# Research Trends in Microbial Remediation of Heavy Metal-Contaminated Soils: A Bibliometric Analysis

**DOI:** 10.3390/microorganisms14051140

**Published:** 2026-05-17

**Authors:** Zhikang Guo, Mu Peng, Haibo Wang

**Affiliations:** 1 Hubei Key Laboratory of Biological Resources Protection and Utilization, Hubei Minzu University, Enshi 445000, China; 15072395917@163.com (Z.G.); pengmu1025@hotmail.com (M.P.); 2 College of Biological and Food Engineering, Hubei Minzu University, Enshi 445000, China

**Keywords:** heavy metal-contaminated soils, microbial remediation, bibliometric analysis, Web of Science Core Collection

## Abstract

Heavy metal contamination in soils threatens ecosystem stability, agricultural productivity, and human health due to its persistence, toxicity, and ecological risks. Microbial remediation has emerged as a sustainable and cost-effective strategy, but the knowledge structure and research trends in this field remain insufficiently summarized. This study conducted a bibliometric analysis of publications on microbial remediation of heavy metal-contaminated soils retrieved from the Web of Science Core Collection from 2000 to 2025. VOSviewer (version 1.6.20), CiteSpace (version 7.0.R0), and the bibliometrix package (version 4.5.0) were used to analyze publication trends, major contributors, influential journals, and keyword evolution. The results showed that the number of publications increased continuously, with rapid growth after 2020. China, India, and the United States were the leading contributors, while Poland, Spain, and the United States played important bridging roles in international collaboration. Ravi Naidu was the most cited author, and Journal of Hazardous Materials was the most productive journal. Keyword analysis revealed a shift from pollutant degradation and microbial screening toward plant–microbe synergistic remediation, co-contaminated soil treatment, microbial community responses, and ecological risk assessment. Future research should emphasize multi-omics-based mechanisms, long-term in situ applications, and integrated evaluation frameworks.

## 1. Introduction

Heavy metal contamination in soils has become a major global environmental challenge because metal pollutants are persistent, toxic, non-biodegradable, and prone to bioaccumulation in terrestrial ecosystems [1]. Excessive accumulation of metals, including cadmium, lead, chromium, arsenic, mercury, copper, zinc, and nickel, can severely impair soil ecological functions and reduce crop productivity and quality. These effects may further threaten food safety and human health through trophic transfer [2]. Unlike many organic contaminants, heavy metals cannot be completely degraded once they are released into the soil environment [3]. Instead, they may persist for long periods, undergo complex transformations among different geochemical fractions, and continuously affect soil microorganisms, plants, and higher organisms [4]. Consequently, the development of effective, environmentally friendly, and sustainable remediation strategies for heavy metal-contaminated soils has become a priority in environmental science, soil science, and agricultural research.

Among the available remediation approaches, microbial remediation has attracted increasing attention because it exploits microbial metabolic potential and ecological functions to reduce heavy metal mobility, bioavailability, and toxicity [5]. Bacteria, fungi, actinomycetes, and plant-associated microorganisms can contribute to heavy metal immobilization, biosorption, extracellular complexation, biomineralization, bioprecipitation, redox transformation, detoxification, and rhizosphere-mediated stabilization processes [6,7]. Compared with conventional physical and chemical remediation methods, microbial remediation offers several important advantages, including lower cost, reduced secondary pollution, greater environmental compatibility, and stronger long-term sustainability [6]. More importantly, microbial processes are closely linked to soil ecological functioning, nutrient cycling, plant performance, and contaminated ecosystem resilience, making microbial remediation not only a technical approach for pollution control but also a key research topic in environmental microbiology and soil microbial ecology [8]. In this study, heavy metal-contaminated soils are defined as soils polluted by common toxic metals and metalloids, including cadmium (Cd), lead (Pb), chromium (Cr), arsenic (As), mercury (Hg), copper (Cu), zinc (Zn), and nickel (Ni). The scope of microbial remediation mainly includes microbe-mediated immobilization, biosorption, bioaccumulation, extracellular complexation, biomineralization, bioprecipitation, redox transformation, detoxification, bioaugmentation, biostimulation, and plant–microbe synergistic remediation. Microorganisms considered in this review include bacteria, fungi, actinomycetes, rhizobacteria, arbuscular mycorrhizal fungi, and other soil- or plant-associated microbial groups involved in reducing the mobility, bioavailability, or toxicity of heavy metals in soils.

Bibliometric analysis is a quantitative and knowledge-mapping approach that uses large-scale literature data to reveal the intellectual structure, research trends, collaboration patterns, and emerging themes in a given field [9]. In recent years, it has been widely applied in environmental science [10], soil science [11], microbial ecology [12], and other related disciplines to identify research hotspots, track thematic evolution, and evaluate the contributions of countries, institutions, authors, and journals. Compared with traditional narrative reviews, bibliometric analysis provides a more systematic and visual perspective on the development of a research area and is particularly useful for understanding rapidly expanding interdisciplinary fields [13]. Although an increasing number of bibliometric studies have examined the remediation of heavy metal-contaminated soils and related topics, most have focused on broader remediation topics, phytoremediation, or contaminated soil remediation [14,15,16,17]. Bibliometric investigations specifically addressing microbial remediation of heavy metal-contaminated soils remain relatively limited [18,19]. Moreover, existing studies have not fully provided a microbe-centered and long-term assessment that integrates microbial functions, collaboration structures, and thematic evolution. Therefore, a systematic bibliometric analysis focusing on microbial functions, collaboration patterns, and thematic evolution in this field remains necessary.

To address this gap, this study performed a bibliometric analysis of publications on the microbial remediation of heavy metal-contaminated soils indexed in the Web of Science Core Collection from 2000 to 2025. Using VOSviewer (version 1.6.20), CiteSpace (version 7.0.R0), and the bibliometrix package (version 4.5.0), we systematically examined publication trends, major contributing countries and institutions, influential authors and journals, and the evolution of research hotspots in this field. The novelty of this study lies in its microbe-centered, long-term, and application-oriented perspective, which provides a comprehensive reference for future work on the screening of functional microorganisms, multi-omics mechanistic exploration, rhizosphere microbiome regulation, remediation stability evaluation, and long-term field application.

## 2. Materials and Methods

### 2.1. Data Source and Literature Search

**Figure 1:**
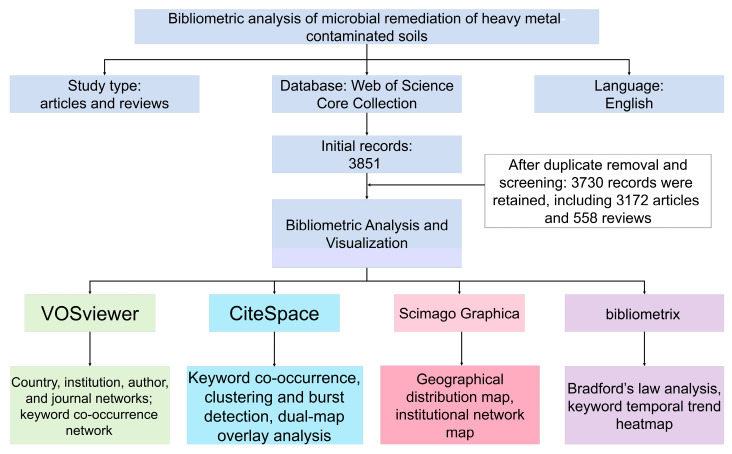
Flowchart of literature retrieval, screening, and bibliometric analysis workflow for research on microbial remediation of heavy metal-contaminated soils.

To systematically explore the development trajectory, major contributors, and evolution of research hotspots in the field of microbial remediation of heavy metal-contaminated soils, the Web of Science Core Collection database was selected as the data source. This database was chosen because it provides high-quality multidisciplinary literature records, standardized bibliographic information, and complete cited reference data, which are essential for bibliometric mapping, co-occurrence analysis, and citation-based evaluation [9]. It has also been widely used as a reliable data source in previous bibliometric studies [13]. The search period covered publications from 1 January 2000 to 31 December 2025. Only documents published in English and classified as Article or Review were considered eligible for inclusion. The retrieval strategy was developed around three key concepts: soil, heavy metal contamination, and microbial remediation. Topic Search (TS) was used in combination with Boolean and proximity operators to improve the relevance of the dataset. The final search formula was as follows: TS = ((soil NEAR/3 (pollut OR contaminat*))) AND TS = (“heavy metal*” OR ((metal* OR metalloid*) NEAR/3 contaminat*) OR cadmium OR lead OR chromium OR arsenic OR mercury OR copper OR zinc OR nickel) AND TS = (bioremediat* OR bioaugment* OR biostimulat* OR ((microb* OR bacteri* OR fung* OR rhizobacter* OR actinomycet*) NEAR/3 (remediat* OR detoxif* OR immobiliz* OR stabiliz*))). A total of 3851 records were initially retrieved. Publications unrelated to heavy metal-contaminated soils or not centered on microbial remediation were excluded from the final dataset. After duplicate removal and screening, 121 records were excluded, and 3730 valid records were retained, including 3172 research articles and 558 reviews, which accounted for 85.0% and 15.0% of the final dataset, respectively (Figure 1). All retained records were exported in plain-text format with the option “Full Record and Cited References” for subsequent statistical analysis and knowledge mapping (Supplementary File S1).

### 2.2. Bibliometric Analysis and Visualization

Several bibliometric and visualization tools were jointly employed to analyze the retrieved literature from different perspectives. VOSviewer (version 1.6.20) was used to construct and visualize collaboration and co-occurrence networks involving countries, institutions, authors, and journals [20]. In these networks, the size of each node reflects the publication output of the corresponding entity, whereas the links between nodes represent collaborative relationships or co-occurrence associations. Thicker links indicate stronger connections. To present the international distribution and cooperation patterns more intuitively, Scimago Graphica (version 1.0.43) was applied to visualize country-level research activity [21]. In addition, the bibliometrix package (version 4.5.0) in R was used to calculate annual publication output, journal-related indicators, and other bibliometric characteristics [22]. Bradford’s law was adopted to identify core journals, and journal impact factors, together with Journal Citation Reports (JCR); quartile information was collected from the corresponding edition of the Journal Citation Reports. To further explore thematic evolution and research hotspots, CiteSpace (version 7.0.R0) was employed for keyword co-occurrence analysis, clustering analysis, and temporal trend analysis [23]. The time span was set from 2000 to 2025, with 1 year per slice, and the node type was defined as keyword. Network simplification was performed using pruning methods such as Pathfinder to enhance the clarity and interpretability of the visualized maps. In the resulting keyword clustering network, larger nodes represent keywords with higher occurrence frequency, different colors correspond to different thematic clusters, and links among nodes indicate co-occurrence relationships between keywords. Denser linkage patterns suggest closer thematic associations.

## 3. Results

### 3.1. Publication Output Trends

**Figure 2:**
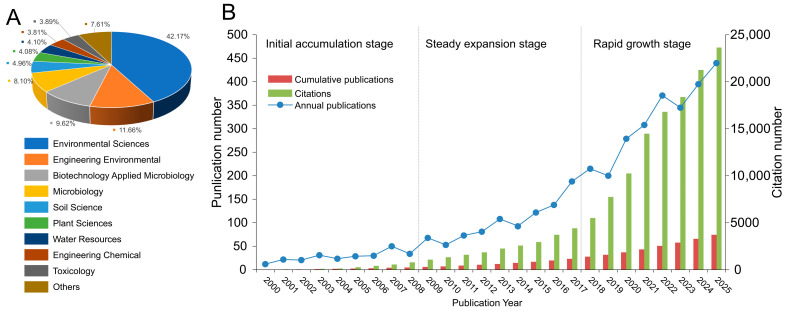
Subject distribution and annual publication trends of studies on microbial remediation of heavy metal-contaminated soils. (**A**), subject distribution; (**B**), annual number of publications and citation counts recorded in WoSCC from 2000 to 2025. “Others” refers to subject categories with relatively small publication shares that are not individually displayed in the figure.

As shown in Figure 2A, research on microbial remediation of heavy metal-contaminated soils shows clear interdisciplinary characteristics. Environmental science accounted for the largest proportion (42.17%), indicating that this field is primarily oriented toward pollution control and ecological restoration. This was followed by environmental engineering (11.66%), applied microbiology and biotechnology (9.62%), and microbiology (8.10%), suggesting that research efforts have mainly focused on remediation technologies and microbial mechanisms. Soil science accounted for 4.96%, with additional contributions from plant science, water resources, chemical engineering, and toxicology. Overall, the field has formed an interdisciplinary research framework centered on environmental science and supported by environmental engineering, microbiology, and soil science.

The annual publication trends from 2000 to 2025 (Figure 2B) showed a continuous increase in the number of publications, indicating growing academic interest. To avoid interpreting the publication increase solely based on total output, the document-type composition of the final dataset was further considered. Among the 3730 records, research articles accounted for 85.0%, whereas reviews accounted for 15.0%, indicating that the growth trend was mainly driven by original research articles rather than reviews alone. Based on the temporal evolution pattern, the development of this field can be divided into three stages. The initial stage (2000–2008) was characterized by relatively low output, generally fewer than 50 publications per year, and slow growth. The second stage (2009–2017) represented a steady expansion phase, with annual publications increasing from approximately 70 to around 140, suggesting that the research framework gradually matured and expanded. The third stage (2018–2025) marked a rapid development phase, with a significant increase in publication output, particularly after 2020. By 2025, the number of annual publications exceeded 400, representing the highest level within the study period. At the same time, citation counts exhibited a similar upward trend. Citations in the early stage were relatively low, whereas a sharp increase was observed after 2018, reaching a peak in 2025. This indicates not only an increase in publication output but also a continuous enhancement of academic impact. Overall, the field has transitioned from an exploratory stage to a phase of rapid expansion and increasing academic influence.

### 3.2. Country/Region Distribution and International Collaboration

At the national level (Table 1), China, India, and the United States were the leading contributors to this field. China ranked first with 1444 publications (39% of the total), significantly exceeding other countries. India and the United States followed with 543 and 259 publications, respectively. Other active countries include Pakistan, Spain, Italy, Australia, Poland, South Korea, and Iran, indicating that, in addition to traditional scientific powers, Asian and some European countries were also highly active in this research area.

**Figure 3:**
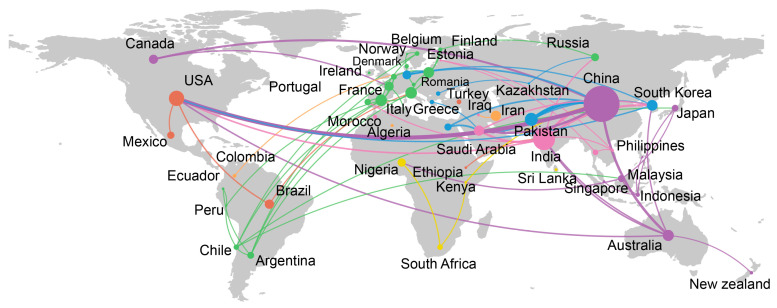
Country/region distribution and collaboration network of research on microbial remediation of heavy metal-contaminated soils based on bibliometric data. Each node represents a country or region, and the node size indicates the number of publications. The connecting lines represent collaborative relationships between countries/regions, with thicker or darker lines indicating stronger collaboration. The node and line colors are mainly used for visual distinction of countries/regions and collaboration links.

However, publication output did not fully correspond to network centrality. As shown in Figure 3, Poland, Spain, and the United States exhibited higher betweenness centrality values (0.20, 0.19, and 0.18, respectively), indicating their important roles as bridges in international collaboration networks. In contrast, although China dominates in publication output, its betweenness centrality value was relatively low (0.04), suggesting that there is room for improvement in international collaboration. Overall, the field demonstrates broad global participation, although collaboration patterns remained somewhat uneven. 

### 3.3. Institutional Distribution and Collaboration

From the perspective of institutional contributions (Table 2), the Chinese Academy of Sciences ranked first with 228 publications and 10,630 citations, making it the leading research institution in this field. It was followed by the University of Chinese Academy of Sciences (78 publications), Zhejiang University (70 publications), and the Council of Scientific and Industrial Research of India (62 publications). Other leading institutions included the Egyptian Knowledge Bank, King Saud University, Nanjing Agricultural University, the French National Centre for Scientific Research (CNRS), China University of Geosciences, and the Spanish National Research Council. This indicates that core research institutions were distributed across China, the Middle East, Europe, and South Asia.

**Figure 4:**
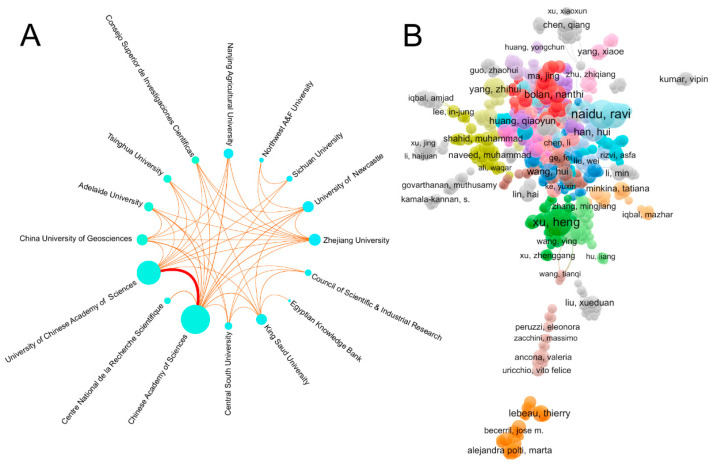
Institution and author collaboration networks in research on microbial remediation of heavy metal-contaminated soils. (**A**) Institution collaboration network. The connecting lines indicate collaborative relationships between institutions; thicker and darker lines represent stronger collaboration strength, with the red line indicating the strongest collaboration link. (**B**) author collaboration network. Different node colors indicate different author collaboration clusters generated by the network analysis. Lines between nodes represent co-authorship relationships.

The collaboration network (Figure 4A) showed that high-output institutions have formed several relatively close collaborative clusters. However, the overall network remained locally clustered and lacked a highly integrated global collaboration structure. Chinese institutions dominated in terms of publication output, whereas some European and American institutions demonstrated stronger international collaboration. This suggests that the research landscape is shifting from regional dominance toward a multicenter structure, but stronger collaboration among leading institutions is still needed.

### 3.4. Author Collaboration Analysis

Research on microbial remediation of heavy metal-contaminated soils involved a large number of scholars from different countries, showing a pattern of broad participation and concentration around leading authors. As shown in Table 3, the most productive authors included Ravi Naidu, Heng Xu, and Mallavarapu Megharaj. Both Ravi Naidu and Heng Xu each published 24 papers and ranked first, followed by Mallavarapu Megharaj with 21 publications. From a geographical perspective, highly productive authors were mainly concentrated in Australia and China, highlighting the strong research presence of these two countries. In terms of citation impact, Ravi Naidu and Mallavarapu Megharaj received 2598 and 2340 citations, respectively, indicating high academic influence. Although authors such as Nanthi Bolan and Andrew S. Ball had fewer publications, their citation counts (1480 and 1317) were also substantial. The author collaboration network (Figure 4B) further showed that productive authors were organized into several collaboration clusters, indicating that author-level cooperation was present but mainly concentrated within specific research groups. Overall, the field was characterized by a core group of highly productive and highly cited researchers.

### 3.5. Journal Distribution and Core Journal Analysis

The core journals identified according to Bradford’s law are presented in Table 4. As shown in Table 4, a total of 15 core journals were identified in the field of microbial remediation of heavy metal-contaminated soils, mainly covering environmental science, pollution control, environmental management, microbiology, and phytoremediation-related disciplines. The leading core journals in terms of publication output were Journal of Hazardous Materials (158 articles), Chemosphere (114 articles), and Environmental Science and Pollution Research (114 articles). This indicates that research outputs in this field were primarily concentrated in a limited number of core journals. In terms of publisher countries, these core journals were mainly distributed in the Netherlands and the United Kingdom, with five journals each; Switzerland accounted for three journals, while the United States and Germany each accounted for one. This suggests that the core journals publishing research in this field were predominantly located in Europe and North America. Regarding journal impact metrics, most of the journals listed in Table 4 showed relatively high academic influence. Among the journals with available 2025 JCR quartile rankings, the majority were classified as Q1. In terms of impact factor, Journal of Hazardous Materials had the highest value at 11.3, followed by Journal of Environmental Management (8.4), Chemosphere (8.1), and Science of the Total Environment (8.0).

**Figure 5:**
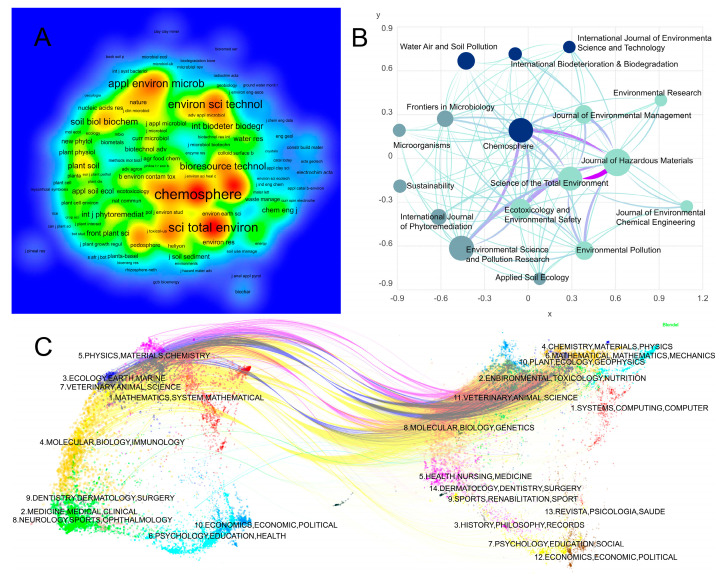
Journal distribution and knowledge flow map in research on microbial remediation of heavy metal-contaminated soils. (**A**) Density map of journals with at least five publications. The color gradient represents journal density, with blue indicating low density and green, yellow, and red indicating progressively higher density; red areas represent the most active journals. (**B**) citation network map of core journals. Node size represents the relative importance or frequency of journals in the network, and lines indicate citation links between journals. The colors of nodes and lines are mainly used for visual distinction. (**C**) journal dual-map overlay. The left side represents citing journals and the right side represents cited journals; colored curves indicate citation paths between subject areas. The colors are used to distinguish different disciplinary regions and citation trajectories.

Figure 5A shows that journals with at least five publications are mainly clustered around nodes such as Chemosphere, Science of the Total Environment, and Journal of Hazardous Materials in the density map. Figure 5B indicates that there are relatively strong connections among the core journals, with Chemosphere, Science of the Total Environment, Environmental Pollution, and Journal of Hazardous Materials occupying prominent positions in the network. Figure 5C presents the results of the journal dual-map overlay analysis, reflecting the main pathways of knowledge input and output in this field. In the figure, the left side represents the disciplines of the citing journals, whereas the right side represents the disciplines of the cited journals, and the colored lines indicate the direction of knowledge flow. Overall, research outputs primarily originated from disciplines such as environmental science, ecology, soil science, and applied microbiology, and showed strong connections with fields such as molecular biology, chemistry, and environmental engineering. This demonstrates that research on the microbial remediation of heavy metal-contaminated soils exhibits a high degree of interdisciplinary knowledge integration characteristics.

### 3.6. Highly Cited Publications

Highly cited publications can, to a certain extent, reflect the foundational knowledge base and core scientific issues of a given research field. As shown in Table 5, the top 10 most cited publications in the field of microbial remediation of heavy metal-contaminated soils had total citation counts ranging from 562 to 1836 and were published between 2003 and 2020. Among them, “Soil Contamination in China: Current Status and Mitigation Strategies” [24], published in Environmental Science & Technology in 2014, ranked first with 1836 citations; “Remediation techniques for heavy metal-contaminated soils: Principles and applicability” [25], published in Science of the Total Environment in 2018, ranked second with 1338 citations; and “Metal contamination and bioremediation of agricultural soils for food safety and sustainability” [26], published in Nature Reviews Earth & Environment in 2020, ranks third with 868 citations.

Overall, the top 10 highly cited publications exhibited strong problem-oriented and application-oriented characteristics. These highly cited studies mainly focused on three aspects: (1) the current status, sources, and mitigation strategies of soil heavy metal contamination; (2) the toxic effects and remediation pathways of typical heavy metals, such as chromium; and (3) microbial processes involved in the remediation of contaminated soils, including remediation technologies for heavy metal-contaminated soils, plant-associated microbial remediation, and biochar–microbe interactions. The highly cited publications listed in Table 5 cover contamination status and ecological risks as well as remediation principles, technical approaches, and microbial mechanisms, indicating that high-impact research in this field mainly focuses on two aspects: understanding pollution effects and developing remediation technologies.

### 3.7. Keyword Co-Occurrence and Research Hotspots Analysis

**Figure 6:**
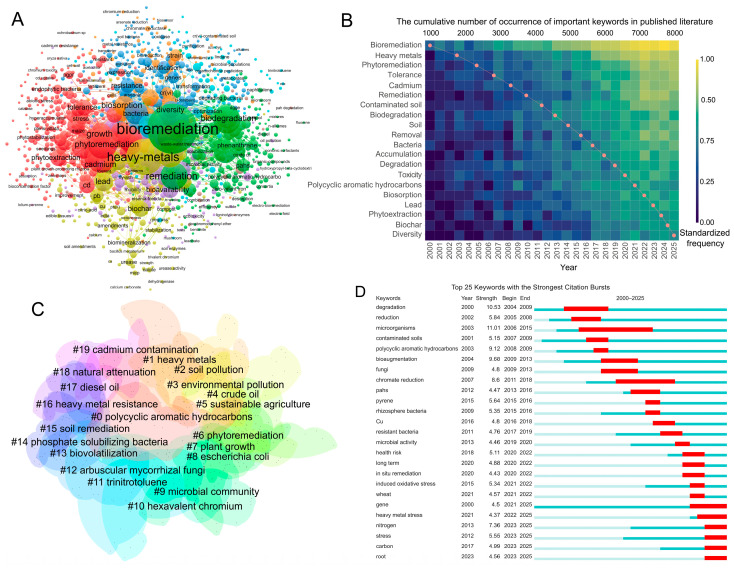
Keyword knowledge map and evolution of research hotspots. (**A**) Keyword co-occurrence network. Different colors represent different keyword clusters. (**B**) Annual heatmap of major keywords. The color gradient represents the standardized frequency of keyword occurrence, with lighter colors indicating higher frequency. (**C**) Keyword clustering map. Different colors represent different keyword clusters. (**D**) Top 25 keywords with the strongest citation bursts. Red bars indicate the burst periods of keywords, blue-green lines indicate the periods after the keywords first appeared but outside the burst periods, and light-colored lines indicate the periods before the keywords first appeared within the analyzed time span.

Keyword co-occurrence analysis indicated that the research field had formed a relatively clear knowledge structure and presented a research network centered on “bioremediation” and “heavy metals” (Figure 6A,B). In the co-occurrence network, keywords such as “bioremediation”, “heavy metals”, “remediation”, “growth”, “biosorption”, “phytoremediation”, “tolerance”, and “cadmium” had larger node sizes and stronger connections, indicating that current research mainly focuses on the bioremediation processes in heavy metal-contaminated soils, while also extending to areas such as phytoremediation enhancement, microbial tolerance mechanisms, regulation of heavy metal bioavailability, and ecological effects of heavy metal pollution. In addition, keywords such as “biodegradation”, “diversity”, “identification”, “genes”, “bioavailability”, “lead”, “Cr(VI)”, “soil”, and “bacteria” also showed strong associations, suggesting that the field has gradually evolved from an early focus on pollutant removal alone to a comprehensive research framework encompassing remediation mechanisms, screening of functional microorganisms, plant–microbe interactions, and ecological risk responses.

Based on the results of keyword clustering (Figure 6C), current research hotspots can be broadly summarized into the following five aspects. First, one major hotspot concerned heavy metal pollution control, removal, and immobilization. This direction was represented by clusters such as “#1 heavy metals”, “#19 cadmium contamination”, “#10 hexavalent chromium”, and “#16 heavy metal resistance”, and was closely associated with keywords such as “biosorption”, “removal”, “lead”, “cadmium”, “Cr(VI)”, and “bioavailability”. This indicates that studies on the adsorption, immobilization, reduction, passivation, and resistance mechanisms of typical heavy metals, such as Cd, Pb, and Cr, have consistently been core topics in this field.

Second, another major hotspot involved bioremediation technologies and the application of functional microorganisms. This theme mainly corresponded to keywords such as “bioremediation”, “remediation”, “biodegradation”, “microorganisms”, “bioaugmentation”, “bacteria”, “genes”, “identification”, and “strain”, reflecting a focus on the screening of functional microorganisms, analysis of degradation/remediation mechanisms, bioaugmentation, and optimization of in situ remediation technologies. These aspects constitute an important foundation for promoting the transition of this field from mechanistic research to engineering applications.

Third, a further hotspot focused on plant–microbe synergistic remediation and agricultural applications. This direction was mainly reflected in clusters such as “#6 phytoremediation”, “#7 plant growth”, “#5 sustainable agriculture”, “#12 arbuscular mycorrhizal fungi”, and “#14 phosphate-solubilizing bacteria”, and is closely associated with keywords such as “phytoremediation”, “phytoextraction”, “rhizosphere bacteria”, “wheat”, and “root”. This indicates that rhizosphere microorganisms, mycorrhizal fungi, and plant growth-promoting bacteria play important roles in enhancing plant growth, improving pollutant uptake or immobilization efficiency, and promoting agricultural ecological restoration.

Fourth, another important hotspot involved organic pollution and the remediation of co-contaminated soils. Clusters such as “#0 polycyclic aromatic hydrocarbons”, “#4 crude oil”, “#11 trinitrotoluene”, “#17 diesel oil”, and “#18 natural attenuation” appeared in the map, and keywords such as “polycyclic aromatic hydrocarbons”, “PAHs”, “pyrene”, “degradation”, and “biodegradation” were also prominent in the burst analysis. This indicates that the field is not limited to heavy metal remediation but has gradually expanded toward the co-remediation of organic pollutants and complex contaminated soils.

Fifth, research also focused on soil microecological processes and ecological risk responses. This direction mainly included cluster “#9 microbial community” and keywords such as “diversity”, “microbial activity”, “tolerance”, “stress”, “heavy metal stress”, “induced oxidative stress”, “health risk”, “nitrogen”, and “carbon”. This suggests that research has shifted from an early focus on single remediation effects to more in-depth issues, including microbial community structure, the maintenance of ecological functions, stress response mechanisms, and environmental health risk assessment.

Based on the keyword temporal distribution heatmap and burst analysis results (Figure 6B,D), the evolution of research hotspots in this field exhibited clear stage-specific characteristics. Early studies mainly focused on topics such as “degradation”, “reduction”, “microorganisms”, “contaminated soils”, and “polycyclic aromatic hydrocarbons”, emphasizing pollutant degradation and basic remediation processes. Subsequently, research gradually shifted toward “bioaugmentation”, “fungi”, “chromate reduction”, “rhizosphere bacteria”, and “resistant bacteria”, reflecting increased attention to functional microorganism applications and remediation mechanisms for specific pollutants. In recent years, research has increasingly focused on topics such as “health risk”, “in situ remediation”, “induced oxidative stress”, “gene”, “heavy metal stress”, “nitrogen”, “stress”, “carbon”, and “root”, indicating that the field is shifting from a traditional pollutant removal-oriented approach toward a stage of development that emphasizes mechanistic understanding, ecological effects, agricultural applications, and risk control.

## 4. Discussion

This study conducted a bibliometric analysis of research on the microbial remediation of heavy metal-contaminated soils based on publications retrieved from the Web of Science Core Collection from 2000 to 2025. The results showed that the number of publications increased continuously from 2000 to 2025, with the growth rate accelerating markedly after 2020. This trend not only reflects an increase in publication volume but also indicates that the field has gradually shifted from an early exploratory stage—mainly focused on the screening of functional microorganisms and the verification of remediation phenomena [34]—to a new stage characterized by parallel advances in mechanistic investigations [34], optimization of integrated remediation strategies, and ecological safety assessment [35]. With the increasing demand for the remediation of heavy metal-contaminated soils and the rapid development of high-throughput sequencing, metagenomics, and multi-omics technologies, research emphasis has gradually moved from assessing remediation feasibility toward understanding the underlying mechanisms, stability, and safety of remediation processes [36,37].

The rapid increase in publications during 2018–2025 may be attributed to several emerging research directions in the microbial remediation of heavy metal-contaminated soils. First, increasing attention has been paid to plant–microbe synergistic remediation, particularly to the use of plant growth-promoting rhizobacteria, endophytic bacteria, arbuscular mycorrhizal fungi, and rhizosphere microbial communities to enhance metal immobilization, phytoextraction, and plant stress tolerance [38]. This trend is consistent with recent studies emphasizing the molecular mechanisms of phytoremediation and the potential of engineered plants and microbes for improving contaminant tolerance and remediation efficiency [39]. Second, the application of multi-omics approaches, including metagenomics, transcriptomics, proteomics, and metabolomics, has promoted a deeper understanding of microbial community responses, functional genes, metal-resistance pathways, and rhizosphere interactions under heavy metal stress [40]. Third, research has increasingly shifted from single-strain remediation toward microbial consortia, bioaugmentation, and combined microbe–plant–material systems, which are considered more suitable for complex and heterogeneous field conditions [41]. In addition, studies on co-contaminated soils, especially those polluted by both heavy metals and organic contaminants, have further expanded the research scope of this field [42].

In terms of microbial groups, bacteria-related studies appeared to dominate dataset, as reflected by the frequent occurrence of keywords such as “bacteria”, “rhizosphere bacteria”, “resistant bacteria”, “bioaugmentation”, and “biosorption” (Figure 6). Fungi-related studies also formed an important component of this field, especially in relation to arbuscular mycorrhizal fungi, plant growth promotion, and metal tolerance [43]. In contrast, studies related to algae and cyanobacteria were less prominent in the keyword network, probably because these microorganisms are more commonly applied in aquatic environments or wastewater treatment than in terrestrial soil systems [44]. These results suggest that bacteria remain the primary microbial group investigated in heavy metal-contaminated soil remediation, whereas fungi and algae provide complementary but less extensively explored pathways.

The distribution of countries, institutions, and authors showed that China, India, and the United States were the major contributors in this field, with China holding a clear advantage in publication output, whereas the United States, Spain, and Poland played more prominent bridging roles in the collaboration network. This suggests that the field has formed a relatively broad pattern of international participation [6], although high productivity and high connectivity do not always coincide, and stronger cross-institutional and cross-national collaboration is still needed. Meanwhile, core institutions and authors, such as the Chinese Academy of Sciences, Zhejiang University, Ravi Naidu, Heng Xu, and Mallavarapu Megharaj, have formed relatively stable knowledge-producing groups, indicating that a comparatively stable core group of authors and dominant research teams has emerged in this field. To further enhance the international influence of this field, it will be necessary not only to maintain high-quality publication output but also to strengthen collaboration across regions, institutions, and disciplines.

In addition to publication output, the geographical distribution of heavy metal-contaminated soils may help explain the strong research activity observed in several countries. Although direct comparisons of contaminated soil areas among countries are difficult because of differences in monitoring standards, sampling density, land-use categories, and national reporting systems, available evidence indicates that countries with intensive mining, smelting, industrialization, wastewater irrigation, and high agricultural inputs often face more extensive heavy metal contamination risks [45]. For example, widespread soil contamination has been reported in China, particularly in agricultural land, industrial areas, mining regions, wastewater-irrigated areas, and areas along transportation routes [46]. This may partly explain why China has become the most productive country in this field. India also faces considerable pressure from heavy metal contamination associated with mining activities, rapid industrialization, urban expansion, wastewater irrigation, and intensive agriculture [47], which is consistent with its high publication output. In contrast, the United States and several European countries have a long history of industrial and mining-related soil contamination [48], but they also have relatively mature monitoring, risk assessment, and remediation management systems. These differences suggest that national publication patterns are shaped not only by research capacity but also by the severity of contamination, food safety concerns, remediation demand, and policy-driven environmental management.

The results of keyword co-occurrence, clustering, and burst analyses indicate that research hotspots in this field have evolved in distinct stages. Early studies mainly focused on topics such as “degradation”, “reduction”, “microorganisms”, and “contaminated soils”, emphasizing the identification functional microorganisms and verification of their potential for pollutant degradation or transformation [49,50]. Subsequently, research gradually shifted toward “bioaugmentation”, “fungi”, “chromate reduction”, “rhizosphere bacteria”, and “resistant bacteria”, reflecting increasing attention to the application of functional microorganisms, remediation mechanisms of specific pollutants, and rhizosphere microbial processes [7,51,52]. In recent years, keywords such as “health risk”, “in situ remediation”, “induced oxidative stress”, “gene”, “heavy metal stress”, “nitrogen”, “stress”, “carbon”, and “root” have shown stronger burst intensity and remained active up to 2025, indicating that the field has moved beyond a sole focus on remediation processes and efficiency [36] toward greater emphasis on soil microecological responses [53], ecological safety, and risk control [54]. The simultaneous appearance of organic pollution-related terms among the high-frequency keywords also suggests that microbial remediation research is gradually extending toward the treatment of co-contaminated soils and system-level remediation [55,56]. This trend is consistent with the recent rapid development of multi-omics technologies, risk assessment frameworks, and studies on plant–microbe synergistic remediation, and further indicates that the field is placing increasing emphasis on the long-term stability, ecological adaptability, and practical safety of remediation systems. Compared with previous bibliometric studies focusing on contaminated-soil remediation in general or shorter observation windows [18,19], the present analysis more clearly reveals a transition from pollutant-removal-oriented research to integrative themes involving plant–microbe interactions, microbial community regulation, and ecological risk assessment. This transition reflects the increasing incorporation of microbial ecology, systems-level analysis, and application-oriented validation into heavy metal remediation research.

It should also be noted that this study has several limitations. First, the data source was limited to the Web of Science Core Collection; Scopus, PubMed, and Chinese databases were not included, which may have resulted in insufficient coverage of some regional contributions and non-English studies. Second, bibliometric results are influenced by the search strategy, database indexing rules, document-type screening, and keyword standardization procedures; therefore, the identified network structures and hotspot identification results may vary under different parameter settings [57]. Third, although keyword co-occurrence and burst analyses can effectively reflect macroscopic hotspots and frontier changes, they cannot fully replace in-depth evaluations of specific research content, remediation efficiency, and engineering applicability [58]. Therefore, future studies should further combine bibliometric analysis with systematic reviews, case comparisons, and field application evidence to develop a more comprehensive and in-depth understanding of research on the microbial remediation of heavy metal-contaminated soils.

In light of recent research progress, at least four directions deserve particular attention in future studies in this field. First, greater efforts should be devoted to multi-omics analyses of the interactions between functional microorganisms and indigenous microbial communities [35], especially key processes such as heavy metal migration and transformation, extracellular polymer secretion, redox regulation, stress-resistance metabolism, and functional gene expression, to establish a mechanistic chain from community structure to functional output. Second, remediation systems should move from single-strain applications toward complex microbial consortia, rhizosphere synergistic systems, and combined microbe–plant–material remediation models to improve adaptability and stability under complex environmental conditions [7]. Third, greater emphasis should be placed on in situ remediation, long-term field validation, and applicability studies across different ecological regions, so as to avoid limiting technology evaluation to indoor pot experiments or short-term simulation studies [6]. Fourth, the evaluation of remediation performance should be expanded beyond reductions in pollutant concentrations to include more comprehensive endpoint indicators, such as stabilization of heavy metal speciation, restoration of soil ecological functions, microbial risk control, and reduction in human health risks, thereby promoting the development of microbial remediation from feasibility toward reliability and practical applicability.

## 5. Plant–Microbe Synergistic Remediation: Mechanisms, Applications, and Challenges

The keyword analyses showed that plant–microbe synergistic remediation has become one of the emerging hotspots in the microbial remediation of heavy metal-contaminated soils (Figure 6). This trend is scientifically important because plant-associated microorganisms can directly or indirectly regulate heavy metal mobility, bioavailability, and plant tolerance [59]. For example, rhizosphere bacteria, endophytic bacteria, arbuscular mycorrhizal fungi, and other plant growth-promoting microorganisms can enhance plant growth under heavy metal stress by producing extracellular polymeric substances, organic acids, siderophores, phytohormones, and stress-regulating enzymes [60]. These microbial processes may promote metal immobilization in the rhizosphere, reduce metal toxicity to plants, or enhance phytoextraction efficiency, depending on the plant species, microbial functional traits, soil properties, and target metals involved [61].

Compared with single microbial inoculation or phytoremediation alone, plant–microbe synergistic remediation provides a more ecologically integrated strategy for contaminated soils by combining plant uptake or stabilization with microbe-mediated metal transformation, immobilization, and plant growth promotion [62]. Plants create rhizosphere niches and release root exudates that support microbial colonization and activity, whereas microorganisms improve nutrient acquisition, regulate metal transformation, and enhance plant stress resistance [7]. This mutualistic interaction is particularly important for heavy metal-contaminated agricultural soils, where remediation strategies should not only reduce metal risks but also maintain soil fertility and plant productivity [63]. Recent studies have also emphasized that molecular mechanisms, microbial community assembly, functional genes, and plant–microbe signaling pathways are critical for improving the stability and predictability of this remediation strategy [64,65].

However, the practical application of plant–microbe synergistic remediation still faces several challenges. Many studies remain limited to pot experiments or short-term laboratory simulations, whereas field-scale validation under heterogeneous soil conditions remains insufficient [66]. In addition, the survival, colonization, and functional stability of introduced microorganisms may be affected by indigenous microbial communities, soil pH, organic matter content, metal speciation, and climatic conditions [6]. Therefore, future application-oriented studies should pay greater attention to microbial colonization stability, plant–microbe compatibility, long-term remediation performance, and ecological safety assessment. This focused discussion supports the bibliometric finding that the field is shifting from simple pollutant removal toward integrated remediation systems involving microbial mechanisms, plant performance, and ecological risk control.

## 6. Conclusions

The results showed that this field experienced continuous growth, with marked acceleration after 2020. China, India, and the United States were the major contributing countries, while the Chinese Academy of Sciences, Ravi Naidu, and Journal of Hazardous Materials represented the important institution, author, and journal, respectively. In terms of remediation implications, microbial remediation contributes to reducing the mobility, bioavailability, and toxicity of heavy metals in soils through processes such as biosorption, immobilization, biomineralization, bioprecipitation, redox transformation, bioaugmentation, and plant–microbe synergistic remediation. Keyword co-occurrence, clustering, and burst analyses further showed that research hotspots have shifted from early pollutant removal and screening of functional microorganisms toward plant–microbe synergistic remediation, co-contaminated soil treatment, microbial community responses, in situ remediation, and ecological risk assessment. These findings indicate that the field is developing from a pollutant-removal-oriented stage toward a broader framework integrating microbial mechanisms, ecological responses, risk assessment, and application-oriented remediation.

## Figures and Tables

**Table 1:** Top 10 countries/regions in terms of publication output in the field of microbial remediation of heavy metal-contaminated soils.

Rank	Country	Publications	Betweenness Centrality	Publication Share *
1	China	1444	0.04	0.39
2	India	543	0.03	0.15
3	United States	259	0.18	0.07
4	Pakistan	190	0.12	0.05
5	Spain	153	0.19	0.04
6	Italy	148	0.07	0.04
7	Australia	140	0.09	0.04
8	Poland	133	0.20	0.04
9	South Korea	132	0.14	0.04
10	Iran	120	0.00	0.03

*: Publication share was calculated as the number of publications from a given country divided by the total number of publications in the dataset.

**Table 2:** Top 10 research institutions in terms of publication output in the field of microbial remediation of heavy metal-contaminated soils.

Rank	Institution	Publications	Citations	Country
1	Chinese Academy of Sciences	228	10,630	China
2	University of Chinese Academy of Sciences	78	2718	China
3	Zhejiang University	70	3513	China
4	Council of Scientific and Industrial Research	62	1781	India
5	Egyptian Knowledge Bank	54	1586	Egypt
6	King Saud University	49	1479	Saudi Arabia
7	Nanjing Agricultural University	47	3297	China
8	French National Centre for Scientific Research	45	1740	France
9	China University of Geosciences	44	4342	China
10	Spanish National Research Council	43	2293	Spain

**Table 3:** Top 10 authors in terms of publication output in the field of microbial remediation of heavy metal-contaminated soils.

Rank	Author	Publications	Citations	Country
1	Naidu, Ravi	24	2598	Australia
2	Xu, Heng	24	1343	China
3	Megharaj, Mallavarapu	21	2340	Australia
4	Han, Hui	12	478	China
5	Achal, Varenyam	12	334	China
6	Thavamani, Palanisami	10	739	Australia
7	Bolan, Nanthi	9	1480	Australia
8	Yao, Jun	8	457	China
9	Minkina, Tatiana	8	179	Russia
10	Ball, Andrew S	8	1317	Australia

**Table 4:** Top 15 journals in terms of publication output in the field of microbial remediation of heavy metal-contaminated soils.

Journal Title	Publications	Country	IF (2025)	JCR (2025)
Journal of Hazardous Materials	158	The Netherlands	11.3	Q1
Chemosphere	114	United Kingdom	8.1	N/A *
Environmental Science and Pollution Research	114	Germany	N/A	N/A
Science of the Total Environment	111	The Netherlands	8.0	Q1
Ecotoxicology and Environmental Safety	92	United Kingdom	6.1	Q1
Environmental Pollution	75	United Kingdom	7.3	Q1
Journal of Environmental Management	67	United Kingdom	8.4	Q1
Water Air and Soil Pollution	61	The Netherlands	3.0	Q2
Frontiers in Microbiology	60	Switzerland	4.5	Q1
International Journal of Phytoremediation	43	United States	3.1	Q2
Sustainability	42	Switzerland	3.3	Q2
Journal of Environmental Chemical Engineering	38	The Netherlands	7.2	Q1
International Biodeterioration and Biodegradation	35	United Kingdom	4.1	Q2
Microorganisms	35	Switzerland	4.2	Q2
Environmental Research	34	The Netherlands	7.7	Q1

*: Not Available.

**Table 5:** Top 10 most cited publications in the field of microbial remediation of heavy metal-contaminated soils.

Rank	Title	Year	Journal	First Author	Total Citations	References
1	Soil Contamination in China: Current Status and Mitigation Strategies	2014	Environmental Science & Technology	Zhao Fang-Jie	1836	[24]
2	Remediation techniques for heavy metal-contaminated soils: Principles and applicability	2018	Science of the Total Environment	Lianwen Liu	1338	[25]
3	Metal contamination and bioremediation of agricultural soils for food safety and sustainability	2020	Nature Reviews Earth & Environment	Deyi Hou	868	[26]
4	Effects and mechanisms of biochar-microbe interactions in soil improvement and pollution remediation: A review	2017	Environmental Pollution	Xiaomin Zhu	819	[27]
5	Chromium in the environment: factors affecting biological remediation	2003	Plant and Soil	Adel M. Zayed	739	[28]
6	Bioremediation techniques-classification based on site of application: principles, advantages, limitations and prospects	2016	World Journal of Microbiology & Biotechnology	Azubuike Christopher Chibueze	678	[29]
7	Perspectives of plant-associated microbes in heavy metal phytoremediation	2012	Biotechnology Advances	Rajkumar M	648	[30]
8	Biosorption and biotransformation of hexavalent chromium [Cr(VI)]: A comprehensive review	2018	Chemosphere	Jobby Renitta	624	[31]
9	Microbial and Plant-Assisted Bioremediation of Heavy Metal Polluted Environments: A Review	2017	International Journal of Environmental Research and Public Health	Ojuederie Omena Bernard	577	[32]
10	Sphingomonas: from diversity and genomics to functional role in environmental remediation and plant growth	2020	Critical Reviews in Biotechnology	Asaf Sajjad	562	[33]

## Data Availability

The original contributions presented in this study are included in the article/Supplementary Materials. Further inquiries can be directed to the corresponding author.

## Supplementary Materials

The following supporting information can be downloaded at: https://www.mdpi.com/article/10.3390/microorganisms14051140/s1, Supplementary File S1 Full records and cited references exported from Web of Science.
