# Supplementary material for: Research Trends in Microbial Remediation of Heavy Metal-Contaminated Soils: A Bibliometric Analysis
Source: Microorganisms. 2026 May 17;14(5):1140. doi: 10.3390/microorganisms14051140 (PMC13209943; doi:10.3390/microorganisms14051140)
Supplement: Supplementary file 1 [file microorganisms-4300880-supplementary.pdf › microorganisms-4300880-supplementary.pdf]

FN Clarivate Analytics Web of Science
VR 1.0
PT J
"AU Liang, L"
"AF Liang, Li"
TI RETRACTED: Numerical Simulation Method for Microbial Remediation Effect
of Nano Heavy Metal Contaminated Soil (Retracted Article)
SO JOURNAL OF NANOMATERIALS
LA English
DT Article; Retracted Publication
ID PHYTOREMEDIATION; WATER
"AB Heavy metal soil remediation is an important component in mitigating environmental problems, and microbial remediation has good treatment effect, good environmental affinity, and high treatment cost treatment efficiency. Numerical simulations of soil remediation effects enable the selection of suitable remediation methods and the determination of the optimal remediation input ratio. However, the current numerical simulation mainly relies on a single mathematical model, and the simulation error is large when applied to the microbial remediation with large variability. Numerical simulation methods of microbial remediation impacts of heavy metal contaminated soils will be investigated to overcome the foregoing problems. By researching the migratory law of heavy metal contaminated soil components, the process of microbial remediation of heavy metal contaminated soil will be examined, and a microbial proliferation model will be constructed. The numerical simulation of microbial remediation effect is realized by using HYDRUS to inverse solve the data of small laboratory tests and obtain the relevant parameters for numerical simulation. The maximum simulation error of the method studied at the time of testing was 2.1%, the simulation trend was consistent with the real remediation effect, and the simulation results were reliable. Although the simulation results of the numerical simulation method of the microbial remediation effect of heavy metal polluted soil proposed in this paper differ from actual values of the microbial remediation of heavy metals in soil, the overall trend of changes in soil heavy metal content is similar. It reveals that the outcomes of the approach investigated in this work are somewhat trustworthy when numerically modeling the effect of microbial remediation of heavy metals in soils. In other words, the numerical modeling approach utilized in this work to examine the impact of microbial remediation of heavy metal-contaminated soil is highly precise and effective."
"C1 [Liang, Li] Chongqing Ind Polytech Coll, Chongqing 401120, Peoples R China."
C3 Chongqing Industry Polytechnic University
"RP Liang, L (corresponding author), Chongqing Ind Polytech Coll, Chongqing 401120, Peoples R China."
EM 19402054@masu.edu.cn
"FU Natural Science Foundation of Chongqing, China; [cstc2021jcyj-msxm2408]"
FX AcknowledgmentsThis work is sponsored by Natural Science Foundation of
"   Chongqing, China (no. cstc2021jcyj-msxm2408)."
"CR Akbarpour F, 2021, ENVIRON EARTH SCI, V80, DOI 10.1007/s12665-021-10024-x"
"   Alazzam MB, 2022, ADV MATER SCI ENG, V2022, DOI 10.1155/2022/7774180"
"   Alazzam MB, 2022, COMPUT INTEL NEUROSC, V2022, DOI 10.1155/2022/1422963"
"   Cheng JM, 2019, INT J PHYTOREMEDIAT, V21, P634, DOI 10.1080/15226514.2018.1556581"
"   Fajardo C, 2015, SCI TOTAL ENVIRON, V535, P79, DOI 10.1016/j.scitotenv.2015.03.067"
"   Gaur VK, 2021, BIOENGINEERED, V12, P7297, DOI 10.1080/21655979.2021.1978616"
"   Gayathri S, 2021, SUST WAT RESOUR MAN, V7, DOI 10.1007/s40899-021-00502-2"
"   Gremion F, 2004, FEMS MICROBIOL ECOL, V48, P273, DOI 10.1016/j.femsec.2004.02.004"
"   Hamad AA, 2022, COMPUT INTEL NEUROSC, V2022, DOI 10.1155/2022/4569879"
"   Issakhov A, 2019, ENG APPL COMP FLUID, V13, P279, DOI 10.1080/19942060.2019.1584126"
"   Joseph MP, 2020, EXP COMPUT MULTI FLO, V2, P162, DOI 10.1007/s42757-019-0043-9"
"   Khan MA, 2021, J ENVIRON CHEM ENG, V9, DOI 10.1016/j.jece.2021.106019"
"   Magdoff F, 2021, MON REV, V72, P1, DOI 10.14452/mr-072-11-2021-04_1"
"   Mohiuddin M, 2021, SUSTAINABILITY-BASEL, V13, DOI 10.3390/su131910967"
"   Mukherjee T, 2020, HELIYON, V6, DOI 10.1016/j.heliyon.2020.e03548"
"   Nishimura Ibuki, 2021, Japanese Geotechnical Society Special Publication, V9, P319, DOI [10.3208/jgssp.v09.cpeg017, 10.3208/jgssp.v09.cpeg017]"
"   Pan Y, 2021, WATER-SUI, V13, DOI 10.3390/w13010096"
"   Pellinen VA, 2021, AGRONOMY-BASEL, V11, DOI 10.3390/agronomy11102007"
"   Qi SQ, 2023, PEDOSPHERE, V33, P61, DOI 10.1016/j.pedsph.2022.07.004"
"   Qiu XQ, 2012, CHEM ENG J, V193, P358, DOI 10.1016/j.cej.2012.04.067"
"   Selvi A, 2021, J ENVIRON CHEM ENG, V9, DOI 10.1016/j.jece.2021.106602"
"   Suebyat K, 2018, ABSTR APPL ANAL, V2018, P1, DOI [10.1155/2018/9025851, 10.1155/2018/9025851, DOI 10.1155/2018/9025851]"
"   Tan H, 2020, CHEMOSPHERE, V242, DOI 10.1016/j.chemosphere.2019.125251"
"   Toki T, 2021, J FLUID MECH, V928, DOI 10.1017/jfm.2021.814"
"   Tseng SC, 2021, INT J ENV RES PUB HE, V18, DOI 10.3390/ijerph18168661"
"   Ubuza LJA, 2020, ENVIRON ENG RES, V25, P977, DOI 10.4491/eer.2019.258"
"   Wang X., 2020, COMPUTER SIMULATION, V37, P435"
"   Yaqoob AA, 2021, POLYMERS-BASEL, V13, DOI 10.3390/polym13010135"
"   Yuhu Luo, 2021, IOP Conference Series: Earth and Environmental Science, V769, DOI [10.1088/1755-1315/769/2/022032, 10.1088/1755-1315/769/2/022032]"
NR 29
TC 1
Z9 1
U1 1
U2 108
PU HINDAWI LTD
PI LONDON
"PA ADAM HOUSE, 3RD FLR, 1 FITZROY SQ, LONDON, W1T 5HF, the United Kingdom"
SN 1687-4110
EI 1687-4129
J9 J NANOMATER
JI J. Nanomater.
PD AUG 5
PY 2022

VL 2022
AR 3318917
DI 10.1155/2022/3318917
PG 9
"WC Nanoscience & Nanotechnology; Materials Science, Multidisciplinary"
WE Science Citation Index Expanded (SCI-EXPANDED)
SC Science & Technology - Other Topics; Materials Science
GA 3Z7SZ
UT WOS:000844618100002
"OA Green Submitted, hybrid"
DA 2026-04-06
ER

PT J
"AU Li, SQ"
"   Yan, X"
"   Zhang, MJ"
"   Sun, Q"
"   Zhu, XZ"
"AF Li, Shuangquan"
"   Yan, Xiao"
"   Zhang, Mingjiang"
"   Sun, Qi"
"   Zhu, Xuezhe"
TI Microbial remediation technology for heavy metal contamination of mine
   soil
SO CHEMOECOLOGY
LA English
DT Review
DE Heavy metal; Mineral soil; Pollution; Remediation technology; Microbial
   remediation
ID HEALTH-RISK ASSESSMENT; ATMOSPHERIC DEPOSITION; SPATIAL-DISTRIBUTION;
   AGRICULTURAL SOIL; POLLUTION; REMOVAL; LEAD; BIOMINERALIZATION;
   SPECIATION; TAILINGS
"AB The problem of persistent and potentially toxic heavy metal-contaminated soils is widespread worldwide. These heavy metal concentrations have continued to accumulate in soils over the past three decades, posing a threat to the environment and human health. Therefore, the problem of heavy metal soil contamination needs to be addressed urgently. In this paper, we describe the sources of heavy metal pollution and their hazardous properties, emphasizing the urgent need for treatment. Traditional remediation techniques for heavy metal contamination include physical, chemical, and biological methods. Each of these methods has its advantages and disadvantages and can be combined to remove (transform) or reduce heavy metals in contaminated soils to safe levels. The article provides a comparative analysis of traditional remediation techniques for heavy metal contamination, focusing on a review of the current status of research and application of microbial remediation methods in heavy metal contamination of mine soils. The paper also discusses the combined microbial and chemical/plant remediation techniques. In conclusion, the remediation and treatment of heavy metal-contaminated soils should consider both the economy and effectiveness of the remediation technology. Microbial remediation is a typical remediation technology that will become the main development trend due to its effectiveness and low cost."
"C1 [Li, Shuangquan; Yan, Xiao; Zhang, Mingjiang; Sun, Qi; Zhu, Xuezhe] GRINM Grp Co Ltd, Natl Engn Res Ctr Environm Friendly Met Producing, Beijing 101407, Peoples R China."
"   [Li, Shuangquan; Yan, Xiao; Zhang, Mingjiang; Sun, Qi; Zhu, Xuezhe] GRINM Resources & Environm Tech Co Ltd, Beijing 101407, Peoples R China."
"   [Li, Shuangquan; Yan, Xiao; Zhang, Mingjiang; Sun, Qi; Zhu, Xuezhe] Gen Res Inst NonFerrous Met, Beijing 100088, Peoples R China."
"   [Li, Shuangquan; Yan, Xiao; Zhang, Mingjiang; Sun, Qi; Zhu, Xuezhe] Beijing Engn Res Ctr Strateg Nonferrous Met Green, Beijing 100088, Peoples R China."
"   [Yan, Xiao; Zhang, Mingjiang; Sun, Qi; Zhu, Xuezhe] GRIMAT Engn Inst Co Ltd, Beijing 101407, Peoples R China."
C3 General Research Institute for Nonferrous Metals
"RP Zhang, MJ (corresponding author), GRINM Grp Co Ltd, Natl Engn Res Ctr Environm Friendly Met Producing, Beijing 101407, Peoples R China.; Zhang, MJ (corresponding author), GRINM Resources & Environm Tech Co Ltd, Beijing 101407, Peoples R China.; Zhang, MJ (corresponding author), Gen Res Inst NonFerrous Met, Beijing 100088, Peoples R China.; Zhang, MJ (corresponding author), Beijing Engn Res Ctr Strateg Nonferrous Met Green, Beijing 100088, Peoples R China.; Zhang, MJ (corresponding author), GRIMAT Engn Inst Co Ltd, Beijing 101407, Peoples R China."
EM zmj0630@163.com
"RI ; Li, Shuangquan/HJI-2585-2023"
"OI Zhang, Mingjiang/0000-0001-5225-0042; "
FU the National Natural Science Foundation of China [51974279]; National
"   Natural Science Foundation of China [2018YFC18027, 2018YFC18018];"
   National Key Research and & Development Program of China
FX This research was funded by the National Natural Science Foundation of
"   China [grant numbers 51974279], the National Key Research and &"
"   Development Program of China [grant numbers 2018YFC18027, 2018YFC18018]."
"CR Acosta-Dacal A, 2022, ENVIRON POLLUT, V301, DOI 10.1016/j.envpol.2022.118979"
"   [Anonymous], 2021, SCI TECHNOL ENG, V21, P9634"
"   Arab F, 2018, ENVIRON SCI POLLUT R, V25, P16202, DOI 10.1007/s11356-018-1770-3"
"   Awasthi G, 2022, MINERALS-BASEL, V12, DOI 10.3390/min12010085"
"   Bai CRGYSYWTG., 2021, NONFERROUS METALS MI, V73, P111"
"   Bhandari G, 2020, CHEMOSPHERE, V253, DOI 10.1016/j.chemosphere.2020.126594"
"   Ceci A, 2019, APPL MICROBIOL BIOT, V103, P53, DOI 10.1007/s00253-018-9451-1"
"   Chakraborty B, 2021, ENVIRON SCI POLLUT R, V28, P47275, DOI 10.1007/s11356-021-14012-3"
"   Chen CF, 2013, SOIL SEDIMENT CONTAM, V22, P546, DOI 10.1080/15320383.2013.750268"

" Chen G.R., 2010, CONSERVATION UTILIZA, V2, P41, DOI [10.13779/j.cnki.issn1001-0076.2010.02.007, DOI 10.13779/J.CNKI.ISSN1001-0076.2010.02.007]"
" Chen P, 2017, ACTA GEOCHIM, V36, P539, DOI 10.1007/s11631-017-0230-y"
" [????????? Chen Shibao], 2019, [????????????, Earth Science Frontiers], V26, P35"
" Chen YX, 2018, PROCESS SAF ENVIRON, V113, P204, DOI 10.1016/j.psep.2017.10.008"
" Chen Z, 2020, WORLD NONFERROUS MET, P217"
" Cornu JY, 2017, WORLD J MICROB BIOT, V33, DOI 10.1007/s11274-016-2191-4"
" [????????? Dai Qingyun], 2018, [?????????????????€???, Environmental Science and Technology], V41, P56"
" Ding Q, 2017, SCI TOTAL ENVIRON, V578, P577, DOI 10.1016/j.scitotenv.2016.11.001"
" Doabi SA, 2018, ECOTOX ENVIRON SAFE, V163, P153, DOI 10.1016/j.ecoenv.2018.07.057"
" Dong LJ, 2020, J CLEAN PROD, V269, DOI 10.1016/j.jclepro.2020.122270"
" Faheem M, 2020, MICROORGANISMS, V8, DOI 10.3390/microorganisms8040505"
" Fu LJ, 2021, J HAZARD MATER, V403, DOI 10.1016/j.jhazmat.2020.123847"
" Fu RB, 2017, CHEM ENG J, V316, P601, DOI 10.1016/j.cej.2017.01.092"
" Fu W, 2017, INT J ENVIRON RES, V11, P515, DOI 10.1007/s41742-017-0045-z"
" GAO Wei-qiang, 2006, J XIAMEN U NATURAL S, P281"
" Gao Y, 2022, ECOTOX ENVIRON SAFE, V231, DOI 10.1016/j.ecoenv.2022.113198"
" Garbisu C, 2001, BIORESOURCE TECHNOL, V77, P229, DOI 10.1016/S0960-8524(00)00108-5"
" Gong YY, 2018, WATER RES, V147, P440, DOI 10.1016/j.watres.2018.10.024"
" Gu WZ, 2021, ECOTOX ENVIRON SAFE, V218, DOI 10.1016/j.ecoenv.2021.112263"
" Hai-yong GTLB-tL, 2022, CHEM ENG DES COMMUN, V48, P187"
" He JY, 2018, ENVIRON SCI POLLUT R, V25, P17682, DOI 10.1007/s11356-018-1741-8"
" Hu BF, 2020, ENVIRON POLLUT, V266, DOI 10.1016/j.envpol.2020.114961"
" Hu L., 2020, Conserv. Util. Miner. Resour, V40, P40"
" Issaka E, 2022, CHEMOSPHERE, V297, DOI 10.1016/j.chemosphere.2022.134163"
" Jalilvand N, 2020, J SOIL SCI PLANT NUT, V20, P206, DOI 10.1007/s42729-019-00121-z"
" Jamal A, 2019, HUM ECOL RISK ASSESS, V25, P1018, DOI 10.1080/10807039.2018.1460191"
" Jiang L, 2019, HUM ECOL RISK ASSESS, V25, P374, DOI 10.1080/10807039.2019.1568856"
" Jiang LH, 2020, ECOTOX ENVIRON SAFE, V191, DOI 10.1016/j.ecoenv.2019.110009"
" Jing WXWCWXWJMXYXHXG., 2019, CHEM BIOENG, V36, P1"
" [?????? Wang Kun], 2018, [??????????????????, Chinese Journal of Engineering], V40, P526, DOI 10.13374/j.issn2095-9389.2018.05.002"
" Khalid S, 2017, J GEOCHEM EXPLOR, V182, P247, DOI 10.1016/j.gexplo.2016.11.021"
" Kou B, 2023, ENVIRON POLLUT, V322, DOI 10.1016/j.envpol.2023.121136"
" Li Y, 2021, ENVIRON POLLUT, V284, DOI 10.1016/j.envpol.2021.117465"
" Lin H, 2021, J HAZARD MATER, V402, DOI 10.1016/j.jhazmat.2020.123829"
" [????????? Liu Dandan], 2015, [????????????, Geoscience], V29, P390"
" Liu HL, 2019, J HAZARD MATER, V362, P9, DOI 10.1016/j.jhazmat.2018.09.032"
" Liu JC, 2020, INT J ENV RES PUB HE, V17, DOI 10.3390/ijerph17124315"
" Liu JP, 2021, J HAZARD MATER, V403, DOI 10.1016/j.jhazmat.2020.123638"
" [?????? Liu Lian], 2013, [???????????????, Geology and Prospecting], V49, P437"
" Liu Ling, 2009, Huanjing Kexue, V30, P3422"
" Liu Wei, 2016, Huan Jing Ke Xue, V37, P1938"
" Long JM, 2018, HUM ECOL RISK ASSESS, V24, P679, DOI 10.1080/10807039.2017.1396531"
" Loukidou MX, 2003, WATER RES, V37, P4544, DOI 10.1016/S0043-1354(03)00415-9"
" Ma YS, 2018, CHEM ENG J, V353, P410, DOI 10.1016/j.cej.2018.07.131"
" Ministry of Ecology and Environment, 2021, RES EC ENV PROT, V3"
" Mitra S, 2022, J KING SAUD UNIV SCI, V34, DOI 10.1016/j.jksus.2022.101865"
" Munir N, 2022, SUSTAINABILITY-BASEL, V14, DOI 10.3390/su14010161"
" Nasraoui R, 2022, ENVIRON EARTH SCI, V81, DOI 10.1007/s12665-022-10610-7"
" Newsome L, 2021, GEOHEALTH, V5, DOI 10.1029/2020GH000380"
" Oyeyiola AO, 2011, ENVIRON MONIT ASSESS, V172, P319, DOI 10.1007/s10661-010-1336-4"
" Peng L.I.U., 2019, Acta Pedol. Sin., V56, P1048, DOI DOI 10.11766/TRXB201812130538"
" Qian QJJBNXXRHYCXQJYYL., 2021, SCI TECHNOL HUM VAL, V44, P132, DOI [10.19672/j.cnki.1003-6504.2209.20.338, DOI 10.19672/J.CNKI.1003-6504.2209.20.338]"
" Qian T., 2020, GUANGZHOU CHEM IND, V48, P116"
" Qin Jiaming, 2021, IOP Conference Series: Earth and Environmental Science, V687, DOI [10.1088/1755-1315/687/1/012041, 10.1088/1755-1315/687/1/012041]"
" Qiu KY, 2016, ATMOS POLLUT RES, V7, P170, DOI 10.1016/j.apr.2015.09.003"
" Riaz M, 2021, J HAZARD MATER, V402, DOI 10.1016/j.jhazmat.2020.123919"
" Rodr??guez L, 2009, J ENVIRON MANAGE, V90, P1106, DOI 10.1016/j.jenvman.2008.04.007"
" Romero-Freire A, 2016, GEODERMA, V263, P132, DOI 10.1016/j.geoderma.2015.09.006"
" [????????? Ruan Yulong], 2015, [???????????????, Earth and Environment], V43, P92"
" Sapkota B, 2012, ENVIRON POLLUT, V170, P131, DOI 10.1016/j.envpol.2012.06.010"
" Schlutow A, 2021, ENVIRON SCI EUR, V33, DOI 10.1186/s12302-020-00391-w"
" Shahid M, 2017, J HAZARD MATER, V325, P36, DOI 10.1016/j.jhazmat.2016.11.063"
" Shasha X, 2016, THESIS"
" Shuguang YJBLJYZ., 2022, J SHANDONG U TECHNOL, V36, P7"
" Tahmasebi P, 2020, B ENG GEOL ENVIRON, V79, P1113, DOI 10.1007/s10064-019-01574-3"
" Tarfeen N, 2022, PROCESSES, V10, DOI 10.3390/pr10071358"
" Tuo Jin, 2021, IOP Conference Series: Earth and Environmental Science, V687, DOI [10.1088/1755-1315/687/1/012012, 10.1088/1755-1315/687/1/012012]"
" Pham VHT, 2022, MICROORGANISMS, V10, DOI 10.3390/microorganisms10030610"
" Vendruscolo F, 2017, INT BIODETER BIODEGR, V119, P87, DOI 10.1016/j.ibiod.2016.10.008"
" Verma S, 2023, CLEAN TECHNOL ENVIR, V25, P1485, DOI 10.1007/s10098-021-02029-8"
" Wang A, 2022, J HAZARD MATER, V429, DOI 10.1016/j.jhazmat.2022.128396"
" Wei DQHQLDHFZ., 2018, SCI TECHNOL HUM VAL, V41, P56, DOI [10.19672/j.cnki.1003-6504.2018.03.009, DOI 10.19672/J.CNKI.1003-6504.2018.03.009]"
" Weiner S, 2003, REV MINERAL GEOCHEM, V54, P1, DOI 10.2113/0540001"
" Wenqing NXC., 2015, SICHUAN CHEM IND, V18, P18"
" Wu CY, 2022, J HAZARD MATER, V429, DOI 10.1016/j.jhazmat.2022.128297"
" Wu HY, 2020, INT J ENVIRON HEAL R, V30, P174, DOI 10.1080/09603123.2019.1584666"
" Wu Y, 2017, APPL GEOCHEM, V77, P44, DOI 10.1016/j.apgeochem.2016.05.012"
" Wu YF, 2022, RESOUR CONSERV RECY, V181, DOI 10.1016/j.resconrec.2022.106261"

"   Wyszkowska J, 2008, J ELEMENTOL, V13, P443"
"   Xu JY, 2019, J SOIL SEDIMENT, V19, P3608, DOI 10.1007/s11368-019-02341-3"
"   Xu L, 2021, CHEMOSPHERE, V285, DOI 10.1016/j.chemosphere.2021.131350"
"   Xu Yanna NM., 2013, RESOUR EC ENV PROT, V02, P55, DOI [10.16317/j.cnki.12-1377/x.2013.02.036, DOI 10.16317/J.CNKI.12-1377/X.2013.02.036]"
"   [????????? XU Youning], 2008, [???????€????, Geological Bulletin of China], V27, P1272"
"   Xu ZN, 2021, CHEMOSPHERE, V273, DOI 10.1016/j.chemosphere.2020.129496"
"   Xue SG, 2023, J ENVIRON SCI, V127, P780, DOI 10.1016/j.jes.2022.07.009"
"   Xue ZF, 2022, J HAZARD MATER, V426, DOI 10.1016/j.jhazmat.2021.128090"
"   Yan X., 2019, EARTH ENV SCI UK, V401, P012009, DOI [10.1088/1755-1315/401/1/012009, DOI 10.1088/1755-1315/401/1/012009]"
"   Yang JY, 2016, GEOMICROBIOL J, V33, P261, DOI 10.1080/01490451.2015.1068889"
"   Yang J, 2015, ENVIRON SCI POLLUT R, V22, P14710, DOI 10.1007/s11356-015-4601-9"
"   Yang Y, 2020, INT J ENVIRON SCI TE, V17, P2477, DOI 10.1007/s13762-020-02668-2"
"   Ye SJ, 2017, CRIT REV BIOTECHNOL, V37, P1062, DOI 10.1080/07388551.2017.1304357"
"   Youhua BGTRYQHHLDM., 2022, RES PROGR CHIN AGR S, V38, P69"
"   Yu EJ, 2022, FRONT ENV SCI-SWITZ, V10, DOI 10.3389/fenvs.2022.777894"
"   Zamulina IV, 2021, ECOTOX ENVIRON SAFE, V208, DOI 10.1016/j.ecoenv.2020.111471"
"   Zhan T. L., 2017, CHIN J SOIL SCI, V48, P474, DOI [10.19336/j.cnki.trtb.2017.02.32, DOI 10.19336/J.CNKI.TRTB.2017.02.32]"
"   Zhang C, 2016, SCI TOTAL ENVIRON, V557, P785, DOI 10.1016/j.scitotenv.2016.01.170"
"   Zhang HY, 2020, CHEM ENG J, V398, DOI 10.1016/j.cej.2020.125657"
"   Zhang SL, 2020, SUSTAINABILITY-BASEL, V12, DOI 10.3390/su12051868"
"   Zhang XM, 2022, SCI TOTAL ENVIRON, V806, DOI 10.1016/j.scitotenv.2021.151302"
"   Zhang Y, 2021, CHEMOSPHERE, V281, DOI 10.1016/j.chemosphere.2021.130908"
"   Zhao XQ, 2020, ENVIRON SCI POLLUT R, V27, P20215, DOI 10.1007/s11356-020-08538-1"
"   Zheng WK, 2022, ENVIRON CHEM LETT, V20, P2043, DOI 10.1007/s10311-022-01407-y"
"   Zhong X, 2020, J HAZARD MATER, V400, DOI 10.1016/j.jhazmat.2020.123289"
"   Ziwei L, 2021, THESIS"
NR 113
TC 21
Z9 25
U1 121
U2 442
PU SPRINGER BASEL AG
PI BASEL
"PA PICASSOPLATZ 4, BASEL, 4052, SWITZERLAND"
SN 0937-7409
EI 1423-0445
J9 CHEMOECOLOGY
JI Chemoecology
PD JUN
PY 2024
VL 34
IS 2
BP 47
EP 59
DI 10.1007/s00049-024-00399-1
EA APR 2024
PG 13
WC Biochemistry & Molecular Biology; Ecology
WE Science Citation Index Expanded (SCI-EXPANDED)
SC Biochemistry & Molecular Biology; Environmental Sciences & Ecology
GA UY2J0
UT WOS:001207636000001
DA 2026-04-06
ER

PT J
"AU Gao, TP"
"   Liu, Y"
"   Yang, D"
"   Liu, XX"
"   Zuo, MB"
"   He, YQ"
"   Wang, HM"
"   Bao, JT"
"   Shen, YY"
"   Tai, XS"
"   Xu, J"
"   Xu, DH"
"AF Gao, Tianpeng"
"   Liu, Yuan"
"   Yang, Deng"
"   Liu, Xiaoxiao"
"   Zuo, Mingbo"
"   He, Yueqing"
"   Wang, Haoming"
"   Bao, Jingting"
"   Shen, Yuanyuan"
"   Tai, Xisheng"
"   Xu, Jing"
"   Xu, Danghui"
TI Inoculation of Exogenous Complex Bacteria to Enhance Resistance in

Alfalfa and Combined Remediation of Heavy Metal-Contaminated Soil
SO CURRENT MICROBIOLOGY
LA English
DT Article
ID PLANT-GROWTH; ENZYME-ACTIVITY; ACCUMULATION; BIOCHAR; COPPER; FRACTIONS;
TOXICITY; BIOMASS; STRESS; CROPS
"AB Heavy metals are considered to be one of the main sources of soil contamination. In this study, three tolerant bacteria were isolated from the heavy metal-contaminated soil in mining area, and immobilized bacteria were constructed using corn straw as the carrier. The combined remediation effect of immobilized bacteria and alfalfa in pot experiments was explored in heavy metal-contaminated soil. Under heavy metal stress, inoculation with immobilized bacteria significantly promoted the growth of alfalfa, in which the dry weights of roots, stems, and leaves increased by 19.8, 6.89, and 14.6%, respectively ($P < 0.05$). Also, inoculation with immobilized bacteria improved the antioxidant capacity of plants and the activity of soil enzymes and improved soil quality ($P < 0.05$). Microbial-phytoremediation technology effectively reduced the heavy metal content in the soil, and can restore the soil contaminated by heavy metals. The results will help to further understand the mechanism of microbial inoculation to reduce the toxicity of heavy metals, and provide guidance for the cultivation of forage grasses in heavy metal-contaminated soils."
[GRAPHICS]
.
"C1 [Gao, Tianpeng; Liu, Yuan; Liu, Xiaoxiao; Zuo, Mingbo; Bao, Jingting; Shen, Yuanyuan; Xu, Jing] Xian Univ, Sch Biol & Environm Engn, Xian 710000, Peoples R China."
" [Yang, Deng; He, Yueqing; Wang, Haoming] Lanzhou Jiaotong Univ, Sch Biol & Pharmaceut Engn, Lanzhou 730070, Peoples R China."
" [Gao, Tianpeng; Tai, Xisheng] Lanzhou City Univ, Engn Ctr Pollut Control & Ecol Restorat Min Gansu, Lanzhou 730070, Peoples R China."
" [Bao, Jingting] Lanzhou Univ Technol, Sch Life Sci & Engn, Lanzhou 730050, Peoples R China."
" [Xu, Danghui] Lanzhou Univ, Coll Ecol, State Key Lab Grassland Agroecosystems, Lanzhou 730000, Peoples R China."
C3 Xi'an University; Lanzhou Jiaotong University; Lanzhou City University;
Lanzhou University of Technology; Lanzhou University
"RP Gao, TP (corresponding author), Xian Univ, Sch Biol & Environm Engn, Xian 710000, Peoples R China.; Gao, TP (corresponding author), Lanzhou City Univ, Engn Ctr Pollut Control & Ecol Restorat Min Gansu, Lanzhou 730070, Peoples R China."
EM zkgtp@163.com
"RI Gao, Tianpeng/ABF-7377-2021"
"OI Gao, Tianpeng/0000-0001-6821-3836"
"FU National Natural Science Foundation of China [NSFC 31860176, 32171611,"
32101415]; Key Research and Development Program of Gansu Province
" [20YF3FA037, 22JR5RA454, 22JR5RA262]; Key Research and Development"
" Program of Shanxi Province [2020ZDLSF06-06, 2021JQ-791, XAWLKYTD012]"
FX This work was supported by the National Natural Science Foundation of
" China (Grant numbers NSFC 31860176, 32171611, and 32101415), Key"
" Research and Development Program of Gansu Province (20YF3FA037,"
" 22JR5RA454, and 22JR5RA262), Key Research and Development Program of"
" Shanxi Province (2020ZDLSF06-06, 2021JQ-791, and XAWLKYTD012). We are"
grateful to all reviewers whose comments improved the quality of the
manuscript.
"CR Ab Aziz NSB, 2015, WORLD CONFERENCE ON TECHNOLOGY, INNOVATION AND ENTREPRENEURSHIP, P2457, DOI 10.1016/j.sbspro.2015.06.288"
" Abd-Alla MH, 2012, INT BIODETER BIODEGR, V67, P48, DOI 10.1016/j.ibiod.2011.10.008"
" Ali H, 2013, CHEMOSPHERE, V91, P869, DOI 10.1016/j.chemosphere.2013.01.075"
" Aponte H, 2020, SCI TOTAL ENVIRON, V737, DOI 10.1016/j.scitotenv.2020.139744"
" Bilal S, 2020, IND CROP PROD, V143, DOI 10.1016/j.indcrop.2019.111931"
" Burd GI, 1998, APPL ENVIRON MICROB, V64, P3663"
" Chatterjee P, 2017, FRONT PLANT SCI, V8, DOI 10.3389/fpls.2017.00705"
" Chen YX, 2005, ENVIRON INT, V31, P861, DOI 10.1016/j.envint.2005.05.044"
" Cui HB, 2017, RSC ADV, V7, P45869, DOI 10.1039/c7ra08786a"
" Etesami H, 2018, ECOTOX ENVIRON SAFE, V147, P175, DOI 10.1016/j.ecoenv.2017.08.032"
" Gasc?? G, 2016, ECOL ENG, V95, P19, DOI 10.1016/j.ecoleng.2016.06.039"
" Ge TD, 2011, J SOIL SEDIMENT, V11, P25, DOI 10.1007/s11368-010-0293-4"
" Grant JJ, 2000, PLANT PHYSIOL, V124, P21, DOI 10.1104/pp.124.1.21"
" Huang Q, 2000, SOIL BIOL BIOCHEM, V32, P1885, DOI 10.1016/S0038-0717(00)00162-0"
" Jia XY, 2020, GEODERMA, V366, DOI 10.1016/j.geoderma.2020.114256"
" Ju WL, 2020, APPL SOIL ECOL, V150, DOI 10.1016/j.apsoil.2019.103450"
" Karn SK, 2017, ENVIRON ENG SCI, V34, P158, DOI 10.1089/ees.2015.0390"
" Kumar A, 2021, MICROBIOL RES, V242, DOI 10.1016/j.micres.2020.126616"
" Li BH, 2021, SCIENCEASIA, V47, P366, DOI 10.2306/scienceasia1513-1874.2021.047"
" Li Y, 2019, ECOTOX ENVIRON SAFE, V184, DOI 10.1016/j.ecoenv.2019.109603"
" Li ZS, 2022, SCI REP-UK, V12, DOI 10.1038/s41598-022-19388-x"
" Lin H, 2021, J HAZARD MATER, V402, DOI 10.1016/j.jhazmat.2020.123829"
" Liu Y, 2022, RES REMEDIATION HEAV, DOI [10.27205/d.cnki.gltec.2022.000363, DOI 10.27205/D.CNKI.GLTEC.2022.000363]"
" Ma Y, 2016, J ENVIRON MANAGE, V174, P14, DOI 10.1016/j.jenvman.2016.02.047"
" Ma Y, 2009, J HAZARD MATER, V166, P1154, DOI 10.1016/j.jhazmat.2008.12.018"
" Medina CA, 2020, INT J MOL SCI, V21, DOI 10.3390/ijms21093361"
" Miralles J, 2006, MAR POLLUT BULL, V52, P1364, DOI 10.1016/j.marpolbul.2006.03.018"
" Mittler R, 2002, TRENDS PLANT SCI, V7, P405, DOI 10.1016/S1360-1385(02)02312-9"
" Nannipieri P, 2003, EUR J SOIL SCI, V54, P655, DOI 10.1046/j.1351-0754.2003.0556.x"
" Naveed M, 2020, PLANTS-BASEL, V9, DOI 10.3390/plants9040530"
" Ojuederie OB, 2017, INT J ENV RES PUB HE, V14, DOI 10.3390/ijerph14121504"
" Qi RM, 2016, APPL SOIL ECOL, V102, P36, DOI 10.1016/j.apsoil.2016.02.004"
" Rizwan M, 2016, ENVIRON SCI POLLUT R, V23, P2230, DOI 10.1007/s11356-015-5697-7"
" Sharma RK, 2016, APPL SOIL ECOL, V107, P66, DOI 10.1016/j.apsoil.2016.05.009"
" Takarina ND, 2017, MAKARA J SCI, V21, P78, DOI 10.7454/mss.v21i2.7308"

"   Tu C, 2020, ENVIRON INT, V137, DOI 10.1016/j.envint.2020.105576"
"   Wang Q, 2016, AGR ECOSYST ENVIRON, V228, P9, DOI 10.1016/j.agee.2016.05.006"
"   Wang XY, 2022, SCI TOTAL ENVIRON, V819, DOI 10.1016/j.scitotenv.2022.153242"
"   Willekens H, 1997, EMBO J, V16, P4806, DOI 10.1093/emboj/16.16.4806"
"   Wu YJ, 2020, J HAZARD MATER, V395, DOI 10.1016/j.jhazmat.2020.122661"
"   Xu ZN, 2021, CHEMOSPHERE, V273, DOI 10.1016/j.chemosphere.2020.129496"
"   Yavari S, 2015, ENVIRON SCI POLLUT R, V22, P13824, DOI 10.1007/s11356-015-5114-2"
"   Yoon J, 2006, SCI TOTAL ENVIRON, V368, P456, DOI 10.1016/j.scitotenv.2006.01.016"
"   Yu FM, 2021, PLANT SOIL, V459, P155, DOI 10.1007/s11104-020-04752-5"
"   Zhang QM, 2014, ENVIRON MONIT ASSESS, V186, P2801, DOI 10.1007/s10661-013-3581-9"
NR 45
TC 9
Z9 9
U1 11
U2 92
PU SPRINGER
PI NEW YORK
"PA ONE NEW YORK PLAZA, SUITE 4600, NEW YORK, NY, UNITED STATES"
SN 0343-8651
EI 1432-0991
J9 CURR MICROBIOL
JI Curr. Microbiol.
PD JUN
PY 2023
VL 80
IS 6
AR 213
DI 10.1007/s00284-023-03299-9
PG 11
WC Microbiology
WE Science Citation Index Expanded (SCI-EXPANDED)
SC Microbiology
GA G8ZG8
UT WOS:000991966800003
PM 37191724
DA 2026-04-06
ER

PT J
"AU Garg, A"
"   Chauhan, P"
"   Kaur, C"
"   Arora, PK"
"   Garg, SK"
"   Singh, VP"
"   Singh, KP"
"   Srivastava, A"
"AF Garg, Akansha"
"   Chauhan, Prerna"
"   Kaur, Charanjeet"
"   Arora, Pankaj Kumar"
"   Garg, Sanjay Kumar"
"   Singh, Vijay Pal"
"   Singh, Krishna Pal"
"   Srivastava, Alok"
TI Comprehensive heavy metal remediation mechanisms with insights into
   CRISPR-Cas9 and biochar innovations
SO BIODEGRADATION
LA English
DT Review
DE Phytoremediation; Microbial remediation; CRISPR-Cas9; Biochar;
   Detoxification
ID GROWTH-PROMOTING BACTERIA; INDUCED OXIDATIVE STRESS; PLANT-GROWTH;
   CONFERS RESISTANCE; CONTAMINATED SOIL; ENHANCED PHYTOREMEDIATION; AIDED
   PHYTOSTABILIZATION; HYPERACCUMULATING PLANTS; SACCHAROMYCES-CEREVISIAE;
   METALLOTHIONEIN GENES
"AB Heavy metal contamination of the environment is a serious issue, and more efficient and effective bioremediation techniques are needed. This review introduces current heavy metal bioremediation techniques, with focus on phytoremediation and microbial remediation, and recent developments in biochar and CRISPR-Cas9 technology. Phytoremediation employs the natural process of plants to accumulate and detoxify metals as an eco-friendly and sustainable technique. Microbial remediation by fungi and bacteria provides an additional approach through reduction, sequestration, and transformation of metals. Biochar as a high-carbon value-added pyrolytic biomass product improves soil quality, increases microbial activity, and adsorbs heavy metals, making bioremediation more effective. The discovery of CRISPR-Cas9 revolutionized gene engineering by allowing gene editing of plants and microbes to improve their metal tolerance and degradation. This review outlines recent developments, synergistic uses of biochar and CRISPR-Cas9, and how they might enhance phytoremediation and microbial remediation. By combining such novel technologies, strong, sustainable, and scalable solutions could be built for curbing heavy metal pollution and safeguarding environmental health."
"C1 [Garg, Akansha; Chauhan, Prerna; Arora, Pankaj Kumar; Garg, Sanjay Kumar; Singh, Vijay Pal; Srivastava, Alok] MJP Rohilkhand Univ, Dept Plant Sci, Bareilly 243006, India."
"   [Kaur, Charanjeet] MJP Rohilkhand Univ, Dept Microbiol, Bareilly 243006, India."
"   [Singh, Krishna Pal] MJP Rohilkhand Univ, Bareilly 243006, India."
C3 Mahatma Jyotiba Phule Rohilkhand University; Mahatma Jyotiba Phule
   Rohilkhand University; Mahatma Jyotiba Phule Rohilkhand University

"RP Srivastava, A (corresponding author), MJP Rohilkhand Univ, Dept Plant Sci, Bareilly 243006, India."
EM gargiakansha1999@gmail.com; prernachauhan7417@gmail.com;
charannbri@gmail.com; arora484@gmail.com; gargskplantscience@gmail.com;
singhvp03@rediffmail.com; vcoffice@mjpru.ac.in;
alokplantscience@gmail.com
"RI Arora, Pankaj Kumar/F-9418-2014; KUMAR, SANJAY/IYS-9415-2023"
"OI Arora, Pankaj Kumar/0000-0002-4106-8859; "
FU DST-INSPIRE INDIA; DST
"FX The author A.G. is thankful to DST for awarding INSPIRE Fellowship,"
"   authors are thankful to department of Plant Science, MJPRU Bareilly for"
providing facility. Authors are thankful to ChatGPT and Mendeley for
improving the language and reference setup in the preparation of
manuscript.
"CR Acosta JA, 2018, CHEMOSPHERE, V204, P71, DOI 10.1016/j.chemosphere.2018.04.027"
"   Adetunji C. O., 2021, Microbial Rejuvenation of Polluted Environment, V3, P303, DOI [10.1007/978-981-15-7459-7_14, DOI 10.1007/978-981-15-7459-7_14]"
"   Adle DJ, 2007, J BIOL CHEM, V282, P947, DOI 10.1074/jbc.M609535200"
"   Afzal I, 2024, SCI REP-UK, V14, DOI 10.1038/s41598-024-82138-8"
"   Agegnehu G, 2017, APPL SOIL ECOL, V119, P156, DOI 10.1016/j.apsoil.2017.06.008"
"   Ahmed E, 2014, MICROB BIOTECHNOL, V7, P196, DOI 10.1111/1751-7915.12117"
"   Ahmed SF, 2022, ENVIRON TECHNOL INNO, V25, DOI 10.1016/j.eti.2021.102114"
"   Akhtar Nahid, 2020, Biotechnology Reports, V26, pe00452, DOI [10.1016/j.btre.2020.e00452, 10.1016/j.btre.2020.e00452]"
"   Al-Sulaiti MM, 2022, CURR POLLUT REP, V8, P249, DOI 10.1007/s40726-022-00226-7"
"   Alabssawy AN, 2024, ARCH MICROBIOL, V206, DOI 10.1007/s00203-023-03793-5"
"   Algahmadi A, 2024, ACS OMEGA, V9, P26245, DOI 10.1021/acsomega.4c02006"
"   Ali H, 2013, CHEMOSPHERE, V91, P869, DOI 10.1016/j.chemosphere.2013.01.075"
"   Ali Q, 2023, Hybrid Advances, V4, P100091, DOI [10.1016/j.hybadv.2023.100091, 10.1016/j.hybadv.2023.100091, DOI 10.1016/J.HYBADV.2023.100091]"
"   Ali Z, 2015, GENOME BIOL, V16, DOI 10.1186/s13059-015-0799-6"
"   Allamin IA, 2020, SCI REP-UK, V10, DOI 10.1038/s41598-020-60668-1"
"   Allen D, 2021, FRONT GENOME EDIT, V2, DOI 10.3389/fgeed.2020.617910"
"   Alotaibi BS, 2021, MICROORGANISMS, V9, DOI 10.3390/microorganisms9081628"
"   Altamirano-Lozano M, 2006, IARC Monographs on the Evaluation of Carcinogenic Risks to Humans,, V86"
"   Aman R, 2018, GENOME BIOL, V19, DOI 10.1186/s13059-017-1381-1"
"   Ambaye TG, 2022, CHEMOSPHERE, V293, DOI 10.1016/j.chemosphere.2022.133572"
"   Ambika S, 2022, CHEM ENG J, V439, DOI 10.1016/j.cej.2022.135716"
"   Andrade AC, 2000, MOL GEN GENET, V263, P966, DOI 10.1007/PL00008697"
"   Angulo-Bejarano PI, 2021, PLANTS-BASEL, V10, DOI 10.3390/plants10040635"
"   Ankit, 2022, HYDROBIOLOGY-BASEL, V1, P288, DOI [10.3390/hydrobiology1030021, 10.3390/hydrobiology1030021]"
"   [Anonymous], 1990, IARC Monogr Eval Carcinog Risks Hum, V49, P1"
"   [Anonymous], 2005, Mercury in Drinking-water: Background document for development of WHO Guidelines for Drinking-water Quality"
"   Anton A, 2004, J BACTERIOL, V186, P7499, DOI 10.1128/JB.186.22.7499-7507.2004"
"   Aransiola SA., 2021, Int J Environ Plan Manag, V7, P102"
"   Arazi T, 1999, PLANT J, V20, P171, DOI 10.1046/j.1365-313x.1999.00588.x"
"   Arisah FM, 2021, SUSTAINABILITY-BASEL, V13, DOI 10.3390/su13179797"
"   Arora PK, 2021, DATA BRIEF, V34, DOI 10.1016/j.dib.2020.106640"
"   Auguy F, 2016, FRONT PLANT SCI, V6, DOI 10.3389/fpls.2015.01231"
"   Ayangbenro AS, 2017, INT J ENV RES PUB HE, V14, DOI 10.3390/ijerph14010094"
"   Ayilara MS, 2023, FRONT AGRON, V5, DOI 10.3389/fagro.2023.1183691"
"   Azevedo BF, 2012, J BIOMED BIOTECHNOL, DOI 10.1155/2012/949048"
"   Aziz KHH, 2023, RSC ADV, V13, P17595, DOI 10.1039/d3ra00723e"
"   Bala S, 2022, TOXICS, V10, DOI 10.3390/toxics10080484"
"   Baldiris R, 2018, MOLECULES, V23, DOI 10.3390/molecules23020406"
"   Baltes NJ, 2015, NAT PLANTS, V1, DOI [10.1038/nplants.2015.145, 10.1038/NPLANTS.2015.145]"
"   Banerjee Aditya, 2022, Vegetos, V35, P1158, DOI [10.1007/s42535-022-00405-3, 10.1007/s42535-022-00405-3]"
"   Bano A, 2018, CHEMOSPHERE, V199, P218, DOI 10.1016/j.chemosphere.2018.02.043"
"   Barbosa B., 2018, Bio-Geotechnologies for Mine Site Rehabilitation, P147, DOI DOI 10.1016/B978-0-12-812986-9.00009-9"
"   Basharat Z, 2018, PLANTS-BASEL, V7, DOI 10.3390/plants7030051"
"   Begum N, 2019, PLANTS-BASEL, V8, DOI 10.3390/plants8110504"
"   Bentley R, 2002, MICROBIOL MOL BIOL R, V66, P250, DOI 10.1128/MMBR.66.2.250-271.2002"
"   Bhat SA, 2022, CHEMOSPHERE, V303, DOI 10.1016/j.chemosphere.2022.134788"
"   Bhattacharyya N, 2023, ENVIRON CHEM LETT, V21, P429, DOI 10.1007/s10311-022-01474-1"
"   Bizily SP, 1999, P NATL ACAD SCI USA, V96, P6808, DOI 10.1073/pnas.96.12.6808"
"   Bizily SP, 2000, NAT BIOTECHNOL, V18, P213, DOI 10.1038/72678"
"   Bleackley MR, 2011, BIOMETALS, V24, P785, DOI 10.1007/s10534-011-9451-4"
"   Bolan NS, 2011, ADV AGRON, V112, P145, DOI 10.1016/B978-0-12-385538-1.00004-4"
"   Bortoloti GA, 2022, Environmental Advances, V8, P100204, DOI [10.1016/j.envadv.2022.100204, 10.1016/j.envadv.2022.100204, DOI 10.1016/J.ENVADV.2022.100204]"
"   Braud A, 2009, CHEMOSPHERE, V74, P280, DOI 10.1016/j.chemosphere.2008.09.013"
"   Bravo D, 2022, LETT APPL MICROBIOL, V74, P311, DOI 10.1111/lam.13594"
"   Briffa J, 2020, HELIYON, V6, DOI 10.1016/j.heliyon.2020.e04691"
"   Briskine RV, 2017, MOL ECOL RESOUR, V17, P1025, DOI 10.1111/1755-0998.12604"
"   Burd GI, 1998, APPL ENVIRON MICROB, V64, P3663"
"   Burd GI, 2000, CAN J MICROBIOL, V46, P237, DOI 10.1139/cjm-46-3-237"
"   Cameselle C, 2019, CHEMOSPHERE, V229, P481, DOI 10.1016/j.chemosphere.2019.04.222"
"   Cameselle C, 2013, CHEMOSPHERE, V93, P626, DOI 10.1016/j.chemosphere.2013.06.029"
"   Campos P, 2020, SOIL BIOL BIOCHEM, V150, DOI 10.1016/j.soilbio.2020.108014"
"   Chandrasekaran J, 2016, MOL PLANT PATHOL, V17, P1140, DOI 10.1111/mpp.12375"
"   Chaturvedi K, 2024, BIOMASS CONVERS BIOR, V14, P17041, DOI 10.1007/s13399-022-03715-3"
"   Chauhan P, 2020, ENVIRON SCI POLLUT R, V27, P29954, DOI 10.1007/s11356-020-09233-x"

"   Chehelgerdi M, 2024, MOL CANCER, V23, DOI 10.1186/s12943-023-01925-5"
"   Chen HX, 2018, J ENVIRON SCI-CHINA, V70, P45, DOI 10.1016/j.jes.2017.11.007"
"   Chen L, 2014, SOIL BIOL BIOCHEM, V68, P300, DOI 10.1016/j.soilbio.2013.10.021"
"   Chen S, 2023, SUSTAINABILITY-BASEL, V15, DOI 10.3390/su152215863"
"   Chilcoat D, 2017, PROG MOL BIOL TRANSL, V149, P27, DOI 10.1016/bs.pmbts.2017.04.005"
"   Choinska-Pulit A, 2018, ECOTOX ENVIRON SAFE, V149, P275, DOI 10.1016/j.ecoenv.2017.12.008"
"   Chugh M, 2022, ENERGY NEXUS, V7, DOI 10.1016/j.nexus.2022.100129"
"   Comte S, 2008, J HAZARD MATER, V151, P185, DOI 10.1016/j.jhazmat.2007.05.070"
"   Costa F, 2017, INT BIODETER BIODEGR, V120, P115, DOI 10.1016/j.ibiod.2017.02.018"
"   CotterHowells J, 1996, APPL GEOCHEM, V11, P335, DOI 10.1016/0883-2927(95)00042-9"
"   Courbot M, 2004, APPL ENVIRON MICROB, V70, P7413, DOI 10.1128/AEM.70.12.7413-7417.2004"
"   Cristaldi A, 2020, ENVIRON RES, V185, DOI 10.1016/j.envres.2020.109427"
"   DalCorso G, 2019, INT J MOL SCI, V20, DOI 10.3390/ijms20143412"
"   Dalvi A.A., 2013, Annals of Plant Sci, V2, P362, DOI DOI 10.1155/2015/756120"
"   Damle MS, 2021, J BIOL CHEM, V297, DOI 10.1016/j.jbc.2021.101078"
"   Danouche M, 2021, HELIYON, V7, DOI 10.1016/j.heliyon.2021.e07609"
"   Daoud L, 2022, PLOS ONE, V17, DOI 10.1371/journal.pone.0277946"
"   de Figueiredo CC, 2021, WASTE MANAGE, V124, P144, DOI 10.1016/j.wasman.2021.01.044"
"   de Souza TD, 2019, CHEMOSPHERE, V234, P402, DOI 10.1016/j.chemosphere.2019.06.004"
"   Dell' Amico E., 2005, FEMS MICROBIOL ECOL, V52, P153"
"   Delmar JA, 2014, ANNU REV BIOPHYS, V43, P93, DOI 10.1146/annurev-biophys-051013-022855"
"   Deshmukh R, 2016, INDIAN J MICROBIOL, V56, P247, DOI 10.1007/s12088-016-0584-6"
"   Dey Uttiya, 2016, Biotechnol Rep (Amst), V10, P1, DOI [10.1016/j.btre.2016.02.002, 10.1016/j.btre.2016.02.002]"
"   Dhama K, 2021, ENVIRON SCI POLLUT R, V28, P34211, DOI 10.1007/s11356-021-14429-w"
"   Dhiman SS, 2017, PLOS ONE, V12, DOI 10.1371/journal.pone.0175845"
"   Di XR, 2019, INT J ENV RES PUB HE, V16, DOI 10.3390/ijerph16245012"
"   Dixit R, 2015, SUSTAINABILITY-BASEL, V7, P2189, DOI 10.3390/su7022189"
"   do Nascimento CWA, 2006, ENVIRON POLLUT, V140, P114, DOI 10.1016/j.envpol.2005.06.017"
"   Dolphen R, 2015, INT J PHYTOREMEDIAT, V17, P686, DOI 10.1080/15226514.2014.964839"
"   Dom??nguez MT, 2009, CHEMOSPHERE, V76, P480, DOI 10.1016/j.chemosphere.2009.03.026"
"   Doukyu N, 2021, AMB EXPRESS, V11, DOI 10.1186/s13568-021-01258-w"
"   Ducret V, 2020, FRONT MICROBIOL, V11, DOI 10.3389/fmicb.2020.00911"
"   Dur??n N, 2000, APPL CATAL B-ENVIRON, V28, P83, DOI 10.1016/S0926-3373(00)00168-5"
"   Egamberdiyeva D, 2004, J ARID ENVIRON, V56, P293, DOI 10.1016/S0140-1963(03)00050-8"
"   El Sayed MT, 2020, HELIYON, V6, DOI 10.1016/j.heliyon.2020.e03866"
"   El-Bassi L, 2021, SCI TOTAL ENVIRON, V755, DOI 10.1016/j.scitotenv.2020.142531"
"   El-Gendy MMAA, 2023, BIOMETALS, V36, P1307, DOI 10.1007/s10534-023-00520-x"
"   El-Naggar NEA, 2022, POLYMERS-BASEL, V14, DOI 10.3390/polym14071339"
"   El-Naggar NE, 2020, SCI REP-UK, V10, DOI 10.1038/s41598-020-70473-5"
"   Elahi A, 2022, SAUDI J BIOL SCI, V29, P2878, DOI 10.1016/j.sjbs.2022.01.027"
"   Elango D, 2022, J AGR FOOD RES, V10, DOI 10.1016/j.jafr.2022.100374"
"   Emamverdian Abolghassem, 2015, ScientificWorldJournal, V2015, P756120, DOI [10.1155/2015/756120, 10.1155/2015/756120]"
"   Emri T, 2021, MICROORGANISMS, V9, DOI 10.3390/microorganisms9071433"
"   Fan JX, 2021, 3 BIOTECH, V11, DOI 10.1007/s13205-021-02998-z"
"   Faramarzi MA, 2020, HYDROMETALLURGY, V191, DOI 10.1016/j.hydromet.2019.105228"
"   Fasani E, 2018, PLANT CELL ENVIRON, V41, P1201, DOI 10.1111/pce.12963"
"   Feng X, 2017, MICROBIOLOGYOPEN, V6, DOI 10.1002/mbo3.485"
"   Fijalkowska G, 2021, CHEMOSPHERE, V276, DOI 10.1016/j.chemosphere.2021.130088"
"   Firinca C, 2024, J XENOBIOTICS, V14, P51, DOI 10.3390/jox14010004"
"   Fischer F, 2016, PLOS PATHOG, V12, DOI 10.1371/journal.ppat.1006018"
"   Fomina M, 2014, BIORESOURCE TECHNOL, V160, P3, DOI 10.1016/j.biortech.2013.12.102"
"   Frankel ML, 2016, BIORESOURCE TECHNOL, V216, P352, DOI 10.1016/j.biortech.2016.05.084"
"   Franken GAC, 2022, CELL MOL LIFE SCI, V79, DOI 10.1007/s00018-022-04442-8"
"   Freedman Z, 2012, APPL ENVIRON MICROB, V78, P6568, DOI 10.1128/AEM.01060-12"
"   Gaballa A, 2021, REDOX BIOL, V42, DOI 10.1016/j.redox.2021.101935"
"   Genchi G, 2020, INT J ENV RES PUB HE, V17, DOI 10.3390/ijerph17113782"
"   Gerhardt KE, 2017, PLANT SCI, V256, P170, DOI 10.1016/j.plantsci.2016.11.016"
"   Gerwien F, 2018, FEMS MICROBIOL REV, V42, P1, DOI 10.1093/femsre/fux050"
"   Ghosh A, 2022, CURR RES MICROB SCI, V3, DOI 10.1016/j.crmicr.2021.100101"
"   Ghosh S, 2023, FRONT BIOENG BIOTECH, V11, DOI 10.3389/fbioe.2023.1106973"
"   Ginn BR, 2008, CHEM GEOL, V253, P130, DOI 10.1016/j.chemgeo.2008.05.001"
"   G??tz M, 2020, COATINGS, V10, DOI 10.3390/coatings10030218"
"   Gonz??lez A, 2021, FRONT PLANT SCI, V12, DOI 10.3389/fpls.2021.669096"
"   Grass G, 2002, J BACTERIOL, V184, P864, DOI 10.1128/JB.184.3.864-866.2002"
"   GRIFFIOEN WAJ, 1994, PLANT SOIL, V158, P83, DOI 10.1007/BF00007920"
"   Grimes RT, 2021, INORG CHEM, V60, P1229, DOI 10.1021/acs.inorgchem.0c03398"
"   Guo Q, 2017, ECOTOX ENVIRON SAFE, V139, P50, DOI 10.1016/j.ecoenv.2016.12.013"
"   Gupta A, 2021, MATERIALS, V14, DOI 10.3390/ma14164702"
"   Gupta DK, 2008, J ENVIRON BIOL, V29, P903"
"   Gupta Pratima, 2017, Biotechnol Rep (Amst), V13, P58, DOI [10.1016/j.btre.2016.12.006, 10.1016/j.btre.2016.12.006]"
"   Gupta P, 2018, SCI REP-UK, V8, DOI 10.1038/s41598-018-23322-5"
"   Gururajan K, 2018, ENVIRON TECHNOL INNO, V9, P91, DOI 10.1016/j.eti.2017.11.001"
"   Hall JL, 2002, J EXP BOT, V53, P1, DOI 10.1093/jexbot/53.366.1"
"   Han Y, 2021, MINERALS-BASEL, V11, DOI 10.3390/min11010041"
"   Hannink N, 2001, NAT BIOTECHNOL, V19, P1168, DOI 10.1038/nbt1201-1168"
"   Hasan MK, 2017, FRONT PLANT SCI, V8, DOI 10.3389/fpls.2017.01492"
"   Hattori H, 2006, SOIL SCI PLANT NUTR, V52, P89, DOI 10.1111/j.1747-0765.2006.00007.x"
"   He JL, 2013, PLANT PHYSIOL, V162, P424, DOI 10.1104/pp.113.215681"
"   He SY, 2012, ADV AGRON, V117, P117, DOI 10.1016/B978-0-12-394278-4.00003-9"
"   He SX, 2020, ENVIRON INT, V141, DOI 10.1016/j.envint.2020.105799"
"   He YX, 2019, CHEMOSPHERE, V221, P793, DOI 10.1016/j.chemosphere.2019.01.039"

"   Hecel A, 2020, INORG CHEM, V59, P1947, DOI 10.1021/acs.inorgchem.9b03298"
"   Hegazy GE, 2023, SCI REP-UK, V13, DOI 10.1038/s41598-023-29456-5"
"   Heidari P, 2020, INT J ENV RES PUB HE, V17, DOI 10.3390/ijerph17114059"
"   Hejna M, 2021, Heavy-Metal Phytoremediation from Livestock Wastewater and Exploitation of Exhausted Biomass"
"   Helbig K, 2008, J BACTERIOL, V190, P5439, DOI 10.1128/JB.00272-08"
"   Hern??ndez-Allica J, 2008, ENVIRON POLLUT, V152, P32, DOI 10.1016/j.envpol.2007.06.002"
"   Hlihor RM, 2017, WATER SCI TECHNOL, V76, P1726, DOI 10.2166/wst.2017.360"
"   Hlozkov?? K, 2016, FUNGAL BIOL-UK, V120, P358, DOI 10.1016/j.funbio.2015.11.007"
"   HOLM HW, 1975, APPL MICROBIOL, V29, P491, DOI 10.1128/AEM.29.4.491-494.1975"
"   Hossain MA., 2012, APPL ENVIRON SOIL SC, V2012, DOI [DOI 10.1155/2012/650206, 10.1155/2012/872875, DOI 10.1155/2012/872875]"
"   Houben D, 2015, CHEMOSPHERE, V139, P644, DOI 10.1016/j.chemosphere.2014.12.036"
"   Huang C, 2017, CHEMOSPHERE, V187, P70, DOI 10.1016/j.chemosphere.2017.08.104"
"   Huang HG, 2012, ENVIRON SCI POLLUT R, V19, P1640, DOI 10.1007/s11356-011-0675-1"
"   Hussein KA, 2011, J GEN APPL MICROBIOL, V57, P347"
"   Hyre Amanda, 2021, EcoSal Plus, V9, peESP00142020, DOI [10.1128/ecosalplus.esp-0014-2020, 10.1128/ecosalplus.ESP-0014-2020]"
"   IARC, 2014, IARC Monogr Eval Carcinog Risks Hum, V100F, P423"
"   IARC, 1993, GLASS, V58, P119"
"   IARC Working Group on the Evaluation of Carcinogenic Risks to Humans, 2012, IARC Monogr Eval Carcinog Risks Hum, V100, P11"
"   Ibn Ferjani A, 2019, RENEW SUST ENERG REV, V107, P425, DOI 10.1016/j.rser.2019.03.034"
"   Ighalo JO, 2022, CHEM ENG RES DES, V184, P419, DOI 10.1016/j.cherd.2022.06.028"
"   Igiri BE, 2018, J TOXICOL-US, V2018, DOI 10.1155/2018/2568038"
"   Ignatius A, 2014, ENVIRON SCI POLLUT R, V21, P13007, DOI 10.1007/s11356-014-3204-1"
"   Ilias M, 2011, INDIAN J MICROBIOL, V51, P76, DOI 10.1007/s12088-011-0095-4"
"   International Agency for Research on Cancer (IARC), 2023, IARC Monographs on the Identification of Carcinogenic Hazards to Humans, V131"
"   Iram S, 2015, ARAB J SCI ENG, V40, P1867, DOI 10.1007/s13369-015-1702-1"
"   ISHINO Y, 1987, J BACTERIOL, V169, P5429, DOI 10.1128/jb.169.12.5429-5433.1987"
"   Ishino Y, 2018, J BACTERIOL, V200, DOI 10.1128/JB.00580-17"
"   Islam MA, 2015, Progressive Agriculture, V26, P97, DOI [10.3329/pa.v26i2.25962, 10.3329/pa.v26i2.25962, DOI 10.3329/PA.V26I2.25962]"
"   Jacob JM, 2018, J ENVIRON MANAGE, V217, P56, DOI 10.1016/j.jenvman.2018.03.077"
"   Jaishankar Monisha, 2014, Interdiscip Toxicol, V7, P60, DOI 10.2478/intox-2014-0009"
"   Jawed Kamran, 2019, Metab Eng Commun, V9, pe00095, DOI 10.1016/j.mec.2019.e00095"
"   Legorreta-Casta??eda AJ, 2020, WATER-SUI, V12, DOI 10.3390/w12041155"
"   Jebril Nadia, 2022, IOP Conference Series: Earth and Environmental Science, V1002, DOI [10.1088/1755-1315/1002/1/012006, 10.1088/1755-1315/1002/1/012006]"
"   Jeyakumar P, 2023, ENVIRON ENG RES, V28, DOI 10.4491/eer.2021.631"
"   Ji X, 2015, NAT PLANTS, V1, DOI [10.1038/NPLANTS.2015.144, 10.1038/nplants.2015.144]"
"   Jia HG, 2017, PLANT BIOTECHNOL J, V15, P817, DOI 10.1111/pbi.12677"
"   Jiang SH, 2013, MICROBES ENVIRON, V28, P312, DOI 10.1264/jsme2.ME12149"
"   Jing Yan-De, 2007, Journal of Zhejiang University-Science B, V8, P192, DOI 10.1631/jzus.2007.B0192"
"   Joshi S, 2023, FRONT MICROBIOL, V14, DOI 10.3389/fmicb.2023.1229828"
"   Jozefczak M, 2012, INT J MOL SCI, V13, P3145, DOI 10.3390/ijms13033145"
"   Kafle A, 2022, Environmental Advances, V8, P100203, DOI [10.1016/j.envadv.2022.100203, 10.1016/j.envadv.2022.100203, DOI 10.1016/J.ENVADV.2022.100203, 10.1016/J.ENVADV.2022.100203]"
"   Kahl G, 2011, The Dictionary of Genomics, Transcriptomics and Proteomics, DOI [10.1002/9783527678679.dg07434, DOI 10.1002/9783527678679.DG07434]"
"   Kang SY, 2007, BIOCHEM ENG J, V36, P54, DOI 10.1016/j.bej.2006.06.005"
"   Kapahi M, 2019, J HEALTH POLLUT, V9, DOI 10.5696/2156-9614-9.24.191203"
"   Karnwal A, 2024, DESALIN WATER TREAT, V319, DOI 10.1016/j.dwt.2024.100523"
"   Karthik C, 2017, J TAIWAN INST CHEM E, V70, P282, DOI 10.1016/j.jtice.2016.11.006"
"   Kathal R, 2016, Journal of Environmental & Analytical Toxicology, V6, DOI [10.4172/2161-0525.1000394, 10.4172/2161-0525.1000394, DOI 10.4172/2161-0525.1000394]"
"   Kaur C., 2020, Microbial technology for health and environment, P249"
"   Kaur G, 2015, BIOLOGIA, V70, P190, DOI 10.1515/biolog-2015-0023"
"   Khan IU, 2023, PLANTS-BASEL, V12, DOI 10.3390/plants12040725"
"   Khan N, 2016, INT J PHYTOREMEDIAT, V18, P1258, DOI 10.1080/15226514.2016.1203287"
"   Khan Z, 2022, J SAUDI CHEM SOC, V26, DOI 10.1016/j.jscs.2022.101569"
"   Khiari B, 2019, RENEW SUST ENERG REV, V108, P253, DOI 10.1016/j.rser.2019.03.057"
"   Khouja HR, 2013, FUNGAL GENET BIOL, V52, P53, DOI 10.1016/j.fgb.2012.11.004"
"   Kim S, 2005, PLANT CELL PHYSIOL, V46, P1809, DOI 10.1093/pcp/pci196"
"   Kiran BR, 2021, ENVIRON GEOCHEM HLTH, V43, P1507, DOI 10.1007/s10653-021-00825-1"
"   Kishor PBK, 2015, FRONT PLANT SCI, V6, DOI 10.3389/fpls.2015.00544"
"   Kuffner M, 2010, J APPL MICROBIOL, V108, P1471, DOI 10.1111/j.1365-2672.2010.04670.x"
"   Kumar A, 2020, HELIYON, V6, DOI 10.1016/j.heliyon.2020.e03170"
"   Kumar A, 2018, MICROBIOL RES, V207, P41, DOI 10.1016/j.micres.2017.11.004"
"   Kumar M, 2021, ENVIRON SUSTAIN IND, V12, DOI 10.1016/j.indic.2021.100155"
"   Kumar M, 2023, FRONT PLANT SCI, V14, DOI 10.3389/fpls.2023.1157678"
"   Kumar Santosh, 2012, J Pet Environ Biotechnol, V3"
"   Kumar V, 2021, ENVIRON SCI POLLUT R, V28, P10375, DOI 10.1007/s11356-020-11491-8"
"   LaVoie SP, 2018, BMC GENOMICS, V19, DOI 10.1186/s12864-017-4413-z"
"   Lebrun M., 2022, Assisted Phytoremediation, P101, DOI DOI 10.1016/B978-0-12-822893-7.00010-0"
"   Leduc DL, 2006, ENVIRON POLLUT, V144, P70, DOI 10.1016/j.envpol.2006.01.008"
"   Lee SH, 2014, J ENVIRON MANAGE, V139, P15, DOI 10.1016/j.jenvman.2014.02.019"
"   Li HY, 2012, FUNGAL DIVERS, V54, P11, DOI 10.1007/s13225-012-0165-x"
"   Li MH, 2020, INT J ENV RES PUB HE, V17, DOI 10.3390/ijerph17062118"
"   Li SN, 2022, ENVIRON SCI ECOTECH, V9, DOI 10.1016/j.ese.2022.100145"
"   Li XX, 2019, CHEMOSPHERE, V224, P716, DOI 10.1016/j.chemosphere.2019.02.184"
"   Li ZJ, 2022, ENVIRON POLLUT, V314, DOI 10.1016/j.envpol.2022.120292"
"   Liang XJ, 2015, ENVIRON MICROBIOL, V17, P2064, DOI 10.1111/1462-2920.12771"

"   Liebensteiner MG, 2014, FRONT MICROBIOL, V5, DOI 10.3389/fmicb.2014.00428"
"   Limmer M, 2016, ENVIRON SCI TECHNOL, V50, P6632, DOI 10.1021/acs.est.5b04113"
"   Lin HY, 2021, ISME J, V15, P1810, DOI 10.1038/s41396-020-00889-4"
"   Lin L, 2022, FRONT ENV SCI-SWITZ, V10, DOI 10.3389/fenvs.2022.880246"
"   Litwin A, 2020, REV ENVIRON SCI BIO, V19, P23, DOI 10.1007/s11157-020-09525-1"
"   Liu JN, 2008, B ENVIRON CONTAM TOX, V80, P260, DOI 10.1007/s00128-008-9357-1"
"   Liu Q, 2022, SEPARATIONS, V9, DOI 10.3390/separations9110374"
"   Liu XY, 2022, SUSTAINABILITY-BASEL, V14, DOI 10.3390/su14021020"
"   Liu YM, 2014, PLOS ONE, V9, DOI 10.1371/journal.pone.0112759"
"   Liu ZQ, 2020, MICROB CELL FACT, V19, DOI 10.1186/s12934-020-01431-z"
"   Liu ZC, 2021, ECOTOX ENVIRON SAFE, V226, DOI 10.1016/j.ecoenv.2021.112821"
"   Loureiro A, 2019, ANTIBIOTICS-BASEL, V8, DOI 10.3390/antibiotics8010018"
"   Lu HP, 2015, CHEMOSPHERE, V119, P209, DOI 10.1016/j.chemosphere.2014.06.024"
"   Lv YY, 2013, PLANT SOIL, V367, P507, DOI 10.1007/s11104-012-1486-y"
"   Ma Y, 2016, FRONT PLANT SCI, V7, DOI 10.3389/fpls.2016.00918"
"   Madanan MT, 2021, ENVIRON CHEM ECOTOX, V3, P17, DOI 10.1016/j.enceco.2020.10.007"
"   Mahas A, 2018, CURR OPIN VIROL, V32, P1, DOI 10.1016/j.coviro.2018.06.002"
"   Mahdavian Kobra, 2022, Proceedings of the Indian National Science Academy Part B Biological Sciences, V92, P319, DOI [10.1007/s40011-021-01345-9, 10.1007/s40011-021-01345-9]"
"   Mahmud HNME, 2016, RSC ADV, V6, P14778, DOI 10.1039/c5ra24358k"
"   Mala JGSM, 2020, Applied Microbiology Theory ??? Technology, P32, DOI [10.37256/amtt.112020222, 10.37256/amtt.112020222, DOI 10.37256/AMTT.112020222]"
"   Malik S, 2023, WATER-SUI, V15, DOI 10.3390/w15122262"
"   Mand??kov?? T, 2015, PLANT PHYSIOL, V169, P674, DOI 10.1104/pp.15.00619"
"   Manikandan SK, 2023, MOLECULES, V28, DOI 10.3390/molecules28020719"
"   Marshall MJ, 2006, PLOS BIOL, V4, P1324, DOI 10.1371/journal.pbio.0040268"
"   Mart??n JF, 2022, GENES-BASEL, V13, DOI 10.3390/genes13081450"
"   Masulili A., 2010, Journal of Agricultural Science (Toronto), V2, P39"
"   Mathur R, 2018, INDIAN J MED RES, V148, P279, DOI 10.4103/0971-5916.245303"
"   Mayak S, 2004, PLANT PHYSIOL BIOCH, V42, P565, DOI 10.1016/j.plaphy.2004.05.009"
"   Mayak S, 2004, PLANT SCI, V166, P525, DOI 10.1016/j.plantsci.2003.10.025"
"   McCarty Kathleen M., 2011, Reviews on Environmental Health, V26, P71, DOI 10.1515/REVEH.2011.010"
"   McGrath SP, 2003, CURR OPIN BIOTECH, V14, P277, DOI 10.1016/S0958-1669(03)00060-0"
"   Mei Y, 2016, J GENET GENOMICS, V43, P63, DOI 10.1016/j.jgg.2016.01.001"
"   Meier S, 2012, ECOTOX ENVIRON SAFE, V75, P8, DOI 10.1016/j.ecoenv.2011.08.029"
"   MENCH M, 1991, PLANT SOIL, V132, P187, DOI 10.1007/BF00010399"
"   Mengstie MA, 2021, BIOL-TARGETS THER, V15, P353, DOI 10.2147/BTT.S326422"
"   Miethke M, 2007, MICROBIOL MOL BIOL R, V71, P413, DOI 10.1128/MMBR.00012-07"
"   Ministry of Environment Forest and Climate Change, GEAC Approvals"
"   Mishra J, 2017, FRONT MICROBIOL, V8, DOI 10.3389/fmicb.2017.01706"
"   Mishra Ritesh, 2012, Toxicol Int, V19, P25, DOI 10.4103/0971-6580.94512"
"   Mishra S., 2015, Plant-Environment Interaction, P327, DOI [DOI 10.1002/9781119081005.CH18, 10.1002/9781119081005.ch, DOI 10.1002/9781119081005.CH]"
"   Miyata N, 2004, FEMS MICROBIOL ECOL, V47, P101, DOI 10.1016/S0168-6496(03)00251-4"
"   Mohamadhasani F, 2022, SCI REP-UK, V12, DOI 10.1038/s41598-022-24349-5"
"   Mohsenzadeh F, 2014, J ENVIRON HEALTH SCI, V12, DOI 10.1186/2052-336X-12-102"
"   Monographs I, 2024, Report of the Advisory Group to Recommend Priorities for the IARC Monographs during"
"   Mosa KA, 2016, FRONT PLANT SCI, V7, DOI 10.3389/fpls.2016.00303"
"   Mosna K, 2023, METALLOMICS, V15, DOI 10.1093/mtomcs/mfad061"
"   Mukherjee S, 2022, ENVIRON POLLUT, V308, DOI 10.1016/j.envpol.2022.119609"
"   Munir N, 2022, SUSTAINABILITY-BASEL, V14, DOI 10.3390/su14010161"
"   Mustapha M. U., 2015, Journal of Microbial and Biochemical Technology, V7, P253, DOI 10.4172/1948-5948.1000219"
"   Mwandira W, 2020, SCI REP-UK, V10, DOI 10.1038/s41598-020-78187-4"
"   Nadarajah K, 2021, J WATER PROCESS ENG, V40, DOI 10.1016/j.jwpe.2021.101929"
"   N??dgorska-Socha A, 2013, ECOTOXICOLOGY, V22, P1422, DOI 10.1007/s10646-013-1129-y"
"   Narayanan M, 2022, FRONT MICROBIOL, V13, DOI 10.3389/fmicb.2022.929730"
"   Naveed M, 2024, SCI REP-UK, V14, DOI 10.1038/s41598-024-59340-9"
"   Nayak AK, 2018, INT J PHYTOREMEDIAT, V20, P682, DOI 10.1080/15226514.2017.1413332"
"   Nedjimi B, 2021, SN APPL SCI, V3, DOI 10.1007/s42452-021-04301-4"
"   Nianiou-Obeidat I, 2017, PLANT CELL REP, V36, P791, DOI 10.1007/s00299-017-2139-7"
"   Nikolic M, 2015, URBAN FOR URBAN GREE, V14, P782, DOI 10.1016/j.ufug.2015.08.002"
"   Nishimasu H, 2014, CELL, V156, P935, DOI 10.1016/j.cell.2014.02.001"
"   Nogueira-Lopez G, 2018, FRONT PLANT SCI, V9, DOI 10.3389/fpls.2018.00409"
"   Nowicka B, 2022, ENVIRON SCI POLLUT R, V29, P16860, DOI 10.1007/s11356-021-18419-w"
"   Nur-E-Alam M, 2020, APPL WATER SCI, V10, DOI 10.1007/s13201-020-01286-0"
"   Nwadinigwe AO., 2018, Overview of nano-phytoremediation applications BT-phytoremediation: management of environmental contaminants, V6, P377"
"   Nworie OE, 2017, TOXICS, V5, DOI 10.3390/toxics5030018"
"   Sadiku OO, 2022, ARH HIG RADA TOKSIKO, V73, P244, DOI 10.2478/aiht-2022-73-3669"
"   Omiadze N. T., 2018, Annals of Agrarian Science, V16, P196, DOI [10.1016/j.aasci.2018.03.009, 10.1016/j.aasci.2018.03.009]"
"   Orji OU, 2021, HELIYON, V7, DOI 10.1016/j.heliyon.2021.e07512"
"   Pacios-Michelena S, 2021, FRONT SUSTAIN FOOD S, V5, DOI 10.3389/fsufs.2021.696518"
"   Padhye LP, 2022, FRONT CHEM ENG, V4, DOI 10.3389/fceng.2022.867228"
"   Palmieri G, 2000, APPL ENVIRON MICROB, V66, P920, DOI 10.1128/AEM.66.3.920-924.2000"
"   Pan B, 1999, EUR J AGRON, V11, P179, DOI 10.1016/S1161-0301(99)00029-5"
"   Pande A, 2022, FRONT PLANT SCI, V13, DOI 10.3389/fpls.2022.1019647"
"   Pande V, 2022, FRONT MICROBIOL, V13, DOI 10.3389/fmicb.2022.824084"
"   Pandey HK, 2015, Regulatory Framework for GE Plants in India, P1"
"   Pandey N, 2016, EUR J SOIL BIOL, V75, P142, DOI 10.1016/j.ejsobi.2016.05.007"
"   Patra DK, 2021, ENVIRON TECHNOL INNO, V21, DOI 10.1016/j.eti.2020.101293"
"   Patra M, 2004, ENVIRON EXP BOT, V52, P199, DOI 10.1016/j.envexpbot.2004.02.009"
"   Paul S., 2007, J Hazard Subst Res, DOI [10.4148/1090-7025.1010, DOI 10.4148/1090-7025.1010]"

"   Paumi CM, 2009, MICROBIOL MOL BIOL R, V73, P577, DOI 10.1128/MMBR.00020-09"
"   Pavel PB, 2014, SCI TOTAL ENVIRON, V479, P125, DOI 10.1016/j.scitotenv.2014.01.097"
"   Peco JD, 2020, ECOTOX ENVIRON SAFE, V201, DOI 10.1016/j.ecoenv.2020.110784"
"   Pecoraro L, 2022, J FUNGI, V8, DOI 10.3390/jof8010021"
"   Pedron F, 2021, APPL SCI-BASEL, V11, DOI 10.3390/app11041923"
"   Pe??a MMO, 2000, J BIOL CHEM, V275, P33244, DOI 10.1074/jbc.M005392200"
"   Peng AH, 2017, PLANT BIOTECHNOL J, V15, P1509, DOI 10.1111/pbi.12733"
"   P??rez-Esteban J, 2014, PLANT SOIL, V377, P97, DOI 10.1007/s11104-013-1629-9"
"   Pesce S, 2020, FRONT MICROBIOL, V11, DOI 10.3389/fmicb.2020.01342"
"   Pianelli K, 2005, TRANSGENIC RES, V14, P739, DOI 10.1007/s11248-005-7159-3"
"   Pilon-Smits EAH, 2009, CURR OPIN BIOTECH, V20, P207, DOI 10.1016/j.copbio.2009.02.001"
"   Priya AK, 2023, TOXICS, V11, DOI 10.3390/toxics11050422"
"   Probanza A, 2002, APPL SOIL ECOL, V20, P75, DOI 10.1016/S0929-1393(02)00007-0"
"   Purakayastha TJ, 2008, INT J PHYTOREMEDIAT, V10, P61, DOI 10.1080/15226510701827077"
"   2023, ZANCO Journal of Pure and Applied Sciences, V35, DOI [10.21271/zjpas.35.3.16, 10.21271/ZJPAS.35.3.16, DOI 10.21271/ZJPAS.35.3.16]"
"   Qasem NAA, 2021, NPJ CLEAN WATER, V4, DOI 10.1038/s41545-021-00127-0"
"   Qayyumi S, 2016, INDIAN J GEO-MAR SCI, V45, P499"
"   Qin HC, 2024, MICROORGANISMS, V12, DOI 10.3390/microorganisms12040700"
"   Qin W, 2020, ADV MATER, V32, DOI 10.1002/adma.201907833"
"   Qureshi FF, 2024, PLANT STRESS, V11, DOI 10.1016/j.stress.2024.100394"
"   Rabani M. S., 2022, Phytoremediation, P91, DOI DOI 10.1016/B978-0-323-89874-4.00006-6"
"   Radons J, 2016, CELL STRESS CHAPERON, V21, P379, DOI 10.1007/s12192-016-0676-6"
"   Rafique M, 2019, ENVIRON SCI POLLUT R, V26, P20689, DOI 10.1007/s11356-019-05323-7"
"   Rajapaksha AU, 2016, CHEMOSPHERE, V148, P276, DOI 10.1016/j.chemosphere.2016.01.043"
"   Rajkumar M, 2008, BIORESOURCE TECHNOL, V99, P3491, DOI 10.1016/j.biortech.2007.07.046"
"   Raklami A, 2022, INT J MOL SCI, V23, DOI 10.3390/ijms23095031"
"   Ramli NN, 2023, MICROBIOL RES, V268, DOI 10.1016/j.micres.2022.127288"
"   Rodr??guez-Rodr??guez DR, 2019, INT J MOL MED, V43, P1559, DOI 10.3892/ijmm.2019.4112"
"   Rascio N, 2011, PLANT SCI, V180, P169, DOI 10.1016/j.plantsci.2010.08.016"
"   Rathika R, 2021, CHEMOSPHERE, V271, DOI 10.1016/j.chemosphere.2020.129513"
"   Rawat K., 2015, INT J SCI ENG RES, V6, P1041"
"   Raza A, 2020, BIOLOGY-BASEL, V9, DOI 10.3390/biology9070177"
"   Refaey M, 2021, Role of Fungi in Bioremediation of Soil Contaminated with Heavy Metals, DOI [10.1007/978-3-030-67561-5_16, DOI 10.1007/978-3-030-67561-5_16]"
"   Rehman A, 2010, WATER AIR SOIL POLL, V205, P149, DOI 10.1007/s11270-009-0062-4"
"   Ren GM, 2015, ECOTOX ENVIRON SAFE, V117, P141, DOI 10.1016/j.ecoenv.2015.03.033"
"   Rezania S, 2016, J HAZARD MATER, V318, P587, DOI 10.1016/j.jhazmat.2016.07.053"
"   Robinson JR, 2021, J FUNGI, V7, DOI 10.3390/jof7030225"
"   Romaniuk JAH, 2015, PHILOS T R SOC B, V370, DOI 10.1098/rstb.2015.0024"
"   Romera E, 2007, BIORESOURCE TECHNOL, V98, P3344, DOI 10.1016/j.biortech.2006.09.026"
"   Romo-Rodriguez P., 2019, Fungal Bioremed, V4, P173, DOI [10.1201/9781315205984-6, DOI 10.1201/9781315205984-6]"
"   Ruiz ON, 2011, PLANT BIOTECHNOL J, V9, P609, DOI 10.1111/j.1467-7652.2011.00616.x"
"   Ruiz ON, 2009, CURR OPIN BIOTECH, V20, P213, DOI 10.1016/j.copbio.2009.02.010"
"   Rylott EL, 2015, J EXP BOT, V66, P6519, DOI 10.1093/jxb/erv384"
"   Sahu PK, 2022, FRONT PLANT SCI, V13, DOI 10.3389/fpls.2022.1042936"
"   Sakakibara M., 2010, [No title captured], V12, P26"
"   Sakamoto M, 2018, TOXICS, V6, DOI 10.3390/toxics6030045"
"   Samadi S, 2017, BIOFUEL RES J, V4, P545, DOI 10.18331/BRJ2017.4.1.4"
"   Sampanpanish P, 2019, B ENVIRON CONTAM TOX, V102, P140, DOI 10.1007/s00128-018-2502-6"
"   Santiba??ez C, 2008, SCI TOTAL ENVIRON, V395, P1, DOI 10.1016/j.scitotenv.2007.12.033"
"   Sarma H, 2021, CURR RES MICROB SCI, V2, DOI 10.1016/j.crmicr.2021.100041"
"   Sasaki Y, 2006, TRANSGENIC RES, V15, P615, DOI 10.1007/s11248-006-9008-4"
"   Saxena P., 2020, Bioremediation of pollutants, P125, DOI [DOI 10.1016/B978-0-12-819025-8.00005-3, 10.1016/B978-0-12-819025-8.00005-3]"
"   Scherer J, 2009, MOL MICROBIOL, V73, P601, DOI 10.1111/j.1365-2958.2009.06792.x"
"   Scutarasu EC, 2023, FOODS, V12, DOI 10.3390/foods12183340"
"   Sebesta M, 2022, INT J MOL SCI, V23, DOI 10.3390/ijms232214084"
"   Shabbir MA, 2019, ANN CLIN MICROB ANTI, V18, DOI 10.1186/s12941-019-0317-x"
"   Shaheen R, 2024, RESULTS CHEM, V11, DOI 10.1016/j.rechem.2024.101763"
"   Shahsavari E., 2020, A handbook of environmental toxicology: human disorders and ecotoxicology, P517, DOI 10.1079/9781786394675.0517"
"   Shaji E, 2021, GEOSCI FRONT, V12, DOI 10.1016/j.gsf.2020.08.015"
"   Shameer S, 2016, 3 BIOTECH, V6, DOI 10.1007/s13205-016-0498-3"
"   Shao M, 2016, ZOOL RES, V37, P191, DOI 10.13918/j.issn.2095-8137.2016.4.191"
"   Sharma A, 2003, SOIL BIOL BIOCHEM, V35, P887, DOI 10.1016/S0038-0717(03)00119-6"
"   Sharma JK, 2023, FRONT PLANT SCI, V14, DOI 10.3389/fpls.2023.1076876"
"   Sharma P, 2022, BIOENGINEERED, V13, P4923, DOI 10.1080/21655979.2022.2037273"
"   Sharma R., 2014, Emerging Technologies and Management of Crop Stress Tolerance, P405, DOI DOI 10.1016/B978-0-12-800875-1.00017-X"
"   Sharma S, 2015, ENVIRON SCI POLLUT R, V22, P946, DOI 10.1007/s11356-014-3635-8"
"   Sheng YW, 2014, CHEM REV, V114, P3854, DOI 10.1021/cr4005296"
"   Shi A, 2023, ENVIRON POLLUT, V327, DOI 10.1016/j.envpol.2023.121559"
"   Shin MN, 2012, J HAZARD MATER, V199, P314, DOI 10.1016/j.jhazmat.2011.11.010"
"   Shiyab S, 2018, AGRICULTURE-BASEL, V8, DOI 10.3390/agriculture8020029"
"   Simiele M, 2020, SCI TOTAL ENVIRON, V710, DOI 10.1016/j.scitotenv.2019.136203"
"   Singh N, 2021, ENVIRON SUSTAIN, V4, P63, DOI 10.1007/s42398-021-00162-0"
"   Singh S., 2016, Proteomics, V6, P1, DOI [10.3389/fpls.2015.01143, DOI 10.3389/FPLS.2015.01143]"
"   Sinhal VK, 2010, J ENVIRON BIOL, V31, P255"
"   Smith AD, 2017, ANNU REV MICROBIOL, V71, P597, DOI 10.1146/annurev-micro-030117-020444"
"   Sohi S., 2009, CSIRO LAND WATER SCI, V5, P17, DOI DOI 10.1139/Z03-132"
"   Solioz M, 1996, TRENDS BIOCHEM SCI, V21, P237"
"   Srivastava S, 2006, CURR MICROBIOL, V53, P232, DOI 10.1007/s00284-006-0103-9"

"   Staats CC, 2013, FRONT CELL INFECT MI, V3, DOI 10.3389/fcimb.2013.00065"
"   Sun GL, 2019, NAT COMMUN, V10, DOI 10.1038/s41467-019-13093-6"
"   Sun Hong, 2015, Curr Environ Health Rep, V2, P295, DOI [10.1007/s40572-015-0054-z, 10.1007/s40572-015-0054-z]"
"   Sun YB, 2016, J ENVIRON MANAGE, V166, P204, DOI 10.1016/j.jenvman.2015.10.017"
"   Supreeth M, 2022, INT J ENVIRON SCI TE, V19, P4587, DOI 10.1007/s13762-021-03354-7"
"   Susilawati A., 2020, IOP Conference Series: Materials Science and Engineering, V980, DOI [10.1088/1757-899x/980/1/012065, 10.1088/1757-899X/980/1/012065]"
"   Suthar V, 2014, ENVIRON MONIT ASSESS, V186, P3957, DOI 10.1007/s10661-014-3671-3"
"   Syc M, 2020, J HAZARD MATER, V393, DOI 10.1016/j.jhazmat.2020.122433"
"   Tahir M, 2022, ENVIRON SCI POLLUT R, V29, P6026, DOI 10.1007/s11356-021-16018-3"
"   Takahashi T, 2010, ANN BOT-LONDON, V105, P1, DOI 10.1093/aob/mcp259"
"   Tamayo E, 2014, FRONT PLANT SCI, V5, DOI 10.3389/fpls.2014.00547"
"   Tang L, 2017, ENVIRON SCI POLLUT R, V24, P19293, DOI 10.1007/s11356-017-9146-7"
"   Tang X, 2021, ECOTOX ENVIRON SAFE, V208, DOI 10.1016/j.ecoenv.2020.111699"
"   Tarfeen N, 2022, PROCESSES, V10, DOI 10.3390/pr10071358"
"   Tasleem M, 2023, TOXICS, V11, DOI 10.3390/toxics11100864"
"   Tattibayeva Z, 2022, HELIYON, V8, DOI 10.1016/j.heliyon.2022.e10468"
"   Tavakoli K, 2021, BIOTECH-BASEL, V10, DOI 10.3390/biotech10030014"
"   Tchounwou P. B., 2012, EXS, V101, P133, DOI [DOI 10.1007/978-3-7643-8340-4_6, 10.1007/978-3-7643-8340-46, 10.1007/978-3-7643-8340-4_6]"
"   Terry N, 2000, ANNU REV PLANT PHYS, V51, P401, DOI 10.1146/annurev.arplant.51.1.401"
"   Thakur K., 2021, Plant-microbial interactions and smart agricultural biotechnology, P253, DOI [10.1201/9781003213864-13, DOI 10.1201/9781003213864-13]"
"   Thandeeswaran M, 2019, Biotechnology Research and Innovation, V3, P159, DOI [10.1016/j.biori.2019.02.003, DOI 10.1016/J.BIORI.2019.02.003]"
"   Tong YP, 2004, TRENDS PLANT SCI, V9, P7, DOI 10.1016/j.tplants.2003.11.009"
"   Topoliantz S, 2005, BIOL FERT SOILS, V41, P15, DOI 10.1007/s00374-004-0804-9"
"   Torres E, 2020, PROCESSES, V8, DOI 10.3390/pr8121584"
"   Traxler L, 2022, J HAZARD MATER, V425, DOI 10.1016/j.jhazmat.2021.127978"
"   Tripathi M, 2023, FRONT MICROBIOL, V14, DOI 10.3389/fmicb.2023.1168954"
"   Trippe RC III, 2021, J HAZARD MATER, V404, DOI 10.1016/j.jhazmat.2020.124178"
"   TSAI KJ, 1992, J BACTERIOL, V174, P116, DOI 10.1128/jb.174.1.116-121.1992"
"   Tumolo M, 2020, INT J ENV RES PUB HE, V17, DOI 10.3390/ijerph17155438"
"   Tunali S, 2006, CHEM ENG J, V115, P203, DOI 10.1016/j.cej.2005.09.023"
"   Turchi A, 2012, PLANT SCI, V183, P50, DOI 10.1016/j.plantsci.2011.11.008"
"   ul Hassan Zaid, 2017, PROBIOTICS AGROECOSY, V1, P517, DOI DOI 10.1007/978-981-10-4059-727"
"   Upadhyay N, 2017, FRONT PLANT SCI, V8, DOI 10.3389/fpls.2017.00778"
"   Vaksmaa A, 2023, FRONT MAR SCI, V10, DOI 10.3389/fmars.2023.1070905"
"   Vald??s J, 2008, BMC GENOMICS, V9, DOI 10.1186/1471-2164-9-597"
"   van der Ent A, 2013, PLANT SOIL, V362, P319, DOI 10.1007/s11104-012-1287-3"
"   Pham VHT, 2022, MICROORGANISMS, V10, DOI 10.3390/microorganisms10030610"
"   Vangronsveld J, 2009, ENVIRON SCI POLLUT R, V16, P765, DOI 10.1007/s11356-009-0213-6"
"   V??zquez S, 2006, WATER AIR SOIL POLL, V177, P349, DOI 10.1007/s11270-006-9178-y"
"   Vecchi V., 2020, PLANTS-BASEL, V9, P67, DOI DOI 10.3390/plants9010067"
"   Verbruggen N, 2009, CURR OPIN PLANT BIOL, V12, P364, DOI 10.1016/j.pbi.2009.05.001"
"   Vignesh KS, 2017, INT J MOL SCI, V18, DOI 10.3390/ijms18102197"
"   Visconti D, 2020, CHEMOSPHERE, V260, DOI 10.1016/j.chemosphere.2020.127661"
"   Vocciante M, 2021, SUSTAINABILITY-BASEL, V13, DOI 10.3390/su13020770"
"   Voumik LC, 2022, HELIYON, V8, DOI 10.1016/j.heliyon.2022.e11457"
"   Wang A, 2022, J HAZARD MATER, V429, DOI 10.1016/j.jhazmat.2022.128396"
"   Wang JX, 2020, J HAZARD MATER, V394, DOI 10.1016/j.jhazmat.2020.122505"
"   Wang M, 2022, FRONT PLANT SCI, V13, DOI 10.3389/fpls.2022.827453"
"   Wang SY, 2017, ENVIRON SCI POLLUT R, V24, P15209, DOI 10.1007/s11356-017-8909-5"
"   Wang Y, 2018, J CELL BIOL, V217, P1915, DOI 10.1083/jcb.201708007"
"   Wang YS, 2012, PLOS ONE, V7, DOI [10.1371/journal.pone.0030635, 10.1371/journal.pone.0047302, 10.1371/journal.pone.0048669]"
"   Wani Ab Latif, 2015, Interdiscip Toxicol, V8, P55, DOI [10.1515/intox-2015-0009, 10.1515/intox-2015-0009]"
"   Weale A, 2010, NEW BIOTECHNOL, V27, P582, DOI 10.1016/j.nbt.2010.08.013"
"   Wei Kang, 2007, Journal of Zhejiang University-Science B, V8, P266, DOI 10.1631/jzus.2007.B0266"
"   Welter T, 2017, Bachelor of Engineering Thesis,, DOI [10.13140/RG.2.2.19572.55688, DOI 10.13140/RG.2.2.19572.55688]"
"   Wen XP, 2010, TRANSGENIC RES, V19, P91, DOI 10.1007/s11248-009-9296-6"
"   Wolfger H, 2001, RES MICROBIOL, V152, P375, DOI 10.1016/S0923-2508(01)01209-8"
"   Wong MH, 2003, CHEMOSPHERE, V50, P775, DOI 10.1016/S0045-6535(02)00232-1"
"   Woraharn S, 2021, WATER AIR SOIL POLL, V232, DOI 10.1007/s11270-021-05156-6"
"   World Health Organization, 2024, Mercury and Health"
"   Wrobel Monika, 2023, Int J Environ Res Public Health, V20, DOI [10.3390/ijerph20064964, 10.3390/ijerph20064964]"
"   Wr??blewski K, 2024, SCI REP-UK, V14, DOI 10.1038/s41598-024-52234-w"
"   Wu QH, 2011, INT J PHYTOREMEDIAT, V13, P788, DOI 10.1080/15226514.2010.525562"
"   Wu YH, 2020, MOL MED REP, V21, P851, DOI 10.3892/mmr.2019.10885"
"   Wuana R.A., 2011, ISRN Ecology, DOI [10.5402/2011/402647, DOI 10.5402/2011/402647]"
"   Yaashikaa P. R., 2020, Biotechnology Reports, V28, pe00570, DOI [10.1016/j.btre.2020.e00570, 10.1016/j.btre.2020.e00570]"
"   Yadav SK, 2023, SCI REP-UK, V13, DOI 10.1038/s41598-023-33466-8"
"   Yan A, 2020, FRONT PLANT SCI, V11, DOI 10.3389/fpls.2020.00359"
"   Yan GW, 2023, FRONT ENV SCI-SWITZ, V11, DOI 10.3389/fenvs.2023.1131204"
"   Yan HL, 2019, J HAZARD MATER, V368, P386, DOI 10.1016/j.jhazmat.2019.01.072"
"   Yang D, 2020, COMPOS PART A-APPL S, V138, DOI 10.1016/j.compositesa.2020.106042"
"   Yang JH, 2016, NAT GENET, V48, P1225, DOI 10.1038/ng.3657"
"   Yang JX, 2017, ECOL ENG, V109, P35, DOI 10.1016/j.ecoleng.2017.09.001"
"   Ye BJ, 2016, ANN OCCUP ENVIRON ME, V28, DOI 10.1186/s40557-015-0086-8"

"   Yorifuji T, 2020, J EPIDEMIOL, V30, P12, DOI 10.2188/jea.JE20190089"
"   Yu KL, 2017, BIORESOURCE TECHNOL, V246, P2, DOI 10.1016/j.biortech.2017.08.009"
"   Yu XF, 2023, CHEMOSPHERE, V315, DOI 10.1016/j.chemosphere.2022.137677"
"   Yuan LZ, 2021, ENVIRON RES, V198, DOI 10.1016/j.envres.2021.111290"
"   Zaidi S, 2006, CHEMOSPHERE, V64, P991, DOI 10.1016/j.chemosphere.2005.12.057"
"   Zeremski T, 2021, PLANTS-BASEL, V10, DOI 10.3390/plants10112340"
"   Zhang JH, 2019, ECOTOX ENVIRON SAFE, V176, P162, DOI 10.1016/j.ecoenv.2019.03.091"
"   Zhang L, 2017, PLANT MOL BIOL, V95, P99, DOI 10.1007/s11103-017-0639-z"
"   Zhang QH, 2015, APPL BIOCHEM BIOTECH, V175, P1281, DOI 10.1007/s12010-014-1298-z"
"   Zhao CR, 2024, J FUNGI, V10, DOI 10.3390/jof10040273"
"   Zhao SY, 2023, SCI REP-UK, V13, DOI 10.1038/s41598-023-43112-y"
"   Zhao YY, 2020, G3-GENES GENOM GENET, V10, P479, DOI 10.1534/g3.119.400933"
"   ZHOU JM, 1994, PLANT CELL, V6, P875, DOI 10.1105/tpc.6.6.875"
NR 462
TC 5
Z9 6
U1 21
U2 23
PU SPRINGER
PI NEW YORK
"PA ONE NEW YORK PLAZA, SUITE 4600, NEW YORK, NY, UNITED STATES"
SN 0923-9820
EI 1572-9729
J9 BIODEGRADATION
JI Biodegradation
PD JUL 25
PY 2025
VL 36
IS 4
AR 69
DI 10.1007/s10532-025-10165-x
PG 50
WC Biotechnology & Applied Microbiology
WE Science Citation Index Expanded (SCI-EXPANDED)
SC Biotechnology & Applied Microbiology
GA 5JK5I
UT WOS:001537109200002
PM 40715906
DA 2026-04-06
ER

PT J
"AU Wei, T"
"   Wang, YY"
"   Ma, XL"
"   Hu, RY"
"   Guo, JK"
"   Hua, L"
"   Zhang, XK"
"AF Wei, Ting"
"   Wang, Yuyao"
"   Ma, Xiulian"
"   Hu, Rongyi"
"   Guo, Junkang"
"   Hua, Li"
"   Zhang, Xingkai"
TI Bacteria-loaded Biochar (BC)/Phosphorus-Modified Biochar (PBC): An
   Efficient Biosorbent for Remediation of Cd and Pb Co-Contaminated Soil
SO WATER AIR AND SOIL POLLUTION
LA English
DT Article
DE Heavy metal stress; Modified biochar; Bacteria-BC/PBC composites; Heavy
   metal immobilization; Remediation
ID MICROBIAL COMMUNITY; HEAVY-METALS; TOXICITY; CADMIUM; AVAILABILITY;
   REMOVAL; SMELTER; CD(II); PLANTS
"AB Heavy metal pollution is a serious environmental risk worldwide. Environmental-friendly strategies for heavy metal remediation are urgently needed. In this study, the bacterial consortium BR7 was constructed using heavy metal immobilizing bacteria Priestia megaterium B2 and Enterobacter sp. B57. The B2, B57 and BR7 (B2:B57 = 3:2) were immobilized on rice husk biochar (BC)/phosphorus-modified biochar (PBC) and their effects on remediation of Pb, Cd co-contaminated soil were evaluated. Immobilization increased bacteria viability in heavy metal polluted soil. Addition of bacteria, BC/PBC, and the immobilized bacteria in soil reduced the DTPA-extractable Cd and Pb by 20.00%-62.18% and 10.77%-43.99%, respectively. Accompanied by the conversion of acid extractable Cd, Pb into residual state. Meanwhile, soil urease, sucrase and catalase activity were stimulated and the bacterial community was improved. Importantly, their application enhanced plant growth and reduced Cd, Pb content in pakchoi, the Cd content in shoot and root decreased by 2.45%-16.58% and 17.92%-59.93%, respectively. The Pb content in shoot and root decreased by 7.37%-70.17% and 2.03%-62.64%, respectively. Collectively, the immobilized bacteria exhibited better remediation performance than bacteria, BC/PBC alone, indicating a synergistic effect has occurred. Among them, BR7-PBC showed the best effect. This study provides a feasible way for remediation of heavy metal polluted soil."
"C1 [Wei, Ting; Wang, Yuyao; Ma, Xiulian; Hu, Rongyi; Guo, Junkang; Hua, Li] Shaanxi Univ Sci & Technol, Sch Environm Sci & Engn, Xian 710021, Peoples R China."
"   [Zhang, Xingkai] Jilin Yige Environm Technol Co LTD, Jilin 13000, Peoples R China."
C3 Shaanxi University of Science & Technology

"RP Wei, T (corresponding author), Shaanxi Univ Sci & Technol, Sch Environm Sci & Engn, Xian 710021, Peoples R China."
EM weiting@sust.edu.cn
FU National Natural Science Foundation of China [22376133]; National
Natural Science Foundation of China
FX This research was financially supported by the National Natural Science
Foundation of China (22376133).
"CR Ali A, 2019, ECOTOX ENVIRON SAFE, V184, DOI 10.1016/j.ecoenv.2019.109635"
" Andrey G, 2019, APPL GEOCHEM, V104, P93, DOI 10.1016/j.apgeochem.2019.03.017"
" Anjitha KS, 2023, FUNCT PLANT BIOL, V50, P965, DOI 10.1071/FP23145"
" Aponte H, 2020, SCI TOTAL ENVIRON, V737, DOI 10.1016/j.scitotenv.2020.139744"
" Arous A, 2024, AGRICULTURE-BASEL, V14, DOI 10.3390/agriculture14091527"
" Ayangbenro AS, 2017, INT J ENV RES PUB HE, V14, DOI 10.3390/ijerph14010094"
" Cai JF, 2021, SCI TOTAL ENVIRON, V788, DOI 10.1016/j.scitotenv.2021.147707"
" Chen JQ, 2018, ECOTOX ENVIRON SAFE, V163, P172, DOI 10.1016/j.ecoenv.2018.07.071"
" Chen JL, 2024, PROCESS SAF ENVIRON, V192, P649, DOI 10.1016/j.psep.2024.10.084"
" Chen MM, 2024, PLANT SOIL, V504, P643, DOI 10.1007/s11104-024-06650-6"
" Cui HB, 2022, SCI TOTAL ENVIRON, V824, DOI 10.1016/j.scitotenv.2022.153957"
" Dhokpande SR, 2024, REV INORG CHEM, V44, P655, DOI 10.1515/revic-2024-0027"
" Du HH, 2017, ENVIRON POLLUT, V229, P871, DOI 10.1016/j.envpol.2017.07.052"
" Ertani A, 2017, WATER AIR SOIL POLL, V228, DOI 10.1007/s11270-017-3356-y"
" Gao RL, 2020, SCI TOTAL ENVIRON, V730, DOI 10.1016/j.scitotenv.2020.139119"
" Gou ZC, 2023, ENVIRON POLLUT, V317, DOI 10.1016/j.envpol.2022.120790"
" Guo KX, 2024, FRONT NUTR, V11, DOI 10.3389/fnut.2024.1337996"
" Guo SY, 2023, ENV POLLUT BIOAVAIL, V35, DOI 10.1080/26395940.2023.2212127"
" Gyanwali Prashant, 2024, Egyptian Journal of Soil Science, V64, P855, DOI [10.21608/ejss.2024.270380.1725, 10.21608/EJSS.2024.270380.1725]"
" Hameed A, 2016, PLANT METAL INTERACTION: EMERGING REMEDIATION TECHNIQUES, P557, DOI 10.1016/B978-0-12-803158-2.00024-2"
" Han JC, 2024, GEOMICROBIOL J, V41, P61, DOI 10.1080/01490451.2023.2272619"
" Heidari P, 2020, INT J ENV RES PUB HE, V17, DOI 10.3390/ijerph17114059"
" Hu XS, 2024, SCI TOTAL ENVIRON, V927, DOI 10.1016/j.scitotenv.2024.172170"
" Hua L, 2021, CHEMOSPHERE, V263, DOI 10.1016/j.chemosphere.2020.127795"
" Huang DL, 2018, J HAZARD MATER, V348, P109, DOI 10.1016/j.jhazmat.2018.01.024"
" Huang J, 2023, SCI TOTAL ENVIRON, V872, DOI 10.1016/j.scitotenv.2023.162021"
" Ji XW, 2022, SCI TOTAL ENVIRON, V811, DOI 10.1016/j.scitotenv.2021.152112"
" Jia LX, 2021, J ENVIRON MANAGE, V292, DOI 10.1016/j.jenvman.2021.112750"
" Jia YF, 2022, B ENVIRON CONTAM TOX, V108, P909, DOI 10.1007/s00128-022-03473-y"
" Juwarkar AA, 2010, BIOREMEDIATION TECHNOLOGY: RECENT ADVANCES, P266"
" Kaushik Pallavi, 2022, Flora and Fauna - Jhansi, V28, P315, DOI [10.33451/florafauna.v28i2pp315-327, 10.33451/florafauna.v28i2pp315-327]"
" Kelly JJ, 2003, BIOL FERT SOILS, V38, P65, DOI 10.1007/s00374-003-0642-1"
" Kumar A., 2023, Bio-Inspired Land Remediation, P317, DOI DOI 10.1007/978-3-031-04931-6_13"
" Kumar R, 2022, SCI TOTAL ENVIRON, V836, DOI 10.1016/j.scitotenv.2022.155619"
" Li F, 2019, ECOTOX ENVIRON SAFE, V184, DOI 10.1016/j.ecoenv.2019.109655"
" Li HX, 2020, CHEMOSPHERE, V244, DOI 10.1016/j.chemosphere.2019.125471"
" Li Q, 2019, POL J ENVIRON STUD, V28, P2697, DOI 10.15244/pjoes/92527"
" [????????? Li Ruimei], 2002, [???????????????, Soil and environmental sciences], V11, P348"
" Li SQ, 2024, CHEMOECOLOGY, V34, P47, DOI 10.1007/s00049-024-00399-1"
" Li Y, 2015, ENVIRON MONIT ASSESS, V187, DOI 10.1007/s10661-015-4892-9"
" [????????? Li Zhiping], 2015, [????€???????, Acta Ecologica Sinica], V35, P3919"
" Liang MA, 2021, SUSTAINABILITY-BASEL, V13, DOI 10.3390/su132414041"
" Lin H, 2023, INT BIODETER BIODEGR, V178, DOI 10.1016/j.ibiod.2022.105544"
" Liu CR, 2024, J ENVIRON SCI, V144, P172, DOI 10.1016/j.jes.2023.07.039"
" Liu C, 2022, J ENVIRON CHEM ENG, V10, DOI 10.1016/j.jece.2022.107393"
" Liu HK, 2018, SCI TOTAL ENVIRON, V645, P702, DOI 10.1016/j.scitotenv.2018.07.115"
" Liu SJ, 2018, CHEMOSPHERE, V208, P655, DOI 10.1016/j.chemosphere.2018.06.023"
" Liu YL, 2020, CHEMOSPHERE, V247, DOI 10.1016/j.chemosphere.2020.125850"
" Luo Y, 2022, SCI TOTAL ENVIRON, V831, DOI 10.1016/j.scitotenv.2022.154845"
" Luo ZX, 2024, FRONT MICROBIOL, V15, DOI 10.3389/fmicb.2024.1365234"
" Ma H, 2020, J HAZARD MATER, V388, DOI 10.1016/j.jhazmat.2020.122065"
" Ma JW, 2010, J HAZARD MATER, V176, P715, DOI 10.1016/j.jhazmat.2009.11.093"
" Menzembere ERGY, 2023, PEDOSPHERE, V33, P23, DOI 10.1016/j.pedsph.2022.06.030"
" Mishra Geetesh Kumar, 2017, Recent Pat Biotechnol, V11, P188, DOI [10.2174/1872208311666170120121025, 10.2174/1872208311666170120121025]"
" Mummey DL, 2002, APPL SOIL ECOL, V21, P251, DOI 10.1016/S0929-1393(02)00090-2"
" Nagajyoti PC, 2010, ENVIRON CHEM LETT, V8, P199, DOI 10.1007/s10311-010-0297-8"
" Nanda SP, 2024, ENVIRON MONIT ASSESS, V196, DOI 10.1007/s10661-024-13166-0"
" Ng CWW, 2022, SCI REP-UK, V12, DOI 10.1038/s41598-022-11170-3"
" Nie CR, 2018, CHEMOSPHERE, V200, P274, DOI 10.1016/j.chemosphere.2018.02.134"
" Oves M, 2019, INT J ENVIRON SCI TE, V16, P6967, DOI 10.1007/s13762-019-02300-y"
" Peng HB, 2017, ENVIRON POLLUT, V229, P846, DOI 10.1016/j.envpol.2017.07.004"
" Qi X, 2021, J HAZARD MATER, V401, DOI 10.1016/j.jhazmat.2020.123823"
" Rahi AA, 2022, SAUDI J BIOL SCI, V29, P743, DOI 10.1016/j.sjbs.2021.09.035"
" Ren J, 2020, J SOIL SEDIMENT, V20, P3041, DOI 10.1007/s11368-019-02403-6"
" Schommer VA, 2023, SCI TOTAL ENVIRON, V881, DOI 10.1016/j.scitotenv.2023.163385"
" Siddika A, 2022, Indonesian Journal of Social and Environmental Issues (IJSEI), V3, P19, DOI [10.47540/ijsei.v3i1.417, 10.47540/ijsei.v3i1.417, DOI 10.47540/IJSEI.V3I1.417]"
" Sivaranjanee R, 2023, CARBON LETT, V33, P1407"
" Siwik-Ziomek A, 2024, AGRICULTURE-BASEL, V14, DOI 10.3390/agriculture14081255"
" Sungur A, 2015, SOIL SEDIMENT CONTAM, V24, P1, DOI 10.1080/15320383.2014.907238"
" Tripathi M, 2023, FRONT MICROBIOL, V14, DOI 10.3389/fmicb.2023.1168954"
" Tu C, 2020, ENVIRON INT, V137, DOI 10.1016/j.envint.2020.105576"
" Vandeuren A, 2023, SCI TOTAL ENVIRON, V902, DOI 10.1016/j.scitotenv.2023.166073"

"   Vera-Bernal M, 2021, MICROBIOL RES-ITALY, V12, P354, DOI 10.3390/microbiolres12020024"
"   Verma S, 2023, CLEAN TECHNOL ENVIR, V25, P1485, DOI 10.1007/s10098-021-02029-8"
"   Wang AN, 2024, PLANT SOIL, V494, P413, DOI 10.1007/s11104-023-06290-2"
"   Wang K, 2024, ENVIRON RES, V240, DOI 10.1016/j.envres.2023.117426"
"   Wang Y, 2020, ECOTOX ENVIRON SAFE, V192, DOI 10.1016/j.ecoenv.2020.110294"
"   Wang Y, 2022, ENVIRON GEOTECH, V9, P135, DOI 10.1680/jenge.18.00091"
"   Wang Y, 2021, SCI TOTAL ENVIRON, V774, DOI 10.1016/j.scitotenv.2021.145136"
"   Wei T, 2022, WATER AIR SOIL POLL, V233, DOI 10.1007/s11270-022-05934-w"
"   Wei T, 2022, J HAZARD MATER, V435, DOI 10.1016/j.jhazmat.2022.129006"
"   Wei T, 2022, ENVIRON SCI POLLUT R, V29, P63886, DOI 10.1007/s11356-022-20344-5"
"   Wu LP, 2019, J CLEAN PROD, V235, P901, DOI 10.1016/j.jclepro.2019.07.043"
"   Wu P, 2021, J HAZARD MATER, V416, DOI 10.1016/j.jhazmat.2021.125915"
"   Wu P, 2020, BIOCHAR, V2, P135, DOI 10.1007/s42773-020-00055-1"
"   Wu YF, 2022, RESOUR CONSERV RECY, V181, DOI 10.1016/j.resconrec.2022.106261"
"   Xiao R, 2019, ECOTOX ENVIRON SAFE, V181, P155, DOI 10.1016/j.ecoenv.2019.06.005"
"   Xu KL, 2024, J ENVIRON CHEM ENG, V12, DOI 10.1016/j.jece.2024.114357"
"   Xu M, 2018, CHEM ECOL, V34, P177, DOI 10.1080/02757540.2017.1404992"
"   Xu YL, 2024, FERMENTATION-BASEL, V10, DOI 10.3390/fermentation10010041"
"   Yao XH, 2006, EUR J SOIL BIOL, V42, P120, DOI 10.1016/j.ejsobi.2005.12.001"
"   Yin GC, 2023, CHEM ENG J, V466, DOI 10.1016/j.cej.2023.143199"
"   Yin YC, 2015, RSC ADV, V5, P47584, DOI 10.1039/c5ra05717e"
"   Zeng XY, 2022, BIOCHAR, V4, DOI 10.1007/s42773-022-00143-4"
"   Zerizghi T, 2022, SCI TOTAL ENVIRON, V814, DOI 10.1016/j.scitotenv.2021.152653"
"   Zhang H, 2020, J HAZARD MATER, V390, DOI 10.1016/j.jhazmat.2019.121349"
"   Zhang JG, 2020, J HAZARD MATER, V385, DOI 10.1016/j.jhazmat.2019.121588"
"   Zhang J, 2018, INT J PHYTOREMEDIAT, V20, P616, DOI 10.1080/15226514.2017.1413328"
"   Zhang R, 2024, FRONT MICROBIOL, V15, DOI 10.3389/fmicb.2024.1376849"
"   Zheng LG, 2019, ANN MICROBIOL, V69, P849, DOI 10.1007/s13213-019-01478-3"
"   Zhong MT, 2023, ECOTOX ENVIRON SAFE, V262, DOI 10.1016/j.ecoenv.2023.115317"
"   Zhou YC, 2023, SCI TOTAL ENVIRON, V861, DOI 10.1016/j.scitotenv.2022.160649"
"   Zhu JP, 2024, HORTICULTURAE, V10, DOI 10.3390/horticulturae10060592"
NR 103
TC 1
Z9 1
U1 20
U2 20
PU SPRINGER INT PUBL AG
PI CHAM
"PA GEWERBESTRASSE 11, CHAM, CH-6330, SWITZERLAND"
SN 0049-6979
EI 1573-2932
J9 WATER AIR SOIL POLL
JI Water Air Soil Pollut.
PD OCT 6
PY 2025
VL 236
IS 15
AR 979
DI 10.1007/s11270-025-08667-8
PG 21
WC Environmental Sciences; Meteorology & Atmospheric Sciences; Water
   Resources
WE Science Citation Index Expanded (SCI-EXPANDED)
SC Environmental Sciences & Ecology; Meteorology & Atmospheric Sciences;
   Water Resources
GA 8HW1Z
UT WOS:001589044100018
DA 2026-04-06
ER

PT J
"AU Cui, ZJ"
"   Zhang, X"
"   Yang, HH"
"   Sun, LQ"
"AF Cui, Zhaojie"
"   Zhang, Xu"
"   Yang, Huanhuan"
"   Sun, Liqun"
TI Bioremediation of heavy metal pollution utilizing composite microbial
"   agent of Mucor circinelloides, Actinomucor sp. and"
   Mortierella sp.
SO JOURNAL OF ENVIRONMENTAL CHEMICAL ENGINEERING
LA English
DT Article
DE Heavy metal; Microbial agents; Microbial community; In situ remediation
ID MINE TAILINGS; SOILS; ACID; COPPER; BIOAVAILABILITY; MICROORGANISMS;
   LEACHABILITY; REMEDIATION
"AB Microbial remediation of local microbes shows great application prospects for in situ remediation for heavy metal pollution especially in harsh soil conditions. Thermodynamics and kinetics of adsorption of Mucor circinelloides, Actinomucor sp and Mortierella sp were consistent with Langmuir adsorption isothermal model and Pseudo-second order kinetics model. Synergies of specific enzymes and organic acids may be the main physiological processes of microbial remediation. The efficiency of immobilization of heavy metals is up to 74.98% (Zn), 85.29% (Pb) and 79.41% (Mn) which shows high bioremediation effect of the composite microbial

agent. Microbial remediation of Mucor circinelloides, Actinomucor sp and Mortierella sp not only effectively reduce the heavy metal activity, but also significantly shorten the remediation period and improve the poor habitat of mine tailings. More interestingly, the study reveals that microbial diversity and community structure sensitively respond to the bioremediation process. This study demonstrates that changes of microbial community can be used to real-time monitoring the bioremediation process."
"C1 [Cui, Zhaojie; Zhang, Xu; Sun, Liqun] Shandong Univ, Sch Environm Sci & Engn, Jinan 250100, Shandong, Peoples R China."
"   [Yang, Huanhuan] Shandong Univ, Sch Life Sci, Jinan 250100, Shandong, Peoples R China."
C3 Shandong University; Shandong University
"RP Cui, ZJ (corresponding author), Shandong Univ, Sch Environm Sci & Engn, Jinan 250100, Shandong, Peoples R China."
EM cuizj@sdu.edu.cn
"RI Sun, Liqun/KTI-6521-2024"
FU Ministry of Science and Technology of China
FX This research was funded by Ministry of Science and Technology of China: The remediation technologies and demonstration for Anshan mine tailings. Special thanks are extended to School of Life Sciences in Shandong University for their suggestions and support to improve the basic research.
"CR [Anonymous], 2012, APPL ENVIRON SOIL SC, DOI DOI 10.1155/2012/395964"
"   Beste DJV, 2007, J BACTERIOL, V189, P3969, DOI 10.1128/JB.01787-06"
"   Chen XC, 2009, BIORESOURCE TECHNOL, V100, P330, DOI 10.1016/j.biortech.2008.04.051"
"   Dowd SE, 1998, APPL ENVIRON MICROB, V64, P333"
"   Gobi KV, 2007, SENSOR ACTUAT B-CHEM, V123, P583, DOI 10.1016/j.snb.2006.09.056"
"   Grzegorczyk S, 2014, J ELEMENTOL, V19, P109, DOI 10.5601/jelem.2014.19.1.583"
"   Guittonny-Philippe A, 2014, ENVIRON INT, V64, P1, DOI 10.1016/j.envint.2013.11.016"
"   Albarrac??n VH, 2010, INT J SYST EVOL MICR, V60, P397, DOI 10.1099/ijs.0.010587-0"
"   Hiol A, 2000, ENZYME MICROB TECH, V26, P421, DOI 10.1016/S0141-0229(99)00173-8"
"   Hseu ZY, 2010, SOIL SCI PLANT NUTR, V56, P31, DOI 10.1111/j.1747-0765.2009.00442.x"
"   Karami H, 2004, ANAL CHIM ACTA, V509, P89, DOI 10.1016/j.aca.2003.12.022"
"   Kidgell JT, 2014, PLOS ONE, V9, DOI 10.1371/journal.pone.0094706"
"   Lalev AI, 2000, J MOL BIOL, V302, P65, DOI 10.1006/jmbi.2000.4015"
"   Levin JD, 2006, NUCLEIC ACIDS RES, V34, DOI 10.1093/nar/gkl756"
"   Li JH, 2015, MICROCHEM J, V122, P89, DOI 10.1016/j.microc.2015.04.015"
"   Lim M, 2009, INT J ENV RES PUB HE, V6, P2865, DOI 10.3390/ijerph6112865"
"   Mascio CTM, 2007, ANTIMICROB AGENTS CH, V51, P4255, DOI 10.1128/AAC.00824-07"
"   Mercier A, 2013, CHEMOSPHERE, V92, P787, DOI 10.1016/j.chemosphere.2013.04.016"
"   Olaniran AO, 2013, INT J MOL SCI, V14, P10197, DOI 10.3390/ijms140510197"
"   Pathak H, 1998, SOIL BIOL BIOCHEM, V30, P695, DOI 10.1016/S0038-0717(97)00208-3"
"   Polti MA, 2011, WATER AIR SOIL POLL, V214, P49, DOI 10.1007/s11270-010-0401-5"
"   Rai A, 2010, TOXICOL SCI, V118, P586, DOI 10.1093/toxsci/kfq266"
"   Rosario K, 2007, J ENVIRON QUAL, V36, P1249, DOI 10.2134/jeq2006.0315"
"   Rudzinski W, 2009, ADSORPTION, V15, P181, DOI 10.1007/s10450-009-9167-8"
"   Shirai K, 2001, ENZYME MICROB TECH, V28, P446, DOI 10.1016/S0141-0229(00)00338-0"
"   Singh J, 2013, ENVIRON SCI POLLUT R, V20, P8974, DOI 10.1007/s11356-013-1848-x"
"   Song XL, 2013, J COLLOID INTERF SCI, V389, P213, DOI 10.1016/j.jcis.2012.08.060"
"   Sucharov?? J, 2011, APPL GEOCHEM, V26, P1205, DOI 10.1016/j.apgeochem.2011.04.009"
"   van den Bosch PLF, 2009, APPL MICROBIOL BIOT, V83, P579, DOI 10.1007/s00253-009-1951-6"
"   Yanai J, 1998, SOIL SCI PLANT NUTR, V44, P367, DOI 10.1080/00380768.1998.10414458"
"   Yin X, 2015, BIOTECHNOL ADV, V33, P830, DOI 10.1016/j.biotechadv.2015.04.006"
"   Zhan J, 2011, J ENVIRON SCI-CHINA, V23, P476, DOI 10.1016/S1001-0742(10)60433-0"
"   Zhang H, 2017, CHEM ENG J, V313, P157, DOI 10.1016/j.cej.2016.12.067"
"   Zhang Xi Zhang Xi, 2010, Environmental Science & Technology (China), V33, P106"
"   Zhang X, 2017, TOXICOL RES-UK, V6, P442, DOI 10.1039/c7tx00110j"
"   Zhang X, 2016, RSC ADV, V6, P27963, DOI 10.1039/c6ra00765a"
NR 36
TC 66
Z9 86
U1 10
U2 295
PU ELSEVIER SCI LTD
PI OXFORD
"PA THE BOULEVARD, LANGFORD LANE, KIDLINGTON, OXFORD OX5 1GB, OXON, the United Kingdom"
EI 2213-3437
J9 J ENVIRON CHEM ENG
JI J. Environ. Chem. Eng.
PD AUG
PY 2017
VL 5
IS 4
BP 3616
EP 3621
DI 10.1016/j.jece.2017.07.021
PG 6
"WC Engineering, Environmental; Engineering, Chemical"
WE Science Citation Index Expanded (SCI-EXPANDED)
SC Engineering
GA FH4QO
UT WOS:000411143200059
DA 2026-04-06
ER

PT J
"AU Agrawal, K"

"   Ruhil, T"
"   Gupta, VK"
"   Verma, P"
"AF Agrawal, Komal"
"   Ruhil, Tannu"
"   Gupta, Vijai Kumar"
"   Verma, Pradeep"
TI Microbial assisted multifaceted amelioration processes of heavy-metal
   remediation: a clean perspective toward sustainable and greener future
SO CRITICAL REVIEWS IN BIOTECHNOLOGY
LA English
DT Review
DE Heavy metals; pollution; microbial remediation; sustainability;
   nanotechnology; LCA; TEA
ID PRINTED-CIRCUIT BOARDS; AQUEOUS-SOLUTIONS; BACTERIAL COMMUNITY;
   CONTAMINATED SOILS; BIOSORPTION; WASTE; BIOREMEDIATION; REMOVAL;
   TOLERANCE; BIOMASS
"AB Rapidly increasing heavy metal waste has adversely affected the environment and the Earth's health. The lack of appropriate remediation technologies has worsened the issue globally, especially in developing countries. Heavy-metals contaminants have severely impacted the environment and led to devastating conditions owing to their abundance and reactivity. As they are nondegradable, the potential risk increases even at a low concentration. However, heavy-metal remediation has increased with the up-gradation of technologies and integration of new approaches. Also, of all the treatment methodologies, microbial-assisted multifaceted approach for ameliorating heavy metals is a promising strategy for propagating the idea of a green and sustainable environment with minimal waste aggregation. Microbial remediation combined with different biotechniques could aid in unraveling new methods for eradicating heavy metals. Thus, the present review focuses on various microbial remediation approaches and their affecting factors, enabling recapitulation of the interplay between heavy-metals ions and microorganisms. Additionally, heavy-metals remediation mechanisms adapted by microorganisms, the role of genetically modified (GM) microorganisms, life cycle assessment (LCA), techno-economic assessment (TEA) limitations, and prospects of microbial-assisted amelioration of heavy-metals have been elaborated in the current review with focus toward ""sustainable and greener future."""
"C1 [Agrawal, Komal; Ruhil, Tannu; Verma, Pradeep] Cent Univ Rajasthan, Dept Microbiol, Bioproc & Bioenergy Lab, Ajmer, India."
"   [Agrawal, Komal] Lovely Profess Univ, Sch Bio Engn & Biosci, Dept Microbiol, Phagwara, India."
"   [Gupta, Vijai Kumar] SRUC, Ctr Safe & Improved Food, Edinburgh, the United Kingdom."
"   [Gupta, Vijai Kumar] SRUC, Biorefining & Adv Mat Res Ctr, Edinburgh, the United Kingdom."
"   [Verma, Pradeep] Cent Univ Rajasthan, Dept Microbiol, Bioproc & Bioenergy Lab, Ajmer 305817, Rajasthan, India."
C3 Central University of Rajasthan (CURAJ); Lovely Professional University;
   Central University of Rajasthan (CURAJ)
"RP Verma, P (corresponding author), Cent Univ Rajasthan, Dept Microbiol, Bioproc & Bioenergy Lab, Ajmer 305817, Rajasthan, India."
EM pradeepverma@curaj.ac.in
"RI agrawal, Komal/HHR-9684-2022; Gupta, Vijai Kumar/O-2445-2014"
"OI agrawal, Komal/0000-0003-2317-7928; "
"CR Abd Elhafez SE, 2017, ENVIRON SCI POLLUT R, V24, P1397, DOI 10.1007/s11356-016-7891-7"
"   Abdel-Monem MO, 2010, WATER SCI TECHNOL, V61, P2994, DOI 10.2166/wst.2010.198"
"   Abdi O., 2015, Journal of Materials and Environmental Sciences, V6, P1386"
"   Acar FN, 2004, BIORESOURCE TECHNOL, V94, P13, DOI 10.1016/j.biortech.2003.10.032"
"   Adama M, 2016, J ENVIRON PUBLIC HEA, V16, P1, DOI DOI 10.1155/2016/"
"   Adeleke R, 2017, S AFR J BOT, V108, P393, DOI 10.1016/j.sajb.2016.09.002"
"   Al-Qodah Z, 2006, DESALINATION, V196, P164, DOI 10.1016/j.desal.2005.12.012"
"   Alam P., 2013, International Journal of Sustainable Development and Green Economics IJSDGE, V2, P165, DOI DOI 10.1504/IJSDEGE.2013.055650"
"   Albert Q, 2018, CHEMOSPHERE, V196, P386, DOI 10.1016/j.chemosphere.2017.12.156"
"   Alengebawy A, 2021, TOXICS, V9, DOI 10.3390/toxics9030042"
"   Ali H, 2019, J CHEM-NY, V2019, DOI 10.1155/2019/6730305"
"   Alkorta I, 2017, FEMS MICROBIOL LETT, V364, DOI 10.1093/femsle/fnx200"
"   Alloway B., 2013, Heavy Metals in Soils. Environmental Pollution, P195, DOI [DOI 10.1007/978-94-007-4470-7_7, 10.1007/978-94-007-4470-77, DOI 10.1007/978-94-007-4470-77, 10.1007/978-94-007-4470-7_7]"
"   Alvarez A, 2017, CHEMOSPHERE, V166, P41, DOI 10.1016/j.chemosphere.2016.09.070"
"   [Anonymous], 2006, FEDERAL REMEDIATION"
"   [Anonymous], 2018, The Effects of pH on Microbial Growth"
"   Aryal M., 2013, Journal of Water Sustainability, V3, P117, DOI DOI 10.1016/J.CEJ.2010.05.026"
"   Aryal M, 2015, ENVIRON MONIT ASSESS, V187, DOI 10.1007/s10661-014-4173-z"
"   Awasthi AK, 2019, EMERGING ECO FRIENDL, P59"
"   Azubuike CC, 2016, WORLD J MICROB BIOT, V32, DOI 10.1007/s11274-016-2137-x"
"   Baker-Austin C, 2007, TRENDS MICROBIOL, V15, P165, DOI 10.1016/j.tim.2007.02.005"
"   Balali-Mood M, 2021, FRONT PHARMACOL, V12, DOI 10.3389/fphar.2021.643972"
"   Barakat MA, 2011, ARAB J CHEM, V4, P361, DOI 10.1016/j.arabjc.2010.07.019"
"   Barkay T, 2003, FEMS MICROBIOL REV, V27, P355, DOI 10.1016/S0168-6445(03)00046-9"
"   Beames A, 2015, J CLEAN PROD, V101, P109, DOI 10.1016/j.jclepro.2015.03.073"
"   Bilal M, 2013, J HAZARD MATER, V263, P322, DOI 10.1016/j.jhazmat.2013.07.071"
"   Bizily SP, 1999, P NATL ACAD SCI USA, V96, P6808, DOI 10.1073/pnas.96.12.6808"
"   Brim H, 2000, NAT BIOTECHNOL, V18, P85, DOI 10.1038/71986"
"   Bwapwa J. K., 2017, South African Journal of Chemical Engineering, V24, P62, DOI [10.1016/j.sajce.2017.06.005, 10.1016/j.sajce.2017.06.005]"
"   Cespi D, 2016, GREEN CHEM, V18, P1625, DOI [10.1039/C5GC02148K, 10.1039/c5gc02148k]"
"   Cespi D, 2015, GREEN CHEM, V17, P343, DOI 10.1039/c4gc01497a"
"   Cespi D, 2015, GREEN CHEM, V17, P3390, DOI 10.1039/c5gc00424a"
"   Chaturvedi AD, 2015, WORLD J MICROB BIOT, V31, P1595, DOI 10.1007/s11274-015-1911-5"
"   Chen C, 2020, J CLEAN PROD, V263, DOI 10.1016/j.jclepro.2020.121422"
"   CHIBUIKE G, 2014, APPL ENVIRON SOIL SC, V2014, P1, DOI [10.1155/2014/752708, DOI 10.1155/2014/752708]"

"   Davis SC, 2016, J ENVIRON STUD SCI, V6, P11, DOI [10.1007/s13412-016-0370-0, DOI 10.1007/S13412-016-0370-0, 10.1007/s13412-016-0370-0]"
"   De J, 2008, MAR BIOTECHNOL, V10, P471, DOI 10.1007/s10126-008-9083-z"
"   Silva AADE, 2012, BRAZ J MICROBIOL, V43, P1620, DOI [10.1590/S1517-83822012000400047, 10.1590/S1517-83822012000400047]"
"   de Wet M MM., 2021, Fungi Bio-Prospects in Sustainable Agriculture, Environment and Nanotechnology, P407, DOI DOI 10.1016/B978-0-12-821925-6.00018-6"
"   Delangiz N, 2020, MOLECULAR ASPECTS OF PLANT BENEFICIAL MICROBES IN AGRICULTURE, P417, DOI 10.1016/B978-0-12-818469-1.00034-1"
"   Diep P, 2018, FRONT BIOENG BIOTECH, V6, DOI 10.3389/fbioe.2018.00157"
"   Dipak Paul Dipak Paul, 2017, Annals of Agrarian Science, V15, P278, DOI [10.1016/j.aasci.2017.04.001, 10.1016/j.aasci.2017.04.001]"
"   D??nmez G??, 1999, PROCESS BIOCHEM, V34, P885"
"   Dubey SK, 2011, AFR J BIOTECHNOL, V10, P1125"
"   Dwivedi S., 2012, Journal of Advance Laboratory Research in Biology, V3, P229"
"   Dzyadevych S., 2014, Biological Identification, P153, DOI DOI 10.1533/9780857099167.2.153"
"   Edwards SJ, 2013, APPL MICROBIOL BIOT, V97, P9909, DOI 10.1007/s00253-013-5216-z"
"   Efaq AN., 2015, 3 INT C SUST SOL WAS, P2"
"   El-Hendawy HH, 2009, EGYPT ACAD J BIOL SC, V1, P23, DOI 10.21608/eajbsg.2009.16714"
"   El-Kassas HY, 2016, ACTA OCEANOL SIN, V35, P89, DOI 10.1007/s13131-016-0880-3"
"   Espinoza, 2014, CHEM WASTE IMPACT AQ"
"   Farhan SN, 2015, INT J IND CHEM, V6, P119, DOI 10.1007/s40090-015-0038-8"
"   Farina M, 2011, LIFE SCI, V89, P555, DOI 10.1016/j.lfs.2011.05.019"
"   Farzadkia M, 2022, J HAZARD MATER, V425, DOI 10.1016/j.jhazmat.2021.127969"
"   Fashola MO, 2016, INT J ENV RES PUB HE, V13, DOI 10.3390/ijerph13111047"
"   Fern??ndez-Calvi??o D, 2010, FEMS MICROBIOL ECOL, V73, P149, DOI 10.1111/j.1574-6941.2010.00873.x"
"   Fran??ois F, 2012, APPL ENVIRON MICROB, V78, P1097, DOI 10.1128/AEM.06522-11"
"   Frederick TM, 2013, BIOTECHNOL LETT, V35, P1291, DOI 10.1007/s10529-013-1200-z"
"   Gavrilescu M, 2004, ENG LIFE SCI, V4, P219, DOI 10.1002/elsc.200420026"
"   Ghosh M., 2005, ASIAN J ENV SCI, V6, p214 231"
"   Girolkar S., 2021, BIOREMEDIATION ENV S, P247, DOI DOI 10.1016/B978-0-12-820318-7.00012-5"
"   Goswami RK, 2022, J CLEAN PROD, V339, DOI 10.1016/j.jclepro.2022.130654"
"   Gupta R, 2000, CURR SCI INDIA, V78, P967"
"   Gupta S, 2015, INT J ENVIRON SCI TE, V12, P995, DOI 10.1007/s13762-013-0484-9"
"   HAMMOUDA O, 1995, ECOTOX ENVIRON SAFE, V31, P205, DOI 10.1016/j.sjbs.2012.04.005"
"   He ZQ, 2011, CHEMOSPHERE, V83, P1249, DOI 10.1016/j.chemosphere.2011.03.004"
"   He ZLL, 2005, J TRACE ELEM MED BIO, V19, P125, DOI 10.1016/j.jtemb.2005.02.010"
"   Hossain MS, 2011, WASTE MANAGE, V31, P754, DOI 10.1016/j.wasman.2010.11.008"
"   Huang JH, 2017, CHEMOSPHERE, V173, P199, DOI 10.1016/j.chemosphere.2016.12.137"
"   Igiri BE, 2018, J TOXICOL-US, V2018, DOI 10.1155/2018/2568038"
"   Ihedioha JN, 2017, ENVIRON GEOCHEM HLTH, V39, P497, DOI 10.1007/s10653-016-9830-4"
"   Iriventi, 2016, CLIN SOLID WASTE MAN"
"   Jagdale S, 2018, BIOSTIMULATION REMED, P259"
"   Jerez CA., 2011, COMPREHENSIVE BIOTEC, V3"
"   Jin QS, 2018, FRONT ENV SCI-SWITZ, V6, DOI 10.3389/fenvs.2018.00101"
"   Jin YY, 2018, APPL SCI-BASEL, V8, DOI 10.3390/app8081336"
"   Joint World Health Organization, 2007, EUR065067592 JOINT W"
"   Joo JO, 2015, BIOTECHNOL BIOPROC E, V20, P937, DOI 10.1007/s12257-015-0287-6"
"   Joshi PM, 2009, ENVIRON SCI TECHNOL, V43, P5884, DOI 10.1021/es900063b"
"   Juwarkar AA, 2007, CHEMOSPHERE, V68, P1996, DOI 10.1016/j.chemosphere.2007.02.027"
"   Juwarkar AA, 2010, BIOREMEDIATION TECHNOLOGY: RECENT ADVANCES, P266"
"   Kachenko A, 2006, WATER AIR SOIL POLL, V169, P101, DOI 10.1007/s11270-006-2027-1"
"   Kaewdoung B, 2016, GEOMICROBIOL J, V33, P283, DOI 10.1080/01490451.2015.1048394"
"   Kalin M, 2006, SCI TOTAL ENVIRON, V366, P395, DOI 10.1016/j.scitotenv.2005.11.015"
"   Kapahi M, 2019, J HEALTH POLLUT, V9, DOI 10.5696/2156-9614-9.24.191203"
"   Karwowska E, 2014, J HAZARD MATER, V264, P203, DOI 10.1016/j.jhazmat.2013.11.018"
"   Kirpichtchikova TA, 2006, GEOCHIM COSMOCHIM AC, V70, P2163, DOI 10.1016/j.gca.2006.02.006"
"   Klemes JJ, 2020, RENEW SUST ENERG REV, V127, DOI 10.1016/j.rser.2020.109883"
"   Kogej A, 2001, CHEM BIOCHEM ENG Q, V15, P75"
"   Kulkarni BN, 2020, SCI TOTAL ENVIRON, V743, DOI 10.1016/j.scitotenv.2020.140693"
"   Kumar A, 2019, FUNG BIOL-US, P437, DOI 10.1007/978-3-030-25506-0_18"
"   Kumar B, 2021, ENERG CONVERS MAN-X, V10, DOI 10.1016/j.ecmx.2020.100063"
"   Kumar NM, 2018, ENERGY ENV SUSTAIN, P53, DOI 10.1007/978-981-10-7485-1_4"
"   Kushwaha A, 2017, ENVIRON SCI POLLUT R, V24, P10652, DOI 10.1007/s11356-017-8752-8"
"   Lakherwal D., 2014, INT J INNOVATIVE RES, V4, P41, DOI [DOI 10.1016/J.MATPR.2019.07.462, 10.1016/j.matpr.2019.07.462]"
"   Lal RM, 2016, ENVIRON RES LETT, V11, DOI 10.1088/1748-9326/11/10/104009"
"   Lauber CL, 2009, APPL ENVIRON MICROB, V75, P5111, DOI 10.1128/AEM.00335-09"
"   Lee BK, 2004, WASTE MANAGE, V24, P143, DOI 10.1016/j.wasman.2003.10.008"
"   Lenart-Boron A, 2015, PLANT SOIL ENVIRON, V61, P273, DOI 10.17221/217/2015-PSE"
"   Lennon JT, 2011, NAT REV MICROBIOL, V9, P119, DOI 10.1038/nrmicro2504"
"   Leong YK, 2020, BIORESOURCE TECHNOL, V303, DOI 10.1016/j.biortech.2020.122886"
"   Li H, 2015, J BASIC MICROB, V55, P398, DOI [10.1002/jobm.201300670, 10.1002/jobm.201570032]"
"   Li J, 2010, INNOV FOOD SCI EMERG, V11, P637, DOI 10.1016/j.ifset.2010.06.004"
"   Li SZ, 2020, J HAZARD MATER, V400, DOI 10.1016/j.jhazmat.2020.123255"
"   Liu LN, 2019, SCI TOTAL ENVIRON, V667, P444, DOI 10.1016/j.scitotenv.2019.02.390"
"   Liu P, 2021, BIOCHEM ENG J, V166, DOI 10.1016/j.bej.2020.107856"
"   Lloyd PJ., 2001, EUROPEAN J POLITICAL, V17, P327"
"   Machado MD, 2010, J HAZARD MATER, V180, P347, DOI 10.1016/j.jhazmat.2010.04.037"
"   Mane P. C., 2011, Advances in Applied Science Research, V2, P202"
"   Martins M, 2017, WATER RES, V108, P160, DOI 10.1016/j.watres.2016.10.071"
"   Mateos LM, 2017, ADV APPL MICROBIOL, V99, P103, DOI 10.1016/bs.aambs.2017.01.001"
"   M??th?? I, 2012, INT BIODETER BIODEGR, V73, P41, DOI 10.1016/j.ibiod.2012.05.018"
"   Maznah WOW, 2012, J ENVIRON SCI, V24, P1386, DOI 10.1016/S1001-0742(11)60931-5"

"   McKone TE, 2011, ENVIRON SCI TECHNOL, V45, P1751, DOI 10.1021/es103579c"
"   Megharaj M., 2014, Encyclopedia of Toxicology, P485, DOI [DOI 10.1016/B978-0-12-386454-3.01001-0, 10.1016/B978-0-12-3864543.01001-0, DOI 10.1016/B978-0-12-3864543.01001-0]"
"   Miller D.Semmens., 2002, Waste management in aquaculture, P8"
"   Mishra D, 2005, MET MATER INT, V11, P249, DOI 10.1007/BF03027450"
"   Mishra Shraddha, 2022, Environmental Nanotechnology, Monitoring and Management, DOI [10.1016/j.enmm.2021.100632, 10.1016/j.enmm.2021.100632]"
"   Mohamed A. T., 2011, Biotechnology, V10, P274"
"   Mohd S, 2019, ECOTOX ENVIRON SAFE, V176, P108, DOI 10.1016/j.ecoenv.2019.03.053"
"   Morton-Bermea O, 2002, B ENVIRON CONTAM TOX, V68, P383, DOI [10.1007/s00128-001-0265-x, 10.1007/s001280265]"
"   Mustapha MU, 2015, PROCEDIA ENVIRON SCI, V30, P33, DOI 10.1016/j.proenv.2015.10.006"
"   Nair A, 2008, J HAZARD MATER, V152, P545, DOI 10.1016/j.jhazmat.2007.07.054"
"   Nanda M, 2019, AQUAT TOXICOL, V212, P1, DOI 10.1016/j.aquatox.2019.04.011"
"   Nanda S, 2021, ENVIRON CHEM LETT, V19, P1433, DOI 10.1007/s10311-020-01100-y"
"   Narayanasamy M, 2018, INT J ENVIRON SCI TE, V15, P119, DOI 10.1007/s13762-017-1372-5"
"   Naseem M., 2021, Fungi Bio-Prospects in Sustainable Agriculture, Environment and Nano-Technology, P433"
"   Nawaz T, 2020, PROCESSES, V8, DOI 10.3390/pr8040384"
"   Nemathaga F, 2008, WASTE MANAGE, V28, P1236, DOI 10.1016/j.wasman.2007.03.033"
"   Nie J, 2020, SCI TOTAL ENVIRON, V707, DOI 10.1016/j.scitotenv.2019.136080"
"   Niti C, 2013, RES J CHEM ENVIRON, V17, P88"
"   NRIAGU JO, 1989, NATURE, V338, P47, DOI 10.1038/338047a0"
"   Obi FO, 2016, Nigerian Journal of Technology, V35, P957, DOI [10.4314/njt.v35i4.34, 10.4314/njt.v35i4.34, DOI 10.4314/NJT.V35I4.34, 10.4314/njt.v35i4]"
"   Parimala G.S., 2020, MICROORGANISMS SUSTA, P119, DOI DOI 10.1016/B978-0-12-819001-2.00006-1"
"   Parvin F, 2019, MICRO NANO TECHNOL, P137, DOI 10.1016/B978-0-12-813902-8.00008-3"
"   Passarini F, 2014, WASTE MANAGE, V34, P753, DOI 10.1016/j.wasman.2013.12.022"
"   Perpetuo EA, 2011, PROGRESS IN MOLECULAR AND ENVIRONMENTAL BIOENGINEERING - FROM ANALYSIS AND MODELING TO TECHNOLOGY APPLICATIONS, P605"
"   Pietik??inen J, 2005, FEMS MICROBIOL ECOL, V52, P49, DOI 10.1016/j.femsec.2004.10.002"
"   Polcaro AM., 2005, HEAVY METALS ENV ORI, P2579"
"   Pollmann K, 2018, BIOTECHNOL ADV, V36, P1048, DOI 10.1016/j.biotechadv.2018.03.006"
"   Priyadarshanee M, 2021, J ENVIRON CHEM ENG, V9, DOI 10.1016/j.jece.2020.104686"
"   Purchase D, 2009, J APPL MICROBIOL, V106, P1163, DOI 10.1111/j.1365-2672.2008.04082.x"
"   Qin J, 2009, P NATL ACAD SCI USA, V106, P5213, DOI 10.1073/pnas.0900238106"
"   Rahimi S, 2020, J CLEAN PROD, V248, DOI 10.1016/j.jclepro.2019.119186"
"   Ram??rez-Rodr??guez AE, 2019, INT J PHYTOREMEDIAT, V21, P617, DOI 10.1080/15226514.2018.1546274"
"   Rana S, 2020, J ENVIRON SCI, V89, P47, DOI 10.1016/j.jes.2019.09.023"
"   Rani A, 2009, MICROBIAL STRATEGIES FOR CROP IMPROVEMENT, P85, DOI 10.1007/978-3-642-01979-1_5"
"   Rasoulnia P, 2016, WASTE MANAGE, V52, P309, DOI 10.1016/j.wasman.2016.04.004"
"   Rayu S, 2012, BIODEGRADATION, V23, P917, DOI 10.1007/s10532-012-9576-3"
"   Righi S., 2019, LIFE CYCLE ASSESS EN, P139, DOI [DOI 10.1007/978-3-319-93740-3_9, 10.1007/978-3-319-93740-3, DOI 10.1007/978-3-319-93740-3]"
"   Ripp S, 2000, ENVIRON SCI TECHNOL, V34, P846, DOI 10.1021/es9908319"
"   Rockne KarlJ., 2003, BIOREMEDIATION CONTA"
"   Rohwerder T, 2003, APPL MICROBIOL BIOT, V63, P239, DOI 10.1007/s00253-003-1448-7"
"   Rojas LA, 2011, PLOS ONE, V6, DOI 10.1371/journal.pone.0017555"
"   RoyChowdhury A., 2018, GREEN CHEM, P359, DOI [10.1016/B978-0-12-809270-5.00015-7, DOI 10.1016/B978-0-12-809270-5.00015-7]"
"   Rozman U, 2020, ENVIRON TECHNOL INNO, V18, DOI 10.1016/j.eti.2020.100742"
"   Sanscartier D, 2011, SOIL SEDIMENT CONTAM, V20, P353, DOI 10.1080/15320383.2011.571311"
"   Satpati G.G., 2021, POLLUT RES, V40, P259"
"   Sayler GS, 2000, CURR OPIN BIOTECH, V11, P286, DOI 10.1016/S0958-1669(00)00097-5"
"   Shah V, 2020, ENVIRON TECHNOL INNO, V18, DOI 10.1016/j.eti.2020.100774"
"   SHARMA GK., 2013, International Journal of Environmental Engineering and Management, V4, P573"
"   Sharma I., 2020, ICINDE TRACE METALS, DOI DOI 10.5772/INTECHOPEN.90453"
"   Shen N., 2017, Chem. Eng. Trans, V61, P25, DOI DOI 10.3303/CET1761002"
"   Singh J, 2018, J NANOBIOTECHNOL, V16, DOI 10.1186/s12951-018-0408-4"
"   Singh S, 2020, ENVIRON SCI POLLUT R, V27, P27181, DOI 10.1007/s11356-019-04974-w"
"   Singh S, 2020, SCI TOTAL ENVIRON, V709, DOI 10.1016/j.scitotenv.2019.135895"
"   Sn C, 2017, Recent Advances in Petrochemical Science, V2, DOI [10.19080/rapsci.2017.02.555586, 10.19080/rapsci.2017.02.555586, DOI 10.19080/RAPSCI.2017.02.555586]"
"   Soni A, 2016, PROCEDIA ENVIRON SCI, V35, P119, DOI 10.1016/j.proenv.2016.07.057"
"   Speight J., 2018, Reaction Mechanisms in Environmental Engineering, V1st"
"   Srivastava V, 2017, FRONT ENV SCI-SWITZ, V5, DOI 10.3389/fenvs.2017.00064"
"   Subramaniyam V, 2015, J APPL PHYCOL, V27, P1861, DOI 10.1007/s10811-014-0492-2"
"   Tahir MB., 2020, ENCY RENEWABLE SUSTA, P320"
"   Tan TW, 2003, APPL BIOCHEM BIOTECH, V104, P119, DOI 10.1385/ABAB:104:2:119"
"   Tangahu BV, 2011, INT J CHEM ENG, V2011, DOI 10.1155/2011/939161"
"   Tao L, 2011, ENVIRON MONIT ASSESS, V175, P227, DOI 10.1007/s10661-010-1508-2"
"   Thornley P, 2015, BIOMASS BIOENERG, V81, P35, DOI 10.1016/j.biombioe.2015.05.002"
"   Tiseo, 2020, GLOBAL POPULATION MS"
"   Trindade PVO, 2005, CHEMOSPHERE, V58, P515, DOI 10.1016/j.chemosphere.2004.09.021"
"   Tripathi RD, 2007, TRENDS BIOTECHNOL, V25, P158, DOI 10.1016/j.tibtech.2007.02.003"
"   Valix M, 2017, CURRENT DEVELOPMENTS IN BIOTECHNOLOGY AND BIOENGINEERING: SOLID WASTE MANAGEMENT, P407, DOI 10.1016/B978-0-444-63664-5.00018-6"
"   Valls M, 2000, J INORG BIOCHEM, V79, P219, DOI 10.1016/S0162-0134(99)00170-1"
"   Vel??squez L, 2009, J HAZARD MATER, V167, P713, DOI 10.1016/j.jhazmat.2009.01.044"
"   Vijver MG, 2004, ENVIRON SCI TECHNOL, V38, P4705, DOI 10.1021/es040354g"
"   Vinti G, 2021, INT J ENV RES PUB HE, V18, DOI 10.3390/ijerph18084331"
"   Wagner-D??bler I, 2000, APPL ENVIRON MICROB, V66, P4559, DOI 10.1128/AEM.66.10.4559-4563.2000"
"   Wang XD, 2021, J HAZARD MATER, V412, DOI 10.1016/j.jhazmat.2021.125200"
"   Wang Y, 2020, ECOTOX ENVIRON SAFE, V192, DOI 10.1016/j.ecoenv.2020.110294"
"   Wiszniewska A, 2016, PEDOSPHERE, V26, P1, DOI 10.1016/S1002-0160(15)60017-0"

"   Wu C, 2021, J ENVIRON MANAGE, V296, DOI 10.1016/j.jenvman.2021.113185"
"   Wu MX, 2017, J HAZARD MATER, V337, P189, DOI 10.1016/j.jhazmat.2017.05.006"
"   Wuana R.A., 2011, ISRN Ecology, DOI [10.5402/2011/402647, DOI 10.5402/2011/402647]"
"   Xie Y, 2016, FRONT PLANT SCI, V7, DOI 10.3389/fpls.2016.00755"
"   Yan A, 2020, FRONT PLANT SCI, V11, DOI 10.3389/fpls.2020.00359"
"   Yao XC, 2021, SCI TOTAL ENVIRON, V761, DOI 10.1016/j.scitotenv.2020.143260"
"   Yin Kun Yin Kun, 2019, Chemical Engineering Journal, V360, P1553, DOI 10.1016/j.cej.2018.10.226"
"   Zaidi A., 2011, BIOMANAGEMENT METAL, P479, DOI DOI 10.1007/978-94-007-1914-9_21"
"   Zainith S., 2021, Bioremediation for Environmental Sustainability, P481, DOI DOI 10.1016/B978-0-12-820524-2.00020-1"
"   Zhalnina K, 2015, MICROB ECOL, V69, P395, DOI 10.1007/s00248-014-0530-2"
"   Ziagova M, 2007, BIORESOURCE TECHNOL, V98, P2859, DOI 10.1016/j.biortech.2006.09.043"
NR 199
TC 26
Z9 31
U1 16
U2 187
PU TAYLOR & FRANCIS LTD
PI ABINGDON
"PA 2-4 PARK SQUARE, MILTON PARK, ABINGDON OR14 4RN, OXON, the United Kingdom"
SN 0738-8551
EI 1549-7801
J9 CRIT REV BIOTECHNOL
JI Crit. Rev. Biotechnol.
PD APR 2
PY 2024
VL 44
IS 3
BP 429
EP 447
DI 10.1080/07388551.2023.2170862
EA FEB 2023
PG 19
WC Biotechnology & Applied Microbiology
WE Science Citation Index Expanded (SCI-EXPANDED)
SC Biotechnology & Applied Microbiology
GA MU2B7
UT WOS:000941861800001
PM 36851851
DA 2026-04-06
ER

PT J
"AU Cheng, JM"
"   Sun, ZH"
"   Yu, YQ"
"   Li, XR"
"   Li, TT"
"AF Cheng, Jiemin"
"   Sun, Zihan"
"   Yu, Yaqin"
"   Li, Xinrui"
"   Li, Tingting"
TI Effects of modified carbon black nanoparticles on plant-microbe remediation of petroleum and heavy metal co-contaminated soils
SO INTERNATIONAL JOURNAL OF PHYTOREMEDIATION
LA English
DT Article
DE Co-contaminated soil; heavy metals; microorganisms; petroleum; plants
ID CRUDE-OIL; BIOREMEDIATION; SPECIATION; BIOAVAILABILITY; BIODEGRADATION; IMPROVEMENT; STRAINS
"AB Little is known about the effect of modified carbon black nanoparticles (MNCB) on the availability of heavy metals and petroleum degradation in petroleum and heavy metals co-contaminated soils. The overall objective of this study was to investigate the simultaneous effect of MNCB on heavy metal immobilization and petroleum biodegradation in co-contaminated soil in plant and plant-microbe combined remediation. The results showed that the petroleum degradation increased by 50% in petroleum-Cd co-contaminated soil and 65% in petroleum-Ni co-contaminated soil in plant-microbe combined remediation, comparing with the plant remediation and the application of MNCB did not show significant improvement on petroleum degradation in both plant and plant-microbe combined remediation. MNCB could significantly reduce the availability of heavy metals in soil and the uptakes of Cd and Ni by Suaeda salsa by roughly 18 and 10% and improve the growth of plant by alleviating the growth inhibition caused by heavy metals. The application of Bacillus subtilis and Sphingobacterium multivorum (heavy metal tolerant bacteria) inhibited the biomass of Suaeda salsa by enhancing the petroleum degradation. It could be concluded that MNCB played a major role in immobilizing the heavy metals and bacteria dominated the petroleum degradation in petroleum-metal co-contaminated soil."
"C1 [Cheng, Jiemin; Sun, Zihan; Yu, Yaqin; Li, Xinrui; Li, Tingting] Shandong Normal Univ, Coll Geog & Environm, Jinan 250014, Shandong, Peoples R China."
C3 Shandong Normal University
"RP Cheng, JM (corresponding author), Shandong Normal Univ, Coll Geog & Environm, Jinan 250014, Shandong, Peoples R China."
EM jmcheng2002@hotmail.com
"RI sun, ZIHAN/KTI-7057-2024; tingting, Li/KYP-5635-2024; Li,"
   Xinrui/KIJ-2564-2024
"FU National Natural Science Fund Committee, China [41471255, 41877119];"
   Shandong Province Natural Science Foundation of China [ZR2016YL002]

FX The authors would like to thank the National Natural Science Fund
"   Committee, China [No. 41471255 and No. 41877119], and the Shandong"
   Province Natural Science Foundation of China [Grant No. ZR2016YL002].
"CR Abd El-Azeem S, 2013, ENVIRON EARTH SCI, V70, P3411, DOI 10.1007/s12665-013-2410-3"
"   Amado GM, 2008, BRAZ J BIOL, V68, P95, DOI 10.1590/S1519-69842008000100013"
"   Amor L, 2001, BIORESOURCE TECHNOL, V78, P181, DOI 10.1016/S0960-8524(00)00182-6"
"   An X, 2006, FISHERIES SCI, V25, P46"
"   [Anonymous], 2001, BCR701 IRMM EUR COMM"
"   ATLAS RM, 1981, MICROBIOL REV, V45, P180, DOI 10.1128/MMBR.45.1.180-209.1981"
"   Bartha R, 1977, Adv Appl Microbiol, V22, P225, DOI 10.1016/S0065-2164(08)70164-3"
"   BOLAN NS, 1991, PLANT SOIL, V134, P53, DOI 10.1007/BF00010717"
"   Borah D, 2008, APPL SURF SCI, V254, P3049, DOI 10.1016/j.apsusc.2007.10.053"
"   BRUEMMER GW, 1986, Z PFLANZ BODENKUNDE, V149, P382, DOI 10.1002/jpln.19861490404"
"   Chang Y, 2014, LECT NOTES ELECT ENG, V250, P1245, DOI [10.1007/978-3-642-37922-2_134., DOI 10.1007/978-3-642-37922-2_134]"
"   Cheng JM, 2014, INT J PHYTOREMEDIAT, V16, P86, DOI 10.1080/15226514.2012.759530"
"   Cheng JM, 2015, J CHEM-NY, V2015, DOI 10.1155/2015/839069"
"   Chuan MC, 1996, WATER AIR SOIL POLL, V90, P543, DOI 10.1007/BF00282668"
"   Cui ZJ., 2015, STUDY REMEDIATION TE"
"   ELLIS B, 1990, ENVIRON TECHNOL, V11, P443, DOI 10.1080/09593339009384884"
"   Erdogmus SF, 2013, WATER AIR SOIL POLL, V224, DOI 10.1007/s11270-013-1449-9"
"   Fu XW, 2014, ENVIRON SCI-PROC IMP, V16, P1737, DOI 10.1039/c3em00618b"
"   Gao YY, 2014, KEY ENG MATER, V609-610, P1538, DOI 10.4028/www.scientific.net/KEM.609-610.1538"
"   Garbisu C, 2001, BIORESOURCE TECHNOL, V77, P229, DOI 10.1016/S0960-8524(00)00108-5"
"   Gargouri B, 2014, J CHEM TECHNOL BIOT, V89, P978, DOI 10.1002/jctb.4188"
"   G??mez-Pastora J, 2014, CHEM ENG J, V256, P187, DOI 10.1016/j.cej.2014.06.119"
"   Head IM, 2006, NAT REV MICROBIOL, V4, P173, DOI 10.1038/nrmicro1348"
"   Iwegbue CMA, 2011, CHEM SPEC BIOAVAILAB, V23, P7, DOI 10.3184/095422911X12964002282100"
"   Kandeler E, 1996, BIOL FERT SOILS, V23, P299, DOI 10.1007/s003740050174"
"   Kang CH, 2016, ECOL ENG, V89, P64, DOI 10.1016/j.ecoleng.2016.01.023"
"   Khosravi E, 2013, ENG GEOL, V166, P11, DOI 10.1016/j.enggeo.2013.08.004"
"   Nadal M, 2007, CHEMOSPHERE, V66, P267, DOI 10.1016/j.chemosphere.2006.05.020"
"   Olaniran AO, 2013, INT J MOL SCI, V14, P10197, DOI 10.3390/ijms140510197"
"   Oyetibo GO, 2017, INT BIODETER BIODEGR, V120, P143, DOI 10.1016/j.ibiod.2017.02.021"
"   REUTER JH, 1977, GEOCHIM COSMOCHIM AC, V41, P325, DOI 10.1016/0016-7037(77)90240-X"
"   Roy AS, 2014, INT BIODETER BIODEGR, V94, P79, DOI 10.1016/j.ibiod.2014.03.024"
"   SORKHOH NA, 1990, ENVIRON POLLUT, V65, P1, DOI 10.1016/0269-7491(90)90162-6"
"   Stafiej A, 2007, SEP PURIF TECHNOL, V58, P49, DOI 10.1016/j.seppur.2007.07.008"
"   Sungur A, 2015, SOIL SEDIMENT CONTAM, V24, P1, DOI 10.1080/15320383.2014.907238"
"   THOMSON CJ, 1993, J PLANT NUTR, V16, P493, DOI 10.1080/01904169309364548"
"   Tungittiplakorn W, 2004, ENVIRON SCI TECHNOL, V38, P1605, DOI 10.1021/es0348997"
"   URE AM, 1993, INT J ENVIRON AN CH, V51, P135, DOI 10.1080/03067319308027619"
"   Varanasi P, 2007, CHEMOSPHERE, V66, P1031, DOI 10.1016/j.chemosphere.2006.07.036"
"   [?????? Yuan Min], 2005, [????€???????, Ecology and Environment], V14, P43"
"   Zanuzzi A, 2013, ENVIRON EARTH SCI, V70, P2623, DOI 10.1007/s12665-013-2313-3"
"   Zhou DM, 2010, J HAZARD MATER, V174, P34, DOI 10.1016/j.jhazmat.2009.09.012"
NR 42
TC 30
Z9 42
U1 1
U2 210
PU TAYLOR & FRANCIS INC
PI PHILADELPHIA
"PA 530 WALNUT STREET, STE 850, PHILADELPHIA, PA 19106 USA"
SN 1522-6514
EI 1549-7879
J9 INT J PHYTOREMEDIAT
JI Int. J. Phytoremediat.
PD JUN 7
PY 2019
VL 21
IS 7
BP 634
EP 642
DI 10.1080/15226514.2018.1556581
PG 9
WC Environmental Sciences
WE Science Citation Index Expanded (SCI-EXPANDED)
SC Environmental Sciences & Ecology
GA HY0UO
UT WOS:000467828900003
PM 30656965
DA 2026-04-06
ER

PT C
"AU Feng, H"
"AF Feng, Hao"
GP IOP
TI Discussion on Microbial Remediation Technology of Heavy Metal
   Contaminated Soil
"SO 5TH INTERNATIONAL CONFERENCE ON ADVANCES IN ENERGY, ENVIRONMENT AND"
   CHEMICAL ENGINEERING
SE IOP Conference Series-Earth and Environmental Science

LA English
DT Proceedings Paper
"CT 5th International Conference on Advances in Energy, Environment and"
Chemical Engineering (AEECE)
"CY AUG 16-18, 2019"
"CL Beijing Univ Agr, Coll Bioscience & Resource Environm, Shanghai, PEOPLES"
R CHINA
"HO Beijing Univ Agr, Coll Bioscience & Resource Environm"
"AB The key issue in the world is to treat heavy metal contaminated soil. This is because environmental pollution, especially the intrusion of heavy metals in the soil, has polluted the ecological environment chain and threatened human survival. It has happened in all countries of the world. At present, through research on the source characteristics of heavy metals and the destruction behavior in soil, all countries have new research topics. This paper analyzes the soil heavy metal pollution and microbial remediation technology, and proposes how to use microbial technology to take action against heavy metal pollution in soil. The corresponding intentions of the measures with strong sexuality and effectiveness, as well as suggestions which are expected to have reference value for solving soil pollution problems."
"C1 [Feng, Hao] ChiFeng Ind Vocat Technol Coll, Chifeng 024000, Peoples R China."
"RP Feng, H (corresponding author), ChiFeng Ind Vocat Technol Coll, Chifeng 024000, Peoples R China."
"CR Chen Jidong, 2019, LOW CARBON WORLD, V9, P43"
"   Liu JL., 2019, SHANDONG IND TECHNOL, V07, P229"
"   Mou YH, 2019, J HEMATOL ONCOL, V12, DOI 10.1186/s13045-019-0720-y"
"   Tian Maoyuan, 2019, MINERAL ROCK, V39, P115"
"   Wang Yimin, 2018, ENV PROTECTION FRONT, V8, P492"
"   Zhang BA, 2019, ACS APPL MATER INTER, V11, P10328, DOI 10.1021/acsami.9b00359"
NR 6
TC 2
Z9 2
U1 10
U2 199
PU IOP PUBLISHING LTD
PI BRISTOL
"PA DIRAC HOUSE, TEMPLE BACK, BRISTOL BS1 6BE, the United Kingdom"
SN 1755-1307
J9 IOP C SER EARTH ENV
JI IOP Conf. Ser. Earth Envir. Sci.
PY 2019
VL 358
AR 022011
DI 10.1088/1755-1315/358/2/022011
PG 5
"WC Energy & Fuels; Engineering, Chemical; Environmental Sciences"
WE Conference Proceedings Citation Index - Science (CPCI-S)
SC Energy & Fuels; Engineering; Environmental Sciences & Ecology
GA BP7JJ
UT WOS:000562374700011
OA gold
DA 2026-04-06
ER

PT J
"AU Selim, S"
"   Harun-Ur-Rashid, M"
"AF Selim, Samy"
"   Harun-Ur-Rashid, Mohammad"
TI Harnessing microbial diversity for effective remediation of heavy
metal-contaminated soils
SO APPLIED SOIL ECOLOGY
LA English
DT Review
DE Soil remediation; Microbial diversity; Bioaugmentation; Heavy metal
remediation
ID BIOREMEDIATION; REMOVAL
"AB Heavy metal contamination in soils presents a significant global environmental challenge, particularly in agricultural regions where it threatens food safety, soil health, and human well-being. This review explores the role of microbial communities in the remediation of heavy metals from contaminated soils, with an emphasis on the diversity and functionality of bacteria and fungi in these processes. Microorganisms play a pivotal role in biogeochemical cycling, employing mechanisms such as bioaccumulation, detoxification, and transformation to mitigate the toxic effects of heavy metals. This review discusses various biotechnological approaches, such as bioaugmentation and biostimulation, which utilize microbes to enhance natural cleanup processes in polluted areas. Case studies from around the world highlight the practical applications of microbial remediation techniques, demonstrating their effectiveness in reducing heavy metal concentrations in soils. The review also addresses the challenges in this field, including the need for tailored remediation strategies and the competition between indigenous and introduced microbial populations. Future research and technological advancements are crucial for optimizing microbial remediation processes, ensuring the sustainability of agricultural practices, and protecting environmental and human health."
"C1 [Selim, Samy] Jouf Univ, Coll Appl Med Sci, Dept Clin Lab Sci, Sakaka 72388, Saudi Arabia."
"   [Harun-Ur-Rashid, Mohammad] Int Univ Business Agr & Technol, Dept Chem, Dhaka 1230, Bangladesh."
C3 Al Jouf University; International University of Business Agriculture &
Technology (IUBAT)
"RP Selim, S (corresponding author), Jouf Univ, Coll Appl Med Sci, Dept Clin Lab Sci, Sakaka 72388, Saudi Arabia."
EM sabdulsalam@ju.edu.sa
"RI Selim, Samy/AAZ-3438-2020"
"CR Abou-Aly HE, 2021, BIOCATAL AGR BIOTECH, V31, DOI 10.1016/j.bcab.2021.101911"

"   Al-Ansari MM, 2021, ENVIRON RES, V199, DOI 10.1016/j.envres.2021.111240"
"   Alkorta I., 2010, Reviews on Environmental Health, V25, P135"
"   Amirnia S, 2015, CHEM ENG J, V264, P863, DOI 10.1016/j.cej.2014.12.016"
"   Arslan B, 2017, REV ENVIRON CONTAM T, V243, P27, DOI 10.1007/398_2016_18"
"   Aryal M, 2021, REV CHEM ENG, V37, P715, DOI 10.1515/revce-2019-0016"
"   Behera BC, 2020, CRIT REV MICROBIOL, V46, P727, DOI 10.1080/1040841X.2020.1828815"
"   Bramhachari P.V., 2017, Marine pollution and microbial remediation, P67, DOI [DOI 10.1007/978-981-10-1044-6_5, 10.1007/978-981-10-1044-6_5]"
"   Pereira IAC, 2011, FRONT MICROBIOL, V2, DOI 10.3389/fmicb.2011.00069"
"   Chauhan P., 2023, GREEN TECHNOLOGIES I, P147, DOI [10.1007/978-3-031-46858-2_8, DOI 10.1007/978-3-031-46858-2_8]"
"   Chen T, 2023, ENVIRON POLLUT, V338, DOI 10.1016/j.envpol.2023.122667"
"   Collin MS, 2022, J HAZARD MATER ADV, V7, DOI 10.1016/j.hazadv.2022.100094"
"   Cursino L, 2000, SCI TOTAL ENVIRON, V261, P109, DOI 10.1016/S0048-9697(00)00629-X"
"   El-Bondkly AMA, 2022, HELIYON, V8, DOI 10.1016/j.heliyon.2022.e09854"
"   El-Mahdy OM, 2021, ENVIRON SCI POLLUT R, V28, P67608, DOI 10.1007/s11356-021-15382-4"
"   Feng G, 2018, BMC MICROBIOL, V18, DOI 10.1186/s12866-018-1152-5"
"   Geoghegan I, 2017, TRENDS MICROBIOL, V25, P957, DOI 10.1016/j.tim.2017.05.015"
"   Ghosh S, 2023, FRONT BIOENG BIOTECH, V11, DOI 10.3389/fbioe.2023.1106973"
"   Henao SG, 2021, FRONT ENV SCI-SWITZ, V9, DOI 10.3389/fenvs.2021.604216"
"   Gupta S, 2015, INT J ENVIRON SCI TE, V12, P995, DOI 10.1007/s13762-013-0484-9"
"   Han Y, 2021, SCI TOTAL ENVIRON, V798, DOI 10.1016/j.scitotenv.2021.149222"
"   Hern??ndez IA, 2024, APPL SCI-BASEL, V14, DOI 10.3390/app142411756"
"   Hussain Fida, 2024, Chemosphere, V352, P141403, DOI [10.1016/j.chemosphere.2024.141403, 10.1016/j.chemosphere.2024.141403]"
"   Iravani S, 2020, ACS SUSTAIN CHEM ENG, V8, P5395, DOI 10.1021/acssuschemeng.0c00292"
"   Jiao WX, 2022, MOL PLANT PATHOL, V23, P304, DOI 10.1111/mpp.13159"
"   Kumar R S., 2020, MICROORGANISMS SUSTA, P137, DOI [10.1016/B978-0-12-819001-2.00007-3, DOI 10.1016/B978-0-12-819001-2.00007-3]"
"   Lebrazi S., 2018, Legumes for Soil Health and Sustainable Management, P205, DOI DOI 10.1007/978-981-13-0253-4_7"
"   Li M, 2021, SCI TOTAL ENVIRON, V760, DOI 10.1016/j.scitotenv.2020.144037"
"   Li Y, 2014, DESALIN WATER TREAT, V52, P7218, DOI 10.1080/19443994.2013.823567"
"   Liu MQ, 2012, PLOS ONE, V7, DOI 10.1371/journal.pone.0045306"
"   Liu YG, 2006, T NONFERR METAL SOC, V16, P681, DOI 10.1016/S1003-6326(06)60121-0"
"   Lounes-Hadj Sahraoui A., 2022, Assisted Phytoremediation, P49, DOI [DOI 10.1016/B978-0-12-822893-7.00008-2, 10.1016/B978-0-12-822893, DOI 10.1016/B978-0-12-822893]"
"   Lu HF, 2021, MINERALS-BASEL, V11, DOI 10.3390/min11030243"
"   L?? HX, 2024, J CLEAN PROD, V451, DOI 10.1016/j.jclepro.2024.141913"
"   Luka Y, 2024, Biological Sciences, V04, DOI [10.55006/biolsciences.2024.4105, 10.55006/biolsciences.2024.4105, DOI 10.55006/BIOLSCIENCES.2024.4105]"
"   Lv Y, 2022, POL J ENVIRON STUD, V31, P1747, DOI 10.15244/pjoes/141326"
"   Maqsood Q, 2022, ARCH MICROBIOL, V204, DOI 10.1007/s00203-022-03087-2"
"   Massoud R, 2019, ELECTRON J BIOTECHN, V37, P56, DOI 10.1016/j.ejbt.2018.11.003"
"   Tarekegn MM, 2020, COGENT FOOD AGR, V6, DOI 10.1080/23311932.2020.1783174"
"   Mergeay M., 2015, Metal response in Cupriavidus metallidurans: volume I: from habitats to genes and proteins, P1, DOI 10.1007/978-3-319-20594-6_1"
"   Mordhorst A, 2022, SCI TOTAL ENVIRON, V817, DOI 10.1016/j.scitotenv.2022.152952"
"   Mortada WI, 2023, CHEMOSPHERE, V331, DOI 10.1016/j.chemosphere.2023.138804"
"   Mukhopadhyay M, 2011, CAN J CHEM ENG, V89, P889, DOI 10.1002/cjce.20460"
"   Nnaji ND, 2023, SN APPL SCI, V5, DOI 10.1007/s42452-023-05351-6"
"   Nunan N, 2017, SCI REP-UK, V7, DOI 10.1038/s41598-017-04485-z"
"   Okpara-Elom IA, 2024, BIOREMEDIAT J, V28, P34, DOI 10.1080/10889868.2022.2143473"
"   Oves M, 2019, INT J ENVIRON SCI TE, V16, P6967, DOI 10.1007/s13762-019-02300-y"
"   Pal A, 2022, CRIT REV MICROBIOL, V48, P327, DOI 10.1080/1040841X.2021.1970512"
"   Pande V, 2022, FRONT MICROBIOL, V13, DOI 10.3389/fmicb.2022.824084"
"   Paulo LM, 2017, FRONT MICROBIOL, V8, DOI 10.3389/fmicb.2017.01341"
"   Pelaez AI, 2013, ENVIRON POLLUT, V181, P190, DOI 10.1016/j.envpol.2013.06.004"
"   Peng C, 2023, PROCESS SAF ENVIRON, V170, P720, DOI 10.1016/j.psep.2022.12.037"
"   Pujari M., 2021, Heavy Metals in the Environment, P1, DOI [10.1016/B978-0-12-821656-9.00001-8, DOI 10.1016/B978-0-12-821656-9.00001-8]"
"   Qiu JJ, 2021, APPL MICROBIOL BIOT, V105, P6477, DOI 10.1007/s00253-021-11497-3"
"   Rabani M.S., 2022, Phytoremediation, P91"
"   Rajendran S, 2022, CHEMOSPHERE, V287, DOI 10.1016/j.chemosphere.2021.132369"
"   Rashid A, 2023, AGRONOMY-BASEL, V13, DOI 10.3390/agronomy13061521"
"   Roy A, 2018, BIORESOURCE TECHNOL, V253, P22, DOI 10.1016/j.biortech.2018.01.004"
"   Roy R, 2024, APPL BIOCHEM BIOTECH, V196, P1712, DOI 10.1007/s12010-023-04614-7"
"   Salamanca D, 2013, CHEM-ING-TECH, V85, P1575, DOI 10.1002/cite.201200144"
"   Saravanan A, 2022, CHEMOSPHERE, V298, DOI 10.1016/j.chemosphere.2022.134341"
"   Scullett-Dean G, 2023, SCI TOTAL ENVIRON, V877, DOI 10.1016/j.scitotenv.2023.162699"
"   Shah V, 2020, ENVIRON TECHNOL INNO, V18, DOI 10.1016/j.eti.2020.100774"
"   Sharma A., 2023, METALS WATER, P39, DOI [10.1016/B978-0-323-95919-3.00015-X, DOI 10.1016/B978-0-323-95919-3.00015-X]"
"   Sharma R, 2022, ENVIRON RES, V212, DOI 10.1016/j.envres.2022.113337"
"   Silva-Castro GA, 2023, J FUNGI, V9, DOI 10.3390/jof9010056"
"   Singh VK, 2024, MYCOLOGY-UK, V15, P585, DOI 10.1080/21501203.2024.2389290"
"   Suhani I, 2021, CURR OPIN TOXICOL, V27, P1, DOI 10.1016/j.cotox.2021.04.004"
"   Sun LJ, 2021, SCI REP-UK, V11, DOI 10.1038/s41598-021-91003-x"
"   Sun QY, 2022, TOXICOLOGY, V469, DOI 10.1016/j.tox.2022.153136"
"   Tavares APM, 2017, Current Biotechnology, V6, P78, DOI [10.2174/2211550105666160510113212, 10.2174/2211550105666160510113212, DOI 10.2174/2211550105666160510113212]"
"   Tripathi S, 2021, BIORESOURCE TECHNOL, V338, DOI 10.1016/j.biortech.2021.125518"
"   Vaksmaa A, 2023, FRONT MAR SCI, V10, DOI 10.3389/fmars.2023.1070905"
"   V??lkel S, 2018, FRONT MICROBIOL, V9, DOI 10.3389/fmicb.2018.03157"

"   Wang GZ, 2020, COLLOID SURFACE B, V195, DOI 10.1016/j.colsurfb.2020.111249"
"   Wani Parvaze Ahmad, 2019, Biotechnology Research & Innovation, V3, P38, DOI [10.1016/j.biori.2019.04.001, 10.1016/j.biori.2019.04.001]"
"   Wu Q, 2022, J HAZARD MATER ADV, V8, DOI 10.1016/j.hazadv.2022.100161"
"   Xia F, 2024, J HAZARD MATER, V465, DOI 10.1016/j.jhazmat.2023.133215"
"   Xu XY, 2024, BIOCHEM ENG J, V210, DOI 10.1016/j.bej.2024.109433"
"   Zhou B, 2023, APPL SCI-BASEL, V13, DOI 10.3390/app13148439"
NR 80
TC 4
Z9 4
U1 41
U2 42
PU ELSEVIER
PI AMSTERDAM
"PA RADARWEG 29, 1043 NX AMSTERDAM, NETHERLANDS"
SN 0929-1393
EI 1873-0272
J9 APPL SOIL ECOL
JI Appl. Soil Ecol.
PD NOV
PY 2025
VL 215
AR 106473
DI 10.1016/j.apsoil.2025.106473
EA SEP 2025
PG 12
WC Soil Science
WE Science Citation Index Expanded (SCI-EXPANDED)
SC Agriculture
GA 7PY3B
UT WOS:001576877900001
DA 2026-04-06
ER

PT J
"AU Li, HX"
"   Cui, XL"
"   Sun, YC"
"   Zheng, P"
"   Wang, L"
"   Shi, XY"
"AF Li, Hongxia"
"   Cui, Xinglan"
"   Sun, Yingchun"
"   Zheng, Peng"
"   Wang, Lei"
"   Shi, Xinyue"
TI Advances in Microbial Remediation of Heavy Metal-Contaminated Soils:
"   Mechanisms, Synergistic Technologies, Field Applications and Future"
   Perspectives
SO TOXICS
LA English
DT Review
DE heavy metal contamination; microbial remediation; mechanisms;
   synergistic technology; field application
"AB Heavy-metal contamination poses a significant global threat to soil environments, underscoring the necessity for effective and sustainable remediation technologies. This review methodically summarizes advances in the field of microbial remediation of heavy metal-contaminated soils, organized around four major dimensions: remediation mechanisms, synergistic technologies, field applications, and future prospects. Firstly, the remediation mechanisms are elucidated, encompassing molecular interactions, cellular adaptation, and community-level cooperative responses. Secondly, the integration of microbes with functional materials and bioelectrochemical systems (BESs) is evaluated, with these materials providing support, electron mediation, and micro-environment regulation that markedly improve remediation efficiency and stability. Moreover, illustrative field cases demonstrate pivotal technological pathways and cost-effectiveness when transitioning from laboratory- to field-scale applications. Finally, emerging frontiers such as synthetic biology-engineered microbes, AI-driven microbial design, circular-economy value recovery, and policy-governance innovations are discussed, proposing essential elements for building a ""predictable-controllable-sustainable"" microbial remediation platform. This review aims to provide a comprehensive knowledge framework for researchers and to offer decision-making guidance for practitioners and policymakers, thereby advancing microbial remediation toward higher efficiency, reliability, and scalability."
"C1 [Li, Hongxia; Cui, Xinglan; Sun, Yingchun; Wang, Lei; Shi, Xinyue] GRINM Resources & Environm Technol Corp Ltd, Natl Engn Res Ctr Environm Friendly Met Producing, Beijing 101407, Peoples R China."
"   [Zheng, Peng] CECEP DADI Hangzhou Environm Remediat Corp Ltd, Hangzhou 310013, Peoples R China."
"RP Cui, XL (corresponding author), GRINM Resources & Environm Technol Corp Ltd, Natl Engn Res Ctr Environm Friendly Met Producing, Beijing 101407, Peoples R China."
EM lihongxia@grinm.com; cuixinglan@grinm.com; 15966001661@163.com;
   hjgczhengp@163.com; wanglei@grinm.com; shixinyue@grinm.com
"RI shi, xinyue/HGA-9757-2022"
FU Scientific Research and Technology Development Program of Guangxi [Guike
"   AB22080078, Guike AB23026043]; National Natural Science Foundation of"
   China [52274272]
FX The project was funded by the National Natural Science Foundation of
   China (Grants No. 52274272) and the Scientific Research and Technology
   Development Program of Guangxi (No. Guike AB22080078 and No. Guike

AB23026043).
"CR Abbas HMM, 2024, SCI TOTAL ENVIRON, V954, DOI 10.1016/j.scitotenv.2024.176104"
"   Aggarwal N, 2023, CHEM REV, V123, P31, DOI 10.1021/acs.chemrev.2c00431"
"   Agrawal K, 2024, CRIT REV BIOTECHNOL, V44, P429, DOI 10.1080/07388551.2023.2170862"
"   Alavian F, 2025, ENVIRON MONIT ASSESS, V197, DOI 10.1007/s10661-025-14666-3"
"   Alsafran M, 2023, J PLANT GROWTH REGUL, V42, P3419, DOI 10.1007/s00344-022-10803-1"
"   Aminian-Dehkordi J, 2023, BIOTECHNOL ADV, V68, DOI 10.1016/j.biotechadv.2023.108239"
"   Chang TT, 2023, NAT COMMUN, V14, DOI 10.1038/s41467-023-42358-4"
"   Che SH, 2024, CHEM ENG J, V497, DOI 10.1016/j.cej.2024.154684"
"   Feng ZV, 2015, CHEM SCI, V6, P5186, DOI 10.1039/c5sc00792e"
"   Garg A, 2025, BIODEGRADATION, V36, DOI 10.1007/s10532-025-10165-x"
"   Gautam K, 2023, ENVIRON RES, V225, DOI 10.1016/j.envres.2023.115592"
"   Ge Y, 2022, J SOIL SEDIMENT, V22, P218, DOI 10.1007/s11368-021-03066-y"
"   Guo DC, 2019, SCI REP-UK, V9, DOI 10.1038/s41598-018-38360-2"
"   Gustave W, 2021, SCI TOTAL ENVIRON, V756, DOI 10.1016/j.scitotenv.2020.143865"
"   Habibul N, 2016, ENVIRON SCI TECHNOL, V50, P3882, DOI 10.1021/acs.est.5b06376"
"   Hassan A, 2019, WATER AIR SOIL POLL, V230, DOI 10.1007/s11270-019-4227-5"
"   He YX, 2019, CHEMOSPHERE, V221, P793, DOI 10.1016/j.chemosphere.2019.01.039"
"   Hong Y, 2022, SCI TOTAL ENVIRON, V851, DOI 10.1016/j.scitotenv.2022.158033"
"   Jaiswal S, 2020, FRONT MICROBIOL, V11, DOI 10.3389/fmicb.2020.00808"
"   Jansson JK, 2023, NAT BIOTECHNOL, V41, P1716, DOI 10.1038/s41587-023-01932-3"
"   Jiang GB, 2025, J HAZARD MATER, V494, DOI 10.1016/j.jhazmat.2025.138753"
"   Jiang Y, 2025, J HAZARD MATER, V498, DOI 10.1016/j.jhazmat.2025.139970"
"   Johns NI, 2016, CURR OPIN MICROBIOL, V31, P146, DOI 10.1016/j.mib.2016.03.010"
"   Khan M, 2025, J ENVIRON MANAGE, V374, DOI 10.1016/j.jenvman.2024.123969"
"   Khatoon Z, 2024, MICROORGANISMS, V12, DOI 10.3390/microorganisms12101945"
"   Kou B, 2024, SCI TOTAL ENVIRON, V917, DOI 10.1016/j.scitotenv.2024.170451"
"   Li XH, 2023, ENVIRON SCI POLLUT R, V30, P118192, DOI 10.1007/s11356-023-30655-w"
"   Li XF, 2021, ECOTOX ENVIRON SAFE, V222, DOI 10.1016/j.ecoenv.2021.112512"
"   Lian ZL, 2025, WATER AIR SOIL POLL, V236, DOI 10.1007/s11270-025-08055-2"
"   Liu C, 2022, SCI TOTAL ENVIRON, V809, DOI 10.1016/j.scitotenv.2021.152075"
"   Liu NQ, 2024, SCI TOTAL ENVIRON, V948, DOI 10.1016/j.scitotenv.2024.174237"
"   Liu SH, 2025, INT BIODETER BIODEGR, V196, DOI 10.1016/j.ibiod.2024.105940"
"   Liu XY, 2022, CHEMOSPHERE, V305, DOI 10.1016/j.chemosphere.2022.135393"
"   Ma F, 2026, INT BIODETER BIODEGR, V206, DOI 10.1016/j.ibiod.2025.106192"
"   Ma WY, 2022, ENVIRON POLLUT, V292, DOI 10.1016/j.envpol.2021.118327"
"   Mandal RR, 2025, J ENVIRON MANAGE, V375, DOI 10.1016/j.jenvman.2025.124199"
"   Mishra S, 2024, REV ENVIRON CONTAM T, V262, DOI 10.1007/s44169-024-00072-2"
"   Nie XY, 2024, TOXICS, V12, DOI 10.3390/toxics12120872"
"   Rehman H, 2024, J ENVIRON MANAGE, V370, DOI 10.1016/j.jenvman.2024.122575"
"   Ren XK, 2025, NAT COMMUN, V16, DOI 10.1038/s41467-025-60117-5"
"   Saghafi D, 2019, 3 BIOTECH, V9, DOI 10.1007/s13205-019-1799-0"
"   Segundo RF, 2024, MOLECULES, V29, DOI 10.3390/molecules29122725"
"   Selim S, 2025, APPL SOIL ECOL, V215, DOI 10.1016/j.apsoil.2025.106473"
"   Shuaib M, 2021, MICROB PATHOGENESIS, V150, DOI 10.1016/j.micpath.2020.104713"
"   Simonin M, 2015, ENVIRON SCI POLLUT R, V22, P13710, DOI 10.1007/s11356-015-4171-x"
"   Song PP, 2022, SCI TOTAL ENVIRON, V838, DOI 10.1016/j.scitotenv.2022.156417"
"   Su HF, 2022, J CLEAN PROD, V373, DOI 10.1016/j.jclepro.2022.133825"
"   Su M, 2020, J ENVIRON MANAGE, V257, DOI 10.1016/j.jenvman.2019.109951"
"   Thai TD, 2023, FRONT BIOENG BIOTECH, V11, DOI 10.3389/fbioe.2023.1178680"
"   The Ministry of Environmental Protection, 2014, The Ministry of Land and Resources Report on the National Soil Contamination Survey"
"   UNEP, 2021, Global Assessment of Soil Pollution: Report"
"   van Bodegom P, 2007, MICROB ECOL, V53, P513, DOI 10.1007/s00248-006-9049-5"
"   Velte JM, 2025, MSYSTEMS, V10, DOI 10.1128/msystems.00921-25"
"   Vitova M, 2024, WORLD J MICROB BIOT, V40, DOI 10.1007/s11274-024-03974-4"
"   Wang HM, 2021, CHEM ENG J, V419, DOI 10.1016/j.cej.2021.129600"
"   Wang MJ, 2025, AGRONOMY-BASEL, V15, DOI 10.3390/agronomy15081876"
"   Wang N, 2024, SCI TOTAL ENVIRON, V912, DOI 10.1016/j.scitotenv.2023.168994"
"   Wang Z, 2022, J CLEAN PROD, V379, DOI 10.1016/j.jclepro.2022.134043"
"   Warner CM, 2020, SYNTHETIC BIOLOGY 2020: FRONTIERS IN RISK ANALYSIS AND GOVERNANCE, P19, DOI 10.1007/978-3-030-27264-7_2"
"   Wu C, 2021, J ENVIRON MANAGE, V296, DOI 10.1016/j.jenvman.2021.113185"
"   Xi H, 2025, J WATER PROCESS ENG, V71, DOI 10.1016/j.jwpe.2025.107416"
"   Xie XG, 2025, J ENVIRON MANAGE, V375, DOI 10.1016/j.jenvman.2025.124290"
"   Xiong BY, 2024, BIORESOURCE TECHNOL, V397, DOI 10.1016/j.biortech.2024.130501"
"   Yu FM, 2024, J HAZARD MATER, V480, DOI 10.1016/j.jhazmat.2024.135883"
"   Yu JA, 2025, J ENVIRON MANAGE, V383, DOI 10.1016/j.jenvman.2025.125402"
"   Zhang D, 2025, J HAZARD MATER, V496, DOI 10.1016/j.jhazmat.2025.139540"
"   Zhang H, 2025, ENVIRON POLLUT, V374, DOI [10.1016/j.envpol.2025.126211, 10.1016/j.envpol.2025.126211]"
"   Zhang JR, 2020, BIOELECTROCHEMISTRY, V136, DOI 10.1016/j.bioelechem.2020.107596"
"   Zhang YN, 2024, SCI TOTAL ENVIRON, V951, DOI 10.1016/j.scitotenv.2024.175369"
"   Zhang YY, 2025, CRIT REV ENV SCI TEC, V55, P1657, DOI 10.1080/10643389.2025.2560435"
"   Zheng K, 2023, FRONT MICROBIOL, V14, DOI 10.3389/fmicb.2023.1272591"
NR 71
TC 6
Z9 6
U1 18
U2 18
PU MDPI
PI BASEL
"PA MDPI AG, Grosspeteranlage 5, CH-4052 BASEL, SWITZERLAND"
EI 2305-6304
J9 TOXICS

JI Toxics
PD DEC 12
PY 2025
VL 13
IS 12
AR 1069
DI 10.3390/toxics13121069
PG 22
WC Environmental Sciences; Toxicology
WE Science Citation Index Expanded (SCI-EXPANDED)
SC Environmental Sciences & Ecology; Toxicology
GA AE6XB
UT WOS:001646347900001
PM 41441290
"OA Green Submitted, gold"
DA 2026-04-06
ER

PT J
"AU Chen, YS"
"   Wang, ZW"
"   Fang, Y"
"   Wang, GW"
"   Zhou, F"
"   Yu, JX"
"   Chi, R"
"   Xiao, CQ"
"AF Chen, Yushan"
"   Wang, Ziwei"
"   Fang, Yun"
"   Wang, Guowei"
"   Zhou, Fang"
"   Yu, Junxia"
"   Chi, Ruan"
"   Xiao, Chunqiao"
TI Occurrence and microbial remediation of polycyclic aromatic hydrocarbons
   and heavy metals pollution in soils
SO WORLD JOURNAL OF MICROBIOLOGY & BIOTECHNOLOGY
LA English
DT Review
DE Compost amendment; Dually polluted soil; Genetically engineered
   microorganisms; Heavy metals; Microbial remediation; Polycyclic aromatic
   hydrocarbons
ID CO-CONTAMINATED SOIL; OXIDATIVE STRESS; BIOREMEDIATION; BACTERIA; PAHS;
   BIOAVAILABILITY; PHENANTHRENE; MECHANISMS; DESORPTION; COMMUNITY
"AB The presence of soils contaminated with polycyclic aromatic hydrocarbons and heavy metals presents a widespread environmental challenge in industrial areas. Unlike single-pollutant systems, co-pollution by polycyclic aromatic hydrocarbons and heavy metals exhibits synergistic toxicity that weakens traditional remediation technologies. This review systematically examines microbial remediation strategies for such co-polluted soils, focusing on three levels of intervention: functional microorganisms, compost-based enhancement, and engineered microbial solutions. We highlight the role of noncovalent interactions, especially cation-pi bonding and hydrophobic partitioning, in decreasing pollutant bioavailability and increasing co-toxicity. Functional microbes improve pollutant removal through intracellular transformation, extracellular secretion of biosurfactants and chelators, and surface adsorption. Compost-enhanced systems incorporating biochar, vermicompost, and mycorrhizal fungi demonstrate improved pollutant breakdown and soil health. Engineered microbes offer advanced remediation capabilities but face challenges related to metabolic load, ecological safety, and regulation. Despite promising laboratory results, field implementation remains limited by low pollutant bioavailability, biosafety concerns, and uncertain cost efficiency. We propose critical future research directions to bridge this gap and enable practical remediation of soils co-contaminated with polycyclic aromatic hydrocarbons and heavy metals."
"C1 [Chen, Yushan; Wang, Ziwei; Fang, Yun; Wang, Guowei; Zhou, Fang; Yu, Junxia; Chi, Ruan; Xiao, Chunqiao] Wuhan Inst Technol, Sch Environm Ecol & Biol Engn, Hubei Key Lab Microbial Transformat & Regulat Biog, Key Lab Green Chem Proc,Minist Educ, 206 Guanggu 1st Rd,Donghu New & High Technol Dev Z, Wuhan 430205, Hubei, Peoples R China."
C3 Wuhan Institute of Technology
"RP Xiao, CQ (corresponding author), Wuhan Inst Technol, Sch Environm Ecol & Biol Engn, Hubei Key Lab Microbial Transformat & Regulat Biog, Key Lab Green Chem Proc,Minist Educ, 206 Guanggu 1st Rd,Donghu New & High Technol Dev Z, Wuhan 430205, Hubei, Peoples R China."
EM chunqiao@wit.edu.cn
"RI Wang, Guowei/PDY-0041-2025; Fang, Yun/OZE-8933-2025; Wang,"
   Ziwei/AEH-5685-2022
"OI Wang, Guowei/0000-0003-0762-9023; "
FU National Natural Science Foundation of China [52174256]; Special Project
   of Central Government for Local Science and Technology Development of
   Hubei Province [2023EGA028]
FX This work was supported by the National Natural Science Foundation of
"   China (52174256), and the Special Project of Central Government for"
   Local Science and Technology Development of Hubei Province (2023EGA028).
"CR Abdellah YAY, 2022, ENVIRON POLLUT, V307, DOI 10.1016/j.envpol.2022.119549"
"   Adnan M, 2024, ECOTOX ENVIRON SAFE, V274, DOI 10.1016/j.ecoenv.2024.116189"
"   Ahammed GJ, 2013, J EXP BOT, V64, P199, DOI 10.1093/jxb/ers323"
"   Ahankoub Maryam, 2020, Recent Pat Biotechnol, V14, P121, DOI [10.2174/1872208314666200128103513, 10.2174/1872208314666200128103513]"
"   Ali M, 2022, ENVIRON POLLUT, V295, DOI 10.1016/j.envpol.2021.118686"

"   Antonangelo JA, 2021, J ENVIRON MANAGE, V277, DOI 10.1016/j.jenvman.2020.111443"
"   APHIS USDA., 2024, Fed Reg, V89, P54762"
"   Aryal Mahendra, 2021, Environmental Technology Reviews, V10, P342, DOI [10.1080/21622515.2021.1989059, 10.1080/21622515.2021.1989059]"
"   Aryal Mahendra, 2024, Heliyon, V10, pe38683, DOI [10.1016/j.heliyon.2024.e38683, 10.1016/j.heliyon.2024.e38683]"
"   Aryal M, 2024, J ENVIRON CHEM ENG, V12, DOI 10.1016/j.jece.2024.112221"
"   Aryal M, 2021, J ENVIRON ENG, V147, DOI 10.1061/(ASCE)EE.1943-7870.0001853"
"   Aryal M, 2021, REV CHEM ENG, V37, P715, DOI 10.1515/revce-2019-0016"
"   Aryal M, 2013, WATER AIR SOIL POLL, V224, DOI 10.1007/s11270-012-1426-8"
"   Ashkanani Z, 2024, J HAZARD MATER, V468, DOI 10.1016/j.jhazmat.2024.133813"
"   Attanayake CP, 2015, J ENVIRON QUAL, V44, P930, DOI 10.2134/jeq2014.09.0400"
"   Barbosa F, 2023, J TOXICOL ENV HEAL B, V26, P28, DOI 10.1080/10937404.2022.2164390"
"   Beesley L, 2010, ENVIRON POLLUT, V158, P2282, DOI 10.1016/j.envpol.2010.02.003"
"   Biswas B, 2015, J HAZARD MATER, V298, P129, DOI 10.1016/j.jhazmat.2015.05.009"
"   Ceci A, 2019, APPL MICROBIOL BIOT, V103, P53, DOI 10.1007/s00253-018-9451-1"
"   Chemla Y, 2025, NAT MICROBIOL, V10, P281, DOI 10.1038/s41564-024-01918-0"
"   Chen F, 2016, J HAZARD MATER, V302, P250, DOI 10.1016/j.jhazmat.2015.09.068"
"   Chen J, 2020, ENVIRON SCI TECHNOL, V54, P7261, DOI 10.1021/acs.est.0c00363"
"   Chlebek D, 2022, INT J MOL SCI, V23, DOI 10.3390/ijms23010214"
"   Czarny J, 2020, J HAZARD MATER, V383, DOI 10.1016/j.jhazmat.2019.121168"
"   Davin M, 2020, INT J ENV RES PUB HE, V17, DOI 10.3390/ijerph17114016"
"   de Lorenzo V, 2024, J BACTERIOL, V206, DOI 10.1128/jb.00136-24"
"   Dou RQ, 2024, SCI TOTAL ENVIRON, V912, DOI 10.1016/j.scitotenv.2023.169020"
"   Fan T, 2008, J HAZARD MATER, V160, P655, DOI 10.1016/j.jhazmat.2008.03.038"
"   Fu J, 2014, J HAZARD MATER, V270, P102, DOI 10.1016/j.jhazmat.2014.01.044"
"   Gauthier PT, 2015, ENVIRON SCI TECHNOL, V49, P11780, DOI 10.1021/acs.est.5b03233"
"   Gran-Scheuch A, 2020, MICROORGANISMS, V8, DOI 10.3390/microorganisms8111749"
"   Gupta G, 2024, GROUNDWATER SUST DEV, V26, DOI 10.1016/j.gsd.2024.101290"
"   Hashemi H, 2016, DESALIN WATER TREAT, V57, P24960, DOI 10.1080/19443994.2016.1144529"
"   He FP, 2009, J ENVIRON SCI-CHINA, V21, P675, DOI 10.1016/S1001-0742(08)62323-2"
"   Hkiri N, 2023, J FUNGI, V9, DOI 10.3390/jof9030299"
"   Huang CW, 2023, BIOENGINEERED, V14, P58, DOI 10.1080/21655979.2022.2095089"
"   Huang Q, 2025, SCI REP-UK, V15, DOI 10.1038/s41598-025-93715-w"
"   Huang ZL, 2022, J HAZARD MATER, V438, DOI 10.1016/j.jhazmat.2022.129492"
"   Jia CY, 2024, J ENVIRON CHEM ENG, V12, DOI 10.1016/j.jece.2024.114347"
"   Kim HJ, 2016, BIOSENS BIOELECTRON, V79, P701, DOI 10.1016/j.bios.2015.12.101"
"   Kravchenko E, 2025, J HAZARD MATER, V484, DOI 10.1016/j.jhazmat.2024.136751"
"   Lechner A, 2016, CSH PERSPECT BIOL, V8, DOI 10.1101/cshperspect.a023903"
"   Lee YY, 2025, PROCESSES, V13, DOI 10.3390/pr13010132"
"   Li GR, 2021, ENVIRON TECHNOL INNO, V24, DOI 10.1016/j.eti.2021.101963"
"   Li LY, 2016, BIOSENS BIOELECTRON, V75, P433, DOI 10.1016/j.bios.2015.09.003"
"   Li Mengting, 2024, Chemosphere, V349, P140954, DOI [10.1016/j.chemosphere.2023.140954, 10.1016/j.chemosphere.2023.140954]"
"   Li QW, 2020, APPL MICROBIOL BIOT, V104, P8999, DOI 10.1007/s00253-020-10854-y"
"   Li SJ, 2024, ENVIRON RES, V263, DOI 10.1016/j.envres.2024.120035"
"   Li Wei, 2023, Chemosphere, V336, P139234, DOI [10.1016/j.chemosphere.2023.139234, 10.1016/j.chemosphere.2023.139234]"
"   Li YQ, 2025, J HAZARD MATER, V486, DOI 10.1016/j.jhazmat.2025.137144"
"   Lin KH, 2020, ENERGY, V199, DOI 10.1016/j.energy.2020.117446"
"   Liu H, 2019, CHEMOSPHERE, V222, P517, DOI 10.1016/j.chemosphere.2019.01.180"
"   Liu SH, 2017, BIORESOURCE TECHNOL, V224, P25, DOI 10.1016/j.biortech.2016.11.095"
"   Liu WC, 2023, SCI TOTAL ENVIRON, V895, DOI 10.1016/j.scitotenv.2023.164947"
"   Liu XX, 2019, FRONT MICROBIOL, V10, DOI 10.3389/fmicb.2019.00408"
"   Ma XK, 2016, APPL MICROBIOL BIOT, V100, P7741, DOI 10.1007/s00253-016-7595-4"
"   Ma XK, 2014, APPL MICROBIOL BIOT, V98, P9817, DOI 10.1007/s00253-014-5905-2"
"   Mao JW, 2024, BIOTECHNOL ADV, V74, DOI 10.1016/j.biotechadv.2024.108401"
"   Min BK, 2018, B KOREAN CHEM SOC, V39, P1047, DOI 10.1002/bkcs.11548"
"   Mohsin MZ, 2021, SYN SYST BIOTECHNO, V6, P180, DOI 10.1016/j.synbio.2021.07.002"
"   Muratova AY, 2024, MICROBIOLOGY+, V93, pS148, DOI 10.1134/S0026261724609643"
"   Obi L, 2020, J CHEM TECHNOL BIOT, V95, P1569, DOI 10.1002/jctb.6352"
"   Obuekwe IS, 2013, ENVIRON POLLUT, V180, P180, DOI 10.1016/j.envpol.2013.05.020"
"   Pacwa-Plociniczak M, 2024, MICROBIOL RES, V284, DOI 10.1016/j.micres.2024.127738"
"   Pan XL, 2010, COLLOID SURFACE B, V80, P103, DOI 10.1016/j.colsurfb.2010.05.002"
"   Pande V, 2022, FRONT MICROBIOL, V13, DOI 10.3389/fmicb.2022.824084"
"   Peng JY, 2021, ENVIRON POLLUT, V272, DOI 10.1016/j.envpol.2020.116038"
"   Qattan SYA, 2025, ENVIRON SCI EUR, V37, DOI 10.1186/s12302-025-01103-y"
"   Qian SX, 2023, SCI TOTAL ENVIRON, V870, DOI 10.1016/j.scitotenv.2023.162024"
"   Qin RL, 2022, MICROBIOL SPECTR, V10, DOI 10.1128/spectrum.00910-22"
"   Qu XY, 2024, ENVIRON RES, V263, DOI 10.1016/j.envres.2024.120211"
"   Rajendran S, 2022, CHEMOSPHERE, V287, DOI 10.1016/j.chemosphere.2021.132369"
"   Ranjard L, 2003, APPL ENVIRON MICROB, V69, P3784, DOI 10.1128/AEM.69.7.3784-3790.2003"
"   Ray S, 2024, EMERG CONTAM, V10, DOI 10.1016/j.emcon.2024.100358"
"   Rijk I, 2024, SCI TOTAL ENVIRON, V936, DOI 10.1016/j.scitotenv.2024.173454"
"   Rorat A, 2017, J ENVIRON MANAGE, V187, P347, DOI 10.1016/j.jenvman.2016.10.062"
"   Saeedi M, 2018, J ENVIRON MANAGE, V214, P204, DOI 10.1016/j.jenvman.2018.02.065"
"   Saparpakorn P, 2007, MOLECULES, V12, P703, DOI 10.3390/12040703"
"   Sayara T, 2020, APPL SCI-BASEL, V10, DOI 10.3390/app10113684"
"   Shang XT, 2024, J HAZARD MATER, V466, DOI 10.1016/j.jhazmat.2024.133684"
"   Shen QH, 2023, FRONT MICROBIOL, V14, DOI 10.3389/fmicb.2023.1143742"
"   Somtrakoon K, 2009, J CHEM TECHNOL BIOT, V84, P1660, DOI 10.1002/jctb.2226"
"   Song LC, 2022, CHEMOSPHERE, V307, DOI 10.1016/j.chemosphere.2022.136084"
"   Tang LS, 2025, J ENVIRON CHEM ENG, V13, DOI 10.1016/j.jece.2025.116081"
"   Tang LY, 2024, SCI TOTAL ENVIRON, V918, DOI 10.1016/j.scitotenv.2024.170744"

"   Tesfaye EL, 2025, EMERG CONTAM, V11, DOI 10.1016/j.emcon.2024.100424"
"   Tomaszewski EJ, 2016, CHEM GEOL, V438, P163, DOI 10.1016/j.chemgeo.2016.06.016"
"   Verma S, 2019, ENVIRON TECHNOL INNO, V14, DOI 10.1016/j.eti.2019.100369"
"   Wang HJ, 2025, J HAZARD MATER, V485, DOI 10.1016/j.jhazmat.2024.136946"
"   Wang K, 2012, J SOIL SEDIMENT, V12, P1089, DOI 10.1007/s11368-012-0539-4"
"   Wang YH, 2024, FRONT PLANT SCI, V15, DOI 10.3389/fpls.2024.1505670"
"   Wu C, 2021, J ENVIRON MANAGE, V296, DOI 10.1016/j.jenvman.2021.113185"
"   Wu ML, 2020, CHEMOSPHERE, V256, DOI 10.1016/j.chemosphere.2020.126998"
"   Wu SH, 2019, J HAZARD MATER, V364, P108, DOI 10.1016/j.jhazmat.2018.09.094"
"   Xu Y, 2021, J HAZARD MATER, V412, DOI 10.1016/j.jhazmat.2021.125340"
"   Yang ZN, 2022, ENVIRON SCI ECOTECH, V10, DOI 10.1016/j.ese.2022.100169"
"   Yi SW, 2023, J ENVIRON SCI, V132, P71, DOI 10.1016/j.jes.2022.07.017"
"   Yi SW, 2022, J HAZARD MATER, V424, DOI 10.1016/j.jhazmat.2021.127514"
"   Yin C, 2020, MICROORGANISMS, V8, DOI 10.3390/microorganisms8060946"
"   Zafra G, 2017, CHEMOSPHERE, V172, P120, DOI 10.1016/j.chemosphere.2016.12.038"
"   Zeng WM, 2020, RSC ADV, V10, P20385, DOI 10.1039/c9ra10560c"
"   Zhang SC, 2017, SCI REP-UK, V7, DOI 10.1038/s41598-017-12437-w"
"   Zhang WH, 2011, CHEMOSPHERE, V83, P302, DOI 10.1016/j.chemosphere.2010.12.056"
"   Zhang X, 2022, SCI REP-UK, V12, DOI 10.1038/s41598-022-07577-7"
"   Zhang Y, 2023, BIOCHAR, V5, DOI 10.1007/s42773-023-00241-x"
"   Zhang ZX, 2023, J CLEAN PROD, V383, DOI 10.1016/j.jclepro.2022.135417"
"   Zheng XC, 2022, WASTE MANAGE, V150, P75, DOI 10.1016/j.wasman.2022.06.033"
"   Zhou M, 2024, SCI TOTAL ENVIRON, V950, DOI 10.1016/j.scitotenv.2024.175364"
"   Zhu XJ, 2024, J HAZARD MATER, V465, DOI 10.1016/j.jhazmat.2023.133119"
NR 109
TC 2
Z9 3
U1 44
U2 59
PU SPRINGER
PI DORDRECHT
"PA VAN GODEWIJCKSTRAAT 30, 3311 GZ DORDRECHT, NETHERLANDS"
SN 0959-3993
EI 1573-0972
J9 WORLD J MICROB BIOT
JI World J. Microbiol. Biotechnol.
PD JUL 28
PY 2025
VL 41
IS 8
AR 280
DI 10.1007/s11274-025-04498-1
PG 13
WC Biotechnology & Applied Microbiology
WE Science Citation Index Expanded (SCI-EXPANDED)
SC Biotechnology & Applied Microbiology
GA 5MQ7E
UT WOS:001539306500006
PM 40719956
DA 2026-04-06
ER

PT J
"AU Si, SC"
"   Ke, YX"
"   Xue, BQ"
"   Zhang, ZY"
"   Zhu, XL"
"AF Si, Shaocheng"
"   Ke, Yuxin"
"   Xue, Boqian"
"   Zhang, Ziye"
"   Zhu, Xiaoli"
TI Immobilized sulfate reducing bacteria (SRB) enhanced passivation
   performance of biochar for Zn
SO SCIENCE OF THE TOTAL ENVIRONMENT
LA English
DT Article
DE Biochar; Sulfate-reducing bacteria; Zn; Passivation; Immobilized
   bacteria
ID HEAVY-METALS; ZINC; COPPER; SOIL; ADSORPTION; POLLUTION; CULTURES;
   IMPACT
"AB The low survival rate in harsh environments has been hindering the transformation of microbial remediation technology for heavy metal-contaminated soil from laboratory to field application. Therefore, biochar was selected as the carrier in this study to immobilize the heavy metal tolerant sulfate-reducing bacteria of SRB14-2-3 for the passivation of Zncontaminated soil. The results showed that the immobilized bacteria of IBWS14-2-3 exhibited the highest passivation performance, as the total content of bioavailable Zn fractions (exchangeable + carbonates) in soil with initial Zn concentration of 350, 750 and 1500 mg &BULL;kg-1 decreased by almost 34.2 %, 30.0 % and 22.2 % compared to the control group, respectively. In addition, the introducing of SRB14-2-3 into biochar has successfully alleviated the possible adverse effects on soil caused by the application of large amounts of biochar, while the protection of biochar against immobilized bacteria significantly improved the reproduction of SRB14-2-3, since the number of SRB14-2-3 increased by 82,278, 42 and 5 times in three different level contaminated soil. Furthermore, the new passivation mechanism for heavy metals brought by SRB14-2-3 is expected to make up for the deficiency of

biochar in long-term application. And more attention should be paid on the performance of immobilized bacteria under field practical application in the future research."
"C1 [Si, Shaocheng; Ke, Yuxin; Xue, Boqian; Zhu, Xiaoli] Northwest Univ, Coll Urban & Environm Sci, Xian 710127, Peoples R China."
"   [Si, Shaocheng; Ke, Yuxin; Xue, Boqian; Zhu, Xiaoli] Shaanxi Key Lab Earth Surface Syst & Environm Carr, Xian 710127, Peoples R China."
"   [Zhang, Ziye] Xian Jinborui Ecol Tech Co Ltd, Xian 710065, Peoples R China."
C3 Northwest University Xi'an
"RP Zhu, XL (corresponding author), Northwest Univ, Coll Urban & Environm Sci, Xian 710127, Peoples R China."
EM xiaolizhu@nwu.edu.cn
FU National Key Research and Development Program of China [2021YFC1808902];
"   Key Research and Development Program of Shaanxi Province [2019NY-200,"
"   2020ZDLNY06-06, 2020ZDLNY07-10]; Science and Technology Program of Xi'an"
"   City [2019-GXYD18.9, 20193057YF045NS045]; Ag-ricultural Science and"
   Technology Innovation Project of Shaanxi Province [NYJK-2022-XA-02]
FX Acknowledgements This work was supported by the National Key Research
"   and Development Program of China (2021YFC1808902) , Key Research and"
   Development Program of Shaanxi Province (Grant No. 2019NY-200;
"   2020ZDLNY06-06; 2020ZDLNY07-10) , Science and Technology Program of"
   Xi'an City (Grant No. 2019-GXYD18.9; 20193057YF045NS045) and
   Ag-ricultural Science and Technology Innovation Project of Shaanxi
   Province (Grant No. NYJK-2022-XA-02) .
"CR Ajeng AA, 2020, ENVIRON TECHNOL INNO, V20, DOI 10.1016/j.eti.2020.101168"
"   Byers HK, 1998, FEMS MICROBIOL ECOL, V25, P391, DOI 10.1016/S0168-6496(98)00013-0"
"   Cai L, 2005, CURR MED CHEM, V12, P2753, DOI 10.2174/092986705774462950"
"   Cai LQ, 2015, ENVIRON EARTH SCI, V74, P803, DOI 10.1007/s12665-015-4084-5"
"   Cao J, 2022, ECOTOXICOLOGY, V31, P259, DOI 10.1007/s10646-021-02505-3"
"   CORDRUWISCH R, 1985, J MICROBIOL METH, V4, P33, DOI 10.1016/0167-7012(85)90005-3"
"   Cui S, 2024, BIOMASS CONVERS BIOR, V14, P12761, DOI 10.1007/s13399-022-03289-0"
"   Cui ZJ, 2017, J ENVIRON CHEM ENG, V5, P3616, DOI 10.1016/j.jece.2017.07.021"
"   Duan MP, 2023, CRIT REV FOOD SCI, V63, P1277, DOI 10.1080/10408398.2021.1963664"
"   Fan JP, 2020, SCI TOTAL ENVIRON, V713, DOI 10.1016/j.scitotenv.2020.136663"
"   Ge YY, 2018, ARCH AGRON SOIL SCI, V64, P708, DOI 10.1080/03650340.2017.1373186"
"   Inyang MI, 2016, CRIT REV ENV SCI TEC, V46, P406, DOI 10.1080/10643389.2015.1096880"
"   Jiang SS, 2016, CHEMOSPHERE, V142, P64, DOI 10.1016/j.chemosphere.2015.06.079"
"   Ke YX, 2023, SCI TOTAL ENVIRON, V867, DOI 10.1016/j.scitotenv.2023.161593"
"   Ke YX, 2022, CHEMOSPHERE, V296, DOI 10.1016/j.chemosphere.2022.134051"
"   Kuppusamy S, 2016, J HAZARD MATER, V317, P169, DOI 10.1016/j.jhazmat.2016.05.066"
"   Li X, 2017, J HAZARD MATER, V324, P340, DOI 10.1016/j.jhazmat.2016.10.067"
"   Li ZJ, 2020, J POROUS MAT, V27, P189, DOI 10.1007/s10934-019-00802-1"
"   Liu YL, 2020, CHEMOSPHERE, V247, DOI 10.1016/j.chemosphere.2020.125850"
"   Lv Y, 2022, POL J ENVIRON STUD, V31, P1747, DOI 10.15244/pjoes/141326"
"   Naz M, 2022, J ENVIRON MANAGE, V321, DOI 10.1016/j.jenvman.2022.115770"
"   Rowell D. L., 1994, Soil science: methods and applications."
"   Sha HC, 2023, ENVIRON RES, V218, DOI 10.1016/j.envres.2022.114769"
"   Sharma S, 2018, 3 BIOTECH, V8, DOI 10.1007/s13205-018-1237-8"
"   Sui L, 2021, J ENVIRON SCI, V106, P116, DOI 10.1016/j.jes.2021.01.001"
"   Tamura K, 2007, MOL BIOL EVOL, V24, P1596, DOI 10.1093/molbev/msm092"
"   Tao M, 2019, J CLEAN PROD, V237, DOI 10.1016/j.jclepro.2019.117833"
"   TESSIER A, 1979, ANAL CHEM, V51, P844, DOI 10.1021/ac50043a017"
"   The National Standard of People""s Republic of China, 2009, 1464352009 GBT NAT S"
"   Tu ZN, 2019, BIOCHAR, V1, P127, DOI 10.1007/s42773-019-00003-8"
"   Utgikar VP, 2003, BIOTECHNOL BIOENG, V82, P306, DOI 10.1002/bit.10575"
"   Wang DW, 2018, LAND DEGRAD DEV, V29, P3783, DOI 10.1002/ldr.3086"
"   Wang JL, 2019, J CLEAN PROD, V227, P1002, DOI 10.1016/j.jclepro.2019.04.282"
"   Wang Y, 2016, BIORESOURCE TECHNOL, V211, P711, DOI 10.1016/j.biortech.2016.03.149"
"   Wang YY, 2022, ENVIRON TECHNOL INNO, V28, DOI 10.1016/j.eti.2022.102622"
"   Wang YP, 2007, J ENVIRON SCI, V19, P848, DOI 10.1016/S1001-0742(07)60141-7"
"   Wu WL, 2022, J HAZARD MATER, V437, DOI 10.1016/j.jhazmat.2022.129330"
"   Xie YL, 2022, ECOTOX ENVIRON SAFE, V230, DOI 10.1016/j.ecoenv.2021.113107"
"   Xing D, 2022, J ENVIRON MANAGE, V321, DOI 10.1016/j.jenvman.2022.115832"
"   Zhai XQ, 2018, SCI TOTAL ENVIRON, V635, P92, DOI 10.1016/j.scitotenv.2018.04.119"
"   Zhou YC, 2023, SCI TOTAL ENVIRON, V861, DOI 10.1016/j.scitotenv.2022.160649"
"   Zhu XL, 2022, ECOTOX ENVIRON SAFE, V237, DOI 10.1016/j.ecoenv.2022.113524"
"   Zhu XL, 2019, GEODERMA, V350, P1, DOI 10.1016/j.geoderma.2019.04.015"
NR 43
TC 36
Z9 39
U1 29
U2 187
PU ELSEVIER
PI AMSTERDAM
"PA RADARWEG 29, 1043 NX AMSTERDAM, NETHERLANDS"
SN 0048-9697
EI 1879-1026
J9 SCI TOTAL ENVIRON
JI Sci. Total Environ.
PD SEP 20
PY 2023
VL 892
AR 164556
DI 10.1016/j.scitotenv.2023.164556
EA JUN 2023
PG 9

WC Environmental Sciences
WE Science Citation Index Expanded (SCI-EXPANDED)
SC Environmental Sciences & Ecology
GA L7JT8
UT WOS:001024992800001
PM 37270015
DA 2026-04-06
ER

PT J
"AU Patil, A"
"   Arya, M"
"   Yadav, BK"
"   Nahar, M"
"   Rajamohan, N"
"AF Patil, A."
"   Arya, M."
"   Yadav, B. K."
"   Nahar, M."
"   Rajamohan, N."
TI Removal of toxic arsenic and chromium from soil using
   biochar-immobilized Geobacillus and Bacillus species: a
   review
SO INTERNATIONAL JOURNAL OF ENVIRONMENTAL SCIENCE AND TECHNOLOGY
LA English
DT Review
DE Heavy metal; Biomineralization; Bacteria; Soil; Feedstock;
   Bioavailability
ID HEAVY-METALS; HEXAVALENT CHROMIUM; THERMOPHILIC BACTERIA;
   AQUEOUS-SOLUTION; MECHANISMS; ADSORPTION; WATER; SORPTION; CD; REDUCTION
"AB Heavy metals are highly hazardous compounds that must be managed carefully, economically, and sustainably. Bioremediation is an essential method for reducing the harmful levels of toxic heavy metal pollutants in the environment. Bacteria employ bio-sorption and bio-accumulation strategies that entail ATP-dependent substrate-specific sequestration to eliminate the contaminants through redox metabolism and enzyme-transformation procedures. Metal ions can be bound and transported to the bacterial cytoplasm via possible chemisorption sites in the bacterial cell wall. Immobilizing bacterial cells on biochar will improve the simultaneous potential, synergistic adsorption, and bioaccumulation of heavy metals from contaminated soil. Bacterial immobilization on biochar can be understood by the adsorption principles. Biochar that has been pyrolyzed at temperatures higher than 500 degrees C is suggested for immobilizing bacteria. Extra polymeric substances (EPS) released from bacterial cells lessen the stress of metal contaminants during bioremediation by immobilizing bacteria on biochar. The use of biochar-immobilized Bacillus spp. and Geobacillus spp. was investigated in this review study. For example, biochar mediated by Bacillus subtilis has a 99.9% maximal capacity to absorb harmful metallic substances from the environment. Bio-adsorption, biomineralization, biosorption, bio-reduction, and bioaccumulation are important steps of this metal elimination procedure. Nevertheless, more investigation is required to comprehend the fundamental mechanisms better, investigate potential solutions, and evaluate the overall effectiveness of this new technology."
"C1 [Patil, A.] Graph Era Deemed Be Univ, Dept Biotechnol, Dehra Dun 248001, Uttarakhand, India."
"   [Arya, M.] Hemvati Nandan Bahuguna Garhwal Univ, Dept Biotechnol, Srinagar 246174, Uttarakhand, India."
"   [Yadav, B. K.] Indian Inst Technol, Dept Hydrol, Roorkee 247667, Uttarakhand, India."
"   [Nahar, M.] Swami Vivekananda Univ, Dept Biotechnol, Barackpore 700121, West Bengal, India."
"   [Rajamohan, N.] Sohar Univ, Fac Engn, Chem Engn Sect, PC-311, Sohar, Oman."
C3 Graphic Era University; Hemwati Nandan Bahuguna Garhwal University;
   Indian Institute of Technology System (IIT System); Indian Institute of
   Technology (IIT) - Roorkee; Sohar University
"RP Rajamohan, N (corresponding author), Sohar Univ, Fac Engn, Chem Engn Sect, PC-311, Sohar, Oman."
EM rnatarajan@su.edu.om
"RI Rajamohan, Natarajan/W-2308-2019"
"OI Rajamohan, Natarajan/0000-0002-5015-0694"
"CR Abdu N, 2017, ENVIRON CHEM LETT, V15, P65, DOI 10.1007/s10311-016-0587-x"
"   Agegnehu G, 2017, APPL SOIL ECOL, V119, P156, DOI 10.1016/j.apsoil.2017.06.008"
"   Ahmad M, 2014, CHEMOSPHERE, V99, P19, DOI 10.1016/j.chemosphere.2013.10.071"
"   Ahmad W, 2021, SCI REP-UK, V11, DOI 10.1038/s41598-021-94616-4"
"   Akhil D, 2021, ENVIRON CHEM LETT, V19, P2261, DOI 10.1007/s10311-020-01167-7"
"   Ali N., 2024, BACTERIAL SECONDARY, P221, DOI [10.1016/B978-0-323-95251-4.00021-, DOI 10.1016/B978-0-323-95251-4.00021, 10.1016/B978-0-323-95251-4.00021-1, DOI 10.1016/B978-0-323-95251-4.00021-1]"
"   An XJ, 2024, J HAZARD MATER, V480, DOI 10.1016/j.jhazmat.2024.136414"
"   Andersen ME, 2000, TOXICOL SCI, V53, P159, DOI 10.1093/toxsci/53.2.159"
"   [Anonymous], 2019, International Agency for Research on Cancer"
"   Antonangelo JA, 2021, J ENVIRON MANAGE, V277, DOI 10.1016/j.jenvman.2020.111443"
"   Anwar S, 2023, PAK J BOT, V55, P1931, DOI 10.30848/PJB2023-5(43)"
"   Arif I, 2020, TRENDS BIOTECHNOL, V38, P1385, DOI 10.1016/j.tibtech.2020.04.015"
"   Arya M., 2023, Water Manag Governance, V10, P194"
"   Atalah J, 2019, BIORESOURCE TECHNOL, V280, P478, DOI 10.1016/j.biortech.2019.02.008"
"   Ayangbenro AS, 2017, INT J ENV RES PUB HE, V14, DOI 10.3390/ijerph14010094"
"   Babau AMC, 2024, SCI REP-UK, V14, DOI 10.1038/s41598-024-60412-z"
"   Bee Aksha, 2024, Journal of Environment and Bio-Sciences, V38, P59, DOI [10.59467/jebs.2024.38.59, 10.59467/JEBS.2024.38.59]"
"   Beesley L, 2013, SCI TOTAL ENVIRON, V454, P598, DOI [10.1016/j.scitotenv.2013.02.047, 10.1016/j.scitotenv2013.02.047]"
"   Bekchanova M, 2024, ENVIRON EVID, V13, DOI 10.1186/s13750-024-00326-5"
"   Briffa J, 2020, HELIYON, V6, DOI 10.1016/j.heliyon.2020.e04691"
"   Burrell LD, 2016, GEODERMA, V282, P96, DOI 10.1016/j.geoderma.2016.07.019"
"   Cao XD, 2009, ENVIRON SCI TECHNOL, V43, P3285, DOI 10.1021/es803092k"
"   Carlin DJ, 2016, ENVIRON HEALTH PERSP, V124, P890, DOI 10.1289/ehp.1510209"

"   Chatterjee SK, 2010, J HAZARD MATER, V175, P117, DOI 10.1016/j.jhazmat.2009.09.136"
"   Chatzimichailidou S, 2023, J COMPOS SCI, V7, DOI 10.3390/jcs7020059"
"   Chen P, 2024, J ENVIRON SCI, V137, P515, DOI 10.1016/j.jes.2023.02.023"
"   Chen Xi, 2023, Chemosphere, V338, P139466, DOI [10.1016/j.chemosphere.2023.139466, 10.1016/j.chemosphere.2023.139466]"
"   Chen X, 2022, J CLEAN PROD, V370, DOI 10.1016/j.jclepro.2022.133483"
"   Chen YY, 2021, FRONT MICROBIOL, V12, DOI 10.3389/fmicb.2021.641913"
"   Cheng C, 2020, ECOTOX ENVIRON SAFE, V206, DOI 10.1016/j.ecoenv.2020.111189"
"   Choppala GK, 2012, J ENVIRON QUAL, V41, P1175, DOI 10.2134/jeq2011.0145"
"   Coelho E, 2020, BIOTECHNOL PROGR, V36, DOI 10.1002/btpr.3032"
"   Dan YT, 2023, ENVIRON POLLUT, V320, DOI 10.1016/j.envpol.2023.121109"
"   Dayan AD, 2001, HUM EXP TOXICOL, V20, P439, DOI 10.1191/096032701682693062"
"   de Figueiredo CC, 2021, WASTE MANAGE, V124, P144, DOI 10.1016/j.wasman.2021.01.044"
"   Demisie W., 2015, African Journal of Agricultural Research, V10, P755, DOI 10.5897/ajar2013.8209"
"   Deng M, 2022, ENVIRON SCI POLLUT R, V29, P18495, DOI 10.1007/s11356-021-17125-x"
"   Desaules A, 2012, SCI TOTAL ENVIRON, V426, P120, DOI 10.1016/j.scitotenv.2012.03.035"
"   Dhaliwal SS, 2020, ENVIRON SCI POLLUT R, V27, P1319, DOI 10.1007/s11356-019-06967-1"
"   Dong XL, 2014, J HAZARD MATER, V267, P62, DOI 10.1016/j.jhazmat.2013.12.027"
"   Dong XL, 2011, J HAZARD MATER, V190, P909, DOI 10.1016/j.jhazmat.2011.04.008"
"   Duarte SD, 2019, AGRONOMY-BASEL, V9, DOI 10.3390/agronomy9040165"
"   El-Bassi L, 2021, SCI TOTAL ENVIRON, V755, DOI 10.1016/j.scitotenv.2020.142531"
"   Embrandiri Asha, 2012, Environmentalist, V32, P111, DOI 10.1007/s10669-011-9367-0"
"   Fahimirad Shohreh, 2017, MED PLANTS ENV CHALL, P189, DOI 10.1007/978-3-319-68717-911"
"   Favas PJC, 2011, J GEOCHEM EXPLOR, V111, P160, DOI 10.1016/j.gexplo.2011.04.009"
"   Fijalkowska G, 2021, CHEMOSPHERE, V276, DOI 10.1016/j.chemosphere.2021.130088"
"   Gautam K, 2023, ENVIRON RES, V225, DOI 10.1016/j.envres.2023.115592"
"   Genchi G, 2022, APPL SCI-BASEL, V12, DOI 10.3390/app12126184"
"   Glaser B, 2012, GEOCHIM COSMOCHIM AC, V82, P39, DOI 10.1016/j.gca.2010.11.029"
"   Godwill EA, 2019, POISONING MODERN WOR"
"   Gong X, 2019, IOP C SER EARTH ENV, V267, DOI 10.1088/1755-1315/267/5/052064"
"   Guo XL, 2020, ACS OMEGA, V5, P27323, DOI 10.1021/acsomega.0c03652"
"   Han YT, 2016, CHEMOSPHERE, V145, P336, DOI 10.1016/j.chemosphere.2015.11.050"
"   Hassan A, 2022, INT J ENVIRON SCI TE, V19, P6729, DOI 10.1007/s13762-021-03626-2"
"   Hassan A, 2019, WATER AIR SOIL POLL, V230, DOI 10.1007/s11270-019-4227-5"
"   He MJ, 2021, BIORESOURCE TECHNOL, V341, DOI 10.1016/j.biortech.2021.125811"
"   Herath HMSK, 2013, GEODERMA, V209, P188, DOI 10.1016/j.geoderma.2013.06.016"
"   Horckmans L, 2007, SCI TOTAL ENVIRON, V376, P86, DOI 10.1016/j.scitotenv.2007.01.077"
"   Huang JR, 2022, BIORESOURCE TECHNOL, V348, DOI 10.1016/j.biortech.2022.126790"
"   Huang YT, 2024, J HAZARD MATER, V469, DOI 10.1016/j.jhazmat.2024.133966"
"   Igiri BE, 2018, J TOXICOL-US, V2018, DOI 10.1155/2018/2568038"
"   Irshad S, 2021, ENVIRON POLLUT, V291, DOI 10.1016/j.envpol.2021.118269"
"   Jardine TD, 2020, PHILOS T R SOC B, V375, DOI 10.1098/rstb.2019.0639"
"   Ji XW, 2022, SCI TOTAL ENVIRON, V811, DOI 10.1016/j.scitotenv.2021.152112"
"   Jiang YT, 2022, WATER-SUI, V14, DOI 10.3390/w14060894"
"   Kalaivanan D., 2016, ABIOTIC STRESS PHYSL, V9, P85, DOI [10.1007/978-81-322-2725-0_5, DOI 10.1007/978-81-322-2725-0_5]"
"   Kameda T, 2024, RESULTS CHEM, V7, DOI 10.1016/j.rechem.2024.101473"
"   Karim S, 2021, RESOUR CONSERV RECY, V170, DOI 10.1016/j.resconrec.2021.105588"
"   Khalid S, 2017, J GEOCHEM EXPLOR, V182, P247, DOI 10.1016/j.gexplo.2016.11.021"
"   Khan M.S., 2011, Biomanagement of Metal-contaminated Soils"
"   Khiari B, 2019, RENEW SUST ENERG REV, V108, P253, DOI 10.1016/j.rser.2019.03.057"
"   Kim Hyun Soo, 2015, J Cancer Prev, V20, P232, DOI [10.15430/jcp.2015.20.4.232, 10.15430/JCP.2015.20.4.232]"
"   Kim I, 2014, J ENVIRON MANAGE, V146, P568, DOI 10.1016/j.jenvman.2014.05.042"
"   Kiran, 2022, MATER TODAY-PROC, V51, P880, DOI 10.1016/j.matpr.2021.06.278"
"   Koedrith P, 2013, INT J HYG ENVIR HEAL, V216, P587, DOI 10.1016/j.ijheh.2013.02.010"
"   Kubier A, 2019, APPL GEOCHEM, V108, DOI 10.1016/j.apgeochem.2019.104388"
"   Kumar V, 2019, CHEMOSPHERE, V216, P449, DOI 10.1016/j.chemosphere.2018.10.066"
"   Lai HJ, 2023, BIOGEOTECHNICS, V1, DOI [10.1016/j.bgtech.2023.100039, 10.1016/j.bgtech.2023.100039]"
"   Laulicht F., 2015, HDB TOXICOLOGY METAL"
"   Lebrun M, 2021, ENVIRON SCI POLLUT R, V28, P11195, DOI 10.1007/s11356-020-11355-1"
"   Li C, 2020, ENVIRON POLLUT, V260, DOI 10.1016/j.envpol.2020.114075"
"   Li HB, 2017, CHEMOSPHERE, V178, P466, DOI 10.1016/j.chemosphere.2017.03.072"
"   Li ZG, 2019, INT J ENV RES PUB HE, V16, DOI 10.3390/ijerph16193676"
"   Liang JL, 2021, SCI TOTAL ENVIRON, V774, DOI 10.1016/j.scitotenv.2021.145762"
"   Liao YH, 2024, BMC PLANT BIOL, V24, DOI 10.1186/s12870-024-05372-2"
"   Lipinska A, 2021, WATER AIR SOIL POLL, V232, DOI 10.1007/s11270-020-04950-y"
"   Liu G, 2020, J CLEAN PROD, V251, DOI 10.1016/j.jclepro.2019.119721"
"   Liu XH, 2022, BIORESOURCE TECHNOL, V345, DOI 10.1016/j.biortech.2021.126523"
"   Liu ZL, 2016, J HYDROL, V533, P461, DOI 10.1016/j.jhydrol.2015.12.007"
"   Ma H, 2020, J HAZARD MATER, V388, DOI 10.1016/j.jhazmat.2020.122065"
"   Ma L, 2018, ENVIRON SCI POLLUT R, V25, P64, DOI 10.1007/s11356-016-7906-4"
"   Mahurpawar M., 2015, International Journal of Research-Granthaalayah, V3, P1, DOI DOI 10.29121/GRANTHAALAYAH.V3.I9SE.2015.3282"
"   Misra V, 2009, ENVIRON MONIT ASSESS, V155, P467, DOI 10.1007/s10661-008-0449-5"
"   Mohammed A. S., 2011, BIOMANAGEMENT METAL, P1, DOI [10.1007/978-94-007-1914-9_1, DOI 10.1007/978-94-007-1914-9_1]"
"   Molaey Rahim, 2024, Science of The Total Environment, V955, P177020, DOI [10.1016/j.scitotenv.2024.177020, 10.1016/j.scitotenv.2024.177020]"
"   Mota R., 2022, Polysaccharides of microbial origin: biomedical applications, P139, DOI [DOI 10.1007/978-3-030-42215-8_11, 10.1007/978-3-030-42215-8_11]"
"   Murtaza G, 2021, J PLANT NUTR, V44, P1677, DOI 10.1080/01904167.2021.1871746"
"   Narayanan M, 2022, FRONT MICROBIOL, V13, DOI 10.3389/fmicb.2022.929730"
"   Nejad ZD, 2018, ENVIRON GEOCHEM HLTH, V40, P927, DOI 10.1007/s10653-017-9964-z"

"   Noreen S, 2024, S AFR J BOT, V172, P720, DOI 10.1016/j.sajb.2024.07.054"
"   Nurzhan A, 2022, EURASIAN SOIL SCI+, V55, P1425, DOI 10.1134/S106422932210012X"
"   ??zdemir S, 2013, BIOREMEDIAT J, V17, P86, DOI 10.1080/10889868.2012.751961"
"   ??zdemir S, 2009, CHEM ENG J, V152, P195, DOI 10.1016/j.cej.2009.04.041"
"   Pan JJ, 2013, J ENVIRON SCI-CHINA, V25, P1957, DOI 10.1016/S1001-0742(12)60305-2"
"   Pandit NR, 2018, SCI TOTAL ENVIRON, V625, P1380, DOI 10.1016/j.scitotenv.2018.01.022"
"   Patil A, 2025, WASTE BIOMASS VALORI, V16, P175, DOI 10.1007/s12649-024-02713-y"
"   Patil Anjali, 2024, Journal of Mountain Research, V19, P171, DOI 10.51220/jmr.v19i1.19"
"   Patil A, 2025, BIOREMEDIAT J, V29, P448, DOI 10.1080/10889868.2024.2375204"
"   Petersen EJ, 2019, ENVIRON SCI-NANO, V6, P1619, DOI [10.1039/c8en01378k, 10.1039/C8EN01378K]"
"   Prakash S, 2021, The International Journal of Biological Innovations, V03, P38, DOI [10.46505/ijbi.2021.3102, 10.46505/IJBI.2021.3102, DOI 10.46505/IJBI.2021.3102]"
"   Pramanick B., 2025, BIOCHAR MITIGATING A, P271"
"   Qi WY, 2023, J HAZARD MATER, V458, DOI 10.1016/j.jhazmat.2023.131921"
"   Qi X, 2022, J HAZARD MATER, V436, DOI 10.1016/j.jhazmat.2022.129147"
"   Qi X, 2021, J HAZARD MATER, V401, DOI 10.1016/j.jhazmat.2020.123823"
"   Qian LB, 2016, BIORESOURCE TECHNOL, V206, P217, DOI 10.1016/j.biortech.2016.01.065"
"   Rai PK, 2019, ENVIRON INT, V125, P365, DOI 10.1016/j.envint.2019.01.067"
"   Rengarajan S, 2025, J TAIWAN INST CHEM E, V166, DOI 10.1016/j.jtice.2023.105330"
"   Sall ML, 2020, ENVIRON SCI POLLUT R, V27, P29927, DOI 10.1007/s11356-020-09354-3"
"   Samsuri AW, 2014, INT J ENVIRON SCI TE, V11, P967, DOI 10.1007/s13762-013-0291-3"
"   S??nchez-Castro I, 2023, HELIYON, V9, DOI 10.1016/j.heliyon.2023.e16692"
"   Sarkhot DV, 2012, J ENVIRON QUAL, V41, P1107, DOI 10.2134/jeq2011.0123"
"   Satyapal G. K., 2016, Journal of Microbial and Biochemical Technology, V8, P256, DOI 10.4172/1948-5948.1000294"
"   Sawicka E, 2021, ANN AGR ENV MED, V28, P1, DOI 10.26444/aaem/118228"
"   Schommer VA, 2024, MICROBIOL RES, V278, DOI 10.1016/j.micres.2023.127534"
"   Schommer VA, 2023, SCI TOTAL ENVIRON, V881, DOI 10.1016/j.scitotenv.2023.163385"
"   Shar AG, 2025, SCIENTIFICA, V2025, DOI 10.1155/sci5/6302968"
"   Sharma PK, 2022, GROUNDWATER SUST DEV, V17, DOI 10.1016/j.gsd.2022.100740"
"   Shettigar H., 2025, PHYTOREMEDIATION FOO, P236"
"   Shin DY, 2023, INT J MOL SCI, V24, DOI 10.3390/ijms24043410"
"   Shrivastava A, 2015, CURR POLLUT REP, V1, P35, DOI 10.1007/s40726-015-0004-2"
"   Song DL, 2018, EUR J SOIL BIOL, V84, P1, DOI 10.1016/j.ejsobi.2017.11.003"
"   Song LC, 2022, CHEMOSPHERE, V307, DOI 10.1016/j.chemosphere.2022.136084"
"   Sreedevi PR, 2022, J WATER PROCESS ENG, V48, DOI 10.1016/j.jwpe.2022.102884"
"   Sun Hong, 2015, Curr Environ Health Rep, V2, P295, DOI [10.1007/s40572-015-0054-z, 10.1007/s40572-015-0054-z]"
"   Sun JZ, 2024, J ENVIRON MANAGE, V352, DOI 10.1016/j.jenvman.2024.120081"
"   Sun YC, 2022, WATER-SUI, V14, DOI 10.3390/w14111691"
"   Thakur M, 2022, CHEMOSPHERE, V287, DOI 10.1016/j.chemosphere.2021.131957"
"   Trakal L, 2014, BIORESOURCE TECHNOL, V171, P442, DOI 10.1016/j.biortech.2014.08.108"
"   Tu C, 2020, ENVIRON INT, V137, DOI 10.1016/j.envint.2020.105576"
"   Turner A, 2019, SCI TOTAL ENVIRON, V657, P1409, DOI 10.1016/j.scitotenv.2018.12.096"
"   Vishwakarma S. K., 2019, PERIODIC RES, V7, P195"
"   Vishwakarma SK, 2024, APPL BIOCHEM BIOTECH, V196, P2518, DOI 10.1007/s12010-023-04682-9"
"   Vishwakarma SK, 2025, GEOMICROBIOL J, V42, P420, DOI 10.1080/01490451.2025.2471448"
"   Wallace DR, 2020, CURR OPIN TOXICOL, V19, P72, DOI 10.1016/j.cotox.2020.01.001"
"   Wang L, 2021, J HAZARD MATER, V414, DOI 10.1016/j.jhazmat.2021.125494"
"   Wang SS, 2015, BIORESOURCE TECHNOL, V181, P13, DOI 10.1016/j.biortech.2015.01.044"
"   Wang SS, 2015, BIORESOURCE TECHNOL, V175, P391, DOI 10.1016/j.biortech.2014.10.104"
"   Wang T, 2018, ECOTOX ENVIRON SAFE, V148, P285, DOI 10.1016/j.ecoenv.2017.10.039"
"   Wang XR, 2020, CHEMOSPHERE, V257, DOI 10.1016/j.chemosphere.2020.127282"
"   Wang ZY, 2025, AGRICULTURE-BASEL, V15, DOI 10.3390/agriculture15010077"
"   Weber K, 2018, FUEL, V217, P240, DOI 10.1016/j.fuel.2017.12.054"
"   Wise JP Jr, 2022, ENVIRON INT, V158, DOI 10.1016/j.envint.2021.106877"
"   Wuana R.A., 2011, ISRN Ecology, DOI [10.5402/2011/402647, DOI 10.5402/2011/402647]"
"   Xu ZB, 2022, J HAZARD MATER, V430, DOI 10.1016/j.jhazmat.2022.128479"
"   Yang CD, 2022, SOIL TILL RES, V224, DOI 10.1016/j.still.2022.105505"
"   Yang X, 2019, J HAZARD MATER, V365, P178, DOI 10.1016/j.jhazmat.2018.10.047"
"   Younas H, 2022, ENVIRON SCI POLLUT R, V29, P57669, DOI 10.1007/s11356-022-19913-5"
"   Yu HW, 2019, J ENVIRON MANAGE, V232, P8, DOI 10.1016/j.jenvman.2018.10.117"
"   Zanganeh F, 2022, ENVIRON SCI POLLUT R, V29, P6040, DOI 10.1007/s11356-021-16061-0"
"   Zayed AM, 2003, PLANT SOIL, V249, P139, DOI 10.1023/A:1022504826342"
"   Zaynab M, 2022, J KING SAUD UNIV SCI, V34, DOI 10.1016/j.jksus.2021.101653"
"   Zhang DX, 2016, AGR ECOSYST ENVIRON, V226, P25, DOI 10.1016/j.agee.2016.04.010"
"   Zhang SN, 2021, ENVIRON TECHNOL INNO, V24, DOI 10.1016/j.eti.2021.101930"
"   Zhang WH, 2015, J ENVIRON QUAL, V44, P1119, DOI 10.2134/jeq2014.12.0536"
"   Zhao N, 2018, BIORESOURCE TECHNOL, V260, P294, DOI 10.1016/j.biortech.2018.03.116"
"   Zheng CJ, 2021, J HAZARD MATER, V407, DOI 10.1016/j.jhazmat.2020.124376"
"   Zhou HH, 2021, SCI TOTAL ENVIRON, V793, DOI 10.1016/j.scitotenv.2021.148529"
"   Zhou L, 2016, BIORESOURCE TECHNOL, V218, P351, DOI 10.1016/j.biortech.2016.06.102"
"   Zhu XJ, 2024, J HAZARD MATER, V465, DOI 10.1016/j.jhazmat.2023.133119"
"   Ziyaadini M, 2017, IRAN J FISH SCI, V16, P1123"
"   Zuo WL, 2022, CHEMOSPHERE, V307, DOI 10.1016/j.chemosphere.2022.135797"
"   Zwolak A, 2019, WATER AIR SOIL POLL, V230, DOI 10.1007/s11270-019-4221-y"
NR 173
TC 3
Z9 3
U1 20
U2 27
PU SPRINGER
PI NEW YORK
"PA ONE NEW YORK PLAZA, SUITE 4600, NEW YORK, NY, UNITED STATES"

SN 1735-1472
EI 1735-2630
J9 INT J ENVIRON SCI TE
JI Int. J. Environ. Sci. Technol.
PD NOV
PY 2025
VL 22
IS 15
BP 16143
EP 16166
DI 10.1007/s13762-025-06676-y
EA AUG 2025
PG 24
WC Environmental Sciences
WE Science Citation Index Expanded (SCI-EXPANDED)
SC Environmental Sciences & Ecology
GA 8EA2M
UT WOS:001541825000001
DA 2026-04-06
ER

PT J
"AU Chen, YL"
"   Zuo, MB"
"   Yang, D"
"   He, YQ"
"   Wang, HM"
"   Liu, XX"
"   Zhao, MJ"
"   Xu, LL"
"   Ji, J"
"   Liu, Y"
"   Gao, TP"
"AF Chen, Yueli"
"   Zuo, Mingbo"
"   Yang, Deng"
"   He, Yueqing"
"   Wang, Haoming"
"   Liu, Xiaoxiao"
"   Zhao, Minjuan"
"   Xu, Lingling"
"   Ji, Jing"
"   Liu, Ying"
"   Gao, Tianpeng"
TI Synergistically Effect of Heavy Metal Resistant Bacteria and Plants on
   Remediation of Soil Heavy Metal Pollution
SO WATER AIR AND SOIL POLLUTION
LA English
DT Article
DE Heavy metal contamination; Heavy metal-tolerant bacterium; Bidens pilosa
   L.; Medicago sativa L.; Microbial-phytoremediation remediation
ID STRESS; PHOSPHATE; GROWTH; ACCUMULATION; L.
"AB Microorganisms in the soil that have been contaminated with heavy metals for a long-time exhibited resistance to heavy metals. In recent years, the use of resistant microbes and heavy metal-tolerant plants to repair heavy metal-contaminated soil has become an increasingly hot subject. In this study, heavy metal-tolerant bacteria ZG7 was isolated from the mining soils and injected into Bidens pilosa L. and Medicago sativa L.. The results revealed that: (1) Compared to the non-inoculated group, inoculation with ZG7 notably increased the dry weight and chlorophyll content in plants under heavy metal stress, as well as promoted the content of several types of antioxidant chemicals; (2) Combined remediation improved soil enzyme activities, with soil catalase, sucrase, and phosphatase activities remarkably increased by 136.06%, 124.17% and 180.14% in the ZG7- Bidens pilosa L.; And soil catalase, urease, and sucrase activities promoted by 68.07%, 130.08% and 239.79% in the ZG7- Medicago sativa L.; (3) The content of Pb and Zn in the ZG7- Medicago sativa L. combination soil were considerably lower (9.28% and 6.2%, respectively) than no strain group, but there was no substantial change in soil heavy metal content when combined with the Bidens pilosa L. remediation when compared to no strain group. In summary, the combination of strain ZG7 and plants had a good effect on the remediation of heavy metal contaminated soils, and ZG7- Medicago sativa L. had the best effect, which is a reliable technique for effective control of heavy metal pollution. This study provided theoretical support for microbial-phytoremediation joint remediation."
"C1 [Chen, Yueli; Yang, Deng; He, Yueqing; Wang, Haoming; Gao, Tianpeng] Lanzhou Jiaotong Univ, Sch Biol & Pharmaceut Engn, Lanzhou 730070, Peoples R China."
"   [Zuo, Mingbo; Liu, Xiaoxiao; Zhao, Minjuan; Xu, Lingling; Gao, Tianpeng] Xian Univ, Sch Biol & Environm Engn, Xian Key Lab Plant Stress Physiol & Ecol Remediat, Xian 710000, Peoples R China."
"   [Ji, Jing] Lanzhou Univ, Sch Life Sci, MOE, Key Lab Cell Act & Stress Adaptat, Lanzhou 730000, Gansu, Peoples R China."
"   [Liu, Ying] Northwest A&F Univ, Coll Life Sci, Shaanxi Key Lab Agr & Environm Microbiol, Yangling 712100, Peoples R China."
"   [Gao, Tianpeng] Lanzhou City Univ, Engn Ctr Pollut Control & Ecol Remediat Restorat M, Lanzhou 730070, Peoples R China."
C3 Lanzhou Jiaotong University; Xi'an University; Lanzhou University;
   Northwest A&F University - China; Lanzhou City University
"RP Gao, TP (corresponding author), Lanzhou Jiaotong Univ, Sch Biol & Pharmaceut Engn, Lanzhou 730070, Peoples R China.; Gao, TP (corresponding author), Xian Univ, Sch Biol & Environm Engn, Xian Key Lab Plant Stress Physiol & Ecol Remediat, Xian 710000, Peoples R China.; Gao, TP (corresponding author), Lanzhou City Univ, Engn Ctr Pollut Control & Ecol Remediat Restorat M, Lanzhou 730070, Peoples R China."

EM zkgtp@163.com
"RI Gao, Tianpeng/ABF-7377-2021; Liu, Xiaoxiao/AAE-7247-2020"
FU Key Research and Development Program of Gansu Province
FX No Statement Available
"CR Abdelkrim S, 2020, ECOTOX ENVIRON SAFE, V192, DOI 10.1016/j.ecoenv.2020.110260"
"   Ahsan MT, 2017, INT J PHYTOREMEDIAT, V19, P937, DOI 10.1080/15226514.2017.1303813"
"   Bai BX, 2020, PLANT SOIL, V455, P41, DOI 10.1007/s11104-020-04660-8"
"   Boscolo PRS, 2003, PHYTOCHEMISTRY, V62, P181, DOI 10.1016/S0031-9422(02)00491-0"
"   Bueis T, 2018, ANN FOREST SCI, V75, DOI 10.1007/s13595-018-0720-z"
"   Cai ZC, 2021, SCI REP-UK, V11, DOI 10.1038/s41598-021-83566-6"
"   Dhaliwal SS, 2020, ENVIRON SCI POLLUT R, V27, P1319, DOI 10.1007/s11356-019-06967-1"
"   Goodarzi A, 2020, ECOTOX ENVIRON SAFE, V201, DOI 10.1016/j.ecoenv.2020.110853"
"   Guo JK, 2015, ANTON LEEUW INT J G, V107, P1591, DOI 10.1007/s10482-015-0453-z"
"   Han F, 2006, PLANT SOIL, V289, P355, DOI 10.1007/s11104-006-9145-9"
"   [????????? Hei Zewen], 2019, [????€???????, Ecologic Science], V38, P218"
"   Hu HQ, 2014, ENVIRON TOXICOL CHEM, V33, P2455, DOI 10.1002/etc.2720"
"   Huang JL, 2023, J HAZARD MATER, V450, DOI 10.1016/j.jhazmat.2023.131013"
"   Hussain I, 2018, SCI TOTAL ENVIRON, V628-629, P1582, DOI 10.1016/j.scitotenv.2018.02.037"
"   Hussein KA, 2014, CURR MICROBIOL, V68, P717, DOI 10.1007/s00284-014-0530-y"
"   Jiang CY, 2008, CHEMOSPHERE, V72, P157, DOI 10.1016/j.chemosphere.2008.02.006"
"   Ju WL, 2020, APPL SOIL ECOL, V150, DOI 10.1016/j.apsoil.2019.103450"
"   Kan XQ, 2021, CHEMOSPHERE, V267, DOI 10.1016/j.chemosphere.2020.128909"
"   Kong Tao, 2021, Journal of Soil and Water Conservation, V35, P321, DOI 10.13870/j.cnki.stbcxb.2021.04.044"
"   Li XX, 2019, ECOTOX ENVIRON SAFE, V168, P1, DOI 10.1016/j.ecoenv.2018.10.012"
"   Lin H, 2021, J HAZARD MATER, V402, DOI 10.1016/j.jhazmat.2020.123829"
"   Liu SM, 2021, PLANTS-BASEL, V10, DOI 10.3390/plants10050912"
"   Miransari M, 2011, BIOTECHNOL ADV, V29, P645, DOI 10.1016/j.biotechadv.2011.04.006"
"   [????????? Mu Wenqiang], 2022, [????????????, Chinese Bulletin of Life Sciences], V34, P118"
"   Murshed R, 2013, PHYSIOL MOL BIOL PLA, V19, P363, DOI 10.1007/s12298-013-0173-7"
"   Pantoja-Guerra M, 2023, ANTON LEEUW INT J G, V116, P615, DOI 10.1007/s10482-023-01828-x"
"   Park JH, 2011, J HAZARD MATER, V185, P829, DOI 10.1016/j.jhazmat.2010.09.095"
"   Rajkumar M, 2012, BIOTECHNOL ADV, V30, P1562, DOI 10.1016/j.biotechadv.2012.04.011"
"   Raturi G, 2023, SCI TOTAL ENVIRON, V864, DOI 10.1016/j.scitotenv.2022.160972"
"   Rizvi A, 2018, ECOTOX ENVIRON SAFE, V157, P9, DOI 10.1016/j.ecoenv.2018.03.063"
"   Saha M, 2016, ENVIRON SCI POLLUT R, V23, P3984, DOI 10.1007/s11356-015-4294-0"
"   Selvi A, 2019, FRONT ENV SCI-SWITZ, V7, DOI 10.3389/fenvs.2019.00066"
"   Sheng XF, 2008, J HAZARD MATER, V155, P17, DOI 10.1016/j.jhazmat.2007.10.107"
"   Suman J, 2018, FRONT PLANT SCI, V9, DOI 10.3389/fpls.2018.01476"
"   Tian HX, 2017, CHEMOSPHERE, V187, P19, DOI 10.1016/j.chemosphere.2017.08.073"
"   Touceda-Gonzalez M, 2015, SOIL BIOL BIOCHEM, V91, P140, DOI 10.1016/j.soilbio.2015.08.038"
"   Wang CR, 2020, CHEMOSPHERE, V252, DOI 10.1016/j.chemosphere.2020.126603"
"   [????????? Wang Qiyuan], 2021, [????????€????€????, Biotechnology Bulletin], V37, P174"
"   Wang YX., 2016, Journal of Microbiology, V56, P1022, DOI [10.13343/j.cnki.wsxb.20150435, DOI 10.13343/J.CNKI.WSXB.20150435]"
"   Xiao CQ, 2013, J CENT SOUTH UNIV, V20, P2109, DOI 10.1007/s11771-013-1714-5"
"   Yang S, 2019, CHEMOSPHERE, V220, P69, DOI 10.1016/j.chemosphere.2018.12.101"
"   Yu FM, 2022, SCI TOTAL ENVIRON, V804, DOI 10.1016/j.scitotenv.2021.150282"
"   Yuan Fang, 2022, Huan Jing Ke Xue, V43, P4655, DOI 10.13227/j.hjkx.202112155"
"   Zhang HH, 2020, ECOTOX ENVIRON SAFE, V195, DOI 10.1016/j.ecoenv.2020.110469"
"   Zhang JH, 2010, WORLD J MICROB BIOT, V26, P371, DOI 10.1007/s11274-009-0163-7"
"   [????????? Zhang Xingyu], 2021, [??????????????????, Plant Physiology Journal], V57, P1437"
"   Zhang Xue-mei, 2017, Shengtaixue Zazhi, V36, P1097, DOI 10.13292/j.1000-4890.201704.001"
"   Zheng WK, 2022, ENVIRON CHEM LETT, V20, P2043, DOI 10.1007/s10311-022-01407-y"
"   [????????? Zhu Li'an], 2021, [????€?????????????, Ecology and Environmental Sciences], V30, P849"
NR 49
TC 16
Z9 16
U1 40
U2 133
PU SPRINGER INT PUBL AG
PI CHAM
"PA GEWERBESTRASSE 11, CHAM, CH-6330, SWITZERLAND"
SN 0049-6979
EI 1573-2932
J9 WATER AIR SOIL POLL
JI Water Air Soil Pollut.
PD MAY
PY 2024
VL 235
IS 5
AR 296
DI 10.1007/s11270-024-07100-w
PG 17
WC Environmental Sciences; Meteorology & Atmospheric Sciences; Water
   Resources
WE Science Citation Index Expanded (SCI-EXPANDED)
SC Environmental Sciences & Ecology; Meteorology & Atmospheric Sciences;
   Water Resources
GA RS1W7
UT WOS:001229569400010
DA 2026-04-06
ER

PT J
"AU Tang, CY"
"   Yao, J"
"   Liu, XY"
"   Masakorala, K"
"   Lv, Y"
"   Liu, JL"
"   Cao, Y"
"AF Tang, Chuiyun"
"   Yao, Jun"
"   Liu, Xingyu"
"   Masakorala, K."
"   Lv, Ying"
"   Liu, Jianli"
"   Cao, Ying"
TI Research progress in utilization of microbial immobilization technology
   in microbially induced mineralization of heavy metals
SO JOURNAL OF ENVIRONMENTAL CHEMICAL ENGINEERING
LA English
DT Article
DE Heavy metals; Immobilization methods; Interaction mechanisms;
   Microorganism; Microbial induced mineralization
ID SULFATE-REDUCING BACTERIA; PB-CONTAMINATED SOIL; WASTE-WATER; CALCITE
   PRECIPITATION; SIMULTANEOUS REMOVAL; NITROGEN REMOVAL; BIOREMEDIATION;
   BIODEGRADATION; DEGRADATION; BIOCHAR
"AB The microbial induced mineralization (MIM) is applied to the remediation of heavy metals contaminated sites, to minimize the risks associated with contaminated environments. However, the efficiency of heavy metal remediation is influenced by environmental factors. There is a high potential to enhance the efficiency of microbial remediation by effectively alleviating the pressure on microbes from environmental conditions by combining microbial immobilization technology (MIT) with MIM. However, research on the interaction mechanisms of MITMIM is limited and most of the studies in the literature are from laboratory studies. In this context, this review aims to summarize the research progress of MIT in MIM in the remediation of water and soil environments contaminated with heavy metals. The review begins by providing an overview of the types of MIM, including microbially induced carbonate precipitation (MICP), microbially induced phosphate precipitation (MIPP), and microbially induced sulfide precipitation (MISP). Subsequently, it introduces MIT methods, including physical adsorption, encapsulation, covalent binding, layer-by-layer self-assembly, and composite methods. Furthermore, the review explores the mechanisms of MIT-MIM and systematically summarizes research on the remediation of contaminated environments with heavy metals by applying MIT-MIM. Finally, it summarizes the challenges in the application of this technology and provides prospects for future research and practical applications."
"C1 [Tang, Chuiyun; Yao, Jun; Liu, Jianli] China Univ Geosci Beijing, Res Ctr Environm Sci & Engn, Sch Water Resources & Environm, 29 Xueyuan Rd, Beijing 100083, Haidian Distric, Peoples R China."
"   [Liu, Xingyu; Lv, Ying] China Univ Geosci Beijing, Inst Earth Sci, 29 Xueyuan Rd, Beijing 100083, Haidian Distric, Peoples R China."
"   [Masakorala, K.] Univ Ruhuna, Fac Sci, Matara 81000, Sri Lanka."
"   [Cao, Ying] Zhengzhou Univ, Sch Ecol & Environm, 100 Kexue Ave, Zhengzhou 450001, Henan, Peoples R China."
C3 China University of Geosciences; China University of Geosciences;
   University Ruhuna; Zhengzhou University
"RP Yao, J (corresponding author), China Univ Geosci Beijing, Res Ctr Environm Sci & Engn, Sch Water Resources & Environm, 29 Xueyuan Rd, Beijing 100083, Haidian Distric, Peoples R China."
EM yaojun@cugb.edu.cn
"RI Liu, Jianli/ITW-1730-2023"
FU National Science Foundation of China [42230716]; Major National R & D
   Projects for the Chinese Ministry of Science and Technology
"   [2023YFC3707600, 2023YFC3207300]; The 111 Project [B21017]"
FX This work has been supported partly by grants received from the project
"   of the National Science Foundation of China (42230716) , the Major"
   National R & D Projects for the Chinese Ministry of Science and
"   Technology (2023YFC3707600 and 2023YFC3207300) , and the 111 Project"
   (B21017) .
"CR Abd Elnabi MK, 2023, TOXICS, V11, DOI 10.3390/toxics11070580"
"   Achal V, 2012, J MICROBIOL BIOTECHN, V22, P244, DOI 10.4014/jmb.1108.08033"
"   Ahmad WHW, 2015, INT BIODETER BIODEGR, V102, P3, DOI 10.1016/j.ibiod.2015.03.007"
"   Alessandrello MJ, 2017, MAR POLLUT BULL, V122, P156, DOI 10.1016/j.marpolbul.2017.06.040"
"   Ali I, 2019, ENVIRON INT, V127, P160, DOI 10.1016/j.envint.2019.03.029"
"   Ali M, 2015, WATER RES, V79, P147, DOI 10.1016/j.watres.2015.04.024"
"   An Q, 2022, SCI TOTAL ENVIRON, V814, DOI 10.1016/j.scitotenv.2021.152635"
"   Angelim AL, 2013, J ENVIRON MANAGE, V127, P10, DOI 10.1016/j.jenvman.2013.04.014"
"   Ashjari M, 2020, CHEM COMMUN, V56, P9683, DOI 10.1039/d0cc03429k"
"   Bayat Zeynab, 2015, Open Microbiol J, V9, P48, DOI 10.2174/1874285801509010048"
"   Berillo D, 2021, POLYMERS-BASEL, V13, DOI 10.3390/polym13071073"
"   Bilal M, 2017, SCI TOTAL ENVIRON, V576, P646, DOI 10.1016/j.scitotenv.2016.10.137"
"   [?????????? ??????????? ??????????????????? Boiko Oksana V.], 2020, [?????????????? ?? ????????????€????, Hygiene and Sanitation, Russian journal, Gigiena i sanitariya], V99, P368, DOI [10.33029/0016-9900-2020-99-4-368-378, 10.47470/0016-9900-2020-99-4-368-378, 10.33029/0016-9900-2020-99-4-368-378]"
"   Bouabidi ZB, 2019, ENVIRON CHEM LETT, V17, P241, DOI 10.1007/s10311-018-0795-7"
"   [????????? Cai Changfeng], 2010, [??????????€???, Technology of Water Treatment], V36, P74"
"   Cai Q, 2023, SCI TOTAL ENVIRON, V874, DOI 10.1016/j.scitotenv.2023.162594"
"   Ch??varri M, 2010, INT J FOOD MICROBIOL, V142, P185, DOI 10.1016/j.ijfoodmicro.2010.06.022"
"   Chen GH, 2015, RSC ADV, V5, P25372, DOI 10.1039/c4ra14451a"
"   [????????? Chen Junli], 2021, [??????????€???, Technology of Water Treatment], V47, P65"
"   Chen L, 2022, SCI TOTAL ENVIRON, V835, DOI 10.1016/j.scitotenv.2022.155441"

"   Chen MJ, 2021, J HAZARD MATER, V411, DOI 10.1016/j.jhazmat.2021.125103"
"   Chen Suhua, 2002, Yingyong Shengtai Xuebao, V13, P239"
"   Chen XY, 2019, J HAZARD MATER, V368, P705, DOI 10.1016/j.jhazmat.2019.01.108"
"   Chen YY, 2021, FRONT MICROBIOL, V12, DOI 10.3389/fmicb.2021.641913"
"   Chen Z, 2016, J HAZARD MATER, V301, P531, DOI 10.1016/j.jhazmat.2015.09.023"
"   Coelho E, 2022, BIOMETALS, V35, P335, DOI 10.1007/s10534-022-00374-9"
"   Costa S.A., 2005, Enzyme immobilization in biodegradable polymers for biomedical applications"
"   Cuaxinque-Flores G, 2020, SCI TOTAL ENVIRON, V724, DOI 10.1016/j.scitotenv.2020.138124"
"   Cui JW, 2014, ADV COLLOID INTERFAC, V207, P14, DOI 10.1016/j.cis.2013.10.012"
"   Date Y, 2008, WATER SCI TECHNOL, V58, P1121, DOI 10.2166/wst.2008.466"
"   de Matos LP, 2018, CHEM ENG J, V334, P1630, DOI 10.1016/j.cej.2017.11.035"
"   Di J., 2020, Part. J. Water Process. Eng., V31, P43"
"   Di J., 2020, Part. J. Water Process. Eng., V31, P29"
"   Di JZ, 2022, SCI REP-UK, V12, DOI 10.1038/s41598-022-12897-9"
"   Diao CY, 2023, J WATER PROCESS ENG, V52, DOI 10.1016/j.jwpe.2023.103537"
"   Dong HH, 2017, BIORESOURCE TECHNOL, V239, P472, DOI 10.1016/j.biortech.2017.05.015"
"   Duarte-Nass C, 2020, J ENVIRON MANAGE, V256, DOI 10.1016/j.jenvman.2019.109938"
"   El-Naas Muftah H, 2017, Environ Sci Pollut Res Int, V24, P7511, DOI 10.1007/s11356-017-8469-8"
"   Erkaya IA, 2014, J RADIOANAL NUCL CH, V299, P1993, DOI 10.1007/s10967-014-2964-x"
"   Fierro S, 2008, BIORESOURCE TECHNOL, V99, P1274, DOI 10.1016/j.biortech.2007.02.043"
"   Gan YL, 2019, BIORESOURCE TECHNOL, V281, P351, DOI 10.1016/j.biortech.2019.02.111"
"   Gao QT, 2011, BIORESOURCE TECHNOL, V102, P10230, DOI 10.1016/j.biortech.2011.08.070"
"   Garcia ACFS, 2019, APPL BIOCHEM BIOTECH, V188, P1168, DOI 10.1007/s12010-019-02980-9"
"   Garc??a-Delgado C, 2015, J HAZARD MATER, V300, P281, DOI 10.1016/j.jhazmat.2015.07.008"
"   Garmroodi M, 2016, INT J BIOL MACROMOL, V86, P208, DOI 10.1016/j.ijbiomac.2016.01.076"
"   George SE, 2023, J HAZARD MATER, V457, DOI 10.1016/j.jhazmat.2023.131738"
"   [????????? Gong Chunjuan], 2018, [????€??????????, Chinese Journal of Ecology], V37, P3713"
"   Gong YZ, 2022, ENVIRON POLLUT, V314, DOI 10.1016/j.envpol.2022.120232"
"   Gonz??lez AG, 2010, J COLLOID INTERF SCI, V350, P305, DOI 10.1016/j.jcis.2010.06.020"
"   [????????? Guan Qingkun], 2017, [??????????????????, Chinese Journal of Environmental Engineering], V11, P1375"
"   Guo XY, 2022, RSC ADV, V12, P4595, DOI 10.1039/d1ra08709f"
"   Han LJ, 2023, SCI TOTAL ENVIRON, V868, DOI 10.1016/j.scitotenv.2023.161628"
"   Han LJ, 2020, SCI TOTAL ENVIRON, V746, DOI 10.1016/j.scitotenv.2020.140967"
"   Hao TW, 2014, WATER RES, V65, P1, DOI 10.1016/j.watres.2014.06.043"
"   Hashem AM, 2016, INT J BIOL MACROMOL, V82, P905, DOI 10.1016/j.ijbiomac.2015.09.076"
"   He N, 2022, J HAZARD MATER, V422, DOI 10.1016/j.jhazmat.2021.126902"
"   Hou SY, 2022, CHEMOSPHERE, V293, DOI 10.1016/j.chemosphere.2022.133582"
"   Hu SB, 2020, RSC ADV, V10, P1142, DOI 10.1039/c9ra07302g"
"   Huang DQ, 2022, SCI TOTAL ENVIRON, V803, DOI 10.1016/j.scitotenv.2021.150009"
"   Huang F, 2020, J CLEAN PROD, V272, DOI 10.1016/j.jclepro.2020.122743"
"   Huang J, 2020, SPECTROSC LETT, V53, P712, DOI 10.1080/00387010.2020.1824196"
"   Huang ZZ, 2015, J HAZARD MATER, V289, P174, DOI 10.1016/j.jhazmat.2015.02.043"
"   Isaka K, 2007, BIOCHEM ENG J, V37, P49, DOI 10.1016/j.bej.2007.03.008"
"   Jeong SW, 2020, MOLECULES, V25, DOI 10.3390/molecules25214916"
"   Jesionowski T, 2014, ADSORPTION, V20, P801, DOI 10.1007/s10450-014-9623-y"
"   Ji XW, 2022, SCI TOTAL ENVIRON, V811, DOI 10.1016/j.scitotenv.2021.152112"
"   Jiang C., 2023, Environ. Sci. Pollut. Res., V30, P8955"
"   Jun LY, 2019, J ENVIRON CHEM ENG, V7, DOI 10.1016/j.jece.2019.102961"
"   Kilonzo P, 2011, BIORESOURCE TECHNOL, V102, P3662, DOI 10.1016/j.biortech.2010.11.055"
"   Kim Y, 2021, FRONT MICROBIOL, V12, DOI 10.3389/fmicb.2021.646748"
"   Kiran MG, 2017, J HAZARD MATER, V324, P62, DOI 10.1016/j.jhazmat.2015.12.042"
"   Kraynak CA, 2020, ACTA BIOMATER, V108, P250, DOI 10.1016/j.actbio.2020.03.041"
"   Kumar AG, 2019, MAR POLLUT BULL, V146, P741, DOI 10.1016/j.marpolbul.2019.07.006"
"   Lao Chang-ling, 2020, Research of Environmental Sciences, V33, P1929, DOI 10.13198/j.issn.1001-6929.2019.12.06"
"   Li F, 2018, J HAZARD MATER, V358, P178, DOI 10.1016/j.jhazmat.2018.06.066"
"   Li GR, 2020, ENVIRON POLLUT, V265, DOI 10.1016/j.envpol.2020.114887"
"   Li L, 2017, WATER RES, V111, P274, DOI 10.1016/j.watres.2017.01.012"
"   [?????? Li Meng], 2016, [????????????, Environment Engineering], V34, P20"
"   Li M, 2013, INT BIODETER BIODEGR, V76, P81, DOI 10.1016/j.ibiod.2012.06.016"
"   Li MM, 2022, ENVIRON POLLUT, V313, DOI 10.1016/j.envpol.2022.120042"
"   Li SQ, 2021, FOOD CHEM, V339, DOI 10.1016/j.foodchem.2020.128016"
"   Li WL, 2022, SCI TOTAL ENVIRON, V802, DOI 10.1016/j.scitotenv.2021.149802"
"   Li X, 2022, ENVIRON TECHNOL, V43, P1068, DOI 10.1080/09593330.2020.1815858"
"   [????????? Li Xiangyu], 2017, [??????????????????, Chinese Journal of Environmental Engineering], V11, P2812"
"   Li XH, 2023, J HAZARD MATER, V441, DOI 10.1016/j.jhazmat.2022.129862"
"   Li X, 2016, CHEM ENG J, V306, P393, DOI 10.1016/j.cej.2016.07.079"
"   Lin H, 2023, INT BIODETER BIODEGR, V178, DOI 10.1016/j.ibiod.2022.105544"
"   Lin JJ, 2015, BIOCHEM ENG J, V100, P76, DOI 10.1016/j.bej.2015.04.014"
"   Liu CC, 2020, BIORESOURCE TECHNOL, V302, DOI 10.1016/j.biortech.2020.122864"
"   Liu J, 2017, INT BIODETER BIODEGR, V120, P15, DOI [10.1016/j.ibiod.2017.01.039, 10.1145/3145749.3149432]"
"   Liu SH, 2019, SCI TOTAL ENVIRON, V655, P1279, DOI 10.1016/j.scitotenv.2018.11.272"
"   Liu YG, 2019, CHEMOSPHERE, V214, P220, DOI 10.1016/j.chemosphere.2018.09.119"
"   Luo XH, 2023, J ENVIRON SCI, V125, P662, DOI 10.1016/j.jes.2022.01.029"
"   Manonmani U, 2018, J CHEM TECHNOL BIOT, V93, P2486, DOI 10.1002/jctb.5632"
"   Martins S. C. S., 2013, African Journal of Biotechnology, V12, P4412"
"   [?????? Meng Chen], 2019, [??????????????????, Chinese Journal of Environmental Engineering], V13, P1995"
"   Mohite BV, 2018, APPL BIOCHEM BIOTECH, V186, P199, DOI 10.1007/s12010-018-2727-1"
"   Moreau JW, 2013, FRONT MICROBIOL, V4, DOI 10.3389/fmicb.2013.00043"
"   Muyzer G, 2008, NAT REV MICROBIOL, V6, P441, DOI 10.1038/nrmicro1892"
"   Nogueira EW, 2021, BIORESOURCE TECHNOL, V330, DOI 10.1016/j.biortech.2021.124968"
"   Onta??on OM, 2017, NEW BIOTECHNOL, V37, P172, DOI 10.1016/j.nbt.2017.02.003"

"   Parsek MR, 2005, TRENDS MICROBIOL, V13, P27, DOI 10.1016/j.tim.2004.11.007"
"   Pereira PP, 2021, APPL MICROBIOL BIOT, V105, P827, DOI 10.1007/s00253-020-11031-x"
"   Qian Lin-bo, 2012, Huanjing Kexue, V33, P1767"
"   Qiang A., 2021, Sci. Total Environ., V25"
"   Qin S., 2014, Water Sci. Technol., V40, P6"
"   Rahman KSM, 2003, BIORESOURCE TECHNOL, V90, P159, DOI 10.1016/S0960-8524(03)00114-7"
"   Ramakrishnan B, 2011, REV ENVIRON CONTAM T, V211, P63, DOI 10.1007/978-1-4419-8011-3_3"
"   Rathore S, 2013, J FOOD ENG, V116, P369, DOI 10.1016/j.jfoodeng.2012.12.022"
"   Saha P, 2021, ENVIRON REV, V29, P277, DOI 10.1139/er-2020-0074"
"   da Silva IGS, 2020, ENERGIES, V13, DOI 10.3390/en13184664"
"   Sarker A, 2023, CHEMOSPHERE, V332, DOI 10.1016/j.chemosphere.2023.138861"
"   Shan B, 2021, ENVIRON SCI POLLUT R, V28, P30486, DOI 10.1007/s11356-021-14045-8"
"   Shi KX, 2020, CHEMOSPHERE, V259, DOI 10.1016/j.chemosphere.2020.127428"
"   [????????? Shi Zheming], 2022, [???????????, Modern Chemical Industry], V42, P24"
"   Si SC, 2023, SCI TOTAL ENVIRON, V892, DOI 10.1016/j.scitotenv.2023.164556"
"   Sun T, 2020, J HAZARD MATER, V398, DOI 10.1016/j.jhazmat.2020.122941"
"   Tao Y, 2019, SCI TOTAL ENVIRON, V682, P59, DOI 10.1016/j.scitotenv.2019.05.134"
"   Teng ZD, 2021, J HAZARD MATER, V419, DOI 10.1016/j.jhazmat.2021.126433"
"   Teng ZD, 2020, J HAZARD MATER, V384, DOI 10.1016/j.jhazmat.2019.121505"
"   Tufail MA, 2022, SCI TOTAL ENVIRON, V850, DOI 10.1016/j.scitotenv.2022.157961"
"   Vacca DJ, 2005, APPL ENVIRON MICROB, V71, P3797, DOI 10.1128/AEM.71.7.3797-3805.2005"
"   Valdivia-Rivera S, 2021, REV ENVIRON SCI BIO, V20, P815, DOI 10.1007/s11157-021-09577-x"
"   Wang CH, 2019, ENVIRON TECHNOL, V40, P2345, DOI 10.1080/09593330.2018.1441328"
"   Wang CY, 2022, SUSTAINABILITY-BASEL, V14, DOI 10.3390/su141912164"
"   Wang F, 2013, CHEMOSPHERE, V92, P1403, DOI 10.1016/j.chemosphere.2013.03.043"
"   Wang GT, 2022, J HAZARD MATER, V437, DOI 10.1016/j.jhazmat.2022.129402"
"   Wang J, 2009, WORLD J MICROB BIOT, V25, P875, DOI 10.1007/s11274-009-9962-0"
"   Wang JX, 2020, BIORESOURCE TECHNOL, V309, DOI 10.1016/j.biortech.2020.123448"
"   Wang L, 2021, SCI TOTAL ENVIRON, V750, DOI 10.1016/j.scitotenv.2020.141672"
"   Wang ML, 2019, J HAZARD MATER, V366, P684, DOI 10.1016/j.jhazmat.2018.12.049"
"   Wang SX, 2012, J ENVIRON SCI, V24, P1662, DOI 10.1016/S1001-0742(11)60963-7"
"   Wang S, 2023, SUSTAINABILITY-BASEL, V15, DOI 10.3390/su15097622"
"   [?????? Wang Shuo], 2020, [?????????????????, China Environmental Science], V40, P640"
"   Wang WD, 2015, J ENVIRON SCI, V31, P68, DOI 10.1016/j.jes.2014.11.005"
"   Wang WeiQi Wang WeiQi, 2018, China Environmental Science, V38, P3343"
"   Wang X, 2015, MAR POLLUT BULL, V92, P195, DOI 10.1016/j.marpolbul.2014.12.033"
"   Wang Y, 2021, SCI TOTAL ENVIRON, V774, DOI 10.1016/j.scitotenv.2021.145136"
"   Wen Y, 2021, J ENVIRON MANAGE, V297, DOI 10.1016/j.jenvman.2021.113415"
"   Wu B, 2019, SCI TOTAL ENVIRON, V686, P719, DOI 10.1016/j.scitotenv.2019.06.041"
"   Wu MH, 2022, ECOTOX ENVIRON SAFE, V243, DOI 10.1016/j.ecoenv.2022.113956"
"   Wu MH, 2019, J HAZARD MATER, V368, P412, DOI 10.1016/j.jhazmat.2019.01.059"
"   Wu Z, 2022, J CLEAN PROD, V366, DOI 10.1016/j.jclepro.2022.132823"
"   Wu ZG, 2012, WORLD J MICROB BIOT, V28, P2679, DOI 10.1007/s11274-012-1078-2"
"   Xu FL, 2023, GEOMICROBIOL J, V40, P503, DOI 10.1080/01490451.2023.2208113"
"   Xu HC, 2022, J ENVIRON CHEM ENG, V10, DOI 10.1016/j.jece.2022.108531"
"   Xu PP, 2020, ALGAL RES, V47, DOI 10.1016/j.algal.2020.101831"
"   Yadav P, 2021, J WATER PROCESS ENG, V41, DOI 10.1016/j.jwpe.2021.102059"
"   Yan J, 2018, BIORESOURCE TECHNOL, V266, P447, DOI 10.1016/j.biortech.2018.07.012"
"   Yang L, 2022, BIORESOURCE TECHNOL, V356, DOI 10.1016/j.biortech.2022.127287"
"   Yang L, 2017, SCI REP-UK, V7, DOI 10.1038/s41598-017-06338-1"
"   Yang MY, 2020, APPL SOIL ECOL, V150, DOI 10.1016/j.apsoil.2019.103472"
"   Yin TT, 2021, J HAZARD MATER, V401, DOI 10.1016/j.jhazmat.2020.123269"
"   Yu GL, 2019, BIORESOURCE TECHNOL, V280, P337, DOI 10.1016/j.biortech.2019.02.043"
"   Yu XN, 2021, CONSTR BUILD MATER, V273, DOI 10.1016/j.conbuildmat.2020.121113"
"   Yu XN, 2019, J ENVIRON MANAGE, V245, P447, DOI 10.1016/j.jenvman.2019.05.091"
"   Zdarta J, 2018, ADV COLLOID INTERFAC, V258, P1, DOI 10.1016/j.cis.2018.07.004"
"   Zeng GQ, 2021, J HAZARD MATER, V412, DOI 10.1016/j.jhazmat.2021.125156"
"   Zerva I, 2021, BIORESOURCE TECHNOL, V325, DOI 10.1016/j.biortech.2021.124694"
"   Zhan FC, 2021, FOOD CHEM, V337, DOI 10.1016/j.foodchem.2020.127763"
"   Zhang C, 2021, J HAZARD MATER, V403, DOI 10.1016/j.jhazmat.2020.123888"
"   Zhang C, 2020, J HAZARD MATER, V398, DOI 10.1016/j.jhazmat.2020.122806"
"   Zhang DY, 2021, BIORESOURCE TECHNOL, V326, DOI 10.1016/j.biortech.2021.124794"
"   Zhang GX, 2020, GEODERMA, V375, DOI 10.1016/j.geoderma.2020.114497"
"   Zhang J, 2019, APPL GEOCHEM, V103, P68, DOI 10.1016/j.apgeochem.2019.02.011"
"   Zhang KY, 2020, J HAZARD MATER, V397, DOI 10.1016/j.jhazmat.2020.122754"
"   Zhang LF, 2023, J HAZARD MATER, V446, DOI 10.1016/j.jhazmat.2022.130704"
"   Zhang Shilun, 2022, Weishengwu Xuebao, V62, P2289, DOI 10.13343/j.cnki.wsxb.20210493"
"   Zhang TR, 2023, SCI TOTAL ENVIRON, V877, DOI 10.1016/j.scitotenv.2023.162812"
"   Zhang WC, 2023, FRONT MICROBIOL, V14, DOI 10.3389/fmicb.2023.1116970"
"   Zhang XF, 2016, AER ADV ENG RES, V49, P103"
"   Zhang XQ, 2017, POL J ENVIRON STUD, V26, P413, DOI 10.15244/pjoes/64908"
"   Zhao CR, 2023, J CLEAN PROD, V392, DOI 10.1016/j.jclepro.2023.136332"
"   Zheng Meilin, 2021, Sheng Wu Gong Cheng Xue Bao, V37, P3535, DOI 10.13345/j.cjb.210389"
"   Zheng Yu, 2016, Weishengwu Xuebao, V56, P1504, DOI 10.13343/j.cnici.wsxb.20150595"
"   Zheng YT, 2021, WORLD J MICROB BIOT, V37, DOI 10.1007/s11274-021-03176-2"
"   [?????? Zhong Juan], 2021, [??€?????????, Chinese Journal of Rare Metals], V45, P93"
"   Zhu TT, 2016, FRONT BIOENG BIOTECH, V4, DOI 10.3389/fbioe.2016.00004"
"   Zhu XL, 2019, GEODERMA, V350, P1, DOI 10.1016/j.geoderma.2019.04.015"
"   Zou B, 2020, COLLOID SURFACE B, V195, DOI 10.1016/j.colsurfb.2020.111275"
"   Ztab D., 2021, J. Hazard. Mater."
"   Z????iga-Barra H, 2022, MINER ENG, V189, DOI 10.1016/j.mineng.2022.107855"
NR 182
TC 27
Z9 28

U1 39
U2 156
PU ELSEVIER SCI LTD
PI London
"PA 125 London Wall, London, the United Kingdom"
SN 2213-2929
EI 2213-3437
J9 J ENVIRON CHEM ENG
JI J. Environ. Chem. Eng.
PD OCT
PY 2024
VL 12
IS 5
AR 114086
DI 10.1016/j.jece.2024.114086
EA SEP 2024
PG 15
"WC Engineering, Environmental; Engineering, Chemical"
WE Science Citation Index Expanded (SCI-EXPANDED)
SC Engineering
GA H3N8M
UT WOS:001322548200001
DA 2026-04-06
ER

PT J
"AU Wei, T"
"   Gao, H"
"   An, FQ"
"   Ma, XL"
"   Hua, L"
"   Guo, JK"
"AF Wei, Ting"
"   Gao, Han"
"   An, Fengqiu"
"   Ma, Xiulian"
"   Hua, Li"
"   Guo, Junkang"
TI Performance of heavy metal-immobilizing bacteria combined with biochar
   on remediation of cadmium and lead co-contaminated soil
SO ENVIRONMENTAL GEOCHEMISTRY AND HEALTH
LA English
DT Article
DE Heavy metal; Biochar; Heavy metal-immobilizing bacteria; Remediation;
   Heavy metal accumulation
ID GROWTH-PROMOTING BACTERIA; CHEMICAL-PROPERTIES; COMMUNITY STRUCTURE;
   ENZYME-ACTIVITIES; CD ACCUMULATION; SALICYLIC-ACID; VIGNA-RADIATA;
   LACTIC-ACID; CELL-WALL; TOXICITY
"AB Heavy metal pollution of soil has become a public concern worldwide since it threats food safety and human health. Sustainable and environmental-friendly remediation technology is urgently needed. Therefore, we investigated the properties and heavy metal removal ability of Enterobacter asburiae G3 (G3), Enterobacter tabaci I12 (I12), and explored the feasibility of remediation Cd, Pb co-contaminated soil by the combination of G3/I12 and biochar. Our results indicated that both strains are highly resistant to Cd, Pb and maintain plant growth-promoting properties. The removal efficiency of G3 for Cd and Pb were 76.79-99.43%, respectively, while the removal efficiency of I12 for Cd and Pb were 62.57-99.55%, respectively. SEM-EDS and XRD analysis revealed that the morphological and structural changes occurred upon heavy metal exposure, metal precipitates were also detected on cell surface. FTIR analysis indicated that functional groups (-OH, -N-H, -C = O, -C-N, -PO4) were involved in Cd/Pb immobilization. Application of the bacteria, biochar, or their combination decreased the acid-extractable Cd, Pb in soil while increased the residual fractions, meanwhile, the bioavailability of both metal elements declined. Besides, these treatments increased soil enzyme (sucrase, catalase and urease) activity and accelerated pakchoi growth, heavy metal accumulation in pakchoi was depressed upon bacteria and/or biochar application, and a synergistic effect was detected when applying bacteria and biochar together. In BC + G3 and BC + I12 treated plants, the Cd and Pb accumulation decreased by 24.42% and 52.19%, 17.55% and 47.36%, respectively. Overall, our study provides an eco-friendly and promising in situ technology that could be applied in heavy metal remediation."
"C1 [Wei, Ting; Gao, Han; Ma, Xiulian; Hua, Li; Guo, Junkang] Shaanxi Univ Sci & Technol, Sch Environm Sci & Engn, Xian 710021, Peoples R China."
"   [An, Fengqiu] Xian Polytech Univ, Coll Environm & Chem Engn, Xian 710048, Shaanxi, Peoples R China."
C3 Shaanxi University of Science & Technology; Xi'an Polytechnic University
"RP Guo, JK (corresponding author), Shaanxi Univ Sci & Technol, Sch Environm Sci & Engn, Xian 710021, Peoples R China."
EM guojunkang029@163.com
FU National Natural Science Foundation of China [41807123]; Natural Science
"   Foundation of Shaanxi Province [2023-JC-YB-265, 2022NY-078]"
FX AcknowledgementsThis research was financially supported by by the
"   National Natural Science Foundation of China (41807123), Natural Science"
"   Foundation of Shaanxi Province (2023-JC-YB-265, 2022NY-078)."
"CR Abebe Nigussie Abebe Nigussie, 2012, American-Eurasian Journal of Agricultural & Environmental Sciences, V12, P369"
"   Ahmad MT, 2015, AGRON J, V107, P2327, DOI 10.2134/agronj15.0212"
"   Andrey G, 2019, APPL GEOCHEM, V104, P93, DOI 10.1016/j.apgeochem.2019.03.017"
"   Awitdrus Awitdrus, 2021, Advanced Materials Research, V1162, P57, DOI [10.4028/www.scientific.net/amr.1162.57, 10.4028/www.scientific.net/AMR.1162.57]"
"   Babu SV, 2013, J SCI IND RES INDIA, V72, P581"

"   BEVERIDGE TJ, 1980, J BACTERIOL, V141, P876, DOI 10.1128/JB.141.2.876-887.1980"
"   Bhattacharjya S, 2021, ECOTOXICOLOGY, V30, P719, DOI 10.1007/s10646-021-02398-2"
"   Burns RG, 2013, SOIL BIOL BIOCHEM, V58, P216, DOI 10.1016/j.soilbio.2012.11.009"
"   Chakravarty R, 2012, BIORESOURCE TECHNOL, V108, P176, DOI 10.1016/j.biortech.2011.12.100"
"   Chen XT, 2020, SCI REP-UK, V10, DOI [10.1038/s41598-020-61776-8, 10.1038/s41598-020-65631-8]"
"   Chen Yi-Chao, 2022, Acta Prataculturae Sinica, V31, P50, DOI 10.11686/cyxb2021233"
"   Coelho LM, 2015, ADVANCES IN BIOREMEDIATION OF WASTEWATER AND POLLUTED SOIL, P1, DOI 10.5772/60770"
"   de Souza R, 2015, GENET MOL BIOL, V38, P401, DOI 10.1590/S1415-475738420150053"
"   Duan YM, 2021, J HAZARD MATER, V420, DOI 10.1016/j.jhazmat.2021.126635"
"   Durak A, 2010, ASIAN J CHEM, V22, P6375"
"   El-Meihy RM, 2019, ENVIRON EXP BOT, V162, P295, DOI 10.1016/j.envexpbot.2019.03.005"
"   Fang HHP, 2002, WATER RES, V36, P4709, DOI 10.1016/S0043-1354(02)00207-5"
"   Fayaz AM, 2011, PROCESS BIOCHEM, V46, P1958, DOI 10.1016/j.procbio.2011.07.003"
"   Feng Hui-lin, 2021, Huanjing Kexue, V42, P422, DOI [10.13227/j.hjkx.202005285, 10.13227/j.hjkx.202005285]"
"   Gao Y, 2010, J ENVIRON SCI-CHINA, V22, P1040, DOI 10.1016/S1001-0742(09)60215-1"
"   Glaser B, 2002, BIOL FERT SOILS, V35, P219, DOI 10.1007/s00374-002-0466-4"
"   Guo JK, 2018, ECOTOX ENVIRON SAFE, V157, P491, DOI 10.1016/j.ecoenv.2018.04.010"
"   Han H, 2020, ENVIRON POLLUT, V267, DOI 10.1016/j.envpol.2020.115432"
"   Hua L, 2021, CHEMOSPHERE, V263, DOI 10.1016/j.chemosphere.2020.127795"
"   Hua L, 2019, J SOIL SEDIMENT, V19, P2836, DOI 10.1007/s11368-019-02251-4"
"   [????????? Huang Huaqian], 2013, [??????????????????, Southwest China Journal of Agricultural Sciences], V26, P2412"
"   Huo Y, 2021, J GINSENG RES, V45, P218"
"   Jiang Y, 2022, ENVIRON POLLUT, V295, DOI 10.1016/j.envpol.2021.118590"
"   Jin K, 2009, SOIL TILL RES, V104, P115, DOI 10.1016/j.still.2009.02.004"
"   Jin Y, 2017, 3 BIOTECH, V7, DOI 10.1007/s13205-017-0721-x"
"   Johnson DB, 2009, ADV MAT RES, V71-73, P693, DOI 10.4028/www.scientific.net/AMR.71-73.693"
"   Kong ZY, 2017, ADV MICROB PHYSIOL, V71, P97, DOI 10.1016/bs.ampbs.2017.04.001"
"   Kowalenko CG, 2009, COMMUN SOIL SCI PLAN, V40, P3139, DOI 10.1080/00103620903261668"
"   Kumi Andy G., 2020, Materials Science Forum, V1008, P202, DOI [10.4028/www.scientific.net/msf.1008.202, 10.4028/www.scientific.net/MSF.1008.202]"
"   Lao Chang-ling, 2020, Research of Environmental Sciences, V33, P1929, DOI 10.13198/j.issn.1001-6929.2019.12.06"
"   Li HongDa Li HongDa, 2018, Journal of Agro-Environment Science, V37, P1856"
"   Liu SJ, 2018, CHEMOSPHERE, V208, P655, DOI 10.1016/j.chemosphere.2018.06.023"
"   Ma H, 2020, J HAZARD MATER, V388, DOI 10.1016/j.jhazmat.2020.122065"
"   [????????? Mao Xiaojie], 2017, [????????€????€????, Biotechnology Bulletin], V33, P148, DOI 10.13560/j.cnki.biotech.bull.1985.2017-0678"
"   Mckone TE, 2007, ENVIRON TOXICOL CHEM, V26, P2494, DOI 10.1897/06-269.1"
"   Mishra Geetesh Kumar, 2017, Recent Pat Biotechnol, V11, P188, DOI [10.2174/1872208311666170120121025, 10.2174/1872208311666170120121025]"
"   Mishra P, 2021, MICROBIOL RES, V252, DOI 10.1016/j.micres.2021.126861"
"   Munir N, 2022, SUSTAINABILITY-BASEL, V14, DOI 10.3390/su14010161"
"   Naik MM, 2011, CURR MICROBIOL, V62, P409, DOI 10.1007/s00284-010-9722-2"
"   Nair A, 2007, WATER AIR SOIL POLL, V180, P199, DOI 10.1007/s11270-006-9263-2"
"   Namgay T, 2010, AUST J SOIL RES, V48, P638, DOI 10.1071/SR10049"
"   Nies DH, 2000, EXTREMOPHILES, V4, P77, DOI 10.1007/s007920050140"
"   Nithya C, 2011, MAR ENVIRON RES, V71, P283, DOI 10.1016/j.marenvres.2011.02.003"
"   Ojuederie OB, 2017, INT J ENV RES PUB HE, V14, DOI 10.3390/ijerph14121504"
"   Oves M, 2019, INT J ENVIRON SCI TE, V16, P6967, DOI 10.1007/s13762-019-02300-y"
"   Pal AK, 2019, SOIL SEDIMENT CONTAM, V28, P601, DOI 10.1080/15320383.2019.1637398"
"   Pan XH, 2011, J NANOSCI NANOTECHNO, V11, P3597, DOI 10.1166/jnn.2011.3720"
"   Qi X, 2021, J HAZARD MATER, V401, DOI 10.1016/j.jhazmat.2020.123823"
"   Ren XH, 2022, ENVIRON SCI POLLUT R, V29, P73856, DOI 10.1007/s11356-022-21000-8"
"   Ren XH, 2018, ENVIRON POLLUT, V242, P1880, DOI 10.1016/j.envpol.2018.07.078"
"   Renu, 2021, BIOREMEDIAT J, V25, P108, DOI 10.1080/10889868.2020.1844133"
"   Seneviratne M, 2017, J ENVIRON MANAGE, V186, P293, DOI 10.1016/j.jenvman.2016.07.024"
"   Shen H, 2016, FRONT MICROBIOL, V7, DOI 10.3389/fmicb.2016.00841"
"   Singh P, 2021, FRONT MICROBIOL, V11, DOI 10.3389/fmicb.2020.600417"
"   Stanciu-Burileanu MM, 2015, ROM BIOTECH LETT, V20, P10107"
"   Tao Q, 2017, J EXP BOT, V68, P739, DOI 10.1093/jxb/erw453"
"   Taraqqi-A-Kamal A, 2021, PLANT SOIL ENVIRON, V67, P183, DOI 10.17221/544/2020-PSE"
"   Teem H, 2008, INT J FOOD MICROBIOL, V125, P170, DOI 10.1016/j.ijfoodmicro.2008.03.041"
"   Thakur S, 2016, ENVIRON MONIT ASSESS, V188, DOI 10.1007/s10661-016-5211-9"
"   Tu C, 2020, ENVIRON INT, V137, DOI 10.1016/j.envint.2020.105576"
"   Ullah I, 2019, ECOTOX ENVIRON SAFE, V174, P197, DOI 10.1016/j.ecoenv.2019.02.074"
"   Venegas A, 2016, GEODERMA, V279, P132, DOI 10.1016/j.geoderma.2016.06.010"
"   Violante A, 2010, J SOIL SCI PLANT NUT, V10, P268, DOI 10.4067/S0718-95162010000100005"
"   Wang XF, 2014, ADV MATER RES-SWITZ, V864-867, P853, DOI 10.4028/www.scientific.net/AMR.864-867.853"
"   Wei T, 2022, J HAZARD MATER, V435, DOI 10.1016/j.jhazmat.2022.129006"
"   Wei T, 2022, ENVIRON SCI POLLUT R, V29, P63886, DOI 10.1007/s11356-022-20344-5"
"   Wei T, 2022, J PLANT GROWTH REGUL, V41, P445, DOI 10.1007/s00344-021-10315-4"
"   Wei T, 2018, J ENVIRON MANAGE, V214, P164, DOI 10.1016/j.jenvman.2018.02.100"
"   Weng ChengWu Weng ChengWu, 2018, Food Research and Development, V39, P194"
"   Wu C, 2019, SCI TOTAL ENVIRON, V647, P1158, DOI 10.1016/j.scitotenv.2018.08.087"
"   Wu P, 2021, J HAZARD MATER, V416, DOI 10.1016/j.jhazmat.2021.125915"
"   Yamato M, 2006, SOIL SCI PLANT NUTR, V52, P489, DOI 10.1111/j.1747-0765.2006.00065.x"
"   [????????? Yang Guodong], 2020, [????????????, Modern Chemical Industry], V40, P50"
"   Yang YH, 2020, EUR FOOD RES TECHNOL, V246, P2055, DOI 10.1007/s00217-020-03555-3"
"   Yao ZT, 2012, PROCEDIA ENVIRON SCI, V16, P722, DOI 10.1016/j.proenv.2012.10.099"
"   Yi YJ, 2017, J MICROBIOL, V55, P296, DOI 10.1007/s12275-017-6642-x"
"   Yu F., 2017, FORESTRY ENV SCI, V33, P107"
"   Yu HY, 2020, ENVIRON SCI EUR, V32, DOI 10.1186/s12302-020-00303-y"

"   Zahir ZA, 2010, PAK J BOT, V42, P1771"
"   Zeng FR, 2011, ENVIRON POLLUT, V159, P84, DOI 10.1016/j.envpol.2010.09.019"
"   Zhang C, 2023, J HAZARD MATER, V442, DOI 10.1016/j.jhazmat.2022.130105"
"   Zhang CY, 2020, ECOTOX ENVIRON SAFE, V190, DOI 10.1016/j.ecoenv.2019.110090"
"   [?????€??? Zhang Xiuxia], 2013, [??????????????????, Chinese Journal of Environmental Engineering], V7, P1156"
"   Zhu XM, 2017, ENVIRON POLLUT, V227, P98, DOI [10.1016/j.envpol.2017.04.032, 10.1016/j.envPol.2017.04.032]"
"   Zhuang XL, 2007, ENVIRON INT, V33, P406, DOI 10.1016/j.envint.2006.12.005"
NR 90
TC 14
Z9 18
U1 19
U2 155
PU SPRINGER
PI DORDRECHT
"PA VAN GODEWIJCKSTRAAT 30, 3311 GZ DORDRECHT, NETHERLANDS"
SN 0269-4042
EI 1573-2983
J9 ENVIRON GEOCHEM HLTH
JI Environ. Geochem. Health
PD AUG
PY 2023
VL 45
IS 8
BP 6009
EP 6026
DI 10.1007/s10653-023-01605-9
EA MAY 2023
PG 18
"WC Engineering, Environmental; Environmental Sciences; Public,"
   Environmental & Occupational Health; Water Resources
WE Science Citation Index Expanded (SCI-EXPANDED)
"SC Engineering; Environmental Sciences & Ecology; Public, Environmental &"
   Occupational Health; Water Resources
GA O3BU6
UT WOS:000991583800001
PM 37204552
DA 2026-04-06
ER

PT J
"AU Wyszkowska, J"
"   Boros, E"
"   Kucharski, J"
"AF Wyszkowska, J."
"   Boros, E."
"   Kucharski, J."
TI Effect of interactions between nickel and other heavy metals on the soil
   microbiological properties
SO PLANT SOIL AND ENVIRONMENT
LA English
DT Article
DE soil microbes; heavy metals; soil
ID MICROORGANISMS; COPPER; AZOTOBACTER; SENSITIVITY; BACTERIA; GROWTH
"AB A pot greenhouse experiment was performed to determine the effect of contamination with nickel interacting with other heavy metals on the microbiological properties of soil. The study was conducted on samples of soils classified under natural conditions as typical Eutric Cambisol developed from heavy loamy sand and typical Eutric Cambisol developed from light silty loam. Soil material was contaminated with nickel in the amount of 50 and 200 mg Ni2+/kg. The treatments with 200 mg Ni2+/kg were additionally contaminated with other heavy metals (Zn2+, Cu2+, Pb2+, Cd2+, Cr6+), in the amount of 50 mg/kg soil. The following treatments, in which the soil was contaminated with heavy metals applied alone or in, combinations, were compared in the study: Ni, Zn, Cu, Pb, Cd, Cr, NiZn, NiCu, NiPb, NiCd, NiCr, NiZnCu, NiZnPb, NiZnCd, NiZnCr, NiZnCuPb, NiZnCuCd, NiZnCuCr, NiZnCuPbCd, NiZnCuPbCr, NiZnCuPbCdCr. The experiment was carried out in four replications. A microbiological analysis was performed on days 28 and 56. The tested crop was oat. It was found that the impact of particular heavy metals on microbiological properties of soils depended on their type, interactions between nickel and zinc, copper, lead, cadmium and chromium (VI), date of analysis and soil species. Soil contamination with heavy metals reduced the population size of Azotobacter spp. The counts of other microbial groups, i.e. copiotrophic bacteria, spore-forming copiotrophic bacteria, oligotrophic bacteria, spore-forming oligotrophic bacteria, ammonifying bacteria, nitrogen immobilizing bacteria, cellulose-decomposing bacteria, Arthrobacter spp., Pseudomonas spp., actinomyces and fungi, showed varied susceptibility to heavy metals."
"C1 [Wyszkowska, J.; Boros, E.; Kucharski, J.] Univ Warmia & Mazury, Olsztyn, Poland."
C3 University of Warmia & Mazury
"RP Wyszkowska, J (corresponding author), Univ Warmia & Mazury, Olsztyn, Poland."
"RI ; Kucharski, Jan/AAW-2978-2020"
"OI Wyszkowska, Jadwiga/0000-0002-2156-3780; Boros-Lajszner,"
"   Edyta/0000-0002-1867-5335; Kucharski, Jan/0000-0002-6364-6357"
"CR Babich H, 1997, APPL ENVIRON MICROB, V33, P681"
"   FENGLERO.W, 1965, ACTA MICROBIOL POL, V14, P203"
"   Giller KE, 1998, SOIL BIOL BIOCHEM, V30, P1389, DOI 10.1016/S0038-0717(97)00270-8"
"   Giridhara M., 2002, Current Research - University of Agricultural Sciences (Bangalore), V31, P4"
"   HATTORI R, 1980, J GEN APPL MICROBIOL, V26, P1, DOI 10.2323/jgam.26.1"
"   Huang Q, 2000, SOIL BIOL BIOCHEM, V32, P1885, DOI 10.1016/S0038-0717(00)00162-0"

"   JASIEWICZ C, 2000, AGRICULTURA, V84, P141"
"   Kabata-Pendias A., 2001, Trace Elements in Soil and Plants, VThird"
"   Kucharski J, 2004, PLANT SOIL ENVIRON, V50, P243, DOI 10.17221/4028-PSE"
"   MARTIN JP, 1950, SOIL SCI, V69, P215, DOI 10.1097/00010694-195003000-00006"
"   MULDER EG, 1963, ANN I PASTEUR PARIS, V105, P46"
"   Pandey N, 2002, PLANT SCI, V163, P753, DOI 10.1016/S0168-9452(02)00210-8"
"   Parkinson D., 1971, IBP HDB"
"   Simon T, 1999, ROST VYROBA, V45, P421"
"   Smejkalov?? M, 2003, PLANT SOIL ENVIRON, V49, P321, DOI 10.17221/4131-PSE"
"   STATSOFT INC., 2003, STAT DAT AN SOFTW SY"
"   Tibazarwa C, 2001, ENVIRON POLLUT, V113, P19, DOI 10.1016/S0269-7491(00)00177-9"
"   Vasundhara G, 2004, B ENVIRON CONTAM TOX, V72, P1122, DOI 10.1007/s00128-004-0360-x"
"   Zaborowska M., 2006, Polish Journal of Environmental Studies, V15, P569"
NR 19
TC 37
Z9 45
U1 1
U2 28
PU CZECH ACADEMY AGRICULTURAL SCIENCES
PI PRAGUE
"PA TESNOV 17, PRAGUE, 117 05, CZECH REPUBLIC"
SN 1214-1178
EI 1805-9368
J9 PLANT SOIL ENVIRON
JI Plant Soil Environ.
PD DEC
PY 2007
VL 53
IS 12
BP 544
EP 552
DI 10.17221/2190-PSE
PG 9
"WC Agriculture, Multidisciplinary; Agronomy"
WE Science Citation Index Expanded (SCI-EXPANDED)
SC Agriculture
GA 286QG
UT WOS:000254860300005
"OA Green Submitted, gold"
DA 2026-04-06
ER

PT J
"AU Han, H"
"   Wu, XJ"
"   Yao, LG"
"   Chen, ZJ"
"AF Han, Hui"
"   Wu, Xuejiao"
"   Yao, Lunguang"
"   Chen, Zhaojin"
TI Heavy metal-immobilizing bacteria combined with calcium polypeptides
   reduced the uptake of Cd in wheat and shifted the rhizosphere bacterial
   communities
SO ENVIRONMENTAL POLLUTION
LA English
DT Article
DE Heavy metal-immobilizing bacteria; Calcium polypeptide; Wheat; Cd;
   Bacterial community structure
ID MICROBIAL COMMUNITIES; CONTAMINATED SOILS; AGRICULTURAL SOILS; PB
   IMMOBILIZATION; RISK-ASSESSMENT; CADMIUM; RICE; ACCUMULATION; TOXICITY;
   BIOCHAR
"AB In situ stabilization techniques for the ""remediation"" of heavy metal-contaminated soil are a novel and inexpensive technology. However, the mechanisms underlying the interaction of exogenous passivators with the bacterial community in wheat rhizosphere soil remain unclear. Soil static culture and pot experiments were conducted to evaluate the effects and mechanisms of the heavy metal-immobilizing bacterium Enterobacter bugandensis TJ6 and calcium polypeptides (CPPs) and their association with Cd uptake in wheat, soil quality and the rhizobacterial community structure. The results showed that compared with the control treatment (CK), the TJ6, CPP, and TJ6+CPP treatments significantly decreased the diethylenetriaminepentaacetic acid (DTPA)-extractable Cd (25.2%-60.1%) content and increased the pH, organic matter content and urease activity in the wheat rhizosphere soil, which resulted in decreases in the Cd (21.5%-77.8%) content in wheat tissues (grain, straw, and roots). In particular, the TJ6-CPP treatment was more effective at decreasing Cd accumulation in grains. Furthermore, the TJ6-CPP treatment improved the diversity of the soil bacterial community in the wheat rhizosphere, and the relative abundances of Proteobacteria, Firmicutes, Arthrobacter, Microvirga, Ensifer, Brevundimonas, Devosia and Pedobacter were enriched. These results suggest that the TJ6-CPP treatment decreased the uptake of Cd in wheat by i) providing essential elements (N and C sources), ii) increasing the pH and reducing the bioavailable Cd content in wheat rhizosphere soil, iii) allowing colonization to promote plant growth and Cd-resistant bacteria, and iv) increasing the abundance of genes associated with ABC transporters, carbon metabolism and oxidative phosphorylation in the rhizosphere bacterial community. Our results showed that the heavy metal-immobilizing bacterium TJ6 combined with CPPs decreased the Cd content and increased the bacterial community diversity of wheat rhizosphere soil. Our results also highlight the potential of using heavy metal-immobilizing bacteria and CPPs to ensure the safe production of crops growing on heavy metal-polluted soils. (C) 2020 Elsevier Ltd. All rights reserved."

"C1 [Han, Hui; Wu, Xuejiao; Yao, Lunguang; Chen, Zhaojin] Nanyang Normal Univ, Coll Agr Engn, Collaborat Innovat Water Secur Water Source Reg M, Nanyang 473061, Peoples R China."
C3 Nanyang Normal University
"RP Chen, ZJ (corresponding author), Nanyang Normal Univ, Coll Agr Engn, Collaborat Innovat Water Secur Water Source Reg M, Nanyang 473061, Peoples R China."
EM zhaojin_chen@163.com
"RI Han, Hui/GYJ-6725-2022"
"OI Han, Hui/0000-0002-5218-0109; ?????????, Zhaojin Chen/0000-0003-1088-6821"
"FU National Natural Science Foundation of China [41907143, 41601332]; Key"
Research Project of Colleges and Universities of Henan Province
Education Department [20A180023]
FX This work was supported by the National Natural Science Foundation of
" China (Grant No. 41907143, 41601332) and the Key Research Project of"
Colleges and Universities of Henan Province Education Department (Grant
No. 20A180023).
"CR Altimira F, 2012, BMC MICROBIOL, V12, DOI 10.1186/1471-2180-12-193"
" [Anonymous], 2011, OFF J EUR COMMUN, VL88/4.4"
" Burkhardt EM, 2011, APPL ENVIRON MICROB, V77, P3132, DOI 10.1128/AEM.02085-10"
" Chen D, 2016, SCI TOTAL ENVIRON, V541, P1489, DOI 10.1016/j.scitotenv.2015.10.052"
" Chen HM, 2019, ENVIRON INT, V127, P395, DOI 10.1016/j.envint.2019.03.068"
" Chen L, 2016, J HAZARD MATER, V312, P123, DOI 10.1016/j.jhazmat.2016.03.042"
" Cheng C, 2020, SCI TOTAL ENVIRON, V740, DOI 10.1016/j.scitotenv.2020.139972"
" Diaconu M, 2020, NEW BIOTECHNOL, V56, P130, DOI 10.1016/j.nbt.2020.01.003"
" EIVAZI F, 1988, SOIL BIOL BIOCHEM, V20, P601, DOI 10.1016/0038-0717(88)90141-1"
" Etesami H, 2018, ECOTOX ENVIRON SAFE, V147, P175, DOI 10.1016/j.ecoenv.2017.08.032"
" Gong XM, 2019, SCI TOTAL ENVIRON, V666, P1126, DOI 10.1016/j.scitotenv.2019.02.215"
" Han H, 2020, ECOTOX ENVIRON SAFE, V195, DOI 10.1016/j.ecoenv.2020.110375"
" Han H, 2018, J HAZARD MATER, V353, P280, DOI 10.1016/j.jhazmat.2018.04.024"
" Hesse E, 2018, ECOL LETT, V21, P117, DOI 10.1111/ele.12878"
" Hidri R, 2019, APPL SOIL ECOL, V143, P161, DOI 10.1016/j.apsoil.2019.06.014"
" Hortal S, 2013, SOIL BIOL BIOCHEM, V64, P139, DOI 10.1016/j.soilbio.2013.04.018"
" Hou DD, 2018, ENVIRON POLLUT, V241, P63, DOI 10.1016/j.envpol.2018.04.121"
" Huang JH, 2017, CHEMOSPHERE, V173, P199, DOI 10.1016/j.chemosphere.2016.12.137"
" Jiang LF, 2017, ENVIRON POLLUT, V231, P173, DOI 10.1016/j.envpol.2017.08.003"
" Ju WL, 2020, APPL SOIL ECOL, V150, DOI 10.1016/j.apsoil.2019.103450"
" Ju WL, 2019, ECOTOX ENVIRON SAFE, V167, P218, DOI 10.1016/j.ecoenv.2018.10.016"
" Kim SW, 2017, J AGR FOOD CHEM, V65, P1239, DOI 10.1021/acs.jafc.6b04339"
" Langille MGI, 2013, NAT BIOTECHNOL, V31, P814, DOI 10.1038/nbt.2676"
" Le??n-Ca??edo JA, 2019, SCI TOTAL ENVIRON, V650, P2535, DOI 10.1016/j.scitotenv.2018.10.003"
" Li RF, 2019, ENVIRON POLLUT, V255, DOI 10.1016/j.envpol.2019.113170"
" Li Y, 2017, ECOTOX ENVIRON SAFE, V138, P56, DOI 10.1016/j.ecoenv.2016.12.024"
" Liu HK, 2020, CHEMOSPHERE, V242, DOI 10.1016/j.chemosphere.2019.125164"
" Liu L, 2020, TRENDS FOOD SCI TECH, V96, P199, DOI 10.1016/j.tifs.2019.12.022"
" Liu L, 2014, J PHARM SCI-US, V103, P1979, DOI 10.1002/jps.24013"
" Lu Q, 2020, ENVIRON POLLUT, V257, DOI 10.1016/j.envpol.2019.113497"
" Mahbub KR, 2017, SCI TOTAL ENVIRON, V576, P303, DOI 10.1016/j.scitotenv.2016.10.083"
" McMahon KD, 2002, APPL ENVIRON MICROB, V68, P4971, DOI 10.1128/AEM.68.10.4971-4978.2002"
" Nicholson FA, 2003, SCI TOTAL ENVIRON, V311, P205, DOI 10.1016/S0048-9697(03)00139-6"
" Nielsen S, 2014, AGR ECOSYST ENVIRON, V191, P73, DOI 10.1016/j.agee.2014.04.006"
" Niemeyer JC, 2012, APPL SOIL ECOL, V59, P96, DOI 10.1016/j.apsoil.2012.03.019"
" Ontiveros-Valencia A, 2014, ENVIRON SCI TECHNOL, V48, P7511, DOI 10.1021/es5012466"
" Pan W, 2019, ECOTOX ENVIRON SAFE, V177, P100, DOI 10.1016/j.ecoenv.2019.04.010"
" Park JH, 2011, SCI TOTAL ENVIRON, V409, P853, DOI 10.1016/j.scitotenv.2010.11.003"
" Rehman MZU, 2018, ENVIRON POLLUT, V241, P557, DOI 10.1016/j.envpol.2018.06.005"
" Ruangdech Thanyaporn, 2017, Agriculture and Natural Resources, V51, P137, DOI [10.1016/j.anres.2016.05.005, 10.1016/j.anres.2016.05.005]"
" Rudnick P, 1997, SOIL BIOL BIOCHEM, V29, P831, DOI 10.1016/S0038-0717(96)00238-6"
" Segata N, 2011, GENOME BIOL, V12, DOI 10.1186/gb-2011-12-6-r60"
" Shahid M, 2017, REV ENVIRON CONTAM T, V241, P73, DOI 10.1007/398_2016_8"
" SSICA (Soil Sci Ch. Acad.), 1980, DISSERTATION"
" Teng ZD, 2019, J ENVIRON MANAGE, V231, P189, DOI 10.1016/j.jenvman.2018.10.012"
" TESSIER A, 1979, ANAL CHEM, V51, P844, DOI 10.1021/ac50043a017"
" Tipayno S, 2012, APPL SOIL ECOL, V61, P137, DOI 10.1016/j.apsoil.2012.06.001"
" Venegas A, 2016, GEODERMA, V279, P132, DOI 10.1016/j.geoderma.2016.06.010"
" Wan MX, 2019, PROCESS SAF ENVIRON, V132, P73, DOI 10.1016/j.psep.2019.09.025"
" Wan WJ, 2020, SCI TOTAL ENVIRON, V700, DOI 10.1016/j.scitotenv.2019.134418"
" Wan YA, 2018, ENVIRON POLLUT, V240, P523, DOI 10.1016/j.envpol.2018.04.044"
" Wang LH, 2020, CHEMOSPHERE, V252, DOI 10.1016/j.chemosphere.2020.126522"
" Wang M, 2019, CHEMOSPHERE, V220, P828, DOI 10.1016/j.chemosphere.2018.12.214"
" Wang TJ, 2020, ENVIRON SCI POLLUT R, V27, P8707, DOI 10.1007/s11356-019-06957-3"
" Wang XH, 2018, ENVIRON POLLUT, V242, P1488, DOI 10.1016/j.envpol.2018.08.039"
" Wang YP, 2007, J ENVIRON SCI, V19, P848, DOI 10.1016/S1001-0742(07)60141-7"
" Xu CR, 2017, CHEMOSPHERE, V173, P622, DOI 10.1016/j.chemosphere.2017.01.005"
" Xu XB, 2019, SCI TOTAL ENVIRON, V684, P537, DOI 10.1016/j.scitotenv.2019.05.015"
" Yang YJ, 2020, SCI TOTAL ENVIRON, V708, DOI 10.1016/j.scitotenv.2019.135186"
" Yu PY, 2020, J HAZARD MATER, V381, DOI 10.1016/j.jhazmat.2019.121203"
" Yu SM, 2019, J SOIL SEDIMENT, V19, P1064, DOI 10.1007/s11368-018-2111-3"
" Zeng FR, 2011, ENVIRON POLLUT, V159, P84, DOI 10.1016/j.envpol.2010.09.019"
NR 62
TC 95
Z9 103
U1 7
U2 198
PU ELSEVIER SCI LTD

PI London
"PA 125 London Wall, London, the United Kingdom"
SN 0269-7491
EI 1873-6424
J9 ENVIRON POLLUT
JI Environ. Pollut.
PD DEC
PY 2020
VL 267
AR 115432
DI 10.1016/j.envpol.2020.115432
PG 13
WC Environmental Sciences
WE Science Citation Index Expanded (SCI-EXPANDED)
SC Environmental Sciences & Ecology
GA RB1XQ
UT WOS:000631909700017
PM 32841909
DA 2026-04-06
ER

PT J
"AU Song, JW"
"   Brookes, PC"
"   Shan, SD"
"   Xu, JM"
"   Liu, XM"
"AF Song, Jiuwei"
"   Brookes, Philip C."
"   Shan, Shengdao"
"   Xu, Jianming"
"   Liu, Xingmei"
TI Effects of remediation agents on microbial community structure and
   function in soil aggregates contaminated with heavy metals
SO GEODERMA
LA English
DT Article
DE Heavy metals; Soil aggregates; Remediation agents; Microbial biomass;
   Microbial community
ID BACTERIAL COMMUNITIES; CADMIUM; MICROORGANISMS; IMMOBILIZATION;
   NITROGEN; REMOVAL; CARBON; CU; STABILIZATION; AVAILABILITY
"AB Heavy metal remediation agents have been widely used in contaminated soils, but the remediation effects on heavy metals in soil aggregates and the effects on the structure and function of the microbial communities in aggregates have rarely been reported. Here, sodium silicate, sepiolite, quicklime and monocalcium phosphate were added to soil contaminated with Cu, Zn and Cd. Rice was grown as a test crop. Rice plants and soil samples were then collected after the rice was harvested. The remediation agents decreased the available heavy metal concentrations of both the soil aggregates and rice plants mainly by increasing the soil pH. The changes in bacterial community structure were mainly related to the contents of available heavy metals, and the addition of remediation agents changed the microbial composition. Sodium silicate and sepiolite increased the microbial biomass content in the soil aggregates, while quicklime decreased both microbial biomass and bacterial alpha-diversity in the soil aggregates. Remediation agents can increase the predicted expression intensity of heavy metal resistance genes and C and N metabolism genes by decreasing the contents of available heavy metals. The influence of heavy metal remediation agents on microbial community structure and function should be considered when selecting heavy metal remediation agents."
"C1 [Song, Jiuwei; Brookes, Philip C.; Xu, Jianming; Liu, Xingmei] Zhejiang Univ, Coll Environm & Resource Sci, Zhejiang Prov Key Lab Agr Resources & Environm, Hangzhou 310058, Peoples R China."
"   [Shan, Shengdao] Zhejiang Univ Sci & Technol, Sch Environm & Nat Resources, Hangzhou 310023, Peoples R China."
C3 Zhejiang University; Zhejiang University of Science & Technology
"RP Liu, XM (corresponding author), Zhejiang Univ, Coll Environm & Resource Sci, Zhejiang Prov Key Lab Agr Resources & Environm, Hangzhou 310058, Peoples R China."
EM xmliu@zju.edu.cn
"RI Brookes, Philip/ABW-6646-2022; Xu, Jianming/C-8752-2016; song,"
   jiuwei/NYT-0175-2025
FU National Key Research Program of China [2020YFC1806700]; National
   Natural Science Foundation of China [41977143]
FX Acknowledgements This work was financially supported by the National Key
   Research Program of China (2020YFC1806700) and the National Natural
   Science Foundation of China (41977143) .
"CR Abiven S, 2009, SOIL BIOL BIOCHEM, V41, P1, DOI 10.1016/j.soilbio.2008.09.015"
"   Pietri JCA, 2009, SOIL BIOL BIOCHEM, V41, P1396, DOI 10.1016/j.soilbio.2009.03.017"
"   Alvani S, 2019, GEODERMA, V350, P19, DOI 10.1016/j.geoderma.2019.05.004"
"   Alvarenga D, 2021, SCI TOTAL ENVIRON, V795, DOI 10.1016/j.scitotenv.2021.148676"
"   Bach EM, 2014, SOIL BIOL BIOCHEM, V69, P54, DOI 10.1016/j.soilbio.2013.10.033"
"   Bailey VL, 2013, ISME J, V7, P2044, DOI 10.1038/ismej.2013.87"
"   Bailey VL, 2012, SOIL BIOL BIOCHEM, V48, P192, DOI 10.1016/j.soilbio.2012.01.004"
"   Beattie RE, 2018, SOIL BIOL BIOCHEM, V126, P57, DOI 10.1016/j.soilbio.2018.08.011"
"   Berg J, 2012, APPL ENVIRON MICROB, V78, P7438, DOI 10.1128/AEM.01071-12"
"   Beulig F, 2015, ISME J, V9, P746, DOI 10.1038/ismej.2014.148"
"   Blumer-Schuette SE, 2014, FEMS MICROBIOL REV, V38, P393, DOI 10.1111/1574-6976.12044"
"   Bolan N, 2014, J HAZARD MATER, V266, P141, DOI 10.1016/j.jhazmat.2013.12.018"
"   Bolan NS, 2003, PLANT SOIL, V251, P187, DOI 10.1023/A:1023037706905"
"   Bolyen E, 2019, NAT BIOTECHNOL, V37, P852, DOI 10.1038/s41587-019-0209-9"
"   Cai T, 2021, J CLEAN PROD, V281, DOI 10.1016/j.jclepro.2020.125390"

"   Callahan BJ, 2016, NAT METHODS, V13, P581, DOI [10.1038/NMETH.3869, 10.1038/nmeth.3869]"
"   Chen D, 2020, J ENVIRON MANAGE, V254, DOI 10.1016/j.jenvman.2019.109788"
"   Chen SF, 2018, BIOINFORMATICS, V34, P884, DOI 10.1093/bioinformatics/bty560"
"   Chodak M, 2013, APPL SOIL ECOL, V64, P7, DOI 10.1016/j.apsoil.2012.11.004"
"   Douglas G.M., 2019, BIORXIV, DOI DOI 10.1101/672295"
"   Fedotov GN, 2008, DOKL CHEM, V420, P125, DOI 10.1134/S0012500808050042"
"   Feng G, 2018, BMC MICROBIOL, V18, DOI 10.1186/s12866-018-1152-5"
"   Fierer N, 2021, SOIL BIOL BIOCHEM, V153, DOI 10.1016/j.soilbio.2020.108111"
"   Finn D, 2020, APPL SOIL ECOL, V148, DOI 10.1016/j.apsoil.2019.103483"
"   FROSTEGARD A, 1993, SOIL BIOL BIOCHEM, V25, P723, DOI 10.1016/0038-0717(93)90113-P"
"   Giller KE, 2009, SOIL BIOL BIOCHEM, V41, P2031, DOI 10.1016/j.soilbio.2009.04.026"
"   Griffiths BS, 2013, FEMS MICROBIOL REV, V37, P112, DOI 10.1111/j.1574-6976.2012.00343.x"
"   Guidi P, 2021, GEODERMA, V397, DOI 10.1016/j.geoderma.2021.115101"
"   Hazra KK, 2019, GEODERMA, V353, P308, DOI 10.1016/j.geoderma.2019.06.039"
"   He HD, 2017, PEDOSPHERE, V27, P569, DOI [10.1016/S1002-0160(17)60352-7, 10.1016/s1002-0160(17)60352-7]"
"   He MJ, 2019, ENVIRON POLLUT, V244, P431, DOI 10.1016/j.envpol.2018.10.070"
"   Huang B, 2019, ENVIRON TECHNOL, V40, P2705, DOI 10.1080/09593330.2018.1449900"
"   Hwang JU, 2016, MOL PLANT, V9, P338, DOI 10.1016/j.molp.2016.02.003"
"   Ilg K, 2004, GEODERMA, V123, P153, DOI 10.1016/j.geoderma.2004.02.006"
"   Jaskulak M, 2020, ECOTOX ENVIRON SAFE, V197, DOI 10.1016/j.ecoenv.2020.110606"
"   Jie SQ, 2016, BMC MICROBIOL, V16, DOI 10.1186/s12866-016-0800-x"
"   Jim??nez-Gonz??lez MA, 2020, GEODERMA, V374, DOI 10.1016/j.geoderma.2020.114443"
"   Kang EZ, 2021, SCI TOTAL ENVIRON, V774, DOI 10.1016/j.scitotenv.2021.145780"
"   Kenarova A, 2014, ECOTOX ENVIRON SAFE, V100, P226, DOI 10.1016/j.ecoenv.2013.11.012"
"   Khan S, 2010, ECOTOX ENVIRON SAFE, V73, P1820, DOI 10.1016/j.ecoenv.2010.08.016"
"   Kieu HTQ, 2011, WATER RES, V45, P3863, DOI 10.1016/j.watres.2011.04.043"
"   Kumpiene J, 2008, WASTE MANAGE, V28, P215, DOI 10.1016/j.wasman.2006.12.012"
"   Li RF, 2019, ENVIRON POLLUT, V255, DOI 10.1016/j.envpol.2019.113170"
"   Li XQ, 2017, ENVIRON POLLUT, V231, P908, DOI 10.1016/j.envpol.2017.08.057"
"   Liu J, 2018, ENVIRON POLLUT, V235, P171, DOI 10.1016/j.envpol.2017.12.008"
"   Ma YJ, 2020, ECOL INDIC, V117, DOI 10.1016/j.ecolind.2020.106626"
"   Ma ZZ, 2021, GEODERMA, V388, DOI 10.1016/j.geoderma.2021.114928"
"   Madsen EL, 2011, CURR OPIN BIOTECH, V22, P456, DOI 10.1016/j.copbio.2011.01.008"
"   Magoc T, 2011, BIOINFORMATICS, V27, P2957, DOI 10.1093/bioinformatics/btr507"
"   M??kil?? M, 2015, GEODERMA, V239, P280, DOI 10.1016/j.geoderma.2014.11.002"
"   Mhh W, 2011, R PACKAGE VERSION 20"
"   Mikhailova EA, 2000, AGR ECOSYST ENVIRON, V80, P213, DOI 10.1016/S0167-8809(00)00148-1"
"   Monreal CM, 2018, RHIZOSPHERE-NETH, V5, P16, DOI 10.1016/j.rhisph.2017.11.004"
"   Morton-Bermea O, 2009, GEODERMA, V151, P121, DOI 10.1016/j.geoderma.2009.03.019"
"   Nawab J, 2016, ENVIRON SCI POLLUT R, V23, P2381, DOI 10.1007/s11356-015-5458-7"
"   Nies DH, 1999, APPL MICROBIOL BIOT, V51, P730, DOI 10.1007/s002530051457"
"   Ren TT, 2020, SOIL BIOL BIOCHEM, V146, DOI 10.1016/j.soilbio.2020.107825"
"   Rillig MC, 2017, ISME J, V11, P1943, DOI 10.1038/ismej.2017.56"
"   Rizwan Muhammad, 2016, Environ Sci Pollut Res Int, V23, P17859, DOI 10.1007/s11356-016-6436-4"
"   Smith MB, 2015, MBIO, V6, DOI 10.1128/mBio.00326-15"
"   Song B, 2017, ENVIRON INT, V105, P43, DOI 10.1016/j.envint.2017.05.001"
"   Song JW, 2021, ENVIRON POLLUT, V268, DOI 10.1016/j.envpol.2020.115933"
"   Song JW, 2018, ENVIRON POLLUT, V243, P510, DOI 10.1016/j.envpol.2018.09.011"
"   Steinnes E, 2006, ENVIRON REV, V14, P169, DOI [10.1139/A06-002, 10.1139/a06-002]"
"   Sun FL, 2021, J ENVIRON MANAGE, V300, DOI 10.1016/j.jenvman.2021.113754"
"   Sun YB, 2012, J ENVIRON SCI, V24, P1799, DOI 10.1016/S1001-0742(11)61010-3"
"   ter Beek J, 2014, J GEN PHYSIOL, V143, P419, DOI 10.1085/jgp.201411164"
"   VANCE ED, 1987, SOIL BIOL BIOCHEM, V19, P703, DOI 10.1016/0038-0717(87)90052-6"
"   Venceslau SS, 2014, BBA-BIOENERGETICS, V1837, P1148, DOI 10.1016/j.bbabio.2014.03.007"
"   Vesterg??rd M, 2018, GCB BIOENERGY, V10, P320, DOI 10.1111/gcbb.12494"
"   Vr??nceanu NO, 2019, CATENA, V176, P336, DOI 10.1016/j.catena.2019.01.015"
"   Wang H, 2017, J HAZARD MATER, V329, P66, DOI 10.1016/j.jhazmat.2017.01.025"
"   Wang XQ, 2021, APPL SOIL ECOL, V166, DOI 10.1016/j.apsoil.2021.104064"
"   Wang YY, 2019, J CLEAN PROD, V231, P556, DOI 10.1016/j.jclepro.2019.04.407"
"   Wyszkowska J, 2013, J ELEMENTOL, V18, P769, DOI 10.5601/jelem.2013.18.4.455"
"   Xu M, 2021, SOIL BIOL BIOCHEM, V154, DOI 10.1016/j.soilbio.2020.108126"
"   Xu Y., 2018, 20 YEARS RES DEV SOI, P399"
"   Xu Y, 2017, PEDOSPHERE, V27, P193, DOI 10.1016/S1002-0160(17)60310-2"
"   Yang YF, 2013, GLOBAL CHANGE BIOL, V19, P637, DOI 10.1111/gcb.12065"
"   Yin HQ, 2015, SCI REP-UK, V5, DOI 10.1038/srep14266"
"   Ying JY, 2017, SOIL BIOL BIOCHEM, V107, P10, DOI 10.1016/j.soilbio.2016.12.023"
"   Yu ZG, 2009, J HAZARD MATER, V167, P38, DOI 10.1016/j.jhazmat.2008.12.082"
"   Zhang MP, 2021, WATER RES, V202, DOI 10.1016/j.watres.2021.117446"
"   Zhang Q, 2017, SOIL BIOL BIOCHEM, V113, P1, DOI 10.1016/j.soilbio.2017.05.022"
"   Zhang Q, 2019, SCI TOTAL ENVIRON, V687, P790, DOI 10.1016/j.scitotenv.2019.06.044"
"   Zhang XP, 2020, J HAZARD MATER, V399, DOI 10.1016/j.jhazmat.2020.123107"
"   Zhou T, 2018, ENVIRON POLLUT, V232, P514, DOI 10.1016/j.envpol.2017.09.081"
"   Zhou ZH, 2020, NAT COMMUN, V11, DOI 10.1038/s41467-020-16881-7"
"   Zhu M, 2022, FOOD RES INT, V153, DOI 10.1016/j.foodres.2022.110955"
NR 89
TC 31
Z9 32
U1 29
U2 366
PU ELSEVIER
PI AMSTERDAM
"PA RADARWEG 29, 1043 NX AMSTERDAM, NETHERLANDS"
SN 0016-7061
EI 1872-6259

J9 GEODERMA
JI Geoderma
PD NOV 1
PY 2022
VL 425
AR 116030
DI 10.1016/j.geoderma.2022.116030
EA JUL 2022
PG 12
WC Soil Science
WE Science Citation Index Expanded (SCI-EXPANDED)
SC Agriculture
GA 4Y6AX
UT WOS:000861609500004
DA 2026-04-06
ER

PT J
"AU Wang, D"
"   Chen, HS"
"   Xin, CY"
"   Yuan, Y"
"   Sun, Q"
"   Cao, CR"
"   Chao, HR"
"   Wu, T"
"   Zheng, SC"
"AF Wang, Di"
"   Chen, Hansong"
"   Xin, Chaoyang"
"   Yuan, Ying"
"   Sun, Qi"
"   Cao, Churong"
"   Chao, Herong"
"   Wu, Ting"
"   Zheng, Shaocheng"
TI Insight into adsorption of Pb(II) with wild resistant bacteria TJ6
   immobilized on biochar composite: Roles of bacterial cell and biochar
SO SEPARATION AND PURIFICATION TECHNOLOGY
LA English
DT Article
DE Biochar; Environmental remediation; Pb; Resistant bacteria; Heavy metals
ID CONTAMINATED SOIL; ACTIVATED CARBON; AQUEOUS-SOLUTION; HEAVY-METALS;
   CADMIUM; LEAD; REMOVAL; BIOSORPTION; REMEDIATION; MECHANISMS
"AB Microbial immobilization is a new strategy for bio-remediation of heavy metals. However, information regarding immobilization of wild resistant bacteria onto biochar for heavy metal separation is limited. This study proposed the use of wild-resistant bacteria TJ6 in combination with biochar produced from agricultural waste (BC-TJ6) for Pb removal and investigated the associated mechanisms using Fourier transform infrared spectroscopy (FT-IR), energy dispersive spectroscopy (EDS), and X-ray photoelectron spectroscopy (XPS). Compared with the theoretical adsorption capacity according to the linear combination calculating by the mass ratio and adsorption capacity of individual component in the composite material, the actual adsorption capacity of BC-TJ6 for Pb(II) was increased by 73.54%. Specifically, the bridge between the bacteria and biochar of Pb(II) may explain the higher observed adsorption capacity of BC-TJ6 compared to theoretical values. EDS analysis based on electron micrograph showed that Pb(II) adsorbed onto TJ6 strain was 0.48%, while that onto biochar component was 0.14%. It indicated that the resistant bacteria contributed more to the adsorption of Pb(II) in BC-TJ6 composites. The FT-IR analysis suggested that the amide, carboxyl, and hydroxyl groups on the surface of the composite were involved in the adsorption of Pb(II). Further, XPS analysis demonstrated that Pb-O (42.12%) and Pb-OOC (27.6%) were the main Pb-molecular structures bound to the surface of the composite. This study is significant for improving the understanding of biochar-wild resistant bacterial combinations for heavy metal adsorption, and can provide guidance for the use of biochar-microbial remediation of heavy metal pollution."
"C1 [Wang, Di; Chen, Hansong; Sun, Qi; Cao, Churong; Wu, Ting; Zheng, Shaocheng] Zhejiang Normal Univ, Coll Xingzhi, Jinhua 321000, Peoples R China."
"   [Wang, Di; Chen, Hansong; Zheng, Shaocheng] Zhejiang Normal Univ, Coll Geog & Environm Sci, Jinhua 321000, Peoples R China."
"   [Xin, Chaoyang; Yuan, Ying] Jinhua Ecol Environm Emergency & Monitoring Ctr, Jinhua 321000, Peoples R China."
"   [Chao, Herong] Chinese Acad Sci, Res Ctr Soil & Water Conservat & Ecol Environm, Yangling 712100, Shaanxi, Peoples R China."
"   [Chao, Herong] Minist Educ, Yangling 712100, Peoples R China."
"   [Chao, Herong] Northwest A&F Univ, Coll Nat Resources & Environm, Yangling 712100, Peoples R China."
C3 Zhejiang Normal University; Zhejiang Normal University; Chinese Academy
"   of Sciences; Institute of Soil & Water Conservation (ISWC), CAS;"
   Northwest A&F University - China
"RP Chen, HS (corresponding author), Zhejiang Normal Univ, Coll Xingzhi, Jinhua 321000, Peoples R China.; Chen, HS (corresponding author), Zhejiang Normal Univ, Coll Geog & Environm Sci, Jinhua 321000, Peoples R China."
EM chenhs@zjnu.edu.cn
"RI Chen, Hansong/JEF-5103-2023; YUAN, YINGFANG/LCD-9628-2024"
"OI Chen, Hansong/0000-0002-0747-7421; "
FU Zhejiang Provincial Natural Science Foundation of China [LTGS23B070003];
"   Key Scien- tific and Technological Project of Jinhua, China"
"   [2021C221870, 20214347]"
FX This research was supported by Zhejiang Provincial Natural Science

Foundation of China under Grant No. LTGS23B070003 and Key Scien- tific
"   and Technological Project of Jinhua, China (2021C221870 and 20214347) ."
"CR Argun ME, 2009, DESALINATION, V249, P519, DOI 10.1016/j.desal.2009.01.020"
"   Chen BY, 2014, BIORESOURCE TECHNOL, V160, P175, DOI 10.1016/j.biortech.2014.02.006"
"   Chen DZ, 2013, BIORESOURCE TECHNOL, V139, P87, DOI 10.1016/j.biortech.2013.04.037"
"   Chen HM, 2019, ENVIRON INT, V127, P395, DOI 10.1016/j.envint.2019.03.068"
"   Cheng S, 2023, J CLEAN PROD, V403, DOI 10.1016/j.jclepro.2023.136821"
"   Choinska-Pulit A, 2018, ECOTOX ENVIRON SAFE, V149, P275, DOI 10.1016/j.ecoenv.2017.12.008"
"   Ding ZH, 2016, J IND ENG CHEM, V33, P239, DOI 10.1016/j.jiec.2015.10.007"
"   El-Naggar A, 2021, ENVIRON INT, V156, DOI 10.1016/j.envint.2021.106638"
"   Fan WH, 2012, CHEMOSPHERE, V88, P751, DOI 10.1016/j.chemosphere.2012.04.047"
"   Fang LC, 2012, WATER RES, V46, P5613, DOI 10.1016/j.watres.2012.07.046"
"   Fang LC, 2010, BIORESOURCE TECHNOL, V101, P5774, DOI 10.1016/j.biortech.2010.02.075"
"   Han H, 2020, ECOTOX ENVIRON SAFE, V195, DOI 10.1016/j.ecoenv.2020.110375"
"   Ho SH, 2017, BIORESOURCE TECHNOL, V246, P142, DOI 10.1016/j.biortech.2017.08.025"
"   Hu XR, 2020, CHEMOSPHERE, V253, DOI 10.1016/j.chemosphere.2020.126737"
"   Jesionowski T, 2014, ADSORPTION, V20, P801, DOI 10.1007/s10450-014-9623-y"
"   Jiang Y, 2022, ENVIRON SCI POLLUT R, V29, P58037, DOI 10.1007/s11356-022-19960-y"
"   Joseph S, 2013, CARBON MANAG, V4, P323, DOI [10.4155/CMT.13.23, 10.4155/cmt.13.23]"
"   Kong LJ, 2017, J CLEAN PROD, V168, P22, DOI 10.1016/j.jclepro.2017.09.005"
"   Li BB, 2018, WATER RES, V146, P55, DOI 10.1016/j.watres.2018.09.006"
"   Li J, 2022, CARBOHYD POLYM, V276, DOI 10.1016/j.carbpol.2021.118789"
"   Li XM, 2016, CHEMOSPHERE, V156, P228, DOI 10.1016/j.chemosphere.2016.04.098"
"   Liu YX, 2017, BIORESOURCE TECHNOL, V235, P332, DOI 10.1016/j.biortech.2017.03.103"
"   Ma ZW, 2021, J ENERGY STORAGE, V40, DOI 10.1016/j.est.2021.102773"
"   Mahbub KR, 2017, SCI TOTAL ENVIRON, V576, P303, DOI 10.1016/j.scitotenv.2016.10.083"
"   Marques APGC, 2011, CRIT REV ENV SCI TEC, V41, P879, DOI 10.1080/10643380903299517"
"   Mathew Blessy Baby, 2018, Groundwater for Sustainable Development, V7, P313, DOI
[10.1016/j.gsd.2018.07.007, 10.1016/j.gsd.2018.07.007]"
"   Mei C, 2022, SCI TOTAL ENVIRON, V839, DOI 10.1016/j.scitotenv.2022.156202"
"   O'Connor D, 2018, ENVIRON INT, V121, P85, DOI 10.1016/j.envint.2018.08.052"
"   Panwichian S, 2010, WORLD J MICROB BIOT, V26, P2199, DOI 10.1007/s11274-010-0405-8"
"   Qiu YP, 2008, WATER RES, V42, P567, DOI 10.1016/j.watres.2007.07.051"
"   Sevak PI, 2021, ENVIRON CHEM LETT, V19, P4463, DOI 10.1007/s10311-021-01296-7"
"   Shen Y, 2017, BIORESOURCE TECHNOL, V244, P1031, DOI 10.1016/j.biortech.2017.08.085"
"   Shen ZT, 2018, CHEMOSPHERE, V190, P260, DOI 10.1016/j.chemosphere.2017.09.140"
"   Teng ZD, 2021, J HAZARD MATER, V419, DOI 10.1016/j.jhazmat.2021.126433"
"   Teng ZD, 2020, J HAZARD MATER, V384, DOI 10.1016/j.jhazmat.2019.121505"
"   Tu C, 2020, ENVIRON INT, V137, DOI 10.1016/j.envint.2020.105576"
"   Wang L, 2020, J CLEAN PROD, V258, DOI 10.1016/j.jclepro.2020.120678"
"   Wang TJ, 2020, ENVIRON SCI POLLUT R, V27, P8707, DOI 10.1007/s11356-019-06957-3"
"   Wang T, 2018, ECOTOX ENVIRON SAFE, V148, P285, DOI 10.1016/j.ecoenv.2017.10.039"
"   Wang YZ, 2021, ENVIRON POLLUT, V289, DOI 10.1016/j.envpol.2021.117829"
"   Yan FF, 2013, BIOCHEM ENG J, V77, P183, DOI 10.1016/j.bej.2013.06.009"
"   Yao YJ, 2011, CHEM ENG J, V170, P82, DOI 10.1016/j.cej.2011.03.031"
"   Zamora-Ledezma C, 2021, ENVIRON TECHNOL INNO, V22, DOI 10.1016/j.eti.2021.101504"
"   Zhang C, 2017, BIORESOURCE TECHNOL, V238, P352, DOI 10.1016/j.biortech.2017.04.051"
"   Zhao XQ, 2021, SCI TOTAL ENVIRON, V777, DOI 10.1016/j.scitotenv.2021.146190"
"   Zheng CF, 2021, J HAZARD MATER, V403, DOI 10.1016/j.jhazmat.2020.123801"
"   Zhu XM, 2017, ENVIRON POLLUT, V227, P98, DOI [10.1016/j.envpol.2017.04.032,
10.1016/j.envPol.2017.04.032]"
NR 47
TC 28
Z9 31
U1 14
U2 116
PU ELSEVIER
PI AMSTERDAM
"PA RADARWEG 29, 1043 NX AMSTERDAM, NETHERLANDS"
SN 1383-5866
EI 1873-3794
J9 SEP PURIF TECHNOL
JI Sep. Purif. Technol.
PD MAR 1
PY 2024
VL 331
AR 125660
DI 10.1016/j.seppur.2023.125660
EA NOV 2023
PG 8
"WC Engineering, Chemical"
WE Science Citation Index Expanded (SCI-EXPANDED)
SC Engineering
GA Z6ZE2
UT WOS:001113528800001
DA 2026-04-06
ER

PT J
"AU Li, XH"
"   Gao, Y"
"   Ning, XL"
"   Li, ZH"
"AF Li, Xianhong"

"   Gao, Yang"
"   Ning, Xiaolin"
"   Li, Zhonghong"
TI Research progress and hotspots on microbial remediation of heavy
   metal-contaminated soil: a systematic review and future perspectives
SO ENVIRONMENTAL SCIENCE AND POLLUTION RESEARCH
LA English
DT Review
DE Heavy metal-contaminated soil; Microbial remediation; Scientometric
   analysis; Web of Science
ID MINE TAILINGS; BIOAUGMENTATION; BACTERIA; BIOREMEDIATION; REDUCTION;
   BIOMINERALIZATION; PHYTOREMEDIATION; BIOSTIMULATION; BIOSURFACTANT;
   REMOVAL
"AB Microbial remediation technology has received much attention as a green, ecological, and inexpensive technology, and there is great potential for the application of microbial remediation technology for heavy metals (HMs) contaminated soil alone and in conjunction with other technologies in environmental remediation. To gain an in-depth understanding of the latest research progress, research hotspots, and development trends on microbial remediation of HMs-contaminated soil, and to objectively reflect the scientific contributions and impacts of relevant countries/regions, institutions, and individuals of this field, in this manuscript, ISI Web of Knowledge's Web of Science (TM) core collection database, data visualization, and analysis software Bibliometrix, VOSviewer, and HistCite Pro were used to collect and analyze the relevant literature from 2000 to 2022, and 1409 publications were subjected to scientometric analyses. It involved 327 journals, 5150 authors, 75 countries/regions, and 2740 keywords. The current progress and hotspots on microbial remediation of HMs-contaminated soil since the twenty-first century were analyzed in terms of the top 10 most productive countries (regions), high-yielding authors, source journals, important research institutions, and hotspots of research directions. Over the past 22 years, China, India, and the USA have been the countries with the most articles. The institution and author with the most publications are the Chinese Acad Sci and Zhu YG, respectively. Journal of Hazardous Materials is the most productive journal. The keywords showed 6 co-occurrence clusters. These findings revealed the research hotspots, knowledge gaps, and future exploration trends related to microbial remediation of HMs-contaminated soil."
"C1 [Li, Xianhong; Gao, Yang; Ning, Xiaolin] Hangzhou Inst Natl Extremely Weak Magnet Field In, Hangzhou 310028, Peoples R China."
"   [Li, Xianhong; Gao, Yang; Ning, Xiaolin] Beihang Univ, Sch Instrumentat & Optoelect Engn, Beijing 100191, Peoples R China."
"   [Li, Zhonghong] Beijing Univ Civil Engn & Architecture, Sch Environm & Energy Engn, Beijing 100044, Peoples R China."
C3 Beihang University; Beijing University of Civil Engineering &
   Architecture
"RP Li, ZH (corresponding author), Beijing Univ Civil Engn & Architecture, Sch Environm & Energy Engn, Beijing 100044, Peoples R China."
EM pl_lizhh@163.com
"RI Li, Zhonghong/ABB-1165-2021"
"OI Li, Zhonghong/0000-0002-8939-0302"
"CR Achal V, 2012, J HAZARD MATER, V201, P178, DOI 10.1016/j.jhazmat.2011.11.067"
"   Agnello AC, 2016, SCI TOTAL ENVIRON, V563, P693, DOI 10.1016/j.scitotenv.2015.10.061"
"   Al Disi Zulfa, 2022, Biotechnology Reports, V35, pe00747, DOI [10.1016/j.btre.2022.e00747, 10.1016/j.btre.2022.e00747]"
"   Al Disi Z, 2022, ENVIRON TECHNOL INNO, V27, DOI 10.1016/j.eti.2022.102513"
"   Ameen FA, 2020, SCI REP-UK, V10, DOI 10.1038/s41598-019-57210-3"
"   Andreazza R, 2010, CHEMOSPHERE, V81, P1149, DOI 10.1016/j.chemosphere.2010.09.047"
"   Antonangelo JA, 2021, J ENVIRON MANAGE, V277, DOI 10.1016/j.jenvman.2020.111443"
"   Aria M, 2017, J INFORMETR, V11, P959, DOI 10.1016/j.joi.2017.08.007"
"   Awasthi MK, 2020, J HAZARD MATER, V389, DOI 10.1016/j.jhazmat.2020.122116"
"   Azhar U, 2022, ENVIRON RES, V214, DOI 10.1016/j.envres.2022.113918"
"   Bennett RM, 2013, CHEM ECOL, V29, P320, DOI 10.1080/02757540.2013.770478"
"   Bodour AA, 2003, APPL ENVIRON MICROB, V69, P3280, DOI 10.1128/AEM.69.6.3280-3287.2003"
"   Braud A, 2009, CHEMOSPHERE, V74, P280, DOI 10.1016/j.chemosphere.2008.09.013"
"   Cabrales-Gonz??lez AM, 2022, REV MEX ING QUIM, V21, DOI 10.24275/rmiq/Bio2723"
"   Chen CM, 2006, J AM SOC INF SCI TEC, V57, P359, DOI 10.1002/asi.20317"
"   Chen P, 2017, ENVIRON SCI POLLUT R, V24, P21739, DOI 10.1007/s11356-017-9816-5"
"   Chen YX, 2022, CHEMOSPHERE, V297, DOI 10.1016/j.chemosphere.2022.133932"
"   Cycon M, 2017, CHEMOSPHERE, V172, P52, DOI 10.1016/j.chemosphere.2016.12.129"
"   Das C, 2011, BIOREMEDIAT J, V15, P90, DOI 10.1080/10889868.2011.570283"
"   Dhaliwal SS, 2020, ENVIRON SCI POLLUT R, V27, P1319, DOI 10.1007/s11356-019-06967-1"
"   Di Z, 2022, FRONT MICROBIOL, V12, DOI 10.3389/fmicb.2021.816312"
"   Diaz MA, 2015, SOIL SEDIMENT CONTAM, V24, P16, DOI 10.1080/15320383.2014.907239"
"   Dixit R, 2015, SUSTAINABILITY-BASEL, V7, P2189, DOI 10.3390/su7022189"
"   Fang LC, 2020, ENVIRON POLLUT, V265, DOI 10.1016/j.envpol.2020.114744"
"   Gao YF, 2022, ECOTOX ENVIRON SAFE, V248, DOI 10.1016/j.ecoenv.2022.114342"
"   Gonzales LGV, 2023, APPL SOIL ECOL, V182, DOI 10.1016/j.apsoil.2022.104695"
"   Graz M, 2011, INT BIODETER BIODEGR, V65, P124, DOI 10.1016/j.ibiod.2010.08.010"
"   Hirsch JE, 2005, P NATL ACAD SCI USA, V102, P16569, DOI 10.1073/pnas.0507655102"
"   Jalilvand N, 2020, J SOIL SCI PLANT NUT, V20, P206, DOI 10.1007/s42729-019-00121-z"
"   Jia L, 2020, CHEM ENG PROCESS, V158, DOI 10.1016/j.cep.2020.108177"
"   Jiang C, 2009, J HAZARD MATER, V167, P1170, DOI 10.1016/j.jhazmat.2009.01.107"
"   Jin YY, 2018, APPL SCI-BASEL, V8, DOI 10.3390/app8081336"
"   Joner EJ, 2000, PLANT SOIL, V226, P227, DOI 10.1023/A:1026565701391"
"   Khalid FE, 2021, J MAR SCI ENG, V9, DOI 10.3390/jmse9020155"
"   Kocar BD, 2006, ENVIRON SCI TECHNOL, V40, P6715, DOI 10.1021/es061540k"
"   Kumar RN, 2009, J HAZARD MATER, V169, P1119, DOI 10.1016/j.jhazmat.2009.04.069"
"   Lal S, 2018, ENVIRON TECHNOL INNO, V10, P243, DOI 10.1016/j.eti.2018.02.011"
"   Lara P, 2017, ENVIRON SCI POLLUT R, V24, P25513, DOI 10.1007/s11356-016-7191-2"
"   Lee E, 2015, J ENVIRON MANAGE, V147, P124, DOI 10.1016/j.jenvman.2014.08.019"
"   Li SZ, 2020, J HAZARD MATER, V400, DOI 10.1016/j.jhazmat.2020.123255"

"   Li YH, 2021, REV ENVIRON CONTAM T, V257, P69, DOI 10.1007/398_2020_63"
"   Lin H, 2023, INT BIODETER BIODEGR, V178, DOI 10.1016/j.ibiod.2022.105544"
"   Lin H, 2022, CHEMOSPHERE, V304, DOI 10.1016/j.chemosphere.2022.135319"
"   Ma YS, 2018, CHEM ENG J, V353, P410, DOI 10.1016/j.cej.2018.07.131"
"   MEP, 2014, China Soil Pollution Survey Communique in Chinese"
"   Mulligan CN, 2001, ENG GEOL, V60, P193, DOI 10.1016/S0013-7952(00)00101-0"
"   Narayanan M, 2023, J ENVIRON MANAGE, V334, DOI 10.1016/j.jenvman.2023.117532"
"   Pan XY, 2021, AGRICULTURE-BASEL, V11, DOI 10.3390/agriculture11030223"
"   Park J, 2014, SEP PURIF TECHNOL, V133, P291, DOI 10.1016/j.seppur.2014.06.054"
"   Park Y, 2022, CHEMPLUSCHEM, V87, DOI 10.1002/cplu.202100457"
"   Qu JH, 2022, J HAZARD MATER, V423, DOI 10.1016/j.jhazmat.2021.127043"
"   Seki H, 2000, J COLLOID INTERF SCI, V229, P196, DOI 10.1006/jcis.2000.6998"
"   Shah V, 2020, ENVIRON TECHNOL INNO, V18, DOI 10.1016/j.eti.2020.100774"
"   Sharma P, 2022, ENVIRON POLLUT, V305, DOI 10.1016/j.envpol.2022.119248"
"   Shi GY, 2023, ENVIRON SCI POLLUT R, V30, P28247, DOI 10.1007/s11356-022-24224-w"
"   Sodhi KK, 2019, ENVIRON TECHNOL INNO, V16, DOI 10.1016/j.eti.2019.100462"
"   Tan H, 2020, J HAZARD MATER, V386, DOI 10.1016/j.jhazmat.2019.121628"
"   Tan YL, 2023, ENVIRON SCI POLLUT R, V30, P2754, DOI 10.1007/s11356-022-22211-9"
"   Teng ZD, 2019, CHEM ENG J, V375, DOI 10.1016/j.cej.2019.122113"
"   Terry LR, 2015, APPL ENVIRON MICROB, V81, P8478, DOI 10.1128/AEM.01970-15"
"   Tran HT, 2023, J ENVIRON MANAGE, V342, DOI 10.1016/j.jenvman.2023.118191"
"   Uddin MM, 2021, TOXICS, V9, DOI 10.3390/toxics9120360"
"   van Eck NJ, 2010, SCIENTOMETRICS, V84, P523, DOI 10.1007/s11192-009-0146-3"
"   Wan J, 2018, J HAZARD MATER, V343, P332, DOI 10.1016/j.jhazmat.2017.09.053"
"   Wang X, 2020, ECOTOX ENVIRON SAFE, V198, DOI 10.1016/j.ecoenv.2020.110655"
"   Wu BH, 2022, SCI TOTAL ENVIRON, V808, DOI 10.1016/j.scitotenv.2021.151995"
"   Xia MQ, 2023, ENVIRON SCI POLLUT R, V30, P69711, DOI 10.1007/s11356-023-27404-4"
"   Xie YL, 2021, J CLEAN PROD, V312, DOI 10.1016/j.jclepro.2021.127749"
"   Xu PF, 2022, J CLEAN PROD, V337, DOI 10.1016/j.jclepro.2022.130510"
"   Yaashikaa PR, 2022, ENVIRON POLLUT, V301, DOI 10.1016/j.envpol.2022.119035"
"   Yang ZH, 2016, J HAZARD MATER, V301, P145, DOI 10.1016/j.jhazmat.2015.08.047"
"   Yin HQ, 2015, SCI REP-UK, V5, DOI 10.1038/srep14266"
"   Zaidi S, 2006, CHEMOSPHERE, V64, P991, DOI 10.1016/j.chemosphere.2005.12.057"
"   Zeng XF, 2015, J SOIL SEDIMENT, V15, P1029, DOI 10.1007/s11368-015-1076-8"
"   Zhai XQ, 2018, SCI TOTAL ENVIRON, V635, P92, DOI 10.1016/j.scitotenv.2018.04.119"
"   Zhao MY, 2022, FRONT BIOENG BIOTECH, V10, DOI 10.3389/fbioe.2022.914253"
"   Zhao WT, 2022, ENVIRON POLLUT, V303, DOI 10.1016/j.envpol.2022.119120"
"   Zhao Y, 2017, ENVIRON SCI POLLUT R, V24, P372, DOI 10.1007/s11356-016-7810-y"
"   Zheng XM, 2022, J HAZARD MATER, V438, DOI 10.1016/j.jhazmat.2022.129557"
"   Zhou QQ, 2020, GLOB ECOL CONSERV, V22, DOI 10.1016/j.gecco.2020.e00925"
NR 80
TC 14
Z9 17
U1 19
U2 135
PU SPRINGER HEIDELBERG
PI HEIDELBERG
"PA TIERGARTENSTRASSE 17, D-69121 HEIDELBERG, GERMANY"
SN 0944-1344
EI 1614-7499
J9 ENVIRON SCI POLLUT R
JI Environ. Sci. Pollut. Res.
PD DEC
PY 2023
VL 30
IS 56
BP 118192
EP 118212
DI 10.1007/s11356-023-30655-w
PG 21
WC Environmental Sciences
WE Science Citation Index Expanded (SCI-EXPANDED)
SC Environmental Sciences & Ecology
GA AQ9L5
UT WOS:001120043100046
PM 37936038
DA 2026-04-06
ER

PT J
"AU Zhang, H"
"   Cui, JL"
"   Xiong, Y"
"   Li, GW"
"   Du, CL"
"   Zhang, LY"
"AF Zhang, Han"
"   Cui, Jianglong"
"   Xiong, Ying"
"   Li, Guowen"
"   Du, Caili"
"   Zhang, Lieyu"
TI Microbial response and the crucial function of predominant phyla in
   Sedum alfredii-mediated remediation of high concentration of multiple

heavy metal soils
SO ENVIRONMENTAL POLLUTION
LA English
DT Article
DE Microbial remediation; Phytoremediation; Microbial reactions; Sedum alfredii
ID HYPERACCUMULATOR; CONTAMINATION; ACCUMULATION; RHIZOSPHERE; CADMIUM; MATTER
"AB Phytoremediation of soils contaminated with high concentrations of multiple heavy metals (HCMHMs) is a promising technique. However, the microbial response mechanisms during the phytoremediation process remain poorly understood. The role of microbes in HCMHMs soil remediation may be underestimated. This study investigated microbial responses and their potential roles in HCMHMs soil remediation using the hyperaccumulator plant Sedum alfredii (S. alfredii). Soil microbial communities were characterised by 16S rRNA sequencing, and metabolic pathways and functions were predicted using PICRUSt2 analysis. The results indicated that the impact of heavy metals on bacterial community structure was more significant than that of S. alfredii. The formation of dominant phyla such as Proteobacteria and Patescibacteria played a crucial role in the bacterial remediation of HCMHMs soils. Proteobacteria utilised the Inorganic ion transport and metabolism gene clusters to translocate heavy metals or reduce their bioavailability and toxicity. Patescibacteria used the Replication, recombination and repair gene clusters to repair damaged genes, enhancing bacterial tolerance of heavy metals. The results provided new insights into the role of microbes during phytoremediation and offered a scientific basis for optimizing phytoremediation technologies. This study demonstrated that dominant phyla effectively mitigated the damage to soil ecological functions from HCMHMs soil."
"C1 [Zhang, Han; Cui, Jianglong; Li, Guowen; Du, Caili; Zhang, Lieyu] Chinese Res Inst Environm Sci, State Key Lab Environm Criteria & Risk Assessment, Beijing 100012, Peoples R China."
"   [Xiong, Ying] Beijing Water Sci & Technol Inst, Beijing, Peoples R China."
C3 Chinese Research Academy of Environmental Sciences
"RP Zhang, LY (corresponding author), Chinese Res Inst Environm Sci, State Key Lab Environm Criteria & Risk Assessment, Beijing 100012, Peoples R China."
EM zhanglieyu@163.com
"FU National Key Research and Devel-opment Program, China [2022YFD1601001];"
Fundamental Research Funds for Central Public Welfare Scientific Research Institutes of China [2021-JY-37]
FX This research was funded by the National Key Research and Devel-opment
"   Program, China (2022YFD1601001) and the Fundamental Research Funds for"
Central Public Welfare Scientific Research Institutes of China (2021-JY-37)
"CR Bian FY, 2023, J SOIL SEDIMENT, V23, P368, DOI 10.1007/s11368-022-03303-y"
"   Chatterjee S, 2009, WORLD J MICROB BIOT, V25, P1829, DOI 10.1007/s11274-009-0084-5"
"   Chen SS, 2018, INT J MOL SCI, V19, DOI 10.3390/ijms19041216"
"   Chen Y, 2018, SCI TOTAL ENVIRON, V637, P1400, DOI 10.1016/j.scitotenv.2018.05.109"
"   Dey P, 2022, FRONT MICROBIOL, V13, DOI 10.3389/fmicb.2022.872576"
"   Douglas GM, 2020, NAT BIOTECHNOL, V38, P685, DOI 10.1038/s41587-020-0548-6"
"   Ettema TJG, 2003, TRENDS BIOCHEM SCI, V28, P170, DOI 10.1016/S0968-0004(03)00037-9"
"   Furukawa K, 2015, MOL CELL, V57, P1088, DOI 10.1016/j.molcel.2015.02.009"
"   HIGHAM DP, 1986, ENVIRON HEALTH PERSP, V65, P5, DOI 10.2307/3430155"
"   Hou DD, 2018, APPL ENVIRON MICROB, V84, DOI [10.1128/AEM.02797-17, 10.1128/aem.02797-17]"
"   Huang WY, 2023, CELL HOST MICROBE, V31, P112, DOI 10.1016/j.chom.2022.11.013"
"   Jin XF, 2008, J HAZARD MATER, V156, P387, DOI 10.1016/j.jhazmat.2007.12.064"
"   Johnson H, 2019, Computational Molecular Bioscience, V09, P1, DOI [10.4236/cmb.2019.91001, 10.4236/cmb.2019.91001, DOI 10.4236/CMB.2019.91001]"
"   Joshi PM, 2009, ENVIRON SCI TECHNOL, V43, P5884, DOI 10.1021/es900063b"
"   Kasemodel MC, 2019, SCI TOTAL ENVIRON, V675, P367, DOI 10.1016/j.scitotenv.2019.04.223"
"   Kumar S, 2019, ENVIRON RES, V179, DOI 10.1016/j.envres.2019.108792"
"   Li TQ, 2013, ENVIRON POLLUT, V182, P248, DOI 10.1016/j.envpol.2013.07.025"
"   Li WC, 2007, J EXP BOT, V58, P4173, DOI 10.1093/jxb/erm274"
"   Li YY, 2022, GUT MICROBES, V14, DOI 10.1080/19490976.2022.2096994"
"   Lima S, 2022, J HAZARD MATER, V431, DOI 10.1016/j.jhazmat.2022.128594"
"   Lin H, 2021, J HAZARD MATER, V402, DOI 10.1016/j.jhazmat.2020.123829"
"   Liu CS, 2016, APPL MICROBIOL BIOT, V100, P1421, DOI 10.1007/s00253-015-7039-6"
"   Ma L, 2024, SCI TOTAL ENVIRON, V906, DOI 10.1016/j.scitotenv.2023.167645"
"   Ma SY, 2022, ENVIRON RES, V214, DOI 10.1016/j.envres.2022.113822"
"   Majumder A, 2013, SCI TOTAL ENVIRON, V463, P1006, DOI 10.1016/j.scitotenv.2013.06.068"
"   Margesin R, 2011, CHEMOSPHERE, V82, P1583, DOI 10.1016/j.chemosphere.2010.11.056"
"   McMaster E., 2020, Parasite or Symbiont: Investigation of the Relationship Between Patescibacteria and Goldbacteria, DOI DOI 10.13140/RG.2.2.19160.57601"
"   Muthusaravanan S, 2018, ENVIRON CHEM LETT, V16, P1339, DOI 10.1007/s10311-018-0762-3"
"   Nemerow N.L., 1974, Scientific Stream Pollution Analysis"
"   Noghabi KA, 2007, PROCESS BIOCHEM, V42, P847, DOI 10.1016/j.procbio.2007.02.004"
"   Qian FH, 2023, J HAZARD MATER, V456, DOI 10.1016/j.jhazmat.2023.131681"
"   Rogers MB, 2016, MICROBIOME, V4, DOI 10.1186/s40168-016-0211-0"
"   Shen X, 2022, CHEMOSPHERE, V291, DOI 10.1016/j.chemosphere.2021.132979"
"   Song WY, 2022, INT J PHYTOREMEDIAT, V24, P744, DOI 10.1080/15226514.2021.1970103"
"   Thompson LR, 2017, NATURE, V551, P457, DOI 10.1038/nature24621"
"   Tian ZJ, 2022, AGR ECOSYST ENVIRON, V339, DOI 10.1016/j.agee.2022.108090"
"   Wang X, 2020, ECOTOX ENVIRON SAFE, V198, DOI 10.1016/j.ecoenv.2020.110655"
"   Wang YX, 2023, CELL, V186, P4803, DOI 10.1016/j.cell.2023.08.017"
"   Wang ZH, 2025, J ENVIRON SCI, V147, P179, DOI 10.1016/j.jes.2023.10.002"
"   Wu BH, 2022, SCI TOTAL ENVIRON, V808, DOI 10.1016/j.scitotenv.2021.151995"
"   Wu FY, 2007, PLANTA, V226, P1363, DOI 10.1007/s00425-007-0575-2"
"   Ye J, 2017, ISME J, V11, P1087, DOI 10.1038/ismej.2016.187"
"   Zhang C, 2016, SCI TOTAL ENVIRON, V557, P785, DOI 10.1016/j.scitotenv.2016.01.170"
"   Zhang H, 2024, SCI TOTAL ENVIRON, V947, DOI 10.1016/j.scitotenv.2024.174565"
"   Zhang RF, 2017, CURR OPIN MICROBIOL, V37, P8, DOI 10.1016/j.mib.2017.03.008"

"   Zheng J, 2017, ONCOTARGET, V8, P82314, DOI 10.18632/oncotarget.19319"
"   Zuo YY, 2023, ECOTOX ENVIRON SAFE, V262, DOI 10.1016/j.ecoenv.2023.115190"
NR 47
TC 5
Z9 6
U1 17
U2 32
PU ELSEVIER SCI LTD
PI London
"PA 125 London Wall, London, the United Kingdom"
SN 0269-7491
EI 1873-6424
J9 ENVIRON POLLUT
JI Environ. Pollut.
PD JUN 1
PY 2025
VL 374
AR 126211
DI 10.1016/j.envpol.2025.126211
EA APR 2025
PG 7
WC Environmental Sciences
WE Science Citation Index Expanded (SCI-EXPANDED)
SC Environmental Sciences & Ecology
GA 2BN0N
UT WOS:001478712700001
PM 40189089
OA hybrid
DA 2026-04-06
ER

PT J
"AU Shan, SP"
"   Cheng, W"
"   Li, YL"
"   Zhang, M"
"   Liu, ZD"
"   Wang, YS"
"   Wei, XW"
"   Fu, ZJ"
"   Wu, SD"
"   Du, DX"
"   Guo, ZH"
"AF Shan, Shiping"
"   Cheng, Wei"
"   Li, Yilu"
"   Zhang, Min"
"   Liu, Zhudong"
"   Wang, Yushuang"
"   Wei, Xiaowu"
"   Fu, Zujiao"
"   Wu, Shandong"
"   Du, Dongxia"
"   Guo, Zhaohui"
TI Comparative genomic analysis of Citrobacter sp. XT1-2-2 reveals
   insights into the molecular mechanism of microbial immobilization of
   heavy metals
SO BMC GENOMICS
LA English
DT Article
DE Citrobacter; Cadmium; Microbial immobilization; Rice; Sulfate reduction
   pathway
ID BIOCHEMICAL-IDENTIFICATION; RESISTANCE; ACCUMULATION; BACTERIA; BIOFILM;
   SYSTEM; GENES; NOV
"AB Background: In our previous study, Citrobacter sp. XT1-2-2 was isolated from high cadmium-contaminated soils, and demonstrated an excellent ability to decrease the bioavailability of cadmium in the soil and inhibit cadmium uptake in rice. In addition, the strain XT1-2-2 could significantly promote rice growth and increase rice biomass. Therefore, the strain XT1-2-2 shows great potential for remediation of cadmium - contaminated soils. However, the genome sequence of this organism has not been reported so far. Results: Here the basic characteristics and genetic diversity of the strain XT1-2-2 were described, together with the draft genome and comparative genomic results. The strain XT1-2-2 is 5040459 bp long with an average G + C content of 52.09%, and contains a total of 4801 genes. Putative genomic islands were predicted in the genome of Citrobacter sp. XT1-2-2. All genes of a complete set of sulfate reduction pathway and various putative heavy metal resistance genes in the genome were identified and analyzed. Conclusions: These analytical results provide insights into the genomic basis of microbial immobilization of heavy metals."
"C1 [Shan, Shiping; Cheng, Wei; Li, Yilu; Zhang, Min; Liu, Zhudong; Wang, Yushuang; Wei, Xiaowu; Fu, Zujiao; Wu, Shandong; Du, Dongxia; Guo, Zhaohui] Hunan Inst Microbiol, Changsha 410009, Hunan, Peoples R China."
"   [Shan, Shiping; Guo, Zhaohui] Hunan Engn & Technol Res Ctr Agr Microbiol Applica, Changsha 410009, Hunan, Peoples R China."
"RP Zhang, M; Du, DX; Guo, ZH (corresponding author), Hunan Inst Microbiol, Changsha 410009, Hunan, Peoples R China."
EM 178488510@qq.com; xiaxia414@126.com; 157263033@qq.com
"RI Fu, Zujiao/OYF-5510-2025; cheng, wei/V-4156-2017"
FU National Natural Science Foundation of China; Natural Science Foundation

of Hunan Province of China [32171633]; Key Technology Research Project
of Hunan Province of China [2021JJ40307]; [2021NK1040]
FX <STRONG> </STRONG>This work was supported by the National Natural
" Science Foundation of China (Project No.32171633), Natural Science"
Foundation of Hunan Province of China (Project No. 2021JJ40307) and Key
Technology Research Project of Hunan Province of China (Project No.
2021NK1040).
"CR [Anonymous], 2005, Int J Syst Evol Microbiol, V55, P1395, DOI 10.1099/ijs.0.63883-0"
" Anton A, 1999, J BACTERIOL, V181, P6876, DOI 10.1128/JB.181.22.6876-6881.1999"
" Arens Sofie, 1997, Clin Microbiol Infect, V3, P53"
" Barkay T, 2003, FEMS MICROBIOL REV, V27, P355, DOI 10.1016/S0168-6445(03)00046-9"
" Bertelli C, 2017, NUCLEIC ACIDS RES, V45, pW30, DOI 10.1093/nar/gkx343"
" Bottacini F, 2010, MICROBIOL-SGM, V156, P3243, DOI 10.1099/mic.0.039545-0"
" Brenner DJ, 1999, J CLIN MICROBIOL, V37, P2619, DOI 10.1128/JCM.37.8.2619-2624.1999"
" Chauhan NS, 2017, INDIAN J MICROBIOL, V57, P299, DOI 10.1007/s12088-017-0658-0"
" Chien CC, 2013, APPL BIOCHEM BIOTECH, V169, P1837, DOI 10.1007/s12010-012-0047-4"
" Finlay JA, 1999, BIOTECHNOL BIOENG, V63, P87, DOI 10.1002/(SICI)1097-0290(19990405)63:1<87::AID-
BIT9>3.0.CO;2-0"
" Franke S, 2003, J BACTERIOL, V185, P3804, DOI 10.1128/JB.185.13.3804-3812.2003"
" Gu RJ, 2020, ECOTOX ENVIRON SAFE, V204, DOI 10.1016/j.ecoenv.2020.111060"
" Guglielmini J, 2011, PLOS GENET, V7, DOI 10.1371/journal.pgen.1002222"
" JANDA JM, 1994, J CLIN MICROBIOL, V32, P1850, DOI 10.1128/JCM.32.8.1850-1854.1994"
" Kanehisa M, 2000, NUCLEIC ACIDS RES, V28, P27, DOI 10.1093/nar/28.1.27"
" Kanehisa M, 2021, NUCLEIC ACIDS RES, V49, pD545, DOI 10.1093/nar/gkaa970"
" Kanehisa M, 2019, PROTEIN SCI, V28, P1947, DOI 10.1002/pro.3715"
" LIPSKY BA, 1980, REV INFECT DIS, V2, P746"
" Liu LH, 2018, J MICROBIOL IMMUNOL, V51, P565, DOI 10.1016/j.jmii.2016.08.016"
" MACASKIE LE, 1995, J CHEM TECHNOL BIOT, V63, P1, DOI 10.1002/jctb.280630102"
" Meier-Kolthoff JP, 2019, NAT COMMUN, V10, DOI 10.1038/s41467-019-10210-3"
" Meier-Kolthoff JP, 2013, BMC BIOINFORMATICS, V14, DOI 10.1186/1471-2105-14-60"
" Miki K, 1996, MICROBIOL IMMUNOL, V40, P915, DOI 10.1111/j.1348-0421.1996.tb01160.x"
" Partridge SR, 2018, CLIN MICROBIOL REV, V31, DOI 10.1128/CMR.00088-17"
" Ramos-Vivas J, 2020, FRONT CELL INFECT MI, V10, DOI 10.3389/fcimb.2020.00447"
" Santos RERS, 2021, INFECT IMMUN, V89, DOI 10.1128/IAI.00311-21"
" Shan SP, 2020, SCI TOTAL ENVIRON, V708, DOI 10.1016/j.scitotenv.2019.135224"
" Shan SP, 2019, ECOTOX ENVIRON SAFE, V169, P292, DOI 10.1016/j.ecoenv.2018.11.030"
" Siche S, 2010, RES MICROBIOL, V161, P824, DOI 10.1016/j.resmic.2010.09.010"
" STACKEBRANDT E, 1988, INT J SYST BACTERIOL, V38, P321, DOI 10.1099/00207713-38-3-321"
" Wang HL, 2021, ANTON LEEUW INT J G, V114, P1285, DOI 10.1007/s10482-021-01601-y"
" Werkman CH, 1932, J BACTERIOL, V23, P167, DOI 10.1128/JB.23.2.167-182.1932"
" WEST MARY G., 1954, PUBL HEALTH MONOG, V22, P1"
" WOESE CR, 1990, P NATL ACAD SCI USA, V87, P4576, DOI 10.1073/pnas.87.12.4576"
" Xia X, 2016, STAND GENOMIC SCI, V11, DOI 10.1186/s40793-016-0169-3"
" Xiao B, 2016, MICROB PATHOGENESIS, V93, P194, DOI 10.1016/j.micpath.2016.01.016"
" Yuan C, 2019, FRONT MICROBIOL, V10, DOI 10.3389/fmicb.2019.02774"
" Zerbino DR, 2008, GENOME RES, V18, P821, DOI 10.1101/gr.074492.107"
" Zhou G, 2017, BMC GENOMICS, V18, DOI 10.1186/s12864-017-4157-9"
" Zinicovscaia I, 2020, ENVIRON SCI POLLUT R, V27, P10495, DOI 10.1007/s11356-020-07690-y"
NR 40
TC 4
Z9 5
U1 2
U2 38
PU BMC
PI LONDON
"PA CAMPUS, 4 CRINAN ST, LONDON N1 9XW, the United Kingdom"
SN 1471-2164
J9 BMC GENOMICS
JI BMC Genomics
PD DEC 19
PY 2022
VL 23
IS 1
AR 838
DI 10.1186/s12864-022-09069-4
PG 13
WC Biotechnology & Applied Microbiology; Genetics & Heredity
WE Science Citation Index Expanded (SCI-EXPANDED)
SC Biotechnology & Applied Microbiology; Genetics & Heredity
GA 7E0ZY
UT WOS:000900908800003
PM 36536293
"OA Green Submitted, gold"
DA 2026-04-06
ER

PT J
"AU Vasarevicius, S"
" Paliuliene, V"
"AF Vasarevicius, Saulius"
" Paliuliene, Vaida"
"TI Immobilization of Cadmium, Lead, and Copper in Soil Using Bacteria: A"
Literature Review
SO LAND

LA English
DT Review
DE heavy metals; bacterial immobilization; cadmium; lead; copper;
biosorption; soil remediation; environmental sustainability
ID HEAVY-METALS; PSEUDOMONAS-AERUGINOSA; BIOSORPTION; CD; GROWTH; ZINC; PB;
NI; CR
"AB The heavy metal contamination of soils is a global environmental challenge threatening water quality, food safety, and human health. Using a systematic literature review approach, this study aimed to assess the potential of bacterial strains to immobilize cadmium (Cd2+), lead (Pb2+), and copper (Cu2+) in contaminated soils. A total of 45 articles were analyzed, focusing on studies that reported heavy metal concentrations before and after bacterial treatment. The analysis revealed that bacterial genera such as Bacillus, Pseudomonas, and Enterobacter were most commonly used for the immobilization of these metals. Immobilization efficiencies ranged from 25% to over 98%, with higher efficiencies generally observed when microbial consortia or amendments (e.g., phosphate compounds and biochar) were applied. The main immobilization mechanisms included biosorption, bioprecipitation (such as carbonate-induced precipitation), bioaccumulation, and biomineralization, which convert mobile metal ions into more stable, less bioavailable forms. These findings highlight the promising role of microbial-assisted immobilization in mitigating heavy metal pollution and reducing ecological risks. Further laboratory and field studies are needed to optimize the use of these microbial strains under site-specific conditions to ensure effective and sustainable soil remediation practices."
"C1 [Vasarevicius, Saulius; Paliuliene, Vaida] Vilnius Gediminas Tech Univ, Dept Environm Protect & Water Engn, LT-10223 Vilnius, Lithuania."
C3 Vilnius Gediminas Technical University
"RP Vasarevicius, S (corresponding author), Vilnius Gediminas Tech Univ, Dept Environm Protect & Water Engn, LT-10223 Vilnius, Lithuania."
EM saulius.vasarevicius@vilniustech.lt; vaida.paliuliene@vilniustech.lt
"RI Vasarevicius, Saulius/E-8342-2017"
"CR Abioye OP, 2018, Brazilian Journal of Biological Sciences, V5, P25, DOI [10.21472/bjbs.050903, 10.21472/bjbs.050903, DOI 10.21472/BJBS.050903]"
" Afridi MS, 2021, BIOLOGIA, V76, P3091, DOI 10.1007/s11756-021-00826-6"
" Aka RJN, 2016, INT J PHYTOREMEDIAT, V18, P200, DOI 10.1080/15226514.2015.1073671"
" Al Disi Zulfa, 2022, Biotechnology Reports, V35, pe00747, DOI [10.1016/j.btre.2022.e00747, 10.1016/j.btre.2022.e00747]"
" Alotaibi BS, 2021, MICROORGANISMS, V9, DOI 10.3390/microorganisms9081628"
" Arifiyanto A, 2017, AIP CONF PROC, V1854, DOI 10.1063/1.4985394"
" Atuchin VV, 2023, MICROORGANISMS, V11, DOI 10.3390/microorganisms11040864"
" Ayangbenro AS, 2019, CHEMOSPHERE, V231, P113, DOI 10.1016/j.chemosphere.2019.05.092"
" Ayangbenro AS, 2018, SUSTAINABILITY-BASEL, V10, DOI 10.3390/su10093028"
" Basha S. A., 2014, International Journal of Current Microbiology and Applied Sciences, V3, P785"
" Basit A, 2024, FOLIA MICROBIOL, V69, P975, DOI 10.1007/s12223-024-01135-6"
" Cephidian A, 2016, INT J ENVIRON SCI TE, V13, P2135, DOI 10.1007/s13762-016-1023-2"
" Chen XC, 2006, CAN J MICROBIOL, V52, P308, DOI 10.1139/W05-157"
" Desoky EM, 2020, ECOTOX ENVIRON SAFE, V198, DOI 10.1016/j.ecoenv.2020.110685"
" Dotaniya M.L., 2021, Handbook of Nanomaterials and Nanocomposites for Energy and Environmental Applications, DOI [10.1007/978-3-030-36268-3_65, DOI 10.1007/978-3-030-36268-3_65]"
" Fashola MO, 2020, INT J ENVIRON RES, V14, P71, DOI 10.1007/s41742-019-00240-6"
" Ghandali MV, 2024, SCI REP-UK, V14, DOI 10.1038/s41598-024-61270-5"
" Han H, 2020, ENVIRON POLLUT, V267, DOI 10.1016/j.envpol.2020.115432"
" Han H, 2020, ECOTOX ENVIRON SAFE, V195, DOI 10.1016/j.ecoenv.2020.110375"
" Hassan SHA, 2009, J GEN APPL MICROBIOL, V55, P27, DOI 10.2323/jgam.55.27"
" He Jing, 2020, Microbiology and Biotechnology Letters, V48, P223, DOI [10.4014/mbl.1912.12011, 10.4014/mbl.1912.12011]"
" Heidari P, 2020, Journal of Applied Biotechnology Reports, V7, P208, DOI [10.30491/jabr.2020.120185, 10.30491/JABR.2020.120185, DOI 10.30491/JABR.2020.120185]"
" Hu XS, 2024, J CLEAN PROD, V436, DOI 10.1016/j.jclepro.2023.140508"
" Huang YT, 2024, J HAZARD MATER, V469, DOI 10.1016/j.jhazmat.2024.133966"
" Jiang MY, 2023, BIOCHAR, V5, DOI 10.1007/s42773-022-00201-x"
" Kapahi M, 2019, J HEALTH POLLUT, V9, DOI 10.5696/2156-9614-9.24.191203"
" Karakagh Rahim Mohammadzadeh, 2012, Ecohydrology & Hydrobiology, V12, P191, DOI 10.1016/S1642-3593(12)70203-3"
" Karimpour M, 2018, DATA BRIEF, V18, P1185, DOI 10.1016/j.dib.2018.04.014"
" Karmakar Diyashree, 2024, Journal of Applied Biology & Biotechnology, V12, P22, DOI [10.7324/jabb.2024.157765, 10.7324/JABB.2024.157765]"
" Kassimghaima K., 2017, International Journal of Applied Environmental Sciences, V12, P1649"
" Khalid S, 2023, SOIL ECOL LETT, V5, DOI 10.1007/s42832-022-0162-2"
" Kim JJ, 2019, J TRACE ELEM MED BIO, V54, P226, DOI 10.1016/j.jtemb.2019.05.003"
" Kim SY, 2015, J BIOSCI BIOENG, V119, P433, DOI 10.1016/j.jbiosc.2014.09.022"
" Kou B, 2023, ENVIRON POLLUT, V322, DOI 10.1016/j.envpol.2023.121136"
" Li Y, 2017, ECOTOX ENVIRON SAFE, V138, P56, DOI 10.1016/j.ecoenv.2016.12.024"
" Liu F, 2024, SCI TOTAL ENVIRON, V912, DOI 10.1016/j.scitotenv.2023.168990"
" Liu TZ, 2018, ENVIRON POLLUT, V238, P359, DOI 10.1016/j.envpol.2018.03.022"
" Tarekegn MM, 2020, COGENT FOOD AGR, V6, DOI 10.1080/23311932.2020.1783174"
" Meng ZW, 2022, J HAZARD MATER, V434, DOI 10.1016/j.jhazmat.2022.128903"
" M??nzel T, 2023, CARDIOVASC RES, V119, P440, DOI 10.1093/cvr/cvac082"
" Nath S., 2014, Journal of Environmental and Analytical Toxicology, V4, P4, DOI 10.4172/2161-0525.1000221"
" Oves M, 2013, SAUDI J BIOL SCI, V20, P121, DOI 10.1016/j.sjbs.2012.11.006"
" Palanivel TM, 2020, J ENVIRON MANAGE, V253, DOI 10.1016/j.jenvman.2019.109706"
" Pan JH, 2007, J ENVIRON SCI-CHINA, V19, P403, DOI 10.1016/S1001-0742(07)60067-9"
" Pande V, 2022, FRONT MICROBIOL, V13, DOI 10.3389/fmicb.2022.824084"
" Park JH, 2011, J HAZARD MATER, V185, P829, DOI 10.1016/j.jhazmat.2010.09.095"
" Parmar P, 2020, MICROBIOL RES, V238, DOI 10.1016/j.micres.2020.126526"
" Peng DH, 2020, J HAZARD MATER, V400, DOI 10.1016/j.jhazmat.2020.123116"
" Pratush A, 2018, INT MICROBIOL, V21, P97, DOI 10.1007/s10123-018-0012-3"
" Qi X, 2021, J HAZARD MATER, V401, DOI 10.1016/j.jhazmat.2020.123823"

"   Qiao SY, 2021, CHEMOSPHERE, V274, DOI 10.1016/j.chemosphere.2021.129661"
"   Qiao WC, 2019, ROY SOC OPEN SCI, V6, DOI 10.1098/rsos.181701"
"   Qin SM, 2023, SCI TOTAL ENVIRON, V868, DOI 10.1016/j.scitotenv.2023.161685"
"   Rajesh Priyalaxmi Rajesh Priyalaxmi, 2014, International Journal of Current Microbiology and Applied Sciences, V3, P326"
"   Rodr??guez-Tirado V, 2012, CHEM ENG J, V181, P352, DOI 10.1016/j.cej.2011.11.091"
"   ROHINI B, 2015, INT J ADV RES BIOL S, V2, P200"
"   Sahoo S., 2020, Int J Sci Res, V9, P1337, DOI [https://doi.org/10.21275/ART20204172, DOI 10.21275/ART20204172, 10.21275/ART20204172]"
"   Shan B, 2023, WORLD J MICROB BIOT, V39, DOI 10.1007/s11274-022-03484-1"
"   Singh S., 2020, Bioremediation of Pollutants"
"   Sodhi KK, 2020, SN APPL SCI, V2, DOI 10.1007/s42452-020-03583-4"
"   Soto DF, 2019, J PROTEOMICS, V191, P143, DOI 10.1016/j.jprot.2018.02.024"
"   Tu C, 2020, ENVIRON INT, V137, DOI 10.1016/j.envint.2020.105576"
"   Wang XY, 2022, SCI TOTAL ENVIRON, V819, DOI 10.1016/j.scitotenv.2022.153242"
"   Wang Y, 2020, ECOTOX ENVIRON SAFE, V192, DOI 10.1016/j.ecoenv.2020.110294"
"   Wei T, 2023, ENVIRON GEOCHEM HLTH, V45, P6009, DOI 10.1007/s10653-023-01605-9"
"   Wei YQ, 2020, BIORESOURCE TECHNOL, V296, DOI 10.1016/j.biortech.2019.122375"
"   Wu P, 2022, J HAZARD MATER, V435, DOI 10.1016/j.jhazmat.2022.129002"
"   Wu Y, 2025, J ENVIRON SCI, V147, P370, DOI 10.1016/j.jes.2023.11.010"
"   Yang GY, 2020, Journal of Geoscience and Environment Protection, V08, P140, DOI [10.4236/gep.2020.89009, 10.4236/gep.2020.89009, DOI 10.4236/GEP.2020.89009]"
"   Yuan ZM, 2017, ENVIRON SCI POLLUT R, V24, P21877, DOI 10.1007/s11356-017-9832-5"
"   Zeng XX, 2020, SCI REP-UK, V10, DOI 10.1038/s41598-020-58259-1"
"   Zhang H, 2023, CHEM ENG J, V459, DOI 10.1016/j.cej.2023.141583"
"   Zhang L, 2024, SCI TOTAL ENVIRON, V921, DOI 10.1016/j.scitotenv.2024.171060"
"   Zhu XL, 2019, GEODERMA, V350, P1, DOI 10.1016/j.geoderma.2019.04.015"
"   Zocca VFB, 2022, CRIT REV BIOTECHNOL, V42, P813, DOI 10.1080/07388551.2021.1983516"
NR 75
TC 2
Z9 2
U1 13
U2 17
PU MDPI
PI BASEL
"PA MDPI AG, Grosspeteranlage 5, CH-4052 BASEL, SWITZERLAND"
EI 2073-445X
J9 LAND-BASEL
JI Land
PD JUL 28
PY 2025
VL 14
IS 8
AR 1547
DI 10.3390/land14081547
PG 18
WC Environmental Studies
WE Social Science Citation Index (SSCI)
SC Environmental Sciences & Ecology
GA 6PA9F
UT WOS:001558614000001
"OA Green Submitted, gold"
DA 2026-04-06
ER

PT J
"AU Savov, V"
"   Chakalov, K"
"   Angelova, G"
"   Popova, T"
"   Hristova, R"
"AF Savov, V."
"   Chakalov, K."
"   Angelova, G."
"   Popova, T."
"   Hristova, R."
TI STUDY OF IMMOBILIZED MICROBIAL COMPOSITION ON THE RECLAMATION PROCESS OF
   HEAVY METAL POLLUTED SOILS
SO BIOTECHNOLOGY & BIOTECHNOLOGICAL EQUIPMENT
LA English
DT Article
DE bioremediation; heavy metal; humic substanses; Trichoderma sp.
"AB Heavy metal pollutions are in certain cases phytotoxic. Bioremediation can sometimes be realized with the using of microorganisms for ""removing"" of problematic pollutions from soil and underground water by helping the phytoremediation processes. The purpose ot the study is to determine the influence of biotransformed lignites' derivatives (HA) alone and as an agent for immobilization of micro fungi upon some bioremediation processes in heavy metal polluted soils. The pot vegetative test is realized with Ap horizons of polluted soils from the regions of Lead-Zink Procesing Plant Plovdiv (Alluvial-Meadow, Eutric Fluvisol, FAO) HA are a matrix for immobilization of T.viride and T. harzianum (10(5)CFU/ml solution), at a background treatment with Fe-zeolite. Test plants - garlic and grass mixture. A strong influence of the studied meliorating agents - bioproducts - upon bioremediation processes in polluted soil is observed. A good effect of immobilized micro flora on bioremediation processes is determined. The total content of the accumulated heavy metals from the test culture demonstrates the possibility of using HA as an immobilizing factor for T.viride and T. harzianum during rehabilitation of the productivity of heavy metal polluted soils."

"C1 [Savov, V.] Sofia Univ St Kliment Ohridski, Fac Biol, 8 Dragan Tsankov Str, Sofia 1164, Bulgaria."
"   [Chakalov, K.; Angelova, G.; Popova, T.] ROMB Ltd, Sofia 1606, Bulgaria."
"   [Hristova, R.] BAS, Cent Lab Ecol, Sofia, Bulgaria."
C3 University of Sofia; Bulgarian Academy of Sciences
"RP Savov, V (corresponding author), Sofia Univ St Kliment Ohridski, Fac Biol, 8 Dragan Tsankov Str, Sofia 1164, Bulgaria."
EM romb_ltd@abv.bg
"CR Benkova M., 2005, P NAT C INT PARTC MA, P432"
"   Chakalov K., 2002, P 6 INT C OCC PROP U"
"   Filcheva E., 2002, P 6 INT C OCC PROP U"
"   Ghosh S. K., 2001, BIOREMEDIATION CLEAN"
NR 4
TC 0
Z9 0
U1 1
U2 8
PU TAYLOR & FRANCIS LTD
PI ABINGDON
"PA 2-4 PARK SQUARE, MILTON PARK, ABINGDON OR14 4RN, OXON, the United Kingdom"
SN 1310-2818
EI 1314-3530
J9 BIOTECHNOL BIOTEC EQ
JI Biotechnol. Biotechnol. Equip.
PY 2009
VL 23
SU 1
BP 262
EP 266
DI 10.1080/13102818.2009.10818415
PG 5
WC Biotechnology & Applied Microbiology
WE Science Citation Index Expanded (SCI-EXPANDED)
SC Biotechnology & Applied Microbiology
GA VH2OZ
UT WOS:000451691400065
OA Bronze
DA 2026-04-06
ER

PT J
"AU Hamid, B"
"   Majeed, N"
"   Ganai, BA"
"   Hassan, S"
"   Bashir, Z"
"   Wani, PA"
"   Perveen, K"
"   Sayyed, RZ"
"AF Hamid, Burhan"
"   Majeed, Neesa"
"   Ganai, Bashir Ahmad"
"   Hassan, Shahnawaz"
"   Bashir, Zaffar"
"   Wani, Parvaze Ahmad"
"   Perveen, Kahkashan"
"   Sayyed, R. Z."
TI Heavy-metal tolerant bacterial strains isolated from industrial sites
"   and scrap yards in Kashmir, India"
SO JOURNAL OF BASIC MICROBIOLOGY
LA English
DT Article
DE bioremediation; heavy metal; iron and copper metal contamination; metal
   tolerance; minimum inhibitory concentration
ID CR VI; BIOSORPTION; IRON; BIOREMEDIATION; DETOXIFICATION; WATER
"AB Heavy metal pollution has posed a severe danger to environmental stability due to its high toxicity and lack of biodegradability. The present study deals with the appraisement of tolerance shown by various bacteria in varied copper and iron concentrations. Among the 20 isolates, four isolates, GN2, SC5, SC8, and SC10, exhibiting more significant iron and copper tolerance, were selected and identified by 16 S ribosomal ribonucleic acid (rRNA) gene sequence analysis as Pantoea agglomerans strain GN2, Pantoea sp. strain SC5, Bacillus sp. strain SC8 and Priestia aryabhattaistrain SC10. The minimum inhibitory concentration of molecularly identified strains revealed that P. agglomerans strain GN2 showed tolerance to iron sulfate and copper sulfate upto 600 and 400 mu g/mL, whereas Bacillus sp. SC8 (OQ202165) showed tolerance of 700 and 250 mu g/mL were tolerant to iron sulfate and copper sulfate up to 700 and 150 mu g/mL, respectively. Pantoea sp. strain SC5 showed significant tolerance to both heavy metals. The isolates were further studied for their ability to grow at varying temperatures and pH ranges. Most of the isolates showed optimal growth at 37 degrees C and pH 7. However, Pantoea sp. SC5 was competent to have prominent growth at 45 degrees C and pH 8.0. Microbial remediation, which is ecofriendly, has proven the most effective method for bioremediation of heavy metal-contaminated environments. Using heavy metal-resistant bacteria for microbial remediation of iron and copper-contaminated environments could be a viable and valuable strategy. These isolates could also be used to decontaminate heavy metal-polluted agricultural soils."
"C1 [Hamid, Burhan; Majeed, Neesa; Ganai, Bashir Ahmad; Bashir, Zaffar; Wani, Parvaze Ahmad] Univ Kashmir, Ctr Res Dev, Srinagar, India."
"   [Hassan, Shahnawaz] Univ Kashmir, Dept Environm Sci, Srinagar, India."
"   [Perveen, Kahkashan] King Saud Univ, Coll Sci, Dept Bot & Microbiol, Riyadh, Saudi Arabia."

"   [Sayyed, R. Z.] G B Patel Sci & STKVS Commerce Coll, PSGVP Mandals Shri S I Patil Arts, Dept Microbiol, Shahada, India."
"   [Sayyed, R. Z.] Auburn Ventures, Asian PGPR Soc, Auburn, AL USA."
"   [Hamid, Burhan; Ganai, Bashir Ahmad] Univ Kashmir, Ctr Res Dev, Srinagar 190006, India."
C3 University of Kashmir; University of Kashmir; King Saud University;
   University of Kashmir
"RP Hamid, B; Ganai, BA (corresponding author), Univ Kashmir, Ctr Res Dev, Srinagar 190006, India."
EM burhan.cordct@uok.edu.in; bbcganai@gmail.com
"RI Sayyed, Riyaz/Q-4313-2016; Ganai, Bashir/KII-4537-2024; Perveen,"
"   Kahkashan/N-1679-2014; Hassan, Shahnawaz/KRP-5659-2024"
"OI Sayyed, Riyaz/0000-0002-1553-1213; Ganai, Bashir/0000-0003-4178-5919;"
"   Perveen, Kahkashan/0000-0002-9618-5876; Hassan,"
"   Shahnawaz/0000-0002-4527-4211; Hamid, Burhan/0000-0003-1630-9929"
FU King Saud University [RSP2023R358]
"FX King Saud University, Grant/Award Number: RSP2023R358"
"CR Adegoke IA, 2022, European Journal of Energy Research, V2, P9, DOI [10.24018/ejenergy.2022.2.2.47, DOI 10.24018/EJENERGY.2022.2.2.47]"
"   Ajibade F O., 2021, MICROBE MEDIATED REM, P321, DOI [10.1016/b978-0-12-821199-1.00025-0, DOI 10.1016/B978-0-12-821199-1.00025-0]"
"   Al-Afify ADG, 2022, WATER AIR SOIL POLL, V233, DOI 10.1007/s11270-022-05759-7"
"   Albretsen J, 2006, VET MED-US, V101, P82"
"   Aliasgharpour M., 2020, INT J PREV MED, V1, P11"
"   Alnaimat S., 2017, Biodiversitas: Journal of Biological Diversity, V18, P1250, DOI [10.13057/biodiv/d180350, 10.13057/biodiv/d180350]"
"   Alshaibani DAMH., 2020, EVALUATION EXPLORATI"
"   APHA, 2005, AM PUBL HLTH ASS AM, P2"
"   Asha B., 2018, ASIAN J MICROBIOL BI, V8, P119"
"   Bahafid W., 2017, Yeast-Industrial Applications, P559"
"   Bhat SA., 2019, INT J MED SCI DIAGN, V3, P106, DOI DOI 10.32553/JMSDR"
"   Bhattacharya A, 2013, ENVIRON SCI POLLUT R, V20, P6628, DOI 10.1007/s11356-013-1728-4"
"   Briffa J, 2020, HELIYON, V6, DOI 10.1016/j.heliyon.2020.e04691"
"   Cappuccino JG, 2011, MICROBIOLOGY LAB MAN"
"   Coates TD, 2014, FREE RADICAL BIO MED, V72, P23, DOI 10.1016/j.freeradbiomed.2014.03.039"
"   Dar MA, 2023, REG STUD MAR SCI, V59, DOI 10.1016/j.rsma.2023.102814"
"   De Beni E, 2022, CHEMOSPHERE, V292, DOI 10.1016/j.chemosphere.2021.133448"
"   Forstner U., 2012, METAL POLLUTION AQUA"
"   Gutnick DL, 2000, APPL MICROBIOL BIOT, V54, P451, DOI 10.1007/s002530000438"
"   Holt J.G., 1994, BERGEYS MANUAL DETER, P786, DOI DOI 10.1089/FPD.2014.180"
"   Iskandar NL, 2011, J ENVIRON SCI, V23, P824, DOI 10.1016/S1001-0742(10)60475-5"
"   Jaishankar Monisha, 2014, Interdiscip Toxicol, V7, P60, DOI 10.2478/intox-2014-0009"
"   Jiang D, 2021, PEST MANAG SCI, V77, P1115, DOI 10.1002/ps.6103"
"   Kalaimurugan D, 2020, INT J ENVIRON SCI TE, V17, P1455, DOI 10.1007/s13762-019-02563-5"
"   Karthik C, 2017, J TAIWAN INST CHEM E, V70, P282, DOI 10.1016/j.jtice.2016.11.006"
"   Kerur SS, 2021, MATER TODAY-PROC, V42, P1112, DOI 10.1016/j.matpr.2020.12.492"
"   Khan AL, 2017, J PLANT INTERACT, V12, P550, DOI 10.1080/17429145.2017.1397203"
"   Koju NK, 2022, WATER AIR SOIL POLL, V233, DOI 10.1007/s11270-022-05645-2"
"   Kumar P, 2021, CURR MICROBIOL, V78, P3258, DOI 10.1007/s00284-021-02602-w"
"   Li L, 2020, ENVIRON SCI POLLUT R, V27, P18392, DOI 10.1007/s11356-020-08173-w"
"   Majhi K., 2021, [No title captured], DOI DOI 10.21203/RS.3.RS-705506/V1"
"   Maqsood Q, 2022, ARCH MICROBIOL, V204, DOI 10.1007/s00203-022-03087-2"
"   Marzan LW, 2017, EGYPT J AQUATIC RES, V43, P65, DOI [10.1016/j.ejar.2016.11.002, 10.1016/j.ejar.2016.11.002, DOI 10.1016/j.ejar.2016.11.002, DOI 10.1016/J.EJAR.2016.11.002]"
"   Tarekegn MM, 2020, COGENT FOOD AGR, V6, DOI 10.1080/23311932.2020.1783174"
"   Mukherjee P, 2019, FRONT MICROBIOL, V10, DOI 10.3389/fmicb.2019.01207"
"   Mu??oz AJ, 2012, CHEM ENG J, V210, P325, DOI 10.1016/j.cej.2012.09.007"
"   Nath S, 2019, SN APPL SCI, V1, DOI 10.1007/s42452-019-0762-3"
"   Oves M, 2013, SAUDI J BIOL SCI, V20, P121, DOI 10.1016/j.sjbs.2012.11.006"
"   Pal A, 2022, CRIT REV MICROBIOL, V48, P327, DOI 10.1080/1040841X.2021.1970512"
"   Pandey S, 2013, J MICROBIOL, V51, P11, DOI 10.1007/s12275-013-2330-7"
"   Paulina P., 2014, ADV MATER RES-KR, V945, P3479"
"   Peletiri I. C., 2021, African Journal of Clinical and Experimental Microbiology, V22, P146, DOI [10.4314/ajcem.v22i2.6, 10.4314/ajcem.v22i2.6]"
"   Priyadarshanee M, 2021, J ENVIRON CHEM ENG, V9, DOI 10.1016/j.jece.2020.104686"
"   Qin GW, 2021, CHEMOSPHERE, V267, DOI 10.1016/j.chemosphere.2020.129205"
"   Rehman AU, 2021, J MOL LIQ, V321, DOI 10.1016/j.molliq.2020.114455"
"   Renshaw MA, 2015, MOL ECOL RESOUR, V15, P168, DOI 10.1111/1755-0998.12281"
"   Rizvi A, 2022, ENVIRON EXP BOT, V200, DOI 10.1016/j.envexpbot.2022.104911"
"   Rizvi A, 2020, RSC ADV, V10, P38379, DOI 10.1039/d0ra05610c"
"   Rizvi A, 2020, ENVIRON MONIT ASSESS, V192, DOI 10.1007/s10661-020-08758-5"
"   Rizwan Muhammad, 2016, Environ Sci Pollut Res Int, V23, P17859, DOI 10.1007/s11356-016-6436-4"
"   Sachdev S, 2021, ANTIOXIDANTS-BASEL, V10, DOI 10.3390/antiox10020277"
"   Sharma P, 2021, ENVIRON TECHNOL INNO, V24, DOI 10.1016/j.eti.2021.101826"
"   Shuaib M, 2021, MICROB PATHOGENESIS, V150, DOI 10.1016/j.micpath.2020.104713"
"   Silva B, 2015, BBA-MOL BASIS DIS, V1852, P1347, DOI 10.1016/j.bbadis.2015.03.011"
"   Sonwani S., 2022, AIRBORNE PARTICULATE, P1"
"   Sun QY, 2022, TOXICOLOGY, V469, DOI 10.1016/j.tox.2022.153136"
"   Tarfeen N, 2022, PROCESSES, V10, DOI 10.3390/pr10071358"
"   Pissarra TCT, 2021, SCI TOTAL ENVIRON, V785, DOI 10.1016/j.scitotenv.2021.147331"
"   Wani PA, 2018, APPL SOIL ECOL, V132, P187, DOI 10.1016/j.apsoil.2018.07.012"
"   Wani PA, 2018, J ENVIRON CHEM ENG, V6, P3798, DOI 10.1016/j.jece.2018.05.042"
"   Wood JC, 2015, BRIT J HAEMATOL, V170, P15, DOI 10.1111/bjh.13374"
"   Xing YH, 2021, J CLEAN PROD, V311, DOI 10.1016/j.jclepro.2021.127514"
"   Yatoo AM, 2022, ENVIRON CHEM LETT, V20, P2573, DOI 10.1007/s10311-022-01386-0"
"   Yin Kun Yin Kun, 2019, Chemical Engineering Journal, V360, P1553, DOI 10.1016/j.cej.2018.10.226"

"   Yun S, 2015, CRIT REV ONCOL HEMAT, V95, P12, DOI 10.1016/j.critrevonc.2015.02.006"
"   Zhang W, 2018, PHYTOMEDICINE, V48, P51, DOI 10.1016/j.phymed.2018.04.058"
NR 66
TC 4
Z9 7
U1 1
U2 8
PU WILEY
PI HOBOKEN
"PA 111 RIVER ST, HOBOKEN 07030-5774, NJ USA"
SN 0233-111X
EI 1521-4028
J9 J BASIC MICROB
JI J. Basic Microbiol.
PD DEC
PY 2023
VL 63
IS 12
BP 1361
EP 1372
DI 10.1002/jobm.202300349
EA SEP 2023
PG 12
WC Microbiology
WE Science Citation Index Expanded (SCI-EXPANDED)
SC Microbiology
GA Z4CV5
UT WOS:001067121700001
PM 37712102
DA 2026-04-06
ER

PT J
"AU Fan, GD"
"   Zhou, JH"
"   Cao, XF"
"   You, W"
"   Lin, C"
"   Luo, J"
"   Zou, JY"
"   Xu, KQ"
"   Luo, QD"
"AF Fan, Gongduan"
"   Zhou, Junhou"
"   Cao, Xingfeng"
"   You, Wu"
"   Lin, Chen"
"   Luo, Jing"
"   Zou, Jianyong"
"   Xu, Kai-Qin"
"   Luo, Quanda"
TI Enhanced Remediation of Lead and Cadmium by the Co-System of
   Phosphate-Solubilizing Bacteria Immobilized on Goethite-Modified Biochar
SO WATER
LA English
DT Article
DE biochar; goethite; phosphate-solubilizing bacteria; lead; cadmium;
   environmental remediation
ID ZERO-VALENT IRON; HEAVY-METALS; ADSORPTION; BIOSORPTION; REDUCTION;
   SEDIMENTS; AMMONIUM; PB2+
"AB Bioremediation has drawn widespread concern in passivating heavy metals, but the intense toxicity of heavy metals to biological cells limits the application of functional strains. Herein, goethite-modified biochar (GMB) was chosen as the carrier to immobilize phosphate-solubilizing bacteria (PSB) of strain L1 for lead and cadmium remediation. Batch experiments showed that the GMB-L1 possessed excellent adsorption performance with a maximum adsorption of 496.54 and 178.18 mg/g for Pb and Cd, respectively. Moreover, adding GMB-L1 in contaminated soil converted heavy metals (Pb and Cd) into more stable fractions and reduced TCLP-extracted heavy metal concentrations (73.24% of Pb and 57.25% of Cd). The GMB-L1 was proved to accomplish Pb and Cd remediation via the process of chemical precipitation, surface complexation, electrostatic attraction, and biomineralization, which was accompanied by the transformation of heavy metals into a more stable crystal structure, such as Pb5(PO4)3OH and Cd5(PO4)3OH. Therefore, the co-system of GMB and strain L1 could be regarded as a prospective option for efficiently remedying environmental heavy metal pollution."
"C1 [Fan, Gongduan; Zhou, Junhou; Cao, Xingfeng; Lin, Chen; Xu, Kai-Qin] Fuzhou Univ, Coll Civil Engn, Fuzhou 350116, Peoples R China."
"   [You, Wu] Fujian Agr & Forestry Univ, Sch Life Sci, Fuzhou 350002, Peoples R China."
"   [You, Wu; Luo, Quanda] Fujian Agr Ecol Environm & Energy Technol Extens S, Fuzhou 350001, Peoples R China."
"   [Luo, Jing] Fujian Jinhuang Environm Sci Tech Co Ltd, Fuzhou 350002, Peoples R China."
"   [Zou, Jianyong] Anhui Urban Construct Design Inst Co Ltd, Hefei 230051, Peoples R China."
C3 Fuzhou University; Fujian Agriculture & Forestry University
"RP Fan, GD; Xu, KQ (corresponding author), Fuzhou Univ, Coll Civil Engn, Fuzhou 350116, Peoples R China."
EM fgdfz@fzu.edu.cn; 210527060@fzu.edu.cn; xingfeng_cao@126.com;
   youwu1986@163.com; 220520096@fzu.edu.cn; irenelj22@163.com;
   medricn@gmail.com; joexu@fzu.edu.cn; fjsnyz@163.com
"RI Xu, Kaiqin/A-6253-2011; Cao, Xingfeng/KIK-1560-2024; Fan,"

Gongduan/K-6910-2017
"OI Xu, Kaiqin/0000-0003-0781-3031; Cao, Xingfeng/0009-0008-4164-6883; Fan,"
Gongduan/0000-0001-7687-1046
"FU Natural Science Foundation of Fujian Province in China [2021N0022,"
" 2021Y3002, 2023J02006]; Science and Technology Project of Fuzhou City"
[2022-P-013]; Science and Technology Project of Anhui Province
" [2021-YF26, 2021-YF27]"
FX This research was funded by the Natural Science Foundation of Fujian
" Province in China (Nos. 2021N0022, 2021Y3002, 2023J02006), the Science"
" and Technology Project of Fuzhou City (No.2022-P-013), and the Science"
" and Technology Project of Anhui Province (Nos. 2021-YF26, 2021-YF27)."
"CR Ahmad A, 2021, ENVIRON TECHNOL INNO, V23, DOI 10.1016/j.eti.2021.101734"
" Ahmed MB, 2017, CHEM ENG J, V322, P571, DOI 10.1016/j.cej.2017.04.063"
" Akar ST, 2012, CHEM ENG J, V185, P82, DOI 10.1016/j.cej.2012.01.032"
" Ali M, 2015, WATER RES, V79, P147, DOI 10.1016/j.watres.2015.04.024"
" An Q, 2023, J ENVIRON CHEM ENG, V11, DOI 10.1016/j.jece.2023.109411"
" Begum W, 2022, RSC ADV, V12, P9139, DOI 10.1039/d2ra00378c"
" Cao XF, 2023, J ENVIRON MANAGE, V325, DOI 10.1016/j.jenvman.2022.116577"
" Chen HM, 2023, J HAZARD MATER, V452, DOI 10.1016/j.jhazmat.2023.131176"
" Chen HM, 2020, ENVIRON POLLUT, V263, DOI 10.1016/j.envpol.2020.114483"
" Chen HM, 2019, ENVIRON INT, V127, P395, DOI 10.1016/j.envint.2019.03.068"
" Chen YY, 2023, ENVIRON SCI POLLUT R, V30, P19427, DOI 10.1007/s11356-022-23544-1"
" Du BH, 2023, ENVIRON RES, V231, DOI 10.1016/j.envres.2023.116249"
" El-Naggar A, 2021, ENVIRON INT, V156, DOI 10.1016/j.envint.2021.106638"
" Fan GD, 2024, SEP PURIF TECHNOL, V338, DOI 10.1016/j.seppur.2024.126522"
" Fan GD, 2023, J WATER PROCESS ENG, V56, DOI 10.1016/j.jwpe.2023.104333"
" Feng C, 2023, BIOCHAR, V5, DOI 10.1007/s42773-023-00253-7"
" Gan L, 2018, COLLOID SURFACE B, V172, P526, DOI 10.1016/j.colsurfb.2018.09.008"
" Ge X, 2017, ACS APPL MATER INTER, V9, P13480, DOI 10.1021/acsami.7b01275"
" Ghosh D, 2015, DESALIN WATER TREAT, V53, P413, DOI 10.1080/19443994.2013.846233"
" Harindintwali JD, 2020, ECOTOX ENVIRON SAFE, V204, DOI 10.1016/j.ecoenv.2020.111020"
" He N, 2023, J HAZARD MATER, V445, DOI 10.1016/j.jhazmat.2022.130465"
" Hou DY, 2020, NAT REV EARTH ENV, V1, P366, DOI 10.1038/s43017-020-0061-y"
" Hu BW, 2017, CHEMOSPHERE, V184, P408, DOI 10.1016/j.chemosphere.2017.05.179"
" Huang F, 2020, J CLEAN PROD, V272, DOI 10.1016/j.jclepro.2020.122743"
" Irshad MK, 2020, CHEMOSPHERE, V242, DOI 10.1016/j.chemosphere.2019.125152"
" Ji XW, 2022, SCI TOTAL ENVIRON, V811, DOI 10.1016/j.scitotenv.2021.152112"
" Jiang JG, 2004, J HAZARD MATER, V113, P141, DOI 10.1016/j.jhazmat.2004.05.030"
" Jiang ZC, 2021, ENVIRON POLLUT, V282, DOI 10.1016/j.envpol.2021.117038"
" Li ZT, 2018, J HAZARD MATER, V344, P1, DOI 10.1016/j.jhazmat.2017.09.036"
" Liang J, 2017, ACS SUSTAIN CHEM ENG, V5, P5049, DOI 10.1021/acssuschemeng.7b00434"
" Liu HW, 2010, J ENVIRON SCI-CHINA, V22, P1513, DOI 10.1016/S1001-0742(09)60282-5"
" Liu ZG, 2009, J HAZARD MATER, V167, P933, DOI 10.1016/j.jhazmat.2009.01.085"
" Ma JF, 2011, TRAC-TREND ANAL CHEM, V30, P691, DOI 10.1016/j.trac.2010.12.008"
" Manikandan SK, 2022, CATALYSTS, V12, DOI 10.3390/catal12121495"
" MURPHY J, 1986, CC/AGR BIOL ENVIRON, P16"
" Nandi R, 2017, RES CHEM INTERMEDIAT, V43, P1619, DOI 10.1007/s11164-016-2719-0"
" Nemati K, 2011, J HAZARD MATER, V192, P402, DOI 10.1016/j.jhazmat.2011.05.039"
" Nemerow N.L., 1974, Scientific Stream Pollution Analysis"
" Oh SY, 2016, BIORESOURCE TECHNOL, V200, P891, DOI 10.1016/j.biortech.2015.11.021"
" Park JH, 2011, J HAZARD MATER, V185, P829, DOI 10.1016/j.jhazmat.2010.09.095"
" Qi X, 2021, J HAZARD MATER, V401, DOI 10.1016/j.jhazmat.2020.123823"
" Qin CC, 2020, CHEMOSPHERE, V261, DOI 10.1016/j.chemosphere.2020.128010"
" Qu JH, 2022, J HAZARD MATER, V423, DOI 10.1016/j.jhazmat.2021.127043"
" Qu JH, 2021, J HAZARD MATER, V401, DOI 10.1016/j.jhazmat.2020.123292"
" Saha R, 2011, J COORD CHEM, V64, P1782, DOI 10.1080/00958972.2011.583646"
" Sarma SJ, 2011, INT BIODETER BIODEGR, V65, P73, DOI 10.1016/j.ibiod.2010.09.004"
" Shao W, 2019, INT J ENV RES PUB HE, V16, DOI 10.3390/ijerph16122172"
" Shen ZT, 2018, CHEMOSPHERE, V190, P260, DOI 10.1016/j.chemosphere.2017.09.140"
" Si SC, 2023, SCI TOTAL ENVIRON, V892, DOI 10.1016/j.scitotenv.2023.164556"
" Song LC, 2021, CHEMOSPHERE, V279, DOI 10.1016/j.chemosphere.2021.130427"
" Tang YY, 2016, RSC ADV, V6, P28579, DOI 10.1039/c6ra00168h"
" Tao KY, 2019, CHEMOSPHERE, V222, P831, DOI 10.1016/j.chemosphere.2019.01.133"
" Tao Y, 2024, SEP PURIF TECHNOL, V330, DOI 10.1016/j.seppur.2023.125392"
" Vu TM, 2017, SCI TOTAL ENVIRON, V579, P612, DOI 10.1016/j.scitotenv.2016.11.050"
" Wang L, 2020, J CLEAN PROD, V258, DOI 10.1016/j.jclepro.2020.120678"
" Wang Q, 2023, ENVIRON SCI POLLUT R, V30, P39154, DOI 10.1007/s11356-022-24952-z"
" Wu G, 2010, J HAZARD MATER, V174, P1, DOI 10.1016/j.jhazmat.2009.09.113"
" Xiang JX, 2021, ENVIRON RES, V195, DOI 10.1016/j.envres.2020.110650"
" Xu R, 2022, J HAZARD MATER, V438, DOI 10.1016/j.jhazmat.2022.129481"
" Xu YJ, 2023, ENVIRON SCI POLLUT R, V30, P101616, DOI 10.1007/s11356-023-29499-1"
" You W, 2024, WATER-SUI, V16, DOI 10.3390/w16121754"
" Zampieri BD, 2016, MICROB ECOL, V72, P582, DOI 10.1007/s00248-016-0821-x"
" Zhang FF, 2023, ENVIRON POLLUT, V330, DOI 10.1016/j.envpol.2023.121681"
" Zhang L, 2019, CHEM ENG J, V356, P151, DOI 10.1016/j.cej.2018.08.224"
" Zhang ML, 2016, CHEMOSPHERE, V154, P215, DOI 10.1016/j.chemosphere.2016.03.103"
" Zhang PZ, 2019, BIORESOURCE TECHNOL, V285, DOI 10.1016/j.biortech.2019.121348"
" Zhang SN, 2024, BIOMASS CONVERS BIOR, V14, P2281, DOI 10.1007/s13399-022-02359-7"
" Zhang Y, 2023, BIOCHAR, V5, DOI 10.1007/s42773-023-00241-x"
" Zhu SH, 2020, J CLEAN PROD, V247, DOI 10.1016/j.jclepro.2019.119579"
NR 69
TC 2
Z9 2
U1 9

U2 63
PU MDPI
PI BASEL
"PA ST ALBAN-ANLAGE 66, CH-4052 BASEL, SWITZERLAND"
EI 2073-4441
J9 WATER-SUI
JI Water
PD JUL
PY 2024
VL 16
IS 13
AR 1917
DI 10.3390/w16131917
PG 18
WC Environmental Sciences; Water Resources
WE Science Citation Index Expanded (SCI-EXPANDED)
SC Environmental Sciences & Ecology; Water Resources
GA YD6T4
UT WOS:001266595000001
"OA Green Submitted, gold"
DA 2026-04-06
ER

PT J
"AU Huang, CC"
"   Liang, CM"
"   Yang, TI"
"   Chen, JL"
"   Wang, WK"
"AF Huang, Chi-Chun"
"   Liang, Chih-Ming"
"   Yang, Ting-, I"
"   Chen, Jiann-Long"
"   Wang, Wei-Kuang"
TI Shift of bacterial communities in heavy metal-contaminated agricultural
   land during a remediation process
SO PLOS ONE
LA English
DT Article
ID 16S RIBOSOMAL-RNA; MICROBIAL COMMUNITIES; SOIL MICROBIOME; HEALTH-RISKS;
   DIVERSITY; SEDIMENTS; ABUNDANCE; PATTERNS; DATABASE; AREAS
"AB Anthropogenic activities accompanied by heavy metal waste threaten the environment. Heavy metal pollution alters the soil microbial community composition, and the microorganisms that adapt to this stress increase in abundance. The remediation process of contaminated soil not only reduces the concentration of heavy metals but also alters the bacterial communities. High-throughput 16S rDNA sequencing techniques were applied to understand the changes in soil microbial communities. Using the remediation approach of the soil mixing, the concentrations of heavy metals in the contaminated areas were diluted and the soil environment was changed. The change of soil environment as a disturbance contributed to the alteration of microbial diversity of the remediated areas. The pH and heavy metals (Cr, Cu, Ni, and Zn) were the most influential factors driving the changes in community structure. The bacterial community structure was significantly different among sample areas. The decrease of heavy metals in soil may be the important factors that changed the microbial composition. This study provides the better understanding of the changes in composition of microbial communities affected by the remediation process in heavy metal-contaminated soil."
"C1 [Huang, Chi-Chun] Endem Species Res Inst, Nantou, Taiwan."
"   [Liang, Chih-Ming; Yang, Ting-, I; Chen, Jiann-Long; Wang, Wei-Kuang] Feng Chia Univ, Dept Environm Engn & Sci, Taichung, Taiwan."
C3 Feng Chia University
"RP Wang, WK (corresponding author), Feng Chia Univ, Dept Environm Engn & Sci, Taichung, Taiwan."
EM wkwang@fcu.edu.tw
"OI Wang, Weikuang/0000-0003-4068-0429; Liang,"
"   Chih-Ming/0000-0002-7818-7253; Huang, chi-chun/0000-0002-6850-1309"
"FU Ministry of Science and Technology, Taiwan [MOST 109-2221-E-035-034]"
FX This work was supported by grants from the Ministry of Science and
"   Technology, Taiwan (MOST 109-2221-E-035-034). The funders had no role in"
"   study design, data collection and analysis, decision to publish, or"
   preparation of the manuscript. Also no authors received a salary from
   the funder.
"CR Ager D, 2010, ENVIRON MICROBIOL, V12, P670, DOI 10.1111/j.1462-2920.2009.02107.x"
"   Ali H, 2019, J CHEM-NY, V2019, DOI 10.1155/2019/6730305"
"   Anderson MJ, 2001, AUSTRAL ECOL, V26, P32, DOI 10.1046/j.1442-9993.2001.01070.x"
"   [Anonymous], 2011, Prescrire Int, V20, P43"
"   [Anonymous], 1991, MONOGRAPH, V17, P1"
"   Azarbad H, 2015, FEMS MICROBIOL ECOL, V91, DOI 10.1093/femsec/fiu003"
"   B????th E, 1998, APPL ENVIRON MICROB, V64, P238"
"   Balaban N, 2019, WATER-SUI, V11, DOI 10.3390/w11102128"
"   Berg J, 2012, APPL ENVIRON MICROB, V78, P7438, DOI 10.1128/AEM.01071-12"
"   Bourceret A, 2016, MICROB ECOL, V71, P711, DOI 10.1007/s00248-015-0682-8"
"   Caporaso JG, 2011, P NATL ACAD SCI USA, V108, P4516, DOI 10.1073/pnas.1000080107"
"   Chellaiah ER, 2018, APPL WATER SCI, V8, DOI 10.1007/s13201-018-0796-5"
"   Cheng BY, 2013, PADDY WATER ENVIRON, V11, P343, DOI 10.1007/s10333-012-0325-3"
"   Cole JR, 2014, NUCLEIC ACIDS RES, V42, pD633, DOI 10.1093/nar/gkt1244"
"   Cui H, 2018, ECOTOX ENVIRON SAFE, V166, P259, DOI 10.1016/j.ecoenv.2018.09.096"
"   Das S, 2013, GEOMICROBIOL J, V30, P635, DOI 10.1080/01490451.2012.746407"
"   David M, 2016, 3 BIOTECH, V6, DOI 10.1007/s13205-016-0475-x"

"   Delgado-Baquerizo M, 2018, SCIENCE, V359, P320, DOI 10.1126/science.aap9516"
"   DeSantis TZ, 2006, APPL ENVIRON MICROB, V72, P5069, DOI 10.1128/AEM.03006-05"
"   Dubey A, 2019, BIODIVERS CONSERV, V28, P2405, DOI 10.1007/s10531-019-01760-5"
"   Fatimawali, 2020, INT J MICROBIOL, V2020, DOI 10.1155/2020/1858732"
"   Feng G, 2018, BMC MICROBIOL, V18, DOI 10.1186/s12866-018-1152-5"
"   Fernandes CC, 2018, BRAZ J MICROBIOL, V49, P489"
"   Frossard A, 2017, SOIL BIOL BIOCHEM, V105, P162, DOI 10.1016/j.soilbio.2016.11.016"
"   Giller KE, 1998, SOIL BIOL BIOCHEM, V30, P1389, DOI 10.1016/S0038-0717(97)00270-8"
"   Golebiewski M, 2014, MICROB ECOL, V67, P635, DOI 10.1007/s00248-013-0344-7"
"   Harpole WS, 2007, NATURE, V446, P791, DOI 10.1038/nature05684"
"   Hemme CL, 2010, ISME J, V4, P660, DOI 10.1038/ismej.2009.154"
"   Hsu MJ, 2006, ENVIRON POLLUT, V143, P327, DOI 10.1016/j.envpol.2005.11.023"
"   Tran HT, 2019, BMC GENOMICS, V20, DOI 10.1186/s12864-019-5875-y"
"   Igiri BE, 2018, J TOXICOL-US, V2018, DOI 10.1155/2018/2568038"
"   Janssen PH, 2006, APPL ENVIRON MICROB, V72, P1719, DOI 10.1128/AEM.72.3.1719-1728.2006"
"   Khudur LS, 2018, ENVIRON POLLUT, V243, P94, DOI 10.1016/j.envpol.2018.08.040"
"   Li XQ, 2017, ENVIRON POLLUT, V231, P908, DOI 10.1016/j.envpol.2017.08.057"
"   McBride M, 1997, EUR J SOIL SCI, V48, P337, DOI 10.1111/j.1365-2389.1997.tb00554.x"
"   Rai PK, 2019, ENVIRON INT, V125, P365, DOI 10.1016/j.envint.2019.01.067"
"   Remen??r M, 2017, BIOLOGIA, V72, P971, DOI 10.1515/biolog-2017-0117"
"   Rognes T, 2016, PEERJ, V4, DOI 10.7717/peerj.2584"
"   Rousk J, 2010, ISME J, V4, P1340, DOI 10.1038/ismej.2010.58"
"   Sandrin TR, 2003, ENVIRON HEALTH PERSP, V111, P1093, DOI 10.1289/ehp.5840"
"   Schloss PD, 2016, MBIO, V7, DOI 10.1128/mBio.00201-16"
"   Song JW, 2018, ENVIRON POLLUT, V243, P510, DOI 10.1016/j.envpol.2018.09.011"
"   Song L, 2020, PEERJ, V8, DOI 10.7717/peerj.10302"
"   Spain AM, 2009, ISME J, V3, P992, DOI 10.1038/ismej.2009.43"
"   Srinivasan S, 2012, PLOS ONE, V7, DOI 10.1371/journal.pone.0037818"
"   Tao JM, 2017, APPL MICROBIOL BIOT, V101, P1289, DOI 10.1007/s00253-016-7938-1"
"   Violante A, 2010, J SOIL SCI PLANT NUT, V10, P268, DOI 10.4067/S0718-95162010000100005"
"   Wang YL, 2020, SCI REP-UK, V10, DOI [10.1038/s41598-020-60384-w, 10.1038/s41598-020-67389-5]"
"   Wu WC, 2017, SCI TOTAL ENVIRON, V601, P57, DOI 10.1016/j.scitotenv.2017.05.165"
"   Wu ZX, 2018, APPL SOIL ECOL, V125, P192, DOI 10.1016/j.apsoil.2018.01.011"
"   Xiao SQ, 2019, FRONT MICROBIOL, V10, DOI 10.3389/fmicb.2019.01867"
"   Xiao SN, 2019, NAT COMMUN, V10, DOI 10.1038/s41467-019-09509-y"
"   Xiong JB, 2014, APPL ENVIRON MICROB, V80, P1919, DOI 10.1128/AEM.03731-13"
"   Xiong JB, 2012, ENVIRON MICROBIOL, V14, P2457, DOI 10.1111/j.1462-2920.2012.02799.x"
"   YANG HY, 2020, SUSTAINABILITY-BASEL, V12, DOI DOI 10.3390/SU122310066"
"   Yang Y, 2018, ENVIRON POLLUT, V238, P263, DOI 10.1016/j.envpol.2018.02.059"
"   Zeng QC, 2019, SCI TOTAL ENVIRON, V680, P124, DOI 10.1016/j.scitotenv.2019.04.184"
"   Zeng XY, 2020, SCI TOTAL ENVIRON, V723, DOI 10.1016/j.scitotenv.2020.138081"
"   Zhang GL, 2017, CHEMOSPHERE, V184, P278, DOI 10.1016/j.chemosphere.2017.05.155"
"   Zhang XM, 2018, ENVIRON MICROBIOL, V20, P3862, DOI 10.1111/1462-2920.14407"
"   2018, AM J GASTROENTEROL, V113"
NR 61
TC 41
Z9 43
U1 10
U2 96
PU PUBLIC LIBRARY SCIENCE
PI SAN FRANCISCO
"PA 1160 BATTERY STREET, STE 100, SAN FRANCISCO, CA 94111 USA"
SN 1932-6203
J9 PLOS ONE
JI PLoS One
PD JUL 23
PY 2021
VL 16
IS 7
AR e0255137
DI 10.1371/journal.pone.0255137
PG 17
WC Multidisciplinary Sciences
WE Science Citation Index Expanded (SCI-EXPANDED)
SC Science & Technology - Other Topics
GA TQ2PW
UT WOS:000678128300010
PM 34297781
"OA Green Submitted, gold"
DA 2026-04-06
ER

PT J
"AU Emenike, CU"
"   Jayanthi, B"
"   Agamuthu, P"
"   Fauziah, SH"
"AF Emenike, C. U."
"   Jayanthi, B."
"   Agamuthu, P."
"   Fauziah, S. H."
TI Biotransformation and removal of heavy metals: a review of
   phytoremediation and microbial remediation assessment on contaminated
   soil

SO ENVIRONMENTAL REVIEWS
LA English
DT Review
DE bioremediation; heavy metals; phytoremediation; pollution; soil
ID TOXIC METALS; ZINC; LEAD; BIOREMEDIATION; CADMIUM; POLLUTION; IMPACT;
COPPER; PLANTS; WASTE
"AB Environmental deterioration is caused by a variety of pollutants; however, heavy metals are often a major issue. Development and globalization has now also resulted in such pollution occurring in developing societies, including Africa and Asia. This review explores the geographical outlook of soil pollution with heavy metals. Various approaches used to remedy metal-polluted soils include physical, chemical, and biological systems, but many of these methods are not economically viable, and they do not ensure restoration without residual effects. This review evaluates the diverse use of plants and microbes in biotransformation and removal of heavy metals from contaminated soil. Mechanisms on how natural processes utilizing plants (phytoremediation) and microorganisms (bioremediation) remove or reduce heavy metals from soil at various levels are presented. This review concludes that remediation technologies are necessary for the recovery of metal-contaminated environments and the prevention of continuous environmentally toxic impacts on living organisms."
"C1 [Emenike, C. U.; Jayanthi, B.; Agamuthu, P.; Fauziah, S. H.] Univ Malaya, Inst Biol Sci, Kuala Lumpur 50603, Malaysia."
" [Emenike, C. U.; Agamuthu, P.; Fauziah, S. H.] Univ Malaya, Inst Res Management & Monitoring, Ctr Res Waste Management, Kuala Lumpur 50603, Malaysia."
" [Emenike, C. U.] Hezekiah Univ, Fac Sci, Umudi, Imo State, Nigeria."
C3 Universiti Malaya; Universiti Malaya
"RP Emenike, CU (corresponding author), Univ Malaya, Inst Biol Sci, Kuala Lumpur 50603, Malaysia.; Emenike, CU (corresponding author), Univ Malaya, Inst Res Management & Monitoring, Ctr Res Waste Management, Kuala Lumpur 50603, Malaysia."
EM emenike@um.edu.my
"RI BARASARATHI, JAYANTHI/AAK-4329-2020; Shahul Hamid, Fauziah/B-5213-2010;"
" Emenike, Chijioke/E-7615-2015"
"OI Shahul Hamid, Fauziah/0000-0002-4083-0602; Emenike,"
Chijioke/0000-0003-4306-0049
FU University of Malaya Research Grant [RP011A-14SUS]; Centre of Research
Grant Management [PG070-2014B]; Ministry of Higher Education Malaysia
(MyBrain15-MyPhD)
"FX Appreciation goes to University of Malaya Research Grant (RP011A-14SUS),"
" Centre of Research Grant Management (PG070-2014B), and Ministry of"
Higher Education Malaysia (MyBrain15-MyPhD) for providing funding.
"CR Abioye P.O., 2011, BIOLOGICAL REMEDIATI, DOI [10.5772/24938, DOI 10.5772/24938]"
" Adriano DC, 2004, GEODERMA, V122, P121, DOI 10.1016/j.geoderma.2004.01.003"
" Adriano DC, 2001, TRACE ELEMENTS TERRE, V2, DOI DOI 10.1007/978-0-387-21510-5"
" Afal A, 2014, METAL TOXICITY"
" Agency for Toxic Substances and Disease Registry (ATSDR), 2003, MERC EXP 3M TART BRA"
" Agency for Toxic Substances and Disease Registry (ATSDR), 1990, TOX PROF SILV"
" Agency for Toxic Substances and Disease Registry (ATSDR), 2008, TOX PROF CHROM DRAFT"
" Ahemad M, 2014, FOLIA MICROBIOL, V59, P321, DOI 10.1007/s12223-014-0304-8"
" Ainza C., 2010, Water Air Soil Pollution, V205, P47, DOI DOI 10.1007/S11270-007-9523-9"
" Al-Sulaimani H., 2010, P SPE EOR C OIL GAS, P378"
" Alloway B. J., 1997, WASTES THEIR DISPOSA, P353"
" [Anonymous], 2000, Phytoremediation of toxic metals: using plants to clean up the environment"
" Ashraf MA, 2012, CHEM SPEC BIOAVAILAB, V24, P1, DOI 10.3184/095422912X13259575370081"
" Atagana HI, 2008, AFR J BIOTECHNOL, V7, P1516"
" Atanasov I., 1993, S MIT OST STAAT UND, P63"
" ATLAS RM, 1981, MICROBIOL REV, V45, P180, DOI 10.1128/MMBR.45.1.180-209.1981"
" BAATH E, 1989, WATER AIR SOIL POLL, V47, P335, DOI 10.1007/BF00279331"
" Baker AJM, 2000, PHYTOREMEDIATION OF CONTAMINATED SOIL AND WATER, P85"
" Ba??uelos GS, 2000, PHYTOREMEDIATION OF CONTAMINATED SOIL AND WATER, P41"
" Basu S., 2014, International Journal of Bioscience, Biochemistry and Bioinformatics, V4, P7, DOI [DOI 10.7763/IJBBB.2014.V4.300, 10.7763/IJBBB.2014.V4.300]"
" Bhattacharya S, 2012, TOXICOL ENVIRON CHEM, V94, P429, DOI 10.1080/02772248.2012.657200"
" Bouchez T, 2000, ENVIRON MICROBIOL, V2, P179"
" Brus DJ, 2009, J ENVIRON QUAL, V38, P1126, DOI 10.2134/jeq2008.0228"
" Bundy JG, 2002, J APPL MICROBIOL, V92, P276, DOI 10.1046/j.1365-2672.2002.01528.x"
" Butow E., 1989, APPROACH MODEL TRANS, P183"
" Castro-Gonz??lez MI, 2008, ENVIRON TOXICOL PHAR, V26, P263, DOI 10.1016/j.etap.2008.06.001"
" CHANDER K, 1995, SOIL BIOL BIOCHEM, V27, P1409, DOI 10.1016/0038-0717(95)00074-O"
" Chao S., 2014, ENV SKEPTICS CRITICS, V3, P24, DOI DOI 10.1037/A0036071"
" Chaperon S, 2008, ECOTOX ENVIRON SAFE, V70, P1, DOI 10.1016/j.ecoenv.2007.10.026"
" Chen Bao Chen Bao, 2011, Journal of Environmental Protection, V2, P130, DOI 10.4236/jep.2011.22015"
" CHEN ZS, 1992, ADV TR SUB, P85"
" Chen ZuengSang Chen ZuengSang, 1997, Remediation of soils contaminated with metals. Proceedings of a conference on the biogeochemistry of trace elements, Taipei, Taiwan, 5-10 September 1993., P209"
" Cheng SP, 2003, ENVIRON SCI POLLUT R, V10, P192, DOI 10.1065/espr2002.11.141.1"
" Chibuike G.U., 2014, APPL ENVIRON SOIL SC, DOI [10.1155/2014/752708, DOI 10.1155/2014/752708]"
" Chou C.-H., 2007, Toxicological profile for arsenic"
" Dadrasnia A, 2015, PETROL SCI TECHNOL, V33, P1030, DOI 10.1080/10916466.2014.925920"
" Das N, 2008, INDIAN J BIOTECHNOL, V7, P159"
" DEGRAEVE N, 1981, MUTAT RES, V86, P115, DOI 10.1016/0165-1110(81)90035-X"
" Dewan S., 2009, NY TIMES"
" DIBBLE JT, 1979, APPL ENVIRON MICROB, V37, P729, DOI 10.1128/AEM.37.4.729-739.1979"
" Diels L, 1999, MOL BIOTECHNOL, V12, P149, DOI 10.1385/MB:12:2:149"
" Dinev N., 2008, General and Applied Plant Physiology, V34, P389"
" Dinev N., 1998, Soil Science, Agro Chemical Ecology, V4, P3"
" Duda-Chodak A, 2008, J ELEM, V13, P685"
" Duruibe JO, 2007, INT J PHYS SCI, V2, P112"

"   Ebbs SD, 1997, J ENVIRON QUAL, V26, P1424, DOI 10.2134/jeq1997.00472425002600050032x"
"   Emenike CU, 2017, J GEOCHEM EXPLOR, V182, P275, DOI 10.1016/j.gexplo.2016.10.002"
"   Emenike CU, 2016, ENVIRON EARTH SCI, V75, DOI 10.1007/s12665-015-4805-9"
"   Emenike C. U., 2014, THESIS"
"   EPA, 2014, MUN SOL WAST LANDF"
"   Farag, 2000, ICEHM2000, P542"
"   Fauziah SH, 2017, ENVIRON EARTH SCI, V76, DOI 10.1007/s12665-017-6739-x"
"   Fletcher T, 2002, LAND USE POLICY, V19, P311, DOI 10.1016/S0264-8377(02)00045-5"
"   Forsyth B, 1995, SUDBURY '95 - MINING AND THE ENVIRONMENT, CONFERENCE PROCEEDINGS, VOLS 1-3, P795"
"   Fulekar MH, 2012, ENVIRON MONIT ASSESS, V184, P7299, DOI 10.1007/s10661-011-2499-3"
"   Garbisu C., 1997, J CLEAN TECHNOL E T, V6, P1"
"   Garbisu C., 2003, The European Journal of Mineral Processing and Environmental Protection, V3, P58"
"   Gulati K, 2010, INDIAN J EXP BIOL, V48, P710"
"   HARTER RD, 1983, SOIL SCI SOC AM J, V47, P47, DOI 10.2136/sssaj1983.03615995004700010009x"
"   Hem JD, 1989, 2254 US GEOL SURV"
"   Hess R., 2002, Journal of Pediatric Hematology/Oncology, V24, P582, DOI DOI 10.1097/00043426-200210000-00020"
"   Hooda V, 2007, J ENVIRON BIOL, V28, P367"
"   Hookoom M., 2013, Current Research in Microbiology and Biotechnology, V1, P119"
"   Hseu ZY, 2010, SOIL SCI PLANT NUTR, V56, P31, DOI 10.1111/j.1747-0765.2009.00442.x"
"   ITRC (Interstate Technology and Regulatory Council), 2009, Phyto-3"
"   Jacobs IA, 2002, AM J IND MED, V41, P285, DOI 10.1002/ajim.10056"
"   Jadia CD, 2009, AFR J BIOTECHNOL, V8, P921"
"   J??rup L, 2003, BRIT MED BULL, V68, P167, DOI 10.1093/bmb/ldg032"
"   Jiang W, 2008, ANN NY ACAD SCI, V1140, P446, DOI 10.1196/annals.1454.050"
"   Jo IS, 2004, ENVIRON GEOCHEM HLTH, V26, P105, DOI 10.1023/B:EGAH.0000039573.05245.cc"
"   Jugaryan O. A., 2000, ECOTOXICOLOGY TECHNO, P1"
"   Kanmani P, 2012, INT J ENVIRON SCI TE, V9, P183, DOI 10.1007/s13762-011-0013-7"
"   Keller BEM, 1998, WETLANDS, V18, P42, DOI 10.1007/BF03161441"
"   Khan MA, 2007, J CHIN CHEM SOC-TAIP, V54, P339, DOI 10.1002/jccs.200700049"
"   Khan S, 2008, ENVIRON POLLUT, V152, P686, DOI 10.1016/j.envpol.2007.06.056"
"   Knopf B., 2010, SOIL BIOL, V19"
"   Knox A. S, 2000, REMEDIATION METALS R, P21"
"   Krishna AK, 2009, J HAZARD MATER, V167, P366, DOI 10.1016/j.jhazmat.2008.12.131"
"   Kumar V., 2013, ROBBINS BASIC PATHOL, V9th"
"   Lai H. Y., 2009, TECHNICAL B FOOD FER, P1"
"   Landberg T, 1996, APPL GEOCHEM, V11, P175, DOI 10.1016/0883-2927(95)00082-8"
"   LEAHY JG, 1990, MICROBIOL REV, V54, P305, DOI 10.1128/MMBR.54.3.305-315.1990"
"   Lejeune K, 1996, ECOTOXICOLOGY, V5, P297, DOI 10.1007/BF00119052"
"   Lenntech, 2004, HEAV MET, P1"
"   Li XD, 2004, ENVIRON POLLUT, V129, P113, DOI 10.1016/j.envpol.2003.09.030"
"   Liao SW, 2004, J AQUAT PLANT MANAGE, V42, P60"
"   Ling T Y., 2007, Journal of Engineering Sciences, V3, P51"
"   Liu WX, 2009, J HAZARD MATER, V161, P479, DOI 10.1016/j.jhazmat.2008.03.123"
"   Loganathan P, 2008, REV ENVIRON CONTAM T, V192, P29, DOI 10.1007/978-0-387-71724-1_2"
"   LOVLEY DR, 1993, APPL ENVIRON MICROB, V59, P3572, DOI 10.1128/AEM.59.11.3572-3576.1993"
"   Marques APGC, 2009, J HAZARD MATER, V165, P174, DOI 10.1016/j.jhazmat.2008.09.102"
"   Mathew A. M., 2005, THESIS"
"   McKeehan P., 2000, The financial, legislative and social aspects of the redevelopment of contaminated commercial and industrial properties"
"   McLaughlin MJ, 2000, COMMUN SOIL SCI PLAN, V31, P1661, DOI 10.1080/00103620009370531"
"   Ming-Ho Y., 2005, Environmental toxicology: Biological and health effects of pollutants"
"   M??ller A, 2005, GEODERMA, V124, P63, DOI 10.1016/j.geoderma.2004.04.003"
"   Mrozik A, 2010, MICROBIOL RES, V165, P363, DOI 10.1016/j.micres.2009.08.001"
"   Nanda S, 2011, AFR J BIOTECHNOL, V10, P11948"
"   Neustadt J., 2007, Journal of Integrative Medicine, V6, P36"
"   Norra S, 2005, APPL GEOCHEM, V20, P1890, DOI 10.1016/j.apgeochem.2005.04.019"
"   NORVELL WA, 1984, SOIL SCI SOC AM J, V48, P1285, DOI 10.2136/sssaj1984.03615995004800060017x"
"   Nouri J, 2009, ENVIRON EARTH SCI, V59, P315, DOI 10.1007/s12665-009-0028-2"
"   Nriagu J. O., 1978, BIOGEOCHEMISTRY LEAD, VII"
"   Nriagu J. O., 1978, BIOGEOCHEMISTRY LEAD, VI"
"   NRIAGU JO, 1988, NATURE, V333, P134, DOI 10.1038/333134a0"
"   NWANKWO JN, 1979, B ENVIRON CONTAM TOX, V22, P625, DOI 10.1007/BF02026998"
"   Nyamangara J, 1998, AGR ECOSYST ENVIRON, V69, P135, DOI 10.1016/S0167-8809(98)00101-7"
"   Ogunkunle C O., 2013, INT J APPL SCI TECHN, V3"
"   Oyeku O. T., 2010, African Journal of Environmental Science and Technology, V4, P201"
"   Paz-Alberto A.M., 2013, American Journal of Climate Change, V02, P71, DOI DOI 10.4236/AJCC.2013.21008"
"   Paz-Ferreiro J, 2014, J SOIL SEDIMENT, V14, P483, DOI 10.1007/s11368-013-0806-z"
"   Phoenix Satellite TV, 2014, SHE HUI NEN JIAN DU"
"   Pilon-Smits E, 2005, ANNU REV PLANT BIOL, V56, P15, DOI 10.1146/annurev.arplant.56.032604.144214"
"   Poovey B., 2001, BLOOMBERG BUSINESS W"
"   Prokisch J, 2009, COMMUN SOIL SCI PLAN, V40, P391, DOI 10.1080/00103620802649039"
"   Puranik PR, 1997, J BIOTECHNOL, V55, P113, DOI 10.1016/S0168-1656(97)00067-9"
"   Ragnarsdottir KV, 2005, GEOCHIM COSMOCHIM AC, V69, pA196"
"   RAMOS L, 1994, J ENVIRON QUAL, V23, P50, DOI 10.2134/jeq1994.00472425002300010009x"
"   Ravindra K, 2008, ATMOS ENVIRON, V42, P2895, DOI 10.1016/j.atmosenv.2007.12.010"
"   Reeves RD, 2003, PLANT SOIL, V249, P57, DOI 10.1023/A:1022572517197"
"   Rodr??guez L, 2009, J ENVIRON MANAGE, V90, P1106, DOI 10.1016/j.jenvman.2008.04.007"
"   Rosselli W, 2003, PLANT SOIL, V256, P265, DOI 10.1023/A:1026100707797"
"   Roy M, 2015, LAND DEGRAD DEV, V26, P785, DOI 10.1002/ldr.2237"
"   Salonen VP, 2007, APPL GEOCHEM, V22, P906, DOI 10.1016/j.apgeochem.2007.02.003"
"   Sayadi M. H., 2014, Proceedings of the International Academy of Ecology and Environmental Sciences, V4, P18"
"   Schm??ger MEV, 2000, PLANT PHYSIOL, V122, P793, DOI 10.1104/pp.122.3.793"

"   Servilia O., 2005, Not. Bio. Sci, P107"
"   Soltan E. S., 2001, P STUTZERI BIOMETALS, V7, P30"
"   Stockwell L. E., 2001, WORLD MINERAL STAT 1"
"   Syakalima M, 2001, J VET MED SCI, V63, P315, DOI 10.1292/jvms.63.315"
"   Sydnor MEW, 2002, J ENVIRON QUAL, V31, P1528, DOI 10.2134/jeq2002.1528"
"   Taiz L., 2006, PLANT PHYSIOL, V4th"
"   Tchounwou P., 2012, MOL CLIN ENV TOXICOL, V101, P133, DOI DOI 10.1007/978-3-7643-8340-4"
"   Thompson IP, 2005, ENVIRON MICROBIOL, V7, P909, DOI 10.1111/j.1462-2920.2005.00804.x"
"   Tomoyuki M., 2007, HEAVY METAL POLLUTIO"
"   Tripathi RD, 2007, TRENDS BIOTECHNOL, V25, P158, DOI 10.1016/j.tibtech.2007.02.003"
"   Tunali S, 2006, CHEM ENG J, V115, P203, DOI 10.1016/j.cej.2005.09.023"
"   TYLER G, 1989, WATER AIR SOIL POLL, V47, P189, DOI 10.1007/BF00279327"
"   U.S. Department of Labor, 2014, SAF HLTH TOP TOX MET"
"   United States Environmental Protection Agency (US EPA), 1997, CLEAN NAT HAZ WAST S"
"   Uslu G, 2006, J HAZARD MATER, V135, P87, DOI 10.1016/j.jhazmat.2005.11.029"
"   Vacca A, 2012, LAND DEGRAD DEV, V23, P350, DOI 10.1002/ldr.2153"
"   VANASSCHE F, 1990, PLANT CELL ENVIRON, V13, P195, DOI 10.1111/j.1365-3040.1990.tb01304.x"
"   vanVeen JA, 1997, MICROBIOL MOL BIOL R, V61, P121, DOI 10.1128/.61.2.121-135.1997"
"   Vinceti M, 2001, Rev Environ Health, V16, P233"
"   WOOD JM, 1974, SCIENCE, V183, P1049, DOI 10.1126/science.183.4129.1049"
"   Wuana R.A., 2011, ISRN Ecology, DOI [10.5402/2011/402647, DOI 10.5402/2011/402647]"
"   Xie Y, 2016, FRONT PLANT SCI, V7, DOI 10.3389/fpls.2016.00755"
"   Yabe J, 2010, J VET MED SCI, V72, P1257, DOI 10.1292/jvms.10-0058"
"   Yang LS, 2012, BIOL TRACE ELEM RES, V145, P81, DOI 10.1007/s12011-011-9159-x"
"   Yang YB, 2009, ENV PPROTECTION SCI, V35, P79"
"   Yao ZT, 2012, PROCEDIA ENVIRON SCI, V16, P722, DOI 10.1016/j.proenv.2012.10.099"
"   Yong-ming L.U. O., 2006, Soil Science, V38, P505"
"   Yoon J, 2006, SCI TOTAL ENVIRON, V368, P456, DOI 10.1016/j.scitotenv.2006.01.016"
"   Zaier H, 2010, ANAL CHIM ACTA, V671, P48, DOI 10.1016/j.aca.2010.04.054"
"   Zhang CX, 2010, GEOPHYS J INT, V181, P1381, DOI 10.1111/j.1365-246X.2010.04590.x"
"   Zhao FJ, 2000, PLANT CELL ENVIRON, V23, P507, DOI 10.1046/j.1365-3040.2000.00569.x"
"   Zheng N, 2008, ENVIRON POLLUT, V154, P135, DOI 10.1016/j.envpol.2008.01.001"
NR 158
TC 85
Z9 105
U1 22
U2 520
PU CANADIAN SCIENCE PUBLISHING
PI OTTAWA
"PA 65 AURIGA DR, SUITE 203, OTTAWA, ON K2E 7W6, CANADA"
SN 1208-6053
EI 1181-8700
J9 ENVIRON REV
JI Environ. Rev.
PD JUN
PY 2018
VL 26
IS 2
BP 156
EP 168
DI 10.1139/er-2017-0045
PG 13
WC Environmental Sciences
WE Science Citation Index Expanded (SCI-EXPANDED)
SC Environmental Sciences & Ecology
GA GH0MV
UT WOS:000433097600004
OA Green Submitted
DA 2026-04-06
ER

PT J
"AU Kou, B"
"   He, Y"
"   Wang, Y"
"   Qu, CT"
"   Tang, J"
"   Wu, YM"
"   Tan, WB"
"   Yuan, Y"
"   Yu, TQ"
"AF Kou, Bing"
"   He, Yue"
"   Wang, Yang"
"   Qu, Chengtun"
"   Tang, Jun"
"   Wu, Yuman"
"   Tan, Wenbing"
"   Yuan, Ying"
"   Yu, Tingqiao"
TI The relationships between heavy metals and bacterial communities in a
   coal gangue site
SO ENVIRONMENTAL POLLUTION
LA English

DT Article
DE Gangue site; Bacterial community; Heavy metal; Soil organic matter
ID DISSOLVED ORGANIC-MATTER; SOIL ENZYME-ACTIVITIES; MICROBIAL COMMUNITIES;
MINING AREA; HEALTH-RISK; HUMIC ACIDS; POLLUTION; NITROGEN; BIOMASS;
MINE
"AB Coal is the main source of energy for China's economic development, but coal gangue dumps are a major source of heavy metal pollution. Bacterial communities have a major effect on the bioremediation of heavy metals in coal gangue dumps. The effects of different concentrations of heavy metals on the composition of bacterial communities in coal gangue sites remain unclear. Soil bacterial communities from four gangue sites that vary in natural heavy metal concentrations were investigated using high-throughput sequencing in this study. Corre-lations among bacterial communities, heavy metal concentrations, physicochemical properties of the soil, and the composition of dissolved organic matter of soil in coal gangue dumps were also analyzed. Our results indicated that Actinobacteriota, Proteobacteria, Chloroflexi, Acidobacteriota, and Gemmatimonadota were the bacterial taxa most resistant to heavy metal stress at gangue sites. Heavy metal contamination may be the main cause of changes in bacterial communities. Heavy metal pollution can foster mutually beneficial symbioses between microbial species. Microbial-derived organic matter was the main source of soil organic matter in unvegetated mining areas, and this could affect the toxicity and transport of heavy metals in soil. Polar functional groups such as hydroxyl and ester groups (A226-400) play an important role in the reaction of cadmium (Cd) and lead (Pb), and organic matter with low molecular weight (SR) tends to bind more to mercury (Hg). In addition to heavy metals, the content of nitrogen (N), phosphorus (P), and total organic carbon (TOC) also affected the composition of the bacterial communities; TOC had the strongest effect, followed by N, SOM, and P. Our findings have implications for the microbial remediation of heavy metal-contaminated soils in coal gangue sites and sustainable development."
"C1 [Kou, Bing; Wang, Yang; Tang, Jun; Wu, Yuman; Tan, Wenbing; Yuan, Ying] Chinese Res Inst Environm Sci, State Environm Protect Key Lab Simulat & Control G, Beijing 100012, Peoples R China."
" [Kou, Bing; Qu, Chengtun; Wu, Yuman] Xian Shiyou Univ, Coll Chem & Chem Engn, Xian 710065, Peoples R China."
" [He, Yue] Beijing Guozhong Biotechnol Co LTD, Beijing 102211, Peoples R China."
" [Yu, Tingqiao] Beijing Vocat Coll Agr, Int Educ Coll, Beijing 102442, Peoples R China."
C3 Chinese Research Academy of Environmental Sciences; Xi'an Shiyou
University
"RP Yuan, Y (corresponding author), Chinese Res Inst Environm Sci, State Environm Protect Key Lab Simulat & Control G, Beijing 100012, Peoples R China."
EM yuanying19880214@126.com
"OI Tan, Wenbing/0000-0001-9896-7604"
"FU Central Level, Shandong Provincial Natural Science Foundation, China;"
National Natural Science Foundation of China [2020YSKY-006]; Scientific
Research Institutes for Basic R & D Special Fund Business
[2020CXGC011403]; [42030704]
"FX This work was supported by the Central Level, Shandong Provincial"
" Natural Science Foundation, China (Grant No:2020CXGC011403) and National"
Natural Science Foundation of China (Grant No. 42030704) and Scientific
Research Institutes for Basic R & D Special Fund Business (Grant No:
2020YSKY-006) .
"CR Aeschbacher M, 2012, ENVIRON SCI TECHNOL, V46, P4916, DOI 10.1021/es300039h"
" Ali A, 2021, CHEMOSPHERE, V273, DOI 10.1016/j.chemosphere.2021.129692"
" Azarbad H, 2015, FEMS MICROBIOL ECOL, V91, DOI 10.1093/femsec/fiu003"
" Bai XJ, 2018, ARID LAND RES MANAG, V32, P438, DOI 10.1080/15324982.2018.1501621"
" Bang L, 2022, J HAZARD MATER, V425, DOI 10.1016/j.jhazmat.2021.127945"
" Beattie RE, 2018, SOIL BIOL BIOCHEM, V126, P57, DOI 10.1016/j.soilbio.2018.08.011"
" Belyaeva ON, 2012, ENVIRON EARTH SCI, V66, P1987, DOI 10.1007/s12665-011-1424-y"
" Bhattacharyya SS, 2022, SCI TOTAL ENVIRON, V815, DOI 10.1016/j.scitotenv.2022.152928"
" Bi YL, 2020, CATENA, V195, DOI 10.1016/j.catena.2020.104791"
" Bruneel O, 2019, J GEOCHEM EXPLOR, V205, DOI 10.1016/j.gexplo.2019.06.009"
" Buch AC, 2021, J HAZARD MATER, V403, DOI 10.1016/j.jhazmat.2020.123852"
" Buckeridge KM, 2020, COMMUN EARTH ENVIRON, V1, DOI 10.1038/s43247-020-00031-4"
" Cao CY, 2017, APPL MICROBIOL BIOT, V101, P6217, DOI 10.1007/s00253-017-8336-z"
" Chakraborti P, 2015, J BIOMOL STRUCT DYN, V33, P2330, DOI 10.1080/07391102.2015.1069214"
" Chamnongpol S, 2002, MOL MICROBIOL, V44, P561, DOI 10.1046/j.1365-2958.2002.02917.x"
" Che RX, 2018, SCI TOTAL ENVIRON, V639, P997, DOI 10.1016/j.scitotenv.2018.05.238"
" Chen J, 2020, CATENA, V194, DOI 10.1016/j.catena.2020.104779"
" Chen J, 2020, APPL SOIL ECOL, V147, DOI 10.1016/j.apsoil.2019.103430"
" Childers SE, 2002, NATURE, V416, P767, DOI 10.1038/416767a"
" CHIN YP, 1994, ENVIRON SCI TECHNOL, V28, P1853, DOI 10.1021/es00060a015"
" Chun SJ, 2021, ENVIRON POLLUT, V289, DOI 10.1016/j.envpol.2021.117851"
" Deng SQ, 2018, ENVIRON POLLUT, V237, P1088, DOI 10.1016/j.envpol.2017.11.037"
" Ding HJ, 2016, ENVIRON SCI POLLUT R, V23, P2831, DOI 10.1007/s11356-015-5479-2"
" Dong J, 2011, ENVIRON EARTH SCI, V64, P1317, DOI 10.1007/s12665-011-0992-1"
" Fine P, 2018, WATER AIR SOIL POLL, V229, DOI 10.1007/s11270-017-3627-7"
" Gadd GM, 2010, MICROBIOL-SGM, V156, P609, DOI 10.1099/mic.0.037143-0"
" Gao M, 2022, J ENVIRON MANAGE, V301, DOI 10.1016/j.jenvman.2021.113837"
" Guo J, 2021, APPL SOIL ECOL, V163, DOI 10.1016/j.apsoil.2021.103916"
" GUPTA RK, 1988, CAN J MICROBIOL, V34, P667, DOI 10.1139/m88-110"
" Han C, 2012, BIOCHEM J, V444, P219, DOI 10.1042/BJ20111623"
" Hansen AM, 2016, LIMNOL OCEANOGR, V61, P1015, DOI 10.1002/lno.10270"
" He XS, 2014, J HAZARD MATER, V268, P256, DOI 10.1016/j.jhazmat.2014.01.030"
" He XS, 2011, CHEMOSPHERE, V82, P541, DOI 10.1016/j.chemosphere.2010.10.057"
" Hower JC, 2008, INT J COAL GEOL, V75, P88, DOI 10.1016/j.coal.2008.03.001"
" Hu HY, 2021, ECOL ENG, V169, DOI 10.1016/j.ecoleng.2021.106305"
" Huang JQ, 2021, J HAZARD MATER, V416, DOI 10.1016/j.jhazmat.2021.125942"
" Huang XF, 2021, ECOTOX ENVIRON SAFE, V227, DOI 10.1016/j.ecoenv.2021.112897"
" Jeong H, 2020, ENVIRON POLLUT, V265, DOI 10.1016/j.envpol.2020.115028"
" Jiang CL, 2022, ECOL INDIC, V136, DOI 10.1016/j.ecolind.2022.108683"
" Jose J, 2011, ENVIRON POLLUT, V159, P2775, DOI 10.1016/j.envpol.2011.05.009"

"   Kalbitz K, 1998, SCI TOTAL ENVIRON, V209, P27, DOI 10.1016/S0048-9697(97)00302-1"
"   Kalbitz K, 2000, SOIL SCI, V165, P277, DOI 10.1097/00010694-200004000-00001"
"   Khan K. S., 1998, Pedosphere, V8, P27"
"   Kjoller R, 2012, NEW PHYTOL, V194, P278, DOI 10.1111/j.1469-8137.2011.04041.x"
"   Kleber M, 2010, ADV AGRON, V106, P77, DOI 10.1016/S0065-2113(10)06003-7"
"   Kong LJ, 2022, CHEMOSPHERE, V292, DOI 10.1016/j.chemosphere.2022.133531"
"   Kumar V., 2022, MICROBIAL EXTREMOZYM, P275, DOI DOI 10.1016/B978-0-12-822945-3.00003-8"
"   Li D, 2018, J CLEAN PROD, V185, P959, DOI 10.1016/j.jclepro.2018.02.216"
"   Li JY, 2019, J CLEAN PROD, V231, P1059, DOI 10.1016/j.jclepro.2019.05.230"
"   Li JY, 2021, CHEMOSPHERE, V274, DOI 10.1016/j.chemosphere.2021.129967"
"   Li SZ, 2020, J HAZARD MATER, V400, DOI 10.1016/j.jhazmat.2020.123255"
"   Li XQ, 2017, ENVIRON POLLUT, V231, P908, DOI 10.1016/j.envpol.2017.08.057"
"   Li YH, 2021, J ENVIRON CHEM ENG, V9, DOI 10.1016/j.jece.2021.106823"
"   Li ZY, 2014, SCI TOTAL ENVIRON, V468, P843, DOI 10.1016/j.scitotenv.2013.08.090"
"   Lin H, 2022, J CLEAN PROD, V351, DOI 10.1016/j.jclepro.2022.131569"
"   Liu B, 2021, CHEMOSPHERE, V282, DOI 10.1016/j.chemosphere.2021.130992"
"   Liu Y, 2018, SCI TOTAL ENVIRON, V625, P1218, DOI 10.1016/j.scitotenv.2018.01.003"
"   Lorenz M, 2021, SCI TOTAL ENVIRON, V781, DOI 10.1016/j.scitotenv.2021.146732"
"   Lovley DR, 2011, ENERG ENVIRON SCI, V4, P4896, DOI 10.1039/c1ee02229f"
"   Manoj SR, 2020, J ENVIRON MANAGE, V254, DOI 10.1016/j.jenvman.2019.109779"
"   Mathivanan K, 2021, ECOTOX ENVIRON SAFE, V226, DOI 10.1016/j.ecoenv.2021.112863"
"   Meena R.K., 2021, 2017 IEEE INT C PROG, V100, P1"
"   Moreno-Pino M, 2016, FEMS MICROBIOL ECOL, V92, DOI 10.1093/femsec/fiw088"
"   Ojuederie OB, 2017, INT J ENV RES PUB HE, V14, DOI 10.3390/ijerph14121504"
"   Paniagua-L??pez M, 2021, SCI TOTAL ENVIRON, V777, DOI 10.1016/j.scitotenv.2021.145128"
"   Polak J, 2009, WATER RES, V43, P4167, DOI 10.1016/j.watres.2009.06.040"
"   Raimondi I.M., 2014, THESIS U SAO PAULO"
"   Ratasuk N, 2007, ENVIRON SCI TECHNOL, V41, P7844, DOI 10.1021/es071389u"
"   Rinc??n A, 2015, ENVIRON MICROBIOL, V17, P3009, DOI 10.1111/1462-2920.12894"
"   Shafer MM, 2012, ENVIRON SCI TECHNOL, V46, P189, DOI 10.1021/es200463c"
"   Shajib MTI, 2019, ENVIRON POLLUT, V248, P584, DOI 10.1016/j.envpol.2019.02.039"
"   Su Z, 2022, SCI TOTAL ENVIRON, V825, DOI 10.1016/j.scitotenv.2022.153767"
"   Sun RY, 2010, INT J COAL GEOL, V81, P81, DOI 10.1016/j.coal.2009.12.001"
"   Sun RY, 2010, ENERGY, V35, P423, DOI 10.1016/j.energy.2009.10.009"
"   Sun YB, 2010, J HAZARD MATER, V174, P455, DOI 10.1016/j.jhazmat.2009.09.074"
"   Tan WB, 2017, ENVIRON SCI TECHNOL, V51, P3176, DOI 10.1021/acs.est.6b04131"
"   Tang JY, 2019, J ENVIRON MANAGE, V242, P121, DOI 10.1016/j.jenvman.2019.04.061"
"   Tang Q, 2018, J GEOCHEM EXPLOR, V186, P1, DOI 10.1016/j.gexplo.2017.11.018"
"   Tang Q, 2013, CHEMOSPHERE, V93, P2473, DOI 10.1016/j.chemosphere.2013.09.015"
"   Vick SHW, 2019, ISCIENCE, V12, P152, DOI 10.1016/j.isci.2019.01.011"
"   Vieira C.K., 2021, SCI TOTAL ENVIRON, V802"
"   Walpen N, 2016, ENVIRON SCI TECHNOL, V50, P6423, DOI 10.1021/acs.est.6b01120"
"   Wang JH, 2018, J ENVIRON SCI-CHINA, V71, P283, DOI 10.1016/j.jes.2018.05.031"
"   Wang JM, 2014, ENVIRON EARTH SCI, V71, P863, DOI 10.1007/s12665-013-2488-7"
"   Wang K, 2021, SCI TOTAL ENVIRON, V767, DOI 10.1016/j.scitotenv.2020.144465"
"   Wang X, 2022, SOIL TILL RES, V216, DOI 10.1016/j.still.2021.105241"
"   Wei Z., 2017, PLOS ONE, V12"
"   Wieder WR, 2014, BIOGEOSCIENCES, V11, P3899, DOI 10.5194/bg-11-3899-2014"
"   Wu BH, 2022, SCI TOTAL ENVIRON, V802, DOI 10.1016/j.scitotenv.2021.149899"
"   Wu F, 2020, J HAZARD MATER, V390, DOI 10.1016/j.jhazmat.2020.122144"
"   Wu LQ, 2020, ENVIRON POLLUT, V265, DOI 10.1016/j.envpol.2020.114998"
"   Wu YJ, 2022, SCI TOTAL ENVIRON, V805, DOI 10.1016/j.scitotenv.2021.150400"
"   Xia X, 2021, J HAZARD MATER, V401, DOI [10.1016/j.jhazmat.2020.143685, 10.1016/j.jhazmat.2020.123685]"
"   Xiao L, 2019, CATENA, V177, P202, DOI 10.1016/j.catena.2019.02.019"
"   Xu LX, 2019, APPL SOIL ECOL, V135, P157, DOI 10.1016/j.apsoil.2018.12.004"
"   Xue Qiang, 2011, Front Biosci (Elite Ed), V3, P830"
"   Yan CC, 2020, SCI TOTAL ENVIRON, V707, DOI 10.1016/j.scitotenv.2019.136116"
"   Ye J, 2017, ISME J, V11, P1087, DOI 10.1038/ismej.2016.187"
"   Yin Y, 2023, J HAZARD MATER, V442, DOI 10.1016/j.jhazmat.2022.130063"
"   Yuan Y, 2017, J HAZARD MATER, V339, P378, DOI 10.1016/j.jhazmat.2017.06.047"
"   Zeng QC, 2019, CATENA, V183, DOI 10.1016/j.catena.2019.104220"
"   Zhang HW, 2021, ENVIRON RES, V202, DOI 10.1016/j.envres.2021.111702"
"   Zhang HN, 2020, BIORESOURCE TECHNOL, V295, DOI 10.1016/j.biortech.2019.122302"
"   Zhang J, 2015, SCI TOTAL ENVIRON, V508, P29, DOI 10.1016/j.scitotenv.2014.11.064"
"   Zhang L, 2018, ENVIRON SCI POLLUT R, V25, P8475, DOI 10.1007/s11356-017-1169-6"
"   Zhang MZ, 2022, J HAZARD MATER, V431, DOI 10.1016/j.jhazmat.2022.128456"
"   Zhang SZ, 2016, HUM ECOL RISK ASSESS, V22, P1469, DOI 10.1080/10807039.2016.1185689"
"   Zhang XQ, 2022, ENVIRON POLLUT, V299, DOI 10.1016/j.envpol.2022.118834"
"   Zhang XW, 2012, ENVIRON MONIT ASSESS, V184, P2261, DOI 10.1007/s10661-011-2115-6"
"   Zhang Y, 2013, J SOIL SCI PLANT NUT, V13, P43, DOI 10.4067/S0718-95162013005000005"
"   Zhao QQ, 2020, APPL SOIL ECOL, V153, DOI 10.1016/j.apsoil.2020.103581"
"   Zhao XY, 2018, BIORESOURCE TECHNOL, V267, P378, DOI 10.1016/j.biortech.2018.07.060"
"   Zhou Y, 2015, PLANT SOIL, V386, P171, DOI 10.1007/s11104-014-2254-y"
NR 113
TC 76
Z9 80
U1 37
U2 366
PU ELSEVIER SCI LTD
PI London
"PA 125 London Wall, London, the United Kingdom"
SN 0269-7491
EI 1873-6424
J9 ENVIRON POLLUT

JI Environ. Pollut.
PD APR 1
PY 2023
VL 322
AR 121136
DI 10.1016/j.envpol.2023.121136
EA FEB 2023
PG 12
WC Environmental Sciences
WE Science Citation Index Expanded (SCI-EXPANDED)
SC Environmental Sciences & Ecology
GA 8Y2AN
UT WOS:000932502700001
PM 36736561
DA 2026-04-06
ER

PT J
"AU Raffa, CM"
"   Chiampo, F"
"   Shanthakumar, S"
"AF Raffa, Carla Maria"
"   Chiampo, Fulvia"
"   Shanthakumar, Subramanian"
TI Remediation of Metal/Metalloid-Polluted Soils: A Short Review
SO APPLIED SCIENCES-BASEL
LA English
DT Review
DE heavy metals and metalloids; removal techniques; contaminated soil
ID HEAVY-METAL POLLUTION; IN-SITU REMEDIATION; ZERO-VALENT IRON;
   CONTAMINATED SOIL; HEXAVALENT CHROMIUM; AGRICULTURAL SOILS;
   RISK-ASSESSMENT; ELECTROKINETIC REMEDIATION; SPATIAL-DISTRIBUTION;
   COPPER TOXICITY
"AB The contamination of soil by heavy metals and metalloids is a worldwide problem due to the accumulation of these compounds in the environment, endangering human health, plants, and animals. Heavy metals and metalloids are normally present in nature, but the rise of industrialization has led to concentrations higher than the admissible ones. They are non-biodegradable and toxic, even at very low concentrations. Residues accumulate in living beings and become dangerous every time they are assimilated and stored faster than they are metabolized. Thus, the potentially harmful effects are due to persistence in the environment, bioaccumulation in the organisms, and toxicity. The severity of the effect depends on the type of heavy metal or metalloid. Indeed, some heavy metals (e.g., Mn, Fe, Co, Ni) at very low concentrations are essential for living organisms, while others (e.g., Cd, Pb, and Hg) are nonessential and are toxic even in trace amounts. It is important to monitor the concentration of heavy metals and metalloids in the environment and adopt methods to remove them. For this purpose, various techniques have been developed over the years: physical remediation (e.g., washing, thermal desorption, solidification), chemical remediation (e.g., adsorption, catalysis, precipitation/solubilization, electrokinetic methods), biological remediation (e.g., biodegradation, phytoremediation, bioventing), and combined remediation (e.g., electrokinetic-microbial remediation; washing-microbial degradation). Some of these are well known and used on a large scale, while others are still at the research level. The main evaluation factors for the choice are contaminated site geology, contamination characteristics, cost, feasibility, and sustainability of the applied process, as well as the technology readiness level. This review aims to give a picture of the main techniques of heavy metal removal, also giving elements to assess their potential hazardousness due to their concentrations."
"C1 [Raffa, Carla Maria; Chiampo, Fulvia] Politecn Torino, Dept Appl Sci & Technol, Corso Duca Abruzzi 24, I-10129 Turin, Italy."
"   [Shanthakumar, Subramanian] Vellore Inst Technol, Sch Civil Engn, Dept Environm & Water Resources Engn, Vellore 632014, Tamil Nadu, India."
C3 Polytechnic University of Turin; Vellore Institute of Technology (VIT);
   VIT Vellore
"RP Chiampo, F (corresponding author), Politecn Torino, Dept Appl Sci & Technol, Corso Duca Abruzzi 24, I-10129 Turin, Italy."
EM carla.raffa@polito.it; fulvia.chiampo@polito.it;
   shanthakumar.s@vit.ac.in
"RI S, Shanthakumar/M-8406-2018"
"OI S, Shanthakumar/0000-0003-1910-2536; Chiampo, Fulvia/0000-0002-1170-4623"
"CR Abdulhamid Y., 2017, KATSINA J NAT APPL S, V6, P168"
"   Ackova DG, 2018, PLANT SCI TODAY, V5, P14, DOI 10.14719/pst.2018.5.1.355"
"   Agnello AC, 2016, SCI TOTAL ENVIRON, V563, P693, DOI 10.1016/j.scitotenv.2015.10.061"
"   Akhter K, 2017, PAK J ZOOL, V49, P1841, DOI [10.11758/journal.pjz/2017.49.5.1841.1846, 10.17582/journal.pjz/2017.49.5.1841.1846]"
"   Al-Taee A, 2019, POLLUT RES, V38, P267"
"   Ali H, 2019, J CHEM-NY, V2019, DOI 10.1155/2019/6730305"
"   Ali H, 2018, TOXICOL ENVIRON CHEM, V100, P6, DOI 10.1080/02772248.2017.1413652"
"   Alkan FA, 2020, J ELEMENTOL, V25, P961, DOI 10.5601/jelem.2020.25.1.1959"
"   Ancona V, 2020, J CHEM TECHNOL BIOT, V95, P940, DOI 10.1002/jctb.6145"
"   Andreoli V, 2017, INT J ENV RES PUB HE, V14, DOI 10.3390/ijerph14010093"
"   [Anonymous], 2006, NEW YORK STATE NEW Y"
"   [Anonymous], 2015, 2016C2021 White Paper"
"   [Anonymous], NTR"
"   [Anonymous], 2011, Soil, Ground Water and Sediment Standards for Use Under Part XV.1 of the Environmental Protection Act"
"   Ashraf S, 2019, ECOTOX ENVIRON SAFE, V174, P714, DOI 10.1016/j.ecoenv.2019.02.068"
"   Atieh MA, 2017, BIOINORG CHEM APPL, V2017, DOI 10.1155/2017/4309198"
"   Awasthi S, 2017, FRONT PLANT SCI, V8, DOI 10.3389/fpls.2017.01007"
"   Ballesteros S, 2017, J GEOCHEM EXPLOR, V174, P132, DOI 10.1016/j.gexplo.2016.07.011"

"   Bano A, 2018, CHEMOSPHERE, V199, P218, DOI 10.1016/j.chemosphere.2018.02.043"
"   Baran A, 2018, ENVIRON GEOCHEM HLTH, V40, P435, DOI 10.1007/s10653-017-9924-7"
"   Bocardi JMB, 2020, J GEOCHEM EXPLOR, V217, DOI 10.1016/j.gexplo.2020.106591"
"   Boente C, 2017, J CLEAN PROD, V142, P2693, DOI 10.1016/j.jclepro.2016.11.007"
"   Cameselle C, 2016, PROCESS SAF ENVIRON, V104, P209, DOI 10.1016/j.psep.2016.09.002"
"   Brito ACC, 2020, WATER AIR SOIL POLL, V231, DOI 10.1007/s11270-020-04760-2"
"   Caporale AG, 2016, CURR POLLUT REP, V2, P15, DOI 10.1007/s40726-015-0024-y"
"   Capotorti G., 2009, P 11 INT C ENV SCI T"
"   Chang TC, 2006, J HAZARD MATER, V128, P208, DOI 10.1016/j.jhazmat.2005.07.053"
"   Chen WY, 2018, WATER AIR SOIL POLL, V229, DOI 10.1007/s11270-018-3784-3"
"   Cheng S, 2020, MOLECULES, V25, DOI 10.3390/molecules25143167"
"   Chirakkara RA, 2015, ELECTROCHIM ACTA, V181, P179, DOI 10.1016/j.electacta.2015.01.025"
"   Cicchella D, 2014, ENVIRON EARTH SCI, V71, P5129, DOI 10.1007/s12665-013-2916-8"
"   Courchesne F, 2017, INT J PHYTOREMEDIAT, V19, P545, DOI [10.1080/15226514.2016.1267700, 10.1080/15226514.2016]"
"   Cui ZJ, 2017, J ENVIRON CHEM ENG, V5, P3616, DOI 10.1016/j.jece.2017.07.021"
"   Dermont G, 2008, J HAZARD MATER, V152, P1, DOI 10.1016/j.jhazmat.2007.10.043"
"   Dhaliwal SS, 2020, ENVIRON SCI POLLUT R, V27, P1319, DOI 10.1007/s11356-019-06967-1"
"   Dinh TTT, 2018, The Journal of Agriculture and Development, V17, P44, DOI 10.52997/jad.6.03.2018"
"   Dixit R, 2015, SUSTAINABILITY-BASEL, V7, P2189, DOI 10.3390/su7022189"
"   dos Santos JV, 2016, ENVIRON SCI POLLUT R, V23, P6735, DOI 10.1007/s11356-015-5904-6"
"   Dumbrava A, 2015, INT J ENVIRON SCI TE, V12, P929, DOI 10.1007/s13762-013-0467-x"
"   Epelde L, 2014, APPL SOIL ECOL, V75, P1, DOI 10.1016/j.apsoil.2013.10.003"
"   Eskandari H, 2016, Journal of Advances in Environmental Health Research, V4, P234, DOI [10.22102/jaehr.2016.45965, 10.22102/JAEHR.2016.45965, DOI 10.22102/JAEHR.2016.45965]"
"   Fan J, 2020, J HAZARD MATER, V398, DOI 10.1016/j.jhazmat.2020.122901"
"   Fan TT, 2016, ENVIRON SCI TECHNOL, V50, P2931, DOI 10.1021/acs.est.5b05281"
"   Farooq MA, 2016, ENVIRON EXP BOT, V132, P42, DOI 10.1016/j.envexpbot.2016.08.004"
"   Feng WJ, 2020, J HAZARD MATER, V381, DOI 10.1016/j.jhazmat.2019.120997"
"   Gidlow DA, 2015, OCCUP MED-OXFORD, V65, P348, DOI 10.1093/occmed/kqv018"
"   Gong YY, 2018, WATER RES, V147, P440, DOI 10.1016/j.watres.2018.10.024"
"   Government Decree on the Assessment, 2007, SOIL CONT REM NEEDS"
"   Gueye MT, 2016, CHEM ENGINEER TRANS, V47, P289, DOI 10.3303/CET1647049"
"   Guo XF, 2016, CHEMOSPHERE, V147, P412, DOI 10.1016/j.chemosphere.2015.12.087"
"   HAKANSON L, 1980, WATER RES, V14, P975, DOI 10.1016/0043-1354(80)90143-8"
"   Han JG, 2010, J HAZARD MATER, V177, P530, DOI 10.1016/j.jhazmat.2009.12.065"
"   Hao XL, 2015, ENVIRON SCI TECHNOL, V49, P2328, DOI 10.1021/es504956a"
"   Hassan A, 2020, J SOIL SEDIMENT, V20, P66, DOI 10.1007/s11368-019-02394-4"
"   Hassan A, 2019, WATER AIR SOIL POLL, V230, DOI 10.1007/s11270-019-4227-5"
"   He F, 2015, ENVIRON SCI POLLUT R, V22, P8124, DOI 10.1007/s11356-015-4316-y"
"   He Z., 2015, J Environ Indic, V9, P17, DOI DOI 10.4236/AS.2019.103029"
"   Hejna M, 2018, ANIMAL, V12, P2156, DOI 10.1017/S175173111700355X"
"   Hussein S, 2007, ENVIRON SCI TECHNOL, V41, P8439, DOI [10.1021/es070908q, 10.1021/es0709O8q]"
"   Iram S, 2015, ARAB J SCI ENG, V40, P1867, DOI 10.1007/s13369-015-1702-1"
"   Islam MN, 2017, ENVIRON SCI POLLUT R, V24, P7177, DOI 10.1007/s11356-016-8330-5"
"   Jamal A., 2018, WORLD SCI NEWS, V105, P1"
"   Jan AT, 2015, INT J MOL SCI, V16, P29592, DOI 10.3390/ijms161226183"
"   Jeong HY, 2007, ENVIRON SCI TECHNOL, V41, P7699, DOI 10.1021/es0702891"
"   Jia WT, 2016, ENVIRON SCI POLLUT R, V23, P18823, DOI 10.1007/s11356-016-7083-5"
"   Jiang YX, 2017, CHEMOSPHERE, V168, P1658, DOI 10.1016/j.chemosphere.2016.11.088"
"   Jin K, 2021, COORDIN CHEM REV, V427, DOI 10.1016/j.ccr.2020.213473"
"   Jin LN, 2015, J COLLOID INTERF SCI, V453, P270, DOI 10.1016/j.jcis.2015.05.005"
"   Kaise, 2006, P ANN INT C SOILS SE, V12, P258"
"   Kelepertzis E, 2014, GEODERMA, V221, P82, DOI 10.1016/j.geoderma.2014.01.007"
"   Khalid S, 2017, J GEOCHEM EXPLOR, V182, P247, DOI 10.1016/j.gexplo.2016.11.021"
"   Kim BK, 2013, WATER AIR SOIL POLL, V224, DOI 10.1007/s11270-013-1698-7"
"   Kinuthia GK, 2020, SCI REP-UK, V10, DOI 10.1038/s41598-020-65359-5"
"   Koller M., 2018, Heavy Met, V13, DOI [10.5772/intechopen.74783, DOI 10.5772/INTECHOPEN.74783]"
"   Krezel A, 2016, ARCH BIOCHEM BIOPHYS, V611, P3, DOI 10.1016/j.abb.2016.04.010"
"   Kumar V, 2015, J TRACE ELEM MED BIO, V29, P269, DOI 10.1016/j.jtemb.2014.06.004"
"   Li LP, 2020, ENVIRON POLLUT, V257, DOI 10.1016/j.envpol.2019.113641"
"   Li ZT, 2020, ENVIRON POLLUT, V260, DOI 10.1016/j.envpol.2020.114098"
"   Li ZT, 2018, J HAZARD MATER, V344, P1, DOI 10.1016/j.jhazmat.2017.09.036"
"   Liao XY, 2016, J ENVIRON SCI, V41, P202, DOI 10.1016/j.jes.2015.06.017"
"   Liu L, 2018, PHOTOSYNTHETICA, V56, P1346, DOI 10.1007/s11099-018-0844-2"
"   Liu SJ, 2018, J HAZARD MATER, V346, P167, DOI 10.1016/j.jhazmat.2017.12.022"
"   Lu KP, 2017, J ENVIRON MANAGE, V186, P285, DOI 10.1016/j.jenvman.2016.05.068"
"   Mahmood-ul-Hassan M, 2020, INT J PHYTOREMEDIAT, V22, P725, DOI 10.1080/15226514.2019.1707772"
"   Mahurpawar M, 2015, International Journal of Research -GRANTHAALAYAH, V3, P1, DOI [10.29121/granthaalayah.v3.i9se.2015.3282, 10.29121/granthaalayah.v3.i9SE.2015.3282, DOI 10.29121/GRANTHAALAYAH.V3.I9SE.2015.3282]"
"   Marrugo-Negrete J, 2017, ENVIRON RES, V154, P380, DOI 10.1016/j.envres.2017.01.021"
"   Mishra S, 2016, J ENVIRON SCI HEAL C, V34, P1, DOI 10.1080/10590501.2015.1096883"
"   Nejad ZD, 2018, ENVIRON GEOCHEM HLTH, V40, P927, DOI 10.1007/s10653-017-9964-z"
"   Nwaichi E. O., 2016, Journal of Environmental Protection, V7, P41, DOI [10.4236/jep.2016.71004, 10.4236/jep.2016.71004]"
"   Pan XD, 2016, SCI REP-UK, V6, DOI 10.1038/srep20317"
"   Park B, 2017, ULTRASON SONOCHEM, V35, P640, DOI 10.1016/j.ultsonch.2016.02.002"
"   Park Jung-Duck, 2012, J Prev Med Public Health, V45, P344, DOI 10.3961/jpmph.2012.45.6.344"
"   Pei GP, 2020, ENVIRON POLLUT, V256, DOI 10.1016/j.envpol.2019.113407"
"   Pilipovic DT, 2018, B ENVIRON CONTAM TOX, V101, P725, DOI 10.1007/s00128-018-2442-1"
"   Rahimzadeh MR, 2017, CASP J INTERN MED, V8, P135, DOI 10.22088/cjim.8.3.135"
"   Reddy KR, 2009, Electro-chemical Remediation Technologies for Polluted Soils, Sediments and Groundwater"
"   Rosa MA, 2017, J TAIWAN INST CHEM E, V78, P409, DOI 10.1016/j.jtice.2017.06.046"

"   Saleem MH, 2020, PLANTS-BASEL, V9, DOI 10.3390/plants9060756"
"   Salomons W., 1995, Heavy metals: problems and solution"
"   Saran A, 2020, INT J PHYTOREMEDIAT, V22, P459, DOI 10.1080/15226514.2019.1675140"
"   Seshadri B, 2017, CHEMOSPHERE, V184, P197, DOI 10.1016/j.chemosphere.2017.05.172"
"   Shekhawat K., 2015, Int. J. Adv. Res, V3, P167, DOI DOI 10.1016/J.IJAR.2015.3.7.167"
"   Shen F, 2017, ECOTOX ENVIRON SAFE, V139, P254, DOI 10.1016/j.ecoenv.2017.01.044"
"   Shi LN, 2011, WATER RES, V45, P886, DOI 10.1016/j.watres.2010.09.025"
"   Shrivastava A, 2015, CURR POLLUT REP, V1, P35, DOI 10.1007/s40726-015-0004-2"
"   Sigua GC, 2019, ENVIRONMENTS, V6, DOI 10.3390/environments6060069"
"   Sivakumar D., 2014, POLLUT RES, V33, P553"
"   Skyllberg U, 2010, ENVIRON SCI TECHNOL, V44, P1254, DOI 10.1021/es902091w"
"   Song B, 2017, ENVIRON INT, V105, P43, DOI 10.1016/j.envint.2017.05.001"
"   Sun YH, 2020, TOXICS, V8, DOI 10.3390/toxics8020026"
"   Sun ZC, 2019, CHEMOSPHERE, V233, P17, DOI 10.1016/j.chemosphere.2019.05.233"
"   Suprasanna P., 2016, PLANT METAL INTERACT"
"   Taleei MM, 2019, B ENVIRON CONTAM TOX, V102, P134, DOI 10.1007/s00128-018-2495-1"
"   T??th G, 2016, ENVIRON INT, V88, P299, DOI 10.1016/j.envint.2015.12.017"
"   Ullah A, 2020, AGRONOMY-BASEL, V10, DOI 10.3390/agronomy10030359"
"   Vacca A, 2012, LAND DEGRAD DEV, V23, P350, DOI 10.1002/ldr.2153"
"   Van Liedekerke M., 2015, 27530 EUR EN"
"   Velkova Z, 2018, ENG LIFE SCI, V18, P871, DOI 10.1002/elsc.201800017"
"   Wang GY, 2020, ENVIRON RES, V186, DOI 10.1016/j.envres.2020.109554"
"   Wang HX, 2019, INT J ENV RES PUB HE, V16, DOI 10.3390/ijerph16224430"
"   Wang L.K., 2009, HEAVY METALS ENV"
"   Wang PX, 2018, INT J ENV RES PUB HE, V15, DOI 10.3390/ijerph15081731"
"   Wang T, 2019, J ENVIRON MANAGE, V231, P679, DOI 10.1016/j.jenvman.2018.10.085"
"   Wang T, 2017, SCI REP-UK, V7, DOI 10.1038/s41598-017-12503-3"
"   Wani Ab Latif, 2015, Interdiscip Toxicol, V8, P55, DOI [10.1515/intox-2015-0009, 10.1515/intox-2015-0009]"
"   Wen YM, 2012, J SOIL SEDIMENT, V12, P900, DOI 10.1007/s11368-012-0520-2"
"   Wu QH, 2015, SCI TOTAL ENVIRON, V506, P217, DOI 10.1016/j.scitotenv.2014.10.121"
"   Xing J, 2019, ECOTOX ENVIRON SAFE, V180, P457, DOI 10.1016/j.ecoenv.2019.05.034"
"   Xu JW, 2019, NAT COMMUN, V10, DOI 10.1038/s41467-019-10472-x"
"   Xue WJ, 2018, J HAZARD MATER, V359, P290, DOI 10.1016/j.jhazmat.2018.07.062"
"   Yan DX, 2020, SUSTAINABILITY-BASEL, V12, DOI 10.3390/su12051907"
"   Yang X, 2020, ENVIRON POLLUT, V264, DOI 10.1016/j.envpol.2020.114745"
"   Yang Y, 2017, CHEMOSPHERE, V188, P148, DOI 10.1016/j.chemosphere.2017.08.140"
"   Yang ZH, 2018, CHEMOSPHERE, V206, P83, DOI 10.1016/j.chemosphere.2018.04.166"
"   Yi YM, 2015, ECOL ENG, V81, P89, DOI 10.1016/j.ecoleng.2015.04.034"
"   Yu FM, 2020, APPL SOIL ECOL, V155, DOI 10.1016/j.apsoil.2020.103669"
"   Zabochnicka-Swiatek M, 2014, POL J ENVIRON STUD, V23, P551"
"   Zacchini M, 2011, BIOL PLANTARUM, V55, P383, DOI 10.1007/s10535-011-0060-4"
"   Zacchini M, 2009, WATER AIR SOIL POLL, V197, P23, DOI 10.1007/s11270-008-9788-7"
"   Zhai XQ, 2018, SCI TOTAL ENVIRON, V635, P92, DOI 10.1016/j.scitotenv.2018.04.119"
"   Zhang GX, 2016, ENVIRON POLLUT, V218, P513, DOI 10.1016/j.envpol.2016.07.031"
"   Zhang MY, 2020, J HAZARD MATER, V388, DOI 10.1016/j.jhazmat.2019.121822"
"   Zhang WH, 2010, J HAZARD MATER, V178, P578, DOI 10.1016/j.jhazmat.2010.01.124"
"   Zhou WX, 2020, INT J ENV RES PUB HE, V17, DOI 10.3390/ijerph17051745"
NR 144
TC 84
Z9 102
U1 10
U2 296
PU MDPI
PI BASEL
"PA MDPI AG, Grosspeteranlage 5, CH-4052 BASEL, SWITZERLAND"
EI 2076-3417
J9 APPL SCI-BASEL
JI Appl. Sci.-Basel
PD MAY
PY 2021
VL 11
IS 9
AR 4134
DI 10.3390/app11094134
PG 23
"WC Chemistry, Multidisciplinary; Engineering, Multidisciplinary; Materials"
"   Science, Multidisciplinary; Physics, Applied"
WE Science Citation Index Expanded (SCI-EXPANDED)
SC Chemistry; Engineering; Materials Science; Physics
GA SB4CR
UT WOS:000649945000001
"OA Green Submitted, gold"
DA 2026-04-06
ER

PT J
"AU Kumar, D"
"   Malik, S"
"   Rani, R"
"   Kumar, R"
"   Duhan, JS"
"AF Kumar, Dharmender"
"   Malik, Sachin"

"   Rani, Ritu"
"   Kumar, Ravinder"
"   Duhan, Joginder Singh"
"TI Behavior, risk, and bioremediation potential of heavy metals/metalloids"
   in the soil system
SO RENDICONTI LINCEI-SCIENZE FISICHE E NATURALI
LA English
DT Review
DE Heavy metal; Biotechnology; Phytoremediation; Fate and transport; Soil;
   Microbial remediation
ID PARTICULATE ORGANIC-MATTER; METAL ACCUMULATION; CONTAMINATED SOIL;
   OXIDATIVE STRESS; AGRICULTURAL SOILS; CADMIUM TOXICITY; SURFACE DISPLAY;
   BRASSICA-JUNCEA; TRACE-ELEMENTS; POLLUTED SOILS
"AB The application of pesticides and chemicals in agriculture and industrialization occasioned in meagre soil quality, which has a serious global concern. The most prevalent type of soil pollutants is inorganic contaminants, metals-metalloids, and organic pollutants like herbicides, pesticides, and polycyclic aromatic hydrocarbons. Even though heavy metals are essential for sustaining soil health, a slight increase in their concentration beyond the permissible limit can harm the soil. These metals cannot be degraded entirely but can be changed from highly toxic to less poisonous by chemical or biological methods. Further, the mobility of pollutants in soil-based environments might be increased due to competition among other heavy metal species for their available adsorption on the soil matrix. These heavy metals are hazardous in high concentrations to plants and animals. In addition to hindering nutrient uptake and limiting seed germination, excessive metal exposure also causes shoot growth to slow down, and lipid peroxidation occurs. In the present article, we see how these heavy metals accumulate continually in the soil and are later absorbed by plants. To appraise the impact of contamination and toxicity by the heavy metals in soil, their fate, transport, and toxicity mechanisms in soil systems have been described. The bioremediation strategies such as biosorption, siderophore formation, phytoremediation, phytovolatilization, and phytostabilization have also been discussed to understand the biodegradation of metalloids. Also, microbial remediation and genetic transformation are the biotechnological aspects employed to increase the degradation efficiency of heavy metals."
"C1 [Kumar, Dharmender; Malik, Sachin; Rani, Ritu] Deenbandhu Chhotu Ram Univ Sci & Technol, Dept Biotechnol, Murthal 131039, Sonepat, India."
"   [Kumar, Ravinder; Duhan, Joginder Singh] Chaudhary Devi Lal Univ, Dept Biotechnol, Sirsa 125055, India."
C3 Deenbandhu Chhotu Ram University of Science & Technology; Chaudhary Devi
   Lal University
"RP Kumar, D (corresponding author), Deenbandhu Chhotu Ram Univ Sci & Technol, Dept Biotechnol, Murthal 131039, Sonepat, India."
EM dkbiology@gmail.com
"RI Duhan, Joginder Singh/Z-2902-2019; Rani, Ritu/IYS-9372-2023; Kumar,"
"   Dharmender/AAB-4252-2022; Kumar, Ravinder/J-2723-2017"
"OI Duhan, Joginder Singh/0000-0001-5089-4442; Kumar,"
   Ravinder/0000-0001-7735-6740
"CR Abbas AA., 2016, INT RES J ENG TECHNO, V3, P1446"
"   Agrawal K., 2020, Combined Application of Physico-Chemical Microbiological Processes for Industrial Effluent Treatment Plant, P119, DOI DOI 10.1007/978-981-15-0497-6_7"
"   Ahirwar N, 2018, International Journal of Research in Applied, Natural and Social Sciences, V2008, P1, DOI DOI 10.21820/23987073.2018.3.1"
"   Ahluwalia SS, 2007, BIORESOURCE TECHNOL, V98, P2243, DOI 10.1016/j.biortech.2005.12.006"
"   Aihemaiti A, 2020, ENVIRON POLLUT, V265, DOI 10.1016/j.envpol.2020.114916"
"   Aihemaiti A, 2018, J ENVIRON MANAGE, V222, P216, DOI 10.1016/j.jenvman.2018.05.081"
"   Aihemaiti A, 2017, ENVIRON SCI POLLUT R, V24, P26839, DOI 10.1007/s11356-017-0250-5"
"   Aldrich MV, 2007, SCI TOTAL ENVIRON, V379, P249, DOI 10.1016/j.scitotenv.2006.08.053"
"   Alengebawy A, 2021, TOXICS, V9, DOI 10.3390/toxics9030042"
"   Ali H, 2013, CHEMOSPHERE, V91, P869, DOI 10.1016/j.chemosphere.2013.01.075"
"   Ali I, 2022, ENVIRON SCI POLLUT R, V29, P37765, DOI 10.1007/s11356-021-18109-7"
"   Almaguer-Cant?? V, 2011, WATER SCI TECHNOL, V63, P1607, DOI 10.2166/wst.2011.225"
"   Ameh EG, 2019, ENVIRON EARTH SCI, V78, DOI 10.1007/s12665-019-8343-8"
"   [Anonymous], ATSDR 1997 CERCLA PR"
"   Antosiewicz DM, 2005, ENVIRON POLLUT, V134, P23, DOI 10.1016/j.envpol.2004.07.019"
"   Arikpo G. E., 2004, Global Journal of Pure and Applied Sciences, V10, P257"
"   Ayangbenro AS, 2017, INT J ENV RES PUB HE, V14, DOI 10.3390/ijerph14010094"
"   Bae W, 2003, APPL ENVIRON MICROB, V69, P3176, DOI 10.1128/AEM.69.6.3176-3180.2003"
"   BAKER A J M, 1989, Biorecovery, V1, P81"
"   Balabane M, 2002, SOIL BIOL BIOCHEM, V34, P1513, DOI 10.1016/S0038-0717(02)00066-4"
"   Barros LM Jr, 2003, BRAZ J CHEM ENG, V20, P229, DOI 10.1590/S0104-66322003000300003"
"   Baun A, 2008, ECOTOXICOLOGY, V17, P387, DOI 10.1007/s10646-008-0208-y"
"   Bayrakli B, 2020, REND LINCEI-SCI FIS, V31, P393, DOI 10.1007/s12210-020-00901-1"
"   Ben-Moshe T, 2010, CHEMOSPHERE, V81, P387, DOI 10.1016/j.chemosphere.2010.07.007"
"   BEVERIDGE TJ, 1989, ANNU REV MICROBIOL, V43, P147, DOI 10.1146/annurev.mi.43.100189.001051"
"   Bhat SA, 2022, CHEMOSPHERE, V303, DOI 10.1016/j.chemosphere.2022.134788"
"   Bhuiyan MAH, 2010, J HAZARD MATER, V173, P384, DOI 10.1016/j.jhazmat.2009.08.085"
"   Blaylock M, 2000, PHYTOEXTRACTION META"
"   Bluskov S, 2005, INT J PHYTOREMEDIAT, V7, P153, DOI 10.1080/16226510590950441"
"   BRUNE A, 1995, J PLANT NUTR, V18, P853, DOI 10.1080/01904169509364943"
"   Brunner I, 2008, ENVIRON POLLUT, V152, P559, DOI 10.1016/j.envpol.2007.07.006"
"   Burges A, 2018, INT J PHYTOREMEDIAT, V20, P384, DOI 10.1080/15226514.2017.1365340"
"   Caussy D, 2003, ECOTOX ENVIRON SAFE, V56, P45, DOI 10.1016/S0147-6513(03)00049-6"
"   Cervantes C, 2001, FEMS MICROBIOL REV, V25, P335, DOI 10.1111/j.1574-6976.2001.tb00581.x"
"   Chakraborty S, 2014, ECOTOX ENVIRON SAFE, V106, P46, DOI 10.1016/j.ecoenv.2014.04.007"
"   Chen CY, 2009, CLEAN-SOIL AIR WATER, V37, P304, DOI 10.1002/clen.200800199"
"   Chen HS, 2019, ENVIRON POLLUT, V250, P118, DOI 10.1016/j.envpol.2019.03.123"
"   Chen L, 2021, J HAZARD MATER, V405, DOI 10.1016/j.jhazmat.2020.124200"
"   Chen WP, 2007, ECOTOX ENVIRON SAFE, V67, P48, DOI 10.1016/j.ecoenv.2006.12.013"
"   Chibuike G.U., 2014, APPL ENVIRON SOIL SC, DOI [10.1155/2014/752708, DOI 10.1155/2014/752708]"
"   Chojnacka K, 2010, ENVIRON INT, V36, P299, DOI 10.1016/j.envint.2009.12.001"

"   Chu BC, 2010, BIOMETALS, V23, P601, DOI 10.1007/s10534-010-9361-x"
"   Cui YS, 2008, GEODERMA, V146, P370, DOI 10.1016/j.geoderma.2008.06.010"
"   Danouche M, 2022, HELIYON, V8, DOI 10.1016/j.heliyon.2022.e08967"
"   Darlington TK, 2009, ENVIRON TOXICOL CHEM, V28, P1191, DOI 10.1897/08-341.1"
"   Debiec-Andrzejewska K, 2020, ENVIRON POLLUT, V264, DOI 10.1016/j.envpol.2020.114692"
"   DEGRAEVE N, 1981, MUTAT RES, V86, P115, DOI 10.1016/0165-1110(81)90035-X"
"   Demarco CF, 2019, INT J PHYTOREMEDIAT, V21, P1145, DOI 10.1080/15226514.2019.1612843"
"   Deng ZJ, 2017, CHEMOSPHERE, V168, P1100, DOI 10.1016/j.chemosphere.2016.10.097"
"   Diatloff E, 2006, BIOCHEM BIOPH RES CO, V344, P807, DOI 10.1016/j.bbrc.2006.03.212"
"   Dixit R, 2015, SUSTAINABILITY-BASEL, V7, P2189, DOI 10.3390/su7022189"
"   Donisa C, 2003, GEODERMA, V111, P75, DOI 10.1016/S0016-7061(02)00254-9"
"   Dotaniya ML, 2020, RAD HEA MET ENVIR, P83, DOI 10.1007/978-3-030-21638-2_5"
"   DRISCOLL CT, 1994, ENVIRON SCI TECHNOL, V28, pA136, DOI 10.1021/es00052a721"
"   Du HH, 2019, CHEM GEOL, V510, P84, DOI 10.1016/j.chemgeo.2019.02.024"
"   Du HH, 2018, ACS EARTH SPACE CHEM, V2, P556, DOI 10.1021/acsearthspacechem.8b00005"
"   Du HH, 2018, CHEMOSPHERE, V207, P404, DOI 10.1016/j.chemosphere.2018.05.092"
"   Du HH, 2017, ENVIRON POLLUT, V229, P871, DOI 10.1016/j.envpol.2017.07.052"
"   Dube BK, 2003, CHEMOSPHERE, V53, P1147, DOI 10.1016/S0045-6535(03)00570-8"
"   Dubey S, 2014, FUNCT INTEGR GENOMIC, V14, P401, DOI 10.1007/s10142-014-0361-8"
"   Edelstein M, 2018, SCI HORTIC-AMSTERDAM, V234, P431, DOI 10.1016/j.scienta.2017.12.039"
"   Elektorowicz M, 2015, INT J PHYTOREMEDIAT, V17, P521, DOI 10.1080/15226514.2013.876966"
"   Evanko C.R, 1997, Remediation of Metals-Contaminated Soils and Groundwater, P5"
"   Fagorzi C, 2018, GENES-BASEL, V9, DOI 10.3390/genes9110542"
"   Falkowski PG, 2008, SCIENCE, V320, P1034, DOI 10.1126/science.1153213"
"   Fang J, 2009, ENVIRON POLLUT, V157, P1101, DOI 10.1016/j.envpol.2008.11.006"
"   Fang LC, 2010, J HAZARD MATER, V181, P1031, DOI 10.1016/j.jhazmat.2010.05.118"
"   Fayiga AO, 2007, ENVIRON POLLUT, V147, P737, DOI 10.1016/j.envpol.2006.09.010"
"   Feng NX, 2017, SCI TOTAL ENVIRON, V583, P352, DOI 10.1016/j.scitotenv.2017.01.075"
"   Fergusson J.E., 1990, HEAVY ELEMENTS CHEM, V614"
"   Fomina M, 2014, BIORESOURCE TECHNOL, V160, P3, DOI 10.1016/j.biortech.2013.12.102"
"   Gadd G.M., 2000, Journal of Chemical Technology Biotechnology, V76, P325"
"   Gajewska E, 2007, BIOMETALS, V20, P27, DOI 10.1007/s10534-006-9011-5"
"   Gajewska E, 2009, ACTA PHYSIOL PLANT, V31, P1291, DOI 10.1007/s11738-009-0370-8"
"   Galal TM, 2016, REND LINCEI-SCI FIS, V27, P241, DOI 10.1007/s12210-015-0468-4"
"   Galunin E, 2014, J HAZARD MATER, V265, P280, DOI 10.1016/j.jhazmat.2013.11.010"
"   Gan CD, 2020, ENVIRON TECHNOL INNO, V20, DOI 10.1016/j.eti.2020.101090"
"   Gan CD, 2020, WATER AIR SOIL POLL, V231, DOI 10.1007/s11270-019-4377-5"
"   Garc??a-Su??rez R, 2017, J APPL MICROBIOL, V122, P1092, DOI 10.1111/jam.13407"
"   Garg N, 2011, ENVIRON CHEM LETT, V9, P303, DOI 10.1007/s10311-011-0313-7"
"   Ghosh S, 2008, LANGMUIR, V24, P12385, DOI 10.1021/la802015f"
"   Gimbert LJ, 2007, ENVIRON CHEM, V4, P8, DOI 10.1071/EN06072"
"   Gisbert C, 2003, BIOCHEM BIOPH RES CO, V303, P440, DOI 10.1016/S0006-291X(03)00349-8"
"   Gong XM, 2019, SCI TOTAL ENVIRON, V666, P1126, DOI 10.1016/j.scitotenv.2019.02.215"
"   Goswami RK, 2022, LETT APPL MICROBIOL, V75, P701, DOI 10.1111/lam.13564"
"   Gulati R, 1999, CURR SCI INDIA, V77, P1359"
"   Gupta VK, 2015, ENVIRON ENG RES, V20, P1, DOI 10.4491/eer.2015.018"
"   Gustafsson JP, 2019, APPL GEOCHEM, V102, P1, DOI 10.1016/j.apgeochem.2018.12.027"
"   Hadi B, 2003, INT J CHEM REACT ENG, V1"
"   Han FXX, 2004, NEW PHYTOL, V162, P489, DOI 10.1111/j.1469-8137.2004.01027.x"
"   Hanisch C, 1998, ENVIRON SCI TECHNOL, V32, p176A, DOI 10.1021/es983470q"
"   Hayat M.T., 2018, Cadmium Toxicity and Tolerance in Plants: From Physiology to Remediation, P163, DOI [10.1016/B978-0-12-814864-8.00007-3, DOI 10.1016/B978-0-12-814864-8.00007-3]"
"   Heikens Alex., 2006, Arsenic Contamination of Irrigation Water, Soil and Crops in Bangladesh: Risk Implications for Sustainable Agriculture and Food Safelty in Asia"
"   Hesterberg D, 2011, J ENVIRON QUAL, V40, P667, DOI 10.2134/jeq2010.0140"
"   Hirschi KD, 2000, PLANT PHYSIOL, V124, P125, DOI 10.1104/pp.124.1.125"
"   Hosseini MJ, 2016, ENVIRON TOXICOL, V31, P979, DOI 10.1002/tox.22107"
"   Hu YN, 2020, J HAZARD MATER, V391, DOI 10.1016/j.jhazmat.2020.122244"
"   Huang JH, 2015, CHEM GEOL, V417, P68, DOI 10.1016/j.chemgeo.2015.09.019"
"   Huang JW, 1996, NEW PHYTOL, V134, P75, DOI 10.1111/j.1469-8137.1996.tb01147.x"
"   HUANG JWW, 1992, PLANT PHYSIOL, V98, P230, DOI 10.1104/pp.98.1.230"
"   Imtiaz M, 2016, ENVIRON SCI POLLUT R, V23, P19787, DOI 10.1007/s11356-016-7192-1"
"   Imtiaz M, 2015, PLANT SOIL, V390, P17, DOI 10.1007/s11104-014-2341-0"
"   Iqbal M, 2013, CHEMOSPHERE, V91, P287, DOI 10.1016/j.chemosphere.2012.11.032"
"   Izquierdo M, 2017, GEODERMA, V295, P41, DOI 10.1016/j.geoderma.2017.02.006"
"   JAMES BR, 1983, J ENVIRON QUAL, V12, P177, DOI 10.2134/jeq1983.00472425001200020005x"
"   J??rup L, 2003, BRIT MED BULL, V68, P167, DOI 10.1093/bmb/ldg032"
"   Jiang B, 2018, ENVIRON SCI POLLUT R, V25, P31272, DOI 10.1007/s11356-018-3069-9"
"   Jiang W, 2000, BIOL PLANTARUM, V43, P603, DOI 10.1023/A:1002804100215"
"   Jibril SA, 2017, ADV AGR, V2017, P1, DOI [10.1155/2017/1236830, 10.1155/2017/1236830]"
"   Johnstone TC, 2015, DALTON T, V44, P6320, DOI 10.1039/c4dt03559c"
"   Jones L., 1981, The Fate of Heavy Metals in the Chemistry of Soil Processes"
"   Kaasalainen M, 2003, ENVIRON POLLUT, V126, P225, DOI 10.1016/S0269-7491(03)00191-X"
"   Kajla S, 2021, REND LINCEI-SCI FIS, V32, P257, DOI 10.1007/s12210-021-00982-6"
"   KAPLAN DI, 1990, WATER AIR SOIL POLL, V49, P81, DOI 10.1007/BF00279512"
"   Kasemodel MC, 2019, SCI TOTAL ENVIRON, V675, P367, DOI 10.1016/j.scitotenv.2019.04.223"
"   Kelepertzis E, 2020, SCI TOTAL ENVIRON, V725, DOI 10.1016/j.scitotenv.2020.138300"
"   Keller C, 2002, J ENVIRON QUAL, V31, P1550, DOI 10.2134/jeq2002.1550"
"   Khan I, 2019, ARAB J CHEM, V12, P908, DOI 10.1016/j.arabjc.2017.05.011"
"   Kim YJ, 2006, FEBS LETT, V580, P206, DOI 10.1016/j.febslet.2005.12.001"
"   KOEDAM N, 1994, BIOMETALS, V7, P287"
"   Kong ZY, 2015, PLANT SOIL, V391, P383, DOI 10.1007/s11104-015-2434-4"
"   Krishnaswamy R, 2000, APPL ENVIRON MICROB, V66, P5383, DOI 10.1128/AEM.66.12.5383-5386.2000"
"   KUMAR PBAN, 1995, ENVIRON SCI TECHNOL, V29, P1232, DOI 10.1021/es00005a014"

"   Kumar S, 2019, ENVIRON RES, V179, DOI 10.1016/j.envres.2019.108792"
"   Kumar V, 2020, ENVIRON TECHNOL INNO, V20, DOI 10.1016/j.eti.2020.101075"
"   Kumar V, 2021, REND LINCEI-SCI FIS, V32, P805, DOI 10.1007/s12210-021-01024-x"
"   Kumar V, 2019, CHEMOSPHERE, V216, P449, DOI 10.1016/j.chemosphere.2018.10.066"
"   Kunene SC, 2020, J HAZARD MATER, V383, DOI 10.1016/j.jhazmat.2019.121167"
"   K??pper H, 2017, METAL IONS LIFE SCI, V17, P491, DOI 10.1515/9783110434330-015"
"   Kushwaha A, 2018, ECOTOX ENVIRON SAFE, V147, P1035, DOI 10.1016/j.ecoenv.2017.09.049"
"   Lajayer BA, 2019, ENVIRON SCI POLLUT R, V26, P8468, DOI 10.1007/s11356-019-04241-y"
"   Lan MM, 2020, INT J ENV RES PUB HE, V17, DOI 10.3390/ijerph17051661"
"   Lasat M., 2000, Journal of Hazardous Substance Research, V2, P5, DOI [10.4148/1090-7025.1015, DOI 10.4148/1090-7025.1015]"
"   Lasota J, 2020, WATER AIR SOIL POLL, V231, DOI 10.1007/s11270-020-4450-0"
"   Li JR, 2016, PLANT CELL PHYSIOL, V57, P1720, DOI 10.1093/pcp/pcw093"
"   Li Q, 2018, ENVIRON POLLUT, V233, P569, DOI 10.1016/j.envpol.2017.10.091"
"   Li QQ, 2009, FEMS MICROBIOL LETT, V299, P44, DOI 10.1111/j.1574-6968.2009.01724.x"
"   Li YJ, 2004, PLANT CELL PHYSIOL, V45, P1787, DOI 10.1093/pcp/pch202"
"   LIANG CN, 1977, ENVIRON POLLUT, V12, P141, DOI 10.1016/0013-9327(77)90017-9"
"   Liao Min, 2005, J Zhejiang Univ Sci B, V6, P324, DOI 10.1631/jzus.2005.B0324"
"   LIU DH, 1994, ISR J PLANT SCI, V42, P143, DOI 10.1080/07929978.1994.10676565"
"   Liu JL, 2019, ENVIRON INT, V129, P461, DOI 10.1016/j.envint.2019.04.068"
"   Liu SL, 2019, J HAZARD MATER, V371, P233, DOI 10.1016/j.jhazmat.2019.03.016"
"   Liu ZC, 2020, J HAZARD MATER, V400, DOI 10.1016/j.jhazmat.2020.123138"
"   Loi N. N., 2018, Russian Agricultural Sciences, V44, P49, DOI [10.3103/s1068367418010111, 10.3103/S1068367418010111]"
"   Losada Benavides L. C., 2018, International Journal of Applied Environmental Sciences, V13, P9"
"   Luo YF, 2019, J SOIL SEDIMENT, V19, P702, DOI 10.1007/s11368-018-2082-4"
"   Luong J.H.T., 2007, OPEN ANAL CHEM J, V1, P7, DOI DOI 10.2174/1874065000701010007"
"   Ma L, 2020, SCI TOTAL ENVIRON, V724, DOI 10.1016/j.scitotenv.2020.138176"
"   Makarova A, 2021, SAUDI J BIOL SCI, V28, P3037, DOI 10.1016/j.sjbs.2021.02.045"
"   Malea P, 2021, ECOTOX ENVIRON SAFE, V208, DOI 10.1016/j.ecoenv.2020.111386"
"   Malik S, 2024, BIOTECHNOL GENET ENG, V40, P154, DOI 10.1080/02648725.2023.2182546"
"   Mani D, 2015, INT J PHYTOREMEDIAT, V17, P256, DOI 10.1080/15226514.2014.883497"
"   Marchiol L, 2004, ENVIRON POLLUT, V132, P21, DOI 10.1016/j.envpol.2004.04.001"
"   Marg BZ, 2011, HAZARDOUS METALS MIN"
"   Mariano C, 2020, ENVIRON SCI POLLUT R, V27, P13550, DOI 10.1007/s11356-020-07913-2"
"   Mart??nez-Trujillo M, 2015, NEW BIOTECHNOL, V32, P396, DOI 10.1016/j.nbt.2015.03.009"
"   Masindi V., 2018, HEAVY METALS PP, P117, DOI DOI 10.5772/INTECHOPEN.76082"
"   Masoom H, 2016, ENVIRON SCI TECHNOL, V50, P1670, DOI 10.1021/acs.est.5b03410"
"   McLaughlin MJ, 2000, AUST J SOIL RES, V38, P1037, DOI 10.1071/SR99128"
"   Mello IS, 2020, ECOTOX ENVIRON SAFE, V202, DOI 10.1016/j.ecoenv.2020.110818"
"   Midhat L, 2019, ECOTOX ENVIRON SAFE, V169, P150, DOI 10.1016/j.ecoenv.2018.11.009"
"   Mishra T, 2020, ENVIRON GEOCHEM HLTH, V42, P4101, DOI 10.1007/s10653-020-00529-y"
"   Mohamed I, 2010, J SOIL SEDIMENT, V10, P973, DOI 10.1007/s11368-010-0199-1"
"   Montiel-Rozas MM, 2016, ENVIRON POLLUT, V216, P273, DOI 10.1016/j.envpol.2016.05.080"
"   MOREL JL, 1986, BIOL FERT SOILS, V2, P29, DOI 10.1007/BF00638958"
"   Mosa KA, 2016, FRONT PLANT SCI, V7, DOI 10.3389/fpls.2016.00303"
"   Nawaz MA, 2018, J PLANT PHYSIOL, V220, P115, DOI 10.1016/j.jplph.2017.11.003"
"   NEILANDS JB, 1995, J BIOL CHEM, V270, P26723, DOI 10.1074/jbc.270.45.26723"
"   Neustadt J., 2007, Journal of Integrative Medicine, V6, P36"
"   Niu H, 1999, J CHEM TECHNOL BIOT, V74, P778, DOI 10.1002/(SICI)1097-4660(199908)74:8<778::AID-JCTB99>3.0.CO;2-Q"
"   Ojuederie OB, 2017, INT J ENV RES PUB HE, V14, DOI 10.3390/ijerph14121504"
"   Olness A, 2005, IND CROP PROD, V21, P165, DOI 10.1016/j.indcrop.2004.02.005"
"   Fari??a AO, 2018, CHEM GEOL, V479, P22, DOI 10.1016/j.chemgeo.2017.12.022"
"   Oumenskou H, 2018, ARAB J GEOSCI, V11, DOI 10.1007/s12517-018-4021-5"
"   Leguizamo MAO, 2017, CHEMOSPHERE, V168, P1230, DOI 10.1016/j.chemosphere.2016.10.075"
"   Pan XM, 2020, SCI TOTAL ENVIRON, V715, DOI 10.1016/j.scitotenv.2020.136904"
"   Peijnenburg W, 2000, ARCH ENVIRON CON TOX, V39, P420, DOI 10.1007/s002440010123"
"   Peralta-Videa JR, 2009, INT J BIOCHEM CELL B, V41, P1665, DOI 10.1016/j.biocel.2009.03.005"
"   P??rez-Palacios P, 2017, ENVIRON SCI POLLUT R, V24, P14910, DOI 10.1007/s11356-017-9092-4"
"   Philip L, 2001, INT J ENVIRON POLLUT, V15, P448, DOI 10.1504/IJEP.2001.004835"
"   Pilon-Smits EAH, 1999, PLANT PHYSIOL, V119, P123, DOI 10.1104/pp.119.1.123"
"   Pinto AP, 2008, J PLANT NUTR, V31, P1746, DOI 10.1080/01904160802324829"
"   Placek A, 2016, INT J PHYTOREMEDIAT, V18, P605, DOI 10.1080/15226514.2015.1086308"
"   Qafoku NP, 2000, COMMUN SOIL SCI PLAN, V31, P1051, DOI [10.1080/00103620009370497, 10.1080/00103620009370532]"
"   Qin CC, 2020, CHEMOSPHERE, V261, DOI 10.1016/j.chemosphere.2020.128010"
"   Qu CC, 2019, ENVIRON SCI TECHNOL, V53, P8147, DOI 10.1021/acs.est.9b00856"
"   Raymond KN., 1984, STRUCT CHEM, DOI [10.1002/chin.198442369, DOI 10.1002/CHIN.198442369]"
"   Rizwan M, 2012, J HAZARD MATER, V209, P326, DOI 10.1016/j.jhazmat.2012.01.033"
"   Rugh CL, 1998, HORTSCIENCE, V33, P618, DOI 10.21273/HORTSCI.33.4.618"
"   Sabreena, 2022, PLANTS-BASEL, V11, DOI 10.3390/plants11091255"
"   Saco D, 2013, BIOL PLANTARUM, V57, P128, DOI 10.1007/s10535-012-0133-z"
"   Sahi SV, 2002, ENVIRON SCI TECHNOL, V36, P4676, DOI 10.1021/es020675x"
"   Sajwan KS, 1996, WATER AIR SOIL POLL, V91, P209, DOI 10.1007/BF00666258"
"   Salem H.M., 2000, Heavy metals in drinking water their environment impact on human health, P542"
"   Samal DPK, 2020, MATER TODAY-PROC, V30, P346, DOI 10.1016/j.matpr.2020.02.165"
"   Samsami S, 2020, PROCESS SAF ENVIRON, V143, P138, DOI 10.1016/j.psep.2020.05.034"
"   Samuelson P, 2000, APPL ENVIRON MICROB, V66, P1243, DOI 10.1128/AEM.66.3.1243-1248.2000"
"   Saran A, 2020, INT J PHYTOREMEDIAT, V22, P459, DOI 10.1080/15226514.2019.1675140"
"   Sebastia J, 2008, EUR J SOIL SCI, V59, P304, DOI 10.1111/j.1365-2389.2007.01001.x"
"   Sen Gupta S, 2012, PHYS CHEM CHEM PHYS, V14, P6698, DOI 10.1039/c2cp40093f"
"   Seregin IV, 2006, RUSS J PLANT PHYSL+, V53, P257, DOI 10.1134/S1021443706020178"
"   Shaheen SM, 2019, ADV COLLOID INTERFAC, V265, P1, DOI 10.1016/j.cis.2019.01.002"

"   Shanker AK, 2005, ENVIRON INT, V31, P739, DOI 10.1016/j.envint.2005.02.003"
"   Sharma P., 2014, Int J Environ Bioremediat Biodegrad, V2, P178, DOI DOI 10.12691/IJEBB-2-4-5"
"   Sharma P, 2021, J ENVIRON CHEM ENG, V9, DOI 10.1016/j.jece.2021.106673"
"   Sheoran V, 2016, PEDOSPHERE, V26, P148, DOI 10.1016/S1002-0160(15)60032-7"
"   Shi MQ, 2021, SCI TOTAL ENVIRON, V752, DOI 10.1016/j.scitotenv.2020.141930"
"   Sidhu GPS, 2017, CHEMOSPHERE, V182, P129, DOI 10.1016/j.chemosphere.2017.05.026"
"   Singh N, 2006, ENVIRON POLLUT, V141, P238, DOI 10.1016/j.envpol.2005.08.050"
"   Smith L.A., 1995, REMEDIATION OPTION M, P17"
"   Soeprobowati T. R., 2012, P 2 INT SEM NEW PAR, V3, P72"
"   Song JW, 2018, ENVIRON POLLUT, V243, P510, DOI 10.1016/j.envpol.2018.09.011"
"   Srinath T., 2003, Indian Journal of Experimental Biology, V41, P986"
"   Stambulska UY, 2018, BIOMED RES INT, V2018, DOI 10.1155/2018/8031213"
"   Steffens M, 2017, ENVIRON SCI TECHNOL, V51, P12182, DOI 10.1021/acs.est.7b03715"
"   Stoikou V, 2017, FOODS, V6, DOI 10.3390/foods6070051"
"   Thakur S, 2016, ENVIRON MONIT ASSESS, V188, DOI 10.1007/s10661-016-5211-9"
"   Tian LY, 2015, ENVIRON SCI POLLUT R, V22, P9215, DOI 10.1007/s11356-014-4031-0"
"   Tian LY, 2014, CHEMOSPHERE, V111, P89, DOI 10.1016/j.chemosphere.2014.03.051"
"   Tiodar ED, 2021, INT J ENV RES PUB HE, V18, DOI 10.3390/ijerph18052435"
"   Tripathi RD, 2007, TRENDS BIOTECHNOL, V25, P158, DOI 10.1016/j.tibtech.2007.02.003"
"   Tsezos M., 2006, Proceedings of EU-Asia Workshop on Clean Production and Nanotechnologies, P23"
"   Turner A, 2019, SCI TOTAL ENVIRON, V657, P1409, DOI 10.1016/j.scitotenv.2018.12.096"
"   Uddin MK, 2017, CHEM ENG J, V308, P438, DOI 10.1016/j.cej.2016.09.029"
"   Unrine J.M., 2008, Nanoscience and Nanotechnology Environmental and Health Impacts, P345, DOI [DOI 10.1002/9780470396612.CH14, 10.1002/9780470396612.ch14]"
"   Uraguchi S, 2009, J EXP BOT, V60, P2677, DOI 10.1093/jxb/erp119"
"   Vardhan KH, 2019, J MOL LIQ, V290, DOI 10.1016/j.molliq.2019.111197"
"   Vel??squez L, 2009, J HAZARD MATER, V167, P713, DOI 10.1016/j.jhazmat.2009.01.044"
"   VOLESKY B, 1995, BIOTECHNOL PROGR, V11, P235, DOI 10.1021/bp00033a001"
"   VOLESKY B, 1995, APPL MICROBIOL BIOT, V42, P797, DOI 10.1007/BF00171964"
"   Wang C, 2021, SCI TOTAL ENVIRON, V777, DOI 10.1016/j.scitotenv.2021.145852"
"   Wang DY, 2016, CHEMOSPHERE, V147, P3, DOI 10.1016/j.chemosphere.2015.11.069"
"   WANG MY, 1994, PLANT PHYSIOL, V104, P899, DOI 10.1104/pp.104.3.899"
"   Wang Q, 2021, TOXICOL SCI, V181, P105, DOI 10.1093/toxsci/kfab017"
"   Wani W, 2018, REND LINCEI-SCI FIS, V29, P709, DOI 10.1007/s12210-018-0702-y"
"   Weyens N, 2011, INT J PHYTOREMEDIAT, V13, P244, DOI 10.1080/15226511003753920"
"   Weyens N, 2010, ENVIRON POLLUT, V158, P2422, DOI 10.1016/j.envpol.2010.04.004"
"   Wu G, 2010, J HAZARD MATER, V174, P1, DOI 10.1016/j.jhazmat.2009.09.113"
"   Wu YH, 2019, ENVIRON POLLUT, V246, P608, DOI 10.1016/j.envpol.2018.12.076"
"   Wu ZZ, 2021, ECOTOX ENVIRON SAFE, V207, DOI 10.1016/j.ecoenv.2020.111297"
"   Wuana R.A., 2011, ISRN Ecology, DOI [10.5402/2011/402647, DOI 10.5402/2011/402647]"
"   Xiong J, 2015, J COLLOID INTERF SCI, V457, P121, DOI 10.1016/j.jcis.2015.07.001"
"   Xu DM, 2021, J CLEAN PROD, V321, DOI 10.1016/j.jclepro.2021.128730"
"   Yadav K.K., 2017, J MAT ENV SCI, V8, P740"
"   Yang J, 2020, ECOL ENG, V156, DOI 10.1016/j.ecoleng.2020.105975"
"   Yang J, 2011, BIOL TRACE ELEM RES, V142, P787, DOI 10.1007/s12011-010-8777-z"
"   Yang JY, 2015, B ENVIRON CONTAM TOX, V94, P448, DOI 10.1007/s00128-015-1498-4"
"   Yang JY, 2017, ENVIRON POLLUT, V231, P48, DOI 10.1016/j.envpol.2017.07.075"
"   Yang K, 2015, CHEMOSPHERE, V138, P657, DOI 10.1016/j.chemosphere.2015.07.061"
"   Yin H, 2016, CHEMOSPHERE, V161, P181, DOI 10.1016/j.chemosphere.2016.07.018"
"   Yu HY, 2016, ENVIRON POLLUT, V209, P38, DOI 10.1016/j.envpol.2015.11.021"
"   Yu Y, 2010, MYCORRHIZA, V20, P407, DOI 10.1007/s00572-009-0296-4"
"   Yuan Y, 2020, INT J ENVIRON SCI TE, V17, P383, DOI 10.1007/s13762-019-02451-y"
"   Yusuf M, 2011, B ENVIRON CONTAM TOX, V86, P1, DOI 10.1007/s00128-010-0171-1"
"   Yusuf M, 2011, ARCH ENVIRON CON TOX, V60, P68, DOI 10.1007/s00244-010-9535-0"
"   Zavala YJ, 2008, ENVIRON SCI TECHNOL, V42, P3856, DOI 10.1021/es702747y"
"   Zhang J, 2017, PLANT SOIL, V416, P259, DOI 10.1007/s11104-017-3207-z"
"   Zhang MK, 2010, COMMUN SOIL SCI PLAN, V41, P820, DOI 10.1080/00103621003592341"
"   Zhang QG, 2021, ENVIRON POLLUT, V272, DOI 10.1016/j.envpol.2020.115989"
"   Zhang WJ, 2018, INT J PHYTOREMEDIAT, V20, P454, DOI 10.1080/15226514.2017.1365345"
"   Zhou J, 2018, PLOS ONE, V13, DOI 10.1371/journal.pone.0191139"
"   Zhou T, 2018, ENVIRON POLLUT, V232, P514, DOI 10.1016/j.envpol.2017.09.081"
"   Zhu HH, 2020, J HAZARD MATER, V388, DOI 10.1016/j.jhazmat.2020.122080"
"   Zhu J, 2019, ENVIRON CHEM LETT, V17, P1271, DOI 10.1007/s10311-019-00881-1"
NR 265
TC 23
Z9 28
U1 12
U2 98
PU SPRINGER-VERLAG ITALIA SRL
PI MILAN
"PA VIA DECEMBRIO, 28, MILAN, 20137, ITALY"
SN 2037-4631
EI 1720-0776
J9 REND LINCEI-SCI FIS
JI Rend. Lincei.-Sci. Fis. Nat.
PD SEP
PY 2023
VL 34
IS 3
SI SI
BP 809
EP 831
DI 10.1007/s12210-023-01166-0
EA JUN 2023

PG 23
WC Multidisciplinary Sciences
WE Science Citation Index Expanded (SCI-EXPANDED)
SC Science & Technology - Other Topics
GA P5ZD7
UT WOS:000999561200001
DA 2026-04-06
ER

PT J
"AU Kamyab, H"
"   Chelliapan, S"
"   Khalili, E"
"   Mediavilla, DPZ"
"   Khorami, M"
"   Aminabhavi, TM"
"   Vasseghian, Y"
"AF Kamyab, Hesam"
"   Chelliapan, Shreeshivadasan"
"   Khalili, Elham"
"   Mediavilla, Diana Patricia Zuleta"
"   Khorami, Majid"
"   Aminabhavi, Tejraj M."
"   Vasseghian, Yasser"
TI Nanobioremediation of heavy metals using microorganisms
SO JOURNAL OF ENVIRONMENTAL MANAGEMENT
LA English
DT Review
DE Heavy metals; Nanobioremediation; Nanophytoremediation; Microorganisms
ID VALENT IRON NZVI; WASTE-WATER; PHANEROCHAETE-CHRYSOSPORIUM;
   DECABROMODIPHENYL ETHER; OXIDE NANOPARTICLES; GOLD NANOPARTICLES;
   CONTAMINATED SOIL; GREEN SYNTHESIS; REMEDIATION; REMOVAL
"AB Heavy metals (HMs) in soil are a big threat to environmental and agricultural sustainability. This review discusses the development of the nanobioremediation paradigm (an approach combining nanotechnology and microbiological processes) for the treatment of HM toxicity. Recent advances in nanoparticle (NP)-based approaches to their ability to improve microbial detoxification methods, such as adsorption, redox transformation, and enzyme activation forms of microbial detoxification are discussed. Key findings suggest that many engineered NPs (e.g., zero-valent iron, biogenic metal oxides) can enhance HM immobilization efficiencies and contour microbial performances and functions of the soil microbiome. Still, there are areas of uncertainty regarding NP-microbe-soil interactions, long-term ecotoxicological effects, and applicability at the field level that are critical to be discussed. Future research directions are advised to focus on the eco-designed NPs, optimizing microbial consortia specific to the soil, and interdisciplinary frameworks linking the laboratory methods and materials to the real-world applications."
"C1 [Kamyab, Hesam] Saveetha Inst Med & Tech Sci, Dept Biomat, Saveetha Dent Coll & Hosp, Chennai 600077, India."
"   [Kamyab, Hesam] Korea Univ, KU KIST Grad Sch Energy & Environm, 145 Anam Ro, Seoul 02841, South Korea."
"   [Kamyab, Hesam] Univ UTE, Quito 170527, Ecuador."
"   [Chelliapan, Shreeshivadasan] Univ Teknol Malaysia, Fac Artificial Intelligence, Dept Smart Engn & Adv Technol, Jalan Sultan Yahya Petra, Kuala Lumpur 54100, Malaysia."
"   [Khalili, Elham] Univ Surrey, Ctr Vis Speech & Signal Proc, Fac Engn & Phys Sci, Guildford, the United Kingdom."
"   [Mediavilla, Diana Patricia Zuleta] UTE Univ, Fac Architecture & Urbanism, Architecture Dept, LL Liminal Lab,Res Grp, Quito 170527, Ecuador."
"   [Khorami, Majid] UTE Univ, Fac Architecture & Urbanism, Architecture Dept, TCEMC Res Grp, Quito 170527, Ecuador."
"   [Aminabhavi, Tejraj M.] KLE Technol Univ, Ctr Energy & Environm, Sch Adv Sci, Hubballi 580031, Karnataka, India."
"   [Aminabhavi, Tejraj M.] Korea Univ, Seoul 02841, South Korea."
"   [Vasseghian, Yasser] Yuan Ze Univ, Dept Chem Engn & Mat Sci, Taoyuan, Taiwan."
C3 Saveetha Institute of Medical & Technical Science; Saveetha Dental
   College & Hospital; Korea University; Universiti Teknologi Malaysia;
   University of Surrey; KLE Technological University; Korea University;
   Yuan Ze University
"RP Kamyab, H (corresponding author), Korea Univ, KU KIST Grad Sch Energy & Environm, 145 Anam Ro, Seoul 02841, South Korea.; Chelliapan, S (corresponding author), Univ Teknol Malaysia, Fac Artificial Intelligence, Dept Smart Engn & Adv Technol, Jalan Sultan Yahya Petra, Kuala Lumpur 54100, Malaysia.; Aminabhavi, TM (corresponding author), KLE Technol Univ, Ctr Energy & Environm, Sch Adv Sci, Hubballi 580031, Karnataka, India."
EM hesam_kamyab@yahoo.com; shreeshivadasan.kl@utm.my; aminabhavit@gmail.com
"RI Aminabhavi, Tejraj/AAY-3470-2021; CHELLIAPAN,"
"   SHREESHIVADASAN/J-6997-2019; Khorami, Majid/AAN-1515-2021; Vasseghian,"
"   Yasser/AAN-4675-2020; Zuleta Mediavilla, Diana Patricia/JCE-8658-2023;"
"   Kamyab, Hesam/H-4583-2016"
"OI Aminabhavi, Tejraj/0000-0002-5613-3916; Khorami,"
"   Majid/0000-0003-4592-0233; Zuleta Mediavilla, Diana"
   Patricia/0000-0001-5146-302X;
"CR Abd Elnabi MK, 2023, TOXICS, V11, DOI 10.3390/toxics11070580"
"   Acharya P, 2020, SCI REP-UK, V10, DOI 10.1038/s41598-020-61696-7"
"   Adeleke Bartholomew Saanu, 2024, Heliyon, V10, pe39636, DOI [10.1016/j.heliyon.2024.e39636, 10.1016/j.heliyon.2024.e39636]"
"   Adenigba VO, 2020, IOP CONF SER-MAT SCI, V805, DOI 10.1088/1757-899X/805/1/012030"
"   Ahmad I, 2012, PAK J BOT, V44, P1569"
"   Akhtar N, 2021, TOXICS, V9, DOI 10.3390/toxics9050113"
"   Alabresm A, 2018, SCI TOTAL ENVIRON, V630, P1292, DOI 10.1016/j.scitotenv.2018.02.277"

"   Alazaiza MYD, 2021, WATER-SUI, V13, DOI 10.3390/w13162186"
"   Aleksandrov V., 2024, Phytoremediation and biofortification, P29"
"   Ali Seyid Amjad, 2024, Environmental Science and Pollution Research International, V31, P42185, DOI [10.1007/s11356-024-33911-9, 10.1007/s11356-024-33911-9]"
"   Allan J, 2021, REGUL TOXICOL PHARM, V122, DOI 10.1016/j.yrtph.2021.104885"
"   Amani AM, 2025, CARBOHYDR POLYM TECH, V10, DOI 10.1016/j.carpta.2025.100863"
"   Amani AM, 2025, J IND ENG CHEM, V149, P275, DOI [10.1016/j.jiec.2025.02.020, 10.1016/j.jiec.2025.02.020]"
"   Amani AM, 2025, ECOTOX ENVIRON SAFE, V291, DOI 10.1016/j.ecoenv.2025.117817"
"   Amani AM, 2025, SENS BIO-SENS RES, V47, DOI 10.1016/j.sbsr.2024.100732"
"   Amani AM, 2025, ADV COMPOS HYBRID MA, V8, DOI 10.1007/s42114-024-01118-8"
"   Amani AM, 2024, INT J PHARMACEUT, V667, DOI 10.1016/j.ijpharm.2024.124847"
"   Amani AM, 2024, J CLEAN PROD, V436, DOI 10.1016/j.jclepro.2024.140606"
"   Amani AM, 2023, ACS OMEGA, V9, P3123, DOI 10.1021/acsomega.3c06590"
"   Amani AM, 2022, APPL PHYS A-MATER, V128, DOI 10.1007/s00339-022-05763-y"
"   Ameen F, 2021, ECOTOX ENVIRON SAFE, V213, DOI 10.1016/j.ecoenv.2021.112027"
"   Angon PB, 2024, HELIYON, V10, DOI 10.1016/j.heliyon.2024.e28357"
"   Ariaeenejad S, 2024, INT J BIOL MACROMOL, V278, DOI 10.1016/j.ijbiomac.2024.134813"
"   Arif N, 2016, FRONT ENV SCI-SWITZ, V4, DOI 10.3389/fenvs.2016.00069"
"   Aryal M, 2024, J ENVIRON CHEM ENG, V12, DOI 10.1016/j.jece.2024.112221"
"   Aseri V., 2024, Letters in Applied NanoBioScience, V13, P57"
"   Ayilara MS, 2023, FRONT AGRON, V5, DOI 10.3389/fagro.2023.1183691"
"   Azizli MJ, 2025, J POLYM ENVIRON, V33, P1947, DOI 10.1007/s10924-025-03505-8"
"   Azizli MJ, 2024, J POLYM ENVIRON, V32, P5576, DOI 10.1007/s10924-024-03327-0"
"   Azizli MJ, 2023, J ADHES SCI TECHNOL, V37, P2754, DOI 10.1080/01694243.2023.2166613"
"   Balaraman P, 2020, WASTE BIOMASS VALORI, V11, P5255, DOI 10.1007/s12649-020-01083-5"
"   Balzano S, 2020, FRONT MICROBIOL, V11, DOI 10.3389/fmicb.2020.00517"
"   Baraga??o D, 2020, SCI REP-UK, V10, DOI 10.1038/s41598-020-58852-4"
"   Behera M, 2023, Drugs and Drug Candidates, V2, P554, DOI [10.3390/ddc2030029, 10.3390/ddc2030029, DOI 10.3390/DDC2030029]"
"   Benjamin SR, 2019, INT J ENVIRON POLLUT, V66, P19, DOI 10.1504/IJEP.2019.104526"
"   Berillo D, 2024, GELS-BASEL, V10, DOI 10.3390/gels10100646"
"   Bhargava A, 2016, J ENVIRON MANAGE, V183, P22, DOI 10.1016/j.jenvman.2016.08.021"
"   Bhatt P, 2022, J HAZARD MATER, V427, DOI 10.1016/j.jhazmat.2021.128033"
"   Bher A, 2022, INT J MOL SCI, V23, DOI 10.3390/ijms232012165"
"   Bhushan Divya, 2024, Chemosphere, V365, P143340, DOI [10.1016/j.chemosphere.2024.143340, 10.1016/j.chemosphere.2024.143340]"
"   Bist P, 2022, BIOL TRACE ELEM RES, V200, P5328, DOI 10.1007/s12011-021-03092-4"
"   Biswas M, 2008, J AM CERAM SOC, V91, P3197, DOI 10.1111/j.1551-2916.2008.02617.x"
"   Blosi M, 2022, J ENVIRON MANAGE, V304, DOI 10.1016/j.jenvman.2021.114187"
"   Bulot A, 2017, RESTOR ECOL, V25, P354, DOI 10.1111/rec.12424"
"   Buta M, 2021, ECOTOX ENVIRON SAFE, V214, DOI 10.1016/j.ecoenv.2021.112070"
"   Campillo-Cora C, 2025, EUR J SOIL BIOL, V124, DOI 10.1016/j.ejsobi.2024.103706"
"   Cao XF, 2020, SCI TOTAL ENVIRON, V704, DOI 10.1016/j.scitotenv.2019.135378"
"   Capeness MJ, 2019, FRONT MICROBIOL, V10, DOI 10.3389/fmicb.2019.00997"
"   Casarrubia S, 2020, FRONT MICROBIOL, V11, DOI 10.3389/fmicb.2020.00341"
"   Castro-Longoria E, 2012, J MICROBIOL BIOTECHN, V22, P1000, DOI 10.4014/jmb.1110.10085"
"   Chakrabarti A.e., 2017, Current Progress in Medical Mycology, P1"
"   Chakraborty SC, 2022, PROCESS SAF ENVIRON, V162, P230, DOI 10.1016/j.psep.2022.04.011"
"   Chandra R, 2016, FOR SCI TECHNOL, V12, P55, DOI 10.1080/21580103.2015.1044024"
"   Chatterjee S, 2020, CHEM ENG J, V385, DOI 10.1016/j.cej.2019.123790"
"   Chaudhary R, 2020, BIOMOLECULES, V10, DOI 10.3390/biom10111498"
"   Chauhan P, 2023, ENVIRON RES, V321, DOI 10.1016/j.envres.2023.116224"
"   Chen GQ, 2013, APPL MICROBIOL BIOT, V97, P3149, DOI 10.1007/s00253-012-4121-1"
"   Chen JY, 2024, J HAZARD MATER, V466, DOI 10.1016/j.jhazmat.2024.133622"
"   Chen YF, 2021, SCI TOTAL ENVIRON, V781, DOI 10.1016/j.scitotenv.2021.146773"
"   Cheng P, 2021, NANOMATERIALS-BASEL, V11, DOI 10.3390/nano11051264"
"   Cheng SY, 2019, TRENDS BIOTECHNOL, V37, P1255, DOI 10.1016/j.tibtech.2019.04.007"
"   Chibuike G.U., 2014, APPL ENVIRON SOIL SC, DOI [10.1155/2014/752708, DOI 10.1155/2014/752708]"
"   Chowardhara B, 2020, ACTA PHYSIOL PLANT, V42, DOI 10.1007/s11738-020-03094-0"
"   Colozza N, 2017, SCI TOTAL ENVIRON, V584, P692, DOI 10.1016/j.scitotenv.2017.01.099"
"   Costa IGF, 2019, J HAZARD MATER, V379, DOI 10.1016/j.jhazmat.2019.120837"
"   Deckwer WD, 2004, ENVIRON SCI TECHNOL, V38, P1858, DOI 10.1021/es0300517"
"   Dehghani F, 2022, APPL PHYS A-MATER, V128, DOI 10.1007/s00339-022-05387-2"
"   Mart??nez-Macias MD, 2019, ENVIRON SCI POLLUT R, V26, P6311, DOI 10.1007/s11356-018-3963-1"
"   Deng JB, 2022, CHEMOSPHERE, V301, DOI 10.1016/j.chemosphere.2022.134753"
"   Dhaarani R., 2025, Intell-Based Med, V11, DOI [10.1016/j.ibmed.2024.100251, DOI 10.1016/J.IBMED.2024.100251]"
"   Ding CL, 2022, FRONT ENV SCI-SWITZ, V10, DOI 10.3389/fenvs.2022.920957"
"   Dixit R, 2015, SUSTAINABILITY-BASEL, V7, P2189, DOI 10.3390/su7022189"
"   Dobrikova A, 2022, PLANTS-BASEL, V11, DOI 10.3390/plants11182407"
"   Dong DX, 2023, ENVIRON INT, V173, DOI 10.1016/j.envint.2023.107842"
"   Dong GW, 2013, BIOCHEM ENG J, V70, P166, DOI 10.1016/j.bej.2012.11.002"
"   Dong HL, 2013, EUR J NUTR, V52, P1853, DOI 10.1007/s00394-012-0487-1"
"   Ebele Anekwe Jennifer, 2017, Emerging Contaminants, V3, P1, DOI [10.1016/j.emcon.2016.12.004, 10.1016/j.emcon.2016.12.004]"
"   El-Kalliny AS, 2023, DISCOV NANO, V18, DOI 10.1186/s11671-023-03787-8"
"   El-Sappah AH, 2024, FRONT PLANT SCI, V15, DOI 10.3389/fpls.2024.1423625"
"   El-Sheekh MM, 2021, INT J PHYTOREMEDIAT, V23, P1538, DOI 10.1080/15226514.2021.1915957"
"   Emenike EC, 2023, CARBOHYDR POLYM TECH, V6, DOI 10.1016/j.carpta.2023.100337"
"   Enez A, 2018, APPL SCI-BASEL, V8, DOI 10.3390/app8101953"
"   Espinosa-Leal CA, 2018, PLANTA, V248, P1, DOI 10.1007/s00425-018-2910-1"
"   Espinosa-Ortiz EJ, 2016, ENVIRON SCI POLLUT R, V23, P21619, DOI 10.1007/s11356-016-7333-6"
"   Fallah S, 2024, PLANT NANO BIOL, V10, DOI 10.1016/j.plana.2024.100105"
"   Fan WH, 2016, WATER RES, V105, P129, DOI 10.1016/j.watres.2016.08.060"

"   Fateminasab F, 2024, APPL FOOD BIOTECHNOL, V11, DOI 10.22037/afb.v11i1.46832"
"   Feitz AJ, 2005, COLLOID SURFACE A, V265, P88, DOI 10.1016/j.colsurfa.2005.01.038"
"   Gayathiri E, 2022, AGRONOMY-BASEL, V12, DOI 10.3390/agronomy12030662"
"   Gholami-Shabani M, 2016, FUNG BIOL-US, P91, DOI 10.1007/978-3-319-42990-8_5"
"   Ghosh S, 2021, FRONT MICROBIOL, V12, DOI 10.3389/fmicb.2021.638068"
"   Giannakoula A, 2021, PLANTS-BASEL, V10, DOI 10.3390/plants10010155"
"   Giese EC, 2020, CRIT REV BIOTECHNOL, V40, P653, DOI 10.1080/07388551.2020.1751583"
"   Gil-D??az M, 2019, SCI TOTAL ENVIRON, V675, P165, DOI 10.1016/j.scitotenv.2019.04.183"
"   Gil-D??az M, 2017, SCI TOTAL ENVIRON, V584, P1324, DOI 10.1016/j.scitotenv.2017.02.011"
"   Gil-D??az M, 2016, CHEMOSPHERE, V149, P137, DOI 10.1016/j.chemosphere.2016.01.106"
"   GILLHAM RW, 1994, GROUND WATER, V32, P958, DOI 10.1111/j.1745-6584.1994.tb00935.x"
"   Gol-Soltani Mehrnoosh, 2024, Environmental Science and Pollution Research International, V31, P39602, DOI [10.1007/s11356-024-33808-7, 10.1007/s11356-024-33808-7]"
"   Gomes Marcelo Pedrosa, 2025, Discover Plants, V2, DOI [10.1007/s44372-025-00090-x, 10.1007/s44372-025-00090-x]"
"   Gomes MP, 2011, SCI AGR, V68, P566, DOI 10.1590/S0103-90162011000500009"
"   Gong YY, 2018, WATER RES, V147, P440, DOI 10.1016/j.watres.2018.10.024"
"   Gong YY, 2012, NANOTECHNOLOGY, V23, DOI 10.1088/0957-4484/23/29/294007"
"   Gonzalez JM, 2023, MICROORGANISMS, V11, DOI 10.3390/microorganisms11071641"
"   Govindan N, 2020, MATER ADV, V1, P3460, DOI 10.1039/d0ma00698j"
"   Gupta P, 2015, ENVIRON EXP BOT, V109, P12, DOI 10.1016/j.envexpbot.2014.08.001"
"   Haghighizadeh A, 2024, ARAB J CHEM, V17, DOI 10.1016/j.arabjc.2024.105777"
"   Haider FU, 2024, ECOTOX ENVIRON SAFE, V281, DOI 10.1016/j.ecoenv.2024.116620"
"   Haider FU, 2021, ECOTOX ENVIRON SAFE, V211, DOI 10.1016/j.ecoenv.2020.111887"
"   Hassan MG, 2024, BMC MICROBIOL, V24, DOI 10.1186/s12866-023-03171-7"
"   Hassan MU, 2019, ENVIRON SCI POLLUT R, V26, P12673, DOI 10.1007/s11356-019-04892-x"
"   Hazra C, 2013, J CHEM TECHNOL BIOT, V88, P1039, DOI 10.1002/jctb.3934"
"   He JS, 2014, BIORESOURCE TECHNOL, V160, P67, DOI 10.1016/j.biortech.2014.01.068"
"   He SY, 2016, CHEMOSPHERE, V147, P195, DOI 10.1016/j.chemosphere.2015.12.055"
"   Heaton L, 2016, ESPA ABER, V6, P35, DOI [10.36403/espacoaberto.2016.5244, 10.36403/espacoaberto.2016.5244, DOI 10.36403/ESPACOABERTO.2016.5244]"
"   Hidangmayum A, 2023, INT J ENVIRON SCI TE, V20, P11693, DOI 10.1007/s13762-022-04560-7"
"   Hu YC, 2024, SCI TOTAL ENVIRON, V918, DOI 10.1016/j.scitotenv.2024.170577"
"   Huang XP, 2021, J MATER RES TECHNOL, V12, P1649, DOI 10.1016/j.jmrt.2021.03.048"
"   Huang ZZ, 2018, CHEMOSPHERE, V211, P573, DOI 10.1016/j.chemosphere.2018.07.192"
"   Hung CM, 2021, J HAZARD MATER, V420, DOI 10.1016/j.jhazmat.2021.126553"
"   Hussain A, 2019, ECOTOX ENVIRON SAFE, V173, P156, DOI 10.1016/j.ecoenv.2019.01.118"
"   Hwang M, 2012, TOX RESEARCH, V28, P73, DOI 10.5487/TR.2012.28.2.073"
"   Ihsanullah, 2016, SEP PURIF TECHNOL, V157, P141, DOI 10.1016/j.seppur.2015.11.039"
"   Ilyas A, 2025, Premier J. Sci., V9"
"   Iqbal B, 2023, PLANT STRESS, V10, DOI 10.1016/j.stress.2023.100283"
"   Jena AB, 2023, BIOMED PHARMACOTHER, V162, DOI 10.1016/j.biopha.2023.114606"
"   Jomova K, 2024, ARCH TOXICOL, V98, P1323, DOI 10.1007/s00204-024-03696-4"
"   Jomova K, 2022, CHEM-BIOL INTERACT, V367, DOI 10.1016/j.cbi.2022.110173"
"   Kamyab H, 2025, J HAZARD MATER ADV, V18, DOI 10.1016/j.hazadv.2025.100673"
"   Kansour MK, 2023, ENVIRON TECHNOL INNO, V32, DOI 10.1016/j.eti.2023.103259"
"   Kazemi S, 2023, MATER TODAY SUSTAIN, V24, DOI 10.1016/j.mtsust.2023.100500"
"   Kebede G, 2021, J CHEM-NY, V2021, DOI 10.1155/2021/9823362"
"   Khan N, 2016, INT J PHYTOREMEDIAT, V18, P1258, DOI 10.1080/15226514.2016.1203287"
"   Khandel P, 2018, J NANOSTRUCTURE CHEM, V8, P217, DOI 10.1007/s40097-018-0267-4"
"   Khanna K, 2021, ECOTOX ENVIRON SAFE, V222, DOI 10.1016/j.ecoenv.2021.112459"
"   Khanna P, 2019, J MICROBIOL METH, V163, DOI 10.1016/j.mimet.2019.105656"
"   Kisku GC, 2018, INT J PHYTOREMEDIAT, V20, P330, DOI 10.1080/15226514.2017.1381942"
"   Kondakindi VR, 2024, ENVIRON CHEM ECOTOX, V6, P408, DOI 10.1016/j.enceco.2024.05.002"
"   Kouhbanani MAJ, 2023, J DRUG DELIV SCI TEC, V85, DOI 10.1016/j.jddst.2023.104541"
"   Koul B., 2018, Biotechnological Strategies for E ective Remediation of Polluted Soils, P240"
"   Kumar V, 2025, BIORESOUR TECH REP, V29, DOI 10.1016/j.biteb.2025.102040"
"   Kumari SA, 2022, MICROMACHINES-BASEL, V13, DOI 10.3390/mi13091416"
"   Kuppan N, 2024, WASTE MANAGE B, V2, P154, DOI [10.1016/j.wmb.2024.07.005, 10.1016/j.wmb.2024.07.005]"
"   Laslo V, 2022, J MOL STRUCT, V1247, DOI 10.1016/j.molstruc.2021.131325"
"   Latif M, 2024, APPL WATER SCI, V15, DOI 10.1007/s13201-024-02341-w"
"   Lee HJ, 2021, J MICROBIOL BIOTECHN, V31, P903, DOI 10.4014/jmb.2106.06056"
"   Lee SJ, 2021, CHEMOSPHERE, V262, DOI 10.1016/j.chemosphere.2020.128358"
"   Li SL, 2017, J HAZARD MATER, V322, P163, DOI 10.1016/j.jhazmat.2016.01.032"
"   Li SL, 2014, ENVIRON SCI-PROC IMP, V16, P524, DOI 10.1039/c3em00578j"
"   Li WL, 2020, SCI TOTAL ENVIRON, V737, DOI 10.1016/j.scitotenv.2020.139745"
"   Li XL, 2021, WATER-SUI, V13, DOI 10.3390/w13202928"
"   Lima EN, 2024, FRONT NANOTECHNOL, V6, DOI 10.3389/fnano.2024.1389107"
"   Lin Bao-shan, 2004, Journal of Forestry Research (Harbin), V15, P138, DOI 10.1007/BF02856749"
"   Lin H, 2022, CHEMOSPHERE, V305, DOI 10.1016/j.chemosphere.2022.135457"
"   Lin H, 2021, J HAZARD MATER, V402, DOI 10.1016/j.jhazmat.2020.123829"
"   Lippy BE, 2025, AM J IND MED, V68, pS45, DOI 10.1002/ajim.23618"
"   Liu BY, 2024, SCI TOTAL ENVIRON, V918, DOI 10.1016/j.scitotenv.2024.170281"
"   Luo HQ, 2022, INT BIODETER BIODEGR, V175, DOI 10.1016/j.ibiod.2022.105492"
"   Mahana A, 2021, ECOTOX ENVIRON SAFE, V208, DOI 10.1016/j.ecoenv.2020.111662"
"   Mahanty Shouvik, 2023, Environmental Nanotechnology, Monitoring and Management, V20, DOI [10.1016/j.enmm.2023.100854, 10.1016/j.enmm.2023.100854]"
"   Mahanty S, 2020, J WATER PROCESS ENG, V37, DOI 10.1016/j.jwpe.2020.101426"
"   Mahdavinia GH, 2012, CHINESE J CHEM, V30, P703, DOI 10.1002/cjoc.201280008"
"   Mahmoud ME, 2018, INT J BIOL MACROMOL, V111, P393, DOI 10.1016/j.ijbiomac.2018.01.014"
"   Maicas S, 2020, MICROORGANISMS, V8, DOI 10.3390/microorganisms8081142"
"   Mandal N., 2020, Soil Analysis: Recent Trends and Applications, P285, DOI [10.1007/978-981-15-2039-6_15, DOI 10.1007/978-981-15-2039-6_15]"
"   Mansoor S, 2023, PLANTS-BASEL, V12, DOI 10.3390/plants12163003"

"   Mart??nez-Toledo A, 2023, CATENA, V222, DOI 10.1016/j.catena.2022.106851"
"   Mechouche MS, 2022, ENVIRON RES, V204, DOI 10.1016/j.envres.2021.112360"
"   Miglani GS, 2017, J CROP IMPROV, V31, P453, DOI 10.1080/15427528.2017.1333192"
"   Milano F, 2024, PLANT PHYSIOL BIOCH, V216, DOI 10.1016/j.plaphy.2024.109090"
"   Mir IR, 2022, S AFR J BOT, V150, P387, DOI 10.1016/j.sajb.2022.07.039"
"   Mitra S, 2022, J KING SAUD UNIV SCI, V34, DOI 10.1016/j.jksus.2022.101865"
"   Mohamadpour F, 2024, RSC ADV, V14, P20609, DOI 10.1039/d4ra03259d"
"   Mondal Pravash, 2019, Journal of Experimental Biology and Agricultural Sciences, V7, P579, DOI [10.18006/2019.7(6).579.586, 10.18006/2019.7(6).579.586]"
"   Moradi N, 2024, RHIZOSPHERE-NETH, V29, DOI 10.1016/j.rhisph.2023.100828"
"   Mosleh-Shirazi S, 2021, APPL PHYS A-MATER, V127, DOI 10.1007/s00339-021-05005-7"
"   Nagar N, 2018, MATER CHEM PHYS, V213, P44, DOI 10.1016/j.matchemphys.2018.04.007"
"   Najafipour I, 2025, POLYM TEST, V142, DOI 10.1016/j.polymertesting.2024.108659"
"   Narayanan KB, 2011, J HAZARD MATER, V189, P519, DOI 10.1016/j.jhazmat.2011.02.069"
"   Nedjimi B, 2021, SN APPL SCI, V3, DOI 10.1007/s42452-021-04301-4"
"   Nguyen HV, 2024, RSC ADV, V14, P23720, DOI 10.1039/d4ra03907f"
"   Nuruzzaman Md, 2025, Science of The Total Environment, V962, P178398, DOI [10.1016/j.scitotenv.2025.178398, 10.1016/j.scitotenv.2025.178398]"
"   Ojuederie OB, 2017, INT J ENV RES PUB HE, V14, DOI 10.3390/ijerph14121504"
"   Oladimeji T., 2024, HELIYON, V10"
"   Olson MR, 2014, CHEMOSPHERE, V114, P144, DOI 10.1016/j.chemosphere.2014.03.109"
"   Omoregie Gloria, 2019, Tanzania Journal of Science, V45, P372"
"   Ondrasek G, 2025, RSC ADV, V15, P3904, DOI 10.1039/d4ra04639k"
"   Ovung A, 2021, BIOPHYS REV-GER, V13, P259, DOI 10.1007/s12551-021-00795-9"
"   Oyebamiji OO, 2019, BIORESOUR TECH REP, V7, P100247, DOI [10.1016/j.biteb.2019.100247, 10.1016/j.biteb.2019.100247, DOI 10.1016/J.BITEB.2019.100247]"
"   Oyewole O.A., 2019, Enhanced degradation of crude oil with Alcaligenes faecalis ADY25 and iron oxide nanoparticle"
"   Pande V, 2022, FRONT MICROBIOL, V13, DOI 10.3389/fmicb.2022.824084"
"   Pasch V, 2025, NEW PHYTOL, V245, P1496, DOI 10.1111/nph.20317"
"   P??rez-Hern??ndez H, 2024, INT J ENVIRON RES, V18, DOI 10.1007/s41742-023-00556-4"
"   Perkovic S, 2022, AGRONOMY-BASEL, V12, DOI 10.3390/agronomy12122945"
"   Pillai H. P. S., 2016, Journal of Environmental Protection, V7, P734, DOI [10.4236/jep.2016.75066, 10.4236/jep.2016.75066]"
"   Prathna TC, 2018, SEP PURIF TECHNOL, V199, P260, DOI 10.1016/j.seppur.2018.01.061"
"   Priya AK, 2022, CHEMOSPHERE, V307, DOI 10.1016/j.chemosphere.2022.135957"
"   Priya M, 2014, ELSEV INSIGHT, P521, DOI 10.1016/B978-0-12-800021-2.00023-6"
"   Priyadarshanee M, 2022, J HAZARD MATER, V423, DOI 10.1016/j.jhazmat.2021.126985"
"   Qiu XH, 2011, J HAZARD MATER, V193, P70, DOI 10.1016/j.jhazmat.2011.07.024"
"   Rad SA, 2023, SUSTAINABILITY-BASEL, V15, DOI 10.3390/su15010876"
"   Rafeeq H, 2023, CHEMOSPHERE, V310, DOI 10.1016/j.chemosphere.2022.136751"
"   Rai PK, 2019, ENVIRON INT, V125, P365, DOI 10.1016/j.envint.2019.01.067"
"   Rajkumari J, 2019, J PHOTOCH PHOTOBIO B, V201, DOI 10.1016/j.jphotobiol.2019.111667"
"   Ramezani M., 2021, MICROBIAL REJUVENATI, P349, DOI DOI 10.1007/978-981-15-7455-9_14"
"   Ramos-Ruiz A, 2016, ENVIRON SCI TECHNOL, V50, P1492, DOI 10.1021/acs.est.5b04074"
"   Raza A, 2024, PLANT CELL REP, V43, DOI 10.1007/s00299-024-03153-7"
"   Rebello S, 2021, BIOENGINEERED, V12, P12839, DOI 10.1080/21655979.2021.2004645"
"   Reddy AS, 2009, J NANOSCI NANOTECHNO, V9, P6693, DOI 10.1166/jnn.2009.1347"
"   Refaey M., 2021, Role of Fungi in Bioremediation of Soil Contaminated with Heavy Metals, P509"
"   Rezaei F, 2010, J SCI FOOD AGR, V90, P2000, DOI 10.1002/jsfa.4044"
"   Riyazuddin R, 2022, BIOMOLECULES, V12, DOI 10.3390/biom12010043"
"   Rizwan M, 2019, ENVIRON POLLUT, V248, P358, DOI 10.1016/j.envpol.2019.02.031"
"   Rostamizadeh S, 2009, J HETEROCYCLIC CHEM, V46, P74, DOI 10.1002/jhet.35"
"   Rozas EE, 2017, J HAZARD MATER, V329, P120, DOI 10.1016/j.jhazmat.2017.01.037"
"   Rucinska-Sobkowiak R, 2016, ACTA PHYSIOL PLANT, V38, DOI 10.1007/s11738-016-2277-5"
"   Rutigliano FA, 2023, PEDOBIOLOGIA, V99, DOI 10.1016/j.pedobi.2023.150894"
"   Saha A., 2021, Biotica Research Today, V3, P1106"
"   Saleem MH, 2022, FRONT PLANT SCI, V13, DOI 10.3389/fpls.2022.1033092"
"   Sangkharak K, 2020, CURR MICROBIOL, V77, P897, DOI 10.1007/s00284-020-01883-x"
"   Saravanan A, 2023, ENVIRON POLLUT, V339, DOI 10.1016/j.envpol.2023.122720"
"   Saravanan A, 2022, CHEMOSPHERE, V298, DOI 10.1016/j.chemosphere.2022.134341"
"   Sarubbo LA, 2022, BIOCHEM ENG J, V181, DOI 10.1016/j.bej.2022.108377"
"   Sarvalkar PD, 2024, RESULTS ENG, V22, DOI 10.1016/j.rineng.2024.102094"
"   Savithramma N., 2012, Nano Vision, V2"
"   Scheffer LK, 2020, ELIFE, V9, DOI 10.7554/eLife.57443"
"   Schwirn K, 2020, INTEGR ENVIRON ASSES, V16, P706, DOI 10.1002/ieam.4267"
"   Shalaby SM, 2021, ENVIRON SCI POLLUT R, V28, P65549, DOI 10.1007/s11356-021-15544-4"
"   Shams A, 2024, ACS SYNTH BIOL, V13, P1412, DOI 10.1021/acssynbio.4c00048"
"   Sharafi S, 2025, SCI REP-UK, V15, DOI 10.1038/s41598-025-91453-7"
"   Sharma P, 2021, J HAZARD MATER, V416, DOI 10.1016/j.jhazmat.2021.125855"
"   Shi C, 2023, CHEMOSPHERE, V329, DOI 10.1016/j.chemosphere.2023.138631"
"   Shi LN, 2011, WATER RES, V45, P886, DOI 10.1016/j.watres.2010.09.025"
"   Shin PK, 1999, J MICROBIOL BIOTECHN, V9, P784"
"   Shiri P, 2021, BEILSTEIN J ORG CHEM, V17, DOI 10.3762/bjoc.17.114"
"   Singh MP, 2013, BIOMED RES INT, V2013, DOI 10.1155/2013/180156"
"   Singh R, 2013, J HAZARD MATER, V258, P35, DOI 10.1016/j.jhazmat.2013.04.016"
"   Sodhi KK, 2022, CURR RES MICROB SCI, V3, DOI 10.1016/j.crmicr.2022.100166"
"   Sohn EK, 2015, BIOMED RES INT-UK, V2015, DOI 10.1155/2015/893049"
"   Soler-Rovira P., 1995, Nutrient Cycl. Agroecosyst., V43, P173"
"   Sone Y, 2013, AMB EXPRESS, V3, DOI 10.1186/2191-0855-3-41"
"   Sood M., 2025, Discover Plants, V2, P187"
"   Sultana N, 2020, SCI REP-UK, V10, DOI 10.1038/s41598-020-72236-8"
"   Sun RG, 2022, CHEMOSPHERE, V291, DOI 10.1016/j.chemosphere.2021.132792"
"   Sun SC, 2021, ARCH MICROBIOL, V203, P2699, DOI 10.1007/s00203-021-02271-0"

"   Sunny NE, 2022, CHEMOSPHERE, V300, DOI 10.1016/j.chemosphere.2022.134612"
"   Suthar S, 2014, ECOTOX ENVIRON SAFE, V109, P177, DOI 10.1016/j.ecoenv.2014.07.030"
"   Tamanreet K., 2020, Organic Agriculture"
"   Tang HY, 2024, FRONT PLANT SCI, V15, DOI 10.3389/fpls.2024.1420408"
"   Tang SQ, 2024, J HAZARD MATER, V465, DOI 10.1016/j.jhazmat.2023.133098"
"   Tangahu BV, 2011, INT J CHEM ENG, V2011, DOI 10.1155/2011/939161"
"   Tian YX, 2024, J ENVIRON CHEM ENG, V12, DOI 10.1016/j.jece.2024.113741"
"   Tokarz E, 2015, J ECOL ENG, V16, P20, DOI [10.12911/22998993/2801, DOI 10.12911/22998993/2801, 10.12911/22998993/2801]"
"   Trujillo-Reyes J, 2014, J HAZARD MATER, V280, P487, DOI 10.1016/j.jhazmat.2014.08.029"
"   Upadhyay SK, 2023, MICROBIOL RES, V273, DOI 10.1016/j.micres.2023.127419"
"   Upadhyay SK, 2021, CHEMOSPHERE, V267, DOI 10.1016/j.chemosphere.2020.129216"
"   Vacondio B, 2015, BIOCATAL AGRIC BIOTE, V4, P266, DOI 10.1016/j.bcab.2015.03.005"
"   Vafa E, 2025, J MATER RES TECHNOL, V36, P4773, DOI [10.1016/j.jmrt.2025.04.055, 10.1016/j.jmrt.2025.04.055]"
"   Doan VD, 2022, CHEMOSPHERE, V286, DOI 10.1016/j.chemosphere.2021.131894"
"   Vanlalveni C, 2024, HELIYON, V10, DOI 10.1016/j.heliyon.2024.e32333"
"   Vasilachi IC, 2023, AGRICULTURE-BASEL, V13, DOI 10.3390/agriculture13101983"
"   Vishnupriya C, 2024, INT J BIOL MACROMOL, V255, DOI 10.1016/j.ijbiomac.2023.128032"
"   Wan Q, 2023, J HAZARD MATER, V453, DOI 10.1016/j.jhazmat.2023.131390"
"   Wang AS, 2006, PLANT SOIL, V281, P325, DOI 10.1007/s11104-005-4642-9"
"   Wang CY, 2024, MINERALS-BASEL, V14, DOI 10.3390/min14050506"
"   Wang HR, 2015, ENVIRON SCI TECHNOL, V49, P529, DOI 10.1021/es504832n"
"   Wang JY, 2023, SCI TOTAL ENVIRON, V902, DOI 10.1016/j.scitotenv.2023.165949"
"   Wang K, 2024, J HAZARD MATER, V465, DOI 10.1016/j.jhazmat.2023.133309"
"   Wang L, 2017, CHEM ENG J, V314, P622, DOI 10.1016/j.cej.2016.12.020"
"   Wang M, 2022, ENVIRON SCI POLLUT R, V29, P91255, DOI 10.1007/s11356-022-22195-6"
"   Wang Q, 2022, J HAZARD MATER, V433, DOI 10.1016/j.jhazmat.2022.128749"
"   Wang W, 2016, CHEM ENG J, V304, P79, DOI 10.1016/j.cej.2016.06.069"
"   Wu G, 2010, J HAZARD MATER, V174, P1, DOI 10.1016/j.jhazmat.2009.09.113"
"   Wu HW, 2024, SCI TOTAL ENVIRON, V909, DOI 10.1016/j.scitotenv.2023.168627"
"   Xiao X, 2017, INT BIODETER BIODEGR, V116, P10, DOI 10.1016/j.ibiod.2016.09.021"
"   Xie YP, 2011, APPL ENVIRON MICROB, V77, P2325, DOI 10.1128/AEM.02149-10"
"   Xie YY, 2016, J ENVIRON MANAGE, V166, P478, DOI 10.1016/j.jenvman.2015.10.042"
"   Yadav VK, 2024, PART PART SYST CHAR, V41, DOI 10.1002/ppsc.202400007"
"   Yan A, 2020, FRONT PLANT SCI, V11, DOI 10.3389/fpls.2020.00359"
"   Yan XX, 2022, SCI TOTAL ENVIRON, V803, DOI 10.1016/j.scitotenv.2021.149871"
"   Yao ZT, 2012, PROCEDIA ENVIRON SCI, V16, P722, DOI 10.1016/j.proenv.2012.10.099"
"   Zandi P, 2022, BIOLOGY-BASEL, V11, DOI 10.3390/biology11020155"
"   Zeng K, 2024, J HAZARD MATER, V465, DOI 10.1016/j.jhazmat.2023.133086"
"   Zhang XW, 2024, SCI TOTAL ENVIRON, V920, DOI 10.1016/j.scitotenv.2024.170951"
"   Zhao XW, 2024, SCI TOTAL ENVIRON, V946, DOI 10.1016/j.scitotenv.2024.174117"
"   Zheng XJ, 2024, ECOTOX ENVIRON SAFE, V284, DOI 10.1016/j.ecoenv.2024.116883"
"   Zuo YA, 2015, J HAZARD MATER, V285, P236, DOI 10.1016/j.jhazmat.2014.12.003"
NR 286
TC 21
Z9 21
U1 12
U2 19
PU ACADEMIC PRESS LTD- ELSEVIER SCIENCE LTD
PI LONDON
"PA 24-28 OVAL RD, LONDON NW1 7DX, the United Kingdom"
SN 0301-4797
EI 1095-8630
J9 J ENVIRON MANAGE
JI J. Environ. Manage.
PD SEP
PY 2025
VL 392
AR 126736
DI 10.1016/j.jenvman.2025.126736
EA AUG 2025
PG 26
WC Environmental Sciences
WE Science Citation Index Expanded (SCI-EXPANDED)
SC Environmental Sciences & Ecology
GA 5VR4U
UT WOS:001545444000008
PM 40752329
DA 2026-04-06
ER

PT C
"AU Kulkarni, K"
"   Jain, N"
"   Babu, GLS"
"AF Kulkarni, Kalyani"
"   Jain, Nehal"
"   Babu, G. L. Sivakumar"
"BE Farid, A"
"   Foye, K"
"   Khire, MV"
TI Effect of Alternate Cementation Reagent and Soil Moisture on
   Leachability of Heavy Metals in Soil Remediated Using Bacillus

subtilis
SO GEO-ENVIRONMEET 2025: GEOENVIRONMENTAL CONTAMINATION AND WASTE CONTAINMENT
SE Geotechnical Special Publication
LA English
DT Proceedings Paper
CT 2025 Geoenvironmental Engineering conference-GeoenvironMeet
"CY MAR 02-05, 2025"
"CL Louisville, KY"
SP American Society of Civil Engineers Geo-Institute
DE Bacterial Remediation; Heavy Metals; Bacillus subtilis
ID MECHANISMS
"AB Using bacteria to improve soil characteristics is a sustainable solution that is gaining popularity. The bacteria are often used in combination with cementation reagents (CR) comprising of calcium chloride (CaCl2) and urea. However, CaCl2, which is also used in soil washing of heavy metal-contaminated soils, showed an increase in the leachability of heavy metals. Hence, calcium hydroxide [Ca(OH)(2)] was experimented as an alternative source of Ca along with Bacillus subtilis and incubation at different moisture contents. Tests were conducted on the leachability of Cadmium (Cd), Nickel (Ni), and Zinc (Zn)- contaminated red soil and dredged sediment. While both were spiked with the same concentration of heavy metals and subjected to the same bacterial concentration, minimum leachability was observed after 4 days for 1M concentration when incubated at 100% moisture in the soil. Ca(OH)(2) was observed to reduce the leachability to the maximum extent for all these heavy metals."
"C1 [Kulkarni, Kalyani] Indian Inst Sci, Ctr Sustainable Technol, Bangalore, Karnataka, India."
"   [Jain, Nehal] Univ Illinois, Dept Civil & Environm Engn, Champaign, IL USA."
"   [Kulkarni, Kalyani; Babu, G. L. Sivakumar] Indian Inst Sci, Civil Engn Dept, Bangalore, Karnataka, India."
C3 Indian Institute of Science (IISC) - Bangalore; University of Illinois System; University of Illinois Urbana-Champaign; Indian Institute of Science (IISC) - Bangalore
"RP Kulkarni, K (corresponding author), Indian Inst Sci, Ctr Sustainable Technol, Bangalore, Karnataka, India."
EM kkalyani@iisc.ac.in; nehalj2@illinois.edu; gls@iisc.ac.in
"RI Babu, Sivakumar/AAK-5287-2020"
"CR Ahmad M, 2018, PEERJ, V6, DOI 10.7717/peerj.5122"
"   Al Disi Z, 2022, ENVIRON TECHNOL INNO, V27, DOI 10.1016/j.eti.2022.102513"
"   Bai J, 2014, J ENVIRON SCI-CHINA, V26, P2056, DOI 10.1016/j.jes.2014.07.015"
"   Bian HF, 2022, FRONT ECOL EVOL, V10, DOI 10.3389/fevo.2022.857185"
"   Kuramshina ZM, 2018, RUSS J PLANT PHYSL+, V65, P269, DOI 10.1134/S1021443718010077"
"   Lee LM, 2012, APPL MECH MATER, V204-208, P326, DOI 10.4028/www.scientific.net/AMM.204-208.326"
"   Lidder P, 2012, ADV GENET, V78, P1, DOI 10.1016/B978-0-12-394394-1.00001-8"
"   Pande V, 2019, SMART BIOREMEDIATION TECHNOLOGIES: MICROBIAL ENZYMES, P255, DOI 10.1016/B978-0-12-818307-6.00014-7"
"   Shi Y, 2023, SCI TOTAL ENVIRON, V865, DOI 10.1016/j.scitotenv.2022.161265"
"   Song HW, 2022, SCI TOTAL ENVIRON, V844, DOI 10.1016/j.scitotenv.2022.157167"
"   STARK JM, 1995, APPL ENVIRON MICROB, V61, P218, DOI 10.1128/AEM.61.1.218-221.1995"
"   Stres B, 2008, FEMS MICROBIOL ECOL, V66, P110, DOI 10.1111/j.1574-6941.2008.00555.x"
"   Torres-Aravena AE, 2018, CRYSTALS, V8, DOI 10.3390/cryst8110438"
"   Xie YL, 2021, J CLEAN PROD, V312, DOI 10.1016/j.jclepro.2021.127749"
"   Xu L, 2022, CHEMOSPHERE, V300, DOI 10.1016/j.chemosphere.2022.134581"
NR 15
TC 0
Z9 0
U1 0
U2 0
PU AMER SOC CIVIL ENGINEERS
PI NEW YORK
"PA UNITED ENGINEERING CENTER, 345 E 47TH ST, NEW YORK, NY 10017-2398 USA"
SN 0895-0563
BN 978-0-7844-8569-9
J9 GEOTECH SP
PY 2025
VL 359
BP 62
EP 70
PG 9
"WC Engineering, Environmental; Engineering, Civil; Engineering, Geological;" Environmental Sciences
WE Conference Proceedings Citation Index - Science (CPCI-S)
SC Engineering; Environmental Sciences & Ecology
GA BZ4SG
UT WOS:001587099700007
DA 2026-04-06
ER

PT J
"AU Pishchik, VN"
"   Vorob'ev, NI"
"   Provorov, NA"
"   Khomyakov, YV"
"AF Pishchik, V. N."
"   Vorob'ev, N. I."
"   Provorov, N. A."
"   Khomyakov, Yu. V."
TI Mechanisms of plant and microbial adaptation to heavy metals in

plant-microbial systems
SO MICROBIOLOGY
LA English
DT Review
DE plant-microbial system; heavy metals; adaptation
ID GROWTH-PROMOTING RHIZOBACTERIA; THLASPI-CAERULESCENS; MATHEMATICAL
SIMULATION; CADMIUM ACCUMULATION; RHIZOSPHERE BACTERIA;
PSEUDOMONAS-PUTIDA; WASTE-WATER; RESISTANCE; TOLERANCE; SOIL
"AB The data on heavy metal (HM) accumulation and detoxification by plants and bacteria in plant-microbial systems (PMS) are reviewed. Bacteria are shown to be the labile component of the system, responsible for a considerable amelioration of HM stress impact on plants and for improved PMS adaptation to heavy metals. Simulation of plant-microbial interactions under conditions of soil contamination by HM revealed the protective role of bacterial migration from the rhizoplane to the rhizosphere."
"C1 [Pishchik, V. N.; Khomyakov, Yu. V.] Inst Agrophys, St Petersburg, Russia."
" [Vorob'ev, N. I.; Provorov, N. A.] All Russia Res Inst Agr Microbiol, St Petersburg, Russia."
"RP Pishchik, VN (corresponding author), Inst Agrophys, St Petersburg, Russia."
EM veronika-bio@rambler.ru
"RI Provorov, Nikolai/AAB-3908-2021; Pishchik, Veronika/B-9029-2017"
"OI Provorov, Nikolai/0000-0001-9091-9384; Pishchik,"
Veronika/0000-0001-6422-4837
FU Russian Science Foundation [14-26-00094]; Russian Science Foundation
[14-26-00094] Funding Source: Russian Science Foundation
FX The team of the authors is grateful to I.N. Ktitorova for her
constructive proposals in the work on the manuscript. The work by N.I.
Vorobyev and N.A. Provorov was supported by the Russian Science
" Foundation, project no. 14-26-00094."
"CR Abou-Shanab RI, 2003, INT J PHYTOREMEDIAT, V5, P367, DOI [10.1080/16226510390268766, 10.1080/15226510309359043]"
" Ahemad Munees, 2015, Journal of Genetic Engineering and Biotechnology, V13, P51, DOI [10.1016/j.jgeb.2015.02.001, 10.1016/j.jgeb.2015.02.001]"
" Aleem A, 2003, BIORESOURCE TECHNOL, V86, P7, DOI 10.1016/S0960-8524(02)00134-7"
" Aleksakhin R.M., 2004, METODIKA OTSENKI EKO"
" Alekseev Yu. V., 2002, Agrokhimiya, P82"
" ALEKSEEV YV, 2008, TYAZHELYE METALLY AG"
" [Anonymous], SCI TOTAL ENV"
" [Anonymous], 1998, PLANTS HYPERACCUMULA, DOI DOI 10.1079/9780851992365.0000"
" Antonyuk LP, 2001, RUSS J PLANT PHYSL+, V48, P364, DOI 10.1023/A:1016622618524"
" Arshad M, 2007, TRENDS BIOTECHNOL, V25, P356, DOI 10.1016/j.tibtech.2007.05.005"
" Bais HP, 2006, ANNU REV PLANT BIOL, V57, P233, DOI 10.1146/annurev.arplant.57.032905.105159"
" BAKER A J M, 1989, Biorecovery, V1, P81"
" BAKER AJM, 1981, J PLANT NUTR, V3, P643, DOI 10.1080/01904168109362867"
" BARCELO J, 1990, J PLANT NUTR, V13, P1, DOI 10.1080/01904169009364057"
" Barkey T., 1985, APPL ENVIRON MICROB, V49, P337"
" BASZYNSKI T, 1980, PHYSIOL PLANTARUM, V48, P365, DOI 10.1111/j.1399-3054.1980.tb03269.x"
" Belimov A. A., 2011, Sel'skokhozyaistvennaya Biologiya, P10"
" Belimov AA, 2000, MICROBIOL RES, V155, P113, DOI 10.1016/S0944-5013(00)80046-4"
" Benabdellah K, 2009, NEW PHYTOL, V184, P682, DOI 10.1111/j.1469-8137.2009.02978.x"
" Bianco A., 2000, ENV BIOTECHNOLOGY CL"
" Bothe H., 2011, Detoxification of Heavy Metals, V30, P35, DOI DOI 10.1007/978-3-642-21408-0_2"
" Boyer M, 2009, FEMS MICROBIOL ECOL, V70, P1, DOI 10.1111/j.1574-6941.2009.00745.x"
" Breckle SW., 1991, Plant Root: The Hidden Half, V7, P351"
" Brencic A, 2005, MICROBIOL MOL BIOL R, V69, P155, DOI 10.1128/MMBR.69.1.155-194.2005"
" Bruins MR, 2000, ECOTOX ENVIRON SAFE, V45, P198, DOI 10.1006/eesa.1999.1860"
" Cailliatte R, 2009, BIOCHEM J, V422, P217, DOI 10.1042/BJ20090655"
" Callahan DL, 2006, J BIOL INORG CHEM, V11, P2, DOI 10.1007/s00775-005-0056-7"
" Cervantes C, 2001, FEMS MICROBIOL REV, V25, P335, DOI 10.1111/j.1574-6976.2001.tb00581.x"
" Chebotar V. K., 2007, EFFEKTIVNOST PRIMENE"
" Choudhury R, 2001, CURR SCI INDIA, V81, P768"
" Clemens S, 2001, PLANTA, V212, P475, DOI 10.1007/s004250000458"
" Delorme TA, 2001, CAN J MICROBIOL, V47, P773, DOI 10.1139/cjm-47-8-773"
" Derzhavin D.M., 1989, METODICHESKIE UKAZAN"
" Dmitrenko GN, 2003, MICROBIOLOGY+, V72, P327, DOI 10.1023/A:1024204200737"
" [???????€?????????? ??.??. Domracheva L.I.], 2009, [???????€?????????????????? ?? ???€???????????????? ???????????????, Teoreticheskaya i prikladnaya ekologiya], P4"
" Drazic G, 2009, ARCH BIOL SCI, V61, P431, DOI 10.2298/ABS0903431D"
" Ebbs S, 2002, PLANTA, V214, P635, DOI 10.1007/s004250100650"
" Ehrlich HL, 1997, APPL MICROBIOL BIOT, V48, P687, DOI 10.1007/s002530051116"
" El-Helow ER, 2000, BIOMETALS, V13, P273, DOI 10.1023/A:1009291931258"
" Ernst W., 1982, PFLANZENOKOLOGIE MIN, P472"
" Ernst W.H.O., 1974, SCHWERMETALLVEGETATI"
" Ernst W.H.O., 1998, Ecotoxicology, P587"
" Fabre B, 2003, ENVIRON CHEM LETT, V1, P141, DOI 10.1007/s10311-003-0025-8"
" Faisal M., 2004, BIOTECHNOL LETT, V26, P1623"
" Filali BK, 2000, CURR MICROBIOL, V41, P151, DOI 10.1007/s0028400"
" GADD GM, 1990, EXPERIENTIA, V46, P834, DOI 10.1007/BF01935534"
" Gerend??s J, 1998, J EXP BOT, V49, P1545, DOI 10.1093/jexbot/49.326.1545"
" Ghasemkheyli F. T., 2014, International Journal of Biosciences (IJB), V4, P294"
" Giller KE, 1998, SOIL BIOL BIOCHEM, V30, P1389, DOI 10.1016/S0038-0717(97)00270-8"
" Glick BR, 2010, BIOTECHNOL ADV, V28, P367, DOI 10.1016/j.biotechadv.2010.02.001"
" Glick BR, 1998, J THEOR BIOL, V190, P63, DOI 10.1006/jtbi.1997.0532"
" Godzik B., 1993, POL BOT STUD, V5, P113"
" Gorshkov V. Yu., 2009, THESIS"
" Grobelak A, 2015, ECOL ENG, V84, P22, DOI 10.1016/j.ecoleng.2015.07.019"
" Gupta A, 1999, NAT MED, V5, P183, DOI 10.1038/5545"

"   Lima AIG, 2006, ENZYME MICROB TECH, V39, P763, DOI 10.1016/j.enzmictec.2005.12.009"
"   Hall JL, 2003, J EXP BOT, V54, P2601, DOI 10.1093/jxb/erg303"
"   Hall JL, 2002, J EXP BOT, V53, P1, DOI 10.1093/jexbot/53.366.1"
"   HIGHAM DP, 1986, ENVIRON HEALTH PERSP, V65, P5, DOI 10.2307/3430155"
"   HOWDEN R, 1995, PLANT PHYSIOL, V107, P1059, DOI 10.1104/pp.107.4.1059"
"   Ignatov VV, 2005, MOL BASES RELATIONSH, P70"
"   Ilyaletdinov A. N., MICROBIOLOGY MOSCOW, V50, P135"
"   Ivanova EP, 2001, MICROBIOLOGY+, V70, P239, DOI 10.1023/A:1010498017807"
"   Iyer A, 2005, MAR POLLUT BULL, V50, P340, DOI 10.1016/j.marpolbul.2004.11.012"
"   Jing Yan-De, 2007, Journal of Zhejiang University-Science B, V8, P192, DOI 10.1631/jzus.2007.B0192"
"   KACABOVA P, 1986, PHOTOSYNTHETICA, V20, P411"
"   Karavaiko GI, 2006, MICROBIOLOGY+, V75, P512, DOI 10.1134/S002626170605002X"
"   Kaznina NM, 2014, RUSS J PLANT PHYSL+, V61, P355, DOI 10.1134/S1021443714030066"
"   KLOEPPER JW, 1989, TRENDS BIOTECHNOL, V7, P39, DOI 10.1016/0167-7799(89)90057-7"
"   Kositsin A. V., 1991, USTOICHIVOST TYAZHEL, P15"
"   Kulaev I.S., 1996, SOROS OBRAZ ZH, P28"
"   Kulaeva O. A., 2011, RUSS J GENET APPL RE, V1, P349, DOI DOI 10.1134/S2079059711050108"
"   Kuzmicheva YV, 2014, RUSS J PLANT PHYSL+, V61, P112, DOI 10.1134/S1021443714010087"
"   Ladonin D.V., 2007, SOVREMENNYE PROBLEMY, V2, P203"
"   Lane TW, 2005, NATURE, V435, P42, DOI 10.1038/435042a"
"   Lanquar V, 2010, PLANT PHYSIOL, V152, P1986, DOI 10.1104/pp.109.150946"
"   Lasat MM, 2002, J ENVIRON QUAL, V31, P109, DOI 10.2134/jeq2002.0109"
"   Lebedeva A. F., 2003, BIOLOGY, P29"
"   Lee SW, 2001, APPL ENVIRON MICROB, V67, P1437, DOI 10.1128/AEM.67.4.1437-1444.2001"
"   LEPP NW, 1977, Z PFLANZENPHYSIOL, V84, P363, DOI 10.1016/S0044-328X(77)80150-5"
"   Llamas A, 2008, PLANT PHYSIOL BIOCH, V46, P905, DOI 10.1016/j.plaphy.2008.05.006"
"   Lombi E, 2000, NEW PHYTOL, V145, P11, DOI 10.1046/j.1469-8137.2000.00560.x"
"   LOVLEY DR, 1988, APPL ENVIRON MICROB, V54, P1472, DOI 10.1128/AEM.54.6.1472-1480.1988"
"   Mari S, 2006, TOP CURR GENET, V14, P273"
"   McDougald D, 2007, ANAL BIOANAL CHEM, V387, P445, DOI 10.1007/s00216-006-0761-2"
"   MEHRA RK, 1995, BIOCHEM BIOPH RES CO, V215, P730, DOI 10.1006/bbrc.1995.2524"
"   Mendoza-C??zatl DG, 2005, BBA-BIOENERGETICS, V1706, P88, DOI 10.1016/j.bbabio.2004.09.010"
"   Mengoni A, 2001, ENVIRON MICROBIOL, V3, P691, DOI 10.1046/j.1462-2920.2001.00243.x"
"   MURAKAMI T, 1989, CHEM LETT, P2137, DOI 10.1246/cl.1989.2137"
"   Nies DH, 2003, FEMS MICROBIOL REV, V27, P313, DOI 10.1016/S0168-6445(03)00048-2"
"   Nies DH, 1999, APPL MICROBIOL BIOT, V51, P730, DOI 10.1007/s002530051457"
"   NIES DH, 1995, J BACTERIOL, V177, P2707, DOI 10.1128/jb.177.10.2707-2712.1995"
"   Nikiforova EM, 2009, EURASIAN SOIL SCI+, V42, P874, DOI 10.1134/S1064229309080055"
"   Ona O, 2005, FEMS MICROBIOL LETT, V246, P125, DOI 10.1016/j.femsle.2005.03.048"
"   Parker DL, 2000, J APPL PHYCOL, V12, P219, DOI 10.1023/A:1008195312218"
"   P??rez-Figueroa A, 2010, J EVOLUTION BIOL, V23, P2267, DOI 10.1111/j.1420-9101.2010.02093.x"
"   [???????????? ??.??. Phockina A.I.], 2008, [???????€?????????????????? ?? ???€????????????????
???????????????, Teoreticheskaya i prikladnaya ekologiya], P4"
"   Pirog T.P., 1999, MIKROBIOL ZH, V61, P64"
"   Pishchik VN, 2015, EURASIAN SOIL SCI+, V48, P77, DOI 10.1134/S1064229315010135"
"   Pishchik V. N., 1997, Mikrobiologiya, V66, P54"
"   Pishchik V. N., 1999, P INT C REG PLANT GR, V2, P232"
"   Pishchik VN, 2005, MICROBIOLOGY+, V74, P735, DOI 10.1007/s11021-005-0132-6"
"   Pishchik VN, 1998, PLANT SOIL, V202, P49, DOI 10.1023/A:1004356431849"
"   Pishchik VN, 2002, PLANT SOIL, V243, P173, DOI 10.1023/A:1019941525758"
"   Polevoi V.V., 1989, FIZIOLOGIYA RASTENII"
"   Prapadgee B., 2015, INT J ENV S IN PRESS"
"   Rajkumar M, 2010, TRENDS BIOTECHNOL, V28, P142, DOI 10.1016/j.tibtech.2009.12.002"
"   Rauser WE, 1999, CELL BIOCHEM BIOPHYS, V31, P19, DOI 10.1007/BF02738153"
"   Rensing C, 1999, J BACTERIOL, V181, P5891, DOI 10.1128/JB.181.19.5891-5897.1999"
"   Roane T.M., 1999, ENVIRON MICROBIOL, P403"
"   Roane TM, 1999, MICROB ECOL, V38, P358, DOI 10.1007/s002489901001"
"   Roane TM, 1996, CAN J MICROBIOL, V42, P593, DOI 10.1139/m96-080"
"   Ruchko R. V., 1984, THESIS"
"   Ryan PR, 2001, ANNU REV PLANT PHYS, V52, P527, DOI 10.1146/annurev.arplant.52.1.527"
"   Sabry SA, 1997, J APPL MICROBIOL, V82, P245, DOI 10.1111/j.1365-2672.1997.tb02858.x"
"   Safronova VI, 2006, BIOL FERT SOILS, V42, P267, DOI 10.1007/s00374-005-0024-y"
"   SALT DE, 1995, PLANT PHYSIOL, V107, P1293, DOI 10.1104/pp.107.4.1293"
"   SALT DE, 1995, BIO-TECHNOL, V13, P468, DOI 10.1038/nbt0595-468"
"   Sanjaya, 2008, PLANT CELL ENVIRON, V31, P1074, DOI 10.1111/j.1365-3040.2008.01819.x"
"   Satoh-Nagasawa N, 2012, PLANT CELL PHYSIOL, V53, P213, DOI 10.1093/pcp/pcr166"
"   Seregin IV, 2001, RUSS J PLANT PHYSL+, V48, P523, DOI 10.1023/A:1016719901147"
"   Sessitsch A, 2013, SOIL BIOL BIOCHEM, V60, P182, DOI 10.1016/j.soilbio.2013.01.012"
"   Shaposhnikov A. I., 2011, Sel'skokhozyaistvennaya Biologiya, P16"
"   Shcherbakov A. V., 2011, DOSTIZH NAUK TEKHN A, V7, P61"
"   Singh V., 2010, Int J Pharm Sci Rev Res, V3, P164"
"   Slobodkina GB, 2007, MICROBIOLOGY+, V76, P530, DOI 10.1134/S0026261707050037"
"   SMILDE KW, 1981, PLANT SOIL, V62, P3, DOI 10.1007/BF02205020"
"   Smith SE, 1998, HEREDITY, V80, P760, DOI 10.1046/j.1365-2540.1998.00351.x"
"   Taboonma, 2014, P INT C FUT TRENDS B, P15"
"   Tak HI, 2013, REV ENVIRON CONTAM T, V223, P33, DOI 10.1007/978-1-4614-5577-6_2"
"   Takagi Sei-Ichi, 1993, P111"
"   Teitzel GM, 2003, APPL ENVIRON MICROB, V69, P2313, DOI 10.1128/AEM.69.4.2313-2320.2003"
"   Thomine S, 2003, PLANT J, V34, P685, DOI 10.1046/j.1365-313X.2003.01760.x"
"   Tikhonovich I.A., 2009, SIMBIOZY RASTENII MI"
"   Titov A.F., 2007, USTOICHIVOST RASTENI"
"   Turkovskaya O. V., 2005, MOLEKULYARNYE OSNOVY, P180"
"   Ueno D, 2010, P NATL ACAD SCI USA, V107, P16500, DOI 10.1073/pnas.1005396107"
"   Ullah A, 2015, ENVIRON EXP BOT, V117, P28, DOI 10.1016/j.envexpbot.2015.05.001"

"   Vangronsveld J., 1998, INTRO CONCEPTS METAL, P1"
"   Vats N, 2001, MICROBIOL-UK, V147, P653, DOI 10.1099/00221287-147-3-653"
"   Viti C, 2003, CURR MICROBIOL, V46, P1, DOI 10.1007/s00284-002-3800-z"
"   Vodyanitskii Yu N., 2013, Agrokhimiya, P88"
"   Wang QYD, 2001, SOIL SEDIMENT CONTAM, V10, P497, DOI 10.1080/20015891109392"
"   Webb R., 1999, J Hazard Subst Res, V2, P1, DOI DOI 10.4148/1090-7025.1011"
"   Whiting SN, 2001, ENVIRON SCI TECHNOL, V35, P3144, DOI 10.1021/es001938v"
"   WIERZBICKA M, 1994, ENVIRON EXP BOT, V34, P173, DOI 10.1016/0098-8472(94)90036-1"
"   Willems G, 2007, GENETICS, V176, P659, DOI 10.1534/genetics.106.064485"
"   WILLIAMS CH, 1973, AUST J SOIL RES, V11, P43, DOI 10.1071/SR9730043"
"   Williams LE, 2000, BBA-BIOMEMBRANES, V1465, P104, DOI 10.1016/S0005-2736(00)00133-4"
"   Wong CKE, 2009, NEW PHYTOL, V181, P71, DOI 10.1111/j.1469-8137.2008.02638.x"
"   Yahya M., 2014, Trends in Bioinformatics, V7, P19, DOI DOI 10.3923/TB.2014.19.26"
"   Yaneva O. D., 2009, MIKROBIOL ZH, V71, P53"
"   Yang YY, 2000, PLANT PHYSIOL, V124, P1019, DOI 10.1104/pp.124.3.1019"
"   Zaripova NR, 2011, RUSS J PLANT PHYSL+, V58, P1040, DOI 10.1134/S1021443711060203"
"   Zha HG, 2004, NEW PHYTOL, V163, P299, DOI 10.1111/j.1469-8137.2004.01113.x"
"   Zhang SJ, 2012, ENVIRON SCI POLLUT R, V19, P3879, DOI 10.1007/s11356-012-1060-4"
NR 156
TC 31
Z9 37
U1 0
U2 124
PU MAIK NAUKA/INTERPERIODICA/SPRINGER
PI NEW YORK
"PA 233 SPRING ST, NEW YORK, NY 10013-1578 USA"
SN 0026-2617
EI 1608-3237
J9 MICROBIOLOGY+
JI Microbiology
PD MAY
PY 2016
VL 85
IS 3
BP 257
EP 271
DI 10.1134/S0026261716030097
PG 15
WC Microbiology
WE Science Citation Index Expanded (SCI-EXPANDED)
SC Microbiology
GA DP8UO
UT WOS:000378773600001
DA 2026-04-06
ER

PT J
"AU Chellaiah, ER"
"AF Chellaiah, Edward Raja"
TI Cadmium (heavy metals) bioremediation by Pseudomonas aeruginosa:
   a minireview
SO APPLIED WATER SCIENCE
LA English
DT Review
DE Bioremediation; Cadmium; Pseudomonas aeruginosa; Resistance bacteria;
   Mechanisms
ID GROWTH-PROMOTING RHIZOBACTERIA; PHOTOSYNTHETIC ACTIVITY; OXIDATIVE
   STRESS; RESISTANT; SOIL; LEAD; BACTERIA; BIOSORPTION; PLASMID;
   IDENTIFICATION
"AB Heavy metal pollution has become an issue of serious international concern. One of the heavy metal cadmium (Cd) is known to be a widespread environmental contaminant and a potent toxin that may adversely affect human health. Microbial remediation has been applied as an efficient strategy to remove or detoxify the heavy metals mainly from soil, water and sediments, etc. Pseudomonas aeruginosa is one of the most significant bacterium present in almost all contaminated sites. They are often resistant to antibiotics, heavy metals, detergents and organic solvents. This review concluded that P. aeruginosa is one of the versatile and high-tolerance cadmium-resistant bacteria isolated from different environment regimens. It can be used as suitable biosorbent for the removal of cadmium and other heavy metals from solution, contaminated waste, water and soil. Apart from this characteristic, P. aeruginosa, used as potent bioinoculant, express Plant growth promoting rhizobacteria (PGPR) activity, biofilm and biosurfactant production, and comprise key role in metal phytoextraction process."
"C1 [Chellaiah, Edward Raja] Madurai Kamaraj Univ, Sch Biol Sci, Dept Mol Biol, Madurai 625021, Tamil Nadu, India."
C3 Madurai Kamaraj University
"RP Chellaiah, ER (corresponding author), Madurai Kamaraj Univ, Sch Biol Sci, Dept Mol Biol, Madurai 625021, Tamil Nadu, India."
EM edwardrajac@gmail.com
"OI Chellaih, Edward Raja/0000-0002-5559-7946"
"CR Abbas S.Z., 2014, J WASTE MANAG, V2014, P6, DOI [10.1155/2014/160398, DOI 10.1155/2014/160398]"
"   Abo-Amer Aly E, 2006, Roum Arch Microbiol Immunol, V65, P113"
"   Adesemoye AO, 2008, BRAZ J MICROBIOL, V39, P423, DOI 10.1590/S1517-83822008000300003"
"   Adesemoye Anthony O., 2009, Archives of Phytopathology and Plant Protection, V42, P188, DOI 10.1080/03235400601014791"
"   Ahemad M, 2010, ARCH ENVIRON CON TOX, V58, P361, DOI 10.1007/s00244-009-9382-z"
"   Aka RJN, 2016, INT J PHYTOREMEDIAT, V18, P200, DOI 10.1080/15226514.2015.1073671"

"   Alkorta I., 2004, Reviews in Environmental Science and Bio/Technology, V3, P71, DOI 10.1023/B:RESB.0000040059.70899.3d"
"   BEACH LR, 1981, P NATL ACAD SCI-BIOL, V78, P2110, DOI 10.1073/pnas.78.4.2110"
"   Benmalek Y., 2014, Advances in Bioscience and Biotechnology, V5, P718"
"   Beveridge T.J., 1989, METAL IONS BACTERIA, P1"
"   BLOM A, 1992, APPL ENVIRON MICROB, V58, P331, DOI 10.1128/AEM.58.1.331-334.1992"
"   Bodour AA, 2003, APPL ENVIRON MICROB, V69, P3280, DOI 10.1128/AEM.69.6.3280-3287.2003"
"   Boj??rquez C, 2016, REV INT CONTAM AMBIE, V32, P407, DOI 10.20937/RICA.2016.32.04.04"
"   BOLLAG JM, 1979, J ENVIRON QUAL, V8, P196, DOI 10.2134/jeq1979.00472425000800020012x"
"   Brar S. K., 2006, Practice Periodical of Hazardous, Toxic and Radioactive Waste Management, V10, P59, DOI 10.1061/(ASCE)1090-025X(2006)10:2(59)"
"   Braud A, 2009, CHEMOSPHERE, V74, P280, DOI 10.1016/j.chemosphere.2008.09.013"
"   Brocklehurst KR, 2003, BIOCHEM BIOPH RES CO, V308, P234, DOI 10.1016/S0006-291X(03)01366-4"
"   Bullied WJ, 2002, CAN J PLANT SCI, V82, P291"
"   Bulut Y, 2007, J ENVIRON SCI, V19, P160, DOI 10.1016/S1001-0742(07)60026-6"
"   Chakraborty J, 2014, ENVIRON SCI POLLUT R, V21, P14188, DOI 10.1007/s11356-014-3308-7"
"   Chang JS, 1997, WATER RES, V31, P1651, DOI 10.1016/S0043-1354(97)00008-0"
"   Chen B, 2016, SCI TOTAL ENVIRON, V539, P17, DOI 10.1016/j.scitotenv.2015.08.117"
"   Chen L, 2014, SOIL BIOL BIOCHEM, V68, P300, DOI 10.1016/j.soilbio.2013.10.021"
"   Chien CC, 2013, BIOCHEM ENG J, V78, P132, DOI 10.1016/j.bej.2013.01.014"
"   Chien CC, 2011, ENVIRON TOXICOL CHEM, V30, P2202, DOI 10.1002/etc.620"
"   Chojnacka K, 2010, ENVIRON INT, V36, P299, DOI 10.1016/j.envint.2009.12.001"
"   Choudhary S, 2009, BIORESOURCE TECHNOL, V100, P2482, DOI 10.1016/j.biortech.2008.12.015"
"   Chovanov?? K, 2004, BIOLOGIA, V59, P817"
"   CLARKE PH, 1975, GENETICS BIOCH PSEUD, P263"
"   Coral G, 2006, POL J ENVIRON STUD, V15, P517"
"   Dell'Amico E, 2008, SOIL BIOL BIOCHEM, V40, P74, DOI 10.1016/j.soilbio.2007.06.024"
"   Drees KP, 2004, THESIS U ARIZ, P52"
"   Duruibe JO, 2007, INT J PHYS SCI, V2, P112"
"   Edward R. C., 2006, WORLD J MICROB BIOT, V22, P577, DOI DOI 10.1007/s11274-005-9074-4"
"   El-Sayed MS, 2008, AFR J BIOTECHNOL, V7, P2375"
"   Feng JP, 2010, SCI HORTIC-AMSTERDAM, V123, P521, DOI 10.1016/j.scienta.2009.10.013"
"   Filali BK, 2000, CURR MICROBIOL, V41, P151, DOI 10.1007/s0028400"
"   GELMI M, 1994, CURR MICROBIOL, V29, P335, DOI 10.1007/BF01570226"
"   G??ksungur Y, 2005, BIORESOURCE TECHNOL, V96, P103, DOI 10.1016/j.biortech.2003.04.002"
"   GREEN SK, 1974, APPL MICROBIOL, V28, P987, DOI 10.1128/AEM.28.6.987-991.1974"
"   Gupta A, 2002, CURR MICROBIOL, V45, P323, DOI 10.1007/s00284-002-3762-1"
"   Haritash AK, 2009, J HAZARD MATER, V169, P1, DOI 10.1016/j.jhazmat.2009.03.137"
"   Hassan MET, 1999, GENE, V238, P417, DOI 10.1016/S0378-1119(99)00349-2"
"   Hassan SHA, 2008, J BASIC MICROB, V48, P168, DOI 10.1002/jobm.200700338"
"   Hassen A, 1998, BIORESOURCE TECHNOL, V64, P7, DOI 10.1016/S0960-8524(97)00161-2"
"   HIGHAM DP, 1984, SCIENCE, V225, P1043, DOI 10.1126/science.225.4666.1043"
"   Hossain Mohammad Anwar, 2010, Physiology and Molecular Biology of Plants, V16, P19, DOI [10.1007/s12298-010-0003-0, 10.1007/s12298-010-0028-4]"
"   HOUBA C, 1980, MICROB ECOL, V6, P55, DOI 10.1007/BF02020375"
"   Huang JL, 2016, J GEN APPL MICROBIOL, V62, P258, DOI 10.2323/jgam.2016.04.007"
"   Ansari MI, 2007, BIORESOURCE TECHNOL, V98, P3149, DOI 10.1016/j.biortech.2006.10.008"
"   Izrael-Zivkovic L, 2018, RSC ADV, V8, P10549, DOI 10.1039/c8ra00371h"
"   JENNI B, 1989, J GEN MICROBIOL, V135, P461"
"   Joutey NT, 2015, REV ENVIRON CONTAM T, V233, P45, DOI 10.1007/978-3-319-10479-9_2"
"   Kabata-Pendias A., 2001, Trace Elements in Soil and Plants, VThird"
"   Karimpour M, 2018, DATA BRIEF, V18, P1185, DOI 10.1016/j.dib.2018.04.014"
"   Kassab DM, 2006, BIODEGRADATION, V17, P379, DOI 10.1007/s10532-005-9010-1"
"   Kassimghaima K., 2017, International Journal of Applied Environmental Sciences, V12, P1649"
"   Kermani AJN, 2010, IRAN J ENVIRON HEALT, V7, P279"
"   Khan NA, 2007, J AGRON CROP SCI, V193, P435, DOI 10.1111/j.1439-037X.2007.00272.x"
"   Khan NA, 2009, RUSS J PLANT PHYSL+, V56, P670, DOI 10.1134/S1021443709050136"
"   Kili?? NK, 2008, J HAZARD MATER, V154, P1019, DOI 10.1016/j.jhazmat.2007.11.008"
"   Kloepper J.W., 1978, Proceedings 4th International Conference on Plant Pathogenic Bacteria, P879"
"   Kloepper JW, 1999, AUSTRALAS PLANT PATH, V28, P21, DOI 10.1071/AP99003"
"   Konig-P??ter A, 2014, J SERB CHEM SOC, V79, P495, DOI 10.2298/JSC130314070K"
"   Krishna M. P., 2012, Environmental Research, Engineering and Management, V61, P58, DOI 10.5755/j01.erem.61.3.1268"
"   LADDAGA RA, 1985, J BACTERIOL, V162, P1100, DOI 10.1128/JB.162.3.1100-1105.1985"
"   Lederberg J.e.a., 2000, ENCY MICROBIOLOGY, V3, P876"
"   Lee SW, 2001, APPL ENVIRON MICROB, V67, P1437, DOI 10.1128/AEM.67.4.1437-1444.2001"
"   Lin XY, 2016, SCI TOTAL ENVIRON, V569, P97, DOI 10.1016/j.scitotenv.2016.06.121"
"   MACASKIE LE, 1987, J GEN MICROBIOL, V133, P539"
"   Maier RM, 2000, APPL MICROBIOL BIOT, V54, P625, DOI 10.1007/s002530000443"
"   Malik A, 2004, ENVIRON INT, V30, P261, DOI 10.1016/j.envint.2003.08.001"
"   Mangwani N, 2014, COLLOID SURFACE B, V114, P301, DOI 10.1016/j.colsurfb.2013.10.003"
"   MCENTEE JD, 1986, APPL ENVIRON MICROB, V51, P515, DOI 10.1128/AEM.51.3.515-520.1986"
"   MITRA RS, 1977, BIOCHEM BIOPH RES CO, V74, P1450, DOI 10.1016/0006-291X(77)90604-0"
"   Mobin M, 2007, J PLANT PHYSIOL, V164, P601, DOI 10.1016/j.jplph.2006.03.003"
"   Mohamed RM, 2012, J BASIC MICROB, V52, P53, DOI 10.1002/jobm.201100133"
"   Mongkolsuk S, 1997, FEMS MICROBIOL LETT, V146, P217, DOI 10.1111/j.1574-6968.1997.tb10196.x"
"   M??ller MM, 2012, J BIOTECHNOL, V162, P366, DOI 10.1016/j.jbiotec.2012.05.022"
"   Mulligan CN, 2005, ENVIRON POLLUT, V133, P183, DOI 10.1016/j.envpol.2004.06.009"
"   Muneer B, 2016, PAK J ZOOL, V48, P1495"
"   Naik MM, 2012, ECOTOX ENVIRON SAFE, V79, P129, DOI 10.1016/j.ecoenv.2011.12.015"
"   Naik MM, 2011, CURR MICROBIOL, V62, P409, DOI 10.1007/s00284-010-9722-2"
"   Nath S., 2014, J ENV ANAL TOXICOL, V4, P4"
"   Neilson JW, 2010, APPL MICROBIOL BIOT, V88, P953, DOI 10.1007/s00253-010-2808-8"
"   Neilson JW, 2003, J ENVIRON QUAL, V32, P899, DOI 10.2134/jeq2003.0899"

"   NIES A, 1989, J BACTERIOL, V171, P5065, DOI 10.1128/jb.171.9.5065-5070.1989"
"   NIES DH, 1989, J BACTERIOL, V171, P896, DOI 10.1128/jb.171.2.896-900.1989"
"   NIES DH, 1992, PLASMID, V27, P17, DOI 10.1016/0147-619X(92)90003-S"
"   Ochoa-Loza FJ, 2001, J ENVIRON QUAL, V30, P479, DOI 10.2134/jeq2001.302479x"
"   PACHECO SV, 1995, ANTON LEEUW INT J G, V67, P333, DOI 10.1007/BF00872931"
"   Pardo R, 2003, ANAL BIOANAL CHEM, V376, P26, DOI 10.1007/s00216-003-1843-z"
"   Park SM, 2006, J MICROBIOL, V44, P255"
"   PELLETT S, 1983, APPL ENVIRON MICROB, V45, P328, DOI 10.1128/AEM.45.1.328-332.1983"
"   Raja CE, 2009, INT J ENVIRON SCI TE, V6, P259"
"   Raja CE, 2012, J BASIC MICROB, V52, P175, DOI 10.1002/jobm.201000408"
"   Raja CE, 2008, BIOLOGIA, V63, P461, DOI 10.2478/s11756-008-0095-y"
"   Rajkumar M, 2008, CHEMOSPHERE, V71, P834, DOI 10.1016/j.chemosphere.2007.11.038"
"   Sanit?? di Toppi L, 1999, ENVIRON EXP BOT, V41, P105, DOI 10.1016/S0098-8472(98)00058-6"
"   Sheng XF, 2008, ENVIRON POLLUT, V156, P1164, DOI 10.1016/j.envpol.2008.04.007"
"   Shi PL, 2016, WATER AIR SOIL POLL, V227, DOI 10.1007/s11270-016-3167-6"
"   Silver S, 1996, ANNU REV MICROBIOL, V50, P753, DOI 10.1146/annurev.micro.50.1.753"
"   Sinha S, 2009, BRAZ J MICROBIOL, V40, P655, DOI 10.1590/S1517-838220090003000030"
"   Spain A., 2003, RUR, V2, P1"
"   Tang X, 2018, SCI TOTAL ENVIRON, V636, P1355, DOI 10.1016/j.scitotenv.2018.04.229"
"   Tang XY, 2006, ENVIRON INT, V32, P682, DOI 10.1016/j.envint.2006.03.003"
"   TYNECKA Z, 1981, J BACTERIOL, V147, P313, DOI 10.1128/JB.147.2.313-319.1981"
"   Vel??squez L, 2009, J HAZARD MATER, V167, P713, DOI 10.1016/j.jhazmat.2009.01.044"
"   Vijayaraghavan K, 2008, BIOTECHNOL ADV, V26, P266, DOI 10.1016/j.biotechadv.2008.02.002"
"   Volesky, 2001, PROCESS METALL, V11B, P69"
"   Wackett L P, 2000, Environ Microbiol, V2, P348, DOI 10.1046/j.1462-2920.2000.00125.x"
"   Wang CL, 1997, APPL ENVIRON MICROB, V63, P4075, DOI 10.1128/AEM.63.10.4075-4078.1997"
"   Wuertz S., 1997, Modern soil microbiology., P607"
"   Xia Y, 2002, INT J ENVIRON STUD, V5, P1"
"   Yang X, 2005, J TRACE ELEM MED BIO, V18, P339, DOI 10.1016/j.jtemb.2005.02.007"
"   Zeng XX, 2009, J CENT SOUTH UNIV T, V16, P416, DOI 10.1007/s11771-009-0070-y"
"   Zeng XX, 2012, CURR MICROBIOL, V65, P799, DOI 10.1007/s00284-012-0224-2"
"   [????????€ Zhang Yuxiu], 2010, [????????????, Journal of China Coal Society], V35, P1735"
"   Ziagova M, 2007, BIORESOURCE TECHNOL, V98, P2859, DOI 10.1016/j.biortech.2006.09.043"
NR 122
TC 181
Z9 228
U1 6
U2 220
PU SPRINGER HEIDELBERG
PI HEIDELBERG
"PA TIERGARTENSTRASSE 17, D-69121 HEIDELBERG, GERMANY"
SN 2190-5487
EI 2190-5495
J9 APPL WATER SCI
JI Appl. Water Sci.
PD OCT
PY 2018
VL 8
IS 6
AR 154
DI 10.1007/s13201-018-0796-5
PG 10
WC Water Resources
WE Science Citation Index Expanded (SCI-EXPANDED)
SC Water Resources
GA GT3AP
UT WOS:000444372400001
"OA Green Submitted, gold"
DA 2026-04-06
ER

PT J
"AU Paul, T"
"   Saha, NC"
"AF Paul, Tanmoy"
"   Saha, Nimai Chandra"
TI Environmental Arsenic and Selenium Contamination and Approaches Towards
   Its Bioremediation Through the Exploration of Microbial Adaptations: A
   Review
SO PEDOSPHERE
LA English
DT Review
DE bioaccumulation; biogeochemistry; chemical species; contaminant;
   decontamination; heavy metal; microbial remediation; microorganism
ID ENTEROBACTER-CLOACAE SLD1A-1; HEALTH-RISK ASSESSMENT; FOOD-CHAIN;
   DRINKING-WATER; PUBLIC-HEALTH; HEAVY-METALS; WEST-BENGAL; REDUCTION;
   SOIL; BACTERIUM
"AB Bioaccumulation of selenium and arsenic resulting from geogenic activities and aggravated by anthropogenic actions has now become a serious environmental issue. Different parts of the world are experiencing issues of selenium and arsenic contamination, especially in soil and groundwater. The present selenium and arsenic contamination scenario has been well represented in contemporary literature, and there are an array of remediation techniques targeting the decontamination of environmental contamination. The use of microbe-mediated remediation of selenium and arsenic contamination is gaining scientific attention for sustainable environmental bioremediation. The present review elucidates the different aspects of selenium and

arsenic contamination, bioaccumulation, and bioremediation with implications for successful decontamination of these heavy metals."
"C1 [Paul, Tanmoy] Natl Inst Cholera & Enter Dis, Kolkata 700010, India."
" [Saha, Nimai Chandra] Univ Burdwan, Fishery & Ecotoxicol Res Lab, Vice Chancellors Res Grp, Dept Zool, Burdwan 713104, W Bengal, India."
C3 Indian Council of Medical Research (ICMR); ICMR - National Institute of
Cholera & Enteric Diseases (NICED); University of Burdwan
"RP Saha, NC (corresponding author), Univ Burdwan, Fishery & Ecotoxicol Res Lab, Vice Chancellors Res Grp, Dept Zool, Burdwan 713104, W Bengal, India."
EM prof.ncsaha@gmail.com
"RI Saha, Nimai Chana/ABH-1455-2020"
"OI Saha, Nimai Chana/0000-0001-5171-3762"
"CR Abbas SZ, 2014, BRAZ J MICROBIOL, V45, P1309, DOI 10.1590/S1517-83822014000400022"
" Abul Fazal M, 2001, WATER INT, V26, P380, DOI 10.1080/02508060108686930"
" Acharyya SK, 1999, NATURE, V401, P545, DOI 10.1038/44052"
" Achour AR, 2007, RES MICROBIOL, V158, P128, DOI 10.1016/j.resmic.2006.11.006"
" Andrahennadi R, 2007, ENVIRON SCI TECHNOL, V41, P7683, DOI 10.1021/es071399v"
" Andren A.W., 1975, Environmental Science and Technology, V9, P856, DOI DOI 10.1021/ES60107A002"
" [Anonymous], [No title captured]"
" [Anonymous], ENV HLTH CONTROL ASP"
" [Anonymous], 2003, TECHN SUMM INF AV BI"
" [Anonymous], 1937, PUBLIC HEALTH REP 1"
" [Anonymous], P S SEL TELL ENV IND"
" [Anonymous], WATER QUALITY TREATM"
" [Anonymous], DRINK WAT SPEC 2 REV"
" [Anonymous], 2018, Guidelines for Drinking Water Quality"
" [Anonymous], WHO TECHN REP SER"
" [Anonymous], 2011, DETOXIFICATION HEAVY"
" [Anonymous], [No title captured]"
" Argos Maria, 2012, Reviews on Environmental Health, V27, P191, DOI 10.1515/reveh-2012-0021"
" Avenda??o R, 2016, SCI REP-UK, V6, DOI 10.1038/srep37155"
" Awual MR, 2015, SENSOR ACTUAT B-CHEM, V209, P194, DOI 10.1016/j.snb.2014.11.010"
" Azubuike CC, 2016, WORLD J MICROB BIOT, V32, DOI 10.1007/s11274-016-2137-x"
" Bahar MM, 2012, BIODEGRADATION, V23, P803, DOI 10.1007/s10532-012-9567-4"
" Bajaj M, 2012, MICROB CELL FACT, V11, DOI 10.1186/1475-2859-11-64"
" Benbrahim-Tallaa L, 2005, TOXICOL APPL PHARM, V206, P288, DOI 10.1016/j.taap.2004.11.017"
" Bentley R, 2002, MICROBIOL MOL BIOL R, V66, P250, DOI 10.1128/MMBR.66.2.250-271.2002"
" Bhattacharjee Hiranmoy, 2008, Journal of Biology (London), V7, P33, DOI 10.1186/jbiol91"
" BOPP BA, 1982, DRUG METAB REV, V13, P271, DOI 10.3109/03602538209030000"
" Caussy D, 2003, ECOTOX ENVIRON SAFE, V56, P164, DOI 10.1016/S0147-6513(03)00059-9"
" Caussy D, 2008, REV ENVIRON CONTAM T, V197, P1, DOI 10.1007/978-0-387-79284-2_1"
" Chaalal O, 2005, ENERG SOURCE, V27, P87, DOI 10.1080/00908310490448136"
" Chakraborti D, 2003, ENVIRON HEALTH PERSP, V111, P1194, DOI 10.1289/ehp.5966"
" Chakraborti D, 2009, MOL NUTR FOOD RES, V53, P542, DOI 10.1002/mnfr.200700517"
" Chou WC, 2001, J CLIN INVEST, V108, P1541, DOI 10.1172/JCI200114064"
" Chung J, 2006, ENVIRON SCI TECHNOL, V40, P1664, DOI 10.1021/es051251g"
" Combs G.F., 1986, ROLE SELENIUM NUTR"
" Crognale S, 2019, SCI TOTAL ENVIRON, V651, P93, DOI 10.1016/j.scitotenv.2018.09.176"
" De Gieter M, 2002, ARCH ENVIRON CON TOX, V43, P406, DOI 10.1007/s00244-002-1193-4"
" Dembitsky VM, 2004, PROG LIPID RES, V43, P403, DOI 10.1016/j.plipres.2004.07.001"
" Dennis Lemly A., 2002, SELENIUM ASSESSMENT, P3, DOI [10.1007/978-1-4613-0073-1_1, DOI 10.1007/978-1-4613-0073-1_1]"
" Dey U, 2017, ENVIRON PROCESS, V4, P183, DOI 10.1007/s40710-016-0201-z"
" Dhanjal S, 2010, MICROB CELL FACT, V9, DOI 10.1186/1475-2859-9-52"
" Draize J. H., 1935, Journal of the American Veterinary Medical Association, V86, P753"
" Dubois M, 2009, ENVIRON POLLUT, V157, P772, DOI 10.1016/j.envpol.2008.11.022"
" Eswayah AS, 2016, APPL ENVIRON MICROB, V82, P4848, DOI 10.1128/AEM.00877-16"
" EVANS DW, 1980, JAPCA J AIR WASTE MA, V30, P567, DOI 10.1080/00022470.1980.10465982"
" Fairweather-Tait SJ, 2011, ANTIOXID REDOX SIGN, V14, P1337, DOI 10.1089/ars.2010.3275"
" Fawell J, 2003, BRIT MED BULL, V68, P199, DOI 10.1093/bmb/ldg027"
" Fendorf S, 2008, WILEY-IUPAC SER BIOP, P313"
" Fendorf S, 2010, SCIENCE, V328, P1123, DOI 10.1126/science.1172974"
" Fern??ndez-Laso V, 2017, SCI REP-UK, V7, DOI 10.1038/srep46679"
" Foster S, 2006, ENVIRON CHEM, V3, P304, DOI 10.1071/EN06026"
" Francesconi KA, 1998, SCI TOTAL ENVIRON, V221, P139, DOI 10.1016/S0048-9697(98)00272-1"
" Francesconi KA, 1997, ADV INORG CHEM, V44, P147"
" Garousi F, 2015, Acta Agraria Debreceniensis, P33, DOI [10.34101/actaagrar/64/1859, DOI 10.34101/ACTAAGRAR/64/1859, 10.34101/actaagrar/64/1859]"
" Gentry PR, 2010, ENVIRON MOL MUTAGEN, V51, P1, DOI 10.1002/em.20505"
" Goessler W, 1997, FRESEN J ANAL CHEM, V359, P434, DOI 10.1007/s002160050605"
" Govarthanan M, 2018, ECOTOX ENVIRON SAFE, V151, P273, DOI 10.1016/j.ecoenv.2018.01.020"
" Govarthanan M, 2016, 3 BIOTECH, V6, DOI 10.1007/s13205-016-0560-1"
" Govarthanan M, 2015, CURR MICROBIOL, V71, P618, DOI 10.1007/s00284-015-0897-4"
" Govarthanan M, 2015, CURR MICROBIOL, V71, P311, DOI 10.1007/s00284-015-0850-6"
" Govarthanan M, 2015, RES MICROBIOL, V166, P196, DOI 10.1016/j.resmic.2015.02.007"
" Harms H, 2011, NAT REV MICROBIOL, V9, P177, DOI 10.1038/nrmicro2519"
" Hartikainen H, 2005, J TRACE ELEM MED BIO, V18, P309, DOI 10.1016/j.jtemb.2005.02.009"
" HARTLEY WJ, 1961, FED PROC, V20, P679"
" He YZ, 2018, ENVIRON RES, V164, P288, DOI 10.1016/j.envres.2018.02.037"
" Hillwalker WE, 2006, SCI TOTAL ENVIRON, V366, P367, DOI 10.1016/j.scitotenv.2005.12.024"
" Hlihor RM, 2017, ENHANCING CLEANUP OF ENVIRONMENTAL POLLUTANTS, VOL 1: BIOLOGICAL APPROACHES, P289, DOI 10.1007/978-3-319-55426-6_14"
" Hoshino YT, 2008, SOIL SCI PLANT NUTR, V54, P701, DOI 10.1111/j.1747-0765.2008.00289.x"
" Hu S, 2012, J ENVIRON SCI-CHINA, V24, P1341, DOI 10.1016/S1001-0742(11)60922-4"

"   Ilialetdinov A N, 1981, Mikrobiologiia, V50, P197"
"   INNES JRM, 1969, JNCI-J NATL CANCER I, V42, P1101"
"   IPCS (International Programme on Chemical Safety), 2001, ENV HLTH CRIT 224 AR"
"   Khoei NS, 2017, NEW BIOTECHNOL, V34, P1, DOI 10.1016/j.nbt.2016.10.002"
"   Kirby J, 2002, APPL ORGANOMET CHEM, V16, P192, DOI 10.1002/aoc.283"
"   Korotkevych O, 2011, FEMS MICROBIOL ECOL, V78, P137, DOI 10.1111/j.1574-6941.2011.01169.x"
"   Kuiper I, 2004, MOL PLANT MICROBE IN, V17, P6, DOI 10.1094/MPMI.2004.17.1.6"
"   Lampis S, 2014, MICROB CELL FACT, V13, DOI 10.1186/1475-2859-13-35"
"   Lee KH, 2012, MOL MED REP, V5, P299, DOI 10.3892/mmr.2011.651"
"   LEITA L, 1995, BIOL FERT SOILS, V19, P103, DOI 10.1007/BF00336144"
"   Lemly AD, 2004, ECOTOX ENVIRON SAFE, V59, P44, DOI 10.1016/S0147-6513(03)00095-2"
"   LEMLY AD, 1993, ENVIRON MONIT ASSESS, V28, P83, DOI 10.1007/BF00547213"
"   LEMLY AD, 1985, REGUL TOXICOL PHARM, V5, P465"
"   Lemly AD, 1997, ECOTOX ENVIRON SAFE, V36, P275, DOI 10.1006/eesa.1996.1515"
"   Liao VHC, 2011, J CONTAM HYDROL, V123, P20, DOI 10.1016/j.jconhyd.2010.12.003"
"   Losi ME, 1997, APPL ENVIRON MICROB, V63, P3079, DOI 10.1128/AEM.63.8.3079-3084.1997"
"   Lu XL, 2018, J HAZARD MATER, V359, P527, DOI 10.1016/j.jhazmat.2018.07.112"
"   Malik A, 2004, ENVIRON INT, V30, P261, DOI 10.1016/j.envint.2003.08.001"
"   Mandal BK, 2002, TALANTA, V58, P201, DOI 10.1016/S0039-9140(02)00268-0"
"   Mandal Paramita, 2017, Emerging Contaminants, V3, P17, DOI [10.1016/j.emcon.2017.01.004, 10.1016/j.emcon.2017.01.004]"
"   McGuinness M, 2009, INT J ENV RES PUB HE, V6, P2226, DOI 10.3390/ijerph6082226"
"   Mehdi Y, 2013, MOLECULES, V18, P3292, DOI 10.3390/molecules18033292"
"   Mukhopadhyay R, 2002, FEMS MICROBIOL REV, V26, P311, DOI 10.1111/j.1574-6976.2002.tb00617.x"
"   Muscatello JR, 2009, SCI TOTAL ENVIRON, V407, P1318, DOI 10.1016/j.scitotenv.2008.10.046"
"   Muscatello JR, 2008, ENVIRON POLLUT, V156, P387, DOI 10.1016/j.envpol.2008.01.039"
"   Nancharaiah YV, 2015, TRENDS BIOTECHNOL, V33, P323, DOI 10.1016/j.tibtech.2015.03.004"
"   Nelson AA, 1943, CANCER RES, V3, P230"
"   Newman DK, 1998, GEOMICROBIOL J, V15, P255, DOI 10.1080/01490459809378082"
"   Nickson R, 1998, NATURE, V395, P338, DOI 10.1038/26387"
"   Nishimura T, 2007, SEPAR SCI TECHNOL, V42, P3155, DOI 10.1080/01496390701513107"
"   Nordstrom DK, 2002, SCIENCE, V296, P2143, DOI 10.1126/science.1072375"
"   OHLENDORF HM, 1988, J TOXICOL ENV HEALTH, V24, P67, DOI 10.1080/15287398809531141"
"   OHLENDORF HM, 1986, SCI TOTAL ENVIRON, V52, P49, DOI 10.1016/0048-9697(86)90104-X"
"   Oremland RS, 2009, GEOMICROBIOL J, V26, P522, DOI 10.1080/01490450903102525"
"   Oremland RS, 2003, SCIENCE, V300, P939, DOI 10.1126/science.1081903"
"   Oremland RS, 2005, TRENDS MICROBIOL, V13, P45, DOI 10.1016/j.tim.2004.12.002"
"   Orr PL, 2006, ECOTOX ENVIRON SAFE, V63, P175, DOI 10.1016/j.ecoenv.2005.09.004"
"   Painter EP, 1941, CHEM REV, V28, P179, DOI 10.1021/cr60090a001"
"   Pan X., 2018, 20 YEARS RES DEV SOI, P477, DOI [10.1007/978-981-10-6029-8_28, DOI 10.1007/978-981-10-6029-8_28]"
"   Paul T, 2018, HEAVY METALS IN THE ENVIRONMENT: MICROORGANISMS AND BIOREMEDIATION, P73"
"   Paul T, 2018, PEDOSPHERE, V28, P299, DOI 10.1016/S1002-0160(17)60493-4"
"   Pond GJ, 2008, J N AM BENTHOL SOC, V27, P717, DOI 10.1899/08-015.1"
"   PRESSER TS, 1994, ENVIRON MANAGE, V18, P423, DOI 10.1007/BF02393871"
"   Ravenscroft P., 2009, ARSENIC POLLUTION GL"
"   Rhine ED, 2006, ENVIRON MICROBIOL, V8, P899, DOI 10.1111/j.1462-2920.2005.00977.x"
"   Rodriguez MJM, 2005, ENVIRON GEOCHEM HLTH, V27, P513, DOI 10.1007/s10653-005-8625-9"
"   Rosen BP, 1999, TRENDS MICROBIOL, V7, P207, DOI 10.1016/S0966-842X(99)01494-8"
"   Rosen BP, 2002, FEBS LETT, V529, P86, DOI 10.1016/S0014-5793(02)03186-1"
"   Rosenfeld CE, 2017, GEOBIOLOGY, V15, P441, DOI 10.1111/gbi.12224"
"   Roychowdhury R, 2018, B ENVIRON CONTAM TOX, V101, P527, DOI 10.1007/s00128-018-2428-z"
"   Sakamoto M, 2015, ENVIRON RES, V143, P55, DOI 10.1016/j.envres.2015.09.010"
"   Sakamoto M, 2012, ECOTOX ENVIRON SAFE, V84, P179, DOI 10.1016/j.ecoenv.2012.07.014"
"   SAKURAI H, 1975, ENVIRON PHYSIOL BIOC, V5, P107"
"   Salt DE, 1998, ANNU REV PLANT PHYS, V49, P643, DOI 10.1146/annurev.arplant.49.1.643"
"   Satyapal Ghanshyam Kumar, 2018, Biotechnology Reports, V17, P117, DOI [10.1016/j.btre.2018.02.002, 10.1016/j.btre.2018.02.002]"
"   SEIFTER J, 1946, SCIENCE, V103, P762, DOI 10.1126/science.103.2687.762"
"   Selvankumar T, 2017, 3 BIOTECH, V7, DOI 10.1007/s13205-017-0901-8"
"   Shankar S, 2014, J MECH MED BIOL, V14, DOI 10.1142/S0219519414500419"
"   Sharma A.K., 2006, Environ. Geoscinces, V1, P17, DOI DOI 10.1306/EG.07310404035"
"   Shi HL, 2004, MOL CELL BIOCHEM, V255, P67, DOI 10.1023/B:MCBI.0000007262.26044.e8"
"   Silver S, 2005, APPL ENVIRON MICROB, V71, P599, DOI 10.1128/AEM.71.2.599-608.2005"
"   Singh M, 2015, J APPL MICROBIOL, V119, P1278, DOI 10.1111/jam.12948"
"   Singh N, 2016, CHEMOSPHERE, V164, P524, DOI 10.1016/j.chemosphere.2016.08.119"
"   Smedley PL, 2002, APPL GEOCHEM, V17, P517, DOI 10.1016/S0883-2927(02)00018-5"
"   Smith AH, 2002, SCIENCE, V296, P2145, DOI 10.1126/science.1072896"
"   Srivastava PK, 2011, SCI TOTAL ENVIRON, V409, P2430, DOI 10.1016/j.scitotenv.2011.03.002"
"   Stewart AR, 2004, ENVIRON SCI TECHNOL, V38, P4519, DOI 10.1021/es0499647"
"   Stolz John., 2010, Microbe, V5, P53, DOI [10.1128/microbe.5.53.1, DOI 10.1128/MICROBE.5.53.1, DOI 10.1128/MICROBE.5.53.1]"
"   Su SM, 2010, CURR MICROBIOL, V61, P261, DOI 10.1007/s00284-010-9605-6"
"   Sun HJ, 2014, ENVIRON INT, V69, P148, DOI 10.1016/j.envint.2014.04.019"
"   Suzuki KT, 2007, BBA-GEN SUBJECTS, V1770, P1053, DOI 10.1016/j.bbagen.2007.03.007"
"   Tapio S, 2006, MUTAT RES-REV MUTAT, V612, P215, DOI 10.1016/j.mrrev.2006.02.001"
"   Uchino T, 2006, FOOD CHEM TOXICOL, V44, P455, DOI 10.1016/j.fct.2005.08.018"
"   Ur??k M, 2016, CLEAN-SOIL AIR WATER, V44, P610, DOI 10.1002/clen.201500100"
"   VALENTINE JL, 1988, ENVIRON RES, V45, P16, DOI 10.1016/S0013-9351(88)80003-3"
"   Nguyen VK, 2016, BIORESOURCE TECHNOL, V212, P182, DOI 10.1016/j.biortech.2016.04.033"
"   Vinceti M, 2017, MOL MED REP, V15, P3323, DOI 10.3892/mmr.2017.6377"
"   Vinceti M, 2013, SCI TOTAL ENVIRON, V443, P633, DOI 10.1016/j.scitotenv.2012.11.025"
"   Wang SL, 2006, ENVIRON GEOCHEM HLTH, V28, P197, DOI 10.1007/s10653-005-9032-y"
"   Wang TS, 2001, FREE RADICAL BIO MED, V31, P321, DOI 10.1016/S0891-5849(01)00581-0"

"   Wang YT, 2018, INT J MOL SCI, V19, DOI 10.3390/ijms19092799"
"   Weeger W, 1999, BIOMETALS, V12, P141, DOI 10.1023/A:1009255012328"
"   Wenzel W.W., 2013, Heavy Metals in Soils: Trace Metals and Metalloids in soils and their bioavailability, P241, DOI DOI 10.1007/978-94-007-4470-7_9"
"   Xia X, 2018, J HAZARD MATER, V342, P499, DOI 10.1016/j.jhazmat.2017.08.051"
"   Yang HC, 2012, CURR TOP MEMBR, V69, P325, DOI 10.1016/B978-0-12-394390-3.00012-4"
"   Yee N, 2007, APPL ENVIRON MICROB, V73, P1914, DOI 10.1128/AEM.02542-06"
"   Yoshida T, 2004, TOXICOL APPL PHARM, V198, P243, DOI 10.1016/j.taap.2003.10.022"
"   Zhang TC, 2003, CARCINOGENESIS, V24, P1811, DOI 10.1093/carcin/bgg141"
"   Zhao FJ, 2010, ANNU REV PLANT BIOL, V61, P535, DOI 10.1146/annurev-arplant-042809-112152"
"   Zhao FJ, 2002, NEW PHYTOL, V156, P27, DOI 10.1046/j.1469-8137.2002.00493.x"
NR 159
TC 32
Z9 36
U1 10
U2 250
PU SCIENCE PRESS
PI BEIJING
"PA 16 DONGHUANGCHENGGEN NORTH ST, BEIJING 100717, PEOPLES R CHINA"
SN 1002-0160
EI 2210-5107
J9 PEDOSPHERE
JI Pedosphere
PD OCT
PY 2019
VL 29
IS 5
BP 554
EP 568
DI 10.1016/S1002-0160(19)60829-5
PG 15
WC Soil Science
WE Science Citation Index Expanded (SCI-EXPANDED)
SC Agriculture
GA JA1LL
UT WOS:000487578400002
DA 2026-04-06
ER

PT J
"AU Zhao, S"
"   Yuan, XT"
"   Wang, XH"
"   Ai, YJ"
"   Li, FP"
"AF Zhao, Shuai"
"   Yuan, Xue-Tao"
"   Wang, Xiao-Hong"
"   Ai, Yan-Jun"
"   Li, Fu-Ping"
TI Research Progress and Hotspots in Microbial Remediation for Polluted
   Soils
SO SUSTAINABILITY
LA English
DT Review
DE microorganisms; soil remediation; CiteSpace; heavy metals; PAHs
ID POLYCYCLIC AROMATIC-HYDROCARBONS; HEAVY-METAL TOLERANCE; CONTAMINATED
   SOILS; ORGANIC POLLUTANTS; DEGRADATION; PETROLEUM; BIOREMEDIATION;
   DIVERSITY; OXYGEN; PAHS
"AB Microbial remediation has become a prominent focus in soil pollution control due to its environmental friendliness, cost-effectiveness, and high efficiency. The effectiveness of microbial remediation is rooted in the interactions between microbial metabolic activities and the soil environment. Various microorganisms employ distinct mechanisms for pollutant treatment, including surface adsorption, intracellular accumulation, and biomineralization. Using the Web of Science Core Collection database, tools such as CiteSpace 6.1.R6, VOSviewer 1.6.20, and HistCite Pro were employed to conduct a quantitative analysis of several key aspects: the volume and thematic distribution of research papers on microbial remediation of soils, the cooperative networks between countries and institutions, the leading journals, major research hotspots, and emerging trends. The analysis reveals that utilizing microbial regulatory mechanisms and functions to remediate inorganic pollutants, such as heavy metals, and organic pollutants, such as PAHs, is becoming a significant frontier in future research. This study provides a valuable reference for scholars aiming to understand the current status of microbial research in soil remediation, both domestically and internationally. It also offers guidance for developing efficient, sustainable, and safe remediation strategies while identifying directions for future innovative research. The specific results are as follows: (1) China, the USA, India, and other countries have a high frequency of citations in this field, and the research is more in-depth. (2) More and more attention has been paid to the use of microbial remediation of contaminated soil in the world, mainly in Environmental Sciences. (3) Major publications include Chemosphere, Journal of Hazardous Materials, and Science of The Total Environment. In the key literature, the use of microorganisms to restore the soil environment and the combination of microorganisms and plants to repair soil contaminated by heavy metals occupy a high proportion. (4) The key areas of focus include the application of microorganisms in soil inorganic pollution remediation, the application of microorganisms in remediation of soil organic pollution (crude oil and polycyclic aromatic hydrocarbons (PAHs)), and the contribution of microorganisms to soil pollutant degradation and toxicity assessment systems. The research and development of combined microbial remediation technology is the current research hotspot in the field of soil remediation, focusing on the symbiosis between mycorrhizal fungi and plant roots, the enhancement in the ability of microorganisms to

absorb and degrade pollutants and their tolerance, and the interaction mechanism between indigenous microorganisms and plants."
"C1 [Zhao, Shuai; Yuan, Xue-Tao; Wang, Xiao-Hong; Ai, Yan-Jun; Li, Fu-Ping] North China Univ Sci & Technol, Coll Min Engn, Tangshan 063210, Peoples R China."
"   [Yuan, Xue-Tao; Wang, Xiao-Hong; Ai, Yan-Jun; Li, Fu-Ping] Hebei Key Lab Min Dev & Secur Technol, Tangshan 063210, Peoples R China."
"   [Yuan, Xue-Tao; Wang, Xiao-Hong; Ai, Yan-Jun; Li, Fu-Ping] Hebei Ind Technol Inst Mine Ecol Remediat, Tangshan 063210, Peoples R China."
C3 North China University of Science & Technology
"RP Yuan, XT; Wang, XH (corresponding author), North China Univ Sci & Technol, Coll Min Engn, Tangshan 063210, Peoples R China.; Yuan, XT; Wang, XH (corresponding author), Hebei Key Lab Min Dev & Secur Technol, Tangshan 063210, Peoples R China.; Yuan, XT; Wang, XH (corresponding author), Hebei Ind Technol Inst Mine Ecol Remediat, Tangshan 063210, Peoples R China."
EM 17332570724@163.com; yuanxuetao@ncst.edu.cn; glwxiaohong@163.com;
aiyanjun11@siian.com; tsxyhk@163.com
"RI ; Zhao, Shuai/F-1126-2019"
"OI Li, Fuping/0009-0007-8956-1929; "
FU National Natural Science Foundation of China; Hebei Industrial Technology Institute of Mine Ecological Remediation; Central Guided Local Science and Technology Development Fund Project of Hebei Province [246Z4201G]; [52274166]
FX This project was supported by the National Natural Science Foundation of
" China (No. 52274166), the Hebei Industrial Technology Institute of Mine"
" Ecological Remediation, and the Central Guided Local Science and"
Technology Development Fund Project of Hebei Province (Grant No. 246Z4201G).
"CR Ai YJ, 2022, SUSTAINABILITY-BASEL, V14, DOI 10.3390/su14159611"
" Aquino B, 2015, BRAZ J MED BIOL RES, V48, P683, DOI [10.1590/1414-431X20154522, 10.1590/1414-431x20154522]"
" Baoune H, 2019, J SOIL SEDIMENT, V19, P2222, DOI 10.1007/s11368-019-02259-w"
" Bolan N, 2014, J HAZARD MATER, V266, P141, DOI 10.1016/j.jhazmat.2013.12.018"
" Bollag J. M., 2008, Revista de la Ciencia del Suelo y Nutricion Vegetal, V8, P28"
" Buzmakov S, 2019, J SOIL SEDIMENT, V19, P38, DOI 10.1007/s11368-018-2025-0"
" Cao MH, 2013, ENVIRON POLLUT, V174, P93, DOI 10.1016/j.envpol.2012.11.015"
" Cerqueira VS, 2011, BIORESOURCE TECHNOL, V102, P11003, DOI 10.1016/j.biortech.2011.09.074"
" Chai XY, 2023, FRONT ENV SCI-SWITZ, V11, DOI 10.3389/fenvs.2023.1141562"
" Chaudhry Q, 2005, ENVIRON SCI POLLUT R, V12, P34, DOI 10.1065/espr2004.08.213"
" Chen M, 2015, BIOTECHNOL ADV, V33, P745, DOI 10.1016/j.biotechadv.2015.05.003"
" Chen WY, 2021, WATER-SUI, V13, DOI 10.3390/w13233404"
" Chunyan X, 2023, SCI TOTAL ENVIRON, V865, DOI 10.1016/j.scitotenv.2022.161112"
" Cui J, 2020, INT BIODETER BIODEGR, V150, DOI 10.1016/j.ibiod.2020.104964"
" Dai XL, 2023, ENVIRON SCI POLLUT R, V30, P93491, DOI 10.1007/s11356-023-29151-y"
" Damar S, 2006, J FOOD SCI, V71, pR1, DOI 10.1111/j.1365-2621.2006.tb12397.x"
" Deepika KV, 2016, ECOTOX ENVIRON SAFE, V134, P1, DOI 10.1016/j.ecoenv.2016.08.008"
" Fan RJ, 2022, J HAZARD MATER, V422, DOI 10.1016/j.jhazmat.2021.126959"
" Gerhardt KE, 2009, PLANT SCI, V176, P20, DOI 10.1016/j.plantsci.2008.09.014"
" Gong YX, 2022, J CLEAN PROD, V358, DOI 10.1016/j.jclepro.2022.132046"
" Guo C.L., 2016, Appl. Chem. Ind, V45, P1156"
" He YQ, 2022, ECOL INDIC, V141, DOI 10.1016/j.ecolind.2022.109145"
" Huang CC, 2021, PLOS ONE, V16, DOI 10.1371/journal.pone.0255137"
" Huang FX, 2022, SCI TOTAL ENVIRON, V836, DOI 10.1016/j.scitotenv.2022.155635"
" K??stner M, 2016, APPL MICROBIOL BIOT, V100, P3433, DOI 10.1007/s00253-016-7378-y"
" Kang CX, 2014, WORLD J MICROB BIOT, V30, P2733, DOI 10.1007/s11274-014-1697-x"
" Karakurt-Fischer S, 2023, WATER RES, V247, DOI 10.1016/j.watres.2023.120756"
" Kim SJ, 2008, BIODEGRADATION, V19, P859, DOI 10.1007/s10532-008-9189-z"
" Kobeticov?? K, 2015, CHEM LISTY, V109, P794"
" KREY R, 1992, MOL GEN GENET, V234, P433, DOI 10.1007/BF00538703"
" Kuppusamy S, 2017, CHEMOSPHERE, V168, P944, DOI 10.1016/j.chemosphere.2016.10.115"
" Lawniczak L, 2020, MOLECULES, V25, DOI 10.3390/molecules25040856"
" Lee KY, 2012, ENVIRON GEOCHEM HLTH, V34, P135, DOI 10.1007/s10653-011-9406-2"
" Lee SH, 2020, SUSTAINABILITY-BASEL, V12, DOI 10.3390/su122310078"
" Li CS, 2013, BIORESOURCE TECHNOL, V143, P46, DOI 10.1016/j.biortech.2013.05.114"
" Li D, 2021, J ENVIRON CHEM ENG, V9, DOI 10.1016/j.jece.2021.106098"
" Li D, 2021, SOIL SEDIMENT CONTAM, V30, P35, DOI 10.1080/15320383.2020.1787328"
" Li SN, 2023, ENVIRON RES, V219, DOI 10.1016/j.envres.2022.115132"
" Li SS, 2021, J CLEAN PROD, V294, DOI 10.1016/j.jclepro.2020.125350"
" Li SQ, 2024, CHEMOECOLOGY, V34, P47, DOI 10.1007/s00049-024-00399-1"
" Li WL, 2022, SCI TOTAL ENVIRON, V802, DOI 10.1016/j.scitotenv.2021.149802"
" Liang G, 2022, PROCESS SAF ENVIRON, V160, P610, DOI 10.1016/j.psep.2022.02.049"
" Liang T, 2023, J CLEAN PROD, V392, DOI 10.1016/j.jclepro.2023.136260"
" Liu J, 2023, J HAZARD MATER, V448, DOI 10.1016/j.jhazmat.2023.130855"
" Liu MX, 2017, PROG CHEM, V29, P1537, DOI 10.7536/PC170739"
" Lu HX, 2024, SCI TOTAL ENVIRON, V912, DOI 10.1016/j.scitotenv.2023.169425"
" Lv YF, 2022, ENERGY REP, V8, P7976, DOI 10.1016/j.egyr.2022.06.034"
" Macek I, 2016, ECOL ENG, V95, P330, DOI 10.1016/j.ecoleng.2016.06.026"
" Mi NA, 2022, RHIZOSPHERE-NETH, V24, DOI 10.1016/j.rhisph.2022.100604"
" Mumtaz MZ, 2024, FRONT MICROBIOL, V14, DOI 10.3389/fmicb.2023.1345161"
" Nejad ZD, 2018, ENVIRON GEOCHEM HLTH, V40, P927, DOI 10.1007/s10653-017-9964-z"
" Popescu G, 2009, BIOTECHNOL BIOTEC EQ, V23, P791, DOI 10.1080/13102818.2009.10818542"
" Qi YW, 2024, J HAZARD MATER, V469, DOI 10.1016/j.jhazmat.2024.133994"
" Qu JH, 2024, ACS EST ENG, V4, P2054, DOI 10.1021/acsestengg.4c00177"
" Qu JH, 2024, BIORESOURCE TECHNOL, V407, DOI 10.1016/j.biortech.2024.131075"
" Qu JH, 2024, CHEM ENG J, V490, DOI 10.1016/j.cej.2024.151662"
" Qu JH, 2023, P NATL ACAD SCI USA, V120, DOI 10.1073/pnas.2304552120"

"   Raiesi F, 2015, ECOL INDIC, V50, P173, DOI 10.1016/j.ecolind.2014.11.008"
"   Rusanescu CO, 2023, APPL SCI-BASEL, V13, DOI 10.3390/app13053246"
"   Shen YY, 2023, HUM ECOL RISK ASSESS, V29, P427, DOI 10.1080/10807039.2022.2102460"
"   Shi L, 2022, ANN MICROBIOL, V72, DOI 10.1186/s13213-022-01679-3"
"   Singh NK, 2021, ENVIRON SCI POLLUT R, V28, P58819, DOI 10.1007/s11356-020-11705-z"
"   Song PP, 2022, SCI TOTAL ENVIRON, V838, DOI 10.1016/j.scitotenv.2022.156417"
"   Song QY, 2023, CHEM ENG J, V475, DOI 10.1016/j.cej.2023.146042"
"   Sreedevi PR, 2022, J WATER PROCESS ENG, V48, DOI 10.1016/j.jwpe.2022.102884"
"   Sun X, 2017, GEOPHYS RES LETT, V44, P7883, DOI 10.1002/2017GL074355"
"   Sun YB, 2011, J HAZARD MATER, V186, P2075, DOI 10.1016/j.jhazmat.2010.12.116"
"   van Beilen JB, 2003, OIL GAS SCI TECHNOL, V58, P427, DOI 10.2516/ogst:2003026"
"   Varjani SJ, 2017, BIORESOURCE TECHNOL, V223, P277, DOI 10.1016/j.biortech.2016.10.037"
"   Wang C, 2020, APPL ECOL ENV RES, V18, P2727, DOI 10.15666/aeer/1802_27272746"
"   Wang HM, 2020, ENVIRON RES, V186, DOI 10.1016/j.envres.2020.109582"
"   Wang JL, 2022, ECOTOX ENVIRON SAFE, V247, DOI 10.1016/j.ecoenv.2022.114262"
"   Wang M, 2023, FRONT MICROBIOL, V14, DOI 10.3389/fmicb.2023.1122278"
"   Wang WC, 2021, J AGR FOOD CHEM, V69, P13386, DOI 10.1021/acs.jafc.1c04121"
"   Wang WJ, 2022, CHEMOSPHERE, V307, DOI 10.1016/j.chemosphere.2022.135783"
"   Wesseldyke ES, 2015, ADV WATER RESOUR, V78, P112, DOI 10.1016/j.advwatres.2015.01.009"
"   Wu SH, 2019, J HAZARD MATER, V364, P108, DOI 10.1016/j.jhazmat.2018.09.094"
"   Xie WQ, 2023, ENVIRON SCI POLLUT R, V30, P30210, DOI 10.1007/s11356-022-24356-z"
"   [????????? Xu Yanbo], 2013, [??????????????????, Chinese Journal of Environmental Engineering], V7, P2763"
"   Yang LQ., 2011, ENV PROT OIL GAS FIE, V21, P24, DOI [10.3969/j.issn.1005-3158.2011.02.009, DOI 10.3969/J.ISSN.1005-3158.2011.02.009]"
"   Yannikos N, 2014, SOIL BIOL BIOCHEM, V70, P5, DOI 10.1016/j.soilbio.2013.11.025"
"   Yu JL, 2023, ENVIRON SCI POLLUT R, V30, P28010, DOI 10.1007/s11356-022-24211-1"
"   Zha FS, 2022, CHEMOSPHERE, V308, DOI 10.1016/j.chemosphere.2022.136422"
"   Zhang K, 2023, ENVIRON EARTH SCI, V82, DOI 10.1007/s12665-023-10899-y"
"   Zhao JT, 2021, ENVIRON POLLUT, V281, DOI 10.1016/j.envpol.2021.116994"
"   Zheng RJ, 2021, J HAZARD MATER, V412, DOI 10.1016/j.jhazmat.2021.125199"
NR 86
TC 9
Z9 9
U1 62
U2 276
PU MDPI
PI BASEL
"PA ST ALBAN-ANLAGE 66, CH-4052 BASEL, SWITZERLAND"
EI 2071-1050
J9 SUSTAINABILITY-BASEL
JI Sustainability
PD SEP
PY 2024
VL 16
IS 17
AR 7458
DI 10.3390/su16177458
PG 18
WC Green & Sustainable Science & Technology; Environmental Sciences;
   Environmental Studies
WE Science Citation Index Expanded (SCI-EXPANDED); Social Science Citation Index (SSCI)
SC Science & Technology - Other Topics; Environmental Sciences & Ecology
GA F7H4O
UT WOS:001311484800001
"OA Green Submitted, gold"
DA 2026-04-06
ER

PT J
"AU Mao, YB"
"   Tan, HF"
"   Wang, MM"
"   Jiang, TH"
"   Wei, HW"
"   Xu, WP"
"   Jiang, Q"
"   Bao, H"
"   Ding, YF"
"   Wang, FJ"
"   Zhu, C"
"AF Mao, Yangbin"
"   Tan, Haifeng"
"   Wang, Maomao"
"   Jiang, Tianheng"
"   Wei, Hewen"
"   Xu, Wenping"
"   Jiang, Qiong"
"   Bao, Hexigeduleng"
"   Ding, Yanfei"
"   Wang, Feijuan"
"   Zhu, Cheng"
TI Research Progress of Soil Microorganisms in Response to Heavy Metals in
   Rice

SO JOURNAL OF AGRICULTURAL AND FOOD CHEMISTRY
LA English
DT Article
DE microorganisms; rice; heavy metals; functional mechanisms
ID ARBUSCULAR MYCORRHIZAL FUNGI; PLANT-GROWTH; PSEUDOMONAS-AERUGINOSA;
CADMIUM ACCUMULATION; BACTERIAL COMMUNITY; OXIDATIVE STRESS; REDUCES CD;
RESISTANT; TOXICITY; L.
"AB Soil heavy-metal pollution leads to excessive heavy metals in rice and other food crops, which has caused serious impacts on the ecological environment and on human health. In recent years, environmental friendly treatment methods that reduce the bioavailability of heavy metals in soil by soil microorganisms improving the tolerance of heavy metals in rice and reducing the transfer of heavy metals from the roots to the above-ground parts of rice have attracted much attention. This paper reviews the role and mechanism of soil microorganisms in alleviating heavy-metal stress in rice at home and abroad in recent years. At present, microorganisms tolerant to heavy metals mainly include bacteria and fungi, and their mechanisms include the adsorption of heavy metals by microorganisms, the secretion of growth-promoting substances (growth hormone, ACC deaminase, IAA), changing the physical and chemical properties of the soil and the composition of the microbial community, changing the transport mode of heavy metals in soil, the improvement of the antioxidant capacity of rice, etc. Hence, soil microorganisms have good application value and prospects in rice and other crops. However, the vast majority of current research focuses on a single strain, the screening principles of strains are limited, the pathogenicities of the strains have not been evaluated, and there are still few field experiments under natural conditions. In the future, we should strengthen the action of soil microorganisms on rice in response to the above problems in heavy metals, to better promote the microbial remediation technology."
"C1 [Mao, Yangbin; Tan, Haifeng; Wang, Maomao; Jiang, Tianheng; Jiang, Qiong; Bao, Hexigeduleng; Ding, Yanfei; Wang, Feijuan; Zhu, Cheng] China Jiliang Univ, Coll Life Sci, Key Lab Specialty Agriprod Qual & Hazard Controll, Hangzhou 310018, Peoples R China."
"   [Wei, Hewen] Jinhua Inst Food & Drug Inspect & Testing, Jinhua 321000, Zhejiang, Peoples R China."
"   [Xu, Wenping] Armed Police Sergeant Sch, Hangzhou 310018, Peoples R China."
C3 China Jiliang University
"RP Wang, FJ; Zhu, C (corresponding author), China Jiliang Univ, Coll Life Sci, Key Lab Specialty Agriprod Qual & Hazard Controll, Hangzhou 310018, Peoples R China."
EM wfj0311@cjlu.edu.cn; pzhch@cjlu.edu.cn
"RI Wang, Maomao/KHZ-5415-2024; Bao, Hexigeduleng/LNQ-8754-2024"
"OI Bao, Hexigeduleng/0000-0002-7097-8616"
FU Jinhua Science and Technology Project [2020-2-035]; Wenling Science and
Technology Planned Agriculture Project [2020N0020002]; Natural Science
"   Foundation of Zhejiang Province, China [LQ18C020001, LY19C020001];"
Fundamental Research Funds for the Provincial Universities of Zhejiang
[2020YW26]
FX This work was supported by the following funding: Jinhua Science and
"   Technology Project (No.2020-2-035), Wenling Science and Technology"
"   Planned Agriculture Project (2020N0020002), the Natural Science"
"   Foundation of Zhejiang Province, China (LQ18C020001, LY19C020001), the"
Fundamental Research Funds for the Provincial Universities of Zhejiang
(2020YW26) and the Fundamental Research Funds for the Provincial
Universities of Zhejiang.
"CR Ahmad I, 2016, J PLANT GROWTH REGUL, V35, P303, DOI 10.1007/s00344-015-9534-5"
"   Alvarez-V??zquez LJ, 2019, APPL MATH MODEL, V73, P387, DOI 10.1016/j.apm.2019.04.011"
"   Ashraf S, 2019, ECOTOX ENVIRON SAFE, V174, P714, DOI 10.1016/j.ecoenv.2019.02.068"
"   Basha DC, 2012, INT J DEV NEUROSCI, V30, P343, DOI 10.1016/j.ijdevneu.2012.01.004"
"   Burd GI, 2000, CAN J MICROBIOL, V46, P237, DOI 10.1139/cjm-46-3-237"
"   Chen DM, 2019, J HAZARD MATER, V367, P447, DOI 10.1016/j.jhazmat.2018.12.111"
"   Chen L, 2013, J EXP BOT, V64, P1565, DOI 10.1093/jxb/ert031"
"   Chen XW, 2019, GEODERMA, V337, P749, DOI 10.1016/j.geoderma.2018.10.029"
"   Chen YM, 2016, APPL ENVIRON MICROB, V82, P1734, DOI 10.1128/AEM.03689-15"
"   Dai S, 2021, ENVIRON POLLUT, V284, DOI 10.1016/j.envpol.2021.117127"
"   De J, 2008, MAR BIOTECHNOL, V10, P471, DOI 10.1007/s10126-008-9083-z"
"   Deng FL, 2013, PLANT PHYSIOL, V163, P1353, DOI 10.1104/pp.113.226225"
"   Deng YN, 2017, POL J ENVIRON STUD, V26, P1011, DOI 10.15244/pjoes/67655"
"   Ding Z., 2016, THESIS HUAZHONG AGR"
"   Dong MF, 2016, PLANT SOIL, V404, P75, DOI 10.1007/s11104-016-2829-x"
"   Fan Mei-Yu, 2021, Journal of Agro-Environment Science, V40, P279"
"   Fang L., 2011, THESIS HUAZHONG AGR"
"   Ghosh A, 2021, ENVIRON SCI POLLUT R, V28, P21633, DOI 10.1007/s11356-020-11816-7"
"   Ghosh PK, 2018, CHEMOSPHERE, V211, P407, DOI 10.1016/j.chemosphere.2018.07.148"
"   Huang F, 2013, COLLOID SURFACE B, V107, P11, DOI 10.1016/j.colsurfb.2013.01.062"
"   Ijaz M, 2019, BRAZ J MICROBIOL, V50, P449, DOI 10.1007/s42770-019-00043-z"
"   Islam F, 2014, ECOTOX ENVIRON SAFE, V104, P285, DOI 10.1016/j.ecoenv.2014.03.008"
"   Jiang CY, 2008, CHEMOSPHERE, V72, P157, DOI 10.1016/j.chemosphere.2008.02.006"
"   [????????? JIANG Chunyu], 2008, [??????????????????, Acta Scientiae Circumstantiae], V28, P1961"
"   Kotoky R, 2019, ENVIRON SUSTAIN, V2, P135, DOI [10.1007/s42398-019-00055-3, 10.1007/s42398-019-00055-3, DOI 10.1007/S42398-019-00055-3]"
"   Li Hesheng, 2012, MODERN PLANT PHYSL, P2"
"   Li H, 2016, SCI TOTAL ENVIRON, V571, P1183, DOI 10.1016/j.scitotenv.2016.07.124"
"   Li J., 1997, SOUTHWEST CHINA J AG, P27"
"   [?????? Li Peng], 2019, [????????????????????????. ???????????????, Journal of Hunan Agricultural University. Natural Sciences], V45, P124"
"   Li Y, 2017, ECOTOX ENVIRON SAFE, V138, P56, DOI 10.1016/j.ecoenv.2016.12.024"
"   Lin XY, 2016, SCI TOTAL ENVIRON, V569, P97, DOI 10.1016/j.scitotenv.2016.06.121"
"   Liu YuLing Liu YuLing, 2019, Journal of Agro-Environment Science, V38, P1855, DOI 10.11654/jaes.2019-0261"
"   Manasi, 2015, INT J BIOL MACROMOL, V79, P300, DOI 10.1016/j.ijbiomac.2015.04.071"
"   Mesa-Mar??n J, 2018, FRONT PLANT SCI, V9, DOI 10.3389/fpls.2018.01500"
"   Mitra S, 2018, ECOTOX ENVIRON SAFE, V156, P183, DOI 10.1016/j.ecoenv.2018.03.001"

"   Nawaz A, 2020, FRONT MICROBIOL, V11, DOI 10.3389/fmicb.2020.02019"
"   Nookongbut P, 2018, ENVIRON SCI POLLUT R, V25, P36530, DOI 10.1007/s11356-018-3568-8"
"   Pramanik K, 2021, CHEMOSPHERE, V274, DOI 10.1016/j.chemosphere.2021.129819"
"   Pramanik K, 2018, J HAZARD MATER, V351, P317, DOI 10.1016/j.jhazmat.2018.03.009"
"   Pramanik K, 2017, ENVIRON SCI POLLUT R, V24, P24419, DOI 10.1007/s11356-017-0033-z"
"   Pramanik K, 2016, SOIL SEDIMENT CONTAM, V25, P313, DOI 10.1080/15320383.2016.1137861"
"   Punjee P, 2018, CAN J MICROBIOL, V64, P131, DOI 10.1139/cjm-2017-0198"
"   Qu Lu-lu, 2020, Yingyong Shengtai Xuebao, V31, P326, DOI 10.13287/j.1001-9332.202001.033"
"   Rajkumar M, 2012, BIOTECHNOL ADV, V30, P1562, DOI 10.1016/j.biotechadv.2012.04.011"
"   Rizvi A, 2017, CHEMOSPHERE, V185, P942, DOI 10.1016/j.chemosphere.2017.07.088"
"   Sahoo RK, 2021, 3 BIOTECH, V11, DOI 10.1007/s13205-021-02835-3"
"   Samanta S, 2022, J PLANT GROWTH REGUL, V41, P2210, DOI 10.1007/s00344-021-10432-0"
"   Sasaki A, 2014, J EXP BOT, V65, P6013, DOI 10.1093/jxb/eru340"
"   Shahid M, 2019, J APPL MICROBIOL, V126, P1708, DOI 10.1111/jam.14252"
"   Shahzad R, 2017, ACTA BIOL HUNG, V68, P175, DOI 10.1556/018.68.2017.2.5"
"   Shan SP, 2020, SCI TOTAL ENVIRON, V708, DOI 10.1016/j.scitotenv.2019.135224"
"   Shi JiYan Shi JiYan, 2004, China Environmental Science, V24, P316"
"   Shi ZJ, 2021, J HAZARD MATER, V403, DOI 10.1016/j.jhazmat.2020.123824"
"   SILVER S, 1989, TRENDS BIOCHEM SCI, V14, P76, DOI 10.1016/0968-0004(89)90048-0"
"   Singh N, 2016, J ENVIRON SCI, V48, P112, DOI 10.1016/j.jes.2015.12.034"
"   Singh N, 2016, ECOTOX ENVIRON SAFE, V125, P25, DOI 10.1016/j.ecoenv.2015.11.020"
"   Suksabye P, 2016, ENVIRON SCI POLLUT R, V23, P962, DOI 10.1007/s11356-015-4590-8"
"   Tan Gaowei, 2019, Chinese Journal of Applied and Environmental Biology, V25, P524, DOI 10.19675/j.cnki.1006-687x.2018.12006"
"   Treesubsuntorn C, 2018, ENVIRON SCI POLLUT R, V25, P25690, DOI 10.1007/s11356-017-9058-6"
"   Wang CR, 2020, CHEMOSPHERE, V252, DOI 10.1016/j.chemosphere.2020.126603"
"   Wang Dun-fei, 2019, Yingyong Shengtai Xuebao, V30, P2767, DOI 10.13287/j.1001-9332.201908.037"
"   [?????? Wang Li], 2014, [????????????????????????, Journal of Agro-Environment Science], V33, P1882"
"   Wang M, 2019, SCI TOTAL ENVIRON, V649, P413, DOI 10.1016/j.scitotenv.2018.08.174"
"   Wang R., 2011, THESIS CHINESE ACAD"
"   Weng B., 2014, ASIAN AGR RES, V6, P19"
"   Wu L., 2019, THESIS JINAN U"
"   Xiao X, 2019, CLEAN-SOIL AIR WATER, V47, DOI 10.1002/clen.201800382"
"   Xie Y, 2018, PLANT SOIL, V422, P409, DOI 10.1007/s11104-017-3465-9"
"   Xie Y, 2014, CHEMOSPHERE, V117, P786, DOI 10.1016/j.chemosphere.2014.10.037"
"   Xu P., 2016, THESIS HUNAN U"
"   Xu SZ, 2019, APPL MICROBIOL BIOT, V103, P3887, DOI 10.1007/s00253-019-09700-7"
"   Sun YW, 2021, BMC PHARMACOL TOXICO, V22, DOI 10.1186/s40360-021-00493-y"
"   You LX, 2021, ECOTOX ENVIRON SAFE, V211, DOI 10.1016/j.ecoenv.2021.111914"
"   Zhang XH, 2009, SOIL BIOL BIOCHEM, V41, P930, DOI 10.1016/j.soilbio.2008.08.011"
"   Zhou JY, 2020, ENVIRON POLLUT, V260, DOI 10.1016/j.envpol.2020.113990"
"   Zhu LL, 2018, ENVIRON SCI POLLUT R, V25, P35232, DOI 10.1007/s11356-018-3456-2"
NR 76
TC 53
Z9 63
U1 27
U2 321
PU AMER CHEMICAL SOC
PI WASHINGTON
"PA 1155 16TH ST, NW, WASHINGTON, DC 20036 USA"
SN 0021-8561
EI 1520-5118
J9 J AGR FOOD CHEM
JI J. Agric. Food Chem.
PD JUL 20
PY 2022
VL 70
IS 28
BP 8513
EP 8522
DI 10.1021/acs.jafc.2c01437
PG 10
"WC Agriculture, Multidisciplinary; Chemistry, Applied; Food Science &"
   Technology
WE Science Citation Index Expanded (SCI-EXPANDED)
SC Agriculture; Chemistry; Food Science & Technology
GA 6W8TU
UT WOS:000896000300001
PM 35816106
DA 2026-04-06
ER

PT J
"AU Tang, JY"
"   Zhang, JC"
"   Ren, LH"
"   Zhou, YY"
"   Gao, J"
"   Luo, L"
"   Yang, Y"
"   Peng, QH"
"   Huang, HL"
"   Chen, AW"
"AF Tang, Jiayi"

"   Zhang, Jiachao"
"   Ren, Liheng"
"   Zhou, Yaoyu"
"   Gao, Jun"
"   Luo, Lin"
"   Yang, Yuan"
"   Peng, Qinghui"
"   Huang, Hongli"
"   Chen, Anwei"
TI Diagnosis of soil contamination using microbiological indices: A review
   on heavy metal pollution
SO JOURNAL OF ENVIRONMENTAL MANAGEMENT
LA English
DT Review
DE Soil diagnosis; Microbiological indicator; Heavy metal; Microbial
   community
ID MICROBIAL COMMUNITY STRUCTURE; AMMONIA-OXIDIZING BACTERIA; SUBSTRATE
   UTILIZATION PATTERN; IN-SITU REMEDIATION; LONG-TERM IMPACT;
   ENZYME-ACTIVITIES; WASTE-WATER; CHEMICAL PROPERTIES; FUNCTIONAL GENES;
   FOREST SOILS
"AB Heavy metal contamination of soil has become a serious global issue because of their persistence in the environment and the non-biodegradable nature leading to their accumulation to toxic levels. In order to achieve early warning and prevent soil quality from deteriorating, it is necessary to select suitable indices to diagnose heavy metal pollution. Microbiological indices for monitoring soil pollution by heavy metals are gaining attention. However, the related researches are scattered, and critical review is imperative. This review is mainly to provide readers with an in-depth understanding of the merits and limitations of microbiological indices for heavy metals contaminated and remediated soils. Microbiological indicators include microbial abundance, community diversity and structure, functional activity. The changes of different microbiological indices and the mechanism of microbial response to heavy metal stress in soils are comprehensively summarized. Furthermore, research gaps and future directions of the microbial ecotoxicological diagnosis of soil contamination by heavy metals are also proposed and discussed."
"C1 [Tang, Jiayi; Zhang, Jiachao; Ren, Liheng; Zhou, Yaoyu; Luo, Lin; Yang, Yuan; Peng, Qinghui; Huang, Hongli; Chen, Anwei] Hunan Agr Univ, Coll Resources & Environm, Changsha 410128, Hunan, Peoples R China."
"   [Gao, Jun] Univ Arkansas Med Sci, Dept Biochem & Mol Biol, Little Rock, AR 72205 USA."
C3 Hunan Agricultural University; University of Arkansas System; University
   of Arkansas Medical Sciences
"RP Zhang, JC; Zhou, YY (corresponding author), Hunan Agr Univ, Coll Resources & Environm, Changsha 410128, Hunan, Peoples R China."
EM jiachao.zhang@163.com; zhouyy@hunau.edu.cn
"RI Chen, Anwei/LGY-0867-2024; GAO, JUN/K-3981-2014; tang,"
"   jiayi/KIJ-0077-2024; Zhou, Yaoyu/S-8248-2016; Ren, Liheng/HNQ-9759-2023"
"OI Ren, Liheng/0009-0008-4788-5527"
FU Hunan Key Scientific Research Project [2017SK2351]; National Key R&D
   Projects of China [2016YFC0403001]; National Natural Science Foundation
"   of China [51709103]; Natural Science Foundation of Hunan Province, China"
   [2018JJ3242]; Hong Kong Scholars Program [XJ2018029]; China Postdoctoral
   Science Foundation [2018M630901]
FX This work was jointly supported by the Hunan Key Scientific Research
   Project (Grant No. 2017SK2351) and the National Key R&D Projects of
"   China (Grant No. 2016YFC0403001), the National Natural Science"
"   Foundation of China (Grant No. 51709103), Natural Science Foundation of"
"   Hunan Province, China (Grant No. 2018JJ3242), Hong Kong Scholars Program"
"   (XJ2018029), China Postdoctoral Science Foundation (Grant No."
   2018M630901).
"CR Akmal M, 2005, CHEMOSPHERE, V60, P508, DOI 10.1016/j.chemosphere.2005.01.001"
"   [Anonymous], 2014, PLOS ONE, DOI DOI 10.1371/JOURNAL.PONE.0240886"
"   [Anonymous], 2015, MBIO, DOI DOI 10.1128/mBio.02288-14"
"   Barajas-Aceves M, 2005, BIORESOURCE TECHNOL, V96, P1405, DOI 10.1016/j.biortech.2004.09.013"
"   Bastida F, 2008, GEODERMA, V147, P159, DOI 10.1016/j.geoderma.2008.08.007"
"   Berg J, 2012, APPL ENVIRON MICROB, V78, P7438, DOI 10.1128/AEM.01071-12"
"   Bhattacharyya P, 2008, CHEMOSPHERE, V72, P543, DOI 10.1016/j.chemosphere.2008.03.035"
"   Bouskill NJ, 2010, ECOTOXICOLOGY, V19, P317, DOI 10.1007/s10646-009-0414-2"
"   Brochier-Armanet C, 2008, NAT REV MICROBIOL, V6, P245, DOI 10.1038/nrmicro1852"
"   Cavani L, 2016, J ENVIRON MANAGE, V182, P37, DOI 10.1016/j.jenvman.2016.07.050"
"   Chao S., 2014, ENV SKEPTICS CRITICS, V3, P24, DOI DOI 10.1037/A0036071"
"   Charlton A, 2016, ENVIRON POLLUT, V219, P1021, DOI 10.1016/j.envpol.2016.07.050"
"   Chen JH, 2014, FEMS MICROBIOL ECOL, V87, P164, DOI 10.1111/1574-6941.12212"
"   Chodak M, 2013, APPL SOIL ECOL, V64, P7, DOI 10.1016/j.apsoil.2012.11.004"
"   Dai J, 2004, APPL SOIL ECOL, V25, P99, DOI 10.1016/j.apsoil.2003.09.003"
"   Das S, 2011, PLANTA, V234, P139, DOI 10.1007/s00425-011-1376-1"
"   Deng LJ, 2015, APPL MICROBIOL BIOT, V99, P8259, DOI 10.1007/s00253-015-6662-6"
"   Desai C, 2009, RES MICROBIOL, V160, P1, DOI 10.1016/j.resmic.2008.10.003"
"   dos Santos JV, 2016, ENVIRON SCI POLLUT R, V23, P6735, DOI 10.1007/s11356-015-5904-6"
"   Feng G, 2018, BMC MICROBIOL, V18, DOI 10.1186/s12866-018-1152-5"
"   Fern??ndez-Calvi??o D, 2016, SOIL BIOL BIOCHEM, V96, P20, DOI 10.1016/j.soilbio.2016.01.010"
"   Fern??ndez-Calvi??o D, 2011, SOIL BIOL BIOCHEM, V43, P2324, DOI 10.1016/j.soilbio.2011.08.001"
"   Gao Y, 2010, ENVIRON EARTH SCI, V60, P603, DOI 10.1007/s12665-009-0200-8"
"   Ge SJ, 2015, CHEMOSPHERE, V140, P85, DOI 10.1016/j.chemosphere.2015.02.004"
"   Giller KE, 1998, SOIL BIOL BIOCHEM, V30, P1389, DOI 10.1016/S0038-0717(97)00270-8"
"   Golebiewski M, 2014, MICROB ECOL, V67, P635, DOI 10.1007/s00248-013-0344-7"
"   Gomez-Alvarez V, 2009, ISME J, V3, P1314, DOI 10.1038/ismej.2009.72"
"   G??mez-Sagasti MT, 2012, WATER AIR SOIL POLL, V223, P3249, DOI 10.1007/s11270-012-1106-8"
"   Griffiths BS, 2013, FEMS MICROBIOL REV, V37, P112, DOI 10.1111/j.1574-6976.2012.00343.x"
"   Hai B, 2009, APPL ENVIRON MICROB, V75, P4993, DOI 10.1128/AEM.02917-08"

"   Hallenbeck PC, 2016, GENOME ANNOUNCEMENTS, V4, DOI 10.1128/genomeA.01571-15"
"   Hou J, 2013, WATER RES, V47, P2285, DOI 10.1016/j.watres.2013.01.042"
"   Hu XF, 2014, J GEOCHEM EXPLOR, V147, P139, DOI 10.1016/j.gexplo.2014.08.001"
"   Huang ZQ, 2016, WORLD J MICROB BIOT, V32, DOI 10.1007/s11274-015-1990-3"
"   Hynes HM, 2012, SOIL BIOL BIOCHEM, V46, P18, DOI 10.1016/j.soilbio.2011.10.018"
"   Imperato M, 2003, ENVIRON POLLUT, V124, P247, DOI 10.1016/S0269-7491(02)00478-5"
"   Jiang JP, 2010, EUR J SOIL BIOL, V46, P18, DOI 10.1016/j.ejsobi.2009.10.001"
"   Kaplan H, 2014, BIOL FERT SOILS, V50, P735, DOI 10.1007/s00374-013-0890-7"
"   Khan S, 2007, J ENVIRON SCI, V19, P834, DOI 10.1016/S1001-0742(07)60139-9"
"   Khan S, 2010, ENVIRON SCI POLLUT R, V17, P288, DOI 10.1007/s11356-009-0134-4"
"   Klotz MG, 1997, FEMS MICROBIOL LETT, V150, P65, DOI 10.1016/S0378-1097(97)00098-0"
"   Kou SM, 2018, FRONT MICROBIOL, V9, DOI 10.3389/fmicb.2018.00366"
"   Kucharski J., 2012, J ELEM, V19, P929"
"   Lebrun JD, 2012, SOIL BIOL BIOCHEM, V49, P150, DOI 10.1016/j.soilbio.2012.02.027"
"   Leininger S, 2006, NATURE, V442, P806, DOI 10.1038/nature04983"
"   LEITA L, 1995, BIOL FERT SOILS, V19, P103, DOI 10.1007/BF00336144"
"   Li MY, 2019, SCI TOTAL ENVIRON, V651, P2166, DOI 10.1016/j.scitotenv.2018.10.152"
"   Li XF, 2009, PLANT SOIL, V324, P209, DOI 10.1007/s11104-009-9947-7"
"   Li XQ, 2017, ENVIRON POLLUT, V231, P908, DOI 10.1016/j.envpol.2017.08.057"
"   Liang Q, 2014, AGR WATER MANAGE, V135, P100, DOI 10.1016/j.agwat.2014.01.003"
"   Liao M, 2007, ECOTOX ENVIRON SAFE, V66, P217, DOI 10.1016/j.ecoenv.2005.12.013"
"   Liu Y, 2012, SUBST ABUSE TREAT PR, V7, DOI 10.1186/1747-597X-7-6"
"   Liu YR, 2010, ENVIRON SCI POLLUT R, V17, P1237, DOI 10.1007/s11356-010-0302-6"
"   Magalhaes CM, 2011, FEMS MICROBIOL ECOL, V77, P274, DOI 10.1111/j.1574-6941.2011.01107.x"
"   Malik S, 2008, ENVIRON INT, V34, P265, DOI 10.1016/j.envint.2007.09.001"
"   Mench M, 2006, ENVIRON POLLUT, V144, P24, DOI 10.1016/j.envpol.2006.01.014"
"   Mertens J, 2009, ISME J, V3, P916, DOI 10.1038/ismej.2009.39"
"   Moffett BF, 2003, FEMS MICROBIOL ECOL, V43, P13, DOI 10.1111/j.1574-6941.2003.tb01041.x"
"   Moreno JL, 2003, EUR J SOIL SCI, V54, P377, DOI 10.1046/j.1365-2389.2003.00533.x"
"   Morton-Bermea O, 2009, J GEOCHEM EXPLOR, V101, P218, DOI 10.1016/j.gexplo.2008.07.002"
"   Nwuche CO, 2008, INT J ENVIRON SCI TE, V5, P409, DOI 10.1007/BF03326036"
"   Ollivier J, 2012, MICROB ECOL, V64, P1038, DOI 10.1007/s00248-012-0078-y"
"   Pan J, 2011, ECOL ENG, V37, P1889, DOI 10.1016/j.ecoleng.2011.07.002"
"   Rajapaksha RMCP, 2004, APPL ENVIRON MICROB, V70, P2966, DOI 10.1128/AEM.70.5.2966-2973.2004"
"   Renella G, 2005, SOIL BIOL BIOCHEM, V37, P1498, DOI 10.1016/j.soilbio.2005.01.013"
"   Ribbons RR, 2016, SOIL BIOL BIOCHEM, V103, P181, DOI 10.1016/j.soilbio.2016.07.024"
"   Ruyters S, 2013, SOIL BIOL BIOCHEM, V58, P244, DOI 10.1016/j.soilbio.2012.12.003"
"   Ruyters S, 2010, SOIL BIOL BIOCHEM, V42, P1581, DOI 10.1016/j.soilbio.2010.05.036"
"   Sheik CS, 2012, PLOS ONE, V7, DOI 10.1371/journal.pone.0040059"
"   Shen TL, 2019, SCI TOTAL ENVIRON, V646, P940, DOI 10.1016/j.scitotenv.2018.07.223"
"   Shukurov N, 2006, ENVIRON INT, V32, P1, DOI 10.1016/j.envint.2004.12.003"
"   Singh BK, 2014, ENVIRON MICROBIOL, V16, P2408, DOI 10.1111/1462-2920.12353"
"   Sobolev Dmitri, 2008, Int J Environ Res Public Health, V5, P450"
"   Song B, 2017, ENVIRON INT, V105, P43, DOI 10.1016/j.envint.2017.05.001"
"   Song B, 2016, ARCH BIOCHEM BIOPHYS, V604, P167, DOI 10.1016/j.abb.2016.04.018"
"   Song JW, 2018, ENVIRON POLLUT, V243, P510, DOI 10.1016/j.envpol.2018.09.011"
"   Sun YB, 2013, ENVIRON SCI POLLUT R, V20, P3290, DOI 10.1007/s11356-012-1261-x"
"   Throb??ck IN, 2007, FEMS MICROBIOL LETT, V270, P189, DOI 10.1111/j.1574-6968.2007.00632.x"
"   Tipayno SC, 2018, ECOL EVOL, V8, P6157, DOI 10.1002/ece3.4170"
"   Valentine DL, 2007, NAT REV MICROBIOL, V5, P316, DOI 10.1038/nrmicro1619"
"   Wang YP, 2007, J ENVIRON SCI, V19, P848, DOI 10.1016/S1001-0742(07)60141-7"
"   Wang YP, 2007, ECOTOX ENVIRON SAFE, V67, P75, DOI 10.1016/j.ecoenv.2006.03.007"
"   Ward BB, 2009, NATURE, V461, P78, DOI 10.1038/nature08276"
"   Wittebolle L, 2009, NATURE, V458, P623, DOI 10.1038/nature07840"
"   Wu B, 2018, ECOTOX ENVIRON SAFE, V166, P116, DOI 10.1016/j.ecoenv.2018.09.076"
"   Wyszkowska J, 2006, POL J ENVIRON STUD, V15, P927"
"   Xian Y, 2015, CHEMOSPHERE, V139, P604, DOI 10.1016/j.chemosphere.2014.12.060"
"   Xiao XY, 2017, ECOTOX ENVIRON SAFE, V142, P200, DOI 10.1016/j.ecoenv.2017.03.047"
"   Yang JS, 2016, ENVIRON POLLUT, V213, P760, DOI 10.1016/j.envpol.2016.03.030"
"   Yang JY, 2014, WATER AIR SOIL POLL, V225, DOI 10.1007/s11270-014-2012-z"
"   Yang ZX, 2006, J ENVIRON SCI-CHINA, V18, P1135, DOI 10.1016/S1001-0742(06)60051-X"
"   Yin HQ, 2015, SCI REP-UK, V5, DOI 10.1038/srep14266"
"   Yoshida M, 2010, MICROBES ENVIRON, V25, P45, DOI 10.1264/jsme2.ME09160"
"   Yu H, 2016, ENVIRON SCI POLLUT R, V23, P19212, DOI 10.1007/s11356-016-6967-8"
"   Zainun MY, 2018, SCI TOTAL ENVIRON, V616, P269, DOI 10.1016/j.scitotenv.2017.10.266"
"   Zhang C, 2016, SCI TOTAL ENVIRON, V557, P785, DOI 10.1016/j.scitotenv.2016.01.170"
"   Zhang FP, 2010, APPL SOIL ECOL, V45, P144, DOI 10.1016/j.apsoil.2010.03.006"
"   Zhang J, 2015, SCI TOTAL ENVIRON, V508, P29, DOI 10.1016/j.scitotenv.2014.11.064"
"   Zhang MY, 2017, CHEMOSPHERE, V184, P892, DOI 10.1016/j.chemosphere.2017.06.081"
"   Zhang MY, 2016, SCI TOTAL ENVIRON, V543, P636, DOI 10.1016/j.scitotenv.2015.11.053"
"   Zhang W, 2016, J HAZARD MATER, V301, P1, DOI 10.1016/j.jhazmat.2015.08.037"
"   Zhang XK, 2013, ENVIRON SCI POLLUT R, V20, P8472, DOI 10.1007/s11356-013-1659-0"
"   Zhang Y, 2008, PEDOSPHERE, V18, P1, DOI 10.1016/S1002-0160(07)60097-6"
"   Zhou ZF, 2012, J ENVIRON SCI, V24, P369, DOI 10.1016/S1001-0742(11)60747-X"
NR 104
TC 315
Z9 368
U1 25
U2 1062
PU ACADEMIC PRESS LTD- ELSEVIER SCIENCE LTD
PI LONDON
"PA 24-28 OVAL RD, LONDON NW1 7DX, the United Kingdom"
SN 0301-4797
EI 1095-8630

J9 J ENVIRON MANAGE
JI J. Environ. Manage.
PD JUL 15
PY 2019
VL 242
BP 121
EP 130
DI 10.1016/j.jenvman.2019.04.061
PG 10
WC Environmental Sciences
WE Science Citation Index Expanded (SCI-EXPANDED)
SC Environmental Sciences & Ecology
GA IB1VV
UT WOS:000470054000015
PM 31028952
DA 2026-04-06
ER

PT J
"AU Devi, R"
"   Behera, B"
"   Raza, MB"
"   Mangal, V"
"   Altaf, MA"
"   Kumar, R"
"   Kumar, A"
"   Tiwari, RK"
"   Lal, MK"
"   Singh, B"
"AF Devi, Rajni"
"   Behera, Biswaranjan"
"   Raza, Md Basit"
"   Mangal, Vikas"
"   Altaf, Muhammad Ahsan"
"   Kumar, Ravinder"
"   Kumar, Awadhesh"
"   Tiwari, Rahul Kumar"
"   Lal, Milan Kumar"
"   Singh, Brajesh"
TI An Insight into Microbes Mediated Heavy Metal Detoxification in Plants:
   a Review
SO JOURNAL OF SOIL SCIENCE AND PLANT NUTRITION
LA English
DT Review
DE Phytoremediation; Heavy metals; Oxidative stress; Bioremediation;
   Rhizobacteria
ID ARBUSCULAR MYCORRHIZAL FUNGI; GROWTH-PROMOTING RHIZOBACTERIA;
   TRITICUM-AESTIVUM L.; ZEA-MAYS L.; WASTE-WATER; SOLANUM-LYCOPERSICON;
   ENDOPHYTIC BACTERIA; CONTAMINATED SOILS; STRESS ALLEVIATION;
   ARSENIC-RESISTANT
"AB In this era of rapid industrialization and modernization, contamination of the environment with heavy metals from natural and anthropogenic sources without proper disposal treatment is a global concern. Huge amounts of toxic heavy metals (HM) released through industrial, agricultural, and military practices into the environment lead to pernicious effects on soils, water, and air. Deposited toxic HM beyond certain permissible limits is producing an obnoxious effect not only on the soil but also on human and animal health. Application of physical and chemical processes for HM remediation is expensive, laborious, and non-sustainable. Under metal stress, soil microbes have developed various mechanisms to cope with metal toxicity. They can accumulate, alter, or detoxify HM. Therefore, the exploitation of microbes acquiring metal detoxifying traits in addition to plant beneficial attributes make the metal remediation process eco-friendly and cost-effective. Plant-microbe interactions aiming at HM stress may provide a new way to existing phytoremediation and rhizoremediation uses. This review includes a brief insight into heavy metal pollution, their effect on plant biology, and metal-microbe association and highlights the various mechanisms of microbes mediated heavy metal detoxification in plants."
"C1 [Devi, Rajni] Punjab Agr Univ, Ludhiana 141004, Punjab, India."
"   [Devi, Rajni; Mangal, Vikas; Kumar, Ravinder; Tiwari, Rahul Kumar; Lal, Milan Kumar; Singh, Brajesh] ICAR Cent Potato Res Inst, Shimla 171001, Himachal Prades, India."
"   [Behera, Biswaranjan] ICAR Indian Inst Water Management, Bhubaneswar 751023, Odisha, India."
"   [Raza, Md Basit; Tiwari, Rahul Kumar; Lal, Milan Kumar] ICAR Indian Agr Res Inst, New Delhi 110012, India."
"   [Altaf, Muhammad Ahsan] Hainan Univ, Coll Hort, Haikou 570228, Hainan, Peoples R China."
"   [Kumar, Awadhesh] ICAR Natl Rice Res Inst, Cuttack 753006, Odisha, India."
C3 Punjab Agricultural University; Indian Council of Agricultural Research
   (ICAR); ICAR - Central Potato Research Institute; Indian Council of
   Agricultural Research (ICAR); ICAR - Indian Institute of Water
   Management; Indian Council of Agricultural Research (ICAR); ICAR -
   Indian Agricultural Research Institute; Hainan University; Indian
   Council of Agricultural Research (ICAR); ICAR - National Rice Research
   Institute
"RP Tiwari, RK; Lal, MK (corresponding author), ICAR Cent Potato Res Inst, Shimla 171001, Himachal Prades, India.; Behera, B (corresponding author), ICAR Indian Inst Water Management, Bhubaneswar 751023, Odisha, India.; Tiwari, RK; Lal, MK (corresponding author), ICAR Indian Agr Res Inst, New Delhi 110012, India."
EM biswaranjanbehera41@gmail.com; rahul.tiwari@icar.gov.in;
   milan2925@gmail.com
"RI Kumar, Ravinder/AAQ-4588-2021; Altaf, Muhammad Ahsan/ABG-7689-2021;"

"   Raza, Basit/HKF-5896-2023; MANGAL, VIKAS/KRQ-2362-2024; Kumar,"
"   Awadhesh/ACF-5693-2022; Tiwari, Rahul Kumar/AAV-1257-2021; Kumar,"
"   Awadhesh/ACF-5693-2022; Lal, Milan Kumar/ABI-7965-2020; Behera,"
Biswaranjan/ABC-2272-2020
"OI Kumar, Ravinder/0000-0001-9034-7742; Altaf, Muhammad"
"   Ahsan/0000-0001-7257-479X; Raza, Basit/0000-0003-4473-0737; MANGAL,"
"   VIKAS/0000-0001-9418-2340; Kumar, Awadhesh/0000-0001-9055-2500; Tiwari,"
"   Rahul Kumar/0000-0001-7789-8144; Kumar, Awadhesh/0000-0002-4428-1030;"
"   Lal, Milan Kumar/0000-0002-2442-9640; Behera,"
Biswaranjan/0000-0002-0337-9808
"CR Abadi VAJM, 2016, SYMBIOSIS, V69, P9, DOI 10.1007/s13199-015-0361-z"
"   Latef AAHA, 2020, PLANTS-BASEL, V9, DOI 10.3390/plants9111513"
"   Abou-Shanab RA, 2003, NEW PHYTOL, V158, P219, DOI 10.1046/j.1469-8137.2003.00721.x"
"   Adediran GA, 2015, J HAZARD MATER, V283, P490, DOI 10.1016/j.jhazmat.2014.09.064"
"   Ahemad M, 2012, IIOAB J, V3, P39"
"   Ahemad M, 2012, CHEMOSPHERE, V86, P945, DOI 10.1016/j.chemosphere.2011.11.013"
"   Ahmad I, 2016, J PLANT GROWTH REGUL, V35, P303, DOI 10.1007/s00344-015-9534-5"
"   Al-Tarawneh MAB, 2022, J AMB INTEL HUM COMP, V13, P445, DOI 10.1007/s12652-021-02910-w"
"   Alengebawy A, 2021, TOXICS, V9, DOI 10.3390/toxics9030042"
"   Alkorta I., 2004, Reviews in Environmental Science and Bio/Technology, V3, P71, DOI 10.1023/B:RESB.0000040059.70899.3d"
"   Alloway B.J., 2013, Heavy Metals in Soils, P11, DOI [10.1007/978-94-007-4470-7-2, DOI 10.1007/978-94-007-4470-7-2, 10.1002/9781119438922.ch18, DOI 10.1007/978-94-007-4470-72]"
"   Amstaetter K, 2010, ENVIRON SCI TECHNOL, V44, P102, DOI 10.1021/es901274s"
"   [Anonymous], 2016, The Mycota III: Biochemistry and Molecular Biology"
"   Arora K, 2016, INT J PHYTOREMEDIAT, V18, P704, DOI 10.1080/15226514.2015.1131232"
"   Ashraf R, 2007, PAK J BOT, V39, P629"
"   Awan SA, 2020, PLANTS-BASEL, V9, DOI 10.3390/plants9070878"
"   Ayangbenro AS, 2017, INT J ENV RES PUB HE, V14, DOI 10.3390/ijerph14010094"
"   Azc??n R, 2010, MICROB ECOL, V59, P668, DOI 10.1007/s00248-009-9618-5"
"   Aziz L, 2021, J PLANT INTERACT, V16, P104, DOI 10.1080/17429145.2021.1903105"
"   Babu AG, 2013, J HAZARD MATER, V250, P477, DOI 10.1016/j.jhazmat.2013.02.014"
"   Badri DV, 2009, CURR OPIN BIOTECH, V20, P642, DOI 10.1016/j.copbio.2009.09.014"
"   Begum N, 2019, FRONT PLANT SCI, V10, DOI 10.3389/fpls.2019.01068"
"   Belimov AA, 2015, WATER AIR SOIL POLL, V226, DOI 10.1007/s11270-015-2537-9"
"   Bhattacharyya P, 2008, GEOSCI J, V12, P41, DOI 10.1007/s12303-008-0006-9"
"   Bijay-Singh, 2018, AGRONOMY-BASEL, V8, DOI 10.3390/agronomy8040048"
"   Carrillo-Casta??eda G, 2003, J PLANT NUTR, V26, P1801, DOI 10.1081/PLN-120023284"
"   Chatterjee S, 2009, WORLD J MICROB BIOT, V25, P1829, DOI 10.1007/s11274-009-0084-5"
"   Chen L, 2014, SOIL BIOL BIOCHEM, V68, P300, DOI 10.1016/j.soilbio.2013.10.021"
"   Chen M, 2015, BIOTECHNOL ADV, V33, P745, DOI 10.1016/j.biotechadv.2015.05.003"
"   Choudhury BU, 2017, SCI TOTAL ENVIRON, V583, P344, DOI 10.1016/j.scitotenv.2017.01.074"
"   Chourasia KN, 2021, LIFE-BASEL, V11, DOI 10.3390/life11060545"
"   Christou A, 2021, ENVIRON EXP BOT, V189, DOI 10.1016/j.envexpbot.2021.104564"
"   Clemens S, 2002, TRENDS PLANT SCI, V7, P309, DOI 10.1016/S1360-1385(02)02295-1"
"   Clemens S, 2016, ANNU REV PLANT BIOL, V67, P489, DOI 10.1146/annurev-arplant-043015-112301"
"   Dabrowska G, 2017, INT J PHYTOREMEDIAT, V19, P597, DOI 10.1080/15226514.2016.1244157"
"   Dary M, 2010, J HAZARD MATER, V177, P323, DOI 10.1016/j.jhazmat.2009.12.035"
"   Dhaliwal SS, 2020, SOIL SEDIMENT CONTAM, V29, P638, DOI 10.1080/15320383.2020.1758031"
"   Dimkpa C, 2008, CAN J MICROBIOL, V54, P163, DOI [10.1139/W07-130, 10.1139/w07-130]"
"   Dimkpa CO, 2009, SOIL BIOL BIOCHEM, V41, P154, DOI 10.1016/j.soilbio.2008.10.010"
"   El-Meihy RM, 2019, ENVIRON EXP BOT, V162, P295, DOI 10.1016/j.envexpbot.2019.03.005"
"   Emamverdian Abolghassem, 2015, ScientificWorldJournal, V2015, P756120, DOI [10.1155/2015/756120, 10.1155/2015/756120]"
"   Fan MC, 2018, MICROBIOL RES, V217, P51, DOI 10.1016/j.micres.2018.09.002"
"   Fatemi H, 2020, ENVIRON POLLUT, V266, DOI 10.1016/j.envpol.2020.114982"
"   Feng YM, 2020, FRONT MICROBIOL, V11, DOI 10.3389/fmicb.2020.00389"
"   Gadd GM, 2010, MICROBIOL-SGM, V156, P609, DOI 10.1099/mic.0.037143-0"
"   Garc??a-S??nchez F, 2020, J HAZARD MATER, V397, DOI 10.1016/j.jhazmat.2020.122713"
"   Garg N, 2011, J PLANT GROWTH REGUL, V30, P286, DOI 10.1007/s00344-010-9191-7"
"   Glick BR, 2010, BIOTECHNOL ADV, V28, P367, DOI 10.1016/j.biotechadv.2010.02.001"
"   Gonnelli C., 2013, Heavy Metals in Soils, P313, DOI [10.1007/978-94-007-4470-7_11, DOI 10.1007/978-94-007-4470-7_11]"
"   Gontia-Mishra I, 2016, J PLANT GROWTH REGUL, V35, P1000, DOI 10.1007/s00344-016-9598-x"
"   Guo W, 2013, APPL SOIL ECOL, V72, P85, DOI 10.1016/j.apsoil.2013.06.001"
"   Gupta DK, 2014, CHEMOSPHERE, V108, P134, DOI 10.1016/j.chemosphere.2014.01.030"
"   Guti??rrez-Corona JF, 2016, WORLD J MICROB BIOT, V32, DOI 10.1007/s11274-016-2150-0"
"   Haferburg G, 2007, J BASIC MICROB, V47, P453, DOI 10.1002/jobm.200700275"
"   Han YL, 2015, J MICROBIOL BIOTECHN, V25, P1119, DOI 10.4014/jmb.1412.12053"
"   Hansda A, 2017, 3 BIOTECH, V7, DOI 10.1007/s13205-017-0757-y"
"   Hartmann A, 2009, PLANT SOIL, V321, P235, DOI 10.1007/s11104-008-9814-y"
"   Hashem A, 2016, SAUDI J BIOL SCI, V23, P272, DOI 10.1016/j.sjbs.2015.11.002"
"   He HD, 2013, CHEMOSPHERE, V90, P1960, DOI 10.1016/j.chemosphere.2012.10.057"
"   Hossain MA., 2012, APPL ENVIRON SOIL SC, V2012, DOI [DOI 10.1155/2012/650206, 10.1155/2012/872875, DOI 10.1155/2012/872875]"
"   Ilieva A., 2014, Journal of Central European Agriculture, V15, P23, DOI 10.5513/JCEA01/15.2.1446"
"   Imtiaz M, 2018, ECOTOX ENVIRON SAFE, V158, P139, DOI 10.1016/j.ecoenv.2018.04.022"
"   Irfan M, 2021, SCI REP-UK, V11, DOI 10.1038/s41598-021-97525-8"
"   Irfan M, 2021, J SAUDI CHEM SOC, V25, DOI 10.1016/j.jscs.2021.101232"
"   Jamil M, 2014, INT J PHYTOREMEDIAT, V16, P554, DOI 10.1080/15226514.2013.798621"
"   Janmohammadi M., 2013, Cercetari Agronomice in Moldova, V46, P49"
"   Johansson EM, 2008, SOIL BIOL BIOCHEM, V40, P2225, DOI 10.1016/j.soilbio.2008.04.016"
"   Jones AM, 2016, SCI TOTAL ENVIRON, V547, P104, DOI 10.1016/j.scitotenv.2016.01.024"
"   Jordao CP, 2006, ENVIRON MONIT ASSESS, V112, P309, DOI 10.1007/s10661-006-1072-y"

"   Carlos MHJ, 2016, MICROBIOL RES, V188, P53, DOI 10.1016/j.micres.2016.05.001"
"   Kabata-Pendias Alina, 2010, P1"
"   Kaewdoung B, 2016, GEOMICROBIOL J, V33, P283, DOI 10.1080/01490451.2015.1048394"
"   Kamran MA, 2015, ENVIRON SCI POLLUT R, V22, P9275, DOI 10.1007/s11356-015-4074-x"
"   Kantachote D, 2016, APPL SOIL ECOL, V100, P154, DOI 10.1016/j.apsoil.2015.12.015"
"   Kanwal A, 2020, SCI REP-UK, V10, DOI 10.1038/s41598-020-68208-7"
"   Khan S, 2008, ENVIRON POLLUT, V152, P686, DOI 10.1016/j.envpol.2007.06.056"
"   Khan WU, 2017, INT J PHYTOREMEDIAT, V19, P470, DOI 10.1080/15226514.2016.1244167"
"   Nguyen KQ, 2018, ANN MICROBIOL, V68, P217, DOI 10.1007/s13213-018-1332-4"
"   Kong LY, 2020, INT J MOL SCI, V21, DOI 10.3390/ijms21041480"
"   Kosev V, 2014, INT J PHARM LIFE SCI, V5"
"   Kotrba P, 2009, BIOTECHNOL ADV, V27, P799, DOI 10.1016/j.biotechadv.2009.06.003"
"   Kr??mer U, 2010, ANNU REV PLANT BIOL, V61, P517, DOI 10.1146/annurev-arplant-042809-112156"
"   Kuiper I, 2004, MOL PLANT MICROBE IN, V17, P6, DOI 10.1094/MPMI.2004.17.1.6"
"   Kumar RN, 2009, J HAZARD MATER, V169, P1119, DOI 10.1016/j.jhazmat.2009.04.069"
"   Lal MK, 2022, PLANT CELL REP, V41, P501, DOI 10.1007/s00299-021-02784-4"
"   Lal MK, 2021, FOOD CHEM, V359, DOI 10.1016/j.foodchem.2021.129939"
"   Lal MK, 2021, STARCH-STARKE, V73, DOI 10.1002/star.201900281"
"   Lermen C, 2015, IND CROP PROD, V76, P734, DOI 10.1016/j.indcrop.2015.07.009"
"   Liu H, 2015, APPL SOIL ECOL, V89, P44, DOI 10.1016/j.apsoil.2015.01.006"
"   Lu YG, 2018, PEDOSPHERE, V28, P680, DOI [10.1016/S1002-0160(17)60417-X, 10.1016/s1002-0160(17)60417-x]"
"   Ma Y, 2009, J ENVIRON MANAGE, V90, P831, DOI 10.1016/j.jenvman.2008.01.014"
"   Ma Y, 2016, FRONT PLANT SCI, V7, DOI 10.3389/fpls.2016.00918"
"   Ma Y, 2015, J ENVIRON MANAGE, V156, P62, DOI 10.1016/j.jenvman.2015.03.024"
"   Mane PC, 2012, INT J ENVIRON RES, V6, P571"
"   Marques APGC, 2013, CHEMOSPHERE, V92, P74, DOI 10.1016/j.chemosphere.2013.02.055"
"   Merdy P, 2009, COLLOID SURFACE A, V347, P192, DOI 10.1016/j.colsurfa.2009.04.004"
"   Mishra J, 2017, FRONT MICROBIOL, V8, DOI 10.3389/fmicb.2017.01706"
"   Mu??oz R, 2006, CHEMOSPHERE, V63, P903, DOI 10.1016/j.chemosphere.2005.09.062"
"   Mushtaq T, 2020, INT J PHYTOREMEDIAT, V22, P1408, DOI 10.1080/15226514.2020.1781052"
"   Nagajyoti PC, 2010, ENVIRON CHEM LETT, V8, P199, DOI 10.1007/s10311-010-0297-8"
"   Naveed M, 2020, PLANTS-BASEL, V9, DOI 10.3390/plants9040530"
"   Naz R, 2021, PLANT PHYSIOL BIOCH, V159, P285, DOI 10.1016/j.plaphy.2020.12.022"
"   Oladipo OG, 2018, BRAZ J MICROBIOL, V49, P29, DOI 10.1016/j.bjm.2017.06.003"
"   Orlowska E, 2013, ENVIRON POLLUT, V175, P100, DOI 10.1016/j.envpol.2012.12.028"
"   Pandey S, 2013, J MICROBIOL, V51, P11, DOI 10.1007/s12275-013-2330-7"
"   Patra M, 2004, ENVIRON EXP BOT, V52, P199, DOI 10.1016/j.envexpbot.2004.02.009"
"   Pokethitiyook P., 2016, Phytoremediation, DOI [DOI 10.1007/978-3-319-40148-54, 10.1007/978-3-319-40148-5_, DOI 10.1007/978-3-319-40148-5]"
"   Pramanik K, 2017, ENVIRON SCI POLLUT R, V24, P24419, DOI 10.1007/s11356-017-0033-z"
"   Purakayastha TJ, 2010, SOIL BIOL, V19, P389, DOI 10.1007/978-3-642-02436-8_18"
"   Qadir M, 2020, CHEMOSPHERE, V258, DOI 10.1016/j.chemosphere.2020.127386"
"   Rajkumar M, 2012, BIOTECHNOL ADV, V30, P1562, DOI 10.1016/j.biotechadv.2012.04.011"
"   Rangel WM, 2017, INT J PHYTOREMEDIAT, V19, P142, DOI 10.1080/15226514.2016.1207600"
"   Ravikumar C., 2021, Journal of Applied and Natural Science, V13, P295, DOI [10.31018/jans.v13i1.2569, 10.31018/jans.v13i1.2569]"
"   Rehman M, 2019, ENVIRON SCI POLLUT R, V26, P5851, DOI 10.1007/s11356-018-4015-6"
"   Rezapour S, 2019, SCI TOTAL ENVIRON, V656, P261, DOI 10.1016/j.scitotenv.2018.11.288"
"   Rizvi A, 2018, ECOTOX ENVIRON SAFE, V157, P9, DOI 10.1016/j.ecoenv.2018.03.063"
"   Rizvi A, 2017, CHEMOSPHERE, V185, P942, DOI 10.1016/j.chemosphere.2017.07.088"
"   Rom??n-Ponce B, 2017, PEDOSPHERE, V27, P511, DOI 10.1016/S1002-0160(17)60347-3"
"   Rouphael Y, 2015, SCI HORTIC-AMSTERDAM, V196, P91, DOI 10.1016/j.scienta.2015.09.002"
"   Saleem M, 2018, CHEMOSPHERE, V195, P606, DOI 10.1016/j.chemosphere.2017.12.117"
"   Saleem MH, 2020, PLANTS-BASEL, V9, DOI 10.3390/plants9030404"
"   Saleem MH, 2020, J ENVIRON MANAGE, V257, DOI 10.1016/j.jenvman.2019.109994"
"   Saleem MH, 2020, ECOTOX ENVIRON SAFE, V189, DOI 10.1016/j.ecoenv.2019.109915"
"   Schneider KD, 2010, J APPL MICROBIOL, V108, P366, DOI 10.1111/j.1365-2672.2009.04489.x"
"   Segura A, 2009, MICROB BIOTECHNOL, V2, P452, DOI 10.1111/j.1751-7915.2009.00113.x"
"   Sepehri M, 2021, MICROB ECOL, V81, P717, DOI 10.1007/s00248-020-01629-z"
"   Sessitsch A, 2013, SOIL BIOL BIOCHEM, V60, P182, DOI 10.1016/j.soilbio.2013.01.012"
"   Sharma RK, 2020, J SCI FOOD AGR, V100, P1532, DOI 10.1002/jsfa.10161"
"   Singh Jiwan Singh Jiwan, 2011, International Journal of Research in Chemistry and Environment (IJRCE), V1, P15"
"   Soto J, 2019, MICROORGANISMS, V7, DOI 10.3390/microorganisms7090348"
"   Srivastava S, 2014, ECOL ENG, V70, P146, DOI 10.1016/j.ecoleng.2014.05.008"
"   Srivastava S, 2013, J HAZARD MATER, V262, P1039, DOI 10.1016/j.jhazmat.2012.08.019"
"   Talukdar D, 2020, ENVIRON TECHNOL INNO, V20, DOI 10.1016/j.eti.2020.101050"
"   Tastan BE, 2010, BIORESOURCE TECHNOL, V101, P870, DOI 10.1016/j.biortech.2009.08.099"
"   Tchounwou Paul B, 2012, Exp Suppl, V101, P133, DOI 10.1007/978-3-7643-8340-4_6"
"   Thirkell TJ, 2017, J ECOL, V105, P921, DOI 10.1111/1365-2745.12788"
"   Tiwari RK, 2021, POSTHARVEST BIOL TEC, V181, DOI 10.1016/j.postharvbio.2021.111638"
"   Tiwari RK, 2021, PHYSIOL PLANTARUM, V172, P1212, DOI 10.1111/ppl.13307"
"   Tiwari RK, 2020, 3 BIOTECH, V10, DOI 10.1007/s13205-020-02496-8"
"   Tiwari S, 2018, FRONT PLANT SCI, V9, DOI 10.3389/fpls.2018.00452"
"   Tripathi P, 2020, SCI TOTAL ENVIRON, V716, DOI 10.1016/j.scitotenv.2020.136758"
"   Ul Hassan T, 2017, INT J PHYTOREMEDIAT, V19, P522, DOI 10.1080/15226514.2016.1267696"
"   Vareda JP, 2019, J ENVIRON MANAGE, V246, P101, DOI 10.1016/j.jenvman.2019.05.126"
"   Wang Q, 2011, J APPL MICROBIOL, V111, P1065, DOI 10.1111/j.1365-2672.2011.05142.x"
"   Wang Q, 2020, BMC PLANT BIOL, V20, DOI 10.1186/s12870-020-2273-1"
"   Wani PA, 2008, BIOTECHNOL LETT, V30, P159, DOI 10.1007/s10529-007-9515-2"
"   Wong JWC, 2006, CHEMOSPHERE, V63, P980, DOI 10.1016/j.chemosphere.2005.08.045"
"   Wuana R.A., 2011, ISRN Ecology, DOI [10.5402/2011/402647, DOI 10.5402/2011/402647]"
"   Xie LP, 2018, ECOTOX ENVIRON SAFE, V162, P71, DOI 10.1016/j.ecoenv.2018.06.072"
"   Yaseen M, 2017, INT J PHYTOREMEDIAT, V19, P798, DOI 10.1080/15226514.2017.1284757"

"   Yu FM, 2021, PLANT SOIL, V459, P155, DOI 10.1007/s11104-020-04752-5"
"   Zaidi S, 2006, CHEMOSPHERE, V64, P991, DOI 10.1016/j.chemosphere.2005.12.057"
"   Zainab N, 2020, PLANT PHYSIOL BIOCH, V152, P90, DOI 10.1016/j.plaphy.2020.04.039"
"   Zanganeh F, 2021, BIOAUGMENTATION BIOA, P1, DOI [10.21203/rs.3.rs-439162/v1, DOI 10.21203/RS.3.RS-439162/V1]"
"   Zhuang P, 2014, PLOS ONE, V9, DOI 10.1371/journal.pone.0094484"
"   Zhuang P, 2013, ENVIRON SCI POLLUT R, V20, P5844, DOI 10.1007/s11356-013-1606-0"
NR 156
TC 66
Z9 71
U1 6
U2 129
PU SPRINGER INT PUBL AG
PI CHAM
"PA GEWERBESTRASSE 11, CHAM, CH-6330, SWITZERLAND"
SN 0718-9508
EI 0718-9516
J9 J SOIL SCI PLANT NUT
JI J. Soil Sci. Plant Nutr.
PD MAR
PY 2022
VL 22
IS 1
BP 914
EP 936
DI 10.1007/s42729-021-00702-x
EA NOV 2021
PG 23
WC Plant Sciences; Environmental Sciences; Soil Science
WE Science Citation Index Expanded (SCI-EXPANDED)
SC Plant Sciences; Environmental Sciences & Ecology; Agriculture
GA ZO6RI
UT WOS:000722123700002
DA 2026-04-06
ER

PT J
"AU Kour, D"
"   Sharma, B"
"   Negi, R"
"   Kumar, S"
"   Kaur, S"
"   Kaur, T"
"   Khan, SS"
"   Kour, H"
"   Ramniwas, S"
"   Rustegi, S"
"   Rai, AK"
"   Singh, S"
"   Shreaz, S"
"   Yadav, AN"
"   Ahluwalia, AS"
"AF Kour, Divjot"
"   Sharma, Babita"
"   Negi, Rajeshwari"
"   Kumar, Sanjeev"
"   Kaur, Simranjeet"
"   Kaur, Tanvir"
"   Khan, Sofia Sharief"
"   Kour, Harpreet"
"   Ramniwas, Seema"
"   Rustegi, Sarvesh"
"   Rai, Ashutosh Kumar"
"   Singh, Sangram"
"   Shreaz, Sheikh"
"   Yadav, Ajar Nath"
"   Ahluwalia, Amrik Singh"
TI Microbial Amelioration of Heavy Metal Toxicity in Plants for
   Agro-Environmental Sustainability
SO WATER AIR AND SOIL POLLUTION
LA English
DT Review
DE Bioavailability; Biodiversity; Heavy metals; Microbes; Mitigation
ID SEEDLING GROWTH PROMOTION; RESISTANT-BACTERIA; OXIDATIVE STRESS;
   MEDIATED ALLEVIATION; CONTAMINATED SOIL; CHROMIUM TOXICITY; MERCURY
   TOXICITY; RISK-ASSESSMENT; LEAD-RESISTANT; CADMIUM STRESS
"AB Heavy metal pollution is one of the modern environmental problems that contaminate soil, water, and air. Heavy metal pollution not only results in significant crop yield losses, but also poses health risks. When subjected to heavy metal toxicity, which happens when the quantity of particular heavy metals in the soil or water exceeds their tolerance levels, plants can exhibit a variety of reactions. Metal accumulation in the tissues of plants causes oxidative stress. The oxidative stress in turn impacts cellular homeostasis and adversely affects growth and development. Plants exhibit different mechanisms to fight stressful conditions and lessen the negative consequences of heavy metal toxicity. In this regard, the first line of defence in response to heavy metal toxicity provided by the plant is restricting the uptake of the metals. The second

line of defence includes various detoxification mechanisms. Microbes especially associated with the rhizospheric region of the crops have role in remediation of the soils from metal contaminants. The present review highlights the impact of heavy metal on plants and role of the microbes in lessening of heavy metal stress in plants."
"C1 [Kour, Divjot; Sharma, Babita] Eternal Univ, Akal Coll Basic Sci, Dept Microbiol, Baru Sahib 173101, Himachal Prades, India."
" [Negi, Rajeshwari; Kaur, Tanvir; Yadav, Ajar Nath] Eternal Univ, Dr Khem Singh Gill Akal Coll Agr, Dept Genet Plant Breeding & Biotechnol, Baru Sahib 173101, Himachal Prades, India."
" [Kumar, Sanjeev] GLA Univ, Fac Agr Sci, Mathura, Uttar Pradesh, India."
" [Kaur, Simranjeet] Eternal Univ, Akal Coll Basic Sci, Dept Zool, Baru Sahib 173101, Himachal Prades, India."
" [Khan, Sofia Sharief] Shri Mata Vaishno Devi Univ, Dept Biotechnol, Katra 182320, Jammu And Kashm, India."
" [Kour, Harpreet] Univ Jammu, Dept Bot, Jammu 180006, India."
" [Ramniwas, Seema] Chandigarh Univ, Univ Ctr Res & Dev, Dept Biotechnol, Mohali 140413, Punjab, India."
" [Rustegi, Sarvesh] Uttaranchal Univ, Sch Appl & Life Sci, Dept Food Technol, Dehra Dun, Uttarakhand, India."
" [Rai, Ashutosh Kumar] Imam Abdulrahman Bin Faisal Univ, Coll Med, Dept Biochem, Dammam Kingdom Saudi Arabia, Dammam, Saudi Arabia."
" [Singh, Sangram] Dr Ram Manohar Lohia Avadh Univ, Dept Biochem, Faizabad, Uttar Pradesh, India."
" [Shreaz, Sheikh] Kuwait Inst Sci Res, Environm & Life Sci Res Ctr, POB 24885, Safat 13109, Kuwait."
" [Ahluwalia, Amrik Singh] Eternal Univ, Akal Coll Basic Sci, Dept Bot, Baru Sahib 173101, Himachal Prades, India."
C3 GLA University; Shri Mata Vaishno Devi University; University of Jammu;
Chandigarh University; Uttaranchal University; Imam Abdulrahman Bin
Faisal University; Kuwait Institute for Scientific Research
"RP Yadav, AN (corresponding author), Eternal Univ, Dr Khem Singh Gill Akal Coll Agr, Dept Genet Plant Breeding & Biotechnol, Baru Sahib 173101, Himachal Prades, India."
EM ajar@eternaluniversity.edu.in
"RI Kour, Harpreet/AAX-5888-2020; Kour, Divjot/AAK-1119-2021; Kaur,"
" Tanvir/AAS-2845-2021; Kaur, Simranjeet/LTE-8086-2024; Rai, Ashutosh"
" Kumar/E-4904-2018; Yadav, Ajar Nath/I-3572-2014; Negi,"
Rajeshwari/HTS-0468-2023
"OI Kaur, Simranjeet/0000-0002-2796-7727; Rai, Ashutosh"
" Kumar/0000-0001-6938-619X; Shreaz, Sheikh/0009-0006-0865-1993; Yadav,"
" Ajar Nath/0000-0002-6911-7050; Negi, Rajeshwari/0009-0009-4445-1237"
FU DEST
FX No Statement Available
"CR Abdollahi S, 2020, INT MICROBIOL, V23, P625, DOI 10.1007/s10123-020-00133-1"
" Abo-Amer AE, 2014, GEOMICROBIOL J, V31, P551, DOI 10.1080/01490451.2013.850561"
" Abou-Aly HE, 2021, BIOCATAL AGR BIOTECH, V31, DOI 10.1016/j.bcab.2021.101911"
" Ahammed GJ, 2022, ENVIRON POLLUT, V292, DOI 10.1016/j.envpol.2021.118475"
" Alaoui-Soss?? B, 2004, PLANT SCI, V166, P1213, DOI 10.1016/j.plantsci.2003.12.032"
" Ali H, 2013, CHEMOSPHERE, V91, P869, DOI 10.1016/j.chemosphere.2013.01.075"
" Angulo-Bejarano PI, 2021, PLANTS-BASEL, V10, DOI 10.3390/plants10040635"
" Anjum SA, 2017, PEDOSPHERE, V27, P262, DOI 10.1016/S1002-0160(17)60315-1"
" [Anonymous], 2003, BIOAVAILABILITY CONT"
" Antoniadis V, 2017, EARTH-SCI REV, V171, P621, DOI 10.1016/j.earscirev.2017.06.005"
" Ayangbenro AS, 2017, INT J ENV RES PUB HE, V14, DOI 10.3390/ijerph14010094"
" Banerjee G, 2015, WATER AIR SOIL POLL, V226, DOI 10.1007/s11270-015-2359-9"
" Banerjee S, 2011, J ENVIRON SCI HEAL A, V46, P1736, DOI 10.1080/10934529.2011.623995"
" Bhaduri AM, 2012, REV ENVIRON SCI BIO, V11, P55, DOI 10.1007/s11157-011-9251-x"
" Brammer H, 2009, ENVIRON INT, V35, P647, DOI 10.1016/j.envint.2008.10.004"
" Bravo G, 2020, MICROORGANISMS, V8, DOI 10.3390/microorganisms8121952"
" Bruins MR, 2000, ECOTOX ENVIRON SAFE, V45, P198, DOI 10.1006/eesa.1999.1860"
" Burges A, 2018, INT J PHYTOREMEDIAT, V20, P384, DOI 10.1080/15226514.2017.1365340"
" Catarecha P, 2007, PLANT CELL, V19, P1123, DOI 10.1105/tpc.106.041871"
" Cervantes C., 2007, MOL MICROBIOLOGY HEA, P407, DOI [DOI 10.1007/7171_2006_087, 10.1007/7171_2006_087]"
" Chakraborty S, 2021, BIOCATAL AGR BIOTECH, V38, DOI 10.1016/j.bcab.2021.102204"
" Chen J, 2012, BIOMETALS, V25, P847, DOI 10.1007/s10534-012-9560-8"
" Clemens S, 2006, J PLANT PHYSIOL, V163, P319, DOI 10.1016/j.jplph.2005.11.010"
" Clemens S., 2013, Encyclopedia of metalloproteins, DOI [10.1007/978-1-4614-1533-6_313, DOI 10.1007/978-1-4614-1533-6_313]"
" Dalvi A.A., 2013, Annals of Plant Sci, V2, P362, DOI DOI 10.1155/2015/756120"
" Dell'Amico E, 2008, SOIL BIOL BIOCHEM, V40, P74, DOI 10.1016/j.soilbio.2007.06.024"
" Devi Rubee, 2022, Journal of Applied Biology & Biotechnology, V10, P25, DOI [10.7324/jabb.2022.10s203, 10.7324/JABB.2022.10s203]"
" Dharni S, 2014, CHEMOSPHERE, V117, P433, DOI 10.1016/j.chemosphere.2014.08.001"
" Durrieu C, 2002, ECOTOX ENVIRON SAFE, V51, P206, DOI 10.1006/eesa.2001.2140"
" El-Ballat EM, 2023, PLANTS-BASEL, V12, DOI 10.3390/plants12112110"
" Emamverdian Abolghassem, 2015, ScientificWorldJournal, V2015, P756120, DOI [10.1155/2015/756120, 10.1155/2015/756120]"
" Esertas ??Z??, 2020, ARCH MICROBIOL, V202, P1817, DOI 10.1007/s00203-020-01900-4"
" Etesami H, 2018, ECOTOX ENVIRON SAFE, V147, P175, DOI 10.1016/j.ecoenv.2017.08.032"
" Feng Z, 2021, TRAC-TREND ANAL CHEM, V143, DOI 10.1016/j.trac.2021.116402"
" Finnegan PM, 2012, FRONT PHYSIOL, V3, DOI 10.3389/fphys.2012.00182"
" Fuentes A, 2016, PLANT SOIL, V407, P355, DOI 10.1007/s11104-016-2893-2"
" Gadd GM, 2008, WILEY-IUPAC SER BIOP, P53"
" Galvez EN., 1990, Survey of Total Arsenic Concentrations in Edible Seaweeds from Selected Oahu Sites"
" Ghori NH, 2019, INT J ENVIRON SCI TE, V16, P1807, DOI 10.1007/s13762-019-02215-8"
" Gontia-Mishra I, 2016, J PLANT GROWTH REGUL, V35, P1000, DOI 10.1007/s00344-016-9598-x"
" Gupta A, 2002, CURR MICROBIOL, V45, P323, DOI 10.1007/s00284-002-3762-1"
" Hall JL, 2002, J EXP BOT, V53, P1, DOI 10.1093/jexbot/53.366.1"

"   Hansen AJ., 2004, Land Change Science, P277, DOI [10.1007/978-1-4020-2562-4_16, DOI 10.1007/978-1-4020-2562-4_16]"
"   Harada E, 2010, PLANT CELL PHYSIOL, V51, P1627, DOI 10.1093/pcp/pcq118"
"   Harmsen J., 2008, Werkgroep ISO/DIS17402, Soil quality"
"   Harmsen J, 2007, J ENVIRON QUAL, V36, P1420, DOI 10.2134/jeq2006.0492"
"   Hasanuzzaman M, 2015, SOIL REMEDIATION AND PLANTS: PROSPECTS AND CHALLENGES, P433"
"   Hauser MT, 2014, FRONT PLANT SCI, V5, DOI [10.3389/fpls.2014.00320, 10.3389/fpsyg.2014.00401]"
"   Herzig R, 2014, INT J PHYTOREMEDIAT, V16, P735, DOI 10.1080/15226514.2013.856846"
"   Hossain MA., 2012, APPL ENVIRON SOIL SC, V2012, DOI [DOI 10.1155/2012/650206, 10.1155/2012/872875, DOI 10.1155/2012/872875]"
"   Hughes MF, 2011, TOXICOL SCI, V123, P305, DOI 10.1093/toxsci/kfr184"
"   Iram Shazia Iram Shazia, 2013, International Research Journal of Biological Sciences, V2, P66"
"   Islam F, 2016, ENVIRON SCI POLLUT R, V23, P220, DOI 10.1007/s11356-015-5354-1"
"   Israr M, 2006, CHEMOSPHERE, V65, P591, DOI 10.1016/j.chemosphere.2006.02.016"
"   Iyer A, 2004, MAR POLLUT BULL, V49, P974, DOI 10.1016/j.marpolbul.2004.06.023"
"   Jiang CY, 2008, CHEMOSPHERE, V72, P157, DOI 10.1016/j.chemosphere.2008.02.006"
"   Jomova K, 2022, CHEM-BIOL INTERACT, V367, DOI 10.1016/j.cbi.2022.110173"
"   Kalaivanan D., 2016, ABIOTIC STRESS PHYSL, V9, P85, DOI [10.1007/978-81-322-2725-0_5, DOI 10.1007/978-81-322-2725-0_5]"
"   Kang SM, 2020, MICROORGANISMS, V8, DOI 10.3390/microorganisms8030382"
"   K??renlampi S, 2000, ENVIRON POLLUT, V107, P225, DOI 10.1016/S0269-7491(99)00141-4"
"   Kaur H, 2021, PLANTA, V253, DOI 10.1007/s00425-021-03642-z"
"   Kayser A, 2000, ENVIRON SCI TECHNOL, V34, P1778, DOI 10.1021/es990697s"
"   Khalid S, 2017, J GEOCHEM EXPLOR, V182, P247, DOI 10.1016/j.gexplo.2016.11.021"
"   Khan I, 2009, ECOTOX ENVIRON SAFE, V72, P626, DOI 10.1016/j.ecoenv.2007.11.016"
"   Kim SU, 2007, WATER SCI TECHNOL, V55, P105, DOI 10.2166/wst.2007.007"
"   Kisa D, 2019, ACTA BOT CROAT, V78, P9, DOI 10.2478/botcro-2018-0023"
"   Knotek-Smith HM, 2003, BIOMETALS, V16, P251, DOI 10.1023/A:1020617013927"
"   Kohli S K., 2017, Reactive Oxygen Species and Antioxidant Systems in Plants: Role and Regulation Under Abiotic Stress, P185, DOI [DOI 10.1007/978-981-10-5254-5_8, 10.1007/978-981, DOI 10.1007/978-981]"
"   Kotala S., 2014, CURR WORLD ENVIRON, V9, P271, DOI [10.12944/CWE.9.2.07, DOI 10.12944/CWE.9.2.07]"
"   K??pper H, 2017, METAL IONS LIFE SCI, V17, P491, DOI 10.1515/9783110434330-015"
"   Lakshmipathy T.D., 2010, Advances in Biological Research, V4, P108"
"   Leduc DL, 2006, ENVIRON POLLUT, V144, P70, DOI 10.1016/j.envpol.2006.01.008"
"   Li N, 2015, SCI TOTAL ENVIRON, V521, P144, DOI 10.1016/j.scitotenv.2015.03.081"
"   Li R, 2017, SCI REP-UK, V7, DOI 10.1038/srep46545"
"   Liao VHC, 2006, ENVIRON POLLUT, V142, P17, DOI 10.1016/j.envpol.2005.09.021"
"   Lin XY, 2016, SCI TOTAL ENVIRON, V569, P97, DOI 10.1016/j.scitotenv.2016.06.121"
"   Lingua G, 2012, PLOS ONE, V7, DOI 10.1371/journal.pone.0038662"
"   Liu AR, 2022, ENVIRON POLLUT, V314, DOI 10.1016/j.envpol.2022.120303"
"   Liu WX, 2015, J SOIL SEDIMENT, V15, P1191, DOI 10.1007/s11368-015-1067-9"
"   Liu Y, 2018, SCI TOTAL ENVIRON, V613, P462, DOI 10.1016/j.scitotenv.2017.09.088"
"   Lodenius A, 2003, SCI TOTAL ENVIRON, V304, P169, DOI 10.1016/S0048-9697(02)00566-1"
"   Malik RN, 2010, PAK J BOT, V42, P291"
"   Mallick I, 2014, ECOTOX ENVIRON SAFE, V107, P236, DOI 10.1016/j.ecoenv.2014.06.007"
"   Maqsood Q, 2023, ENVIRON RES, V227, DOI 10.1016/j.envres.2023.115665"
"   Marzan LW, 2017, EGYPT J AQUATIC RES, V43, P65, DOI [10.1016/j.ejar.2016.11.002, 10.1016/j.ejar.2016.11.002, DOI 10.1016/j.ejar.2016.11.002, DOI 10.1016/J.EJAR.2016.11.002]"
"   McLean JS, 2000, ENVIRON MICROBIOL, V2, P611, DOI 10.1046/j.1462-2920.2000.00143.x"
"   Mishra J, 2017, FRONT MICROBIOL, V8, DOI 10.3389/fmicb.2017.01706"
"   Mitra S, 2018, MICROBIOL RES, V210, P12, DOI 10.1016/j.micres.2018.03.003"
"   Mondal M, 2022, ENVIRON RES, V214, DOI 10.1016/j.envres.2022.113937"
"   Morkunas I, 2018, MOLECULES, V23, DOI 10.3390/molecules23092320"
"   Mountouris A, 2002, MAR POLLUT BULL, V44, P1136, DOI 10.1016/S0025-326X(02)00168-6"
"   Mourato M, 2012, ADVANCES IN SELECTED PLANT PHYSIOLOGY ASPECTS, P23"
"   Muhammad S, 2011, MICROCHEM J, V98, P334, DOI 10.1016/j.microc.2011.03.003"
"   Mujahid Farid Mujahid Farid, 2013, International Journal of Chemical and Biochemical Sciences, V3, P53"
"   Mutiat FBY, 2018, BIOREMEDIAT J, V22, P1, DOI 10.1080/10889868.2018.1445695"
"   Nagajyoti PC, 2010, ENVIRON CHEM LETT, V8, P199, DOI 10.1007/s10311-010-0297-8"
"   Naik MM, 2012, BIODEGRADATION, V23, P775, DOI 10.1007/s10532-012-9552-y"
"   Nazir A, 2011, PAK J BOT, V43, P1925"
"   Nies DH, 1999, APPL MICROBIOL BIOT, V51, P730, DOI 10.1007/s002530051457"
"   Njoku KL, 2020, SCI AFR, V10, DOI 10.1016/j.sciaf.2020.e00545"
"   Oladipo OG, 2016, BIOREMEDIAT J, V20, P287, DOI 10.1080/10889868.2016.1250722"
"   Olaniran AO, 2013, INT J MOL SCI, V14, P10197, DOI 10.3390/ijms140510197"
"   Oubohssaine M, 2022, MICROORGANISMS, V10, DOI 10.3390/microorganisms10050838"
"   Oves M, 2013, EUR J SOIL BIOL, V56, P72, DOI 10.1016/j.ejsobi.2013.02.002"
"   Paithankar JG, 2021, CHEMOSPHERE, V262, DOI 10.1016/j.chemosphere.2020.128350"
"   Pal AK, 2019, SOIL SEDIMENT CONTAM, V28, P601, DOI 10.1080/15320383.2019.1637398"
"   Pandey S, 2013, J MICROBIOL, V51, P11, DOI 10.1007/s12275-013-2330-7"
"   Paria K, 2022, CHEMOSPHERE, V286, DOI 10.1016/j.chemosphere.2021.131874"
"   Patra M, 2000, BOT REV, V66, P379, DOI 10.1007/BF02868923"
"   Pourrut B, 2011, REV ENVIRON CONTAM T, V213, P113, DOI 10.1007/978-1-4419-9860-6_4"
"   Pramanik Krishnendu, 2018, Agriculture and Natural Resources, V52, P215, DOI [10.1016/j.anres.2018.09.007, 10.1016/j.anres.2018.09.007]"
"   Qadir S, 2014, REV ENVIRON CONTAM T, V229, P51, DOI 10.1007/978-3-319-03777-6_4"
"   Qiu GL, 2008, J AGR FOOD CHEM, V56, P2465, DOI 10.1021/jf073391a"
"   Rajkumar M, 2008, J BASIC MICROB, V48, P500, DOI 10.1002/jobm.200800073"
"   Reale L, 2016, CHEMOSPHERE, V145, P98, DOI 10.1016/j.chemosphere.2015.11.047"
"   Rehman A, 2007, WORLD J MICROB BIOT, V23, P753, DOI 10.1007/s11274-006-9291-5"
"   Renu S, 2022, FRONT MICROBIOL, V12, DOI 10.3389/fmicb.2021.758144"
"   Rinklebe J, 2016, GEODERMA, V270, P21, DOI 10.1016/j.geoderma.2015.10.011"
"   Rizvi A, 2019, RSC ADV, V9, P6125, DOI 10.1039/c9ra00333a"
"   Rout GR, 2009, SUSTAINABLE AGRICULTURE, P873, DOI 10.1051/agro:2002073"

"   Rubiya Syed Rubiya Syed, 2018, Plant Archives, V18, P1229"
"   Sabae S, 2006, BIOSCI RES, V3, P189"
"   Saha J, 2022, GEOMICROBIOL J, V39, P891, DOI 10.1080/01490451.2022.2089781"
"   Sakpirom J, 2017, RES MICROBIOL, V168, P266, DOI 10.1016/j.resmic.2016.12.001"
"   Sandrin ToddR., 2007, Environmental Bioremediation Technologies, P1, DOI DOI 10.1007/978-3-540-34793-4_1"
"   Sangthong C, 2016, ENVIRON SCI POLLUT R, V23, P756, DOI 10.1007/s11356-015-5318-5"
"   Saxena Deepa, 2001, Indian Journal of Experimental Biology, V39, P590"
"   Say R, 2003, SEP SCI TECHNOL, V38, P2039, DOI 10.1081/SS-120020133"
"   Schwesig D, 2003, PLANT SOIL, V253, P445, DOI 10.1023/A:1024891014028"
"   Seregin IV, 2008, RUSS J PLANT PHYSL+, V55, P1, DOI 10.1134/S1021443708010019"
"   Shah FUR, 2010, PLANT ADAPTATION AND PHYTOREMEDIATION, P71, DOI 10.1007/978-90-481-9370-7_4"
"   Shah M., 2021, WORLD J MICROB BIOT, V37, P195, DOI [10.1007/s11274-021-03156-6, DOI 10.1007/s11274-021-03156-6]"
"   Shameer K, 2009, Int J Plant Genomics, V2009, P583429, DOI 10.1155/2009/583429"
"   Shanker AK, 2005, ENVIRON INT, V31, P739, DOI 10.1016/j.envint.2005.02.003"
"   Shilev S, 2020, J CHEM TECHNOL BIOT, V95, P932, DOI 10.1002/jctb.6077"
"   Shreya D, 2020, ARCH MICROBIOL, V202, P887, DOI 10.1007/s00203-019-01801-1"
"   Singh HP, 2013, ENVIRON CHEM LETT, V11, P229, DOI 10.1007/s10311-013-0407-5"
"   Singh N, 2006, PLANT SCI, V170, P274, DOI 10.1016/j.plantsci.2005.08.013"
"   Singh N, 2016, ECOTOX ENVIRON SAFE, V125, P25, DOI 10.1016/j.ecoenv.2015.11.020"
"   Singh S, 2016, FRONT PLANT SCI, V6, DOI 10.3389/fpls.2015.01143"
"   Sinha D, 2023, PLANTS-BASEL, V12, DOI 10.3390/plants12091815"
"   Sinha S, 2008, CURR MICROBIOL, V56, P55, DOI 10.1007/s00284-007-9038-z"
"   Sparks DL, 2005, ELEMENTS, V1, P193, DOI 10.2113/gselements.1.4.193"
"   Srivastava V, 2018, INT J RECYCLING ORG, V7, P241, DOI 10.1007/s40093-018-0210-1"
"   Srivastava V, 2017, FRONT ENV SCI-SWITZ, V5, DOI 10.3389/fenvs.2017.00064"
"   Sultana R, 2023, ENVIRON TECHNOL INNO, V31, DOI 10.1016/j.eti.2023.103160"
"   Suman J, 2018, FRONT PLANT SCI, V9, DOI 10.3389/fpls.2018.01476"
"   Syed A, 2023, SCI REP-UK, V13, DOI 10.1038/s41598-023-31330-3"
"   Sytar O, 2013, ACTA PHYSIOL PLANT, V35, P985, DOI 10.1007/s11738-012-1169-6"
"   Yazdi MET, 2021, ENVIRON SCI POLLUT R, V28, P26359, DOI [10.1007/s11356-021-13687-y, 10.1007/s11356-021-16285-0]"
"   Thomas J, 2023, BIOCATAL AGR BIOTECH, V47, DOI 10.1016/j.bcab.2022.102592"
"   Tiwari S, 2018, FRONT PLANT SCI, V9, DOI 10.3389/fpls.2018.00452"
"   Tlustos P, 2006, PLANT SOIL ENVIRON, V52, P413, DOI 10.17221/3460-PSE"
"   Tripathi M, 2005, CURR MICROBIOL, V50, P233, DOI 10.1007/s00284-004-4459-4"
"   Tripathi M, 2023, ENERGIES, V16, DOI 10.3390/en16114508"
"   Twiss MR, 2001, CHEM SPEC BIOAVAILAB, V13, P9, DOI 10.3184/095422901782775462"
"   Ul Hassan T, 2017, INT J PHYTOREMEDIAT, V19, P522, DOI 10.1080/15226514.2016.1267696"
"   Vald??s J, 2014, CIENC MAR, V40, P45, DOI 10.7773/cm.v40i1.2318"
"   van der Heijden MGA, 2008, ECOL LETT, V11, P296, DOI 10.1111/j.1461-0248.2007.01139.x"
"   Vangronsveld J, 2009, ENVIRON SCI POLLUT R, V16, P765, DOI 10.1007/s11356-009-0213-6"
"   Vaughan DJ, 2006, ELEMENTS, V2, P71, DOI 10.2113/gselements.2.2.71"
"   Venkatachalam P, 2017, CHEMOSPHERE, V171, P544, DOI 10.1016/j.chemosphere.2016.12.092"
"   Verma J, 2016, OCTA J ENV RES, V4, P146"
"   Verma S, 2019, ENVIRON TECHNOL INNO, V14, DOI 10.1016/j.eti.2019.100369"
"   Violante A, 2010, J SOIL SCI PLANT NUT, V10, P268, DOI 10.4067/S0718-95162010000100005"
"   Vivas A, 2005, MICROB ECOL, V49, P416, DOI 10.1007/s00248-004-0044-4"
"   Wang NN, 2023, WATER AIR SOIL POLL, V234, DOI 10.1007/s11270-023-06296-7"
"   Wang R, 2013, INT J MOL SCI, V14, P11125, DOI 10.3390/ijms140611125"
"   Wang YD, 2004, J ENVIRON QUAL, V33, P1779, DOI 10.2134/jeq2004.1779"
"   Wani PA, 2013, B ENVIRON CONTAM TOX, V91, P117, DOI 10.1007/s00128-013-1002-y"
"   Wei GH, 2009, J HAZARD MATER, V162, P50, DOI 10.1016/j.jhazmat.2008.05.040"
"   Wolterbeek HT, 2002, SCI TOTAL ENVIRON, V287, P13, DOI 10.1016/S0048-9697(01)00989-5"
"   Xie Y, 2021, ENVIRON RES, V200, DOI 10.1016/j.envres.2021.111730"
"   Xiong TT, 2016, ENVIRON GEOCHEM HLTH, V38, P1283, DOI 10.1007/s10653-016-9796-2"
"   Xu SZ, 2019, APPL MICROBIOL BIOT, V103, P3887, DOI 10.1007/s00253-019-09700-7"
"   Yan A, 2020, FRONT PLANT SCI, V11, DOI 10.3389/fpls.2020.00359"
"   Yruela Inmaculada, 2005, Braz. J. Plant Physiol., V17, P145, DOI 10.1590/S1677-04202005000100012"
"   Zaid A, 2020, PHYSIOL MOL BIOL PLA, V26, P25, DOI 10.1007/s12298-019-00715-y"
"   Zaynab M, 2022, J KING SAUD UNIV SCI, V34, DOI 10.1016/j.jksus.2021.101653"
"   Zhu HH, 2012, PLOS ONE, V7, DOI 10.1371/journal.pone.0050225"
NR 180
TC 6
Z9 9
U1 12
U2 57
PU SPRINGER INT PUBL AG
PI CHAM
"PA GEWERBESTRASSE 11, CHAM, CH-6330, SWITZERLAND"
SN 0049-6979
EI 1573-2932
J9 WATER AIR SOIL POLL
JI Water Air Soil Pollut.
PD JUN
PY 2024
VL 235
IS 6
AR 431
DI 10.1007/s11270-024-07251-w
PG 25
WC Environmental Sciences; Meteorology & Atmospheric Sciences; Water
   Resources
WE Science Citation Index Expanded (SCI-EXPANDED)

SC Environmental Sciences & Ecology; Meteorology & Atmospheric Sciences;
   Water Resources
GA UP1C4
UT WOS:001249158800004
DA 2026-04-06
ER

PT J
"AU Feng, HP"
"   Wang, BJ"
"   Wang, MM"
"   Ye, DD"
"   Wang, M"
"   Sun, XY"
"   Duan, YL"
"   Li, D"
"   Zhang, X"
"   Zhu, ZQ"
"AF Feng, Huiping"
"   Wang, Baijie"
"   Wang, Miaomiao"
"   Ye, Dandan"
"   Wang, Meng"
"   Sun, Xiaoyan"
"   Duan, Yali"
"   Li, Dong"
"   Zhang, Xin"
"   Zhu, Zhiqiang"
TI Effective soil remediation with fungal Co-inoculation and king grass for
   robust cadmium and chromium phytoextraction
SO ENVIRONMENTAL POLLUTION
LA English
DT Article
DE Cd-Cr resilient fungi; Cadmium sorption; Chromium (VI) reduction;
   Efficacy of removal; Plant-microbial remediation combo
ID MUCOR-CIRCINELLOIDES; AQUEOUS-SOLUTIONS; POLLUTION; PLANT;
   PHYTOREMEDIATION
"AB Bioremediation, an economical and environmentally friendly approach, provides a sustainable solution for mitigating heavy metal contamination in soils. This study identifies four fungal strains- Trichoderma harzianum DAA8, Trichoderma reesei DAA9, Rhizomucor variabilis s DFB3, and Trichoderma asperellum LDA4-that exhibit tolerance to cadmium (Cd) and chromium (Cr). These strains were isolated from soils impacted by heavy metal contamination in mining regions. Rigorous examinations of these strains led us to determine their Minimum Inhibitory Concentrations (MICs) and optimal absorption-reduction conditions. Our microscopic data and GC-MS analysis indicate that these strains can accumulate Cd and Cr by generating compounds, such as ketones and imines, in heavy metal environments. We evaluated the remediation efficacy of both single and co-cultures of Rhizomucor variabilis DFB3 and Trichoderma asperellum LDA4 in conjunction with king grass, a plant known for its heavy metal accumulation capabilities. Our findings indicated an impressive 41.9% increase in plant biomass and 47.2% and 64.4% increase in Cd and Cr accumulation respectively. The removal rates of Cd and Cr were 16.5% and 19.0%, respectively, following the co-inoculation of Rhizomucor variabilis DFB3 and Trichoderma asperellum LDA4. These rates represent increases of 37.1% and 33.7% compared to the removal rates achieved with king grass alone. This study not only advances strategies to manage Cd-Cr contamination but also sets a pathway for efficient heavy metal soil remediation using a microbial-plant combined technique."
"C1 [Feng, Huiping; Wang, Baijie; Wang, Miaomiao; Ye, Dandan; Wang, Meng; Sun, Xiaoyan; Duan, Yali; Li, Dong; Zhu, Zhiqiang] Hainan Univ, Sch Trop Agr & Forestry, Sch Environm Sci & Engn, Haikou 570228, Peoples R China."
"   [Zhang, Xin] Chinese Acad Sci, Res Ctr Ecoenvironm Sci, State Key Lab Urban & Reg Ecol, Beijing 100085, Peoples R China."
"   [Zhang, Xin] Univ Chinese Acad Sci, Beijing 100049, Peoples R China."
C3 Hainan University; Chinese Academy of Sciences; Research Center for
"   Eco-Environmental Sciences (RCEES), CAS; Chinese Academy of Sciences;"
"   University of Chinese Academy of Sciences, CAS"
"RP Li, D; Zhu, ZQ (corresponding author), Hainan Univ, Sch Trop Agr & Forestry, Sch Environm Sci & Engn, Haikou 570228, Peoples R China."
EM lidong2021@hainanu.edu.cn; zqzhu@hainanu.edu.cn
"RI Feng, Huiping/KVB-9265-2024; Li, Dong/OHR-5404-2025; Wang,"
   miaomiao/JGD-4088-2023
"OI Zhu, Zhiqiang/0000-0002-7479-9279; ???, ???/0000-0001-8504-5213"
"FU National Natural Science Foundation of China [42207467, 42377016,"
"   42067027, 41867025]; Graduate Innovation Research Project of Hainan"
"   Province, China [Qhys2022-84]; National Key Research and Devel-opment"
"   Program of China [2023YFC3709103, 2023YFD1901405]"
FX This work was supported by the National Natural Science Foundation of
"   China (Grant Nos. 42207467, 42377016, 42067027 and 41867025) , the"
"   Graduate Innovation Research Project of Hainan Province, China (Grant"
"   No. Qhys2022-84) , and the National Key Research and Devel-opment"
   Program of China (Grant Nos. 2023YFC3709103 and 2023YFD1901405) .
"CR Anahid S, 2011, SCI IRAN, V18, P502, DOI 10.1016/j.scient.2011.05.015"
"   Asghar W, 2024, PLANT STRESS, V12, DOI 10.1016/j.stress.2024.100455"
"   Azhar U, 2022, ENVIRON RES, V214, DOI 10.1016/j.envres.2022.113918"
"   Babu AG, 2014, ECOL ENG, V69, P186, DOI 10.1016/j.ecoleng.2014.03.055"
"   Bao S D., 2010, Soil Agro-Chemistrical Analysis, V3rd"
"   Borah Pallabi, 2018, Groundwater for Sustainable Development, V6, P189, DOI [10.1016/j.gsd.2018.01.002, 10.1016/j.gsd.2018.01.002]"

"   Chang F, 2016, CHEM ENG J, V298, P75, DOI 10.1016/j.cej.2016.04.023"
"   Chen YH, 2017, J SOIL SEDIMENT, V17, P2786, DOI 10.1007/s11368-017-1703-7"
"   Cui ZJ, 2017, J ENVIRON CHEM ENG, V5, P3616, DOI 10.1016/j.jece.2017.07.021"
"   Deng Y, 2021, MINERALS-BASEL, V11, DOI 10.3390/min11060622"
"   Deshmukh R, 2016, INDIAN J MICROBIOL, V56, P247, DOI 10.1007/s12088-016-0584-6"
"   Ding QF, 2017, CHEMOSPHERE, V188, P320, DOI 10.1016/j.chemosphere.2017.08.124"
"   Elhamouly NA, 2022, FRONT PLANT SCI, V13, DOI 10.3389/fpls.2022.1044896"
"   Gola D, 2018, ENVIRON SCI POLLUT R, V25, P20486, DOI 10.1007/s11356-017-0246-1"
"   Henao SG, 2021, FRONT ENV SCI-SWITZ, V9, DOI 10.3389/fenvs.2021.604216"
"   Gui H, 2023, ENVIRON RES, V222, DOI 10.1016/j.envres.2023.115328"
"   Guti??rrez JC, 2015, FRONT MICROBIOL, V6, DOI 10.3389/fmicb.2015.00048"
"   Harman GE, 2004, NAT REV MICROBIOL, V2, P43, DOI 10.1038/nrmicro797"
"   Hassan A, 2022, APPL SOIL ECOL, V170, DOI 10.1016/j.apsoil.2021.104307"
"   He MY, 2022, ECOTOX ENVIRON SAFE, V236, DOI 10.1016/j.ecoenv.2022.113497"
"   Hu BF, 2020, ENVIRON POLLUT, V266, DOI 10.1016/j.envpol.2020.114961"
"   Imran M, 2020, J PURE APPL MICROBIO, V14, P157, DOI 10.22207/JPAM.14.1.17"
"   Lakhdari W, 2023, MOLECULES, V28, DOI 10.3390/molecules28135025"
"   Li D, 2022, J HAZARD MATER, V422, DOI 10.1016/j.jhazmat.2021.126936"
"   Li Q, 2022, SCI TOTAL ENVIRON, V825, DOI 10.1016/j.scitotenv.2022.154136"
"   Liu HT, 2023, J HAZARD MATER, V444, DOI 10.1016/j.jhazmat.2022.130376"
"   Manoj SR, 2020, J ENVIRON MANAGE, V254, DOI 10.1016/j.jenvman.2019.109779"
"   Mart??nez-Medina A, 2011, PHYTOCHEMISTRY, V72, P223, DOI 10.1016/j.phytochem.2010.11.008"
"   Meier K, 2016, BIOCHEM ENG J, V109, P228, DOI 10.1016/j.bej.2016.01.014"
"   Narayanan M, 2023, J ENVIRON MANAGE, V345, DOI 10.1016/j.jenvman.2023.118732"
"   Oshiquiri LH, 2020, FUNGAL GENET BIOL, V134, DOI 10.1016/j.fgb.2019.103281"
"   Pehlivan N, 2021, J HAZARD MATER, V403, DOI 10.1016/j.jhazmat.2020.123609"
"   Qin HQ, 2020, ENVIRON POLLUT, V258, DOI 10.1016/j.envpol.2019.113777"
"   Singh DP, 2020, MICROBIOL RES, V239, DOI 10.1016/j.micres.2020.126538"
"   Singh M, 2015, J APPL MICROBIOL, V119, P1278, DOI 10.1111/jam.12948"
"   Sol?? VA, 2007, SPECTROCHIM ACTA B, V62, P63, DOI 10.1016/j.sab.2006.12.002"
"   Sud D, 2008, BIORESOURCE TECHNOL, V99, P6017, DOI 10.1016/j.biortech.2007.11.064"
"   Sun XY, 2023, ENVIRON RES, V222, DOI 10.1016/j.envres.2023.115376"
"   Talukdar D, 2020, ENVIRON TECHNOL INNO, V20, DOI 10.1016/j.eti.2020.101050"
"   Vaksmaa A, 2023, FRONT MAR SCI, V10, DOI 10.3389/fmars.2023.1070905"
"   Vinale F, 2008, SOIL BIOL BIOCHEM, V40, P1, DOI 10.1016/j.soilbio.2007.07.002"
"   Wang JJ, 2023, J HAZARD MATER, V445, DOI 10.1016/j.jhazmat.2022.130507"
"   Wang JJ, 2021, SEP PURIF TECHNOL, V268, DOI 10.1016/j.seppur.2021.118689"
"   Wang X, 2021, ENVIRON POLLUT, V277, DOI 10.1016/j.envpol.2021.116758"
"   Wu SH, 2023, J HAZARD MATER, V443, DOI 10.1016/j.jhazmat.2022.130221"
"   Xiang MT, 2021, ENVIRON POLLUT, V278, DOI 10.1016/j.envpol.2021.116911"
"   Xie Y, 2021, ENVIRON RES, V200, DOI 10.1016/j.envres.2021.111730"
"   Yilmazer P, 2009, J CHEM TECHNOL BIOT, V84, P604, DOI 10.1002/jctb.2088"
"   Zeng SY, 2019, SCI TOTAL ENVIRON, V687, P642, DOI 10.1016/j.scitotenv.2019.05.291"
"   Zerizghi T, 2022, SCI TOTAL ENVIRON, V814, DOI 10.1016/j.scitotenv.2021.152653"
"   Zhang HX, 2024, J HAZARD MATER, V465, DOI 10.1016/j.jhazmat.2023.133266"
"   Zhang KL, 2024, J HAZARD MATER, V462, DOI 10.1016/j.jhazmat.2023.132728"
"   Zhang X, 2018, ECOTOX ENVIRON SAFE, V157, P21, DOI 10.1016/j.ecoenv.2018.03.047"
"   Zhou ZQ, 2020, ENVIRON SCI POLLUT R, V27, P35249, DOI 10.1007/s11356-020-09844-4"
NR 54
TC 6
Z9 6
U1 15
U2 60
PU ELSEVIER SCI LTD
PI London
"PA 125 London Wall, London, the United Kingdom"
SN 0269-7491
EI 1873-6424
J9 ENVIRON POLLUT
JI Environ. Pollut.
PD DEC 1
PY 2024
VL 362
AR 124979
DI 10.1016/j.envpol.2024.124979
EA SEP 2024
PG 11
WC Environmental Sciences
WE Science Citation Index Expanded (SCI-EXPANDED)
SC Environmental Sciences & Ecology
GA H0T8M
UT WOS:001320664900001
PM 39293658
DA 2026-04-06
ER

PT J
"AU He, YY"
"   Li, CY"
"   Sun, ZY"
"   Zhang, W"
"   He, JN"
"   Zhao, YL"
"   Xu, ZG"

"   Zhao, WP"
"AF He, Yiying"
"   Li, Chaoyang"
"   Sun, Zhongyu"
"   Zhang, Wan"
"   He, Jianing"
"   Zhao, Yunlin"
"   Xu, Zhenggang"
"   Zhao, Weiping"
"TI Penicillium spp. XK10, Fungi with Potential to Repair Cadmium and"
Antimony Pollution
SO APPLIED SCIENCES-BASEL
LA English
DT Article
DE Penicillium; heavy metal pollution; Cd; Sb; biosorption
ID HEAVY-METAL POLLUTION; BROUSSONETIA-PAPYRIFERA; BIOSORPTION;
OPTIMIZATION; LEAD; CHRYSOGENUM; SOILS
"AB Soil heavy-metal pollution is one of the most important environmental problems in the world, and seriously endangers plant growth and human health. Microbial remediation has become a key technology in the field of soil heavy-metal remediation due to its advantages of being harmless, green and environmental. In this study, a fungus Penicillium spp. XK10 with high tolerance to cadmium (Cd) and antimony (Sb) was screened from mine slag, and its adsorption characteristics to heavy metals under different environmental conditions were studied. The results showed that at pH(0) = 6, C-0 (Cd) = 0.1 mM, and the adsorption time was 4 days, the maximum removal rate of cadmium by XK10 was 32.2%. Under the conditions of pH(0) = 4, T = 7d, and the initial antimony concentration of 1 mM, the removal rate of antimony by XK10 was the highest, which was 15.5%. This study provides potential microbial materials for bioremediation of heavy metal-contaminated soils."
"C1 [He, Yiying; Li, Chaoyang; Zhang, Wan; Zhao, Yunlin; Xu, Zhenggang] Cent South Univ Forestry & Technol, Hunan Res Ctr Engn Technol Utilizat Environm & Res, Changsha 410004, Peoples R China."
"   [Sun, Zhongyu; He, Jianing; Xu, Zhenggang] Northwest A&F Univ, Coll Forestry, Key Lab Natl Forestry & Grassland Adm Management W, Yangling 712100, Peoples R China."
"   [Zhao, Weiping] Hunan Agr Univ, Sch Business, Changsha 410128, Peoples R China."
C3 Central South University of Forestry & Technology; Northwest A&F
University - China; Hunan Agricultural University
"RP Zhao, YL; Xu, ZG (corresponding author), Cent South Univ Forestry & Technol, Hunan Res Ctr Engn Technol Utilizat Environm & Res, Changsha 410004, Peoples R China.; Xu, ZG (corresponding author), Northwest A&F Univ, Coll Forestry, Key Lab Natl Forestry & Grassland Adm Management W, Yangling 712100, Peoples R China."
EM zyl8291290@163.com; xuzhenggang@nwafu.edu.cn
"RI Zhenggang, Xu/ABE-9095-2020"
"OI Zhenggang, Xu/0000-0002-7401-5163"
FU Key Projects of National Forestry and Grassland Bureau [201801]; China
Postdoctoral Science Foundation [2020M683592]; Open Fund of Key
"   Laboratory of Microbial Resources Collection and Preservation, Ministry"
of Agriculture and Rural Affairs [KLMRCP2021-07]
FX This research was funded by the Key Projects of National Forestry and
"   Grassland Bureau (201801), China Postdoctoral Science Foundation"
"   (2020M683592), and Open Fund of Key Laboratory of Microbial Resources"
"   Collection and Preservation, Ministry of Agriculture and Rural Affairs"
(KLMRCP2021-07). The funding bodies played no role in the design of the
"   study and collection, analysis, and interpretation of data and in"
writing the manuscript.
"CR Amini M, 2008, J HAZARD MATER, V154, P694, DOI 10.1016/j.jhazmat.2007.10.114"
"   Arwidsson Z, 2010, WATER AIR SOIL POLL, V207, P5, DOI 10.1007/s11270-009-0222-6"
"   Benbrahim-Tallaa L, 2007, PROSTATE, V67, P135, DOI 10.1002/pros.20479"
"   Cai Y, 2018, INT J ENV RES PUB HE, V15, DOI 10.3390/ijerph15040702"
"   CHANG CH, 1995, COLLOID SURFACE A, V100, P1, DOI 10.1016/0927-7757(94)03061-4"
"   Chien CC, 2013, BIOCHEM ENG J, V78, P132, DOI 10.1016/j.bej.2013.01.014"
"   Choinska-Pulit A, 2018, ECOTOX ENVIRON SAFE, V149, P275, DOI 10.1016/j.ecoenv.2017.12.008"
"   Deng XH, 2012, J HAZARD MATER, V233, P25, DOI 10.1016/j.jhazmat.2012.06.054"
"   Di Z, 2022, FRONT MICROBIOL, V12, DOI 10.3389/fmicb.2021.816312"
"   FOUREST E, 1994, FEMS MICROBIOL REV, V14, P325"
"   Hassan SHA, 2018, CHEMOSPHERE, V202, P750, DOI 10.1016/j.chemosphere.2018.03.114"
"   Huang HM, 2022, FRONT MICROBIOL, V13, DOI 10.3389/fmicb.2022.871581"
"   Huang HM, 2020, CHEMOSPHERE, V260, DOI 10.1016/j.chemosphere.2020.127614"
"   Huang HM, 2019, ECOTOX ENVIRON SAFE, V181, P18, DOI 10.1016/j.ecoenv.2019.05.063"
"   Huang HM, 2018, PLOS ONE, V13, DOI 10.1371/journal.pone.0203285"
"   Jacob JM, 2018, J ENVIRON MANAGE, V217, P56, DOI 10.1016/j.jenvman.2018.03.077"
"   JONES RD, 1994, OCCUP ENVIRON MED, V51, P772, DOI 10.1136/oem.51.11.772"
"   Kapoor A, 1999, BIORESOURCE TECHNOL, V70, P95, DOI 10.1016/S0960-8524(98)00192-8"
"   Khan S, 2008, ENVIRON POLLUT, V152, P686, DOI 10.1016/j.envpol.2007.06.056"
"   Kim JW, 2006, PROCESS BIOCHEM, V41, P1253, DOI 10.1016/j.procbio.2005.12.021"
"   Kiran I, 2006, BIOCHEM ENG J, V31, P197, DOI 10.1016/j.bej.2006.07.008"
"   Kumar V, 2019, CHEMOSPHERE, V237, DOI 10.1016/j.chemosphere.2019.124567"
"   Li F, 2018, J HAZARD MATER, V358, P178, DOI 10.1016/j.jhazmat.2018.06.066"
"   Li JK, 2018, GEOMICROBIOL J, V35, P713, DOI 10.1080/01490451.2018.1454556"
"   Li ZY, 2014, SCI TOTAL ENVIRON, V468, P843, DOI 10.1016/j.scitotenv.2013.08.090"
"   Liu C., 2012, AM J CLIN NUTR, V31, P213"
"   Martins LR, 2016, J ENVIRON ENG, V142, DOI 10.1061/(ASCE)EE.1943-7870.0000998"
"   Mwandira W, 2020, SCI REP-UK, V10, DOI 10.1038/s41598-020-78187-4"
"   Pan JL, 2010, ENVIRON GEOCHEM HLTH, V32, P1, DOI 10.1007/s10653-009-9273-2"
"   Rajendran S, 2022, CHEMOSPHERE, V287, DOI 10.1016/j.chemosphere.2021.132369"
"   Singh M, 2015, J APPL MICROBIOL, V119, P1278, DOI 10.1111/jam.12948"
"   Tian D, 2019, ENVIRON MICROBIOL, V21, P471, DOI 10.1111/1462-2920.14478"
"   Tian D, 2018, ENVIRON SCI POLLUT R, V25, P21118, DOI 10.1007/s11356-018-2243-4"

"   Tong FP, 2008, CHIN AGR SCI B, V12, P179"
"   Tu X., 2021, GUANGDONG CHEM IND, V48, P93"
"   Wang JL, 2009, BIOTECHNOL ADV, V27, P195, DOI 10.1016/j.biotechadv.2008.11.002"
"   Wang Y, 2020, ECOTOX ENVIRON SAFE, V192, DOI 10.1016/j.ecoenv.2020.110294"
"   Wang YJ, 2019, CHEMOSPHERE, V225, P200, DOI 10.1016/j.chemosphere.2019.03.027"
"   Wilson NJ, 2004, ENVIRON POLLUT, V129, P257, DOI 10.1016/j.envpol.2003.10.014"
"   Xia L, 2015, INT J MOL SCI, V16, P15670, DOI 10.3390/ijms160715670"
"   Xu H, 2021, WATER ENVIRON RES, V93, P844, DOI 10.1002/wer.1472"
"   Xu Shaohui, 2019, Chinese Journal of Applied and Environmental Biology, V25, P532, DOI 10.19675/j.cnki.1006-687x.2018.08028"
"   Xu XJ, 2015, FRONT MICROBIOL, V6, DOI 10.3389/fmicb.2015.01422"
"   Xu XJ, 2012, ENVIRON TECHNOL, V33, P1661, DOI 10.1080/09593330.2011.641591"
"   Xu ZG, 2019, POL J ENVIRON STUD, V28, P463, DOI 10.15244/pjoes/84838"
"   [????????? Yang Yongkang], 2020, [??????????????????, Jiangsu Journal of Agricultural Sciences], V36, P1322"
"   Zhang M., 2018, ADV ENERGY MATER, V46, P1321"
NR 47
TC 18
Z9 18
U1 3
U2 66
PU MDPI
PI BASEL
"PA ST ALBAN-ANLAGE 66, CH-4052 BASEL, SWITZERLAND"
EI 2076-3417
J9 APPL SCI-BASEL
JI Appl. Sci.-Basel
PD FEB
PY 2023
VL 13
IS 3
AR 1228
DI 10.3390/app13031228
PG 13
"WC Chemistry, Multidisciplinary; Engineering, Multidisciplinary; Materials"
"   Science, Multidisciplinary; Physics, Applied"
WE Science Citation Index Expanded (SCI-EXPANDED)
SC Chemistry; Engineering; Materials Science; Physics
GA 8T3WN
UT WOS:000929196700001
"OA Green Submitted, gold"
DA 2026-04-06
ER

PT J
"AU Achal, V"
"   Zhang, QZ"
"   Xiang, WN"
"   Wang, DC"
"AF Achal, Varenyam"
"   Zhang, Qiuzhuo"
"   Xiang, Wei-Ning"
"   Wang, Duanchao"
TI ABILITY OF Shewanella oneidensis FOR THE REMEDIATION OF HEAVY
   METALS FROM URBAN ROAD DUSTS
SO FRESENIUS ENVIRONMENTAL BULLETIN
LA English
DT Article
DE Dusts; Shewanella oneidensis; Heavy metals; Calcite; Bioremediation
ID STREET DUST; CONTAMINATED SOIL; TRACE-ELEMENTS; CALCITE; SPECIATION;
   BACTERIUM; SEDIMENTS
"AB Heavy metals present in urban road dusts are hazardous to human health as well as environment. Although there are many reports of such pollution, remediation process for urban road dusts has not been studied. In the present study, road dusts were collected from different parts of Shanghai, China and were analyzed for the presence of heavy metals. A novel concept of microbially induced calcite precipitation (MICP) was employed in the remediation process. Shewanella oneidensis VA6 a river isolate, with multiple metal resistant abilities, was identified based on 16S rDNA sequencing and used in this study. This bacterium remediated more than 90% of six different heavy metals (Zn, Cr, Pb, Cu, Cd, and Co) from urban road dusts. Further the efficiency of MICP was analyzed by X-Ray Diffraction (XRD), which plays a role for various biomineralization products such as calcite, aragonite, vaterite and gwihabaite in the process of remediation. This is the first extensive study where remediation of heavy metals from road dusts was studied."
"C1 [Achal, Varenyam; Zhang, Qiuzhuo; Xiang, Wei-Ning; Wang, Duanchao] E China Normal Univ, Coll Resources & Environm Sci, Shanghai Key Lab Urban Ecol Proc & Ecorestorat SH, Shanghai 200241, Peoples R China."
C3 East China Normal University
"RP Achal, V (corresponding author), E China Normal Univ, Coll Resources & Environm Sci, Shanghai Key Lab Urban Ecol Proc & Ecorestorat SH, Shanghai 200241, Peoples R China."
EM varenyam@re.ecnu.edu.cn
"RI achal, varenyam/D-3442-2011"
"OI achal, varenyam/0000-0001-5444-7746"
FU Natural Science Foundation of China [41130525]; Shanghai Science and
   Technology Committee [12231205300]; Shanghai Key Lab for Urban
   Ecological Processes and Eco-Restoration [44503545]
FX This work was supported by grants from Natural Science Foundation of
"   China (No.41130525), Shanghai Science and Technology Committee"

(No.12231205300) and Shanghai Key Lab for Urban Ecological Processes and
Eco-Restoration (Project number 44503545).
"CR Achal V, 2009, J IND MICROBIOL BIOT, V36, P981, DOI 10.1007/s10295-009-0578-z"
"   Achal V, 2012, J MICROBIOL BIOTECHN, V22, P244, DOI 10.4014/jmb.1108.08033"
"   Achal V, 2011, ECOL ENG, V37, P1601, DOI 10.1016/j.ecoleng.2011.06.008"
"   Achal V, 2011, CURR MICROBIOL, V62, P894, DOI 10.1007/s00284-010-9801-4"
"   Al-Khashman OA, 2004, ATMOS ENVIRON, V38, P6803, DOI 10.1016/j.atmosenv.2004.09.011"
"   Alexandratos VG, 2007, GEOCHIM COSMOCHIM AC, V71, P4172, DOI 10.1016/j.gca.2007.06.055"
"   Altschul SF, 1997, NUCLEIC ACIDS RES, V25, P3389, DOI 10.1093/nar/25.17.3389"
"   Banerjee ADK, 2003, ENVIRON POLLUT, V123, P95, DOI 10.1016/S0269-7491(02)00337-8"
"   BelzunceSegarra MJ, 1997, J ENVIRON SCI HEAL A, V32, P1271, DOI 10.1080/10934529709376609"
"   Chompoothawat N, 2010, FRESEN ENVIRON BULL, V19, P2919"
"   Cook AG, 2005, BIOL TRACE ELEM RES, V103, P1, DOI 10.1385/BTER:103:1:001"
"   deMiguel E, 1997, ATMOS ENVIRON, V31, P2733, DOI 10.1016/S1352-2310(97)00101-5"
"   Ferreira-Baptista L, 2005, ATMOS ENVIRON, V39, P4501, DOI 10.1016/j.atmosenv.2005.03.026"
"   Gadd GM, 2000, CURR OPIN BIOTECH, V11, P271, DOI 10.1016/S0958-1669(00)00095-1"
"   Han YM, 2006, SCI TOTAL ENVIRON, V355, P176, DOI 10.1016/j.scitotenv.2005.02.026"
"   Martini J.E.J., 1996, S AFRICAN SPELEOLOGI, V36, P19"
"   PARDO R, 1990, WATER RES, V24, P373, DOI 10.1016/0043-1354(90)90016-Y"
"   Rom??n-Ross G, 2006, CHEM GEOL, V233, P328, DOI 10.1016/j.chemgeo.2006.04.007"
"   Tamura K, 2007, MOL BIOL EVOL, V24, P1596, DOI 10.1093/molbev/msm092"
"   TESSIER A, 1979, ANAL CHEM, V51, P844, DOI 10.1021/ac50043a017"
"   Wang XS, 2007, ENVIRON GEOCHEM HLTH, V29, P11, DOI 10.1007/s10653-006-9052-2"
"   Wei BG, 2010, ENVIRON EARTH SCI, V61, P1187, DOI 10.1007/s12665-009-0441-6"
"   Wei BG, 2010, MICROCHEM J, V94, P99, DOI 10.1016/j.microc.2009.09.014"
"   Yoshida N, 2010, APPL ENVIRON MICROB, V76, P7322, DOI 10.1128/AEM.01767-10"
NR 24
TC 1
Z9 1
U1 1
U2 28
PU PARLAR SCIENTIFIC PUBLICATIONS (P S P)
PI FREISING
"PA ANGERSTR. 12, 85354 FREISING, GERMANY"
SN 1018-4619
EI 1610-2304
J9 FRESEN ENVIRON BULL
JI Fresenius Environ. Bull.
PY 2013
VL 22
IS 11
BP 3223
EP 3228
PG 6
WC Environmental Sciences
WE Science Citation Index Expanded (SCI-EXPANDED)
SC Environmental Sciences & Ecology
GA 260DR
UT WOS:000327575700015
DA 2026-04-06
ER

PT J
"AU Han, H"
"   Wu, XJ"
"   Bolan, N"
"   Kirkham, MB"
"   Yang, JJ"
"   Chen, ZJ"
"AF Han, Hui"
"   Wu, Xuejiao"
"   Bolan, Nanthi"
"   Kirkham, M. B."
"   Yang, Jianjun"
"   Chen, Zhaojin"
TI Inhibition of cadmium uptake by wheat with urease-producing bacteria
combined with sheep manure under field conditions
SO CHEMOSPHERE
LA English
DT Article
DE Urease-producing bacteria; Immobilization; Sheep manure; Wheat; Field
experiment
ID HEAVY METAL-RESISTANT; PLANT-GROWTH; CONTAMINATED SOIL; FARMLAND SOIL;
YANGTZE-RIVER; CD TOXICITY; RICE; ACCUMULATION; IMMOBILIZATION;
FERTILIZER
"AB In heavy metal-contaminated farmland, microorganisms or organic fertilizers can be used to minimize heavy metal uptake by crops to ensure food safety. However, the mechanisms by which urease-producing and metal-immobilizing bacteria combined with manure inhibit Cd uptake in wheat (Triticum aestivum L.) remain unclear. Herein, the effects of Enterobacter bugandensis TJ6, sheep manure (SM), and TJ6 combined with SM on Cd uptake by wheat and the mechanisms involved were investigated under field conditions. The results showed that strain TJ6 increased the urease activity and the proportion of strains with a high Cd adsorption capacity in SM, thereby enhancing the Cd adsorption capacity of SM in solution. Strain TJ6 combined with SM improved the rhizosphere soil urease activity, NH4+/NO3- ratio, and pH, thus reducing the Cd content (75.9%) in wheat grain. In addition, TJ6+SM reduced the bacterial community diversity but shifted the structure of the

bacterial community in rhizosphere soil. Interestingly, the relative abundances of urease-producing bacteria and metal-immobilizing bacteria (Enterobacter, Bacillus, Exiguobacterium, Rhizobium, and Serratia) in rhizosphere soil were enriched, which enhanced wheat resistance to Cd toxicity. These results showed that urease-producing and metal-immobilizing bacteria combined with sheep manure can inhibit the uptake of Cd by wheat."
"C1 [Han, Hui; Yang, Jianjun] Chinese Acad Agr Sci, Inst Environm & Sustainable Dev Agr, Beijing 100081, Peoples R China."
"   [Han, Hui; Wu, Xuejiao; Chen, Zhaojin] Nanyang Normal Univ, Coll Life Sci & Agr Engn, Collaborat Innovat Water Secur Water Source Reg M, Nanyang 473061, Peoples R China."
"   [Bolan, Nanthi] Univ Newcastle, Global Ctr Environm Remediat, Callaghan Campus, Callaghan, NSW 2308, Australia."
"   [Kirkham, M. B.] Kansas State Univ, Dept Agron, Manhattan, KS 66506 USA."
C3 Chinese Academy of Agricultural Sciences; Institute of Environment &
"   Sustainable Development in Agriculture, CAAS; Nanyang Normal University;"
   University of Newcastle; Kansas State University
"RP Yang, JJ (corresponding author), Chinese Acad Agr Sci, Inst Environm & Sustainable Dev Agr, Beijing 100081, Peoples R China."
EM yangjianjun@caas.cn
"RI ; Bolan, Nanthi/E-8535-2011; Han, Hui/GYJ-6725-2022"
"OI ?????????, Zhaojin Chen/0000-0003-1088-6821; Bolan,"
"   Nanthi/0000-0003-2056-1692; Han, Hui/0000-0002-5218-0109"
"FU National Natural Science Foundation of China [41907143, U2004145]; China"
   Postdoctoral Science Foundation [2020M680775]; Youth Talent Promotion
   Project of Henan Province [2022HYTP033]; Major Science and Technology
   Special Projects of Nanyang [2019ZDZX10]; National Key Research and
   Development Program of China [2018YFD0800305]
FX This research was funded by the National Natural Science Foundation of
"   China (41907143, U2004145) , the China Postdoctoral Science Foundation"
"   (2020M680775), the Youth Talent Promotion Project of Henan Province"
"   (2022HYTP033), the Major Science and Technology Special Projects of"
   Nanyang (2019ZDZX10) and National Key Research and Development Program
   of China (2018YFD0800305).
"CR Ahmadabadi Z, 2021, ECOTOX ENVIRON SAFE, V208, DOI 10.1016/j.ecoenv.2020.111408"
"   Akahane I., 2010, Pedologist, V53, P101"
"   Alam M, 2020, SCI HORTIC-AMSTERDAM, V262, DOI 10.1016/j.scienta.2019.109067"
"   Altimira F, 2012, BMC MICROBIOL, V12, DOI 10.1186/1471-2180-12-193"
"   Bhattacharya A, 2018, J ENVIRON MANAGE, V215, P143, DOI 10.1016/j.jenvman.2018.03.055"
"   Blonska E, 2021, APPL SOIL ECOL, V157, DOI 10.1016/j.apsoil.2020.103723"
"   Bolan NS, 2013, ADV AGRON, V119, P183, DOI 10.1016/B978-0-12-407247-3.00004-4"
"   Bravo S, 2017, J GEOCHEM EXPLOR, V174, P79, DOI 10.1016/j.gexplo.2015.12.012"
"   Burkhardt EM, 2011, APPL ENVIRON MICROB, V77, P3132, DOI 10.1128/AEM.02085-10"
"   Carpio IEM, 2018, ENVIRON SCI TECHNOL, V52, P184, DOI 10.1021/acs.est.7b04131"
"   Chen L, 2016, J HAZARD MATER, V312, P123, DOI 10.1016/j.jhazmat.2016.03.042"
"   Chen Z., 2021, SCI TOTAL ENVIRON, V753"
"   Cheng C, 2020, SCI TOTAL ENVIRON, V740, DOI 10.1016/j.scitotenv.2020.139972"
"   Chuaphasuk C, 2019, ENVIRON SCI POLLUT R, V26, P23679, DOI 10.1007/s11356-019-05661-6"
"   Edgar RC, 2010, BIOINFORMATICS, V26, P2460, DOI 10.1093/bioinformatics/btq461"
"   Elouear Z, 2016, SUSTAIN ENVIRON RES, V26, P131, DOI 10.1016/j.serj.2016.04.004"
"   Etesami H, 2018, ECOTOX ENVIRON SAFE, V147, P175, DOI 10.1016/j.ecoenv.2017.08.032"
"   Fan MC, 2018, MICROBIOL RES, V217, P51, DOI 10.1016/j.micres.2018.09.002"
"   Fisher KA, 2017, GEODERMA, V285, P1, DOI 10.1016/j.geoderma.2016.09.012"
"   FLORIJN PJ, 1992, J PLANT NUTR, V15, P2405, DOI 10.1080/01904169209364483"
"   Gong LD, 2021, CHEMOSPHERE, V262, DOI 10.1016/j.chemosphere.2020.127828"
"   Han H, 2021, CHEMOSPHERE, V276, DOI 10.1016/j.chemosphere.2021.130157"
"   Han H, 2020, ENVIRON POLLUT, V267, DOI 10.1016/j.envpol.2020.115432"
"   Harindintwali JD, 2020, ECOTOX ENVIRON SAFE, V204, DOI 10.1016/j.ecoenv.2020.111020"
"   Hassan MJ, 2005, PLANT SOIL, V277, P359, DOI 10.1007/s11104-005-8160-6"
"   Hassan MJ, 2008, PLANT GROWTH REGUL, V54, P125, DOI 10.1007/s10725-007-9235-6"
"   Jalloh MA, 2009, J HAZARD MATER, V162, P1081, DOI 10.1016/j.jhazmat.2008.05.146"
"   Ju WL, 2019, ECOTOX ENVIRON SAFE, V167, P218, DOI 10.1016/j.ecoenv.2018.10.016"
"   Kemper W. D., 1986, Methods of soil analysis. Part 1. Physical and mineralogical methods, P425"
"   Khatiwada B, 2020, ALGAL RES, V45, DOI 10.1016/j.algal.2019.101764"
"   Kikot P, 2010, HYDROMETALLURGY, V104, P494, DOI 10.1016/j.hydromet.2010.02.026"
"   Kumari D, 2014, INT BIODETER BIODEGR, V94, P98, DOI 10.1016/j.ibiod.2014.07.007"
"   Langille MGI, 2013, NAT BIOTECHNOL, V31, P814, DOI 10.1038/nbt.2676"
"   Li M, 2013, INT BIODETER BIODEGR, V76, P81, DOI 10.1016/j.ibiod.2012.06.016"
"   Li RF, 2019, ENVIRON POLLUT, V255, DOI 10.1016/j.envpol.2019.113170"
"   Li Y, 2017, ECOTOX ENVIRON SAFE, V138, P56, DOI 10.1016/j.ecoenv.2016.12.024"
"   Liu LH, 2021, J HAZARD MATER, V403, DOI 10.1016/j.jhazmat.2020.123648"
"   Liu YL, 2020, CHEMOSPHERE, V247, DOI 10.1016/j.chemosphere.2020.125850"
"   Lu Q, 2020, ENVIRON POLLUT, V257, DOI 10.1016/j.envpol.2019.113497"
"   Lu WB, 2006, J HAZARD MATER, V134, P80, DOI 10.1016/j.jhazmat.2005.10.036"
"   Luo BF, 2012, J EXP BOT, V63, P3127, DOI 10.1093/jxb/ers036"
"   Madhaiyan M, 2007, CHEMOSPHERE, V69, P220, DOI 10.1016/j.chemosphere.2007.04.017"
"   Maier NA, 2002, J PLANT NUTR, V25, P523, DOI 10.1081/PLN-120003380"
"   Mayanna S, 2015, CHEM GEOL, V402, P6, DOI 10.1016/j.chemgeo.2015.02.029"
"   Medina-Sauza R.M., RHIZOSPHERE-NETH, V21, P2022"
"   Mitchell LG, 2000, CAN J SOIL SCI, V80, P107, DOI 10.4141/S98-085"
"   Park JH, 2011, J ENVIRON MANAGE, V92, P1115, DOI 10.1016/j.jenvman.2010.11.031"
"   Pathania P, 2020, BIOCATAL AGR BIOTECH, V30, DOI 10.1016/j.bcab.2020.101842"
"   Rehman MZU, 2018, ENVIRON POLLUT, V241, P557, DOI 10.1016/j.envpol.2018.06.005"
"   Rigby H, 2020, ENVIRON INT, V143, DOI 10.1016/j.envint.2020.105844"
"   de Miera LES, 2014, INT J GREENH GAS CON, V29, P176, DOI 10.1016/j.ijggc.2014.08.014"
"   Shan SP, 2020, SCI TOTAL ENVIRON, V708, DOI 10.1016/j.scitotenv.2019.135224"

"   Shao DW, 2016, ENVIRON POLLUT, V219, P329, DOI 10.1016/j.envpol.2016.10.023"
"   Shi L, 2019, ECOTOX ENVIRON SAFE, V171, P425, DOI 10.1016/j.ecoenv.2019.01.006"
"   Sizmur T, 2020, SOIL BIOL BIOCHEM, V148, DOI 10.1016/j.soilbio.2020.107865"
"   Skowronska M, 2020, CATENA, V189, DOI 10.1016/j.catena.2020.104484"
"   SSICA (Soil Sci Ch. Acad.), 1980, DISSERTATION"
"   Tahir M, 2018, BRAZ J MICROBIOL, V49, P15, DOI 10.1016/j.bjm.2017.11.005"
"   Tang X, 2016, J ENVIRON MANAGE, V181, P646, DOI 10.1016/j.jenvman.2016.08.043"
"   Teng ZD, 2019, J ENVIRON MANAGE, V231, P189, DOI 10.1016/j.jenvman.2018.10.012"
"   TESSIER A, 1979, ANAL CHEM, V51, P844, DOI 10.1021/ac50043a017"
"   Touceda-Gonzalez M, 2015, SOIL BIOL BIOCHEM, V91, P140, DOI 10.1016/j.soilbio.2015.08.038"
"   Wan WJ, 2020, SCI TOTAL ENVIRON, V700, DOI 10.1016/j.scitotenv.2019.134418"
"   Wang TJ, 2020, ENVIRON SCI POLLUT R, V27, P8707, DOI 10.1007/s11356-019-06957-3"
"   Wang XB, 2020, PEDOSPHERE, V30, P555, DOI 10.1016/S1002-0160(17)60457-0"
"   Wu YC, 2017, SCI REP-UK, V7, DOI [10.1038/srep40093, 10.1038/srep41076]"
"   Wu ZX, 2020, RHIZOSPHERE-NETH, V16, DOI 10.1016/j.rhisph.2020.100264"
"   Yang YJ, 2020, SCI TOTAL ENVIRON, V708, DOI 10.1016/j.scitotenv.2019.135186"
"   Yi YJ, 2017, ECOTOX ENVIRON SAFE, V145, P295, DOI 10.1016/j.ecoenv.2017.07.022"
"   Zeng FR, 2011, ENVIRON POLLUT, V159, P84, DOI 10.1016/j.envpol.2010.09.019"
"   Zhou JY, 2020, ENVIRON POLLUT, V260, DOI 10.1016/j.envpol.2020.113990"
"   Zhou J, 2020, ENVIRON POLLUT, V265, DOI 10.1016/j.envpol.2020.115045"
"   Zia A, 2020, ENVIRON POLLUT, V265, DOI 10.1016/j.envpol.2020.114981"
"   Zuo XJ, 2019, SCI TOTAL ENVIRON, V676, P447, DOI 10.1016/j.scitotenv.2019.04.340"
NR 74
TC 25
Z9 28
U1 7
U2 152
PU PERGAMON-ELSEVIER SCIENCE LTD
PI OXFORD
"PA THE BOULEVARD, LANGFORD LANE, KIDLINGTON, OXFORD OX5 1GB, the United Kingdom"
SN 0045-6535
EI 1879-1298
J9 CHEMOSPHERE
JI Chemosphere
PD APR
PY 2022
VL 293
AR 133534
DI 10.1016/j.chemosphere.2022.133534
EA JAN 2022
PG 10
WC Environmental Sciences
WE Science Citation Index Expanded (SCI-EXPANDED)
SC Environmental Sciences & Ecology
GA ZD6BK
UT WOS:000758283100004
PM 34999099
DA 2026-04-06
ER

PT J
"AU Wang, ZH"
"   Zhang, H"
"   Xiong, Y"
"   Zhang, LY"
"   Cui, JL"
"   Li, GW"
"   Du, CL"
"   Wen, KY"
"AF Wang, Zihe"
"   Zhang, Han"
"   Xiong, Ying"
"   Zhang, Lieyu"
"   Cui, Jianglong"
"   Li, Guowen"
"   Du, Caili"
"   Wen, Kaiyang"
TI Remediation mechanism of high concentrations of multiple heavy metals in
   contaminated soil by Sedum alfredii and native microorganisms
SO JOURNAL OF ENVIRONMENTAL SCIENCES
LA English
DT Article
DE Microbial remediation; Phytoremediation; Sedum alfredii
ID CADMIUM; TOPSOIL; HEALTH; GROWTH; ZINC
"AB Pollution accident of nonferrous metallurgy industry often lead to serious heavy metal pollution of the surrounding soil. Phytoremediation of contaminated soil is an environmental and sustainable technology, and soil native microorganisms in the process of phytoremediation also participate in the remediation of heavy metals. However, the effects of high concentrations of multiple heavy metals (HCMHMs) on plants and native soil microorganisms remain uncertain. Thus, further clarification of the mechanism of phytoremediation of HCMHMs soil by plants and native soil microorganisms is required. Using the plant Sedum alfredii (S. alfredii) to restore HCMHM-contaminated soil, we further explored the mechanism of S. alfredii and native soil microorganisms in the remediation of HCMHM soils. The results showed that (i) S. alfredii can promote heavy metals from non-rhizosphere soil to rhizosphere soil, which is conducive to the effect of plants on heavy metals. In addition, it can also enrich the absorbed heavy metals in its roots and leaves; (ii) native

soil bacteria can increase the abundance of signal molecule-synthesizing enzymes, such as trpE, trpG, bjaI, rpfF, ACSL, and yidC, and promote the expression of the pathway that converts serine to cysteine, then synthesize substances to chelate heavy metals. In addition, we speculated that genes such as K19703, K07891, K09711, K19703, K07891, and K09711 in native bacteria may be involved in the stabilization or absorption of heavy metals. The results provide scientific basis for S. alfredii to remediate heavy metals contaminated soils, and confirm the potential of phytoremediation of HCMHM contaminated soil. (c) 2024 The Research Center for Eco-Environmental Sciences, Chinese Academy of Sciences. Published by Elsevier B.V."
"C1 [Wang, Zihe; Zhang, Han; Zhang, Lieyu; Cui, Jianglong; Li, Guowen; Du, Caili] Chinese Res Inst Environm Sci, State Key Lab Environm Criteria & Risk Assessment, Beijing 100012, Peoples R China."
"   [Xiong, Ying] Beijing Water Sci & Technol Inst, Beijing 100044, Peoples R China."
"   [Wen, Kaiyang] Chengdu Univ, Sch Architecture & Civil Engn, Chengdu 610106, Peoples R China."
C3 Chinese Research Academy of Environmental Sciences; Chengdu University
"RP Zhang, H (corresponding author), Chinese Res Inst Environm Sci, State Key Lab Environm Criteria & Risk Assessment, Beijing 100012, Peoples R China."
EM 851747344@qq.com
"RI Zhang, Han/KCL-1470-2024"
FU Fundamental Research Funds for Central Public Welfare Scientific
Research Institutes of China [2021-JY-37]; Yellow River Basin Ecological
Protection and High-quality Development Joint Study (Phase I)
[2022-YRUC-01-0202]
FX This research was supported by the Fundamental Research Funds for
Central Public Welfare Scientific Research Institutes of China (No.
2021-JY-37) and the Yellow River Basin Ecological Protection and
High-quality Development Joint Study (Phase I) (No. 2022-YRUC-01-0202) .
"CR Ali H, 2013, CHEMOSPHERE, V91, P869, DOI 10.1016/j.chemosphere.2013.01.075"
"   Broadley MR, 2007, NEW PHYTOL, V173, P677, DOI 10.1111/j.1469-8137.2007.01996.x"
"   Cai YM, 2019, ENVIRON POLLUT, V253, P959, DOI 10.1016/j.envpol.2019.07.077"
"   Chen M, 2015, BIOTECHNOL ADV, V33, P745, DOI 10.1016/j.biotechadv.2015.05.003"
"   Chen SS, 2018, INT J MOL SCI, V19, DOI 10.3390/ijms19041216"
"   Chen XD, 2018, ECOTOX ENVIRON SAFE, V151, P153, DOI 10.1016/j.ecoenv.2018.01.010"
"   Chen ZQ, 2022, ENVIRON POLLUT, V293, DOI 10.1016/j.envpol.2021.118510"
"   De Marco P, 1999, J BACTERIOL, V181, P2244, DOI 10.1128/JB.181.7.2244-2251.1999"
"   Doran JW, 2002, AGR ECOSYST ENVIRON, V88, P119, DOI 10.1016/S0167-8809(01)00246-8"
"   Du ST, 2022, J HAZARD MATER, V424, DOI 10.1016/j.jhazmat.2021.127638"
"   Eckhardt M, 2005, BIOCHEM J, V388, P245, DOI 10.1042/BJ20041451"
"   Gallardo-Benavente C, 2021, GENES-BASEL, V12, DOI 10.3390/genes12020187"
"   Guo JK, 2020, J ENVIRON SCI, V88, P361, DOI 10.1016/j.jes.2019.10.001"
"   HIGHAM DP, 1986, ENVIRON HEALTH PERSP, V65, P5, DOI 10.2307/3430155"
"   Hou DD, 2018, APPL ENVIRON MICROB, V84, DOI [10.1128/AEM.02797-17, 10.1128/aem.02797-17]"
"   Jia FX, 2021, WATER ENVIRON RES, V93, P2549, DOI 10.1002/wer.1529"
"   Jin JQ, 2020, APPL ENVIRON MICROB, V86, DOI 10.1128/AEM.01359-20"
"   Kemper T, 2002, ENVIRON SCI TECHNOL, V36, P2742, DOI 10.1021/es015747j"
"   Kredich Nicholas M, 2008, EcoSal Plus, V3, DOI 10.1128/ecosalplus.3.6.1.11"
"   Kumar PS, 2018, ENERGY ENV SUSTAIN, P165, DOI 10.1007/978-981-10-7485-1_9"
"   Liu KH, 2020, CHEMOSPHERE, V251, DOI 10.1016/j.chemosphere.2020.126415"
"   Magad??n JG, 2006, MOL CELL BIOL, V26, P2595, DOI 10.1128/MCB.26.7.2595-2614.2006"
"   Martins L. S., 2011, Journal of Applied Sciences Research, P160"
"   Mas??k J, 2014, FEMS MICROBIOL ECOL, V89, P1, DOI 10.1111/1574-6941.12344"
"   Munshi S, 2021, DALTON T, V50, P5590, DOI 10.1039/d0dt04421k"
"   Nemerow N.L., 1974, Scientific Stream Pollution Analysis"
"   Noghabi KA, 2007, PROCESS BIOCHEM, V42, P847, DOI 10.1016/j.procbio.2007.02.004"
"   O'Toole G, 2000, ANNU REV MICROBIOL, V54, P49, DOI 10.1146/annurev.micro.54.1.49"
"   Raspanti E, 2009, CHEMOSPHERE, V76, P48, DOI 10.1016/j.chemosphere.2009.02.030"
"   Rizwan M, 2019, ENVIRON SCI POLLUT R, V26, P6279, DOI 10.1007/s11356-019-04174-6"
"   Ryan RP, 2015, PLOS PATHOG, V11, DOI 10.1371/journal.ppat.1004986"
"   Schomburg D., 1998, Enzyme Handbook 16: First Supplement Part 2 Class 3: Hydrolases, P553"
"   Shah V, 2020, ENVIRON TECHNOL INNO, V18, DOI 10.1016/j.eti.2020.100774"
"   Sharma P, 2021, BIORESOURCE TECHNOL, V328, DOI 10.1016/j.biortech.2021.124835"
"   Song PP, 2022, SCI TOTAL ENVIRON, V838, DOI 10.1016/j.scitotenv.2022.156417"
"   Song SH, 2020, ISME J, V14, P2248, DOI 10.1038/s41396-020-0682-7"
"   Tan LT, 2020, PLANT PHYSIOL, V183, P1235, DOI 10.1104/pp.19.01569"
"   Tian ZJ, 2022, AGR ECOSYST ENVIRON, V339, DOI 10.1016/j.agee.2022.108090"
"   Valentin C., 2018, Soils as a Key Component of the critical Zone 5: Degradation and Rehabilitation, V5, P39, DOI [10.1002/9781119438298.ch3, DOI 10.1002/9781119438298.CH3]"
"   Wei LL, 2016, CHEMOSPHERE, V165, P100, DOI 10.1016/j.chemosphere.2016.09.027"
"   Xv LL, 2020, ENVIRON POLLUT, V263, DOI 10.1016/j.envpol.2020.114401"
"   Yang L, 2022, ECOTOX ENVIRON SAFE, V234, DOI 10.1016/j.ecoenv.2022.113403"
"   Yang X, 2002, CHINESE SCI BULL, V47, P1634, DOI 10.1360/02tb9359"
"   Yao ZT, 2012, PROCEDIA ENVIRON SCI, V16, P722, DOI 10.1016/j.proenv.2012.10.099"
"   Zeng P, 2022, CHEMOSPHERE, V290, DOI 10.1016/j.chemosphere.2021.133107"
"   Zhang J, 2019, RSC ADV, V9, P20603, DOI 10.1039/c9ra01986c"
"   Zhang M.L., 2011, IEEE, P5936, DOI [10.1109/RSETE.2011.5965706, DOI 10.1109/RSETE.2011.5965706]"
"   Zhang ZC, 2008, PHYTOCHEMISTRY, V69, P911, DOI 10.1016/j.phytochem.2007.10.012"
"   Zhao QH, 2014, SCI TOTAL ENVIRON, V470, P340, DOI 10.1016/j.scitotenv.2013.09.086"
NR 49
TC 22
Z9 23
U1 34
U2 170
PU SCIENCE PRESS
PI BEIJING
"PA 16 DONGHUANGCHENGGEN NORTH ST, BEIJING 100717, PEOPLES R CHINA"
SN 1001-0742
EI 1878-7320

J9 J ENVIRON SCI
JI J. Environ. Sci.
PD JAN
PY 2025
VL 147
BP 179
EP 188
DI 10.1016/j.jes.2023.10.002
EA JAN 2024
PG 10
WC Environmental Sciences
WE Science Citation Index Expanded (SCI-EXPANDED)
SC Environmental Sciences & Ecology
GA IT1L6
UT WOS:001168491500001
PM 39003038
DA 2026-04-06
ER

PT J
"AU Campillo-Cora, C"
"   Soto-G??mez, D"
"   Arias-Est??vez, M"
"   Fern??ndez-Calvi??o, D"
"AF Campillo-Cora, Claudia"
"   Soto-Gomez, Diego"
"   Arias-Estevez, Manuel"
"   Fernandez-Calvino, David"
TI Assessment of Polluted Soil Remediation Using Bacterial Community
   Tolerance to Heavy Metals as an Indicator
SO AGRONOMY-BASEL
LA English
DT Article
DE metal pollution; soil bioremediation; risk assessment; bacterial growth;
   Pollution-Induced Community Tolerance (PICT); bio-sorbents; soil washing
ID IN-SITU REMEDIATION; MICROBIAL BIOMASS; PINE BARK; LEUCINE
   INCORPORATION; ENZYME-ACTIVITIES; CONTAMINATED SOILS; VINEYARD SOILS;
   CU; COPPER; EDTA
"AB The assessment of remediation on metal-polluted soils is usually focused on total and/or bioavailable metal content. However, these chemical variables do not provide direct information about reductions in heavy metals pressure on soil microorganisms. We propose the use of bacterial communities to evaluate the efficiency of three remediation techniques: crushed mussel shell (CMS) and pine bark (PB) as soil amendments and EDTA-washing. A soil sample was polluted with different doses of Cu, Ni, and Zn (separately). After 30 days of incubation, the remediation techniques were applied, and bacterial community tolerance to heavy metals determined. If bacterial communities develop tolerance, it is an indicator that the metal is exerting toxicity on them. Soil bacterial communities developed tolerance to Cu, Ni, and Zn in response to metal additions. After remediation, bacterial communities showed decreases in bacterial community tolerance to Cu, Ni, and Zn for all remediation techniques. For Cu and Ni, soil EDTA-washing showed the greatest reduction of bacterial community tolerance to Cu and Ni, respectively, while for Zn the soil amendment with PB was the most effective remediation technique. Thus, bacterial community tolerance to heavy metals successfully detect differences in the effectiveness of the three remediation techniques."
"C1 [Campillo-Cora, Claudia; Arias-Estevez, Manuel; Fernandez-Calvino, David] Univ Vigo, Fac Ciencias, Dept Biol Vexetal & Ciencia Solo, As Lagoas S-N, Orense 32004, Spain."
"   [Soto-Gomez, Diego] Tech Univ Cartagena, Dept Agr Engn, Paseo Alfonso XIII 48, Cartagena 30203, Spain."
"   [Soto-Gomez, Diego] Lund Univ, Dept Biol, Microbial Ecol MEMEG, Ecol Bldg, SE-22362 Lund, Sweden."
C3 Universidade de Vigo; Universidad Politecnica de Cartagena; Lund
   University
"RP Campillo-Cora, C (corresponding author), Univ Vigo, Fac Ciencias, Dept Biol Vexetal & Ciencia Solo, As Lagoas S-N, Orense 32004, Spain."
EM ccampillo@uvigo.es
"RI Campillo, Claudia/PJB-8256-2026; Arias-Est??vez, Manuel/M-8629-2016; Soto"
"   G??mez, Diego/JFK-1351-2023; Fern??ndez-Calvi??o, David/C-5756-2015"
"OI Campillo-Cora, Claudia/0000-0001-8239-3896; Arias-Est??vez,"
"   Manuel/0000-0002-9162-1587; Soto G??mez, Diego/0000-0001-7439-1356;"
"   Fern??ndez-Calvi??o, David/0000-0003-0864-7039"
FU Spanish Ministry of Economy and Competitiveness [CTM2015-73422-JIN];
"   Spanish Ministry of Economy, Industry and Competitiveness"
   [RYC-2016-20411]; European Union-Next Generation EU; Seneca Foundation
"   [21525/EE/21]; Conselleria de Educacion, Universidade e Formacion"
   Profesional [ED401A-2020/084]
FX This study has been funded by the Spanish Ministry of Economy and
   Competitiveness through the project CTM2015-73422-JIN (FEDER Funds)
   David Fernandez Calvino holds a Ramon y Cajal contract (RYC-2016-20411)
"   financed by the Spanish Ministry of Economy, Industry and"
   Competitiveness. Diego Soto-Gomez has a post-doctoral contract
"   ""Margarita Salas"" funded by European Union-NextGenerationEU, and a"
   Seneca Foundation grant for research stays in international centres
   (21525/EE/21). Claudia Campillo-Cora holds a predoctoral fellowship with
"   Xunta de Galicia (ED401A-2020/084) funded by Conselleria de Educacion,"
   Universidade e Formacion Profesional.
"CR Abd El-Azeem S, 2013, ENVIRON EARTH SCI, V70, P3411, DOI 10.1007/s12665-013-2410-3"
"   Ahmad M, 2012, ENVIRON EARTH SCI, V67, P1023, DOI 10.1007/s12665-012-1550-1"
"   Aliasgharzad N., 2011, Int. J. Environ. Ecol. Eng., V5, P838"
"   B????th E, 2001, SOIL BIOL BIOCHEM, V33, P1571, DOI 10.1016/S0038-0717(01)00073-6"

"   BAATH E, 1994, MICROBIAL ECOL, V27, P267, DOI 10.1007/BF00182410"
"   Barona A, 2001, ENVIRON POLLUT, V113, P79, DOI 10.1016/S0269-7491(00)00158-5"
"   B??rard A, 2016, ENVIRON SCI POLLUT R, V23, P4271, DOI 10.1007/s11356-015-5089-z"
"   Berg J, 2012, APPL ENVIRON MICROB, V78, P7438, DOI 10.1128/AEM.01071-12"
"   Blanck H, 2002, HUM ECOL RISK ASSESS, V8, P1003, DOI 10.1080/1080-700291905792"
"   Bradl HB, 2004, J COLLOID INTERF SCI, V277, P1, DOI 10.1016/j.jcis.2004.04.005"
"   Brandt KK, 2010, SOIL BIOL BIOCHEM, V42, P748, DOI 10.1016/j.soilbio.2010.01.008"
"   Clemente R, 2007, J ENVIRON QUAL, V36, P1137, DOI 10.2134/jeq2006.0304"
"   Conde-Cid M, 2019, CHEMOSPHERE, V234, P978, DOI 10.1016/j.chemosphere.2019.06.121"
"   Cutillas-Barreiro L, 2014, J ENVIRON MANAGE, V144, P258, DOI [10.1016/j.jenvman.2014.06.008, 10.10]"
"   Cutillas-Barreiro L, 2016, ECOTOX ENVIRON SAFE, V131, P118, DOI 10.1016/j.ecoenv.2016.05.007"
"   Day PR., 1965, VIE MICROBIENNE SOL, V9, P545"
"   de Mora AP, 2005, APPL SOIL ECOL, V28, P125, DOI 10.1016/j.apsoil.2004.07.006"
"   Demoling LA, 2008, ENVIRON SCI TECHNOL, V42, P6917, DOI 10.1021/es8004706"
"   D??az-Rav????a M, 2007, J ENVIRON QUAL, V36, P1760, DOI 10.2134/jeq2006.0476"
"   DiazRavina M, 1996, APPL ENVIRON MICROB, V62, P2970, DOI 10.1128/AEM.62.8.2970-2977.1996"
"   DIAZRAVINA M, 1994, APPL ENVIRON MICROB, V60, P2238"
"   Enya O, 2020, SCI TOTAL ENVIRON, V724, DOI 10.1016/j.scitotenv.2020.138223"
"   Fait G, 2006, ENVIRON TOXICOL CHEM, V25, P2000, DOI 10.1897/05-517R.1"
"   Fern??ndez-Calvi??o D, 2015, APPL SOIL ECOL, V85, P65, DOI 10.1016/j.apsoil.2014.09.010"
"   Fern??ndez-Calvi??o D, 2018, APPL SOIL ECOL, V127, P102, DOI 10.1016/j.apsoil.2018.03.010"
"   Fern??ndez-Calvi??o D, 2017, LAND DEGRAD DEV, V28, P762, DOI 10.1002/ldr.2634"
"   Fern??ndez-Calvi??o D, 2017, J ENVIRON MANAGE, V192, P79, DOI 10.1016/j.jenvman.2017.01.042"
"   Fern??ndez-Calvi??o D, 2016, SOIL BIOL BIOCHEM, V96, P20, DOI 10.1016/j.soilbio.2016.01.010"
"   Fern??ndez-Calvi??o D, 2016, LAND DEGRAD DEV, V27, P1276, DOI 10.1002/ldr.2505"
"   Fern??ndez-Calvi??o D, 2011, SOIL BIOL BIOCHEM, V43, P2324, DOI 10.1016/j.soilbio.2011.08.001"
"   Garrido-Rodr??guez B, 2013, INT J ENVIRON SCI TE, V10, P983, DOI 10.1007/s13762-013-0201-8"
"   Guitian F., 1976, Tecnicas de analisis de suelos"
"   Hoogsteen MJJ, 2015, EUR J SOIL SCI, V66, P320, DOI 10.1111/ejss.12224"
"   Kamitani T, 2006, WATER AIR SOIL POLL, V172, P185, DOI 10.1007/s11270-005-9073-y"
"   Kaurin A, 2018, ENVIRON POLLUT, V243, P238, DOI 10.1016/j.envpol.2018.08.079"
"   Kaurin A, 2018, CHEMOSPHERE, V193, P726, DOI 10.1016/j.chemosphere.2017.11.082"
"   Kelly JJ, 2003, BIOL FERT SOILS, V38, P65, DOI 10.1007/s00374-003-0642-1"
"   Kelly JJ, 1998, J ENVIRON QUAL, V27, P609, DOI 10.2134/jeq1998.00472425002700030019x"
"   KOKALISBURELLE N, 1994, PLANT SOIL, V162, P169, DOI 10.1007/BF01347703"
"   Lakanen E., 1971, ACTA AGR SCAND, V17, P131, DOI [10.1080/00015126709433148, DOI 10.1080/00015126709433148]"
"   Lekfeldt JDS, 2014, ENVIRON POLLUT, V194, P78, DOI 10.1016/j.envpol.2014.07.013"
"   Lestan D, 2008, ENVIRON POLLUT, V153, P3, DOI 10.1016/j.envpol.2007.11.015"
"   Li YX, 2015, J HAZARD MATER, V283, P512, DOI 10.1016/j.jhazmat.2014.09.061"
"   Liu HB, 2022, SCI TOTAL ENVIRON, V825, DOI 10.1016/j.scitotenv.2022.154004"
"   Liu SJ, 2018, CHEMOSPHERE, V208, P655, DOI 10.1016/j.chemosphere.2018.06.023"
"   Lock K, 2005, ENVIRON POLLUT, V136, P275, DOI 10.1016/j.envpol.2004.12.038"
"   Macdonald CA, 2010, SOIL BIOL BIOCHEM, V42, P1408, DOI 10.1016/j.soilbio.2010.05.002"
"   Marrugo-Negrete J, 2017, ENVIRON RES, V154, P380, DOI 10.1016/j.envres.2017.01.021"
"   Medynska-Juraszek A., 2013, FUNCTIONS NATURAL OR, P365"
"   Meisner A, 2013, SOIL BIOL BIOCHEM, V66, P188, DOI 10.1016/j.soilbio.2013.07.014"
"   Mirlean N, 2007, ENVIRON POLLUT, V149, P10, DOI 10.1016/j.envpol.2006.12.024"
"   Nagajyoti PC, 2010, ENVIRON CHEM LETT, V8, P199, DOI 10.1007/s10311-010-0297-8"
"   Naghipour D, 2017, J ENVIRON ENG, V143, DOI [10.1061/(ASCE)EE.1943-7870.0001219, 10.1061/(asce)ee.1943-7870.0001219]"
"   Nannipieri P, 2003, EUR J SOIL SCI, V54, P655, DOI 10.1046/j.1351-0754.2003.0556.x"
"   Niklinska M, 2006, APPL SOIL ECOL, V32, P265, DOI 10.1016/j.apsoil.2005.08.002"
"   Paradelo R, 2016, CHEMOSPHERE, V149, P146, DOI 10.1016/j.chemosphere.2016.01.087"
"   Pascual JA, 2000, SOIL BIOL BIOCHEM, V32, P1877, DOI 10.1016/S0038-0717(00)00161-9"
"   Ram??rez-P??rez AM, 2013, J HAZARD MATER, V248, P122, DOI 10.1016/j.jhazmat.2012.12.045"
"   Rasool B, 2022, WATER AIR SOIL POLL, V233, DOI 10.1007/s11270-022-05807-2"
"   Reed S. T., 1996, Methods of soil analysis. Part 3 - chemical methods., P703"
"   Rodr??guez-Seijo A, 2016, LAND DEGRAD DEV, V27, P1721, DOI 10.1002/ldr.2497"
"   Romar-Gasalla A, 2018, J ENVIRON MANAGE, V222, P3, DOI 10.1016/j.jenvman.2018.05.050"
"   Rousk J, 2011, FEMS MICROBIOL ECOL, V78, P17, DOI 10.1111/j.1574-6941.2011.01106.x"
"   Rutgers M, 1998, ENVIRON TOXICOL CHEM, V17, P2210, DOI [10.1897/1551-5028(1998)017<2210:RMFAPI>2.3.CO;2, 10.1002/etc.5620171111]"
"   Santas-Miguel V, 2018, SPAN J SOIL SCI, V8, P1, DOI 10.3232/SJSS.2018.V8.N1.01"
"   Sant??s-Miguel V, 2022, SOIL-GERMANY, V8, P437, DOI 10.5194/soil-8-437-2022"
"   Sant??s-Miguel V, 2021, ENVIRON RES, V193, DOI 10.1016/j.envres.2020.110404"
"   Stefanowicz AM, 2009, EUR J SOIL BIOL, V45, P363, DOI 10.1016/j.ejsobi.2009.05.005"
"   Sun B, 2001, ENVIRON POLLUT, V113, P111, DOI 10.1016/S0269-7491(00)00176-7"
"   Tang JY, 2019, J ENVIRON MANAGE, V242, P121, DOI 10.1016/j.jenvman.2019.04.061"
"   Tauqeer HM, 2022, ENVIRON POLLUT, V313, DOI 10.1016/j.envpol.2022.120064"
"   Tlili A, 2016, FRESHWATER BIOL, V61, P2141, DOI 10.1111/fwb.12558"
"   Turan V, 2020, CHEMOSPHERE, V245, DOI 10.1016/j.chemosphere.2019.125611"
"   Turan V, 2019, ECOTOX ENVIRON SAFE, V183, DOI 10.1016/j.ecoenv.2019.109594"
"   Turan V, 2018, ECOTOX ENVIRON SAFE, V161, P409, DOI 10.1016/j.ecoenv.2018.05.082"
"   Turan V, 2018, ARCH AGRON SOIL SCI, V64, P1053, DOI 10.1080/03650340.2017.1410542"
"   Uchimiya M, 2020, J AGR FOOD CHEM, V68, P12856, DOI 10.1021/acs.jafc.0c00183"
"   Udovic M, 2012, CHEMOSPHERE, V88, P718, DOI 10.1016/j.chemosphere.2012.04.040"
"   Van Beelen P, 2001, ENVIRON TOXICOL CHEM, V20, P1133"
"   Viti C, 2008, WORLD J MICROB BIOT, V24, P309, DOI 10.1007/s11274-007-9472-x"
"   Wang SY, 2022, INT J ENV RES PUB HE, V19, DOI 10.3390/ijerph19063335"
"   Wasay SA, 2001, WATER AIR SOIL POLL, V127, P301, DOI 10.1023/A:1005251915165"
"   Wei H, 2020, SCI TOTAL ENVIRON, V715, DOI 10.1016/j.scitotenv.2020.137032"
"   Zhang CL, 2013, B ENVIRON CONTAM TOX, V90, P736, DOI 10.1007/s00128-013-0987-6"
"   Zhang WH, 2010, J HAZARD MATER, V173, P369, DOI 10.1016/j.jhazmat.2009.08.087"

"   Zhang Y, 2022, ENVIRON SCI POLLUT R, V29, P54641, DOI 10.1007/s11356-022-19734-6"
NR 86
TC 4
Z9 4
U1 3
U2 32
PU MDPI
PI BASEL
"PA MDPI AG, Grosspeteranlage 5, CH-4052 BASEL, SWITZERLAND"
EI 2073-4395
J9 AGRONOMY-BASEL
JI Agronomy-Basel
PD OCT
PY 2022
VL 12
IS 10
AR 2280
DI 10.3390/agronomy12102280
PG 14
WC Agronomy; Plant Sciences
WE Science Citation Index Expanded (SCI-EXPANDED)
SC Agriculture; Plant Sciences
GA 5N9SG
UT WOS:000872125100001
"OA Green Submitted, gold"
DA 2026-04-06
ER

PT J
"AU Fu, SL"
"   Iqbal, B"
"   Li, GL"
"   Alabbosh, KF"
"   Khan, KA"
"   Zhao, X"
"   Raheem, A"
"   Du, DL"
"AF Fu, Shilin"
"   Iqbal, Babar"
"   Li, Guanlin"
"   Alabbosh, Khulood Fahad"
"   Khan, Khalid Ali"
"   Zhao, Xin"
"   Raheem, Abdulkareem"
"   Du, Daolin"
TI The role of microbial partners in heavy metal metabolism in plants: a
   review
SO PLANT CELL REPORTS
LA English
DT Review
DE Stress signaling; Plant-microbe symbiosis; Microbial detoxification;
   Plant defense mechanisms; Genetic mechanisms
ID ARBUSCULAR MYCORRHIZAL FUNGI; TO-SHOOT COMMUNICATION; CADMIUM STRESS;
   MICROPLASTIC CONTAMINATION; BIOFILM FORMATION; MERCURY TOXICITY;
   OXIDATIVE STRESS; LEAD TOXICITY; EXPRESSION; TOLERANCE
"AB Heavy metal pollution threatens plant growth and development as well as ecological stability. Here, we synthesize current research on the interplay between plants and their microbial symbionts under heavy metal stress, highlighting the mechanisms employed by microbes to enhance plant tolerance and resilience. Several key strategies such as bioavailability alteration, chelation, detoxification, induced systemic tolerance, horizontal gene transfer, and methylation and demethylation, are examined, alongside the genetic and molecular basis governing these plant-microbe interactions. However, the complexity of plant-microbe interactions, coupled with our limited understanding of the associated mechanisms, presents challenges in their practical application. Thus, this review underscores the necessity of a more detailed understanding of how plants and microbes interact and the importance of using a combined approach from different scientific fields to maximize the benefits of these microbial processes. By advancing our knowledge of plant-microbe synergies in the metabolism of heavy metals, we can develop more effective bioremediation strategies to combat the contamination of soil by heavy metals."
"C1 [Fu, Shilin; Iqbal, Babar; Li, Guanlin; Raheem, Abdulkareem] Jiangsu Univ, Sch Environm & Safety Engn, Sch Emergency Management, Jiangsu Prov Engn Res Ctr Green Technol & Continge, Zhenjiang 212013, Peoples R China."
"   [Li, Guanlin] Suzhou Univ Sci & Technol, Jiangsu Collaborat Innovat Ctr Technol & Mat Water, Suzhou 215009, Peoples R China."
"   [Alabbosh, Khulood Fahad] Univ Hail, Coll Sci, Dept Biol, Hail 2440, Saudi Arabia."
"   [Khan, Khalid Ali] King Khalid Univ, Appl Coll, Ctr Bee Res & its Prod CBRP, Unit Bee Res & Honey Prod, Abha 61413, Saudi Arabia."
"   [Khan, Khalid Ali] King Khalid Univ, Res Ctr Adv Mat Sci RCAMS, Abha 61413, Saudi Arabia."
"   [Zhao, Xin] Seoul Natl Univ, Coll Engn, Dept Civil & Environm Engn, Seoul 08826, South Korea."
"   [Du, Daolin] Jiangsu Univ, Inst Environm & Ecol, Sch Emergency Management, Sch Environm & Safety Engn,Jingjiang Coll,Sch Agr, Zhenjiang 212013, Peoples R China."
C3 Jiangsu University; Suzhou University of Science & Technology;
   University Ha'il; King Khalid University; King Khalid University; Seoul
   National University (SNU); Jiangsu University
"RP Li, GL; Raheem, A (corresponding author), Jiangsu Univ, Sch Environm & Safety Engn, Sch Emergency Management, Jiangsu Prov Engn Res Ctr Green Technol & Continge, Zhenjiang 212013, Peoples R China.; Li, GL

(corresponding author), Suzhou Univ Sci & Technol, Jiangsu Collaborat Innovat Ctr Technol & Mat Water, Suzhou 215009, Peoples R China.; Du, DL (corresponding author), Jiangsu Univ, Inst Environm & Ecol, Sch Emergency Management, Sch Environm & Safety Engn,Jingjiang Coll,Sch Agr, Zhenjiang 212013, Peoples R China."
EM liguanlin@ujs.edu.cn; abdulkareemraheem@gmail.com; ddl@ujs.edu.cn
"RI Raheem, Abdulkareem/AAM-6141-2020; Alabbosh, Khulood/KPA-0685-2024;"
"   Iqbal, Babar/AAQ-9244-2021; Du, Daolin/HHC-9359-2022; ???,"
"   ???/GSO-3867-2022; Khan, Khalid/AAR-5403-2020"
"OI Raheem, Abdulkareem/0000-0002-9636-7813; Alabbosh,"
   Khulood/0000-0003-2971-7593;
FU Open Project of the Carbon Peak and Carbon Neutrality Technology
   Innovation Foundation of Jiangsu Province [BK20220030]; Open Project of
   the Carbon Peak and Carbon Neutrality Technology Innovation Foundation
"   of Jiangsu Province [32271587, 3235041400]; National Natural Science"
   Foundation of China [18JDG039]; Senior Talent Foundation of Jiangsu
   University [RGP2/360/44]; Deanship of Scientific Research at King Khalid
   University Saudi Arabia
FX This work was supported by the Open Project of the Carbon Peak and
   Carbon Neutrality Technology Innovation Foundation of Jiangsu Province
"   (BK20220030), the National Natural Science Foundation of China"
"   (32271587; 3235041400), and the Senior Talent Foundation of Jiangsu"
   University (18JDG039). The authors extend their appreciation to the
   Deanship of Scientific Research at King Khalid University Saudi Arabia
   for funding this work through Large Groups Project under grant number
   RGP2/360/44.
"CR Abbasi Q, 2023, ECOTOX ENVIRON SAFE, V267, DOI 10.1016/j.ecoenv.2023.115619"
"   Afriyie RZ, 2023, J TRACE ELEM MINER, V4, DOI [10.1016/j.jtemin.2023.100075,
10.1016/j.jtemin.2023.100075]"
"   Agarwal P, 2019, J PLANT PHYSIOL, V240, DOI 10.1016/j.jplph.2019.153010"
"   Ahmad N, 2023, SCI HORTIC-AMSTERDAM, V321, DOI 10.1016/j.scienta.2023.112369"
"   Ahmad P, 2021, J HAZARD MATER, V408, DOI 10.1016/j.jhazmat.2020.124852"
"   Ahmad P, 2020, CHEMOSPHERE, V244, DOI 10.1016/j.chemosphere.2019.125480"
"   Alp-Turgut FN, 2024, ENVIRON POLLUT, V342, DOI 10.1016/j.envpol.2023.123117"
"   Alves LR, 2017, ENVIRON EXP BOT, V134, P102, DOI 10.1016/j.envexpbot.2016.11.008"
"   Ambreetha S, 2018, RHIZOSPHERE-NETH, V5, P57, DOI 10.1016/j.rhisph.2017.12.001"
"   Amna S, 2020, PLANT PHYSIOL BIOCH, V157, P348, DOI 10.1016/j.plaphy.2020.11.002"
"   An TT, 2023, PLANT PHYSIOL BIOCH, V205, DOI 10.1016/j.plaphy.2023.108135"
"   Arif MS, 2019, CHEMOSPHERE, V234, P70, DOI 10.1016/j.chemosphere.2019.06.024"
"   Asif M, 2023, PHYSIOL MOL PLANT P, V127, DOI 10.1016/j.pmpp.2023.102097"
"   Bai B, 2024, ENVIRON TECHNOL INNO, V33, DOI 10.1016/j.eti.2023.103485"
"   Bai B, 2020, INT J HEAT MASS TRAN, V153, DOI 10.1016/j.ijheatmasstransfer.2020.119573"
"   Banerjee A, 2016, PLANT BIOTECHNOL REP, V10, P185, DOI 10.1007/s11816-016-0401-z"
"   Barrios AFG, 2006, J BACTERIOL, V188, P305, DOI 10.1128/JB.188.1.305-316.2006"
"   Ben Y, 2023, ECOL INDIC, V154, DOI 10.1016/j.ecolind.2023.110784"
"   Besson-Bard A, 2008, ANNU REV PLANT BIOL, V59, P21, DOI 10.1146/annurev.arplant.59.032607.092830"
"   Bhatta D, 2023, ECOTOX ENVIRON SAFE, V263, DOI 10.1016/j.ecoenv.2023.115377"
"   Bilal S, 2020, IND CROP PROD, V143, DOI 10.1016/j.indcrop.2019.111931"
"   Bisht A, 2023, J PLANT GROWTH REGUL, V42, P3947, DOI 10.1007/s00344-022-10864-2"
"   Bisht A, 2022, PLANT GROWTH REGUL, V96, P409, DOI 10.1007/s10725-021-00791-9"
"   Boudjabi S, 2023, HELIYON, V9, DOI 10.1016/j.heliyon.2023.e14615"
"   Brooker RW, 2023, J PLANT ECOL, V16, DOI 10.1093/jpe/rtad015"
"   Buzhdygan OY, 2023, J PLANT ECOL, V16, DOI 10.1093/jpe/rtad019"
"   Calatrava-Morales N, 2018, GENES-BASEL, V9, DOI 10.3390/genes9050263"
"   Campos NV, 2015, PLANT PHYSIOL BIOCH, V97, P28, DOI 10.1016/j.plaphy.2015.09.011"
"   Capone R, 2004, PLANT PHYSIOL BIOCH, V42, P425, DOI 10.1016/j.plaphy.2004.03.005"
"   Caracciolo AB, 2021, MICROORGANISMS, V9, DOI 10.3390/microorganisms9071462"
"   Chakraborty J, 2014, ENVIRON SCI POLLUT R, V21, P14188, DOI 10.1007/s11356-014-3308-7"
"   Chang JJ, 2022, J HAZARD MATER, V425, DOI 10.1016/j.jhazmat.2021.127948"
"   Chapotin SM, 2007, TRANSGENIC RES, V16, P675, DOI 10.1007/s11248-007-9122-y"
"   Chen XP, 2023, J PLANT ECOL, V16, DOI 10.1093/jpe/rtac093"
"   Chen Y, 2021, PLANT COMMUN, V2, DOI 10.1016/j.xplc.2021.100234"
"   Cordon G, 2018, CHEMOSPHERE, V204, P398, DOI 10.1016/j.chemosphere.2018.04.048"
"   Costa TR, 2023, J PLANT ECOL, V16, DOI 10.1093/jpe/rtac089"
"   Crupper SS, 1999, J BACTERIOL, V181, P4071, DOI 10.1128/JB.181.13.4071-4075.1999"
"   Cui WT, 2014, ECOTOX ENVIRON SAFE, V105, P103, DOI 10.1016/j.ecoenv.2014.04.009"
"   Czajka K, 2022, GENES GENOM, V44, P279, DOI 10.1007/s13258-021-01191-z"
"   de Oliveira NT, 2023, PLANT PHYSIOL BIOCH, V194, P335, DOI 10.1016/j.plaphy.2022.11.024"
"   Delias DS, 2022, ENVIRON SCI POLLUT R, V29, P22427, DOI 10.1007/s11356-021-17397-3"
"   Ding GH, 2019, ENVIRON SCI POLLUT R, V26, P3428, DOI 10.1007/s11356-018-3864-3"
"   Ejileugha C, 2022, HELIYON, V8, DOI 10.1016/j.heliyon.2022.e09543"
"   Aviles-Garcia ME, 2016, REV ARGENT MICROBIOL, V48, P342, DOI 10.1016/j.ram.2016.07.004"
"   ELMELEIGY MA, 1992, BIOL TRACE ELEM RES, V34, P177, DOI 10.1007/BF02785245"
"   Etesami H, 2023, CURR RES BIOTECHNOL, V6, DOI 10.1016/j.crbiot.2023.100158"
"   Faizan M, 2021, ECOTOX ENVIRON SAFE, V220, DOI 10.1016/j.ecoenv.2021.112401"
"   Fan MC, 2018, MICROBIOL RES, V217, P51, DOI 10.1016/j.micres.2018.09.002"
"   Fang GY, 2024, SCI TOTAL ENVIRON, V912, DOI 10.1016/j.scitotenv.2023.168908"
"   Fedenko VS, 2022, INT J MOL SCI, V23, DOI 10.3390/ijms231911370"
"   Feng SJ, 2021, ENVIRON POLLUT, V288, DOI 10.1016/j.envpol.2021.117837"
"   Feng SJ, 2020, ENVIRON POLLUT, V256, DOI 10.1016/j.envpol.2019.113464"
"   Fesharaki-Esfahani M, 2021, PHYTOCHEMISTRY, V187, DOI 10.1016/j.phytochem.2021.112766"
"   Figueira E, 2021, ENVIRON EXP BOT, V191, DOI 10.1016/j.envexpbot.2021.104619"
"   Figueredo MS, 2023, PLANT SOIL, V486, P375, DOI 10.1007/s11104-023-05876-0"
"   Flores Aurora, 2022, Microbiol Res, V264, P127158, DOI [10.1016/j.micres.2022.127158,
10.1016/j.micres.2022.127158]"
"   Gaddam SR, 2024, J HAZARD MATER, V465, DOI 10.1016/j.jhazmat.2023.133100"

"   Galati S, 2021, ENVIRON MOL MUTAGEN, V62, P133, DOI 10.1002/em.22421"
"   Ge J, 2022, ECOTOX ENVIRON SAFE, V241, DOI 10.1016/j.ecoenv.2022.113795"
"   Ghouri F, 2024, SCI TOTAL ENVIRON, V911, DOI 10.1016/j.scitotenv.2023.168709"
"   Gong J, 2019, CZECH J GENET PLANT, V55, P76, DOI 10.17221/57/2018-CJGPB"
"   Gratao PL, 2015, BIOMETALS, V28, P803, DOI 10.1007/s10534-015-9867-3"
"   Grosse C., 2023, J Bacteriol, V4, P1"
"   Grosskinsky DK, 2018, J EXP BOT, V69, P825, DOI 10.1093/jxb/erx333"
"   Gu YL, 2015, ENVIRON SCI POLLUT R, V22, P6271, DOI 10.1007/s11356-014-3856-x"
"   Guan J, 2023, PLANT PHYSIOL BIOCH, V203, DOI 10.1016/j.plaphy.2023.108043"
"   Hak K, 2020, ECOTOX ENVIRON SAFE, V189, DOI 10.1016/j.ecoenv.2019.110049"
"   Han H, 2021, CHEMOSPHERE, V276, DOI 10.1016/j.chemosphere.2021.130157"
"   Han YS, 2014, ENVIRON EXP BOT, V100, P74, DOI 10.1016/j.envexpbot.2013.12.021"
"   Hao SF, 2022, J HAZARD MATER, V424, DOI 10.1016/j.jhazmat.2021.127720"
"   Hareem M, 2023, J KING SAUD UNIV SCI, V35, DOI 10.1016/j.jksus.2023.102765"
"   Hartmann A, 2021, FRONT PLANT SCI, V12, DOI 10.3389/fpls.2021.683546"
"   Hashem A, 2016, SAUDI J BIOL SCI, V23, P272, DOI 10.1016/j.sjbs.2015.11.002"
"   He CM, 2021, MOL PLANT, V14, P488, DOI 10.1016/j.molp.2020.12.015"
"   He MY, 2023, SPECTROCHIM ACTA B, V209, DOI 10.1016/j.sab.2023.106781"
"   Hobman JL, 2012, BMC MICROBIOL, V12, DOI 10.1186/1471-2180-12-109"
"   Huang HL, 2019, FRONT PLANT SCI, V10, DOI 10.3389/fpls.2019.00800"
"   Huang SH, 2014, PLANT PHYSIOL BIOCH, V85, P9, DOI 10.1016/j.plaphy.2014.10.006"
"   Iqbal B, 2024, INT J PHYTOREMEDIAT, V26, P1269, DOI 10.1080/15226514.2024.2310001"
"   Iqbal B, 2023, PLANT STRESS, V10, DOI 10.1016/j.stress.2023.100283"
"   Iqbal B, 2024, SCI TOTAL ENVIRON, V906, DOI 10.1016/j.scitotenv.2023.167756"
"   Iqbal B, 2023, S AFR J BOT, V160, P369, DOI 10.1016/j.sajb.2023.07.025"
"   Islam F, 2014, ECOTOX ENVIRON SAFE, V110, P143, DOI 10.1016/j.ecoenv.2014.08.020"
"   Jain M, 2004, J PLANT PHYSIOL, V161, P251, DOI 10.1078/0176-1617-00879"
"   Jalil Sanaullah, 2023, Chemosphere, V338, P139566, DOI 10.1016/j.chemosphere.2023.139566"
"   Jing MY, 2022, FRONT PLANT SCI, V13, DOI 10.3389/fpls.2022.845108"
"   Karmakar R, 2019, SCI TOTAL ENVIRON, V650, P858, DOI 10.1016/j.scitotenv.2018.09.078"
"   Khalid MF, 2023, ENVIRON TECHNOL INNO, V32, DOI 10.1016/j.eti.2023.103283"
"   Khan MIR, 2021, ECOTOX ENVIRON SAFE, V222, DOI 10.1016/j.ecoenv.2021.112535"
"   Khan Z, 2022, J SAUDI CHEM SOC, V26, DOI 10.1016/j.jscs.2022.101569"
"   Kirolinko C, 2021, FRONT PLANT SCI, V12, DOI 10.3389/fpls.2021.659061"
"   Kumar RS, 2023, J HAZARD MATER, V452, DOI 10.1016/j.jhazmat.2023.131359"
"   Kumar S, 2022, ENVIRON EXP BOT, V199, DOI 10.1016/j.envexpbot.2022.104871"
"   Li A, 2024, ECOTOX ENVIRON SAFE, V269, DOI 10.1016/j.ecoenv.2023.115789"
"   Li CY, 2021, ENVIRON EXP BOT, V191, DOI 10.1016/j.envexpbot.2021.104602"
"   Li JL, 2018, CHEMOSPHERE, V209, P392, DOI 10.1016/j.chemosphere.2018.06.092"
"   Li MJ, 2017, PLANT CELL REP, V36, P1585, DOI 10.1007/s00299-017-2176-2"
"   Li Q, 2020, CHEMOSPHERE, V249, DOI 10.1016/j.chemosphere.2020.126143"
"   Li XR, 2022, NAT COMMUN, V13, DOI 10.1038/s41467-022-33908-3"
"   Li ZQ, 2023, ENVIRON POLLUT, V326, DOI 10.1016/j.envpol.2023.121488"
"   Liu JP, 2021, J HAZARD MATER, V403, DOI 10.1016/j.jhazmat.2020.123638"
"   Liu YY, 2023, PLANT PHYSIOL BIOCH, V202, DOI 10.1016/j.plaphy.2023.107989"
"   Luo DJ, 2023, PLANT SCI, V331, DOI 10.1016/j.plantsci.2023.111663"
"   Luo YF, 2023, CHEMOSPHERE, V313, DOI 10.1016/j.chemosphere.2022.137556"
"   Lv FL, 2023, PLANT CELL REP, V42, P1777, DOI 10.1007/s00299-023-03065-y"
"   Ma XC, 2023, ECOTOX ENVIRON SAFE, V261, DOI 10.1016/j.ecoenv.2023.115110"
"   Maity S, 2023, APPL BIOCHEM BIOTECH, V195, P4832, DOI 10.1007/s12010-022-04288-7"
"   Mart??nez M, 2006, CHEMOSPHERE, V64, P478, DOI 10.1016/j.chemosphere.2005.10.044"
"   Meng M, 2021, ECOTOX ENVIRON SAFE, V215, DOI 10.1016/j.ecoenv.2021.112106"
"   Mishra D, 2021, INT J BIOL MACROMOL, V183, P513, DOI 10.1016/j.ijbiomac.2021.04.137"
"   Mohammed AE, 2023, PLANT PHYSIOL BIOCH, V205, DOI 10.1016/j.plaphy.2023.108148"
"   Mu??oz-Garc??a A, 2022, MAR POLLUT BULL, V184, DOI 10.1016/j.marpolbul.2022.114204"
"   Mwelwa S, 2023, DATA BRIEF, V49, DOI 10.1016/j.dib.2023.109434"
"   Naik MM, 2012, ECOTOX ENVIRON SAFE, V79, P129, DOI 10.1016/j.ecoenv.2011.12.015"
"   Niu DD, 2011, MOL PLANT MICROBE IN, V24, P533, DOI 10.1094/MPMI-09-10-0213"
"   Onyango DA, 2020, FUNCT PLANT BIOL, V47, P145, DOI 10.1071/FP19054"
"   Palacios OA, 2016, FEMS MICROBIOL ECOL, V92, DOI 10.1093/femsec/fiw077"
"   Pang B, 2024, PLANT PHYSIOL BIOCH, V206, DOI 10.1016/j.plaphy.2023.108299"
"   Pavl??k M, 2010, ECOTOX ENVIRON SAFE, V73, P1309, DOI 10.1016/j.ecoenv.2010.07.008"
"   Pelcov?? P, 2022, CHEMOSPHERE, V288, DOI 10.1016/j.chemosphere.2021.132545"
"   Peng RH, 2014, ENVIRON SCI TECHNOL, V48, P12824, DOI 10.1021/es5015357"
"   Pirzadah TB, 2020, INT BIODETER BIODEGR, V151, DOI 10.1016/j.ibiod.2020.104992"
"   Praveen A, 2020, PEDOSPHERE, V30, P517, DOI 10.1016/S1002-0160(17)60435-1"
"   Qu LL, 2024, J HAZARD MATER, V461, DOI 10.1016/j.jhazmat.2023.132641"
"   Rai R, 2021, PLANT PHYSIOL BIOCH, V168, P353, DOI 10.1016/j.plaphy.2021.09.037"
"   Safari F, 2019, ECOTOX ENVIRON SAFE, V183, DOI 10.1016/j.ecoenv.2019.109542"
"   Sarma H, 2021, J HAZARD MATER, V414, DOI 10.1016/j.jhazmat.2021.125493"
"   Schikora A, 2016, PLANT MOL BIOL, V90, P605, DOI 10.1007/s11103-016-0457-8"
"   Serrato-Gami??o N, 2018, WORLD J MICROB BIOT, V34, DOI 10.1007/s11274-018-2526-4"
"   Shabala S, 2016, FUNCT PLANT BIOL, V43, P87, DOI 10.1071/FP15252"
"   Shabani L, 2016, MYCORRHIZA, V26, P67, DOI 10.1007/s00572-015-0647-2"
"   Shah TR, 2024, J ENVIRON CHEM ENG, V12, DOI 10.1016/j.jece.2023.111619"
"   Shah T, 2023, PLANT PHYSIOL BIOCH, V202, DOI 10.1016/j.plaphy.2023.107916"
"   Shah T, 2020, CHEMOSPHERE, V246, DOI 10.1016/j.chemosphere.2019.125766"
"   Shahid M, 2014, J GEOCHEM EXPLOR, V144, P290, DOI 10.1016/j.gexplo.2014.01.003"
"   Sharma P, 2022, FRONT MICROBIOL, V13, DOI 10.3389/fmicb.2022.802512"
"   Shi WG, 2019, J HAZARD MATER, V362, P275, DOI 10.1016/j.jhazmat.2018.09.024"
"   Shi WG, 2019, PLANT CELL ENVIRON, V42, P1087, DOI 10.1111/pce.13471"
"   Shukla D, 2021, ENVIRON EXP BOT, V188, DOI 10.1016/j.envexpbot.2021.104483"
"   Siddiqui MH, 2020, J HAZARD MATER, V398, DOI 10.1016/j.jhazmat.2020.122882"
"   Sieburth LE, 2010, J INTEGR PLANT BIOL, V52, P77, DOI 10.1111/j.1744-7909.2010.00902.x"

"   Silva-Gigante M., 2023, Chemosphere, V335, P139010, DOI [10.1016/j.chemosphere.2023.139010, 10.1016/j.chemosphere.2023.139010]"
"   Singh A, 2023, PLANT CELL REP, V42, P961, DOI 10.1007/s00299-023-03013-w"
"   Singh Prince Kumar, 2022, Toxicol Rep, V9, P1906, DOI [10.1016/j.toxrep.2022.10.010, 10.1016/j.toxrep.2022.10.010]"
"   Singh P, 2021, PLANT CELL REP, V40, P1617, DOI 10.1007/s00299-021-02736-y"
"   Sobral-Souza CE, 2019, ECOTOX ENVIRON SAFE, V170, P461, DOI 10.1016/j.ecoenv.2018.12.007"
"   Song JY, 2019, PLANT SCI, V287, DOI 10.1016/j.plantsci.2019.110206"
"   Spaepen S., 2015, Principles of plant-microbe interactions, P247, DOI [10.1007/978-, DOI 10.1007/978, 10.1007/978-3-319-08575-3_26]"
"   Stuwe E, 2014, GENE DEV, V28, P423, DOI 10.1101/gad.236414.113"
"   Su SM, 2017, ENVIRON POLLUT, V225, P620, DOI 10.1016/j.envpol.2017.03.030"
"   Su ZG, 2023, ENVIRON INT, V171, DOI 10.1016/j.envint.2022.107714"
"   Subhanullah M, 2024, SCI REP-UK, V14, DOI 10.1038/s41598-024-53340-5"
"   Sun YP, 2023, ECOTOX ENVIRON SAFE, V263, DOI 10.1016/j.ecoenv.2023.115214"
"   Swift S, 2001, ADV MICROB PHYSIOL, V45, P199, DOI 10.1016/S0065-2911(01)45005-3"
"   Tagumira Abudallah, 2022, Toxicol Rep, V9, P2042, DOI [10.1016/j.toxrep.2022.11.007, 10.1016/j.toxrep.2022.11.007]"
"   Tang L, 2017, SCI REP-UK, V7, DOI 10.1038/s41598-017-14832-9"
"   Tang Y, 2023, ECOTOX ENVIRON SAFE, V267, DOI 10.1016/j.ecoenv.2023.115640"
"   Tariq M, 2024, PLANT CELL REP, V43, DOI 10.1007/s00299-024-03162-6"
"   Tatung M, 2024, S AFR J BOT, V165, P217, DOI 10.1016/j.sajb.2023.12.039"
"   Terzi H, 2021, ECOTOX ENVIRON SAFE, V209, DOI 10.1016/j.ecoenv.2020.111784"
"   Teshita A, 2024, PLANT STRESS, V11, DOI 10.1016/j.stress.2023.100345"
"   Tewari RK, 2021, J SOIL SCI PLANT NUT, V21, P832, DOI 10.1007/s42729-020-00405-9"
"   Thaden JT, 2010, J BACTERIOL, V192, P2557, DOI 10.1128/JB.01528-09"
"   Thornhill SG, 2017, MICROBIOL-SGM, V163, P1429, DOI 10.1099/mic.0.000531"
"   Tiecher TL, 2016, ECOTOX ENVIRON SAFE, V129, P109, DOI 10.1016/j.ecoenv.2016.03.016"
"   Tu C, 2020, ENVIRON INT, V137, DOI 10.1016/j.envint.2020.105576"
"   Turco F, 2022, ACS SYNTH BIOL, V11, P3617, DOI 10.1021/acssynbio.2c00130"
"   Ummara U, 2022, CHEMOSPHERE, V290, DOI 10.1016/j.chemosphere.2021.133327"
"   Vega LM, 2014, INT BIODETER BIODEGR, V91, P82, DOI 10.1016/j.ibiod.2014.03.013"
"   Verma P, 2021, PLANT CELL REP, V40, P2111, DOI 10.1007/s00299-021-02772-8"
"   Wan JSH, 2017, EVOL BIOL, V44, P411, DOI 10.1007/s11692-017-9419-6"
"   Wang W, 2016, PLANT GENE, V6, P18, DOI [10.1016/j.plgene.2016.02.002, 10.1016/j.plgene.2016.02.002, DOI 10.1016/J.PLGENE.2016.02.002]"
"   Wang XY, 2022, SCI TOTAL ENVIRON, V819, DOI 10.1016/j.scitotenv.2022.153242"
"   Xiang MT, 2022, ECOL INDIC, V143, DOI 10.1016/j.ecolind.2022.109412"
"   Xiang Y, 2024, ECOTOX ENVIRON SAFE, V270, DOI 10.1016/j.ecoenv.2023.115859"
"   Xie QL, 2022, PLANT CELL REP, V41, P1631, DOI 10.1007/s00299-022-02876-9"
"   Xing QF, 2023, J HAZARD MATER, V456, DOI 10.1016/j.jhazmat.2023.131670"
"   Xu XJ, 2015, FRONT MICROBIOL, V6, DOI 10.3389/fmicb.2015.01422"
"   Xu ZG, 2019, ECOTOX ENVIRON SAFE, V171, P301, DOI 10.1016/j.ecoenv.2018.12.084"
"   Yadav S, 2023, ENVIRON RES, V226, DOI 10.1016/j.envres.2023.115618"
"   Yang YX, 2022, ENVIRON POLLUT, V307, DOI 10.1016/j.envpol.2022.119561"
"   Yi SW, 2022, J HAZARD MATER, V424, DOI 10.1016/j.jhazmat.2021.127514"
"   Yoo Sung-Je, 2017, [Research in Plant Disease, ????????? ??????], V23, P99, DOI 10.5423/rpd.2017.23.2.99"
"   Yu XZ, 2019, CHEMOSPHERE, V220, P300, DOI 10.1016/j.chemosphere.2018.12.119"
"   Yuan M, 2019, BMC GENOMICS, V20, DOI 10.1186/s12864-019-5513-8"
"   Zare L, 2023, ENVIRON SCI POLLUT R, V30, P93602, DOI 10.1007/s11356-023-28969-w"
"   Zhang LD, 2023, J HAZARD MATER, V459, DOI 10.1016/j.jhazmat.2023.132321"
"   Zhang X., 2024, Carbohydr Polym, V246"
"   Zhao JQ, 2022, ENVIRON POLLUT, V315, DOI 10.1016/j.envpol.2022.120410"
"   Zhao Q, 2018, PLANT CELL REP, V37, P741, DOI 10.1007/s00299-018-2264-y"
"   Zhi JK, 2020, PLANT CELL TISS ORG, V143, P211, DOI 10.1007/s11240-020-01914-2"
"   Zhou ZS, 2008, CHEMOSPHERE, V70, P1500, DOI 10.1016/j.chemosphere.2007.08.028"
"   Zhu NL, 2020, ACS APPL MATER INTER, V12, P22948, DOI 10.1021/acsami.0c04512"
"   Zlobin IE, 2017, PLANT PHYSIOL BIOCH, V118, P333, DOI 10.1016/j.plaphy.2017.06.029"
"   Zuo D, 2023, PLANT PHYSIOL BIOCH, V201, DOI 10.1016/j.plaphy.2023.107900"
NR 196
TC 20
Z9 24
U1 19
U2 111
PU SPRINGER
PI NEW YORK
"PA ONE NEW YORK PLAZA, SUITE 4600, NEW YORK, NY, UNITED STATES"
SN 0721-7714
EI 1432-203X
J9 PLANT CELL REP
JI Plant Cell Reports
PD APR
PY 2024
VL 43
IS 4
AR 111
DI 10.1007/s00299-024-03194-y
PG 20
WC Plant Sciences
WE Science Citation Index Expanded (SCI-EXPANDED)
SC Plant Sciences
GA MX7V0
UT WOS:001197009700002
PM 38568247

DA 2026-04-06
ER

PT J
"AU Li, WJ"
"   Chen, XW"
"   Zhang, YA"
"   Ling, WT"
"   Qin, C"
"   Gao, YZ"
"AF Li, Wenjie"
"   Chen, Xuwen"
"   Zhang, Youai"
"   Ling, Wanting"
"   Qin, Chao"
"   Gao, Yanzheng"
TI Synergistic remediation of phthalate and Cd co-contaminated soil with an
   immobilized bacterial agent
SO CHEMICAL ENGINEERING JOURNAL
LA English
DT Article
DE Microbial agent; PAEs; Cd; Bacterial community; Agricultural soil
ID ADSORPTION BEHAVIOR; HEAVY-METALS; DEGRADATION; MONTMORILLONITE;
   VEGETABLES; EXUDATION; RESIDUES; REMOVAL; ESTERS; CLAY
"AB Developing novel immobilized microbial agents for the synergistic remediation of Phthalate (PAE)-Cd contaminated soil is one of the main focuses of environmental governance. This work optimized the preparation of an immobilized bacterial agent (BSMA) based on biochar-sulfhydryl modified montmorillonite composites and Cdresistant bacteria with PAEs efficient degradation for the co-contaminated soil in karst areas. The results showed that BSMA had a significant advantage over biochar immobilized bacterial agent (BMA) in the PAEs-Cd synergistic remediation. It can effectively reduce the content of PAEs in the soil-plant system, and promote the conversion of soil exchangeable Cd to bound and residual Cd, reducing the bioavailability of heavy metals. Despite divergent responses of soil bacterial community structures to BSMA, BSMA consistently strengthened the connectivity of key genera (Sphingomonas and Bacillus) related to synergetic remediation in the microbial network. This regulated the ecological function of the bacterial community and enhanced the soil resistance to pollutant stress. Clay and UR are the key env-factors for the BSMA synergistic remediation. These findings collectively indicate that BSMA enhances soil stress resistance by improving soil texture and regulating the niche of key genera, thereby achieving PAE-Cd synergistic remediation. This study provides novel ideas with which immobilized microbial agents can be further improved and designed to accomplish the highly important goal of soil synergistic remediation."
"C1 [Li, Wenjie; Chen, Xuwen; Zhang, Youai; Ling, Wanting; Qin, Chao; Gao, Yanzheng] Nanjing Agr Univ, Inst Organ Contaminant Control & Soil Remediat, Coll Resources & Environm Sci, Nanjing 210095, Peoples R China."
C3 Nanjing Agricultural University
"RP Gao, YZ (corresponding author), Nanjing Agr Univ, Inst Organ Contaminant Control & Soil Remediat, Coll Resources & Environm Sci, Nanjing 210095, Peoples R China."
EM gaoyanzheng@njau.edu.cn
"RI Gao, Yanzheng/G-6391-2010; CHEN, XUWEN/E-1975-2016"
"FU National Natural Science Foundation of China [U22A20590, 42430703,"
"   42261144738, 42177016]; Jiangsu Agricultural Science and Technology"
   Innovation Fund [CX (24) 1002]; Fundamental Research Funds for the
   Central Universities [QTPY2025004]
FX This work was financially supported by the National Natural Science
"   Foundation of China (U22A20590, 42430703, 42261144738, 42177016) ,"
   Jiangsu Agricultural Science and Technology Innovation Fund (CX (24)
"   1002) , and Fundamental Research Funds for the Central Universities"
   (QTPY2025004) .
"CR Almihyawi RAH, 2024, SCI REP-UK, V14, DOI 10.1038/s41598-024-61619-w"
"   Alothman ZA, 2020, SEP SCI TECHNOL, V55, P1766, DOI 10.1080/01496395.2019.1608242"
"   Alsohaimi IH, 2015, CHEM ENG J, V270, P9, DOI 10.1016/j.cej.2015.02.008"
"   Bretti C, 2012, J CHEM ENG DATA, V57, P2838, DOI 10.1021/je300755y"
"   Burns RG, 2013, SOIL BIOL BIOCHEM, V58, P216, DOI 10.1016/j.soilbio.2012.11.009"
"   Cai QY, 2008, SCI TOTAL ENVIRON, V389, P209, DOI 10.1016/j.scitotenv.2007.08.026"
"   Chaloner TM, 2020, NAT COMMUN, V11, DOI 10.1038/s41467-020-16778-5"
"   Cottom JW, 2024, NATURE, V633, P101, DOI 10.1038/s41586-024-07758-6"
"   Ewis D, 2022, APPL CLAY SCI, V229, DOI 10.1016/j.clay.2022.106686"
"   Fan SH, 2023, ENVIRON TECHNOL INNO, V32, DOI 10.1016/j.eti.2023.103269"
"   Fu XW, 2011, J AGR FOOD CHEM, V59, P11585, DOI 10.1021/jf203502e"
"   Fuchs G, 2011, NAT REV MICROBIOL, V9, P803, DOI 10.1038/nrmicro2652"
"   Ganesamurthi J, 2022, COLLOID SURFACE A, V647, DOI 10.1016/j.colsurfa.2022.129077"
"   Gao YZ, 2015, SOIL TILL RES, V145, P103, DOI 10.1016/j.still.2014.09.008"
"   Gao YZ, 2013, SOIL SCI SOC AM J, V77, P1572, DOI 10.2136/sssaj2013.04.0128"
"   Ghaffar A, 2015, ENVIRON POLLUT, V206, P502, DOI 10.1016/j.envpol.2015.08.001"
"   Hernandez DJ, 2021, ISME J, V15, P1722, DOI 10.1038/s41396-020-00882-x"
"   Hou DY, 2020, NAT REV EARTH ENV, V1, P366, DOI 10.1038/s43017-020-0061-y"
"   Javed MT, 2017, ECOTOX ENVIRON SAFE, V141, P216, DOI 10.1016/j.ecoenv.2017.03.027"
"   Kenawy ER, 2018, J ENVIRON MANAGE, V219, P285, DOI 10.1016/j.jenvman.2018.04.121"
"   Krulwich TA, 2011, NAT REV MICROBIOL, V9, P330, DOI 10.1038/nrmicro2549"
"   Lehmann J, 2011, SOIL BIOL BIOCHEM, V43, P1812, DOI 10.1016/j.soilbio.2011.04.022"
"   Li JT, 2025, J HAZARD MATER, V485, DOI 10.1016/j.jhazmat.2024.136941"
"   Li WJ, 2025, J HAZARD MATER, V494, DOI 10.1016/j.jhazmat.2025.138526"
"   Li XZ, 2024, J ENVIRON CHEM ENG, V12, DOI 10.1016/j.jece.2024.113170"
"   Li Y, 2025, NATURE, V641, DOI 10.1038/s41586-025-08831-4"
"   Li Y, 2020, CHEMOSPHERE, V249, DOI 10.1016/j.chemosphere.2020.126457"
"   Liang GW, 2019, SCI TOTAL ENVIRON, V695, DOI 10.1016/j.scitotenv.2019.133800"
"   Lin MX, 2023, ENVIRON CHEM LETT, V21, P1837, DOI 10.1007/s10311-023-01582-6"

"   Liu K, 2020, J HAZARD MATER, V395, DOI 10.1016/j.jhazmat.2020.122623"
"   Liu YN, 2020, P NATL ACAD SCI USA, V117, P27148, DOI 10.1073/pnas.2010136117"
"   Lu J, 2020, SCI TOTAL ENVIRON, V708, DOI 10.1016/j.scitotenv.2019.134773"
"   Maiti BK, 2025, COORDIN CHEM REV, V529, DOI 10.1016/j.ccr.2025.216442"
"   Maszenan AM, 2022, WATER RES, V221, DOI 10.1016/j.watres.2022.118729"
"   Mehrotra T, 2021, J ENVIRON CHEM ENG, V9, DOI 10.1016/j.jece.2021.105920"
"   Mittal A, 2016, DESALIN WATER TREAT, V57, P21863, DOI 10.1080/19443994.2015.1125805"
"   Mo CH, 2009, ARCH ENVIRON CON TOX, V56, P181, DOI 10.1007/s00244-008-9177-7"
"   Muthukutty B, 2021, ELECTROCHIM ACTA, V386, DOI 10.1016/j.electacta.2021.138482"
"   Nahurira R, 2017, CURR MICROBIOL, V74, P309, DOI 10.1007/s00284-016-1159-9"
"   Nazari MT, 2022, J ENVIRON MANAGE, V323, DOI 10.1016/j.jenvman.2022.116220"
"   Net S, 2015, ENVIRON SCI TECHNOL, V49, P4019, DOI 10.1021/es505233b"
"   Palansooriya KN, 2022, ENVIRON SCI TECHNOL, V56, P4187, DOI 10.1021/acs.est.1c08302"
"   Ruan ZP, 2024, NAT COMMUN, V15, DOI 10.1038/s41467-024-49098-z"
"   Sinsabaugh RL, 2009, NATURE, V462, P795, DOI 10.1038/nature08632"
"   Su XX, 2023, J COLLOID INTERF SCI, V646, P129, DOI 10.1016/j.jcis.2023.05.041"
"   Sun JQ, 2015, ENVIRON SCI TECHNOL, V49, P8471, DOI 10.1021/acs.est.5b01233"
"   Sun SW, 2025, CHEM ENG J, V508, DOI 10.1016/j.cej.2025.160640"
"   Sun T, 2024, J HAZARD MATER, V468, DOI 10.1016/j.jhazmat.2024.133845"
"   Torres NI, 2018, ENVIRON POLLUT, V237, P298, DOI 10.1016/j.envpol.2018.01.106"
"   Tu C, 2020, ENVIRON INT, V137, DOI 10.1016/j.envint.2020.105576"
"   Uddin MK, 2017, CHEM ENG J, V308, P438, DOI 10.1016/j.cej.2016.09.029"
"   Wan PX, 2023, CATENA, V223, DOI 10.1016/j.catena.2023.106913"
"   Wang K., 2023, Chem. Eng. J., V462"
"   Wang TJ, 2020, J AGR FOOD CHEM, V68, P14907, DOI 10.1021/acs.jafc.0c04752"
"   Wei N, 2025, J HAZARD MATER, V486, DOI 10.1016/j.jhazmat.2025.137100"
"   Xia Y, 2016, BIOTECHNOL BIOFUELS, V9, DOI 10.1186/s13068-016-0524-z"
"   Zeng FR, 2008, ENVIRON POLLUT, V155, P284, DOI 10.1016/j.envpol.2007.11.019"
"   Zhang HX, 2024, J HAZARD MATER, V461, DOI 10.1016/j.jhazmat.2023.132619"
"   Zhang Y, 2023, BIOCHAR, V5, DOI 10.1007/s42773-023-00241-x"
"   Zhao GZ, 2009, J HAZARD MATER, V169, P402, DOI 10.1016/j.jhazmat.2009.03.110"
"   Zhao GQ, 2025, ENVIRON POLLUT, V367, DOI 10.1016/j.envpol.2024.125548"
"   Zhao HM, 2018, SCI TOTAL ENVIRON, V640, P646, DOI 10.1016/j.scitotenv.2018.05.337"
"   Zuo XZ, 2024, ENVIRON POLLUT, V363, DOI 10.1016/j.envpol.2024.125079"
"   Zuo XZ, 2024, ENVIRON POLLUT, V361, DOI 10.1016/j.envpol.2024.124925"
NR 64
TC 8
Z9 8
U1 59
U2 74
PU ELSEVIER SCIENCE SA
PI LAUSANNE
"PA PO BOX 564, 1001 LAUSANNE, SWITZERLAND"
SN 1385-8947
EI 1873-3212
J9 CHEM ENG J
JI Chem. Eng. J.
PD SEP 15
PY 2025
VL 520
AR 165972
DI 10.1016/j.cej.2025.165972
EA JUL 2025
PG 9
"WC Engineering, Environmental; Engineering, Chemical"
WE Science Citation Index Expanded (SCI-EXPANDED)
SC Engineering
GA 5NI7D
UT WOS:001539775600001
DA 2026-04-06
ER

PT J
"AU Majzlik, P"
"   Strasky, A"
"   N??mec, M"
"   Trnkova, L"
"   Havel, L"
"   Zehnalek, J"
"   Babula, P"
"   Kizek, R"
"AF Majzlik, Petr"
"   Strasky, Andrej"
"   Nemec, Miroslav"
"   Trnkova, Libuse"
"   Havel, Ladislav"
"   Zehnalek, Josef"
"   Babula, Petr"
"   Kizek, Rene"
TI Remediation potential of bacteria for removing heavy metals from
   environment
SO LISTY CUKROVARNICKE A REPARSKE
LA Czech
DT Editorial Material

DE heavy metals; differential pulse voltammetry; bioremediation;
   Streptomyces
"AB Heavy metals are toxic and can be harmful for organisms For this reason a number of organisms including bacteria develop processes which are able to withstand the effects of these pollutants An accumulation of heavy metals in different bacterial strains in soil has been observed and could be used in the process of remediation of soils contaminated by heavy metal-pollutants In this study Streptomyces, well known for their capacity to accumulate he ivy metals from the environment were selected The ability to accumulate he ivy metals in biomass was determined for four strains studied"
"C1 [Majzlik, Petr; Havel, Ladislav; Zehnalek, Josef; Kizek, Rene] Mendelova Univ Brne, Brno, Czech Republic."
"   [Strasky, Andrej; Nemec, Miroslav; Trnkova, Libuse] Masaryk Univ, CS-60177 Brno, Czech Republic."
"   [Babula, Petr] Vet & Farmaceut Univ Brne, Brno, Czech Republic."
C3 Mendel University in Brno; Masaryk University
"RP Majzlik, P (corresponding author), Mendelova Univ, Ustav Chem & Biochem, Zemedelska 1, Brno 61300, Czech Republic."
"RI Babula, Petr/AAL-6510-2020; Havel, Ladislav/C-8954-2012; Kizek,"
"   Rene/D-7748-2012; Zehnalek, Josef/F-5551-2012"
"OI Havel, Ladislav/0000-0003-3378-2785; "
NR 0
TC 3
Z9 3
U1 0
U2 8
PU LISTY CUKROVARNICKE REPARSKE
PI PRAGUE
"PA V U C PRAHA, A.S., U JEDNOTY 7, PRAGUE 142 00, CZECH REPUBLIC"
SN 1210-3306
J9 LISTY CUKROV REPAR
JI Lis. Cukrov. Repar.
PD NOV
PY 2010
VL 126
IS 11
BP 417
EP 417
PG 1
WC Food Science & Technology
WE Science Citation Index Expanded (SCI-EXPANDED)
SC Food Science & Technology
GA 686VV
UT WOS:000284725700031
DA 2026-04-06
ER

PT J
"AU He, N"
"   Xie, YJ"
"AF He, Ning"
"   Xie, Yajun"
TI Responses of Soil Microbial Diversity and Composition To Heavy Metals
   Contamination in the Largest Lithium Mine in China
SO JOURNAL OF SOIL SCIENCE AND PLANT NUTRITION
LA English
DT Article
DE Microbiome; Multiple contamination; Nemerow pollution index;
   Generalists; Microbial interactions
ID SPHINGOMONAS SP; LAND-USE; RISK; SEQUENCES
"AB PurposeThe exponential growth of the market for lithium-ion batteries has resulted in expansion of mining activities for lithium on a global scale. However, the detailed consequences of exposure to heavy metals introduced by lithium mining on ecosystem processes are still not well understood.MethodsWe investigated the diversity and composition of bacteria and their potential interactions in response to a multi-metal contamination gradient in area potentially affected by the largest lithium mine in China.ResultsThe pollution level of Cd, indicated by Nemerow pollution index, is two orders of magnitude larger than that of other heavy metals. Contamination with multiple heavy metals decreased the microbial diversity and altered community structure. As the potential microbial bio-remediators of multi-metal contamination in soils, habitat generalists (genera which were found in all soil samples) were more resistant to contamination than specialists (genera which were found in some soil samples).ConclusionsOur results provide information for potential microbial bio-remediators of multi-metal contamination in soils. Future studies are needed to explore the potential application of resistant in remediation of contaminated lands affected by lithium-mining activities."
"C1 [He, Ning] Yichun Univ, Coll Life Sci & Resources & Environm, Yichun 336000, Peoples R China."
"   [Xie, Yajun] Hunan Univ Technol, Coll Urban & Environm Sci, Zhuzhou 412007, Peoples R China."
C3 Yichun University; Hunan University of Technology
"RP Xie, YJ (corresponding author), Hunan Univ Technol, Coll Urban & Environm Sci, Zhuzhou 412007, Peoples R China."
EM xieyajun@hut.edu.cn
FU Foundation by Jiangxi Province Institute of Water Sciences [2024SKTR05];
   Geophysical and Geochemical Survey institute of HuNan [GRE202310G];
"   Natural Science Foundation of Jiangxi, China [20242BAB25186]"
FX This research was funded by the Foundation by Jiangxi Province Institute
"   of Water Sciences (2024SKTR05), open project program in Hunan Provincial"
   Key Laboratory of Geochemical Processes and Resource Environmental
"   Effects, Geophysical and Geochemical Survey institute of HuNan"
"   (GRE202310G), Natural Science Foundation of Jiangxi, China"

(20242BAB25186).
"CR Asaf S, 2017, J PLANT INTERACT, V12, P31, DOI 10.1080/17429145.2016.1274060"
"   Awasthi AK, 2016, ENVIRON POLLUT, V211, P259, DOI 10.1016/j.envpol.2015.11.027"
"   Bilal S, 2018, ECOTOX ENVIRON SAFE, V164, P648, DOI 10.1016/j.ecoenv.2018.08.043"
"   Bolan N, 2021, ENVIRON POLLUT, V290, DOI 10.1016/j.envpol.2021.118067"
"   [????????? Cai Xixi], 2018, [????€?????????????, Ecology and Environmental Sciences], V27, P1160"
"   Cao YS, 2024, J SOIL SCI PLANT NUT, V24, P1761, DOI 10.1007/s42729-024-01769-y"
"   Caporaso JG, 2010, NAT METHODS, V7, P335, DOI 10.1038/nmeth.f.303"
"   Delgado-Baquerizo M, 2018, SCIENCE, V359, P320, DOI 10.1126/science.aap9516"
"   Denelle P, 2020, J BIOGEOGR, V47, P1922, DOI 10.1111/jbi.13848"
"   Don A, 2011, GLOBAL CHANGE BIOL, V17, P1658, DOI 10.1111/j.1365-2486.2010.02336.x"
"   Dong LL, 2017, CHIN MED-UK, V12, DOI 10.1186/s13020-017-0139-8"
"   Dordevic D, 2024, SCI REP-UK, V14, DOI 10.1038/s41598-024-68072-9"
"   Edgar RC, 2013, NAT METHODS, V10, P996, DOI [10.1038/NMETH.2604, 10.1038/nmeth.2604]"
"   Fouts DE, 2012, PLOS ONE, V7, DOI 10.1371/journal.pone.0048289"
"   Fridley JD, 2007, J ECOL, V95, P707, DOI 10.1111/j.1365-2745.2007.01236.x"
"   Frossard A, 2017, SOIL BIOL BIOCHEM, V105, P162, DOI 10.1016/j.soilbio.2016.11.016"
"   Guo Wei, 2011, Huanjing Kexue, V32, P3099"
"   He J, 2006, CHINA ENV SCI"
"   Hughes JB, 2005, METHOD ENZYMOL, V397, P292, DOI 10.1016/S0076-6879(05)97017-1"
"   Kurm V, 2019, ENVIRON MICROBIOL, V21, P750, DOI 10.1111/1462-2920.14492"
"   Li BQ, 2021, CHEMOSPHERE, V263, DOI 10.1016/j.chemosphere.2020.128227"
"   Li Dong, 2024, Chemosphere, V360, P142339, DOI [10.1016/j.chemosphere.2024.142339, 10.1016/j.chemosphere.2024.142339]"
"   [????????? Li Zhuoyang], 2022, [??????????????????, Chinese Journal of Tropical Crops], V43, P589"
"   Lion GN, 2013, S AFR J BOT, V88, P178, DOI 10.1016/j.sajb.2013.07.014"
"   Liu W, 2024, GONDWANA RES, V135, P198, DOI 10.1016/j.gr.2024.07.024"
"   Magoc T, 2011, BIOINFORMATICS, V27, P2957, DOI 10.1093/bioinformatics/btr507"
"   Mao YB, 2022, J AGR FOOD CHEM, V70, P8513, DOI 10.1021/acs.jafc.2c01437"
"   Monard C, 2016, SCI REP-UK, V6, DOI 10.1038/srep37719"
"   Nam IH, 2019, MINERALS-BASEL, V9, DOI 10.3390/min9100568"
"   Nemerow N.L., 1974, Scientific Stream Pollution Analysis"
"   Price MN, 2009, MOL BIOL EVOL, V26, P1641, DOI 10.1093/molbev/msp077"
"   Qi Q, 2022, ENVIRON POLLUT, V306, DOI 10.1016/j.envpol.2022.119406"
"   Sinkko H, 2013, PLOS ONE, V8, DOI 10.1371/journal.pone.0067061"
"   Soliman MA, 2024, J SOIL SCI PLANT NUT, V24, P8005, DOI 10.1007/s42729-024-02094-0"
"   Wang Q, 2007, APPL ENVIRON MICROB, V73, P5261, DOI 10.1128/AEM.00062-07"
"   Wang XL, 2015, ENVIRON SCI POLLUT R, V22, P264, DOI 10.1007/s11356-014-3340-7"
"   Willis AD, 2019, FRONT MICROBIOL, V10, DOI 10.3389/fmicb.2019.02407"
"   Xu LH, 2017, STAND GENOMIC SCI, V12, DOI 10.1186/s40793-017-0231-9"
"   Yan Min, 2020, Weishengwu Xuebao, V60, P2423, DOI 10.13343/j.cnki.wsxb.20200014"
"   Yang YR, 2015, SOIL BIOL BIOCHEM, V86, P146, DOI 10.1016/j.soilbio.2015.03.018"
"   Yao YR, 2021, B ENVIRON CONTAM TOX, V107, P996, DOI 10.1007/s00128-021-03350-0"
"   Zhang Y, 2021, PB ZN ISOTOPIC CHARA, DOI [10.26945/d.cnki.gbjku.2021.000319, DOI 10.26945/D.CNKI.GBJKU.2021.000319]"
"   Zhang Z, 2014, EFFECTS PECTIN XYLAN"
"   Zhao FJ, 2015, ENVIRON SCI TECHNOL, V49, P750, DOI 10.1021/es5047099"
NR 44
TC 1
Z9 1
U1 13
U2 17
PU SPRINGER INT PUBL AG
PI CHAM
"PA GEWERBESTRASSE 11, CHAM, CH-6330, SWITZERLAND"
SN 0718-9508
EI 0718-9516
J9 J SOIL SCI PLANT NUT
JI J. Soil Sci. Plant Nutr.
PD SEP
PY 2025
VL 25
IS 3
BP 8044
EP 8053
DI 10.1007/s42729-025-02653-z
EA AUG 2025
PG 10
WC Plant Sciences; Environmental Sciences; Soil Science
WE Science Citation Index Expanded (SCI-EXPANDED)
SC Plant Sciences; Environmental Sciences & Ecology; Agriculture
GA 7EZ9D
UT WOS:001544160600001
DA 2026-04-06
ER

PT J
"AU Tang, HY"
"   Xiang, GH"
"   Xiao, W"
"   Yang, ZL"
"   Zhao, BY"
"AF Tang, Haiying"
"   Xiang, Guohong"

"   Xiao, Wen"
"   Yang, Zeliang"
"   Zhao, Baoyi"
TI Microbial mediated remediation of heavy metals toxicity: mechanisms and
   future prospects
SO FRONTIERS IN PLANT SCIENCE
LA English
DT Review
DE bio-sorption; genetic engineering; heavy metals; bioremediation;
   nano-particles
ID LEAD-CONTAMINATED SOIL; ACID-MINE DRAINAGE; HEXAVALENT CHROMIUM;
   BACILLUS-SUBTILIS; OXIDATIVE STRESS; CARBONATE PRECIPITATION; NICKEL
   PHYTOEXTRACTION; PSEUDOMONAS-AERUGINOSA; RESISTANT BACTERIUM;
   AQUEOUS-SOLUTIONS
"AB Heavy metal pollution has become a serious concern across the globe due to their persistent nature, higher toxicity, and recalcitrance. These toxic metals threaten the stability of the environment and the health of all living beings. Heavy metals also enter the human food chain by eating contaminated foods and cause toxic effects on human health. Thus, remediation of HMs polluted soils is mandatory and it needs to be addressed at higher priority. The use of microbes is considered as a promising approach to combat the adverse impacts of HMs. Microbes aided in the restoration of deteriorated environments to their natural condition, with long-term environmental effects. Microbial remediation prevents the leaching and mobilization of HMs and they also make the extraction of HMs simple. Therefore, in this context recent technological advancement allowed to use of bioremediation as an imperative approach to remediate polluted soils. Microbes use different mechanisms including bio-sorption, bioaccumulation, bioleaching, bio-transformation, bio-volatilization and bio-mineralization to mitigate toxic the effects of HMs. Thus, keeping in the view toxic HMs here in this review explores the role of bacteria, fungi and algae in bioremediation of polluted soils. This review also discusses the various approaches that can be used to improve the efficiency of microbes to remediate HMs polluted soils. It also highlights different research gaps that must be solved in future study programs to improve bioremediation efficency."
"C1 [Tang, Haiying; Xiang, Guohong; Xiao, Wen; Yang, Zeliang] Hunan Univ Humanities Sci & Technol, Sch Agr & Biotechnol, Loudi, Peoples R China."
"   [Zhao, Baoyi] Shuangfeng Agr & Rural Bur, Loudi, Hunan, Peoples R China."
"C3 Hunan University Of Humanities, Science & Technology"
"RP Yang, ZL (corresponding author), Hunan Univ Humanities Sci & Technol, Sch Agr & Biotechnol, Loudi, Peoples R China.; Zhao, BY (corresponding author), Shuangfeng Agr & Rural Bur, Loudi, Hunan, Peoples R China."
EM bioinfo@qq.com; zby13397388807@163.com
FU National Natural Science Foundation of China [32060318]; Hunan
"   Provincial Natural Science Foundation of China [2023JJ50474,"
   2023JJ50475]; The 2023 Industry-University Collaborative Education
   Project of the Higher Education Department of the Ministry of Education
"   [230801720230445, 2023]; Hunan University Students Innovation and"
   Entrepreneurship Training Program [S202310553008]; Ministry of Education
   Supply and Demand docking Employment Education [2024011153277]
"FX The author(s) declare financial support was received for the research,"
"   authorship, and/or publication of this article. This work was supported"
   and funded by National Natural Science Foundation of China (32060318);
"   Hunan Provincial Natural Science Foundation of China (2023JJ50474,"
   2023JJ50475); 2023 Industry-University Collaborative Education Project
   of the Higher Education Department of the Ministry of Education
   (230801720230445). 2023 Hunan University Students Innovation and
   Entrepreneurship Training Program (S202310553008). 2024 Ministry of
   Education Supply and Demand docking Employment Education Project:
   Research on Employment-oriented Practical Teaching Model Reform of Plant
   Production Courses (2024011153277).
"CR Abbas SZ, 2018, INT J ENVIRON SCI TE, V15, P243, DOI 10.1007/s13762-017-1400-5"
"   Abbaszadeh-Dahaji P, 2019, ENVIRON SCI POLLUT R, V26, P31822, DOI 10.1007/s11356-019-06334-0"
"   Abhijit Gupta Abhijit Gupta, 2016, Journal of Microbial and Biochemical Technology, V8, P364, DOI 10.4172/1948-5948.1000310"
"   Acar FN, 2004, BIORESOURCE TECHNOL, V94, P13, DOI 10.1016/j.biortech.2003.10.032"
"   Achal V, 2012, J HAZARD MATER, V201, P178, DOI 10.1016/j.jhazmat.2011.11.067"
"   Acosta JA, 2011, J GEOCHEM EXPLOR, V109, P8, DOI 10.1016/j.gexplo.2011.01.004"
"   Adetunji AI, 2023, MINERALS-BASEL, V13, DOI 10.3390/min13060828"
"   Adrees M, 2015, ENVIRON SCI POLLUT R, V22, P8148, DOI 10.1007/s11356-015-4496-5"
"   AL-Huqail AA, 2022, INT J ENVIRON SCI TE, V19, P5301, DOI 10.1007/s13762-021-03532-7"
"   Alaboudi KA, 2018, ANN AGR SCI-CAIRO, V63, P123, DOI 10.1016/j.aoas.2018.05.007"
"   Alboghobeish H, 2014, J ENVIRON HEALTH SCI, V12, DOI 10.1186/2052-336X-12-44"
"   Alkorta I, 2017, FEMS MICROBIOL LETT, V364, DOI 10.1093/femsle/fnx200"
"   Alsokari S., 2011, J. Appl. Sci. Res., V7, P376"
"   Aluko OA, 2021, J ENVIRON MANAGE, V281, DOI 10.1016/j.jenvman.2020.111892"
"   Alves ARA, 2022, SCI TOTAL ENVIRON, V838, DOI 10.1016/j.scitotenv.2022.156435"
"   Alviz-Gazitua P, 2019, FRONT MICROBIOL, V10, DOI 10.3389/fmicb.2019.01499"
"   Amirnia S, 2015, CHEM ENG J, V264, P863, DOI 10.1016/j.cej.2014.12.016"
"   Arce-Inga M, 2022, MICROORGANISMS, V10, DOI 10.3390/microorganisms10112108"
"   Armah Frederick Ato, 2014, Int Sch Res Notices, V2014, P252148, DOI 10.1155/2014/252148"
"   Arshad F, 2019, SEP PURIF TECHNOL, V209, P870, DOI 10.1016/j.seppur.2018.06.035"
"   Aslam F, 2020, INT MICROBIOL, V23, P253, DOI 10.1007/s10123-019-00098-w"
"   Asyakina L. K., 2021, Reclamation of post-technological landscapes: International experience, DOI [10.21603/2074-9414-2021-4, DOI 10.21603/2074-9414-2021-4]"
"   Ayangbenro AS, 2017, INT J ENV RES PUB HE, V14, DOI 10.3390/ijerph14010094"
"   Azad MA, 2014, CHINESE SCI BULL, V59, P703, DOI 10.1007/s11434-013-0058-8"
"   Bahafid W, 2013, J APPL MICROBIOL, V115, P727, DOI 10.1111/jam.12282"
"   Bakshi S, 2018, BURL DODDS AGR SCI, V49, P63, DOI 10.19103/AS.2017.0033.20"
"   Bala S, 2022, TOXICS, V10, DOI 10.3390/toxics10080484"

"   Bandara T, 2022, CHEMOSPHERE, V303, DOI 10.1016/j.chemosphere.2022.135153"
"   Bandowe BAM, 2014, ENVIRON INT, V65, P135, DOI 10.1016/j.envint.2013.12.018"
"   Bansal N, 2019, ECOTOX ENVIRON SAFE, V172, P281, DOI 10.1016/j.ecoenv.2019.01.094"
"   Baraga??o D, 2020, SCI REP-UK, V10, DOI 10.1038/s41598-020-58852-4"
"   Barquilha CER, 2017, J CLEAN PROD, V150, P58, DOI 10.1016/j.jclepro.2017.02.199"
"   Barsainya M, 2016, International Journal of Current Microbiology and Applied Sciences, V5, P274, DOI [10.20546/ijcmas.2016.501.027, DOI 10.20546/IJCMAS.2016.501.027]"
"   Bayramoglu G, 2011, WATER AIR SOIL POLL, V221, P391, DOI 10.1007/s11270-011-0798-5"
"   Bazihizina N, 2015, J PLANT PHYSIOL, V174, P137, DOI 10.1016/j.jplph.2014.10.011"
"   Bellion M, 2006, FEMS MICROBIOL LETT, V254, P173, DOI 10.1111/j.1574-6968.2005.00044.x"
"   Bentley R, 2002, MICROBIOL MOL BIOL R, V66, P250, DOI 10.1128/MMBR.66.2.250-271.2002"
"   BEVERIDGE TJ, 1989, ANNU REV MICROBIOL, V43, P147, DOI 10.1146/annurev.mi.43.100189.001051"
"   BEWLEY RJF, 1983, SCI TOTAL ENVIRON, V31, P41, DOI 10.1016/0048-9697(83)90055-4"
"   Bhati Tushar, 2019, Microbiology and Biotechnology Letters, V47, P269, DOI [10.4014/mbl.1808.08006, 10.4014/mbl.1808.08006]"
"   Bhatt P, 2022, J BIOMOL STRUCT DYN, V40, P3285, DOI 10.1080/07391102.2020.1846623"
"   Bilal M, 2018, MAR DRUGS, V16, DOI 10.3390/md16020065"
"   Bisht Jyoti., 2014, Oct Jour Env Res, V2"
"   Boj??rquez C, 2016, REV INT CONTAM AMBIE, V32, P407, DOI 10.20937/RICA.2016.32.04.04"
"   Boriov?? K, 2014, LETT APPL MICROBIOL, V59, P217, DOI 10.1111/lam.12266"
"   Brandt KK, 2010, SOIL BIOL BIOCHEM, V42, P748, DOI 10.1016/j.soilbio.2010.01.008"
"   Bravo D, 2022, FRONT ENV SCI-SWITZ, V10, DOI 10.3389/fenvs.2022.910234"
"   Briffa J, 2020, HELIYON, V6, DOI 10.1016/j.heliyon.2020.e04691"
"   Burns RG, 2013, SOIL BIOL BIOCHEM, V58, P216, DOI 10.1016/j.soilbio.2012.11.009"
"   Cabello-Conejo MI, 2014, PLANT SOIL, V379, P35, DOI 10.1007/s11104-014-2043-7"
"   Cao LX, 2008, CHEMOSPHERE, V71, P1769, DOI 10.1016/j.chemosphere.2008.01.066"
"   Cao XF, 2020, SCI TOTAL ENVIRON, V704, DOI 10.1016/j.scitotenv.2019.135378"
"   Casarrubia S, 2020, FRONT MICROBIOL, V11, DOI 10.3389/fmicb.2020.00341"
"   Cepoi L, 2022, ENVIRONMENTS, V9, DOI 10.3390/environments9010001"
"   Chan WF, 2013, J HAZARD MATER, V262, P1116, DOI 10.1016/j.jhazmat.2012.08.020"
"   Chandra R, 2020, INORG CHIM ACTA, V499, DOI 10.1016/j.ica.2019.119192"
"   Chang JN, 2020, CHEMOSPHERE, V241, DOI 10.1016/j.chemosphere.2019.125121"
"   CHANMUGATHAS P, 1988, ARCH ENVIRON CON TOX, V17, P229, DOI 10.1007/BF01056029"
"   Chen YY, 2021, FRONT MICROBIOL, V12, DOI 10.3389/fmicb.2021.641913"
"   Cheng Y., 2012, Elements, V8, P107112"
"   Cheng ZY, 2022, J HAZARD MATER, V426, DOI 10.1016/j.jhazmat.2021.128095"
"   Cheung KH, 2007, INT BIODETER BIODEGR, V59, P8, DOI 10.1016/j.ibiod.2006.05.002"
"   Chowardhara B, 2020, ACTA PHYSIOL PLANT, V42, DOI 10.1007/s11738-020-03094-0"
"   Christoforidis AK, 2015, CHEM ENG J, V277, P334, DOI 10.1016/j.cej.2015.04.138"
"   Costa F, 2017, INT BIODETER BIODEGR, V120, P115, DOI 10.1016/j.ibiod.2017.02.018"
"   Cozzolino V, 2016, ENVIRON SCI POLLUT R, V23, P11312, DOI 10.1007/s11356-016-6337-6"
"   Cui LW, 2014, ENVIRON TOXICOL CHEM, V33, P334, DOI 10.1002/etc.2443"
"   Das P, 2014, BIOREMEDIAT J, V18, P169, DOI 10.1080/10889868.2014.889071"
"   De Francisco P, 2021, INT J ENV RES PUB HE, V18, DOI 10.3390/ijerph182212226"
"   Debonne N, 2021, GLOBAL ENVIRON CHANG, V66, DOI 10.1016/j.gloenvcha.2020.102221"
"   Deckwer WD, 2004, ENVIRON SCI TECHNOL, V38, P1858, DOI 10.1021/es0300517"
"   Demarco CF, 2023, SUSTAINABILITY-BASEL, V15, DOI 10.3390/su15021411"
"   Demey H, 2018, CHEM ENG J, V332, P582, DOI 10.1016/j.cej.2017.09.083"
"   Deng Y, 2021, MINERALS-BASEL, V11, DOI 10.3390/min11060622"
"   Dermont G, 2008, J HAZARD MATER, V152, P1, DOI 10.1016/j.jhazmat.2007.10.043"
"   Dhanapal AR, 2024, ACS OMEGA, V9, P13522, DOI 10.1021/acsomega.3c09776"
"   Dickerhof N, 2019, J BIOL CHEM, V294, P13502, DOI 10.1074/jbc.RA119.009934"
"   Diep P, 2018, FRONT BIOENG BIOTECH, V6, DOI 10.3389/fbioe.2018.00157"
"   Ding YF, 2020, J AGR FOOD CHEM, V68, P1958, DOI 10.1021/acs.jafc.9b07468"
"   Dixit R, 2015, SUSTAINABILITY-BASEL, V7, P2189, DOI 10.3390/su7022189"
"   Du JK, 2016, WATER RES, V102, P73, DOI 10.1016/j.watres.2016.06.009"
"   Durand A, 2016, PLANT SOIL, V399, P179, DOI 10.1007/s11104-015-2691-2"
"   Elahi Amina, 2019, Biotechnology Reports, V21, pe00307, DOI [10.1016/j.btre.2019.e00307, 10.1016/j.btre.2019.e00307]"
"   Eneh Onyenekenwa C, 2023, Environ Anal Health Toxicol, V38, pe2023001, DOI [10.5620/eaht.2023001, 10.5620/eaht.2023001]"
"   Fang LC, 2011, J HAZARD MATER, V190, P810, DOI 10.1016/j.jhazmat.2011.03.122"
"   Faskhutdinova Elizaveta R., 2021, Food Processing: Techniques and Technology, V51, P883, DOI [10.21603/2074-9414-2021-4-883-904, 10.21603/2074-9414-2021-4-883-904]"
"   Fawzy E. M., 2017, Journal of Bioremediation and Biodegradation, V8, P394, DOI [10.5897/jtehs2017.0385, 10.4172/2155-6199.1000394]"
"   Feng GF, 2023, MINERALS-BASEL, V13, DOI 10.3390/min13040546"
"   Feria-C??ceres PF, 2022, MICROBIOL RES-ITALY, V13, P556, DOI 10.3390/microbiolres13030039"
"   Fern??ndez PM, 2018, CHEMOSPHERE, V208, P139, DOI 10.1016/j.chemosphere.2018.05.166"
"   Ferreira V, 2016, ENVIRON POLLUT, V210, P261, DOI 10.1016/j.envpol.2015.12.060"
"   Foyer CH, 2016, PLANT CELL ENVIRON, V39, P951, DOI 10.1111/pce.12621"
"   Freedman Z, 2012, APPL ENVIRON MICROB, V78, P6568, DOI 10.1128/AEM.01060-12"
"   Pinter IF, 2017, APPL SOIL ECOL, V109, P60, DOI 10.1016/j.apsoil.2016.10.003"
"   Gadd GM, 2010, MICROBIOL-SGM, V156, P609, DOI 10.1099/mic.0.037143-0"
"   Galiulin RV, 2008, HER RUSS ACAD SCI+, V78, P141, DOI 10.1134/S1019331608020044"
"   Gao ML, 2020, PLANT PHYSIOL BIOCH, V154, P287, DOI 10.1016/j.plaphy.2020.06.021"
"   Ghosh Sougata, 2022, Bacilli in agrobiotechnology: plant stress tolerance, bioremediation, and bioprospecting, P1, DOI 10.1007/978-3-030-85465-2_1"
"   Gill Sarvajeet S., 2012, The Plant Family Brassicaceae, P99"
"   Giovanella P, 2016, NEW BIOTECHNOL, V33, P216, DOI 10.1016/j.nbt.2015.05.006"
"   Gomes MP, 2011, SCI AGR, V68, P566, DOI 10.1590/S0103-90162011000500009"
"   Gong YY, 2018, WATER RES, V147, P440, DOI 10.1016/j.watres.2018.10.024"
"   Gontia-Mishra I, 2016, J PLANT GROWTH REGUL, V35, P1000, DOI 10.1007/s00344-016-9598-x"
"   Grujic S, 2017, WATER AIR SOIL POLL, V228, DOI 10.1007/s11270-017-3259-y"

"   Gu YL, 2015, ENVIRON SCI POLLUT R, V22, P6271, DOI 10.1007/s11356-014-3856-x"
"   Guan QY, 2022, SCI TOTAL ENVIRON, V833, DOI 10.1016/j.scitotenv.2022.155096"
"   Guilherme Maria de Fatima de Souza, 2015, Acta Scientiarum Biological Sciences, V37, P499, DOI 10.4025/actascibiolsci.v37i4.28148"
"   Gupta P, 2015, ENVIRON EXP BOT, V109, P12, DOI 10.1016/j.envexpbot.2014.08.001"
"   Lima AIG, 2006, ENZYME MICROB TECH, V39, P763, DOI 10.1016/j.enzmictec.2005.12.009"
"   Hadia-e-Fatima, 2018, PEERJ, V6, DOI 10.7717/peerj.6076"
"   Hakeem K.R., 2020, BIOREMEDIATION BIOTE, DOI [DOI 10.1007/978-3-030-35691-0, 10.1007/978-3-030-35691-0_3, DOI 10.1007/978-3-030-35691-0_10]"
"   Hammaini A, 2007, J ENVIRON MANAGE, V84, P419, DOI 10.1016/j.jenvman.2006.06.015"
"   Hamsa N, 2017, International Journal of Current Microbiology and Applied Sciences, V6, P816, DOI [10.20546/ijcmas.2017.605.092, DOI 10.20546/IJCMAS.2017.605.092]"
"   Hamzah A, 2015, J RADIOANAL NUCL CH, V304, P151, DOI 10.1007/s10967-014-3775-9"
"   Haque S, 2022, SCI TOTAL ENVIRON, V833, DOI 10.1016/j.scitotenv.2022.155222"
"   Hassan MU, 2019, ENVIRON SCI POLLUT R, V26, P12673, DOI 10.1007/s11356-019-04892-x"
"   Hassan SE, 2013, NEW BIOTECHNOL, V30, P780, DOI 10.1016/j.nbt.2013.07.002"
"   He J, 2019, INT BIODETER BIODEGR, V140, P67, DOI 10.1016/j.ibiod.2019.03.012"
"   Heidari J, 2020, INT J PHYTOREMEDIAT, V22, P1175, DOI 10.1080/15226514.2020.1748564"
"   Henson M. W., 2015, isolated chromate contaminated sediment. PeerJ, V3, P1395, DOI [10.7717/peerj.1395, DOI 10.7717/PEERJ.1395]"
"   HIGHAM DP, 1986, ENVIRON HEALTH PERSP, V65, P5, DOI 10.2307/3430155"
"   Hlihor RM, 2017, PROCESS SAF ENVIRON, V108, P44, DOI 10.1016/j.psep.2016.06.016"
"   Hockmann K, 2014, J HAZARD MATER, V275, P215, DOI 10.1016/j.jhazmat.2014.04.065"
"   Hogsden KL, 2012, FRESHW SCI, V31, P108, DOI 10.1899/11-091.1"
"   Hu NingJing Hu NingJing, 2010, Acta Pedologica Sinica, V47, P246"
"   Huang C, 2017, CHEMOSPHERE, V187, P70, DOI 10.1016/j.chemosphere.2017.08.104"
"   Huang F, 2013, COLLOID SURFACE B, V107, P11, DOI 10.1016/j.colsurfb.2013.01.062"
"   Huang HJ, 2020, CHEMOSPHERE, V251, DOI 10.1016/j.chemosphere.2020.126428"
"   Huang HM, 2022, FRONT MICROBIOL, V13, DOI 10.3389/fmicb.2022.871581"
"   Huang K, 2015, APPL ENVIRON MICROB, V81, P6718, DOI 10.1128/AEM.01535-15"
"   Ibarrolaza A, 2011, J APPL MICROBIOL, V111, P26, DOI 10.1111/j.1365-2672.2011.05036.x"
"   Iram S, 2015, ARAB J SCI ENG, V40, P1867, DOI 10.1007/s13369-015-1702-1"
"   Iram Shazia Iram Shazia, 2013, International Research Journal of Biological Sciences, V2, P66"
"   Isaac CPJ, 2013, DESALIN WATER TREAT, V51, P7700, DOI 10.1080/19443994.2013.778218"
"   Jaiswal S, 2019, FRONT MICROBIOL, V10, DOI 10.3389/fmicb.2019.00087"
"   Jiang M, 2008, LETT APPL MICROBIOL, V47, P561, DOI 10.1111/j.1472-765X.2008.02454.x"
"   Joner EJ, 1997, NEW PHYTOL, V135, P353, DOI 10.1046/j.1469-8137.1997.00633.x"
"   Joutey NT, 2015, REV ENVIRON CONTAM T, V233, P45, DOI 10.1007/978-3-319-10479-9_2"
"   Kaksonen AH, 2020, J CLEAN PROD, V264, DOI 10.1016/j.jclepro.2020.121586"
"   Kalita Debajit, 2017, Biotechnol Rep (Amst), V16, P48, DOI [10.1016/j.btre.2017.11.003, 10.1016/j.btre.2017.11.003]"
"   Kang CH, 2016, ECOL ENG, V89, P64, DOI 10.1016/j.ecoleng.2016.01.023"
"   Kang JW, 2014, BIOTECHNOL LETT, V36, P1129, DOI 10.1007/s10529-014-1466-9"
"   Kang X, 2018, FRONT MICROBIOL, V9, DOI 10.3389/fmicb.2018.01853"
"   Ke T, 2021, ENVIRON POLLUT, V271, DOI 10.1016/j.envpol.2020.116314"
"   Khalid S, 2017, J GEOCHEM EXPLOR, V182, P247, DOI 10.1016/j.gexplo.2016.11.021"
"   Khan I, 2019, ENVIRON MONIT ASSESS, V191, DOI 10.1007/s10661-019-7769-5"
"   Khan I, 2021, NOT BOT HORTI AGROBO, V49, DOI 10.15835/nbha49212303"
"   Khati P, 2017, CLEAN-SOIL AIR WATER, V45, DOI 10.1002/clen.201600458"
"   Kicinska A, 2022, EUR J SOIL SCI, V73, DOI 10.1111/ejss.13203"
"   Kim JJ, 2019, J TRACE ELEM MED BIO, V54, P226, DOI 10.1016/j.jtemb.2019.05.003"
"   Kothe E., 2010, Soil heavy metals, V225-235, DOI [10.1007/978-3-642-02436-8, DOI 10.1007/978-3-642-02436-8]"
"   Kubrak OI, 2010, COMP BIOCHEM PHYS C, V152, P360, DOI 10.1016/j.cbpc.2010.06.003"
"   Kulp TR, 2014, ENVIRON SCI TECHNOL, V48, P218, DOI 10.1021/es403312j"
"   Kumar R., 2014, J OFNUCLEAR PHYS MAT, V7, P213, DOI DOI 10.15415/JNP.2014.12018"
"   Kushwaha A, 2017, ENVIRON SCI POLLUT R, V24, P10652, DOI 10.1007/s11356-017-8752-8"
"   Kwak HW, 2015, ALGAL RES, V7, P92, DOI 10.1016/j.algal.2014.12.006"
"   Lai CY, 2018, ENVIRON SCI TECHNOL, V52, P8693, DOI 10.1021/acs.est.8b02035"
"   Lai CY, 2016, WATER RES, V88, P467, DOI 10.1016/j.watres.2015.10.042"
"   Latif U, 2020, PLANTS-BASEL, V9, DOI 10.3390/plants9091084"
"   Leal MFC, 2023, TRACE ELEM ELECTROLY, V40, P1, DOI 10.5414/TE500038"
"   Lee HJ, 2021, J MICROBIOL BIOTECHN, V31, P903, DOI 10.4014/jmb.2106.06056"
"   Lerebours A, 2016, J APPL TOXICOL, V36, P1551, DOI 10.1002/jat.3313"
"   Leudo AM, 2020, SUSTAINABILITY-BASEL, V12, DOI 10.3390/su12093795"
"   Li CC, 2020, SCI TOTAL ENVIRON, V749, DOI 10.1016/j.scitotenv.2020.141555"
"   Li DD, 2017, J ENVIRON MANAGE, V196, P8, DOI 10.1016/j.jenvman.2017.02.076"
"   Li J, 2021, J HYDROL, V596, DOI 10.1016/j.jhydrol.2021.126092"
"   Li WW, 2015, BIOTECHNOL ADV, V33, P1, DOI 10.1016/j.biotechadv.2014.12.011"
"   Li XM, 2016, CHEMOSPHERE, V156, P228, DOI 10.1016/j.chemosphere.2016.04.098"
"   Li XX, 2017, GEOMICROBIOL J, V34, P346, DOI 10.1080/01490451.2016.1193571"
"   Li YL, 2023, ENVIRON POLLUT, V325, DOI 10.1016/j.envpol.2023.121433"
"   Lin WT, 2016, J ENVIRON SCI-CHINA, V45, P94, DOI 10.1016/j.jes.2015.12.010"
"   Liu CJ, 2023, CRIT REV ENV SCI TEC, V53, P723, DOI 10.1080/10643389.2023.2168365"
"   Liu SH, 2017, BIORESOURCE TECHNOL, V224, P25, DOI 10.1016/j.biortech.2016.11.095"
"   Liu S, 2011, J ENVIRON SCI-CHINA, V23, P1544, DOI 10.1016/S1001-0742(10)60570-0"
"   Liu WX, 2013, PLANT SOIL, V371, P533, DOI 10.1007/s11104-013-1717-x"
"   Lytras G, 2017, J HAZARD MATER, V336, P41, DOI 10.1016/j.jhazmat.2017.04.049"
"   Ma H, 2020, J HAZARD MATER, V388, DOI 10.1016/j.jhazmat.2020.122065"
"   Ma Y, 2015, J ENVIRON MANAGE, V156, P62, DOI 10.1016/j.jenvman.2015.03.024"
"   Magnin JP, 2014, CAN J MICROBIOL, V60, P829, DOI 10.1139/cjm-2014-0231"
"   Malekzadeh E, 2012, BIOREMEDIAT J, V16, P204, DOI 10.1080/10889868.2012.703258"
"   Malik A, 2004, ENVIRON INT, V30, P261, DOI 10.1016/j.envint.2003.08.001"
"   Malla MA, 2018, FRONT MICROBIOL, V9, DOI 10.3389/fmicb.2018.01132"

"   Manoj SR, 2020, J ENVIRON MANAGE, V254, DOI 10.1016/j.jenvman.2019.109779"
"   Manorma K., 2023, Integr Strateg Bioremediat Environ Contamin, V2, P113"
"   Mariano C, 2020, ENVIRON SCI POLLUT R, V27, P13550, DOI 10.1007/s11356-020-07913-2"
"   Marschner B, 2003, GEODERMA, V113, P211, DOI 10.1016/S0016-7061(02)00362-2"
"   Martis SB, 2021, CURR RES MICROB SCI, V2, DOI 10.1016/j.crmicr.2021.100029"
"   Mathew DC, 2015, PLOS ONE, V10, DOI 10.1371/journal.pone.0121178"
"   Mathur S, 2016, PHOTOSYNTHETICA, V54, P185, DOI 10.1007/s11099-016-0198-6"
"   Meng LD, 2023, FRONT PLANT SCI, V14, DOI 10.3389/fpls.2023.1112002"
"   Mestrot A, 2011, ENVIRON SCI TECHNOL, V45, P1798, DOI 10.1021/es103463d"
"   Miglani GS, 2017, J CROP IMPROV, V31, P453, DOI 10.1080/15427528.2017.1333192"
"   Mishra Devendra Kumar, 2022, J Complement Integr Med, V19, P513, DOI [10.1515/jcim-2020-0325, 10.1515/jcim-2020-0325]"
"   de Oliveira EC, 2023, CHEMOSPHERE, V319, DOI 10.1016/j.chemosphere.2023.138030"
"   Morton-Bermea O, 2002, B ENVIRON CONTAM TOX, V68, P383, DOI [10.1007/s00128-001-0265-x, 10.1007/s001280265]"
"   Murthy MK, 2023, REV ENVIRON HEALTH, V38, P461, DOI 10.1515/reveh-2021-0139"
"   Muzaffar S, 2023, CELL BIOL TOXICOL, V39, P85, DOI 10.1007/s10565-022-09710-8"
"   Nagvenkar GS, 2010, ECOTOXICOLOGY, V19, P604, DOI 10.1007/s10646-009-0429-8"
"   Narayanan M, 2023, J ENVIRON MANAGE, V345, DOI 10.1016/j.jenvman.2023.118732"
"   Naskar A, 2020, ENVIRON TECHNOL INNO, V20, DOI 10.1016/j.eti.2020.101057"
"   Nayak AK, 2015, ECOTOX ENVIRON SAFE, V114, P257, DOI 10.1016/j.ecoenv.2014.03.033"
"   Neelima P., 2003, J ENVIRON BIOL, V24, P453"
"   Nguyen VK, 2017, INT J HYDROGEN ENERG, V42, P27832, DOI 10.1016/j.ijhydene.2017.08.056"
"   Nikolaou KE, 2022, HORTICULTURAE, V8, DOI 10.3390/horticulturae8060493"
"   Nizamutdinov S. A. R., 2022, Food Process Tech Technol, V52, P2022, DOI [10.21603/2074-9414-2022-2, DOI 10.21603/2074-9414-2022-2]"
"   Nurzhan A, 2022, EURASIAN SOIL SCI+, V55, P1425, DOI 10.1134/S106422932210012X"
"   Nwuche CO, 2008, INT J ENVIRON SCI TE, V5, P409, DOI 10.1007/BF03326036"
"   Nziguheba G, 2008, SCI TOTAL ENVIRON, V390, P53, DOI 10.1016/j.scitotenv.2007.09.031"
"   Obani IS, 2023, IPS Journal of Public Health, V3, P10, DOI [10.54117/ijph.v3i1.15, 10.54117/ijph.v3i1.15, DOI 10.54117/IJPH.V3I1.15]"
"   Oyewole OA, 2019, SN APPL SCI, V1, DOI 10.1007/s42452-019-0879-4"
"   Pan J, 2011, ECOL ENG, V37, P1889, DOI 10.1016/j.ecoleng.2011.07.002"
"   Pattanapipitpaisal P, 2001, APPL MICROBIOL BIOT, V57, P257"
"   Paul A, 2016, POL J MICROBIOL, V65, P115, DOI 10.5604/17331331.1197284"
"   Paunov M, 2018, INT J MOL SCI, V19, DOI 10.3390/ijms19030787"
"   Pena-Castro JM, 2023, MICROORGANISMS, V11, DOI 10.3390/microorganisms11092197"
"   Pervaiz I, 2013, APPL BIOCHEM MICRO+, V49, P437, DOI 10.1134/S0003683813050098"
"   Philippot L, 2008, FEMS MICROBIOL LETT, V285, P51, DOI 10.1111/j.1574-6968.2008.01210.x"
"   Pietro-Souza W, 2020, CHEMOSPHERE, V240, DOI 10.1016/j.chemosphere.2019.124874"
"   Freitas APP, 2011, MINER ENG, V24, P1215, DOI 10.1016/j.mineng.2011.04.013"
"   Pirselov?? B, 2021, PLANTS-BASEL, V10, DOI 10.3390/plants10051007"
"   Poo KM, 2018, J ENVIRON MANAGE, V206, P364, DOI 10.1016/j.jenvman.2017.10.056"
"   Povedano-Priego C, 2017, MINER ENG, V106, P46, DOI 10.1016/j.mineng.2016.11.007"
"   Preetha JSY, 2023, MOLECULES, V28, DOI 10.3390/molecules28031474"
"   Purchase D, 2009, J APPL MICROBIOL, V106, P1163, DOI 10.1111/j.1365-2672.2008.04082.x"
"   Puyen ZM, 2012, BIORESOURCE TECHNOL, V126, P233, DOI 10.1016/j.biortech.2012.09.036"
"   Qian XY, 2017, J CLEAN PROD, V164, P198, DOI 10.1016/j.jclepro.2017.06.195"
"   Qin JQ, 2023, PROCESSES, V11, DOI 10.3390/pr11051562"
"   Quiroga-Mateus R, 2022, PROCESSES, V10, DOI 10.3390/pr10081457"
"   Rad SA, 2023, SUSTAINABILITY-BASEL, V15, DOI 10.3390/su15010876"
"   Rahman Z, 2020, ENVIRON SCI POLLUT R, V27, P27563, DOI 10.1007/s11356-020-08903-0"
"   Rahman Z, 2019, ENVIRON MONIT ASSESS, V191, DOI 10.1007/s10661-019-7528-7"
"   Rajkumar M, 2010, TRENDS BIOTECHNOL, V28, P142, DOI 10.1016/j.tibtech.2009.12.002"
"   Rajput V, 2018, SCI TOTAL ENVIRON, V645, P1103, DOI 10.1016/j.scitotenv.2018.07.211"
"   Rajput VD, 2022, NANOMATERIALS-BASEL, V12, DOI 10.3390/nano12050769"
"   Raklami A, 2021, J ENVIRON SCI, V99, P210, DOI 10.1016/j.jes.2020.06.032"
"   Ramesh A, 2010, RNA BIOL, V7, P77, DOI 10.4161/rna.7.1.10490"
"   Ramrakhiani L, 2011, CHEM ENG J, V171, P1060, DOI 10.1016/j.cej.2011.05.002"
"   Rani A, 2009, MICROBIAL STRATEGIES FOR CROP IMPROVEMENT, P85, DOI 10.1007/978-3-642-01979-1_5"
"   Raza A, 2024, PLANT CELL REP, V43, DOI 10.1007/s00299-024-03153-7"
"   Raza A, 2022, PLANT CELL REP, V41, P741, DOI 10.1007/s00299-020-02635-8"
"   Remen??r M, 2018, POL J MICROBIOL, V67, P191, DOI 10.21307/pjm-2018-022"
"   Ren GM, 2015, ECOTOX ENVIRON SAFE, V117, P141, DOI 10.1016/j.ecoenv.2015.03.033"
"   Rezaei H, 2016, ARAB J CHEM, V9, P846, DOI 10.1016/j.arabjc.2013.11.008"
"   Rhee YJ, 2012, CURR BIOL, V22, P237, DOI 10.1016/j.cub.2011.12.017"
"   Martins JPR, 2020, PLANT CELL TISS ORG, V143, P303, DOI 10.1007/s11240-020-01917-z"
"   Rodr??guez-Tirado V, 2012, CHEM ENG J, V181, P352, DOI 10.1016/j.cej.2011.11.091"
"   Rucinska-Sobkowiak R, 2016, ACTA PHYSIOL PLANT, V38, DOI 10.1007/s11738-016-2277-5"
"   Rugnini L, 2018, PLANT PHYSIOL BIOCH, V125, P45, DOI 10.1016/j.plaphy.2018.01.032"
"   Sati D, 2023, J SOIL SCI PLANT NUT, V23, P106, DOI 10.1007/s42729-021-00724-5"
"   Sayyed RZ, 2019, ENVIRON SUSTAIN, V2, P117, DOI [10.1007/s42398-019-00070-4, 10.1007/s42398-019-00070-4, DOI 10.1007/S42398-019-00070-4, 10.1007/s42398-019, DOI 10.1007/S42398-019]"
"   Sch??ck M, 2023, ENVIRON SCI POLLUT R, V30, P58030, DOI 10.1007/s11356-023-26490-8"
"   Shahpiri A, 2018, ANN MICROBIOL, V68, P145, DOI 10.1007/s13213-018-1326-2"
"   Shahzad B, 2018, PLANT PHYSIOL BIOCH, V132, P641, DOI 10.1016/j.plaphy.2018.10.014"
"   Shanab S, 2012, PLANT SIGNAL BEHAV, V7, P392, DOI 10.4161/psb.19173"
"   Shanker AK, 2005, ENVIRON INT, V31, P739, DOI 10.1016/j.envint.2005.02.003"
"   Sharma A, 2020, J PLANT GROWTH REGUL, V39, P509, DOI 10.1007/s00344-019-10018-x"
"   Sharma B, 2017, WASTE MANAGE, V64, P117, DOI 10.1016/j.wasman.2017.03.002"
"   Sher S, 2019, APPL MICROBIOL BIOT, V103, P6007, DOI 10.1007/s00253-019-09933-6"
"   Singh Jiwan Singh Jiwan, 2011, International Journal of Research in Chemistry and Environment (IJRCE), V1, P15"
"   Singh P, 2021, J ENVIRON MANAGE, V279, DOI 10.1016/j.jenvman.2020.111547"

"   Singh R., 2020, Bioremediation of Industrial Waste for Environmental Safety: Volume II: Biological Agents and Methods for Industrial Waste Management, P165"
"   Singh S., 2023, J. Popul Ther. Clin. Pharmacol, V30, P504"
"   Sofo A, 2017, J PLANT PHYSIOL, V216, P174, DOI 10.1016/j.jplph.2017.06.008"
"   Sone Y, 2013, AMB EXPRESS, V3, DOI 10.1186/2191-0855-3-41"
"   Soto DF, 2019, J PROTEOMICS, V191, P143, DOI 10.1016/j.jprot.2018.02.024"
"   Jeyasundar PGSA, 2021, ENVIRON POLLUT, V277, DOI 10.1016/j.envpol.2021.116789"
"   Souri Z, 2020, PHOTOSYNTHESIS, PRODUCTIVITY, AND ENVIRONMENTAL STRESS, P107"
"   Spagnoletti FN, 2016, ECOTOX ENVIRON SAFE, V133, P47, DOI 10.1016/j.ecoenv.2016.06.012"
"   Srichandan H, 2014, HYDROMETALLURGY, V150, P130, DOI 10.1016/j.hydromet.2014.09.019"
"   Srivastav A., 2018, PHYTOREMEDIATION, V6, P383, DOI [DOI 10.1007/978-3-319-99651-6_16, 10.1007/978-3-319-99651-6_16]"
"   Sun LQ, 2017, ENVIRON SCI POLLUT R, V24, P9681, DOI 10.1007/s11356-017-8637-x"
"   Sun SC, 2021, ARCH MICROBIOL, V203, P2699, DOI 10.1007/s00203-021-02271-0"
"   Talukdar D, 2020, ENVIRON TECHNOL INNO, V20, DOI 10.1016/j.eti.2020.101050"
"   Tan WJ, 2018, ENVIRON SCI-NANO, V5, P257, DOI 10.1039/c7en00985b"
"   Taran M, 2019, IRAN J MICROBIOL, V11, P60, DOI 10.18502/ijm.v11i1.707"
"   Teitzel GM, 2003, APPL ENVIRON MICROB, V69, P2313, DOI 10.1128/AEM.69.4.2313-2320.2003"
"   Thatoi H, 2014, J ENVIRON MANAGE, V146, P383, DOI 10.1016/j.jenvman.2014.07.014"
"   Thelwell C, 1998, P NATL ACAD SCI USA, V95, P10728, DOI 10.1073/pnas.95.18.10728"
"   Tirry N, 2021, J GENET ENG BIOTECHN, V19, DOI 10.1186/s43141-021-00254-8"
"   Tripathi DK, 2016, J PHOTOCH PHOTOBIO B, V154, P89, DOI 10.1016/j.jphotobiol.2015.11.008"
"   Uchimiya M, 2020, J AGR FOOD CHEM, V68, P12856, DOI 10.1021/acs.jafc.0c00183"
"   Umrania V, 2006, BIORESOURCE TECHNOL, V97, P1237, DOI 10.1016/j.biortech.2005.04.048"
"   Ur??k M, 2007, WATER AIR SOIL POLL, V186, P337, DOI 10.1007/s11270-007-9489-7"
"   Ustiatik R, 2022, BIOREMEDIAT J, V26, P208, DOI 10.1080/10889868.2021.1973950"
"   Aguirre CFV, 2018, ENVIRON EXP BOT, V156, P197, DOI 10.1016/j.envexpbot.2018.09.005"
"   Vel??squez L, 2009, J HAZARD MATER, V167, P713, DOI 10.1016/j.jhazmat.2009.01.044"
"   Verasoundarapandian G, 2022, AGRONOMY-BASEL, V12, DOI 10.3390/agronomy12010117"
"   Villadangos AF, 2014, APPL MICROBIOL BIOT, V98, P10143, DOI 10.1007/s00253-014-6055-2"
"   Villegas-Plazas M, 2019, J ENVIRON MANAGE, V251, DOI 10.1016/j.jenvman.2019.109581"
"   Wang LY, 2022, FRONT ENV SCI-SWITZ, V10, DOI 10.3389/fenvs.2022.895646"
"   Wang SL, 2009, J ENVIRON MANAGE, V90, P2367, DOI 10.1016/j.jenvman.2009.02.001"
"   Wang XJ, 2017, SCI REP-UK, V7, DOI 10.1038/s41598-017-14601-8"
"   Wang YM, 2020, CHEMOSPHERE, V242, DOI [10.1016/j.chemosphere.2019.125265, 10.1016/j.chemosphere.2019.125266]"
"   Wang Yaxiong, 2001, Huanjing Kexue, V22, P72"
"   Wen ZY, 2023, TOXICS, V11, DOI 10.3390/toxics11040371"
"   Wierzba S, 2015, POL J CHEM TECHNOL, V17, P79, DOI 10.1515/pjct-2015-0012"
"   Wu CC, 2020, SCI TOTAL ENVIRON, V703, DOI 10.1016/j.scitotenv.2019.134812"
"   Wu G, 2010, J HAZARD MATER, V174, P1, DOI 10.1016/j.jhazmat.2009.09.113"
"   Wu QH, 2015, SCI TOTAL ENVIRON, V506, P217, DOI 10.1016/j.scitotenv.2014.10.121"
"   Xi L, 2022, SCI TOTAL ENVIRON, V804, DOI 10.1016/j.scitotenv.2021.149904"
"   Xian Y, 2015, CHEMOSPHERE, V139, P604, DOI 10.1016/j.chemosphere.2014.12.060"
"   Yaashikaa PR, 2022, ENVIRON POLLUT, V312, DOI 10.1016/j.envpol.2022.120031"
"   Yadav G, 2014, BIOL TRACE ELEM RES, V158, P410, DOI 10.1007/s12011-014-9950-6"
"   Yan A, 2020, FRONT PLANT SCI, V11, DOI 10.3389/fpls.2020.00359"
"   Yang HC, 2016, BIOMED J, V39, P5, DOI 10.1016/j.bj.2015.08.003"
"   Yang T, 2015, TRAC-TREND ANAL CHEM, V66, P90, DOI 10.1016/j.trac.2014.11.016"
"   Yin K, 2016, WATER RES, V103, P383, DOI 10.1016/j.watres.2016.07.053"
"   Yin XZ, 2021, SCI TOTAL ENVIRON, V750, DOI 10.1016/j.scitotenv.2020.141233"
"   Yotsova E, 2020, PLANT PHYSIOL BIOCH, V155, P789, DOI 10.1016/j.plaphy.2020.06.042"
"   Yuan L, 2015, ENVIRON SCI TECHNOL, V49, P824, DOI 10.1021/es504207c"
"   Yue ZB, 2015, BIORESOURCE TECHNOL, V194, P399, DOI 10.1016/j.biortech.2015.07.042"
"   Zaidi S, 2006, CHEMOSPHERE, V64, P991, DOI 10.1016/j.chemosphere.2005.12.057"
"   Zammit CM, 2016, METALLOMICS, V8, P1204, DOI 10.1039/c6mt00142d"
"   Zapana-Huarache SV, 2020, BRAZ J MICROBIOL, V51, P271, DOI 10.1007/s42770-019-00209-9"
"   Zhang WW, 2012, APPL MICROBIOL BIOT, V93, P1305, DOI 10.1007/s00253-011-3454-5"
"   Zheng YJ, 2018, FRONT MICROBIOL, V9, DOI 10.3389/fmicb.2018.01490"
"   Zhou GT, 2016, WATER AIR SOIL POLL, V227, DOI 10.1007/s11270-016-3040-7"
"   Zhou W, 2012, APPL MICROBIOL BIOT, V95, P1097, DOI 10.1007/s00253-012-4245-3"
"   Zhu XJ, 2016, ENVIRON POLLUT, V219, P149, DOI 10.1016/j.envpol.2016.10.047"
"   Zhuang P, 2009, SCI TOTAL ENVIRON, V407, P1551, DOI 10.1016/j.scitotenv.2008.10.061"
NR 320
TC 71
Z9 88
U1 53
U2 110
PU FRONTIERS MEDIA SA
PI LAUSANNE
"PA AVENUE DU TRIBUNAL FEDERAL 34, LAUSANNE, CH-1015, SWITZERLAND"
SN 1664-462X
J9 FRONT PLANT SCI
JI Front. Plant Sci.
PD JUL 19
PY 2024
VL 15
AR 1420408
DI 10.3389/fpls.2024.1420408
PG 23
WC Plant Sciences
WE Science Citation Index Expanded (SCI-EXPANDED)
SC Plant Sciences
GA A3X2O

UT WOS:001281887900001
PM 39100088
"OA Green Submitted, gold"
DA 2026-04-06
ER

PT J
"AU Liu, NQ"
"   Zhao, J"
"   Du, JW"
"   Hou, C"
"   Zhou, XF"
"   Chen, JB"
"   Zhang, YL"
"AF Liu, Nengqian"
"   Zhao, Jiang"
"   Du, Jiawen"
"   Hou, Cheng"
"   Zhou, Xuefei"
"   Chen, Jiabin"
"   Zhang, Yalei"
TI Non-phytoremediation and phytoremediation technologies of integrated
   remediation for water and soil heavy metal pollution: A comprehensive
   review
SO SCIENCE OF THE TOTAL ENVIRONMENT
LA English
DT Review
DE Heavy metals; Pollution; Integrated remediation; Non-phytoremediation;
   Phytoremediation
ID CONTAMINATED SOILS; PLANT-GROWTH; LEAD IMMOBILIZATION; ORGANIC
   POLLUTANTS; NUTRIENT ELEMENTS; AQUEOUS-SOLUTIONS; WASTE-WATER; REMOVAL;
   BIOCHAR; ACCUMULATION
"AB Since the 1980s, there has been increasing concern over heavy metal pollution remediation. However, most research focused on the individual remediation technologies for heavy metal pollutants in either soil or water. Considering the potential migration of these pollutants, it is necessary to explore effective integrated remediation technologies for soil and water heavy metals. This review thoroughly examines non-phytoremediation technologies likes physical, chemical, and microbial remediation, as well as green remediation approaches involving terrestrial and aquatic phytoremediation. Non-phytoremediation technologies suffer from disadvantages like high costs, secondary pollution risks, and susceptibility to environmental factors. Conversely, phytoremediation technologies have gained significant attention due to their sustainable and environmentally friendly nature. Enhancements through chelating agents, biochar, microorganisms, and genetic engineering have demonstrated improved phytoremediation remediation efficiency. However, it is essential to address the environmental and ecological risks that may arise from the prolonged utilization of these materials and technologies. Lastly, this paper presents an overview of integrated remediation approaches for addressing heavy metal contamination in groundwater-soil-surface water systems and discusses the reasons for the research gaps and future directions. This paper offers valuable insights for comprehensive solutions to heavy metal pollution in water and soil, promoting integrated remediation and sustainable development."
"C1 [Liu, Nengqian; Du, Jiawen; Hou, Cheng; Zhou, Xuefei; Chen, Jiabin; Zhang, Yalei] Tongji Univ, Coll Environm Sci & Engn, State Key Lab Pollut Control & Resources Reuse, Shanghai 200092, Peoples R China."
"   [Zhao, Jiang] Shanghai Rural Revitalizat Res Ctr, Shanghai, Peoples R China."
"   [Zhou, Xuefei; Chen, Jiabin; Zhang, Yalei] Shanghai Inst Pollut Control & Ecol Secur, Shanghai 200092, Peoples R China."
C3 Tongji University; Shanghai Institute of Pollution Control & Ecological
   Security
"RP Zhang, YL (corresponding author), Tongji Univ, Coll Environm Sci & Engn, State Key Lab Pollut Control & Resources Reuse, Shanghai 200092, Peoples R China."
EM zhangyalei@tongji.edu.cn
"RI Zhang, Lei/CAJ-1245-2022; Du, Jiawen/LWK-3891-2024"
"OI Hou, Cheng/0009-0008-6674-1019; "
FU Nation Key R & D Program of China [2021YFC2102200]; China Baowu Low
   Carbon Metallurgy Innovation Foundation [BWLCF202105]
FX This work was supported by the Nation Key R & D Program of China (No:
   2021YFC2102200) and China Baowu Low Carbon Metallurgy Innovation
   Foundation (No: BWLCF202105) .
"CR Abdullah NH, 2019, COMPOS PART B-ENG, V162, P538, DOI 10.1016/j.compositesb.2018.12.075"
"   Abou-Shanab RAI, 2020, ECOTOX ENVIRON SAFE, V195, DOI 10.1016/j.ecoenv.2020.110458"
"   Adeyi AA, 2020, INT J ENVIR WASTE MA, V25, P474, DOI 10.1504/IJEWM.2020.107566"
"   Aghelan N, 2021, J ENVIRON HEALTH SCI, V19, P503, DOI 10.1007/s40201-021-00623-y"
"   Ahemad Munees, 2014, Journal of King Saud University Science, V26, P1, DOI 10.1016/j.jksus.2013.05.001"
"   Ahmad M, 2014, CHEMOSPHERE, V99, P19, DOI 10.1016/j.chemosphere.2013.10.071"
"   Ali H, 2013, CHEMOSPHERE, V91, P869, DOI 10.1016/j.chemosphere.2013.01.075"
"   Ali S, 2022, ENVIRON POLLUT, V300, DOI 10.1016/j.envpol.2022.118940"
"   Alok A., 2017, Metabolic engineering for bioactive compounds, P91, DOI DOI 10.1007/978-981-10-5511-9_5"
"   Anastopoulos I, 2019, ENVIRON CHEM LETT, V17, P755, DOI 10.1007/s10311-018-00829-x"
"   Arabyarmohammadi H, 2018, J POLYM ENVIRON, V26, P2107, DOI 10.1007/s10924-017-1102-6"
"   Azad MA, 2014, CHINESE SCI BULL, V59, P703, DOI 10.1007/s11434-013-0058-8"
"   Azadi N, 2021, BIOCHAR, V3, P485, DOI 10.1007/s42773-021-00123-0"
"   Bahemmat M, 2015, ENVIRON EARTH SCI, V74, P4639, DOI 10.1007/s12665-015-4429-0"
"   Bardos P, 2018, REMEDIATION, V28, P43, DOI 10.1002/rem.21559"
"   Basharat Z, 2018, PLANTS-BASEL, V7, DOI 10.3390/plants7030051"
"   Bhat SA, 2022, CHEMOSPHERE, V303, DOI 10.1016/j.chemosphere.2022.134788"
"   Bhatta D, 2023, ECOTOX ENVIRON SAFE, V263, DOI 10.1016/j.ecoenv.2023.115377"
"   Bi R, 2019, J ENVIRON MANAGE, V238, P484, DOI 10.1016/j.jenvman.2019.02.064"

"   Bian FY, 2021, J HAZARD MATER, V416, DOI 10.1016/j.jhazmat.2021.125898"
"   Bian FY, 2020, CHEMOSPHERE, V246, DOI 10.1016/j.chemosphere.2019.125750"
"   Bisht A, 2022, PLANT GROWTH REGUL, V96, P409, DOI 10.1007/s10725-021-00791-9"
"   Bokhari SH, 2022, INT J PHYTOREMEDIAT, V24, P1465, DOI 10.1080/15226514.2022.2035673"
"   Cabral L, 2015, WORLD J MICROB BIOT, V31, P1655, DOI 10.1007/s11274-015-1918-y"
"   Cao YN, 2022, SCI TOTAL ENVIRON, V813, DOI 10.1016/j.scitotenv.2021.152482"
"   Chandra P, 2004, BOT REV, V70, P313, DOI 10.1663/0006-8101(2004)070[0313:CAATIA]2.0.CO;2"
"   Chang JJ, 2022, J HAZARD MATER, V425, DOI 10.1016/j.jhazmat.2021.127948"
"   Chen L, 2020, J CLEAN PROD, V263, DOI 10.1016/j.jclepro.2020.121491"
"   Chen M, 2022, J HAZARD MATER, V424, DOI 10.1016/j.jhazmat.2021.127272"
"   Chen QY, 2018, J WATER PROCESS ENG, V26, P289, DOI 10.1016/j.jwpe.2018.11.003"
"   Chen W, 2022, CHEMOSPHERE, V289, DOI 10.1016/j.chemosphere.2021.133260"
"   Chen ZB, 2022, J HAZARD MATER, V439, DOI 10.1016/j.jhazmat.2022.129562"
"   Chien SWC, 2021, J HAZARD MATER, V403, DOI 10.1016/j.jhazmat.2020.123549"
"   Choi JY, 2009, ENVIRON SCI TECHNOL, V43, P3030, DOI 10.1021/es802388s"
"   Cui EP, 2021, SCI TOTAL ENVIRON, V784, DOI 10.1016/j.scitotenv.2021.147093"
"   Cui G, 2017, APPL MICROBIOL BIOT, V101, P6821, DOI 10.1007/s00253-017-8406-2"
"   Cui HB, 2017, RSC ADV, V7, P45869, DOI 10.1039/c7ra08786a"
"   Cui J, 2024, J HAZARD MATER, V465, DOI 10.1016/j.jhazmat.2023.133109"
"   Cui LQ, 2021, BIOCHAR, V3, P457, DOI 10.1007/s42773-021-00106-1"
"   Dadari S, 2022, CHEM ENG J, V431, DOI 10.1016/j.cej.2021.134116"
"   Das SK, 2021, J ENVIRON MANAGE, V278, DOI 10.1016/j.jenvman.2020.111501"
"   Deng B, 2022, J PLANT GROWTH REGUL, V41, P639, DOI 10.1007/s00344-021-10327-0"
"   Deng TL, 2017, CHEMOSPHERE, V168, P450, DOI 10.1016/j.chemosphere.2016.09.152"
"   Dietz S, 2020, SCI REP-UK, V10, DOI 10.1038/s41598-019-54309-5"
"   Durand A, 2016, PLANT SOIL, V399, P179, DOI 10.1007/s11104-015-2691-2"
"   Edenborn SL, 2015, J ENVIRON MANAGE, V150, P226, DOI 10.1016/j.jenvman.2014.11.023"
"   Eid EM, 2020, ENVIRON SCI POLLUT R, V27, P12138, DOI 10.1007/s11356-020-07839-9"
"   El-Nagar DA, 2020, ARAB J CHEM, V13, P7695, DOI 10.1016/j.arabjc.2020.09.005"
"   Fang W, 2016, J HAZARD MATER, V310, P1, DOI 10.1016/j.jhazmat.2016.02.025"
"   Farahat E, 2018, ENVIRON SCI POLLUT R, V25, P4214, DOI 10.1007/s11356-017-0808-2"
"   Fei XF, 2019, ECOL INDIC, V106, DOI 10.1016/j.ecolind.2019.105508"
"   Fern??ndez V, 2021, PLANT J, V105, P870, DOI 10.1111/tpj.15090"
"   Gao CX, 2021, CELL, V184, P1621, DOI 10.1016/j.cell.2021.01.005"
"   Gao Jun, 2023, Chemosphere, V335, P139088, DOI [10.1016/j.chemosphere.2023.139088, 10.1016/j.chemosphere.2023.139088]"
"   Gao XP, 2020, INT J BIOL MACROMOL, V164, P4423, DOI 10.1016/j.ijbiomac.2020.09.046"
"   Geng N, 2022, J HAZARD MATER, V430, DOI 10.1016/j.jhazmat.2022.128455"
"   Ghazaryan KA, 2022, ENVIRON GEOCHEM HLTH, V44, P1203, DOI 10.1007/s10653-021-01151-2"
"   Ghosh D, 2021, INT J PHYTOREMEDIAT, V23, P559, DOI 10.1080/15226514.2020.1840510"
"   Gil-D??az M, 2020, SCI REP-UK, V10, DOI 10.1038/s41598-020-73144-7"
"   Gong LD, 2021, CHEMOSPHERE, V262, DOI 10.1016/j.chemosphere.2020.127828"
"   Gong YY, 2018, WATER RES, V147, P440, DOI 10.1016/j.watres.2018.10.024"
"   Gu SQ, 2019, ENVIRON CHEM LETT, V17, P629, DOI 10.1007/s10311-018-0813-9"
"   Guan X, 2022, SCI TOTAL ENVIRON, V838, DOI 10.1016/j.scitotenv.2022.155693"
"   Guarino F, 2020, CHEMOSPHERE, V251, DOI 10.1016/j.chemosphere.2020.126310"
"   Guo L, 2018, WATER ENVIRON J, V32, P333, DOI 10.1111/wej.12329"
"   Guo P, 2014, ENVIRON SCI POLLUT R, V21, P631, DOI 10.1007/s11356-013-1899-z"
"   Gustave W, 2021, SCI TOTAL ENVIRON, V756, DOI 10.1016/j.scitotenv.2020.143865"
"   Hamed SM, 2022, J ENVIRON CHEM ENG, V10, DOI 10.1016/j.jece.2022.108804"
"   Hamid Y, 2019, CHEMOSPHERE, V214, P259, DOI 10.1016/j.chemosphere.2018.09.113"
"   Han T, 2022, B ENVIRON CONTAM TOX, V108, P1132, DOI 10.1007/s00128-022-03532-4"
"   Han WJ, 2016, J HAZARD MATER, V302, P437, DOI 10.1016/j.jhazmat.2015.09.041"
"   Han X, 2021, CHEMOSPHERE, V264, DOI 10.1016/j.chemosphere.2020.128421"
"   Handa Y, 2015, PLANT CELL PHYSIOL, V56, P1490, DOI 10.1093/pcp/pcv071"
"   Haroon B, 2019, J WATER REUSE DESAL, V9, P47, DOI 10.2166/wrd.2018.008"
"   Hattab N, 2014, ENVIRON SCI POLLUT R, V21, P10307, DOI 10.1007/s11356-014-2938-0"
"   He T, 2023, CHEM ENG J, V462, DOI 10.1016/j.cej.2023.142041"
"   He Y, 2017, CHEMOSPHERE, V184, P235, DOI 10.1016/j.chemosphere.2017.05.177"
"   Hou DY, 2020, NAT REV EARTH ENV, V1, P366, DOI 10.1038/s43017-020-0061-y"
"   Hu BW, 2020, BIOCHAR, V2, P47, DOI 10.1007/s42773-020-00044-4"
"   Huang F, 2013, COLLOID SURFACE B, V107, P11, DOI 10.1016/j.colsurfb.2013.01.062"
"   Huang XX, 2019, INT J ENV RES PUB HE, V16, DOI 10.3390/ijerph16081328"
"   Ibarra-Rodr??guez D, 2017, CHEM ENG J, V323, P19, DOI 10.1016/j.cej.2017.04.087"
"   Ievinsh G, 2022, LIFE-BASEL, V12, DOI 10.3390/life12121959"
"   Irshad S, 2020, J HAZARD MATER, V381, DOI 10.1016/j.jhazmat.2019.120903"
"   Ji ZH, 2022, CHEMOSPHERE, V286, DOI 10.1016/j.chemosphere.2021.131564"
"   Jiang B, 2018, ENVIRON SCI POLLUT R, V25, P31272, DOI 10.1007/s11356-018-3069-9"
"   Jiang CA, 2010, ECOL ENG, V36, P391, DOI 10.1016/j.ecoleng.2009.11.005"
"   Jiang DM, 2022, SEP PURIF TECHNOL, V295, DOI 10.1016/j.seppur.2022.121317"
"   Jiang SJ, 2022, CHEMOSPHERE, V287, DOI 10.1016/j.chemosphere.2021.132082"
"   Jiang SJ, 2021, ENVIRON SCI POLLUT R, V28, P68886, DOI 10.1007/s11356-021-14757-x"
"   Jin Y, 2024, SCI TOTAL ENVIRON, V908, DOI 10.1016/j.scitotenv.2023.168394"
"   Karalija E, 2021, ECOTOX ENVIRON SAFE, V210, DOI 10.1016/j.ecoenv.2020.111882"
"   Keochaiyom B, 2017, J COLLOID INTERF SCI, V505, P824, DOI 10.1016/j.jcis.2017.06.056"
"   Khalid M, 2023, SCI TOTAL ENVIRON, V904, DOI 10.1016/j.scitotenv.2023.167226"
"   Khalid S, 2017, J GEOCHEM EXPLOR, V182, P247, DOI 10.1016/j.gexplo.2016.11.021"
"   Khan I, 2019, INT J ENVIRON SCI TE, V16, P6473, DOI 10.1007/s13762-019-02431-2"
"   Khosravi MJ, 2022, CHEMOSPHERE, V290, DOI 10.1016/j.chemosphere.2021.133335"
"   Kumar M, 2021, J ENVIRON MANAGE, V278, DOI 10.1016/j.jenvman.2020.111555"
"   Kumar V, 2021, ENVIRON SCI POLLUT R, V28, P10375, DOI 10.1007/s11356-020-11491-8"
"   Kuppusamy S, 2017, CHEMOSPHERE, V168, P944, DOI 10.1016/j.chemosphere.2016.10.115"
"   Lebrun M, 2021, SCI TOTAL ENVIRON, V791, DOI 10.1016/j.scitotenv.2021.148400"
"   Lee J, 2014, ECOL ENG, V73, P386, DOI 10.1016/j.ecoleng.2014.09.053"

"   Li FL, 2020, ECOTOX ENVIRON SAFE, V191, DOI 10.1016/j.ecoenv.2020.110185"
"   Li HY, 2017, J ENVIRON SCI, V52, P141, DOI 10.1016/j.jes.2016.04.005"
"   Li HS, 2019, FRONT ENV SCI ENG, V13, DOI 10.1007/s11783-019-1130-7"
"   Li Q, 2022, ENVIRON POLLUT, V298, DOI 10.1016/j.envpol.2022.118855"
"   Li YC, 2024, J ENVIRON CHEM ENG, V12, DOI 10.1016/j.jece.2023.111628"
"   Li Y, 2021, CHEM ENG J, V412, DOI 10.1016/j.cej.2021.128706"
"   Li ZL, 2023, SCI TOTAL ENVIRON, V904, DOI 10.1016/j.scitotenv.2023.166918"
"   Li ZL, 2021, SCI TOTAL ENVIRON, V781, DOI 10.1016/j.scitotenv.2021.146757"
"   Li ZL, 2021, J HAZARD MATER, V407, DOI 10.1016/j.jhazmat.2020.124786"
"   Lin G, 2023, CHEM ENG J, V460, DOI 10.1016/j.cej.2023.141710"
"   Lin JY, 2021, ISCIENCE, V24, DOI 10.1016/j.isci.2021.102518"
"   Lino CA, 2018, DRUG DELIV, V25, P1234, DOI 10.1080/10717544.2018.1474964"
"   Liu BL, 2022, J HAZARD MATER, V436, DOI 10.1016/j.jhazmat.2022.129242"
"   Liu J, 2020, ENVIRON RES, V188, DOI 10.1016/j.envres.2020.109886"
"   Liu LW, 2018, SCI TOTAL ENVIRON, V633, P206, DOI 10.1016/j.scitotenv.2018.03.161"
"   Liu LY, 2019, ACS SUSTAIN CHEM ENG, V7, P22, DOI 10.1021/acssuschemeng.8b04253"
"   Liu SL, 2023, CHEM ENG J, V473, DOI 10.1016/j.cej.2023.145389"
"   Liu ZC, 2020, J HAZARD MATER, V400, DOI 10.1016/j.jhazmat.2020.123138"
"   Lou XY, 2020, MEMBRANES-BASEL, V10, DOI 10.3390/membranes10010019"
"   Lu YY, 2017, ENVIRON SCI POLLUT R, V24, P24409, DOI 10.1007/s11356-017-0065-4"
"   Ma WC, 2021, J HAZARD MATER, V408, DOI 10.1016/j.jhazmat.2020.124809"
"   Ma Y, 2016, J ENVIRON MANAGE, V174, P14, DOI 10.1016/j.jenvman.2016.02.047"
"   Mallampati SR, 2015, J ENVIRON RADIOACTIV, V139, P118, DOI 10.1016/j.jenvrad.2014.10.006"
"   Manoj SR, 2020, J ENVIRON MANAGE, V254, DOI 10.1016/j.jenvman.2019.109779"
"   Mao YF, 2019, NATL SCI REV, V6, P421, DOI 10.1093/nsr/nwz005"
"   Marcon L, 2021, SCI TOTAL ENVIRON, V791, DOI 10.1016/j.scitotenv.2021.148324"
"   Mohamed BA, 2021, CHEMOSPHERE, V271, DOI 10.1016/j.chemosphere.2021.129808"
"   Mohamed BA, 2019, APPL CATAL B-ENVIRON, V253, P226, DOI 10.1016/j.apcatb.2019.04.058"
"   Moreira-Grez B, 2019, FRONT MICROBIOL, V10, DOI 10.3389/fmicb.2019.01617"
"   Mousavi A, 2021, J ENVIRON CHEM ENG, V9, DOI 10.1016/j.jece.2021.105456"
"   Nayeri S, 2023, J CLEAN PROD, V428, DOI 10.1016/j.jclepro.2023.139512"
"   Nekouei RK, 2019, J HAZARD MATER, V371, P389, DOI 10.1016/j.jhazmat.2019.03.013"
"   Niu LJ, 2023, ECOTOX ENVIRON SAFE, V256, DOI 10.1016/j.ecoenv.2023.114882"
"   Niu XY, 2021, FRONT MICROBIOL, V12, DOI 10.3389/fmicb.2021.686812"
"   Noor I, 2022, CHEMOSPHERE, V303, DOI 10.1016/j.chemosphere.2022.135196"
"   Oliva J, 2011, J HAZARD MATER, V194, P312, DOI 10.1016/j.jhazmat.2011.07.104"
"   Ozyigit II, 2021, ENVIRON CHEM LETT, V19, P669, DOI 10.1007/s10311-020-01095-6"
"   Pavithra KG, 2020, ENVIRON CHEM LETT, V18, P1905, DOI 10.1007/s10311-020-01046-1"
"   Petrovic A, 2016, INT J ENVIRON SCI TE, V13, P1761, DOI 10.1007/s13762-016-1015-2"
"   Qiao PW, 2023, SCI TOTAL ENVIRON, V857, DOI 10.1016/j.scitotenv.2022.159119"
"   Qin HQ, 2020, ENVIRON POLLUT, V258, DOI 10.1016/j.envpol.2019.113777"
"   Qu LY, 2018, ENVIRON POLLUT, V237, P639, DOI 10.1016/j.envpol.2018.02.020"
"   Rahman MA, 2011, CHEMOSPHERE, V83, P633, DOI 10.1016/j.chemosphere.2011.02.045"
"   Rajkumar M, 2012, BIOTECHNOL ADV, V30, P1562, DOI 10.1016/j.biotechadv.2012.04.011"
"   Rees F, 2016, CHEMOSPHERE, V142, P48, DOI 10.1016/j.chemosphere.2015.03.068"
"   Reshmy R, 2022, J HAZARD MATER, V424, DOI 10.1016/j.jhazmat.2021.127516"
"   Riaz M, 2021, J HAZARD MATER, V402, DOI 10.1016/j.jhazmat.2020.123919"
"   Saeed T, 2016, CHEM ENG J, V287, P62, DOI 10.1016/j.cej.2015.10.118"
"   Saeedi M, 2019, J ENVIRON MANAGE, V248, DOI 10.1016/j.jenvman.2019.07.029"
"   Samal K, 2019, J ENVIRON MANAGE, V251, DOI 10.1016/j.jenvman.2019.109550"
"   Sandhi A, 2018, ENVIRON POLLUT, V237, P1098, DOI 10.1016/j.envpol.2017.11.038"
"   Sarfaraz Q, 2020, SCI REP-UK, V10, DOI 10.1038/s41598-020-57987-8"
"   Sarma H, 2021, J HAZARD MATER, V414, DOI 10.1016/j.jhazmat.2021.125493"
"   Sarwar N, 2017, CHEMOSPHERE, V171, P710, DOI 10.1016/j.chemosphere.2016.12.116"
"   Sellaoui L, 2021, CHEM ENG J, V420, DOI 10.1016/j.cej.2020.127712"
"   Seo EY, 2013, SCI WORLD J, DOI 10.1155/2013/857670"
"   Shah T, 2023, PLANT PHYSIOL BIOCH, V202, DOI 10.1016/j.plaphy.2023.107916"
"   Shahid M, 2014, SOIL SEDIMENT CONTAM, V23, P389, DOI 10.1080/15320383.2014.831029"
"   Shahjahan M, 2022, CHEMOSPHERE, V300, DOI 10.1016/j.chemosphere.2022.134519"
"   Shang XT, 2024, J HAZARD MATER, V466, DOI 10.1016/j.jhazmat.2024.133684"
"   Sharma P, 2021, BIORESOURCE TECHNOL, V320, DOI 10.1016/j.biortech.2020.124353"
"   Singh R, 2012, B ENVIRON CONTAM TOX, V88, P210, DOI 10.1007/s00128-011-0425-6"
"   Singh S, 2022, ECOL ENG, V175, DOI 10.1016/j.ecoleng.2021.106476"
"   Song PP, 2022, SCI TOTAL ENVIRON, V838, DOI 10.1016/j.scitotenv.2022.156417"
"   Srivastava S, 2021, Microbe mediated remediation of environmental contaminants, V177, P198, DOI DOI 10.1016/B978-0-12-821199-1.00016-X"
"   Stathatou PM, 2022, COMMUN EARTH ENVIRON, V3, DOI 10.1038/s43247-022-00463-0"
"   Sui CM, 2022, SCI TOTAL ENVIRON, V812, DOI 10.1016/j.scitotenv.2021.151473"
"   Suman J, 2018, FRONT PLANT SCI, V9, DOI 10.3389/fpls.2018.01476"
"   Sun YM, 2016, J HAZARD MATER, V304, P543, DOI 10.1016/j.jhazmat.2015.11.006"
"   Sun YY, 2022, FRONT MICROBIOL, V13, DOI 10.3389/fmicb.2022.997732"
"   Sun YJ, 2020, CHEM ENG J, V385, DOI 10.1016/j.cej.2019.123911"
"   Tian SS, 2017, CHINESE J CHEM ENG, V25, P477, DOI 10.1016/j.cjche.2016.09.009"
"   Tovar-S??nchez E, 2023, PLANTS-BASEL, V12, DOI 10.3390/plants12061338"
"   Ai TN, 2018, FRONT PLANT SCI, V9, DOI 10.3389/fpls.2018.01388"
"   Uchimiya M, 2012, J AGR FOOD CHEM, V60, P5035, DOI 10.1021/jf300825n"
"   Ullah A, 2015, ENVIRON EXP BOT, V117, P28, DOI 10.1016/j.envexpbot.2015.05.001"
"   Ullmann A, 2013, J HAZARD MATER, V260, P676, DOI 10.1016/j.jhazmat.2013.06.027"
"   Vaidya BP, 2022, ENVIRON POLLUT, V312, DOI 10.1016/j.envpol.2022.120007"
"   Vardhan KH, 2019, J MOL LIQ, V290, DOI 10.1016/j.molliq.2019.111197"
"   Ventura D, 2021, SCI TOTAL ENVIRON, V754, DOI 10.1016/j.scitotenv.2020.142221"
"   Verbon EH, 2016, TRENDS PLANT SCI, V21, P218, DOI 10.1016/j.tplants.2016.01.013"
"   Vergani L, 2017, SCI TOTAL ENVIRON, V575, P1395, DOI 10.1016/j.scitotenv.2016.09.218"
"   Vinod VTP, 2017, RSC ADV, V7, P13997, DOI 10.1039/c7ra00464h"

"   Vymazal J, 2016, CHEM ENG J, V290, P232, DOI 10.1016/j.cej.2015.12.108"
"   Wan XM, 2022, SCI TOTAL ENVIRON, V841, DOI 10.1016/j.scitotenv.2022.156708"
"   Wang JJ, 2021, SEP PURIF TECHNOL, V268, DOI 10.1016/j.seppur.2021.118689"
"   Wang T, 2019, J ENVIRON MANAGE, V231, P679, DOI 10.1016/j.jenvman.2018.10.085"
"   Wang XH, 2021, CHEMOSPHERE, V279, DOI 10.1016/j.chemosphere.2021.130504"
"   Wang X, 2020, ENVIRON SCI TECHNOL, V54, P15731, DOI 10.1021/acs.est.0c03114"
"   Wang YT, 2020, SCI TOTAL ENVIRON, V701, DOI 10.1016/j.scitotenv.2019.134736"
"   Wang YT, 2020, SCI TOTAL ENVIRON, V720, DOI 10.1016/j.scitotenv.2020.137666"
"   Wang YC, 2021, CHEMOSPHERE, V265, DOI 10.1016/j.chemosphere.2020.129071"
"   Wang Z, 2022, J CLEAN PROD, V379, DOI 10.1016/j.jclepro.2022.134043"
"   Wiangkham N, 2018, CHEMOSPHERE, V201, P511, DOI 10.1016/j.chemosphere.2018.03.039"
"   Woraharn S, 2021, WATER AIR SOIL POLL, V232, DOI 10.1007/s11270-021-05156-6"
"   Wu H, 2015, ENVIRON SCI POLLUT R, V22, P13038, DOI 10.1007/s11356-015-4547-y"
"   Wu JW, 2019, BIORESOURCE TECHNOL, V291, DOI 10.1016/j.biortech.2019.121859"
"   Wu YJ, 2020, J HAZARD MATER, V395, DOI 10.1016/j.jhazmat.2020.122661"
"   Wuana R.A., 2011, ISRN Ecology, DOI [10.5402/2011/402647, DOI 10.5402/2011/402647]"
"   Xiang MT, 2021, ENVIRON POLLUT, V278, DOI 10.1016/j.envpol.2021.116911"
"   Xiao J, 2024, ENVIRON POLLUT, V341, DOI 10.1016/j.envpol.2023.123019"
"   Xing JF, 2016, SPRINGERPLUS, V5, DOI 10.1186/s40064-016-2492-y"
"   Xu CB, 2019, SCI TOTAL ENVIRON, V686, P476, DOI 10.1016/j.scitotenv.2019.05.330"
"   Xu DM, 2021, J CLEAN PROD, V321, DOI 10.1016/j.jclepro.2021.128730"
"   Xu L, 2021, J HAZARD MATER, V407, DOI 10.1016/j.jhazmat.2020.124764"
"   Xue SG, 2023, CHEM ENG J, V473, DOI 10.1016/j.cej.2023.145499"
"   Yadav KK, 2018, ECOL ENG, V120, P274, DOI 10.1016/j.ecoleng.2018.05.039"
"   Yan A, 2020, FRONT PLANT SCI, V11, DOI 10.3389/fpls.2020.00359"
"   Yang GL, 2021, J HAZARD MATER, V419, DOI 10.1016/j.jhazmat.2021.126410"
"   Yang Q, 2021, CHEMOSPHERE, V269, DOI 10.1016/j.chemosphere.2020.129373"
"   Yang X, 2019, J HAZARD MATER, V365, P178, DOI 10.1016/j.jhazmat.2018.10.047"
"   Yang ZM, 2016, ECOTOX ENVIRON SAFE, V132, P224, DOI 10.1016/j.ecoenv.2016.06.008"
"   Yang Z, 2022, SCI TOTAL ENVIRON, V809, DOI 10.1016/j.scitotenv.2021.151099"
"   Ye M, 2014, J ENVIRON MANAGE, V141, P161, DOI 10.1016/j.jenvman.2014.03.025"
"   Yin K, 2019, CHEM ENG J, V360, P1553, DOI [10.1016/j.cej.2018.10.226, 10.1016/j.jhazmat.2024.134410]"
"   Yin K, 2016, WATER RES, V103, P383, DOI 10.1016/j.watres.2016.07.053"
"   Yu GL, 2022, SCI TOTAL ENVIRON, V821, DOI 10.1016/j.scitotenv.2022.153516"
"   Yu GL, 2021, WORLD J MICROB BIOT, V37, DOI 10.1007/s11274-021-03123-1"
"   Yu SH, 2020, SPECTROCHIM ACTA A, V229, DOI 10.1016/j.saa.2019.118007"
"   Zakari S, 2021, ENVIRON POLLUT, V288, DOI 10.1016/j.envpol.2021.117820"
"   Zeng JQ, 2023, CHEM ENG J, V454, DOI 10.1016/j.cej.2022.140307"
"   Zeng WZ, 2021, J HAZARD MATER, V408, DOI 10.1016/j.jhazmat.2020.124868"
"   Zhang AL, 2020, J ENVIRON CHEM ENG, V8, DOI 10.1016/j.jece.2020.104196"
"   Zhang HX, 2024, J HAZARD MATER, V465, DOI 10.1016/j.jhazmat.2023.133266"
"   Zhang HZ, 2016, ECOTOX ENVIRON SAFE, V133, P57, DOI 10.1016/j.ecoenv.2016.05.036"
"   Zhang L, 2024, SEP PURIF TECHNOL, V342, DOI 10.1016/j.seppur.2024.126981"
"   Zhang MY, 2019, GEODERMA, V348, P115, DOI 10.1016/j.geoderma.2019.04.031"
"   Zhang RH, 2022, ECOTOX ENVIRON SAFE, V248, DOI 10.1016/j.ecoenv.2022.114316"
"   Zhang X, 2015, CHEMOSPHERE, V119, P224, DOI 10.1016/j.chemosphere.2014.06.042"
"   Zhang Y, 2016, NAT COMMUN, V7, DOI 10.1038/ncomms12617"
"   Zhao AQ, 2019, ENVIRON SCI POLLUT R, V26, P34818, DOI 10.1007/s11356-019-06563-3"
"   Zhao CZ, 2014, PLOS ONE, V9, DOI 10.1371/journal.pone.0103771"
"   Zhao F, 2023, SCI TOTAL ENVIRON, V896, DOI 10.1016/j.scitotenv.2023.165232"
"   Zhao K, 2023, ENVIRON POLLUT, V334, DOI 10.1016/j.envpol.2023.122110"
"   Zhao LZ, 2019, WATER AIR SOIL POLL, V230, DOI 10.1007/s11270-019-4166-1"
"   Zhao ML, 2021, PLANT CELL ENVIRON, V44, P613, DOI 10.1111/pce.13928"
"   Zhao X, 2022, J CLEAN PROD, V376, DOI 10.1016/j.jclepro.2022.134253"
"   Zhou JJ, 2022, J ENVIRON CHEM ENG, V10, DOI 10.1016/j.jece.2022.108360"
"   Zhu F, 2016, CHEM ENG J, V302, P663, DOI 10.1016/j.cej.2016.05.072"
"   Zhu YC, 2024, ENVIRON CHEM LETT, V22, P355, DOI 10.1007/s10311-023-01663-6"
"   Zhu YX, 2023, SCI TOTAL ENVIRON, V905, DOI 10.1016/j.scitotenv.2023.167398"
"   Zhu Y, 2019, ENVIRON RES, V174, P54, DOI 10.1016/j.envres.2019.04.014"
"   Zou WT, 2023, RESTOR ECOL, V31, DOI 10.1111/rec.13702"
NR 245
TC 68
Z9 80
U1 201
U2 576
PU ELSEVIER
PI AMSTERDAM
"PA RADARWEG 29, 1043 NX AMSTERDAM, NETHERLANDS"
SN 0048-9697
EI 1879-1026
J9 SCI TOTAL ENVIRON
JI Sci. Total Environ.
PD OCT 20
PY 2024
VL 948
AR 174237
DI 10.1016/j.scitotenv.2024.174237
EA JUL 2024
PG 22
WC Environmental Sciences
WE Science Citation Index Expanded (SCI-EXPANDED)
SC Environmental Sciences & Ecology
GA A3E6F
UT WOS:001281397700001

PM 38942300
DA 2026-04-06
ER

PT J
"AU Zhai, XQ"
"   Li, ZW"
"   Huang, B"
"   Luo, NL"
"   Huang, M"
"   Zhang, Q"
"   Zeng, GM"
"AF Zhai, Xiuqing"
"   Li, Zhongwu"
"   Huang, Bin"
"   Luo, Ninglin"
"   Huang, Mei"
"   Zhang, Qiu"
"   Zeng, Guangming"
TI Remediation of multiple heavy metal-contaminated soil through the
   combination of soil washing and in situ immobilization
SO SCIENCE OF THE TOTAL ENVIRONMENT
LA English
DT Article
DE Soil washing; In situ immobilization; Combined remediation; Heavy
   metals; Soil enzyme activity
ID ORGANIC-MATTER; FERRIC-CHLORIDE; SIZE FRACTIONS; PADDY SOILS; CD;
   ADSORPTION; CU; ZN; CADMIUM; BIOCHAR
"AB The remediation of heavy metal-contaminated soils is a great challenge for global environmental sciences and engineering. To control the ecological risks of heavy metal-contaminated soil more effectively, the present study focused on the combination of soil washing (with FeCl3) and in situ immobilization (with lime, biochar, and black carbon). The results showed that the removal rate of Cd, Pb, Zn, and Cu was 62.9%, 52.1%, 30.0%, and 16.7%, respectively, when washed with FeCl3. After the combined remediation (immobilization with 1% (w/w) lime), the contaminated soils showed 36.5%, 73.6%, 70.9%, and 53.4% reductions in the bioavailability of Cd, Cu, Pb, and Zn (extracted with 0.11 Macetic acid), respectively, than those of the soils washed with FeCl3 only. However, the immobilization with 1% (w/w) biochar or 1% (w/w) carbon black after washing exhibited low effects on stabilizing the metals. The differences in effects between the immobilization with lime, biochar, and carbon black indicated that the soil pH had a significant influence on the lability of heavy metals during the combined remediation process. The activity of the soil enzymes (urease, sucrase, and catalase) showed that the addition of all the materials, including lime, biochar, and carbon black, exhibited positive effects on microbial remediation after soil washing. Furthermore, lime was the most effective material, indicating that low soil pH and high acid-soluble metal concentrations might restrain the activity of soil enzymes. Soil pH and nutrition were the major considerations for microbial remediation during the combined remediation. These findings suggest that the combination of soil washing and in situ immobilization is an effective method to amend the soils contaminated with multiple heavy metals. (C) 2018 Elsevier B.V. All rights reserved."
"C1 [Zhai, Xiuqing; Li, Zhongwu; Luo, Ninglin; Huang, Mei; Zhang, Qiu; Zeng, Guangming] Hunan Univ, Coll Environm Sci & Engn, Changsha 410082, Hunan, Peoples R China."
"   [Zhai, Xiuqing; Li, Zhongwu; Luo, Ninglin; Huang, Mei; Zhang, Qiu; Zeng, Guangming] Hunan Univ, Minist Educ, Key Lab Environm Biol & Pollut Control, Changsha 410082, Hunan, Peoples R China."
"   [Huang, Bin] Guangdong Inst Ecoenvironm Sci & Technol, Guangdong Key Lab Integrated Agroenvironm Pollut, Guangzhou 510650, Guangdong, Peoples R China."
C3 Hunan University; Hunan University; Guangdong Academy of Sciences;
"   Institute of Eco-environmental & Soil Sciences, Guangdong Academy of"
   Sciences
"RP Li, ZW (corresponding author), Hunan Univ, Coll Environm Sci & Engn, Changsha 410082, Hunan, Peoples R China."
EM lizw@hnu.edu.cn
"RI guangming, zeng/AHD-0360-2022; Li, Zhongwu/AAF-9530-2020; Huang,"
   Mei/L-5322-2019
"OI Huang, Mei/0000-0002-3533-0996; "
FU National Natural Science Foundation of China [51521006]; Key R&D Program
   of Science and Technology of Hunan Province in China [2017SK2351]
FX This work was financially supported by the National Natural Science
   Foundation of China (No. 51521006) and Key R&D Program of Science and
   Technology of Hunan Province in China (No. 2017SK2351).
"CR Ai C, 2015, BIOL FERT SOILS, V51, P465, DOI 10.1007/s00374-015-0994-3"
"   [Anonymous], 1995, GB15618 MIN ENV PROT"
"   BALL DF, 1964, J SOIL SCI, V15, P84, DOI 10.1111/j.1365-2389.1964.tb00247.x"
"   BARROW NJ, 1981, AUST J SOIL RES, V19, P309, DOI 10.1071/SR9810309"
"   Becker M, 2005, J PLANT NUTR SOIL SC, V168, P558, DOI 10.1002/jpln.200520504"
"   Beiyuan JZ, 2017, CHEMOSPHERE, V166, P489, DOI 10.1016/j.chemosphere.2016.09.110"
"   Bolan N, 2014, J HAZARD MATER, V266, P141, DOI 10.1016/j.jhazmat.2013.12.018"
"   Cao XD, 2008, ENVIRON POLLUT, V152, P184, DOI 10.1016/j.envpol.2007.05.008"
"   Chaney R, 1973, CROP FOOD CHAIN EFFE"
"   Cui HB, 2016, ENVIRON SCI POLLUT R, V23, P10808, DOI 10.1007/s11356-016-6271-7"
"   Cui HBA, 2016, J SOIL SEDIMENT, V16, P1498, DOI 10.1007/s11368-015-1317-x"
"   Dermont G, 2008, J HAZARD MATER, V152, P1, DOI 10.1016/j.jhazmat.2007.10.043"
"   Derome J, 2000, ENVIRON POLLUT, V107, P79, DOI 10.1016/S0269-7491(99)00132-3"
"   DONNET JB, 1992, KAUT GUMMI KUNSTST, V45, P459"
"   Echeverr??a JC, 1999, EUR J SOIL SCI, V50, P497, DOI 10.1046/j.1365-2389.1999.00261.x"
"   Fontaine S, 2007, NATURE, V450, P277, DOI 10.1038/nature06275"
"   Fran??a FCSS, 2017, FOOD CHEM, V215, P171, DOI 10.1016/j.foodchem.2016.07.168"
"   Garforth JM, 2016, CHEMOSPHERE, V155, P534, DOI 10.1016/j.chemosphere.2016.04.096"

"   Guo XF, 2016, CHEMOSPHERE, V147, P412, DOI 10.1016/j.chemosphere.2015.12.087"
"   Haidouti C, 1997, SCI TOTAL ENVIRON, V208, P105, DOI 10.1016/S0048-9697(97)00284-2"
"   He XS, 2014, J HAZARD MATER, V268, P256, DOI 10.1016/j.jhazmat.2014.01.030"
"   Hou D., 2017, LAND DEGRAD DEV, DOI [10.1002/ldr.2748/full, DOI 10.1002/LDR.2748/FULL]"
"   Hou DY, 2017, ENVIRON POLLUT, V231, P1188, DOI 10.1016/j.envpol.2017.07.021"
"   Hou DY, 2017, LAND DEGRAD DEV, V28, P2315, DOI 10.1002/ldr.2741"
"   Hou D, 2015, P I CIVIL ENG-ENG SU, V168, P61, DOI 10.1680/ensu.14.00025"
"   Hou DY, 2014, J CLEAN PROD, V83, P87, DOI 10.1016/j.jclepro.2014.07.062"
"   Hu B, 2014, ECOTOX ENVIRON SAFE, V110, P41, DOI 10.1016/j.ecoenv.2014.08.007"
"   Huang B, 2017, ENVIRON SCI POLLUT R, V24, P23939, DOI 10.1007/s11356-017-0012-4"
"   Huang B, 2014, J HAZARD MATER, V264, P176, DOI 10.1016/j.jhazmat.2013.10.074"
"   Huang DL, 2016, WATER RES, V106, P15, DOI 10.1016/j.watres.2016.09.050"
"   Huang S, 2009, SCI CHINA SER E, V52, P3293, DOI 10.1007/s11431-009-0348-1"
"   J??rup L, 2003, BRIT MED BULL, V68, P167, DOI 10.1093/bmb/ldg032"
"   Jiang JP, 2010, EUR J SOIL BIOL, V46, P18, DOI 10.1016/j.ejsobi.2009.10.001"
"   Jiang XJ, 2003, CHEMOSPHERE, V50, P813, DOI 10.1016/S0045-6535(02)00224-2"
"   K??sel K, 2002, ENVIRON MICROBIOL, V4, P414, DOI 10.1046/j.1462-2920.2002.00314.x"
"   Labanowski J, 2008, ENVIRON POLLUT, V152, P693, DOI 10.1016/j.envpol.2007.06.054"
"   Lee CH, 2010, SOIL SCI, V175, P552, DOI 10.1097/SS.0b013e3181fba6d1"
"   Lee SH, 2009, CHEMOSPHERE, V77, P1069, DOI 10.1016/j.chemosphere.2009.08.056"
"   Li ZW, 2016, T NONFERR METAL SOC, V26, P536, DOI 10.1016/S1003-6326(16)64142-0"
"   Li ZG, 2011, APPL GEOCHEM, V26, P160, DOI 10.1016/j.apgeochem.2010.11.014"
"   Liu J, 2009, PLANT SOIL, V322, P187, DOI 10.1007/s11104-009-9907-2"
"   Liu XL, 2013, PROCEEDINGS OF THE 35TH IAHR WORLD CONGRESS, VOLS I AND II, P2264"
"   Lombi E, 2003, ENVIRON SCI TECHNOL, V37, P979, DOI 10.1021/es026083w"
"   Lu HL, 2012, WATER RES, V46, P854, DOI 10.1016/j.watres.2011.11.058"
"   Makino T, 2008, CHEMOSPHERE, V70, P1035, DOI 10.1016/j.chemosphere.2007.07.080"
"   Makino T, 2006, ENVIRON POLLUT, V144, P2, DOI 10.1016/j.envpol.2006.01.017"
"   Makino T, 2016, GEODERMA, V270, P3, DOI 10.1016/j.geoderma.2016.01.006"
"   Mallampati SR, 2012, CHEMOSPHERE, V89, P717, DOI 10.1016/j.chemosphere.2012.06.030"
"   McBride M.B., 1994, ENV CHEM SOILS"
"   Mengel K, 1982, Principles of plant nutrition."
"   Mulligan CN, 2001, ENG GEOL, V60, P193, DOI 10.1016/S0013-7952(00)00101-0"
"   O'Connor D, 2018, SCI TOTAL ENVIRON, V619, P815, DOI 10.1016/j.scitotenv.2017.11.132"
"   Pan YY, 2014, J SOIL SEDIMENT, V14, P1713, DOI 10.1007/s11368-014-0919-z"
"   Park JH, 2013, WATER AIR SOIL POLL, V224, DOI 10.1007/s11270-013-1711-1"
"   Porter SK, 2004, CRIT REV ENV SCI TEC, V34, P495, DOI 10.1080/10643380490492412"
"   Rao MA, 2014, CHEMOSPHERE, V107, P145, DOI 10.1016/j.chemosphere.2013.12.059"
"   Shaheen SM, 2013, ADV COLLOID INTERFAC, V201, P43, DOI 10.1016/j.cis.2013.10.005"
"   Shen ZT, 2018, SCI TOTAL ENVIRON, V619, P185, DOI 10.1016/j.scitotenv.2017.11.038"
"   Songyin G., 1984, Acta Pedologica Sinica, V21, P368"
"   Tan WN, 2011, PEDOSPHERE, V21, P223, DOI 10.1016/S1002-0160(11)60121-5"
"   Tang JC, 2013, J BIOSCI BIOENG, V116, P653, DOI 10.1016/j.jbiosc.2013.05.035"
"   Uchimiya M, 2011, J AGR FOOD CHEM, V59, P2501, DOI 10.1021/jf104206c"
"   Van Nevel L, 2007, ENVIRON POLLUT, V150, P34, DOI 10.1016/j.envpol.2007.05.024"
"   Wahla IH, 2008, ENVIRON POLLUT, V155, P271, DOI 10.1016/j.envpol.2007.11.020"
"   Wang Y, 2015, J CENT SOUTH UNIV, V22, P1269, DOI 10.1007/s11771-015-2643-2"
"   Wei M, 2016, CHEMOSPHERE, V156, P252, DOI 10.1016/j.chemosphere.2016.04.106"
"   Xu HJ, 2014, ENVIRON SCI TECHNOL, V48, P9391, DOI 10.1021/es5021058"
"   [????????? Yang Jinyan], 2005, [????????????????????????, Journal of Agro-environment science], V24, P469"
"   Zhang Q, 2017, ENVIRON SCI POLLUT R, V24, P2734, DOI 10.1007/s11356-016-7853-0"
"   Zhang XK, 2013, ENVIRON SCI POLLUT R, V20, P8472, DOI 10.1007/s11356-013-1659-0"
"   Zhao FJ, 2015, ENVIRON SCI TECHNOL, V49, P750, DOI 10.1021/es5047099"
"   Zhou DM, 2010, J HAZARD MATER, V174, P34, DOI 10.1016/j.jhazmat.2009.09.012"
"   Zupanc V, 2014, ENVIRON POLLUT, V186, P56, DOI 10.1016/j.envpol.2013.11.027"
NR 73
TC 225
Z9 273
U1 20
U2 601
PU ELSEVIER
PI AMSTERDAM
"PA RADARWEG 29, 1043 NX AMSTERDAM, NETHERLANDS"
SN 0048-9697
EI 1879-1026
J9 SCI TOTAL ENVIRON
JI Sci. Total Environ.
PD SEP 1
PY 2018
VL 635
BP 92
EP 99
DI 10.1016/j.scitotenv.2018.04.119
PG 8
WC Environmental Sciences
WE Science Citation Index Expanded (SCI-EXPANDED)
SC Environmental Sciences & Ecology
GA GK8RI
UT WOS:000436494400011
PM 29660731
DA 2026-04-06
ER

PT J
"AU Tian, LP"
"   Zhu, HY"
"   Hao, Y"
"   Tong, Y"
"   Chang, ZF"
"   Yang, D"
"   Du, W"
"   Yu, YJ"
"   Pan, B"
"AF Tian, Luping"
"   Zhu, Huaying"
"   Hao, Yi"
"   Tong, Yu"
"   Chang, Zhaofeng"
"   Yang, Dong"
"   Du, Wei"
"   Yu, Yunjiang"
"   Pan, Bo"
TI Environmental aging enhanced the ability of biochar to remediate heavy
   metal-contaminated soil through combined adsorption and microbial
   community regulation: A mechanistic analysis
SO INDUSTRIAL CROPS AND PRODUCTS
LA English
DT Article
DE Heavy metals; Environmental aging; Biochar-mineral complexes;
   Immobilization; Microbial communities
ID GEN. NOV.; CD
"AB The remediation of heavy metal-contaminated soils via biochar involves both adsorption and microbial community regulation, yet the impact of environmental aging on this synergistic mechanism remains unclear. This study analyzed the aforementioned mechanisms through biochar-soil co-cultivation, batch adsorption experiments and 16S rRNA gene sequencing. The results revealed that the initial introduction of biochar did not significantly enhance heavy metal immobilization. After one year of natural aging, however, the efficacy of biochar in immobilizing heavy metals was closely associated with their initial residual fractions. When the inherent residual fraction was high e.g., Cu (> 94 %), biochar amendment induced negligible change in metal speciation. In contrast, in soils with low residual fractions, a 5 % biochar addition promoted the transformation of heavy metals into residual forms, resulting in maximum increases of 3.2 % for Pb and 7.2 % for Cd. Moreover, the stable fractions of heavy metals in biochar-remediated soils increased slightly with extended incubation time. After natural aging for one year, the content of mineral-related fractions increased significantly, substantially enhancing the adsorption (e.g., K-d increase(d) to 499, 607 and 2114 mg/kg for Cu2 +, Cd2+ and Pb2+ with 5 % biochar addition after 1-year incubation) of heavy metals on biochar-remediated soil. Additionally, after natural aging, biochar amendment enriched key metal-immobilizing microbial genera (e.g., Gemmatimonas, Sphingomonas, and Acidiferrimicrobium), while reducing the abundance of metal-mobilizing Kouleothrix, which exhibited dose-dependent. This study demonstrated environmental aging enhanced the ability of biochar to remediate heavy metal-contaminated soil through combined adsorption and microbial community regulation."
"C1 [Tian, Luping; Zhu, Huaying; Tong, Yu; Chang, Zhaofeng; Du, Wei; Pan, Bo] Kunming Univ Sci & Technol, Fac Environm Sci & Engn, Yunnan Prov Key Lab Soil Carbon Sequestrat & Pollu, Kunming 650500, Peoples R China."
"   [Tian, Luping; Zhu, Huaying; Tong, Yu; Chang, Zhaofeng; Du, Wei; Pan, Bo] Yunnan Int Joint Lab Emiss Reduct & Carbon Sequest, Kunming 650500, Yunnan, Peoples R China."
"   [Hao, Yi] Guangdong Urban Rural Planning & Design Res Inst T, Guangzhou 510290, Guangdong, Peoples R China."
"   [Yu, Yunjiang] Shanghai Jiao Tong Univ, Sch Environm Sci & Engn, Shanghai 200240, Peoples R China."
"   [Yang, Dong] Arable Soil Qual & Fertilizer Adm Bur Zhejiang Pro, Hangzhou 310020, Peoples R China."
C3 Kunming University of Science & Technology; Shanghai Jiao Tong
   University
"RP Chang, ZF (corresponding author), Kunming Univ Sci & Technol, Fac Environm Sci & Engn, Yunnan Prov Key Lab Soil Carbon Sequestrat & Pollu, Kunming 650500, Peoples R China."
EM Changzhaofeng545@163.com
"FU National Natural Science Foundation of China [42130711, 42567071];"
   Yunnan Provincial Department of Education Science Research Fund Project
   [2024J0059]; Yunnan Fundamental Research Projects [202401AU070185];
   Natural Science Foundation of KUST [KKZ3202422049]; Zhejiang Province
"   ""Three Rural Nine Strategies"" Science and Technol-ogy Cooperation"
   Program [2024SNJF068]
FX <BOLD>Funding</BOLD> This work was supported by National Natural Science
"   Foundation of China (grant no. 42130711, 42567071) , Yunnan Provincial"
   Department of Education Science Research Fund Project (grant no.
"   2024J0059) , Yunnan Fundamental Research Projects (grant no."
"   202401AU070185) , Natural Science Foundation of KUST (grant no."
"   KKZ3202422049) , and Zhejiang Province ""Three Rural Nine Strategies"""
   Science and Technol-ogy Cooperation Program (grant no. 2024SNJF068) .
"CR Abhishek K, 2022, SCI TOTAL ENVIRON, V853, DOI 10.1016/j.scitotenv.2022.158562"
"   Bandara T, 2020, CHEMOSPHERE, V254, DOI 10.1016/j.chemosphere.2020.126745"
"   Bradl HB, 2004, J COLLOID INTERF SCI, V277, P1, DOI 10.1016/j.jcis.2004.04.005"
"   Chang ZF, 2022, CHEMOSPHERE, V301, DOI 10.1016/j.chemosphere.2022.134422"
"   Chen H, 2009, APPL CLAY SCI, V46, P102, DOI 10.1016/j.clay.2009.07.006"
"   Chen XK, 2024, SCI TOTAL ENVIRON, V912, DOI 10.1016/j.scitotenv.2023.169384"
"   Chu G, 2019, J HAZARD MATER, V373, P106, DOI 10.1016/j.jhazmat.2019.03.078"
"   Cui ZW, 2024, AGRICULTURE-BASEL, V14, DOI 10.3390/agriculture14091631"
"   Dermawan D, 2025, MINER ENG, V232, DOI 10.1016/j.mineng.2025.109575"
"   Fan JP, 2020, SCI TOTAL ENVIRON, V713, DOI 10.1016/j.scitotenv.2020.136663"
"   Fan QY, 2018, CHEMOSPHERE, V207, P33, DOI 10.1016/j.chemosphere.2018.05.044"
"   Fathollahzadeh H, 2014, ENVIRON SCI POLLUT R, V21, P2455, DOI 10.1007/s11356-013-2173-0"

"   Feng LK, 2024, CHEM ENG J, V488, DOI 10.1016/j.cej.2024.150868"
"   Gao YN, 2022, J ENVIRON MANAGE, V313, DOI 10.1016/j.jenvman.2022.114973"
"   Gonz??lez D, 2020, INT J SYST EVOL MICR, V70, P3348, DOI 10.1099/ijsem.0.004179"
"   Hao SF, 2022, J HAZARD MATER, V424, DOI 10.1016/j.jhazmat.2021.127720"
"   Hug LA, 2013, MICROBIOME, V1, DOI 10.1186/2049-2618-1-22"
"   Jin HM, 2014, BIORESOURCE TECHNOL, V169, P622, DOI 10.1016/j.biortech.2014.06.103"
"   Khan AL, 2016, ENVIRON TOXICOL, V31, P887, DOI 10.1002/tox.22100"
"   Khan S, 2015, J HAZARD MATER, V300, P243, DOI 10.1016/j.jhazmat.2015.06.050"
"   Kim HB, 2025, J HAZARD MATER, V495, DOI 10.1016/j.jhazmat.2025.139123"
"   Kumar A, 2018, SCI TOTAL ENVIRON, V626, P953, DOI 10.1016/j.scitotenv.2018.01.157"
"   Li GR, 2020, ENVIRON POLLUT, V265, DOI 10.1016/j.envpol.2020.114887"
"   Li HY, 2016, J HAZARD MATER, V304, P40, DOI 10.1016/j.jhazmat.2015.10.048"
"   Li JY, 2024, J HAZARD MATER, V469, DOI 10.1016/j.jhazmat.2024.133937"
"   Li X, 2022, BIOCHAR, V4, DOI 10.1007/s42773-022-00132-7"
"   Li XZ, 2025, IND CROP PROD, V225, DOI 10.1016/j.indcrop.2025.120464"
"   Liu CW, 2024, SCI REP-UK, V14, DOI 10.1038/s41598-024-72771-8"
"   Lu YY, 2025, HORTICULTURAE, V11, DOI 10.3390/horticulturae11070800"
"   Meng ZW, 2024, J HAZARD MATER, V469, DOI 10.1016/j.jhazmat.2024.133938"
"   Mohamed BA, 2017, ENVIRON POLLUT, V230, P329, DOI 10.1016/j.envpol.2017.06.075"
"   Nazari S, 2019, J ENVIRON CHEM ENG, V7, DOI 10.1016/j.jece.2019.103064"
"   Ng KH, 2021, J CLEAN PROD, V313, DOI 10.1016/j.jclepro.2021.127726"
"   Nie TH, 2021, J HAZARD MATER, V408, DOI 10.1016/j.jhazmat.2020.124850"
"   Qayyum MF, 2014, PLANT SOIL ENVIRON, V60, P337, DOI 10.17221/768/2013-PSE"
"   Qian LB, 2015, J SOIL SEDIMENT, V15, P1130, DOI 10.1007/s11368-015-1073-y"
"   Qian LB, 2014, J AGR FOOD CHEM, V62, P373, DOI 10.1021/jf404624h"
"   Si TR, 2024, J ENVIRON MANAGE, V370, DOI 10.1016/j.jenvman.2024.122848"
"   Gonzaga MIS, 2020, CHEMOSPHERE, V240, DOI 10.1016/j.chemosphere.2019.124828"
"   Tu C, 2020, ENVIRON INT, V137, DOI 10.1016/j.envint.2020.105576"
"   Waigi MG, 2017, TRENDS BIOTECHNOL, V35, P883, DOI 10.1016/j.tibtech.2017.06.014"
"   Wang BY, 2022, BIOCHAR, V4, DOI 10.1007/s42773-022-00159-w"
"   Wang GL, 2023, J HAZARD MATER, V443, DOI 10.1016/j.jhazmat.2022.130148"
"   Wang YF, 2024, J HAZARD MATER, V477, DOI 10.1016/j.jhazmat.2024.135356"
"   Wang ZP, 2021, J HAZARD MATER, V419, DOI 10.1016/j.jhazmat.2021.126468"
"   Xu KL, 2024, J ENVIRON CHEM ENG, V12, DOI 10.1016/j.jece.2024.114357"
"   Xu Z., 2018, Contrasting impacts of preand postapplication aging of biochar on the immobilization of Cd in contaminated soils, V242, P1362, DOI [10.1016/j.envpol.2018.08.012, DOI 10.1016/J.ENVPOL.2018.08.012]"
"   Yadav N, 2023, HYG ENVIRON HLTH ADV, V7, DOI 10.1016/j.heha.2023.100071"
"   Yang C, 2023, ENVIRON GEOCHEM HLTH, V45, P1311, DOI 10.1007/s10653-022-01343-4"
"   Yi QQ, 2020, SCI TOTAL ENVIRON, V721, DOI 10.1016/j.scitotenv.2020.137670"
"   Zhang H, 2003, INT J SYST EVOL MICR, V53, P1155, DOI 10.1099/ijs.0.02520-0"
"   Zhang RH, 2022, ECOTOX ENVIRON SAFE, V248, DOI 10.1016/j.ecoenv.2022.114316"
"   Zhao ZD, 2019, ENVIRON POLLUT, V245, P208, DOI 10.1016/j.envpol.2018.11.013"
"   Zheng XM, 2022, J HAZARD MATER, V438, DOI 10.1016/j.jhazmat.2022.129557"
"   Zuluaga J, 2011, BIOL TRACE ELEM RES, V144, P1302, DOI 10.1007/s12011-011-9140-8"
NR 55
TC 1
Z9 1
U1 24
U2 24
PU ELSEVIER
PI AMSTERDAM
"PA RADARWEG 29, 1043 NX AMSTERDAM, NETHERLANDS"
SN 0926-6690
EI 1872-633X
J9 IND CROP PROD
JI Ind. Crop. Prod.
PD JAN
PY 2026
VL 239
AR 122477
DI 10.1016/j.indcrop.2025.122477
EA DEC 2025
PG 13
WC Agricultural Engineering; Agronomy
WE Science Citation Index Expanded (SCI-EXPANDED)
SC Agriculture
GA Y2381
UT WOS:001641408200001
"OA Green Submitted, gold"
DA 2026-04-06
ER

PT J
"AU Lal, S"
"   Ratna, S"
"   Ben Said, O"
"   Kumar, R"
"AF Lal, Shatrohan"
"   Ratna, Sheel"
"   Ben Said, Olfa"
"   Kumar, Rajesh"
TI Biosurfactant and exopolysaccharide-assisted rhizobacterial technique
   for the remediation of heavy metal contaminated soil: An advancement in
   metal phytoremediation technology

SO ENVIRONMENTAL TECHNOLOGY & INNOVATION
LA English
DT Article
DE Biosurfactants; Exopolysaccharide; Biosorption; Phytoremediation; Heavy
metals; PGPR
ID EXTRACELLULAR POLYMERIC SUBSTANCES; INDUCED OXIDATIVE STRESS; GROWTH
PROMOTING RHIZOBACTERIA; POTENTIALLY TOXIC ELEMENTS; PLANT-GROWTH;
PSEUDOMONAS SP; PETROLEUM-HYDROCARBONS; HEALTH-RISK; DNA-DAMAGE;
RHAMNOLIPID BIOSURFACTANTS
"AB Biosurfactant and exopolysaccharide producing plant growth promoting rhizobacteria can be a best tool for increasing the efficiency of plant assisted remediation of heavy metal contaminated soil; because they can thrive in the stressful environment in a luxuriant way, support plant growth and also contribute to the remediation process. Heavy metals are a noteworthy environmental pollutant and are regarded as biosphere hazard. Numerous chemically based techniques are used to enhance the efficacy of phytoremediation; however, most of them are ecotoxic, highly expensive and lead to incomplete removal of pollutants. To mitigate these technical inherent and to ensure complete removal of toxic heavy metals from soil, an advanced biological tool is the use of biosurfactant and exopolysaccharide-producing rhizobacteria. This can be a promising technique, that has been operative in nature and is cost effective, eco-friendly, efficient and having socioeconomic importance over other conventional remediation techniques as well as sustainable for the environment. The present article critically reviews the potential role of rootassociated metal resistant, exopolysaccharide and biosurfactant producing rhizospheric bacteria to remediate heavy metal contaminated soil and highlight some insight mechanisms for exploitation of plants and associated rhizobacterial interactions for enhancing heavy metal remediation. (C) 2018 Published by Elsevier B.V."
"C1 [Lal, Shatrohan; Ratna, Sheel; Ben Said, Olfa; Kumar, Rajesh] Babasaheb Bhimrao Ambedkar A Cent Univ, Sch Environm Sci, Dept Environm Microbiol, Rhizosphere Biol Lab, Raebareli Rd, Lucknow 226025, Uttar Pradesh, India."
" [Ben Said, Olfa] Univ Carthage, Fac Sci Bizerte, Coastal Ecol & Ecotoxicol Unit, Lab Environm Biomonitoring, Zarzouna 7021, Tunisia."
C3 Babasaheb Bhimrao Ambedkar University; Universite de Carthage
"RP Kumar, R (corresponding author), Babasaheb Bhimrao Ambedkar A Cent Univ, Sch Environm Sci, Dept Environm Microbiol, Rhizosphere Biol Lab, Raebareli Rd, Lucknow 226025, Uttar Pradesh, India."
EM rajesh4971@yahoo.com
"OI Lal, Shatrohan/0000-0003-1950-7365"
"FU University Grant Commission, New Delhi, India"
[NFSC/2017-18/SC/UTT/29727]
FX Research grant no. NFSC/2017-18/SC/UTT/29727 received from University
" Grant Commission, New Delhi, India to support this study is duly"
acknowledged.
"CR Abbasi H, 2012, J BIOSCI BIOENG, V113, P211, DOI 10.1016/j.jbiosc.2011.10.002"
" Acikel YS., 2011, Biomanagement of Metal-Contaminated Soils, P183, DOI DOI 10.1007/978-94-007-1914-9_8"
" Acosta MP, 2005, WORLD J MICROB BIOT, V21, P1157, DOI 10.1007/s11274-005-0381-6"
" Adrees M, 2015, ENVIRON SCI POLLUT R, V22, P8148, DOI 10.1007/s11356-015-4496-5"
" Agnello AC, 2016, SCI TOTAL ENVIRON, V563, P693, DOI 10.1016/j.scitotenv.2015.10.061"
" Ahemad Munees, 2015, Journal of Genetic Engineering and Biotechnology, V13, P51, DOI [10.1016/j.jgeb.2015.02.001, 10.1016/j.jgeb.2015.02.001]"
" Akhtar MJ, 2018, CHEMOSPHERE, V190, P234, DOI 10.1016/j.chemosphere.2017.09.136"
" Alam MZ, 2013, ANN MICROBIOL, V63, P1137, DOI 10.1007/s13213-012-0571-z"
" Ali H, 2013, CHEMOSPHERE, V91, P869, DOI 10.1016/j.chemosphere.2013.01.075"
" Alissa EM, 2011, J TOXICOL-US, V2011, DOI 10.1155/2011/870125"
" Alkorta I., 2004, Reviews in Environmental Science and Bio/Technology, V3, P55, DOI 10.1023/B:RESB.0000040057.45006.34"
" Alslaibi TM, 2013, J ENVIRON CHEM ENG, V1, P589, DOI 10.1016/j.jece.2013.06.028"
" [Anonymous], INT J PHARM LIFE SCI"
" [Anonymous], 2014, INTERDISCIP TOXICOL"
" [Anonymous], RESOUR PROCESS"
" [Anonymous], GEOMICROBIOL J"
" [Anonymous], THESIS"
" [Anonymous], 2011, INT J CHEM ENG, DOI DOI 10.1155/2011/939161"
" [Anonymous], NAT ED KNOWL"
" [Anonymous], 2009, J ENV HLTH"
" [Anonymous], SCI REP"
" [Anonymous], WATER AIR SOIL POLLU"
" [Anonymous], ENV GEOCHEM HLTH"
" [Anonymous], 2014, NANO RES LETT, DOI DOI 10.1371/J0URNAL.P0NE.0106618"
" [Anonymous], INT J PUBL HLTH"
" [Anonymous], J BIOREMEDIAT BIODEG"
" [Anonymous], 2015, J GLOBAL BIOSCI"
" [Anonymous], 2017, Handbook of Metal Microbe Interactions and Bioremediation"
" [Anonymous], J POLYM"
" [Anonymous], 2016, WATER AIR SOIL POLL"
" As??i YA, 2008, J ENVIRON MANAGE, V88, P383, DOI 10.1016/j.jenvman.2007.03.006"
" Ayangbenro AS, 2017, INT J ENV RES PUB HE, V14, DOI 10.3390/ijerph14010094"
" Babu AG, 2015, J ENVIRON MANAGE, V151, P160, DOI 10.1016/j.jenvman.2014.12.045"
" BAGCHI D, 1995, COMP BIOCHEM PHYS C, V110, P177, DOI 10.1016/0742-8413(94)00093-P"
" BAGCHI D, 1995, COMP BIOCHEM PHYS C, V110, P281, DOI 10.1016/0742-8413(94)00103-H"
" Bagchi D, 2002, TOXICOLOGY, V180, P5, DOI 10.1016/S0300-483X(02)00378-5"
" Barajas-Aceves M, 2015, SOIL SEDIMENT CONTAM, V24, P223, DOI 10.1080/15320383.2015.946593"
" Barathi S, 2001, ENVIRON INT, V26, P413, DOI 10.1016/S0160-4120(01)00021-6"
" Bauer H, 2013, CURR BIOL, V23, P53, DOI 10.1016/j.cub.2012.11.022"
" Bay??u G, 2017, ENVIRON SCI POLLUT R, V24, P2840, DOI 10.1007/s11356-016-8048-4"
" Beier EE, 2013, ENVIRON HEALTH PERSP, V121, P97, DOI 10.1289/ehp.1205374"
" Bento FM, 2005, MICROBIOL RES, V160, P249, DOI 10.1016/j.micres.2004.08.005"
" Bodour AA, 2003, APPL ENVIRON MICROB, V69, P3280, DOI 10.1128/AEM.69.6.3280-3287.2003"
" Bolan N, 2014, J HAZARD MATER, V266, P141, DOI 10.1016/j.jhazmat.2013.12.018"

"   Braud Armelle., 2006, Water, Air, and Soil Pollution, V6, P261, DOI [10.1007/s11267-005-9022-1, DOI 10.1007/S11267-005-9022-1]"
"   Cameotra SS, 2009, MICROB CELL FACT, V8, DOI 10.1186/1475-2859-8-16"
"   Cerqueira VS, 2012, WORLD J MICROB BIOT, V28, P1203, DOI 10.1007/s11274-011-0923-z"
"   Cerqueira VS, 2011, BIORESOURCE TECHNOL, V102, P11003, DOI 10.1016/j.biortech.2011.09.074"
"   Chaudhary P, 2011, B ENVIRON CONTAM TOX, V86, P268, DOI 10.1007/s00128-011-0211-5"
"   Chen J, 2012, J APPL MICROBIOL, V112, P660, DOI 10.1111/j.1365-2672.2012.05242.x"
"   Chen L, 2014, SOIL BIOL BIOCHEM, V68, P300, DOI 10.1016/j.soilbio.2013.10.021"
"   Chen SY, 2007, BIOTECHNOL PROGR, V23, P661, DOI 10.1021/bp0700152"
"   Chen Z, 2017, PLANT PHYSIOL BIOCH, V111, P179, DOI 10.1016/j.plaphy.2016.11.027"
"   Chhaya Verma Chhaya Verma, 2015, Archives of Applied Science Research, V7, P37"
"   Chibuike G.U., 2014, APPL ENVIRON SOIL SC, DOI [10.1155/2014/752708, DOI 10.1155/2014/752708]"
"   Chouchene L, 2016, J APPL TOXICOL, V36, P863, DOI 10.1002/jat.3285"
"   Clemens S, 2016, ANNU REV PLANT BIOL, V67, P489, DOI 10.1146/annurev-arplant-043015-112301"
"   Cobbett C, 2002, ANNU REV PLANT BIOL, V53, P159, DOI 10.1146/annurev.arplant.53.100301.135154"
"   Cogliano VJ, 2011, JNCI-J NATL CANCER I, V103, P1827, DOI 10.1093/jnci/djr483"
"   Coman V, 2013, RESOUR CONSERV RECY, V73, P229, DOI 10.1016/j.resconrec.2013.01.019"
"   Coppotelli BM, 2010, MICROB ECOL, V59, P266, DOI 10.1007/s00248-009-9563-3"
"   Costa SGVAO, 2010, PROCESS BIOCHEM, V45, P1511, DOI 10.1016/j.procbio.2010.05.033"
"   Cruz K, 2017, APPL MICROBIOL BIOT, V101, P5493, DOI 10.1007/s00253-017-8239-z"
"   Gomes MAD, 2016, ECOTOX ENVIRON SAFE, V134, P133, DOI 10.1016/j.ecoenv.2016.08.024"
"   Dahrazma B, 2007, CHEMOSPHERE, V69, P705, DOI 10.1016/j.chemosphere.2007.05.037"
"   Damek-Poprawa M, 2003, TOXICOLOGY, V186, P1, DOI 10.1016/S0300-483X(02)00595-4"
"   Darvishi P, 2011, COLLOID SURFACE B, V84, P292, DOI 10.1016/j.colsurfb.2011.01.011"
"   Das AJ, 2017, INT J ENVIRON SCI TE, V14, P1343, DOI 10.1007/s13762-016-1183-0"
"   Das AJ, 2016, J ENVIRON MANAGE, V174, P79, DOI 10.1016/j.jenvman.2016.01.031"
"   Das KK, 2008, INDIAN J MED RES, V128, P412"
"   Das K, 2007, BIORESOURCE TECHNOL, V98, P1339, DOI 10.1016/j.biortech.2006.05.032"
"   de Lima CJB, 2009, APPL BIOCHEM BIOTECH, V152, P156, DOI 10.1007/s12010-008-8188-1"
"   Martins PSD, 2008, BRAZ J MICROBIOL, V39, P780, DOI [10.1590/S1517-83822008000400034, 10.1590/S1517-838220080004000034]"
"   Deikman J, 1997, PHYSIOL PLANTARUM, V100, P561, DOI 10.1034/j.1399-3054.1997.1000318.x"
"   Department of Biotechnology Guru Ghasidas Vishwavidyalaya Bilaspur-495009 India., 2016, International Journal of Advanced Research, V4, P116, DOI [10.21474/ijar01/111, 10.21474/IJAR01/111]"
"   Desai C, 2008, PROCESS BIOCHEM, V43, P713, DOI 10.1016/j.procbio.2008.02.015"
"   Deschatre M, 2013, APPL BIOCHEM BIOTECH, V171, P1313, DOI 10.1007/s12010-013-0343-7"
"   Dixit R, 2015, SUSTAINABILITY-BASEL, V7, P2189, DOI 10.3390/su7022189"
"   do Amaral FP, 2016, PLANT MOL BIOL, V90, P689, DOI 10.1007/s11103-016-0449-8"
"   Dobrowolski R, 2017, BIORESOURCE TECHNOL, V225, P113, DOI 10.1016/j.biortech.2016.11.040"
"   Domenech J, 2008, BBA-PROTEINS PROTEOM, V1784, P693, DOI 10.1016/j.bbapap.2008.01.008"
"   Dusek P, 2015, J TRACE ELEM MED BIO, V31, P193, DOI 10.1016/j.jtemb.2014.05.007"
"   Eddouaouda K, 2012, J BASIC MICROB, V52, P408, DOI 10.1002/jobm.201100268"
"   Eisler R., 2000, HDB CHEM RISK ASSESS, V2"
"   Eqani SAMAS, 2016, CHEMOSPHERE, V155, P259, DOI 10.1016/j.chemosphere.2016.04.036"
"   Farag, 2000, ICEHM2000, P542"
"   Feng MQ, 2012, J FOOD SCI, V77, pT111, DOI 10.1111/j.1750-3841.2012.02734.x"
"   Filipic M, 2012, MUTAT RES-FUND MOL M, V733, P69, DOI 10.1016/j.mrfmmm.2011.09.002"
"   Flora Gagan, 2012, Interdiscip Toxicol, V5, P47, DOI 10.2478/v10102-012-0009-2"
"   Flora SJS, 2008, INDIAN J MED RES, V128, P501"
"   Franchi E, 2017, J SOIL SEDIMENT, V17, P1224, DOI 10.1007/s11368-015-1346-5"
"   Freitas F, 2011, CARBOHYD POLYM, V83, P159, DOI 10.1016/j.carbpol.2010.07.034"
"   Gadd GM, 2004, GEODERMA, V122, P109, DOI 10.1016/j.geoderma.2004.01.002"
"   Garc??a-Garc??a JD, 2016, BIOTECHNOL ADV, V34, P859, DOI 10.1016/j.biotechadv.2016.05.003"
"   Gawali Ashruta A., 2014, International Journal of Life-Sciences, V2, P373"
"   Gesheva V, 2010, CURR MICROBIOL, V61, P112, DOI 10.1007/s00284-010-9584-7"
"   Gibb H, 2014, ENVIRON HEALTH PERSP, V122, P667, DOI 10.1289/ehp.1307864"
"   Gillis BS, 2012, BMC GENOMICS, V13, DOI 10.1186/1471-2164-13-344"
"   Glick BR, 2010, BIOTECHNOL ADV, V28, P367, DOI 10.1016/j.biotechadv.2010.02.001"
"   Govarthanan M, 2017, BIOTECHNOL BIOPROC E, V22, P186, DOI 10.1007/s12257-016-0652-0"
"   Grennan AK, 2011, PLANT PHYSIOL, V155, P1750, DOI 10.1104/pp.111.900407"
"   Gunaratnam M, 2008, TOXICOL IN VITRO, V22, P879, DOI 10.1016/j.tiv.2008.01.005"
"   Gunther NW, 2005, APPL ENVIRON MICROB, V71, P2288, DOI 10.1128/AEM.71.5.2288-2293.2005"
"   Guo JB, 2008, CHEMOSPHERE, V72, P1020, DOI 10.1016/j.chemosphere.2008.04.018"
"   Gupta Pratima, 2017, Biotechnol Rep (Amst), V13, P58, DOI [10.1016/j.btre.2016.12.006, 10.1016/j.btre.2016.12.006]"
"   Gupta P, 2017, WORLD J MICROB BIOT, V33, DOI 10.1007/s11274-016-2176-3"
"   Guti??rrez JC, 2011, J BIOL INORG CHEM, V16, P1025, DOI 10.1007/s00775-011-0820-9"
"   Haq F, 2016, DESALIN WATER TREAT, V57, P19946, DOI 10.1080/19443994.2015.1109561"
"   Hatef A, 2011, ENVIRON TOXICOL CHEM, V30, P905, DOI 10.1002/etc.461"
"   Hawkes SJ, 1999, J CHEM EDUC, V76, P1099, DOI 10.1021/ed076p1099"
"   Henkler Frank, 2010, Cancers (Basel), V2, P376, DOI 10.3390/cancers2020376"
"   Hossain MA., 2012, APPL ENVIRON SOIL SC, V2012, DOI [DOI 10.1155/2012/650206, 10.1155/2012/872875, DOI 10.1155/2012/872875]"
"   Hossain SM, 2006, AFR J BIOTECHNOL, V5, P189"
"   Hua F, 2012, J APPL MICROBIOL, V112, P25, DOI 10.1111/j.1365-2672.2011.05178.x"
"   Hussain MB, 2014, INT J AGRIC BIOL, V16, P3"
"   Jacquart A, 2017, CHEM-BIOL INTERACT, V267, P2, DOI 10.1016/j.cbi.2016.09.002"
"   Johansson C, 2007, NEUROTOX RES, V11, P241, DOI 10.1007/BF03033570"
"   Jomova K, 2011, TOXICOLOGY, V283, P65, DOI 10.1016/j.tox.2011.03.001"
"   Joshi PM, 2009, ENVIRON SCI TECHNOL, V43, P5884, DOI 10.1021/es900063b"
"   Juwarkar AA, 2007, CHEMOSPHERE, V68, P1996, DOI 10.1016/j.chemosphere.2007.02.027"
"   Karthik C, 2017, GEOMICROBIOL J, V34, P434, DOI 10.1080/01490451.2016.1219429"
"   Karthik C, 2016, J ADV RES, V7, P839, DOI 10.1016/j.jare.2016.08.007"
"   Kasperczyk S, 2004, BIOL TRACE ELEM RES, V102, P61, DOI 10.1385/BTER:102:1-3:061"

"   Kaushal M, 2016, AGR ECOSYST ENVIRON, V231, P68, DOI 10.1016/j.agee.2016.06.031"
"   Khan MS, 2009, ENVIRON CHEM LETT, V7, P1, DOI 10.1007/s10311-008-0155-0"
"   Khan S, 2015, J HAZARD MATER, V300, P243, DOI 10.1016/j.jhazmat.2015.06.050"
"   Khan S, 2014, ENVIRON INT, V68, P154, DOI 10.1016/j.envint.2014.03.017"
"   Kibria MG, 2008, SOIL ENVRON, V27, P23"
"   Kidd PS, 2017, ADV BOT RES, V83, P87, DOI 10.1016/bs.abr.2016.12.004"
"   Kim IH, 2015, J MICROBIOL BIOTECHN, V25, P1542, DOI 10.4014/jmb.1504.04067"
"   Kim KH, 2016, J HAZARD MATER, V306, P376, DOI 10.1016/j.jhazmat.2015.11.031"
"   Kim PI, 2010, J MICROBIOL BIOTECHN, V20, P138, DOI 10.4014/jmb.0905.05007"
"   Kloepper J.W., 1978, Proceedings 4th International Conference on Plant Pathogenic Bacteria, P879"
"   Koedrith P, 2013, INT J HYG ENVIR HEAL, V216, P587, DOI 10.1016/j.ijheh.2013.02.010"
"   Kruijt M, 2009, J APPL MICROBIOL, V107, P546, DOI 10.1111/j.1365-2672.2009.04244.x"
"   Kumar A, 2015, J PURE APPL MICROBIO, V9, P715"
"   Kumar CG, 2012, BIOTECHNOL PROGR, V28, P1507, DOI 10.1002/btpr.1634"
"   Lakzian A, 2008, ASIAN J CHEM, V20, P6075"
"   Lam TV, 2007, SCI TOTAL ENVIRON, V372, P455, DOI 10.1016/j.scitotenv.2006.10.018"
"   Latvala S, 2016, PLOS ONE, V11, DOI 10.1371/journal.pone.0159684"
"   Lawniczak L, 2013, APPL MICROBIOL BIOT, V97, P2327, DOI 10.1007/s00253-013-4740-1"
"   Lebeau T, 2008, ENVIRON POLLUT, V153, P497, DOI 10.1016/j.envpol.2007.09.015"
"   Lee HJ, 2015, J NUTR BIOCHEM, V26, P1414, DOI 10.1016/j.jnutbio.2015.07.008"
"   Leitenmaier B, 2013, FRONT PLANT SCI, V4, DOI 10.3389/fpls.2013.00374"
"   Li N, 2017, J COLLOID INTERF SCI, V490, P754, DOI 10.1016/j.jcis.2016.12.006"
"   Limcharoensuk T, 2015, ECOTOX ENVIRON SAFE, V122, P322, DOI 10.1016/j.ecoenv.2015.08.013"
"   Liu Y, 2001, WATER SCI TECHNOL, V43, P59, DOI 10.2166/wst.2001.0340"
"   Liu ZM, 2012, WORLD J MICROB BIOT, V28, P1585, DOI 10.1007/s11274-011-0962-5"
"   Luo SL, 2012, APPL MICROBIOL BIOT, V93, P1745, DOI 10.1007/s00253-011-3483-0"
"   Ma Y, 2016, J HAZARD MATER, V320, P36, DOI 10.1016/j.jhazmat.2016.08.009"
"   Mahrukh Zubair Mahrukh Zubair, 2016, Environmental Technology Reviews, V5, P112, DOI [10.1080/21622515.2016.1259358, 10.1080/21622515.2016.1259358]"
"   Maier RM, 2000, APPL MICROBIOL BIOT, V54, P625, DOI 10.1007/s002530000443"
"   Mallick I, 2018, SCI TOTAL ENVIRON, V610, P1239, DOI 10.1016/j.scitotenv.2017.07.234"
"   Mao XH, 2015, J HAZARD MATER, V285, P419, DOI 10.1016/j.jhazmat.2014.12.009"
"   Martorell I, 2011, BIOL TRACE ELEM RES, V142, P309, DOI 10.1007/s12011-010-8787-x"
"   Mir SA, 2017, PROTEOMICS, V17, DOI 10.1002/pmic.201600257"
"   Mokaddem H, 2014, CHEM ENGINEER TRANS, V38, P31, DOI 10.3303/CET1438006"
"   Moore F, 2016, ENVIRON EARTH SCI, V75, DOI 10.1007/s12665-015-5026-y"
"   Morillo JA, 2006, CURR MICROBIOL, V53, P189, DOI 10.1007/s00284-005-0438-7"
"   Mulligan CN, 2005, ENVIRON POLLUT, V133, P183, DOI 10.1016/j.envpol.2004.06.009"
"   Nagajyoti PC, 2010, ENVIRON CHEM LETT, V8, P199, DOI 10.1007/s10311-010-0297-8"
"   Naja G., 2011, Microbial Biosorption of Metals, Chapter 3: The mechanism of metal cation and anion biosorption, DOI DOI 10.1007/978-94-007-0443-5_3"
"   Neeti K., 2013, International Research Journal of Environmental Sciences, V2, P88"
"   Nguyen THH, 2011, BIORESOURCE TECHNOL, V102, P2228, DOI 10.1016/j.biortech.2010.10.014"
"   Nie MQ, 2010, BIOTECHNOL ADV, V28, P635, DOI 10.1016/j.biotechadv.2010.05.013"
"   Nielsen TH, 2003, APPL ENVIRON MICROB, V69, P861, DOI 10.1128/AEM.69.2.861-868.2003"
"   Nonnoi F, 2012, APPL SOIL ECOL, V61, P49, DOI 10.1016/j.apsoil.2012.06.004"
"   Nouri J, 2009, ENVIRON EARTH SCI, V59, P315, DOI 10.1007/s12665-009-0028-2"
"   Olaniran AO, 2013, INT J MOL SCI, V14, P10197, DOI 10.3390/ijms140510197"
"   Oner E.T., 2013, PRETREATMENT TECHNIQ, P35, DOI DOI 10.1007/978-3-642-32735-3_2"
"   Onwosi CO, 2012, WORLD J MICROB BIOT, V28, P937, DOI 10.1007/s11274-011-0891-3"
"   Oshima T, 2008, REACT FUNCT POLYM, V68, P376, DOI 10.1016/j.reactfunctpolym.2007.07.046"
"   Ouzounidou G, 2006, ARCH ENVIRON CON TOX, V50, P346, DOI 10.1007/s00244-005-5076-3"
"   Oyaro N, 2007, J FOOD AGRIC ENVIRON, V5, P119"
"   Ozdemir G, 2005, BIORESOURCE TECHNOL, V96, P1677, DOI 10.1016/j.biortech.2004.12.031"
"   Ozdemir G, 2005, WORLD J MICROB BIOT, V21, P163, DOI 10.1007/s11274-004-1563-3"
"   Ozdemir G, 2003, BIORESOURCE TECHNOL, V90, P71, DOI 10.1016/S0960-8524(03)00088-9"
"   Paredes-P??liz KI, 2016, ENVIRON SCI POLLUT R, V23, P19825, DOI 10.1007/s11356-016-7184-1"
"   Patlolla AK, 2009, ENVIRON TOXICOL, V24, P66, DOI 10.1002/tox.20395"
"   Perneel M, 2007, J APPL MICROBIOL, V103, P1007, DOI 10.1111/j.1365-2672.2007.03345.x"
"   Petrus R, 2003, MICROPOR MESOPOR MAT, V61, P137, DOI 10.1016/S1387-1811(03)00361-5"
"   Pourahmad J, 2005, TOXICOLOGY, V206, P449, DOI 10.1016/j.tox.2004.08.002"
"   Raj R, 2016, J COLLOID INTERF SCI, V462, P166, DOI 10.1016/j.jcis.2015.10.004"
"   Rajkumar M, 2009, CHEMOSPHERE, V77, P153, DOI 10.1016/j.chemosphere.2009.06.047"
"   Rasulov B.A., 2013, J ENV PROTECT, V4, P989"
"   Rice Kevin M, 2014, J Prev Med Public Health, V47, P74, DOI 10.3961/jpmph.2014.47.2.74"
"   Rigouin C, 2013, PLOS NEGLECT TROP D, V7, DOI 10.1371/journal.pntd.0002037"
"   Robison MM, 2001, J PHYTOPATHOL, V149, P385, DOI 10.1046/j.1439-0434.2001.00639.x"
"   Rogival D, 2007, ENVIRON POLLUT, V145, P516, DOI 10.1016/j.envpol.2006.04.019"
"   Rold??n-Carrillo T, 2011, COLLOID SURFACE B, V86, P384, DOI 10.1016/j.colsurfb.2011.04.026"
"   Rom??n-Ponce B, 2017, PEDOSPHERE, V27, P511, DOI 10.1016/S1002-0160(17)60347-3"
"   Romano RL, 2017, J APPL PHYCOL, V29, P811, DOI 10.1007/s10811-016-0927-z"
"   Saikia T, 2017, Asian Journal of Soil Science and Plant Nutrition, V1, P1, DOI 10.9734/ajsspn/2017/33653"
"   Salehizadeh H, 2003, WATER RES, V37, P4231, DOI 10.1016/S0043-1354(03)00418-4"
"   Saraswat S, 2009, CHEM ECOL, V25, P1, DOI 10.1080/02757540802657185"
"   Sarubbo LA, 2015, CHEM ECOL, V31, P707, DOI 10.1080/02757540.2015.1095293"
"   Sarwar N, 2017, CHEMOSPHERE, V171, P710, DOI 10.1016/j.chemosphere.2016.12.116"
"   Sastoque-Cala Leonardo, 2010, Univ. Sci., V15, P251"
"   Schauml??ffel D, 2012, J TRACE ELEM MED BIO, V26, P1, DOI 10.1016/j.jtemb.2012.01.002"
"   Schue M, 2011, PLOS ONE, V6, DOI 10.1371/journal.pone.0026771"
"   Sekhon KK, 2011, MICROB CELL FACT, V10, DOI 10.1186/1475-2859-10-49"
"   Sessitsch A, 2013, SOIL BIOL BIOCHEM, V60, P182, DOI 10.1016/j.soilbio.2013.01.012"
"   Shahid M, 2017, CHEMOSPHERE, V178, P513, DOI 10.1016/j.chemosphere.2017.03.074"
"   Shahid M, 2017, REV ENVIRON CONTAM T, V241, P73, DOI 10.1007/398_2016_8"
"   Sharma J, 2017, SCI TOTAL ENVIRON, V579, P359, DOI 10.1016/j.scitotenv.2016.11.089"

"   Sharma SS, 2017, PLANT PHYSIOL BIOCH, V114, P29, DOI 10.1016/j.plaphy.2017.02.017"
"   Sheng GP, 2010, BIOTECHNOL ADV, V28, P882, DOI 10.1016/j.biotechadv.2010.08.001"
"   Sheng XF, 2008, J HAZARD MATER, V155, P17, DOI 10.1016/j.jhazmat.2007.10.107"
"   Singh AK, 2013, ENVIRON SCI POLLUT R, V20, P7367, DOI 10.1007/s11356-013-1752-4"
"   Singh PB, 2009, LETT APPL MICROBIOL, V49, P378, DOI 10.1111/j.1472-765X.2009.02672.x"
"   Singh R, 2011, INDIAN J PHARMACOL, V43, P246, DOI 10.4103/0253-7613.81505"
"   Snook ME, 2009, J AGR FOOD CHEM, V57, P4287, DOI 10.1021/jf900164h"
"   Soudani N, 2013, ENVIRON TOXICOL, V28, P11, DOI 10.1002/tox.20692"
"   Sriprang R, 2002, J BIOTECHNOL, V99, P279, DOI 10.1016/S0168-1656(02)00219-5"
"   Stolt JP, 2003, ENVIRON EXP BOT, V49, P21, DOI 10.1016/S0098-8472(02)00045-X"
"   Sughis M, 2011, ENVIRON HEALTH-GLOB, V10, DOI 10.1186/1476-069X-10-104"
"   Sultan S, 2012, AFR J MICROBIOL RES, V6, P3329, DOI 10.5897/AJMR12.226"
"   Suriyagoda LDB, 2017, PLANT SOIL, V410, P299, DOI 10.1007/s11104-016-3012-0"
"   Takeyama H, 2002, APPL BIOCHEM BIOTECH, V98, P319, DOI 10.1385/ABAB:98-100:1-9:319"
"   Tripathi RD, 2007, TRENDS BIOTECHNOL, V25, P158, DOI 10.1016/j.tibtech.2007.02.003"
"   Tuleva B, 2008, J APPL MICROBIOL, V104, P1703, DOI 10.1111/j.1365-2672.2007.03680.x"
"   Ullah A, 2015, ENVIRON EXP BOT, V117, P28, DOI 10.1016/j.envexpbot.2015.05.001"
"   Ullah A, 2015, ENVIRON SCI POLLUT R, V22, P2505, DOI 10.1007/s11356-014-3699-5"
"   Upadhyay A., 2017, FRONT MICROBIOL, V8"
"   Vacheron J, 2013, FRONT PLANT SCI, V4, DOI 10.3389/fpls.2013.00356"
"   Valverde A, 2011, CHEMOSPHERE, V85, P129, DOI 10.1016/j.chemosphere.2011.06.025"
"   Vanderlinde EM, 2010, FEMS MICROBIOL ECOL, V71, P327, DOI 10.1111/j.1574-6941.2009.00824.x"
"   Vasileva-Tonkova E, 2007, CURR MICROBIOL, V54, P136, DOI 10.1007/s00284-006-0345-6"
"   Verougstraete V, 2002, MUTAT RES-REV MUTAT, V511, P15, DOI 10.1016/S1383-5742(01)00070-9"
"   Violante A, 2010, J SOIL SCI PLANT NUT, V10, P268, DOI 10.4067/S0718-95162010000100005"
"   Vogel-Mikus K, 2008, PLANT CELL ENVIRON, V31, P1484, DOI 10.1111/j.1365-3040.2008.01858.x"
"   Wang SL, 2004, WATER AIR SOIL POLL, V157, P315, DOI 10.1023/B:WATE.0000038904.91977.f0"
"   Wang XF, 2006, TOXICOLOGY, V228, P16, DOI 10.1016/j.tox.2006.08.005"
"   Waqas M, 2015, ENVIRON SCI POLLUT R, V22, P12114, DOI 10.1007/s11356-015-4432-8"
"   Wattanaphon HT, 2008, J APPL MICROBIOL, V105, P416, DOI 10.1111/j.1365-2672.2008.03755.x"
"   Wei X, 2011, ENVIRON POLLUT, V159, P1369, DOI 10.1016/j.envpol.2011.01.006"
"   Williams GP, 2013, GEOMICROBIOL J, V30, P497, DOI 10.1080/01490451.2012.732663"
"   Wilson SC, 2014, ENVIRON SCI POLLUT R, V21, P11671, DOI 10.1007/s11356-014-2577-5"
"   WOOD R, 1990, S AFR MED J, V77, P640"
"   Wu ZL, 2015, FRONT PLANT SCI, V6, DOI 10.3389/fpls.2015.00136"
"   Wuana R.A., 2011, ISRN Ecology, DOI [10.5402/2011/402647, DOI 10.5402/2011/402647]"
"   Xu XJ, 2012, INT BIODETER BIODEGR, V71, P55, DOI 10.1016/j.ibiod.2012.03.006"
"   Yadav SK, 2010, S AFR J BOT, V76, P167, DOI 10.1016/j.sajb.2009.10.007"
"   Yan P, 2017, BIORESOURCE TECHNOL, V232, P354, DOI 10.1016/j.biortech.2017.02.067"
"   Yang H, 2013, ENVIRON SCI POLLUT R, V20, P4441, DOI 10.1007/s11356-012-1366-2"
"   Yang QW, 2011, ECOTOX ENVIRON SAFE, V74, P1664, DOI 10.1016/j.ecoenv.2011.05.006"
"   Yang XH, 2017, MAR POLLUT BULL, V115, P489, DOI 10.1016/j.marpolbul.2016.11.046"
"   Yao ZT, 2012, PROCEDIA ENVIRON SCI, V16, P722, DOI 10.1016/j.proenv.2012.10.099"
"   Zeinali M, 2007, LETT APPL MICROBIOL, V45, P622, DOI 10.1111/j.1472-765X.2007.02241.x"
"   Zhang YF, 2017, ENVIRON GEOCHEM HLTH, V39, P29, DOI 10.1007/s10653-016-9805-5"
"   Zhu A, 2017, J NANOSCI NANOTECHNO, V17, P1753, DOI 10.1166/jnn.2017.12849"
"   Zhuang P, 2009, SCI TOTAL ENVIRON, V407, P1551, DOI 10.1016/j.scitotenv.2008.10.061"
"   Ziller A, 2017, J INORG BIOCHEM, V167, P1, DOI [10.1016/j.jinorgbio.2016.11.07,
10.1016/j.jinorgbio.2016.11.017]"
NR 253
TC 86
Z9 103
U1 2
U2 116
PU ELSEVIER
PI AMSTERDAM
"PA RADARWEG 29, 1043 NX AMSTERDAM, NETHERLANDS"
SN 2352-1864
J9 ENVIRON TECHNOL INNO
JI Environ. Technol. Innov.
PD MAY
PY 2018
VL 10
BP 243
EP 263
DI 10.1016/j.eti.2018.02.011
PG 21
"WC Biotechnology & Applied Microbiology; Engineering, Environmental;"
   Environmental Sciences
WE Science Citation Index Expanded (SCI-EXPANDED)
SC Biotechnology & Applied Microbiology; Engineering; Environmental
   Sciences & Ecology
GA GN3TF
UT WOS:000438930800024
DA 2026-04-06
ER

PT J
"AU Ojuederie, OB"
"   Babalola, OO"
"AF Ojuederie, Omena Bernard"
"   Babalola, Olubukola Oluranti"
TI Microbial and Plant-Assisted Bioremediation of Heavy Metal Polluted
   Environments: A Review

SO INTERNATIONAL JOURNAL OF ENVIRONMENTAL RESEARCH AND PUBLIC HEALTH
LA English
DT Review
DE anthropogenic sources; environmental pollution; genetically engineered
organisms; heavy metals; microbial remediation; phytoremediation
ID GROWTH-PROMOTING RHIZOBACTERIA; POLYCYCLIC AROMATIC-HYDROCARBONS;
CONTAMINATED SOILS; HELIANTHUS-ANNUUS; ACC-DEAMINASE; AQUEOUS-SOLUTIONS;
INDIAN MUSTARD; PHYTOREMEDIATION; REMOVAL; BIOSORPTION
"AB Environmental pollution from hazardous waste materials, organic pollutants and heavy metals, has adversely affected the natural ecosystem to the detriment of man. These pollutants arise from anthropogenic sources as well as natural disasters such as hurricanes and volcanic eruptions. Toxic metals could accumulate in agricultural soils and get into the food chain, thereby becoming a major threat to food security. Conventional and physical methods are expensive and not effective in areas with low metal toxicity. Bioremediation is therefore an eco-friendly and efficient method of reclaiming environments contaminated with heavy metals by making use of the inherent biological mechanisms of microorganisms and plants to eradicate hazardous contaminants. This review discusses the toxic effects of heavy metal pollution and the mechanisms used by microbes and plants for environmental remediation. It also emphasized the importance of modern biotechnological techniques and approaches in improving the ability of microbial enzymes to effectively degrade heavy metals at a faster rate, highlighting recent advances in microbial bioremediation and phytoremediation for the removal of heavy metals from the environment as well as future prospects and limitations. However, strict adherence to biosafety regulations must be followed in the use of biotechnological methods to ensure safety of the environment."
"C1 [Ojuederie, Omena Bernard; Babalola, Olubukola Oluranti] North West Univ, Fac Nat & Agr Sci, Food Secur & Safety Niche Area, Private Mail Bag X2046, ZA-2735 Mmabatho, South Africa."
C3 North West University - South Africa
"RP Babalola, OO (corresponding author), North West Univ, Fac Nat & Agr Sci, Food Secur & Safety Niche Area, Private Mail Bag X2046, ZA-2735 Mmabatho, South Africa."
EM 30783887@nwu.ac.za; olubukola.babalola@nwu.ac.za
"RI Babalola, Olubukola Oluranti/Y-7564-2019; Ojuederie, Omena/GVU-6409-2022"
"OI Babalola, Olubukola Oluranti/0000-0003-4344-1909; "
FU North-West University; National Research Foundation of South Africa
"   [UID81192, UID86625, UID105248]"
FX The first author is grateful for the Postdoctoral fellowship awarded him
by North-West University. Works in OOB laboratory were supported by the
"   National Research Foundation of South Africa, grants UID81192, UID86625"
and UID105248.
"CR Abbas S.H., 2014, Journal of Chemical Science and Technology, V3, P74"
"   Abbaszadeh-Dahaji P., 2016, PLANT MICROBE INTERA, P187"
"   Abhijit Gupta Abhijit Gupta, 2016, Journal of Microbial and Biochemical Technology, V8, P364, DOI 10.4172/1948-5948.1000310"
"   Achal V, 2012, J HAZARD MATER, V201, P178, DOI 10.1016/j.jhazmat.2011.11.067"
"   Adesodun JK, 2010, WATER AIR SOIL POLL, V207, P195, DOI 10.1007/s11270-009-0128-3"
"   Ahemad M, 2011, BACTERIOL J, V2, P12, DOI DOI 10.3923/BJ.2012.12.21"
"   Ahemad Munees, 2014, Journal of King Saud University Science, V26, P1, DOI 10.1016/j.jksus.2013.05.001"
"   Ahmed E, 2014, MICROB BIOTECHNOL, V7, P196, DOI 10.1111/1751-7915.12117"
"   Ahmed MB, 2016, BIORESOURCE TECHNOL, V214, P836, DOI 10.1016/j.biortech.2016.05.057"
"   Aka RJN, 2016, INT J PHYTOREMEDIAT, V18, P200, DOI 10.1080/15226514.2015.1073671"
"   Alburquerque JA, 2011, J ENVIRON MANAGE, V92, P733, DOI 10.1016/j.jenvman.2010.10.018"
"   Alford ??R, 2012, AM J BOT, V99, P1930, DOI 10.3732/ajb.1200124"
"   Ali H, 2013, CHEMOSPHERE, V91, P869, DOI 10.1016/j.chemosphere.2013.01.075"
"   Angelova VR, 2016, International Journal of Environmental and Ecological Engineering, V10, P576, DOI DOI 10.5281/ZENODO.1126371"
"   [Anonymous], PLANT SOIL"
"   [Anonymous], 2012, Journal of Cell and Molecular Research"
"   [Anonymous], 2014, INTERDISCIP TOXICOL"
"   [Anonymous], 2015, J BIOREMED BIODEGRAD, DOI [10.4172/2155-6199.1000323, DOI 10.4172/2155-6199.1000323]"
"   [Anonymous], J BIOREMED BIODEGRAD"
"   [Anonymous], 2012, J I INTEGRATIVE OMIC"
"   [Anonymous], THESIS"
"   [Anonymous], 2014, ENV RISK ASSESSMENT"
"   [Anonymous], HYDROPONICS STANDARD"
"   [Anonymous], 2012, CURR TRENDS BIOTECHN"
"   [Anonymous], ENV REV"
"   [Anonymous], BIOTECHNOL ADV"
"   [Anonymous], 2012, THESIS"
"   [Anonymous], CLIM CHANG PLANT ABI"
"   [Anonymous], 2015, ADV BIOREMEDIATION W"
"   [Anonymous], 2012, SCIENCE"
"   [Anonymous], P IND GEOT C IGC2016"
"   [Anonymous], THESIS"
"   [Anonymous], INT J RECENT SCI RES"
"   [Anonymous], 2009, ADV ENV GEOTECHNICS, DOI DOI 10.1007/978-3-642-04460-1.25"
"   [Anonymous], 2011, DETOXIFICATION HEAVY, DOI DOI 10.1007/978-3-642-21408-0"
"   Arora K, 2016, INT J PHYTOREMEDIAT, V18, P704, DOI 10.1080/15226514.2015.1131232"
"   Arshad M, 2007, TRENDS BIOTECHNOL, V25, P356, DOI 10.1016/j.tibtech.2007.05.005"
"   Ayangbenro AS, 2017, INT J ENV RES PUB HE, V14, DOI 10.3390/ijerph14010094"
"   Azad MA, 2014, CHINESE SCI BULL, V59, P703, DOI 10.1007/s11434-013-0058-8"
"   Azubuike CC, 2016, WORLD J MICROB BIOT, V32, DOI 10.1007/s11274-016-2137-x"
"   Babalola OO, 2010, BIOTECHNOL LETT, V32, P1559, DOI 10.1007/s10529-010-0347-0"
"   Balamurugan D., 2014, International Journal of Environmental Sciences, V5, P522"
"   Balcazar JL, 2015, FRONT MICROBIOL, V6, DOI 10.3389/fmicb.2015.01216"
"   Barkay T, 2003, FEMS MICROBIOL REV, V27, P355, DOI 10.1016/S0168-6445(03)00046-9"
"   Beiyuan J, 2017, CHEMOSPHERE, V178, P110, DOI 10.1016/j.chemosphere.2017.03.022"
"   Bhattacharyya PN, 2012, WORLD J MICROB BIOT, V28, P1327, DOI 10.1007/s11274-011-0979-9"

"   Bielen A, 2013, INT J MOL SCI, V14, P6382, DOI 10.3390/ijms14036382"
"   Blaylock MJ, 1997, ENVIRON SCI TECHNOL, V31, P860, DOI 10.1021/es960552a"
"   Bolan NS, 2013, SCI TOTAL ENVIRON, V465, P216, DOI 10.1016/j.scitotenv.2012.12.093"
"   Bosso L, 2015, NEW BIOTECHNOL, V32, P21, DOI 10.1016/j.nbt.2014.08.001"
"   Brandt S, 1997, BIOTECHNOL BIOENG, V55, P480, DOI 10.1002/(SICI)1097-0290(19970805)55:3<480::AID-BIT3>3.0.CO;2-8"
"   Broadhurst CL, 2016, FRONT PLANT SCI, V7, DOI 10.3389/fpls.2016.00451"
"   Bucker-Neto L, 2017, GENET MOL BIOL, V40, P373, DOI [10.1590/1678-4685-GMB-2016-0087, 10.1590/1678-4685-gmb-2016-0087]"
"   Buermans HPJ, 2014, BBA-MOL BASIS DIS, V1842, P1932, DOI 10.1016/j.bbadis.2014.06.015"
"   Chandra K., 2015, International Journal of Pharmacognosy and Phytochemical Research, V7, P383"
"   Chandra P., 2016, Plant-Microbe Interaction: An Approach to Sustainable Agriculture, P37, DOI [10.1007/978-981-10-2854-0, DOI 10.1007/978-981-10-2854-0]"
"   Chaturvedi AD, 2015, WORLD J MICROB BIOT, V31, P1595, DOI 10.1007/s11274-015-1911-5"
"   Chen Boxia, 2013, [Frontiers in Biology, ???????????????], V8, P599"
"   Chen M, 2015, BIOTECHNOL ADV, V33, P745, DOI 10.1016/j.biotechadv.2015.05.003"
"   Chen SL, 1997, BIODEGRADATION, V8, P97, DOI 10.1023/A:1008233704719"
"   Chibuike GU., 2014, APPLIE ENV SOIL SCI, V2014, P1"
"   Choudhary D.K., 2016, Microbial-mediated induced systematic resistance in plants"
"   Choudhary D.K., 2017, Plant-Microbe Interaction: An Approach to Sustainable Agriculture"
"   Claire-Lise M, 2012, NEW BIOTECHNOL, V30, P9, DOI 10.1016/j.nbt.2012.07.009"
"   Martins MNC, 2016, CHEMOSPHERE, V148, P481, DOI 10.1016/j.chemosphere.2016.01.071"
"   D'Souza SF, 2001, BIOSENS BIOELECTRON, V16, P337, DOI 10.1016/S0956-5663(01)00125-7"
"   Datta R, 2017, SCI REP-UK, V7, DOI 10.1038/s41598-017-03194-x"
"   Davison John, 2002, Environmental Biosafety Research, V1, P9, DOI 10.1051/ebr:2002001"
"   Deckwer WD, 2004, ENVIRON SCI TECHNOL, V38, P1858, DOI 10.1021/es0300517"
"   Dell'mour M, 2012, ELECTROPHORESIS, V33, P726, DOI 10.1002/elps.201100466"
"   Divya B, 2011, RES J BIOTECHNOL, V6, P72"
"   Dixit R, 2015, SUSTAINABILITY-BASEL, V7, P2189, DOI 10.3390/su7022189"
"   Dong GW, 2013, BIOCHEM ENG J, V70, P166, DOI 10.1016/j.bej.2012.11.002"
"   Donot F, 2012, CARBOHYD POLYM, V87, P951, DOI 10.1016/j.carbpol.2011.08.083"
"   Du J, 2012, PLANT OMICS, V5, P46"
"   Ebbs SD, 1997, J ENVIRON QUAL, V26, P776, DOI 10.2134/jeq1997.00472425002600030026x"
"   Ekperusi OA, 2015, 3 BIOTECH, V5, P957, DOI 10.1007/s13205-015-0298-1"
"   Emamverdian A., 2015, SCI WORLD J, DOI DOI 10.1155/2015/756120"
"   Farhan SN, 2015, INT J IND CHEM, V6, P119, DOI 10.1007/s40090-015-0038-8"
"   Fomina M, 2014, BIORESOURCE TECHNOL, V160, P3, DOI 10.1016/j.biortech.2013.12.102"
"   Fran??ois F, 2012, APPL ENVIRON MICROB, V78, P1097, DOI 10.1128/AEM.06522-11"
"   Freitas EV, 2013, CHEMOSPHERE, V92, P213, DOI 10.1016/j.chemosphere.2013.01.103"
"   Fu YongQian Fu YongQian, 2012, African Journal of Biotechnology, V11, P1403"
"   Fulekar MH, 2016, International Journal of Current Microbiology and Applied Sciences, V5, P392, DOI [10.20546/ijcmas.2016.507.043, 10.20546/ijcmas.2016.507.043, DOI 10.20546/IJCMAS.2016.507.043]"
"   Gadd GM, 2010, MICROBIOL-SGM, V156, P609, DOI 10.1099/mic.0.037143-0"
"   Gaiero JR, 2013, AM J BOT, V100, P1738, DOI 10.3732/ajb.1200572"
"   Gaur N, 2014, ENVIRON SCI-PROC IMP, V16, P180, DOI 10.1039/c3em00491k"
"   Ghosal D, 2016, FRONT MICROBIOL, V7, DOI 10.3389/fmicb.2016.01369"
"   Glick BR, 2012, SCIENTIFICA, V2012, DOI 10.6064/2012/963401"
"   Glick BR, 2014, MICROBIOL RES, V169, P30, DOI 10.1016/j.micres.2013.09.009"
"   Graber ER, 2014, EUR J SOIL SCI, V65, P162, DOI 10.1111/ejss.12071"
"   Grennan AK, 2011, PLANT PHYSIOL, V155, P1750, DOI 10.1104/pp.111.900407"
"   Guo JL, 2013, BIOMED RES INT, V2013, DOI 10.1155/2013/904769"
"   Gupta DK, 2013, ENVIRON SCI POLLUT R, V20, P2150, DOI 10.1007/s11356-013-1485-4"
"   Gupta Pratima, 2017, Biotechnol Rep (Amst), V13, P58, DOI [10.1016/j.btre.2016.12.006, 10.1016/j.btre.2016.12.006]"
"   Gupta VK, 2015, ENVIRON ENG RES, V20, P1, DOI 10.4491/eer.2015.018"
"   Herrero EM, 2003, INT J PHYTOREMEDIAT, V5, P153, DOI 10.1080/713610177"
"   Hossain MA., 2012, APPL ENVIRON SOIL SC, V2012, DOI [DOI 10.1155/2012/650206, 10.1155/2012/872875, DOI 10.1155/2012/872875]"
"   Islam MS, 2013, INT J PHYTOREMEDIAT, V15, P1010, DOI 10.1080/15226514.2012.751356"
"   Jabeen R, 2009, BOT REV, V75, P339, DOI 10.1007/s12229-009-9036-x"
"   Jadia CD, 2009, AFR J BIOTECHNOL, V8, P921"
"   Jan AT, 2014, CRIT REV ENV SCI TEC, V44, P519, DOI 10.1080/10643389.2012.728811"
"   Jan S., 2016, Approaches to heavy metal tolerance in plants"
"   Jutsz AM, 2015, ARCH ENVIRON PROT, V41, P104, DOI 10.1515/aep-2015-0045"
"   Kabata-Pendias Alina, 2010, P1"
"   Kang CH, 2016, ECOL ENG, V89, P64, DOI 10.1016/j.ecoleng.2016.01.023"
"   Kang JW, 2014, BIOTECHNOL LETT, V36, P1129, DOI 10.1007/s10529-014-1466-9"
"   Kanmani P, 2012, INT J ENVIRON SCI TE, V9, P183, DOI 10.1007/s13762-011-0013-7"
"   Kluepfel L, 2014, ENVIRON SCI TECHNOL, V48, P5601, DOI 10.1021/es500906d"
"   Kobayashi T, 2012, ANNU REV PLANT BIOL, V63, P131, DOI 10.1146/annurev-arplant-042811-105522"
"   Krumova E, 2016, CLEAN-SOIL AIR WATER, V44, P657, DOI 10.1002/clen.201500139"
"   Kumar Ramasamy R., 2011, Asian J. Exp. Biol. Sci., V2, P342"
"   Kumar S., 2013, Biotechnology for Environmental Management and Resource Recovery, P191, DOI [10.1007/978-81-322-0876-1_11, DOI 10.1007/978-81-322-0876-1_11]"
"   Lakherwal D., 2014, International Journal of Environmental Research and Development, V4, P2249, DOI DOI 10.1016/J.MATPR.2019.07.462"
"   Lee YC, 2011, BIORESOURCE TECHNOL, V102, P5297, DOI 10.1016/j.biortech.2010.12.103"
"   Lin JX, 2003, BIORESOURCE TECHNOL, V86, P151, DOI 10.1016/S0960-8524(02)00152-9"
"   Liu S, 2011, J ENVIRON SCI-CHINA, V23, P1544, DOI 10.1016/S1001-0742(10)60570-0"
"   Lone MI, 2008, J ZHEJIANG UNIV-SC B, V9, P210, DOI 10.1631/jzus.B0710633"
"   Lu L, 2014, ENVIRON SCI TECHNOL, V48, P4021, DOI 10.1021/es4057906"
"   Ma Y, 2011, BIOTECHNOL ADV, V29, P248, DOI 10.1016/j.biotechadv.2010.12.001"
"   Machado MD, 2010, J HAZARD MATER, V180, P347, DOI 10.1016/j.jhazmat.2010.04.037"
"   Macovei A., 2010, AN UNIV ORADEA FASC, P194"

"   Madej??n P, 2003, SCI TOTAL ENVIRON, V307, P239, DOI 10.1016/S0048-9697(02)00609-5"
"   Mane PC, 2012, INT J ENVIRON RES, V6, P571"
"   Mani D, 2014, INT J ENVIRON SCI TE, V11, P843, DOI 10.1007/s13762-013-0299-8"
"   Mani S., 2015, Free radicals in human health and disease, P3, DOI [10.1007/978-81-322-2035-0_1, DOI 10.1007/978-81-322-2035-0_1]"
"   Marchiol L, 2007, PLANT PHYSIOL BIOCH, V45, P379, DOI 10.1016/j.plaphy.2007.03.018"
"   Marconi AM, 1997, BIOTECHNOL LETT, V19, P603, DOI 10.1023/A:1018366126573"
"   Martinez-Viveros O, 2010, J SOIL SCI PLANT NUT, V10, P293"
"   Mathialagan T, 2009, BIORESOURCE TECHNOL, V100, P549, DOI 10.1016/j.biortech.2008.06.054"
"   Mengoni A, 2012, BIOGEOINTERACTIONS M, V31, P279"
"   Mesjasz-Przybylowicz J, 2004, ACTA BIOL CRACOV BOT, V46, P75"
"   Milosevic N. A., 2012, Matica Srpska Proceedings for Natural Sciences, P17"
"   Mohan D, 2014, BIORESOURCE TECHNOL, V160, P191, DOI 10.1016/j.biortech.2014.01.120"
"   Mukhopadhyay S, 2010, APPL ECOL ENV RES, V8, P207"
"   Mustapha M. U., 2015, Journal of Microbial and Biochemical Technology, V7, P253, DOI 10.4172/1948-5948.1000219"
"   Muszynska Ewa, 2015, Biotechnologia (Poznan), V96, P265, DOI [10.5114/bta.2015.57730, 10.5114/bta.2015.57730]"
"   Nadeem SM, 2013, ANN MICROBIOL, V63, P225, DOI 10.1007/s13213-012-0465-0"
"   Nalla S, 2012, INSTRUM SCI TECHNOL, V40, P17, DOI 10.1080/10739149.2011.633143"
"   Nehra V., 2015, Journal of Applied and Natural Science, V7, P540, DOI 10.31018/jans.v7i1.642"
"   Nematian M. A., 2013, International Journal of Agriculture and Crop Sciences (IJACS), V5, P426"
"   Ok Y.S., 2015, BIOCHAR PRODUCTION C"
"   Oner E.T., 2013, PRETREATMENT TECHNIQ, P35, DOI DOI 10.1007/978-3-642-32735-3_2"
"   Paul D, 2014, AGRON SUSTAIN DEV, V34, P737, DOI 10.1007/s13593-014-0233-6"
"   Paz-Ferreiro J, 2014, SOLID EARTH, V5, P65, DOI 10.5194/se-5-65-2014"
"   Pourrut B, 2011, REV ENVIRON CONTAM T, V213, P113, DOI 10.1007/978-1-4419-9860-6_4"
"   Rahman A, 2015, J ENVIRON SCI HEAL A, V50, P1136, DOI 10.1080/10934529.2015.1047670"
"   Rahman MA, 2016, PHYTOREMEDIATION TOX, P161"
"   Rajapaksha AU, 2013, ENVIRON SCI TECHNOL, V47, P9722, DOI 10.1021/es4015025"
"   Rajkumar M, 2012, BIOTECHNOL ADV, V30, P1562, DOI 10.1016/j.biotechadv.2012.04.011"
"   Rajkumar M, 2010, TRENDS BIOTECHNOL, V28, P142, DOI 10.1016/j.tibtech.2009.12.002"
"   Ramadan E. M., 2016, African Journal of Microbiology Research, V10, P486, DOI 10.5897/ajmr2015.7714"
"   Rascio N, 2011, PLANT SCI, V180, P169, DOI 10.1016/j.plantsci.2010.08.016"
"   Rastgoo L, 2011, PLANT OMICS, V4, P377"
"   Rathinasabapathi B, 2011, WORKING WITH FERNS: ISSUES AND APPLICATIONS, P261, DOI 10.1007/978-1-4419-7162-3_19"
"   Rayu S, 2012, BIODEGRADATION, V23, P917, DOI 10.1007/s10532-012-9576-3"
"   Rizwan M, 2016, ENVIRON SCI POLLUT R, V23, P2230, DOI 10.1007/s11356-015-5697-7"
"   Rojas LA, 2011, PLOS ONE, V6, DOI 10.1371/journal.pone.0017555"
"   Saba H., 2013, INT RES J ENV SCI, V2, P74"
"   Samanta SK, 2002, TRENDS BIOTECHNOL, V20, P243, DOI 10.1016/S0167-7799(02)01943-1"
"   Saquing JM, 2016, ENVIRON SCI TECH LET, V3, P62, DOI 10.1021/acs.estlett.5b00354"
"   Selatnia A, 2004, BIOCHEM ENG J, V19, P127, DOI 10.1016/j.bej.2003.12.007"
"   Sharma P., 2012, J BOT, V2012"
"   Shruti Mishra Shruti Mishra, 2006, International Journal of Agricultural Research, V1, P122"
"   Silva P, 2016, ECOTOX ENVIRON SAFE, V131, P151, DOI 10.1016/j.ecoenv.2016.05.014"
"   Singh A, 2016, CHEM ECOL, V32, P684, DOI 10.1080/02757540.2016.1182994"
"   Singh JS, 2011, AGR ECOSYST ENVIRON, V140, P339, DOI 10.1016/j.agee.2011.01.017"
"   Skorzynska-Polit E, 2010, ACTA PHYSIOL PLANT, V32, P169, DOI 10.1007/s11738-009-0393-1"
"   Smith E, 2015, INT BIODETER BIODEGR, V101, P56, DOI 10.1016/j.ibiod.2015.03.029"
"   Solanki R, 2011, BIOLOGIA, V66, P195, DOI 10.2478/s11756-011-0005-6"
"   Sone Y, 2013, AMB EXPRESS, V3, DOI 10.1186/2191-0855-3-41"
"   Spaepen S, 2011, CSH PERSPECT BIOL, V3, DOI 10.1101/cshperspect.a001438"
"   Srivastava S, 2015, ENVIRON SCI POLLUT R, V22, P1499, DOI 10.1007/s11356-014-3442-2"
"   Su Y, 2008, INT J PHYTOREMEDIAT, V10, P547, DOI 10.1080/15226510802115091"
"   Sukumar M., 2010, African Journal of Environmental Science and Technology, V4, P412"
"   Tak HI, 2013, REV ENVIRON CONTAM T, V223, P33, DOI 10.1007/978-1-4614-5577-6_2"
"   Tandon PK, 2013, IND ENG CHEM RES, V52, P10052, DOI 10.1021/ie400702k"
"   Tandon PK, 2016, ENVIRON CHEM LETT, V14, P15, DOI 10.1007/s10311-015-0540-4"
"   Tastan BE, 2010, BIORESOURCE TECHNOL, V101, P870, DOI 10.1016/j.biortech.2009.08.099"
"   Teschler JK, 2015, NAT REV MICROBIOL, V13, P255, DOI 10.1038/nrmicro3433"
"   Tschirhart C, 2012, HUM ECOL, V40, P511, DOI 10.1007/s10745-012-9500-9"
"   Upadhyay N, 2017, FRONT PLANT SCI, V8, DOI 10.3389/fpls.2017.00778"
"   van der Ent A, 2013, PLANT SOIL, V362, P319, DOI 10.1007/s11104-012-1287-3"
"   Van Nostrand JD, 2012, FRONT MICROBIOL, V3, DOI 10.3389/fmicb.2012.00339"
"   Varenyam Achal Varenyam Achal, 2011, Research Journal of Microbiology, V6, P166, DOI 10.3923/jm.2011.166.171"
"   Varsha Mudgal Varsha Mudgal, 2010, Agriculture and Biology Journal of North America, V1, P40"
"   Vassilev Andon, 2004, ScientificWorldJournal, V4, P9"
"   Vejan P, 2016, MOLECULES, V21, DOI 10.3390/molecules21050573"
"   Verkleij JAC, 2003, SULPHUR IN PLANTS, P163"
"   Verma JP, 2016, FRONT MICROBIOL, V6, DOI [10.3389/fmicb.2015.01555, DOI 10.3389/FMICB.2015.01555, 10.3389/fmicb.2015.01555]"
"   Verma N, 2005, BIOMETALS, V18, P121, DOI 10.1007/s10534-004-5787-3"
"   Violante A, 2010, J SOIL SCI PLANT NUT, V10, P268, DOI 10.4067/S0718-95162010000100005"
"   Vullo DL, 2008, BIORESOURCE TECHNOL, V99, P5574, DOI 10.1016/j.biortech.2007.10.060"
"   Wang JL, 2000, BIORESOURCE TECHNOL, V75, P157, DOI 10.1016/S0960-8524(00)00041-9"
"   Wei SH, 2011, J SOIL SEDIMENT, V11, P915, DOI 10.1007/s11368-011-0389-5"
"   Wolejko E, 2016, ECOL CHEM ENG S, V23, P155, DOI 10.1515/eces-2016-0011"
"   Xie QE, 2009, ENVIRON SCI TECHNOL, V43, P8488, DOI 10.1021/es9014647"
"   Yuan Y, 2017, BIORESOURCE TECHNOL, V246, P271, DOI 10.1016/j.biortech.2017.06.154"
"   Zafar-ul-Hye M, 2014, INT J AGRIC BIOL, V16, P591"

"   Zaidi A., 2012, TOXICITY HEAVY METAL, P101, DOI [DOI 10.1007/978-3-7091-0730-0_6, 10.1007/978-3-7091-0730-0_6]"
"   Zhuang P, 2005, PLANT SOIL, V276, P153, DOI 10.1007/s11104-005-3901-0"
NR 201
TC 577
Z9 690
U1 17
U2 473
PU MDPI
PI BASEL
"PA ST ALBAN-ANLAGE 66, CH-4052 BASEL, SWITZERLAND"
EI 1660-4601
J9 INT J ENV RES PUB HE
JI Int. J. Environ. Res. Public Health
PD DEC
PY 2017
VL 14
IS 12
AR 1504
DI 10.3390/ijerph14121504
PG 26
"WC Environmental Sciences; Public, Environmental & Occupational Health"
WE Science Citation Index Expanded (SCI-EXPANDED); Social Science Citation Index (SSCI)
"SC Environmental Sciences & Ecology; Public, Environmental & Occupational"
   Health
GA FU2SD
UT WOS:000423699400067
PM 29207531
"OA Green Submitted, gold"
DA 2026-04-06
ER

PT J
"AU Ye, SJ"
"   Zeng, GM"
"   Wu, HP"
"   Zhang, C"
"   Dai, J"
"   Liang, J"
"   Yu, JF"
"   Ren, XY"
"   Yi, H"
"   Cheng, M"
"   Zhang, C"
"AF Ye, Shujing"
"   Zeng, Guangming"
"   Wu, Haipeng"
"   Zhang, Chang"
"   Dai, Juan"
"   Liang, Jie"
"   Yu, Jiangfang"
"   Ren, Xiaoya"
"   Yi, Huan"
"   Cheng, Min"
"   Zhang, Chen"
TI Biological technologies for the remediation of co-contaminated soil
SO CRITICAL REVIEWS IN BIOTECHNOLOGY
LA English
DT Review
DE Soil remediation; co-contaminated soil; biological technologies;
   mechanisms; heavy metals; organic pollutants
ID POLYCYCLIC AROMATIC-HYDROCARBONS; PERSISTENT FREE-RADICALS;
   HEAVY-METALS; ORGANIC CONTAMINANTS; ENHANCED PHYTOREMEDIATION;
   AQUEOUS-SOLUTIONS; PHANEROCHAETE-CHRYSOSPORIUM; MICROBIAL FORMULA;
   SEDUM-ALFREDII; TOXIC METALS
"AB Compound contamination in soil, caused by unreasonable waste disposal, has attracted increasing attention on a global scale, particularly since multiple heavy metals and/or organic pollutants are entering natural ecosystem through human activities, causing an enormous threat. The remediation of co-contaminated soil is more complicated and difficult than that of single contamination, due to the disparate remediation pathways utilized for different types of pollutants. Several modern remediation technologies have been developed for the treatment of co-contaminated soil. Biological remediation technologies, as the eco-friendly methods, have received widespread concern due to soil improvement besides remediation. This review summarizes the application of biological technologies, which contains microbial technologies (function microbial remediation and composting or compost addition), biochar, phytoremediation technologies, genetic engineering technologies and biochemical technologies, for the remediation of co-contaminated soil with heavy metals and organic pollutants. Mechanisms of these technologies and their remediation efficiencies are also reviewed. Based on this study, this review also identifies the future research required in this field."
"C1 [Ye, Shujing; Zeng, Guangming; Wu, Haipeng; Zhang, Chang; Dai, Juan; Liang, Jie; Yu, Jiangfang; Ren, Xiaoya; Yi, Huan; Cheng, Min; Zhang, Chen] Hunan Univ, Coll Environm Sci & Engn, Changsha, Hunan, Peoples R China."
"   [Ye, Shujing; Zeng, Guangming; Wu, Haipeng; Zhang, Chang; Dai, Juan; Liang, Jie; Yu, Jiangfang; Ren, Xiaoya; Yi, Huan; Cheng, Min; Zhang, Chen] Hunan Univ, Minist Educ, Key Lab Environm Biol & Pollut Control, Changsha, Hunan, Peoples R China."
"   [Wu, Haipeng; Dai, Juan] Changjiang River Sci Res Inst, Wuhan, Hubei, Peoples R China."

C3 Hunan University; Hunan University; Yangtze River Water Resources
Protection Bureau; Changjiang River Scientific Research Institute
"RP Zeng, GM; Wu, HP (corresponding author), Hunan Univ, Coll Environm Sci & Engn, Changsha, Hunan, Peoples R China."
EM zgming@hnu.edu.cn; wuhaipeng0701@126.com
"RI Cheng, Min/AAM-6401-2021; Yi, Huan/NYT-4474-2025; Yu,"
"   Jiangfang/ABD-9643-2021; Ren, Xiaoya/HCH-6468-2022; zhang,"
"   chang/H-5712-2016; guangming, zeng/AHD-0360-2022; Liang,"
"   Jie/P-4649-2015; Zhang, Chen/AAY-8585-2021; Wu, Haipeng/O-2852-2015"
"OI Cheng, Min/0000-0003-1086-4008; Yu, Jiangfang/0000-0002-6529-5263;"
"   Liang, Jie/0000-0002-1979-9356; Zhang, Chen/0000-0002-3579-6980; Wu,"
Haipeng/0000-0002-9888-6959
"FU National Natural Science Foundation of China [51521006, 51479072,"
51378190]; Program for Changjiang Scholars and Innovative Research Team
in University [IRT-13R17]
FX This research was financially supported by the National Natural Science
"   Foundation of China (51521006, 51479072 and 51378190) and the Program"
for Changjiang Scholars and Innovative Research Team in University
(IRT-13R17).
"CR Abhilash PC, 2012, TRENDS BIOTECHNOL, V30, P416, DOI 10.1016/j.tibtech.2012.04.004"
"   Ahmad M, 2012, ECOTOX ENVIRON SAFE, V79, P225, DOI 10.1016/j.ecoenv.2012.01.003"
"   Alisi C, 2009, SCI TOTAL ENVIRON, V407, P3024, DOI 10.1016/j.scitotenv.2009.01.011"
"   Arshad M, 2007, TRENDS BIOTECHNOL, V25, P356, DOI 10.1016/j.tibtech.2007.05.005"
"   Atagana HI, 2011, WATER AIR SOIL POLL, V215, P261, DOI 10.1007/s11270-010-0476-z"
"   Bai HJ, 2008, BIORESOURCE TECHNOL, V99, P7716, DOI 10.1016/j.biortech.2008.01.071"
"   Beesley L, 2014, ENVIRON POLLUT, V186, P195, DOI 10.1016/j.envpol.2013.11.026"
"   Beesley L, 2010, ENVIRON POLLUT, V158, P2282, DOI 10.1016/j.envpol.2010.02.003"
"   Ben Achiba W, 2009, AGR ECOSYST ENVIRON, V130, P156, DOI 10.1016/j.agee.2009.01.001"
"   Bian RJ, 2014, J HAZARD MATER, V272, P121, DOI 10.1016/j.jhazmat.2014.03.017"
"   Braud A, 2009, CHEMOSPHERE, V74, P280, DOI 10.1016/j.chemosphere.2008.09.013"
"   Brennan A, 2014, ENVIRON POLLUT, V193, P79, DOI 10.1016/j.envpol.2014.06.016"
"   Cameselle C, 2013, CHEMOSPHERE, V93, P626, DOI 10.1016/j.chemosphere.2013.06.029"
"   Cao MH, 2013, ENVIRON POLLUT, V174, P93, DOI 10.1016/j.envpol.2012.11.015"
"   Cao XD, 2011, ENVIRON SCI TECHNOL, V45, P4884, DOI 10.1021/es103752u"
"   Cao XD, 2010, BIORESOURCE TECHNOL, V101, P5222, DOI 10.1016/j.biortech.2010.02.052"
"   Cao XD, 2009, ENVIRON SCI TECHNOL, V43, P3285, DOI 10.1021/es803092k"
"   Cheema SA, 2010, J HAZARD MATER, V177, P384, DOI 10.1016/j.jhazmat.2009.12.044"
"   Chen BL, 2012, J SOIL SEDIMENT, V12, P1350, DOI 10.1007/s11368-012-0554-5"
"   Chen BL, 2010, J HAZARD MATER, V179, P845, DOI 10.1016/j.jhazmat.2010.03.082"
"   Chen F, 2016, CHEMOSPHERE, V146, P289, DOI 10.1016/j.chemosphere.2015.12.050"
"   Chen F, 2016, J HAZARD MATER, V302, P250, DOI 10.1016/j.jhazmat.2015.09.068"
"   Chen M, 2015, BIOTECHNOL ADV, V33, P745, DOI 10.1016/j.biotechadv.2015.05.003"
"   Chen R, 2015, J SOIL SEDIMENT, V15, P1083, DOI 10.1007/s11368-015-1093-7"
"   Cheng M, 2016, CHEM ENG J, V284, P582, DOI 10.1016/j.cej.2015.09.001"
"   Choppala GK, 2012, J ENVIRON QUAL, V41, P1175, DOI 10.2134/jeq2011.0145"
"   Clabeaux BL, 2013, ECOTOX ENVIRON SAFE, V98, P236, DOI 10.1016/j.ecoenv.2013.08.014"
"   Deive FJ, 2009, BIORESOURCE TECHNOL, V100, P3630, DOI 10.1016/j.biortech.2009.02.053"
"   Dodgen LK, 2013, ENVIRON POLLUT, V182, P150, DOI 10.1016/j.envpol.2013.06.038"
"   Dong XL, 2014, J HAZARD MATER, V267, P62, DOI 10.1016/j.jhazmat.2013.12.027"
"   Dong ZY, 2013, J HAZARD MATER, V260, P399, DOI 10.1016/j.jhazmat.2013.05.003"
"   Epelde L, 2009, SOIL BIOL BIOCHEM, V41, P1788, DOI 10.1016/j.soilbio.2008.04.001"
"   Fan T, 2008, J HAZARD MATER, V160, P655, DOI 10.1016/j.jhazmat.2008.03.038"
"   Fang GD, 2015, ENVIRON SCI TECHNOL, V49, P5645, DOI 10.1021/es5061512"
"   Fang GD, 2015, BIORESOURCE TECHNOL, V176, P210, DOI 10.1016/j.biortech.2014.11.032"
"   Fang GD, 2014, ENVIRON SCI TECHNOL, V48, P1902, DOI 10.1021/es4048126"
"   Fellet G, 2014, SCI TOTAL ENVIRON, V468, P598, DOI 10.1016/j.scitotenv.2013.08.072"
"   Fellet G, 2011, CHEMOSPHERE, V83, P1262, DOI 10.1016/j.chemosphere.2011.03.053"
"   Feng YA, 2010, CHEM ENG J, V162, P487, DOI 10.1016/j.cej.2010.05.049"
"   Gao YZ, 2011, J HAZARD MATER, V185, P703, DOI 10.1016/j.jhazmat.2010.09.076"
"   Gao YZ, 2010, BIORESOURCE TECHNOL, V101, P6895, DOI 10.1016/j.biortech.2010.03.122"
"   Gao YC, 2014, CHEMOSPHERE, V117, P486, DOI 10.1016/j.chemosphere.2014.08.070"
"   Gill RT, 2014, CHEMOSPHERE, V107, P31, DOI 10.1016/j.chemosphere.2014.03.019"
"   Glick BR, 2010, BIOTECHNOL ADV, V28, P367, DOI 10.1016/j.biotechadv.2010.02.001"
"   Gomez-Eyles JL, 2011, ENVIRON POLLUT, V159, P616, DOI 10.1016/j.envpol.2010.09.037"
"   Gong J, 2009, J HAZARD MATER, p[s2, 1517]"
"   Graber ER, 2012, PLANT SOIL, V353, P95, DOI 10.1007/s11104-011-1012-7"
"   Guo HJ, 2010, BIORESOURCE TECHNOL, V101, P8599, DOI 10.1016/j.biortech.2010.06.085"
"   Guo SH, 2014, CHEMOSPHERE, V109, P226, DOI 10.1016/j.chemosphere.2014.02.007"
"   Hao X, 2014, INT J PHYTOREMEDIAT, V16, P179, DOI 10.1080/15226514.2013.773273"
"   Hirose S, 2005, J AGR FOOD CHEM, V53, P3461, DOI 10.1021/jf050064z"
"   Hoffman DR, 2010, BIORESOURCE TECHNOL, V101, P2672, DOI 10.1016/j.biortech.2009.10.073"
"   Hu XJ, 2011, J HAZARD MATER, V185, P306, DOI 10.1016/j.jhazmat.2010.09.034"
"   Hua L, 2012, ENVIRON TECHNOL, V33, P61, DOI 10.1080/09593330.2010.549845"
"   Huang DL, 2008, ENVIRON SCI TECHNOL, V42, P4946, DOI 10.1021/es800072c"
"   Iqbal M, 2012, ENVIRON POLLUT, V170, P222, DOI 10.1016/j.envpol.2012.07.008"
"   Jiang J, 2015, ECOTOX ENVIRON SAFE, V120, P386, DOI 10.1016/j.ecoenv.2015.06.028"
"   Jiang J, 2012, J HAZARD MATER, V229, P145, DOI 10.1016/j.jhazmat.2012.05.086"
"   Jin J, 2014, SCI TOTAL ENVIRON, V473, P308, DOI 10.1016/j.scitotenv.2013.12.033"
"   Jin QJ, 2013, PLANT CELL ENVIRON, V36, P956, DOI 10.1111/pce.12029"
"   Karami N, 2011, J HAZARD MATER, V191, P41, DOI 10.1016/j.jhazmat.2011.04.025"
"   Kemper JM, 2008, ENVIRON SCI TECHNOL, V42, P2118, DOI 10.1021/es702402a"
"   Kobayashi T, 2009, SCI TOTAL ENVIRON, V407, P5805, DOI 10.1016/j.scitotenv.2009.06.041"
"   Kohler J, 2015, SCI TOTAL ENVIRON, V514, P42, DOI 10.1016/j.scitotenv.2015.01.085"
"   Kookana RS, 2011, ADV AGRON, V112, P103, DOI 10.1016/B978-0-12-385538-1.00003-2"

"   Kutrowska A, 2014, ACTA PHYSIOL PLANT, V36, P1957, DOI 10.1007/s11738-014-1576-y"
"   Lamon L, 2009, ENVIRON POLLUT, V157, P1971, DOI 10.1016/j.envpol.2009.02.016"
"   Lee KY, 2012, ENVIRON GEOCHEM HLTH, V34, P135, DOI 10.1007/s10653-011-9406-2"
"   Lee KY, 2011, ENVIRON GEOCHEM HLTH, V33, P3, DOI 10.1007/s10653-010-9359-x"
"   Lee SH, 2008, J HAZARD MATER, V153, P892, DOI 10.1016/j.jhazmat.2007.09.041"
"   Lim, 2012, J HAZARD MATER, P209"
"   Limcharoensuk T, 2015, ECOTOX ENVIRON SAFE, V122, P322, DOI 10.1016/j.ecoenv.2015.08.013"
"   Liu HY, 2015, J HAZARD MATER, V294, P121, DOI 10.1016/j.jhazmat.2015.04.004"
"   Liu J, 2008, BIODEGRADATION, V19, P695, DOI 10.1007/s10532-007-9174-y"
"   Lu M, 2014, PLANT SOIL, V382, P89, DOI 10.1007/s11104-014-2147-0"
"   Ma JW, 2010, J HAZARD MATER, V176, P715, DOI 10.1016/j.jhazmat.2009.11.093"
"   Ma Y, 2011, BIOTECHNOL ADV, V29, P248, DOI 10.1016/j.biotechadv.2010.12.001"
"   Maestri E, 2010, ENVIRON EXP BOT, V68, P1, DOI 10.1016/j.envexpbot.2009.10.011"
"   Onta??on OM, 2014, INT BIODETER BIODEGR, V88, P192, DOI 10.1016/j.ibiod.2013.10.017"
"   Mandal A, 2016, SCI TOTAL ENVIRON, V550, P611, DOI 10.1016/j.scitotenv.2016.01.164"
"   Many?? JJ, 2012, ENVIRON SCI TECHNOL, V46, P7939, DOI 10.1021/es301029g"
"   Masip L, 2006, ANTIOXID REDOX SIGN, V8, P753, DOI 10.1089/ars.2006.8.753"
"   Melo LCA, 2016, J SOIL SEDIMENT, V16, P226, DOI 10.1007/s11368-015-1199-y"
"   Park JH, 2011, J HAZARD MATER, V185, P549, DOI 10.1016/j.jhazmat.2010.09.082"
"   P??rez DV, 2007, BIORESOURCE TECHNOL, V98, P525, DOI 10.1016/j.biortech.2006.02.025"
"   Plaza C, 2009, ENVIRON POLLUT, V157, P257, DOI 10.1016/j.envpol.2008.07.016"
"   Qiu ZQ, 2014, J HAZARD MATER, V267, P17, DOI 10.1016/j.jhazmat.2013.12.043"
"   Rajkumar M, 2010, TRENDS BIOTECHNOL, V28, P142, DOI 10.1016/j.tibtech.2009.12.002"
"   Sandrin TR, 2000, APPL ENVIRON MICROB, V66, P4585, DOI 10.1128/AEM.66.10.4585-4588.2000"
"   Shahid M, 2014, REV ENVIRON CONTAM T, V232, P1, DOI 10.1007/978-3-319-06746-9_1"
"   Shen ZT, 2016, SCI TOTAL ENVIRON, V542, P771, DOI 10.1016/j.scitotenv.2015.10.057"
"   Shih YH, 2011, APPL CATAL B-ENVIRON, V105, P24, DOI 10.1016/j.apcatb.2011.03.024"
"   Sprocati AR, 2012, PROCESS BIOCHEM, V47, P1649, DOI 10.1016/j.procbio.2011.10.001"
"   Sun K, 2012, BIORESOURCE TECHNOL, V118, P120, DOI 10.1016/j.biortech.2012.05.008"
"   Sun YB, 2011, J HAZARD MATER, V186, P2075, DOI 10.1016/j.jhazmat.2010.12.116"
"   Sung MH, 2011, J HAZARD MATER, V190, P744, DOI 10.1016/j.jhazmat.2011.03.113"
"   Tang L, 2008, ENVIRON SCI TECHNOL, V42, P1207, DOI 10.1021/es7024593"
"   Tang WW, 2014, SCI TOTAL ENVIRON, V468, P1014, DOI 10.1016/j.scitotenv.2013.09.044"
"   Tejada M, 2009, GEODERMA, V153, P254, DOI 10.1016/j.geoderma.2009.08.009"
"   Teng Y, 2015, FRONT PLANT SCI, V6, DOI [10.20546/ijcmas.2018.703.200, 10.3389/fpls.2015.00032]"
"   Thavamani P, 2015, ENVIRON SCI POLLUT R, V22, P8957, DOI 10.1007/s11356-013-1850-3"
"   Thion C, 2012, INT BIODETER BIODEGR, V68, P28, DOI 10.1016/j.ibiod.2011.10.012"
"   Tripathi V, 2015, ECOL ENG, V82, P330, DOI 10.1016/j.ecoleng.2015.05.027"
"   Uchimiya M, 2012, J AGR FOOD CHEM, V60, P2989, DOI 10.1021/jf205110g"
"   Villa RD, 2008, CHEMOSPHERE, V71, P43, DOI 10.1016/j.chemosphere.2007.10.043"
"   Wang CP, 2014, J HAZARD MATER, V264, P439, DOI 10.1016/j.jhazmat.2013.10.073"
"   Wang CP, 2012, IND ENG CHEM RES, V51, P4397, DOI 10.1021/ie2023096"
"   Wang T, 2014, J HAZARD MATER, V278, P483, DOI 10.1016/j.jhazmat.2014.06.028"
"   Wang WY, 2014, MAR POLLUT BULL, V84, P306, DOI 10.1016/j.marpolbul.2014.04.046"
"   Wang XL, 2007, ENVIRON SCI TECHNOL, V41, P8342, DOI 10.1021/es071290n"
"   Wang ZY, 2011, J HAZARD MATER, V190, P677, DOI 10.1016/j.jhazmat.2011.03.103"
"   Weyens N, 2011, INT J PHYTOREMEDIAT, V13, P244, DOI 10.1080/15226511003753920"
"   Wild E, 2005, ENVIRON SCI TECHNOL, V39, P3695, DOI 10.1021/es048136a"
"   Wu HP, 2017, CRIT REV BIOTECHNOL, V37, P754, DOI [10.24966/ctb-9107/100009, 10.1080/07388551.2016.1232696]"
"   Xiao WD, 2013, ENVIRON SCI POLLUT R, V20, P380, DOI 10.1007/s11356-012-0902-4"
"   Xu NN, 2013, BIORESOURCE TECHNOL, V149, P22, DOI 10.1016/j.biortech.2013.09.024"
"   Xu PA, 2012, SCI TOTAL ENVIRON, V424, P1, DOI 10.1016/j.scitotenv.2012.02.023"
"   Xu WQ, 2013, ENVIRON SCI TECHNOL, V47, P7129, DOI 10.1021/es4012367"
"   Yan DYS, 2013, ENVIRON POLLUT, V178, P15, DOI 10.1016/j.envpol.2013.02.030"
"   Yang X, 2016, ENVIRON SCI POLLUT R, V23, P974, DOI 10.1007/s11356-015-4233-0"
"   Yu Z, 2011, PROCESS BIOCHEM, V46, P1285, DOI 10.1016/j.procbio.2011.02.018"
"   Zeng G., 2013, NATURE, V499, P239"
"   Zeng GM, 2015, RSC ADV, V5, P34541, DOI 10.1039/c5ra04834f"
"   Zeng GM, 2013, SCIENCE, V340, P1403, DOI 10.1126/science.340.6139.1403-a"
"   Zhang P, 2013, J HAZARD MATER, V244, P217, DOI 10.1016/j.jhazmat.2012.11.046"
"   Zhang WH, 2007, J ENVIRON ENG, V133, P548, DOI 10.1061/(ASCE)0733-9372(2007)133:5(548)"
"   Zhang XK, 2013, ENVIRON SCI POLLUT R, V20, P8472, DOI 10.1007/s11356-013-1659-0"
"   Zhang Y, 2007, BIOSENS BIOELECTRON, V22, P2121, DOI 10.1016/j.bios.2006.09.030"
"   Zhang YY, 2013, J HAZARD MATER, V260, P1100, DOI 10.1016/j.jhazmat.2013.06.065"
"   Zheng ZM, 2002, ENZYME MICROB TECH, V31, P3, DOI 10.1016/S0141-0229(02)00091-1"
"   Zhu ZQ, 2012, J HAZARD MATER, p[235, 144]"
NR 131
TC 416
Z9 454
U1 39
U2 1524
PU TAYLOR & FRANCIS LTD
PI ABINGDON
"PA 2-4 PARK SQUARE, MILTON PARK, ABINGDON OR14 4RN, OXON, the United Kingdom"
SN 0738-8551
EI 1549-7801
J9 CRIT REV BIOTECHNOL
JI Crit. Rev. Biotechnol.
PY 2017
VL 37
IS 8
BP 1062
EP 1076

DI 10.1080/07388551.2017.1304357
PG 15
WC Biotechnology & Applied Microbiology
WE Science Citation Index Expanded (SCI-EXPANDED)
SC Biotechnology & Applied Microbiology
GA FH3UT
UT WOS:000411076200008
PM 28427272
DA 2026-04-06
ER

PT J
"AU Ali, M"
"   Song, X"
"   Ding, D"
"   Wang, Q"
"   Zhang, ZX"
"   Tang, ZW"
"AF Ali, Mukhtiar"
"   Song, Xin"
"   Ding, Da"
"   Wang, Qing"
"   Zhang, Zhuanxia"
"   Tang, Zhiwen"
TI Bioremediation of PAHs and heavy metals co-contaminated soils:
   Challenges and enhancement strategies
SO ENVIRONMENTAL POLLUTION
LA English
DT Article
DE Co-contamination; Polycyclic aromatic hydrocarbons (PAHs); Heavy metals;
   Bioremediation; Cation-pi interactions
ID POLYCYCLIC AROMATIC-HYDROCARBONS; CATION-PI INTERACTIONS; LONG-TERM;
   POLLUTED SOIL; URBAN SOILS; ELECTROKINETIC REMEDIATION; RHAMNOLIPID
   BIOSURFACTANT; BACKGROUND CONCENTRATIONS; ANTIOXIDANT RESPONSES;
   SIMULTANEOUS REMOVAL
"AB Systemic studies on the bioremediation of co-contaminated PAHs and heavy metals are lacking, and this paper provides an in-depth review on the topic. The released sources and transport of co-contaminated PAHs and heavy metals, including their co-occurrence through formation of cation-pi interactions and their adsorption in soil are examined. Moreover, it is investigated that co-contamination of PAHs and heavy metals can drive a synergistic positive influence on bioremediation through enhanced secretion of extracellular polymeric substances (EPSs), production of biosynthetic genes, organic acid and enzymatic proliferation. However, PAHs molecular structure, PAHs-heavy metals bioavailability and their interactive cytotoxic effects on microorganisms can exert a challenging influence on the bioremediation under co-contaminated conditions. The fluctuations in bioavailability for microorganisms are associated with soil properties, chemical coordinative interactions, and biological activities under the co-contaminated PAHs-heavy metals conditions. The interactive cytotoxicity caused by the emergence of co-contaminants includes microbial cell disruption, denaturation of DNA and protein structure, and deregulation of antioxidant biological molecules. Finally, this paper presents the emerging strategies to overcome the bioavailability problems and recommends the use of biostimulation and bioaugmentation along with the microbial immobilization for enhanced bioremediation of PAHs-heavy metals co-contaminated sites. Better knowledge of the bioremediation potential is imperative to improve the use of these approaches for the sustainable and cost-effective remediation of PAHs and heavy metals co-contamination in the near future."
"C1 [Ali, Mukhtiar; Song, Xin; Wang, Qing; Zhang, Zhuanxia; Tang, Zhiwen] Chinese Acad Sci, Inst Soil Sci, Key Lab Soil Environm & Pollut Remediat, Nanjing 210008, Peoples R China."
"   [Ali, Mukhtiar; Song, Xin; Zhang, Zhuanxia; Tang, Zhiwen] Univ Chinese Acad Sci, Beijing 100049, Peoples R China."
"   [Ding, Da] Minist Ecol & Environm Peoples Republ China, Nanjing Inst Environm Sci, Nanjing 210042, Peoples R China."
"C3 Chinese Academy of Sciences; Nanjing Institute of Soil Science, CAS;"
"   Chinese Academy of Sciences; University of Chinese Academy of Sciences,"
"   CAS; Nanjing Institute of Environmental Sciences, Ministry of Ecology &"
   Environment
"RP Song, X (corresponding author), Chinese Acad Sci, Inst Soil Sci, Key Lab Soil Environm & Pollut Remediat, Nanjing 210008, Peoples R China."
EM xsong@issas.ac.cn
"RI Ali, Mukhtiar/LQK-8623-2024; Ding, Da/JLM-4086-2023"
"OI Ding, Da/0000-0002-6221-4464; Ali, Mukhtiar/0000-0003-2366-4427"
"CR Abdel-Shafy Hussein I., 2016, Egyptian Journal of Petroleum, V25, P107, DOI [10.1016/j.ejpe.2015.03.011, 10.1016/j.ejpe.2015.03.011]"
"   Aguilar-May B, 2007, J APPL PHYCOL, V19, P181, DOI 10.1007/s10811-006-9132-9"
"   Akdogan HA, 2011, PROCESS BIOCHEM, V46, P840, DOI 10.1016/j.procbio.2011.01.008"
"   Al Ali S, 2017, ENVIRON SCI POLLUT R, V24, P26497, DOI 10.1007/s11356-017-0238-1"
"   Alaba PA, 2018, J ENVIRON CHEM ENG, V6, P1651, DOI 10.1016/j.jece.2018.02.019"
"   Ali N, 2020, SCI TOTAL ENVIRON, V704, DOI 10.1016/j.scitotenv.2019.135391"
"   Almeida CMR, 2008, MAR ENVIRON RES, V66, P311, DOI 10.1016/j.marenvres.2008.04.005"
"   Alves D, 2019, J ENVIRON MANAGE, V239, P30, DOI 10.1016/j.jenvman.2019.03.028"
"   Ammami MT, 2015, CHEMOSPHERE, V125, P1, DOI 10.1016/j.chemosphere.2014.12.087"
"   Andreolli M, 2015, J ENVIRON MANAGE, V153, P121, DOI 10.1016/j.jenvman.2015.02.007"
"   Aschi M, 2002, J MOL STRUC-THEOCHEM, V587, P177, DOI 10.1016/S0166-1280(02)00112-4"
"   Asemoloye MD, 2017, J ENVIRON MANAGE, V200, P253, DOI 10.1016/j.jenvman.2017.05.090"
"   Ashraf U, 2020, CHEMOSPHERE, V242, DOI 10.1016/j.chemosphere.2019.125160"
"   Atagana HI, 2006, WORLD J MICROB BIOT, V22, P1145, DOI 10.1007/s11274-006-9155-z"
"   Azzolina NA, 2016, ENVIRON FORENSICS, V17, P294, DOI 10.1080/15275922.2016.1230905"
"   Bacosa HP, 2015, J HAZARD MATER, V283, P689, DOI 10.1016/j.jhazmat.2014.09.068"

"   Bae SY, 2002, ATMOS ENVIRON, V36, P5491, DOI 10.1016/S1352-2310(02)00666-0"
"   Baldrian P, 2000, APPL ENVIRON MICROB, V66, P2471, DOI 10.1128/AEM.66.6.2471-2478.2000"
"   Bamforth SM, 2005, J CHEM TECHNOL BIOT, V80, P723, DOI 10.1002/jctb.1276"
"   Baraga??o D, 2020, CHEM ENG J, V399, DOI 10.1016/j.cej.2020.125809"
"   Bascik-Remisiewicz A, 2011, ARCH ENVIRON CON TOX, V60, P610, DOI 10.1007/s00244-010-9585-3"
"   Perini BLB, 2020, J ENVIRON CHEM ENG, V8, DOI 10.1016/j.jece.2020.104013"
"   Bi CL, 2020, CHEMOSPHERE, V238, DOI 10.1016/j.chemosphere.2019.124620"
"   Biswas B, 2020, MICROB BIOTECHNOL, V13, P522, DOI 10.1111/1751-7915.13510"
"   Biswas B, 2015, J HAZARD MATER, V298, P129, DOI 10.1016/j.jhazmat.2015.05.009"
"   Blokhina O, 2003, ANN BOT-LONDON, V91, P179, DOI 10.1093/aob/mcf118"
"   Booth SC, 2015, FRONT MICROBIOL, V6, DOI 10.3389/fmicb.2015.00827"
"   BROWN NL, 1992, PLASMID, V27, P41, DOI 10.1016/0147-619X(92)90005-U"
"   Calvo C, 2008, J IND MICROBIOL BIOT, V35, P1493, DOI 10.1007/s10295-008-0451-5"
"   CARRELL CJ, 1994, CARCINOGENESIS, V15, P2931, DOI 10.1093/carcin/15.12.2931"
"   Chakravarty R, 2012, BIORESOURCE TECHNOL, V108, P176, DOI 10.1016/j.biortech.2011.12.100"
"   Chen C, 2016, ENVIRON SCI POLLUT R, V23, P6861, DOI 10.1007/s11356-015-5926-0"
"   Chen F, 2016, J HAZARD MATER, V302, P250, DOI 10.1016/j.jhazmat.2015.09.068"
"   Chen J, 2020, ENVIRON SCI TECHNOL, V54, P7261, DOI 10.1021/acs.est.0c00363"
"   Chen SN, 2014, BIORESOURCE TECHNOL, V158, P181, DOI 10.1016/j.biortech.2014.02.020"
"   Chen TF, 2016, J ENVIRON ENG SCI, V11, P7, DOI [10.1680/jenes.16.00002, DOI 10.1680/jenes.16.00002, 10.1680/jenes.16.00002, DOI 10.1680/JENES.16.00002]"
"   Chen X, 2017, J HAZARD MATER, V325, P319, DOI 10.1016/j.jhazmat.2016.12.009"
"   Chen Z, 2020, J HAZARD MATER, V395, DOI 10.1016/j.jhazmat.2020.122665"
"   Cheng QL, 2020, J HAZARD MATER, V395, DOI 10.1016/j.jhazmat.2020.122644"
"   Chettri B, 2019, BIORESOURCE TECHNOL, V294, DOI 10.1016/j.biortech.2019.122190"
"   Chrzanowski L, 2012, BIORESOURCE TECHNOL, V111, P328, DOI 10.1016/j.biortech.2012.01.181"
"   Ciarkowska K, 2019, CHEMOSPHERE, V229, P214, DOI 10.1016/j.chemosphere.2019.04.209"
"   Lerner JEC, 2018, ENVIRON SCI POLLUT R, V25, P8487, DOI 10.1007/s11356-017-1101-0"
"   Cormier SA, 2006, ENVIRON HEALTH PERSP, V114, P810, DOI 10.1289/ehp.8629"
"   Cristaldi A, 2017, ENVIRON TECHNOL INNO, V8, P309, DOI 10.1016/j.eti.2017.08.002"
"   Czarny J, 2020, J HAZARD MATER, V383, DOI 10.1016/j.jhazmat.2019.121168"
"   da Silva EB, 2020, ENVIRON POLLUT, V264, DOI 10.1016/j.envpol.2020.114737"
"   Dai YY, 2020, ENVIRON INT, V136, DOI 10.1016/j.envint.2019.105421"
"   Davin M, 2020, INT J ENV RES PUB HE, V17, DOI 10.3390/ijerph17114016"
"   Martins PSD, 2008, BRAZ J MICROBIOL, V39, P780, DOI [10.1590/S1517-83822008000400034, 10.1590/S1517-838220080004000034]"
"   Deary ME, 2016, J HAZARD MATER, V307, P240, DOI 10.1016/j.jhazmat.2015.12.015"
"   do Vale TO, 2020, FUEL, V280, DOI 10.1016/j.fuel.2020.118512"
"   El-Mufleh A, 2014, ENVIRON SCI POLLUT R, V21, P9764, DOI 10.1007/s11356-014-2850-7"
"   Elgh-Dalgren K, 2009, J HAZARD MATER, V172, P1033, DOI 10.1016/j.jhazmat.2009.07.092"
"   Elyamine AM, 2019, INT J MOL SCI, V20, DOI 10.3390/ijms20092189"
"   Emoyan OO, 2018, GLOBAL NEST J, V20, P181, DOI 10.30955/gnj.002277"
"   Falciglia PP, 2019, CLEAN TECHNOL ENVIR, V21, P655, DOI 10.1007/s10098-019-01665-5"
"   Fijalkowska S, 1998, J BASIC MICROB, V38, P361"
"   Flemming H.C., 1999, Biofilms in the Aquatic Environment, P1, DOI DOI 10.1007/978-3-642-60147-7"
"   Fonseca B, 2011, CHEM ENG J, V167, P84, DOI 10.1016/j.cej.2010.12.004"
"   Franzetti A, 2009, CHEMOSPHERE, V75, P801, DOI 10.1016/j.chemosphere.2008.12.052"
"   Fry KL, 2020, ENVIRON POLLUT, V262, DOI 10.1016/j.envpol.2020.114235"
"   Gao P, 2021, ENVIRON SCI TECHNOL, V55, P2790, DOI 10.1021/acs.est.0c07033"
"   Gao P, 2019, SCI TOTAL ENVIRON, V685, P463, DOI 10.1016/j.scitotenv.2019.05.403"
"   Gao P, 2019, SCI TOTAL ENVIRON, V670, P800, DOI 10.1016/j.scitotenv.2019.03.247"
"   Gao P, 2018, CRIT REV ENV SCI TEC, V48, P923, DOI 10.1080/10643389.2018.1495983"
"   Gao P, 2018, ENVIRON INT, V119, P466, DOI 10.1016/j.envint.2018.07.017"
"   Gao QT, 2011, BIORESOURCE TECHNOL, V102, P10230, DOI 10.1016/j.biortech.2011.08.070"
"   Gao YZ, 2006, CHEMOSPHERE, V65, P1355, DOI 10.1016/j.chemosphere.2006.04.030"
"   Garc??a-Delgado C, 2015, J HAZARD MATER, V300, P281, DOI 10.1016/j.jhazmat.2015.07.008"
"   Gauthier PT, 2014, AQUAT TOXICOL, V154, P253, DOI 10.1016/j.aquatox.2014.05.026"
"   Gbeddy G, 2020, ENVIRON POLLUT, V257, DOI 10.1016/j.envpol.2019.113510"
"   Giller KE, 2009, SOIL BIOL BIOCHEM, V41, P2031, DOI 10.1016/j.soilbio.2009.04.026"
"   Gitaric J, 2020, POLYHEDRON, V188, DOI 10.1016/j.poly.2020.114688"
"   Gonz??lez AG, 2010, J COLLOID INTERF SCI, V350, P305, DOI 10.1016/j.jcis.2010.06.020"
"   Guarino C, 2019, SCI REP-UK, V9, DOI 10.1038/s41598-019-48005-7"
"   Haleyur N, 2019, J ENVIRON MANAGE, V238, P49, DOI 10.1016/j.jenvman.2019.02.115"
"   Heim S, 2004, ORG GEOCHEM, V35, P1409, DOI 10.1016/j.orggeochem.2004.03.008"
"   Hino S, 1997, J FERMENT BIOENG, V84, P528, DOI 10.1016/S0922-338X(97)81906-X"
"   Hou Y, 2016, INT J ENVIRON SCI TE, V13, P887, DOI 10.1007/s13762-015-0927-6"
"   Hou YY, 2015, ENVIRON SCI POLLUT R, V22, P17780, DOI 10.1007/s11356-015-4995-4"
"   Hrenovic J, 2007, CENT EUR J BIOL, V2, P405, DOI 10.2478/s11535-007-0029-7"
"   Hua XF, 2010, CHEMOSPHERE, V80, P951, DOI 10.1016/j.chemosphere.2010.05.002"
"   Huang YL, 2019, INT BIODETER BIODEGR, V138, P78, DOI 10.1016/j.ibiod.2019.01.006"
"   Huguenot D, 2015, J ENVIRON MANAGE, V153, P40, DOI 10.1016/j.jenvman.2015.01.037"
"   Hwang HM, 2008, J ENVIRON SCI HEAL A, V43, P1243, DOI 10.1080/10934520802177771"
"   Islam MN, 2019, ENVIRON TECHNOL, V40, P125, DOI 10.1080/09593330.2017.1380713"
"   Janus A, 2020, ENVIRON SCI POLLUT R, V27, P1852, DOI 10.1007/s11356-019-06764-w"
"   Ji XW, 2019, ATMOS CHEM PHYS, V19, P13789, DOI 10.5194/acp-19-13789-2019"
"   Jia H, 2016, MAR POLLUT BULL, V110, P112, DOI 10.1016/j.marpolbul.2016.06.075"
"   Jiang J, 2015, ECOTOX ENVIRON SAFE, V120, P386, DOI 10.1016/j.ecoenv.2015.06.028"
"   Johnsen AR, 2005, ENVIRON POLLUT, V133, P71, DOI 10.1016/j.envpol.2004.04.015"
"   Karthick A, 2019, J ENVIRON MANAGE, V243, P187, DOI 10.1016/j.jenvman.2019.04.092"
"   Kazy SK, 2002, WORLD J MICROB BIOT, V18, P583, DOI 10.1023/A:1016354713289"
"   Kazy SK, 1999, WORLD J MICROB BIOT, V15, P599, DOI 10.1023/A:1008997718811"
"   Khan Z, 2015, APPL MICROBIOL BIOT, V99, P10745, DOI 10.1007/s00253-015-6901-x"
"   Khatoon K, 2019, HELIYON, V5, DOI 10.1016/j.heliyon.2019.e02742"
"   Kim H, 2020, ENVIRON ENG RES, V25, P659, DOI 10.4491/eer.2019.233"

"   Kim I, 2019, ENVIRON GEOCHEM HLTH, V41, P391, DOI 10.1007/s10653-018-0139-3"
"   Koproch N, 2019, CHEMOSPHERE, V217, P166, DOI 10.1016/j.chemosphere.2018.10.153"
"   Kotoky R, 2020, CHEMOSPHERE, V258, DOI 10.1016/j.chemosphere.2020.127175"
"   Kumar K, 2018, CHEM SCI, V9, P2655, DOI 10.1039/c7sc04905f"
"   Kuppusamy S, 2017, ENVIRON EARTH SCI, V76, DOI 10.1007/s12665-016-6337-3"
"   Kuppusamy S, 2016, CHEMOSPHERE, V162, P31, DOI 10.1016/j.chemosphere.2016.07.052"
"   Kuppusamy S, 2016, J HAZARD MATER, V317, P169, DOI 10.1016/j.jhazmat.2016.05.066"
"   Kuppusamy S, 2016, J HAZARD MATER, V307, P99, DOI 10.1016/j.jhazmat.2015.12.068"
"   Kuppusamy S, 2016, WATER AIR SOIL POLL, V227, DOI 10.1007/s11270-015-2709-7"
"   Lan GH, 2019, FRESEN ENVIRON BULL, V28, P3917"
"   Lee SC, 2008, BIORESOURCE TECHNOL, V99, P2288, DOI 10.1016/j.biortech.2007.05.020"
"   Lei P, 2016, ENVIRON SCI POLLUT R, V23, P22072, DOI 10.1007/s11356-016-7467-6"
"   Leung HM, 2013, PEDOSPHERE, V23, P549, DOI 10.1016/S1002-0160(13)60049-1"
"   Li GX, 2017, RSC ADV, V7, P31018, DOI 10.1039/c7ra02105d"
"   Li J, 2020, ENVIRON POLLUT, V266, DOI 10.1016/j.envpol.2020.115217"
"   Li L, 2020, BMC BIOTECHNOL, V20, DOI 10.1186/s12896-020-00613-2"
"   Li XN, 2019, ENVIRON POLLUT, V255, DOI 10.1016/j.envpol.2019.113312"
"   Liang X, 2016, J SOIL SEDIMENT, V16, P1509, DOI 10.1007/s11368-015-1341-x"
"   Ling WT, 2010, J SOIL SEDIMENT, V10, P799, DOI 10.1007/s11368-010-0187-5"
"   Lipinska A, 2019, WATER AIR SOIL POLL, V230, DOI 10.1007/s11270-019-4344-1"
"   Liu J, 2018, PLANT SOIL, V425, P335, DOI 10.1007/s11104-018-3575-z"
"   Liu SH, 2019, SCI TOTAL ENVIRON, V655, P1279, DOI 10.1016/j.scitotenv.2018.11.272"
"   Liu SH, 2017, BIORESOURCE TECHNOL, V224, P25, DOI 10.1016/j.biortech.2016.11.095"
"   Liu XR, 2019, MAR POLLUT BULL, V142, P419, DOI 10.1016/j.marpolbul.2019.03.064"
"   Liu YG, 2019, CHEMOSPHERE, V214, P220, DOI 10.1016/j.chemosphere.2018.09.119"
"   Lors C, 2012, ENVIRON POLLUT, V165, P11, DOI 10.1016/j.envpol.2012.02.004"
"   Louzon M, 2020, SCI TOTAL ENVIRON, V721, DOI 10.1016/j.scitotenv.2020.137789"
"   Lu C, 2019, ENVIRON POLLUT, V251, P773, DOI 10.1016/j.envpol.2019.05.044"
"   Lv D, 2018, INT J ENV RES PUB HE, V15, DOI 10.3390/ijerph15040698"
"   Ma JC, 1997, CHEM REV, V97, P1303, DOI 10.1021/cr9603744"
"   Ma XK, 2014, APPL MICROBIOL BIOT, V98, P9817, DOI 10.1007/s00253-014-5905-2"
"   Ma YS, 2018, CHEM ENG J, V353, P410, DOI 10.1016/j.cej.2018.07.131"
"   Madrid F, 2019, SCI TOTAL ENVIRON, V660, P705, DOI 10.1016/j.scitotenv.2018.12.376"
"   Madrid F, 2016, SCI TOTAL ENVIRON, V571, P42, DOI 10.1016/j.scitotenv.2016.07.110"
"   Madrid F, 2019, SCI TOTAL ENVIRON, V653, P384, DOI 10.1016/j.scitotenv.2018.10.316"
"   Mahadevi AS, 2013, CHEM REV, V113, P2100, DOI 10.1021/cr300222d"
"   Makkar RS, 2003, ENVIRON TOXICOL CHEM, V22, P2280, DOI 10.1897/02-472"
"   Malawska M, 2001, WATER AIR SOIL POLL, V127, P339, DOI 10.1023/A:1005236016074"
"   Mangwani N, 2017, PEDOSPHERE, V27, P548, DOI 10.1016/S1002-0160(17)60350-3"
"   Reis APM, 2016, SCI TOTAL ENVIRON, V573, P324, DOI 10.1016/j.scitotenv.2016.08.119"
"   Mehr MR, 2020, CONT SHELF RES, V193, DOI 10.1016/j.csr.2019.104036"
"   Merhaby D, 2019, MAR POLLUT BULL, V149, DOI 10.1016/j.marpolbul.2019.110646"
"   Meyer T, 2009, ENVIRON SCI TECHNOL, V43, P657, DOI 10.1021/es8020217"
"   Meyer T, 2011, WATER RES, V45, P1147, DOI 10.1016/j.watres.2010.11.004"
"   Min BK, 2018, B KOREAN CHEM SOC, V39, P1047, DOI 10.1002/bkcs.11548"
"   Moeckel C, 2020, ENVIRON INT, V137, DOI 10.1016/j.envint.2020.105563"
"   Mohammadi A, 2019, INT J ENVIRON SCI TE, V16, P181, DOI 10.1007/s13762-017-1564-z"
"   Mohite BV, 2018, APPL BIOCHEM BIOTECH, V186, P199, DOI 10.1007/s12010-018-2727-1"
"   Mouvenchery YK, 2018, J PLANT NUTR SOIL SC, V181, P453, DOI 10.1002/jpln.201600506"
"   M??hlbachov?? G, 2011, ECOL ENG, V37, P1064, DOI 10.1016/j.ecoleng.2010.08.004"
"   M??ller S, 2007, EUR J SOIL SCI, V58, P918, DOI 10.1111/j.1365-2389.2007.00930.x"
"   Nguyen XP, 2013, ENG GEOL, V166, P52, DOI 10.1016/j.enggeo.2013.08.009"
"   Obi L, 2020, J CHEM TECHNOL BIOT, V95, P1569, DOI 10.1002/jctb.6352"
"   Obuekwe IS, 2013, J SOIL SEDIMENT, V13, P1589, DOI 10.1007/s11368-013-0759-2"
"   Obuekwe IS, 2013, ENVIRON POLLUT, V180, P180, DOI 10.1016/j.envpol.2013.05.020"
"   Pajewski R, 2007, NEW J CHEM, V31, P1960, DOI 10.1039/b705179b"
"   Pan XL, 2010, COLLOID SURFACE B, V80, P103, DOI 10.1016/j.colsurfb.2010.05.002"
"   Paneque P, 2020, J HAZARD MATER, V389, DOI 10.1016/j.jhazmat.2019.121820"
"   Patel AB, 2019, BIORESOURCE TECHNOL, V284, P115, DOI 10.1016/j.biortech.2019.03.097"
"   Peng C, 2013, ENVIRON POLLUT, V178, P426, DOI 10.1016/j.envpol.2013.03.058"
"   Ping LF, 2006, ENVIRON GEOCHEM HLTH, V28, P189, DOI 10.1007/s10653-005-9030-0"
"   Piscitelli L, 2019, SOIL RES, V57, P807, DOI 10.1071/SR19075"
"   Priyadarshini E, 2021, CHEMOSPHERE, V274, DOI 10.1016/j.chemosphere.2021.129976"
"   Qu XL, 2008, ENVIRON SCI TECHNOL, V42, P1109, DOI 10.1021/es071613f"
"   Qu XL, 2007, ENVIRON SCI TECHNOL, V41, P8321, DOI 10.1021/es0718117"
"   Qu YJ, 2020, ENVIRON POLLUT, V260, DOI 10.1016/j.envpol.2020.114016"
"   Rabani MS, 2022, POLYCYCL AROMAT COMP, V42, P978, DOI 10.1080/10406638.2020.1759663"
"   Rachmilevitch S, 2007, PHYSIOL PLANTARUM, V129, P163, DOI 10.1111/j.1399-3054.2006.00784.x"
"   Rachwal M, 2015, CHEMOSPHERE, V138, P863, DOI 10.1016/j.chemosphere.2014.11.077"
"   Rajendran S, 2022, CHEMOSPHERE, V287, DOI 10.1016/j.chemosphere.2021.132369"
"   Raza W, 2011, BIORESOURCE TECHNOL, V102, P6095, DOI 10.1016/j.biortech.2011.02.033"
"   Reddy KR, 2019, INT J GEOTECH ENG, V13, P32, DOI 10.1080/19386362.2017.1318231"
"   Redfern LK, 2019, J HAZARD MATER, V378, DOI 10.1016/j.jhazmat.2019.120859"
"   Rodr??guez-Morgado B, 2015, ENVIRON TECHNOL, V36, P2217, DOI 10.1080/09593330.2015.1024760"
"   Rosner A, 2021, SCI TOTAL ENVIRON, V771, DOI 10.1016/j.scitotenv.2020.144565"
"   Saeedi M, 2019, J ENVIRON MANAGE, V248, DOI 10.1016/j.jenvman.2019.07.029"
"   Saeedi M, 2018, ENVIRON EARTH SCI, V77, DOI 10.1007/s12665-018-7489-0"
"   Saint-Denis M, 2001, SOIL BIOL BIOCHEM, V33, P395, DOI 10.1016/S0038-0717(00)00177-2"
"   Saison C, 2004, CHEMOSPHERE, V55, P477, DOI 10.1016/j.chemosphere.2003.10.059"
"   Sandrin TR, 2000, APPL ENVIRON MICROB, V66, P4585, DOI 10.1128/AEM.66.10.4585-4588.2000"
"   Sandrin TR, 2003, ENVIRON HEALTH PERSP, V111, P1093, DOI 10.1289/ehp.5840"
"   Santos EC, 2008, BIORESOURCE TECHNOL, V99, P2644, DOI 10.1016/j.biortech.2007.04.050"
"   Sarkar B, 2012, CRIT REV ENV SCI TEC, V42, P435, DOI 10.1080/10643389.2010.518524"
"   Sarma H, 2019, ECOTOX ENVIRON SAFE, V176, P288, DOI 10.1016/j.ecoenv.2019.03.081"

"   Sokhn J, 2001, LETT APPL MICROBIOL, V33, P164, DOI 10.1046/j.1472-765x.2001.00972.x"
"   Soltani N, 2015, SCI TOTAL ENVIRON, V505, P712, DOI 10.1016/j.scitotenv.2014.09.097"
"   Song SS, 2008, ENVIRON POLLUT, V156, P1368, DOI 10.1016/j.envpol.2008.06.018"
"   Stajic JM, 2016, CHEMOSPHERE, V146, P68, DOI 10.1016/j.chemosphere.2015.12.006"
"   Staninska-Pieta J, 2020, MOLECULES, V25, DOI 10.3390/molecules25020319"
"   Subashchandrabose SR, 2019, SCI TOTAL ENVIRON, V659, P724, DOI 10.1016/j.scitotenv.2018.12.453"
"   Sun JS, 2012, MAR POLLUT BULL, V64, P13, DOI 10.1016/j.marpolbul.2011.10.034"
"   Sun X, 2020, ENVIRON SCI-PROC IMP, V22, P239, DOI [10.1039/c9em00529c, 10.1039/C9EM00529C]"
"   Sun YM, 2021, ENVIRON INT, V146, DOI 10.1016/j.envint.2020.106162"
"   Sutherland IW, 2001, MICROBIOL-UK, V147, P3, DOI 10.1099/00221287-147-1-3"
"   Tao KL, 2018, ECOTOX ENVIRON SAFE, V161, P237, DOI 10.1016/j.ecoenv.2018.05.091"
"   Tejada M, 2011, APPL SOIL ECOL, V50, P21, DOI 10.1016/j.apsoil.2011.08.002"
"   Tejada M, 2011, ECOTOX ENVIRON SAFE, V74, P668, DOI 10.1016/j.ecoenv.2010.10.018"
"   Teng JH, 2020, CHEMOSPHERE, V255, DOI [10.1016/j.chemosphere.2020.126953,
10.1016/j.chemosphere.2020.126053]"
"   Thavamani P, 2015, ENVIRON SCI POLLUT R, V22, P8957, DOI 10.1007/s11356-013-1850-3"
"   Thavamani P, 2012, BIODEGRADATION, V23, P823, DOI 10.1007/s10532-012-9572-7"
"   Thavamani P, 2012, J ENVIRON MANAGE, V99, P10, DOI 10.1016/j.jenvman.2011.12.030"
"   Tian Y, 2017, SCI TOTAL ENVIRON, V607, P944, DOI 10.1016/j.scitotenv.2017.07.094"
"   Usman ARA, 2013, J HAZARD MATER, V262, P1022, DOI 10.1016/j.jhazmat.2013.04.032"
"   Uzoigwe C, 2015, FRONT MICROBIOL, V6, DOI 10.3389/fmicb.2015.00245"
"   Vaidya S, 2017, 3 BIOTECH, V7, DOI 10.1007/s13205-017-0598-8"
"   Vasudevan D, 2013, ENVIRON SCI TECHNOL, V47, P14119, DOI 10.1021/es403389a"
"   Vijay D, 2008, PHYS CHEM CHEM PHYS, V10, P582, DOI 10.1039/b713703f"
"   Vijayan A, 2019, WATER AIR SOIL POLL, V230, DOI 10.1007/s11270-019-4201-2"
"   Vitali M, 2019, ENVIRON POLLUT, V254, DOI 10.1016/j.envpol.2019.113013"
"   Wang BY, 2020, ENVIRON SCI POLLUT R, V27, P18717, DOI 10.1007/s11356-020-08461-5"
"   Wang C, 2020, ENVIRON POLLUT, V266, DOI 10.1016/j.envpol.2020.115296"
"   Wang C, 2018, CHEMOSPHERE, V210, P247, DOI 10.1016/j.chemosphere.2018.06.177"
"   Wang CY, 2020, MAR POLLUT BULL, V150, DOI 10.1016/j.marpolbul.2019.110787"
"   Wang P, 2013, ENVIRON TOXICOL CHEM, V32, P673, DOI 10.1002/etc.2090"
"   Wang RY, 2020, REV CHEM ENG, V36, P947, DOI 10.1515/revce-2018-0076"
"   Wang WP, 2018, APPL ENVIRON MICROB, V84, DOI 10.1128/AEM.01261-18"
"   Wang YP, 2008, INT J BIOL MACROMOL, V43, P283, DOI 10.1016/j.ijbiomac.2008.06.011"
"   Wang Z, 2014, ECOTOX ENVIRON SAFE, V106, P1, DOI 10.1016/j.ecoenv.2014.04.019"
"   Wania F, 1998, ENVIRON POLLUT, V102, P3, DOI 10.1016/S0269-7491(98)00072-4"
"   Wawra A, 2018, SCI TOTAL ENVIRON, V628-629, P1287, DOI 10.1016/j.scitotenv.2018.02.156"
"   Wolf DC, 2018, ENVIRON POLLUT, V243, P1846, DOI 10.1016/j.envpol.2018.10.031"
"   Wu L, 2020, J OCEAN U CHINA, V19, P874, DOI 10.1007/s11802-020-4266-y"
"   Wu ML, 2020, SCI TOTAL ENVIRON, V713, DOI 10.1016/j.scitotenv.2019.136331"
"   Wu MH, 2016, APPL MICROBIOL BIOT, V100, P7249, DOI 10.1007/s00253-016-7551-3"
"   Wu SH, 2019, J HAZARD MATER, V364, P108, DOI 10.1016/j.jhazmat.2018.09.094"
"   Wu YY, 2020, ENVIRON POLLUT, V261, DOI 10.1016/j.envpol.2020.114093"
"   Xia L, 2020, CHEM PHYS LETT, V754, DOI 10.1016/j.cplett.2020.137689"
"   Xiao JJ, 2010, J HAZARD MATER, V174, P818, DOI 10.1016/j.jhazmat.2009.09.126"
"   Xie QT, 2020, ENVIRON POLLUT, V263, DOI 10.1016/j.envpol.2020.114102"
"   Yang MY, 2020, APPL SOIL ECOL, V150, DOI 10.1016/j.apsoil.2019.103472"
"   Yang Y, 2020, INT J ENVIRON SCI TE, V17, P2477, DOI 10.1007/s13762-020-02668-2"
"   Ye JS, 2013, CHEM ENG J, V219, P1, DOI 10.1016/j.cej.2012.12.093"
"   Yu KSH, 2005, MAR POLLUT BULL, V51, P1071, DOI 10.1016/j.marpolbul.2005.06.006"
"   Zegzouti Y, 2020, ENVIRON ENG RES, V25, P722, DOI 10.4491/eer.2019.317"
"   Zeneli A, 2019, J ENVIRON CHEM ENG, V7, DOI 10.1016/j.jece.2019.103054"
"   Zeng J, 2016, CHEMOSPHERE, V148, P1, DOI 10.1016/j.chemosphere.2016.01.019"
"   Zeng XY, 2018, J ANAL APPL PYROL, V132, P82, DOI 10.1016/j.jaap.2018.03.012"
"   Zhang JM, 2020, ECOTOX ENVIRON SAFE, V192, DOI 10.1016/j.ecoenv.2020.110269"
"   Zhang J, 2020, ARCH AGRON SOIL SCI, V66, P1089, DOI 10.1080/03650340.2019.1655732"
"   Zhang LB, 2012, COMP BIOCHEM PHYS C, V155, P517, DOI 10.1016/j.cbpc.2012.01.002"
"   Zhang SC, 2017, SCI REP-UK, V7, DOI 10.1038/s41598-017-12437-w"
"   Zhang SJ, 2017, SCI TOTAL ENVIRON, V601, P968, DOI 10.1016/j.scitotenv.2017.05.234"
"   Zhang WH, 2015, J SOIL SEDIMENT, V15, P1463, DOI 10.1007/s11368-015-1112-8"
"   Zhang WH, 2011, CHEMOSPHERE, V83, P302, DOI 10.1016/j.chemosphere.2010.12.056"
"   Zhang ZH, 2012, GEODERMA, V175, P1, DOI 10.1016/j.geoderma.2012.01.020"
"   Zhang ZH, 2011, J HAZARD MATER, V189, P119, DOI 10.1016/j.jhazmat.2011.02.007"
"   Zhao ZY, 2011, BIORESOURCE TECHNOL, V102, P3999, DOI 10.1016/j.biortech.2010.11.088"
"   Zhou L, 2020, SCI TOTAL ENVIRON, V719, DOI 10.1016/j.scitotenv.2020.137410"
"   Zhu DQ, 2004, J ENVIRON QUAL, V33, P1322, DOI 10.2134/jeq2004.1322"
"   Zloch M, 2016, CHEMOSPHERE, V156, P312, DOI 10.1016/j.chemosphere.2016.04.130"
NR 248
TC 175
Z9 200
U1 51
U2 752
PU ELSEVIER SCI LTD
PI London
"PA 125 London Wall, London, the United Kingdom"
SN 0269-7491
EI 1873-6424
J9 ENVIRON POLLUT
JI Environ. Pollut.
PD FEB 15
PY 2022
VL 295
AR 118686
DI 10.1016/j.envpol.2021.118686

PG 18
WC Environmental Sciences
WE Science Citation Index Expanded (SCI-EXPANDED)
SC Environmental Sciences & Ecology
GA ZP4KY
UT WOS:000766394300005
PM 34920044
DA 2026-04-06
ER

PT J
"AU Dong, YB"
"   Guo, J"
"   Lin, H"
"AF Dong, Yingbo"
"   Guo, Jing"
"   Lin, Hai"
TI Microbe-Plant Combined Remediation Technology for Heavy Metals in Soil:
   A Comprehensive Review
SO WATER AIR AND SOIL POLLUTION
LA English
DT Article
DE Combined microbial-plant remediation; Heavy metals pollution; Soil
   environment; Plant growth-promoting bacteria; Bioavailability
ID ARBUSCULAR MYCORRHIZAL FUNGI; GROWTH-PROMOTING BACTERIA;
   PHOSPHATE-SOLUBILIZING BACTERIA; SEPTATE ENDOPHYTE DSE; CONTAMINATED
   SOILS; TOLERANT PLANT; ARSENIC ACCUMULATION; RESISTANT BACTERIA;
   BENEFICIAL ROLE; PTERIS-VITTATA
"AB Contaminating the soil with heavy metals(HMs,This abbreviation is used in all subsequent content) is a serious problem.Combining the two can be a more effective solution to soil heavy metal remediation. In this paper, Detailed overview of the contaminated soil remediation in a cooperative manner by microorganisms and plants was presented.The first is the growth-promoting mechanism of microorganisms, which enhances restoration by altering the nitrogen, phosphorus, iron and phytohormones in the plant growth environment. Secondly, by reducing oxidative stress in plants and enhance heavy metals bioavailability, heavy metals can be enriched. In addition, plants can provide nutrients for microbes by releasing some organic matter, and at the same time, they can enrich HMs and provide a good environment for fungus. In general, plant growth-promoting bacteria, mycorrhizal fungi and plant growth-promoting rhizobacteria are usually combined with plants as tools for soil remediation. Finally, prominent cases of soil remediation in recent years were summarised. This review helps to understand how the most newer developments in the area of microbial-phytoremediation and will further strengthen the understanding of the mechanisms and Using microbial-plant cooperation to clean up contaminated soil,and it will make it easier to learn more about what are some of the more notable accomplishments and developments in the field in recent years, which are relevant for the expansion of this remediation methodology."
"C1 [Dong, Yingbo; Guo, Jing; Lin, Hai] Univ Sci & Technol, Sch Energy & Environm Engn, Beijing 100083, Peoples R China."
"   [Dong, Yingbo; Lin, Hai] Beijing Key Lab Resource Oriented Treatment Ind Po, Beijing 100083, Peoples R China."
C3 University of Science & Technology Beijing
"RP Lin, H (corresponding author), Univ Sci & Technol, Sch Energy & Environm Engn, Beijing 100083, Peoples R China.; Lin, H (corresponding author), Beijing Key Lab Resource Oriented Treatment Ind Po, Beijing 100083, Peoples R China."
EM linhai@ces.ustb.edu.cn
FU National Key R&D Program of China [2023YFC3706703]
FX This study was funded by the National Key R&D Program of China
   (2023YFC3706703).
"CR Abou-Shanab RA, 2003, NEW PHYTOL, V158, P219, DOI 10.1046/j.1469-8137.2003.00721.x"
"   Adeleke BS, 2021, RHIZOSPHERE-NETH, V20, DOI 10.1016/j.rhisph.2021.100433"
"   Agarwal P, 2020, CURR GENOMICS, V21, P334, DOI 10.2174/1389202921999200623133240"
"   Ahemad M, 2015, 3 BIOTECH, V5, P111, DOI 10.1007/s13205-014-0206-0"
"   Ahsan MT, 2018, ENVIRON SCI POLLUT R, V25, P7021, DOI 10.1007/s11356-017-0987-x"
"   Akafia MM, 2014, GEOCHIM COSMOCHIM AC, V141, P258, DOI 10.1016/j.gca.2014.06.024"
"   Ali H, 2019, HUM ECOL RISK ASSESS, V25, P1353, DOI 10.1080/10807039.2018.1469398"
"   Ali H, 2013, CHEMOSPHERE, V91, P869, DOI 10.1016/j.chemosphere.2013.01.075"
"   Alinejad Z, 2024, INT J PHYTOREMEDIAT, V26, P250, DOI 10.1080/15226514.2023.2236729"
"   Amari T, 2017, S AFR J BOT, V111, P99, DOI 10.1016/j.sajb.2017.03.011"
"   Ansari A, 2021, ENVIRON SCI POLLUT R, V28, P58640, DOI 10.1007/s11356-021-14821-6"
"   Babu AG, 2014, SCI TOTAL ENVIRON, V476, P561, DOI 10.1016/j.scitotenv.2013.12.119"
"   Babu AG, 2014, J ENVIRON MANAGE, V132, P129, DOI 10.1016/j.jenvman.2013.10.009"
"   Baldrian P, 2003, ENZYME MICROB TECH, V32, P78, DOI 10.1016/S0141-0229(02)00245-4"
"   Ban YH, 2012, PLOS ONE, V7, DOI 10.1371/journal.pone.0047968"
"   Bandurska KM, 2020, POL J ENVIRON STUD, V29, P3049, DOI 10.15244/pjoes/111322"
"   Belimov AA, 2005, SOIL BIOL BIOCHEM, V37, P241, DOI 10.1016/j.soilbio.2004.07.033"
"   Belimov AA, 2004, MICROBIOLOGY+, V73, P99, DOI 10.1023/B:MICI.0000016377.62060.d3"
"   Berthelot C, 2016, FEMS MICROBIOL ECOL, V92, DOI 10.1093/femsec/fiw144"
"   Bonaventure P, 2023, APPL SCI-BASEL, V13, DOI 10.3390/app13074228"
"   Braud A, 2009, CHEMOSPHERE, V74, P280, DOI 10.1016/j.chemosphere.2008.09.013"
"   Bulgarelli D, 2012, NATURE, V488, P91, DOI 10.1038/nature11336"
"   Bunge J, 2006, SCIENCE, V313, DOI 10.1126/science.1126593"
"   Cabello-Conejo MI, 2014, PLANT SOIL, V379, P35, DOI 10.1007/s11104-014-2043-7"
"   Chen LH, 2015, ECOTOX ENVIRON SAFE, V113, P460, DOI 10.1016/j.ecoenv.2014.12.033"
"   [?????? Chen Tao], 2021, [????????????, Pratacultural Science], V38, P1097"
"   Chi YW, 2022, CHEMOSPHERE, V305, DOI 10.1016/j.chemosphere.2022.135488"
"   Chiang PN, 2011, SOIL SCI, V176, P33, DOI 10.1097/SS.0b013e318202fdc9"
"   De Vrieze M, 2018, FRONT MICROBIOL, V9, DOI 10.3389/fmicb.2018.02573"

"   Ortega-Larrocea MD, 2010, ENVIRON POLLUT, V158, P1922, DOI 10.1016/j.envpol.2009.10.034"
"   Deng ZJ, 2012, WATER AIR SOIL POLL, V223, P5321, DOI 10.1007/s11270-012-1282-6"
"   Deram A, 2008, SOIL BIOL BIOCHEM, V40, P845, DOI 10.1016/j.soilbio.2007.09.023"
"   Duan CJ, 2022, FRONT MICROBIOL, V12, DOI 10.3389/fmicb.2021.781831"
"   Etesami H, 2020, ENVIRON EXP BOT, V178, DOI 10.1016/j.envexpbot.2020.104124"
"   Fu L, 2022, INT J PHYTOREMEDIAT, V24, P1107, DOI 10.1080/15226514.2021.2002809"
"   Fudala-Ksiazek S, 2016, WASTE MANAGE, V49, P516, DOI 10.1016/j.wasman.2016.01.022"
"   Gamalero E, 2009, CAN J MICROBIOL, V55, P501, DOI [10.1139/W09-010, 10.1139/w09-010]"
"   Gao MJ, 2022, WATER AIR SOIL POLL, V233, DOI 10.1007/s11270-022-05933-x"
"   Glick BR, 2007, EUR J PLANT PATHOL, V119, P329, DOI 10.1007/s10658-007-9162-4"
"   Glick BR, 2007, CRIT REV PLANT SCI, V26, P227, DOI 10.1080/07352680701572966"
"   Glick BR, 2012, SCIENTIFICA, V2012, DOI 10.6064/2012/963401"
"   Glick BR, 2010, BIOTECHNOL ADV, V28, P367, DOI 10.1016/j.biotechadv.2010.02.001"
"   Glick BR, 2003, BIOTECHNOL ADV, V21, P383, DOI 10.1016/S0734-9750(03)00055-7"
"   Gonzalez-Chavez C, 2002, NEW PHYTOL, V155, P163, DOI 10.1046/j.1469-8137.2002.00430.x"
"   Govarthanan M, 2016, 3 BIOTECH, V6, DOI 10.1007/s13205-016-0560-1"
"   Guarino C, 2014, ENVIRON SCI TECHNOL, V48, P11487, DOI 10.1021/es502070m"
"   Hao X, 2014, INT J PHYTOREMEDIAT, V16, P179, DOI 10.1080/15226514.2013.773273"
"   Harish S, 2008, APPL SOIL ECOL, V39, P187, DOI 10.1016/j.apsoil.2007.12.006"
"   Haroon U, 2021, GEOMICROBIOL J, V38, P768, DOI 10.1080/01490451.2021.1946624"
"   Hassan SE, 2013, NEW BIOTECHNOL, V30, P780, DOI 10.1016/j.nbt.2013.07.002"
"   He H.J., 2020, Journal of Northeast Agricultural Sciences, V45, P70"
"   Herrera-Quiterio A, 2020, REV ARGENT MICROBIOL, V52, P231, DOI 10.1016/j.ram.2019.08.003"
"   Hou LF, 2020, FRONT MICROBIOL, V10, DOI 10.3389/fmicb.2019.03061"
"   Ikram M, 2018, PLOS ONE, V13, DOI 10.1371/journal.pone.0208150"
"   Jain R, 2021, FRONT MICROBIOL, V12, DOI 10.3389/fmicb.2021.696667"
"   Jalili F, 2009, J PLANT PHYSIOL, V166, P667, DOI 10.1016/j.jplph.2008.08.004"
"   Jeong S, 2013, J HAZARD MATER, V263, P441, DOI 10.1016/j.jhazmat.2013.09.062"
"   [????????? Jin Yinjia], 2016, [??????????????????, Chinese Journal of Environmental Engineering], V10, P415"
"   Johnstone TC, 2015, DALTON T, V44, P6320, DOI 10.1039/c4dt03559c"
"   Jumpponen A, 1998, NEW PHYTOL, V140, P295, DOI 10.1046/j.1469-8137.1998.00265.x"
"   Kavroulakis N, 2007, J EXP BOT, V58, P3853, DOI 10.1093/jxb/erm230"
"   Kawahara H, 2001, CURR MICROBIOL, V43, P365, DOI 10.1007/s002840010317"
"   Nguyen KBT, 2023, ENVIRON SCI ENG, P199, DOI 10.1007/978-3-031-17808-5_13"
"   Kong ZY, 2017, ADV MICROB PHYSIOL, V71, P97, DOI 10.1016/bs.ampbs.2017.04.001"
"   Landwehr M, 2002, MYCORRHIZA, V12, P199, DOI 10.1007/s00572-002-0172-y"
"   Lebeau T, 2008, ENVIRON POLLUT, V153, P497, DOI 10.1016/j.envpol.2007.09.015"
"   Li W., 2023, Industrial Crops and Products, V203"
"   Li WC, 2010, PLANT SOIL, V326, P453, DOI 10.1007/s11104-009-0025-y"
"   Likar M, 2009, SCI TOTAL ENVIRON, V407, P6179, DOI 10.1016/j.scitotenv.2009.08.045"
"   Liu BH, 2020, SCI TOTAL ENVIRON, V719, DOI 10.1016/j.scitotenv.2020.137475"
"   Liu CJ, 2022, J HAZARD MATER, V434, DOI 10.1016/j.jhazmat.2022.128829"
"   Liu CJ, 2022, J HAZARD MATER, V431, DOI 10.1016/j.jhazmat.2022.128546"
"   [????????? Liu Jianu], 2016, [????????????????????, Journal of Safety and Environment], V16, P290"
"   Liu LW, 2018, SCI TOTAL ENVIRON, V633, P206, DOI 10.1016/j.scitotenv.2018.03.161"
"   Liu L, 2018, CHEMOSPHERE, V194, P495, DOI 10.1016/j.chemosphere.2017.12.025"
"   Liu SM, 2021, PLANTS-BASEL, V10, DOI 10.3390/plants10050912"
"   Liu ZB, 2018, INT J AGRIC BIOL, V20, P621, DOI [10.17957/ijab/15.0532, 10.17957/IJAB/15.0532]"
"   [?????? Lu Jun], 2023, [?????????????????????, Soil and Fertilizer Sciences in China], P231"
"   Luo Q, 2014, PLOS ONE, V9, DOI 10.1371/journal.pone.0115581"
"   Luo SL, 2011, CHEMOSPHERE, V85, P1130, DOI 10.1016/j.chemosphere.2011.07.053"
"   Luo SL, 2011, APPL MICROBIOL BIOT, V89, P1637, DOI 10.1007/s00253-010-2927-2"
"   Ma Y, 2011, BIOTECHNOL ADV, V29, P248, DOI 10.1016/j.biotechadv.2010.12.001"
"   Ma Y, 2016, FRONT PLANT SCI, V7, DOI 10.3389/fpls.2016.00918"
"   Ma Y, 2016, J ENVIRON MANAGE, V174, P14, DOI 10.1016/j.jenvman.2016.02.047"
"   Ma Y, 2015, J TOXICOL ENV HEAL A, V78, P931, DOI 10.1080/15287394.2015.1051205"
"   Ma Y, 2011, J HAZARD MATER, V195, P230, DOI 10.1016/j.jhazmat.2011.08.034"
"   Mallick I, 2018, SCI TOTAL ENVIRON, V610, P1239, DOI 10.1016/j.scitotenv.2017.07.234"
"   Mandyam K, 2005, STUD MYCOL, P173"
"   Tarekegn MM, 2020, COGENT FOOD AGR, V6, DOI 10.1080/23311932.2020.1783174"
"   Mejia LC, 2008, BIOL CONTROL, V46, P4, DOI 10.1016/j.biocontrol.2008.01.012"
"   Mesa V, 2017, APPL ENVIRON MICROB, V83, DOI [10.1128/AEM.03411-16, 10.1128/aem.03411-16]"
"   Moreno ML, 2012, J APPL MICROBIOL, V113, P550, DOI 10.1111/j.1365-2672.2012.05366.x"
"   Mosa KA, 2016, FRONT PLANT SCI, V7, DOI 10.3389/fpls.2016.00303"
"   Narendrula-Kotha R, 2017, PLOS ONE, V12, DOI 10.1371/journal.pone.0168497"
"   Nicoara A, 2014, ENVIRON SCI POLLUT R, V21, P6905, DOI 10.1007/s11356-013-2489-9"
"   Nie M., 2023, Environmental Pollution, V31, P29113, DOI [10.1007/S11356-024-32959-X, DOI 10.1007/S11356-024-32959-X]"
"   Nurzhanova AA, 2023, ENVIRON SCI POLLUT R, DOI 10.1007/s11356-023-29031-5"
"   Oh JJ, 2021, CHEMOSPHERE, V272, DOI 10.1016/j.chemosphere.2021.129884"
"   Orlowska E, 2012, ENVIRON POLLUT, V168, P121, DOI 10.1016/j.envpol.2012.04.026"
"   Ouatiki E, 2021, POL J ENVIRON STUD, V30, P5669, DOI 10.15244/pjoes/131979"
"   Padmavathiamma PK, 2007, WATER AIR SOIL POLL, V184, P105, DOI 10.1007/s11270-007-9401-5"
"   Parmar S, 2022, FRONT MICROBIOL, V12, DOI 10.3389/fmicb.2021.783475"
"   Peng H, 2021, CHEMOSPHERE, V283, DOI 10.1016/j.chemosphere.2021.131186"
"   Peng W., 2017, Guangdong Chemical Industry, V44, P127"
"   Penrose DM, 2001, CAN J MICROBIOL, V47, P368, DOI 10.1139/cjm-47-4-368"
"   Petriccione M, 2013, WATER AIR SOIL POLL, V224, DOI 10.1007/s11270-013-1645-7"
"   Phurailatpam L, 2022, FRONT SUSTAIN FOOD S, V5, DOI 10.3389/fsufs.2021.817932"
"   Pinto E, 2014, CRIT REV PLANT SCI, V33, P351, DOI 10.1080/07352689.2014.885729"
"   Prasad MNV, 2003, ELECTRON J BIOTECHN, V6, P285, DOI 10.2225/vol6-issue3-fulltext-6"
"   Priyadarshini E, 2021, CHEMOSPHERE, V274, DOI 10.1016/j.chemosphere.2021.129976"
"   Qadir M, 2023, ENVIRON SCI POLLUT R, V30, P9471, DOI 10.1007/s11356-022-22678-6"

"   Qin Y, 2016, BIOTECHNOL ADV, V34, P1245, DOI 10.1016/j.biotechadv.2016.08.005"
"   Qiu L, 2021, ENVIRON SCI POLLUT R, V28, P67472, DOI 10.1007/s11356-021-15326-y"
"   Quan Lingtong, 2023, Chemosphere, V334, P138857, DOI [10.1016/j.chemosphere.2023.138857, 10.1016/j.chemosphere.2023.138857]"
"   Radhakrishnan R, 2013, J MICROBIOL BIOTECHN, V23, P856, DOI 10.4014/jmb.1209.09045"
"   Rajkumar M, 2012, BIOTECHNOL ADV, V30, P1562, DOI 10.1016/j.biotechadv.2012.04.011"
"   Rajkumar M, 2009, CHEMOSPHERE, V77, P153, DOI 10.1016/j.chemosphere.2009.06.047"
"   Rangel TS, 2023, ENVIRON SCI POLLUT R, V30, P68271, DOI 10.1007/s11356-023-27126-7"
"   Rangel WM, 2017, INT J PHYTOREMEDIAT, V19, P142, DOI 10.1080/15226514.2016.1207600"
"   Shahid M, 2015, ANN MICROBIOL, V65, P1525, DOI 10.1007/s13213-014-0991-z"
"   Sharma B, 2021, BIORESOURCE TECHNOL, V320, DOI 10.1016/j.biortech.2020.124330"
"   Sharma P, 2021, BIORESOURCE TECHNOL, V339, DOI 10.1016/j.biortech.2021.125589"
"   Sharma P, 2021, BIORESOURCE TECHNOL, V328, DOI 10.1016/j.biortech.2021.124835"
"   Sharma P, 2021, BIORESOURCE TECHNOL, V320, DOI 10.1016/j.biortech.2020.124353"
"   Shi GY, 2022, ENVIRON TECHNOL INNO, V27, DOI 10.1016/j.eti.2022.102489"
"   Shin MN, 2012, J HAZARD MATER, V199, P314, DOI 10.1016/j.jhazmat.2011.11.010"
"   Silambarasan S, 2020, PLANT SOIL, V457, P241, DOI 10.1007/s11104-020-04734-7"
"   Soldi E, 2020, J FUNGI, V6, DOI 10.3390/jof6040254"
"   Song LP, 2023, SCI TOTAL ENVIRON, V873, DOI 10.1016/j.scitotenv.2023.162314"
"   Spaepen S, 2007, FEMS MICROBIOL REV, V31, P425, DOI 10.1111/j.1574-6976.2007.00072.x"
"   Sturz AV, 2000, CRIT REV PLANT SCI, V19, P1, DOI 10.1016/S0735-2689(01)80001-0"
"   Sun CY, 2021, ECOTOX ENVIRON SAFE, V228, DOI 10.1016/j.ecoenv.2021.113000"
"   Sun J., 2023, Int. J. Environ. Res. Public Health, V20"
"   Tang ST, 2023, ECOTOX ENVIRON SAFE, V254, DOI 10.1016/j.ecoenv.2023.114764"
"   Tiwari P, 2023, MICROBIOL RES, V266, DOI 10.1016/j.micres.2022.127241"
"   Tiwari S, 2018, FRONT PLANT SCI, V9, DOI 10.3389/fpls.2018.00452"
"   Tong Z., 2022, Chemical Engineering Design Communications, V48, P200"
"   Ullah A, 2015, ENVIRON SCI POLLUT R, V22, P2505, DOI 10.1007/s11356-014-3699-5"
"   Virk ZA, 2022, SUSTAINABILITY-BASEL, V14, DOI 10.3390/su14126975"
"   Wan Y., 2009, Application of endophytic bacterium isolated from Cd hyperaccumulator Solanum Nigrum L for phytoremediation enhancement"
"   Wan Y., 2013, Hunan University, V12, P136"
"   Wan Y, 2012, CHEMOSPHERE, V89, P743, DOI 10.1016/j.chemosphere.2012.07.005"
"   Wang JL, 2016, SCI REP-UK, V6, DOI 10.1038/srep22028"
"   Wang L, 2020, CHEMOSPHERE, V240, DOI 10.1016/j.chemosphere.2019.124891"
"   Wang L, 2018, J HAZARD MATER, V341, P1, DOI 10.1016/j.jhazmat.2017.07.036"
"   Wang LW, 2020, CRIT REV ENV SCI TEC, V50, P2724, DOI 10.1080/10643389.2019.1705724"
"   Wang Y, 2021, J ENVIRON CHEM ENG, V9, DOI 10.1016/j.jece.2021.106533"
"   Wani PA, 2008, ARCH ENVIRON CON TOX, V55, P33, DOI 10.1007/s00244-007-9097-y"
"   Wenxing Z., 2021, Journal of Agriculture, V11, P46"
"   Wu FY, 2009, CHEMOSPHERE, V76, P1258, DOI 10.1016/j.chemosphere.2009.05.020"
"   Xiao YH, 2023, CURR MICROBIOL, V80, DOI 10.1007/s00284-023-03453-3"
"   Xiong TT, 2016, J ENVIRON SCI, V46, P16, DOI 10.1016/j.jes.2015.08.029"
"   Xu C, 2015, ENVIRON SCI POLLUT R, V22, P5070, DOI 10.1007/s11356-014-3931-3"
"   Yang XQ, 2024, INT J PHYTOREMEDIAT, V26, P382, DOI 10.1080/15226514.2023.2243631"
"   Yang YR, 2022, ENVIRON SCI POLLUT R, V29, P32988, DOI 10.1007/s11356-022-18588-2"
"   Yasin NA, 2019, POL J ENVIRON STUD, V28, P425, DOI 10.15244/pjoes/76179"
"   Yeganeh E, 2023, GESUNDE PFLANZ, V75, P921, DOI 10.1007/s10343-022-00752-w"
"   Youzheng Z., 2023, Shandong Chemical Industry, V52, P243"
"   Yu J, 2023, J SOIL SEDIMENT, V23, P1745, DOI 10.1007/s11368-022-03415-5"
"   Yunzhu S., 2019, Science and Technology of Sichuan Agriculture, P68"
"   Zhan FD, 2016, B ENVIRON CONTAM TOX, V96, P235, DOI 10.1007/s00128-015-1676-4"
"   Zhang WH, 2012, CHEMOSPHERE, V87, P1171, DOI 10.1016/j.chemosphere.2012.02.036"
"   Zhang YJ, 2008, J MICROBIOL, V46, P624, DOI 10.1007/s12275-008-0163-6"
"   Zhang YK, 2023, J SOIL SEDIMENT, V23, P3472, DOI 10.1007/s11368-023-03559-y"
"   Zhaodong L., 2020, Inner Mongolia Environmental Sciences, V32, P105"
NR 165
TC 13
Z9 14
U1 31
U2 145
PU SPRINGER INT PUBL AG
PI CHAM
"PA GEWERBESTRASSE 11, CHAM, CH-6330, SWITZERLAND"
SN 0049-6979
EI 1573-2932
J9 WATER AIR SOIL POLL
JI Water Air Soil Pollut.
PD NOV
PY 2024
VL 235
IS 11
AR 711
DI 10.1007/s11270-024-07538-y
PG 22
WC Environmental Sciences; Meteorology & Atmospheric Sciences; Water
   Resources
WE Science Citation Index Expanded (SCI-EXPANDED)
SC Environmental Sciences & Ecology; Meteorology & Atmospheric Sciences;
   Water Resources
GA H8G9L
UT WOS:001325780700007
DA 2026-04-06
ER

PT J
"AU Wang, TJ"
"   Wang, XY"
"   Tian, W"
"   Yao, LG"
"   Li, YD"
"   Chen, ZJ"
"   Han, H"
"AF Wang, Tiejun"
"   Wang, Xiaoyu"
"   Tian, Wei"
"   Yao, Lunguang"
"   Li, Yadong"
"   Chen, Zhaojin"
"   Han, Hui"
TI Screening of Heavy Metal-Immobilizing Bacteria and Its Effect on
   Reducing Cd2+ and Pb2+ Concentrations in Water
   Spinach (Ipomoea aquatic Forsk.)
SO INTERNATIONAL JOURNAL OF ENVIRONMENTAL RESEARCH AND PUBLIC HEALTH
LA English
DT Article
DE heavy metal-immobilizing bacteria; Cd2+ and Pb2+; water spinach;
   immobilization; community composition
ID SUBSTRATE UTILIZATION PATTERN; GROWTH PROMOTING BACTERIA; MICROBIAL
   COMMUNITIES; MEDIATED ALLEVIATION; ENDOPHYTIC BACTERIA; CONTAMINATED
   SOILS; PLANT; ACCUMULATION; CADMIUM; BIOMASS
"AB Microbial immobilization is considered as a novel and environmentally friendly technology that uses microbes to reduce heavy metals accumulation in plants. To explore microbial resources which are useful in these applications, three water spinach rhizosphere soils polluted by different levels of heavy metals (heavy pollution (CQ), medium pollution (JZ), and relative clean (NF)) were collected. The community composition of heavy metal-immobilizing bacteria in rhizosphere soils and its effects on reducing the Cd2+ and Pb2+ concentrations in water spinach were evaluated. Four hundred strains were isolated from the CQ (belonging to 3 phyla and 14 genera), JZ (belonging to 4 phyla and 25 genera) and NF (belonged to 6 phyla and 34 genera) samples, respectively. In the CQ sample, 137 strains showed a strong ability to immobilize Cd2+ and Pb2+, giving Cd2+ and Pb2+ removal rates of greater than 80% in solution; Brevundimonas, Serratia, and Pseudoarthrobacter were the main genera. In total, 62 strains showed a strong ability to immobilize Cd2+ and Pb2+ in the JZ sample and Bacillus and Serratia were the main genera. A total of 22 strains showed a strong ability to immobilize Cd2+ and Pb2+ in the NF sample, and Bacillus was the main genus. Compared to the control, Enterobacter bugandensis CQ-7, Bacillus thuringensis CQ-33, and Klebsiella michiganensis CQ-169 significantly increased the dry weight (17.16-148%) of water spinach and reduced the contents of Cd2+ (59.78-72.41%) and Pb2+ (43.36-74.21%) in water spinach. Moreover, the soluble protein and Vc contents in the shoots of water spinach were also significantly increased (72.1-193%) in the presence of strains CQ-7, CQ-33 and CQ-169 compared to the control. In addition, the contents of Cd and Pb in the shoots of water spinach meet the standard for limit of Cd2+ and Pb2+ in vegetables in the presence of strains CQ-7, CQ-33 and CQ-169. Thus, the results provide strains as resources and a theoretical basis for the remediation of Cd- and Pb-contaminated farmlands for the safe production of vegetables."
"C1 [Wang, Tiejun; Wang, Xiaoyu; Yao, Lunguang; Chen, Zhaojin; Han, Hui] Nanyang Normal Univ, Coll Agr Engn, Collaborat Innovat Water Secur Water Source Reg M, Nanyang 473061, Peoples R China."
"   [Wang, Tiejun; Li, Yadong] Hubei Univ, Sch Life Sci, State Key Lab Biocatalysis & Enzyme Engn, Wuhan 43006, Peoples R China."
"   [Tian, Wei] Minist Ecol & Environm, Nanjing Inst Environm Sci, Nanjing 210042, Peoples R China."
C3 Nanyang Normal University; Hubei University; Nanjing Institute of
"   Environmental Sciences, Ministry of Ecology & Environment"
"RP Chen, ZJ; Han, H (corresponding author), Nanyang Normal Univ, Coll Agr Engn, Collaborat Innovat Water Secur Water Source Reg M, Nanyang 473061, Peoples R China."
EM liuyangkun@nynu.edu.cn; tcd530@nynu.edu.cn; tianwei@nies.org;
   lunguangyao@nynu.edu.cn; hbchen7112@hubu.edu.cn; zjchen@nynu.edu.cn;
   hanhui2018@nynu.edu.cn
"RI ; Wang, Tiejun/A-4671-2010; Han, Hui/GYJ-6725-2022; wang,"
   xiaoyu/HJP-6901-2023
"OI ?????????, Zhaojin Chen/0000-0003-1088-6821; Han, Hui/0000-0002-5218-0109; "
"FU National Natural Science Foundation of China [41907143, 31870917]; Key"
   Research Project of Colleges and Universities of Henan Province
   Education Department [20A180023]; Central Public-interest Scientific
   Institution Basal Research Fund [GYZX200202]
FX This work was supported by the National Natural Science Foundation of
"   China (41907143, 31870917), the Key Research Project of Colleges and"
"   Universities of Henan Province Education Department (20A180023), and the"
   Central Public-interest Scientific Institution Basal Research Fund
   (GYZX200202).
"CR Azarbad H, 2015, FEMS MICROBIOL ECOL, V91, DOI 10.1093/femsec/fiu003"
"   Banik A, 2018, SCI TOTAL ENVIRON, V630, P231, DOI 10.1016/j.scitotenv.2018.02.227"
"   Blakely JK, 2002, APPL SOIL ECOL, V21, P71, DOI 10.1016/S0929-1393(02)00023-9"
"   Burger J, 2008, SCI TOTAL ENVIRON, V389, P37, DOI 10.1016/j.scitotenv.2007.08.037"
"   Cai K, 2019, INT J ENV RES PUB HE, V16, DOI 10.3390/ijerph16142625"
"   Cankovic M, 2019, SYST APPL MICROBIOL, V42, DOI 10.1016/j.syapm.2019.126016"
"   Chen JL, 2013, AGR WATER MANAGE, V129, P152, DOI 10.1016/j.agwat.2013.07.011"
"   Chen L, 2016, J HAZARD MATER, V312, P123, DOI 10.1016/j.jhazmat.2016.03.042"
"   Chen Y, 2018, SCI TOTAL ENVIRON, V637, P1400, DOI 10.1016/j.scitotenv.2018.05.109"
"   Chonokhuu S, 2019, INT J ENV RES PUB HE, V16, DOI 10.3390/ijerph16142552"
"   Deng WW, 2020, J ENVIRON MANAGE, V257, DOI 10.1016/j.jenvman.2019.109980"
"   El-Meihy RM, 2019, ENVIRON EXP BOT, V162, P295, DOI 10.1016/j.envexpbot.2019.03.005"
"   Etesami H, 2018, ECOTOX ENVIRON SAFE, V147, P175, DOI 10.1016/j.ecoenv.2017.08.032"

"   FAO/WHO (Food and Agriculture Organization/World Health Organization), 2011, JOINT FAO WHO FOOD S"
"   Goswami D, 2015, J MICROBIOL METH, V110, P7, DOI 10.1016/j.mimet.2015.01.001"
"   Han H, 2020, ECOTOX ENVIRON SAFE, V195, DOI 10.1016/j.ecoenv.2020.110375"
"   Han H, 2018, ECOTOX ENVIRON SAFE, V161, P526, DOI 10.1016/j.ecoenv.2018.06.033"
"   Han H, 2018, J HAZARD MATER, V353, P280, DOI 10.1016/j.jhazmat.2018.04.024"
"   Hesse E, 2018, ECOL LETT, V21, P117, DOI 10.1111/ele.12878"
"   Idris R, 2004, APPL ENVIRON MICROB, V70, P2667, DOI 10.1128/AEM.70.5.2667-2677.2004"
"   Jalilvand N, 2020, J SOIL SCI PLANT NUT, V20, P206, DOI 10.1007/s42729-019-00121-z"
"   Kannan SK, 2006, ARCH MICROBIOL, V185, P202, DOI 10.1007/s00203-006-0088-6"
"   Ke XB, 2019, SYST APPL MICROBIOL, V42, P248, DOI 10.1016/j.syapm.2018.10.010"
"   Kim SW, 2017, J AGR FOOD CHEM, V65, P1239, DOI 10.1021/acs.jafc.6b04339"
"   Kjeldah J., 1883, Z. Anal. Chem, V22, P366, DOI [10.1007/BF01338151, DOI 10.1007/BF01338151]"
"   Le??n-Ca??edo JA, 2019, SCI TOTAL ENVIRON, V650, P2535, DOI 10.1016/j.scitotenv.2018.10.003"
"   Li Y, 2017, ECOTOX ENVIRON SAFE, V138, P56, DOI 10.1016/j.ecoenv.2016.12.024"
"   Li ZG, 2019, INT J ENV RES PUB HE, V16, DOI 10.3390/ijerph16193676"
"   Liao M, 2007, ECOTOX ENVIRON SAFE, V66, P217, DOI 10.1016/j.ecoenv.2005.12.013"
"   Masih L, 2002, FOOD RES INT, V35, P697, DOI 10.1016/S0963-9969(02)00065-0"
"   Nicholson FA, 2003, SCI TOTAL ENVIRON, V311, P205, DOI 10.1016/S0048-9697(03)00139-6"
"   Noisangiam R, 2011, SYST APPL MICROBIOL, V34, P166, DOI 10.1016/j.syapm.2011.01.002"
"   Pishchik VN, 2016, MICROBIOLOGY+, V85, P257, DOI 10.1134/S0026261716030097"
"   Rajkumar M, 2010, TRENDS BIOTECHNOL, V28, P142, DOI 10.1016/j.tibtech.2009.12.002"
"   Rajkumar M, 2009, CHEMOSPHERE, V77, P153, DOI 10.1016/j.chemosphere.2009.06.047"
"   Rehman MZU, 2018, ENVIRON POLLUT, V241, P557, DOI 10.1016/j.envpol.2018.06.005"
"   Saleem M, 2018, CHEMOSPHERE, V195, P606, DOI 10.1016/j.chemosphere.2017.12.117"
"   Singh N, 2016, ECOTOX ENVIRON SAFE, V125, P25, DOI 10.1016/j.ecoenv.2015.11.020"
"   SSICA (Soil Sci Ch. Acad.), 1980, DISSERTATION"
"   Teng ZD, 2019, J ENVIRON MANAGE, V231, P189, DOI 10.1016/j.jenvman.2018.10.012"
"   Torsvik V, 2002, CURR OPIN MICROBIOL, V5, P240, DOI 10.1016/S1369-5274(02)00324-7"
"   Udeigwe TK, 2011, ENVIRON INT, V37, P258, DOI 10.1016/j.envint.2010.08.008"
"   Wan YA, 2018, ENVIRON POLLUT, V240, P523, DOI 10.1016/j.envpol.2018.04.044"
"   Wang DF, 2017, J AGR FOOD CHEM, V65, P8999, DOI 10.1021/acs.jafc.7b02370"
"   Wang GY, 2018, J CLEAN PROD, V197, P479, DOI 10.1016/j.jclepro.2018.06.225"
"   WATANABE F. S., 1965, SOIL SCI SOC AMER PROC, V29, P677"
"   Wei Y, 2014, EUR J SOIL BIOL, V62, P15, DOI 10.1016/j.ejsobi.2014.02.011"
"   Wheaton G, 2015, MINERALS-BASEL, V5, P397, DOI 10.3390/min5030397"
"   Wood JL, 2016, SCI REP-UK, V6, DOI 10.1038/srep36067"
"   Xu QR, 2018, J AGR FOOD CHEM, V66, P5229, DOI 10.1021/acs.jafc.8b00598"
"   Yang UJ, 2012, FOOD CHEM TOXICOL, V50, P3862, DOI 10.1016/j.fct.2012.07.020"
"   Yao HY, 2003, GEODERMA, V115, P139, DOI 10.1016/S0016-7061(03)00083-1"
"   [????????? YUAN Jinhua], 2011, [????€?????????????, Ecology and Environmental Sciences], V20, P779"
"   Zhang C, 2016, SCI TOTAL ENVIRON, V557, P785, DOI 10.1016/j.scitotenv.2016.01.170"
"   Zhao XQ, 2019, INT J ENV RES PUB HE, V16, DOI 10.3390/ijerph16020268"
"   Zhao Yong Zhao Yong, 2006, Transactions of the Chinese Society of Agricultural Engineering, V22, P149"
"   Zornoza P, 2010, J ENVIRON SCI-CHINA, V22, P421, DOI 10.1016/S1001-0742(09)60124-8"
NR 57
TC 12
Z9 12
U1 1
U2 54
PU MDPI
PI BASEL
"PA MDPI AG, Grosspeteranlage 5, CH-4052 BASEL, SWITZERLAND"
EI 1660-4601
J9 INT J ENV RES PUB HE
JI Int. J. Environ. Res. Public Health
PD MAY
PY 2020
VL 17
IS 9
AR 3122
DI 10.3390/ijerph17093122
PG 16
"WC Environmental Sciences; Public, Environmental & Occupational Health"
WE Science Citation Index Expanded (SCI-EXPANDED); Social Science Citation Index (SSCI)
"SC Environmental Sciences & Ecology; Public, Environmental & Occupational"
   Health
GA LR5OY
UT WOS:000535745400146
PM 32365834
"OA Green Submitted, gold"
DA 2026-04-06
ER

PT J
"AU Sharma, A"
"   Maurya, N"
"   Singh, SK"
"   Sundaram, S"
"AF Sharma, Abhijeet"
"   Maurya, Neetu"
"   Singh, Shailendra Kumar"
"   Sundaram, Shanthy"
TI Investigation on synergetic strategy for the rejuvenation of Cr (VI)
   contaminated soil using biochar-immobilized bacteria and cyanobacteria

consortia
SO JOURNAL OF ENVIRONMENTAL CHEMICAL ENGINEERING
LA English
DT Article
DE Biochar; Soil remediation; Multi-heavy metal tolerant; Adsorption;
Bacillus spp
ID HEXAVALENT CHROMIUM; BACILLUS-SUBTILIS; ACTIVATED CARBON; REMEDIATION;
REMOVAL; BIOREMEDIATION; CR(VI)
"AB The study highlights the potential of Bacillus spp. SSAU-2 in rejuvenating soil fertility and emphasizes the importance of microbe adsorption on activated biochar. The primary objective is to restructure the quality of Cr (VI)-contaminated soil through the strategic use of bacteria and cyanobacteria immobilized on biochar derived from rice straw. The synthesized activated charcoal is highly porous, and immobilization significantly boosts the bacteria's ability to remove Cr (VI) from the soil. This ability increased from a threshold value of 60 ppm Cr (VI) to 500 ppm Cr (VI), indicating its efficacy. The microbe demonstrated multi-heavy metal tolerance, surviving even in 1000 ppm heavy metal concentrations. Moreover, the study revealed increased phosphate solubilization (49%), conductivity (175%), and salinity (53%), demonstrating the microbe's efficiency in solubilizing metal salts in the soil. The study also focuses on the community behavior of the bacterium in the soil in which it was found that the SSAU-2 mingles quite efficiently with the soil. Scanning Electron Microscope images confirmed the effective colonization of microbes within the pores of activated charcoal. Thus study presents a novel approach for the effective remediation of Cr (VI)-contaminated soil through the application of Bacillus sp. SSAU-2 immobilized on biochar. The research explored the dual benefits of heavy metal removal and repurposing of agricultural waste through open burning."
"C1 [Sharma, Abhijeet; Maurya, Neetu; Sundaram, Shanthy] Univ Allahabad, Ctr Biotechnol, Prayagraj 211002, Uttar Pradesh, India."
" [Singh, Shailendra Kumar] SS Khanna Girls Degree Coll, Dept Biochem, Prayagraj 211002, Uttar Pradesh, India."
C3 University of Allahabad
"RP Sundaram, S (corresponding author), Univ Allahabad, Ctr Biotechnol, Prayagraj 211002, Uttar Pradesh, India."
EM shanthy.cbt@gmail.com
"RI ; Sharma, Abhijeet/PHD-9671-2026; SINGH, SHAILENDRA/T-1882-2017"
"OI SHARMA, ABHIJEET/0009-0001-2137-6563; "
"FU Department of Biotechnology, New Delhi [BT/PR31340/BCE/8/1505/2019]"
FX Authors are thankful to National Centre of Experimental Petrology and
" Mineralogy, University of Allahabad, Prayagraj, India for providing"
necessary laboratory facilities. Mr. Abhijeet Sharma is grateful to the
" Department of Biotechnology, New Delhi for providing him with the Junior"
Research Fellowship for his work under the sanction order
BT/PR31340/BCE/8/1505/2019.
"CR Al-Badri BAS, 2020, ALL LIFE, V13, P92, DOI 10.1080/26895293.2020.1728393"
" Ali B, 2009, WORLD J MICROB BIOT, V25, P519, DOI 10.1007/s11274-008-9918-9"
" Alori ET, 2017, FRONT MICROBIOL, V8, DOI 10.3389/fmicb.2017.00971"
" An Q, 2022, SCI TOTAL ENVIRON, V814, DOI 10.1016/j.scitotenv.2021.152635"
" Arora PK, 2020, FRONT BIOENG BIOTECH, V8, DOI 10.3389/fbioe.2020.570307"
" Aziz S, 2023, ENVIRON RES, V238, DOI 10.1016/j.envres.2023.116909"
" Basu S., 2021, Phytomicrobiome for Sustainable Agriculture, P149, DOI DOI 10.1016/B978-0-444-64325-4.00013-4"
" Bhagat N, 2021, J MICROBIOL BIOTECHN, V31, P1045, DOI 10.4014/jmb.2105.05009"
" Bibi S, 2018, CHEMOSPHERE, V211, P653, DOI 10.1016/j.chemosphere.2018.07.197"
" Cai XC, 2019, ECOTOX ENVIRON SAFE, V181, P472, DOI 10.1016/j.ecoenv.2019.06.036"
" Chatterjee R, 2020, FRONT ENERGY RES, V8, DOI 10.3389/fenrg.2020.00085"
" Chen MJ, 2021, J HAZARD MATER, V411, DOI 10.1016/j.jhazmat.2021.125103"
" Chen Q, 2019, FRONT MICROBIOL, V10, DOI 10.3389/fmicb.2019.02171"
" Chen YY, 2021, FRONT MICROBIOL, V12, DOI 10.3389/fmicb.2021.641913"
" Chopra S, 2020, BIORESOUR BIOPROCESS, V7, DOI 10.1186/s40643-020-0297-x"
" de Bittencourt MA, 2020, J CLEAN PROD, V268, DOI 10.1016/j.jclepro.2020.122164"
" Diaconu M, 2020, NEW BIOTECHNOL, V56, P130, DOI 10.1016/j.nbt.2020.01.003"
" Gangola S, 2023, ENVIRON RES, V232, DOI 10.1016/j.envres.2023.116332"
" Goldstein AH., 2000, Proceedings of 4th International Association for Technical Conference, P220"
" Gorovtsov AV, 2020, ENVIRON GEOCHEM HLTH, V42, P2495, DOI 10.1007/s10653-019-00412-5"
" Gu P, 2020, ECOTOX ENVIRON SAFE, V189, DOI 10.1016/j.ecoenv.2019.109976"
" Guo H., 2023, Process Saf. Environ. Prot."
" Hartmann M, 2023, NAT REV EARTH ENV, V4, P4, DOI 10.1038/s43017-022-00366-w"
" Kavindi GAG, 2022, BIORESOUR TECH REP, V18, DOI [10.1016/j.biteb.2022.101052, 10.1016/j.biteb.2022.101052]"
" Lafi H, 2023, ENVIRON SCI-WAT RES, V9, P2021, DOI [10.1039/d3ew00117b, 10.1039/D3EW00117B]"
" Liu YP, 2016, MOL PLANT MICROBE IN, V29, P324, DOI 10.1094/MPMI-10-15-0239-R"
" Lu HL, 2022, ECOTOX ENVIRON SAFE, V234, DOI 10.1016/j.ecoenv.2022.113409"
" Minari GD, 2020, ECOTOX ENVIRON SAFE, V204, DOI 10.1016/j.ecoenv.2020.111038"
" Oves M, 2013, EUR J SOIL BIOL, V56, P72, DOI 10.1016/j.ejsobi.2013.02.002"
" Pattnaik S, 2020, CHEMOSPHERE, V240, DOI 10.1016/j.chemosphere.2019.124895"
" Peiris C, 2019, RSC ADV, V9, P17612, DOI 10.1039/c9ra02729g"
" Pradhan D, 2019, J CLEAN PROD, V209, P617, DOI 10.1016/j.jclepro.2018.10.288"
" Rafique MI, 2021, CHEMOSPHERE, V266, DOI 10.1016/j.chemosphere.2020.129198"
" Rajesh Y, 2023, Chemosphere, V341"
" Cruz-M??ndez AS, 2021, APPL SCI-BASEL, V11, DOI 10.3390/app11146502"
" Sato JH, 2014, SCI AGR, V71, P302, DOI 10.1590/0103-9016-2013-0306"
" Si YY, 2022, ADV COMPOS HYBRID MA, V5, P1180, DOI 10.1007/s42114-022-00446-x"
" Singh B. R., 2020, SOIL PROCESSES WATER, P233"
" Sireesha S, 2022, BIOMASS CONVERS BIOR, DOI 10.1007/s13399-021-02186-2"
" Su HJ, 2016, J HAZARD MATER, V318, P533, DOI 10.1016/j.jhazmat.2016.07.039"
" Sukumar C, 2017, CLEAN TECHNOL ENVIR, V19, P251, DOI 10.1007/s10098-016-1203-2"
" Sukumar C, 2014, ENVIRON EARTH SCI, V72, P839, DOI 10.1007/s12665-013-3007-6"

"   Tahir HAS, 2017, FRONT MICROBIOL, V8, DOI 10.3389/fmicb.2017.00171"
"   Tirry N., 2018, Journal of Genetic Engineering and Biotechnology, V16, P613, DOI [10.1016/j.jgeb.2018.06.004, 10.1016/j.jgeb.2018.06.004]"
"   Tu C, 2020, ENVIRON INT, V137, DOI 10.1016/j.envint.2020.105576"
"   Wang BH, 2020, ENVIRON POLLUT, V267, DOI [10.1016/j.envpol.2020.115411, 10.1016/J.envpol.2020.115411]"
"   Wang DF, 2017, ACS SUSTAIN CHEM ENG, V5, P2246, DOI 10.1021/acssuschemeng.6b02569"
"   Wang FY, 2020, TOXICS, V8, DOI 10.3390/toxics8040102"
"   Wang SW, 2021, PLANT SOIL, V466, P491, DOI 10.1007/s11104-021-05055-z"
"   Wang YC, 2021, ENVIRON POLLUT, V283, DOI 10.1016/j.envpol.2021.117111"
"   Wong JWC, 2021, ENVIRON SCI POLLUT R, V28, P46894, DOI 10.1007/s11356-021-14803-8"
"   Xia SP, 2020, ENVIRON GEOCHEM HLTH, V42, P1543, DOI 10.1007/s10653-019-00445-w"
"   Xia SP, 2019, CRIT REV ENV SCI TEC, V49, P1027, DOI 10.1080/10643389.2018.1564526"
"   Xia X, 2021, J HAZARD MATER, V401, DOI [10.1016/j.jhazmat.2020.143685, 10.1016/j.jhazmat.2020.123685]"
"   Yang XR, 2021, SCI TOTAL ENVIRON, V780, DOI 10.1016/j.scitotenv.2021.146580"
"   Yihunu EW, 2019, SN APPL SCI, V1, DOI 10.1007/s42452-019-0936-z"
"   Yong JJJY, 2021, BIOTECHNOL ADV, V47, DOI 10.1016/j.biotechadv.2020.107684"
"   Yusuf I, 2016, J ENVIRON MANAGE, V183, P182, DOI 10.1016/j.jenvman.2016.08.059"
"   Zhang BF, 2019, RSC ADV, V9, P35304, DOI 10.1039/c9ra06726d"
"   Zhang Y, 2023, BIOCHAR, V5, DOI 10.1007/s42773-023-00241-x"
"   Zheng ZJ, 2021, BIORESOURCE TECHNOL, V341, DOI 10.1016/j.biortech.2021.125803"
"   Zhitkovich A, 2011, CHEM RES TOXICOL, V24, P1617, DOI 10.1021/tx200251t"
"   Zhou X, 2021, J HAZARD MATER, V411, DOI 10.1016/j.jhazmat.2021.125087"
"   Zhu XL, 2022, ECOTOX ENVIRON SAFE, V237, DOI 10.1016/j.ecoenv.2022.113524"
"   Zhu XM, 2017, ENVIRON POLLUT, V227, P98, DOI [10.1016/j.envpol.2017.04.032, 10.1016/j.envPol.2017.04.032]"
NR 65
TC 19
Z9 25
U1 9
U2 68
PU ELSEVIER SCI LTD
PI London
"PA 125 London Wall, London, the United Kingdom"
SN 2213-2929
EI 2213-3437
J9 J ENVIRON CHEM ENG
JI J. Environ. Chem. Eng.
PD APR
PY 2024
VL 12
IS 2
AR 112034
DI 10.1016/j.jece.2024.112034
EA FEB 2024
PG 16
"WC Engineering, Environmental; Engineering, Chemical"
WE Science Citation Index Expanded (SCI-EXPANDED)
SC Engineering
GA KV0E0
UT WOS:001182612700001
DA 2026-04-06
ER

PT J
"AU Qin, HC"
"   Wang, ZX"
"   Sha, WY"
"   Song, SH"
"   Qin, FJ"
"   Zhang, WC"
"AF Qin, Haichen"
"   Wang, Zixiao"
"   Sha, Wenya"
"   Song, Shuhong"
"   Qin, Fenju"
"   Zhang, Wenchao"
TI Role of Plant-Growth-Promoting Rhizobacteria in Plant Machinery for Soil Heavy Metal Detoxification
SO MICROORGANISMS
LA English
DT Review
DE heavy metal; plant-growth-promoting rhizobacteria (PGPR); soil contamination; bioremediation; heavy metal detoxification
ID POLYMERIC SUBSTANCES EPS; PHOSPHATE-SOLUBILIZING BACTERIA; WEIGHT ORGANIC-ACIDS; PSEUDOMONAS-AERUGINOSA; ACC DEAMINASE; BACILLUS SPP.; ANTIOXIDANT DEFENSE; NITROGEN-FIXATION; VEGETATIVE GROWTH; OXIDATIVE STRESS
"AB Heavy metals migrate easily and are difficult to degrade in the soil environment, which causes serious harm to the ecological environment and human health. Thus, soil heavy metal pollution has become one of the main environmental issues of global concern. Plant-growth-promoting rhizobacteria (PGPR) is a kind of microorganism that grows around the rhizosphere and can promote plant growth and increase crop yield. PGPR can change the bioavailability of heavy metals in the rhizosphere microenvironment, increase heavy metal uptake by phytoremediation plants, and enhance the phytoremediation efficiency of heavy-metal-contaminated soils. In recent years, the number of studies on the phytoremediation efficiency of heavy-metal-contaminated

soil enhanced by PGPR has increased rapidly. This paper systematically reviews the mechanisms of PGPR that promote plant growth (including nitrogen fixation, phosphorus solubilization, potassium solubilization, iron solubilization, and plant hormone secretion) and the mechanisms of PGPR that enhance plant-heavy metal interactions (including chelation, the induction of systemic resistance, and the improvement of bioavailability). Future research on PGPR should address the challenges in heavy metal removal by PGPR-assisted phytoremediation."
"C1 [Qin, Haichen; Wang, Zixiao; Sha, Wenya; Song, Shuhong; Qin, Fenju; Zhang, Wenchao] Suzhou Univ Sci & Technol, Sch Chem & Life Sci, Suzhou 215009, Peoples R China."
C3 Suzhou University of Science & Technology
"RP Qin, FJ; Zhang, WC (corresponding author), Suzhou Univ Sci & Technol, Sch Chem & Life Sci, Suzhou 215009, Peoples R China."
EM qinfenju@usts.edu.cn; wenchaozhang@usts.edu.cn
"OI zhang, wenchao/0000-0002-7372-6027"
FU National Natural Science Foundation of China
FX No Statement Available
"CR Abbaszadeh-Dahaji P, 2021, CURR MICROBIOL, V78, P1335, DOI 10.1007/s00284-021-02408-w"
"   Abdelkrim S, 2018, J BASIC MICROB, V58, P579, DOI 10.1002/jobm.201700626"
"   Ahemad M, 2019, ARAB J CHEM, V12, P1365, DOI 10.1016/j.arabjc.2014.11.020"
"   Al Mahmud J, 2018, ECOTOX ENVIRON SAFE, V147, P990, DOI 10.1016/j.ecoenv.2017.09.045"
"   Al-Hakimi AMA, 2007, PLANT SOIL ENVIRON, V53, P129, DOI 10.17221/2228-PSE"
"   Ali S, 2017, Functional importance of the plant microbiome, P89, DOI 10.1007/978-3-319-65897-1_6"
"   Amara U, 2015, SUSTAIN DEV BIODIVER, V12, P87, DOI 10.1007/978-3-319-24654-3_5"
"   Annapurna K., 2013, Bacteria in Agrobiology: Disease Management, DOI [10.1007/978-3-642-33639-3_15, DOI 10.1007/978-3-642-33639-315, DOI 10.1007/978-3-642-33639-3_15]"
"   [Anonymous], 2012, Journal of Cell and Molecular Research"
"   Asadulla, 2021, PAK J BOT, V53, P31, DOI 10.30848/PJB2021-1(39)"
"   Ashrafuzzaman M, 2009, AFR J BIOTECHNOL, V8, P1247"
"   Baldani JI, 2005, AN ACAD BRAS CIENC, V77, P549, DOI 10.1590/S0001-37652005000300014"
"   Bao T, 2011, AFR J BIOTECHNOL, V10, P17180, DOI 10.5897/AJB11.1617"
"   Barriuso J., 2008, PLANTBACTERIA INTERA, P1, DOI 10.1002/9783527621989.ch1"
"   Basti??n F, 1998, PLANT GROWTH REGUL, V24, P7, DOI 10.1023/A:1005964031159"
"   BATAINEH ZM, 1986, PROSTATE, V9, P397, DOI 10.1002/pros.2990090409"
"   Ben Massoud M, 2018, J PLANT PHYSIOL, V221, P51, DOI 10.1016/j.jplph.2017.11.014"
"   Biswas JK, 2017, ENVIRON GEOCHEM HLTH, V39, P1583, DOI 10.1007/s10653-017-9950-5"
"   Cai LM, 2012, ECOTOX ENVIRON SAFE, V78, P2, DOI 10.1016/j.ecoenv.2011.07.004"
"   ??akmak??i R, 2007, J PLANT NUTR SOIL SC, V170, P288, DOI 10.1002/jpln.200625105"
"   Camilios-Neto D, 2014, BMC GENOMICS, V15, DOI 10.1186/1471-2164-15-378"
"   Capdevila M, 2011, J BIOL INORG CHEM, V16, P977, DOI 10.1007/s00775-011-0798-3"
"   Chai MinWei Chai MinWei, 2012, African Journal of Biotechnology, V11, P6091"
"   Chen B, 2017, FRONT MICROBIOL, V8, DOI 10.3389/fmicb.2017.02538"
"   Chen HC, 2020, ECOTOX ENVIRON SAFE, V187, DOI 10.1016/j.ecoenv.2019.109790"
"   Chen J, 2018, ECOTOX ENVIRON SAFE, V162, P312, DOI 10.1016/j.ecoenv.2018.07.001"
"   Comte S, 2006, PROCESS BIOCHEM, V41, P815, DOI 10.1016/j.procbio.2005.10.014"
"   Danish S, 2019, ECOTOX ENVIRON SAFE, V185, DOI 10.1016/j.ecoenv.2019.109706"
"   Dary M, 2010, J HAZARD MATER, V177, P323, DOI 10.1016/j.jhazmat.2009.12.035"
"   de Souza LA, 2012, ACTA PHYSIOL PLANT, V34, P523, DOI 10.1007/s11738-011-0849-y"
"   De Zutter N, 2021, MICROB BIOTECHNOL, V14, P1594, DOI 10.1111/1751-7915.13824"
"   Dhiman VK, 2022, PLANT STRESS, V4, DOI 10.1016/j.stress.2022.100080"
"   Di Francesco A, 2021, BIOL CONTROL, V152, DOI 10.1016/j.biocontrol.2020.104456"
"   Dixit R, 2015, SUSTAINABILITY-BASEL, V7, P2189, DOI 10.3390/su7022189"
"   Dobrowolski R, 2017, BIORESOURCE TECHNOL, V225, P113, DOI 10.1016/j.biortech.2016.11.040"
"   Dong B, 2013, BIORESOURCE TECHNOL, V131, P152, DOI 10.1016/j.biortech.2012.12.112"
"   Du J, 2012, PLANT OMICS, V5, P46"
"   Du MM, 2020, ANNU REV PLANT BIOL, V71, P379, DOI 10.1146/annurev-arplant-073019-025907"
"   Farid M, 2019, INT J PHYTOREMEDIAT, V21, P760, DOI 10.1080/15226514.2018.1556595"
"   Gavrilescu M, 2022, CURR OPIN BIOTECH, V74, DOI 10.1016/j.copbio.2021.10.024"
"   Geries LSM, 2021, ARCH MICROBIOL, V203, P169, DOI 10.1007/s00203-020-01991-z"
"   Glick BR, 2007, EUR J PLANT PATHOL, V119, P329, DOI 10.1007/s10658-007-9162-4"
"   Glick BR, 1998, J THEOR BIOL, V190, P63, DOI 10.1006/jtbi.1997.0532"
"   Gomes GLB, 2021, PLANT BIOLOGY, V23, P894, DOI 10.1111/plb.13303"
"   Gopalakrishnan S, 2015, 3 BIOTECH, V5, P355, DOI 10.1007/s13205-014-0241-x"
"   Goswami Dweipayan, 2016, Cogent Food & Agriculture, V2, DOI 10.1080/23311932.2015.1127500"
"   Guarino F, 2020, CHEMOSPHERE, V251, DOI 10.1016/j.chemosphere.2020.126310"
"   Guo JK, 2014, PLANT SOIL, V375, P205, DOI 10.1007/s11104-013-1952-1"
"   Gupta P, 2018, SCI REP-UK, V8, DOI 10.1038/s41598-018-23322-5"
"   Gupta P, 2018, CHEMOSPHERE, V192, P318, DOI 10.1016/j.chemosphere.2017.10.164"
"   Gupta R, 2024, APPL BIOCHEM BIOTECH, V196, P2928, DOI 10.1007/s12010-023-04545-3"
"   Gururani MA, 2013, J PLANT GROWTH REGUL, V32, P245, DOI 10.1007/s00344-012-9292-6"
"   HALLIWELL B, 1992, FEBS LETT, V307, P108, DOI 10.1016/0014-5793(92)80911-Y"
"   Hamidpour M, 2020, ENVIRON GEOCHEM HLTH, V42, P2535, DOI 10.1007/s10653-019-00422-3"
"   Hansda A, 2017, 3 BIOTECH, V7, DOI 10.1007/s13205-017-0757-y"
"   Hao X, 2014, INT J PHYTOREMEDIAT, V16, P179, DOI 10.1080/15226514.2013.773273"
"   Hartmann A, 2008, PLANT SOIL, V312, P7, DOI 10.1007/s11104-007-9514-z"
"   Hassan W, 2016, ENVIRON EARTH SCI, V75, DOI 10.1007/s12665-015-4902-9"
"   Hassen A, 1998, BIORESOURCE TECHNOL, V65, P73, DOI 10.1016/S0960-8524(98)00011-X"
"   Hayat R, 2010, ANN MICROBIOL, V60, P579, DOI 10.1007/s13213-010-0117-1"
"   He CQ, 2010, APPL SOIL ECOL, V44, P1, DOI 10.1016/j.apsoil.2009.07.003"
"   Hodge A, 2000, TRENDS PLANT SCI, V5, P304, DOI 10.1016/S1360-1385(00)01656-3"
"   Hou DY, 2021, ENVIRON SCI TECHNOL, V55, P15572, DOI 10.1021/acs.est.1c06044"
"   Huo Y, 2021, J GINSENG RES, V45, P218"
"   Hussain I, 2023, J PLANT GROWTH REGUL, V42, P2258, DOI 10.1007/s00344-022-10700-7"
"   Islam F, 2014, ECOTOX ENVIRON SAFE, V110, P143, DOI 10.1016/j.ecoenv.2014.08.020"
"   Islam F, 2014, ECOTOX ENVIRON SAFE, V104, P285, DOI 10.1016/j.ecoenv.2014.03.008"
"   Israr D, 2016, PLANT PHYSIOL BIOCH, V108, P304, DOI 10.1016/j.plaphy.2016.07.023"

"   Jeong S, 2012, CHEMOSPHERE, V88, P204, DOI 10.1016/j.chemosphere.2012.03.013"
"   Jha C.K., 2015, E3 J AGR RES DEV, V5, P108, DOI [DOI 10.13140/RG.2.1.5171.2164, 10.1080/01904167.2014.920384, DOI 10.1080/01904167.2014.920384]"
"   Jian LR, 2019, PEERJ, V7, DOI 10.7717/peerj.6875"
"   Jin ZY, 2018, NAT COMMUN, V9, DOI 10.1038/s41467-018-03791-y"
"   Jing Yan-De, 2007, Journal of Zhejiang University-Science B, V8, P192, DOI 10.1631/jzus.2007.B0192"
"   Joo GJ, 2009, J MICROBIOL, V47, P167, DOI 10.1007/s12275-008-0273-1"
"   Jorquera MA, 2016, MICROB ECOL, V72, P633, DOI 10.1007/s00248-016-0813-x"
"   Carlos MHJ, 2016, MICROBIOL RES, V188, P53, DOI 10.1016/j.micres.2016.05.001"
"   Ju WL, 2020, CHEMOSPHERE, V254, DOI 10.1016/j.chemosphere.2020.126724"
"   Juwarkar AA, 2007, CHEMOSPHERE, V68, P1996, DOI 10.1016/j.chemosphere.2007.02.027"
"   Kamal N, 2021, MICROBIOL RES, V242, DOI 10.1016/j.micres.2020.126594"
"   Kaushal M, 2016, ANN MICROBIOL, V66, P35, DOI 10.1007/s13213-015-1112-3"
"   Keesstra S, 2018, LAND-BASEL, V7, DOI 10.3390/land7040133"
"   Khan WU, 2017, INT J PHYTOREMEDIAT, V19, P470, DOI 10.1080/15226514.2016.1244167"
"   Khanna K, 2023, J PLANT GROWTH REGUL, V42, P6474, DOI 10.1007/s00344-022-10879-9"
"   Khanna K, 2019, CHEMOSPHERE, V230, P628, DOI 10.1016/j.chemosphere.2019.05.072"
"   Khanna K, 2019, CHEMOSPHERE, V217, P463, DOI 10.1016/j.chemosphere.2018.11.005"
"   Kieber JJ, 2018, DEVELOPMENT, V145, DOI 10.1242/dev.149344"
"   Kloepper JW., 1978, P 4 INT C PLANT PATH"
"   Krishnamurti GSR, 1997, J ENVIRON QUAL, V26, P271, DOI 10.2134/jeq1997.00472425002600010038x"
"   Kudoyarova G, 2019, FRONT PLANT SCI, V10, DOI 10.3389/fpls.2019.01368"
"   Kushwaha S, 2012, BIORESOURCE TECHNOL, V116, P15, DOI 10.1016/j.biortech.2012.04.009"
"   Lebrazi S, 2020, J GENET ENG BIOTECHN, V18, DOI 10.1186/s43141-020-00035-9"
"   Leong YK, 2020, BIORESOURCE TECHNOL, V303, DOI 10.1016/j.biortech.2020.122886"
"   Li SM, 2021, PLANT CELL REP, V40, P271, DOI 10.1007/s00299-020-02612-1"
"   Li WC, 2010, PLANT SOIL, V326, P453, DOI 10.1007/s11104-009-0025-y"
"   Li WW, 2014, BIORESOURCE TECHNOL, V160, P15, DOI 10.1016/j.biortech.2013.11.074"
"   Liang YQ, 2021, CHEMOSPHERE, V285, DOI 10.1016/j.chemosphere.2021.131420"
"   Lin ZF, 2009, J EXP BOT, V60, P3311, DOI 10.1093/jxb/erp204"
"   Liu AR, 2022, CHEMOSPHERE, V302, DOI 10.1016/j.chemosphere.2022.134900"
"   Liu H, 2002, BIOTECHNOL BIOENG, V80, P806, DOI 10.1002/bit.10432"
"   Lu YZ, 2018, WATER RES, V130, P263, DOI 10.1016/j.watres.2017.11.045"
"   Lukatkin AS, 2007, RUSS J PLANT PHYSL+, V54, P381, DOI 10.1134/S1021443707030132"
"   Ma Y, 2016, J HAZARD MATER, V320, P36, DOI 10.1016/j.jhazmat.2016.08.009"
"   Ma Y, 2016, FRONT PLANT SCI, V7, DOI 10.3389/fpls.2016.00918"
"   Ma Y, 2015, FRONT PLANT SCI, V5, DOI 10.3389/fpls.2014.00757"
"   Manoj SR, 2020, J ENVIRON MANAGE, V254, DOI 10.1016/j.jenvman.2019.109779"
"   McCully M., 2005, Proceedings of the 4th World Congress on Allelopathy, ""Establishing the Scientific Base"", Wagga Wagga, New South the United Kingdom, Australia, 21-26 August 2005, P43"
"   Mehta P., 2019, ENDOPHYTIC FUNGI ROL, P183, DOI [10.1007/978-3-030-03589-1_9, DOI 10.1007/978-3-030-03589-1_9]"
"   Miller G, 2010, PLANT CELL ENVIRON, V33, P453, DOI 10.1111/j.1365-3040.2009.02041.x"
"   Misra S, 2020, 3 BIOTECH, V10, DOI 10.1007/s13205-020-2104-y"
"   Mitra S, 2018, MICROBIOL RES, V210, P12, DOI 10.1016/j.micres.2018.03.003"
"   Mousavi SM, 2018, ECOTOX ENVIRON SAFE, V147, P206, DOI 10.1016/j.ecoenv.2017.08.045"
"   Murthy S, 2011, AFR J BIOTECHNOL, V10, P15966, DOI 10.5897/AJB11.1645"
"   Mus F, 2016, APPL ENVIRON MICROB, V82, P3698, DOI 10.1128/AEM.01055-16"
"   Nagel R, 2018, FRONT MICROBIOL, V9, DOI 10.3389/fmicb.2018.02916"
"   Naila A, 2019, ENVIRON SCI POLLUT R, V26, P26449, DOI 10.1007/s11356-019-05992-4"
"   Naseem H, 2014, J PLANT INTERACT, V9, P689, DOI 10.1080/17429145.2014.902125"
"   NIETO KF, 1991, PLANT SOIL, V135, P213, DOI 10.1007/BF00010909"
"   Nocelli N, 2016, MATERIALS, V9, DOI 10.3390/ma9060418"
"   Ohyama T., 2010, NITROGEN ASSIM PLANT, V37, P1, DOI DOI 10.1109/TAC.2007.900829"
"   Olenska E, 2020, SCI TOTAL ENVIRON, V743, DOI 10.1016/j.scitotenv.2020.140682"
"   Padhan D., 2021, Sustain. Technol., P285, DOI DOI 10.1002/9781119547976.CH12"
"   Pandey N, 2016, EUR J SOIL BIOL, V75, P142, DOI 10.1016/j.ejsobi.2016.05.007"
"   Pandey S, 2013, J MICROBIOL, V51, P11, DOI 10.1007/s12275-013-2330-7"
"   Pantoja-Guerra M, 2023, CHEM BIOL TECHNOL AG, V10, DOI 10.1186/s40538-022-00370-8"
"   Patel K, 2015, SUSTAIN DEV BIODIVER, V12, P1, DOI 10.1007/978-3-319-24654-3_1"
"   Pattyn J, 2021, NEW PHYTOL, V229, P770, DOI 10.1111/nph.16873"
"   Penrose DM, 2001, CAN J MICROBIOL, V47, P368, DOI 10.1139/cjm-47-4-368"
"   Piotrowska-Niczyporuk A, 2018, PLANT PHYSIOL BIOCH, V132, P535, DOI 10.1016/j.plaphy.2018.09.038"
"   Prapagdee B, 2013, CHEMOSPHERE, V92, P659, DOI 10.1016/j.chemosphere.2013.01.082"
"   Renella G, 2004, GEODERMA, V122, P311, DOI 10.1016/j.geoderma.2004.01.018"
"   Rovira A. D., 1963, Plant and Soil, V19, P304, DOI 10.1007/BF01379484"
"   Sabat?? DC, 2018, MICROBIOL RES, V211, P21, DOI 10.1016/j.micres.2018.04.003"
"   Saha M, 2016, ENVIRON SCI POLLUT R, V23, P3984, DOI 10.1007/s11356-015-4294-0"
"   Samreen T, 2019, INT J AGRIC BIOL, V21, P538, DOI 10.17957/IJAB/15.0926"
"   Santos KFDN, 2017, PLANT SOIL, V415, P245, DOI 10.1007/s11104-016-3140-6"
"   Schmidt W, 2003, TRENDS PLANT SCI, V8, P188, DOI 10.1016/S1360-1385(03)00048-7"
"   Shen SL, 2023, J HAZARD MATER, V449, DOI 10.1016/j.jhazmat.2023.131040"
"   Si MF, 2018, J HEMATOL ONCOL, V11, DOI 10.1186/s13045-018-0645-x"
"   Singh RP, 2015, FRONT MICROBIOL, V6, DOI 10.3389/fmicb.2015.00937"
"   Sobariu DL, 2017, NEW BIOTECHNOL, V39, P125, DOI 10.1016/j.nbt.2016.09.002"
"   Stefan B., 2018, FEMS Microbiol. Lett, V365"
"   Strigul NS, 2006, ENVIRON MODELL SOFTW, V21, P1158, DOI 10.1016/j.envsoft.2005.06.003"
"   Sukul P, 2021, PLANT ARCH, V21, P628, DOI [10.51470/plantarchives.2021.v21.s1.096, DOI 10.51470/PLANTARCHIVES.2021.V21.S1.096, 10.51470/PLANTARCHIVES.2021.v21.S1.096]"
"   Taghavi S, 2009, APPL ENVIRON MICROB, V75, P748, DOI 10.1128/AEM.02239-08"
"   Tahir HAS, 2017, FRONT MICROBIOL, V8, DOI 10.3389/fmicb.2017.00171"
"   Tanimoto E, 2005, CRIT REV PLANT SCI, V24, P249, DOI 10.1080/07352680500196108"
"   Tank N, 2009, J BASIC MICROB, V49, P195, DOI 10.1002/jobm.200800090"
"   Tassi E, 2008, CHEMOSPHERE, V71, P66, DOI 10.1016/j.chemosphere.2007.10.027"

"   Teng ZD, 2019, J ENVIRON MANAGE, V231, P189, DOI 10.1016/j.jenvman.2018.10.012"
"   Tirry N, 2021, J GENET ENG BIOTECHN, V19, DOI 10.1186/s43141-021-00254-8"
"   Ul Hassan T, 2019, GEOMICROBIOL J, V36, P376, DOI 10.1080/01490451.2018.1562498"
"   Van de Poel B, 2015, PLANT PHYSIOL, V169, P61, DOI 10.1104/pp.15.00724"
"   van Loon LC, 1998, ANNU REV PHYTOPATHOL, V36, P453, DOI 10.1146/annurev.phyto.36.1.453"
"   Vaverkov?? MD, 2019, LAND USE POLICY, V89, DOI 10.1016/j.landusepol.2019.104234"
"   Vimalnath S, 2018, COLLOID SURFACE B, V172, P60, DOI 10.1016/j.colsurfb.2018.08.024"
"   Wagner S.C., 2011, NATURE ED KNOWLEDGE, V3, P15"
"   Wang JF, 2018, PLANT GROWTH REGUL, V84, P507, DOI 10.1007/s10725-017-0357-1"
"   Wang TJ, 2020, ENVIRON SCI POLLUT R, V27, P8707, DOI 10.1007/s11356-019-06957-3"
"   Wu Juanzi, 2019, Biotechnology Reports, V24, pe00374, DOI [10.1016/j.btre.2019.e00374, 10.1016/j.btre.2019.e00374]"
"   Yamaguchi S, 2008, ANNU REV PLANT BIOL, V59, P225, DOI 10.1146/annurev.arplant.59.032607.092804"
"   Yang L, 2021, ENVIRON PROG SUSTAIN, V40, DOI 10.1002/ep.13655"
"   Yang P, 2018, INT J ENV RES PUB HE, V15, DOI 10.3390/ijerph15071330"
"   Yin YR, 2011, INT BIODETER BIODEGR, V65, P1012, DOI 10.1016/j.ibiod.2011.08.001"
"   Yu G, 2020, INT J PHYTOREMEDIAT, V22, P383, DOI 10.1080/15226514.2019.1663488"
"   Yu S, 2024, SCI TOTAL ENVIRON, V912, DOI 10.1016/j.scitotenv.2023.168993"
"   Zaidi S, 2006, CHEMOSPHERE, V64, P991, DOI 10.1016/j.chemosphere.2005.12.057"
"   Zayed A, 1998, PLANTA, V206, P284, DOI 10.1007/s004250050402"
"   Zhang BG, 2020, GEOCHIM COSMOCHIM AC, V268, P296, DOI 10.1016/j.gca.2019.10.011"
"   Zhang QC, 2020, WATER AIR SOIL POLL, V231, DOI 10.1007/s11270-020-04728-2"
NR 169
TC 51
Z9 57
U1 104
U2 397
PU MDPI
PI BASEL
"PA ST ALBAN-ANLAGE 66, CH-4052 BASEL, SWITZERLAND"
EI 2076-2607
J9 MICROORGANISMS
JI Microorganisms
PD APR
PY 2024
VL 12
IS 4
AR 700
DI 10.3390/microorganisms12040700
PG 20
WC Microbiology
WE Science Citation Index Expanded (SCI-EXPANDED)
SC Microbiology
GA OY0X0
UT WOS:001210730500001
PM 38674644
"OA Green Submitted, gold"
DA 2026-04-06
ER

PT C
"AU Lopareva, A"
"   Goncharova, N"
"AF Lopareva, Alena"
"   Goncharova, Nadezhda"
"BE Hull, RN"
"   Barbu, CH"
"   Goncharova, N"
TI Plant-microbe symbiosis for bioremediation of heavy metal contaminated soil: Perspectives for Belarus
SO STRATEGIES TO ENHANCE ENVIRONMENTAL SECURITY IN TRANSITION COUNTRIES
SE NATO Science for Peace and Security Series C-Environmental Security
LA English
DT Proceedings Paper
CT NATO Advanced Research Workshop on Enhance Environmental Security in Transition Countries
"CY SEP 06-09, 2006"
"CL Sibiu, ROMANIA"
SP NATO
ID THLASPI-CAERULESCENS; ACCUMULATION; POPULATIONS; MODEL
"AB Problems of heavy metal and radionuclide contamination are very important for Belarus. About 23% of its territory is contaminated with radionuclides, some areas are heavily contaminated with Cu, Zn, Pb, and Cd. Heavy metals can be carcinogenic and mutagenic even at low doses, are not biodegradable and are easily accumulated by live organisms. Phytoremediation and bioremediation technologies are just beginning to be developed in Belarus. Some research studies in the field of phytostabilization and phytoextraction of heavy metals in soil are realized. However, mechanisms of heavy metal accumulation and hyperaccumulation for cleanup of contaminated soils need more consideration. The problem of hyperaccumulator plants consists of their low biomass and slow growth. So, different ways to improve heavy metal phytoremediation should be considered. Bacteria play an important role in soil-plant interaction and their influence on heavy metal contamination attracted attention. Plant-assisted bioremediation involves remediation of contaminated soils with both plants and microorganisms associated to their rhizosphere. Alfalfa with its symbiotic bacteria was successfully applied to the final step of remediation of organically polluted areas. But the mechanisms and the role of bacteria in heavy metal accumulation by plants need further research. We present our results concerning the role of bacterial polysaccharides in metal accumulation by plants. It is shown that metal

transfer into solution from elemental form depends on the exopolysaccharide (EPS) concentration in solution and glucuronic acid content in EPS. Bacterial polysaccharides do influence zinc uptake by the hyperaccumulator plant Thlaspi caerulescens. Possible perspectives for plant-bacterial remediation systems are discussed."
"C1 [Lopareva, Alena; Goncharova, Nadezhda] Int Sakharov Environm Univ, Lab Environm Monitoring, Minsk, BELARUS."
"RP Lopareva, A (corresponding author), Int Sakharov Environm Univ, Lab Environm Monitoring, Minsk, BELARUS."
EM lopareva@gmail.com; hotelinka@tut.by
FU NATO
FX The present work was carried out in collaboration with Laboratoire Sols
"   et Environnement (LSE), ENSAIA/INPL INRA, Nancy, France and supported by"
   NATO basic fellowship Effects of cultivation in association of heavy
   metal hyperaccumulator plants and leguminous plants in contaminated
   soils. The authors are grateful to professors Josiane and Bernard
"   Courtois for EPS (glucuronan, succinoglycan property of LPMV-CNRS FRE"
"   2779, IUT Amiens) used in this study."
"CR ALLISON JD, 1991, 600391021 EPA US EPA"
"   Assun??ao AGL, 2001, PLANT CELL ENVIRON, V24, P217, DOI 10.1111/j.1365-3040.2001.00666.x"
"   Assun??ao AGL, 2003, NEW PHYTOL, V159, P351, DOI 10.1046/j.1469-8137.2003.00820.x"
"   BAKER AJM, 1994, NEW PHYTOL, V127, P61, DOI 10.1111/j.1469-8137.1994.tb04259.x"
"   Banfield JF, 1999, P NATL ACAD SCI USA, V96, P3404, DOI 10.1073/pnas.96.7.3404"
"   Barker WW, 1998, AM MINERAL, V83, P1551, DOI 10.2138/am-1998-11-1243"
"   Chenu C, 1996, SOIL BIOL BIOCHEM, V28, P877, DOI 10.1016/0038-0717(96)00070-3"
"   Echevarria G, 1998, J ENVIRON QUAL, V27, P1064, DOI 10.2134/jeq1998.00472425002700050011x"
"   Lasat MM, 2000, J EXP BOT, V51, P71, DOI 10.1093/jexbot/51.342.71"
"   Lombi E, 2000, NEW PHYTOL, V145, P11, DOI 10.1046/j.1469-8137.2000.00560.x"
"   LOPAREVA A, 2003, P WORK GROUP 3 SOIL, P42"
"   LUKASHEV VK, 1996, HEAVY METAL CONTAMIN"
"   MAMARIL JC, 1989, ASEAN J SCI TECHNOL, V6, P75"
"   MCGRATH SP, 1995, J IND MICROBIOL, V14, P94, DOI 10.1007/BF01569890"
"   Mej??re M, 2001, TRENDS BIOTECHNOL, V19, P67, DOI 10.1016/S0167-7799(00)01534-1"
"   Schwartz C, 1999, PLANT SOIL, V208, P103, DOI 10.1023/A:1004519611152"
"   Welch SA, 1999, GEOCHIM COSMOCHIM AC, V63, P1405, DOI 10.1016/S0016-7037(99)00031-9"
NR 17
TC 2
Z9 2
U1 0
U2 25
PU SPRINGER
PI DORDRECHT
"PA PO BOX 17, 3300 AA DORDRECHT, NETHERLANDS"
SN 1871-4668
BN 978-1-4020-5994-0
J9 NATO SCI PEACE SECUR
JI NATO Sci. Peace Secur. Ser. C- Environ. Secur.
PY 2007
BP 401
EP +
DI 10.1007/978-1-4020-5996-4_29
PG 2
WC Environmental Sciences; Environmental Studies
WE Conference Proceedings Citation Index - Science (CPCI-S); Conference Proceedings Citation Index - Social Science & Humanities (CPCI-SSH)
SC Environmental Sciences & Ecology
GA BGF63
UT WOS:000246474500029
DA 2026-04-06
ER

PT J
"AU Tayang, A"
"   Songachan, LS"
"AF Tayang, Amanso"
"   Songachan, L. S."
TI Microbial bioremediation of heavy metals
SO CURRENT SCIENCE
LA English
DT Review
DE Bioremediation; heavy metals; microorganisms; metal-microbe
   interactions; resistance mechanisms
ID SACCHAROMYCES-CEREVISIAE; BACTERIAL-RESISTANCE; HEXAVALENT CHROMIUM;
   PROKARYOTIC METALLOTHIONEIN; POLLUTED ENVIRONMENTS; FUNCTIONAL-ANALYSIS;
   MERCURY RESISTANCE; PSEUDOMONAS-PUTIDA; AQUEOUS-SOLUTIONS;
   OUTER-MEMBRANE
"AB Heavy metals are persistent in nature and toxic to all life forms. Increase in industrialization, urbanization and unsafe agriculture practices is constantly adding heavy metals to the environment, and consequently causing heavy metal pollution of water and soil. Considering the negative impacts of heavy metals on the environment, several strategies have been devised to remediate them. However, most of these have their own limitations. Bioremediation of metals by microorganisms is efficient, cost-effective and environment friendly method of metal detoxification. Microbes can utilize metal contaminants as their energy source and transform them to less toxic forms. When exposed to metals for a considerable period of time, microorganisms interact with them and become tolerant by developing resistance mechanism against them. Metal-microbe interactions can occur in several ways such as biosorption, bioleaching, biomineralization, bioaccumulation and biotransformation. Study of these interactions is important to understand resistance

mechanisms against metals which include barriers, efflux system, sequestration and reduction of metals. These mechanisms are encoded by the resistance genes localized in chromosomes and plasmids. Understanding resistance mechanisms against metals in microorganisms becomes crucial for devising strategies for bioremediation of metals."
"C1 [Tayang, Amanso; Songachan, L. S.] Banaras Hindu Univ, Dept Bot, Varanasi 221005, Uttar Pradesh, India."
C3 Banaras Hindu University (BHU)
"RP Songachan, LS (corresponding author), Banaras Hindu Univ, Dept Bot, Varanasi 221005, Uttar Pradesh, India."
EM rayskybl@yahoo.co.in
"RI Songachan, L S/HOC-4363-2023"
"CR Ahemad M, 2012, IIOAB J, V3, P39"
"   Amirnia S, 2015, CHEM ENG J, V264, P863, DOI 10.1016/j.cej.2014.12.016"
"   Anjum F, 2012, HYDROMETALLURGY, V117, P1, DOI 10.1016/j.hydromet.2012.01.007"
"   Asghari I, 2013, J IND ENG CHEM, V19, P1069, DOI 10.1016/j.jiec.2012.12.005"
"   Asha S, 2009, BIOTECHNOL ADV, V27, P16, DOI 10.1016/j.biotechadv.2008.07.005"
"   Audina E., PHYCOBILIPROTEINS PR"
"   Ayangbenro AS, 2017, INT J ENV RES PUB HE, V14, DOI 10.3390/ijerph14010094"
"   Aziz HA, 2008, BIORESOURCE TECHNOL, V99, P1578, DOI 10.1016/j.biortech.2007.04.007"
"   Azzam AM, 2015, J ENVIRON ENG LANDSC, V23, P288, DOI 10.3846/16486897.2015.1068781"
"   Baaziz H, 2017, PLOS ONE, V12, DOI 10.1371/journal.pone.0188516"
"   Babak L., 2012, Acta Universitatis Agriculturae et Silviculturae Mendelianae Brunensis, V60, P9"
"   Bahobil A, 2017, International Journal of Current Microbiology and Applied Sciences, V6, P2138, DOI [10.20546/ijcmas.2017.606.253, DOI 10.20546/IJCMAS.2017.606.253, 10.20546/ijcmas.2017.606.253]"
"   Balzano S, 2020, FRONT MICROBIOL, V11, DOI 10.3389/fmicb.2020.00517"
"   Barakat MA, 2011, ARAB J CHEM, V4, P361, DOI 10.1016/j.arabjc.2010.07.019"
"   Barkay T, 2003, FEMS MICROBIOL REV, V27, P355, DOI 10.1016/S0168-6445(03)00046-9"
"   Barkay T, 2001, CURR OPIN MICROBIOL, V4, P318, DOI 10.1016/S1369-5274(00)00210-1"
"   Barquilha CER, 2017, J CLEAN PROD, V150, P58, DOI 10.1016/j.jclepro.2017.02.199"
"   Bazrafshan E, 2016, DESALIN WATER TREAT, V57, P14598, DOI 10.1080/19443994.2015.1065764"
"   BEARD SJ, 1995, FEMS MICROBIOL LETT, V131, P205, DOI 10.1016/0378-1097(95)00260-C"
"   Bhati Tushar, 2019, Microbiology and Biotechnology Letters, V47, P269, DOI [10.4014/mbl.1808.08006, 10.4014/mbl.1808.08006]"
"   Blindauer CA, 2008, METAL IONS BIOL MED, V10, P167"
"   Borremans B, 2001, J BACTERIOL, V183, P5651, DOI 10.1128/JB.183.19.5651-5658.2001"
"   Bosecker K, 1997, FEMS MICROBIOL REV, V20, P591, DOI 10.1111/j.1574-6976.1997.tb00340.x"
"   Bramhachari PV, 2007, J MICROBIOL BIOTECHN, V17, P44"
"   Brandl H., 2001, Biotechnology. Special processes, V10, P191, DOI [10.1002/9783527620999.ch8k, DOI 10.1002/9783527620999.CH8K]"
"   Bruins MR, 2000, ECOTOX ENVIRON SAFE, V45, P198, DOI 10.1006/eesa.1999.1860"
"   Burbank MB, 2011, GEOMICROBIOL J, V28, P301, DOI 10.1080/01490451.2010.499929"
"   C??novas D, 2003, ENVIRON MICROBIOL, V5, P1242, DOI [10.1111/j.1462-2920.2003.00463.x, 10.1046/j.1462-2920.2003.00463.x]"
"   Cardoso SL, 2017, J CLEAN PROD, V165, P491, DOI 10.1016/j.jclepro.2017.07.114"
"   Cervantes C., 2007, MOL MICROBIOLOGY HEA, P407, DOI [DOI 10.1007/7171_2006_087, 10.1007/7171_2006_087]"
"   CHA JS, 1991, P NATL ACAD SCI USA, V88, P8915, DOI 10.1073/pnas.88.20.8915"
"   Chang JJ, 2020, J HAZARD MATER, V396, DOI 10.1016/j.jhazmat.2020.122638"
"   Chen SN, 2014, BIORESOURCE TECHNOL, V158, P181, DOI 10.1016/j.biortech.2014.02.020"
"   Chibuike G.U., 2014, APPL ENVIRON SOIL SC, DOI [10.1155/2014/752708, DOI 10.1155/2014/752708]"
"   Christoforidis AK, 2015, CHEM ENG J, V277, P334, DOI 10.1016/j.cej.2015.04.138"
"   Clemens S, 2003, NEW PHYTOL, V159, P323, DOI 10.1046/j.1469-8137.2003.00811.x"
"   COOKSEY DA, 1993, MOL MICROBIOL, V7, P1, DOI 10.1111/j.1365-2958.1993.tb01091.x"
"   De Muynck W, 2010, ECOL ENG, V36, P118, DOI 10.1016/j.ecoleng.2009.02.006"
"   Demey H, 2018, CHEM ENG J, V332, P582, DOI 10.1016/j.cej.2017.09.083"
"   Dhal B, 2018, SUSTAIN ENVIRON RES, V28, P289, DOI 10.1016/j.serj.2018.05.002"
"   Edelstein M, 2018, SCI HORTIC-AMSTERDAM, V234, P431, DOI 10.1016/j.scienta.2017.12.039"
"   Fashola MO, 2016, INT J ENV RES PUB HE, V13, DOI 10.3390/ijerph13111047"
"   Fomina M, 2014, BIORESOURCE TECHNOL, V160, P3, DOI 10.1016/j.biortech.2013.12.102"
"   Franke S, 2003, J BACTERIOL, V185, P3804, DOI 10.1128/JB.185.13.3804-3812.2003"
"   Fu FL, 2011, J ENVIRON MANAGE, V92, P407, DOI 10.1016/j.jenvman.2010.11.011"
"   Fu YongQian Fu YongQian, 2012, African Journal of Biotechnology, V11, P1403"
"   Garg N, 2011, ENVIRON CHEM LETT, V9, P303, DOI 10.1007/s10311-011-0313-7"
"   Gavrilescu M, 2004, ENG LIFE SCI, V4, P219, DOI 10.1002/elsc.200420026"
"   Geva P, 2016, ENVIRON SCI POLLUT R, V23, P19613, DOI 10.1007/s11356-016-7157-4"
"   Gilotra U, 1997, CURR MICROBIOL, V34, P378, DOI 10.1007/s002849900199"
"   Gordon T, 2003, MUTAT RES-FUND MOL M, V533, P99, DOI 10.1016/j.mrfmmm.2003.08.022"
"   Grujic S, 2017, WATER AIR SOIL POLL, V228, DOI 10.1007/s11270-017-3259-y"
"   Gupta Pratima, 2017, Biotechnol Rep (Amst), V13, P58, DOI [10.1016/j.btre.2016.12.006, 10.1016/j.btre.2016.12.006]"
"   Gupta VK, 2015, ENVIRON ENG RES, V20, P1, DOI 10.4491/eer.2015.018"
"   Guti??rrez-Corona JF, 2016, WORLD J MICROB BIOT, V32, DOI 10.1007/s11274-016-2150-0"
"   Hlihor RM, 2017, PROCESS SAF ENVIRON, V108, P44, DOI 10.1016/j.psep.2016.06.016"
"   Hobman JL, 1996, MOL GEN GENET, V250, P129, DOI 10.1007/s004380050059"
"   HOYLE B, 1983, APPL ENVIRON MICROB, V46, P749, DOI 10.1128/AEM.46.3.749-752.1983"
"   HUCKLE JW, 1993, MOL MICROBIOL, V7, P177, DOI 10.1111/j.1365-2958.1993.tb01109.x"
"   Ianeva O D, 2009, Mikrobiol Z, V71, P54"
"   Igiri BE, 2018, J TOXICOL-US, V2018, DOI 10.1155/2018/2568038"
"   Iram S., 2015, International Journal of Scientific and Research Publications, V5, P1, DOI DOI 10.1007/S13369-015-1702-1"
"   Jafari M, 2019, MIN PROC EXT MET REV, V40, P87, DOI 10.1080/08827508.2018.1481063"
"   Jaishankar Monisha, 2014, Interdiscip Toxicol, V7, P60, DOI 10.2478/intox-2014-0009"
"   Jalilvand N, 2020, J SOIL SCI PLANT NUT, V20, P206, DOI 10.1007/s42729-019-00121-z"
"   Jalmi SK, 2018, FRONT PLANT SCI, V9, DOI 10.3389/fpls.2018.00012"
"   JI GY, 1995, J IND MICROBIOL, V14, P61, DOI 10.1007/BF01569887"
"   JI GY, 1992, P NATL ACAD SCI USA, V89, P9474, DOI 10.1073/pnas.89.20.9474"

"   JI GY, 1994, BIOCHEMISTRY-US, V33, P7294, DOI 10.1021/bi00189a034"
"   Jiang J, 2015, ECOTOX ENVIRON SAFE, V120, P386, DOI 10.1016/j.ecoenv.2015.06.028"
"   Joshi N, 2018, FRONT ENV SCI-SWITZ, V6, DOI 10.3389/fenvs.2018.00127"
"   Kang CH, 2016, ECOL ENG, V89, P64, DOI 10.1016/j.ecoleng.2016.01.023"
"   Karigar Chandrakant S, 2011, Enzyme Res, V2011, P805187, DOI 10.4061/2011/805187"
"   Kazy SK, 2002, WORLD J MICROB BIOT, V18, P583, DOI 10.1023/A:1016354713289"
"   Khadivinia E, 2014, J IND ENG CHEM, V20, P4304, DOI 10.1016/j.jiec.2014.01.037"
"   Khan A. A., INT J ENVIRON AN CH, P1"
"   Krebs W, 1997, FEMS MICROBIOL REV, V20, P605, DOI 10.1111/j.1574-6976.1997.tb00341.x"
"   Kumar A., 2011, International Journal of Environmental Sciences, V1, P1079"
"   Kumar NM, 2018, ENERGY ENV SUSTAIN, P53, DOI 10.1007/978-981-10-7485-1_4"
"   Kumar R., 2014, J OFNUCLEAR PHYS MAT, V7, P213, DOI DOI 10.15415/JNP.2014.12018"
"   Kumari S, 2019, ENVIRON SCI POLLUT R, V26, P28763, DOI 10.1007/s11356-019-05916-2"
"   Kushwaha A, 2017, ENVIRON SCI POLLUT R, V24, P10652, DOI 10.1007/s11356-017-8752-8"
"   Lee YC, 2011, BIORESOURCE TECHNOL, V102, P5297, DOI 10.1016/j.biortech.2010.12.103"
"   Leong YK, 2020, BIORESOURCE TECHNOL, V303, DOI 10.1016/j.biortech.2020.122886"
"   Li CS, 2013, BIORESOURCE TECHNOL, V143, P46, DOI 10.1016/j.biortech.2013.05.114"
"   Li JX, 1996, J BIOL CHEM, V271, P25247, DOI 10.1074/jbc.271.41.25247"
"   Li LT, 1997, J BIOL CHEM, V272, P28485, DOI 10.1074/jbc.272.45.28485"
"   Liang K, 2016, CHEM COMMUN, V52, P473, DOI 10.1039/c5cc07577g"
"   Lin ZY, 2020, ECOTOX ENVIRON SAFE, V190, DOI 10.1016/j.ecoenv.2019.110089"
"   Liu SJ, 2018, CHEMOSPHERE, V208, P655, DOI 10.1016/j.chemosphere.2018.06.023"
"   Liu XF, 1997, J BIOL CHEM, V272, P11763, DOI 10.1074/jbc.272.18.11763"
"   Lloyd JR, 2001, CURR OPIN BIOTECH, V12, P248, DOI 10.1016/S0958-1669(00)00207-X"
"   Lloyd JR., 2002, Microbiol. Today, V29, P67"
"   Ma LL, 2019, ECOTOX ENVIRON SAFE, V170, P763, DOI 10.1016/j.ecoenv.2018.12.041"
"   Ma XK, 2014, APPL MICROBIOL BIOT, V98, P9817, DOI 10.1007/s00253-014-5905-2"
"   Machado MD, 2010, J HAZARD MATER, V180, P347, DOI 10.1016/j.jhazmat.2010.04.037"
"   Magnin JP, 2014, CAN J MICROBIOL, V60, P829, DOI 10.1139/cjm-2014-0231"
"   MERGEAY M, 1985, J BACTERIOL, V162, P328, DOI 10.1128/JB.162.1.328-334.1985"
"   Michael G, 2014, FUNGAL BIOL REV, V28, P36, DOI 10.1016/j.fbr.2014.05.001"
"   Mishra A, 2013, CRIT REV ENV SCI TEC, V43, P1162, DOI 10.1080/10934529.2011.627044"
"   Mishra D, 2005, MET MATER INT, V11, P249, DOI 10.1007/BF03027450"
"   Mukhopadhyay R, 2002, FEMS MICROBIOL REV, V26, P311, DOI 10.1111/j.1574-6976.2002.tb00617.x"
"   Nagajyoti PC, 2010, ENVIRON CHEM LETT, V8, P199, DOI 10.1007/s10311-010-0297-8"
"   Nagvenkar GS, 2010, ECOTOXICOLOGY, V19, P604, DOI 10.1007/s10646-009-0429-8"
"   Nanda Manisha, 2011, International Journal of Environmental Sciences, V2, P781"
"   Nanda M., 2011, Global Journal of Applied Environmental Sciences, V2, P127"
"   Nanda M, 2019, AQUAT TOXICOL, V212, P1, DOI 10.1016/j.aquatox.2019.04.011"
"   Naskar A, 2020, ENVIRON TECHNOL INNO, V20, DOI 10.1016/j.eti.2020.101057"
"   Hakizimana JN, 2017, DESALINATION, V404, P1, DOI 10.1016/j.desal.2016.10.011"
"   Nies DH, 1999, APPL MICROBIOL BIOT, V51, P730, DOI 10.1007/s002530051457"
"   NIES DH, 1989, J BACTERIOL, V171, P4073, DOI 10.1128/jb.171.7.4073-4075.1989"
"   NIES DH, 1995, J IND MICROBIOL, V14, P186, DOI 10.1007/BF01569902"
"   NIES DH, 1992, J BACTERIOL, V174, P8102, DOI 10.1128/JB.174.24.8102-8110.1992"
"   NIES DH, 1989, P NATL ACAD SCI USA, V86, P7351, DOI 10.1073/pnas.86.19.7351"
"   Ojuederie OB, 2017, INT J ENV RES PUB HE, V14, DOI 10.3390/ijerph14121504"
"   OLAFSON RW, 1979, BIOCHEM BIOPH RES CO, V89, P36, DOI 10.1016/0006-291X(79)90939-2"
"   Onta??on OM, 2018, ENVIRON SCI POLLUT R, V25, P16111, DOI 10.1007/s11356-018-1764-1"
"   Pervaiz I, 2013, Prikl Biokhim Mikrobiol, V49, P435"
"   Pinto E, 2003, J PHYCOL, V39, P1008, DOI 10.1111/j.0022-3646.2003.02-193.x"
"   Porphyridium sp, 2017, IOP C SERIES EARTH E, V160"
"   Pradhan D, 2019, J CLEAN PROD, V209, P617, DOI 10.1016/j.jclepro.2018.10.288"
"   Rahman Z, 2019, J BASIC MICROB, V59, P477, DOI 10.1002/jobm.201900024"
"   Ramrakhiani L, 2011, CHEM ENG J, V171, P1060, DOI 10.1016/j.cej.2011.05.002"
"   Rani A, 2009, MICROBIAL STRATEGIES FOR CROP IMPROVEMENT, P85, DOI 10.1007/978-3-642-01979-1_5"
"   Rani M. J., 2010, African Journal of Environmental Science and Technology, V4, P77"
"   Rasulov B.A., 2013, J. Environ. Prot, V4, P989, DOI DOI 10.4236/JEP.2013.49114"
"   RICHARDS RI, 1984, CELL, V37, P263, DOI 10.1016/0092-8674(84)90322-2"
"   Riskuwa-Shehu ML, 2019, Microbiology Research Journal International, P1, DOI [10.9734/mrji/2019/v27i630114, https://doi.org/10.9734/mrji/2019/v27i630114, DOI 10.9734/MRJI/2019/V27I630114, 10.9734/MRJI/2019/v27i630114]"
"   Rohwerder T, 2003, APPL MICROBIOL BIOT, V63, P239, DOI 10.1007/s00253-003-1448-7"
"   Rosen BP, 2002, FEBS LETT, V529, P86, DOI 10.1016/S0014-5793(02)03186-1"
"   ROSENSTEIN R, 1992, J BACTERIOL, V174, P3676, DOI 10.1128/JB.174.11.3676-3683.1992"
"   Saavedra R, 2018, BIORESOURCE TECHNOL, V263, P49, DOI 10.1016/j.biortech.2018.04.101"
"   Sahmoune MN, 2018, MICROCHEM J, V141, P87, DOI 10.1016/j.microc.2018.05.009"
"   Samarth D. P., 2012, INT J PURE APPL SCI, V10, P12"
"   Saranya K, 2018, J ENVIRON MANAGE, V222, P396, DOI 10.1016/j.jenvman.2018.05.083"
"   Saxena D, 1998, WORLD J MICROB BIOT, V14, P921, DOI 10.1023/A:1008827623994"
"   Say R, 2001, BIORESOURCE TECHNOL, V76, P67, DOI 10.1016/S0960-8524(00)00071-7"
"   Sayadi MH, 2019, J ENVIRON CHEM ENG, V7, DOI 10.1016/j.jece.2019.103169"
"   SCHIERING N, 1991, NATURE, V352, P168, DOI 10.1038/352168a0"
"   SCHMIDT T, 1994, J BACTERIOL, V176, P7045, DOI 10.1128/JB.176.22.7045-7054.1994"
"   Shafiquzzaman Siddiquee Shafiquzzaman Siddiquee, 2015, Journal of Microbial and Biochemical Technology, V7, P384, DOI 10.4172/1948-5948.1000243"
"   Shahpiri A, 2018, ANN MICROBIOL, V68, P145, DOI 10.1007/s13213-018-1326-2"
"   Sharma PK, 2000, APPL ENVIRON MICROB, V66, P3083, DOI 10.1128/AEM.66.7.3083-3087.2000"
"   Sher S, 2019, APPL MICROBIOL BIOT, V103, P6007, DOI 10.1007/s00253-019-09933-6"
"   Shukla SK, 2020, J HAZARD MATER, V384, DOI 10.1016/j.jhazmat.2019.121316"
"   Silver S, 1996, GENE, V179, P9, DOI 10.1016/S0378-1119(96)00323-X"
"   Silver S, 1996, ANNU REV MICROBIOL, V50, P753, DOI 10.1146/annurev.micro.50.1.753"
"   SILVER S, 1989, TRENDS BIOCHEM SCI, V14, P76, DOI 10.1016/0968-0004(89)90048-0"
"   SILVER S, 1994, ENVIRON HEALTH PERSP, V102, P107, DOI 10.2307/3431771"

"   SILVER S, 1992, MICROBIOL REV, V56, P195, DOI 10.1128/MMBR.56.1.195-228.1992"
"   Smirnova G F, 2005, Mikrobiol Z, V67, P11"
"   Solisio C, 2019, ENVIRON TECHNOL, V40, P664, DOI 10.1080/09593330.2017.1400114"
"   Srichandan H, 2014, HYDROMETALLURGY, V150, P130, DOI 10.1016/j.hydromet.2014.09.019"
"   SUMMERS AO, 1972, J BACTERIOL, V112, P1228, DOI 10.1128/JB.112.3.1228-1236.1972"
"   Talukdar D, 2020, ENVIRON TECHNOL INNO, V20, DOI 10.1016/j.eti.2020.101050"
"   Teitzel GM, 2003, APPL ENVIRON MICROB, V69, P2313, DOI 10.1128/AEM.69.4.2313-2320.2003"
"   Thatoi H, 2014, J ENVIRON MANAGE, V146, P383, DOI 10.1016/j.jenvman.2014.07.014"
"   Tolbatov I, 2020, INORG CHEM, V59, P790, DOI 10.1021/acs.inorgchem.9b03059"
"   Tsekova K, 2010, BIORESOURCE TECHNOL, V101, P1727, DOI 10.1016/j.biortech.2009.10.012"
"   Ungureanu G, 2015, ENVIRON ENG MANAG J, V14, P455, DOI 10.30638/eemj.2015.047"
"   Valko M, 2016, ARCH TOXICOL, V90, P1, DOI 10.1007/s00204-015-1579-5"
"   Vasudevan P, 2001, J SCI IND RES INDIA, V60, P112"
"   Vats N, 2001, MICROBIOL-UK, V147, P653, DOI 10.1099/00221287-147-3-653"
"   Verma S, 2019, ENVIRON TECHNOL INNO, V14, DOI 10.1016/j.eti.2019.100369"
"   Vestola EA, 2010, HYDROMETALLURGY, V103, P74, DOI 10.1016/j.hydromet.2010.02.017"
"   Webb R., 1999, J Hazard Subst Res, V2, P1, DOI DOI 10.4148/1090-7025.1011"
"   WU JH, 1993, MOL MICROBIOL, V8, P615, DOI 10.1111/j.1365-2958.1993.tb01605.x"
"   Wu SJ, 2019, APPL MICROBIOL BIOT, V103, P2701, DOI 10.1007/s00253-019-09666-6"
"   Yang YR, 2016, GEOMICROBIOL J, V33, P179, DOI 10.1080/01490451.2015.1068888"
"   Yin K, 2019, CHEM ENG J, V360, P1553, DOI [10.1016/j.cej.2018.10.226, 10.1016/j.jhazmat.2024.134410]"
"   Yin K, 2016, WATER RES, V103, P383, DOI 10.1016/j.watres.2016.07.053"
"   Zhang KJ, 2019, CHEMOSPHERE, V224, P272, DOI 10.1016/j.chemosphere.2019.02.140"
"   Zhang WM, 2007, T NONFERR METAL SOC, V17, P1123, DOI 10.1016/S1003-6326(07)60236-2"
NR 173
TC 39
Z9 50
U1 16
U2 418
PU INDIAN ACAD SCIENCES
PI BANGALORE
"PA C V RAMAN AVENUE, SADASHIVANAGAR, P B #8005, BANGALORE 560 080, INDIA"
SN 0011-3891
J9 CURR SCI INDIA
JI Curr. Sci.
PD MAR 25
PY 2021
VL 120
IS 6
BP 1013
EP 1025
DI 10.18520/cs/v120/i6/1013-1025
PG 13
WC Multidisciplinary Sciences
WE Science Citation Index Expanded (SCI-EXPANDED)
SC Science & Technology - Other Topics
GA RE9HX
UT WOS:000634462300026
OA gold
DA 2026-04-06
ER

PT J
"AU Yu, JA"
"   Chen, Z"
"   Gao, WH"
"   He, S"
"   Xiao, D"
"   Fan, W"
"   Huo, MX"
"   Nugroho, WA"
"AF Yu, Jia'ao"
"   Chen, Zhen"
"   Gao, Wenhao"
"   He, Shuang"
"   Xiao, Dan"
"   Fan, Wei"
"   Huo, Mingxin"
"   Nugroho, Wahyunanto Agung"
TI Global trends and prospects in research on heavy metal pollution at
   contaminated sites
SO JOURNAL OF ENVIRONMENTAL MANAGEMENT
LA English
DT Article
DE Contaminated site; Heavy metal pollution; Pollution assessment;
   Bibliometric analysis
ID SOIL; CHINA; REMEDIATION; TECHNOLOGIES
"AB Soil contamination by heavy metals is a global environmental challenge, poses a serious risk to plant life, human health, and global food supply. In recent years, advanced and effective remediation strategies for heavy metal-contaminated soils have developed rapidly, and a systematic summarization of this progress is important. Based on 2822 bibliographic data (2014-2023) acquired from the Web of Science Core Collection, the research status, hotspots and trends of heavy metal-contaminated sites worldwide had been synthetically analyzed in scientometrics. The results showed that China, India, and the USA were the most active countries in related research, and the main research subjects were the assessment of contaminated sites and remediation

methods. The keywords showed 3 co-occurrence clusters, focusing on site characterization, phytoremediation and physical-chemical remediation. The time zone view shows a multidisciplinary amalgamation tendency particular in methods such as using artificial intelligence and remote sensing to predict spatial distribution and migration characteristics of heavy metals. Regarding the types of contaminated sites, the most frequently studied are mining sites and industrial waste sites. Among heavy metals, Cd, Pb, and Zn are the most commonly studied pollutants. The heavy metal contamination in soil, ranked by the geo-accumulation index, is as follows: Cd (5.91) > Pb (4.12) > Zn (3.73) > Cu (2.37) > Cr (1.85) > Ni (1.34). In terms of remediation technologies, the frequency of utilization is ranked as follows: phytoremediation > microbial remediation > soil washing > electrokinetic remediation > oxidation reduction > solidification/stabilization > thermal desorption > soil replacement. Additionally, phytoremediation and soil washing are the most effective technologies for removing Cd, Pb, and Zn. The insights derived from this study contribute to a deeper understanding of the state-of-the-art in this field and help to develop/select applicable methods for the effective remediation of heavy metal-contaminated sites."
"C1 [Yu, Jia'ao; Chen, Zhen; Gao, Wenhao; He, Shuang; Fan, Wei; Huo, Mingxin] Northeast Normal Univ, Sch Environm, 2555 Jingyue St, Changchun 130117, Peoples R China."
"   [Xiao, Dan] Jilin Acad Agr Sci, Changchun 130033, Peoples R China."
"   [Nugroho, Wahyunanto Agung] Brawijaya Univ, Dept Biosyst Engn, Malang 65145, Indonesia."
C3 Northeast Normal University - China; Jilin Academy of Agricultural
   Sciences; Brawijaya University
"RP Fan, W (corresponding author), Northeast Normal Univ, Sch Environm, 2555 Jingyue St, Changchun 130117, Peoples R China."
EM fanw100@nenu.edu.cn
"RI Nugroho, Wahyunanto Agung/JSL-0182-2023"
FU National Key Research and Develop-ment Program of China
   [2023YFC3706700]; National Natural Science Foundation of China
"   [52370032, 52270089]"
FX This work was funded by the National Key Research and Develop-ment
   Program of China (No. 2023YFC3706700) . It was also supported by the
   National Natural Science Foundation of China (No. 52370032 and 52270089)
   .
"CR Amin K., 2021, Nat. Commun., V12"
"   Anas R., 2022, Int. J. Mol. Sci., V23"
"   Chen CM, 2006, J AM SOC INF SCI TEC, V57, P359, DOI 10.1002/asi.20317"
"   Chiroma T.M., 2014, Int. J. Eng. Sci., V3"
"   Dada E., 2015, Ethiop. J. Environ. Stud. Manag, V8"
"   Dermont G, 2008, J HAZARD MATER, V152, P1, DOI 10.1016/j.jhazmat.2007.10.043"
"   Dong ZY, 2013, J HAZARD MATER, V260, P399, DOI 10.1016/j.jhazmat.2013.05.003"
"   Falciglia PP, 2011, J HAZARD MATER, V185, P392, DOI 10.1016/j.jhazmat.2010.09.046"
"   Foley JA, 2011, NATURE, V478, P337, DOI 10.1038/nature10452"
"   Gao B., 2023, Environ. Prot. Sci., V21"
"   Han Y, 2019, PROCESS SAF ENVIRON, V125, P189, DOI 10.1016/j.psep.2019.03.020"
"   Hou DM, 2019, J ENVIRON MANAGE, V251, DOI 10.1016/j.jenvman.2019.109542"
"   Juan D.A., 2022, J. For. Environ., V10"
"   Kong LL, 2018, J HAZARD MATER, V343, P276, DOI 10.1016/j.jhazmat.2017.09.040"
"   Li H, 2019, SCI TOTAL ENVIRON, V687, P687, DOI 10.1016/j.scitotenv.2019.06.013"
"   Li K, 2023, ISCIENCE, V26, DOI 10.1016/j.isci.2023.108124"
"   Li M, 2022, CHEMOSPHERE, V304, DOI 10.1016/j.chemosphere.2022.135337"
"   Li XN, 2017, J CLEAN PROD, V147, P485, DOI 10.1016/j.jclepro.2017.01.107"
"   Liu G, 2020, J CLEAN PROD, V251, DOI 10.1016/j.jclepro.2019.119721"
"   Liu XJ, 2024, J ENVIRON MANAGE, V360, DOI 10.1016/j.jenvman.2024.121165"
"   Luo MF, 2024, ENVIRON SCI TECHNOL, V58, P18041, DOI 10.1021/acs.est.4c06557"
"   Luo XH, 2023, J ENVIRON SCI, V125, P662, DOI 10.1016/j.jes.2022.01.029"
"   Ma Y., 2022, Stabilization and Remediation of Heavy Metal-Contaminated Soils in China: Insights from a Decade-Long National Survey, V39"
"   Mahendra K., 2021, Environmental and Sustainability Indicators, V12"
"   Malgorzata C.D., 2020, Sci. Rep., V10"
"   Min T, 2021, ECOTOX ENVIRON SAFE, V226, DOI 10.1016/j.ecoenv.2021.112842"
"   Muller G., 1969, GEOLOGICAL J, V2, P108"
"   Nathaniel D.M., 2012, Nature, V490"
"   Nguyen TB, 2023, ENVIRON POLLUT, V337, DOI 10.1016/j.envpol.2023.122571"
"   Ossai IC, 2020, ENVIRON TECHNOL INNO, V17, DOI 10.1016/j.eti.2019.100526"
"   Qu C.S., 2016, Environ. Sci. Technol., V24"
"   Ronald A., 2015, Science, V348"
"   Sathish P.G., 2018, Process. Saf. Environ, V118"
"   Shen ZT, 2019, ENVIRON SCI TECHNOL, V53, P11615, DOI 10.1021/acs.est.9b04990"
"   Song PP, 2022, SCI TOTAL ENVIRON, V838, DOI 10.1016/j.scitotenv.2022.156417"
"   Steliga T, 2020, ECOTOX ENVIRON SAFE, V194, DOI 10.1016/j.ecoenv.2020.110409"
"   Sushma D., 2022, J. Environ. Manag., V320"
"   Tang XJ, 2010, J HAZARD MATER, V173, P653, DOI 10.1016/j.jhazmat.2009.08.134"
"   Thanh K.N., 2021, J. Environ. Manag., V296"
"   Tian ZX, 2022, ENVIRON MONIT ASSESS, V194, DOI 10.1007/s10661-022-10470-5"
"   Tilman D., 2011, P NATL ACAD SCI, P108"
"   Tran HT, 2022, J HAZARD MATER, V421, DOI 10.1016/j.jhazmat.2021.126767"
"   Ubando AT, 2021, J HAZARD MATER, V402, DOI 10.1016/j.jhazmat.2020.123431"
"   Wang H.W., 2024, Soil. Sci. Environ, V17"
"   Wang JX, 2021, INT J ENV RES PUB HE, V18, DOI 10.3390/ijerph18168560"
"   Wang LX, 2008, J GEOGR SCI, V18, P353, DOI 10.1007/s11442-008-0353-5"
"   Wang S.C., 2022, Waste Manage. (Tucson, Ariz.), V139, P2014"
"   Wang XQ, 2021, BIORESOURCE TECHNOL, V320, DOI 10.1016/j.biortech.2020.124331"
"   Wang Y, 2020, ECOTOX ENVIRON SAFE, V192, DOI 10.1016/j.ecoenv.2020.110294"
"   Wei JJ, 2023, ENVIRON POLLUT, V337, DOI 10.1016/j.envpol.2023.122562"
"   Xiao L, 2019, ENVIRON SCI POLLUT R, V26, P22596, DOI 10.1007/s11356-019-04706-0"
"   Xu DM, 2021, J CLEAN PROD, V321, DOI 10.1016/j.jclepro.2021.128730"
"   Xu M.J., 2022, Int. J. Environ. Res. Publ. Health, V20"

"   Xv LL, 2020, ENVIRON POLLUT, V263, DOI 10.1016/j.envpol.2020.114401"
"   Yan K, 2022, J CLEAN PROD, V373, DOI 10.1016/j.jclepro.2022.133780"
"   Yang SH, 2022, ENVIRON RES, V206, DOI 10.1016/j.envres.2021.112277"
"   Yu JJ, 2024, SUSTAINABILITY-BASEL, V16, DOI 10.3390/su16041703"
"   Yuan XH, 2021, J ENVIRON SCI, V101, P217, DOI [10.1016/j.jes.2020.08.01.3, 10.1016/j.jes.2020.08.013]"
"   Zhang Q, 2022, J HAZARD MATER, V429, DOI 10.1016/j.jhazmat.2022.128362"
"   Zhang SQ, 2023, WATER AIR SOIL POLL, V234, DOI 10.1007/s11270-023-06394-6"
"   Zhao C, 2019, CHEMOSPHERE, V221, P841, DOI 10.1016/j.chemosphere.2019.01.079"
"   Zhu JX, 2022, ENVIRON RES, V203, DOI 10.1016/j.envres.2021.111802"
NR 62
TC 20
Z9 20
U1 73
U2 140
PU ACADEMIC PRESS LTD- ELSEVIER SCIENCE LTD
PI LONDON
"PA 24-28 OVAL RD, LONDON NW1 7DX, the United Kingdom"
SN 0301-4797
EI 1095-8630
J9 J ENVIRON MANAGE
JI J. Environ. Manage.
PD MAY
PY 2025
VL 383
AR 125402
DI 10.1016/j.jenvman.2025.125402
EA APR 2025
PG 14
WC Environmental Sciences
WE Science Citation Index Expanded (SCI-EXPANDED)
SC Environmental Sciences & Ecology
GA 1ZV2F
UT WOS:001477567000001
PM 40262497
OA hybrid
DA 2026-04-06
ER

PT J
"AU Lv, Y"
"   Zhu, XZ"
"   Zhang, MJ"
"   Liu, XY"
"   Wang, JL"
"AF Lv, Ying"
"   Zhu, Xuezhe"
"   Zhang, Mingjiang"
"   Liu, Xingyu"
"   Wang, Jianlei"
TI In-situ Bioremediation of Multiple Heavy Metals Contaminated
   Farmland Soil by Sulfate-Reducing Bacteria
SO POLISH JOURNAL OF ENVIRONMENTAL STUDIES
LA English
DT Article
DE Keywords; multiple heavy metals; farmland; sulfate-reducing bacteria;
   bioremediation; community
ID REMEDIATION; BIOCHAR
"AB Heavy metal pollution of farmland soil deteriorates the cultivated land quality, and adversely affects the growth, yield, and quality of crops. Besides, it hampers food safety, and poses a tremendous threat to human health. In this study, an in-situ bioremediation process was investigated in maize planting areas by spraying sulfate-reducing bacteria solution based on previous studies. The altered basic physical and chemical properties of heavy metals in soil were studied using the Tessier sequential extraction technique to investigate the efficiency of the bioremediation process. The environmental effects of the bioremediation process were also explored by investigating the crop characteristics. The analysis of microbial species composition was used to explore the influence of exogenous microbes on microbial composition in farmland soil. The outcomes of remediation experiments showed that the sulfate-reducing bacteria could stabilize heavy metals in soil, inhibit the uptake of heavy metals by plants, improve the quality of maize products and promote the growth of maize plants. In this study, we investigated the in-situ bioremediation of multiple heavy metals contaminated farmland soil using sulfate-reducing bacteria. The outcomes of the study revealed the unique advantages of bioremediation technology in practical application of heavy metals contaminated farmland soil."
"C1 [Lv, Ying; Zhu, Xuezhe; Zhang, Mingjiang; Liu, Xingyu; Wang, Jianlei] GRINM Grp Corp Ltd, Natl Engn Lab Biohydromet, Beijing, Peoples R China."
"   [Lv, Ying] Univ Sci & Technol, Sch Met & Ecol Engn, Beijing, Peoples R China."
"   [Lv, Ying; Zhu, Xuezhe; Zhang, Mingjiang; Liu, Xingyu; Wang, Jianlei] GRINM Resources & Environm Tech Co Ltd, Beijing, Peoples R China."
"   [Lv, Ying; Zhu, Xuezhe; Zhang, Mingjiang; Liu, Xingyu; Wang, Jianlei] Gen Res Inst Nonferrous Met, Beijing, Peoples R China."
"   [Zhang, Mingjiang; Liu, Xingyu; Wang, Jianlei] GRIMAT Engn Inst Co Ltd, Beijing, Peoples R China."
C3 University of Science & Technology Beijing; General Research Institute
   for Nonferrous Metals
"RP Zhang, MJ; Liu, XY (corresponding author), GRINM Grp Corp Ltd, Natl Engn Lab Biohydromet, Beijing, Peoples R China.; Zhang, MJ; Liu, XY (corresponding author), GRINM Resources & Environm Tech Co Ltd, Beijing,

Peoples R China.; Zhang, MJ; Liu, XY (corresponding author), Gen Res Inst Nonferrous Met, Beijing, Peoples R China.; Zhang, MJ; Liu, XY (corresponding author), GRIMAT Engn Inst Co Ltd, Beijing, Peoples R China."
EM zmj0630@163.com; wellwoodliu@163.com
"OI Lv, Ying/0000-0003-3026-0498"
"FU National Natural Science Foundation of China [U1402234, 41573074,"
51974279]; National Key Research & Development Program of China
"   [2018YFC18018, 2018YFC18027, 2019YFC18059]; Guangxi Scientific Research"
"   and Technology Development Plan [GuikeAB16380287, GuikeAB17129025];"
KeJunPing [2018] [159]
FX This project is financially supported by the National Natural Science
"   Foundation of China [grant numbers U1402234, 41573074 and 51974279], the"
National Key Research & Development Program of China [grant numbers
"   2018YFC18018, 2018YFC18027 and 2019YFC18059], KeJunPing [2018] No. 159,"
the Guangxi Scientific Research and Technology Development Plan [grant
"   numbers GuikeAB16380287 and GuikeAB17129025], which are greatly"
appreciated.
"CR [Anonymous], 112122006 NYT"
"   Cameselle C, 2016, PROCESS SAF ENVIRON, V104, P209, DOI 10.1016/j.psep.2016.09.002"
"   Chen Z, 2020, J HAZARD MATER, V395, DOI 10.1016/j.jhazmat.2020.122665"
"   Deng MH, 2020, J ENVIRON MANAGE, V260, DOI 10.1016/j.jenvman.2020.110092"
"   Dolev N, 2020, ENVIRON RES, V183, DOI 10.1016/j.envres.2020.109140"
"   Fan JJ, 2020, J HAZARD MATER, V388, DOI 10.1016/j.jhazmat.2020.122037"
"   Gao JT, 2020, BIOCHEM ENG J, V160, DOI 10.1016/j.bej.2020.107625"
"   Gnanasundar V.M., MATER TODAY-PROC"
"   Han H, 2020, ECOTOX ENVIRON SAFE, V195, DOI 10.1016/j.ecoenv.2020.110375"
"   Huang JQ, 2020, ARCH MICROBIOL, V202, P843, DOI 10.1007/s00203-019-01787-w"
"   Kumar S, 2019, ENVIRON RES, V179, DOI 10.1016/j.envres.2019.108792"
"   Lahori AH, 2017, PEDOSPHERE, V27, P991, DOI 10.1016/S1002-0160(17)60490-9"
"   Li Y, 2019, J CLEAN PROD, V212, P81, DOI 10.1016/j.jclepro.2018.11.294"
"   Liu LW, 2018, SCI TOTAL ENVIRON, V633, P206, DOI 10.1016/j.scitotenv.2018.03.161"
"   Liu SJ, 2018, CHEMOSPHERE, V208, P655, DOI 10.1016/j.chemosphere.2018.06.023"
"   Liu XY, 2020, SCI TOTAL ENVIRON, V708, DOI 10.1016/j.scitotenv.2019.134479"
"   Luna JM, 2016, PROCESS SAF ENVIRON, V102, P558, DOI 10.1016/j.psep.2016.05.010"
"   Ma YS, 2018, CHEM ENG J, V353, P410, DOI 10.1016/j.cej.2018.07.131"
"   Masud MM, 2020, ECOTOX ENVIRON SAFE, V202, DOI 10.1016/j.ecoenv.2020.110865"
"   O'Connor D, 2018, SCI TOTAL ENVIRON, V619, P815, DOI 10.1016/j.scitotenv.2017.11.132"
"   Otunola BO, 2020, ENVIRON TECHNOL INNO, V18, DOI 10.1016/j.eti.2020.100692"
"   Paul T, 2019, PEDOSPHERE, V29, P554, DOI 10.1016/S1002-0160(19)60829-5"
"   Peng WH, 2018, CHEMOSPHERE, V197, P33, DOI 10.1016/j.chemosphere.2018.01.017"
"   Sun RG, 2020, CHEMOSPHERE, V254, DOI 10.1016/j.chemosphere.2020.126828"
"   TESSIER A, 1979, ANAL CHEM, V51, P844, DOI 10.1021/ac50043a017"
"   Wang GY, 2020, ENVIRON RES, V186, DOI 10.1016/j.envres.2020.109554"
"   Wang L, 2019, ENVIRON POLLUT, V245, P820, DOI 10.1016/j.envpol.2018.11.062"
"   [?????? Yan Xiao], 2019, [?????????????€????, Microbiology China], V46, P1907"
"   Yin HB, 2016, CHEM ENG J, V285, P112, DOI 10.1016/j.cej.2015.09.108"
"   Zeraatkar AK, 2016, J ENVIRON MANAGE, V181, P817, DOI 10.1016/j.jenvman.2016.06.059"
"   Zhai XQ, 2018, SCI TOTAL ENVIRON, V635, P92, DOI 10.1016/j.scitotenv.2018.04.119"
"   Zhang C, 2018, ENVIRON POLLUT, V242, P1768, DOI 10.1016/j.envpol.2018.07.091"
"   Zhang JR, 2018, ENVIRON POLLUT, V235, P710, DOI 10.1016/j.envpol.2017.12.106"
NR 33
TC 10
Z9 10
U1 20
U2 145
PU HARD
PI OLSZTYN 5
"PA POST-OFFICE BOX, 10-718 OLSZTYN 5, POLAND"
SN 1230-1485
EI 2083-5906
J9 POL J ENVIRON STUD
JI Pol. J. Environ. Stud.
PY 2022
VL 31
IS 2
BP 1747
EP 1755
DI 10.15244/pjoes/141326
PG 9
WC Environmental Sciences
WE Science Citation Index Expanded (SCI-EXPANDED)
SC Environmental Sciences & Ecology
GA 0E2LJ
UT WOS:000776515100025
OA gold
DA 2026-04-06
ER

PT J
"AU Jalal, A"
"   Zhu, DC"
"AF Jalal, Abdul"
"   Zhu, Daochen"
TI A hypothetical model of phytoremediation for bioremediation of heavy
metals toxicity in agricultural system

SO PLANT GROWTH REGULATION
LA English
DT Article
DE Heavy metals; Sources; Phytoremediation; Agricultural system;
Hypothetical model
"AB Heavy metals contamination in agricultural soils has become a significant concern due to its detrimental effects on crop growth, productivity and food safety. Understanding the sources of heavy metals and implementing effective remediation strategies is crucial for ensuring sustainable agricultural productivity. In this hypothetical model, we aim to explore the intricate dynamics of heavy metals contamination in agricultural systems, identify potential sources, and propose innovative remediation approach through phytoremediation. This model suggests that heavy metals in agricultural systems can originate from various pathways including, natural sources, anthropogenic activities, and atmospheric deposition. Once the heavy metals enter to agricultural soils, they can be taken up by plants either through root uptake which are then translocate to various plant tissues or via foliar absorption through direct contact with contaminated air or irrigation water. This high amount of heavy metals uptake mainly interfere with the uptake and assimilation of essential nutrients, leading to nutrient deficiencies in plants, and the excessive accumulation of heavy metals in plant tissues induce toxicity and oxidative stress, resulting in reduced photosynthetic efficiency and impaired plant growth. To mitigate these adverse effects of heavy metals contamination, a phytoremediation strategies can be implemented to remove heavy metal contaminants from the soil. Specific plant species, based on the climatic zone, that have the ability to accumulate and tolerate heavy metals, could either be implanted on irrigation channels or grown as covers/farm forestry around agricultural fields. This could prevent field crops from toxic heavy metals and ensure the long-term agricultural productivity and safety of our agricultural lands."
"   The model showcases the capability of phytoremediation to address environmental pollution by utilizing particular plant species to reduce harmful contaminants in soil, water, and air, promoting sustainable agriculture."
"C1 [Jalal, Abdul; Zhu, Daochen] Jiangsu Univ, Biofuels Inst, Sch Emergency Management, Sch Environm & Safety Engn, Zhenjiang 212013, Peoples R China."
"   [Jalal, Abdul; Zhu, Daochen] Suzhou Univ Sci & Technol, Jiangsu Collaborat Innovat Ctr Water Treatment, Suzhou 215009, Peoples R China."
C3 Jiangsu University; Suzhou University of Science & Technology
"RP Zhu, DC (corresponding author), Jiangsu Univ, Biofuels Inst, Sch Emergency Management, Sch Environm & Safety Engn, Zhenjiang 212013, Peoples R China.; Zhu, DC (corresponding author), Suzhou Univ Sci & Technol, Jiangsu Collaborat Innovat Ctr Water Treatment, Suzhou 215009, Peoples R China."
EM agriculturist.201@gmail.com; dczhucn@ujs.edu.cn
"RI Jalal, Abdul/GZK-5844-2022; Zhu, Daochen/HPG-3968-2023"
"OI Jalal, Abdul/0000-0002-8579-9792; Zhu, Daochen/0000-0001-9646-9556"
FU Key Research and Development Program of Jiangsu Province [BE2021691];
Innovation Program for Carbon Neutralization of Jiangsu Province
[BK20220003]; Jiangsu Collaborative Innovation Center of Technology and
"   Material of Water Treatment, Suzhou University of Science and"
"   Technology, Suzhou, China [215009]"
FX This work was supported by the Key Research and Development Program of
"   Jiangsu Province (Grant No. BE2021691), the Innovation Program for"
"   Carbon Neutralization of Jiangsu Province (BK20220003), and the and the"
Jiangsu Collaborative Innovation Center of Technology and Material of
"   Water Treatment, Suzhou University of Science and Technology, Suzhou"
"   215009, China."
"CR Alengebawy A, 2021, TOXICS, V9, DOI 10.3390/toxics9030042"
"   Ali H, 2019, J CHEM-NY, V2019, DOI 10.1155/2019/6730305"
"   Ashraf S, 2019, ECOTOX ENVIRON SAFE, V174, P714, DOI 10.1016/j.ecoenv.2019.02.068"
"   Bhat SA, 2022, CHEMOSPHERE, V303, DOI 10.1016/j.chemosphere.2022.134788"
"   Bian FY, 2020, CHEMOSPHERE, V246, DOI 10.1016/j.chemosphere.2019.125750"
"   Boi ME, 2023, PLANTS-BASEL, V12, DOI 10.3390/plants12223823"
"   Briffa J, 2020, HELIYON, V6, DOI 10.1016/j.heliyon.2020.e04691"
"   Chen XF, 2023, PLANT PHYSIOL BIOCH, V200, DOI 10.1016/j.plaphy.2023.107802"
"   Daghan H, 2019, TRANSGENIC PLANT TECHNOLOGY FOR REMEDIATION OF TOXIC METALS AND METALLOIDS, P279, DOI 10.1016/B978-0-12-814389-6.00013-4"
"   Paes ED, 2023, J HAZARD MATER, V449, DOI 10.1016/j.jhazmat.2023.131034"
"   Eid EM, 2020, ENVIRON SCI POLLUT R, V27, P12138, DOI 10.1007/s11356-020-07839-9"
"   Ezemonye LI, 2019, TOXICOL REP, V6, P1, DOI 10.1016/j.toxrep.2018.11.010"
"   Gavrilescu M, 2022, CURR OPIN BIOTECH, V74, DOI 10.1016/j.copbio.2021.10.024"
"   Sierra BGE, 2021, SUSTAINABILITY-BASEL, V13, DOI 10.3390/su13052574"
"   He MY, 2023, SPECTROCHIM ACTA B, V209, DOI 10.1016/j.sab.2023.106781"
"   Huang H, 2023, IEEE T GEOSCI REMOTE, V61, DOI 10.1109/TGRS.2023.3259742"
"   Jogawat A, 2021, PHYSIOL PLANTARUM, V173, P259, DOI 10.1111/ppl.13370"
"   Khan AHA, 2021, ENVIRON RES, V195, DOI 10.1016/j.envres.2021.110780"
"   Khan IU, 2023, PLANTS-BASEL, V12, DOI 10.3390/plants12040725"
"   Kharazi A, 2021, J FOOD COMPOS ANAL, V100, DOI 10.1016/j.jfca.2021.103890"
"   Liu J, 2018, ENVIRON REV, V26, P43, DOI 10.1139/er-2017-0022"
"   Mahajan P, 2018, J TOXICOL-US, V2018, DOI 10.1155/2018/4864365"
"   Manoj SR, 2020, J ENVIRON MANAGE, V254, DOI 10.1016/j.jenvman.2019.109779"
"   Mitra S, 2022, J KING SAUD UNIV SCI, V34, DOI 10.1016/j.jksus.2022.101865"
"   Naz M, 2022, J ENVIRON MANAGE, V323, DOI 10.1016/j.jenvman.2022.116296"
"   Nyiramigisha P, 2021, Reviews in Agricultural Science, V9, P271, DOI [10.7831/ras.9.0_271, DOI 10.7831/RAS.9.0271, 10.7831/ras.9.0271, 10.7831/ras.9.0_271]"
"   Odoemelam SA, 2008, AFR J BIOTECHNOL, V7, P4301"
"   PhD research Scholar Department of General Engineering ICT Mumbai, 2023, INDIAN J SCI TECHNOL, V16, P1787, DOI 10.17485/ijst/v16i24.800"
"   Rai PK, 2019, ENVIRON INT, V125, P365, DOI 10.1016/j.envint.2019.01.067"
"   Rashid A, 2023, AGRONOMY-BASEL, V13, DOI 10.3390/agronomy13061521"
"   Roozbahani M, 2015, IRAN J TOXICOL, V8"
"   Shen X, 2022, CHEMOSPHERE, V291, DOI 10.1016/j.chemosphere.2021.132979"
"   Shi L, 2023, J HAZARD MATER, V441, DOI 10.1016/j.jhazmat.2022.129904"

"   Shumaker Ketia L, 2005, Int J Environ Res Public Health, V2, P293, DOI 10.3390/ijerph2005020013"
"   Srivastava V, 2017, FRONT ENV SCI-SWITZ, V5, DOI 10.3389/fenvs.2017.00064"
"   Sun JF, 2023, ENVIRON EXP BOT, V215, DOI 10.1016/j.envexpbot.2023.105492"
"   Sutkowska K, 2020, MINERALS-BASEL, V10, DOI 10.3390/min10121140"
"   Tan HW, 2023, ENVIRON TECHNOL INNO, V30, DOI 10.1016/j.eti.2023.103043"
"   Wang XY, 2022, NAT ECOL EVOL, V6, P890, DOI 10.1038/s41559-022-01774-3"
"   Wei SH, 2008, J HAZARD MATER, V150, P662, DOI 10.1016/j.jhazmat.2007.05.014"
"   Wen ZD, 2024, INT SOIL WATER CONSE, V12, P200, DOI 10.1016/j.iswcr.2023.07.002"
"   Yan A, 2020, FRONT PLANT SCI, V11, DOI 10.3389/fpls.2020.00359"
"   Yang L, 2022, ECOTOX ENVIRON SAFE, V234, DOI 10.1016/j.ecoenv.2022.113403"
NR 43
TC 1
Z9 1
U1 3
U2 25
PU SPRINGER
PI DORDRECHT
"PA VAN GODEWIJCKSTRAAT 30, 3311 GZ DORDRECHT, NETHERLANDS"
SN 0167-6903
EI 1573-5087
J9 PLANT GROWTH REGUL
JI Plant Growth Regul.
PD SEP
PY 2024
VL 104
IS 1
SI SI
BP 81
EP 87
DI 10.1007/s10725-024-01171-9
EA JUN 2024
PG 7
WC Plant Sciences
WE Science Citation Index Expanded (SCI-EXPANDED)
SC Plant Sciences
GA H8T9B
UT WOS:001254175200001
DA 2026-04-06
ER

PT J
"AU Wang, T"
"   Sun, HW"
"   Ren, XH"
"   Li, B"
"   Mao, HJ"
"AF Wang, Ting"
"   Sun, Hongwen"
"   Ren, Xinhao"
"   Li, Bing"
"   Mao, Hongjun"
TI Evaluation of biochars from different stock materials as carriers of
   bacterial strain for remediation of heavy metal-contaminated soil
SO SCIENTIFIC REPORTS
LA English
DT Article
ID SEQUENTIAL EXTRACTION PROCEDURE; IMMOBILIZATION; IMPACT; LEAD;
   BIOAUGMENTATION; RESTORATION; BIOSORPTION; SORPTION; BIOMASS; CARBON
"AB Two kinds of biochars, one derived from corn straw and one from pig manure, were studied as carriers of a mutant genotype from Bacillus subtilis (B38) for heavy metal contaminated soil remediation. After amendment with biochar, the heavy metal bioavailability decreased. Moreover, the heavy metal immobilization ability of the biochar was enhanced by combining it with B38. The simultaneous application of B38 and pig manure-derived biochar exhibited a superior effect on the promotion of plant growth and the immobilization of heavy metals in soil. The plant biomass increased by 37.9% and heavy metal concentrations in the edible part of lettuce decreased by 69.9-96.1%. The polymerase chain reaction-denaturing gradient gel electrophoresis (PCR-DGGE) profiles revealed that pig manure-derived biochar could enhance the proliferation of both exotic B38 and native microbes. These results suggest that B38 carried by pig manure-derived biochar may be a promising candidate for the remediation of soils contaminated by multiple heavy metals."
"C1 [Wang, Ting; Sun, Hongwen; Ren, Xinhao; Li, Bing] Nankai Univ, Key Lab Pollut Proc & Environm Criteria, Minist Educ Peoples Republ China, Coll Environm Sci & Engn, Tianjin 300071, Peoples R China."
"   [Wang, Ting; Mao, Hongjun] Nankai Univ, Coll Environm Sci & Engn, Ctr Urban Transport Emiss Res, Tianjin 300071, Peoples R China."
"   [Ren, Xinhao] Shaanxi Univ Sci & Technol, Sch Environm Sci & Engn, Xian 710021, Peoples R China."
C3 Nankai University; Nankai University; Shaanxi University of Science &
   Technology
"RP Sun, HW (corresponding author), Nankai Univ, Key Lab Pollut Proc & Environm Criteria, Minist Educ Peoples Republ China, Coll Environm Sci & Engn, Tianjin 300071, Peoples R China."
EM sunhongwen@nankai.edu.cn
"RI Wang, Ting/AGT-9455-2022"
"OI Wang, Ting/0000-0001-6532-1110"
"FU Natural Science Foundation of Tianjin City [15JCQNJC15200,"
   16YFZCSF00410]
FX This work was financially supported by Natural Science Foundation of
   Tianjin City (Grants No. 15JCQNJC15200 and 16YFZCSF00410).

"CR Akcil A, 2004, MINER ENG, V17, P477, DOI 10.1016/j.mineng.2003.10.016"
"   Bedada W, 2016, AGR ECOSYST ENVIRON, V218, P220, DOI 10.1016/j.agee.2015.11.024"
"   Bei W., 1997, J ENV SCI CHINA, V2, P47"
"   Bian RJ, 2014, J HAZARD MATER, V272, P121, DOI 10.1016/j.jhazmat.2014.03.017"
"   Brewer CE, 2011, BIOENERG RES, V4, P312, DOI 10.1007/s12155-011-9133-7"
"   Brierley JA, 2008, HYDROMETALLURGY, V94, P2, DOI 10.1016/j.hydromet.2008.05.014"
"   Cao XD, 2011, ENVIRON SCI TECHNOL, V45, P4884, DOI 10.1021/es103752u"
"   Cao XD, 2009, ENVIRON SCI TECHNOL, V43, P3285, DOI 10.1021/es803092k"
"   Cavani L, 2016, J ENVIRON MANAGE, V182, P37, DOI 10.1016/j.jenvman.2016.07.050"
"   Chen YX, 2006, SCI TOTAL ENVIRON, V356, P247, DOI 10.1016/j.scitotenv.2005.04.028"
"   El-Bestawy E, 1998, WORLD J MICROB BIOT, V14, P853, DOI 10.1023/A:1008827830024"
"   Han YX, 2013, J ENVIRON MANAGE, V118, P196, DOI 10.1016/j.jenvman.2013.01.001"
"   Jaiswal AK, 2017, SCI REP-UK, V7, DOI 10.1038/srep44382"
"   Jiang C, 2009, J HAZARD MATER, V167, P1170, DOI 10.1016/j.jhazmat.2009.01.107"
"   Jiang J, 2012, J HAZARD MATER, V229, P145, DOI 10.1016/j.jhazmat.2012.05.086"
"   Juwarkar AA, 2008, ENVIRON MONIT ASSESS, V139, P355, DOI 10.1007/s10661-007-9842-8"
"   Keiluweit M, 2010, ENVIRON SCI TECHNOL, V44, P1247, DOI 10.1021/es9031419"
"   Khan S, 2008, J HAZARD MATER, V152, P506, DOI 10.1016/j.jhazmat.2007.07.014"
"   Ledin M, 1999, SOIL BIOL BIOCHEM, V31, P1639, DOI 10.1016/S0038-0717(99)00073-5"
"   Lehmann J., 2006, Mitigation and Adaptation Strategies for Global Change, V11, P403, DOI 10.1007/s11027-005-9006-5"
"   Lehmann J, 2011, SOIL BIOL BIOCHEM, V43, P1812, DOI 10.1016/j.soilbio.2011.04.022"
"   Lindsay W., 1969, AGRON ABSTR, P1"
"   Lu KP, 2014, AGR ECOSYST ENVIRON, V191, P124, DOI 10.1016/j.agee.2014.04.010"
"   Lucchini P, 2014, AGR ECOSYST ENVIRON, V184, P149, DOI 10.1016/j.agee.2013.11.018"
"   MEHLICH A, 1984, COMMUN SOIL SCI PLAN, V15, P1409, DOI 10.1080/00103628409367568"
"   Moreno-Jim??nez E, 2016, AGR ECOSYST ENVIRON, V219, P171, DOI 10.1016/j.agee.2015.12.001"
"   MUYZER G, 1993, APPL ENVIRON MICROB, V59, P695, DOI 10.1128/AEM.59.3.695-700.1993"
"   Nemati K, 2011, J HAZARD MATER, V192, P402, DOI 10.1016/j.jhazmat.2011.05.039"
"   Pal S., 2010, NATURAL RESOURCES, V1, DOI [10.4236/nr.2010.12012, DOI 10.4236/NR.2010.12012]"
"   PAYAPEREZ A, 1993, INT J ENVIRON AN CH, V51, P223, DOI 10.1080/03067319308027628"
"   Qiu GZ, 2008, T NONFERR METAL SOC, V18, P1295, DOI 10.1016/S1003-6326(09)60001-7"
"   Reddy KR, 2003, ENERG SOURCE, V25, P931, DOI 10.1080/00908310390221318"
"   Roane TM, 2001, APPL ENVIRON MICROB, V67, P3208, DOI 10.1128/AEM.67.7.3208-3215.2001"
"   Rombol?? AG, 2015, J AGR FOOD CHEM, V63, P6660, DOI 10.1021/acs.jafc.5b01540"
"   Sager M, 2007, WATER AIR SOIL POLL, V179, P265, DOI 10.1007/s11270-006-9230-y"
"   Sarkhot DV, 2012, J ENVIRON QUAL, V41, P1107, DOI 10.2134/jeq2011.0123"
"   Spokas KA, 2011, CHEMOSPHERE, V85, P869, DOI 10.1016/j.chemosphere.2011.06.108"
"   Trigo C, 2014, J AGR FOOD CHEM, V62, P10855, DOI 10.1021/jf5034398"
"   Uchimiya M, 2012, J AGR FOOD CHEM, V60, P5035, DOI 10.1021/jf300825n"
"   Uchimiya M, 2010, J AGR FOOD CHEM, V58, P5538, DOI 10.1021/jf9044217"
"   Udeigwe TK, 2011, ENVIRON INT, V37, P258, DOI 10.1016/j.envint.2010.08.008"
"   Valls M, 2002, FEMS MICROBIOL REV, V26, P327, DOI 10.1111/j.1574-6976.2002.tb00618.x"
"   Wang T, 2014, J HAZARD MATER, V278, P483, DOI 10.1016/j.jhazmat.2014.06.028"
"   Wang T, 2013, ENVIRON SCI POLLUT R, V20, P7450, DOI 10.1007/s11356-013-1767-x"
"   WHALLEY C, 1994, ANAL CHIM ACTA, V291, P287, DOI 10.1016/0003-2670(94)80024-3"
"   Xu XY, 2016, J COLLOID INTERF SCI, V463, P55, DOI 10.1016/j.jcis.2015.10.003"
"   Yang YN, 2007, NUCL INSTRUM METH B, V262, P220, DOI 10.1016/j.nimb.2007.05.016"
"   Yin H, 2008, WATER RES, V42, P3981, DOI 10.1016/j.watres.2008.07.005"
NR 48
TC 61
Z9 71
U1 11
U2 179
PU NATURE PORTFOLIO
PI BERLIN
"PA HEIDELBERGER PLATZ 3, BERLIN, 14197, GERMANY"
SN 2045-2322
J9 SCI REP-UK
JI Sci Rep
PD SEP 21
PY 2017
VL 7
AR 12114
DI 10.1038/s41598-017-12503-3
PG 10
WC Multidisciplinary Sciences
WE Science Citation Index Expanded (SCI-EXPANDED)
SC Science & Technology - Other Topics
GA FH8BL
UT WOS:000411416700017
PM 28935871
"OA Green Submitted, gold"
DA 2026-04-06
ER

PT J
"AU Ni, ZJ"
"   Zhang, XR"
"   Gong, X"
"   Gong, ZQ"
"   Song, L"
"   Xu, PF"
"   Zhang, ZM"

"   Guo, SH"
"AF Ni, Zijun"
"   Zhang, Xiaorong"
"   Gong, Xuan"
"   Gong, Zongqiang"
"   Song, Lei"
"   Xu, Peifeng"
"   Zhang, Zhimin"
"   Guo, Shuhai"
TI Enhanced remediation of co-contaminated agricultural soils under cold
   stress by immobilized bacterial agents: A perspective based on abundance
   differences
SO JOURNAL OF HAZARDOUS MATERIALS
LA English
DT Article
DE Community assembly; Immobilized bioagent; Winter bioremediation;
   Antibiotic-heavy metal co-contaminated soil; Antibiotic resistance
   genes; Community assembly; Immobilized bioagent; Winter bioremediation;
   Antibiotic-heavy metal co-contaminated soil; Antibiotic resistance genes
ID HEAVY-METALS; SP NOV.; BIOCHAR; MECHANISMS; BIODEGRADATION;
   TETRACYCLINE; POLLUTION
"AB This study explored the enhanced remediation effects and microbial mechanisms of the immobilized microbial agent B&Ma19, compared to the microbial agent Ma19, in cold-region farmland co-contaminated with antibiotics and heavy metals during winter. B&Ma19 achieved superior fluoroquinolone degradation and significantly reduced the bioavailability of copper and zinc, while Ma19 treatment only reduced the content of available zinc. Microbial community analysis revealed that B&Ma19 promoted the proliferation of Firmicutes and increased the relative abundance of rare taxa during the freeze-thaw and freezing phases. Functional predictions indicated that B&Ma19 enhanced the expression of proteins in the winter microbial community involved in resistance to antibiotics, metabolic activity, and nutrient acquisition capacity. A random forest model identified Sporosarcina as a potential key genus for co-contamination remediation. Moreover, increased in overall community dispersal limitation and reduced drift-driven succession were observed. The co-occurrence network became more stable, characterized by a higher proportion of moderately abundant keystone taxa. Mantel tests showed that B&Mal 9 weakened the correlation between antibiotic resistance genes (ARGs) and mobile genetic elements, and reduced the impact of temperature fluctuations on contaminant concentrations. In contrast, Ma19 strengthened ARGantibiotic associations. These findings provide a theoretical basis for bioremediation of co-contaminated farmland in cold regions."
"C1 [Ni, Zijun; Zhang, Xiaorong; Gong, Zongqiang; Song, Lei; Xu, Peifeng; Zhang, Zhimin] Chinese Acad Sci, Inst Appl Ecol, Shenyang 110016, Peoples R China."
"   [Zhang, Xiaorong; Gong, Zongqiang; Guo, Shuhai] Natl Local Joint Engn Lab Contaminated Soil Remedi, Shenyang 110016, Peoples R China."
"   [Ni, Zijun; Song, Lei; Xu, Peifeng; Zhang, Zhimin] Univ Chinese Acad Sci, Beijing 100049, Peoples R China."
"   [Gong, Xuan] Northeastern Univ, Sch Resources & Civil Engn, Shenyang 110819, Peoples R China."
"C3 Chinese Academy of Sciences; Shenyang Institute of Applied Ecology, CAS;"
"   Chinese Academy of Sciences; University of Chinese Academy of Sciences,"
   CAS; Northeastern University - China
"RP Gong, ZQ (corresponding author), Chinese Acad Sci, Inst Appl Ecol, Shenyang 110016, Peoples R China."
EM zgong@iae.ac.cn
"OI Gong, Zongqiang/0000-0002-5646-6227"
FU Shenyang Science and Technology Program of China [23-409-2-01]; National
   Key Research and Development Program of China [2019YFD1100504]; Shenyang
   Science and Technology Program of China; National Key Research and
   Development Program of China
FX This study was financially supported by Shenyang Science and Technology
   Program of China (23-409-2-01) and the National Key Research and
   Development Program of China (2019YFD1100504) . Au-thors thank the
   researchers for their valuable suggestions on the manuscript.
"CR Afzal A, 2024, FRONT PHARMACOL, V15, DOI 10.3389/fphar.2024.1375137"
"   Al Masud MA, 2024, SCI TOTAL ENVIRON, V926, DOI 10.1016/j.scitotenv.2024.171944"
"   Azizi-Rad M, 2022, SOIL BIOL BIOCHEM, V165, DOI 10.1016/j.soilbio.2021.108488"
"   Bagwell CE, 2010, PLOS ONE, V5, DOI 10.1371/journal.pone.0012427"
"   Beaman TW, 2002, PROTEIN SCI, V11, P974, DOI 10.1110/ps.4310102"
"   Brin LD, 2018, AGR ECOSYST ENVIRON, V258, P91, DOI 10.1016/j.agee.2018.01.033"
"   Callahan BJ, 2016, NAT METHODS, V13, P581, DOI [10.1038/NMETH.3869, 10.1038/nmeth.3869]"
"   Caporaso JG, 2011, P NATL ACAD SCI USA, V108, P4516, DOI 10.1073/pnas.1000080107"
"   Deshoux M, 2023, SCI TOTAL ENVIRON, V902, DOI 10.1016/j.scitotenv.2023.166079"
"   Dixon P, 2003, J VEG SCI, V14, P927, DOI 10.1111/j.1654-1103.2003.tb02228.x"
"   Douglas GM, 2020, NAT BIOTECHNOL, V38, P685, DOI 10.1038/s41587-020-0548-6"
"   Du Y, 2012, INT J SYST EVOL MICR, V62, P2356, DOI 10.1099/ijs.0.034306-0"
"   Edet UO, 2023, HELIYON, V9, DOI 10.1016/j.heliyon.2023.e13457"
"   Ellabaan MMH, 2021, NAT COMMUN, V12, DOI 10.1038/s41467-021-22757-1"
"   Fajardo C, 2019, APPL SOIL ECOL, V135, P56, DOI 10.1016/j.apsoil.2018.10.022"
"   Fang J, 2022, J HAZARD MATER, V423, DOI 10.1016/j.jhazmat.2021.127150"
"   Fareed A, 2019, HELIYON, V5, DOI 10.1016/j.heliyon.2019.e02740"
"   Fei JC, 2024, WATER RES, V267, DOI 10.1016/j.watres.2024.122506"
"   Fiaz A, 2021, ENVIRON SCI EUR, V33, DOI 10.1186/s12302-021-00505-y"
"   Filippidou S, 2016, FRONT MICROBIOL, V7, DOI 10.3389/fmicb.2016.01707"
"   Fu YH, 2021, ENVIRON SCI TECHNOL, V55, P16369, DOI 10.1021/acs.est.1c03434"
"   Gabrielaite M, 2021, J CLIN MICROBIOL, V59, DOI 10.1128/JCM.02911-20"
"   Ghimire N, 2020, CURR MICROBIOL, V77, P2940, DOI 10.1007/s00284-020-02120-1"
"   Gorka S, 2023, GEODERMA, V432, DOI 10.1016/j.geoderma.2023.116399"
"   Griffiths RI, 2016, APPL SOIL ECOL, V97, P61, DOI 10.1016/j.apsoil.2015.06.018"
"   Guo LL, 2020, AMB EXPRESS, V10, DOI 10.1186/s13568-019-0942-y"
"   Guo Y, 2014, J ENVIRON SCI, V26, P2123, DOI 10.1016/j.jes.2014.08.012"

"   Hamidjaja R, 2020, ISME J, V14, P2890, DOI 10.1038/s41396-020-00760-6"
"   He Y, 2020, NPJ CLEAN WATER, V3, DOI 10.1038/s41545-020-0051-0"
"   He ZB, 2022, SCI TOTAL ENVIRON, V828, DOI 10.1016/j.scitotenv.2022.154364"
"   Hejna M, 2018, ANIMAL, V12, P2156, DOI 10.1017/S175173111700355X"
"   Hou RJ, 2022, SCI TOTAL ENVIRON, V831, DOI 10.1016/j.scitotenv.2022.154880"
"   Jin YY, 2018, APPL SCI-BASEL, V8, DOI 10.3390/app8081336"
"   Kaale SE, 2024, MICROBIOL RES, V278, DOI 10.1016/j.micres.2023.127543"
"   Kikukawa T, 2006, BBA-BIOMEMBRANES, V1758, P673, DOI 10.1016/j.bbamem.2006.04.004"
"   Kim J, 2018, SCI TOTAL ENVIRON, V612, P903, DOI 10.1016/j.scitotenv.2017.08.227"
"   Kim SH, 2023, APPL BIOL CHEM, V66, DOI 10.1186/s13765-023-00822-1"
"   Kr??l A, 2020, J HAZARD MATER, V384, DOI 10.1016/j.jhazmat.2019.121502"
"   Kuppusamy S, 2018, AGR ECOSYST ENVIRON, V257, P47, DOI 10.1016/j.agee.2018.01.026"
"   Lan JR, 2021, J HAZARD MATER, V420, DOI 10.1016/j.jhazmat.2021.126588"
"   Langfelder P, 2008, BMC BIOINFORMATICS, V9, DOI 10.1186/1471-2105-9-559"
"   Lee H, 2017, ANTON LEEUW INT J G, V110, P751, DOI 10.1007/s10482-017-0845-3"
"   Letunic I, 2007, BIOINFORMATICS, V23, P127, DOI 10.1093/bioinformatics/btl529"
"   Leuther F, 2021, SOIL-GERMANY, V7, P179, DOI 10.5194/soil-7-179-2021"
"   Li CT, 2022, J HAZARD MATER, V429, DOI 10.1016/j.jhazmat.2022.128353"
"   Li JX, 2024, RSC ADV, V14, P12720, DOI 10.1039/d4ra01510j"
"   [?????? Li Ke], 2021, [????€???????, Acta Ecologica Sinica], V41, P4827, DOI 10.5846/stxb202001190150"
"   Li LM, 2021, GEOCHEM-EXPLOR ENV A, V21, DOI 10.1144/geochem2021-029"
"   Liu CW, 2024, SCI REP-UK, V14, DOI 10.1038/s41598-024-72771-8"
"   Liu HG, 2023, BIORESOURCE TECHNOL, V378, DOI 10.1016/j.biortech.2023.128987"
"   Liu L, 2023, APPL ENVIRON MICROB, V89, DOI 10.1128/aem.01072-23"
"   Liu NN, 2021, MICROORGANISMS, V9, DOI 10.3390/microorganisms9091929"
"   Liu SH, 2017, BIORESOURCE TECHNOL, V224, P25, DOI 10.1016/j.biortech.2016.11.095"
"   Liu XY, 2025, SCI REP-UK, V15, DOI 10.1038/s41598-025-91771-w"
"   Ma YC, 2023, MOLECULES, V28, DOI 10.3390/molecules28217433"
"   Mackelprang R, 2022, MSYSTEMS, V7, DOI 10.1128/msystems.01419-21"
"   Martz F, 2016, PLOS ONE, V11, DOI 10.1371/journal.pone.0156620"
"   Mazhar SH, 2021, SCI TOTAL ENVIRON, V755, DOI 10.1016/j.scitotenv.2020.142702"
"   Muurinen J, 2021, SCI REP-UK, V11, DOI 10.1038/s41598-021-84759-9"
"   Najim AA, 2024, J CHEM TECHNOL BIOT, V99, P1707, DOI 10.1002/jctb.7638"
"   Nedwell DB, 1999, FEMS MICROBIOL ECOL, V30, P101"
"   Ni ZJ, 2024, TOXICS, V12, DOI 10.3390/toxics12090667"
"   Ning DL, 2020, NAT COMMUN, V11, DOI 10.1038/s41467-020-18560-z"
"   Ojuederie OB, 2017, INT J ENV RES PUB HE, V14, DOI 10.3390/ijerph14121504"
"   Parks DH, 2014, BIOINFORMATICS, V30, P3123, DOI 10.1093/bioinformatics/btu494"
"   Pascoal F, 2021, FEMS MICROBIOL ECOL, V97, DOI 10.1093/femsec/fiaa227"
"   Rao MPN, 2022, ENVIRON RES, V209, DOI 10.1016/j.envres.2022.112888"
"   Rooney EC, 2022, J GEOPHYS RES-BIOGEO, V127, DOI 10.1029/2022JG006889"
"   Sang CP, 2021, ECOL PROCESS, V10, DOI 10.1186/s13717-021-00337-x"
"   Sen U, 2023, APPL SCI-BASEL, V13, DOI 10.3390/app132111963"
"   Sezmis AL, 2023, MOL BIOL EVOL, V40, DOI 10.1093/molbev/msad028"
"   Shi GX, 2024, ENVIRON POLLUT, V362, DOI 10.1016/j.envpol.2024.125003"
"   Shi GX, 2024, J HAZARD MATER, V471, DOI 10.1016/j.jhazmat.2024.134397"
"   Shu YX, 2025, GREEN ENERGY ENVIRON, V10, P869, DOI [10.1016/j.gee.2024.07.007,
10.1016/j.gee.2024.07.007]"
"   Song JX, 2017, ENVIRON SCI TECHNOL, V51, P3040, DOI 10.1021/acs.est.6b05342"
"   Su D, 2024, SCI TOTAL ENVIRON, V939, DOI 10.1016/j.scitotenv.2024.173414"
"   Su HF, 2022, J CLEAN PROD, V373, DOI 10.1016/j.jclepro.2022.133825"
"   Su Y, 2022, SCI REP-UK, V12, DOI 10.1038/s41598-022-07285-2"
"   Sun SJ, 2022, ENVIRON POLLUT, V293, DOI 10.1016/j.envpol.2021.118524"
"   Thou J., 2017, Microbiol Mol Biol Rev"
"   Totubaeva N, 2023, PLANT SOIL ENVIRON, V69, P188, DOI 10.17221/433/2022-PSE"
"   Viana AT, 2018, ENVIRON POLLUT, V241, P1048, DOI 10.1016/j.envpol.2018.06.047"
"   Wang C, 2023, SCI TOTAL ENVIRON, V867, DOI 10.1016/j.scitotenv.2023.161457"
"   Wang X, 2017, WATER AIR SOIL POLL, V228, DOI 10.1007/s11270-017-3376-7"
"   Wang YC, 2024, BIORESOURCE TECHNOL, V402, DOI 10.1016/j.biortech.2024.130838"
"   Wang YL, 2023, FRONT MICROBIOL, V14, DOI 10.3389/fmicb.2023.1141545"
"   Wang ZH, 2024, NAT COMMUN, V15, DOI 10.1038/s41467-024-54417-5"
"   Warnock DD, 2007, PLANT SOIL, V300, P9, DOI 10.1007/s11104-007-9391-5"
"   Wen T, 2022, IMETA, V1, DOI 10.1002/imt2.32"
"   Wilkes RA, 2023, NAT CHEM BIOL, V19, P651, DOI 10.1038/s41589-022-01237-7"
"   Wu T, 2023, ENVIRON RES, V224, DOI 10.1016/j.envres.2023.115513"
"   Wu YF, 2021, ACS EARTH SPACE CHEM, V5, P2115, DOI 10.1021/acsearthspacechem.1c00155"
"   Wu YC, 2018, FRONT MICROBIOL, V9, DOI 10.3389/fmicb.2018.03096"
"   Xiao LW, 2014, ENVIRON EARTH SCI, V71, P5195, DOI 10.1007/s12665-013-2922-x"
"   Xue P, 2023, J ENVIRON CHEM ENG, V11, DOI 10.1016/j.jece.2023.110928"
"   Xun WB, 2021, MICROBIOME, V9, DOI 10.1186/s40168-020-00985-9"
"   Yan H, 2025, AGRONOMY-BASEL, V15, DOI 10.3390/agronomy15010040"
"   Yin HL, 2020, APPL CATAL B-ENVIRON, V269, DOI 10.1016/j.apcatb.2020.118829"
"   Zhang C, 2024, NAT COMMUN, V15, DOI 10.1038/s41467-024-50342-9"
"   Zhang GX, 2018, GEODERMA, V328, P100, DOI 10.1016/j.geoderma.2018.05.009"
"   Zhang GX, 2013, J SOIL SEDIMENT, V13, P1600, DOI 10.1007/s11368-013-0755-6"
"   Zhang L, 2015, BMC GENOMICS, V16, DOI 10.1186/s12864-015-2326-2"
"   Zhang XR, 2022, J ENVIRON MANAGE, V317, DOI 10.1016/j.jenvman.2022.115320"
"   Zhou HH, 2021, SCI TOTAL ENVIRON, V793, DOI 10.1016/j.scitotenv.2021.148529"
"   Zhou T, 2023, BIOINFORMATICS, V39, DOI 10.1093/bioinformatics/btad339"
"   Zhou XG, 2022, CHEMOSPHERE, V307, DOI 10.1016/j.chemosphere.2022.136138"
"   Zhu XM, 2017, ENVIRON POLLUT, V227, P98, DOI [10.1016/j.envpol.2017.04.032,
10.1016/j.envPol.2017.04.032]"
"   Zhu YF, 2022, FRONT MICROBIOL, V13, DOI 10.3389/fmicb.2022.972300"
NR 108

TC 2
Z9 2
U1 24
U2 45
PU ELSEVIER
PI AMSTERDAM
"PA RADARWEG 29, 1043 NX AMSTERDAM, NETHERLANDS"
SN 0304-3894
EI 1873-3336
J9 J HAZARD MATER
JI J. Hazard. Mater.
PD AUG 15
PY 2025
VL 494
AR 138552
DI 10.1016/j.jhazmat.2025.138552
EA MAY 2025
PG 17
"WC Engineering, Environmental; Environmental Sciences"
WE Science Citation Index Expanded (SCI-EXPANDED)
SC Engineering; Environmental Sciences & Ecology
GA 3MN5O
UT WOS:001503896900020
PM 40373411
DA 2026-04-06
ER

PT J
"AU Kim, SU"
"   Cheong, YH"
"   Seo, DC"
"   Hur, JS"
"   Heo, JS"
"   Cho, JS"
"AF Kim, S. U."
"   Cheong, Y. H."
"   Seo, D. C."
"   Hur, J. S."
"   Heo, J. S."
"   Cho, J. S."
TI Characterisation of heavy metal tolerance and biosorption capacity of
   bacterium strain CPB4 (Bacillus spp.)
SO WATER SCIENCE AND TECHNOLOGY
LA English
DT Article; Proceedings Paper
CT 5th International Conference on Wastewater Reclamation and Reuse for
   Sustainabiltiy
"CY NOV 08-11, 2005"
"CL Cheju Isl, SOUTH KOREA"
DE biosorption; heavy metal; localisation and distribution of heavy metal;
   resistance
ID IONS
"AB A heavy metal resistant bacterium Bacillus spp. strain CPB4 was isolated from heavy metal contaminated soil in Korea and further characterised. The CPB4 strain showed a high capacity for uptake of heavy metal Pb (Pb > Cd > Cu > Ni > Co > Mn > Cr > Zn) both in single and in mixed heavy metal solution. Optimal conditions for heavy metal uptakes of CPB4 strain were 20-40 degrees C culture temperature, 5-7 pH and 24 h pre-culture times. TEM showed that large amounts of the electron-dense granules (heavy metal complexes) were found mainly on the cell wall and cell membrane. Furthermore, more than 90% of adsorbed heavy metals were distributed both in cell wall and in cell membrane fractions. The amount of heavy metal uptake was remarkably decreased by reducing the crude protein contents when cells were treated by alkali solutions. Therefore, this study showed one of the possible examples for useful bioremediation."
"C1 Sunchon Natl Univ, Dept Environm Biol, Sunchon 540742, South Korea."
"   Univ Calif Berkeley, Dept Plant & Microbial Biol, Berkeley, CA 94720 USA."
"   Gyeongsang Natl Univ, Div Appl Life Sci, Jinju 660701, South Korea."
"   Sunchon Natl Univ, Dept Environm Educ, Sunchon 540742, South Korea."
C3 Sunchon National University; University of California System; University
   of California Berkeley; Gyeongsang National University; Sunchon National
   University
"RP Kim, SU (corresponding author), Sunchon Natl Univ, Dept Environm Biol, Sunchon 540742, South Korea."
EM chojs@sunchon.ac.kr
"RI Seo, Dong-Chul/A-6935-2009"
"CR [Anonymous], 1990, Official methods of the analysis of the AOAC, V15th, P237"
"   Arica MY, 2005, PROCESS BIOCHEM, V40, P2351, DOI 10.1016/j.procbio.2004.09.008"
"   BEVERIDGE TJ, 1976, J BACTERIOL, V127, P1502, DOI 10.1128/JB.127.3.1502-1518.1976"
"   Davis TA, 2003, WATER RES, V37, P4311, DOI 10.1016/S0043-1354(03)00293-8"
"   Kadukov?? J, 2005, ENVIRON INT, V31, P227, DOI 10.1016/j.envint.2004.09.020"
"   Malik A, 2004, ENVIRON INT, V30, P261, DOI 10.1016/j.envint.2003.08.001"
"   MULLEN MD, 1989, APPL ENVIRON MICROB, V55, P3143, DOI 10.1128/AEM.55.12.3143-3149.1989"
"   NAKAJIMA A, 1981, EUR J APPL MICROBIOL, V12, P76, DOI 10.1007/BF01970038"
NR 8
TC 54
Z9 59
U1 2
U2 48

PU IWA PUBLISHING
PI LONDON
"PA ALLIANCE HOUSE, 12 CAXTON ST, LONDON SW1H0QS, the United Kingdom"
SN 0273-1223
J9 WATER SCI TECHNOL
JI Water Sci. Technol.
PY 2007
VL 55
IS 1-2
BP 105
EP 111
DI 10.2166/wst.2007.007
PG 7
"WC Engineering, Environmental; Environmental Sciences; Water Resources"
WE Conference Proceedings Citation Index - Science (CPCI-S); Science Citation Index Expanded (SCI-EXPANDED)
SC Engineering; Environmental Sciences & Ecology; Water Resources
GA 158IU
UT WOS:000245786000014
PM 17305129
DA 2026-04-06
ER

PT J
"AU Ge, LQ"
"   Yuan, X"
"   Zhang, LL"
"   Li, H"
"   Liu, XY"
"   Zhu, XH"
"AF Ge, Liqiang"
"   Yuan, Xin"
"   Zhang, Longlong"
"   Li, Hang"
"   Liu, Xiaoyu"
"   Zhu, Xiaohua"
TI Uncovering the Relationship Between Soil Bacterial Community and Heavy
   Metals in a Copper Waste Pile
SO MINERALS
LA English
DT Article
DE copper waste piles; heavy metals; soil bacterial community; heavy metal
   tolerant flora; microbial remediation
ID 16S RIBOSOMAL-RNA; LONG-TERM; MICROBIAL COMMUNITY; CONTAMINATED SOILS;
   MINE TAILINGS; REMEDIATION; PHYTOREMEDIATION; STRATEGIES; SEQUENCES
"AB In the present study, the relationship between the microbial community and heavy metal content of soil was analyzed based on 16S rRNA gene high-throughput sequencing, in order to screen the corresponding heavy metal-resistant bacteria in a copper mine waste dump and adjacent shrubbery. Approximately 22 phyla, 57 classes, 128 orders, 173 families, 263 genera, 433 species, and 954 OUTs obtained from soil sample species annotation indicated the Spearman relevance analysis at the phylum level. Specifically, Gemmatimonadota is positively correlated with arsenic (As); Patescibacteria is positively correlated with arsenic (As), copper (Cu), and cadmium (Cd); Proteobacteria is positively correlated with chromium (Cr); and Acidobacteriota is positively correlated with cadmium (Cd), respectively. Meanwhile, at the genus level, Acidibacter is positively correlated with arsenic (As); norank_f__LWQ8, norank_f__Gemmataceae, and Bryobacter are positively correlated with cadmium (Cd); Acidiphilium and Conexiactor are positively correlated with Zinc (Zn); norank_f__norank_o__IMCC26256 is positively correlated with nickel (Ni); norank_f__norank_o__norank_c__AD3 is positively correlated with manganese (Mn), and nickel (Ni); and Alicyclobacillus and unclassified_f__Acidiferobactereae are positively correlated with chromium (Cr). These bacterial flora are significantly and positively related to the resistance of heavy metals, which provides a promising reference for the development of in situ remediation of heavy metal pollution in mines."
"C1 [Ge, Liqiang; Yuan, Xin; Zhang, Longlong; Li, Hang; Liu, Xiaoyu; Zhu, Xiaohua] Natl Res Ctr Geoanal, Beijing 100037, Peoples R China."
"   [Ge, Liqiang; Yuan, Xin; Zhang, Longlong; Li, Hang; Liu, Xiaoyu; Zhu, Xiaohua] Minist Nat Resources, Key Lab Ecogeochem, Beijing 100037, Peoples R China."
"   [Zhu, Xiaohua] Minist Nat Resources, Land Consolidat & Rehabil Ctr, Beijing 100035, Peoples R China."
C3 Ministry of Natural Resources of the People's Republic of China;
   Ministry of Natural Resources of the People's Republic of China
"RP Yuan, X; Zhu, XH (corresponding author), Natl Res Ctr Geoanal, Beijing 100037, Peoples R China.; Yuan, X; Zhu, XH (corresponding author), Minist Nat Resources, Key Lab Ecogeochem, Beijing 100037, Peoples R China.; Zhu, XH (corresponding author), Minist Nat Resources, Land Consolidat & Rehabil Ctr, Beijing 100035, Peoples R China."
EM geliqiang220@163.com; yuanxin7618@163.com; zhanglonglong88@126.com;
   lihangg2020@163.com; liuxiaoyu0725@163.com; zglzby@163.com
"OI Ge, Liqiang/0000-0002-0754-0064"
FU Basic Research Funds of Chinese Academy of Geological Sciences; National
   Key Technology R&D Program of China [2019YFC1805104]; [CSJ-2021-10]
FX This research was funded by Basic Research Funds of Chinese Academy of
   Geological Sciences (grant No. CSJ-2021-10) and National Key Technology
   R&D Program of China (grant No. 2019YFC1805104).
"CR Abollino O, 2002, CHEMOSPHERE, V49, P545, DOI 10.1016/S0045-6535(02)00352-1"
"   Adriano DC, 2004, GEODERMA, V122, P121, DOI 10.1016/j.geoderma.2004.01.003"
"   [Anonymous], 2018, Ministry of Ecology and Environment of the Peoples Republic of China. Soil Environmental Quality-Risk Control Standard for Soil Contamination of Agricultural Land, P1"
"   [Anonymous], 2018, National Standardization Administration of the Peoples Republic of China. Soil Quality-Guidance on Sampling Techniques, P1"

"   Azarbad H, 2015, FEMS MICROBIOL ECOL, V91, DOI 10.1093/femsec/fiu003"
"   Brady JP, 2014, MAR POLLUT BULL, V81, P248, DOI 10.1016/j.marpolbul.2014.01.031"
"   Cai L, 2009, BMC MICROBIOL, V9, DOI 10.1186/1471-2180-9-4"
"   Chen SP, 2023, RHIZOSPHERE-NETH, V27, DOI 10.1016/j.rhisph.2023.100755"
"   Chen Xinyao, 2017, Huanjing Huaxue-Environmental Chemistry, V36, P356, DOI 10.7524/j.issn.0254-6108.2017.02.2016062401"
"   Chen Y, 2018, SCI TOTAL ENVIRON, V637, P1400, DOI 10.1016/j.scitotenv.2018.05.109"
"   Chinas State Environmental Protection Administration, 1990, Background Values of Soil Elements in China"
"   Crits-Christoph A, 2018, NATURE, V558, P440, DOI 10.1038/s41586-018-0207-y"
"   Dhaliwal SS, 2020, ENVIRON SCI POLLUT R, V27, P1319, DOI 10.1007/s11356-019-06967-1"
"   Edgar RC, 2013, NAT METHODS, V10, P996, DOI [10.1038/NMETH.2604, 10.1038/nmeth.2604]"
"   Feng G, 2018, BMC MICROBIOL, V18, DOI 10.1186/s12866-018-1152-5"
"   Frey B, 2006, SOIL BIOL BIOCHEM, V38, P1745, DOI 10.1016/j.soilbio.2005.11.032"
"   [????????? Gao Zhirui], 2017, [????????????????????, Acta Petrologica et Mineralogica], V36, P785"
"   Giller KE, 2009, SOIL BIOL BIOCHEM, V41, P2031, DOI 10.1016/j.soilbio.2009.04.026"
"   Gong XZ, 2024, MICROBIOME, V12, DOI 10.1186/s40168-024-01871-4"
"   Guo HH, 2017, ECOTOX ENVIRON SAFE, V144, P300, DOI 10.1016/j.ecoenv.2017.06.048"
"   Gupta S., 2017, Role of Genetically Modified Microorganisms in Heavy Metal Bioremediation, P197"
"   Huang YL, 2017, INT BIODETER BIODEGR, V117, P22, DOI 10.1016/j.ibiod.2016.11.010"
"   Islam MS, 2015, SCI TOTAL ENVIRON, V512, P94, DOI 10.1016/j.scitotenv.2014.12.100"
"   Issotta F, 2018, RES MICROBIOL, V169, P608, DOI 10.1016/j.resmic.2018.08.001"
"   Jacquiod S, 2018, J HAZARD MATER, V344, P299, DOI 10.1016/j.jhazmat.2017.09.046"
"   Janssen PH, 2006, APPL ENVIRON MICROB, V72, P1719, DOI 10.1128/AEM.72.3.1719-1728.2006"
"   Kang CH, 2016, ECOL ENG, V89, P64, DOI 10.1016/j.ecoleng.2016.01.023"
"   Katsaveli K, 2012, EXTREMOPHILES, V16, P285, DOI 10.1007/s00792-012-0429-0"
"   Keiblinger KM, 2018, ECOTOXICOLOGY, V27, P217, DOI 10.1007/s10646-017-1888-y"
"   Kepel Billy, 2020, Pak J Biol Sci, V23, P63, DOI [10.3923/pjbs.2020.63.67, 10.3923/pjbs.2020.63.67]"
"   Khan S, 2010, ENVIRON SCI POLLUT R, V17, P288, DOI 10.1007/s11356-009-0134-4"
"   KISHIMOTO N, 1993, CURR MICROBIOL, V27, P131, DOI 10.1007/BF01576009"
"   Kulichevskaya IS, 2010, INT J SYST EVOL MICR, V60, P301, DOI 10.1099/ijs.0.013250-0"
"   Li SQ, 2024, CHEMOECOLOGY, V34, P47, DOI 10.1007/s00049-024-00399-1"
"   Li XQ, 2017, ENVIRON POLLUT, V231, P908, DOI 10.1016/j.envpol.2017.08.057"
"   Limcharoensuk T, 2015, ECOTOX ENVIRON SAFE, V122, P322, DOI 10.1016/j.ecoenv.2015.08.013"
"   Liu LW, 2018, SCI TOTAL ENVIRON, V633, P206, DOI 10.1016/j.scitotenv.2018.03.161"
"   Logares R, 2013, ISME J, V7, P937, DOI 10.1038/ismej.2012.168"
"   Luo LY, 2019, APPL SOIL ECOL, V139, P100, DOI 10.1016/j.apsoil.2019.03.022"
"   Macdonald SJ, 2017, SOIL RES, V55, P692, DOI 10.1071/SR16190"
"   Mahar A, 2016, ECOTOX ENVIRON SAFE, V126, P111, DOI 10.1016/j.ecoenv.2015.12.023"
"   Meier LA, 2019, BIOGEOSCIENCES, V16, P2481, DOI 10.5194/bg-16-2481-2019"
"   Mendez MO, 2008, APPL ENVIRON MICROB, V74, P3899, DOI 10.1128/AEM.02883-07"
"   Pacwa-Plociniczak M, 2018, WATER AIR SOIL POLL, V229, DOI 10.1007/s11270-017-3655-3"
"   Panyushkina AE, 2019, SCI REP-UK, V9, DOI 10.1038/s41598-019-51486-1"
"   Quast C, 2013, NUCLEIC ACIDS RES, V41, pD590, DOI 10.1093/nar/gks1219"
"   Ren Y, 2022, IMETA, V1, DOI 10.1002/imt2.12"
"   Sarwar N, 2017, CHEMOSPHERE, V171, P710, DOI 10.1016/j.chemosphere.2016.12.116"
"   [????????? Shui Xinfang], 2021, [???????????, Geological Review], V67, P752"
"   Smit Y, 2011, FOOD MICROBIOL, V28, P331, DOI 10.1016/j.fm.2010.11.008"
"   STACKEBRANDT E, 1994, INT J SYST BACTERIOL, V44, P846, DOI 10.1099/00207713-44-4-846"
"   Taran M, 2019, IRAN J MICROBIOL, V11, P60, DOI 10.18502/ijm.v11i1.707"
"   Vangronsveld J, 2009, ENVIRON SCI POLLUT R, V16, P765, DOI 10.1007/s11356-009-0213-6"
"   Wang Q, 2007, APPL ENVIRON MICROB, V73, P5261, DOI 10.1128/AEM.00062-07"
"   Wang QY, 2009, EUR J SOIL BIOL, V45, P229, DOI 10.1016/j.ejsobi.2008.12.002"
"   Wang Y., 2014, Practical Handbook of Reference Materials for Geoanalysis, V2nd ed., P39"
"   Wang YX, 2023, CELL, V186, P4803, DOI 10.1016/j.cell.2023.08.017"
"   Wu ZX, 2018, APPL SOIL ECOL, V125, P192, DOI 10.1016/j.apsoil.2018.01.011"
"   Xu N, 2016, EUR J SOIL BIOL, V74, P1, DOI 10.1016/j.ejsobi.2016.02.004"
"   Xu YL, 2018, SCI TOTAL ENVIRON, V621, P148, DOI 10.1016/j.scitotenv.2017.11.214"
"   Yin HQ, 2015, SCI REP-UK, V5, DOI 10.1038/srep14266"
"   Yin K, 2019, CHEM ENG J, V360, P1553, DOI [10.1016/j.cej.2018.10.226, 10.1016/j.jhazmat.2024.134410]"
"   Zeng P, 2019, CHEMOSPHERE, V220, P237, DOI 10.1016/j.chemosphere.2018.12.073"
"   Zhang H, 2018, ECOTOX ENVIRON SAFE, V165, P314, DOI 10.1016/j.ecoenv.2018.09.010"
"   Zhang J, 2015, WATER AIR SOIL POLL, V226, DOI 10.1007/s11270-015-2309-6"
"   Zhang TR, 2024, ENVIRON POLLUT, V358, DOI 10.1016/j.envpol.2024.124492"
NR 66
TC 3
Z9 5
U1 10
U2 35
PU MDPI
PI BASEL
"PA MDPI AG, Grosspeteranlage 5, CH-4052 BASEL, SWITZERLAND"
EI 2075-163X
J9 MINERALS-BASEL
JI Minerals
PD DEC
PY 2024
VL 14
IS 12
AR 1237
DI 10.3390/min14121237
PG 13
WC Geochemistry & Geophysics; Mineralogy; Mining & Mineral Processing
WE Science Citation Index Expanded (SCI-EXPANDED)
SC Geochemistry & Geophysics; Mineralogy; Mining & Mineral Processing

GA Q5E0W
UT WOS:001384899600001
OA gold
DA 2026-04-06
ER

PT J
"AU Xing, YH"
"   Luo, XS"
"   Liu, S"
"   Wan, WJ"
"   Huang, QY"
"   Chen, WL"
"AF Xing, Yonghui"
"   Luo, Xuesong"
"   Liu, Song"
"   Wan, Wenjie"
"   Huang, Qiaoyun"
"   Chen, Wenli"
TI Synergistic effect of biofilm growth and cadmium adsorption via
   compositional changes of extracellular matrix in montmorillonite system
SO BIORESOURCE TECHNOLOGY
LA English
DT Article
DE Montmorillonite; Cd2+; Biofilm; Extracellular polymeric substance;
   Bioremediation
ID POLYMERIC SUBSTANCES; ESCHERICHIA-COLI; EXTRACTION; MEMBRANE; CD(II);
   ACID; SOIL; DNA
"AB The interaction of bacterial biofilm and clay minerals provides great potential for heavy metal remediation in contaminated soil, yet, little is known about how heavy metal, clay minerals and their combinations affect the bacterial biofilm performance and heavy metal adsorption. In this study, the response of biofilm development as well as Cd2+ adsorption in the presence of Cd2+ and montmorillonite has been deciphered. Low concentrations of Cd2+ and montmorillonite or their combinations enhanced biofilm formation by increasing polysaccharides proportion in the biofilm matrix, and the maximum adsorption capacity of Cd2+ by biofilm was increased by 1.5 times. Furthermore, the immobilization of Cd2+ by soil was significantly improved when S14-biofilm was introduced. Such results could gain deeper insight into bacterial survival tactics in the complex systems which makes major contribution to microbial remediation of heavy metal polluted environments."
"C1 [Xing, Yonghui; Luo, Xuesong; Liu, Song; Wan, Wenjie; Huang, Qiaoyun; Chen, Wenli] Huazhong Agr Univ, State Key Lab Agr Microbiol, Wuhan 430070, Peoples R China."
"   [Luo, Xuesong; Huang, Qiaoyun] Huazhong Agr Univ, Coll Resources & Environm, Hubei Key Lab Soil Environm & Pollut Remediat, Wuhan 430070, Peoples R China."
C3 Huazhong Agricultural University; Huazhong Agricultural University
"RP Chen, WL (corresponding author), Huazhong Agr Univ, State Key Lab Agr Microbiol, Wuhan 430070, Peoples R China."
EM wlchen@mail.hzau.edu.cn
"RI Wan, Wenjie/AAT-7226-2021; Xing, Yonghui/NGS-3691-2025; Huang,"
"   Qiaoyun/A-8418-2012; ???, ??????/JAO-5524-2023"
"OI Wan, Wenjie/0000-0001-7150-6138; "
"FU National Key Research and Development Program of China [2017YFA0605001,"
   2016YFD0800206]; Technical Innovation Major Projects of Hubei Province
   [2018ABA092]
FX This study was funded by The National Key Research and Development
"   Program of China (2017YFA0605001 and 2016YFD0800206), and the Technical"
   Innovation Major Projects of Hubei Province (2018ABA092).
"CR [Anonymous], 2012, MBIO"
"   [Anonymous], 2019, SCI TOTAL ENVIRON, DOI DOI 10.1016/J.SCITOTENV.2018.12.419"
"   [Anonymous], 2019, BIORESOURCE TECHNOL, DOI DOI 10.1016/J.BIORTECH.2018.12.100"
"   [Anonymous], 2019, J BACTERIOL, DOI DOI 10.1128/JB.00385-19"
"   [Anonymous], 1998, GEOL SOC SPEC PUBL"
"   [Anonymous], 2008, J APPL MICROBIOL, DOI DOI 10.1111/J.1365-2672.2007.03688.X"
"   Coolon JD, 2010, MOL ECOL, V19, P67, DOI 10.1111/j.1365-294X.2009.04485.x"
"   Du HH, 2017, ENVIRON POLLUT, V229, P871, DOI 10.1016/j.envpol.2017.07.052"
"   Flemming HC, 2019, NAT REV MICROBIOL, V17, P247, DOI 10.1038/s41579-019-0158-9"
"   Flemming HC, 2016, NAT REV MICROBIOL, V14, P563, DOI 10.1038/nrmicro.2016.94"
"   Flemming HC, 2010, NAT REV MICROBIOL, V8, P623, DOI [10.1038/nrmicro2415, 10.1080/0892701031000072190]"
"   Frolund B, 1996, WATER RES, V30, P1749, DOI 10.1016/0043-1354(95)00323-1"
"   Fulaz S, 2019, TRENDS MICROBIOL, V27, P915, DOI 10.1016/j.tim.2019.07.004"
"   Gong XM, 2018, BIORESOURCE TECHNOL, V253, P64, DOI 10.1016/j.biortech.2018.01.018"
"   Grandy AS, 2000, SOIL BIOL BIOCHEM, V32, P725, DOI 10.1016/S0038-0717(99)00203-5"
"   Herten M, 2017, FRONT MICROBIOL, V8, DOI 10.3389/fmicb.2017.02333"
"   Hua XY, 2019, SCI TOTAL ENVIRON, V670, P478, DOI 10.1016/j.scitotenv.2019.03.246"
"   Huang Q., 2015, SCI REP, V20, P168"
"   Jermy A, 2010, NAT REV MICROBIOL, V8, P612, DOI 10.1038/nrmicro2424"
"   Jiao YQ, 2010, APPL ENVIRON MICROB, V76, P2916, DOI 10.1128/AEM.02289-09"
"   LaPaglia C, 1997, APPL ENVIRON MICROB, V63, P3158, DOI 10.1128/AEM.63.8.3158-3163.1997"
"   Lennon JT, 2016, ENVIRON MICROBIOL, V18, P2732, DOI 10.1111/1462-2920.13331"
"   Li F, 2018, J HAZARD MATER, V358, P178, DOI 10.1016/j.jhazmat.2018.06.066"
"   Liao SM, 2014, J BACTERIOL, V196, P2355, DOI 10.1128/JB.01493-14"
"   Liu Z, 2011, MINER ENG, V24, P839, DOI 10.1016/j.mineng.2011.03.005"
"   Lynch JM, 2004, BIOL FERT SOILS, V40, P363, DOI 10.1007/s00374-004-0784-9"
"   Ma WT, 2017, NPJ BIOFILMS MICROBI, V3, DOI 10.1038/s41522-017-0013-6"
"   Miller MB, 2001, ANNU REV MICROBIOL, V55, P165, DOI 10.1146/annurev.micro.55.1.165"
"   Nagar E, 2015, ENVIRON MICROBIOL, V17, P1477, DOI 10.1111/1462-2920.12583"

"   Nkoh JN, 2019, ENVIRON POLLUT, V247, P136, DOI 10.1016/j.envpol.2019.01.038"
"   Omoike A, 2004, BIOMACROMOLECULES, V5, P1219, DOI 10.1021/bm034461z"
"   Raklami A, 2019, MICROORGANISMS, V7, DOI 10.3390/microorganisms7070212"
"   Raynaud X, 2014, PLOS ONE, V9, DOI 10.1371/journal.pone.0087217"
"   R??hs PA, 2014, COLLOID SURFACE B, V117, P174, DOI 10.1016/j.colsurfb.2014.02.023"
"   Sheng GP, 2005, APPL MICROBIOL BIOT, V67, P125, DOI 10.1007/s00253-004-1704-5"
"   SMALL P, 1994, J BACTERIOL, V176, P1729, DOI 10.1128/jb.176.6.1729-1737.1994"
"   Stoodley P, 2002, ANNU REV MICROBIOL, V56, P187, DOI 10.1146/annurev.micro.56.012302.160705"
"   Sule P, 2009, LETT APPL MICROBIOL, V49, P299, DOI 10.1111/j.1472-765X.2009.02659.x"
"   Sun M, 2012, SEP PURIF TECHNOL, V95, P216, DOI 10.1016/j.seppur.2012.05.010"
"   Sun Y, 2020, BIORESOURCE TECHNOL, V302, DOI 10.1016/j.biortech.2020.122797"
"   Sun YD, 1999, VET MICROBIOL, V65, P153, DOI 10.1016/S0378-1135(98)00286-7"
"   Uppuluri P, 2018, MBIO, V9, DOI [10.1128/mBio.01338-18, 10.1128/mbio.01338-18]"
"   Uroz S, 2015, TRENDS MICROBIOL, V23, P751, DOI 10.1016/j.tim.2015.10.004"
"   Velmourougane K, 2017, J BASIC MICROB, V57, P548, DOI 10.1002/jobm.201700046"
"   Vlamakis H, 2013, NAT REV MICROBIOL, V11, P157, DOI 10.1038/nrmicro2960"
"   Xu S., 2020, ENV POLLUT, V258, P113"
"   Xu XJ, 2015, FRONT MICROBIOL, V6, DOI 10.3389/fmicb.2015.01422"
"   Yan SJ, 2019, ENVIRON POLLUT, V252, P1509, DOI 10.1016/j.envpol.2019.06.071"
"   Yang KJ, 2019, BIORESOURCE TECHNOL, V293, DOI 10.1016/j.biortech.2019.122095"
"   Yang W, 2018, MICROBIOL RES, V215, P1, DOI 10.1016/j.micres.2018.06.002"
"   Yu GH, 2011, ENVIRON SCI TECHNOL, V45, P9224, DOI 10.1021/es201483f"
"   Yuan SJ, 2011, ENVIRON SCI TECHNOL, V45, P1152, DOI 10.1021/es1030905"
NR 52
TC 34
Z9 40
U1 4
U2 214
PU ELSEVIER SCI LTD
PI London
"PA 125 London Wall, London, the United Kingdom"
SN 0960-8524
EI 1873-2976
J9 BIORESOURCE TECHNOL
JI Bioresour. Technol.
PD NOV
PY 2020
VL 315
AR 123742
DI 10.1016/j.biortech.2020.123742
PG 8
WC Agricultural Engineering; Biotechnology & Applied Microbiology; Energy &
   Fuels
WE Science Citation Index Expanded (SCI-EXPANDED)
SC Agriculture; Biotechnology & Applied Microbiology; Energy & Fuels
GA NB7TR
UT WOS:000560717900019
PM 32659425
DA 2026-04-06
ER

PT J
"AU Ahemad, M"
"AF Ahemad, Munees"
TI Remediation of metalliferous soils through the heavy metal resistant
   plant growth promoting bacteria: Paradigms and prospects
SO ARABIAN JOURNAL OF CHEMISTRY
LA English
DT Review
DE Bioremediation; Bioinoculant; Heavy metals; Plant growth promoting
   bacteria; Metal resistance; Rhizobacteria
ID RADIATA L. WILCZEK; BRADYRHIZOBIUM SP VIGNA; QUIZALAFOP-P-ETHYL;
   BRASSICA-JUNCEA; PSEUDOMONAS-PUTIDA; INSECTICIDE-TOLERANT; ENDOPHYTIC
   BACTERIA; CONTAMINATED SOIL; INDIAN MUSTARD; NICKEL
"AB Various industrial, agricultural and military operations have released huge amounts of toxic heavy metals into the environment with deleterious effects on soils, water and air. Under metal stress, soil microorganisms including plant growth promoting bacteria (PGPB) have developed many strategies to evade the toxicity generated by the various heavy metals. Such metal resistant PGPB, when used as bioinoculant or biofertilizers, significantly improved the growth of plants in heavy metal contaminated/stressed soils. Application of bacteria possessing metal detoxifying traits along with plant-beneficial properties is a cost effective and environmental friendly metal bioremediation approach. This review highlights the different mechanisms of metal resistance and plant growth promotion of metal resistant PGPB as well as the recent development in exploitation of these bacteria in bioremediation of heavy metals in different agroecosystems. (C) 2014 The Author. Production and hosting by Elsevier B.V. on behalf of King Saud University."
"C1 [Ahemad, Munees] Aligarh Muslim Univ, Fac Agr Sci, Dept Agr Microbiol, Aligarh 202002, Uttar Pradesh, India."
C3 Aligarh Muslim University
"RP Ahemad, M (corresponding author), Aligarh Muslim Univ, Fac Agr Sci, Dept Agr Microbiol, Aligarh 202002, Uttar Pradesh, India."
EM muneesmicro@rediffmail.com
"CR Abou-Shanab RA, 2003, NEW PHYTOL, V158, P219, DOI 10.1046/j.1469-8137.2003.00721.x"
"   Adriano DC, 2001, TRACE ELEMENTS TERRE, V2, DOI DOI 10.1007/978-0-387-21510-5"
"   Ahemad M, 2011, J AGR SCI TECH-IRAN, V13, P1209"
"   Ahemad M., 2010, Research Journal of Microbiology, V5, P849, DOI 10.3923/jm.2010.849.857"

"   Ahemad M., 2013, Biochem. Mol. Biol, V1, P19, DOI [DOI 10.12966/bmb.06.02.2013, 10.12966/bmb.06.02.2013, DOI 10.12966/BMB.06.02.2013]"
"   Ahemad M, 2011, BACTERIOL J, V2, P12, DOI DOI 10.3923/BJ.2012.12.21"
"   Ahemad M, 2009, MICROBES SUSTAINABLE"
"   Ahemad M., 2011, MICROBIOL J, V1, P54, DOI [DOI 10.3923/MJ.2011.54.64, 10.3923/mj.2011.54.64]"
"   Ahemad M., 2010, EURASIA J BIOSCI, P88, DOI [DOI 10.5053/EJOBIOS.2010.4.0.11, 10.5053/ejobios.2010.4.0.11]"
"   AHEMAD M, 2010, AFR J BASIC APPL SCI, V2, P111"
"   Ahemad M, 2015, 3 BIOTECH, V5, P111, DOI 10.1007/s13205-014-0206-0"
"   Ahemad M, 2014, FOLIA MICROBIOL, V59, P321, DOI 10.1007/s12223-014-0304-8"
"   Ahemad Munees, 2014, Journal of King Saud University Science, V26, P1, DOI 10.1016/j.jksus.2013.05.001"
"   Ahemad M, 2012, IIOAB J, V3, P39"
"   Ahemad M, 2012, ANN MICROBIOL, V62, P1531, DOI 10.1007/s13213-011-0407-2"
"   Ahemad M, 2012, SAUDI J BIOL SCI, V19, P451, DOI 10.1016/j.sjbs.2012.06.003"
"   Ahemad M, 2012, J PEST SCI, V85, P29, DOI 10.1007/s10340-011-0402-1"
"   Ahemad M, 2012, CHEMOSPHERE, V86, P945, DOI 10.1016/j.chemosphere.2011.11.013"
"   Ahemad M, 2012, ACTA PHYSIOL PLANT, V34, P245, DOI 10.1007/s11738-011-0823-8"
"   Ahemad M, 2011, ACTA MICROBIOL IMM H, V58, P169, DOI 10.1556/AMicr.58.2011.3.1"
"   Ahemad M, 2011, SYMBIOSIS, V54, P17, DOI 10.1007/s13199-011-0122-6"
"   Ahemad M, 2011, SCI HORTIC-AMSTERDAM, V129, P266, DOI 10.1016/j.scienta.2011.03.036"
"   Ahemad M, 2011, PEST MANAG SCI, V67, P423, DOI 10.1002/ps.2080"
"   Ahemad M, 2011, J PEST SCI, V84, P123, DOI 10.1007/s10340-010-0335-0"
"   Ahemad M, 2011, PESTIC BIOCHEM PHYS, V100, P51, DOI 10.1016/j.pestbp.2011.02.004"
"   Ahemad M, 2011, B ENVIRON CONTAM TOX, V86, P384, DOI 10.1007/s00128-011-0231-1"
"   Ahemad M, 2011, ECOTOXICOLOGY, V20, P661, DOI 10.1007/s10646-011-0606-4"
"   Ahemad M, 2011, J PLANT GROWTH REGUL, V30, P334, DOI 10.1007/s00344-011-9195-y"
"   Ahemad M, 2011, CURR MICROBIOL, V62, P532, DOI 10.1007/s00284-010-9740-0"
"   Ahemad M, 2010, ANN MICROBIOL, V60, P735, DOI 10.1007/s13213-010-0124-2"
"   Ahemad M, 2010, PESTIC BIOCHEM PHYS, V98, P183, DOI 10.1016/j.pestbp.2010.06.005"
"   Ahemad M, 2010, CROP PROT, V29, P325, DOI 10.1016/j.cropro.2010.01.005"
"   Ahemad M, 2010, ARCH ENVIRON CON TOX, V58, P361, DOI 10.1007/s00244-009-9382-z"
"   Ahemad M, 2009, B ENVIRON CONTAM TOX, V82, P761, DOI 10.1007/s00128-009-9692-x"
"   Alkorta I., 2004, Reviews in Environmental Science and Bio/Technology, V3, P71, DOI 10.1023/B:RESB.0000040059.70899.3d"
"   Alloway BJ., 1995, HEAVY METALS SOILS, V2nd, P3"
"   Barzanti R, 2007, MICROB ECOL, V53, P306, DOI 10.1007/s00248-006-9164-3"
"   Belimov AA, 2005, SOIL BIOL BIOCHEM, V37, P241, DOI 10.1016/j.soilbio.2004.07.033"
"   BIRO B, 1995, ACTA BIOL HUNG, V46, P9"
"   Bishop PL., 2002, Pollution prevention: fundamentals and practice"
"   Braud A, 2009, CHEMOSPHERE, V74, P280, DOI 10.1016/j.chemosphere.2008.09.013"
"   Braud Armelle., 2006, Water, Air, and Soil Pollution, V6, P261, DOI [10.1007/s11267-005-9022-1, DOI 10.1007/S11267-005-9022-1]"
"   Burd GI, 2000, CAN J MICROBIOL, V46, P237, DOI 10.1139/cjm-46-3-237"
"   Carrillo-Casta??eda G, 2003, J PLANT NUTR, V26, P1801, DOI 10.1081/PLN-120023284"
"   Cazorla FM, 2007, J APPL MICROBIOL, V103, P1950, DOI 10.1111/j.1365-2672.2007.03433.x"
"   COOKSEY DA, 1994, FEMS MICROBIOL REV, V14, P381, DOI 10.1111/j.1574-6976.1994.tb00112.x"
"   Dary M, 2010, J HAZARD MATER, V177, P323, DOI 10.1016/j.jhazmat.2009.12.035"
"   Di Gregorio S, 2006, CHEMOSPHERE, V63, P293, DOI 10.1016/j.chemosphere.2005.07.020"
"   DiazRavina M, 1996, APPL ENVIRON MICROB, V62, P2970, DOI 10.1128/AEM.62.8.2970-2977.1996"
"   Diels L, 2003, HYDROMETALLURGY, V71, P235, DOI 10.1016/S0304-386X(03)00161-0"
"   Dimkpa C, 2008, CAN J MICROBIOL, V54, P163, DOI [10.1139/W07-130, 10.1139/w07-130]"
"   Dimkpa CO, 2009, SOIL BIOL BIOCHEM, V41, P154, DOI 10.1016/j.soilbio.2008.10.010"
"   Faisal M, 2006, LETT APPL MICROBIOL, V43, P461, DOI 10.1111/j.1472-765X.2006.01977.x"
"   Faisal M, 2005, BIOTECHNOL LETT, V27, P943, DOI 10.1007/s10529-005-7188-2"
"   Fischerov?? Z, 2006, ENVIRON POLLUT, V144, P93, DOI 10.1016/j.envpol.2006.01.005"
"   Ganesan V, 2008, CURR MICROBIOL, V56, P403, DOI 10.1007/s00284-008-9099-7"
"   Gilis A, 1998, J IND MICROBIOL BIOT, V20, P61, DOI 10.1038/sj.jim.2900478"
"   Giller KE, 1998, SOIL BIOL BIOCHEM, V30, P1389, DOI 10.1016/S0038-0717(97)00270-8"
"   Glick BR, 2012, PLANT GROWTH PROMOTI"
"   G??hre V, 2006, PLANTA, V223, P1115, DOI 10.1007/s00425-006-0225-0"
"   Gray EJ, 2005, SOIL BIOL BIOCHEM, V37, P395, DOI 10.1016/j.soilbio.2004.08.030"
"   Gupta A, 2002, CURR MICROBIOL, V45, P323, DOI 10.1007/s00284-002-3762-1"
"   Gupta A., 2005, MICROBIOL RES, V160, P385"
"   Haferburg G, 2007, J BASIC MICROB, V47, P453, DOI 10.1002/jobm.200700275"
"   Hashim MA, 2011, J ENVIRON MANAGE, V92, P2355, DOI 10.1016/j.jenvman.2011.06.009"
"   Hemambika B., 2011, Journal of Ecology and the Natural Environment, V3, P168"
"   Hewitt E.J, 1983, TALS MICRONUTRIENTS, P277"
"   Hutchinson TC, 1997, J GEOCHEM EXPLOR, V58, P323, DOI 10.1016/S0375-6742(96)00067-2"
"   Hynninen A, 2010, THESIS, P1"
"   Ilieva A., 2014, Journal of Central European Agriculture, V15, P23, DOI 10.5513/JCEA01/15.2.1446"
"   Indiragandhi P, 2008, CURR MICROBIOL, V56, P327, DOI 10.1007/s00284-007-9086-4"
"   Jahanian A., 2012, International Journal of Agriculture and Crop Sciences (IJACS), V4, P923"
"   Jha PN, 2007, J APPL MICROBIOL, V103, P1311, DOI 10.1111/j.1365-2672.2007.03383.x"
"   Jiang CY, 2008, CHEMOSPHERE, V72, P157, DOI 10.1016/j.chemosphere.2008.02.006"
"   Jing Yan-De, 2007, Journal of Zhejiang University-Science B, V8, P192, DOI 10.1631/jzus.2007.B0192"
"   Kabata-Pendias A., 2001, Trace Elements in Soil and Plants, VThird"
"   Kehres DG, 2003, FEMS MICROBIOL REV, V27, P263, DOI 10.1016/S0168-6445(03)00052-4"
"   Khan AG, 2005, J TRACE ELEM MED BIO, V18, P355, DOI 10.1016/j.jtemb.2005.02.006"
"   Kosev V., 2014, International Journal of Pharmacy and Life Sciences (IJPLS), V5, P3570"
"   Kotrba P, 2009, BIOTECHNOL ADV, V27, P799, DOI 10.1016/j.biotechadv.2009.06.003"
"   Kumar KV, 2008, CHEMOSPHERE, V72, P678, DOI 10.1016/j.chemosphere.2008.03.025"
"   Lasat MM, 2002, J ENVIRON QUAL, V31, P109, DOI 10.2134/jeq2002.0109"
"   Lena QM, 1997, J ENVIRON QUAL, V26, P264"
"   Lovley D.R., 1997, Iron and Related Transition Metals in Microbial Metabolism"

"   LOVLEY DR, 1995, J IND MICROBIOL, V14, P85, DOI 10.1007/BF01569889"
"   Ma Y, 2011, INT J PHYTOREMEDIAT, V13, P126, DOI 10.1080/15226511003671403"
"   Ma Y, 2009, J ENVIRON MANAGE, V90, P831, DOI 10.1016/j.jenvman.2008.01.014"
"   Ma Y, 2011, J HAZARD MATER, V195, P230, DOI 10.1016/j.jhazmat.2011.08.034"
"   Ma Y, 2009, J HAZARD MATER, V166, P1154, DOI 10.1016/j.jhazmat.2008.12.018"
"   Ma Y, 2009, CHEMOSPHERE, V75, P719, DOI 10.1016/j.chemosphere.2009.01.056"
"   Madhaiyan M, 2007, CHEMOSPHERE, V69, P220, DOI 10.1016/j.chemosphere.2007.04.017"
"   Madhaiyan M, 2008, J MICROBIOL BIOTECHN, V18, P1005"
"   Munees Ahemad Munees Ahemad, 2012, Journal of the Saudi Society of Agricultural Sciences, V11, P63, DOI 10.1016/j.jssas.2011.10.001"
"   Munees Ahemad Munees Ahemad, 2012, Emirates Journal of Food and Agriculture, V24, P334"
"   Munees Ahemad Munees Ahemad, 2009, Journal of Crop Science and Biotechnology, V12, P213"
"   Mu??oz R, 2006, CHEMOSPHERE, V63, P903, DOI 10.1016/j.chemosphere.2005.09.062"
"   Nies DH, 1999, APPL MICROBIOL BIOT, V51, P730, DOI 10.1007/s002530051457"
"   NIES DH, 1995, J IND MICROBIOL, V14, P186, DOI 10.1007/BF01569902"
"   Pajuelo E, 2008, ENVIRON POLLUT, V154, P203, DOI 10.1016/j.envpol.2007.10.015"
"   Rajkumar M, 2006, CHEMOSPHERE, V62, P741, DOI 10.1016/j.chemosphere.2005.04.117"
"   Rajkumar M, 2005, J ENVIRON BIOL, V26, P693"
"   Rajkumar M, 2008, J BASIC MICROB, V48, P500, DOI 10.1002/jobm.200800073"
"   Rajkumar M, 2008, BIORESOURCE TECHNOL, V99, P3491, DOI 10.1016/j.biortech.2007.07.046"
"   Rajkumar M, 2010, TRENDS BIOTECHNOL, V28, P142, DOI 10.1016/j.tibtech.2009.12.002"
"   Rani A, 2009, INT BIODETER BIODEGR, V63, P62, DOI 10.1016/j.ibiod.2008.07.002"
"   Ranjard L, 2003, APPL ENVIRON MICROB, V69, P3784, DOI 10.1128/AEM.69.7.3784-3790.2003"
"   Roane TM, 2000, ENVIRON MICROBIOL, P55"
"   Robinson B, 2001, AGR ECOSYST ENVIRON, V87, P315, DOI 10.1016/S0167-8809(01)00146-3"
"   Rodrigues EP, 2008, PLANT SOIL, V302, P249, DOI 10.1007/s11104-007-9476-1"
"   Rubio LM, 2008, ANNU REV MICROBIOL, V62, P93, DOI 10.1146/annurev.micro.62.081307.162737"
"   Saravanakumar D, 2007, CROP PROT, V26, P556, DOI 10.1016/j.cropro.2006.05.007"
"   Selvakumar G, 2008, LETT APPL MICROBIOL, V46, P171, DOI 10.1111/j.1472-765X.2007.02282.x"
"   Seth CS, 2008, ECOTOX ENVIRON SAFE, V71, P76, DOI 10.1016/j.ecoenv.2007.10.030"
"   Shaharoona B, 2008, APPL MICROBIOL BIOT, V79, P147, DOI 10.1007/s00253-008-1419-0"
"   Shaw B.P., 2004, HEAVY METAL STRESS P, P84, DOI [10.1007/978-3-662-07743-6_4, DOI 10.1007/978-3-662-07743-6_4]"
"   Sheng XF, 2008, CAN J MICROBIOL, V54, P417, DOI [10.1139/W08-020, 10.1139/w08-020]"
"   Sheng XF, 2006, CHEMOSPHERE, V64, P1036, DOI 10.1016/j.chemosphere.2006.01.051"
"   SHIER WT, 1994, J TOXICOL-TOXIN REV, V13, P205, DOI 10.3109/15569549409089960"
"   Singh V., 2010, Int J Pharm Sci Rev Res, V3, P164"
"   Sinha S, 2008, CURR MICROBIOL, V56, P55, DOI 10.1007/s00284-007-9038-z"
"   Tank N, 2010, J PLANT INTERACT, V5, P51, DOI 10.1080/17429140903125848"
"   Tank N, 2009, J BASIC MICROB, V49, P195, DOI 10.1002/jobm.200800090"
"   Tian F, 2009, BRAZ J MICROBIOL, V40, P276, DOI [10.1590/S1517-83822009000200013, 10.1590/S1517-838220090002000013]"
"   Tripathi M, 2005, CURR MICROBIOL, V50, P233, DOI 10.1007/s00284-004-4459-4"
"   Tsezos M, 2009, ADV MATER RES-SWITZ, V71-73, P527, DOI 10.4028/www.scientific.net/AMR.71-73.527"
"   Vangronsveld Jaco, 1994, P149, DOI 10.1002/9783527615919.ch6"
"   Vivas A, 2006, CHEMOSPHERE, V62, P1523, DOI 10.1016/j.chemosphere.2005.06.053"
"   Wang JL, 2006, BIOTECHNOL ADV, V24, P427, DOI 10.1016/j.biotechadv.2006.03.001"
"   WANG YT, 1995, J IND MICROBIOL, V14, P159, DOI 10.1007/BF01569898"
"   Wani P. A., 2007, Acta Agronomica Hungarica, V55, P315, DOI 10.1556/AAgr.55.2007.3.7"
"   Wani PA, 2007, CHEMOSPHERE, V70, P36, DOI 10.1016/j.chemosphere.2007.07.028"
"   Wani PA, 2008, BIOTECHNOL LETT, V30, P159, DOI 10.1007/s10529-007-9515-2"
"   Wani PA, 2010, FOOD CHEM TOXICOL, V48, P3262, DOI 10.1016/j.fct.2010.08.035"
"   Whiting SN, 2001, ENVIRON SCI TECHNOL, V35, P3144, DOI 10.1021/es001938v"
"   Wu CH, 2006, APPL ENVIRON MICROB, V72, P1129, DOI 10.1128/AEM.72.2.1129-1134.2006"
"   Zaidi S, 2006, CHEMOSPHERE, V64, P991, DOI 10.1016/j.chemosphere.2005.12.057"
NR 136
TC 166
Z9 195
U1 5
U2 209
PU ELSEVIER
PI AMSTERDAM
"PA RADARWEG 29, 1043 NX AMSTERDAM, NETHERLANDS"
SN 1878-5352
EI 1878-5379
J9 ARAB J CHEM
JI Arab. J. Chem.
PD NOV
PY 2019
VL 12
IS 7
BP 1365
EP 1377
DI 10.1016/j.arabjc.2014.11.020
PG 13
"WC Chemistry, Multidisciplinary"
WE Science Citation Index Expanded (SCI-EXPANDED)
SC Chemistry
GA JK6LT
UT WOS:000494954400045
"OA Green Submitted, gold"
DA 2026-04-06
ER

PT J
"AU Luo, N"
"   Zhang, XJ"
"   Gong, LF"
"   Yang, LQ"
"   Yao, Q"
"   Song, JF"
"AF Luo, N."
"   Zhang, X. J."
"   Gong, L. F."
"   Yang, L. Q."
"   Yao, Q."
"   Song, J. F."
TI Review of Remediation Methods for Soil Contaminated with Cadmium
SO EURASIAN SOIL SCIENCE
LA English
DT Article
DE bioremediation; cadmium (Cd) pollution; chemical remediation; combined remediation; soil
ID HEAVY-METALS; GRAPHENE OXIDE; STRAIN; CD; PHYTOREMEDIATION; MECHANISM; GROWTH; LEAD; PHYTOEXTRACTION; ENHANCEMENT
"AB The heavy metal pollution is increasing with the continuous development of industrialisation and urbanisation. Among them, cadmium (Cd) contamination of soil (Anthrosol) is particularly serious. In order to solve this global problem, the technologies of soil remediation for heavy metal-contaminated sites have been studied in detail by taking into account the uniqueness of Cd-contaminated soil, including physical remediation, chemical remediation and bioremediation. Among them, chemical remediation (chemical leaching and chemical immobilisation), bioremediation (phytoremediation and microbial remediation) and their combined remediation have become the main technologies for soil remediation. In this paper, various composite remediation methods for soil with Cd contamination are reviewed, and there influencing factors, advantages and disadvantages, remediation mechanisms and practical applications are discussed in depth. In summary, this paper will help to understand current research progress, challenges and opportunities of soil remediation technologies for the sites contaminated with Cd and other heavy metals with similar properties, and provide a reference for the rational selection and development of remediation technologies for soil with Cd and other heavy metal contamination."
"C1 [Luo, N.; Yang, L. Q.; Yao, Q.] Baotou Bur Ecol & Environm, Baotou 014060, Peoples R China."
"   [Zhang, X. J.] Northeast Agr Univ, Sch Water Conservancy & Civil Engn, Int Cooperat Joint Lab Hlth Cold Reg Black Soil Ha, Minist Educ, Harbin 150030, Peoples R China."
"   [Gong, L. F.] Northeast Forestry Univ, Sch Foreign Languages, Harbin 150040, Peoples R China."
"   [Song, J. F.] Northeast Forestry Univ, Sch Ecol, Harbin 150040, Peoples R China."
C3 Northeast Agricultural University - China; Northeast Forestry University - China; Northeast Forestry University - China
"RP Song, JF (corresponding author), Northeast Forestry Univ, Sch Ecol, Harbin 150040, Peoples R China."
EM songnefu@126.com
"RI Song, Jinfeng/J-3625-2015"
FU Natural Science Foundation of Heilongjiang Province [TD2023C006]
FX This work was supported by the Natural Science Foundation of Heilongjiang Province (TD2023C006).
"CR Ahmed F, 2013, J HAZARD MATER, V256, P33, DOI 10.1016/j.jhazmat.2013.03.064"
"   Ayub MA, 2023, EURASIAN SOIL SCI+, V56, pS300, DOI 10.1134/S1064229323601555"
"   Bianucci E, 2013, ENZYME MICROB TECH, V53, P345, DOI 10.1016/j.enzmictec.2013.07.007"
"   Bianucci E, 2012, BIOMETALS, V25, P23, DOI 10.1007/s10534-011-9480-z"
"   [????????? Bu Heshen], 2020, [????????????, Acta Agrestia Sinica], V28, P64"
"   Chang HX, 2017, FRONT MICROBIOL, V8, DOI 10.3389/fmicb.2017.00519"
"   Chen HY, 2015, SCI TOTAL ENVIRON, V512, P143, DOI 10.1016/j.scitotenv.2015.01.025"
"   Chen T., 2018, Twenty years of research and development on soil pollution and remediation in China, P465, DOI 10.1007/978-981-10-6029-8_27"
"   Chen Yao-ning, 2017, Huanjing Kexue, V38, P4398, DOI 10.13227/j.hjkx.201703039"
"   Chen Yaoning, 2017, Journal of Hunan University (Natural Sciences), V44, P127, DOI 10.16339/j.cnki.hdxbzkb.2017.12.020"
"   Dawwam G. E., 2013, Annals of Agricultural Science (Cairo), V58, P195, DOI 10.1016/j.aoas.2013.07.007"
"   [????????? Deng Pingxiang], 2016, [??????????????????, Chinese Journal of Environmental Engineering], V10, P5245"
"   Deng XiaoXia Deng XiaoXia, 2018, Southwest China Journal of Agricultural Sciences, V31, P1221"
"   [????????? Ding Zhuhong], 2009, [????€?????????????, Ecology and Environmental Sciences], V18, P777"
"   Dong C. X., China Patent, Patent No. [2013-07-15, 20130715]"
"   Du Aixue, 2008, Chinese Journal of Applied and Environmental Biology, V14, P650, DOI 10.3724/SP.J.1145.2008.00650"
"   Du JJ, 2015, RSC ADV, V5, P27009, DOI 10.1039/c5ra01045d"
"   [????????? DUAN Xuejun], 2007, [????€???????, Ecology and Environment], V16, P1665"
"   Evangelou MWH, 2007, CHEMOSPHERE, V68, P989, DOI 10.1016/j.chemosphere.2007.01.062"
"   Falkowski PG, 2008, SCIENCE, V320, P1034, DOI 10.1126/science.1153213"
"   Fan YQ, 2019, INT J PHYTOREMEDIAT, V21, P733, DOI 10.1080/15226514.2018.1556592"
"   Fang Z. R., 2015, J. Xichang Univ. (Nat. Sci. Ed.), V29, P15, DOI [10.3969/j.issn.1673-1891.2015.04.002, DOI 10.3969/J.ISSN.1673-1891.2015.04.002]"
"   Fu Jin, 2011, Chinese Journal of Applied and Environmental Biology, V17, P717, DOI [10.3724/sp.j.1145.2011.00717, 10.3724/SP.J.1145.2011.00717]"
"   [????????? Gan Xinhong], 2017, [??????, Soils], V49, P135"
"   ?????????, 2013, [??????????????????, Nonferrous Metals Engineering], V3, P49"
"   Houssou AA, 2022, BIOCHAR, V4, DOI 10.1007/s42773-021-00126-x"
"   Hu Hong-Qing, 2017, Journal of Plant Nutrition and Fertilizers, V23, P1676, DOI 10.11674/zwyf.17299"
"   Hu N., 2019, J. Nanhua University. Sci. Tech., V33, P16, DOI DOI 10.19431/J.CNKI.1673-0062.2019.02.003"
"   Huang Chengtao, 2018, Chinese Journal of Applied and Environmental Biology, V24, P901, DOI 10.19675/j.cnki.1006-687x.2017.09030"
"   Jiang J. R., 2018, Master's Thesis"

"   Laguna-Teno F, 2020, FRONT MICROBIOL, V11, DOI 10.3389/fmicb.2020.01943"
"   [?????? Lan Xiang], 2016, [??????????????????, Acta Scientiae Circumstantiae], V36, P2095"
"   Li Ting, 2021, Chinese Journal of Applied and Environmental Biology, V27, P1405, DOI 10.19675/j.cnki.1006-687x.2020.06062"
"   Li X., 2008, Ph. D. Thesis"
"   [????????? LI Xiaoyun], 2007, [??????????????????, Journal of Jilin Agricultural Sciences], V32, P26"
"   Li Y, 2019, J APPL MICROBIOL, V126, P919, DOI 10.1111/jam.14165"
"   Li Y, 2018, CHEMOSPHERE, V202, P280, DOI 10.1016/j.chemosphere.2018.03.097"
"   [????????? Li Yunshi], 2015, [????€???????, Acta Ecologica Sinica], V35, P6881"
"   [????????? Lin Xiaomin], 2020, [?????????????????€????????????, Journal of Central South University of Forestry & Technology], V40, P149"
"   Liu F, 2024, SCI TOTAL ENVIRON, V912, DOI 10.1016/j.scitotenv.2023.168990"
"   Liu H., 2016, Master's Thesis"
"   Liu SB, 2011, ACS NANO, V5, P6971, DOI 10.1021/nn202451x"
"   [????????? Long Meirong], 2013, [?????????????€????, Microbiology China], V40, P2203"
"   Lu Y. H., 2018, Environ. Sci. Technol., V31, P84, DOI [10.3969/j.issn.1674-4829.2018.05.0, DOI 10.3969/J.ISSN.1674-4829.2018.05.0]"
"   Lv B., 2017, Environ. Sustainability Dev, V42, P4, DOI [10.3969/j.issn.1673-288X.2017.02.020, DOI 10.3969/J.ISSN.1673-288X.2017.02.020]"
"   Ma L. Y., 2017, Curr. Biotechnol, V7, P241, DOI [10.19586/j.2095-2341.2016.0091, DOI 10.19586/J.2095-2341.2016.0091]"
"   Maldonado-Gonz??lez MM, 2015, ENVIRON MICROBIOL, V17, P3139, DOI 10.1111/1462-2920.12725"
"   Mo TD, 2022, ECOL PROCESS, V11, DOI 10.1186/s13717-021-00347-9"
"   Nayak AK, 2018, INT J PHYTOREMEDIAT, V20, P682, DOI 10.1080/15226514.2017.1413332"
"   Nejad ZD, 2018, ENVIRON GEOCHEM HLTH, V40, P927, DOI 10.1007/s10653-017-9964-z"
"   Nicolova M, 2017, J GEOCHEM EXPLOR, V182, P242, DOI 10.1016/j.gexplo.2016.11.003"
"   Nies DH, 2000, EXTREMOPHILES, V4, P77, DOI 10.1007/s007920050140"
"   Ning WJ, 2023, LAND DEGRAD DEV, V34, P534, DOI 10.1002/ldr.4477"
"   Park YJ, 2008, BIORESOURCE TECHNOL, V99, P7458, DOI 10.1016/j.biortech.2008.02.024"
"   Prapagdee B, 2015, INT J ENVIRON SCI TE, V12, P3843, DOI 10.1007/s13762-015-0816-z"
"   Qian X. Y., 2017, Master's Thesis"
"   Rees F, 2014, EUR J SOIL SCI, V65, P149, DOI 10.1111/ejss.12107"
"   Ren AZ, 2011, INT J PHYTOREMEDIAT, V13, P233, DOI 10.1080/15226511003671387"
"   Rong Y, 2017, J AGR FOOD CHEM, V65, P9191, DOI 10.1021/acs.jafc.7b03646"
"   Shabayev VP, 2020, EURASIAN SOIL SCI+, V53, P809, DOI 10.1134/S1064229320060113"
"   She W, 2023, ACTA PHYSIOL PLANT, V45, DOI 10.1007/s11738-023-03621-9"
"   Shi PL, 2016, WATER AIR SOIL POLL, V227, DOI 10.1007/s11270-016-3167-6"
"   Shukla L, 2020, Asian Journal of Research in Chemistry, V13, P299, DOI [10.5958/0974-4150.2020.00058.9, 10.1080/15320383.2019.1592108, DOI 10.1080/15320383.2019.1592108, DOI 10.12691/AEES-10-1-3]"
"   Song A, 2021, SCI TOTAL ENVIRON, V796, DOI 10.1016/j.scitotenv.2021.148797"
"   Su YL, 2015, CHEM ENG J, V279, P47, DOI 10.1016/j.cej.2015.05.005"
"   [????????? SUN Jialong], 2007, [??????????????????, Guizhou Agricultural Sciences], V35, P147"
"   Tan WN, 2011, PEDOSPHERE, V21, P223, DOI 10.1016/S1002-0160(11)60121-5"
"   Tang YeTao Tang YeTao, 2005, Acta Scientiarum Naturalium Universitatis Sunyatseni, V44, P135"
"   Tseng SC, 2021, INT J ENV RES PUB HE, V18, DOI 10.3390/ijerph18168661"
"   Rehman Z, 2023, SCI TOTAL ENVIRON, V867, DOI 10.1016/j.scitotenv.2023.161468"
"   van der Meer JR, 2006, FRONT ECOL ENVIRON, V4, P35, DOI 10.1890/1540-9295(2006)004[0035:EPPSOM]2.0.CO;2"
"   Wang C. M., 2023, Master's Thesis"
"   Wang XH, 2018, ENVIRON POLLUT, V242, P1488, DOI 10.1016/j.envpol.2018.08.039"
"   Wang XM, 2017, CHEMOSPHERE, V168, P699, DOI 10.1016/j.chemosphere.2016.11.005"
"   Watcharamusik A., 2008, Environment Asia, V1, P37"
"   ?????????, 2004, [???????€????, Chinese science bulletin], V49, P2568"
"   Wu HanLin Wu HanLin, 2016, Guizhou Agricultural Sciences, V44, P94"
"   Xie H., 2015, Master's Thesis"
"   Xie Y, 2016, FRONT PLANT SCI, V7, DOI 10.3389/fpls.2016.00755"
"   Yang T. L., 2023, Nonferrous Met. (Extr. Metall.), V11, P128, DOI [10.3969/j.issn.1007-7545.2023.11.017, DOI 10.3969/J.ISSN.1007-7545.2023.11.017]"
"   Yang W, 2018, MICROBIOL RES, V215, P1, DOI 10.1016/j.micres.2018.06.002"
"   Yang ZH, 2018, ENVIRON SCI POLLUT R, V25, P11970, DOI 10.1007/s11356-018-1492-6"
"   [?????? Yin Fei], 2015, [????????????????????????, Journal of Agro-Environment Science], V34, P438"
"   Yin Yong-chao, 2014, Shengtaixue Zazhi, V33, P3060"
"   Ying JiaoYan Ying JiaoYan, 2003, China Environmental Science, V23, P575"
"   Zhang B., China patent, Patent No. [CN201610018191.3, 20161005]"
"   Zhang X. B., 2022, Mod. Min, V5, P209, DOI [10.3969/j.issn.1674-6082.2022.05.051, DOI 10.3969/J.ISSN.1674-6082.2022.05.051]"
"   Zhang X, 2018, ECOTOX ENVIRON SAFE, V157, P21, DOI 10.1016/j.ecoenv.2018.03.047"
"   Zhao Xiao-feng, 2023, Huanjing Kexue, V44, P2786, DOI 10.13227/j.hjkx.202205305"
"   Zhao Yu, 2022, Asian Journal of Ecotoxicology, V17, P144, DOI 10.7524/AJE.1673-5897.20220104002"
"   [?????? Zhou Geng], 2017, [??????????????????, Acta Scientiae Circumstantiae], V37, P2076"
NR 91
TC 7
Z9 7
U1 69
U2 177
PU PLEIADES PUBLISHING LTD
PI TORTOLA
"PA TROPIC ISLE BLDG, PO BOX 3331. ROAD TOWN, TORTOLA, BRITISH VIRGIN ISL"
SN 1064-2293
EI 1556-195X
J9 EURASIAN SOIL SCI+
JI Eurasian Soil Sci.
PD FEB 20
PY 2025
VL 58

IS 1
AR 3
DI 10.1134/S1064229324602087
PG 15
WC Soil Science
WE Science Citation Index Expanded (SCI-EXPANDED)
SC Agriculture
GA X8Z8T
UT WOS:001428168800012
DA 2026-04-06
ER

PT J
"AU Khattak, MR"
"   Shah, G"
"   Naeem, R"
"   Fayyaz, M"
"   Rehman, M"
"   Anees, M"
"   Rha, ES"
"   Jamil, M"
"AF Khattak, Mehvish Riaz"
"   Shah, Gulmeena"
"   Naeem, Rehan"
"   Fayyaz, Muhammad"
"   Rehman, Maha"
"   Anees, Muhammad"
"   Rha, Eui Shik"
"   Jamil, Muhammad"
TI ASSESSMENT OF HEAVY METAL TOLERANCE AND REDUCTION POTENTIAL OF BACILLUS
   SP. ISOLATED FROM THE SOILS CONTAMINATED WITH INDUSTRIAL EFFLUENTS
SO FRESENIUS ENVIRONMENTAL BULLETIN
LA English
DT Article
DE Bacillus sp.; Heavy metals; Tolerance; Reduction potential; 16S rDNA
ID ALCALIGENES-EUTROPHUS; RESISTANCE; BACTERIA; CADMIUM; REMOVAL;
   IDENTIFICATION; BIOSORPTION; COBALT; LEAD
"AB Globally, rapid industrial growth has increased the heavy metals concentrations in the soils, which emphasize the need to explore the soils of Gadoon, Sawabi and Hayatabad industrial estates, Peshawar, Pakistan for the presence of heavy metals tolerant bacteria. A total of 12 bacterial isolates were identified on the basis of phenotypic, biochemical and molecular approaches. 16S rRNA gene sequences of these heavy metals tolerant bacterial isolates were compared with reference data bases and clustered using Maximum Likelihood, which confirmed that Lysinibacillus sp. (PMBL-7) was differentiated from the rest of Bacillus sp. These bacterial isolates were also evaluated for their metals reducing ability, which revealed that Bacillus altitudinis (PMBL-4) was the most optimal strain showing maximum reducing ability against multiple heavy metals. It had shown a maximum reductions of Cr+6 (34.1 %), Mn+2 (32.5 %), Cu+2 (13.5 %) and Cd+2 (28.65 %). The present study advocates that Bacillus sp. isolated from heavy metal concentrated soils has high tolerance and reduction potentials against multiple heavy metals, which emphasize the importance of evaluating metals tolerance and reducing ability of bacterial isolates in the remediation of heavy metals concentrated sites of Gadoon, Sawabi and Hayatabad industrial estates of Pakistan."
"C1 [Khattak, Mehvish Riaz; Shah, Gulmeena; Naeem, Rehan; Fayyaz, Muhammad; Rehman, Maha; Jamil, Muhammad] Kohat Univ Sci & Technol, Dept Biotechnol & Genet Engn, Kohat 26000, Pakistan."
"   [Anees, Muhammad] Kohat Univ Sci & Technol, Dept Microbiol, Kohat 26000, Pakistan."
"   [Rha, Eui Shik] Sunchon Natl Univ, Dept Well Being Resources, Suncheon Si, Jeollanam Do, South Korea."
C3 Kohat University of Science & Technology; Kohat University of Science &
   Technology; Sunchon National University
"RP Jamil, M (corresponding author), Kohat Univ Sci & Technol, Dept Biotechnol & Genet Engn, Kohat 26000, Pakistan."
EM dr.jamil@kust.edu.pk
"RI ; Anees, Muhammad/AAQ-6920-2020; Naeem, Rehan/NJR-8348-2025; Jamil,"
   Muhammad/D-1489-2012
"OI Shah, Gulmeena/0000-0001-8632-6092; Jamil, Muhammad/0000-0002-2889-6676"
FU HEC [20-4372]
FX This work was supported by research grant received from HEC (Grant No.
   20-4372)
"CR Arg??ello JM, 2003, J MEMBRANE BIOL, V195, P93, DOI 10.1007/s00232-003-2048-2"
"   Baker-Austin C, 2006, TRENDS MICROBIOL, V14, P176, DOI 10.1016/j.tim.2006.02.006"
"   Basha S. A., 2014, International Journal of Current Microbiology and Applied Sciences, V3, P785"
"   Batool R, 2014, BRAZ J MICROBIOL, V45, P573, DOI 10.1590/S1517-83822014000200028"
"   Berlanga GA, 2009, TEX J SCI, V61, P259"
"   Campbell PGC, 2006, ENVIRON CHEM, V3, P387, DOI 10.1071/EN06075"
"   COOKSEY DA, 1994, FEMS MICROBIOL REV, V14, P381, DOI 10.1111/j.1574-6976.1994.tb00112.x"
"   Dalsgarrd A., 2002, 722 DANN EPA RROYAL, P1"
"   Das N, 2008, INDIAN J BIOTECHNOL, V7, P159"
"   Dewhirst FE, 2005, J BACTERIOL, V187, P6106, DOI 10.1128/JB.187.17.6106-6118.2005"
"   Duruibe JO, 2007, INT J PHYS SCI, V2, P112"
"   Fritze D., 2002, Applications and systematics of bacillus and relatives"
"   Gikas P, 2008, J HAZARD MATER, V159, P187, DOI 10.1016/j.jhazmat.2008.02.048"
"   GUPTA SD, 1995, J BACTERIOL, V177, P4207, DOI 10.1128/jb.177.15.4207-4215.1995"
"   Gustays L., 2009, PROTOPLASMA, V243, P3"
"   Hameed MSA, 2006, AFR J BIOTECHNOL, V5, P1819"
"   HAMER DH, 1986, ANNU REV BIOCHEM, V55, P913, DOI 10.1146/annurev.bi.55.070186.004405"
"   Hassen A, 1998, BIORESOURCE TECHNOL, V64, P7, DOI 10.1016/S0960-8524(97)00161-2"
"   Hemambika B., 2011, Journal of Ecology and the Natural Environment, V3, P168"

"   Holt J.G., 1994, BERGEYS MANUAL DETER"
"   Huston WM, 2002, MOL MICROBIOL, V45, P1741, DOI 10.1046/j.1365-2958.2002.03132.x"
"   Jain A.N., 2012, International Journal of Science and Research, V6, P2319"
"   Kamaludeen Sara Parwin Banu, 2003, Indian Journal of Experimental Biology, V41, P972"
"   Kamika I, 2013, BMC MICROBIOL, V13, DOI 10.1186/1471-2180-13-28"
"   Kermani AJN, 2010, IRAN J ENVIRON HEALT, V7, P279"
"   Kim J, 2006, J ENVIRON ENG-ASCE, V132, P777, DOI 10.1061/(ASCE)0733-9372(2006)132:7(777)"
"   Kratochvil D, 1998, TRENDS BIOTECHNOL, V16, P291, DOI 10.1016/S0167-7799(98)01218-9"
"   Malik A, 2000, WORLD J MICROB BIOT, V16, P177, DOI 10.1023/A:1008905902282"
"   MERGEAY M, 1985, J BACTERIOL, V162, P328, DOI 10.1128/JB.162.1.328-334.1985"
"   Murugesan A. G., 2007, Journal of Applied Sciences and Environmental Management, V11, P71"
"   Nanda M., 2011, INT J ENV SCI, V2, P789"
"   Naz T, 2016, TOXICOL IND HEALTH, V32, P1619, DOI 10.1177/0748233715569900"
"   Nichols CAM, 2005, MAR BIOTECHNOL, V7, P253, DOI 10.1007/s10126-004-5118-2"
"   Nies DH, 1999, APPL MICROBIOL BIOT, V51, P730, DOI 10.1007/s002530051457"
"   NIES DH, 1995, J BACTERIOL, V177, P2707, DOI 10.1128/jb.177.10.2707-2712.1995"
"   Pal A, 2008, INDIAN J MICROBIOL, V48, P49, DOI 10.1007/s12088-008-0006-5"
"   Peitzsch N, 1998, APPL ENVIRON MICROB, V64, P453"
"   Pelczar M.J., 1957, Manual of Microbiological Methods"
"   Raja CE., 2009, International Joint Symposium on Geo-disaster Prevention and Geo-environment in Asia, P205"
"   Rajeshkumar R., 2011, Our Nature, V9, P49"
"   Ram??rez-D??az MI, 2008, BIOMETALS, V21, P321, DOI 10.1007/s10534-007-9121-8"
"   Roane TM, 2001, APPL ENVIRON MICROB, V67, P3208, DOI 10.1128/AEM.67.7.3208-3215.2001"
"   SAITOU N, 1987, MOL BIOL EVOL, V4, P406, DOI 10.1093/oxfordjournals.molbev.a040454"
"   Salem HM, 2000, INT C ENV HAZ MIT, P542"
"   SCHMIDT T, 1989, FEMS MICROBIOL ECOL, V62, P315, DOI 10.1111/j.1574-6968.1989.tb03386.x"
"   Sevgi E, 2010, TURK J BIOL, V34, P423, DOI 10.3906/biy-0901-23"
"   Sharma PK, 2000, APPL ENVIRON MICROB, V66, P3083, DOI 10.1128/AEM.66.7.3083-3087.2000"
"   Shi ZH, 2013, PLOS ONE, V8, DOI 10.1371/journal.pone.0064351"
"   Singh S, 2007, WORLD J MICROB BIOT, V23, P1747, DOI 10.1007/s11274-007-9424-5"
"   Singh V., 2010, Int J Pharm Sci Rev Res, V3, P164"
"   Smrithi A, 2012, INT J ADV BIOTECHNOL, V3, P644"
"   Sparks DL, 2005, ELEMENTS, V1, P193, DOI 10.2113/gselements.1.4.193"
"   Tsezos M., 2011, WORKSH CLEAN PROD NA"
"   TURNER JS, 1993, J BIOL CHEM, V268, P4494"
"   Vijayaraghavan K, 2008, BIOTECHNOL ADV, V26, P266, DOI 10.1016/j.biotechadv.2008.02.002"
NR 55
TC 8
Z9 8
U1 0
U2 8
PU PARLAR SCIENTIFIC PUBLICATIONS (P S P)
PI FREISING
"PA ANGERSTR. 12, 85354 FREISING, GERMANY"
SN 1018-4619
EI 1610-2304
J9 FRESEN ENVIRON BULL
JI Fresenius Environ. Bull.
PY 2021
VL 30
IS 4A
BP 4391
EP 4401
PG 11
WC Environmental Sciences
WE Science Citation Index Expanded (SCI-EXPANDED)
SC Environmental Sciences & Ecology
GA SN3DD
UT WOS:000658172800051
DA 2026-04-06
ER

PT J
"AU Hu, W"
"   Duan, RY"
"   Dai, Q"
"   Yang, H"
"   Zhang, Y"
"   Meng, FM"
"   Lin, YX"
"AF Hu, Wei"
"   Duan, Renyan"
"   Dai, Qian"
"   Yang, Hui"
"   Zhang, Yu"
"   Meng, Fumin"
"   Lin, Yuxiang"
TI Effects of biochar immobilization Bacillus subtilis on heavy metal
"   accumulation, rhizosphere microorganisms, and metabolism in rice"
SO JOURNAL OF APPLIED MICROBIOLOGY
LA English
DT Article
DE heavy metal pollution; Bacillus subtilis; microorganisms; metabolites

ID BACTERIA; CADMIUM; RARE
"AB Aim The mining of heavy metals leads to environmental pollution in the soil of surrounding farmland. A method for reducing heavy metal concentrations in crops grown on heavy metal-enriched soils is utilizing functional microorganisms and biochar. The 9311 rice variety was used as a model plant to investigate the effects and underlying mechanisms of biochar-immobilized functional microbial materials on reducing the accumulation of heavy metals (Sb, Cr, Pb, and Cd) in plants.Materials and methods Three remediation materials, including Bacillus subtilis (BS), coconut shell biochar (bc), and biochar-immobilized B. subtilis (MIX) were evaluated for their effects on heavy metal accumulation, rhizosphere microbial diversity, and metabolism in rice. All three treatments (BS, bc, and MIX) significantly increased the yield of rice plants and reduced the accumulation of heavy metals in rice. The MIX group reduced heavy metal enrichment the most in all three test treatments while also increasing crop yield. The 16S rRNA analysis revealed that the MIX treatment significantly altered the abundance of some non-dominant bacteria (e.g. Spirochaeta, RB41, S0134, and Bryobacter). The LC-MS analysis showed that compared to the control group, the BS, bc, and MIX treatment groups had 35, 94, and 74 differential metabolites, respectively, mainly including esters, amino acids and their derivatives, ketones, and terpenoids. The MIX group had a significant effect on the metabolic pathways involved in purine metabolism, glyoxylate and dicarboxylate metabolism, and phenylpropanoid biosynthesis in rice. The overall effect of the MIX treatment was superior to that of applying BS and bc separately.Conclusion The application of BS, bc, and MIX treatments in Sb-contaminated farmland can increase rice yield and reduce the absorption of heavy metals. The overall effect of the MIX treatment is superior to applying each separately (BS and BC). These three treatments had no significant effect on the rhizosphere biodiversity or the dominant bacterial structure of the rice rhizosphere but had an impact on non-dominant bacteria and root metabolism in rice roots."
"C1 [Hu, Wei; Duan, Renyan; Dai, Qian; Yang, Hui; Zhang, Yu; Meng, Fumin; Lin, Yuxiang] Hunan Univ Humanities Sci & Technol, Coll Agr & Biotechnol, Loudi 417000, Hunan, Peoples R China."
"   [Duan, Renyan] Key Lab Dev Utilizat Qual & Safety Control Charact, Loudi 417000, Hunan, Peoples R China."
"C3 Hunan University Of Humanities, Science & Technology"
"RP Duan, RY (corresponding author), Hunan Univ Humanities Sci & Technol, Coll Agr & Biotechnol, Loudi 417000, Hunan, Peoples R China."
EM renyanduan@huhst.edu.cn
FU National Natural Science Foundation of China [32371589]; Natural Science Foundation of Hunan Province [2024JJ7238]; Aid program for Science and Technology Innovative Research Team in Higher Educational Institutions of Hunan Province [201937924]
FX This work was supported by grants from the National Natural Science
"   Foundation of China (32371589), Natural Science Foundation of Hunan"
"   Province (2024JJ7238), and the Aid program for Science and Technology"
Innovative Research Team in Higher Educational Institutions of Hunan Province (201937924).
"CR Albqmi M, 2024, BMC PLANT BIOL, V24, DOI 10.1186/s12870-024-05044-1"
"   Basak BB, 2017, NATL ACAD SCI LETT, V40, P245, DOI 10.1007/s40009-017-0561-6"
"   Bolan N, 2022, ENVIRON INT, V158, DOI 10.1016/j.envint.2021.106908"
"   Cao H, 2023, PLANT PHYSIOL BIOCH, V199, DOI 10.1016/j.plaphy.2023.107727"
"   Cao Y, 2024, MICROBIOL RES, V283, DOI 10.1016/j.micres.2024.127690"
"   Chen YH, 2021, ENVIRON SCI POLLUT R, V28, P23036, DOI 10.1007/s11356-020-12203-y"
"   Chen YL, 2024, SCI TOTAL ENVIRON, V912, DOI 10.1016/j.scitotenv.2023.168874"
"   Chu JW, 2021, SCI TOTAL ENVIRON, V771, DOI 10.1016/j.scitotenv.2020.144643"
"   Chu YT, 2023, ANTIOXIDANTS-BASEL, V12, DOI 10.3390/antiox12111917"
"   Duan RY, 2021, PEERJ, V9, DOI 10.7717/peerj.12302"
"   Gao YN, 2022, J ENVIRON MANAGE, V313, DOI 10.1016/j.jenvman.2022.114973"
"   Hong Y, 2022, SCI TOTAL ENVIRON, V851, DOI 10.1016/j.scitotenv.2022.158033"
"   Ibrahim IA, 2019, IRAQ J SCI, P2618, DOI [10.24996/ijs.2019.60.12.10, 10.24996/ijs.2019.60.12.10, DOI 10.24996/IJS.2019.60.12.10]"
"   Jiao S, 2017, MOL ECOL, V26, P5305, DOI 10.1111/mec.14218"
"   Kaushik S, 2024, BIOMETALS, V37, P755, DOI 10.1007/s10534-023-00569-8"
"   Krishnen G, 2011, CAN J MICROBIOL, V57, P914, DOI [10.1139/W11-059, 10.1139/w11-059]"
"   Kumar Abhishek, 2023, Chemosphere, V344, P140417, DOI [10.1016/j.chemosphere.2023.140417, 10.1016/j.chemosphere.2023.140417]"
"   Lai ZY, 2022, ECOTOX ENVIRON SAFE, V233, DOI 10.1016/j.ecoenv.2022.113317"
"   Li C, 2019, ANAL CHIM ACTA, V1063, P47, DOI 10.1016/j.aca.2019.03.012"
"   Li H, 2022, INT J MOL SCI, V23, DOI 10.3390/ijms23031594"
"   Li Q, 2022, SCI TOTAL ENVIRON, V825, DOI 10.1016/j.scitotenv.2022.154136"
"   Li YB, 2024, J HAZARD MATER, V462, DOI 10.1016/j.jhazmat.2023.132807"
"   Li YK, 2018, INT J MOL SCI, V19, DOI 10.3390/ijms19051471"
"   Lin XY, 2016, SCI TOTAL ENVIRON, V569, P97, DOI 10.1016/j.scitotenv.2016.06.121"
"   Liu N, 2023, FRONT MICROBIOL, V13, DOI 10.3389/fmicb.2022.1079114"
"   Lynch MDJ, 2015, NAT REV MICROBIOL, V13, P217, DOI 10.1038/nrmicro3400"
"   Mao WJ, 2022, ENVIRON SCI POLLUT R, V29, P49322, DOI 10.1007/s11356-021-18276-7"
"   Ortiz A, 2022, SUSTAINABILITY-BASEL, V14, DOI 10.3390/su14095358"
"   Phringpaen Waranya, 2023, Trends in Sciences, V20, DOI [10.48048/tis.2023.7032, 10.48048/tis.2023.7032]"
"   Qi WY, 2023, J HAZARD MATER, V458, DOI 10.1016/j.jhazmat.2023.131921"
"   Ros R, 2014, TRENDS PLANT SCI, V19, P564, DOI 10.1016/j.tplants.2014.06.003"
"   Shan SP, 2019, ECOTOX ENVIRON SAFE, V169, P292, DOI 10.1016/j.ecoenv.2018.11.030"
"   Shang Er-ping, 2018, Huanjing Kexue, V39, P4670, DOI 10.13227/j.hjkx.201802139"
"   Si SC, 2023, SCI TOTAL ENVIRON, V892, DOI 10.1016/j.scitotenv.2023.164556"
"   Song PP, 2023, PEERJ, V11, DOI 10.7717/peerj.14841"
"   Sun ML, 2024, FRONT MICROBIOL, V15, DOI 10.3389/fmicb.2024.1352354"
"   Sun WJ, 2021, CURR POLLUT REP, V7, P201, DOI 10.1007/s40726-021-00175-7"
"   Sun YP, 2023, ECOTOX ENVIRON SAFE, V263, DOI 10.1016/j.ecoenv.2023.115214"
"   Verma S, 2019, ENVIRON TECHNOL INNO, V14, DOI 10.1016/j.eti.2019.100369"
"   Wang CY, 2022, SUSTAINABILITY-BASEL, V14, DOI 10.3390/su141912164"
"   Wang WB, 2020, AMB EXPRESS, V10, DOI 10.1186/s13568-020-01074-8"
"   Wrobel Monika, 2023, Int J Environ Res Public Health, V20, DOI [10.3390/ijerph20064964, 10.3390/ijerph20064964]"

"   Xu M, 2017, SCI TOTAL ENVIRON, V599, P1297, DOI [10.1016/j.scitotenv.2017.05.086, 10.1016/j.scitotenv2017.05.086]"
"   Xu WJ, 2024, J HAZARD MATER, V466, DOI 10.1016/j.jhazmat.2024.133486"
"   Yang WT, 2023, LAND DEGRAD DEV, V34, P2791, DOI 10.1002/ldr.4647"
"   Yi-cheng W, 2020, J CHEM-NY, V2020, DOI 10.1155/2020/8758432"
"   Yu XJ, 2024, SCI TOTAL ENVIRON, V926, DOI 10.1016/j.scitotenv.2024.171614"
"   Yu Y, 2022, ENVIRON RES, V203, DOI 10.1016/j.envres.2021.111801"
"   Yuan ZG, 2018, MOLECULES, V23, DOI 10.3390/molecules23112840"
"   Zhang CG, 2020, CARBOHYD RES, V494, DOI 10.1016/j.carres.2020.108056"
"   Zhang QG, 2021, ENVIRON POLLUT, V272, DOI 10.1016/j.envpol.2020.115989"
"   Zhang RJ, 2023, PLOS ONE, V18, DOI 10.1371/journal.pone.0279192"
"   Zhang RH, 2022, ECOTOX ENVIRON SAFE, V248, DOI 10.1016/j.ecoenv.2022.114316"
"   Zhao L, 2020, SCI TOTAL ENVIRON, V719, DOI 10.1016/j.scitotenv.2020.137499"
"   Zheng RL, 2015, ENVIRON SCI POLLUT R, V22, P11097, DOI 10.1007/s11356-015-4268-2"
NR 55
TC 5
Z9 5
U1 31
U2 54
PU OXFORD UNIV PRESS
PI OXFORD
"PA GREAT CLARENDON ST, OXFORD OX2 6DP, the United Kingdom"
SN 1364-5072
EI 1365-2672
J9 J APPL MICROBIOL
JI J. Appl. Microbiol.
PD APR
PY 2025
VL 136
IS 4
AR lxaf083
DI 10.1093/jambio/lxaf083
PG 11
WC Biotechnology & Applied Microbiology; Microbiology
WE Science Citation Index Expanded (SCI-EXPANDED)
SC Biotechnology & Applied Microbiology; Microbiology
GA 1ES6K
UT WOS:001463272200001
PM 40180586
DA 2026-04-06
ER

PT J
"AU Mao, SX"
"   Zhao, QC"
"   Ma, SY"
"   Du, YB"
"   Shi, JS"
"   Zou, JC"
"   Qiu, ZL"
"   Yu, CH"
"AF Mao, Shuaixian"
"   Zhao, Qiancheng"
"   Ma, Suya"
"   Du, Yanbin"
"   Shi, Jinshuai"
"   Zou, Jiacheng"
"   Qiu, Ziliang"
"   Yu, Caihong"
TI Heavy metal pollution pressure in gold mines shows overall suppressed
   biochemical sulfur cycle
SO INTERNATIONAL BIODETERIORATION & BIODEGRADATION
LA English
DT Article
DE Sulfur cycling gene; Sulfur cycling path; Sulfur metabolizing
   microorganisms; Heavy metals; Mine tailings
ID ESCHERICHIA-COLI; ACCUMULATION; OXIDATION; IMPACT
"AB Sulfur cycle is an important material cycle in soil, and soil sulfur-metabolizing microorganisms are one of its prominent drivers. However, the fate of the sulfur cycle under the stressful condition of soil heavy metal pollution due to mining is unknown. In this study, three representative areas with low (L), medium (M) and high (H) levels of heavy metal pollution were selected to investigate the effects of heavy metal contamination levels on sulfur metabolizing microorganisms, sulfur cycling pathways and sulfur cycling genes by using metagenome sequencing and SCycDB sulfur cycle database annotation. The results showed that the relative abundance of sulfur cycle genes in the L, M, and H regions was 6.45 +/- 0.12, 6.29 +/- 0.15, and 5.75 +/- 0.21, respectively; the abundance of sulfur cycle genes showed a decreasing trend with the increase of heavy metal pollution, and the sulfur cycle pathways and microbial sulfur metabolism were inhibited, and the dominant bacteria of the sulfur metabolizing bacterial community evolved gradually from Actinobacteria to Proteobacteria with abundances changing from 0.38 to 0.22 to 0.29 and 0.30, respectively; the proportion of sulfur cycle genes of different types in the sulfur metabolizing metabolizers did not vary with heavy metal pollution, indicating that different sulfur cycling genes in sulfur metabolizers show a uniform decline with increasing heavy metal pollution; dmsA may be a key gene mediating the adaptation and remediation of sulfur metabolizing bacteria to heavy metal pollution. The results reveal the effects of heavy metal pollution in mining areas on the sulfur cycle and provide new insights into the mechanisms of adaptation and remediation of heavy metal pollution by sulfur metabolizing microorganisms."

"C1 [Mao, Shuaixian; Zhao, Qiancheng; Ma, Suya; Du, Yanbin; Shi, Jinshuai; Zou, Jiacheng; Qiu, Ziliang; Yu, Caihong] China Univ Min & Technol Beijing, Sch Chem & Environm Engn, Beijing 100083, Peoples R China."
C3 China University of Mining & Technology
"RP Yu, CH (corresponding author), China Univ Min & Technol Beijing, Sch Chem & Environm Engn, Beijing 100083, Peoples R China."
EM maoshuaixian@hotmail.com; 1104729565@qq.com; 18865559160@163.com;
457958745@qq.com; 168682863@qq.com; 2282217549@qq.com; 952924211@qq.com;
caihongyu@cumtb.edu.cn
"RI zou, jiacheng/PGA-2505-2026; zhao, qiancheng/HHS-6608-2022; Du,"
Yanbin/HMP-4281-2023
"OI Du, Yanbin/0009-0001-3858-8319"
FU National Natural Science Foundation of China [41977029]
FX This work was funded by the National Natural Science Foundation of China
(Grant No. 41977029) .
"CR Baldwin DS, 2012, WATER RES, V46, P965, DOI 10.1016/j.watres.2011.11.065"
" Boetius A, 2000, NATURE, V407, P623, DOI 10.1038/35036572"
" Breiman L, 2001, MACH LEARN, V45, P5, DOI 10.1023/A:1010933404324"
" Cavani L, 2016, J ENVIRON MANAGE, V182, P37, DOI 10.1016/j.jenvman.2016.07.050"
" Erickson AI, 2015, BIOCHEMISTRY-US, V54, P6830, DOI 10.1021/acs.biochem.5b01000"
" Feng YB, 2023, SCI TOTAL ENVIRON, V858, DOI 10.1016/j.scitotenv.2022.160013"
" Fike DA, 2015, ANNU REV EARTH PL SC, V43, P593, DOI 10.1146/annurev-earth-060313-054802"
" Gao L, 2021, INT BIODETER BIODEGR, V162, DOI 10.1016/j.ibiod.2021.105253"
" Gregory GJ, 2021, APPL ENVIRON MICROB, V87, DOI 10.1128/AEM.02235-20"
" Gu J.D., 2016, Biodegradation Testing: So Many Tests but Very Little New Innovation, DOI 10.26789/AEB.2016.01.007"
" Hou TT, 2023, SCI TOTAL ENVIRON, V858, DOI 10.1016/j.scitotenv.2022.159954"
" Hsieh TC, 2016, METHODS ECOL EVOL, V7, P1451, DOI 10.1111/2041-210X.12613"
" Hu XS, 2025, J ENVIRON SCI, V147, P571, DOI 10.1016/j.jes.2023.12.027"
" Hu XS, 2021, SCI TOTAL ENVIRON, V775, DOI 10.1016/j.scitotenv.2021.144946"
" Huang H, 2022, CHEM ENG J, V446, DOI 10.1016/j.cej.2022.137121"
" Huang Q, 2023, J HAZARD MATER, V448, DOI 10.1016/j.jhazmat.2023.130898"
" Joo YC, 2018, J AGR FOOD CHEM, V66, P13454, DOI 10.1021/acs.jafc.8b05093"
" Kadnikov VV, 2016, MICROBIOLOGY+, V85, P449, DOI [10.1134/S002626171604010X, 10.7868/S0026365616040108]"
" LEYH TS, 1992, J BIOL CHEM, V267, P10405"
" Li KC, 2023, CHEM ENG J, V452, DOI 10.1016/j.cej.2022.139136"
" Li SJ, 2022, ENVIRON POLLUT, V305, DOI 10.1016/j.envpol.2022.119322"
" Li SZ, 2020, J HAZARD MATER, V400, DOI 10.1016/j.jhazmat.2020.123255"
" Lia XP, 2010, J HAZARD MATER, V174, P662, DOI 10.1016/j.jhazmat.2009.09.102"
" Liang XJ, 2023, INT BIODETER BIODEGR, V184, DOI 10.1016/j.ibiod.2023.105647"
" Liao XJ, 2021, J CLEAN PROD, V316, DOI 10.1016/j.jclepro.2021.128319"
" Liu HK, 2022, ENVIRON RES, V208, DOI 10.1016/j.envres.2021.112621"
" Liu JR, 2020, EARTH PLANET SC LETT, V536, DOI 10.1016/j.epsl.2020.116165"
" Moran MA, 2019, NAT REV MICROBIOL, V17, P665, DOI 10.1038/s41579-019-0250-1"
" Nogueira EW, 2021, BIORESOURCE TECHNOL, V330, DOI 10.1016/j.biortech.2021.124968"
" Patil PB, 2023, INT BIODETER BIODEGR, V181, DOI 10.1016/j.ibiod.2023.105615"
" Peddie BA, 1999, ANTON LEEUW INT J G, V75, P183, DOI 10.1023/A:1001701400801"
" Qian Y., 2024, J. Hazard Mater., V461"
" RAMSEY MH, 1987, J ANAL ATOM SPECTROM, V2, P497, DOI 10.1039/ja9870200497"
" Segata N, 2011, GENOME BIOL, V12, DOI 10.1186/gb-2011-12-6-r60"
" Senga Y, 2006, ESTUAR COAST SHELF S, V67, P231, DOI 10.1016/j.ecss.2005.11.021"
" Shi X, 2020, ENVIRON INT, V143, DOI 10.1016/j.envint.2020.105923"
" Sivan O, 2014, P NATL ACAD SCI USA, V111, pE4139, DOI 10.1073/pnas.1412269111"
" Sunagawa S, 2015, SCIENCE, V348, DOI 10.1126/science.1261359"
" Swift R. S., 1996, Methods of soil analysis. Part 3 - chemical methods., P1011"
" Tang K, 2020, FRONT MAR SCI, V7, DOI 10.3389/fmars.2020.00068"
" Tao JM, 2017, APPL MICROBIOL BIOT, V101, P1289, DOI 10.1007/s00253-016-7938-1"
" Teng ZJ, 2021, NAT MICROBIOL, V6, P1351, DOI 10.1038/s41564-021-00981-1"
" Trieber CA, 1996, J BIOL CHEM, V271, P4620"
" van der Ploeg JR, 2001, ARCH MICROBIOL, V176, P1, DOI 10.1007/s002030100298"
" van der Ploeg R, 2012, APPL ENVIRON MICROB, V78, P7733, DOI 10.1128/AEM.02093-12"
" Walker AT, 2022, LITHOS, V408-409, DOI 10.1016/j.lithos.2021.106536"
" Wang HL, 2023, ENVIRON SCI TECHNOL, DOI 10.1021/acs.est.2c06546"
" Wang N., 2024, Comput Biol Med, V171"
" Wang X, 2020, SCI TOTAL ENVIRON, V738, DOI 10.1016/j.scitotenv.2020.139709"
" Wang XD, 2023, SOIL BIOL BIOCHEM, V179, DOI 10.1016/j.soilbio.2023.108982"
" Wu B, 2021, SCI TOTAL ENVIRON, V778, DOI 10.1016/j.scitotenv.2021.146085"
" Wu QQ, 2023, FRONT ENV SCI-SWITZ, V10, DOI 10.3389/fenvs.2022.1060277"
" Xi BD, 2021, J HAZARD MATER, V403, DOI 10.1016/j.jhazmat.2020.123853"
" Yao KY, 2023, SCI TOTAL ENVIRON, V874, DOI 10.1016/j.scitotenv.2023.162562"
" Ye JP, 2020, ENVIRON RES, V187, DOI 10.1016/j.envres.2020.109705"
" Yin HQ, 2015, SCI REP-UK, V5, DOI 10.1038/srep14266"
" Yu XL, 2021, MOL ECOL RESOUR, V21, P924, DOI 10.1111/1755-0998.13306"
" Zhang J, 2021, J HAZARD MATER, V402, DOI 10.1016/j.jhazmat.2020.123454"
" Zhao WY, 2022, BIORESOURCE TECHNOL, V349, DOI 10.1016/j.biortech.2021.126431"
" Zhou JZ, 2011, NAT REV MICROBIOL, V9, P452, DOI 10.1038/nrmicro2575"
" Zhou LJ, 2021, SCI TOTAL ENVIRON, V772, DOI 10.1016/j.scitotenv.2021.145464"
" Zhu JJ, 2022, ENVIRON SCI TECHNOL, DOI 10.1021/acs.est.2c00488"
NR 62
TC 7
Z9 8
U1 16
U2 67
PU ELSEVIER SCI LTD
PI London

"PA 125 London Wall, London, the United Kingdom"
SN 0964-8305
EI 1879-0208
J9 INT BIODETER BIODEGR
JI Int. Biodeterior. Biodegrad.
PD JUN
PY 2024
VL 191
AR 105807
DI 10.1016/j.ibiod.2024.105807
EA MAY 2024
PG 10
WC Biotechnology & Applied Microbiology; Environmental Sciences
WE Science Citation Index Expanded (SCI-EXPANDED)
SC Biotechnology & Applied Microbiology; Environmental Sciences & Ecology
GA TL0Y9
UT WOS:001241312900001
DA 2026-04-06
ER

PT J
"AU Zhu, GX"
"   Wang, XF"
"   Chen, RK"
"   Wang, YY"
"   Zhao, XY"
"   Wu, PP"
"   Tu, Q"
"AF Zhu, Guangxu"
"   Wang, Xingfeng"
"   Chen, Rongkun"
"   Wang, Yunyan"
"   Zhao, Xingyun"
"   Wu, Panpan"
"   Tu, Qiang"
TI Remediation of lead-zinc-cadmium co-contaminated soil using distiller's
   grain biochar immobilized with Lactobacillus plantarum
SO CHEMICAL ENGINEERING JOURNAL
LA English
DT Article
DE Heavy metals co-contaminated soil; Microbial immobilization technology;
   Immobilization remediation; Lactobacillus plantarum; Microbe loaded
   biochar
ID WATER
"AB Biochar-immobilized microorganism technology has garnered considerable attention for remediating heavy metal-contaminated soil due to its advantages of high microbial density, rapid reaction kinetics, and enhanced stability. However, remediation efficiency critically depends on biochar type, microbial strain selection, and immobilization methodology. In this study, biochar derived from distiller's grains was combined with Lactobacillus plantarum- a microorganism exhibiting robust heavy metal tolerance- to develop biosorption- immobilized biochar (BIM) and encapsulation- immobilized biochar (EIM). A controlled pot experiment evaluated the effects of BIM, EIM, raw distiller's grains biochar, and free Lactobacillus plantarum at two application rates (0.5 % and 2 % w/w) on (1) physicochemical properties of Pb-Zn-Cd co-contaminated soil, (2) soil enzyme activity, (3) heavy metal speciation, and (4) physiological responses and heavy metal accumulation in chicory (Cichorium intybus). Results revealed that all amendments induced varying degrees of heavy metal passivation, with BIM and EIM demonstrating superior efficacy for metal immobilization. Maximum reductions in bioavailable Pb, Zn, and Cd concentrations were achieved by BIM (50.0 %, 20.0 %, and 39.3 %, respectively) and EIM (28.3 %, 18.8 %, and 38.1 %, respectively). Both BIM and EIM significantly reduced acid-extractable and oxidizable fractions of Pb, Zn, and Cd while enhancing residual fractions, with the 2 % application rate consistently outperforming 0.5 %. Furthermore, these treatments improved critical soil parameters including pH, organic matter content, alkalihydrolyzable nitrogen, available phosphorus, and potassium levels, alongside elevated soil enzyme activities. Crucially, BIM and EIM treatments markedly decreased heavy metal accumulation in chicory, mitigating metalinduced stress while enhancing plant growth and nutrient assimilation. This investigation establishes a theoretical framework and empirical support for utilizing Lactobacillus plantarum-immobilized distiller's grains biochar in remediation multi-metal co-contaminated soils."
"C1 [Zhu, Guangxu; Wang, Xingfeng; Chen, Rongkun; Wang, Yunyan; Zhao, Xingyun; Wu, Panpan] Guiyang Univ, Coll Biol & Environm Engn, Guiyang 550005, Peoples R China."
"   [Tu, Qiang] Shandong Univ, State Key Lab Microbial Technol, Helmholtz Inst Biotechnol, Helmholtz Int Lab Antiinfect, Qingdao 266237, Peoples R China."
"   [Tu, Qiang] Hunan Univ Arts & Sci, Inst Synthet Biol Ind, Changde 415000, Peoples R China."
C3 Guiyang University; Shandong University; Hunan University of Arts &
   Science
"RP Zhu, GX (corresponding author), Guiyang Univ, Coll Biol & Environm Engn, Guiyang 550005, Peoples R China.; Tu, Q (corresponding author), Shandong Univ, State Key Lab Microbial Technol, Helmholtz Inst Biotechnol, Helmholtz Int Lab Antiinfect, Qingdao 266237, Peoples R China."
EM zhugx.10b@igsnrr.ac.cn; tqiang@sdu.edu.cn
"RI Rongkun, Chen/GSM-8976-2022"
FU National Natural Science Foundation of China [42163011]; Guizhou
   Provincial Science and Technology Foundation [ZK [2022] general 016];
   Natural Science Foundation of Shandong Province [ZR2021QC170]; Sixth
   Batch of High-level Innovative Talents Project in Guizhou Province (GCC)
   [[2022] 007]; Guiyang Univeristy Multidisciplinary Team Construction
   Projects in 2025 [GYXK202506]; PhD Research Startup Foundation of
   Guiyang University [GYU-ZRD [2018] 008]

FX This work was financially supported by the National Natural Science
"   Foundation of China (No. 42163011) , Guizhou Provincial Science and"
"   Technology Foundation (ZK [2022] general 016) , Natural Science"
"   Foundation of Shandong Province (ZR2021QC170) , the Sixth Batch of"
High-level Innovative Talents Project in Guizhou Province (GCC [2022]
"   007) , Guiyang Univeristy Multidisciplinary Team Construction Projects"
"   in 2025 [GYXK202506] , and PhD Research Startup Foundation of Guiyang"
University (GYU-ZRD [2018] 008) .
"CR Agrawal K, 2024, CRIT REV BIOTECHNOL, V44, P429, DOI 10.1080/07388551.2023.2170862"
"   Ahmed SF, 2022, ENVIRON TECHNOL INNO, V25, DOI 10.1016/j.eti.2021.102114"
"   Alsafran M, 2023, J PLANT GROWTH REGUL, V42, P3419, DOI 10.1007/s00344-022-10803-1"
"   Ameen FA, 2020, SCI REP-UK, V10, DOI 10.1038/s41598-019-57210-3"
"   Awad M, 2021, BIOMOLECULES, V11, DOI 10.3390/biom11030448"
"   Bouabidi ZB, 2019, ENVIRON CHEM LETT, V17, P241, DOI 10.1007/s10311-018-0795-7"
"   Burns RG, 2013, SOIL BIOL BIOCHEM, V58, P216, DOI 10.1016/j.soilbio.2012.11.009"
"   Cheng N, 2021, BIORESOURCE TECHNOL, V340, DOI 10.1016/j.biortech.2021.125696"
"   Chi WC, 2024, BIOCHAR, V6, DOI 10.1007/s42773-024-00336-z"
"   Gall JE, 2015, ENVIRON MONIT ASSESS, V187, DOI 10.1007/s10661-015-4436-3"
"   Gholizadeh M, 2021, J ENVIRON CHEM ENG, V9, DOI 10.1016/j.jece.2021.105830"
"   Gong YZ, 2022, ENVIRON POLLUT, V314, DOI 10.1016/j.envpol.2022.120232"
"   Guo WP, 2024, J ENVIRON MANAGE, V371, DOI 10.1016/j.jenvman.2024.123212"
"   Han H, 2020, ECOTOX ENVIRON SAFE, V195, DOI 10.1016/j.ecoenv.2020.110375"
"   He JY, 2019, GEODERMA, V337, P359, DOI 10.1016/j.geoderma.2018.09.038"
"   Hou LA, 2024, BIORESOURCE TECHNOL, V413, DOI 10.1016/j.biortech.2024.131518"
"   Huang F, 2020, J CLEAN PROD, V272, DOI 10.1016/j.jclepro.2020.122743"
"   Jeffery S, 2011, AGR ECOSYST ENVIRON, V144, P175, DOI 10.1016/j.agee.2011.08.015"
"   Kalsi A, 2020, ENVIRON TECHNOL INNO, V18, DOI 10.1016/j.eti.2020.100721"
"   Kayoumu M, 2025, BIOCHAR, V7, DOI 10.1007/s42773-025-00452-4"
"   Kebede G, 2021, J CHEM-NY, V2021, DOI 10.1155/2021/9823362"
"   Keiluweit M, 2010, ENVIRON SCI TECHNOL, V44, P1247, DOI 10.1021/es9031419"
"   Khalid S, 2017, J GEOCHEM EXPLOR, V182, P247, DOI 10.1016/j.gexplo.2016.11.021"
"   Khan S, 2021, J HAZARD MATER, V417, DOI 10.1016/j.jhazmat.2021.126039"
"   Kisa D, 2019, ACTA BOT CROAT, V78, P9, DOI 10.2478/botcro-2018-0023"
"   Kumar M, 2024, J TRACE ELEM MED BIO, V83, DOI 10.1016/j.jtemb.2023.127377"
"   Kumar S, 2019, ENVIRON RES, V179, DOI 10.1016/j.envres.2019.108792"
"   Liu HL, 2020, J SOIL SEDIMENT, V20, P2121, DOI 10.1007/s11368-020-02591-6"
"   Lu HL, 2012, WATER RES, V46, P854, DOI 10.1016/j.watres.2011.11.058"
"   Ma H, 2020, J HAZARD MATER, V388, DOI 10.1016/j.jhazmat.2020.122065"
"   Maghraby YR, 2023, ACS OMEGA, V8, P5184, DOI 10.1021/acsomega.2c07560"
"   Manzoor J, 2018, J PLANT NUTR, V41, P1744, DOI 10.1080/01904167.2018.1462382"
"   Maqbool Z, 2024, FUNCT PLANT BIOL, V51, DOI 10.1071/FP23257"
"   Mohanpuria P, 2007, ENVIRON TOXICOL, V22, P368, DOI 10.1002/tox.20273"
"   Mu LY, 2025, AGRICULTURE-BASEL, V15, DOI 10.3390/agriculture15050447"
"   Murshid S, 2021, CHINESE J CHEM ENG, V29, P391, DOI 10.1016/j.cjche.2020.04.008"
"   Najim AA, 2024, J CHEM TECHNOL BIOT, V99, P1707, DOI 10.1002/jctb.7638"
"   Nejad ZD, 2018, ENVIRON GEOCHEM HLTH, V40, P927, DOI 10.1007/s10653-017-9964-z"
"   Nie TT, 2019, SCI TOTAL ENVIRON, V673, P522, DOI 10.1016/j.scitotenv.2019.04.089"
"   Pirzadah TB, 2020, INT BIODETER BIODEGR, V151, DOI 10.1016/j.ibiod.2020.104992"
"   Podg??rska M, 2024, INT J ENVIRON SCI TE, V21, P4645, DOI 10.1007/s13762-023-05206-y"
"   Qi WY, 2023, J HAZARD MATER, V458, DOI 10.1016/j.jhazmat.2023.131921"
"   Qi X, 2022, J HAZARD MATER, V436, DOI 10.1016/j.jhazmat.2022.129147"
"   Qi X, 2021, J HAZARD MATER, V401, DOI 10.1016/j.jhazmat.2020.123823"
"   Qi Xin Qi Xin, 2018, Journal of Agro-Environment Science, V37, P1683"
"   Reddy N, 2015, TRENDS BIOTECHNOL, V33, P362, DOI 10.1016/j.tibtech.2015.03.008"
"   Saravanan A, 2023, ENVIRON RES, V236, DOI 10.1016/j.envres.2023.116723"
"   Schommer VA, 2023, SCI TOTAL ENVIRON, V881, DOI 10.1016/j.scitotenv.2023.163385"
"   Shentu J, 2023, ECOTOX ENVIRON SAFE, V256, DOI 10.1016/j.ecoenv.2023.114870"
"   Struzak M, 2024, J ENVIRON ENG, V150, DOI 10.1061/JOEEDU.EEENG-7487"
"   Tang JY, 2020, SCI TOTAL ENVIRON, V701, DOI 10.1016/j.scitotenv.2019.134751"
"   Taraqqi-A-Kamal A, 2021, PLANT SOIL ENVIRON, V67, P183, DOI 10.17221/544/2020-PSE"
"   Tu C, 2020, ENVIRON INT, V137, DOI 10.1016/j.envint.2020.105576"
"   Rehman Z, 2023, SCI TOTAL ENVIRON, V867, DOI 10.1016/j.scitotenv.2023.161468"
"   Vardhan KH, 2019, J MOL LIQ, V290, DOI 10.1016/j.molliq.2019.111197"
"   Velkova Z, 2018, ENG LIFE SCI, V18, P871, DOI 10.1002/elsc.201800017"
"   Wang LJ, 2023, ECOL ENG, V194, DOI 10.1016/j.ecoleng.2023.107034"
"   Wang Y, 2022, BIORESOURCE TECHNOL, V348, DOI 10.1016/j.biortech.2022.126818"
"   Wu CC, 2022, ENVIRON RES, V212, DOI 10.1016/j.envres.2022.113226"
"   Wu P, 2021, J HAZARD MATER, V416, DOI 10.1016/j.jhazmat.2021.125915"
"   Xie J, 2008, ENVIRON MONIT ASSESS, V146, P473, DOI 10.1007/s10661-008-0250-5"
"   Yaashikaa PR, 2022, ENVIRON POLLUT, V301, DOI 10.1016/j.envpol.2022.119035"
"   Yang X, 2023, CHEM BIOL TECHNOL AG, V10, DOI 10.1186/s40538-023-00516-2"
"   Yang ZH, 2025, BIOCHAR, V7, DOI 10.1007/s42773-024-00395-2"
"   Yuan JH, 2011, SOIL USE MANAGE, V27, P110, DOI 10.1111/j.1475-2743.2010.00317.x"
"   Zhai QX, 2014, APPL ENVIRON MICROB, V80, P4063, DOI 10.1128/AEM.00762-14"
"   Zhang Y, 2024, SUSTAINABILITY-BASEL, V16, DOI 10.3390/su16031001"
"   Zheng XJ, 2024, ECOTOX ENVIRON SAFE, V284, DOI 10.1016/j.ecoenv.2024.116883"
"   Zhou FW, 2018, SCI TOTAL ENVIRON, V633, P776, DOI 10.1016/j.scitotenv.2018.03.217"
"   Zhu GX, 2024, MICROORGANISMS, V12, DOI 10.3390/microorganisms12071406"
"   Zhu GX, 2022, PLANT PHYSIOL BIOCH, V184, P40, DOI 10.1016/j.plaphy.2022.05.013"
"   [????????? Zhu Hengliang], 2014, [???????????????, Earth and Environment], V42, P505"
"   Zimmerman Amanda Jo, 2010, Int J Anal Chem, V2010, P387803, DOI 10.1155/2010/387803"
NR 73
TC 1
Z9 2

U1 37
U2 40
PU ELSEVIER SCIENCE SA
PI LAUSANNE
"PA PO BOX 564, 1001 LAUSANNE, SWITZERLAND"
SN 1385-8947
EI 1873-3212
J9 CHEM ENG J
JI Chem. Eng. J.
PD OCT 15
PY 2025
VL 522
AR 167304
DI 10.1016/j.cej.2025.167304
EA AUG 2025
PG 12
"WC Engineering, Environmental; Engineering, Chemical"
WE Science Citation Index Expanded (SCI-EXPANDED)
SC Engineering
GA 6TX5A
UT WOS:001561926200019
DA 2026-04-06
ER

PT J
"AU Lin, H"
"   Zhou, MY"
"   Li, B"
"   Dong, YB"
"AF Lin, Hai"
"   Zhou, Mengying"
"   Li, Bing"
"   Dong, Yingbo"
"TI Mechanisms, application advances and future perspectives of"
   microbial-induced heavy metal precipitation: A review
SO INTERNATIONAL BIODETERIORATION & BIODEGRADATION
LA English
DT Review
DE Biomineralization; Bioremediation; Heavy metals; Mechanism;
   Microorganism; Influencing factors
ID ACID-MINE DRAINAGE; CARBONATE PRECIPITATION; CONTAMINATED SOIL;
   HEXAVALENT CHROMIUM; BIO-MINERALIZATION; AQUEOUS-SOLUTION;
   BIOMINERALIZATION; BIOREMEDIATION; REMEDIATION; UREASE
"AB Heavy metal pollution is insidious, long-term and irreversible, which seriously endangers the environment and human health. Compared with physical and chemical remediation techniques, microbial mineralization tech-nology has been widely studied in the remediation of heavy metal contaminated soil and water due to wide occurrence. This paper mainly reviews the mechanisms, application examples and influencing factors of microbial-induced carbonate precipitation (MICP), microbial-induced phosphate precipitation (MIPP), and microbial-induced sulfide precipitation (MISP). It was found that MICP is more widely studied, but the miner-alization products formed by the MIPP process are more stable in the natural environment. Besides, MISP is more suitable for use in anaerobic environments. In addition to various types of bacteria, the biomineralization effects and mechanisms of algae which are more environmentally friendly and fungi which are more abundant and have better tolerance to heavy metals should be intensively studied subsequently. We also summarized the recent research progress of these three mechanisms for biomineralization of heavy metals and radionuclides and analyzed the influencing factors including microbial characteristics, environmental factors, and engineering application processes. Finally, the subsequent research directions that need to be continued in depth for the remediation of heavy metal pollution by microbial mineralization were proposed, so as to promote the large-scale application of microbial mineralization technology in the remediation of heavy metal-contaminated soil and water bodies."
"C1 [Lin, Hai; Zhou, Mengying; Li, Bing; Dong, Yingbo] Univ Sci & Technol Beijing, Sch Energy & Environm Engn, Beijing 100083, Peoples R China."
"   [Lin, Hai; Li, Bing; Dong, Yingbo] Beijing Key Lab Resource oriented Treatment Ind Po, Beijing 100083, Peoples R China."
C3 University of Science & Technology Beijing
"RP Li, B; Dong, YB (corresponding author), Univ Sci & Technol Beijing, Sch Energy & Environm Engn, Beijing 100083, Peoples R China."
EM ybdong@ustb.edu.cn
FU National Natural Science Foundation of China [51874018]; Major Science
   and Technology Program for Water Pollution Control and Treatment of
   China [2015ZX07205003]; Fundamental Research Funds for the Central
   Universities [FRFMP-20-35]
FX We are grateful for grants from the National Natural Science Foundation
"   of China (51874018) , the Major Science and Technology Program for Water"
"   Pollution Control and Treatment of China (2015ZX07205003) , and the"
   Fundamental Research Funds for the Central Universities (FRFMP-20-35) .
"CR Achal V, 2012, J MICROBIOL BIOTECHN, V22, P244, DOI 10.4014/jmb.1108.08033"
"   Achal V, 2012, J HAZARD MATER, V201, P178, DOI 10.1016/j.jhazmat.2011.11.067"
"   Almajed A, 2021, CRYSTALS, V11, DOI 10.3390/cryst11040370"
"   Anbu P, 2016, SPRINGERPLUS, V5, DOI 10.1186/s40064-016-1869-2"
"   Aoki M, 2020, DESALIN WATER TREAT, V194, P172, DOI 10.5004/dwt.2020.25843"
"   Ayangbenro AS, 2018, FRONT MICROBIOL, V9, DOI 10.3389/fmicb.2018.01986"
"   Ayangbenro AS, 2017, INT J ENV RES PUB HE, V14, DOI 10.3390/ijerph14010094"
"   Bai H, 2021, INT BIODETER BIODEGR, V165, DOI 10.1016/j.ibiod.2021.105311"
"   Bhattacharya A, 2019, J ENVIRON SCI HEAL A, V54, P526, DOI 10.1080/10934529.2019.1567184"

"   Bhattacharya A, 2018, J ENVIRON MANAGE, V215, P143, DOI 10.1016/j.jenvman.2018.03.055"
"   Brookshaw DR, 2012, MINERAL MAG, V76, P777, DOI 10.1180/minmag.2012.076.3.25"
"   Catelani T, 2018, GEOCHIM COSMOCHIM AC, V222, P642, DOI 10.1016/j.gca.2017.11.013"
"   Chandwadkar P, 2018, METALLOMICS, V10, P1078, DOI 10.1039/c8mt00061a"
"   Chen L, 2022, SCI TOTAL ENVIRON, V835, DOI 10.1016/j.scitotenv.2022.155441"
"   Chen X, 2021, WATER AIR SOIL POLL, V232, DOI 10.1007/s11270-021-05206-z"
"   Chen Z, 2021, J HAZARD MATER, V408, DOI 10.1016/j.jhazmat.2020.124973"
"   Cheng YJ, 2016, GEOMICROBIOL J, V33, P948, DOI 10.1080/01490451.2015.1137660"
"   Cheung KH, 2007, INT BIODETER BIODEGR, V59, P8, DOI 10.1016/j.ibiod.2006.05.002"
"   Cuaxinque-Flores G, 2020, SCI TOTAL ENVIRON, V724, DOI 10.1016/j.scitotenv.2020.138124"
"   Dean J.A., 1998, Lange's Handbook of Chemistry, V15th, p5.165, DOI [DOI 10.1080/10426919008953291, 10.1080/10426919008953291, DOI 10.1080/1042691900895329]"
"   Demers I, 2017, MINER ENG, V107, P43, DOI 10.1016/j.mineng.2016.11.017"
"   Deng LY, 2022, J CLEAN PROD, V340, DOI 10.1016/j.jclepro.2022.130741"
"   Dhami NK, 2016, ECOL ENG, V94, P443, DOI 10.1016/j.ecoleng.2016.06.013"
"   Do HT, 2020, ENVIRON POLLUT, V260, DOI 10.1016/j.envpol.2020.114051"
"   Do HT, 2020, ECOTOX ENVIRON SAFE, V198, DOI 10.1016/j.ecoenv.2020.110672"
"   Dong YH, 2019, CRYSTALS, V9, DOI 10.3390/cryst9120633"
"   Duarte-Nass C, 2020, J ENVIRON MANAGE, V256, DOI 10.1016/j.jenvman.2019.109938"
"   Edelstein M, 2018, SCI HORTIC-AMSTERDAM, V234, P431, DOI 10.1016/j.scienta.2017.12.039"
"   Eltarahony M, 2020, SCI REP-UK, V10, DOI 10.1038/s41598-020-60951-1"
"   Environmental Engineering Guangdong Technion Israel Institute of Technology 241 Daxue Road Shantou Guangdong 515063 China, 2021, Applied Environmental Biotechnology, V5, P3, DOI [10.26789/aeb.2020.02.002, 10.26789/AEB.2020.02.002, DOI 10.26789/AEB.2020.02.002]"
"   Fan YJ, 2020, ADV POWDER TECHNOL, V31, P4095, DOI 10.1016/j.apt.2020.08.014"
"   Fang LY, 2021, SCI TOTAL ENVIRON, V787, DOI 10.1016/j.scitotenv.2021.147627"
"   Fathollahzadeh H, 2019, APPL MICROBIOL BIOT, V103, P1043, DOI 10.1007/s00253-018-9526-z"
"   Fern??ndez PM, 2018, CHEMOSPHERE, V208, P139, DOI 10.1016/j.chemosphere.2018.05.166"
"   Gao YQ, 2022, ACTA GEOTECH, V17, P3799, DOI 10.1007/s11440-022-01489-6"
"   Gerber U, 2018, PLOS ONE, V13, DOI 10.1371/journal.pone.0201903"
"   Ghorbanzadeh N, 2020, ARID LAND RES MANAG, V34, P319, DOI 10.1080/15324982.2020.1720866"
"   Gorospe CM, 2013, BIOTECHNOL BIOPROC E, V18, P903, DOI 10.1007/s12257-013-0030-0"
"   Govarthanan M, 2019, ECOTOX ENVIRON SAFE, V174, P699, DOI 10.1016/j.ecoenv.2019.03.034"
"   Gu S, 2020, SUSTAINABILITY-BASEL, V12, DOI 10.3390/su12031263"
"   Gupta P, 2017, WORLD J MICROB BIOT, V33, DOI 10.1007/s11274-016-2176-3"
"   Hamdan N, 2017, GEOMICROBIOL J, V34, P139, DOI 10.1080/01490451.2016.1154117"
"   Han LJ, 2020, SCI TOTAL ENVIRON, V746, DOI 10.1016/j.scitotenv.2020.140967"
"   He J, 2019, INT BIODETER BIODEGR, V140, P67, DOI 10.1016/j.ibiod.2019.03.012"
"   He N, 2022, J HAZARD MATER, V422, DOI 10.1016/j.jhazmat.2021.126902"
"   Hu L, 2021, WATER RES, V190, DOI 10.1016/j.watres.2020.116753"
"   Jaishankar Monisha, 2014, Interdiscip Toxicol, V7, P60, DOI 10.2478/intox-2014-0009"
"   Jiang LH, 2020, ECOTOX ENVIRON SAFE, V191, DOI 10.1016/j.ecoenv.2019.110009"
"   Jiang NJ, 2019, SCI TOTAL ENVIRON, V672, P722, DOI 10.1016/j.scitotenv.2019.03.294"
"   Jiang YF, 2020, METABOLITES, V10, DOI 10.3390/metabo10110441"
"   Kang CH, 2014, APPL BIOCHEM BIOTECH, V172, P1929, DOI 10.1007/s12010-013-0626-z"
"   Khadim HJ, 2019, ENVIRON TECHNOL INNO, V14, DOI 10.1016/j.eti.2019.100315"
"   Kim JH, 2019, J MATER CYCLES WASTE, V21, P239, DOI 10.1007/s10163-018-0779-5"
"   Kimber RL, 2020, APPL ENVIRON MICROB, V86, DOI 10.1128/AEM.00967-20"
"   Krajewska B, 2018, J ADV RES, V13, P59, DOI 10.1016/j.jare.2017.10.009"
"   Krause S, 2018, GEOCHIM COSMOCHIM AC, V243, P116, DOI 10.1016/j.gca.2018.09.018"
"   Li F, 2018, J HAZARD MATER, V358, P178, DOI 10.1016/j.jhazmat.2018.06.066"
"   Li M, 2016, J ENVIRON ENG, V142, DOI 10.1061/(ASCE)EE.1943-7870.0000970"
"   Li M, 2013, INT BIODETER BIODEGR, V76, P81, DOI 10.1016/j.ibiod.2012.06.016"
"   Li QW, 2019, ENVIRON SCI-NANO, V6, P1866, DOI 10.1039/c9en00385a"
"   Li QW, 2014, ENVIRON SCI TECHNOL, V48, P14409, DOI 10.1021/es5042546"
"   Li WL, 2022, SCI TOTAL ENVIRON, V802, DOI 10.1016/j.scitotenv.2021.149802"
"   Lin CY, 2018, GEOCHIM COSMOCHIM AC, V237, P184, DOI 10.1016/j.gca.2018.06.019"
"   Liu FX, 2021, CURR BIOL, V31, P358, DOI 10.1016/j.cub.2020.10.044"
"   Liu LW, 2018, SCI TOTAL ENVIRON, V633, P206, DOI 10.1016/j.scitotenv.2018.03.161"
"   Liu P, 2021, J MATER CIVIL ENG, V33, DOI 10.1061/(ASCE)MT.1943-5533.0003691"
"   Liu P, 2021, BIOCHEM ENG J, V166, DOI 10.1016/j.bej.2020.107856"
"   Lopez-Fernandez M, 2021, MICROB BIOTECHNOL, V14, P810, DOI 10.1111/1751-7915.13718"
"   Luo J, 2018, CONSTR BUILD MATER, V164, P275, DOI 10.1016/j.conbuildmat.2017.12.233"
"   Ma N, 2021, J HAZARD MATER, V417, DOI 10.1016/j.jhazmat.2021.126102"
"   Ma N, 2021, ENV MICROBIOL REP, V13, P325, DOI 10.1111/1758-2229.12933"
"   Ma N, 2021, ENVIRON MICROBIOL, V23, P934, DOI 10.1111/1462-2920.15205"
"   Maity JP, 2019, GEOMICROBIOL J, V36, P612, DOI 10.1080/01490451.2019.1597216"
"   Martinez RJ, 2014, PLOS ONE, V9, DOI 10.1371/journal.pone.0100383"
"   Meister P, 2018, PLOS ONE, V13, DOI 10.1371/journal.pone.0207305"
"   Meng L, 2020, CHEMOSPHERE, V259, DOI 10.1016/j.chemosphere.2020.127507"
"   Moore J., 2014, CHEM MOL SCI"
"   Morcillo F, 2014, PLOS ONE, V9, DOI 10.1371/journal.pone.0091305"
"   Niedermeier M, 2018, J PLANT PHYSIOL, V230, P80, DOI 10.1016/j.jplph.2018.08.008"
"   Nogueira EW, 2021, BIORESOURCE TECHNOL, V330, DOI 10.1016/j.biortech.2021.124968"
"   Ojuederie OB, 2017, INT J ENV RES PUB HE, V14, DOI 10.3390/ijerph14121504"
"   Oshiro H, 2018, ENVIRON EARTH SCI, V77, DOI 10.1007/s12665-018-7775-x"
"   Park I, 2019, CHEMOSPHERE, V219, P588, DOI 10.1016/j.chemosphere.2018.11.053"
"   Park Y, 2022, CHEMPLUSCHEM, V87, DOI 10.1002/cplu.202100457"
"   Pasquale V, 2019, INT BIODETER BIODEGR, V140, P57, DOI 10.1016/j.ibiod.2019.03.005"
"   Qian CX, 2018, AIP ADV, V8, DOI 10.1063/1.5040730"
"   Qian CX, 2016, J CHIN CHEM SOC-TAIP, V63, P635, DOI 10.1002/jccs.201600002"
"   Qian XY, 2017, J CLEAN PROD, V164, P198, DOI 10.1016/j.jclepro.2017.06.195"
"   Rahman Z, 2020, ENVIRON SCI POLLUT R, V27, P27563, DOI 10.1007/s11356-020-08903-0"
"   S??nchez-Castro I, 2021, J ENVIRON MANAGE, V296, DOI 10.1016/j.jenvman.2021.113411"

"   S??nchez-Castro I, 2020, WATER RES, V183, DOI 10.1016/j.watres.2020.116110"
"   Sch??tze E, 2013, BIOGEOSCIENCES, V10, P3605, DOI 10.5194/bg-10-3605-2013"
"   Seifan M, 2019, APPL MICROBIOL BIOT, V103, P4693, DOI 10.1007/s00253-019-09861-5"
"   Sep??lveda S, 2021, MINERALS-BASEL, V11, DOI 10.3390/min11080905"
"   Sevak PI, 2021, ENVIRON CHEM LETT, V19, P4463, DOI 10.1007/s10311-021-01296-7"
"   Shan B, 2021, ENVIRON SCI POLLUT R, V28, P30486, DOI 10.1007/s11356-021-14045-8"
"   Shar SS, 2019, METALS-BASEL, V9, DOI 10.3390/met9040407"
"   Sharma J, 2018, J ENVIRON MANAGE, V217, P754, DOI 10.1016/j.jenvman.2018.04.027"
"   Shen YH, 2018, APPL MICROBIOL BIOT, V102, P4217, DOI 10.1007/s00253-018-8918-4"
"   Shiraishi F, 2020, GEOCHIM COSMOCHIM AC, V285, P55, DOI 10.1016/j.gca.2020.06.033"
"   Song MZ, 2022, CHEMOSPHERE, V290, DOI 10.1016/j.chemosphere.2021.133229"
"   Sousa T, 2013, METALLOMICS, V5, P390, DOI 10.1039/c3mt00052d"
"   Su Z, 2022, SCI TOTAL ENVIRON, V825, DOI 10.1016/j.scitotenv.2022.153767"
"   Sui M, 2018, IOP C SER EARTH ENV, V208, DOI 10.1088/1755-1315/208/1/012112"
"   Sun LZ, 2020, ECOTOX ENVIRON SAFE, V201, DOI 10.1016/j.ecoenv.2020.110810"
"   Suyamud B, 2020, ENVIRON MICROBIOL, V22, P1588, DOI 10.1111/1462-2920.14949"
"   Tan JQ, 2022, J HAZARD MATER, V423, DOI 10.1016/j.jhazmat.2021.127007"
"   Tang F, 2022, J HAZARD MATER, V439, DOI 10.1016/j.jhazmat.2022.129675"
"   Tarver S, 2019, INT BIODETER BIODEGR, V143, DOI 10.1016/j.ibiod.2019.104724"
"   Tayang A, 2021, CURR SCI INDIA, V120, P1013, DOI 10.18520/cs/v120/i6/1013-1025"
"   Tu H, 2019, NUCL ENG TECHNOL, V51, P1322, DOI 10.1016/j.net.2019.03.002"
"   Vassilev N, 2006, APPL MICROBIOL BIOT, V71, P137, DOI 10.1007/s00253-006-0380-z"
"   Wang ML, 2021, J HAZARD MATER, V412, DOI 10.1016/j.jhazmat.2021.125261"
"   Wu Y, 2021, MICROORGANISMS, V9, DOI 10.3390/microorganisms9112396"
"   Wu ZZ, 2021, J HAZARD MATER, V405, DOI 10.1016/j.jhazmat.2020.124255"
"   Xu SZ, 2021, APPL MICROBIOL BIOT, V105, P2171, DOI 10.1007/s00253-021-11155-8"
"   Xu SZ, 2019, 3 BIOTECH, V9, DOI 10.1007/s13205-019-1649-0"
"   Xue ZF, 2022, J HAZARD MATER, V426, DOI 10.1016/j.jhazmat.2021.128090"
"   Yan X, 2021, ENVIRON SCI POLLUT R, V28, P22359, DOI 10.1007/s11356-020-11852-3"
"   Ye BH, 2018, BIORESOURCE TECHNOL, V264, P206, DOI 10.1016/j.biortech.2018.05.066"
"   Yin TT, 2021, J HAZARD MATER, V419, DOI 10.1016/j.jhazmat.2021.126479"
"   Yin TT, 2021, J HAZARD MATER, V401, DOI 10.1016/j.jhazmat.2020.123269"
"   Yu QH, 2022, J HAZARD MATER, V424, DOI 10.1016/j.jhazmat.2021.127758"
"   Yu XN, 2021, CONSTR BUILD MATER, V273, DOI 10.1016/j.conbuildmat.2020.121113"
"   Yu XN, 2020, ECOTOX ENVIRON SAFE, V191, DOI 10.1016/j.ecoenv.2020.110224"
"   Yu XN, 2019, J CLEAN PROD, V240, DOI 10.1016/j.jclepro.2019.118156"
"   Yu XN, 2019, J ENVIRON MANAGE, V245, P447, DOI 10.1016/j.jenvman.2019.05.091"
"   Zamora-Ledezma C, 2021, ENVIRON TECHNOL INNO, V22, DOI 10.1016/j.eti.2021.101504"
"   Zeng GQ, 2021, J HAZARD MATER, V412, DOI 10.1016/j.jhazmat.2021.125156"
"   Zhan QW, 2016, ELECTRON J BIOTECHN, V19, P29, DOI 10.1016/j.ejbt.2015.11.003"
"   Zhang J, 2018, CHEMOSPHERE, V206, P682, DOI 10.1016/j.chemosphere.2018.04.181"
"   Zhang KJ, 2022, J HAZARD MATER, V421, DOI 10.1016/j.jhazmat.2021.126765"
"   Zhang KJ, 2021, J HAZARD MATER, V416, DOI 10.1016/j.jhazmat.2021.125800"
"   Zhang KJ, 2020, CHEMOSPHERE, V241, DOI 10.1016/j.chemosphere.2019.125041"
"   Zhang KJ, 2019, CHEMOSPHERE, V224, P272, DOI 10.1016/j.chemosphere.2019.02.140"
"   Zhao WW, 2020, J CLEAN PROD, V260, DOI 10.1016/j.jclepro.2020.120980"
"   Zhao XM, 2019, J HAZARD MATER, V368, P133, DOI 10.1016/j.jhazmat.2019.01.029"
"   Zhao XQ, 2019, INT J ENV RES PUB HE, V16, DOI 10.3390/ijerph16020268"
"   Zhao Y, 2015, BIORESOURCE TECHNOL, V186, P52, DOI 10.1016/j.biortech.2015.02.017"
"   Zhao Y, 2017, ENVIRON SCI POLLUT R, V24, P372, DOI 10.1007/s11356-016-7810-y"
"   Zhao ZJ, 2020, GEOMICROBIOL J, V37, P764, DOI 10.1080/01490451.2020.1774687"
"   Zheng LG, 2019, ENVIRON POLLUT, V251, P484, DOI 10.1016/j.envpol.2019.04.131"
"   Zheng XY, 2018, CHEMOSPHERE, V203, P109, DOI 10.1016/j.chemosphere.2018.03.165"
"   Zheng YT, 2021, WORLD J MICROB BIOT, V37, DOI 10.1007/s11274-021-03176-2"
"   Zhou XF, 2021, ENVIRON TECHNOL INNO, V23, DOI 10.1016/j.eti.2021.101626"
"   Zhu TT, 2016, FRONT BIOENG BIOTECH, V4, DOI 10.3389/fbioe.2016.00004"
"   Zhu XJ, 2016, ENVIRON POLLUT, V219, P149, DOI 10.1016/j.envpol.2016.10.047"
NR 147
TC 100
Z9 118
U1 31
U2 380
PU ELSEVIER SCI LTD
PI London
"PA 125 London Wall, London, the United Kingdom"
SN 0964-8305
EI 1879-0208
J9 INT BIODETER BIODEGR
JI Int. Biodeterior. Biodegrad.
PD MAR
PY 2023
VL 178
AR 105544
DI 10.1016/j.ibiod.2022.105544
EA DEC 2022
PG 15
WC Biotechnology & Applied Microbiology; Environmental Sciences
WE Science Citation Index Expanded (SCI-EXPANDED)
SC Biotechnology & Applied Microbiology; Environmental Sciences & Ecology
GA 8U1VO
UT WOS:000929739700005
DA 2026-04-06
ER

PT J
"AU Sumathi, S"
"   Priyanka, V"
"   Krishnapriya, V"
"   Suganya, K"
"AF Sumathi, S."
"   Priyanka, V"
"   Krishnapriya, V"
"   Suganya, K."
TI Identification of a novel strain of fungus Kalmusia italica from
   untouched marine soil and its heavy metal tolerance activity
SO BIOREMEDIATION JOURNAL
LA English
DT Article
DE Biosorption; fungus; heavy metals; Kalmusia italica; marine soil
"AB Heavy metal pollution is one of the most important environmental problems. Heavy metal discharge to the atmosphere has increased currently because of industrial activity and technological growth and creates serious problems for the climate, soil, and water supplies. Many conventional techniques were used for heavy metals separation and treatment. Micro-organisms play a significant role in the bioremediation of polluted soil and waste products from heavy metals. The most common heavy metal tolerant microbes are fungi and yeast. Fungal isolates are potential microbes that are tolerant of heavy metals. The research aims to isolate and classify the fungal species collected from the soil. The fungal isolates identified were tested for their biosorption property and the tolerance index of stressed strains was investigated by examining the minimum inhibitory concentration (MIC). The ability of the strain to remove heavy metals was investigated. Fungal isolates were molecularly identified using 18S rRNA sequencing. For the isolated fungus, BLAST and the phylogenetic trees were built and were known as Kalmusia italica. The effect of the isolates on the growth in the presence of heavy metals was determined by measuring the colony extension radius against control. The results showed that fungus was tolerant to a higher concentration of heavy metals and was able to absorb the heavy metals. MIC determination is indicative of the fact that the levels of tolerance to individual metals depend on the type of fungus. Biological treatment based on living fungi, which is cheaper and more environmentally friendly, has emerged as a good alternative technique to existing conventional methods for metal removal."
"C1 [Sumathi, S.; Priyanka, V; Krishnapriya, V; Suganya, K.] Avinashilingam Inst Home Sci & Higher Educ Women, Dept Biochem Biotechnol & Bioinformat, Coimbatore, Tamil Nadu, India."
C3 Avinashilingam University for Women
"RP Sumathi, S (corresponding author), Avinashilingam Inst Home Sci & Higher Educ Women, Dept Biochem Biotechnol & Bioinformat, Coimbatore, Tamil Nadu, India."
EM sumii.venkat@gmail.com
"RI Sundaravadivelu, Sumathi/ABF-2196-2020; Kanagaraj, Suganya/KGM-4893-2024"
"OI Kanagaraj, Suganya/0000-0001-9249-3364"
"CR Ahsan T, 2017, BIOTECHNOL BIOTEC EQ, V31, P91, DOI 10.1080/13102818.2016.1259016"
"   Ayad F, 2018, J PURE APPL MICROBIO, V12, P855, DOI 10.22207/JPAM.12.2.48"
"   Ayangbenro AS, 2017, INT J ENV RES PUB HE, V14, DOI 10.3390/ijerph14010094"
"   Ayres R.U., 2013, The Life Cycle of Copper, Its Co-Products and Byproducts, V13"
"   Azimi A, 2017, CHEMBIOENG REV, V4, P37, DOI 10.1002/cben.201600010"
"   Bajgai Y, 2015, NUTR CYCL AGROECOSYS, V101, P1, DOI 10.1007/s10705-014-9656-3"
"   Carolin CF, 2017, J ENVIRON CHEM ENG, V5, P2782, DOI 10.1016/j.jece.2017.05.029"
"   Congeevaram S, 2007, J HAZARD MATER, V146, P270, DOI 10.1016/j.jhazmat.2006.12.017"
"   Dirbaba NB, 2018, WATER-SUI, V10, DOI 10.3390/w10050535"
"   Domka A, 2019, J BASIC MICROB, V59, P24, DOI 10.1002/jobm.201800406"
"   Fern??ndez PM, 2018, CHEMOSPHERE, V208, P139, DOI 10.1016/j.chemosphere.2018.05.166"
"   Santana JKG, 2018, CIENC RURAL, V48, DOI 10.1590/0103-8478cr20180071"
"   Gul H, 2018, Environmental Contaminants Reviews, V1, P04, DOI [10.26480/ecr.02.2018.04.08, 10.26480/ecr.02.2018.04.08, DOI 10.26480/ECR.02.2018.04.08]"
"   Guney M, 2010, ENVIRON MONIT ASSESS, V164, P101, DOI 10.1007/s10661-009-0878-9"
"   Gupta Pratima, 2017, Biotechnol Rep (Amst), V13, P58, DOI [10.1016/j.btre.2016.12.006, 10.1016/j.btre.2016.12.006]"
"   Gururajan K, 2018, ENVIRON TECHNOL INNO, V9, P91, DOI 10.1016/j.eti.2017.11.001"
"   Hua M, 2012, J HAZARD MATER, V211, P317, DOI 10.1016/j.jhazmat.2011.10.016"
"   Iram S, 2015, ARAB J SCI ENG, V40, P1867, DOI 10.1007/s13369-015-1702-1"
"   Johney, 2018, LIFE SCI INFORMATICS, V4, P209, DOI [10.26479/2018.0405.16, DOI 10.26479/2018.0405.16]"
"   Kan GF, 2019, MICROBIOLOGYOPEN, V8, DOI 10.1002/mbo3.657"
"   Kumar S.S., 2017, Plant-microbe Interactions in Agro-Ecological Perspectives. microbial interactions and agro-ecological impacts, P367"
"   Laskar MA, 2016, J SUSTAIN DEV ENERGY, V4, P320, DOI 10.13044/j.sdewes.2016.04.0025"
"   Lawrence DP, 2018, PLANT DIS, V102, P1081, DOI [10.1094/PDIS-08-17-1247-RE, 10.1094/pdis-08-17-1247-re]"
"   Lep??re C, 2019, PEERJ, V7, DOI 10.7717/peerj.6247"
"   Marzan LW, 2017, EGYPT J AQUATIC RES, V43, P65, DOI [10.1016/j.ejar.2016.11.002, 10.1016/j.ejar.2016.11.002, DOI 10.1016/j.ejar.2016.11.002, DOI 10.1016/J.EJAR.2016.11.002]"
"   Mohammadian E, 2017, CHEMOSPHERE, V185, P290, DOI 10.1016/j.chemosphere.2017.07.022"
"   Nayak N, 2018, Research & Reviews: A Journal of Biotechnology, V8, P16, DOI [10.37591/(rrjobt).v8i1.111, 10.37591/(rrjobt).v8i1.111, DOI 10.37591/(RRJOBT).V8I1.111]"
"   Oladipo OG, 2018, BRAZ J MICROBIOL, V49, P29, DOI 10.1016/j.bjm.2017.06.003"
"   Panzer K, 2015, PLOS ONE, V10, DOI 10.1371/journal.pone.0134377"
"   Plum LM, 2010, INT J ENV RES PUB HE, V7, P1342, DOI 10.3390/ijerph7041342"
"   Prakash A, 2018, MYCOSES, V61, P305, DOI 10.1111/myc.12741"
"   Rafiq A, 2018, INT J PHARM SCI RES, V9, P1002, DOI 10.13040/IJPSR.0975-8232.9(3).1002-11"
"   Rose PK, 2018, Beni-Suef University Journal of Basic and Applied Sciences, V7, P688, DOI [10.1016/j.bjbas.2018.08.001, 10.1016/j.bjbas.2018.08.001, DOI 10.1016/J.BJBAS.2018.08.001]"
"   GS, 2017, JOURNAL OF ADVANCES IN CHEMISTRY, V13, P6018, DOI [10.24297/jac.v13i11.5770, 10.24297/jac.v13i11.5770, DOI 10.24297/JAC.V13I11.5770]"
"   Saba G, 2017, International Research Journal of Pharmacy, V8, P120, DOI [10.7897/2230-8407.086108, 10.7897/2230-8407.086108, DOI 10.7897/2230-8407.086108]"

"   Shafiquzzaman Siddiquee Shafiquzzaman Siddiquee, 2013, Advances in Bioscience and Biotechnology, V4, P570, DOI 10.4236/abb.2013.44075"
"   Sun ST, 2018, SCI REP-UK, V8, DOI 10.1038/s41598-018-23255-z"
"   Yarza P, 2017, MAR GENOM, V36, P33, DOI 10.1016/j.margen.2017.05.009"
NR 38
TC 5
Z9 7
U1 1
U2 22
PU TAYLOR & FRANCIS INC
PI PHILADELPHIA
"PA 530 WALNUT STREET, STE 850, PHILADELPHIA, PA 19106 USA"
SN 1088-9868
EI 1547-6529
J9 BIOREMEDIAT J
JI Bioremediat. J.
PD APR 3
PY 2021
VL 25
IS 2
BP 91
EP 107
DI 10.1080/10889868.2020.1853029
EA NOV 2020
PG 17
"WC Engineering, Environmental; Environmental Sciences"
WE Science Citation Index Expanded (SCI-EXPANDED)
SC Engineering; Environmental Sciences & Ecology
GA TY9DQ
UT WOS:000598212100001
DA 2026-04-06
ER

PT J
"AU Zuo, YY"
"   Li, Y"
"   Chen, H"
"   Ran, G"
"   Liu, XM"
"AF Zuo, Yingying"
"   Li, Ying"
"   Chen, Hu"
"   Ran, Gang"
"   Liu, Xiuming"
TI Effects of multi-heavy metal composite pollution on microorganisms
"   around a lead-zinc mine in typical karst areas, southwest China"
SO ECOTOXICOLOGY AND ENVIRONMENTAL SAFETY
LA English
DT Article
DE Karst areas; Lead-zinc mines; Heavy metal pollution; Bacterial diversity
ID MICROBIAL COMMUNITY STRUCTURE; SURFACE-WATER; SOIL; DIVERSITY;
   CONTAMINATION; RHIZOSPHERE; GROUNDWATER; RESISTANCE; SYSTEMS; CARBON
"AB Heavy metal pollution poses a serious hazard to the soil bacterial community. The purpose of this study is to understand the characteristics of soil heavy metal pollution in lead-zinc mines in karst areas and the response of Pb, Zn, Cd, and As-induced composite pollution to soil microorganisms. This paper selected soil samples from the lead-zinc mining area of Xiangrong Mining Co., Ltd., Puding County, Guizhou Province, China. The soil in the mining area is contaminated by multiple heavy metals such as Pb, Zn, Cd and As. The average levels of Pb, Zn, Cd and As in the Pb-Zn mining soil were 14.5, 7.8, 5.5 and 4.4 times higher than the soil background in this area, respectively. Bacterial community structures and functions were analyzed using 16 S rRNA high-throughput sequencing technology and the PICRUSt method. A total of 19 bacterial phyla, 34 classes and 76 orders were detected in the tested soil. At the phylum level, the Proteobacteria are the dominant flora of the soil in the tailings reservoir area of the lead-zinc mine, respectively GWK1 (49.64%), GWK2 (81.89%), GWK3 (95.16%); and for the surrounding farmland soil, the Proteobacteria, Actinobacteriota, Acidobacteriota, Chloroflexi and Firmicutes are the most abundant in five bacterial groups. RDA analyses revealed that the heavy metal pollution of the lead-zinc mining area has a significant impact on the diversity of soil microorganisms. As the distance from the mining area increased, the heavy metal comprehensive pollution and potential risk value decreased, and the bacterial diversity increased. Additionally, various types of heavy metals have different effects on bacterial communities, and soil heavy metal content will also change the bacterial community structure. Proteobacteria positively related to Pb, Cd, and Zn, therefore, Proteobacteria were highly resistant to heavy metals. PICRUSt analysis suggested that heavy metals significantly affect the metabolic function of microorganisms. Microorganisms might generate resistance and enable themselves to survive by increasing the transport of metal ions and excreting metal ions. These results can be used as a basis for the microbial remediation of heavy metal-contaminated farmland in mining areas."
"C1 [Zuo, Yingying] Liaoning Univ, Coll Chem, Shenyang 110036, Peoples R China."
"   [Zuo, Yingying; Li, Ying; Chen, Hu; Ran, Gang; Liu, Xiuming] Chinese Acad Sci, Inst Geochem, State Key Lab Environm Geochem, Guiyang 550081, Peoples R China."
"   [Zuo, Yingying; Li, Ying; Chen, Hu; Ran, Gang; Liu, Xiuming] Chinese Acad Sci, Puding Karst Ecosyst Res Stn, Puding 562100, Peoples R China."
C3 Liaoning University; Chinese Academy of Sciences; Institute of
"   Geochemistry, CAS; Chinese Academy of Sciences"
"RP Liu, XM (corresponding author), Chinese Acad Sci, Inst Geochem, State Key Lab Environm Geochem, Guiyang 550081, Peoples R China."
EM liuxiuming@vip.skleg.cn

"OI Zuo, Yingying/0009-0008-2556-8387"
FU Strategic Priority Research Program of the Chinese Academy of Science
[XDA23060102]; Key R amp; D projects of Guangxi [2020-159]; Project on
Social Development by Department of Science and Technology of Guizhou
Province [SY-2014-3041]
FX This work was supported by the Strategic Priority Research Program of
" the Chinese Academy of Science (XDA23060102), the Key R & amp; D"
" projects of Guangxi (2020-159), and the Project on Social Development by"
Department of Science and Technology of Guizhou Province (SY-2014-3041).
"CR Alsabhan AH, 2022, J KING SAUD UNIV SCI, V34, DOI 10.1016/j.jksus.2021.101671"
" Bai YE, 2019, CARBONATE EVAPORITE, V34, P45, DOI 10.1007/s13146-017-0386-x"
" Bang L, 2022, J HAZARD MATER, V425, DOI 10.1016/j.jhazmat.2021.127945"
" Chen H, 2018, FUNCT ECOL, V32, P1400, DOI 10.1111/1365-2435.13069"
" Chen QW, 2021, EUR J REMOTE SENS, V54, P405, DOI 10.1080/22797254.2020.1793688"
" Cheng XY, 2021, SCI TOTAL ENVIRON, V751, DOI 10.1016/j.scitotenv.2020.142263"
" Cui HB, 2018, AGR ECOSYST ENVIRON, V267, P165, DOI 10.1016/j.agee.2018.08.017"
" Martins PSD, 2008, BRAZ J MICROBIOL, V39, P780, DOI [10.1590/S1517-83822008000400034, 10.1590/S1517-838220080004000034]"
" Deng LJ, 2015, APPL MICROBIOL BIOT, V99, P8259, DOI 10.1007/s00253-015-6662-6"
" Deng SQ, 2018, ENVIRON POLLUT, V237, P1088, DOI 10.1016/j.envpol.2017.11.037"
" Feng G, 2018, BMC MICROBIOL, V18, DOI 10.1186/s12866-018-1152-5"
" Ghodhbane-Gtari F, 2013, GENOME ANNOUNCEMENTS, V1, DOI 10.1128/genomeA.00085-13"
" Guan HL, 2021, AGRONOMY-BASEL, V11, DOI 10.3390/agronomy11010173"
" Guo GX, 2015, APPL MICROBIOL BIOT, V99, P8765, DOI 10.1007/s00253-015-6723-x"
" Hu JJ, 2019, ENVIRON GEOCHEM HLTH, V41, P967, DOI 10.1007/s10653-018-0193-x"
" Huang LM, 2013, ENVIRON EARTH SCI, V68, P1331, DOI 10.1007/s12665-012-1831-8"
" Huang Y, 2018, J HAZARD MATER, V354, P161, DOI 10.1016/j.jhazmat.2018.05.006"
" Hur M, 2019, MICROORGANISMS, V7, DOI 10.3390/microorganisms7090357"
" Kielak AM, 2016, ARCH MICROBIOL, V198, P987, DOI 10.1007/s00203-016-1260-2"
" Kn??b M, 2018, ACTA MICROBIOL IMM H, V65, P439, DOI 10.1556/030.65.2018.002"
" Li CC, 2020, SCI TOTAL ENVIRON, V749, DOI 10.1016/j.scitotenv.2020.141555"
" Li CZ, 2021, HUM ECOL RISK ASSESS, V27, P724, DOI 10.1080/10807039.2020.1752143"
" Li DJ, 2017, FOREST ECOL MANAG, V400, P85, DOI 10.1016/j.foreco.2017.05.050"
" Li Q, 2021, FRONT MICROBIOL, V12, DOI 10.3389/fmicb.2021.636405"
" Li XQ, 2017, ENVIRON POLLUT, V231, P908, DOI 10.1016/j.envpol.2017.08.057"
" Li Y, 2014, EVID-BASED COMPL ALT, V2014, DOI 10.1155/2014/160373"
" Lin YB, 2019, ECOTOX ENVIRON SAFE, V180, P557, DOI 10.1016/j.ecoenv.2019.05.057"
" Liu C, 2020, CHEMOSPHERE, V253, DOI 10.1016/j.chemosphere.2020.126721"
" Lu AX, 2018, J GEOCHEM EXPLOR, V194, P210, DOI 10.1016/j.gexplo.2018.08.003"
" Luo LY, 2019, APPL SOIL ECOL, V139, P100, DOI 10.1016/j.apsoil.2019.03.022"
" Obasi PN, 2019, INT J ENVIRON SCI TE, V16, P7869, DOI 10.1007/s13762-019-02337-z"
" Patel Miral, 2004, Indian Journal of Experimental Biology, V42, P728"
" Prabhakaran P, 2016, RSC ADV, V6, P109862, DOI 10.1039/c6ra10966g"
" Radeva G, 2013, WATER AIR SOIL POLL, V224, DOI 10.1007/s11270-013-1748-1"
" Risue??o Y, 2020, J ENVIRON MANAGE, V266, DOI 10.1016/j.jenvman.2020.110613"
" Shen TL, 2019, SCI TOTAL ENVIRON, V646, P940, DOI 10.1016/j.scitotenv.2018.07.223"
" Silver S, 2003, FEMS MICROBIOL REV, V27, P341, DOI 10.1016/S0168-6445(03)00047-0"
" Singh BK, 2014, ENVIRON MICROBIOL, V16, P2408, DOI 10.1111/1462-2920.12353"
" Song SZ, 2022, FRONT MICROBIOL, V13, DOI 10.3389/fmicb.2022.922989"
" Sonthiphand P, 2021, J ENVIRON SCI HEAL A, V56, P769, DOI 10.1080/10934529.2021.1927421"
" Tang J, 2019, SCI TOTAL ENVIRON, V649, P1250, DOI 10.1016/j.scitotenv.2018.08.242"
" Tian XY, 2017, FRONT MICROBIOL, V8, DOI 10.3389/fmicb.2017.02288"
" Tseng SC, 2021, INT J ENV RES PUB HE, V18, DOI 10.3390/ijerph18168661"
" Vishnivetskaya TA, 2018, ENVIRON SCI-PROC IMP, V20, P673, DOI 10.1039/c7em00558j"
" Wang H, 2021, ENVIRON EARTH SCI, V80, DOI 10.1007/s12665-021-09539-0"
" Wang NN, 2018, ENVIRON SCI POLLUT R, V25, P141, DOI 10.1007/s11356-016-8159-y"
" Wang YW, 2020, AGR ECOSYST ENVIRON, V292, DOI 10.1016/j.agee.2020.106821"
" Wang YT, 2021, ENVIRON POLLUT, V276, DOI 10.1016/j.envpol.2021.116666"
" Wu D, 2018, WORLD J MICROB BIOT, V34, DOI 10.1007/s11274-018-2557-x"
" Xiang YW, 2020, WATER AIR SOIL POLL, V231, DOI 10.1007/s11270-020-04878-3"
" Xiao D, 2022, APPL SOIL ECOL, V170, DOI 10.1016/j.apsoil.2021.104227"
" Xiao EZ, 2016, APPL MICROBIOL BIOT, V100, P7751, DOI 10.1007/s00253-016-7598-1"
" Xu YL, 2019, ENVIRON INT, V125, P478, DOI 10.1016/j.envint.2019.01.071"
" Yan CC, 2020, ENVIRON RES, V189, DOI 10.1016/j.envres.2020.109869"
" Yan CC, 2020, SCI TOTAL ENVIRON, V707, DOI 10.1016/j.scitotenv.2019.136116"
" Yin K, 2019, CHEM ENG J, V360, P1553, DOI [10.1016/j.cej.2018.10.226, 10.1016/j.jhazmat.2024.134410]"
" Zhang H, 2018, ECOTOX ENVIRON SAFE, V165, P314, DOI 10.1016/j.ecoenv.2018.09.010"
" Zhang Q., 2019, OBS SW CHINA ENV EAR, V78, P1"
" Zhang QC, 2020, WATER AIR SOIL POLL, V231, DOI 10.1007/s11270-020-04728-2"
" Zhang YX, 2022, J HAZARD MATER, V424, DOI 10.1016/j.jhazmat.2021.127308"
" Zhao XQ, 2020, INT J ENV RES PUB HE, V17, DOI 10.3390/ijerph17165680"
" Zhao XQ, 2019, ECOTOX ENVIRON SAFE, V170, P218, DOI 10.1016/j.ecoenv.2018.11.136"
" Zhou XY, 2021, INT J ENV RES PUB HE, V18, DOI 10.3390/ijerph18147684"
" Zou HY, 2021, SCI TOTAL ENVIRON, V772, DOI 10.1016/j.scitotenv.2021.145516"
NR 64
TC 52
Z9 58
U1 16
U2 221
PU ACADEMIC PRESS INC ELSEVIER SCIENCE
PI SAN DIEGO
"PA 525 B ST, STE 1900, SAN DIEGO, CA 92101-4495 USA"
SN 0147-6513
EI 1090-2414

J9 ECOTOX ENVIRON SAFE
JI Ecotox. Environ. Safe.
PD SEP 1
PY 2023
VL 262
AR 115190
DI 10.1016/j.ecoenv.2023.115190
EA JUN 2023
PG 11
WC Environmental Sciences; Toxicology
WE Science Citation Index Expanded (SCI-EXPANDED)
SC Environmental Sciences & Ecology; Toxicology
GA Q1JX5
UT WOS:001055160000001
PM 37390724
"OA Green Submitted, gold"
DA 2026-04-06
ER

PT J
"AU Oladipo, OG"
"   Awotoye, OO"
"   Olayinka, A"
"   Ezeokoli, OT"
"   Maboeta, MS"
"   Bezuidenhout, CC"
"AF Oladipo, Oluwatosin Gbemisola"
"   Awotoye, Olusegun Olufemi"
"   Olayinka, Akin"
"   Ezeokoli, Obinna Tobechukwu"
"   Maboeta, Mark Steve"
"   Bezuidenhout, Cornelius Carlos"
TI Heavy metal tolerance potential of Aspergillus strains isolated
   from mining sites
SO BIOREMEDIATION JOURNAL
LA English
DT Article
DE Aspergillus spp; bioremediation; fungi; heavy metal; metal tolerance;
   mining environment; soil remediation
ID REMEDIATION TECHNOLOGIES; RESISTANT FUNGAL; BIOSORPTION; SOIL;
   BIOREMEDIATION; LEAD; BIOACCUMULATION; WASTEWATERS; REMOVAL; COPPER
"AB Increased heavy metal pollution generated through anthropogenic activities into the environment has necessitated the need for eco-friendly remediation strategies such as mycoremediation. With a view to prospecting for fungi with heavy metal remediation potentials, the tolerance of five Aspergillus species isolated from soils of three active gold and gemstone mining sites in southwestern Nigeria to varied heavy metal concentrations was investigated. Isolated Aspergillus strains were identified based on the internal transcribed spacer 1 and 2 (ITS 1 and ITS 2) regions. Growth of Aspergillus strains were challenged with a range of varied concentrations of heavy metals: cadmium (Cd) (0-100), copper (Cu) (0-1000), lead (Pb) (0-400), arsenic (As) (0-500), and iron (Fe) (0-800) concentrations (ppm) incorporated into Malt Extract Agar (MEA) in triplicates. Mycelial radial growths were recorded at intervals of 3days during a 13-day incubation period. Aspergillus strains were identified as A. tubingensis, A. fumigatus, A. terreus, A. nidulans, and A. nomius. A. tubingensis tolerated Cd, Cu, Pb, As, and Fe at all test concentrations (100-1000 ppm), showing no significant (p > .05) difference compared with the control. Similarly, A. nomius tolerated all concentrations of Cu, Pb, As, and Fe and only 50ppm Cd concentrations. A. nidulans, A. terreus, and A. fumigatus, on the other hand, tolerated all concentrations of Cu, Pb, and Fe with no statistical significance (p > .05) difference from the controls. Overall, the Aspergillus species showed tolerance to heavy metal concentrations above permissible limits for contaminated soils globally. These heavy metal tolerance traits exhibited by the Aspergillus isolates may suggest that they are potential candidates for bioremediation of heavy metal-polluted environments."
"C1 [Oladipo, Oluwatosin Gbemisola; Ezeokoli, Obinna Tobechukwu; Maboeta, Mark Steve; Bezuidenhout, Cornelius Carlos] North West Univ, Unit Environm Sci & Management, Private Bag X6001, ZA-2520 Potchefstroom, South Africa."
"   [Oladipo, Oluwatosin Gbemisola; Awotoye, Olusegun Olufemi] Obafemi Awolowo Univ, Inst Ecol & Environm Studies, Ife, Nigeria."
"   [Olayinka, Akin] Obafemi Awolowo Univ, Dept Soil & Land Resources, Ife, Nigeria."
"   [Ezeokoli, Obinna Tobechukwu] Agr Res Council, Inst Soil Climate & Water, Microbiol & Environm Biotechnol Res Grp, Pretoria, South Africa."
C3 North West University - South Africa; Obafemi Awolowo University;
   Obafemi Awolowo University; Agricultural Research Council of South
"   Africa; Institute for Soil, Climate and Water, Agricultural Research"
   Council
"RP Oladipo, OG (corresponding author), North West Univ, Unit Environm Sci & Management, Private Bag X6001, ZA-2520 Potchefstroom, South Africa."
EM tosin1oladipo@gmail.com
"RI ; Ezeokoli, Obinna Tobechukwu/I-3290-2019; Maboeta, Mark/O-4619-2014;"
"   Bezuidenhout, Carlos/D-3179-2011; Oladipo, Oluwatosin/P-3242-2019"
"OI Awotoye, Olusegun/0000-0002-1791-0313; Olayinka,"
"   Akinyemi/0000-0003-3990-9599; Ezeokoli, Obinna"
"   Tobechukwu/0000-0003-1819-8804; Bezuidenhout,"
   Carlos/0000-0002-6047-4991;
"FU North-West University, Potchefstroom, South Africa"
"FX The authors acknowledge North-West University, Potchefstroom, South"
"   Africa, for the financial support."
"CR Anahid S, 2011, SCI IRAN, V18, P502, DOI 10.1016/j.scient.2011.05.015"

"   Arland P, 2006, BIORESOURCE TECHNOL, V97, P1018, DOI 10.1016/j.biortech.2005.04.046"
"   Bhattacharya S, 2012, TOXICOL ENVIRON CHEM, V94, P429, DOI 10.1080/02772248.2012.657200"
"   Boyd RS, 2010, J CHEM ECOL, V36, P46, DOI 10.1007/s10886-009-9730-5"
"   Luna MAC, 2015, ELECTRON J BIOTECHN, V18, P418, DOI 10.1016/j.ejbt.2015.09.006"
"   Chakraborty R, 2012, CURR OPIN BIOTECH, V23, P483, DOI 10.1016/j.copbio.2012.01.015"
"   Congeevaram S, 2007, J HAZARD MATER, V146, P270, DOI 10.1016/j.jhazmat.2006.12.017"
"   DalCorso G, 2008, J INTEGR PLANT BIOL, V50, P1268, DOI 10.1111/j.1744-7909.2008.00737.x"
"   Dias MA, 2002, LETT APPL MICROBIOL, V34, P46, DOI 10.1046/j.1472-765x.2002.01040.x"
"   Dixit R, 2015, SUSTAINABILITY-BASEL, V7, P2189, DOI 10.3390/su7022189"
"   Dowarah J, 2009, J EARTH SYST SCI, V118, P597, DOI 10.1007/s12040-009-0042-5"
"   Edgar RC, 2004, NUCLEIC ACIDS RES, V32, P1792, DOI 10.1093/nar/gkh340"
"   Eijsackers H, 2014, ENVIRON SCI POLICY, V44, P174, DOI 10.1016/j.envsci.2014.07.017"
"   Fazli MM, 2015, J ENVIRON HEALTH SCI, V13, DOI 10.1186/s40201-015-0176-0"
"   Fomina M, 2007, ENVIRON MICROBIOL, V9, P308, DOI 10.1111/j.1462-2920.2006.01139.x"
"   Food and Agricultural Organization of the United Nations (FAO), 1984, PLANT PROD PROT SER, V1"
"   Fu FL, 2011, J ENVIRON MANAGE, V92, P407, DOI 10.1016/j.jenvman.2010.11.011"
"   Gadd G.M., 1986, Immobilization of Ion by Biosorption, P135"
"   Gadd GM, 2007, MYCOL RES, V111, P3, DOI 10.1016/j.mycres.2006.12.001"
"   GADD GM, 1993, NEW PHYTOL, V124, P25, DOI 10.1111/j.1469-8137.1993.tb03796.x"
"   Gavrilescu M, 2004, ENG LIFE SCI, V4, P219, DOI 10.1002/elsc.200420026"
"   Gazem MAH, 2013, ANN MICROBIOL, V63, P503, DOI 10.1007/s13213-012-0495-7"
"   Hashim MA, 2011, J ENVIRON MANAGE, V92, P2355, DOI 10.1016/j.jenvman.2011.06.009"
"   Hu H, 2014, SUSTAINABILITY-BASEL, V6, P5820, DOI 10.3390/su6095820"
"   Iram S, 2015, ARAB J SCI ENG, V40, P1867, DOI 10.1007/s13369-015-1702-1"
"   Iram S, 2013, POL J ENVIRON STUD, V22, P691"
"   Iram S, 2009, PAK J BOT, V41, P2583"
"   Iskandar NL, 2011, J ENVIRON SCI, V23, P824, DOI 10.1016/S1001-0742(10)60475-5"
"   Kabata-Pendias Alina, 2010, P1"
"   Kapoor A, 1995, BIORESOURCE TECHNOL, V53, P195, DOI 10.1016/0960-8524(95)00072-1"
"   Leitao AL, 2009, INT J ENV RES PUB HE, V6, P1393, DOI 10.3390/ijerph6041393"
"   Li T, 2011, SCI TOTAL ENVIRON, V409, P1069, DOI 10.1016/j.scitotenv.2010.12.012"
"   Maheswari S, 2009, ENVIRON TECHNOL, V30, P921, DOI 10.1080/09593330902971279"
"   Malik A, 2004, ENVIRON INT, V30, P261, DOI 10.1016/j.envint.2003.08.001"
"   Mani D, 2014, INT J ENVIRON SCI TE, V11, P843, DOI 10.1007/s13762-013-0299-8"
"   Mishra A, 2013, CRIT REV ENV SCI TEC, V43, P1162, DOI 10.1080/10934529.2011.627044"
"   Mukherjee A, 2010, ECOTOX ENVIRON SAFE, V73, P172, DOI 10.1016/j.ecoenv.2009.09.015"
"   Oladipo O. G., 2014, Environmental and Experimental Biology, V12, P179"
"   Oladipo OG, 2016, J ENVIRON MANAGE, V181, P435, DOI 10.1016/j.jenvman.2016.07.009"
"   Safahieh A., 2014, AFR J BIOTECHNOL, V11, P4418"
"   Samson R. A., 1984, INTRO FOOD BORNE FUN"
"   Sanyal A, 2005, LANGMUIR, V21, P7220, DOI 10.1021/la047132g"
"   Singh A, 2015, INT J ENVIRON SCI TE, V12, P353, DOI 10.1007/s13762-014-0542-y"
"   Srinath T, 2002, CHEMOSPHERE, V48, P427, DOI 10.1016/S0045-6535(02)00089-9"
"   Strong Peter James, 2008, Bioremediation Journal, V12, P70, DOI 10.1080/10889860802060063"
"   Tamas MJ, 2014, BIOMOLECULES, V4, P252, DOI 10.3390/biom4010252"
"   Tamura K, 2021, MOL BIOL EVOL, V38, P3022, DOI 10.1093/molbev/msab120"
"   Tj White., 1990, PCR Protocols: A Guide to Methods and Applications, P315, DOI [DOI 10.1016/B978-0-12-372180-8.50042-1, 10.1016/B978-0-12-372180-8.50042-1]"
"   Todorova D, 2008, J APPL MICROBIOL, V104, P178, DOI 10.1111/j.1365-2672.2007.03538.x"
"   Vala AK, 2012, JUNDISHAPUR J MICROB, V5, P542, DOI 10.5812/jjm.3383"
"   Valix M, 2003, MINER ENG, V16, P193, DOI 10.1016/S0892-6875(03)00004-9"
"   Valls M, 2002, FEMS MICROBIOL REV, V26, P327, DOI 10.1111/j.1574-6976.2002.tb00618.x"
"   Vieira Regine H. S. F., 2000, International Microbiology, V3, P17"
"   Volesky B, 2007, WATER RES, V41, P4017, DOI 10.1016/j.watres.2007.05.062"
"   Xia XH, 2013, MOL BIOL EVOL, V30, P1720, DOI 10.1093/molbev/mst064"
"   Yao ZT, 2012, PROCEDIA ENVIRON SCI, V16, P722, DOI 10.1016/j.proenv.2012.10.099"
"   Zafar S, 2007, BIORESOURCE TECHNOL, V98, P2557, DOI 10.1016/j.biortech.2006.09.051"
"   Zhou JM, 2007, PEDOSPHERE, V17, P588, DOI 10.1016/S1002-0160(07)60069-1"
NR 58
TC 34
Z9 42
U1 0
U2 52
PU TAYLOR & FRANCIS INC
PI PHILADELPHIA
"PA 530 WALNUT STREET, STE 850, PHILADELPHIA, PA 19106 USA"
SN 1088-9868
EI 1547-6529
J9 BIOREMEDIAT J
JI Bioremediat. J.
PY 2016
VL 20
IS 4
BP 287
EP 297
DI 10.1080/10889868.2016.1250722
PG 11
"WC Engineering, Environmental; Environmental Sciences"
WE Science Citation Index Expanded (SCI-EXPANDED)
SC Engineering; Environmental Sciences & Ecology
GA EH1WO
UT WOS:000391558700002
DA 2026-04-06
ER

PT J
"AU Yang, X"
"   Yin, ML"
"   Huan, ZL"
"   Zhu, YB"
"   Zhao, SP"
"   Xi, HL"
"AF Yang, Xu"
"   Yin, Mao -ling"
"   Huan, Zheng-lai"
"   Zhu, Yong-bing"
"   Zhao, San -ping"
"   Xi, Hai -ling"
TI Microecological characteristics of water bodies/sediments and microbial
   remediation strategies after 50 years of pollution exposure in
   ammunition destruction sites in China
SO ENVIRONMENTAL RESEARCH
LA English
DT Article
DE Ammunition destruction yard; Water/sediment; Community structure and
   function; Microbial remediation; Ion group
"ID TNT-CONTAMINATED SOIL; 2,4,6-TRINITROTOLUENE TNT; TRINITROTOLUENE TNT;"
   HEAVY-METALS; BIOTRANSFORMATION; BIODEGRADATION; BIOREMEDIATION;
   EXPLOSIVES; RESPONSES; SEAWATER
"AB The effects of long-term ammunition pollution on microecological characteristics were analyzed to formulate microbial remediation strategies. Specifically, the response of enzyme systems, N/O stable isotopes, ion networks, and microbial community structure/function levels were analyzed in long-term (50 years) ammunitioncontaminated water/sediments from a contamination site, and a compound bacterial agent capable of efficiently degrading trinitrotoluene (TNT) while tolerating many heavy metals was selected to remediate the ammunitioncontaminated soil. The basic physical and chemical properties of the water/sediment (pH (up: 0.57-0.64), nitrate (up: 1.31-4.28 times), nitrite (up: 1.51-5.03 times), and ammonium (up: 7.06-70.93 times)) were changed significantly, and the significant differences in stable isotope ratios of N and O (nitrate nitrogen) confirmed the degradability of TNT by indigenous microorganisms exposed to long-term pollution. Heavy metals, such as Pb, Zn, Cu, Cd, Cs, and Sb, have synergistic toxic effects in ammunition-contaminated sites, and significantly decreased the microbial diversity and richness in the core pollution area. However, long-term exposure in the edge pollution area induced microorganisms to use TNT as a carbon and nitrogen sources for life activities and growth and development. The Bacteroidales microbial group was significantly inhibited by ammunition contamination, whereas microorganisms such as Proteobacteria, Acidobacteriota, and Comamonadaceae gradually adapted to this environmental stress by regulating their development and stress responses. Ammunition pollution significantly affected DNA replication and gene regulation in the microecological genetic networks and increased the risk to human health. Mg and K were significantly involved in the internal mechanism of microbial transport, enrichment, and metabolism of TNT. Nine strains of TNT-utilizing microbes were screened for efficient TNT degradation and tolerance to typical heavy metals (copper, zinc and lead) found in contaminated sites, and a compound bacterial agent prepared for effective repair of ammunition-contaminated soil significantly improved the soil ecological environment."
"C1 [Yang, Xu; Yin, Mao -ling; Huan, Zheng-lai; Zhu, Yong-bing; Zhao, San -ping; Xi, Hai -ling] State Key Lab NBC Protect Civilian, Beijing 102205, Peoples R China."
C3 State Key Laboratory of NBC Protection for Civilian Researchers
"RP Xi, HL (corresponding author), State Key Lab NBC Protect Civilian, Beijing 102205, Peoples R China."
EM xihailing@sklnbcpc.cn
"RI YANG, Xu/AEL-9291-2022"
"OI YANG, Xu/0000-0001-9212-9781"
FU Outstanding Talent Program of the Academy of Military Sciences
   [ZQ2021-01]; National Natural Science Foundation of China
"   [22176222,22106182]"
FX This study was financially supported by the Outstanding Talent Program
   of the Academy of Military Sciences (ZQ2021-01) and the National Natural
"   Science Foundation of China (22176222,22106182) . In addition, we thank"
"   Shanghai luming biology Co., Ltd. for their assistance in the analysis"
   of metabolome data.
"CR Ali NA, 2006, ECOTOXICOLOGY, V15, P437, DOI 10.1007/s10646-006-0065-5"
"   Ameen F, 2023, FUNCT PLANT BIOL, V50, P623, DOI 10.1071/FP23027"
"   Amin MM, 2016, J WATER SUPPLY RES T, V65, P564, DOI 10.2166/aqua.2016.044"
"   Anasonye F, 2015, INT BIODETER BIODEGR, V105, P7, DOI 10.1016/j.ibiod.2015.08.003"
"   Bai DS, 2022, J ENVIRON MANAGE, V305, DOI 10.1016/j.jenvman.2021.114400"
"   Barman D, 2022, BIOLOGIA, V77, P533, DOI 10.1007/s11756-021-00963-y"
"   Beck AJ, 2018, FRONT MAR SCI, V5, DOI 10.3389/fmars.2018.00141"
"   Beldowski J, 2020, MAR ENVIRON RES, V162, DOI 10.1016/j.marenvres.2020.105189"
"   Berthe-Corti L, 1998, CHEMOSPHERE, V37, P209, DOI 10.1016/S0045-6535(98)00039-3"
"   Chatterjee S, 2017, CHEMOSPHERE, V184, P438, DOI 10.1016/j.chemosphere.2017.06.008"
"   Coffin RB, 2001, ENVIRON TOXICOL CHEM, V20, P2676, DOI 10.1002/etc.5620201204"
"   Cordero I, 2019, SOIL BIOL BIOCHEM, V134, P72, DOI 10.1016/j.soilbio.2019.03.014"
"   Dai MD, 2023, ENVIRON TECHNOL INNO, V30, DOI 10.1016/j.eti.2023.103042"
"   Bui DN, 2021, SCI TOTAL ENVIRON, V756, DOI 10.1016/j.scitotenv.2020.143852"
"   Frau LJ, 2020, GEODERMA REG, V21, DOI 10.1016/j.geodrs.2020.e00278"
"   Fuchs J, 2011, ENVIRON MONIT ASSESS, V175, P127, DOI 10.1007/s10661-010-1499-z"
"   Hartemink AE, 2023, PLANT SOIL, V486, P209, DOI 10.1007/s11104-022-05861-z"
"   Hoehamer CF, 2006, INT J PHYTOREMEDIAT, V8, P95, DOI 10.1080/15226510600678423"
"   Hosseininasabl V, 2022, NAT CHEM, V14, P1265, DOI 10.1038/s41557-022-01025-9"
"   Hou TT, 2023, SCI TOTAL ENVIRON, V858, DOI 10.1016/j.scitotenv.2022.159954"
"   Kao CM, 2016, BIOREMEDIAT J, V20, P165, DOI 10.1080/10889868.2016.1148007"
"   Kobayashi T, 2020, MAR FRESHWATER RES, V71, P617, DOI 10.1071/MF18468"
"   Lamba J, 2022, ARCH MICROBIOL, V204, DOI 10.1007/s00203-022-03057-8"

"   Lamba J, 2021, ENVIRON MONIT ASSESS, V193, DOI 10.1007/s10661-021-08869-7"
"   Li ZY, 2018, MATER CHEM FRONT, V2, P537, DOI 10.1039/c7qm00547d"
"   Liadze I, 2023, WORLD ECON, V46, P874, DOI 10.1111/twec.13336"
"   Liou MJ, 2003, WATER RES, V37, P3172, DOI 10.1016/S0043-1354(03)00158-1"
"   Logsdon SD, 2018, SCI TOTAL ENVIRON, V627, P39, DOI 10.1016/j.scitotenv.2018.01.182"
"   Luo JP, 2023, J HAZARD MATER, V443, DOI 10.1016/j.jhazmat.2022.130290"
"   Mohammed KS, 2023, ENVIRON SCI POLLUT R, V30, P36782, DOI 10.1007/s11356-022-24833-5"
"   Nerlinger M, 2022, FINANC RES LETT, V50, DOI 10.1016/j.frl.2022.103243"
"   Nyanhongo GS, 2005, BIOCATAL BIOTRANSFOR, V23, P53, DOI 10.1080/10242420500090169"
"   O'Callaghan M, 2022, SOIL USE MANAGE, V38, P1340, DOI 10.1111/sum.12811"
"   Oh SY, 2015, SOIL SEDIMENT CONTAM, V24, P157, DOI 10.1080/15320383.2014.929633"
"   Olaniyan FT, 2022, ANN MICROBIOL, V72, DOI 10.1186/s13213-022-01701-8"
"   Parolo ME, 2017, J ENVIRON MANAGE, V196, P316, DOI 10.1016/j.jenvman.2017.03.018"
"   Rezaei MR, 2010, SOIL SEDIMENT CONTAM, V19, P504, DOI 10.1080/15320383.2010.486049"
"   Rousk J, 2018, ENVIRON POLLUT, V240, P297, DOI 10.1016/j.envpol.2018.04.087"
"   Schlichting D, 2017, PLOS ONE, V12, DOI 10.1371/journal.pone.0184946"
"   Sens C, 1998, ENVIRON SCI POLLUT R, V5, P202, DOI 10.1007/BF02986402"
"   Serrano-Gonz??lez MY, 2018, DEF TECHNOL, V14, P151, DOI 10.1016/j.dt.2018.01.004"
"   Sigman DM, 2001, ANAL CHEM, V73, P4145, DOI 10.1021/ac010088e"
"   Stauffer M, 2017, B ENVIRON CONTAM TOX, V98, P14, DOI 10.1007/s00128-016-1976-3"
"   Taillefer M, 2018, MSYSTEMS, V3, DOI 10.1128/mSystems.00240-18"
"   Takano C, 2022, J BIOSCI BIOENG, V134, P521, DOI 10.1016/j.jbiosc.2022.08.009"
"   Via SM, 2014, ENVIRON EXP BOT, V99, P67, DOI 10.1016/j.envexpbot.2013.11.002"
"   Vukicevich E, 2016, AGRON SUSTAIN DEV, V36, DOI 10.1007/s13593-016-0385-7"
"   Yakovleva G, 2022, BIODEGRADATION, V33, P593, DOI 10.1007/s10532-022-09997-8"
"   Yang X, 2023, J CLEAN PROD, V432, DOI 10.1016/j.jclepro.2023.139715"
"   Yang X, 2022, SCI TOTAL ENVIRON, V815, DOI 10.1016/j.scitotenv.2021.152856"
"   Yang X, 2021, ENVIRON POLLUT, V285, DOI 10.1016/j.envpol.2021.117478"
"   Yang X, 2021, CHEMOSPHERE, V270, DOI 10.1016/j.chemosphere.2020.129280"
"   Yang ZL, 2018, PHYS CHEM CHEM PHYS, V20, P12157, DOI 10.1039/c8cp00345a"
"   Zhang AG, 2017, REG STUD MAR SCI, V11, P32, DOI 10.1016/j.rsma.2017.02.002"
"   Zheng S, 2022, ECOL ENG, V182, DOI 10.1016/j.ecoleng.2022.106719"
"   Zhou TT, 2018, ARCH ENVIRON CON TOX, V74, P577, DOI 10.1007/s00244-017-0497-3"
NR 56
TC 8
Z9 10
U1 6
U2 40
PU ACADEMIC PRESS INC ELSEVIER SCIENCE
PI SAN DIEGO
"PA 525 B ST, STE 1900, SAN DIEGO, CA 92101-4495 USA"
SN 0013-9351
EI 1096-0953
J9 ENVIRON RES
JI Environ. Res.
PD JUN 15
PY 2024
VL 251
AR 118640
DI 10.1016/j.envres.2024.118640
EA MAR 2024
PN 2
PG 12
"WC Environmental Sciences; Public, Environmental & Occupational Health"
WE Science Citation Index Expanded (SCI-EXPANDED)
"SC Environmental Sciences & Ecology; Public, Environmental & Occupational"
   Health
GA QM5Z2
UT WOS:001221316300001
PM 38479720
DA 2026-04-06
ER

PT J
"AU Tiwari, S"
"   Ullah, A"
"   Feng, YX"
"   Yu, XZ"
"AF Tiwari, Sarita"
"   Ullah, Abid"
"   Feng, Yu-Xi"
"   Yu, Xiao-Zhang"
TI Exploring plant symbiotic microbial dynamics in metal hyperaccumulators
   for phytoremediation
SO INTERNATIONAL BIODETERIORATION & BIODEGRADATION
LA English
DT Article
DE Endophytic microbes; Heavy metals; Phytoremediation; Microbial
   remediation; Hyperaccumulators; Plant microbe interactions
ID RESISTANT ENDOPHYTIC BACTERIA; HEAVY-METALS; RHIZOSPHERE MICROBIOME;
   COMMUNITY DIVERSITY; SEDUM-ALFREDII; CD PHYTOEXTRACTION; POLLUTED SOILS;
   GROWTH; CADMIUM; BIOREMEDIATION
"AB Metal hyperaccumulators (HAs) are renowned for their remarkable capacity to sequester heavy metals (HMs) within their tissues, a trait that is closely linked to their symbiotic relationship with a unique set of

microbes inhabiting their rhizosphere and endosphere. Despite the pivotal role of HAs in the remediation of HMs, the intricacies of the microbiome characteristics associated with HAs remain largely elusive. This review navigates the intricate microbial landscape of the rhizosphere associated with hyperaccumulating plants. Emphasizing the assembly and colonization dynamics of microbiomes by HAs, it sheds light on the substantial variations in community structure and function during exposure to HMs. Furthermore, this study delves into the multifaceted role of HA-associated microbes in HMs remediation, elucidating both direct and indirect mechanisms employed by these microbial communities in assisting phytoremediation. By providing new insights into the interaction between HAs and their microbial partners, this review highlights the vital contributions of these symbiotic relationships to the efficient extraction and detoxification of toxic metals through phytoremediation."
"C1 [Tiwari, Sarita; Ullah, Abid; Feng, Yu-Xi; Yu, Xiao-Zhang] Guilin Univ Technol, Coll Environm Sci & Engn, Guilin 541004, Peoples R China."
C3 Guilin University of Technology
"RP Yu, XZ (corresponding author), Guilin Univ Technol, Coll Environm Sci & Engn, Guilin 541004, Peoples R China."
EM xzyu@glut.edu.cn
"RI Ullah, Abid/AAD-1241-2020"
FU National Natural Science Foundation of China [42277361]
FX This work is financially supported by the National Natural Science
Foundation of China (No: 42277361) .
"CR Abou-Shanab RAI, 2022, INT J PHYTOREMEDIAT, V24, P420, DOI 10.1080/15226514.2021.1951654"
"   Adedayo AA, 2023, J FUNGI, V9, DOI 10.3390/jof9020239"
"   Adnan M, 2024, ECOTOX ENVIRON SAFE, V274, DOI 10.1016/j.ecoenv.2024.116189"
"   Ahemad M, 2014, FOLIA MICROBIOL, V59, P321, DOI 10.1007/s12223-014-0304-8"
"   Ahsan MT, 2019, WORLD J MICROB BIOT, V35, DOI 10.1007/s11274-019-2757-z"
"   Aioub AAA, 2021, ENVIRON SCI POLLUT R, V28, P43992, DOI 10.1007/s11356-021-13853-2"
"   Ajuna HB, 2023, INT J MOL SCI, V24, DOI 10.3390/ijms242316889"
"   Alford ??R, 2010, PLANT SOIL, V337, P33, DOI 10.1007/s11104-010-0482-3"
"   Alloway B.J., 2013, Heavy Metals in Soils, P11, DOI [10.1007/978-94-007-4470-7-2, DOI 10.1007/978-94-007-4470-7-2, 10.1002/9781119438922.ch18, DOI 10.1007/978-94-007-4470-72]"
"   Alves ARA, 2022, SCI TOTAL ENVIRON, V838, DOI 10.1016/j.scitotenv.2022.156435"
"   Aqsa, 2022, Advances in Microbe-Assisted Phytoremediation of Polluted Sites, P147"
"   Asad SA, 2019, CHEMOSPHERE, V217, P925, DOI 10.1016/j.chemosphere.2018.11.021"
"   Aubin E, 2021, LIFE-BASEL, V11, DOI 10.3390/life11080857"
"   Baker AJM, 2000, PHYTOREMEDIATION OF CONTAMINATED SOIL AND WATER, P85"
"   Bakker PAHM, 2013, FRONT PLANT SCI, V4, DOI 10.3389/fpls.2013.00165"
"   Barnawal D, 2017, PHYSIOL PLANTARUM, V161, P502, DOI 10.1111/ppl.12614"
"   Barnes EM, 2020, FRONT MICROBIOL, V11, DOI 10.3389/fmicb.2020.00101"
"   Barzanti R, 2007, MICROB ECOL, V53, P306, DOI 10.1007/s00248-006-9164-3"
"   Berendsen RL, 2012, TRENDS PLANT SCI, V17, P478, DOI 10.1016/j.tplants.2012.04.001"
"   Bledsoe R, 2016, INT BIODETER BIODEGR, V113, P155, DOI 10.1016/j.ibiod.2016.02.010"
"   Boller T, 2009, SCIENCE, V324, P742, DOI 10.1126/science.1171647"
"   Boorboori MR, 2022, J FUNGI, V8, DOI 10.3390/jof8020176"
"   Chaparro JM, 2014, ISME J, V8, P790, DOI 10.1038/ismej.2013.196"
"   Chen HM, 2018, INT J MOL SCI, V19, DOI 10.3390/ijms19030672"
"   Chen L, 2024, BIOLOGY-BASEL, V13, DOI 10.3390/biology13020095"
"   Chen S, 2013, INT J PHYTOREMEDIAT, V15, P477, DOI 10.1080/15226514.2012.716100"
"   Chen YT, 2017, CURR OPIN PLANT BIOL, V39, P66, DOI 10.1016/j.pbi.2017.06.004"
"   Cheung KH, 2007, INT BIODETER BIODEGR, V59, P8, DOI 10.1016/j.ibiod.2006.05.002"
"   Copeland JK, 2015, MOL PLANT MICROBE IN, V28, P274, DOI 10.1094/MPMI-10-14-0331-FI"
"   Costa OYA, 2018, FRONT MICROBIOL, V9, DOI 10.3389/fmicb.2018.01636"
"   Dai HP, 2021, CHEMOSPHERE, V273, DOI 10.1016/j.chemosphere.2021.129652"
"   Danyal Y, 2023, ENVIRON SCI POLLUT R, V30, P5296, DOI 10.1007/s11356-022-23967-w"
"   Deicke M, 2013, J CHROMATOGR A, V1298, P50, DOI 10.1016/j.chroma.2013.05.008"
"   Montero-Calasanz MD, 2013, INT J SYST EVOL MICR, V63, P4386, DOI 10.1099/ijs.0.052456-0"
"   Edwards J, 2015, P NATL ACAD SCI USA, V112, pE911, DOI 10.1073/pnas.1414592112"
"   El-Deeb B, 2012, GEOMICROBIOL J, V29, P906, DOI 10.1080/01490451.2011.635764"
"   El-Mahdy OM, 2023, BMC MICROBIOL, V23, DOI 10.1186/s12866-023-03100-8"
"   L??pez-P??rez ME, 2021, ARCH MICROBIOL, V203, P499, DOI 10.1007/s00203-020-01973-1"
"   Ellermann M, 2017, FREE RADICAL BIO MED, V105, P68, DOI 10.1016/j.freeradbiomed.2016.10.489"
"   Erlandson S, 2018, MOL ECOL, V27, P2007, DOI 10.1111/mec.14576"
"   Gadd GM, 2010, MICROBIOL-SGM, V156, P609, DOI 10.1099/mic.0.037143-0"
"   Gateta T, 2023, J FUNGI, V9, DOI 10.3390/jof9090937"
"   Gervais-Bergeron B, 2023, PLANT SOIL, V488, P187, DOI 10.1007/s11104-023-05996-7"
"   Giotta L, 2011, LANGMUIR, V27, P3762, DOI 10.1021/la104868m"
"   Glick BR, 2010, BIOTECHNOL ADV, V28, P367, DOI 10.1016/j.biotechadv.2010.02.001"
"   Golestanifard A, 2020, ENVIRON POLLUT, V266, DOI 10.1016/j.envpol.2020.115088"
"   Goolsby EW, 2016, FRONT PLANT SCI, V6, DOI 10.3389/fpls.2015.01252"
"   Guo HJ, 2010, BIORESOURCE TECHNOL, V101, P8599, DOI 10.1016/j.biortech.2010.06.085"
"   Gupta Pratima, 2017, Biotechnol Rep (Amst), V13, P58, DOI [10.1016/j.btre.2016.12.006, 10.1016/j.btre.2016.12.006]"
"   Hacquard S, 2015, CELL HOST MICROBE, V17, P603, DOI 10.1016/j.chom.2015.04.009"
"   Haichar FE, 2014, SOIL BIOL BIOCHEM, V77, P69, DOI 10.1016/j.soilbio.2014.06.017"
"   Hardoim PR, 2008, TRENDS MICROBIOL, V16, P463, DOI 10.1016/j.tim.2008.07.008"
"   He LY, 2009, ECOTOX ENVIRON SAFE, V72, P1343, DOI 10.1016/j.ecoenv.2009.03.006"
"   Hou JY, 2021, PLANT SOIL, V468, P375, DOI 10.1007/s11104-021-05123-4"
"   Idris R, 2004, APPL ENVIRON MICROB, V70, P2667, DOI 10.1128/AEM.70.5.2667-2677.2004"
"   Idris R, 2006, SYST APPL MICROBIOL, V29, P634, DOI 10.1016/j.syapm.2006.01.011"
"   Islam M, 2022, SOIL HLTH ENV SUSTAI, P615, DOI DOI 10.1007/978-3-031-09270-127"
"   Jan AT, 2014, CRIT REV ENV SCI TEC, V44, P519, DOI 10.1080/10643389.2012.728811"
"   Jayanthi B, 2017, INT BIODETER BIODEGR, V119, P467, DOI 10.1016/j.ibiod.2016.10.053"
"   Jayaprakashvel M., 2019, Secondary Metabolites of Plant Growth Promoting Rhizomicroorganisms: Discovery and Applications, P293, DOI DOI 10.1007/978-981-13-5862-3_15"
"   Jayaprakashvel M., 2019, Sustainable Agriculture Reviews, V35, P117"

"   Jiang XW, 2021, ENVIRON POLLUT, V280, DOI 10.1016/j.envpol.2021.116947"
"   Johnston-Monje D, 2016, PLANT SOIL, V405, P337, DOI 10.1007/s11104-016-2826-0"
"   Jones DL, 2004, NEW PHYTOL, V163, P459, DOI 10.1111/j.1469-8137.2004.01130.x"
"   Kemen E, 2014, CURR OPIN PLANT BIOL, V20, P75, DOI 10.1016/j.pbi.2014.04.005"
"   Koley A., 2024, Bioremediation for Sustainable Environmental Cleanup, P238, DOI DOI 10.1201/9781003277941"
"   Koley A., 2022, BIOREMEDIATION TOXIC, P95, DOI [DOI 10.1201/9781003229940-6, 10.1201/9781003229940-6]"
"   Kumar A, 2020, J ADV RES, V24, P337, DOI 10.1016/j.jare.2020.04.014"
"   Laforest-Lapointe I, 2016, MICROBIOME, V4, DOI 10.1186/s40168-016-0174-1"
"   Laiq M, 2024, APPL SOIL ECOL, V195, DOI 10.1016/j.apsoil.2023.105258"
"   Lebeis SL, 2015, SCIENCE, V349, P860, DOI 10.1126/science.aaa8764"
"   Li Q, 2021, ECOTOX ENVIRON SAFE, V212, DOI 10.1016/j.ecoenv.2021.112016"
"   Li TQ, 2013, CHEMOSPHERE, V91, P970, DOI 10.1016/j.chemosphere.2013.01.100"
"   Li WC, 2007, J EXP BOT, V58, P4173, DOI 10.1093/jxb/erm274"
"   Lin H, 2023, INT BIODETER BIODEGR, V178, DOI 10.1016/j.ibiod.2022.105544"
"   Lin HY, 2022, FRONT MICROBIOL, V13, DOI 10.3389/fmicb.2022.872034"
"   Liu WX, 2015, J SOIL SEDIMENT, V15, P1191, DOI 10.1007/s11368-015-1067-9"
"   Liu YY, 2019, SOIL ECOL LETT, V1, P59, DOI 10.1007/s42832-019-0009-7"
"   Lodewyckx C, 2001, INT J PHYTOREMEDIAT, V3, P173, DOI 10.1080/15226510108500055"
"   Lone MI, 2008, J ZHEJIANG UNIV-SC B, V9, P210, DOI 10.1631/jzus.B0710633"
"   Lopez S, 2020, ENVIRON MICROBIOL, V22, P1649, DOI 10.1111/1462-2920.14970"
"   Lopez S, 2019, APPL SOIL ECOL, V133, P70, DOI 10.1016/j.apsoil.2018.09.007"
"   Lopez S, 2019, SCI TOTAL ENVIRON, V654, P237, DOI 10.1016/j.scitotenv.2018.11.056"
"   Lopez S, 2017, SOIL BIOL BIOCHEM, V114, P121, DOI 10.1016/j.soilbio.2017.07.010"
"   Lundberg DS, 2012, NATURE, V488, P86, DOI 10.1038/nature11237"
"   Luo JP, 2019, ENVIRON SCI TECHNOL, V53, P6954, DOI 10.1021/acs.est.9b01093"
"   Luo JP, 2017, APPL MICROBIOL BIOT, V101, P7961, DOI 10.1007/s00253-017-8469-0"
"   Luo SL, 2011, CHEMOSPHERE, V85, P1130, DOI 10.1016/j.chemosphere.2011.07.053"
"   Ma Y, 2016, J ENVIRON MANAGE, V174, P14, DOI 10.1016/j.jenvman.2016.02.047"
"   Ma Y, 2013, CHEMOSPHERE, V93, P1386, DOI 10.1016/j.chemosphere.2013.06.077"
"   Mahrukh Zubair Mahrukh Zubair, 2016, Environmental Technology Reviews, V5, P112, DOI [10.1080/21622515.2016.1259358, 10.1080/21622515.2016.1259358]"
"   Marschner H., 1995, Mineral Nutrition of Higher Plants, DOI [DOI 10.1016/C2009-0-63043-9, DOI 10.1016/B978-0-12-473542-2.X5000-7]"
"   Martos S, 2021, FRONT MICROBIOL, V12, DOI 10.3389/fmicb.2021.639997"
"   Memon A.R., 2016, Phytoremediat.: Manag. Environ. Contam., V3, P239, DOI [DOI 10.1007/978-3-319-40148-58, 10.1007/]"
"   Moran NA, 2015, PLOS BIOL, V13, DOI 10.1371/journal.pbio.1002311"
"   Mular A, 2024, COORDIN CHEM REV, V501, DOI 10.1016/j.ccr.2023.215551"
"   M??ller DB, 2016, ANNU REV GENET, V50, P211, DOI 10.1146/annurev-genet-120215-034952"
"   Nagajyoti PC, 2010, ENVIRON CHEM LETT, V8, P199, DOI 10.1007/s10311-010-0297-8"
"   Narayanan M, 2023, J ENVIRON MANAGE, V345, DOI 10.1016/j.jenvman.2023.118732"
"   Nejad P, 2000, BIOL CONTROL, V18, P208, DOI 10.1006/bcon.2000.0837"
"   Nirola R, 2016, INT BIODETER BIODEGR, V113, P208, DOI 10.1016/j.ibiod.2016.03.007"
"   Novo L.A., 2018, Bio-Geotechnologies for Mine Site Rehabilitation, P281, DOI DOI 10.1016/B978-0-12-812986-9.00016-6"
"   Olenska E, 2023, SCI REP-UK, V13, DOI 10.1038/s41598-023-39852-6"
"   Oner E.T., 2013, PRETREATMENT TECHNIQ, P35, DOI DOI 10.1007/978-3-642-32735-3_2"
"   Pan FS, 2016, CHEMOSPHERE, V154, P358, DOI 10.1016/j.chemosphere.2016.03.120"
"   Pande V, 2022, FRONT MICROBIOL, V13, DOI 10.3389/fmicb.2022.824084"
"   Pasricha S., 2021, Environ. Chall, V4, P100197, DOI DOI 10.1016/J.ENVC.2021.100197"
"   Pollard AJ, 2002, CRIT REV PLANT SCI, V21, P539, DOI 10.1080/0735-260291044359"
"   Pramanik K., 2021, MICROBES FDN STONE B, P441, DOI [10.1007/978-3-030-63512-1_22, DOI 10.1007/978-3-030-63512-1_22]"
"   Pramanik K, 2021, CHEMOSPHERE, V274, DOI 10.1016/j.chemosphere.2021.129819"
"   Prasad S, 2021, J ENVIRON MANAGE, V285, DOI 10.1016/j.jenvman.2021.112174"
"   Prashar P, 2014, REV ENVIRON SCI BIO, V13, P63, DOI 10.1007/s11157-013-9317-z"
"   Priya AK, 2023, TOXICS, V11, DOI 10.3390/toxics11050422"
"   Pujari M., 2021, Heavy Metals in the Environment, P1, DOI [10.1016/B978-0-12-821656-9.00001-8, DOI 10.1016/B978-0-12-821656-9.00001-8]"
"   Qiao YB, 2024, ENVIRON SCI TECHNOL, V58, P14726, DOI 10.1021/acs.est.4c01418"
"   Qin HC, 2024, MICROORGANISMS, V12, DOI 10.3390/microorganisms12040700"
"   Rajkumar M, 2012, BIOTECHNOL ADV, V30, P1562, DOI 10.1016/j.biotechadv.2012.04.011"
"   Raklami A, 2022, INT J MOL SCI, V23, DOI 10.3390/ijms23095031"
"   Rani R., 2015, INT C BIOL ENV FOOD"
"   Rascio N, 2011, PLANT SCI, V180, P169, DOI 10.1016/j.plantsci.2010.08.016"
"   Reed GP, 2005, ENERG FUEL, V19, P298, DOI 10.1021/ef049943y"
"   Reeves RD, 2018, NEW PHYTOL, V218, P407, DOI 10.1111/nph.14907"
"   Reinhold-Hurek B, 2015, ANNU REV PHYTOPATHOL, V53, P403, DOI 10.1146/annurev-phyto-082712-102342"
"   Rezania S, 2016, J HAZARD MATER, V318, P587, DOI 10.1016/j.jhazmat.2016.07.053"
"   Roskova Z, 2022, SCI TOTAL ENVIRON, V819, DOI 10.1016/j.scitotenv.2022.153144"
"   Saeed Q, 2021, INT J MOL SCI, V22, DOI 10.3390/ijms221910529"
"   Saleh Tawfik A., 2022, Environmental Nanotechnology, Monitoring and Management, DOI [10.1016/j.enmm.2021.100617, 10.1016/j.enmm.2021.100617]"
"   Selatnia A, 2004, BIOCHEM ENG J, V19, P127, DOI 10.1016/j.bej.2003.12.007"
"   Sessitsch A, 2013, SOIL BIOL BIOCHEM, V60, P182, DOI 10.1016/j.soilbio.2013.01.012"
"   Sharma P, 2022, ENVIRON TECHNOL INNO, V25, DOI 10.1016/j.eti.2021.102182"
"   Sharma P, 2021, BIORESOURCE TECHNOL, V339, DOI 10.1016/j.biortech.2021.125589"
"   Sheng XF, 2008, ENVIRON POLLUT, V156, P1164, DOI 10.1016/j.envpol.2008.04.007"
"   Gonzaga MIS, 2009, SCI TOTAL ENVIRON, V407, P4711, DOI 10.1016/j.scitotenv.2009.04.037"
"   Singh R, 2022, PHYTOFRONTIERS, V2, P158, DOI 10.1094/PHYTOFR-04-21-0026-RVW"
"   Siripan O, 2018, INT J PHYTOREMEDIAT, V20, P1096, DOI 10.1080/15226514.2017.1365338"
"   Skuza L, 2022, INT J MOL SCI, V23, DOI 10.3390/ijms23169335"
"   Song PP, 2022, SCI TOTAL ENVIRON, V838, DOI 10.1016/j.scitotenv.2022.156417"

"   Sytar O, 2021, PHYSIOL PLANTARUM, V173, P148, DOI 10.1111/ppl.13285"
"   Theis KR, 2016, MSYSTEMS, V1, DOI 10.1128/mSystems.00028-16"
"   Thijs S, 2017, ADV BOT RES, V83, P43, DOI 10.1016/bs.abr.2016.12.003"
"   Tiwari S, 2016, J ENVIRON MANAGE, V180, P359, DOI 10.1016/j.jenvman.2016.05.029"
"   Tkacz A, 2021, PLANTS PEOPLE PLANET, V3, P124, DOI 10.1002/ppp3.10167"
"   Tokuda M, 2024, MICROB BIOTECHNOL, V17, DOI 10.1111/1751-7915.14408"
"   Tovar-S??nchez E, 2023, PLANTS-BASEL, V12, DOI 10.3390/plants12061338"
"   T??rkmen D, 2022, FRONT SUSTAIN, V3, DOI 10.3389/frsus.2022.765592"
"   Turner TR, 2013, GENOME BIOL, V14, DOI 10.1186/gb-2013-14-6-209"
"   Uddin MM, 2021, TOXICS, V9, DOI 10.3390/toxics9120360"
"   Ullah A, 2024, INT BIODETER BIODEGR, V190, DOI 10.1016/j.ibiod.2024.105787"
"   Ullah A, 2024, TOXICS, V12, DOI 10.3390/toxics12010004"
"   Ullah A, 2019, MICROB ECOL, V77, P429, DOI 10.1007/s00248-018-1260-7"
"   Ullah A, 2015, ENVIRON EXP BOT, V117, P28, DOI 10.1016/j.envexpbot.2015.05.001"
"   van der Ent A, 2013, PLANT SOIL, V362, P319, DOI 10.1007/s11104-012-1287-3"
"   van der Pas L, 2019, PLANTS-BASEL, V8, DOI 10.3390/plants8010011"
"   Verbon EH, 2016, TRENDS PLANT SCI, V21, P218, DOI 10.1016/j.tplants.2016.01.013"
"   Vincent SA, 2022, FRONT MICROBIOL, V13, DOI 10.3389/fmicb.2022.809940"
"   Wan YA, 2024, TOXICS, V12, DOI 10.3390/toxics12010063"
"   Wang Q, 2019, CHEMOSPHERE, V234, P769, DOI 10.1016/j.chemosphere.2019.06.132"
"   Wang Q, 2019, ECOTOX ENVIRON SAFE, V172, P97, DOI 10.1016/j.ecoenv.2019.01.009"
"   Wang YY, 2014, J AGR FOOD CHEM, V62, P1783, DOI 10.1021/jf404152u"
"   Wei XM, 2023, CHIN HERB MED, V15, P45, DOI 10.1016/j.chmed.2022.01.008"
"   White LJ, 2015, MOL PLANT MICROBE IN, V28, P22, DOI 10.1094/MPMI-08-14-0247-R"
"   Wu LK, 2023, FRONT PLANT SCI, V14, DOI 10.3389/fpls.2023.1281010"
"   Wu YJ, 2020, J HAZARD MATER, V395, DOI 10.1016/j.jhazmat.2020.122661"
"   Wu YH, 2021, BIOMOLECULES, V11, DOI 10.3390/biom11111715"
"   Yadav KK, 2018, ECOL ENG, V120, P274, DOI 10.1016/j.ecoleng.2018.05.039"
"   Yan HL, 2019, J HAZARD MATER, V368, P386, DOI 10.1016/j.jhazmat.2019.01.072"
"   Yang CY, 2020, ECOTOX ENVIRON SAFE, V190, DOI 10.1016/j.ecoenv.2019.110075"
"   Yang L, 2022, ECOTOX ENVIRON SAFE, V234, DOI 10.1016/j.ecoenv.2022.113403"
"   Yuan M, 2014, CHEMOSPHERE, V103, P99, DOI 10.1016/j.chemosphere.2013.11.040"
"   Zhang KP, 2017, ENVIRON MICROBIOL, V19, P3649, DOI 10.1111/1462-2920.13865"
"   Zhang N, 2014, PLANT SOIL, V374, P689, DOI 10.1007/s11104-013-1915-6"
"   Zhang Y, 2022, FRONT PLANT SCI, V13, DOI 10.3389/fpls.2022.912350"
"   Zhang YH, 2024, ECOTOX ENVIRON SAFE, V269, DOI 10.1016/j.ecoenv.2023.115739"
"   Zhu L, 2022, FRONT PLANT SCI, V13, DOI 10.3389/fpls.2022.952397"
"   Zipfel C, 2014, TRENDS IMMUNOL, V35, P345, DOI 10.1016/j.it.2014.05.004"
"   Zloch M, 2016, CHEMOSPHERE, V156, P312, DOI 10.1016/j.chemosphere.2016.04.130"
NR 176
TC 6
Z9 9
U1 3
U2 23
PU ELSEVIER SCI LTD
PI London
"PA 125 London Wall, London, the United Kingdom"
SN 0964-8305
EI 1879-0208
J9 INT BIODETER BIODEGR
JI Int. Biodeterior. Biodegrad.
PD JAN
PY 2025
VL 196
AR 105941
DI 10.1016/j.ibiod.2024.105941
EA OCT 2024
PG 12
WC Biotechnology & Applied Microbiology; Environmental Sciences
WE Science Citation Index Expanded (SCI-EXPANDED)
SC Biotechnology & Applied Microbiology; Environmental Sciences & Ecology
GA J1P0T
UT WOS:001334848800001
OA hybrid
DA 2026-04-06
ER

PT J
"AU Dou, RQ"
"   Xie, YL"
"   Liu, FX"
"   Xu, F"
"   Xiao, KM"
"AF Dou, Ruqiang"
"   Xie, Yanluo"
"   Liu, Frank X."
"   Xu, Fei"
"   Xiao, Kemeng"
TI Reconstruction of microecosystem in extreme soil environment by
   biochar-bacteria enhanced mycoremediation
SO JOURNAL OF ENVIRONMENTAL CHEMICAL ENGINEERING
LA English
DT Article
DE Soil remediation; Biochar; Heavy metals; Stropharia rugoso-annulata;

Metal-resistant bacterium
ID GROWTH-PROMOTING BACTERIA; HEAVY-METALS; CONTAMINATED SOIL; REMEDIATION;
PHYTOREMEDIATION; BIOREMEDIATION; CARBON; AVAILABILITY; STRATEGY; UREASE
"AB Soil contamination by heavy metals threatens the biodiversity and even disrupt microecosystems in extreme conditions. Mycoremediation, utilizing fungi as accumulators, is a promising technique for heavy metals removal in situ, however, the remediation efficiency is often constrained. In this study, we presented a novel remediation approach to tackle cadmium (Cd) and copper (Cu) contamination with high efficiency and short disposing period. The metal-resistant bacteria Serratia spp. Strain CTZ4 was isolated and domesticated. Then, a straw biochar-bacteria hybrid system with porous structure was developed as an amendment for Stropharia rugosoannulata. The hybrid system was observed to largely increase the acid extractable Cd and Cu, while reduce the residual component, indicating improved metal activation and bioavailability. Immobilized bacteria on biochar significantly enhanced fungal biomass and the metal uptake capacity, with the realized increment of more than similar to 150 % and similar to 220 %, respectively. Furthermore, biochar-bacteria demonstrated a synergistic effect with Stropharia rugoso-annulata, leading to enhancements in soil pH, respiration rates, microbial populations, enzyme activities, bacterial diversity and abundance. This improvement in soil physicochemical properties and microbial community help to reconstruct the microecosystem, facilitating the continuous removal of heavy metals in extreme soil environment."
"C1 [Dou, Ruqiang] Univ Elect Sci & Technol China, Shenzhen Inst Adv Study, Shenzhen 518110, Peoples R China."
"   [Dou, Ruqiang; Xiao, Kemeng] Chinese Univ Hong Kong, Dept Chem, Shatin, Hong Kong, Peoples R China."
"   [Dou, Ruqiang; Xiao, Kemeng] Chinese Univ Hong Kong, Ctr Cell & Dev Biol, Shatin, Hong Kong, Peoples R China."
"   [Dou, Ruqiang; Xie, Yanluo; Xu, Fei; Xiao, Kemeng] Sichuan Univ, Coll Life Sci, Key Lab Bioresources & Ecoenvironm, Minist Educ, Chengdu 610064, Sichuan, Peoples R China."
"   [Xie, Yanluo] Chengdu Med Coll, Coll Pharm, Chengdu 610064, Sichuan, Peoples R China."
"   [Liu, Frank X.] Hong Kong Univ Sci & Technol, Dept Mech & Aerosp Engn, Kowloon, Clear Water Bay, Hong Kong, Peoples R China."
"C3 Shenzhen Institute for Advanced Study, UESTC; University of Electronic"
Science & Technology of China; Chinese University of Hong Kong; Chinese
University of Hong Kong; Sichuan University; Chengdu Medical College;
Hong Kong University of Science & Technology
"RP Dou, RQ (corresponding author), Univ Elect Sci & Technol China, Shenzhen Inst Adv Study, Shenzhen 518110, Peoples R China.; Dou, RQ; Xiao, KM (corresponding author), Chinese Univ Hong Kong, Dept Chem, Shatin, Hong Kong, Peoples R China.; Dou, RQ; Xiao, KM (corresponding author), Chinese Univ Hong Kong, Ctr Cell & Dev Biol, Shatin, Hong Kong, Peoples R China.; Dou, RQ; Xu, F; Xiao, KM (corresponding author), Sichuan Univ, Coll Life Sci, Key Lab Bioresources & Ecoenvironm, Minist Educ, Chengdu 610064, Sichuan, Peoples R China."
EM ruokin@link.cuhk.edu.hk; xufei@scu.edu.cn; kemengxiao@cuhk.edu.hk
"RI ; Xiao, Kemeng/OIC-5102-2025"
"OI DOU, Ruqiang/0000-0003-2642-3610; "
"CR Adhikari S, 2022, SCI TOTAL ENVIRON, V851, DOI 10.1016/j.scitotenv.2022.158043"
"   Adnan M, 2024, ECOTOX ENVIRON SAFE, V274, DOI 10.1016/j.ecoenv.2024.116189"
"   Afshar M, 2024, RESULTS ENG, V23, DOI 10.1016/j.rineng.2024.102433"
"   Ahemad Munees, 2015, Journal of Genetic Engineering and Biotechnology, V13, P51, DOI [10.1016/j.jgeb.2015.02.001, 10.1016/j.jgeb.2015.02.001]"
"   Ajeng AA, 2020, ENVIRON TECHNOL INNO, V20, DOI 10.1016/j.eti.2020.101168"
"   Ali H, 2013, CHEMOSPHERE, V91, P869, DOI 10.1016/j.chemosphere.2013.01.075"
"   Antoniadis V, 2021, ENVIRON INT, V146, DOI 10.1016/j.envint.2020.106233"
"   Arnot JA, 2006, ENVIRON REV, V14, P257, DOI [10.1139/A06-005, 10.1139/a06-005]"
"   Azhar U, 2022, ENVIRON RES, V214, DOI 10.1016/j.envres.2022.113918"
"   BENEFIELD CB, 1977, SOIL BIOL BIOCHEM, V9, P67, DOI 10.1016/0038-0717(77)90063-3"
"   Bolan N, 2014, J HAZARD MATER, V266, P141, DOI 10.1016/j.jhazmat.2013.12.018"
"   Bolan S, 2023, SCI TOTAL ENVIRON, V886, DOI 10.1016/j.scitotenv.2023.163968"
"   Brassard P., Char and Carbon Materials Derived from Biomass: Production, Characterization and Applications, V2019, P109, DOI [10.1016/B978-0-12-814893-8.00004-3, DOI 10.1016/B978-0-12-814893-8.00004-3, 10.1016/b978-0-12-814893-8.00004-3]"
"   Burd GI, 2000, CAN J MICROBIOL, V46, P237, DOI 10.1139/cjm-46-3-237"
"   Castellet-Rovira F, 2018, J ENVIRON MANAGE, V207, P396, DOI 10.1016/j.jenvman.2017.07.052"
"   Chaudhary P, 2023, J ENVIRON CHEM ENG, V11, DOI 10.1016/j.jece.2023.109591"
"   Chen BL, 2012, J SOIL SEDIMENT, V12, P1350, DOI 10.1007/s11368-012-0554-5"
"   Chen L, 2014, SOIL BIOL BIOCHEM, V68, P300, DOI 10.1016/j.soilbio.2013.10.021"
"   Chen YG, 2021, ECOTOX ENVIRON SAFE, V219, DOI 10.1016/j.ecoenv.2021.112336"
"   Egamberdieva D, 2018, ENVIRON SUSTAIN, V1, P19, DOI [10.1007/s42398-018-0010-6, 10.1007/s42398-018-0010-6, DOI 10.1007/S42398-018-0010-6]"
"   Gautam K, 2023, ENVIRON RES, V225, DOI 10.1016/j.envres.2023.115592"
"   Geng N, 2022, ECOTOX ENVIRON SAFE, V232, DOI 10.1016/j.ecoenv.2022.113241"
"   GIANFREDA L, 1993, PESTIC SCI, V39, P237, DOI 10.1002/ps.2780390310"
"   Grice EA, 2009, SCIENCE, V324, P1190, DOI 10.1126/science.1171700"
"   Hale L, 2015, SOIL BIOL BIOCHEM, V81, P228, DOI 10.1016/j.soilbio.2014.11.023"
"   Hou RJ, 2021, J HAZARD MATER, V415, DOI 10.1016/j.jhazmat.2021.125650"
"   Huang L, 2023, J FUNGI, V9, DOI 10.3390/jof9080792"
"   Iravani S, 2020, ACS SUSTAIN CHEM ENG, V8, P5395, DOI 10.1021/acssuschemeng.0c00292"
"   Kamali M, 2022, CHEM ENG J, V428, DOI 10.1016/j.cej.2021.131189"
"   KANDELER E, 1988, BIOL FERT SOILS, V6, P68, DOI 10.1007/BF00257924"
"   Kim SR, 2007, J MICROBIOL METH, V70, P132, DOI 10.1016/j.mimet.2007.04.003"
"   Kumar A, 2021, CHEMOSPHERE, V284, DOI 10.1016/j.chemosphere.2021.131325"
"   Kumar S, 2019, ENVIRON RES, V179, DOI 10.1016/j.envres.2019.108792"
"   Largeteau ML, 2011, APPL MICROBIOL BIOT, V92, P897, DOI 10.1007/s00253-011-3630-7"
"   Lehmann J, 2011, SOIL BIOL BIOCHEM, V43, P1812, DOI 10.1016/j.soilbio.2011.04.022"
"   Li CC, 2020, SCI TOTAL ENVIRON, V749, DOI 10.1016/j.scitotenv.2020.141555"
"   Li JH, 2010, J ENVIRON MONITOR, V12, P466, DOI 10.1039/b916389a"
"   Li X, 2016, RSC ADV, V6, P65816, DOI 10.1039/c6ra11767h"
"   Liu GH, 2016, WASTE BIOMASS VALORI, V7, P1469, DOI 10.1007/s12649-016-9534-0"
"   Liu HY, 2015, J HAZARD MATER, V294, P121, DOI 10.1016/j.jhazmat.2015.04.004"
"   Ma Y, 2016, J HAZARD MATER, V320, P36, DOI 10.1016/j.jhazmat.2016.08.009"

"   Mai XR, 2025, J ENVIRON SCI, V149, P1, DOI 10.1016/j.jes.2024.01.045"
"   Masoudzadeh N, 2011, J HAZARD MATER, V197, P190, DOI 10.1016/j.jhazmat.2011.09.075"
"   Mokrani S, 2024, ECOL ENG, V207, DOI 10.1016/j.ecoleng.2024.107338"
"   Nannipieri P, 2018, BIOL FERT SOILS, V54, P11, DOI 10.1007/s00374-017-1245-6"
"   Ouyang P, 2023, BIOCHAR, V5, DOI 10.1007/s42773-023-00265-3"
"   Rajendran S, 2022, CHEMOSPHERE, V287, DOI 10.1016/j.chemosphere.2021.132369"
"   Rayment GE., 1992, The Australian Laboratory Handbook of Soil and Water Chemical Methods"
"   ROSS DJ, 1992, SOIL BIOL BIOCHEM, V24, P343, DOI 10.1016/0038-0717(92)90194-3"
"   Sellami K, 2022, SCI TOTAL ENVIRON, V806, DOI 10.1016/j.scitotenv.2021.150500"
"   Sharma P, 2022, Cleaner Chemical Engineering, V2, P100020, DOI [10.1016/j.clce.2022.100020, 10.1016/j.clce.2022.100020, DOI 10.1016/J.CLCE.2022.100020]"
"   Shen TT, 2015, RSC ADV, V5, P58704, DOI 10.1039/c5ra06686g"
"   Sivaram AK, 2023, ADV AGRON, V180, P35, DOI 10.1016/bs.agron.2023.03.001"
"   Sultan H, 2024, J ENVIRON MANAGE, V355, DOI 10.1016/j.jenvman.2024.120448"
"   Tabatabai M. A., 1969, Soil Biology and Biochemistry, V1, P301, DOI 10.1016/0038-0717(69)90012-1"
"   Vessey JK, 2003, PLANT SOIL, V255, P571, DOI 10.1023/A:1026037216893"
"   Wang GL, 2023, J HAZARD MATER, V443, DOI 10.1016/j.jhazmat.2022.130148"
"   Wang L, 2020, NANOSCALE, V12, P4790, DOI 10.1039/c9nr09274a"
"   Wang YS, 2017, COMPOS PART B-ENG, V121, P9, DOI 10.1016/j.compositesb.2017.06.023"
"   Wei Z, 2024, J ENVIRON SCI, V138, P350, DOI 10.1016/j.jes.2023.04.008"
"   Wu B, 2016, SCI TOTAL ENVIRON, V562, P732, DOI 10.1016/j.scitotenv.2016.04.079"
"   Xiang XZ, 2023, J HAZARD MATER, V443, DOI 10.1016/j.jhazmat.2022.130247"
"   Xiao KM, 2017, ACS APPL MATER INTER, V9, P30448, DOI 10.1021/acsami.7b09520"
"   Xiao KM, 2016, RSC ADV, V6, P86068, DOI 10.1039/c6ra14417a"
"   Xu L, 2019, FOOD CHEM, V285, P406, DOI 10.1016/j.foodchem.2019.01.179"
"   Xu S, 2024, CHEM ENG J, V482, DOI 10.1016/j.cej.2024.149170"
"   Xue SG, 2023, CHEM ENG J, V473, DOI 10.1016/j.cej.2023.145499"
"   Yang Y, 2022, J FUNGI, V8, DOI 10.3390/jof8020162"
"   Yin K, 2019, CHEM ENG J, V360, P1553, DOI [10.1016/j.cej.2018.10.226, 10.1016/j.jhazmat.2024.134410]"
"   Zhang GX, 2016, ENVIRON POLLUT, V218, P513, DOI 10.1016/j.envpol.2016.07.031"
"   Zhang HR, 2016, J ENVIRON SCI, V47, P7, DOI 10.1016/j.jes.2015.12.023"
"   Zheng XJ, 2024, ECOTOX ENVIRON SAFE, V284, DOI 10.1016/j.ecoenv.2024.116883"
"   Zheng XM, 2022, J HAZARD MATER, V438, DOI 10.1016/j.jhazmat.2022.129557"
"   Zulfiqar U, 2023, PLANTS-BASEL, V12, DOI 10.3390/plants12173147"
NR 74
TC 2
Z9 2
U1 23
U2 42
PU ELSEVIER SCI LTD
PI London
"PA 125 London Wall, London, the United Kingdom"
SN 2213-2929
EI 2213-3437
J9 J ENVIRON CHEM ENG
JI J. Environ. Chem. Eng.
PD JUN
PY 2025
VL 13
IS 3
AR 116512
DI 10.1016/j.jece.2025.116512
EA APR 2025
PG 10
"WC Engineering, Environmental; Engineering, Chemical"
WE Science Citation Index Expanded (SCI-EXPANDED)
SC Engineering
GA 1JR3D
UT WOS:001466624600001
DA 2026-04-06
ER

PT J
"AU Iimaa, T"
"   Batmunkh, M"
"   Dulguun, B"
"   Dorjsuren, B"
"   Turmunkh, T"
"   Tserennadmid, E"
"   Surenjav, U"
"   Choidash, B"
"   Gereltuya, R"
"AF Iimaa, Tuyajargal"
"   Batmunkh, Munkhjin"
"   Dulguun, Batbold"
"   Dorjsuren, Batsuren"
"   Turmunkh, Telmen"
"   Tserennadmid, Enkhjargal"
"   Surenjav, Unursaikhan"
"   Choidash, Battsetseg"
"   Gereltuya, Renchinkhand"
TI Bacterial Heavy Metal Resistance in Contaminated Soil
SO JOURNAL OF MICROBIOLOGY AND BIOTECHNOLOGY
LA English

DT Review
DE Resistance; microorganisms; bioremediation; heavy metals; plasmids
ID BIOREMEDIATION; DETOXIFICATION; DIVERSITY; MICROORGANISMS; TECHNOLOGIES;
COMMUNITIES; REMOVAL; IMPACTS; GENES; WASTE
"AB Soil heavy metal contamination poses a significant global threat to both environmental and human health. The accumulation of heavy metals in the Earth's crust, driven by urbanization, industrialization, agricultural practices, improper waste disposal, and mining, triggers harmful ecological cascades. Microorganisms, particularly bacteria, play a vital role in the decomposition and detoxification of these contaminants. In contaminated environments, bacteria exhibit resistance to heavy metals through various strategies, including the production of metal-chelating molecules, alterations to cell surface properties, efflux pumps, and activation of detoxification pathways. Notable microbial species such as Bacillus, Enterobacter, Pseudomonas, Aspergillus, and Penicillium show promising potential for bioremediation efforts. Harnessing bacterial resistance to heavy metals offers a costeffective and sustainable approach to mitigate the adverse impacts of contamination. This review explores the mechanisms of heavy metal resistance in bacteria, the role of soil microbiota, and the implications for bioremediation strategies. This review emphasizes the critical importance of bacterial resistance in addressing soil contamination and highlight the need for further research to elucidate underlying mechanisms and enhance bioremediation applications in this urgent global challenge."
"C1 [Iimaa, Tuyajargal] Mongolian Natl Univ Med Sci, Div Grad Educ Policy & Management, Ulaanbaatar 14210, Mongolia."
" [Batmunkh, Munkhjin; Dulguun, Batbold; Tserennadmid, Enkhjargal; Surenjav, Unursaikhan] Minist Hlth, Natl Ctr Publ Hlth, Dept Publ Hlth Reference Lab, Ulaanbaatar 13381, Mongolia."
" [Dorjsuren, Batsuren] Natl Univ Mongolia, Sch Engn & Technol, Dept Environm & Forest Engn, Lab Air & Environm Monitoring, Ulaanbaatar 14201, Mongolia."
" [Turmunkh, Telmen] Korea Univ, Dept Earth & Environm Sci, Seoul 02841, South Korea."
" [Choidash, Battsetseg; Gereltuya, Renchinkhand] Natl Univ Mongolia, Sch Arts & Sci, Dept Biol, Ulaanbaatar 14201, Mongolia."
C3 Mongolian National University of Medical Sciences; Ministry of Health
Mongolia; National University of Mongolia; Korea University; National
University of Mongolia
"RP Gereltuya, R (corresponding author), Natl Univ Mongolia, Sch Arts & Sci, Dept Biol, Ulaanbaatar 14201, Mongolia."
EM renchinkhand@num.edu.mn
"RI Dorjsuren, Batsuren/Q-6986-2018"
"OI Turmunkh, Telmen/0000-0002-7704-4891; Iimaa,"
Tuyajargal/0000-0002-3175-5110
FU Mongolian Foundation for Science and Technology (MFST)
" [ShUTBIHHZG-2022/143, TWN-02-2022/02]"
FX The team acknowledges the Mongolian Foundation for Science and
Technology (MFST) financial support for providing the Grant
" (No.ShUTBIHHZG-2022/143, TWN-02-2022/02) during the research."
"CR Abbas SZ., 2014, J. Waste Manag., V2014, P1, DOI [10.1155/2014/160398, DOI 10.1155/2014/160398]"
" Abhijit Gupta Abhijit Gupta, 2016, Journal of Microbial and Biochemical Technology, V8, P364, DOI 10.4172/1948-5948.1000310"
" Adl SM, 2019, J EUKARYOT MICROBIOL, V66, P4, DOI 10.1111/jeu.12691"
" Ahmed Shabbir, 2018, Biotechnologia (Poznan), V99, P137, DOI [10.5114/bta.2018.75657, 10.5114/bta.2018.75657]"
" Alboghobeish H, 2014, J ENVIRON HEALTH SCI, V12, DOI 10.1186/2052-336X-12-44"
" Ali S., 2013, Greener Journal of Environmental Management and Public Safety, V2, P172, DOI [10.15580/GJEMPS.2013.4.060413652, DOI 10.15580/GJEMPS.2013.4.060413652]"
" Alloway B.J., 1999, CADMIUM SOILS PLANTS, P97, DOI [DOI 10.1007/978-94-011-4473-5_5, 10.1007/978-94-011-4473-5_5]"
" Altimira F, 2012, BMC MICROBIOL, V12, DOI 10.1186/1471-2180-12-193"
" Andrade-Linares DR, 2021, SCI TOTAL ENVIRON, V780, DOI 10.1016/j.scitotenv.2021.146672"
" Andreazza R, 2011, BRAZ J MICROBIOL, V42, P66, DOI 10.1590/S1517-83822011000100009"
" Aryal M, 2021, REV CHEM ENG, V37, P715, DOI 10.1515/revce-2019-0016"
" Atibu EK, 2023, INT J ENVIRON RES, V17, DOI 10.1007/s41742-023-00524-y"
" Atikpo E, 2017, J. Niger. Assoc. Math Phys., V41, P339"
" Atuchin VV, 2023, MICROORGANISMS, V11, DOI 10.3390/microorganisms11040864"
" Baquero F, 2013, FRONT MICROBIOL, V4, DOI 10.3389/fmicb.2013.00015"
" Bardgett RD, 2014, NATURE, V515, P505, DOI 10.1038/nature13855"
" Bhatti AA, 2017, MICROB PATHOGENESIS, V111, P458, DOI 10.1016/j.micpath.2017.09.036"
" Bouchez T, 2000, ENVIRON MICROBIOL, V2, P179"
" Braga RM, 2016, BRAZ J MICROBIOL, V47, P86"
" Branco R, 2008, BMC MICROBIOL, V8, DOI 10.1186/1471-2180-8-95"
" Butt HA, 2016, J TEKNOL, V78, P47"
" Cai LM, 2019, SCI TOTAL ENVIRON, V650, P725, DOI 10.1016/j.scitotenv.2018.09.081"
" Chatterjee S, 2020, METALLOMICS, V12, P1637, DOI 10.1039/d0mt00140f"
" CHAUDHARY H.S., 2013, Journal of Applied Pharmaceutical Science, V3, pS83, DOI DOI 10.7324/JAPS.2013.38.S14"
" Chen H., 2023, PREPRINT"
" Chonokhuu S, 2019, INT J ENV RES PUB HE, V16, DOI 10.3390/ijerph16142552"
" Chu D, 2018, IOP C SER EARTH ENV, V113, DOI 10.1088/1755-1315/113/1/012009"
" Clemens S, 2003, NEW PHYTOL, V159, P323, DOI 10.1046/j.1469-8137.2003.00811.x"
" Creamer RE, 2022, SOIL BIOL BIOCHEM, V166, DOI 10.1016/j.soilbio.2022.108561"
" Das S, 2014, ELSEV INSIGHT, P1, DOI 10.1016/B978-0-12-800021-2.00001-7"
" Delgado-Baquerizo M, 2014, J ECOL, V102, P1592, DOI 10.1111/1365-2745.12303"
" Dixit R, 2015, SUSTAINABILITY-BASEL, V7, P2189, DOI 10.3390/su7022189"
" Dobrovol'skaya TG, 2015, EURASIAN SOIL SCI+, V48, P959, DOI 10.1134/S1064229315090033"
" Durve A., 2012, ADV APPL SCI RES, V3, P2801"
" El Baz Soraia, 2015, ScientificWorldJournal, V2015, P761834, DOI 10.1155/2015/761834"
" Elbert W, 2012, NAT GEOSCI, V5, P459, DOI 10.1038/ngeo1486"
" Elnahas MO, 2021, POLYSACCHARIDES-BASE, V2, P47, DOI 10.3390/polysaccharides2010004"
" Fakhar A, 2022, CRIT REV ENV SCI TEC, V52, P1868, DOI 10.1080/10643389.2020.1863112"

"   Fang L, 2024, FRONT CELL INFECT MI, V14, DOI 10.3389/fcimb.2024.1472876"
"   Farda B, 2022, MICROORGANISMS, V10, DOI 10.3390/microorganisms10020453"
"   Franke S, 2001, MICROBIOL-SGM, V147, P965, DOI 10.1099/00221287-147-4-965"
"   Gadd GM, 2004, GEODERMA, V122, P109, DOI 10.1016/j.geoderma.2004.01.002"
"   Garbisu C, 2017, FRONT MICROBIOL, V8, DOI 10.3389/fmicb.2017.01966"
"   Gaurav A, 2023, MICROBIOL-SGM, V169, DOI 10.1099/mic.0.001333"
"   Geisen S, 2017, SOIL BIOL BIOCHEM, V111, P94, DOI 10.1016/j.soilbio.2017.04.001"
"   Henao SG, 2021, FRONT ENV SCI-SWITZ, V9, DOI 10.3389/fenvs.2021.604216"
"   Gullberg E, 2014, MBIO, V5, DOI 10.1128/mBio.01918-14"
"   Gupta Pratima, 2017, Biotechnol Rep (Amst), V13, P58, DOI [10.1016/j.btre.2016.12.006, 10.1016/j.btre.2016.12.006]"
"   Hoorman JJ, 2011, OSU Extension, P1"
"   Hou SN, 2019, ENVIRON INT, V128, P430, DOI 10.1016/j.envint.2019.04.046"
"   Hu Shi-lei, 2023, Huanjing Kexue, V44, P336, DOI 10.13227/j.hjkx.202202175"
"   Islam M. S., 2022, SOIL HLTH ENV SUSTAI, DOI [DOI 10.1007/978-3-031-09270-1_27, 10.1007/978-3-031-09270-1_27]"
"   Jiang B, 2019, J HAZARD MATER, V362, P187, DOI 10.1016/j.jhazmat.2018.08.060"
"   Johnson H, 2019, Computational Molecular Bioscience, V09, P1, DOI [10.4236/cmb.2019.91001, 10.4236/cmb.2019.91001, DOI 10.4236/CMB.2019.91001]"
"   Kalaimurugan D, 2020, INT J ENVIRON SCI TE, V17, P1455, DOI 10.1007/s13762-019-02563-5"
"   Kamala-Kannan S., 2008, BIOTECHNOLOGY, V7, P149, DOI [10.3923/biotech.2008.149.152, DOI 10.3923/BIOTECH.2008.149.152]"
"   Khalid N, 2018, B ENVIRON CONTAM TOX, V100, P250, DOI 10.1007/s00128-017-2247-7"
"   Kotrba P, 1999, APPL ENVIRON MICROB, V65, P1092"
"   Kumar SA, 2022, International Journal of Plant & Soil Science, P62, DOI [10.9734/ijpss/2022/v34i1831055, 10.9734/ijpss/2022/v34i1831055, DOI 10.9734/IJPSS/2022/V34I1831055]"
"   Kumar V., 2022, MICROBIAL EXTREMOZYM, P275, DOI DOI 10.1016/B978-0-12-822945-3.00003-8"
"   Lakzian A, 2008, ASIAN J CHEM, V20, P6075"
"   Li ZJ, 2006, APPL MICROBIOL BIOT, V73, P458, DOI 10.1007/s00253-006-0473-8"
"   Megharaj M, 2011, ENVIRON INT, V37, P1362, DOI 10.1016/j.envint.2011.06.003"
"   MERGEAY M, 1985, J BACTERIOL, V162, P328, DOI 10.1128/JB.162.1.328-334.1985"
"   Mishra M., 2021, Woodhead publishing series in Food Science, Technology and Nutrition, P47, DOI DOI 10.1016/B978-0-12-821199-1.00005-5"
"   MISHRA S., 2019, Environmental biotechnology: For sustainable future, P103, DOI [DOI 10.1007/978-981-10-7284-0_5, DOI 10.1007/978-981-10-7284-05]"
"   Mishra V, 2016, INT J PHYTOREMEDIAT, V18, P697, DOI 10.1080/15226514.2015.1131231"
"   Nadzir MM, 2021, POLYMERS-BASEL, V13, DOI 10.3390/polym13040530"
"   Nath S, 2018, INT MICROBIOL, V21, P35, DOI 10.1007/s10123-018-0003-4"
"   Nguyen CC, 2019, FRONT ENV SCI ENG, V13, DOI 10.1007/s11783-019-1129-0"
"   Nies DH, 1999, APPL MICROBIOL BIOT, V51, P730, DOI 10.1007/s002530051457"
"   Nirbhavane H M, 2018, Indian J. Microbiol. Res., V5, P229"
"   Oladipo OG, 2018, BRAZ J MICROBIOL, V49, P29, DOI 10.1016/j.bjm.2017.06.003"
"   Olaniran AO, 2013, INT J MOL SCI, V14, P10197, DOI 10.3390/ijms140510197"
"   Qiao WC, 2019, ROY SOC OPEN SCI, V6, DOI 10.1098/rsos.181701"
"   Qin WT, 2019, FRONT MICROBIOL, V10, DOI 10.3389/fmicb.2019.00278"
"   Qishlaqi A., 2007, American-Eurasian Journal of Agricultural and Environmental Science, V2, P565"
"   Radwan SS, 2019, SCI REP-UK, V9, DOI 10.1038/s41598-019-56099-2"
"   Rahman Z, 2020, J HAZARD MATER, V396, DOI 10.1016/j.jhazmat.2020.122682"
"   Raj KK, 2018, CARBOHYD POLYM, V199, P353, DOI 10.1016/j.carbpol.2018.07.037"
"   Rasulov B.A., 2013, J. Environ. Prot, V4, P989, DOI DOI 10.4236/JEP.2013.49114"
"   Riyam NK, 2022, Environmental Impact and Remediation of Heavy Metals"
"   Rodr??guez-S??nchez V, 2017, WORLD J MICROB BIOT, V33, DOI 10.1007/s11274-017-2314-6"
"   Rosen BP, 2002, COMP BIOCHEM PHYS A, V133, P689, DOI 10.1016/S1095-6433(02)00201-5"
"   Saha J, 2023, BIOLOGIA, V78, P2975, DOI 10.1007/s11756-023-01465-9"
"   Salehizadeh H, 2003, WATER RES, V37, P4231, DOI 10.1016/S0043-1354(03)00418-4"
"   Saranya K, 2017, BIOMED RES INT, V2017, DOI 10.1155/2017/6509648"
"   Sevim E, 2015, Suleyman Demirel Univ. Fen Bilim. Enst. Deg., V19, DOI [10.19113/sdufbed.22055, DOI 10.19113/SDUFBED.22055]"
"   Singh RK, 2011, J ANTIBIOT, V64, P401, DOI 10.1038/ja.2011.21"
"   Smith E, 2015, INT BIODETER BIODEGR, V101, P56, DOI 10.1016/j.ibiod.2015.03.029"
"   Song LC, 2022, CHEMOSPHERE, V307, DOI 10.1016/j.chemosphere.2022.136084"
"   Sreedevi PR, 2022, J WATER PROCESS ENG, V48, DOI 10.1016/j.jwpe.2022.102884"
"   Staninska-Pieta J, 2020, MOLECULES, V25, DOI 10.3390/molecules25020319"
"   Su JQ, 2015, ENVIRON SCI TECHNOL, V49, P7356, DOI 10.1021/acs.est.5b01012"
"   Tecon R, 2017, FEMS MICROBIOL REV, V41, P599, DOI 10.1093/femsre/fux039"
"   Thatoi H., 2021, Bioprospecting of Enzymes in Industry, Healthcare and Sustainable Environment, P29, DOI [10.1007/978-981-33-4195-1, DOI 10.1007/978-981-33-4195-1]"
"   Titah HS, 2018, INT J MICROBIOL, V2018, DOI 10.1155/2018/3101498"
"   Verma JP, 2016, FRONT MICROBIOL, V6, DOI [10.3389/fmicb.2015.01555, DOI 10.3389/FMICB.2015.01555, 10.3389/fmicb.2015.01555]"
"   Verma S, 2023, CLEAN TECHNOL ENVIR, V25, P1485, DOI 10.1007/s10098-021-02029-8"
"   Viti C, 2014, FEMS MICROBIOL REV, V38, P633, DOI 10.1111/1574-6976.12051"
"   von Rozycki T, 2009, ANTON LEEUW INT J G, V96, P115, DOI 10.1007/s10482-008-9284-5"
"   Weisberg AJ, 2023, ANNU REV MICROBIOL, V77, P603, DOI 10.1146/annurev-micro-032521-022006"
"   Welbaum GE, 2004, CRIT REV PLANT SCI, V23, P175, DOI 10.1080/07352680490433295"
"   Wessel AK, 2013, NAT REV MICROBIOL, V11, P337, DOI 10.1038/nrmicro3010"
"   White C, 1997, FEMS MICROBIOL REV, V20, P503, DOI 10.1016/S0168-6445(97)00029-6"
"   Wuana R.A., 2011, ISRN Ecology, DOI [10.5402/2011/402647, DOI 10.5402/2011/402647]"
"   Wyatt TT, 2013, ADV APPL MICROBIOL, V85, P43, DOI 10.1016/B978-0-12-407672-3.00002-2"
"   Yang L, 2018, SOIL BIOL BIOCHEM, V122, P131, DOI 10.1016/j.soilbio.2018.04.017"
"   Yang RQ, 2024, ANTIOXIDANTS-BASEL, V13, DOI 10.3390/antiox13070825"
"   Yao ZT, 2012, PROCEDIA ENVIRON SCI, V16, P722, DOI 10.1016/j.proenv.2012.10.099"
"   Yin K, 2019, CHEM ENG J, V360, P1553, DOI [10.1016/j.cej.2018.10.226, 10.1016/j.jhazmat.2024.134410]"
"   Zglobicki W, 2018, ENVIRON EARTH SCI, V77, DOI 10.1007/s12665-018-7969-2"

"   Zhu YG, 2013, P NATL ACAD SCI USA, V110, P3435, DOI 10.1073/pnas.1222743110"
NR 113
TC 4
Z9 5
U1 20
U2 28
PU KOREAN SOC MICROBIOLOGY & BIOTECHNOLOGY
PI SEOUL
"PA KOREA SCI TECHNOL CENTER #402, 635-4 YEOGSAM-DONG, KANGNAM-GU, SEOUL,"
SOUTH KOREA
SN 1017-7825
EI 1738-8872
J9 J MICROBIOL BIOTECHN
JI J. Microbiol. Biotechnol.
PY 2025
VL 35
AR e2411073
DI 10.4014/jmb.2411.11073
PG 13
WC Biotechnology & Applied Microbiology; Microbiology
WE Science Citation Index Expanded (SCI-EXPANDED)
SC Biotechnology & Applied Microbiology; Microbiology
GA 5DI9U
UT WOS:001532996000005
PM 40659549
"OA Green Submitted, Bronze"
DA 2026-04-06
ER

PT J
"AU Sharma, JK"
"   Kumar, N"
"   Singh, NP"
"   Santal, AR"
"AF Sharma, Jitendra Kumar"
"   Kumar, Nitish"
"   Singh, N. P."
"   Santal, Anita Rani"
TI Phytoremediation technologies and their mechanism for removal of heavy
metal from contaminated soil: An approach for a sustainable environment
SO FRONTIERS IN PLANT SCIENCE
LA English
DT Review
DE phytoremediation; heavy metals; soil contamination; crops;
phytoextraction; metal-binding proteins
ID ARBUSCULAR MYCORRHIZAL FUNGI; PHYTOCHELATIN SYNTHASE GENE; CADMIUM
TOLERANCE; PLANT-GROWTH; EXPRESSION; ACCUMULATION; CD; TOXICITY;
BIOCHAR; REMEDIATION
"AB The contamination of soils with heavy metals and its associated hazardous effects are a thrust area of today's research. Rapid industrialization, emissions from automobiles, agricultural inputs, improper disposal of waste, etc., are the major causes of soil contamination with heavy metals. These contaminants not only contaminate soil but also groundwater, reducing agricultural land and hence food quality. These contaminants enter the food chain and have a severe effect on human health. It is important to remove these contaminants from the soil. Various economic and ecological strategies are required to restore the soils contaminated with heavy metals. Phytoremediation is an emerging technology that is non-invasive, cost-effective, and aesthetically pleasing. Many metal-binding proteins (MBPs) of the plants are significantly involved in the phytoremediation of heavy metals; the MBPs include metallothioneins; phytochelatins; metalloenzymes; metal-activated enzymes; and many metal storage proteins, carrier proteins, and channel proteins. Plants are genetically modified to enhance their phytoremediation capacity. In Arabidopsis, the expression of the mercuric ion-binding protein in Bacillus megaterium improves the metal accumulation capacity. The phytoremediation efficiency of plants is also enhanced when assisted with microorganisms, biochar, and/or chemicals. Removing heavy metals from agricultural land without challenging food security is almost impossible. As a result, crop selections with the ability to sequester heavy metals and provide food security are in high demand. This paper summarizes the role of plant proteins and plant-microbe interaction in remediating soils contaminated with heavy metals. Biotechnological approaches or genetic engineering can also be used to tackle the problem of heavy metal contamination."
"C1 [Sharma, Jitendra Kumar; Singh, N. P.] Maharshi Dayanand Univ, Ctr Biotechnol, Rohtak, India."
"   [Kumar, Nitish] Cent Univ South Bihar, Dept Biotechnol, Gaya, Bihar, India."
"   [Santal, Anita Rani] Maharshi Dayanand Univ, Dept Microbiol, Rohtak, India."
C3 Maharshi Dayanand University; Central University of South Bihar;
Maharshi Dayanand University
"RP Singh, NP (corresponding author), Maharshi Dayanand Univ, Ctr Biotechnol, Rohtak, India.; Santal, AR (corresponding author), Maharshi Dayanand Univ, Dept Microbiol, Rohtak, India."
EM npsingh.cbt@mdurohtak.ac.in; anita.gangotra@gmail.com
"RI ; Singh, Nater Pal/AAE-2596-2019; Santal, Anita/ABB-2652-2020; Sharma,"
Jitendra/ABD-9917-2021
"OI Kumar, Nitish/0000-0001-5479-0755; Singh, Nater Pal/0000-0003-3778-8711;"

"CR AbdElgawad H, 2021, J HAZARD MATER, V417, DOI 10.1016/j.jhazmat.2021.126055"
"   AHLFELD DP, 1994, J WATER RES PL-ASCE, V120, P350, DOI 10.1061/(ASCE)0733-9496(1994)120:3(350)"
"   Ahmad M, 2014, CHEMOSPHERE, V99, P19, DOI 10.1016/j.chemosphere.2013.10.071"
"   Alengebawy A, 2021, TOXICS, V9, DOI 10.3390/toxics9030042"
"   Andr??s P, 2016, WEB ECOL, V16, P81, DOI 10.5194/we-16-81-2016"
"   [Anonymous], 2018, BIORXIV, DOI DOI 10.1101/379511"

"   Antoniadis V, 2021, ENVIRON INT, V146, DOI 10.1016/j.envint.2020.106233"
"   Azhar U, 2022, ENVIRON RES, V214, DOI 10.1016/j.envres.2022.113918"
"   Bahraminia M, 2016, INT J PHYTOREMEDIAT, V18, P730, DOI 10.1080/15226514.2015.1131242"
"   Banerjee Aditya, 2022, Vegetos, V35, P1158, DOI [10.1007/s42535-022-00405-3, 10.1007/s42535-022-00405-3]"
"   Berglund M, 2015, ENVIRON RES, V141, P69, DOI 10.1016/j.envres.2014.09.042"
"   Bolan NS, 2011, ADV AGRON, V112, P145, DOI 10.1016/B978-0-12-385538-1.00004-4"
"   Brewer CE, 2014, BIOMASS BIOENERG, V66, P176, DOI 10.1016/j.biombioe.2014.03.059"
"   Brunetti G, 2012, J PLANT INTERACT, V7, P160, DOI 10.1080/17429145.2011.603438"
"   Brunetti P, 2015, J EXP BOT, V66, P3815, DOI 10.1093/jxb/erv185"
"   Cao FB, 2020, CHEMOSPHERE, V240, DOI 10.1016/j.chemosphere.2019.124907"
"   Chandramohan Rajeev, 2015, AIAA Guidance, Navigation and Control Conference, P1"
"   Chang JD, 2022, PLANT SOIL, V476, P79, DOI 10.1007/s11104-022-05323-6"
"   Chang JD, 2020, PLANT CELL ENVIRON, V43, P2476, DOI 10.1111/pce.13843"
"   Chaudhary K, 2018, FUNG BIOL-US, P39, DOI 10.1007/978-3-319-77386-5_2"
"   Chen D., 2018, J ENVIRON MANAGE, V222, P76, DOI DOI 10.1016/j.jenvman.2018.05.004"
"   Chen QQ, 2020, SELENIUM RESEARCH FOR ENVIRONMENT AND HUMAN HEALTH: PERSPECTIVES, TECHNOLOGIES AND ADVANCEMENTS, P25"
"   Chia J.C., 2021, Biodegradation Technology of Organic and Inorganic Pollutants, P1, DOI DOI 10.5772/INTECHOPEN.99077"
"   Dada E. O., 2015, Ethiopian Journal of Environmental Studies and Management, V8, P606, DOI [10.4314/ejesm.v8i5.13, 10.4314/ejesm.v8i5.13]"
"   de Souza TD, 2019, CHEMOSPHERE, V234, P402, DOI 10.1016/j.chemosphere.2019.06.004"
"   Del Bubba M, 2013, J HAZARD MATER, V262, P394, DOI 10.1016/j.jhazmat.2013.08.073"
"   Din BU, 2020, PLANT PHYSIOL BIOCH, V146, P249, DOI 10.1016/j.plaphy.2019.11.010"
"   Drozdova I, 2019, APPL GEOCHEM, V108, DOI 10.1016/j.apgeochem.2019.104377"
"   Ennis CJ, 2012, CRIT REV ENV SCI TEC, V42, P2311, DOI 10.1080/10643389.2011.574115"
"   Feki K, 2021, ENVIRON SCI POLLUT R, V28, P64967, DOI 10.1007/s11356-021-16805-y"
"   Gao CH, 2022, BIOL RES, V55, DOI 10.1186/s40659-021-00368-w"
"   Ghosh D, 2021, INT J PHYTOREMEDIAT, V23, P559, DOI 10.1080/15226514.2020.1840510"
"   Ghosh M., 2005, Applied Ecology and Environmental Research, V3, P1"
"   Gong XM, 2019, SCI TOTAL ENVIRON, V666, P1126, DOI 10.1016/j.scitotenv.2019.02.215"
"   Gorjanovic S, 2004, ENVIRON CHEM LETT, V2, P113, DOI 10.1007/s10311-004-0084-5"
"   Gouiaa S, 2019, ENVIRON SCI POLLUT R, V26, P37037, DOI 10.1007/s11356-019-06852-x"
"   Grill E., 2007, ENV BIOREMEDIATION T, P101, DOI DOI 10.1007/978-3-540-34793-4_5"
"   Guarino F, 2020, CHEMOSPHERE, V251, DOI 10.1016/j.chemosphere.2020.126310"
"   Hartmann T, 2004, PLANTA, V219, P1, DOI 10.1007/s00425-004-1249-y"
"   Hossain MM, 2018, INT J PHYTOREMEDIAT, V20, P796, DOI 10.1080/15226514.2018.1425669"
"   Hsieh JL, 2009, J HAZARD MATER, V161, P920, DOI 10.1016/j.jhazmat.2008.04.079"
"   Jabeen R, 2009, BOT REV, V75, P339, DOI 10.1007/s12229-009-9036-x"
"   Jach ME, 2022, BIOLOGY-BASEL, V11, DOI 10.3390/biology11050676"
"   Jia Y, 2012, ENVIRON SCI TECHNOL, V46, P8090, DOI 10.1021/es300499a"
"   Jin DM, 2022, ACTA PHYSIOL PLANT, V44, DOI 10.1007/s11738-022-03451-1"
"   Jin ZM, 2019, SCI TOTAL ENVIRON, V697, DOI 10.1016/j.scitotenv.2019.134148"
"   Joshi R, 2016, PLANT METAL INTERACTION: EMERGING REMEDIATION TECHNIQUES, P239, DOI 10.1016/B978-0-12-803158-2.00009-6"
"   Ju WL, 2019, ECOTOX ENVIRON SAFE, V167, P218, DOI 10.1016/j.ecoenv.2018.10.016"
"   Kang YC, 2022, J HAZARD MATER, V425, DOI 10.1016/j.jhazmat.2021.127875"
"   Khanna K, 2023, J PLANT GROWTH REGUL, V42, P6474, DOI 10.1007/s00344-022-10879-9"
"   Kim YN, 2011, PLANT BIOTECHNOL REP, V5, P323, DOI 10.1007/s11816-011-0186-z"
"   Kintlov?? M, 2021, FRONT PLANT SCI, V12, DOI 10.3389/fpls.2021.629089"
"   Kumar B, 2017, ARAB J CHEM, V10, pS2335, DOI 10.1016/j.arabjc.2013.08.010"
"   Kumar K, 2023, J PLANT BIOCHEM BIOT, V32, P12, DOI 10.1007/s13562-022-00776-3"
"   Kumar S, 2018, FRONT PLANT SCI, V9, DOI 10.3389/fpls.2018.00751"
"   Kumar U, 2022, J INDIAN CHEM SOC, V99, DOI 10.1016/j.jics.2021.100334"
"   Lanquar V, 2005, EMBO J, V24, P4041, DOI 10.1038/sj.emboj.7600864"
"   Leal-Alvarado DA, 2018, ECOTOX ENVIRON SAFE, V147, P1056, DOI 10.1016/j.ecoenv.2017.09.046"
"   Leung Molly., 2004, BIOTEACH J, V2, P18"
"   Li AM, 2009, INT J MOL SCI, V10, P3269, DOI 10.3390/ijms10083269"
"   Li Can, 2022, E3S Web of Conferences, V350, DOI [10.1051/e3sconf/202235001006, 10.1051/e3sconf/202235001006]"
"   Li CF, 2019, SOIL SEDIMENT CONTAM, V28, P380, DOI 10.1080/15320383.2019.1592108"
"   Li HC, 2023, ENVIRON SCI POLLUT R, V30, P3774, DOI 10.1007/s11356-022-22470-6"
"   Li Q, 2021, ECOTOX ENVIRON SAFE, V212, DOI 10.1016/j.ecoenv.2021.112016"
"   Liu H, 2015, APPL SOIL ECOL, V89, P44, DOI 10.1016/j.apsoil.2015.01.006"
"   Liu SL, 2019, J HAZARD MATER, V371, P233, DOI 10.1016/j.jhazmat.2019.03.016"
"   Losada Benavides L. C., 2018, International Journal of Applied Environmental Sciences, V13, P9"
"   Lu KP, 2017, J ENVIRON MANAGE, V186, P285, DOI 10.1016/j.jenvman.2016.05.068"
"   Luo JS, 2021, CROP J, V9, P521, DOI 10.1016/j.cj.2021.02.001"
"   Ma Y, 2016, FRONT PLANT SCI, V7, DOI 10.3389/fpls.2016.00918"
"   Madanan MT, 2021, ENVIRON CHEM ECOTOX, V3, P17, DOI 10.1016/j.enceco.2020.10.007"
"   Mahardika G, 2018, IOP C SER EARTH ENV, V106, DOI 10.1088/1755-1315/106/1/012120"
"   Mahdavian Kobra, 2022, Proceedings of the Indian National Science Academy Part B Biological Sciences, V92, P319, DOI [10.1007/s40011-021-01345-9, 10.1007/s40011-021-01345-9]"
"   Manzoor M, 2021, CHEMOSPHERE, V277, DOI 10.1016/j.chemosphere.2021.130243"
"   Marrugo-Negrete J, 2016, SCI TOTAL ENVIRON, V542, P809, DOI 10.1016/j.scitotenv.2015.10.117"
"   Mathur J., 2020, SUSTAINABLE SOLUTION, P335, DOI [DOI 10.1007/978-981-15-8636-113, DOI 10.1007/978-981-15-8636-1_13]"
"   Mazumdar K, 2015, ENVIRON SCI POLLUT R, V22, P701, DOI 10.1007/s11356-014-3377-7"
"   McGrath SP, 2002, ADV AGRON, V75, P1, DOI 10.1016/S0065-2113(02)75002-5"
"   Mej??re M, 2001, TRENDS BIOTECHNOL, V19, P67, DOI 10.1016/S0167-7799(00)01534-1"
"   Mello IS, 2020, ECOTOX ENVIRON SAFE, V202, DOI 10.1016/j.ecoenv.2020.110818"
"   Memon AR, 2009, ENVIRON SCI POLLUT R, V16, P162, DOI 10.1007/s11356-008-0079-z"
"   Mesa V, 2017, APPL ENVIRON MICROB, V83, DOI [10.1128/AEM.03411-16, 10.1128/aem.03411-16]"
"   Mills RF, 2012, PLOS ONE, V7, DOI 10.1371/journal.pone.0042640"

"   Mills RF, 2003, PLANT J, V35, P164, DOI 10.1046/j.1365-313X.2003.01790.x"
"   Mishra J, 2017, FRONT MICROBIOL, V8, DOI 10.3389/fmicb.2017.01706"
"   Moreira H, 2016, GEODERMA, V278, P1, DOI 10.1016/j.geoderma.2016.05.003"
"   Muthusaravanan S, 2018, ENVIRON CHEM LETT, V16, P1339, DOI 10.1007/s10311-018-0762-3"
"   Nayak AK, 2018, INT J PHYTOREMEDIAT, V20, P682, DOI 10.1080/15226514.2017.1413332"
"   Onyia PC, 2021, Geology Ecology and Landscapes, V5, P308, DOI [10.1080/24749508.2020.1809058, 10.1080/24749508.2020.1809058, DOI 10.1080/24749508.2020.1809058]"
"   Ovecka M, 2014, BIOTECHNOL ADV, V32, P73, DOI 10.1016/j.biotechadv.2013.11.011"
"   Ozyigit II, 2021, ENVIRON CHEM LETT, V19, P669, DOI 10.1007/s10311-020-01095-6"
"   Pandey SK, 2019, J HEALTH POLLUT, V9, DOI 10.5696/2156-9614-9.24.191206"
"   Park J, 2012, PLANT J, V69, P278, DOI 10.1111/j.1365-313X.2011.04789.x"
"   Rahimzadeh MR, 2017, CASP J INTERN MED, V8, P135, DOI 10.22088/cjim.8.3.135"
"   Rahman MM, 2022, GESUNDE PFLANZ, V74, P571, DOI 10.1007/s10343-022-00632-3"
"   Rajkumar M, 2012, BIOTECHNOL ADV, V30, P1562, DOI 10.1016/j.biotechadv.2012.04.011"
"   Repkina N, 2019, BIOL PLANTARUM, V63, P70, DOI 10.32615/bp.2019.009"
"   Rizaludin MS, 2021, METABOLITES, V11, DOI 10.3390/metabo11060357"
"   Rolfe SA, 2019, CURR OPIN MICROBIOL, V49, P73, DOI 10.1016/j.mib.2019.10.003"
"   Rolli E, 2021, ENVIRON MICROBIOL, V23, P5690, DOI 10.1111/1462-2920.15647"
"   Rono JK, 2021, CHEMOSPHERE, V265, DOI 10.1016/j.chemosphere.2020.129136"
"   Safronova VI, 2012, SYMBIOSIS, V58, P149, DOI 10.1007/s13199-012-0207-x"
"   SALT DE, 1995, BIO-TECHNOL, V13, P468, DOI 10.1038/nbt0595-468"
"   Sarwar N, 2017, CHEMOSPHERE, V171, P710, DOI 10.1016/j.chemosphere.2016.12.116"
"   Sasaki A, 2012, PLANT CELL, V24, P2155, DOI 10.1105/tpc.112.096925"
"   Seo SG, 2010, GENES GENOM, V32, P544, DOI 10.1007/s13258-010-0093-7"
"   Shah V, 2020, ENVIRON TECHNOL INNO, V18, DOI 10.1016/j.eti.2020.100774"
"   Sharma P, 2021, BIORESOURCE TECHNOL, V326, DOI 10.1016/j.biortech.2021.124750"
"   Sharma S, 2015, ENVIRON SCI POLLUT R, V22, P946, DOI 10.1007/s11356-014-3635-8"
"   Sheoran V, 2009, MINER ENG, V22, P1007, DOI 10.1016/j.mineng.2009.04.001"
"   Shiyab S, 2018, AGRICULTURE-BASEL, V8, DOI 10.3390/agriculture8020029"
"   Shukla D, 2013, PROTOPLASMA, V250, P1263, DOI 10.1007/s00709-013-0508-9"
"   Shukla D, 2012, PLANT CELL REP, V31, P1687, DOI 10.1007/s00299-012-1283-3"
"   Singh N.P., 2016, Phytoremediation, P131, DOI [10.1007/978-3-319-41811-7_8, DOI 10.1007/978-3-319-41811-7_8]"
"   Singh NP., 2015, Phytoremediation, P115, DOI DOI 10.1007/978-3-319-10969-5_10"
"   Su HX, 2020, PLANT PHYSIOL BIOCH, V155, P743, DOI 10.1016/j.plaphy.2020.08.012"
"   Suman J, 2018, FRONT PLANT SCI, V9, DOI 10.3389/fpls.2018.01476"
"   Sun LP, 2018, BIOCHEM BIOPH RES CO, V497, P997, DOI 10.1016/j.bbrc.2018.02.133"
"   Suthar V, 2014, ENVIRON MONIT ASSESS, V186, P3957, DOI 10.1007/s10661-014-3671-3"
"   Tabrizi L, 2015, INT J PHYTOREMEDIAT, V17, P1244, DOI 10.1080/15226514.2015.1045131"
"   Tahir M, 2022, ENVIRON SCI POLLUT R, V29, P6026, DOI 10.1007/s11356-021-16018-3"
"   Talebi M, 2019, CHEMOSPHERE, V230, P488, DOI 10.1016/j.chemosphere.2019.05.098"
"   Taufikurahman T, 2019, IOP C SER EARTH ENV, V308, DOI 10.1088/1755-1315/308/1/012020"
"   Terry N, 2000, ANNU REV PLANT PHYS, V51, P401, DOI 10.1146/annurev.arplant.51.1.401"
"   Tiwari M, 2022, J HAZARD MATER, V426, DOI 10.1016/j.jhazmat.2021.128100"
"   T??r??k A, 2015, J HAZARD MATER, V299, P371, DOI 10.1016/j.jhazmat.2015.06.042"
"   Ullah S, 2021, CHEMOSPHERE, V269, DOI 10.1016/j.chemosphere.2020.128728"
"   Uraguchi S, 2019, PLANTA, V250, P667, DOI 10.1007/s00425-019-03186-3"
"   Usman K, 2019, SCI REP-UK, V9, DOI 10.1038/s41598-019-42029-9"
"   Vangronsveld J, 2009, ENVIRON SCI POLLUT R, V16, P765, DOI 10.1007/s11356-009-0213-6"
"   Vershinina ZR, 2022, RUSS J PLANT PHYSL+, V69, DOI 10.1134/S1021443722040185"
"   Wang JX, 2020, J HAZARD MATER, V394, DOI 10.1016/j.jhazmat.2020.122505"
"   Wang LW, 2020, CRIT REV ENV SCI TEC, V50, P2724, DOI 10.1080/10643389.2019.1705724"
"   Wang W., 2019, Cadmium Toxicity:New Aspects in Human Disease, Rice Contamination, and Cytotoxicity, P115, DOI DOI 10.1007/978-981-13-3630-0_9"
"   Wong CKE, 2009, NEW PHYTOL, V181, P71, DOI 10.1111/j.1469-8137.2008.02638.x"
"   Woraharn S, 2021, WATER AIR SOIL POLL, V232, DOI 10.1007/s11270-021-05156-6"
"   Wu DZ, 2016, PLANT PHYSIOL, V172, P1899, DOI 10.1104/pp.16.01189"
"   Wu MX, 2018, J PLANT GROWTH REGUL, V37, P709, DOI 10.1007/s00344-017-9765-8"
"   Wu MX, 2018, ECOTOX ENVIRON SAFE, V162, P35, DOI 10.1016/j.ecoenv.2018.06.049"
"   Yan A, 2020, FRONT PLANT SCI, V11, DOI 10.3389/fpls.2020.00359"
"   Yang Q, 2021, CHEMOSPHERE, V269, DOI 10.1016/j.chemosphere.2020.129373"
"   Yang YR, 2016, SCI REP-UK, V6, DOI 10.1038/srep20469"
"   Yanitch A, 2020, INT J PHYTOREMEDIAT, V22, P1505, DOI 10.1080/15226514.2020.1785387"
"   Yu Y, 2018, J INTEGR AGR, V17, P54, DOI [10.1016/S2095-3119(16)61599-X, 10.1016/s2095-3119(16)61599-x]"
"   Zamora-Ledezma C, 2021, ENVIRON TECHNOL INNO, V22, DOI 10.1016/j.eti.2021.101504"
"   Zhan J, 2016, ENVIRON SCI POLLUT R, V23, P18524, DOI 10.1007/s11356-016-7037-y"
"   Zhang C, 2020, ECOTOX ENVIRON SAFE, V188, DOI 10.1016/j.ecoenv.2019.109756"
"   Zhang J, 2020, ENVIRON EXP BOT, V176, DOI 10.1016/j.envexpbot.2020.104121"
"   Zhao FJ, 2010, ANNU REV PLANT BIOL, V61, P535, DOI 10.1146/annurev-arplant-042809-112152"
"   Zhong LS, 2019, ENVIRON SCI POLLUT R, V26, P13311, DOI 10.1007/s11356-019-04702-4"
NR 150
TC 218
Z9 266
U1 68
U2 613
PU FRONTIERS MEDIA SA
PI LAUSANNE
"PA AVENUE DU TRIBUNAL FEDERAL 34, LAUSANNE, CH-1015, SWITZERLAND"
SN 1664-462X
J9 FRONT PLANT SCI
JI Front. Plant Sci.
PD JAN 27
PY 2023
VL 14

AR 1076876
DI 10.3389/fpls.2023.1076876
PG 13
WC Plant Sciences
WE Science Citation Index Expanded (SCI-EXPANDED)
SC Plant Sciences
GA 8Y9OU
UT WOS:000933021200001
PM 36778693
"OA Green Submitted, gold"
DA 2026-04-06
ER

PT J
"AU Zeng, WM"
"   Li, F"
"   Wu, CH"
"   Yu, RL"
"   Wu, XL"
"   Shen, L"
"   Liu, YD"
"   Qiu, GH"
"   Li, JK"
"AF Zeng, Weimin"
"   Li, Fang"
"   Wu, Chenchen"
"   Yu, Runlan"
"   Wu, Xueling"
"   Shen, Li"
"   Liu, Yuandong"
"   Qiu, Guanzhou"
"   Li, Jiaokun"
TI Role of extracellular polymeric substance (EPS) in toxicity response of
   soil bacteria Bacillus sp. S3 to multiple heavy metals
SO BIOPROCESS AND BIOSYSTEMS ENGINEERING
LA English
DT Article
DE Metal tolerance; Extracellular polymeric substance; Intracellular
   accumulation; Metal adsorption; Bioremediation
ID CHINA POLLUTION; ADSORPTION; BIOSORPTION; RESISTANCE; ANTIMONY; REMOVAL;
   CADMIUM; IONS; MINE; PRODUCTS
"AB Heavy metal resistant bacteria are of great interest because of their potential use in bioremediation. Understanding the survival and adaptive strategies of these bacteria under heavy metal stress is important for better utilization of these bacteria in remediation. The objective of this study was to investigate the role of bacterial extracellular polymeric substance (EPS) in detoxifying against different heavy metals in Bacillus sp. S3, a new hyper antimony-oxidizing bacterium previously isolated from contaminated mine soils. The results showed that Bacillus sp. S3 is a multi-metal resistant bacterial strain, especially to Sb(III), Cu(II) and Cr(VI). Toxic Cd(II), Cr(VI) and Cu(II) could stimulate the secretion of EPS in Bacillus sp. S3, significantly enhancing the adsorption and detoxification capacity of heavy metals. Both Fourier transform infrared spectroscopy (FTIR) and three-dimensional excitation-emission matrix (3D-EEM) analysis further confirmed that proteins were the main compounds of EPS for metal binding. In contrast, the EPS production was not induced under Sb(III) stress. Furthermore, the TEM-EDX micrograph showed that Bacillus sp. S3 strain preferentially transported the Sb(III) to the inside of the cell rather than adsorbed it on the extracellular surface, indicating intracellular detoxification rather than extracellular EPS precipitation played an important role in microbial resistance towards Sb(III). Together, our study suggests that the toxicity response of EPS to heavy metals is associated with difference in EPS properties, metal types and corresponding environmental conditions, which is likely to contribute to microbial-mediated remediation."
"C1 [Zeng, Weimin; Li, Fang; Wu, Chenchen; Yu, Runlan; Wu, Xueling; Shen, Li; Liu, Yuandong; Qiu, Guanzhou; Li, Jiaokun] Cent S Univ CSU, Sch Minerals Proc & Bioengn, Changsha 410083, Peoples R China."
"   [Zeng, Weimin; Yu, Runlan; Wu, Xueling; Shen, Li; Liu, Yuandong; Qiu, Guanzhou; Li, Jiaokun] Cent S Univ CSU, Key Lab BioMet, Minist Educ, Changsha 410083, Peoples R China."
"   [Zeng, Weimin] CSIRO Mineral Resources, Clayton, Vic 3169, Australia."
C3 Central South University; Central South University; Commonwealth
   Scientific & Industrial Research Organisation (CSIRO); CSIRO Mineral
   Resources
"RP Li, JK (corresponding author), Cent S Univ CSU, Sch Minerals Proc & Bioengn, Changsha 410083, Peoples R China."
EM lijiaokun@csu.edu.cn
"OI Zeng, Weimin/0000-0002-7155-6386; Yu, Runlan/0000-0003-3817-9897"
FU Natural Science Foundation of Hunan Province of China [2019JJ40361]
   Funding Source: Medline; the National Natural Science Foundation of
"   China [51320105006, 31470230, 51604308] Funding Source: Medline; Key"
   Research and Development Projects in Hunan Province [2018WK2012] Funding
   Source: Medline; Fundamental Research Funds for the Central Universities
   of Central South University [2018zzts763] Funding Source: Medline; The
   Youth Talent Foundation of Hunan Province of China [2017RS3003] Funding
   Source: Medline
"CR Aquino SF, 2004, WATER RES, V38, P255, DOI 10.1016/j.watres.2003.09.031"
"   Ball MM, 2007, CURR MICROBIOL, V54, P149, DOI 10.1007/s00284-006-0347-4"
"   Baptista MS, 2006, CRIT REV MICROBIOL, V32, P127, DOI 10.1080/10408410600822934"
"   Boonaert CJP, 2001, COLLOID SURFACE B, V22, P171, DOI 10.1016/S0927-7765(01)00196-5"
"   Bruins MR, 2000, ECOTOX ENVIRON SAFE, V45, P198, DOI 10.1006/eesa.1999.1860"
"   Chen J, 2002, CHEMOSPHERE, V48, P59, DOI 10.1016/S0045-6535(02)00041-3"
"   Chen W, 2003, ENVIRON SCI TECHNOL, V37, P5701, DOI 10.1021/es034354c"

"   Choudhary S, 2009, BIORESOURCE TECHNOL, V100, P2482, DOI 10.1016/j.biortech.2008.12.015"
"   Davis TA, 2003, WATER RES, V37, P4311, DOI 10.1016/S0043-1354(03)00293-8"
"   Ding PF, 2018, BIOPROC BIOSYST ENG, V41, P781, DOI 10.1007/s00449-018-1911-6"
"   DUBOIS M, 1956, ANAL CHEM, V28, P350, DOI 10.1021/ac60111a017"
"   Eboigbodin KE, 2008, BIOMACROMOLECULES, V9, P686, DOI 10.1021/bm701043c"
"   Fang LC, 2011, BIORESOURCE TECHNOL, V102, P1137, DOI 10.1016/j.biortech.2010.09.006"
"   Freundlich H, 1906, Z PHYS CHEM-STOCH VE, V57, P385"
"   FROLUND B, 1995, APPL MICROBIOL BIOT, V43, P755, DOI 10.1007/s002530050481"
"   Fu FL, 2011, J ENVIRON MANAGE, V92, P407, DOI 10.1016/j.jenvman.2010.11.011"
"   Giovanella P, 2017, ECOTOX ENVIRON SAFE, V140, P162, DOI 10.1016/j.ecoenv.2017.02.010"
"   Gonz??lez AG, 2010, J COLLOID INTERF SCI, V350, P305, DOI 10.1016/j.jcis.2010.06.020"
"   Guibaud G, 2003, CHEMOSPHERE, V52, P1701, DOI 10.1016/S0045-6535(03)00355-2"
"   Guibaud G, 2005, CHEMOSPHERE, V59, P629, DOI 10.1016/j.chemosphere.2004.10.028"
"   Ha J, 2010, GEOCHIM COSMOCHIM AC, V74, P1, DOI 10.1016/j.gca.2009.06.031"
"   Hamamura N, 2013, MICROBES ENVIRON, V28, P257, DOI 10.1264/jsme2.ME12217"
"   HOLMES JD, 1995, ARCH MICROBIOL, V163, P143, DOI 10.1007/s002030050184"
"   Huang F, 2013, COLLOID SURFACE B, V107, P11, DOI 10.1016/j.colsurfb.2013.01.062"
"   Joshi PM, 2009, ENVIRON SCI TECHNOL, V43, P5884, DOI 10.1021/es900063b"
"   Langmuir I, 1918, J AM CHEM SOC, V40, P1361, DOI 10.1021/ja02242a004"
"   Li JK, 2018, GEOMICROBIOL J, V35, P713, DOI 10.1080/01490451.2018.1454556"
"   Li J, 2013, INT BIODETER BIODEGR, V76, P76, DOI 10.1016/j.ibiod.2012.06.009"
"   Li WW, 2014, BIORESOURCE TECHNOL, V160, P15, DOI 10.1016/j.biortech.2013.11.074"
"   Li ZY, 2014, SCI TOTAL ENVIRON, V468, P843, DOI 10.1016/j.scitotenv.2013.08.090"
"   Loukidou MX, 2004, COLLOID SURFACE A, V242, P93, DOI 10.1016/j.colsurfa.2004.03.030"
"   Merroun ML, 2003, CHEMOSPHERE, V52, P113, DOI 10.1016/S0045-6535(03)00220-0"
"   Miao LZ, 2017, SCI TOTAL ENVIRON, V579, P588, DOI 10.1016/j.scitotenv.2016.11.056"
"   Mikes J, 2005, WATER SCI TECHNOL, V52, P151, DOI 10.2166/wst.2005.0689"
"   Mishra A, 2013, CRIT REV ENV SCI TEC, V43, P1162, DOI 10.1080/10934529.2011.627044"
"   Mohite BV, 2018, APPL BIOCHEM BIOTECH, V186, P199, DOI 10.1007/s12010-018-2727-1"
"   More TT, 2014, J ENVIRON MANAGE, V144, P1, DOI 10.1016/j.jenvman.2014.05.010"
"   Naja G., 2011, Microbial Biosorption of Metals, Chapter 3: The mechanism of metal cation and anion biosorption, DOI DOI 10.1007/978-94-007-0443-5_3"
"   Nguyen TAH, 2013, BIORESOURCE TECHNOL, V148, P574, DOI 10.1016/j.biortech.2013.08.124"
"   Nies DH, 1999, APPL MICROBIOL BIOT, V51, P730, DOI 10.1007/s002530051457"
"   Nithya C, 2011, MAR ENVIRON RES, V71, P283, DOI 10.1016/j.marenvres.2011.02.003"
"   Pagnanelli F, 2009, CHEMOSPHERE, V75, P1028, DOI 10.1016/j.chemosphere.2009.01.043"
"   Pan XL, 2010, COLLOID SURFACE B, V80, P103, DOI 10.1016/j.colsurfb.2010.05.002"
"   Paul ML, 2012, IND ENG CHEM RES, V51, P15242, DOI 10.1021/ie302140a"
"   Pourret O, 2018, SCI TOTAL ENVIRON, V610, P419, DOI 10.1016/j.scitotenv.2017.08.043"
"   Qu FS, 2012, WATER RES, V46, P2881, DOI 10.1016/j.watres.2012.02.045"
"   SENESI N, 1990, ANAL CHIM ACTA, V232, P51, DOI 10.1016/S0003-2670(00)81225-8"
"   Sheng GP, 2005, APPL MICROBIOL BIOT, V69, P216, DOI 10.1007/s00253-005-1990-6"
"   Sheng GP, 2005, APPL MICROBIOL BIOT, V67, P125, DOI 10.1007/s00253-004-1704-5"
"   Sheng GP, 2013, CHEMOSPHERE, V93, P1436, DOI 10.1016/j.chemosphere.2013.07.076"
"   Sheng GP, 2010, BIOTECHNOL ADV, V28, P882, DOI 10.1016/j.biotechadv.2010.08.001"
"   Tourney J, 2009, J COLLOID INTERF SCI, V337, P381, DOI 10.1016/j.jcis.2009.05.067"
"   Tyagi AK, 2010, MICRON, V41, P797, DOI 10.1016/j.micron.2010.05.007"
"   Ueshima M, 2008, GEOCHIM COSMOCHIM AC, V72, P5885, DOI 10.1016/j.gca.2008.09.014"
"   Wang ZC, 2014, CHEM ENG J, V251, P165, DOI 10.1016/j.cej.2014.04.078"
"   Wei BG, 2010, MICROCHEM J, V94, P99, DOI 10.1016/j.microc.2009.09.014"
"   Wei X, 2011, ENVIRON POLLUT, V159, P1369, DOI 10.1016/j.envpol.2011.01.006"
"   Wu XL, 2019, FRONT MICROBIOL, V10, DOI 10.3389/fmicb.2019.00033"
"   Yamamura S, 2014, J BIOSCI BIOENG, V118, P1, DOI 10.1016/j.jbiosc.2013.12.011"
"   Yang QQ, 2018, SCI TOTAL ENVIRON, V642, P690, DOI 10.1016/j.scitotenv.2018.06.068"
"   Zhao WQ, 2015, CHEM GEOL, V410, P79, DOI 10.1016/j.chemgeo.2015.06.013"
NR 61
TC 162
Z9 182
U1 17
U2 327
PU SPRINGER
PI NEW YORK
"PA ONE NEW YORK PLAZA, SUITE 4600, NEW YORK, NY, UNITED STATES"
SN 1615-7591
EI 1615-7605
J9 BIOPROC BIOSYST ENG
JI Bioprocess. Biosyst. Eng.
PD JAN
PY 2020
VL 43
IS 1
BP 153
EP 167
DI 10.1007/s00449-019-02213-7
PG 15
"WC Biotechnology & Applied Microbiology; Engineering, Chemical"
WE Science Citation Index Expanded (SCI-EXPANDED)
SC Biotechnology & Applied Microbiology; Engineering
GA KI9SU
UT WOS:000511698800013
PM 31549306
DA 2026-04-06
ER

PT J
"AU Ghorbel, S"
"   Aldilami, M"
"   Zouari-Mechichi, H"
"   Mechichi, T"
"   AlSherif, EA"
"AF Ghorbel, Sofiane"
"   Aldilami, Mohammad"
"   Zouari-Mechichi, Hela"
"   Mechichi, Tahar"
"   AlSherif, Emad Ali"
TI Isolation and characterization of a plant growth-promoting
   rhizobacterium strain MD36 that promotes barley seedlings and growth
   under heavy metals stress
SO 3 BIOTECH
LA English
DT Article
DE MD36; PGPR; Heavy metal; Germination; Growth
ID BACTERIA; TOXICITY
"AB Plant growth, promoting, bacteria, (PGPB) can improve plant germination and growth in heavy metal-contaminated land and enhance heavy metal removal efficiency. In this study, we isolated PGPR bacterial strains which can withstand heavy metal pollution and tested their ability to improve barley germination under heavy metal stress. Out of 16 multi-resistant heavy metal isolates, strain MD36 was identified by 16S rRNA sequencing and shared close relation to different species of the genus Glutamicibacter. The new isolated strain showed other important PGPR activities, mainly IAA production and salt tolerance. The effect of adding the strain MD36 to barley grains under heavy metal stress enhanced their germination up to 100%, while the percentage of germination ranged between 0 and 20% for non-inoculated grains. In addition, in these conditions, MD36 can significantly enhance barley growth by reducing the heavy metal effect. This study strongly recommends the use of MD36 as seed coatings trials in the field to enhance growth and yield in soils contaminated with heavy metals, as well as in bioremediation of HM-contaminated salt-containing soils and water."
"C1 [Ghorbel, Sofiane; Aldilami, Mohammad; AlSherif, Emad Ali] Univ Jeddah, Coll Sci & Arts Khulais, Biol Dept, Jeddah, Saudi Arabia."
"   [Ghorbel, Sofiane] Univ Sfax, Inst Biotechnol, Plant Physiol & Funct Genom Res Unit, Sfax 3038, Tunisia."
"   [Zouari-Mechichi, Hela; Mechichi, Tahar] Univ Sfax, Natl Sch Engineers Sfax, Lab Biochem & Enzymat Engn Lipases, Sfax 3038, Tunisia."
"   [AlSherif, Emad Ali] Beni Suef Univ, Fac Sci, Bot & Microbiol Dept, Bani Suwayf 62521, Egypt."
C3 University of Jeddah; Centre de Biotechnologie de Borj Cedria;
   Universite de Sfax; Universite de Sfax; Ecole Nationale dIngenieurs de
   Sfax (ENIS); Egyptian Knowledge Bank (EKB); Beni Suef University
"RP Ghorbel, S (corresponding author), Univ Jeddah, Coll Sci & Arts Khulais, Biol Dept, Jeddah, Saudi Arabia.; Ghorbel, S (corresponding author), Univ Sfax, Inst Biotechnol, Plant Physiol & Funct Genom Res Unit, Sfax 3038, Tunisia."
EM so_fian@yahoo.fr
"RI ; Alsherif, Emad/I-7042-2012"
"OI Ghorbel, Sofiane/0000-0001-8793-4882; "
"FU University of Jeddah, Jeddah, Saudi Arabia [UJ-21-074-DR]; University of"
   Jeddah
"FX This work was funded by the University of Jeddah, Jeddah, Saudi Arabia,"
   under grant number (UJ-21-074-DR). The authors therefore acknowledge
   with thanks the university of Jeddah technical and financial support
"CR AbdElgawad H, 2020, ENVIRON POLLUT, V258, DOI 10.1016/j.envpol.2019.113705"
"   Alves ARA, 2022, SCI TOTAL ENVIRON, V838, DOI 10.1016/j.scitotenv.2022.156435"
"   Belimov AA, 2004, MICROBIOLOGY+, V73, P99, DOI 10.1023/B:MICI.0000016377.62060.d3"
"   Belimov AA, 2000, MICROBIOL RES, V155, P113, DOI 10.1016/S0944-5013(00)80046-4"
"   Bhattacharyya PN, 2012, WORLD J MICROB BIOT, V28, P1327, DOI 10.1007/s11274-011-0979-9"
"   Bravo G, 2020, MICROORGANISMS, V8, DOI 10.3390/microorganisms8121952"
"   Burd GI, 2000, CAN J MICROBIOL, V46, P237, DOI 10.1139/cjm-46-3-237"
"   Dell'Amico E, 2008, SOIL BIOL BIOCHEM, V40, P74, DOI 10.1016/j.soilbio.2007.06.024"
"   Dey G, 2022, MAR POLLUT BULL, V181, DOI 10.1016/j.marpolbul.2022.113905"
"   Goswami Dweipayan, 2016, Cogent Food & Agriculture, V2, DOI 10.1080/23311932.2015.1127500"
"   Hamayun M, 2021, BIOCELL, V45, P363, DOI 10.32604/biocell.2021.013907"
"   Harris J, 2009, SCIENCE, V325, P573, DOI 10.1126/science.1172975"
"   HASEGAWA M, 1991, J MOL EVOL, V32, P443, DOI 10.1007/BF02101285"
"   Hussain A, 2015, FRONT PLANT SCI, V6, DOI 10.3389/fpls.2015.00046"
"   Hussein KA, 2018, J MICROBIOL BIOTECHN, V28, P938, DOI 10.4014/jmb.1712.12027"
"   Ismail Hamayun M., 2020, J APPL BOT FOOD QUAL, V93, P112, DOI DOI 10.5073/JABFQ.2020.093.014"
"   Javed Hina, 2020, Eurasian Journal of Soil Science, V9, P85, DOI [10.18393/ejss.650546, 10.18393/ejss.650546]"
"   Manoj SR, 2020, J ENVIRON MANAGE, V254, DOI 10.1016/j.jenvman.2019.109779"
"   Meena VS, 2017, ECOL ENG, V107, P8, DOI 10.1016/j.ecoleng.2017.06.058"
"   Nagajyoti PC, 2010, ENVIRON CHEM LETT, V8, P199, DOI 10.1007/s10311-010-0297-8"
"   Patten CL, 2002, APPL ENVIRON MICROB, V68, P3795, DOI 10.1128/AEM.68.8.3795-3801.2002"
"   Paz-Ferreiro J, 2016, LAND DEGRAD DEV, V27, P14, DOI 10.1002/ldr.2262"
"   Qadir M, 2020, CHEMOSPHERE, V258, DOI 10.1016/j.chemosphere.2020.127386"
"   Rao N.S. Subba., 1977, SOIL MICROORGANISMS"
"   Santos RG, 2020, GENE, V741, DOI 10.1016/j.gene.2020.144566"
"   Schimel J, 2007, ECOLOGY, V88, P1386, DOI 10.1890/06-0219"
"   Sobariu DL, 2017, NEW BIOTECHNOL, V39, P125, DOI 10.1016/j.nbt.2016.09.002"
"   Verma S, 2019, ENVIRON TECHNOL INNO, V14, DOI 10.1016/j.eti.2019.100369"
"   Wang XY, 2022, SCI TOTAL ENVIRON, V819, DOI 10.1016/j.scitotenv.2022.153242"
"   Wang Y, 2022, FRONT MICROBIOL, V13, DOI 10.3389/fmicb.2022.966226"
"   World Health Organization Regional Office for the Eastern Mediterranean, 2020, World Health Organization annual report 2019 WHO country office Lebanon: health for all"

"   Yao YX, 2015, SCI REP-UK, V5, DOI 10.1038/srep08642"
"   Zaidi S, 2006, CHEMOSPHERE, V64, P991, DOI 10.1016/j.chemosphere.2005.12.057"
"   Zainab N, 2020, PLANT PHYSIOL BIOCH, V152, P90, DOI 10.1016/j.plaphy.2020.04.039"
NR 34
TC 9
Z9 10
U1 3
U2 43
PU SPRINGER HEIDELBERG
PI HEIDELBERG
"PA TIERGARTENSTRASSE 17, D-69121 HEIDELBERG, GERMANY"
SN 2190-572X
EI 2190-5738
J9 3 BIOTECH
JI 3 Biotech
PD MAY
PY 2023
VL 13
IS 5
AR 145
DI 10.1007/s13205-023-03566-3
PG 8
WC Biotechnology & Applied Microbiology
WE Science Citation Index Expanded (SCI-EXPANDED)
SC Biotechnology & Applied Microbiology
GA E8YX9
UT WOS:000978343900005
PM 37124983
OA Green Submitted
DA 2026-04-06
ER

PT J
"AU Ou, JB"
"   Wang, X"
"   Gao, KN"
"   Ma, MY"
"   Wu, J"
"   Xu, SH"
"   Li, DX"
"   Sang, WJ"
"AF Ou, Jinbao"
"   Wang, Xi"
"   Gao, Kangning"
"   Ma, Mingyu"
"   Wu, Juan"
"   Xu, Shihong"
"   Li, Dengxin"
"   Sang, Wenjing"
TI Removal of Typical Heavy Metals from Soil by the Synergistic Interaction
"   between Microbubbles and Washing Agents: Experiments, Influences, and"
   Mechanisms
SO WATER AIR AND SOIL POLLUTION
LA English
DT Article
DE Heavy metals; Intensive washing; Microbubbles; Soil remediation
ID CONTAMINATED SOILS; REMEDIATION; SEPARATION; PILOT
"AB Soil contamination by heavy metals poses serious risks to human health due to their high toxicity and resistance to biodegradation, especially when they accumulate through the food chain. Soil drenching technology is one of the most promising methods for soil pollution remediation. In this research, microbubbles were introduced into the disodium ethylenediamine tetraacetate(EDTA-2Na) solution during soil drenching, combined with Polysorbate 80(TW-80) to remove heavy metals(Cu, Cd) from the soil. The microbubbles contributed to concentrating heavy metals, thus lowering their levels in the solution. The efficiency of heavy metal removal was found to be positively correlated with both time and detergent concentration, and negatively correlated with pH levels. Under conditions where the washing time was 120 minutes, EDTA-2Na concentration was 1 g/L, TW-80 concentration was 2 g/L, the solid-liquid ratio was 1:30, the gas-liquid ratio was 1:3, and the pH was maintained at 3, the removal rates for Cu and Cd were 86.67% and 85.52%, respectively. The Community Bureau of Reference (BCR) sequential extraction procedure revealed that intensive was Ahile et al., 2021 hing with microbubbles effectively eliminated acid-soluble (F1) and reducible (F2) heavy metals from the soil, thereby mitigating their detrimental effects on soil ecosystems. These insights offer a theoretical and practical foundation for employing microbubble technology in the remediation of heavy metal ion pollution in soils."
"C1 [Ou, Jinbao; Wang, Xi; Gao, Kangning; Ma, Mingyu; Wu, Juan; Xu, Shihong; Li, Dengxin; Sang, Wenjing] Donghua Univ, Dept Environm Sci & Engn, Shanghai 201620, Peoples R China."
C3 Donghua University
"RP Li, DX (corresponding author), Donghua Univ, Dept Environm Sci & Engn, Shanghai 201620, Peoples R China."
EM lidengxin@mail.dhu.edu.cn
"RI mingyu, ma/HPE-2588-2023"
FU National Key Research and Development Program: Key Projects of
   International Scientific and Technological Innovation Cooperation
   Between Governments [2022YFE0120600]; National Natural Science
   Foundation of China [52270151]
FX This work was financially supported by the National Key Research and
   Development Program: Key Projects of International Scientific and

   Technological Innovation Cooperation Between Governments
"   (2022YFE0120600), National Natural Science Foundation of China (No."
   52270151)
"CR Adnan M, 2024, ECOTOX ENVIRON SAFE, V274, DOI 10.1016/j.ecoenv.2024.116189"
"   Ahile UJ, 2021, J ENVIRON MANAGE, V278, DOI 10.1016/j.jenvman.2020.111566"
"   Ahile UJ, 2020, J WATER PROCESS ENG, V36, DOI 10.1016/j.jwpe.2020.101320"
"   Azpeitia J, 2023, APPL SURF SCI, V639, DOI 10.1016/j.apsusc.2023.158231"
"   Cakaj A, 2024, ENVIRON RES, V256, DOI 10.1016/j.envres.2024.119222"
"   Chen WT, 2023, J CLEAN PROD, V432, DOI 10.1016/j.jclepro.2023.139776"
"   Cheng SL, 2020, ARAB J CHEM, V13, P5160, DOI 10.1016/j.arabjc.2020.02.015"
"   Deng KM, 2024, J COLLOID INTERF SCI, V656, P466, DOI 10.1016/j.jcis.2023.11.128"
"   Diarra I, 2021, CHEMOSPHERE, V273, DOI 10.1016/j.chemosphere.2020.128483"
"   Efligenir A, 2013, J ENVIRON CHEM ENG, V1, P448, DOI 10.1016/j.jece.2013.06.010"
"   Feng G, 2024, SCI TOTAL ENVIRON, V925, DOI 10.1016/j.scitotenv.2024.171785"
"   Foudas AW, 2023, CHEM ENG RES DES, V189, P64, DOI 10.1016/j.cherd.2022.11.013"
"   Gautam P, 2020, CHEMOSPHERE, V250, DOI 10.1016/j.chemosphere.2020.126206"
"   Ghassemi-Golezani Kazem, 2024, Chemosphere, V362, P142623, DOI [10.1016/j.chemosphere.2024.142623, 10.1016/j.chemosphere.2024.142623]"
"   Han JC, 2024, J CLEAN PROD, V450, DOI 10.1016/j.jclepro.2024.141918"
"   He ZL, 2023, J CLEAN PROD, V393, DOI 10.1016/j.jclepro.2023.136235"
"   Hsi HC, 2018, SCI TOTAL ENVIRON, V627, P844, DOI 10.1016/j.scitotenv.2018.01.308"
"   Hu GY, 2024, J CLEAN PROD, V453, DOI 10.1016/j.jclepro.2024.142148"
"   Hu PJ, 2014, CHEMOSPHERE, V117, P532, DOI 10.1016/j.chemosphere.2014.08.081"
"   Huang JT, 2024, J HAZARD MATER, V470, DOI 10.1016/j.jhazmat.2024.134115"
"   Jia JG, 2023, CHEM ENG J, V471, DOI 10.1016/j.cej.2023.144621"
"   Khatun J, 2022, TOXICOLOGY, V477, DOI 10.1016/j.tox.2022.153274"
"   Kim H, 2024, RESULTS ENG, V22, DOI 10.1016/j.rineng.2024.102312"
"   Kou B, 2024, SCI TOTAL ENVIRON, V917, DOI 10.1016/j.scitotenv.2024.170451"
"   Kou YY, 2020, SCI TOTAL ENVIRON, V707, DOI 10.1016/j.scitotenv.2019.135866"
"   Kumm ZA, 2024, APPL CLAY SCI, V259, DOI 10.1016/j.clay.2024.107513"
"   Li YP, 2022, J ENVIRON MANAGE, V312, DOI 10.1016/j.jenvman.2022.114921"
"   Liu SY, 2024, ENVIRON RES, V253, DOI 10.1016/j.envres.2024.119153"
"   Lohr CD, 2024, MAR PETROL GEOL, V164, DOI 10.1016/j.marpetgeo.2023.106655"
"   Lu L, 2023, J HAZARD MATER, V442, DOI 10.1016/j.jhazmat.2022.130021"
"   Pang Zhongzheng, 2024, Chemosphere, V356, P141917, DOI [10.1016/j.chemosphere.2024.141917, 10.1016/j.chemosphere.2024.141917]"
"   Priyanka M, 2022, J CLEAN PROD, V334, DOI 10.1016/j.jclepro.2021.130198"
"   Schwartz AM., 1989, Journal of Colloid and Interface Science, V130, P296, DOI [10.1016/0021-9797(89)90106-9, DOI 10.1016/0021-9797(89)90106-9]"
"   Sheets RG, 1999, J HAZARD MATER, V66, P137, DOI 10.1016/S0304-3894(99)00009-6"
"   Shi XN, 2021, LANGMUIR, V37, P2514, DOI 10.1021/acs.langmuir.0c03574"
"   Song PP, 2022, SCI TOTAL ENVIRON, V838, DOI 10.1016/j.scitotenv.2022.156417"
"   Sun H, 2023, ELECTROCHIM ACTA, V448, DOI 10.1016/j.electacta.2023.142178"
"   Rehman Z, 2023, SCI TOTAL ENVIRON, V867, DOI 10.1016/j.scitotenv.2023.161468"
"   Ure A.M., 1995, TECH INSTRUM ANAL CH, V17, P505, DOI [10.1016/S0167-9244(06)80021-1, DOI 10.1016/S0167-9244(06)80021-1]"
"   Vancsik A, 2024, J HAZARD MATER, V474, DOI 10.1016/j.jhazmat.2024.134704"
"   Wang A, 2024, CHEM ENG SCI, V288, DOI 10.1016/j.ces.2024.119812"
"   Wang FE, 2023, ENVIRON POLLUT, V316, DOI 10.1016/j.envpol.2022.120608"
"   Wang J, 2024, CHEM ENG J, V488, DOI 10.1016/j.cej.2024.150935"
"   Wang N, 2024, J ENVIRON SCI, V135, P242, DOI 10.1016/j.jes.2022.12.005"
"   Xia F, 2024, J HAZARD MATER, V465, DOI 10.1016/j.jhazmat.2023.133215"
"   Xiao ZG, 2020, SEP PURIF TECHNOL, V242, DOI 10.1016/j.seppur.2020.116788"
"   Xu JY, 2014, CHEMOSPHERE, V109, P99, DOI 10.1016/j.chemosphere.2014.02.058"
"   Ylivainio K, 2010, ENVIRON POLLUT, V158, P3194, DOI 10.1016/j.envpol.2010.07.004"
"   Yuan SH, 2010, GEODERMA, V159, P165, DOI 10.1016/j.geoderma.2010.07.008"
"   Zhang C, 2024, J HAZARD MATER, V465, DOI 10.1016/j.jhazmat.2023.133330"
"   Zhang HJ, 2017, ENVIRON SCI POLLUT R, V24, P9506, DOI 10.1007/s11356-017-8660-y"
"   Zhang J, 2024, SEP PURIF TECHNOL, V340, DOI 10.1016/j.seppur.2024.126767"
"   Zhang Kailu, 2024, Chemosphere, V352, P141293, DOI [10.1016/j.chemosphere.2024.141293, 10.1016/j.chemosphere.2024.141293]"
"   Zhang M, 2017, WATER RES, V126, P399, DOI 10.1016/j.watres.2017.09.051"
"   Zhang YL, 2024, CHEM ENG J, V497, DOI 10.1016/j.cej.2024.154649"
"   Zhou Xian, 2023, Chemosphere, V344, P140394, DOI [10.1016/j.chemosphere.2023.140394, 10.1016/j.chemosphere.2023.140394]"
"   Zhou ZW, 2024, J HAZARD MATER, V473, DOI 10.1016/j.jhazmat.2024.134712"
"   Zhu ZH, 2021, SURF INTERFACES, V27, DOI 10.1016/j.surfin.2021.101563"
"   Zinjenab ZT, 2023, CHEM ENG PROCESS, V189, DOI 10.1016/j.cep.2023.109401"
NR 59
TC 1
Z9 1
U1 4
U2 33
PU SPRINGER INT PUBL AG
PI CHAM
"PA GEWERBESTRASSE 11, CHAM, CH-6330, SWITZERLAND"
SN 0049-6979
EI 1573-2932
J9 WATER AIR SOIL POLL
JI Water Air Soil Pollut.
PD NOV
PY 2024
VL 235
IS 11

AR 710
DI 10.1007/s11270-024-07547-x
PG 13
WC Environmental Sciences; Meteorology & Atmospheric Sciences; Water
   Resources
WE Science Citation Index Expanded (SCI-EXPANDED)
SC Environmental Sciences & Ecology; Meteorology & Atmospheric Sciences;
   Water Resources
GA H1X5U
UT WOS:001321439100001
DA 2026-04-06
ER

PT J
"AU Tseng, SC"
"   Liang, CM"
"   Chia, T"
"   Ton, SS"
"AF Tseng, Shu-chun"
"   Liang, Chih-ming"
"   Chia, Taipau"
"   Ton, Shan-shin"
TI Changes in the Composition of the Soil Bacterial Community in Heavy
   Metal-Contaminated Farmland
SO INTERNATIONAL JOURNAL OF ENVIRONMENTAL RESEARCH AND PUBLIC HEALTH
LA English
DT Article
DE heavy metals; soil bacteria; bacterial community
ID MICROBIAL ACTIVITY; DIVERSITY; BACILLUS; BIOSORPTION; RIVER
"AB The structural changes of microorganisms in soil are the focus of soil indicators research. The purpose of this study was to investigate the changes in the composition of the soil bacterial community in heavy metal-contaminated soil. A total of six soil samples (two sampling times) were collected from contaminated farmland at three different depths (surface, middle, and deep layer). The pH value was measured. The concentrations of heavy metals (Cr, Ni, Cu, Zn, Cd, and Pb) and the soil bacterial community were analyzed using ICP-OES and 16S rRNA gene sequencing. The results of the two samplings showed that the pH value in the deep layer decreased from 6.88 to 6.23, and the concentrations of Cu, Zn, Cd, and Pb, with a smaller ion radius, increased by 16-28%, and Shannon, Chao1 increased by similar to 13%. The bacteria community composition at the three depths changed, but Proteobacteria, Acidobacteria, and Actinobacteria were the dominant phyla. In the copper and zinc tolerance test, the isolated bacterium that was able to tolerate copper and zinc was Bacillus sp. We found that, the longer the heavy metal pollution was of concern, the higher the tolerance. These results can be used as references for the microbial remediation of heavy metal-contaminated farmland."
"C1 [Tseng, Shu-chun; Liang, Chih-ming; Ton, Shan-shin] Feng Chia Univ, Dept Environm Engn & Sci, Taichung 407802, Taiwan."
"   [Chia, Taipau] Hung Kuang Univ, Dept Safety Hlth & Environm Engn, 1018,Sect 6,Taiwan Blvd, Taichung 433304, Taiwan."
C3 Feng Chia University
"RP Ton, SS (corresponding author), Feng Chia Univ, Dept Environm Engn & Sci, Taichung 407802, Taiwan."
EM sctseng0315@gmail.com; cmliang@fcu.edu.tw; tpchia38@gmail.com;
   sston@fcu.edu.tw
"OI Liang, Chih-Ming/0000-0002-7818-7253; Ton, Shanshin/0000-0003-0440-4566;"
"   Shu-Chun, Tseng/0000-0002-5519-7598"
"CR Abu Hasan H, 2016, J IND ENG CHEM, V37, P295, DOI 10.1016/j.jiec.2016.03.038"
"   Abu Hasan H, 2012, J ENVIRON MANAGE, V111, P34, DOI 10.1016/j.jenvman.2012.06.027"
"   Alawiye TT, 2021, AGRICULTURE-BASEL, V11, DOI 10.3390/agriculture11020167"
"   Ayangbenro AS, 2020, SCI REP-UK, V10, DOI 10.1038/s41598-020-75170-x"
"   Ayangbenro AS, 2017, INT J ENV RES PUB HE, V14, DOI 10.3390/ijerph14010094"
"   Bandeira B, 2013, ECOL INDIC, V29, P522, DOI 10.1016/j.ecolind.2013.01.028"
"   Beattie RE, 2018, SOIL BIOL BIOCHEM, V126, P57, DOI 10.1016/j.soilbio.2018.08.011"
"   Bohn H. L., 1979, Soil Chemistry."
"   Bolger AM, 2014, BIOINFORMATICS, V30, P2114, DOI 10.1093/bioinformatics/btu170"
"   Bouskill NJ, 2010, ECOTOXICOLOGY, V19, P317, DOI 10.1007/s10646-009-0414-2"
"   Brandt KK, 2010, SOIL BIOL BIOCHEM, V42, P748, DOI 10.1016/j.soilbio.2010.01.008"
"   CHAO A, 1987, BIOMETRICS, V43, P783, DOI 10.2307/2531532"
"   Chodak M, 2013, APPL SOIL ECOL, V64, P7, DOI 10.1016/j.apsoil.2012.11.004"
"   Cui H, 2018, ECOTOX ENVIRON SAFE, V166, P259, DOI 10.1016/j.ecoenv.2018.09.096"
"   Ding ZL, 2017, SOIL SCI PLANT NUTR, V63, P75, DOI 10.1080/00380768.2016.1247385"
"   Fagnano M, 2020, SCI TOTAL ENVIRON, V734, DOI 10.1016/j.scitotenv.2020.139434"
"   Goes-Neto A., 2005, Biotemas, V18, P19"
"   Griffiths BS, 2013, FEMS MICROBIOL REV, V37, P112, DOI 10.1111/j.1574-6976.2012.00343.x"
"   Hu WG, 2015, PLOS ONE, V10, DOI 10.1371/journal.pone.0145747"
"   Huang F, 2013, COLLOID SURFACE B, V107, P11, DOI 10.1016/j.colsurfb.2013.01.062"
"   Idris R, 2004, APPL ENVIRON MICROB, V70, P2667, DOI 10.1128/AEM.70.5.2667-2677.2004"
"   Keiblinger KM, 2018, ECOTOXICOLOGY, V27, P217, DOI 10.1007/s10646-017-1888-y"
"   Khadhar S, 2020, ENVIRON POLLUT, V263, DOI 10.1016/j.envpol.2020.114543"
"   Lane D.J., 1911, NUCL ACID TECHNIQUES"
"   Lee HJ, 2015, FRONT MICROBIOL, V6, DOI 10.3389/fmicb.2015.00639"
"   Li CZ, 2021, HUM ECOL RISK ASSESS, V27, P724, DOI 10.1080/10807039.2020.1752143"
"   Li Q, 2021, INT J ENV RES PUB HE, V18, DOI 10.3390/ijerph18136859"
"   Li XQ, 2017, ENVIRON POLLUT, V231, P908, DOI 10.1016/j.envpol.2017.08.057"
"   Liang XL, 2020, SOIL BIOL BIOCHEM, V144, DOI 10.1016/j.soilbio.2020.107767"
"   Lin YB, 2019, ECOTOX ENVIRON SAFE, V180, P557, DOI 10.1016/j.ecoenv.2019.05.057"
"   Luo LY, 2019, APPL SOIL ECOL, V139, P100, DOI 10.1016/j.apsoil.2019.03.022"
"   Mohammadian E, 2017, CHEMOSPHERE, V185, P290, DOI 10.1016/j.chemosphere.2017.07.022"

"   Naveed M, 2020, INT J ENV RES PUB HE, V17, DOI 10.3390/ijerph17238859"
"   Nunoura T, 2013, MICROBES ENVIRON, V28, P228, DOI 10.1264/jsme2.ME12193"
"   Park K., 2012, FDN MICROBIOLOGY"
"   P??rez-Esteban J, 2013, ENVIRON EXP BOT, V88, P53, DOI 10.1016/j.envexpbot.2011.12.003"
"   RELMAN DA, 1992, NEW ENGL J MED, V327, P293, DOI 10.1056/NEJM199207303270501"
"   Ren GM, 2015, ECOTOX ENVIRON SAFE, V117, P141, DOI 10.1016/j.ecoenv.2015.03.033"
"   Slimane M., 2021, HEAVY METALS ENV, P39, DOI [10.1016/B978-0-12-821656-9.00003-1, DOI 10.1016/B978-0-12-821656-9.00003-1]"
"   Soil and Groundwater Pollution Fund Management Board, 2018 ANN REP SOIL GR"
"   Song JW, 2018, ENVIRON POLLUT, V243, P510, DOI 10.1016/j.envpol.2018.09.011"
"   Thavamani P, 2012, J ENVIRON MANAGE, V99, P10, DOI 10.1016/j.jenvman.2011.12.030"
"   Wilbrink B, 1998, ARTHRITIS RHEUM-US, V41, P535, DOI 10.1002/1529-0131(199803)41:3<535::AID-ART20>3.0.CO;2-4"
"   Wolna-Maruwka A, 2021, AGRONOMY-BASEL, V11, DOI 10.3390/agronomy11071286"
"   Yamamoto N, 2009, ASIAN AUSTRAL J ANIM, V22, P900, DOI 10.5713/ajas.2009.80663"
"   Yang JJ, 2020, INT J ENV RES PUB HE, V17, DOI 10.3390/ijerph17197101"
"   Yin HQ, 2015, SCI REP-UK, V5, DOI 10.1038/srep14266"
"   Zhao XQ, 2019, ECOTOX ENVIRON SAFE, V170, P218, DOI 10.1016/j.ecoenv.2018.11.136"
"   Zhu JY, 2013, MAR POLLUT BULL, V70, P134, DOI 10.1016/j.marpolbul.2013.02.023"
NR 49
TC 27
Z9 29
U1 8
U2 96
PU MDPI
PI BASEL
"PA MDPI AG, Grosspeteranlage 5, CH-4052 BASEL, SWITZERLAND"
EI 1660-4601
J9 INT J ENV RES PUB HE
JI Int. J. Environ. Res. Public Health
PD AUG
PY 2021
VL 18
IS 16
AR 8661
DI 10.3390/ijerph18168661
PG 15
"WC Environmental Sciences; Public, Environmental & Occupational Health"
WE Science Citation Index Expanded (SCI-EXPANDED); Social Science Citation Index (SSCI)
"SC Environmental Sciences & Ecology; Public, Environmental & Occupational"
Health
GA UI2RL
UT WOS:000690461100001
PM 34444410
"OA Green Submitted, gold"
DA 2026-04-06
ER

PT J
"AU Asad, SA"
"   Farooq, M"
"   Afzal, A"
"   West, H"
"AF Asad, Saeed Ahmad"
"   Farooq, Muhammad"
"   Afzal, Aftab"
"   West, Helen"
TI Integrated phytobial heavy metal remediation strategies for a
sustainable clean environment - A review
SO CHEMOSPHERE
LA English
DT Review
DE Bioremediation; Heavy metals; Hyperaccumulators; PGPR; Toxicity
ID GROWTH-PROMOTING RHIZOBACTERIA; ARBUSCULAR MYCORRHIZAL FUNGI;
PLANT-ASSOCIATED MICROBES; CANOLA BRASSICA-NAPUS; ENDOPHYTIC BACTERIA;
ACC-DEAMINASE; CONTAMINATED SOILS; SIDEROPHORE PRODUCTION;
THLASPI-CAERULESCENS; PSEUDOMONAS-PUTIDA
"AB Heavy metal contamination in the environment is a global threat which accelerated after the industrial revolution. Remediation of these noxious elements has been widely investigated and multifarious technologies have been practiced for many decades. Phytoremediation has attracted much attention from researchers. Under this technology, heavy metal hyperaccumulator plants have been extensively employed to extract extraordinary concentrations of heavy metals but slow growth, limited biomass and stresses caused by heavy metals imperil the efficiency of hyperaccumulators. Plant growth promoting rhizobacteria (PGPR) can help overcome/lessen heavy metal-induced adversities. PGPR produce several metabolites, including growth hormones, siderophores and organic acids, which aid in solubilization and provision of essential nutrients (e.g. Fe and Mg) to the plant. Hyperaccumulator plants may be employed to remediate metal contaminated sites. Use of PGPR to enhance growth of hyperaccumulator plant species may enhance their metal accumulating capacity by increasing metal availability and also by alleviating plant stress induced by the heavy metals. Combined use of hyperaccumulator plants and PGPR may prove to be a cost effective and environmentally friendly technology to clean heavy metal contaminated sites on a sustainable basis. This review discusses the current status of PGPR in improving the growth and development of hyperaccumulator plants growing in metal contaminated environments. The mechanisms used by these rhizosphere bacteria in increasing the availability of heavy metals to plants and coping with heavy metal stresses are also described. (C) 2018 Elsevier Ltd. All rights reserved."

"C1 [Asad, Saeed Ahmad] COMSATS Univ, Ctr Climate Res & Dev, Pk Rd, Chak Shahzad Islamabad 45550, Pakistan."
"   [Farooq, Muhammad] Sultan Qaboos Univ, Coll Agr & Marine Sci, Dept Crop Sci, Al Khoud 123, Oman."
"   [Farooq, Muhammad] Univ Agr Faisalabad, Dept Agron, Faisalabad, Pakistan."
"   [Afzal, Aftab] Hazara Univ Mansehra, Dept Bot, Mansehra, Pakistan."
"   [West, Helen] Univ Nottingham, Sch Biosci, Sutton Bonington Campus, Loughborough LE12 5RD, Leics, the United Kingdom."
C3 COMSATS University Islamabad (CUI); Sultan Qaboos University; University of Agriculture Faisalabad; Hazara University; University of Nottingham
"RP Asad, SA (corresponding author), COMSATS Univ, Ctr Climate Res & Dev, Pk Rd, Chak Shahzad Islamabad 45550, Pakistan."
EM saeed.asad@comsats.edu.pk
"RI Farooq, Muhammad/F-1617-2010; Asad, Saeed A/E-6236-2019; Afzal," Aftab/F-4057-2017
"OI Farooq, Muhammad/0000-0003-4368-9357; Asad, Saeed A/0000-0002-9242-3806;"

"CR Abou-Shanab RAI, 2006, SOIL BIOL BIOCHEM, V38, P2882, DOI 10.1016/j.soilbio.2006.04.045"
"   Abou-Shanab RA, 2003, NEW PHYTOL, V158, P219, DOI 10.1046/j.1469-8137.2003.00721.x"
"   Aboudrar W, 2013, J SOIL SEDIMENT, V13, P501, DOI 10.1007/s11368-012-0614-x"
"   Aftab Afzal Aftab Afzal, 2005, International Journal of Agriculture and Biology, V7, P207"
"   Ali S.S., 2013, INT J CURR MICROBIOL, V2, P303, DOI DOI 10.1016/S0022-2836(05)80360-2"
"   Altin??zl?? H, 2012, TURK J BOT, V36, P269, DOI 10.3906/bot-1101-10"
"   Alvin A, 2014, MICROBIOL RES, V169, P483, DOI 10.1016/j.micres.2013.12.009"
"   Amari T, 2017, S AFR J BOT, V111, P99, DOI 10.1016/j.sajb.2017.03.011"
"   [Anonymous], 1987, SERPENTINE ITS VEGET"
"   [Anonymous], apportionment of heavy metals from Kushui Rose growing soils"
"   [Anonymous], LIFE SCI MED RES LSM"
"   [Anonymous], THESIS"
"   [Anonymous], PHYTOREMEDIATION CON"
"   [Anonymous], 2012, J BOT"
"   [Anonymous], NATO SCI 4"
"   [Anonymous], FIZIOL RAST"
"   [Anonymous], 2012, RESOUR ENV"
"   [Anonymous], INT J CHEM ENG"
"   [Anonymous], NATO SECURITY SCI SE"
"   [Anonymous], 2010, NAT ED KNOW"
"   [Anonymous], SCI TOTAL ENV"
"   Aravind R, 2010, CROP PROT, V29, P318, DOI 10.1016/j.cropro.2009.12.005"
"   Asad S. A., 2004, International Journal of Agriculture and Biology, V6, P837"
"   Asad SA, 2015, SCI TOTAL ENVIRON, V511, P21, DOI 10.1016/j.scitotenv.2014.12.029"
"   Asad SA, 2013, PAK J BOT, V45, P495"
"   Ashraf MA, 2017, J ENVIRON MANAGE, V198, P132, DOI 10.1016/j.jenvman.2017.04.060"
"   Audet P, 2007, ENVIRON POLLUT, V147, P231, DOI 10.1016/j.envpol.2006.08.011"
"   BAKER AJM, 1994, NEW PHYTOL, V127, P61, DOI 10.1111/j.1469-8137.1994.tb04259.x"
"   Baldwin PR, 2007, MICROCHEM J, V85, P297, DOI 10.1016/j.microc.2006.07.005"
"   Barazani O, 1999, J CHEM ECOL, V25, P2397, DOI 10.1023/A:1020890311499"
"   BARCELO J, 1990, J PLANT NUTR, V13, P1, DOI 10.1080/01904169009364057"
"   Barsukova V.S., 1999, Agrokhimiya, V1, P80"
"   Barzanti R, 2007, MICROB ECOL, V53, P306, DOI 10.1007/s00248-006-9164-3"
"   Becerra-Castro C, 2013, APPL ENVIRON MICROB, V79, P5094, DOI 10.1128/AEM.00402-13"
"   Belimov AA, 2005, SOIL BIOL BIOCHEM, V37, P241, DOI 10.1016/j.soilbio.2004.07.033"
"   Berg G, 2009, APPL MICROBIOL BIOT, V84, P11, DOI 10.1007/s00253-009-2092-7"
"   Bert V, 2002, NEW PHYTOL, V155, P47, DOI 10.1046/j.1469-8137.2002.00432.x"
"   Bhattacharyya PN, 2012, WORLD J MICROB BIOT, V28, P1327, DOI 10.1007/s11274-011-0979-9"
"   Bolan N, 2014, J HAZARD MATER, V266, P141, DOI 10.1016/j.jhazmat.2013.12.018"
"   Bonanno G, 2017, SCI TOTAL ENVIRON, V576, P796, DOI 10.1016/j.scitotenv.2016.10.171"
"   Boyd RS, 2004, NEW PHYTOL, V162, P563, DOI 10.1111/j.1469-8137.2004.01079.x"
"   Braud A, 2009, CHEMOSPHERE, V74, P280, DOI 10.1016/j.chemosphere.2008.09.013"
"   BROOKES PC, 1984, J SOIL SCI, V35, P341, DOI 10.1111/j.1365-2389.1984.tb00288.x"
"   Brooks M.A., 1998, THESIS"
"   BROOKS RR, 1977, J GEOCHEM EXPLOR, V7, P49, DOI 10.1016/0375-6742(77)90074-7"
"   Burd GI, 1998, APPL ENVIRON MICROB, V64, P3663"
"   Burd GI, 2000, CAN J MICROBIOL, V46, P237, DOI 10.1139/cjm-46-3-237"
"   Cabello-Conejo MI, 2014, PLANT SOIL, V379, P35, DOI 10.1007/s11104-014-2043-7"
"   Callahan DL, 2006, J BIOL INORG CHEM, V11, P2, DOI 10.1007/s00775-005-0056-7"
"   Chaney R. L., 2010, Trace elements in soils, P311"
"   Chen L, 2014, SOIL BIOL BIOCHEM, V68, P300, DOI 10.1016/j.soilbio.2013.10.021"
"   Chen YP, 2006, APPL SOIL ECOL, V34, P33, DOI 10.1016/j.apsoil.2005.12.002"
"   Chen ZJ, 2013, J HAZARD MATER, V244, P709, DOI 10.1016/j.jhazmat.2012.10.063"
"   Chiboub M, 2016, CR BIOL, V339, P391, DOI 10.1016/j.crvi.2016.04.015"
"   Chibuike GU., 2014, APPLIE ENV SOIL SCI, V2014, P1"
"   Cocozza C, 2015, ENVIRON SCI POLLUT R, V22, P19546, DOI 10.1007/s11356-015-5097-z"
"   Collins J. C., 1981, Effect of heavy metal pollution on plants. Vol.1. Effects of trace metals on plant function, P145"
"   Compant S, 2005, APPL ENVIRON MICROB, V71, P4951, DOI 10.1128/AEM.71.9.4951-4959.2005"
"   Cunningham SD, 1996, PLANT PHYSIOL, V110, P715, DOI 10.1104/pp.110.3.715"
"   Gomes MAD, 2016, ECOTOX ENVIRON SAFE, V134, P133, DOI 10.1016/j.ecoenv.2016.08.024"
"   Daniels R, 2004, FEMS MICROBIOL REV, V28, P261, DOI 10.1016/j.femsre.2003.09.004"
"   Delvasto P, 2009, MINER ENG, V22, P1, DOI 10.1016/j.mineng.2008.03.001"
"   Deng H, 2010, FRESEN ENVIRON BULL, V19, P94"
"   Di Fiori S., 1995, AZOSPIRILLUM 6 RELAT, P169, DOI [10.1007/978-3-642-79906-8_18, DOI 10.1007/978-3-642-79906-8_18]"
"   Dimkpa CO, 2008, CHEMOSPHERE, V74, P19, DOI 10.1016/j.chemosphere.2008.09.079"
"   Dimkpa CO, 2009, SOIL BIOL BIOCHEM, V41, P154, DOI 10.1016/j.soilbio.2008.10.010"
"   Doornbos RF, 2012, AGRON SUSTAIN DEV, V32, P227, DOI 10.1007/s13593-011-0028-y"

"   Durand A, 2016, PLANT SOIL, V399, P179, DOI 10.1007/s11104-015-2691-2"
"   Emamverdian A., 2015, SCI WORLD J, DOI DOI 10.1155/2015/756120"
"   Fernando DR, 2008, NEW PHYTOL, V177, P178, DOI 10.1111/j.1469-8137.2007.02253.x"
"   Fernando DR, 2010, NEW PHYTOL, V188, P1014, DOI 10.1111/j.1469-8137.2010.03431.x"
"   Foyer CH, 2005, PLANT CELL, V17, P1866, DOI 10.1105/tpc.105.033589"
"   Fuentes A, 2016, PLANT SOIL, V407, P355, DOI 10.1007/s11104-016-2893-2"
"   Gadd GM, 2010, MICROBIOL-SGM, V156, P609, DOI 10.1099/mic.0.037143-0"
"   Gamalero E, 2009, CAN J MICROBIOL, V55, P501, DOI [10.1139/W09-010, 10.1139/w09-010]"
"   Gao YZ, 2003, ENVIRON INT, V29, P613, DOI 10.1016/S0160-4120(03)00048-5"
"   Garc??a-Salgado S, 2012, WATER AIR SOIL POLL, V223, P559, DOI 10.1007/s11270-011-0882-x"
"   Gechev TS, 2006, BIOESSAYS, V28, P1091, DOI 10.1002/bies.20493"
"   Gill SS, 2010, PLANT PHYSIOL BIOCH, V48, P909, DOI 10.1016/j.plaphy.2010.08.016"
"   Glick B., 1999, BIOCH GENETIC MECH U, DOI DOI 10.1142/9781848160521"
"   Glick BR, 2007, EUR J PLANT PATHOL, V119, P329, DOI 10.1007/s10658-007-9162-4"
"   Glick BR, 2010, BIOTECHNOL ADV, V28, P367, DOI 10.1016/j.biotechadv.2010.02.001"
"   GLICK BR, 1995, CAN J MICROBIOL, V41, P109, DOI 10.1139/m95-015"
"   Glick BR, 1998, J THEOR BIOL, V190, P63, DOI 10.1006/jtbi.1997.0532"
"   GODBOLD DL, 1985, ENVIRON POLLUT A, V38, P375, DOI 10.1016/0143-1471(85)90108-4"
"   G??hre V, 2006, PLANTA, V223, P1115, DOI 10.1007/s00425-006-0225-0"
"   Gravel V, 2007, SOIL BIOL BIOCHEM, V39, P1968, DOI 10.1016/j.soilbio.2007.02.015"
"   Grichko VP, 2001, PLANT PHYSIOL BIOCH, V39, P11, DOI 10.1016/S0981-9428(00)01212-2"
"   Grobelak A, 2018, J ECOL ENG, V19, P150, DOI 10.12911/22998993/89818"
"   Guarino C, 2017, ECOL ENG, V99, P70, DOI 10.1016/j.ecoleng.2016.11.051"
"   Guarino C, 2014, ENVIRON SCI TECHNOL, V48, P11487, DOI 10.1021/es502070m"
"   Guo JK, 2014, PLANT SOIL, V375, P205, DOI 10.1007/s11104-013-1952-1"
"   Guo JK, 2011, WORLD J MICROB BIOT, V27, P2835, DOI 10.1007/s11274-011-0762-y"
"   Gupta A, 2002, CURR MICROBIOL, V45, P323, DOI 10.1007/s00284-002-3762-1"
"   Gupta DK, 2014, CHEMOSPHERE, V108, P134, DOI 10.1016/j.chemosphere.2014.01.030"
"   Guti??rrez-Ma??ero FJ, 2001, PHYSIOL PLANTARUM, V111, P206, DOI 10.1034/j.1399-3054.2001.1110211.x"
"   HAGHIRI F, 1974, J ENVIRON QUAL, V3, P180, DOI 10.2134/jeq1974.00472425000300020021x"
"   Harish S, 2009, BIOCONTROL SCI TECHN, V19, P843, DOI 10.1080/09583150903145000"
"   Hartmann A, 2009, PLANT SOIL, V321, P235, DOI 10.1007/s11104-008-9814-y"
"   HASSAN M., 1996, THESIS"
"   Hassan W, 2016, ENVIRON EARTH SCI, V75, DOI 10.1007/s12665-015-4902-9"
"   Hayat R, 2010, ANN MICROBIOL, V60, P579, DOI 10.1007/s13213-010-0117-1"
"   Hewitt E. J., 1983, Metals and micronutrients: uptake and utilization by plants, P277"
"   HOFLICH G, 1994, EXPERIENTIA, V50, P897, DOI 10.1007/BF01923476"
"   Hooda V, 2007, J ENVIRON BIOL, V28, P367"
"   HUYER M, 1988, APPL ENVIRON MICROB, V54, P2625, DOI 10.1128/AEM.54.11.2625-2631.1988"
"   Ibrahim M. A., 2015, Environ. Rev, V23, P1"
"   Idris R, 2004, APPL ENVIRON MICROB, V70, P2667, DOI 10.1128/AEM.70.5.2667-2677.2004"
"   Islam F, 2014, ECOTOX ENVIRON SAFE, V110, P143, DOI 10.1016/j.ecoenv.2014.08.020"
"   JAFFRE T, 1976, SCIENCE, V193, P579, DOI 10.1126/science.193.4253.579"
"   Jaffr?? T, 2013, FRONT PLANT SCI, V4, DOI 10.3389/fpls.2013.00279"
"   Jalili F, 2009, J PLANT PHYSIOL, V166, P667, DOI 10.1016/j.jplph.2008.08.004"
"   Jamil M, 2014, INT J PHYTOREMEDIAT, V16, P554, DOI 10.1080/15226514.2013.798621"
"   Jeong S, 2014, J HAZARD MATER, V280, P536, DOI 10.1016/j.jhazmat.2014.08.057"
"   Jiang CY, 2008, CHEMOSPHERE, V72, P157, DOI 10.1016/j.chemosphere.2008.02.006"
"   Jing YX, 2014, INT J PHYTOREMEDIAT, V16, P321, DOI 10.1080/15226514.2013.773283"
"   Jones DL, 2003, PLANT SOIL, V248, P31, DOI 10.1023/A:1022304332313"
"   Kalve S, 2011, CURR SCI INDIA, V100, P888"
"   Kamran MA, 2015, ENVIRON SCI POLLUT R, V22, P9275, DOI 10.1007/s11356-015-4074-x"
"   Karimi A, 2017, CHEM ECOL, V33, P963, DOI 10.1080/02757540.2017.1391798"
"   Kavino M, 2010, APPL SOIL ECOL, V45, P71, DOI 10.1016/j.apsoil.2010.02.003"
"   Kaymak HC, 2010, MICROBIOL MONOGR, V18, P45, DOI 10.1007/978-3-642-13612-2_3"
"   Khan MS, 2007, AGRON SUSTAIN DEV, V27, P29, DOI 10.1051/agro:2006011"
"   Khan MS, 2009, SUSTAIN AGR REV, V1, P319, DOI 10.1007/978-1-4020-9654-9_15"
"   Khan MU, 2013, CHEMOSPHERE, V93, P2230, DOI 10.1016/j.chemosphere.2013.07.067"
"   Kidd KA, 2012, SCI TOTAL ENVIRON, V438, P135, DOI 10.1016/j.scitotenv.2012.08.057"
"   Kloepper J.W., 1978, P 4 INT C PLANT PATH"
"   KLOEPPER JW, 1992, CAN J MICROBIOL, V38, P1219, DOI 10.1139/m92-202"
"   Koptsik GN, 2014, EURASIAN SOIL SCI+, V47, P923, DOI 10.1134/S1064229314090075"
"   Kr??mer U, 2010, ANNU REV PLANT BIOL, V61, P517, DOI 10.1146/annurev-arplant-042809-112156"
"   Kucharski R, 2005, PLANT SOIL, V273, P291, DOI 10.1007/s11104-004-8068-6"
"   Kuffner M, 2010, J APPL MICROBIOL, V108, P1471, DOI 10.1111/j.1365-2672.2010.04670.x"
"   Kuffner M, 2008, PLANT SOIL, V304, P35, DOI 10.1007/s11104-007-9517-9"
"   K??pper H, 1999, PLANT PHYSIOL, V119, P305, DOI 10.1104/pp.119.1.305"
"   K??pper H, 2004, PLANT PHYSIOL, V134, P748, DOI 10.1104/pp.103.032953"
"   Lamba DT, 2009, J HAZARD MATER, V171, P1150, DOI 10.1016/j.jhazmat.2009.06.124"
"   Lasat MM, 2002, J ENVIRON QUAL, V31, P109, DOI 10.2134/jeq2002.0109"
"   Lasat MM, 2000, J EXP BOT, V51, P71, DOI 10.1093/jexbot/51.342.71"
"   Lee S, 2004, J BACTERIOL, V186, P5384, DOI 10.1128/JB.186.16.5384-5391.2004"
"   Li PZ, 2015, ECOTOX ENVIRON SAFE, V113, P391, DOI 10.1016/j.ecoenv.2014.12.025"
"   Li WC, 2010, PLANT SOIL, V326, P453, DOI 10.1007/s11104-009-0025-y"
"   Liu P, 2010, PLANT SOIL, V335, P385, DOI 10.1007/s11104-010-0427-x"
"   Liu WX, 2015, J SOIL SEDIMENT, V15, P1191, DOI 10.1007/s11368-015-1067-9"
"   Lombi E, 2001, J ENVIRON QUAL, V30, P1919, DOI 10.2134/jeq2001.1919"
"   Lu HL, 2007, ENVIRON EXP BOT, V61, P159, DOI 10.1016/j.envexpbot.2007.05.007"
"   Luo SL, 2011, CHEMOSPHERE, V85, P1130, DOI 10.1016/j.chemosphere.2011.07.053"
"   Luo SL, 2012, APPL MICROBIOL BIOT, V93, P1745, DOI 10.1007/s00253-011-3483-0"
"   Lynch JM, 2005, ANN APPL BIOL, V146, P217, DOI 10.1111/j.1744-7348.2005.040115.x"
"   Ma Y, 2011, BIOTECHNOL ADV, V29, P248, DOI 10.1016/j.biotechadv.2010.12.001"
"   Ma Y, 2016, J ENVIRON MANAGE, V174, P14, DOI 10.1016/j.jenvman.2016.02.047"
"   Machuca A, 2007, LETT APPL MICROBIOL, V44, P7, DOI 10.1111/j.1472-765X.2006.02046.x"

"   Macnair MR, 2003, ADV BOT RES, V40, P63, DOI 10.1016/S0065-2296(05)40002-6"
"   Mahrukh Zubair Mahrukh Zubair, 2016, Environmental Technology Reviews, V5, P112, DOI [10.1080/21622515.2016.1259358, 10.1080/21622515.2016.1259358]"
"   MALEA P, 1995, ISRAEL J PLANT SCI, V43, P21, DOI 10.1080/07929978.1995.10676587"
"   Marchiol L, 2013, ECOL ENG, V53, P343, DOI 10.1016/j.ecoleng.2012.12.072"
"   Mastretta C, 2009, INT J PHYTOREMEDIAT, V11, P251, DOI 10.1080/15226510802432678"
"   McCutcheon S.C., 2003, PHYTOREMEDIATION"
"   MCGRATH SP, 1995, J IND MICROBIOL, V14, P94, DOI 10.1007/BF01569890"
"   Meers E, 2010, CHEMOSPHERE, V78, P35, DOI 10.1016/j.chemosphere.2009.08.015"
"   Mehnaz S., 2013, Plant Microbe Symbiosis: Fundamentals and Advances, P373, DOI [10.1007/978-81-322-1287-4_14, DOI 10.1007/978-81-322-1287-4_14]"
"   Miransari M, 2011, BIOTECHNOL ADV, V29, P645, DOI 10.1016/j.biotechadv.2011.04.006"
"   Molas J, 1997, PHOTOSYNTHETICA, V34, P513, DOI 10.1023/A:1006805327340"
"   Muhammad S, 2013, BIOMED RES INT, V2013, DOI 10.1155/2013/194765"
"   Nabulo G, 2011, ENVIRON POLLUT, V159, P368, DOI 10.1016/j.envpol.2010.11.007"
"   Nadeem SM, 2014, BIOTECHNOL ADV, V32, P429, DOI 10.1016/j.biotechadv.2013.12.005"
"   Nadeem SM, 2010, CRIT REV PLANT SCI, V29, P360, DOI 10.1080/07352689.2010.524518"
"   Naidu R.Kookuna., 1996, Contaminants and the Soil Environment in the Australasia Pacific Region"
"   Navarro L, 2006, SCIENCE, V312, P436, DOI 10.1126/science.1126088"
"   Naz I, 2009, AFR J BIOTECHNOL, V8, P5762"
"   NEILANDS JB, 1981, ANNU REV BIOCHEM, V50, P715, DOI 10.1146/annurev.bi.50.070181.003435"
"   Nies DH, 1999, APPL MICROBIOL BIOT, V51, P730, DOI 10.1007/s002530051457"
"   Pan FS, 2017, INT J PHYTOREMEDIAT, V19, P281, DOI 10.1080/15226514.2016.1225280"
"   Pandey S, 2013, J MICROBIOL, V51, P11, DOI 10.1007/s12275-013-2330-7"
"   Pandey VC, 2016, INT J PHYTOREMEDIAT, V18, P450, DOI 10.1080/15226514.2015.1109605"
"   PANDOLFINI T, 1992, PLANT CELL ENVIRON, V15, P719, DOI 10.1111/j.1365-3040.1992.tb01014.x"
"   Paredes-P??liz K, 2018, PLANT BIOLOGY, V20, P497, DOI 10.1111/plb.12693"
"   Paredes-P??liz KI, 2016, MAR POLLUT BULL, V110, P396, DOI 10.1016/j.marpolbul.2016.06.036"
"   Pavel VL, 2013, EUR J SCI THEOL, V9, P211"
"   PEARSON JN, 1995, J EXP BOT, V46, P833, DOI 10.1093/jxb/46.7.833"
"   Phillips DA, 1999, P NATL ACAD SCI USA, V96, P12275, DOI 10.1073/pnas.96.22.12275"
"   Phillips RP, 2011, ECOL LETT, V14, P187, DOI 10.1111/j.1461-0248.2010.01570.x"
"   PICCINI DF, 1992, J PLANT NUTR, V15, P2343, DOI 10.1080/01904169209364478"
"   Poynton CY, 2004, PLANTA, V219, P1080, DOI 10.1007/s00425-004-1304-8"
"   Prapagdee B, 2013, CHEMOSPHERE, V92, P659, DOI 10.1016/j.chemosphere.2013.01.082"
"   Radjacommare R, 2004, PLANT SOIL, V266, P165"
"   Rai PK, 2008, INT J PHYTOREMEDIAT, V10, P430, DOI 10.1080/15226510802100606"
"   Rajkumar M, 2012, BIOTECHNOL ADV, V30, P1562, DOI 10.1016/j.biotechadv.2012.04.011"
"   Rajkumar M, 2010, TRENDS BIOTECHNOL, V28, P142, DOI 10.1016/j.tibtech.2009.12.002"
"   Rajkumar M, 2009, CHEMOSPHERE, V77, P153, DOI 10.1016/j.chemosphere.2009.06.047"
"   RASCIO N, 1977, OIKOS, V29, P250, DOI 10.2307/3543610"
"   Rascio N, 2011, PLANT SCI, V180, P169, DOI 10.1016/j.plantsci.2010.08.016"
"   Raven P.H., 1998, ENVIRONMENT, V2"
"   Reed MLE, 2005, CAN J MICROBIOL, V51, P1061, DOI 10.1139/W05-094"
"   Reeves RD, 2000, PHYTOREMEDIATION OF TOXIC METALS, P193"
"   [????????? REN Limin], 2007, [??????????????????, Journal of Soil and Water Conservation], V21, P81"
"   Robinson BH, 1998, PLANT SOIL, V203, P47, DOI 10.1023/A:1004328816645"
"   Rodr??guez H, 2006, PLANT SOIL, V287, P15, DOI 10.1007/s11104-006-9056-9"
"   Rodriguez L, 2007, INT J PHYTOREMEDIAT, V9, P1, DOI 10.1080/15226510601139359"
"   Rout GR, 2009, SUSTAINABLE AGRICULTURE, P873, DOI 10.1051/agro:2002073"
"   RUBIO MI, 1994, PLANT GROWTH REGUL, V14, P151, DOI 10.1007/BF00025217"
"   Ryan PR, 2001, ANNU REV PLANT PHYS, V52, P527, DOI 10.1146/annurev.arplant.52.1.527"
"   Ryan RP, 2008, FEMS MICROBIOL LETT, V278, P1, DOI 10.1111/j.1574-6968.2007.00918.x"
"   Safronova VI, 2006, BIOL FERT SOILS, V42, P267, DOI 10.1007/s00374-005-0024-y"
"   Sakakibara M, 2011, CLEAN-SOIL AIR WATER, V39, P735, DOI 10.1002/clen.201000488"
"   Salisbury F.B., 1992, Plant Physiology"
"   Salt DE, 1998, ANNU REV PLANT PHYS, V49, P643, DOI 10.1146/annurev.arplant.49.1.643"
"   Saravanan VS, 2007, CHEMOSPHERE, V66, P1794, DOI 10.1016/j.chemosphere.2006.07.067"
"   Sas-Nowosielska A, 2008, ENVIRON MONIT ASSESS, V137, P101, DOI 10.1007/s10661-007-9732-0"
"   Sasmaz M, 2016, INT J PHYTOREMEDIAT, V18, P69, DOI 10.1080/15226514.2015.1058334"
"   Sasmaz M, 2015, J ENVIRON MANAGE, V163, P246, DOI 10.1016/j.jenvman.2015.08.029"
"   Schalk IJ, 2011, ENVIRON MICROBIOL, V13, P2844, DOI 10.1111/j.1462-2920.2011.02556.x"
"   Seregin IV, 2001, RUSS J PLANT PHYSL+, V48, P523, DOI 10.1023/A:1016719901147"
"   Shah MT, 2014, CLEAN-SOIL AIR WATER, V42, P339, DOI 10.1002/clen.201200632"
"   Shahinpoor M., 2015, CHAPTER 1, V1, P1, DOI DOI 10.1007/978-3-319-23162-41"
"   Sheng XF, 2008, ENVIRON POLLUT, V156, P1164, DOI 10.1016/j.envpol.2008.04.007"
"   Sheng XF, 2008, J HAZARD MATER, V155, P17, DOI 10.1016/j.jhazmat.2007.10.107"
"   Sheoran N, 2015, MICROBIOL RES, V173, P66, DOI 10.1016/j.micres.2015.02.001"
"   Sheoran V, 2009, MINER ENG, V22, P1007, DOI 10.1016/j.mineng.2009.04.001"
"   Shi YW, 2009, BIOL FERT SOILS, V45, P645, DOI 10.1007/s00374-009-0376-9"
"   SHIER WT, 1994, J TOXICOL-TOXIN REV, V13, P205, DOI 10.3109/15569549409089960"
"   Shin MN, 2012, J HAZARD MATER, V199, P314, DOI 10.1016/j.jhazmat.2011.11.010"
"   Silver S., 2009, Encyclopedia of Microbiology, P220, DOI DOI 10.1016/B978-012373944-5.00267-4"
"   Sinha S, 2008, CURR MICROBIOL, V56, P55, DOI 10.1007/s00284-007-9038-z"
"   Souza LA, 2013, SCI AGR, V70, P290, DOI 10.1590/S0103-90162013000400010"
"   Spaepen S, 2007, FEMS MICROBIOL REV, V31, P425, DOI 10.1111/j.1574-6976.2007.00072.x"
"   Sresty TVS, 1999, ENVIRON EXP BOT, V41, P3, DOI 10.1016/S0098-8472(98)00034-3"
"   Sriprang R, 2003, APPL ENVIRON MICROB, V69, P1791, DOI 10.1128/AEM.69.3.1791-1796.2003"
"   Srivastava M, 2006, SCI TOTAL ENVIRON, V364, P24, DOI 10.1016/j.scitotenv.2005.11.002"
"   Sturz AV, 1996, PLANT SOIL, V184, P265, DOI 10.1007/BF00010455"
"   Sun RL, 2006, PLANT SOIL, V285, P125, DOI 10.1007/s11104-006-0064-6"
"   Sun Y., 2010, J ENV CHEM ECOTOX, V33, P45"
"   Tailor AJ, 2014, J PLANT NUTR, V37, P1534, DOI 10.1080/01904167.2014.911319"
"   Tank N, 2009, J BASIC MICROB, V49, P195, DOI 10.1002/jobm.200800090"

"   TARAFDAR JC, 1988, BIOL FERT SOILS, V5, P308, DOI 10.1007/BF00262137"
"   Thiem D, 2018, J BASIC MICROB, V58, P623, DOI 10.1002/jobm.201800034"
"   Tiwari S, 2018, FRONT PLANT SCI, V9, DOI 10.3389/fpls.2018.00452"
"   Traina SJ, 1999, P NATL ACAD SCI USA, V96, P3365, DOI 10.1073/pnas.96.7.3365"
"   Tripathi M, 2005, CURR MICROBIOL, V50, P233, DOI 10.1007/s00284-004-4459-4"
"   Trotta A, 2006, CHEMOSPHERE, V65, P74, DOI 10.1016/j.chemosphere.2006.02.048"
"   TYLER G, 1989, WATER AIR SOIL POLL, V47, P189, DOI 10.1007/BF00279327"
"   Ullah A, 2015, ENVIRON EXP BOT, V117, P28, DOI 10.1016/j.envexpbot.2015.05.001"
"   Ullah A, 2015, ENVIRON SCI POLLUT R, V22, P2505, DOI 10.1007/s11356-014-3699-5"
"   V??zquez MM, 2000, APPL SOIL ECOL, V15, P261, DOI 10.1016/S0929-1393(00)00075-5"
"   Verbon EH, 2016, TRENDS PLANT SCI, V21, P218, DOI 10.1016/j.tplants.2016.01.013"
"   Viterbo A, 2010, FEMS MICROBIOL LETT, V305, P42, DOI 10.1111/j.1574-6968.2010.01910.x"
"   Wachs B., 2000, Arch. Hjydrobiol. Suppl, V115/, P533"
"   Wakelin SA, 2004, BIOL FERT SOILS, V40, P36, DOI 10.1007/s00374-004-0750-6"
"   Wan Y, 2012, CHEMOSPHERE, V89, P743, DOI 10.1016/j.chemosphere.2012.07.005"
"   Wang JX, 2012, J HAZARD MATER, V221, P1, DOI 10.1016/j.jhazmat.2012.04.035"
"   Wang Q, 2011, J APPL MICROBIOL, V111, P1065, DOI 10.1111/j.1365-2672.2011.05142.x"
"   Wang WF, 2014, BIOMETALS, V27, P623, DOI 10.1007/s10534-014-9739-2"
"   Wani PA, 2007, CHEMOSPHERE, V70, P36, DOI 10.1016/j.chemosphere.2007.07.028"
"   Wei SH, 2008, J HAZARD MATER, V150, P662, DOI 10.1016/j.jhazmat.2007.05.014"
"   Wenzel WW, 2009, PLANT SOIL, V321, P385, DOI 10.1007/s11104-008-9686-1"
"   Weyens N, 2009, TRENDS BIOTECHNOL, V27, P591, DOI 10.1016/j.tibtech.2009.07.006"
"   Whiting SN, 2001, ENVIRON SCI TECHNOL, V35, P3144, DOI 10.1021/es001938v"
"   Yang J, 2009, TRENDS PLANT SCI, V14, P1, DOI 10.1016/j.tplants.2008.10.004"
"   Yang SX, 2008, PLANT SOIL ENVIRON, V54, P441, DOI 10.17221/401-PSE"
"   Yang X, 2006, PLANTA, V224, P185, DOI 10.1007/s00425-005-0194-8"
"   Zaidi S, 2006, CHEMOSPHERE, V64, P991, DOI 10.1016/j.chemosphere.2005.12.057"
"   Zarei M, 2008, ENVIRON POLLUT, V156, P1277, DOI 10.1016/j.envpol.2008.03.006"
"   Zarei M, 2010, ENVIRON POLLUT, V158, P2757, DOI 10.1016/j.envpol.2010.04.017"
"   Zhang XF, 2011, J HAZARD MATER, V189, P414, DOI 10.1016/j.jhazmat.2011.02.053"
"   Zloch M, 2016, CHEMOSPHERE, V156, P312, DOI 10.1016/j.chemosphere.2016.04.130"
NR 264
TC 141
Z9 164
U1 6
U2 385
PU PERGAMON-ELSEVIER SCIENCE LTD
PI OXFORD
"PA THE BOULEVARD, LANGFORD LANE, KIDLINGTON, OXFORD OX5 1GB, the United Kingdom"
SN 0045-6535
EI 1879-1298
J9 CHEMOSPHERE
JI Chemosphere
PD FEB
PY 2019
VL 217
BP 925
EP 941
DI 10.1016/j.chemosphere.2018.11.021
PG 17
WC Environmental Sciences
WE Science Citation Index Expanded (SCI-EXPANDED)
SC Environmental Sciences & Ecology
GA HI1RU
UT WOS:000456223500101
PM 30586789
DA 2026-04-06
ER

PT J
"AU Xu, MH"
"   Li, X"
"   Ye, QN"
"   Gong, F"
"   He, XL"
"AF Xu, Minghui"
"   Li, Xia"
"   Ye, Qiannan"
"   Gong, Feng"
"   He, Xueli"
TI Occurrence of dark septate endophytes in Phragmites australis in the
   Baiyang Lake and their resistance to Cd stress
SO PEDOSPHERE
LA English
DT Article
DE ascomycete; colonization rate; contaminated site; evenness index; heavy
   metal; Shannon-Wiener index; Simpson index; wetland
ID HEAVY-METALS; PLANT-GROWTH; MYCORRHIZAL FUNGI; DSE FUNGI; CADMIUM;
   TOLERANCE; SOIL; PHYTOREMEDIATION; ACCUMULATION; RHIZOSPHERE
"AB Heavy metal pollution poses a serious hazard to human health, and microbial remediation of heavy metals in soil has been widely studied. A group of ascomycetes classified as dark septate endophytes (DSEs) colonize plant roots and benefit host plants under abiotic stress conditions. In this study, Phragmites australis , a common remediation plant in the Baiyang Lake in North China, was investigated. Soils and roots of P. australis were collected in typical heavy metal-contaminated sites, and the species diversity and community

structure of DSEs in P. australis roots were studied. In addition, DSE strains were isolated, cultured, and tested for their tolerance to Cd stress. The results showed that DSEs occurred extensively in P. australis roots, forming typical dark septate hyphae, with a total colonization rate of 19.7%-83.1%. Morphological and internal transcribed spacer sequencing analyses were used to identify 10 species within 9 genera of DSE fungi. Among these fungi, 6 strains with considerable resistance to Cd stress were identified. The biomasses of Poaceascoma helicoides , Alternaria doliconidium , and Acrocalymma vagum strains increased as the Cd levels increased. These results can not only help to understand plant-DSE interactions in wetland environments, but also provide a theoretical basis for making full use of DSE fungi to alleviate heavy metal contamination in soil."
"C1 [Xu, Minghui; Li, Xia; Ye, Qiannan; Gong, Feng; He, Xueli] Hebei Univ, Sch Life Sci, Baoding 071002, Peoples R China."
"   [Li, Xia; He, Xueli] Hebei Univ, Key Lab Microbial Divers Res & Applicat Hebei Prov, Baoding 071002, Peoples R China."
C3 Hebei University; Hebei University
"RP Li, X; He, XL (corresponding author), Hebei Univ, Sch Life Sci, Baoding 071002, Peoples R China.; Li, X; He, XL (corresponding author), Hebei Univ, Key Lab Microbial Divers Res & Applicat Hebei Prov, Baoding 071002, Peoples R China."
EM wulan2050@126.com; xlh3615@126.com
"FU National Natural Science Foundation of China [31800345, 31770561];"
"   National Science Foundation of Hebei Province, China [C2020201043,"
   22E50029D]; Natural Science Interdisciplinary Research Program of Hebei
"   University, China [DXK202311]; Post-graduate's Innovation Fund Project"
"   of Hebei Province, China [CXZ-ZSS2023010]"
FX This research was supported by the National Natural Science Foundation
"   of China (Nos. 31800345 and 31770561) , the National Science Foundation"
"   of Hebei Province, China (Nos. C2020201043 and 22E50029D) , the Natural"
"   Science Interdisciplinary Research Program of Hebei University, China"
"   (No. DXK202311) , and the Post-graduate's Inno- vation Fund Project of"
"   Hebei Province, China (No. CXZ-ZSS2023010) ."
"CR Abhijit Gupta Abhijit Gupta, 2016, Journal of Microbial and Biochemical Technology, V8, P364, DOI 10.4172/1948-5948.1000310"
"   An HM, 2015, MICROBIOL RES, V176, P29, DOI 10.1016/j.micres.2015.03.013"
"   Arias JA, 2010, ENVIRON SCI TECHNOL, V44, P7272, DOI 10.1021/es1008664"
"   Ayangbenro AS, 2017, INT J ENV RES PUB HE, V14, DOI 10.3390/ijerph14010094"
"   Baba H, 2013, MODERN PATHOL, V26, P1228, DOI 10.1038/modpathol.2013.62"
"   Ban YH, 2021, J HAZARD MATER, V416, DOI 10.1016/j.jhazmat.2021.126095"
"   Ban YH, 2017, PEDOSPHERE, V27, P283, DOI [10.1016/s1002-0160(17)60316-3, 10.1016/S1002-0160(17)60316-3]"
"   Ban YH, 2015, ANN MICROBIOL, V65, P503, DOI 10.1007/s13213-014-0886-z"
"   Ban YH, 2012, PLOS ONE, V7, DOI 10.1371/journal.pone.0047968"
"   Berthelot C, 2020, MICROORGANISMS, V8, DOI 10.3390/microorganisms8040537"
"   Berthelot C, 2016, FEMS MICROBIOL ECOL, V92, DOI 10.1093/femsec/fiw144"
"   Berthelot C, 2018, MYCORRHIZA, V28, P301, DOI 10.1007/s00572-018-0826-z"
"   Bhargava P, 2017, NAT REV NEPHROL, V13, P629, DOI 10.1038/nrneph.2017.107"
"   BIERMANN B, 1981, NEW PHYTOL, V87, P63, DOI 10.1111/j.1469-8137.1981.tb01690.x"
"   BREMNER J. M., 1960, JOUR AGRIC SCI, V55, P11, DOI 10.1017/S0021859600021572"
"   Clay K, 2016, BIOL INVASIONS, V18, P2703, DOI 10.1007/s10530-016-1137-y"
"   Cornelissen JHC, 2003, AUST J BOT, V51, P335, DOI 10.1071/BT02124"
"   Diene O, 2014, PLOS ONE, V9, DOI 10.1371/journal.pone.0109233"
"   Din BU, 2020, PLANT PHYSIOL BIOCH, V146, P249, DOI 10.1016/j.plaphy.2019.11.010"
"   Dixon P, 2003, J VEG SCI, V14, P927, DOI 10.1111/j.1654-1103.2003.tb02228.x"
"   DRAPER MH, 1994, SCI TOTAL ENVIRON, V148, P263, DOI 10.1016/0048-9697(94)90402-2"
"   Egidi E, 2019, NAT COMMUN, V10, DOI 10.1038/s41467-019-10373-z"
"   ELLIS DH, 1974, CAN J MICROBIOL, V20, P1379, DOI 10.1139/m74-212"
"   Feng NX, 2017, SCI TOTAL ENVIRON, V583, P352, DOI 10.1016/j.scitotenv.2017.01.075"
"   Gessler NN, 2014, APPL BIOCHEM MICRO+, V50, P105, DOI 10.1134/S0003683814020094"
"   Gheju M, 2017, J ENVIRON MANAGE, V203, P920, DOI 10.1016/j.jenvman.2017.04.094"
"   Guo HM, 2020, ENVIRON SCI TECHNOL, V54, P10068, DOI 10.1021/acs.est.0c02357"
"   Hejna M, 2020, CHEMOSPHERE, V241, DOI 10.1016/j.chemosphere.2019.125018"
"   Hou LF, 2020, FRONT MICROBIOL, V10, DOI 10.3389/fmicb.2019.03061"
"   Ji ZH, 2019, ECOTOX ENVIRON SAFE, V174, P417, DOI 10.1016/j.ecoenv.2019.02.062"
"   Jin ZM, 2019, SCI TOTAL ENVIRON, V697, DOI 10.1016/j.scitotenv.2019.134148"
"   Jumpponen A, 1998, NEW PHYTOL, V140, P295, DOI 10.1046/j.1469-8137.1998.00265.x"
"   Khan AR, 2017, ECOTOX ENVIRON SAFE, V136, P180, DOI 10.1016/j.ecoenv.2016.03.014"
"   Knapp DG, 2019, FRONT MICROBIOL, V10, DOI 10.3389/fmicb.2019.02565"
"   Knapp DG, 2012, PLOS ONE, V7, DOI 10.1371/journal.pone.0032570"
"   K??bbing JF, 2013, MIRES PEAT, V13"
"   Kumari M, 2018, BIORESOUR TECH REP, V3, P59, DOI [10.1016/j.biteb.2018.06.001, 10.1016/j.biteb.2018.06.001, DOI 10.1016/J.BITEB.2018.06.001]"
"   Lahori AH, 2017, ECOTOX ENVIRON SAFE, V145, P313, DOI 10.1016/j.ecoenv.2017.07.049"
"   Li HY, 2012, FUNGAL ECOL, V5, P309, DOI 10.1016/j.funeco.2011.06.002"
"   Li LJ, 2021, J HAZARD MATER, V401, DOI 10.1016/j.jhazmat.2020.123370"
"   Li SZ, 2020, J HAZARD MATER, V400, DOI 10.1016/j.jhazmat.2020.123255"
"   Li T, 2011, SCI TOTAL ENVIRON, V409, P1069, DOI 10.1016/j.scitotenv.2010.12.012"
"   Likar M, 2013, PLANT SOIL, V370, P593, DOI 10.1007/s11104-013-1656-6"
"   Likar M, 2009, SCI TOTAL ENVIRON, V407, P6179, DOI 10.1016/j.scitotenv.2009.08.045"
"   Maadon SN, 2018, SAINS MALAYS, V47, P3025, DOI 10.17576/jsm-2018-4712-12"
"   Mateu MG, 2020, ISME J, V14, P1943, DOI 10.1038/s41396-020-0654-y"
"   McGrath SP, 2001, PLANT SOIL, V232, P207, DOI 10.1023/A:1010358708525"
"   Ndinga-Muniania C, 2021, MYCOLOGIA, V113, P1181, DOI 10.1080/00275514.2021.1965852"
"   Newsham KK, 2011, NEW PHYTOL, V190, P783, DOI 10.1111/j.1469-8137.2010.03611.x"
"   Niu Y, 2020, SCI TOTAL ENVIRON, V700, DOI 10.1016/j.scitotenv.2019.134509"
"   Onakpa MM, 2018, ANN GLOB HEALTH, V84, P488, DOI 10.29024/aogh.2314"
"   PHILLIPS JM, 1970, T BRIT MYCOL SOC, V55, P158, DOI 10.1016/S0007-1536(70)80110-3"
"   Potisek M, 2021, ECOTOX ENVIRON SAFE, V222, DOI 10.1016/j.ecoenv.2021.112493"

"   Riaz M, 2021, J HAZARD MATER, V402, DOI 10.1016/j.jhazmat.2020.123919"
"   Rocha AG, 2018, MITOCHONDRION, V40, P29, DOI 10.1016/j.mito.2017.09.003"
"   Rowell D. L., 1994, Soil science: methods and applications."
"   Shadmani L, 2021, BRAZ J MICROBIOL, V52, P1097, DOI 10.1007/s42770-021-00493-4"
"   Shi YA, 2017, ENVIRON SCI POLLUT R, V24, P417, DOI 10.1007/s11356-016-7693-y"
"   Sieber TN, 2013, PLANT ROOTS: THE HIDDEN HALF, 4TH EDITION"
"   Sprenger HG, 2019, TRENDS CELL BIOL, V29, P888, DOI 10.1016/j.tcb.2019.08.003"
"   Su ZZ, 2021, J HAZARD MATER, V419, DOI 10.1016/j.jhazmat.2021.126435"
"   Tamura K, 2013, MOL BIOL EVOL, V30, P2725, DOI 10.1093/molbev/mst197"
"   Wang L, 2017, ENVIRON SCI POLLUT R, V24, P3593, DOI 10.1007/s11356-016-7984-3"
"   Wei W, 2014, ENVIRON SCI TECHNOL, V48, P3363, DOI 10.1021/es4046567"
"   Xie LL, 2017, FUNGAL ECOL, V26, P135, DOI 10.1016/j.funeco.2017.01.007"
"   Xu Z, 2022, CHEM ENG J, V429, DOI 10.1016/j.cej.2021.131688"
"   Yang X, 2020, ENVIRON POLLUT, V264, DOI 10.1016/j.envpol.2020.114745"
"   Yu Y, 2021, BMC MICROBIOL, V21, DOI 10.1186/s12866-021-02098-1"
"   Yung L, 2021, ENVIRON SCI POLLUT R, V28, P16544, DOI 10.1007/s11356-020-11793-x"
"   Zhan FD, 2016, B ENVIRON CONTAM TOX, V96, P235, DOI 10.1007/s00128-015-1676-4"
"   Zhan FD, 2015, ENVIRON SCI POLLUT R, V22, P17897, DOI 10.1007/s11356-015-5012-7"
"   Zhang JL, 2022, FRONT PLANT SCI, V13, DOI 10.3389/fpls.2022.882359"
"   Zhang Shurong, 2013, ACTA BOTANICA BOREALI-OCCIDENTALIA SINICA, V33, P1891"
"   Zhang YJ, 2008, J MICROBIOL, V46, P624, DOI 10.1007/s12275-008-0163-6"
"   Zhong X, 2023, J HAZARD MATER, V443, DOI 10.1016/j.jhazmat.2022.130241"
"   Zhu LL, 2018, ENVIRON SCI POLLUT R, V25, P35232, DOI 10.1007/s11356-018-3456-2"
NR 76
TC 11
Z9 12
U1 10
U2 50
PU SCIENCE PRESS
PI BEIJING
"PA 16 DONGHUANGCHENGGEN NORTH ST, BEIJING 100717, PEOPLES R CHINA"
SN 1002-0160
EI 2210-5107
J9 PEDOSPHERE
JI Pedosphere
PD APR
PY 2024
VL 34
IS 2
BP 484
EP 496
DI 10.1016/j.pedsph.2023.07.009
EA APR 2024
PG 13
WC Soil Science
WE Science Citation Index Expanded (SCI-EXPANDED)
SC Agriculture
GA SO2I5
UT WOS:001235323300001
DA 2026-04-06
ER

PT J
"AU Damodaran, D"
"   Shetty, KV"
"   Mohan, BR"
"AF Damodaran, Dilna"
"   Shetty, K. Vidya"
"   Mohan, B. Raj"
"TI Effect of chelaters on bioaccumulation of Cd (II), Cu (II), Cr (VI), Pb"
   (II) and Zn (II) in Galerina vittiformis from soil
SO INTERNATIONAL BIODETERIORATION & BIODEGRADATION
LA English
DT Article
DE Bioremediation; Galerina vittiformis; Heavy metals; Mycoremediation;
   Soil pollution
ID HEAVY-METAL POLLUTION; CONTAMINATED SOILS; PHANEROCHAETE-CHRYSOSPORIUM;
   CITRIC-ACID; PHYTOEXTRACTION; EDTA; LEAD; BIOREMEDIATION; BIOSORPTION;
   MUSHROOMS
"AB Remediation of heavy metal contaminated soil and water streams are of great necessity as heavy metals are toxic and pose hazardous ecological impacts. Low cost mitigation measures like phytoremediation and mycoremediation are commonly employed. Mycoremediation using macro fungi (mushroom) have proven to provide effective tolerance using an efficient accumulation mechanism in removing heavy metals from soil. The current paper reports the heavy metal remediation potential of macro fungi on soil artificially contaminated with 50 mg kg(-1) of Cu (II), Cd (II), Cr (VI), Pb (II), and Zn (II) ions. Galerina vittiformis belonging to Strophariacea family was found to be effective in removing the heavy metal from the soil under study within 30 days. Both chemical and biological chelaters at 1, 5, and 10 mmol kg(-1) concentrations were found to increase the mycoremediation potential of the organism. This study showed that G. vittiformis are efficient in remediating heavy metal from contaminated soil and that their remediation potential can be enhanced by the addition of chelaters. (C) 2013 Elsevier Ltd. All rights reserved."
"C1 [Damodaran, Dilna; Shetty, K. Vidya; Mohan, B. Raj] Natl Inst Technol Karnataka, Dept Chem Engn, Bangalore 575025, Karnataka, India."
C3 National Institute of Technology (NIT System); National Institute of
   Technology Karnataka

"RP Mohan, BR (corresponding author), Natl Inst Technol Karnataka, Dept Chem Engn, Bangalore 575025, Karnataka, India."
EM rajmohanbala@gmail.com
"CR [Anonymous], 1997, TE9801 GROUND WAT RE"
"   [Anonymous], 2000, J YANTAI U NAT SCI E"
"   Atanassova I, 1999, WATER AIR SOIL POLL, V113, P115, DOI 10.1023/A:1005076821325"
"   Baldrian P, 2003, ENZYME MICROB TECH, V32, P78, DOI 10.1016/S0141-0229(02)00245-4"
"   Bardan J.S., 2012, CLEAN-SOIL AIR WATER, V40, P607"
"   Basile A, 2008, ENVIRON POLLUT, V151, P401, DOI 10.1016/j.envpol.2007.07.004"
"   Blaylock MJ, 2000, PHYTOREMEDIATION OF TOXIC METALS, P53"
"   Bliefert C., 1994, UMWELTCHEMIE, P336"
"   Bothe H, 2010, MICROBIOL MOL BIOL R, V74, P529, DOI 10.1128/MMBR.00033-10"
"   Bushnell LD, 1941, J BACTERIOL, V41, P653, DOI 10.1128/JB.41.5.653-673.1941"
"   Cao A, 2007, EUR J SOIL BIOL, V43, P200, DOI 10.1016/j.ejsobi.2007.02.002"
"   ??ayir A, 2010, BIOL TRACE ELEM RES, V134, P212, DOI 10.1007/s12011-009-8464-0"
"   Chen H, 2001, CHEMOSPHERE, V45, P21, DOI 10.1016/S0045-6535(01)00031-5"
"   Chen HM, 2000, CHEMOSPHERE, V41, P229, DOI 10.1016/S0045-6535(99)00415-4"
"   Chen LA, 2010, APPL SOIL ECOL, V46, P383, DOI 10.1016/j.apsoil.2010.10.003"
"   Chen XH, 2009, B ENVIRON CONTAM TOX, V83, P280, DOI 10.1007/s00128-009-9767-8"
"   Cheng SP, 2003, ENVIRON SCI POLLUT R, V10, P192, DOI 10.1065/espr2002.11.141.1"
"   Cho YG, 2000, BIOTECHNOL LETT, V22, P915, DOI 10.1023/A:1005612232079"
"   Demirbas A, 2001, FOOD CHEM, V75, P453, DOI 10.1016/S0308-8146(01)00236-9"
"   Diels L, 1999, MOL BIOTECHNOL, V12, P149, DOI 10.1385/MB:12:2:149"
"   do Nascimento CWA, 2006, ENVIRON POLLUT, V140, P114, DOI 10.1016/j.envpol.2005.06.017"
"   Evangelou MWH, 2007, CHEMOSPHERE, V68, P989, DOI 10.1016/j.chemosphere.2007.01.062"
"   Falandysz J, 2007, J ENVIRON SCI HEAL B, V42, P735, DOI 10.1080/03601230701466005"
"   GAST CH, 1988, CHEMOSPHERE, V17, P789, DOI 10.1016/0045-6535(88)90258-5"
"   Ge W, 2011, ADSORPTION, V17, P901, DOI 10.1007/s10450-011-9359-x"
"   Gupta VK, 2001, WATER RES, V35, P1125, DOI 10.1016/S0043-1354(00)00389-4"
"   Hamman S., 2004, J MOL BIOL, V23, P234, DOI DOI 10.1007/978-3-642-233342-5"
"   Haswell SJ., 1991, Atomic absorption spectrometry"
"   Hornburg V., 1995, EXTRAKTION MOBILER S, V158, P137"
"   Huang DL, 2008, ENVIRON SCI TECHNOL, V42, P4946, DOI 10.1021/es800072c"
"   Huang DL, 2006, J HAZARD MATER, V134, P268, DOI 10.1016/j.jhazmat.2005.11.021"
"   Isildak ??, 2004, FOOD CHEM, V86, P547, DOI 10.1016/j.foodchem.2003.09.007"
"   Isildak O, 2007, ANAL LETT, V40, P1099, DOI 10.1080/00032710701297042"
"   Ita B. N., 2006, ENZYME MICROBIAL TEC, V32, P78"
"   Jean L, 2008, ENVIRON POLLUT, V153, P555, DOI 10.1016/j.envpol.2007.09.013"
"   Kalac P, 2000, FOOD CHEM, V69, P273, DOI 10.1016/S0308-8146(99)00264-2"
"   Kamyotra S., 2009, ECOLOGICAL IMPACT AS, P56"
"   Konuk M, 2007, FRESEN ENVIRON BULL, V16, P1359"
"   Kos B., 2004, J PLANT SOIL BIOL, V253, P403"
"   Lai HY, 2005, CHEMOSPHERE, V60, P1062, DOI 10.1016/j.chemosphere.2005.01.020"
"   Li ZB, 1996, SOIL SCI, V161, P656, DOI 10.1097/00010694-199610000-00003"
"   Lombi E, 2001, J ENVIRON QUAL, V30, P1919, DOI 10.2134/jeq2001.1919"
"   Luciano A, 2012, CHEM ENGINEER TRANS, V28, P151, DOI 10.3303/CET1228026"
"   Luo CL, 2006, CHEMOSPHERE, V63, P1773, DOI 10.1016/j.chemosphere.2005.09.050"
"   Machuca A, 2001, WORLD J MICROB BIOT, V17, P687, DOI 10.1023/A:1012929112523"
"   MAHAVI P, 2005, CHEMOSPHERE, V54, P1522"
"   Malinowska E, 2004, FOOD CHEM, V84, P405, DOI 10.1016/S0308-8146(03)00250-4"
"   Mancini G, 2011, CHEM ECOL, V27, P251, DOI 10.1080/02757540.2010.547492"
"   Milenkovic N, 2005, POL J ENVIRON STUD, V14, P781"
"   Mishra V, 2010, CLEAN-SOIL AIR WATER, V38, P1062, DOI 10.1002/clen.201000030"
"   Mitra K., 1994, THESIS U CALCUTTA IN"
"   Moore R., 1998, BOTANY, V2nd"
"   Morsy FM, 2011, CLEAN-SOIL AIR WATER, V39, P680, DOI 10.1002/clen.201000312"
"   Niu ZX, 2007, J ENVIRON SCI, V19, P961, DOI 10.1016/S1001-0742(07)60158-2"
"   Oei P., 1996, Mushroom cultivation: With special emphasis on appropriate techniques for developing countries"
"   REDDY KJ, 1995, PLANT SOIL, V171, P53, DOI 10.1007/BF00009564"
"   Rudawska M, 2005, FOOD CHEM, V92, P499, DOI 10.1016/j.foodchem.2004.08.017"
"   Saifullah, 2009, CHEMOSPHERE, V74, P1279, DOI 10.1016/j.chemosphere.2008.11.007"
"   Schmidt U, 2003, J ENVIRON QUAL, V32, P1939, DOI 10.2134/jeq2003.1939"
"   Sheoran V., 2010, INT J SOIL SEDIMENT, V3, P15"
"   Sinhal VK, 2010, J ENVIRON BIOL, V31, P255"
"   Srivastava S, 2006, CURR MICROBIOL, V53, P232, DOI 10.1007/s00284-006-0103-9"
"   Sun B, 2005, J APPL POLYM SCI, V97, P2215, DOI 10.1002/app.21867"
"   Sun YB, 2009, GEODERMA, V150, P106, DOI 10.1016/j.geoderma.2009.01.016"
"   Thomet U, 1999, EUR FOOD RES TECHNOL, V209, P317, DOI 10.1007/s002170050502"
"   Turkekul I, 2004, FOOD CHEM, V84, P389, DOI 10.1016/S0308-8146(03)00245-0"
"   TYLER G, 1980, T BRIT MYCOL SOC, V74, P41, DOI 10.1016/S0007-1536(80)80005-2"
"   Ullah R, 2011, AFR J BIOTECHNOL, V10, P17192, DOI 10.5897/AJB11.1832"
"   Vimala R, 2009, J HAZARD MATER, V168, P376, DOI 10.1016/j.jhazmat.2009.02.062"
"   VOLESKY B, 1995, BIOTECHNOL PROGR, V11, P235, DOI 10.1021/bp00033a001"
"   Wenger K., 2005, Biogeochemistry of Chelating Agents, V910, P204, DOI [10.1021/bk-2005-0910.ch012, DOI 10.1021/BK-2005-0910.CH012]"
"   Wuyep PA, 2007, SCI RES ESSAYS, V2, P217"
"   Yilmaz Fadime, 2003, Turkish Journal of Botany, V27, P45"
"   Zhao ZQ, 2010, J HAZARD MATER, V181, P455, DOI 10.1016/j.jhazmat.2010.05.032"
"   Zhu FK, 2011, ENVIRON MONIT ASSESS, V179, P191, DOI 10.1007/s10661-010-1728-5"
NR 75
TC 45
Z9 54
U1 1

U2 97
PU ELSEVIER SCI LTD
PI OXFORD
"PA THE BOULEVARD, LANGFORD LANE, KIDLINGTON, OXFORD OX5 1GB, OXON, the United Kingdom"
SN 0964-8305
EI 1879-0208
J9 INT BIODETER BIODEGR
JI Int. Biodeterior. Biodegrad.
PD NOV
PY 2013
VL 85
BP 182
EP 188
DI 10.1016/j.ibiod.2013.05.031
PG 7
WC Biotechnology & Applied Microbiology; Environmental Sciences
WE Science Citation Index Expanded (SCI-EXPANDED)
SC Biotechnology & Applied Microbiology; Environmental Sciences & Ecology
GA 254LD
UT WOS:000327166300025
DA 2026-04-06
ER

PT J
"AU Zhao, ZG"
"   Wang, YF"
"   Kong, LH"
"   Zhao, LY"
"   Li, YC"
"   He, T"
"   Liu, SG"
"   Han, XC"
"   Gu, SS"
"   Wu, SY"
"   Zhang, JH"
"   Zhang, JT"
"   Li, B"
"AF Zhao, Zhigang"
"   Wang, Yongfeng"
"   Kong, Linghao"
"   Zhao, Liyuan"
"   Li, Yachao"
"   He, Teng"
"   Liu, Shigao"
"   Han, Xiangcai"
"   Gu, Songsong"
"   Wu, Shuyu"
"   Zhang, Jiahao"
"   Zhang, Jintao"
"   Li, Bing"
TI Response of soil microbial community composition and function to
   prolonged heavy metal exposure
SO ENVIRONMENTAL MONITORING AND ASSESSMENT
LA English
DT Article
DE Heavy metal exposure; Microbial community composition; Microbial
   function; Surface soil; High-throughput sequencing
ID BACTERIA; RESISTANCE; CADMIUM; MECHANISMS; REVEALS; IMPACT;
   BIOMINERALIZATION; MICROORGANISMS; CONTAMINATION; POLLUTION
"AB Understanding the effects of heavy metals on microbial community composition and function is crucial for environmental restoration. In this paper, soil samples with low, medium, and high levels of potential ecological risk (RI) associated with heavy metals were collected from a gold mining area in northern Laizhou, Shandong Province, Eastern China. The impact of heavy metals on soil microbial communities was assessed through Illumina high-throughput sequencing of 16S rRNA gene amplicons. The results demonstrated that while microbial community evenness remained relatively stable across varying RI levels, significant differences were observed in microbial community richness and composition. Canonical correlation analysis (CCA) revealed that nutrients were the primary factors shaping microbial communities under low RI levels, whereas pH and heavy metals played dominant roles under high RI levels. At the genus level, several taxa, including Acinetobacter, Paracoccus, Marinobacter, Halomonas, Streptococcus, Lactobacillus, Sulfobacillus, Sulfurifustis, Bacillus, and Pseudomonas, were identified as particularly tolerant to heavy metal stress. Co-occurrence network analysis showed that microbial networks were more complex and stable under low contamination, while increased cooperative interactions were observed under high contamination. At the phylum level, Proteobacteria, Firmicutes, and Bacteroidetes emerged as the key taxa in high RI soils. Functional predictions indicated that microbial processes related to replication and repair, extracellular polymeric substance (EPS) biosynthesis, membrane transport, and heavy metal resistance were significantly enhanced in high-risk environments. Keystone taxa employed various survival strategies, including extracellular polymerization, nutrient uptake, intracellular sequestration, active efflux systems, and collaboration with plants, to resist heavy metal stress. These findings provide new insights into the mechanisms of microbial adaptation and remediation in heavy metal-contaminated soils."
"C1 [Zhao, Zhigang] China Univ Geosci, Sch Earth Sci, Wuhan 430074, Peoples R China."
"   [Zhao, Zhigang; Wang, Yongfeng; Kong, Linghao; Zhao, Liyuan; Li, Yachao; He, Teng; Liu, Shigao; Han, Xiangcai; Gu, Songsong; Wu, Shuyu; Zhang, Jiahao; Zhang, Jintao; Li, Bing] China Geol Survey, Yantai Ctr Coastal Zone Geol Survey, Yantai 264000, Peoples R China."
C3 China University of Geosciences; China Geological Survey

"RP Wang, YF (corresponding author), China Geol Survey, Yantai Ctr Coastal Zone Geol Survey, Yantai 264000, Peoples R China."
EM 156513449@qq.com; wangyongfeng1204@163.com; 1245046910@qq.com;
353840397@qq.com; 1095779284@qq.com; 15169762229@163.com;
758187672@qq.com; 1771879956@qq.com; 372978364@qq.com;
hnwushuyu@163.com; 18769441868@163.com; 1026176676@qq.com;
493907595@qq.com
"RI ; Zhang, Jiahao/PIH-5391-2026"
"OI wu, shuyu/0000-0002-0746-7477; Zhao, Liyuan/0000-0002-4332-6380; "
FU China Geological Survey [DD20208080]; Science and Technology Innovation
Fund of Command Center of Natural Resources Comprehensive Survey
[KC20220011]; Characterization of Carboniferous-Early Permian
heterogeneous porous carbonate reservoirs and hydrocarbon potential
analysis in the central uplift of the South Yellow Sea Basin [KLSG2304];
"   Key Laboratory of Submarine Science, Ministry of Natural Resources"
FX This work was supported by the China Geological Survey (Grant No.
"   DD20208080), Science and Technology Innovation Fund of Command Center of"
"   Natural Resources Comprehensive Survey entitled ""Temporal and spatial"
distri-bution of palaeochannel and origin of organic carbon burial in
"   the Western Bohai Sea since 2.28 Ma"" (KC20220011), and ""Characterization"
of Carboniferous-Early Permian heterogeneous porous carbonate reservoirs
and hydrocarbon potential analysis in the central uplift of the South
"   Yellow Sea Basin"" (KLSG2304) found by the Key Laboratory of Submarine"
"   Science, Ministry of Natural Resources"
"CR Azarbad H, 2015, FEMS MICROBIOL ECOL, V91, DOI 10.1093/femsec/fiu003"
"   Banerjee S, 2016, SOIL BIOL BIOCHEM, V97, P188, DOI 10.1016/j.soilbio.2016.03.017"
"   Chakravarty R, 2012, BIORESOURCE TECHNOL, V108, P176, DOI 10.1016/j.biortech.2011.12.100"
"   Chen LM, 2023, SCI TOTAL ENVIRON, V856, DOI 10.1016/j.scitotenv.2022.159223"
"   Chen Y, 2018, SCI TOTAL ENVIRON, V637, P1400, DOI 10.1016/j.scitotenv.2018.05.109"
"   Cheng HF, 2009, J HAZARD MATER, V165, P13, DOI 10.1016/j.jhazmat.2008.10.070"
"   [????????? Dai Jierui], 2011, [????????????, Geochimica], V40, P577"
"   Das SK, 2012, ACS NANO, V6, P6165, DOI 10.1021/nn301502s"
"   Deng Y, 2016, ENVIRON MICROBIOL, V18, P205, DOI 10.1111/1462-2920.12981"
"   Deng Y, 2012, BMC BIOINFORMATICS, V13, DOI 10.1186/1471-2105-13-113"
"   Drobnik T, 2018, ECOL INDIC, V94, P151, DOI 10.1016/j.ecolind.2018.06.052"
"   Fairbrother A, 2007, ECOTOX ENVIRON SAFE, V68, P145, DOI 10.1016/j.ecoenv.2007.03.015"
"   Feng Hui-lin, 2021, Huanjing Kexue, V42, P422, DOI [10.13227/j.hjkx.202005285, 10.13227/j.hjkx.202005285]"
"   Gadd GM, 2010, MICROBIOL-SGM, V156, P609, DOI 10.1099/mic.0.037143-0"
"   Gan CD, 2022, J HAZARD MATER, V439, DOI 10.1016/j.jhazmat.2022.129624"
"   Gan CD, 2022, J HAZARD MATER, V429, DOI 10.1016/j.jhazmat.2021.128032"
"   Gu JD., 2019, Biotechnology, V4, P1"
"   Guo HH, 2017, ECOTOX ENVIRON SAFE, V144, P300, DOI 10.1016/j.ecoenv.2017.06.048"
"   Guo SY, 2021, ENVIRON CHEM LETT, V19, P1413, DOI 10.1007/s10311-020-01114-6"
"   HAKANSON L, 1980, WATER RES, V14, P975, DOI 10.1016/0043-1354(80)90143-8"
"   [????????? HAN Chunmei], 2005, [????€??????????, Chinese Journal of Ecology], V24, P1499"
"   [????????? Han Guiqi], 2010, [?????????????????, Journal of Soil and Water Conservation], V24, P238"
"   Hou D. M., 2020, Doctor's thesis,"
"   [????????? Hu Yonghao], 2023, [????????????, Geoscience], V37, P758"
"   Huang Shanshan, 2020, Weishengwu Xuebao, V60, P1985, DOI 10.13343/j.cnki.wsxb.20200048"
"   Huang Y, 2018, J ENVIRON MANAGE, V207, P159, DOI 10.1016/j.jenvman.2017.10.072"
"   International Organization for Standardization, 2022, Soil, treated biowaste and sludge-Determination of pH (ISO 10390)"
"   Jiang B, 2019, J HAZARD MATER, V362, P187, DOI 10.1016/j.jhazmat.2018.08.060"
"   Kamika I, 2013, BMC MICROBIOL, V13, DOI 10.1186/1471-2180-13-28"
"   Kang S, 2013, FEMS MICROBIOL ECOL, V86, P200, DOI 10.1111/1574-6941.12152"
"   Kaplan H, 2014, BIOL FERT SOILS, V50, P735, DOI 10.1007/s00374-013-0890-7"
"   Karelov?? E, 2011, BIOLOGIA, V66, P18, DOI 10.2478/s11756-010-0145-0"
"   Kolton M, 2011, APPL ENVIRON MICROB, V77, P4924, DOI 10.1128/AEM.00148-11"
"   Lengke M, 2006, GEOCHIM COSMOCHIM AC, V70, P3646, DOI 10.1016/j.gca.2006.04.018"
"   Li M, 2013, INT BIODETER BIODEGR, V76, P81, DOI 10.1016/j.ibiod.2012.06.016"
"   Li Q, 2020, ECOTOX ENVIRON SAFE, V204, DOI 10.1016/j.ecoenv.2020.111083"
"   Li SZ, 2020, J HAZARD MATER, V400, DOI 10.1016/j.jhazmat.2020.123255"
"   [????????? Li Weifeng], 2017, [??????????????????, Journal of Natural Resources], V32, P1942"
"   [?????? Liu Wei], 2025, [????????????????€???, Environmental Science and Technology], V48, P190"
"   Liu YR, 2018, MICROBIOME, V6, DOI 10.1186/s40168-018-0572-7"
"   LOEPPERT RH, 1984, SOIL SCI SOC AM J, V48, P1030, DOI 10.2136/sssaj1984.03615995004800050016x"
"   Loni PC, 2020, J HAZARD MATER, V385, DOI 10.1016/j.jhazmat.2019.121561"
"   Losada L, 2016, PLASMID, V83, P8, DOI 10.1016/j.plasmid.2015.12.001"
"   Luo Y, 2021, ECOTOX ENVIRON SAFE, V225, DOI 10.1016/j.ecoenv.2021.112749"
"   Ma JJ, 2021, CHEMOSPHERE, V269, DOI 10.1016/j.chemosphere.2020.128735"
"   Ma SY, 2022, ENVIRON RES, V214, DOI 10.1016/j.envres.2022.113822"
"   Martin M, 2014, GEODERMA, V228, P132, DOI 10.1016/j.geoderma.2013.09.020"
"   Ministry of Ecology and Environment of the People's Republic of China, 2018, SOIL ENV QUALITY RIS"
"   Ministry of Environmental Protection of the People's Republic of China, 2013, Soil and sediment Determination of mercury, arsenic, selenium, bismuth, antimony microwave dissolution/atomic fluorescence spectrometry (HJ608-2013)"
"   Ministry of Environmental Protection of the People's Republic of China, 2011, Soil-determination of organic carbon-potassium dichromate oxidation spectrophotometric method (HJ 615-2011)"
"   Ministry of Environmental Protection of the People's Republic of China, 2016, Soil and Sediment-Determination of Aqua Regia Extracts of 12 Metal Elements-Inductively Coupled Plasma Mass Spectrometry, HJ 803-2016"
"   Narendrula-Kotha R, 2020, REV ENVIRON CONTAM T, V249, P1, DOI 10.1007/398_2018_22"

"   Narendrula-Kotha R, 2017, Ecological Genetics and Genomics, V2, P13, DOI [10.1016/j.egg.2016.11.001, 10.1016/ j.egg.2016.11.001., DOI 10.1016/J.EGG.2016.11.001]"
"   Oka M, 2018, ENVIRON POLLUT, V243, P713, DOI 10.1016/j.envpol.2018.09.024"
"   Pagnanelli F, 2010, BIORESOURCE TECHNOL, V101, P2981, DOI 10.1016/j.biortech.2009.12.009"
"   Perrett M, 2021, ENVIRON POLLUT, V278, DOI 10.1016/j.envpol.2021.116815"
"   Qiao LK, 2021, SCI TOTAL ENVIRON, V779, DOI 10.1016/j.scitotenv.2021.146502"
"   Qin GW, 2021, CHEMOSPHERE, V267, DOI 10.1016/j.chemosphere.2020.129205"
"   Qiu XW, 2021, ECOTOX ENVIRON SAFE, V216, DOI 10.1016/j.ecoenv.2021.112190"
"   Simpson SL, 2000, ENVIRON SCI TECHNOL, V34, P4533, DOI 10.1021/es991440x"
"   Sinkko H, 2013, PLOS ONE, V8, DOI 10.1371/journal.pone.0067061"
"   Sobariu DL, 2017, NEW BIOTECHNOL, V39, P125, DOI 10.1016/j.nbt.2016.09.002"
"   Song HJ, 2019, ECOTOX ENVIRON SAFE, V184, DOI 10.1016/j.ecoenv.2019.109620"
"   Tang JY, 2019, J ENVIRON MANAGE, V242, P121, DOI 10.1016/j.jenvman.2019.04.061"
"   Tipayno SC, 2018, ECOL EVOL, V8, P6157, DOI 10.1002/ece3.4170"
"   Valenzuela EI, 2020, J ENVIRON MANAGE, V270, DOI 10.1016/j.jenvman.2020.110873"
"   Wang XJ, 2021, MICROORGANISMS, V9, DOI 10.3390/microorganisms9071417"
"   [????????? Wu Guangmei], 2020, [??????, Soils], V52, P920"
"   Xie P, 2015, J ENVIRON SCI, V27, P179, DOI 10.1016/j.jes.2014.07.017"
"   Xu R, 2022, ENVIRON MICROBIOL, V24, P803, DOI 10.1111/1462-2920.15612"
"   Xu YL, 2018, SCI TOTAL ENVIRON, V621, P148, DOI 10.1016/j.scitotenv.2017.11.214"
"   Yin HQ, 2015, SCI REP-UK, V5, DOI 10.1038/srep14266"
"   [????????? Yu Fangming], 2020, [??????????????????, China Environmental Science], V40, P2262"
"   Zeng FR, 2011, ENVIRON POLLUT, V159, P84, DOI 10.1016/j.envpol.2010.09.019"
"   Zeng GM, 2017, J HAZARD MATER, V339, P354, DOI 10.1016/j.jhazmat.2017.05.038"
"   [?????? Zeng Yuan], 2017, [????????????, Rock and Mineral Analysis], V36, P209"
"   Zhang BG, 2018, SOIL BIOL BIOCHEM, V118, P178, DOI 10.1016/j.soilbio.2017.12.011"
"   Zhang J, 2023, J ENVIRON CHEM ENG, V11, DOI 10.1016/j.jece.2023.109895"
"   Zhang LX, 2022, J HAZARD MATER, V424, DOI 10.1016/j.jhazmat.2021.127154"
"   Zhao QQ, 2020, ECOHYDROL HYDROBIOL, V20, P537, DOI 10.1016/j.ecohyd.2019.10.008"
"   Zhao Si-yu, 2025, Huanjing Kexue, V46, DOI 10.13227/j.hjkx.202401094"
"   [????????? Zhao Xiaoxue], 2017, [??????????????????, Environmental Monitoring in China], V33, P68"
"   Zhao XQ, 2020, ENVIRON SCI POLLUT R, V27, P20215, DOI 10.1007/s11356-020-08538-1"
"   Zhao XQ, 2019, ECOTOX ENVIRON SAFE, V170, P218, DOI 10.1016/j.ecoenv.2018.11.136"
"   Zhao ZG, 2021, WATER-SUI, V13, DOI 10.3390/w13212940"
"   Zhong X, 2020, J HAZARD MATER, V400, DOI 10.1016/j.jhazmat.2020.123289"
"   Zou Shuhua, 2019, Weishengwu Xuebao, V59, P2306, DOI 10.13343/j.cnki.wsxb.20190029"
NR 87
TC 4
Z9 4
U1 28
U2 33
PU SPRINGER
PI DORDRECHT
"PA VAN GODEWIJCKSTRAAT 30, 3311 GZ DORDRECHT, NETHERLANDS"
SN 0167-6369
EI 1573-2959
J9 ENVIRON MONIT ASSESS
JI Environ. Monit. Assess.
PD JUL 9
PY 2025
VL 197
IS 8
AR 883
DI 10.1007/s10661-025-14228-7
PG 17
WC Environmental Sciences
WE Science Citation Index Expanded (SCI-EXPANDED)
SC Environmental Sciences & Ecology
GA 4SE3O
UT WOS:001525398300004
PM 40632334
DA 2026-04-06
ER

PT J
"AU Liu, MB"
"   Wang, SL"
"   Yang, M"
"   Wu, YN"
"   Nan, ZR"
"AF Liu, Mengbo"
"   Wang, Shengli"
"   Yang, Meng"
"   Wu, Yining"
"   Nan, Zhongren"
TI Combined treatment of heavy metals in water and soil by biochar and
   manganese-oxidizing bacteria
SO JOURNAL OF SOILS AND SEDIMENTS
LA English
DT Article
DE Biological iron manganese oxide; Biochar; Compound pollution; Arsenic;
   Lead; Cadmium
ID TRACE-ELEMENTS; ADSORPTION; MECHANISMS; OXIDATION; DYNAMICS; CADMIUM;
   OXIDES; ALLEVIATION; TEMPERATURE; AMENDMENTS

"AB Purpose The combined pollution of soil heavy metals has always been a difficult problem for remediation. The combined pollution in soil also has great potential risks to crop safety and groundwater safety, and more research needs to be carried out on the remediation of soil heavy metal combined pollution. In addition, there is a large gap in the application of biological manganese oxide in soil. Materials and methods In this study, the treatment effects of As, Pb, and Cd in water and soil systems were studied by combining biochar with manganese-oxidizing bacteria repair agent. The orthogonal experiment was used to study the effect of different proportions of addition on the remediation effect of three heavy metals, and the contribution of each component added in the remediation was discussed by the control experiment. The effects of soil physicochemical properties including pH, EC, and DOM (dissolved organic matter) on the transformation and migration of heavy metals were also analyzed in detail. Results and discussion The combined remediation showed a high removal rate of Pb (97.71%) in water and increased the removal rate of Cd alone to 96.5%. Compared with the microbial remediation, the removal rate of As decreased (25.5%), which may be related to the adsorption of Fe2+ and Mn2+ by biochar. The combined application of biochar and manganese-oxidizing bacteria in soil could significantly improve the passivation of Pb, reduce the passivation rate of As, and increase the concentration of exchangeable Cd. Treatment of heavy metal combined pollution by combined remediation can achieve the best benefits, and the most suitable ratio is 2% (w/w) of biochar and 60% (v/w) of manganese oxide bacteria remediation agent. The migration of cadmium in soil induced by biochar and manganese-oxidizing bacteria may be due to the increase of DOM. Conclusions Compared with the single application, the combined remediation of biochar and manganese-oxidizing bacteria has better effect on composite pollution. Combined remediation is more suitable for soil contaminated by Pb and As, and it is difficult to efficiently solve the simultaneous Cd pollution. At the same time, combined remediation has greater development potential for Pb and Cd pollution in water."
"C1 [Liu, Mengbo; Wang, Shengli; Yang, Meng; Wu, Yining; Nan, Zhongren] Lanzhou Univ, Coll Earth & Environm Sci, 222 Tianshui South Rd, Lanzhou 730000, Peoples R China."
C3 Lanzhou University
"RP Wang, SL (corresponding author), Lanzhou Univ, Coll Earth & Environm Sci, 222 Tianshui South Rd, Lanzhou 730000, Peoples R China."
EM liumb2019@lzu.edu.cn; wangshengl@lzu.edu.cn; 2460681019@qq.com;
wuyn20@lzu.edu.cn; nanzhongren@lzu.edu.cn
FU National Key Research and Development Program of China [2018YFC1802905]
FX This work was supported by the National Key Research and Development
Program of China (2018YFC1802905).
"CR Abdelhafez AA, 2014, CHEMOSPHERE, V117, P66, DOI 10.1016/j.chemosphere.2014.05.086"
" Ahmad N, 2019, PAK J ANAL ENV CHEM, V20, P82, DOI 10.21743/pjaec/2019.06.11"
" Al-Dhabi NA, 2022, ENVIRON RES, V204, DOI 10.1016/j.envres.2021.112115"
" Ali S, 2017, ENVIRON SCI POLLUT R, V24, P12700, DOI 10.1007/s11356-017-8904-x"
" Andeer PF, 2015, ENVIRON MICROBIOL, V17, P3925, DOI 10.1111/1462-2920.12893"
" Asere TG, 2019, SCI TOTAL ENVIRON, V676, P706, DOI 10.1016/j.scitotenv.2019.04.237"
" Bai YH, 2016, WATER RES, V98, P119, DOI 10.1016/j.watres.2016.03.068"
" Baker H, 2004, ANAL CHIM ACTA, V516, P179, DOI 10.1016/j.aca.2004.03.068"
" Bao, 2020, APPLICABILITY BIOGEN"
" Barboza NR, 2015, BIOMED RES INT, V2015, DOI 10.1155/2015/925972"
" Beesley L, 2010, ENVIRON POLLUT, V158, P2282, DOI 10.1016/j.envpol.2010.02.003"
" Borggaard OK, 2019, GEODERMA, V343, P235, DOI 10.1016/j.geoderma.2019.02.041"
" Cheng J, 2021, J TRACE ELEM MED BIO, V67, DOI 10.1016/j.jtemb.2021.126778"
" Comert S, 2020, GEOMICROBIOL J, V37, P753, DOI 10.1080/01490451.2020.1770900"
" Cui Q, 2021, SCI TOTAL ENVIRON, V756, DOI 10.1016/j.scitotenv.2020.143801"
" Fan DW, 2021, SCI TOTAL ENVIRON, V783, DOI 10.1016/j.scitotenv.2021.147494"
" Farhangi-Abriz S, 2021, ECOTOX ENVIRON SAFE, V211, DOI 10.1016/j.ecoenv.2021.111904"
" Haider FU, 2021, ECOTOX ENVIRON SAFE, V214, DOI 10.1016/j.ecoenv.2021.112112"
" Hamid Y, 2019, CHEMOSPHERE, V214, P259, DOI 10.1016/j.chemosphere.2018.09.113"
" He BH, 2021, ECOTOX ENVIRON SAFE, V207, DOI 10.1016/j.ecoenv.2020.111234"
" He ZF, 2020, CHEMOSPHERE, V260, DOI 10.1016/j.chemosphere.2020.127621"
" He ZF, 2019, CHEMOSPHERE, V236, DOI [10.1016/j.chemosphere.2019.07.124353, 10.1016/j.chemosphere.2019.124353]"
" Hohmann C, 2011, GEOCHIM COSMOCHIM AC, V75, P4699, DOI 10.1016/j.gca.2011.02.044"
" Holguera JG, 2018, CHEM GEOL, V502, P76, DOI 10.1016/j.chemgeo.2018.10.020"
" Houben D, 2015, CHEMOSPHERE, V139, P644, DOI 10.1016/j.chemosphere.2014.12.036"
" Kumar A, 2022, B ENVIRON CONTAM TOX, V108, P415, DOI 10.1007/s00128-020-03095-2"
" Laird D, 2010, GEODERMA, V158, P436, DOI 10.1016/j.geoderma.2010.05.012"
" Lata S, 2019, ENVIRON SCI POLLUT R, V26, P32175, DOI 10.1007/s11356-019-06300-w"
" Li D, 2020, CHEMOSPHERE, V241, DOI 10.1016/j.chemosphere.2019.125039"
" Li HB, 2017, CHEMOSPHERE, V178, P466, DOI 10.1016/j.chemosphere.2017.03.072"
" Li YP, 2019, SCI TOTAL ENVIRON, V663, P307, DOI 10.1016/j.scitotenv.2019.01.257"
" Liu MB, 2022, ENVIRON SCI POLLUT R, V29, P5526, DOI 10.1007/s11356-021-15475-0"
" Lucchini P, 2014, AGR ECOSYST ENVIRON, V184, P149, DOI 10.1016/j.agee.2013.11.018"
" Madadi R, 2021, MAR POLLUT BULL, V166, DOI 10.1016/j.marpolbul.2021.112247"
" Meng YT, 2009, ENVIRON POLLUT, V157, P2577, DOI 10.1016/j.envpol.2009.02.035"
" Ming H, 2016, GEODERMA, V268, P60, DOI 10.1016/j.geoderma.2016.01.021"
" Mishrra A, 2021, ENVIRON SCI TECH LET, V8, P878, DOI 10.1021/acs.estlett.1c00580"
" Nelson YM, 2002, ENVIRON SCI TECHNOL, V36, P421, DOI 10.1021/es010907c"
" Novak JM, 2013, BIOENERG RES, V6, P114, DOI 10.1007/s12155-012-9228-9"
" Ofomaja AE, 2010, IND ENG CHEM RES, V49, P2562, DOI 10.1021/ie901150x"
" Qiu BB, 2021, J ANAL APPL PYROL, V155, DOI 10.1016/j.jaap.2021.105081"
" Qu CC, 2022, ENVIRON POLLUT, V295, DOI 10.1016/j.envpol.2021.118651"
" Rizwan M, 2016, ENVIRON SCI POLLUT R, V23, P2230, DOI 10.1007/s11356-015-5697-7"
" Samsuri A, 2013, J ENVIRON CHEM ENG, V1, P981, DOI 10.1016/j.jece.2013.08.009"
" Sharma MD, 2022, ENVIRON RES, V207, DOI 10.1016/j.envres.2021.112198"
" Suda A, 2016, GEODERMA, V270, P68, DOI 10.1016/j.geoderma.2015.12.017"
" Tani Y, 2004, ENVIRON SCI TECHNOL, V38, P6618, DOI 10.1021/es049226i"
" Uchimiya M, 2010, J AGR FOOD CHEM, V58, P5538, DOI 10.1021/jf9044217"
" Wang F, 2021, SCI TOTAL ENVIRON, V786, DOI 10.1016/j.scitotenv.2021.147595"
" Wang YN, 2020, ENVIRON POLLUT, V258, DOI 10.1016/j.envpol.2019.113481"
" Wang YY, 2020, SCI TOTAL ENVIRON, V729, DOI 10.1016/j.scitotenv.2020.139060"

"   Wright MH, 2016, APPL ENVIRON MICROB, V82, P5402, DOI 10.1128/AEM.00663-16"
"   Xiao AW, 2020, ECOTOX ENVIRON SAFE, V190, DOI 10.1016/j.ecoenv.2019.110136"
"   Yan DYS, 2012, J HAZARD MATER, V199, P51, DOI 10.1016/j.jhazmat.2011.10.054"
"   Yang HY, 2020, CHEM ENG J, V382, DOI 10.1016/j.cej.2019.123033"
"   Yin DX, 2017, CHEMOSPHERE, V186, P928, DOI 10.1016/j.chemosphere.2017.07.126"
"   Yu ZH, 2015, J ENVIRON MANAGE, V163, P155, DOI 10.1016/j.jenvman.2015.08.020"
"   Yuan JH, 2011, BIORESOURCE TECHNOL, V102, P3488, DOI 10.1016/j.biortech.2010.11.018"
"   Zhang HY, 2020, CHEM ENG J, V398, DOI 10.1016/j.cej.2020.125657"
"   Zhang JQ, 2021, AGR ECOSYST ENVIRON, V317, DOI 10.1016/j.agee.2021.107453"
"   Zhang Y, 2019, J HAZARD MATER, V367, P539, DOI 10.1016/j.jhazmat.2019.01.012"
"   Zhao X, 2018, CHEMOSPHERE, V205, P553, DOI 10.1016/j.chemosphere.2018.04.130"
NR 62
TC 8
Z9 9
U1 21
U2 224
PU SPRINGER HEIDELBERG
PI HEIDELBERG
"PA TIERGARTENSTRASSE 17, D-69121 HEIDELBERG, GERMANY"
SN 1439-0108
EI 1614-7480
J9 J SOIL SEDIMENT
JI J. Soils Sediments
PD JAN
PY 2023
VL 23
IS 1
BP 145
EP 155
DI 10.1007/s11368-022-03298-6
EA AUG 2022
PG 11
WC Environmental Sciences; Soil Science
WE Science Citation Index Expanded (SCI-EXPANDED)
SC Environmental Sciences & Ecology; Agriculture
GA 7V1ZA
UT WOS:000837957500001
DA 2026-04-06
ER

PT J
"AU Li, QR"
"   Zhang, WB"
"   Liao, ST"
"   Xing, DX"
"   Xiao, Y"
"   Zhou, DL"
"   Yang, Q"
"AF Li, Qingrong"
"   Zhang, Wenbo"
"   Liao, Sentai"
"   Xing, Dongxu"
"   Xiao, Yang"
"   Zhou, Donglai"
"   Yang, Qiong"
TI Mechanism of lead adsorption by a Bacillus cereus strain with
   indole-3-acetic acid secretion and inorganic phosphorus dissolution
   functions
SO BMC MICROBIOLOGY
LA English
DT Article
DE Plant growth promoting; Heavy metal; Lead; Microbial remediation
ID METAL-CONTAMINATED SOILS; PLANT-GROWTH PROMOTION; OXIDATIVE STRESS;
   AQUEOUS-SOLUTION; ARSENIC UPTAKE; HEAVY-METALS; BIOSORPTION; BACTERIA;
   PB(II); BIOMINERALIZATION
"AB BackgroundHeavy metal pollution has become a major source of environmental pollution because of increasing industrialization. Microbial remediation is a promising approach to remediate lead-contaminated environments owing to its cost-effective, environment-friendly, ecologically sustainable, and highly efficient properties. In this study, the growth-promoting functions and lead-adsorption ability of Bacillus cereus SEM-15 were examined, and the functional mechanism of the strain was preliminarily identified using scanning electron microscopy, energy spectrum, infrared spectrum, and genome analyses, providing theoretical support for utilization of B. cereus SEM-15 in heavy metals remediation.ResultsB. cereus SEM-15 showed strong ability to dissolve inorganic phosphorus and secrete indole-3-acetic acid. The lead adsorption efficiency of the strain at lead ion concentration of 150 mg/L was more than 93%. Single factor analysis revealed the optimal conditions for heavy metal adsorption by B. cereus SEM-15 (adsorption time, initial lead ion concentration, pH, and inoculum amount were 10 min, 50-150 mg/L, 6-7, and 5 g/L, respectively) in nutrient-free environment, with the lead adsorption rate reaching 96.58%. Scanning electron microscopy of B. cereus SEM-15 cells before and after lead adsorption showed adherence of a large number of granular precipitates to the cell surface after lead adsorption. X-Ray photoelectron spectroscopy and Fourier transform infrared spectroscopy results indicated the characteristic peaks of Pb-O, Pb-O-R (R = functional group), and Pb-S bonds after lead adsorption, and a shift in the characteristic peaks of bonds and groups related to C, N, and O. Genome annotation results showed the presence of genes related to heavy metals tolerance and plant growth promotion in B. cereus SEM-15, providing a molecular basis for the strain's heavy metals tolerance and plant growth promotion functions.ConclusionsThis study analyzed the lead adsorption characteristics of B. cereus

SEM-15 and the associated influencing factors, and discussed the adsorption mechanism and related functional genes, providing a basis for clarifying the underlying molecular mechanism and offering a reference for further research on plant-microorganisms combined remediation of heavy metals polluted environments."
"C1 [Li, Qingrong; Zhang, Wenbo; Liao, Sentai; Xing, Dongxu; Xiao, Yang; Zhou, Donglai; Yang, Qiong] Guangdong Acad Agr Sci, Sericultural & Agrifood Res Inst, Guangzhou 510610, Peoples R China."
"   [Li, Qingrong; Xing, Dongxu; Xiao, Yang; Yang, Qiong] Minist Agr & Rural Affairs, Key Lab Urban Agr South China, Guangzhou 510610, Peoples R China."
C3 Guangdong Academy of Agricultural Sciences
"RP Yang, Q (corresponding author), Guangdong Acad Agr Sci, Sericultural & Agrifood Res Inst, Guangzhou 510610, Peoples R China.; Yang, Q (corresponding author), Minist Agr & Rural Affairs, Key Lab Urban Agr South China, Guangzhou 510610, Peoples R China."
EM serilover@126.com
"OI Zhou, Donglai/0000-0003-1884-4749"
FU Science and Technology Plan Project of Guangxi Province [AB21196040];
   Project for Agricultural Advantage Industry Discipline Team Construction
   of GAAS [XT202221]; Low Carbon Agriculture and Carbon Neutralization
"   Research Center, GDAAS; [202119TD]"
FX This work was financially supported by Science and Technology Plan
"   Project of Guangxi Province (AB21196040), Low Carbon Agriculture and"
"   Carbon Neutralization Research Center, GDAAS (XT202221), and the Project"
   for Agricultural Advantage Industry Discipline Team Construction of GAAS
   (No. 202119TD).
"CR Abdollahi S, 2020, INT MICROBIOL, V23, P625, DOI 10.1007/s10123-020-00133-1"
"   Akar T, 2006, BIORESOURCE TECHNOL, V97, P1780, DOI 10.1016/j.biortech.2005.09.009"
"   Akhter S, 2012, NUCLEIC ACIDS RES, V40, DOI 10.1093/nar/gks406"
"   Anton A, 1999, J BACTERIOL, V181, P6876, DOI 10.1128/JB.181.22.6876-6881.1999"
"   Benson G, 1999, NUCLEIC ACIDS RES, V27, P573, DOI 10.1093/nar/27.2.573"
"   Bhat S, 2011, J ENVIRON SCI-CHINA, V23, P95, DOI 10.1016/S1001-0742(10)60378-6"
"   Bhojiya AA, 2022, B ENVIRON CONTAM TOX, V108, P468, DOI 10.1007/s00128-021-03232-5"
"   CARLIN A, 1995, J BACTERIOL, V177, P981, DOI 10.1128/jb.177.4.981-986.1995"
"   Chen HM, 2019, ENVIRON INT, V127, P395, DOI 10.1016/j.envint.2019.03.068"
"   Chen Z, 2016, J HAZARD MATER, V301, P531, DOI 10.1016/j.jhazmat.2015.09.023"
"   Cho DH, 2003, BIOPROC BIOSYST ENG, V25, P271, DOI 10.1007/s00449-002-0315-8"
"   Couvin D, 2018, NUCLEIC ACIDS RES, V46, pW246, DOI 10.1093/nar/gky425"
"   Dahmani MA, 2020, FRONT PLANT SCI, V11, DOI 10.3389/fpls.2020.00124"
"   De Bartolomeo A, 2004, CHEMOSPHERE, V55, P1323, DOI 10.1016/j.chemosphere.2003.12.005"
"   Defez R, 2019, BIOMOLECULES, V9, DOI 10.3390/biom9100522"
"   Delcher AL, 2007, BIOINFORMATICS, V23, P673, DOI 10.1093/bioinformatics/btm009"
"   Dietrich M, 2021, ENVIRON POLLUT, V288, DOI 10.1016/j.envpol.2021.117815"
"   Efe D, 2020, CURR MICROBIOL, V77, P3861, DOI 10.1007/s00284-020-02208-8"
"   El Aafi N, 2012, INT J PHYTOREMEDIAT, V14, P261, DOI 10.1080/15226514.2011.604693"
"   Fereidouni M, 2009, J HAZARD MATER, V168, P1437, DOI 10.1016/j.jhazmat.2009.03.041"
"   Garbinski LD, 2019, ENVIRON INT, V126, P585, DOI 10.1016/j.envint.2019.02.058"
"   Gardner PP, 2009, NUCLEIC ACIDS RES, V37, pD136, DOI 10.1093/nar/gkn766"
"   Gidlow DA, 2015, OCCUP MED-OXFORD, V65, P348, DOI 10.1093/occmed/kqv018"
"   Govarthanan M, 2013, CHEMOSPHERE, V90, P2267, DOI 10.1016/j.chemosphere.2012.10.038"
"   Grass G, 2000, J BACTERIOL, V182, P1390, DOI 10.1128/JB.182.5.1390-1398.2000"
"   Gupta P, 2019, J HAZARD MATER, V377, P391, DOI 10.1016/j.jhazmat.2019.05.054"
"   Hidangmayum A, 2023, INT J ENVIRON SCI TE, V20, P11693, DOI 10.1007/s13762-022-04560-7"
"   Huang Y, 2019, SCI TOTAL ENVIRON, V651, P3034, DOI 10.1016/j.scitotenv.2018.10.185"
"   Islam F, 2014, ECOTOX ENVIRON SAFE, V104, P285, DOI 10.1016/j.ecoenv.2014.03.008"
"   Kabir E, 2012, SCI WORLD J, DOI 10.1100/2012/916705"
"   Koistinen J, 2020, METHODS MOL BIOL, V1980, P87, DOI 10.1007/7651_2017_104"
"   Lagesen K, 2007, NUCLEIC ACIDS RES, V35, P3100, DOI 10.1093/nar/gkm160"
"   Landajo A, 2004, CHEMOSPHERE, V56, P1033, DOI 10.1016/j.chemosphere.2004.06.005"
"   Li QR, 2019, CAN J MICROBIOL, V65, P45, DOI 10.1139/cjm-2018-0350"
"   Liu LW, 2018, SCI TOTAL ENVIRON, V633, P206, DOI 10.1016/j.scitotenv.2018.03.161"
"   Lodeiro P, 2006, ENVIRON POLLUT, V142, P264, DOI 10.1016/j.envpol.2005.10.001"
"   Lowe TM, 1997, NUCLEIC ACIDS RES, V25, P955, DOI 10.1093/nar/25.5.955"
"   Ma Y, 2015, J TOXICOL ENV HEAL A, V78, P931, DOI 10.1080/15287394.2015.1051205"
"   Mathew Blessy Baby, 2018, Groundwater for Sustainable Development, V7, P313, DOI [10.1016/j.gsd.2018.07.007, 10.1016/j.gsd.2018.07.007]"
"   Mergeay M, 2003, FEMS MICROBIOL REV, V27, P385, DOI 10.1016/S0168-6445(03)00045-7"
"   Mesa-Mar??n J, 2020, FRONT MICROBIOL, V11, DOI 10.3389/fmicb.2020.553018"
"   Moreno-Ram??rez L, 2015, GENET MOL RES, V14, P2793, DOI 10.4238/2015.March.31.9"
"   Ortiz-Ojeda P, 2017, AIMS MICROBIOL, V3, P279, DOI 10.3934/microbiol.2017.2.279"
"   Pan XH, 2017, WORLD J MICROB BIOT, V33, DOI 10.1007/s11274-017-2211-z"
"   Pourrut B, 2011, REV ENVIRON CONTAM T, V213, P113, DOI 10.1007/978-1-4419-9860-6_4"
"   Rahman K, 2022, BIOMASS CONVERS BIOR, V12, P1703, DOI 10.1007/s13399-021-01354-8"
"   Sagar S, 2012, CHEMOSPHERE, V86, P847, DOI 10.1016/j.chemosphere.2011.11.031"
"   Sari A, 2009, J HAZARD MATER, V164, P1004, DOI 10.1016/j.jhazmat.2008.09.002"
"   Shan B, 2021, ENVIRON SCI POLLUT R, V28, P30486, DOI 10.1007/s11356-021-14045-8"
"   Sharma B, 2021, J HAZARD MATER, V401, DOI 10.1016/j.jhazmat.2020.123285"
"   Sharma P, 2021, ENVIRON TECHNOL INNO, V24, DOI 10.1016/j.eti.2021.101826"
"   Sheng MM, 2020, J MICROBIOL BIOTECHN, V30, P689, DOI 10.4014/jmb.1910.10066"
"   Shin MN, 2012, J HAZARD MATER, V199, P314, DOI 10.1016/j.jhazmat.2011.11.010"
"   Singh P, 2022, FRONT MICROBIOL, V13, DOI 10.3389/fmicb.2022.898979"
"   Sun YX, 2022, CURR MICROBIOL, V79, DOI 10.1007/s00284-022-02777-w"
"   Teng ZD, 2019, J ENVIRON MANAGE, V231, P189, DOI 10.1016/j.jenvman.2018.10.012"
"   Tirry N., 2018, Journal of Genetic Engineering and Biotechnology, V16, P613, DOI [10.1016/j.jgeb.2018.06.004, 10.1016/j.jgeb.2018.06.004]"
"   T??z??n I, 2005, J ENVIRON MANAGE, V77, P85, DOI 10.1016/j.jenvman.2005.01.028"
"   Upadhyay SK, 2023, CHEMOSPHERE, V313, DOI 10.1016/j.chemosphere.2022.137551"
"   Upadhyay SK, 2022, FRONT MICROBIOL, V13, DOI 10.3389/fmicb.2022.916488"

"   Upadhyay SK, 2021, CHEMOSPHERE, V267, DOI 10.1016/j.chemosphere.2020.129216"
"   Vishnupradeep R, 2022, ENVIRON TECHNOL INNO, V25, DOI 10.1016/j.eti.2021.102154"
"   Waday YA, 2022, HELIYON, V8, DOI 10.1016/j.heliyon.2022.e12224"
"   Wang JL, 2006, BIOTECHNOL ADV, V24, P427, DOI 10.1016/j.biotechadv.2006.03.001"
"   Wang TQ, 2018, ENVIRON SCI POLLUT R, V25, P3527, DOI 10.1007/s11356-017-0694-7"
"   [????????? Wang Yaqi], 2022, [????????????, Acta Pedologica Sinica], V59, P808"
"   Wenzel WW, 2009, PLANT SOIL, V321, P385, DOI 10.1007/s11104-008-9686-1"
"   Yang HC, 2012, CURR TOP MEMBR, V69, P325, DOI 10.1016/B978-0-12-394390-3.00012-4"
"   Yang Q, 2012, INT J PHYTOREMEDIAT, V14, P89, DOI 10.1080/15226510903567471"
"   Zhang J, 2018, CHEMOSPHERE, V206, P682, DOI 10.1016/j.chemosphere.2018.04.181"
"   [????????? Zhang Wenbo], 2022, [?????????????€????, Microbiology China], V49, P3617"
NR 71
TC 21
Z9 25
U1 6
U2 43
PU BMC
PI LONDON
"PA CAMPUS, 4 CRINAN ST, LONDON N1 9XW, the United Kingdom"
SN 1471-2180
J9 BMC MICROBIOL
JI BMC Microbiol.
PD MAR 4
PY 2023
VL 23
IS 1
AR 57
DI 10.1186/s12866-023-02795-z
PG 14
WC Microbiology
WE Science Citation Index Expanded (SCI-EXPANDED)
SC Microbiology
GA 9N9FH
UT WOS:000943213400001
PM 36869296
"OA Green Submitted, gold"
DA 2026-04-06
ER

PT J
"AU Guo, SY"
"   Xiao, CQ"
"   Zhou, N"
"   Chi, R"
"AF Guo, Shuyu"
"   Xiao, Chunqiao"
"   Zhou, Na"
"   Chi, Ruan"
"TI Speciation, toxicity, microbial remediation and phytoremediation of soil"
   chromium contamination
SO ENVIRONMENTAL CHEMISTRY LETTERS
LA English
DT Review
DE Chromium contamination; Bioremediation; Phytoremediation; Microbial
   remediation; Biotransformation; Biosorption
ID HEXAVALENT CHROMIUM; PLANT-GROWTH; WASTE-WATER; PSEUDOMONAS-PUTIDA;
   HEAVY-METALS; MOLECULAR-MECHANISMS; PHENOLIC-COMPOUNDS; CR(VI)
   REDUCTION; BACTERIAL STRAIN; TANNERY EFFLUENT
"AB Contamination of soil by toxic chromium (Cr) is a rising health issue due to over-exploitation and industrial production. Toxicity can be decreased by bioremediation because some microorganisms are able to convert highly toxic hexavalent chromium Cr(VI) into less toxic trivalent chromium Cr(III) by secreting chromate reductase. Moreover, microorganisms are able to remove Cr by adsorption on microbial cell walls. Plants can also be used for phytoremediation by uptaking Cr from soil into plant organs. Here, we review the speciation of Cr in soil, remediation methods to remove Cr, bioremediation challenges, and remaining ecological impacts after bioremediation. We present the mechanisms of microbial remediation, phytoremediation and plant-microbial combined remediation and applications."
"C1 [Guo, Shuyu; Xiao, Chunqiao; Zhou, Na; Chi, Ruan] Wuhan Inst Technol, Sch Environm Ecol & Biol Engn, Minist Educ, Key Lab Green Chem Proc, Wuhan 430205, Hubei, Peoples R China."
C3 Wuhan Institute of Technology
"RP Xiao, CQ (corresponding author), Wuhan Inst Technol, Sch Environm Ecol & Biol Engn, Minist Educ, Key Lab Green Chem Proc, Wuhan 430205, Hubei, Peoples R China."
EM chunqiao@wit.edu.cn
"RI guo, shuyu/LBH-1222-2024"
"OI Xiao, Chunqiao/0000-0002-9942-3235"
FU National Natural Science Foundation of China [51674178]; Frontier
   Projects of Applied Foundation of Wuhan Science and Technology Bureau
   [2019020701011498]
FX This work was funded by the National Natural Science Foundation of China
   (51674178) and Frontier Projects of Applied Foundation of Wuhan Science
   and Technology Bureau (2019020701011498).
"CR Abou-Shanab RA, 2008, WORLD J MICROB BIOT, V24, P253, DOI 10.1007/s11274-007-9464-x"
"   Ackerley DF, 2004, APPL ENVIRON MICROB, V70, P873, DOI 10.1128/AEM.70.2.873-882.2004"
"   Agarwal R, 2006, ACTA CRYSTALLOGR D, V62, P383, DOI [10.1107/S0907444906001600,
10.1107/S0904777906001600]"

"   Ahemad Munees, 2015, Journal of Genetic Engineering and Biotechnology, V13, P51, DOI [10.1016/j.jgeb.2015.02.001, 10.1016/j.jgeb.2015.02.001]"
"   Ahmad WA, 2013, INT BIODETER BIODEGR, V85, P571, DOI 10.1016/j.ibiod.2013.01.008"
"   Alahmad W, 2019, ANAL CHIM ACTA, V1085, P98, DOI 10.1016/j.aca.2019.08.002"
"   Ali I, 2017, J MOL LIQ, V236, P205, DOI 10.1016/j.molliq.2017.04.028"
"   Amatussalam A, 2011, INDIAN J EXP BIOL, V49, P925"
"   Andreozzi R, 2020, J ENVIRON MANAGE, V268, DOI 10.1016/j.jenvman.2020.110584"
"   Anju M., 2017, Plant Biotechnology: Recent Advancements and Developments, P315"
"   Avdalovic J, 2015, MINER ENG, V72, P17, DOI 10.1016/j.mineng.2014.12.010"
"   Bali AS, 2020, ENVIRON CHEM LETT, V18, P1243, DOI 10.1007/s10311-020-01012-x"
"   Bolan NS, 2013, REV ENVIRON CONTAM T, V225, P1, DOI 10.1007/978-1-4614-6470-9_1"
"   Brown S, 2005, ENVIRON POLLUT, V138, P34, DOI 10.1016/j.envpol.2005.02.020"
"   Browning CL, 2017, TOXICOL APPL PHARM, V331, P101, DOI 10.1016/j.taap.2017.05.030"
"   CARLOS FS, 2016, WATER AIR SOIL POLL, V227"
"   Chai LY, 2009, J HAZARD MATER, V167, P516, DOI 10.1016/j.jhazmat.2009.01.030"
"   Chen HX, 2018, J SOIL SEDIMENT, V18, P897, DOI 10.1007/s11368-017-1811-4"
"   Chen S, 2019, ECOTOX ENVIRON SAFE, V169, P134, DOI 10.1016/j.ecoenv.2018.11.004"
"   Cheng YJ, 2009, J ENVIRON SCI, V21, P1673, DOI 10.1016/S1001-0742(08)62472-9"
"   Cheung KH, 2007, INT BIODETER BIODEGR, V59, P8, DOI 10.1016/j.ibiod.2006.05.002"
"   Chhikara S, 2010, J ENVIRON BIOL, V31, P561"
"   Chitraprabha K, 2018, SUSTAIN ENVIRON RES, V28, P128, DOI 10.1016/j.serj.2018.01.002"
"   CLARKSON DT, 1980, ANNU REV PLANT PHYS, V31, P239, DOI 10.1146/annurev.pp.31.060180.001323"
"   Coutinho FP, 2012, ECOL ENG, V42, P85, DOI 10.1016/j.ecoleng.2012.02.002"
"   Das PK, 2021, ENVIRON CHEM LETT, V19, P1369, DOI 10.1007/s10311-020-01102-w"
"   Das S, 2014, CHEMOSPHERE, V96, P112, DOI 10.1016/j.chemosphere.2013.08.080"
"   de Oliveira LM, 2014, ENVIRON POLLUT, V184, P187, DOI 10.1016/j.envpol.2013.08.025"
"   Deghles A, 2016, DESALIN WATER TREAT, V57, P14798, DOI 10.1080/19443994.2015.1074622"
"   Deng P, 2015, EXP THER MED, V9, P795, DOI 10.3892/etm.2014.2148"
"   Dhal B, 2013, J HAZARD MATER, V250, P272, DOI 10.1016/j.jhazmat.2013.01.048"
"   Dias-Ferreira C, 2015, CHEMOSPHERE, V119, P889, DOI 10.1016/j.chemosphere.2014.08.064"
"   Dong J, 2020, APPL ENERG, V267, DOI 10.1016/j.apenergy.2020.114792"
"   Feng YX, 2019, J AGR FOOD CHEM, V67, P9738, DOI 10.1021/acs.jafc.9b01720"
"   Fu JW, 2017, J HAZARD MATER, V330, P68, DOI 10.1016/j.jhazmat.2017.01.049"
"   Garc??a-Gonzalo P, 2017, ENVIRON SCI POLLUT R, V24, P25713, DOI 10.1007/s11356-016-6667-4"
"   Gardea-Torresdey JL, 2004, BIORESOURCE TECHNOL, V92, P229, DOI 10.1016/j.biortech.2003.10.002"
"   Garg N, 2012, PLANT GROWTH REGUL, V66, P9, DOI 10.1007/s10725-011-9624-8"
"   Gunasundari D, 2013, ENVIRON SCI POLLUT R, V20, P6563, DOI 10.1007/s11356-013-1718-6"
"   He CW, 2020, ENVIRON CHEM LETT, V18, P561, DOI 10.1007/s10311-019-00960-3"
"   He JS, 2014, BIORESOURCE TECHNOL, V160, P67, DOI 10.1016/j.biortech.2014.01.068"
"   Helbig K, 2008, J BACTERIOL, V190, P5431, DOI 10.1128/JB.00271-08"
"   Hughes JM, 2002, REV MINERAL GEOCHEM, V48, P1, DOI 10.2138/rmg.2002.48.1"
"   Hussain S, 2020, INT J AGRIC BIOL, V23, P241, DOI 10.17957/IJAB/15.1282"
"   Igwe JC, 2006, AFR J BIOTECHNOL, V5, P1167"
"   Jalili B, 2020, J HAZARD MATER, V393, DOI 10.1016/j.jhazmat.2020.122375"
"   Jasso-Chavez R, 2019, FRONT MICROBIOL, V10, DOI 10.3389/fmicb.2019.02432"
"   Jobby R, 2018, CHEMOSPHERE, V207, P255, DOI 10.1016/j.chemosphere.2018.05.050"
"   Joseph SD, 2010, AUST J SOIL RES, V48, P501, DOI 10.1071/SR10009"
"   Joutey NT, 2015, REV ENVIRON CONTAM T, V233, P45, DOI 10.1007/978-3-319-10479-9_2"
"   Kamaludeen SPB, 2003, REV ENVIRON CONTAM T, V178, P93, DOI 10.1007/0-387-21728-2_4"
"   Karthik C, 2017, J TAIWAN INST CHEM E, V70, P282, DOI 10.1016/j.jtice.2016.11.006"
"   Kathiravan MN, 2011, CHEM ENG J, V169, P107, DOI 10.1016/j.cej.2011.02.060"
"   KATO FH, 2018, PLANT PHYSL BIOCH, V77"
"   Khan Mohammad Saghir, 2010, Archives of Agronomy and Soil Science, V56, P73, DOI 10.1080/03650340902806469"
"   Khan MS, 2009, ENVIRON CHEM LETT, V7, P1, DOI 10.1007/s10311-008-0155-0"
"   Kili?? NK, 2008, J HAZARD MATER, V154, P1019, DOI 10.1016/j.jhazmat.2007.11.008"
"   KLC NK, 2010, BIORESOURCE TECHNOL, V101, P2134"
"   Kleiman ID, 1997, ENVIRON POLLUT, V97, P131, DOI 10.1016/S0269-7491(97)00063-8"
"   Komives T., 2006, Phytoremediation Rhiyoremediation, P23, DOI [DOI 10.1007/978-1-4020-4999-4_3, 10.1007/978-1- 4020-4999-4_3.]"
"   Ksheminska H, 2005, PROCESS BIOCHEM, V40, P1565, DOI 10.1016/j.procbio.2004.05.012"
"   Kumar P, 2008, J HAZARD MATER, V153, P635, DOI 10.1016/j.jhazmat.2007.09.007"
"   Lacalle RG, 2020, ENVIRON SCI POLLUT R, V27, P31726, DOI 10.1007/s11356-020-09449-x"
"   Lee CC, 2016, SOIL SEDIMENT CONTAM, V25, P505, DOI 10.1080/15320383.2016.1169500"
"   Li B, 2019, WATER AIR SOIL POLL, V230, DOI 10.1007/s11270-019-4353-0"
"   Li H., 2016, FRONT PLANT SCI, V7"
"   Li HC, 2014, ENERG POLICY, V71, P180, DOI 10.1016/j.enpol.2014.04.018"
"   Li JG, 2020, J CLEAN PROD, V252, DOI 10.1016/j.jclepro.2019.119868"
"   Li J, 2019, J CLEAN PROD, V233, P1182, DOI 10.1016/j.jclepro.2019.06.061"
"   Li Z, 2016, J HAZARD MATER, V320, P386, DOI 10.1016/j.jhazmat.2016.08.051"
"   Liu HL, 2015, GEOMICROBIOL J, V32, P616, DOI 10.1080/01490451.2014.971200"
"   Liu X, 2015, INT J MOL SCI, V16, P11892, DOI 10.3390/ijms160611892"
"   L??pez-Arredondo DL, 2014, ANNU REV PLANT BIOL, V65, P95, DOI 10.1146/annurev-arplant-050213-035949"
"   Ma LL, 2020, CHEMOSPHERE, V240, DOI 10.1016/j.chemosphere.2019.124896"
"   Onta??on OM, 2014, INT BIODETER BIODEGR, V88, P192, DOI 10.1016/j.ibiod.2013.10.017"
"   Mandina S., 2013, Acad. J, V7, P310"
"   Manqui??n-Cerda K, 2018, ECOTOX ENVIRON SAFE, V150, P320, DOI 10.1016/j.ecoenv.2017.12.050"
"   McGowen SL, 2001, J ENVIRON QUAL, V30, P493, DOI 10.2134/jeq2001.302493x"
"   Mehes-Smith M, 2015, WATER AIR SOIL POLL, V226, DOI 10.1007/s11270-015-2382-x"
"   Mellem J. J., 2012, African Journal of Agricultural Research, V7, P591"
"   Mereuta I, 2020, POL J ENVIRON STUD, V29, P197, DOI 10.15244/pjoes/94810"
"   Michalak A, 2006, POL J ENVIRON STUD, V15, P523"
"   MIKALSEN A, 1991, CARCINOGENESIS, V12, P825, DOI 10.1093/carcin/12.5.825"
"   Muthusaravanan S, 2018, ENVIRON CHEM LETT, V16, P1339, DOI 10.1007/s10311-018-0762-3"

"   Narendrula-Kotha R, 2020, REV ENVIRON CONTAM T, V249, P1, DOI 10.1007/398_2018_22"
"   Nasiri A, 2020, ENVIRON POLLUT, V266, DOI 10.1016/j.envpol.2020.115197"
"   Nayak S, 2020, INT J PHYTOREMEDIAT, V22, P804, DOI 10.1080/15226514.2020.1717432"
"   Neha Gupta Neha Gupta, 2012, Archives of Applied Science Research, V4, P577"
"   Niu XY, 2019, FORESTS, V10, DOI 10.3390/f10020077"
"   Oyetibo GO, 2013, ENVIRON MONIT ASSESS, V185, P6809, DOI 10.1007/s10661-013-3066-x"
"   Pant BD, 2015, PLANT CELL ENVIRON, V38, P172, DOI 10.1111/pce.12378"
"   Park CH, 2000, APPL ENVIRON MICROB, V66, P1788, DOI 10.1128/AEM.66.5.1788-1795.2000"
"   Patra DK, 2020, ENVIRON TECHNOL INNO, V18, DOI 10.1016/j.eti.2020.100672"
"   Pattnaik S, 2020, CHEMOSPHERE, V240, DOI 10.1016/j.chemosphere.2019.124895"
"   PENG H, 2020, ENVIRON CHEM LE 0723"
"   Peuelas J., 2013, NATURE COMMUN, V4, P2934, DOI [10.1038/ncomms3934, DOI 10.1038/NCOMMS3934]"
"   Prasad MNV, 2010, ENVIRON POLLUT, V158, P18, DOI 10.1016/j.envpol.2009.07.038"
"   Rahman Z, 2020, ENVIRON SCI POLLUT R, V27, P27563, DOI 10.1007/s11356-020-08903-0"
"   Raicevic S, 2005, J HAZARD MATER, V117, P41, DOI 10.1016/j.jhazmat.2004.07.024"
"   Ram??rez V, 2019, FRONT MICROBIOL, V10, DOI 10.3389/fmicb.2019.01833"
"   Redondo-G??mez S, 2011, J HAZARD MATER, V185, P862, DOI 10.1016/j.jhazmat.2010.09.101"
"   Ren LX, 2014, CHEM ENG J, V247, P111, DOI 10.1016/j.cej.2014.02.107"
"   Robins KJ, 2013, PLOS ONE, V8, DOI 10.1371/journal.pone.0059200"
"   Rosling A, 2007, GEOBIOLOGY, V5, P265, DOI 10.1111/j.1472-4669.2007.00107.x"
"   S??nchez-Calder??n L, 2010, PLANT CELL MONOGR, V17, P173, DOI 10.1007/978-3-642-10613-2_8"
"   Shaheen SM, 2014, GEODERMA, V228, P142, DOI 10.1016/j.geoderma.2013.10.012"
"   Shahid M, 2017, CHEMOSPHERE, V178, P513, DOI 10.1016/j.chemosphere.2017.03.074"
"   Shahid M, 2015, ANN MICROBIOL, V65, P1525, DOI 10.1007/s13213-014-0991-z"
"   Sharan A, 2008, WORLD J MICROB BIOT, V24, P753, DOI 10.1007/s11274-007-9535-z"
"   Sharma S, 2016, FRONT MICROBIOL, V7, DOI 10.3389/fmicb.2016.01600"
"   Singh DK, 2019, MICROBIOLOGYOPEN, V8, DOI 10.1002/mbo3.838"
"   Singh HP, 2013, ENVIRON CHEM LETT, V11, P229, DOI 10.1007/s10311-013-0407-5"
"   Sivakumar D, 2016, GLOB J ENVIRON SCI M, V2, P105, DOI 10.7508/gjesm.2016.02.002"
"   Smith L.M., 1994, Engineering with Clad Steel, P1"
"   Sun FL, 2019, J HAZARD MATER, V368, P523, DOI 10.1016/j.jhazmat.2019.01.076"
"   Sytar O, 2013, ACTA PHYSIOL PLANT, V35, P985, DOI 10.1007/s11738-012-1169-6"
"   Takahashi CK, 2012, MAR POLLUT BULL, V64, P133, DOI 10.1016/j.marpolbul.2011.10.010"
"   Tang RG, 2019, ENVIRON POLLUT, V251, P910, DOI 10.1016/j.envpol.2019.05.069"
"   Tang RG, 2018, ENVIRON SCI POLLUT R, V25, P23117, DOI 10.1007/s11356-018-2436-x"
"   Tariq M, 2019, PEERJ, V7, DOI 10.7717/peerj.7726"
"   Thawornchaisit U, 2009, J HAZARD MATER, V165, P1109, DOI 10.1016/j.jhazmat.2008.10.103"
"   Trivedi P, 2008, CURR MICROBIOL, V56, P140, DOI 10.1007/s00284-007-9058-8"
"   Vallero DA, 2020, J ENVIRON ENG, V146, DOI 10.1061/(ASCE)EE.1943-7870.0001676"
"   van der Ent A, 2013, PLANT SOIL, V362, P319, DOI 10.1007/s11104-012-1287-3"
"   Vankar PS, 2008, DESALINATION, V222, P255, DOI 10.1016/j.desal.2007.01.168"
"   Velez PA, 2017, ENVIRON TECHNOL, V38, P2164, DOI 10.1080/09593330.2016.1247198"
"   Vendruscolo F, 2017, INT BIODETER BIODEGR, V119, P87, DOI 10.1016/j.ibiod.2016.10.008"
"   Viti C, 2014, FEMS MICROBIOL REV, V38, P633, DOI 10.1111/1574-6976.12051"
"   Wang L, 2004, ENZYME MICROB TECH, V34, P474, DOI 10.1016/j.enzmictec.2003.12.006"
"   Wani PA, 2019, B ENVIRON CONTAM TOX, V103, P476, DOI 10.1007/s00128-019-02683-1"
"   Wani PA, 2018, J ENVIRON CHEM ENG, V6, P3798, DOI 10.1016/j.jece.2018.05.042"
"   Wen ZH, 2017, PLANT SOIL, V416, P377, DOI 10.1007/s11104-017-3214-0"
"   Winkel-Shirley B, 2002, CURR OPIN PLANT BIOL, V5, P218, DOI 10.1016/S1369-5266(02)00256-X"
"   Wu JH, 2021, CHEMOSPHERE, V262, DOI 10.1016/j.chemosphere.2020.127733"
"   Xia SP, 2019, CRIT REV ENV SCI TEC, V49, P1027, DOI 10.1080/10643389.2018.1564526"
"   Xiao WD, 2015, ECOTOX ENVIRON SAFE, V113, P439, DOI 10.1016/j.ecoenv.2014.12.030"
"   Yang P, 2019, ENVIRON EXP BOT, V167, DOI 10.1016/j.envexpbot.2019.103827"
"   Yang X, 2019, APPL SCI BASEL, V9"
"   Yaseen M, 2009, J PLANT NUTR, V32, P410, DOI 10.1080/01904160802660735"
"   Zeb A, 2020, SCI TOTAL ENVIRON, V740, DOI 10.1016/j.scitotenv.2020.140145"
"   Zeidan AA, 2017, FEMS MICROBIOL REV, V41, pS168, DOI 10.1093/femsre/fux017"
"   Zeng FR, 2011, ENVIRON POLLUT, V159, P84, DOI 10.1016/j.envpol.2010.09.019"
"   Zhang MX, 2019, INT J ENV RES PUB HE, V16, DOI 10.3390/ijerph16122174"
"   Zhang ZL, 2014, J INTEGR PLANT BIOL, V56, P192, DOI 10.1111/jipb.12163"
"   Zhou AF, 2012, APPL ENVIRON MICROB, V78, P1168, DOI 10.1128/AEM.05666-11"
"   Zupancic M, 2012, J ENVIRON MONITOR, V14, P704, DOI 10.1039/c2em10798h"
NR 146
TC 124
Z9 140
U1 32
U2 649
PU SPRINGER BASEL AG
PI BASEL
"PA PICASSOPLATZ 4, BASEL, 4052, SWITZERLAND"
SN 1610-3653
EI 1610-3661
J9 ENVIRON CHEM LETT
JI Environ. Chem. Lett.
PD APR
PY 2021
VL 19
IS 2
BP 1413
EP 1431
DI 10.1007/s10311-020-01114-6
EA OCT 2020
PG 19
"WC Chemistry, Multidisciplinary; Engineering, Environmental; Environmental"

Sciences
WE Science Citation Index Expanded (SCI-EXPANDED)
SC Chemistry; Engineering; Environmental Sciences & Ecology
GA RO1IS
UT WOS:000583444000001
DA 2026-04-06
ER

PT J
"AU Yang, ZX"
"   Wang, TT"
"   Wang, HJ"
"   Wang, HB"
"AF Yang, Zhenxi"
"   Wang, Tingting"
"   Wang, Haijuan"
"   Wang, Hongbin"
TI Intercropping remediation of heavy metal polluted soil: Recent advances
   and future perspectives
SO APPLIED SOIL ECOLOGY
LA English
DT Review
DE Intercropping; Phytoremediation; Rhizospheric interaction; Heavy metals
ID PTERIS-VITTATA; RHIZOSPHERE MICROBIOME; OXALATE SECRETION; ROOT APEX;
   CADMIUM; PHYTOREMEDIATION; PHYTOEXTRACTION; FACILITATION; CD
"AB Phytoremediation, as an alternative to physical and chemical methods, offers a more cost-effective and ecofriendly approach for in situ remediation of heavy metal polluted soil. Despite many advantages, traditional phytoremediation often disrupts agricultural production and decreases economic benefits. Therefore, intercropping remediation integrating heavy metal hyperaccumulators with crops has been developed to achieve ""remediation while production"". Up to now, substantial researches have focused on elucidating the mechanisms of intercropping remediation and enhancing its efficiency, primarily through the identification of optimal plant species combination. However, it is essential to consider the impact of intercropping distance on remediation efficiency, and how rhizospheric interactions influence the effectiveness of intercropping remediation. This review systematically synthesizes recent advances in intercropping remediation of heavy metal contaminated soils, with an emphasis on the underlying mechanisms of intercropping remediation of heavy metal polluted soil, the impacts of intercropping remediation on soil quality, and the factors influencing the effectiveness of intercropping remediation. In the future, in-depth researches on combined root exudates-microbial intercropping remediation are needed to systematically understand the molecular composition of rhizospheric metabolites and their functional mechanisms in mediating plant-soil-microbe interactions in the rhizosphere of both crops and heavy metal hyperaccumulators. Furthermore, intercropping distance should be prioritized as a critical factor and in-depth investigations are necessary to probe into the related mechanisms governing interspecific relationship and intercropping remediation. Our review will provide an insight into a theoretical basis and practical application of intercropping remediation technology during phytoremediation of heavy metal polluted soil for simultaneously ensuring food safety and soil remediation."
"C1 [Yang, Zhenxi; Wang, Tingting; Wang, Haijuan; Wang, Hongbin] Kunming Univ Sci & Technol, Fac Environm Sci & Engn, Yunnan Key Lab Soil Carbon Sequestrat & Pollut Con, Kunming 650500, Peoples R China."
C3 Kunming University of Science & Technology
"RP Wang, HB (corresponding author), Kunming Univ Sci & Technol, Fac Environm Sci & Engn, Yunnan Key Lab Soil Carbon Sequestrat & Pollut Con, Kunming 650500, Peoples R China."
EM yangzx@stu.kust.edu.cn; 20232107007@stu.kust.edu.cn; whj79@kust.edu.cn;
   whb1974@kust.edu.cn
FU National Natural Science Foundation of China [31960264]
FX This work was financially supported by the National Natural Science
   Foundation of China (31960264).
"CR Alam O, 2024, J ENVIRON CHEM ENG, V12, DOI 10.1016/j.jece.2024.113149"
"   Alam O, 2020, J ENVIRON HEALTH SCI, V18, P1401, DOI 10.1007/s40201-020-00556-y"
"   Bakker PAHM, 2018, CELL, V172, P1178, DOI 10.1016/j.cell.2018.02.024"
"   Bali AS, 2020, ENVIRON CHEM LETT, V18, P1243, DOI 10.1007/s10311-020-01012-x"
"   Bayala J, 2020, PLANT SOIL, V453, P17, DOI 10.1007/s11104-019-04173-z"
"   Berendsen RL, 2012, TRENDS PLANT SCI, V17, P478, DOI 10.1016/j.tplants.2012.04.001"
"   Bian FY, 2021, J HAZARD MATER, V416, DOI 10.1016/j.jhazmat.2021.125898"
"   Bian FY, 2018, INT J PHYTOREMEDIAT, V20, P490, DOI 10.1080/15226514.2017.1374339"
"   Brooker RW, 2021, J ECOL, V109, P2054, DOI 10.1111/1365-2745.13592"
"   Cao XR, 2024, ENVIRON TECHNOL INNO, V35, DOI 10.1016/j.eti.2024.103709"
"   Cao XR, 2020, SCI TOTAL ENVIRON, V723, DOI 10.1016/j.scitotenv.2020.138152"
"   Chen WZ, 2023, ENVIRON SCI POLLUT R, V30, P53037, DOI 10.1007/s11356-023-26076-4"
"   Cheng H, 2022, CHEMOSPHERE, V306, DOI 10.1016/j.chemosphere.2022.135550"
"   Chi GY, 2024, J HAZARD MATER, V461, DOI 10.1016/j.jhazmat.2023.132727"
"   Cong WF, 2020, TRENDS PLANT SCI, V25, P967, DOI 10.1016/j.tplants.2020.04.013"
"   Cui T, 2018, J CHEM-NY, V2018, DOI 10.1155/2018/7803408"
"   Deng SX, 2024, BIOTECHNOL ADV, V72, DOI 10.1016/j.biotechadv.2024.108337"
"   Ding CH, 2022, ECOTOX ENVIRON SAFE, V232, DOI 10.1016/j.ecoenv.2022.113273"
"   Ding Y, 2021, J SOIL SEDIMENT, V21, P364, DOI 10.1007/s11368-020-02733-w"
"   Dong JL, 2021, SCI TOTAL ENVIRON, V754, DOI 10.1016/j.scitotenv.2020.142434"
"   Fahy A, 2015, MICROB ECOL, V70, P154, DOI 10.1007/s00248-014-0558-3"
"   Feng HC, 2018, MOL PLANT MICROBE IN, V31, P995, DOI [10.1094/mpmi-01-18-0003-r, 10.1094/MPMI-01-18-0003-R]"
"   Feng ZW, 2024, ENVIRON MICROBIOME, V19, DOI 10.1186/s40793-024-00638-6"
"   Glick BR, 2012, SCIENTIFICA, V2012, DOI 10.6064/2012/963401"
"   Han R, 2023, ENVIRON SCI TECHNOL, V57, P190, DOI 10.1021/acs.est.2c05659"
"   Hassan S, 2012, J EXP BOT, V63, P3429, DOI 10.1093/jxb/err430"
"   He MF, 2022, FRONT ECOL EVOL, V10, DOI 10.3389/fevo.2022.1036765"
"   Huang R, 2024, SCI TOTAL ENVIRON, V914, DOI 10.1016/j.scitotenv.2024.169900"

"   Huo CF, 2017, SOIL BIOL BIOCHEM, V111, P78, DOI 10.1016/j.soilbio.2017.04.003"
"   Jiang P, 2024, NEW PHYTOL, V243, P1506, DOI 10.1111/nph.19906"
"   Kang ZM, 2021, SCI TOTAL ENVIRON, V800, DOI 10.1016/j.scitotenv.2021.149600"
"   Kang ZM, 2020, ECOTOX ENVIRON SAFE, V190, DOI 10.1016/j.ecoenv.2019.110102"
"   Lei LL, 2021, J ENVIRON MANAGE, V298, DOI 10.1016/j.jenvman.2021.113516"
"   Li B, 2016, P NATL ACAD SCI USA, V113, P6496, DOI 10.1073/pnas.1523580113"
"   Li Junchun, 2023, Chemosphere, V341, P139975, DOI [10.1016/j.chemosphere.2023.139975, 10.1016/j.chemosphere.2023.139975]"
"   Li Q, 2021, B ENVIRON CONTAM TOX, V107, P1059, DOI 10.1007/s00128-021-03361-x"
"   Li XF, 2021, NAT SUSTAIN, V4, P943, DOI 10.1038/s41893-021-00767-7"
"   Li YF, 2023, SCI TOTAL ENVIRON, V879, DOI 10.1016/j.scitotenv.2023.163034"
"   Ling N, 2022, NAT COMMUN, V13, DOI 10.1038/s41467-022-28448-9"
"   Liu X, 2018, ENVIRON POLLUT, V241, P240, DOI 10.1016/j.envpol.2018.05.054"
"   Liu YR, 2023, SCI TOTAL ENVIRON, V875, DOI 10.1016/j.scitotenv.2023.162700"
"   Ma LY, 2024, ECOTOX ENVIRON SAFE, V282, DOI 10.1016/j.ecoenv.2024.116715"
"   Ma LY, 2022, J ENVIRON SCI, V115, P383, DOI 10.1016/j.jes.2021.08.005"
"   Ma LY, 2020, ENVIRON POLLUT, V264, DOI 10.1016/j.envpol.2020.114796"
"   Ma WM, 2022, FRONT MICROBIOL, V13, DOI 10.3389/fmicb.2022.937940"
"   Macias-Benitez S, 2020, FRONT PLANT SCI, V11, DOI 10.3389/fpls.2020.00633"
"   Manoj SR, 2020, J ENVIRON MANAGE, V254, DOI 10.1016/j.jenvman.2019.109779"
"   Ng CWW, 2023, ENVIRON POLLUT, V318, DOI 10.1016/j.envpol.2022.120930"
"   Omae N, 2022, MOL PLANT MICROBE IN, V35, P511, DOI 10.1094/MPMI-11-21-0281-FI"
"   Peiffer JA, 2013, P NATL ACAD SCI USA, V110, P6548, DOI 10.1073/pnas.1302837110"
"   Philippot L, 2024, NAT REV MICROBIOL, V22, P226, DOI 10.1038/s41579-023-00980-5"
"   Picone R, 2025, PLANT SOIL, DOI 10.1007/s11104-025-07723-w"
"   Qiang BB, 2024, IND CROP PROD, V222, DOI 10.1016/j.indcrop.2024.119506"
"   Qiao MF, 2014, CHEM ECOL, V30, P555, DOI 10.1080/02757540.2013.868891"
"   Quilb?? J, 2021, NAT COMMUN, V12, DOI 10.1038/s41467-021-21094-7"
"   Rajkumar M, 2012, BIOTECHNOL ADV, V30, P1562, DOI 10.1016/j.biotechadv.2012.04.011"
"   Rehman M, 2023, ENVIRON SCI POLLUT R, V30, P89638, DOI 10.1007/s11356-023-28757-6"
"   Riaz M, 2021, J HAZARD MATER, V402, DOI 10.1016/j.jhazmat.2020.123919"
"   Sasse J, 2018, TRENDS PLANT SCI, V23, P25, DOI 10.1016/j.tplants.2017.09.003"
"   Shu DT, 2024, SCI TOTAL ENVIRON, V949, DOI 10.1016/j.scitotenv.2024.174953"
"   Sun D, 2024, ENVIRON SCI TECHNOL, V58, P7346, DOI 10.1021/acs.est.3c09335"
"   Tang L, 2021, CHEMOSPHERE, V270, DOI 10.1016/j.chemosphere.2020.128634"
"   Tang L, 2020, ENVIRON POLLUT, V265, DOI 10.1016/j.envpol.2020.114861"
"   Tang Y, 2020, INT J ENVIRON AN CH, V100, P567, DOI 10.1080/03067319.2019.1637430"
"   Tao Q, 2016, PLANT SOIL, V398, P139, DOI 10.1007/s11104-015-2651-x"
"   Thilakarathna MS, 2016, AGRON SUSTAIN DEV, V36, DOI 10.1007/s13593-016-0396-4"
"   van der Bom FJT, 2020, J EXP BOT, V71, P5752, DOI 10.1093/jxb/eraa324"
"   Wan Tianying, 2024, Chemosphere, V359, P142331, DOI [10.1016/j.chemosphere.2024.142331, 10.1016/j.chemosphere.2024.142331]"
"   Wan TY, 2021, ENVIRON TECHNOL INNO, V24, DOI 10.1016/j.eti.2021.101923"
"   Wang G, 2020, J HAZARD MATER, V389, DOI 10.1016/j.jhazmat.2019.121873"
"   Wang JX, 2020, J HAZARD MATER, V394, DOI 10.1016/j.jhazmat.2020.122505"
"   Wang L, 2020, ECOTOX ENVIRON SAFE, V206, DOI 10.1016/j.ecoenv.2020.111383"
"   Wang Shuguang, 2024, Science of The Total Environment, V956, P177350, DOI [10.1016/j.scitotenv.2024.177350, 10.1016/j.scitotenv.2024.177350]"
"   Wang XH, 2023, ECOTOX ENVIRON SAFE, V256, DOI 10.1016/j.ecoenv.2023.114849"
"   Wang YL, 2022, PLANT DIVERSITY, V44, P127, DOI 10.1016/j.pld.2021.06.005"
"   Wang YL, 2020, PLANT SOIL, V447, P135, DOI 10.1007/s11104-019-03972-8"
"   Wang YZ, 2025, AGR ECOSYST ENVIRON, V381, DOI 10.1016/j.agee.2024.109450"
"   Wu F, 2021, J HAZARD MATER, V410, DOI 10.1016/j.jhazmat.2020.124550"
"   Xiao ZH, 2020, ECOTOX ENVIRON SAFE, V205, DOI 10.1016/j.ecoenv.2020.111162"
"   Xue T, 2023, SCI TOTAL ENVIRON, V878, DOI 10.1016/j.scitotenv.2023.163133"
"   Yan Ren-Jun, 2020, Journal of Agro-Environment Science, V39, P2162"
"   Yan YX, 2022, SCI TOTAL ENVIRON, V812, DOI 10.1016/j.scitotenv.2021.152475"
"   Yang JX, 2024, ECOL ENG, V207, DOI 10.1016/j.ecoleng.2024.107356"
"   Yang JX, 2017, ECOL ENG, V109, P35, DOI 10.1016/j.ecoleng.2017.09.001"
"   Yang X, 2021, J HAZARD MATER, V406, DOI 10.1016/j.jhazmat.2020.124325"
"   Yu H, 2024, ENVIRON SCI TECHNOL, V58, P16891, DOI 10.1021/acs.est.4c05291"
"   Yu RP, 2022, PLANT SOIL, V476, P263, DOI 10.1007/s11104-022-05487-1"
"   Yu RP, 2021, TRENDS PLANT SCI, V26, P1227, DOI 10.1016/j.tplants.2021.07.014"
"   Yu S, 2024, SCI TOTAL ENVIRON, V951, DOI 10.1016/j.scitotenv.2024.175336"
"   Zeng P, 2019, CHEMOSPHERE, V220, P237, DOI 10.1016/j.chemosphere.2018.12.073"
"   Zeng P, 2019, SCI TOTAL ENVIRON, V650, P594, DOI 10.1016/j.scitotenv.2018.09.055"
"   Zeng WB, 2024, SCI TOTAL ENVIRON, V915, DOI 10.1016/j.scitotenv.2024.170074"
"   Zhalnina K, 2018, NAT MICROBIOL, V3, P470, DOI 10.1038/s41564-018-0129-3"
"   Zhang DG, 2022, SCI TOTAL ENVIRON, V850, DOI 10.1016/j.scitotenv.2022.157767"
"   Zhang WP, 2024, PLANT SOIL, V502, P7, DOI 10.1007/s11104-023-06464-y"
"   Zhang WP, 2021, J APPL ECOL, V58, P2603, DOI 10.1111/1365-2664.13989"
"   Zhang X, 2023, J HAZARD MATER, V452, DOI 10.1016/j.jhazmat.2023.131342"
"   Zhao M, 2025, BIOCHAR, V7, DOI 10.1007/s42773-024-00418-y"
"   Zhao XY, 2023, CHEM ENG J, V477, DOI 10.1016/j.cej.2023.147311"
"   Zhao YN, 2020, TRENDS PLANT SCI, V25, P446, DOI 10.1016/j.tplants.2020.01.010"
"   Zheng XJ, 2024, J ENVIRON CHEM ENG, V12, DOI 10.1016/j.jece.2024.114366"
"   Zheng XJ, 2024, ECOTOX ENVIRON SAFE, V284, DOI 10.1016/j.ecoenv.2024.116883"
"   Zhu XF, 2011, PLANT CELL ENVIRON, V34, P1055, DOI 10.1111/j.1365-3040.2011.02304.x"
"   Zhuang Y, 2024, PLANT PHYSIOL BIOCH, V210, DOI 10.1016/j.plaphy.2024.108619"
"   Zou JC, 2021, CHEMOSPHERE, V276, DOI 10.1016/j.chemosphere.2021.130223"
NR 105
TC 1
Z9 1
U1 41

U2 41
PU ELSEVIER
PI AMSTERDAM
"PA RADARWEG 29, 1043 NX AMSTERDAM, NETHERLANDS"
SN 0929-1393
EI 1873-0272
J9 APPL SOIL ECOL
JI Appl. Soil Ecol.
PD FEB
PY 2026
VL 218
AR 106673
DI 10.1016/j.apsoil.2025.106673
EA DEC 2025
PG 14
WC Soil Science
WE Science Citation Index Expanded (SCI-EXPANDED)
SC Agriculture
GA U5414
UT WOS:001638703000001
DA 2026-04-06
ER

PT J
"AU Dusengemungu, L"
"   Gwanama, C"
"   Simuchimba, G"
"   Mubemba, B"
"AF Dusengemungu, Leonce"
"   Gwanama, Cousins"
"   Simuchimba, Grant"
"   Mubemba, Benjamin"
TI Potential of bioaugmentation of heavy metal contaminated soils in the
   Zambian Copperbelt using autochthonous filamentous fungi
SO FRONTIERS IN MICROBIOLOGY
LA English
DT Article
DE bioaugmentation; Aspergillus transmontanensis; Geotrichum candidum;
   Cladosporium; heavy metal; contaminated soil
ID GEOTRICHUM-CANDIDUM; BIOSORPTION; TOLERANCE; BIOREMEDIATION;
   IDENTIFICATION; COBALT; BIOACCUMULATION; MECHANISMS; LEAD;
   MICROORGANISMS
"AB There is great potential to remediate heavy metal contaminated environments through bioaugmentation with filamentous fungi. However, these fungi have been poorly investigated in most developing countries, such as Zambia. Therefore, the present study aimed at isolating indigenous filamentous fungi from heavy metal contaminated soil and to explore their potential for use in bioaugmentation. The conventional streak plate method was used to isolate fungi from heavy metal-contaminated soil. Filamentous fungal isolates were identified using morphological and molecular techniques. The radial growth diameter technique was used to evaluate heavy metal tolerance of the fungi. The most abundant and highly tolerant fungi, identified as Aspergillus transmontanensis, Cladosporium cladosporioides, and Geotrichum candidum species, were used to bioremediate heavy metal contaminated soil samples with uncontaminated soil sample being employed as a control. A maximum tolerance index (TI) between 0.7 and 11.0 was observed for A. transmontanensis, and G. candidum while C. cladosporioides displayed the TI between 0.2 and 1.2 in the presence of 1,000 ppm of Cu, Co, Fe, Mn, and Zn. The interspecific interaction was analyzed to determine the compatibility among isolates. Our results showed mutual intermingling between the three evaluated fungal species, which confirms their common influence in biomineralization of heavy metals in contaminated soils. Maximum bio-removal capacities after 90 days were 72% for Cu, 99.8% for Co, 60.6% for Fe, 82.2% for Mn, and 100% for both Pb and Zn. This study has demonstrated the potential of highly resistant autochthonous fungal isolates to remediate the heavy metal contamination problem."
"C1 [Dusengemungu, Leonce] Copperbelt Univ, Sch Math & Nat Sci, Kitwe, Zambia."
"   [Dusengemungu, Leonce] Copperbelt Univ, Africa Ctr Excellence Sustainable Min, Kitwe, Zambia."
"   [Gwanama, Cousins; Simuchimba, Grant; Mubemba, Benjamin] Copperbelt Univ, Sch Nat Resources, Kitwe, Zambia."
C3 Copperbelt University; Copperbelt University; Copperbelt University
"RP Dusengemungu, L (corresponding author), Copperbelt Univ, Sch Math & Nat Sci, Kitwe, Zambia.; Dusengemungu, L (corresponding author), Copperbelt Univ, Africa Ctr Excellence Sustainable Min, Kitwe, Zambia."
EM dusengeleonce@yahoo.fr
"RI Dusengemungu, Leonce/HKE-7763-2023"
"OI Dusengemungu, Leonce/0000-0003-2908-2110"
"CR Abd El-Rahim WM, 2003, J BASIC MICROB, V43, P367, DOI 10.1002/jobm.200310267"
"   Abu-Elsaoud AM, 2017, PLOS ONE, V12, DOI 10.1371/journal.pone.0188220"
"   Acosta-Rodr??guez I, 2018, BIOINORG CHEM APPL, V2018, DOI 10.1155/2018/3457196"
"   Adeyemi AO, 2005, BIOMETALS, V18, P269, DOI 10.1007/s10534-005-1539-2"
"   Ahemad Munees, 2014, Journal of King Saud University Science, V26, P1, DOI 10.1016/j.jksus.2013.05.001"
"   Ahmad E., 2012, TOXICITY HEAVY METAL, P29, DOI DOI 10.1007/978-3-7091-0730-0_2"
"   Alori E., 2012, Journal of Agricultural Science (Toronto), V4, P246, DOI 10.5539/jas.v4n8p246"
"   Amorim SS, 2018, J ENVIRON CHEM ENG, V6, P4538, DOI 10.1016/j.jece.2018.06.061"
"   Anahid S, 2011, SCI IRAN, V18, P502, DOI 10.1016/j.scient.2011.05.015"
"   [Anonymous], 2020, R: A language and environment for statistical computing"
"   [Anonymous], 2020, RStudio: Integrated Development for R"
"   Arita C, 2014, EUR J PLANT PATHOL, V139, P655, DOI 10.1007/s10658-014-0438-1"
"   Arland P, 2006, BIORESOURCE TECHNOL, V97, P1018, DOI 10.1016/j.biortech.2005.04.046"
"   Arriaga FL., 2017, SOIL HLTH INTENSIFIC, P109, DOI DOI 10.1016/B978-0-12-805317-1.00005-1"

"   Bellion M, 2006, FEMS MICROBIOL LETT, V254, P173, DOI 10.1111/j.1574-6968.2005.00044.x"
"   Bhandari G., 2021, Microbes and Signaling Biomolecules against Plant Stress: Strategies of Plant-Microbe Relationships for Better Survival, P55, DOI 10.1007/978-981-15-7094-04"
"   Boddy L., 2016, INTERACTIONS FUNGI O"
"   Bosco F., 2019, Environmental chemistry and recent pollution control approaches"
"   Burford EP, 2003, MINERAL MAG, V67, P1127, DOI 10.1180/0026461036760154"
"   Chakravarty R, 2012, BIORESOURCE TECHNOL, V108, P176, DOI 10.1016/j.biortech.2011.12.100"
"   Chileshe MN, 2020, J FORESTRY RES, V31, P1283, DOI 10.1007/s11676-019-00921-0"
"   Coelho E, 2020, CHEMOSPHERE, V248, DOI 10.1016/j.chemosphere.2020.126068"
"   Comensoli L, 2017, ADV APPL MICROBIOL, V98, P31, DOI 10.1016/bs.aambs.2016.11.001"
"   Cook JM, 1997, MAR POLLUT BULL, V34, P637, DOI 10.1016/S0025-326X(96)00186-5"
"   Das AP, 2015, SOIL BIOL, V45, P313, DOI 10.1007/978-3-319-19018-1_16"
"   Das SK, 2007, ENVIRON SCI TECHNOL, V41, P8281, DOI 10.1021/es070814g"
"   Deshmukh R, 2016, INDIAN J MICROBIOL, V56, P247, DOI 10.1007/s12088-016-0584-6"
"   Dhankhar R, 2011, ENVIRON TECHNOL, V32, P467, DOI 10.1080/09593330.2011.572922"
"   Dursun AY, 2003, PROCESS BIOCHEM, V38, P1647, DOI 10.1016/S0032-9592(02)00075-4"
"   Dusengemungu L, 2022, SCI REP-UK, V12, DOI 10.1038/s41598-022-15458-2"
"   Dusengemungu L, 2021, Environmental Advances, V5, P100083, DOI [10.1016/j.envadv.2021.100083, 10.1016/j.envadv.2021.100083, DOI 10.1016/J.ENVADV.2021.100083]"
"   Dusengemungu L, 2020, FRONT MICROBIOL, V11, DOI 10.3389/fmicb.2020.582016"
"   Emenike CU, 2017, J GEOCHEM EXPLOR, V182, P275, DOI 10.1016/j.gexplo.2016.10.002"
"   Emenike CU, 2016, ENVIRON EARTH SCI, V75, DOI 10.1007/s12665-015-4805-9"
"   Esshaimi M, 2017, Linnaeus Eco-Tech, P975, DOI [10.15626/eco-tech.2010.102, 10.15626/eco-tech.2010.102, DOI 10.15626/ECO-TECH.2010.102]"
"   Ettler V, 2011, GEODERMA, V164, P73, DOI 10.1016/j.geoderma.2011.05.014"
"   Ezekoye CC, 2018, SUSTAIN CHEM PHARM, V10, P148, DOI 10.1016/j.scp.2018.11.003"
"   Ezzouhri L, 2009, AFR J MICROBIOL RES, V3, P35"
"   Fawzy E. M., 2017, Journal of Bioremediation and Biodegradation, V8, P394, DOI [10.5897/jtehs2017.0385, 10.4172/2155-6199.1000394]"
"   Festin ES, 2019, J FORESTRY RES, V30, P381, DOI 10.1007/s11676-018-0621-x"
"   Frey-Klett P, 2011, MICROBIOL MOL BIOL R, V75, P583, DOI 10.1128/MMBR.00020-11"
"   Gadd G. M., 2004, Mycologist, V18, P60, DOI [10.1017/s0269-915x(04)00202-2, 10.1017/S0269915X04002022]"
"   GADD GM, 1993, NEW PHYTOL, V124, P25, DOI 10.1111/j.1469-8137.1993.tb03796.x"
"   Gathuru G., 2011, PERFORMANCE SELECTED"
"   Gente S, 2006, J IND MICROBIOL BIOT, V33, P1019, DOI 10.1007/s10295-006-0130-3"
"   Gherbawy Y, 2010, MOLECULAR IDENTIFICATION OF FUNGI, P213, DOI 10.1007/978-3-642-05042-8_11"
"   Gola D, 2016, BIORESOURCE TECHNOL, V218, P388, DOI 10.1016/j.biortech.2016.06.096"
"   Goltapeh E.M., 2013, Fungi as bioremediators"
"   Griffin D.H., 1995, METAL IONS FUNGI"
"   Guindon S, 2005, NUCLEIC ACIDS RES, V33, pW557, DOI 10.1093/nar/gki352"
"   Gupta S, 2017, PLANT MICROBE INTERA, V2, P593, DOI DOI 10.1007/978-981-10-6593-4_24"
"   Halfeld-Vieira BA, 2020, PLANT DIS, V104, P1537, DOI 10.1094/PDIS-11-19-2484-PDN"
"   Hansda A, 2016, WORLD J MICROB BIOT, V32, DOI 10.1007/s11274-016-2117-1"
"   Harms H, 2011, NAT REV MICROBIOL, V9, P177, DOI 10.1038/nrmicro2519"
"   Hassan A, 2020, BIOCHEM ENG J, V157, DOI 10.1016/j.bej.2020.107550"
"   Hassan A, 2019, WATER AIR SOIL POLL, V230, DOI 10.1007/s11270-019-4227-5"
"   He MY, 2022, ECOTOX ENVIRON SAFE, V236, DOI 10.1016/j.ecoenv.2022.113497"
"   Iram S, 2015, ARAB J SCI ENG, V40, P1867, DOI 10.1007/s13369-015-1702-1"
"   Iskandar NL, 2011, J ENVIRON SCI, V23, P824, DOI 10.1016/S1001-0742(10)60475-5"
"   Jayaraman M., 2014, Int. J. Sci. Res., V3, P335"
"   Joshi PK, 2011, INDIAN J MICROBIOL, V51, P482, DOI 10.1007/s12088-011-0110-9"
"   Kapungwe E., 2013, J GEOGRAPHY GEOLOGY, V5, P55, DOI [10.5539/jgg.v5n2p55, DOI 10.5539/JGG.V5N2P55]"
"   Katoh K, 2019, BRIEF BIOINFORM, V20, P1160, DOI 10.1093/bib/bbx108"
"   Kausar H, 2010, INT BIODETER BIODEGR, V64, P594, DOI 10.1016/j.ibiod.2010.06.012"
"   Khan I, 2019, ENVIRON MONIT ASSESS, V191, DOI 10.1007/s10661-019-7781-9"
"   Khan I, 2019, ENVIRON MONIT ASSESS, V191, DOI 10.1007/s10661-019-7769-5"
"   Khatri N, 2015, FRONT LIFE SCI, V8, P23, DOI 10.1080/21553769.2014.933716"
"   Leck A., 1999, COMMUNITY EYE HLTH, V12, P30"
"   Lee S, 2015, FUNGAL BIOL-UK, V119, P1311, DOI 10.1016/j.funbio.2015.10.006"
"   Lefort V, 2017, MOL BIOL EVOL, V34, P2422, DOI 10.1093/molbev/msx149"
"   Lestan D, 1996, J IND MICROBIOL, V16, P286, DOI 10.1007/BF01570036"
"   Letunic I, 2021, NUCLEIC ACIDS RES, V49, pW293, DOI 10.1093/nar/gkab301"
"   Liang JJ, 2022, MOLECULES, V27, DOI 10.3390/molecules27175562"
"   Lobos A, 2021, J APPL MICROBIOL, V131, P743, DOI 10.1111/jam.14947"
"   Malik A, 2004, ENVIRON INT, V30, P261, DOI 10.1016/j.envint.2003.08.001"
"   Mancera-L??pez M, 2008, INT BIODETER BIODEGR, V61, P151, DOI 10.1016/j.ibiod.2007.05.012"
"   Medaura MC, 2021, FRONT MICROBIOL, V12, DOI 10.3389/fmicb.2021.626436"
"   Mensah A. K., 2015, International Journal of Biodiversity and Conservation, V7, P57, DOI 10.5897/ijbc2014.0775"
"   Michael G, 2014, FUNGAL BIOL REV, V28, P36, DOI 10.1016/j.fbr.2014.05.001"
"   Migahed F, 2017, INT J ENVIRON SCI TE, V14, P1169, DOI 10.1007/s13762-016-1229-3"
"   Mohanty S, 2017, GEOMICROBIOL J, V34, P309, DOI 10.1080/01490451.2016.1189016"
"   Moll J, 2016, PLOS ONE, V11, DOI 10.1371/journal.pone.0148130"
"   More TT, 2010, BIORESOURCE TECHNOL, V101, P7691, DOI 10.1016/j.biortech.2010.05.033"
"   Moretti A., 2017, Mycotoxigenic fungi Methods and Protocols"
"   Mu??oz AJ, 2012, CHEM ENG J, V210, P325, DOI 10.1016/j.cej.2012.09.007"
"   Murugesan K, 2014, BIORESOURCE TECHNOL, V168, P198, DOI 10.1016/j.biortech.2014.04.063"
"   Oladipo OG, 2018, BRAZ J MICROBIOL, V49, P29, DOI 10.1016/j.bjm.2017.06.003"
"   Opaluwa O. D., 2012, Advances in Applied Science Research, V3, P780"
"   Pal A, 2006, BIORESOURCE TECHNOL, V97, P1253, DOI 10.1016/j.biortech.2005.01.043"
"   Pehoiu G, 2020, PLANT SOIL, V456, P405, DOI 10.1007/s11104-020-04725-8"
"   Pettersson UT, 2001, CHEM GEOL, V177, P399, DOI 10.1016/S0009-2541(00)00422-8"
"   Philpott CC, 2006, BBA-MOL CELL RES, V1763, P636, DOI 10.1016/j.bbamcr.2006.05.008"
"   Pourret O, 2016, APPL GEOCHEM, V64, P43, DOI 10.1016/j.apgeochem.2015.07.012"

"   Prescott C.E., 2021, Soils and landscape restoration, P299, DOI [DOI 10.1016/B978-0-12-813193-0.00011-4, 10.1016/B978-0-12- 813193-0.00011-4]"
"   Priyadarshini E, 2021, CHEMOSPHERE, V274, DOI 10.1016/j.chemosphere.2021.129976"
"   Rambaut A, 2016, VIRUS EVOL, V2, DOI 10.1093/ve/vew007"
"   Rashmi K, 2004, SCI TOTAL ENVIRON, V328, P1, DOI 10.1016/j.scitotenv.2004.02.009"
"   Ren WX, 2009, J HAZARD MATER, V167, P164, DOI 10.1016/j.jhazmat.2008.12.104"
"   Robinson JR, 2021, J FUNGI, V7, DOI 10.3390/jof7030225"
"   Rousk J, 2010, SOIL BIOL BIOCHEM, V42, P926, DOI 10.1016/j.soilbio.2010.02.009"
"   Sa W, 2020, ACTA SCIENTIFIC MICROBIOLOGY, V3, P220, DOI [10.31080/asmi.2020.03.0572, 10.31080/ASMI.2020.03.0572]"
"   Sabra N, 2012, Adv Chem Eng Sci, V2, P1, DOI DOI 10.4236/ACES.2012.21001"
"   Samson RA, 2014, STUD MYCOL, P141, DOI 10.1016/j.simyco.2014.07.004"
"   Santos F, 2019, ECOSYSTEM CONSEQUENCES OF SOIL WARMING: MICROBES, VEGETATION, FAUNA, AND SOIL BIOGEOCHEMISTRY, P209, DOI 10.1016/B978-0-12-813493-1.00010-7"
"   Schubert K, 2007, STUD MYCOL, P105, DOI 10.3114/sim.2007.58.05"
"   Sey E., 2021, HEAVY METALS TOLERAN"
"   Shafiquzzaman Siddiquee Shafiquzzaman Siddiquee, 2013, Advances in Bioscience and Biotechnology, V4, P570, DOI 10.4236/abb.2013.44075"
"   Silva IS, 2009, BIORESOURCE TECHNOL, V100, P4669, DOI 10.1016/j.biortech.2009.03.079"
"   Simate GS, 2010, HYDROMETALLURGY, V103, P150, DOI 10.1016/j.hydromet.2010.03.012"
"   Simonovicov?? A, 2019, ECOTOX ENVIRON SAFE, V172, P194, DOI 10.1016/j.ecoenv.2019.01.064"
"   Singh A., 2021, FUNGAL COMMUNITIES R, P127"
"   Singh M, 2015, J APPL MICROBIOL, V119, P1278, DOI 10.1111/jam.12948"
"   STAHL PD, 1992, SOIL BIOL BIOCHEM, V24, P309, DOI 10.1016/0038-0717(92)90190-9"
"   Staniland S, 2010, GEOMICROBIOL J, V27, P656, DOI 10.1080/01490450903527994"
"   Szada-Borzyszkowska A, 2022, INT J ENVIRON SCI TE, V19, P9365, DOI 10.1007/s13762-021-03783-4"
"   Talibi I, 2012, CROP PROT, V35, P41, DOI 10.1016/j.cropro.2011.12.016"
"   Talukdar D, 2020, ENVIRON TECHNOL INNO, V20, DOI 10.1016/j.eti.2020.101050"
"   Termorshuizen AJ, 2016, MICROBIOL SPECTR, V4, DOI 10.1128/microbiolspec.FUNK-0013-2016"
"   TOBIN JM, 1994, J IND MICROBIOL, V13, P126, DOI 10.1007/BF01584110"
"   Torri D., 2017, PLOS ONE, V12, P1, DOI [DOI 10.1371/JOURNAL.PONE.017078, DOI 10.1371/JOURNAL.PONE.0170782]"
"   Tsekova K, 2007, CR ACAD BULG SCI, V60, P63"
"   Tutu H, 2008, APPL GEOCHEM, V23, P3666, DOI 10.1016/j.apgeochem.2008.09.002"
"   Tuzen M, 2007, ANAL CHIM ACTA, V581, P241, DOI 10.1016/j.aca.2006.08.040"
"   Ullah R, 2019, WATER AIR SOIL POLL, V230, DOI 10.1007/s11270-019-4089-x"
"   Vacar CL, 2021, J FUNGI, V7, DOI 10.3390/jof7050386"
"   V??zquez-Campos X, 2015, ENVIRON SCI TECHNOL, V49, P8487, DOI 10.1021/acs.est.5b01342"
"   Velkova Z, 2018, ENG LIFE SCI, V18, P871, DOI 10.1002/elsc.201800017"
"   Visagie CM, 2020, STUD MYCOL, P253, DOI 10.1016/j.simyco.2020.02.003"
"   V??tkov?? M, 2010, MINERAL MAG, V74, P581, DOI 10.1180/minmag.2010.074.4.581"
"   Wang MX, 2015, CHEM ENG J, V259, P837, DOI 10.1016/j.cej.2014.08.003"
"   Watanabe T., 2016, PICT ATL SOIL SEED"
"   Wickham H, 2009, USE R, P1, DOI 10.1007/978-0-387-98141-3"
"   Wong C, 2018, LETT APPL MICROBIOL, V67, P384, DOI 10.1111/lam.13049"
"   Wood JL, 2016, AIMS BIOENG, V3, P211, DOI 10.3934/bioeng.2016.2.211"
"   Wu BH, 2022, SCI TOTAL ENVIRON, V808, DOI 10.1016/j.scitotenv.2021.151995"
"   Yin GH, 2017, J FUNGI, V3, DOI 10.3390/jof3010012"
"   Yu X., 2020, NANOMATERIALS TOXICI"
"   Zafar A, 2007, WORLD BANK RES OBSER, V22, P103, DOI 10.1093/wbro/lkm001"
"   Zafar S, 2007, BIORESOURCE TECHNOL, V98, P2557, DOI 10.1016/j.biortech.2006.09.051"
"   Zhang XC, 2022, ENVIRON SCI POLLUT R, V29, P1937, DOI 10.1007/s11356-021-15776-4"
NR 140
TC 16
Z9 17
U1 3
U2 47
PU FRONTIERS MEDIA SA
PI LAUSANNE
"PA AVENUE DU TRIBUNAL FEDERAL 34, LAUSANNE, CH-1015, SWITZERLAND"
EI 1664-302X
J9 FRONT MICROBIOL
JI Front. Microbiol.
PD DEC 1
PY 2022
VL 13
AR 1045671
DI 10.3389/fmicb.2022.1045671
PG 18
WC Microbiology
WE Science Citation Index Expanded (SCI-EXPANDED)
SC Microbiology
GA 6Y6JE
UT WOS:000897198400001
PM 36532421
"OA Green Submitted, gold"
DA 2026-04-06
ER

PT J
"AU Mohamed, HI"
"   Ullah, I"
"   Toor, MD"
"   Tanveer, NA"

"   Din, MMU"
"   Basit, A"
"   Sultan, Y"
"   Muhammad, M"
"   Rehman, MU"
"AF Mohamed, Heba I."
"   Ullah, Izhar"
"   Toor, Muhammad Danish"
"   Tanveer, Nouraiz Ahmed"
"   Din, Muhammad Mughees Ud"
"   Basit, Abdul"
"   Sultan, Yaqoob"
"   Muhammad, Murad"
"   Rehman, Muneeb Ur"
TI Heavy metals toxicity in plants: understanding mechanisms and developing
   coping strategies for remediation: a review
SO BIORESOURCES AND BIOPROCESSING
LA English
DT Review
DE Heavy metal; Remediation techniques; Soil health and ecosystem;
   Sustainable agriculture
ID ARBUSCULAR MYCORRHIZAL FUNGI; GROWTH-PROMOTING BACTERIA; HEXAVALENT
   CHROMIUM; CONTAMINATED SOIL; BACILLUS-SUBTILIS; CADMIUM STRESS;
   TRACE-METALS; CD; PHYTOREMEDIATION; LEAD
"AB Heavy metal (HM) contamination is an increasing environmental and agricultural concern due to the persistence, toxicity, and bioaccumulative nature of metals such as cadmium (Cd), lead (Pb), mercury (Hg), and arsenic (As). These pollutants are primarily introduced through industrial effluents, mining, and agrochemicals, negatively impacting soil health, crop productivity, and food safety, ultimately posing serious risks to both ecosystems and human health. Conventional remediation methods can be costly, labor-intensive, and environmentally disruptive. Heavy metals like Cd, Pb, Hg, and As disrupt cellular homeostasis, inhibit photosynthesis, generate oxidative stress, and interfere with nutrient uptake, leading to significant yield losses in plants. In response to these stresses, plants utilize complex molecular mechanisms for tolerance, including the activation of antioxidant enzymes, upregulation of metal transporters, production of metal-chelating molecules, and modulation of stress-responsive genes and transcription factors. In contrast, bioremediation offers a sustainable and eco-friendly alternative by leveraging the detoxification capabilities of plants, microbes, and their symbiotic interactions. Techniques such as phytoremediation, microbial-assisted remediation, and integrated strategies involving biochar and organic amendments have demonstrated promising results in restoring heavy metal-contaminated soils. Recent advancements in molecular biology and synthetic biology have further improved the efficiency of bioremediation through the genetic engineering of hyperaccumulator plant species and metal-resistant microbes. This review examines the toxic effects of heavy metals on plants and highlights innovative, nature-based remediation strategies, emphasizing their potential for scalable and sustainable environmental cleanup."
"C1 [Mohamed, Heba I.] Ain Shams Univ, Fac Educ, Dept Biol & Geol Sci, Cairo 11341, Egypt."
"   [Ullah, Izhar] Ondokuz Mayis Univ, Fac Agr, Dept Hort, Samsun, Turkey."
"   [Toor, Muhammad Danish] Ondokuz Mayis Univ, Dept Soil Sci & Plant Nutr, Samsun, Turkey."
"   [Tanveer, Nouraiz Ahmed] Ondokuz Mayis Univ, Fac Agr, Dept Agr Biotechnol, Samsun, Turkey."
"   [Din, Muhammad Mughees Ud] Univ Agr Faisalabad, Inst Soil & Environm Sci, Faisalabad, Pakistan."
"   [Basit, Abdul] Kyungpook Natl Univ, Dept Hort Sci, Daegu 41566, South Korea."
"   [Sultan, Yaqoob] Lithuanian Res Ctr Agr & Forestry, Inst Agr, Dept Grass Breeding, LT-58344 Kedainiu, Lithuania."
"   [Muhammad, Murad] Chinese Acad Sci, Xinjiang Inst Ecol & Geog, State Key Lab Desert & Oasis Ecol, Key Lab Ecol Safety & Sustainable Dev Arid Lands, Urumqi 830011, Peoples R China."
"   [Muhammad, Murad] Univ Chinese Acad Sci, Beijing 100049, Peoples R China."
"   [Muhammad, Murad] Chinese Acad Sci, Xinjiang Inst Ecol & Geog 10, Xinjiang Key Lab Biodivers Conservat & Applicat Ar, Beijing, Peoples R China."
"   [Rehman, Muneeb Ur] Agr Univ, Fac Agron, Dept Agrochem & Soil Sci, Plovdiv, Bulgaria."
C3 Egyptian Knowledge Bank (EKB); Ain Shams University; Ondokuz Mayis
   University; Ondokuz Mayis University; Ondokuz Mayis University;
   University of Agriculture Faisalabad; Kyungpook National University
   (KNU); Chinese Academy of Sciences; Xinjiang Institute of Ecology &
"   Geography, CAS; Chinese Academy of Sciences; University of Chinese"
"   Academy of Sciences, CAS; Chinese Academy of Sciences; Agricultural"
   University of Plovdiv
"RP Mohamed, HI (corresponding author), Ain Shams Univ, Fac Educ, Dept Biol & Geol Sci, Cairo 11341, Egypt."
EM hebaibrahim79@gmail.com
"RI ullah, izhar/ABM-1357-2022; Sultan, Yaqoob/MTB-3585-2025; Basit,"
"   Abdul/AAX-2414-2021; MUHAMMAD, MURAD/KLZ-2989-2024; Mohamed,"
   Heba/U-8673-2019
"OI Sultan, Yaqoob/0000-0001-6585-0768; Basit, Abdul/0000-0003-3324-6296;"
"   Mohamed, Heba/0000-0002-6892-3376"
"FU Science, Technology & Innovation Funding Authority (STDF); Egyptian"
   Knowledge Bank (EKB)
"FX Open access funding provided by The Science, Technology & Innovation"
   Funding Authority (STDF) in cooperation with The Egyptian Knowledge Bank
   (EKB).
"CR Abdel Wahab Mostafa M., 2023, Nanotechnology for Environmental Engineering, P347, DOI [10.1007/s41204-022-00274-w, 10.1007/s41204-022-00274-w]"
"   Abu-Elsaoud AM, 2017, PLOS ONE, V12, DOI 10.1371/journal.pone.0188220"
"   Abu-Shahba MS, 2022, SCI HORTIC-AMSTERDAM, V293, DOI 10.1016/j.scienta.2021.110727"
"   Adepoju AO., 2024, Soil pollution and management practices, DOI [10.1016/B978-0-323-95967-4.00011-8, DOI 10.1016/B978-0-323-95967-4.00011-8]"
"   Adeyemi NO, 2021, RHIZOSPHERE-NETH, V18, DOI 10.1016/j.rhisph.2021.100325"
"   Adhikary S, 2024, GEOMICROBIOL J, DOI 10.1080/01490451.2024.2340517"
"   Adrees M, 2021, ECOTOX ENVIRON SAFE, V208, DOI 10.1016/j.ecoenv.2020.111627"

"   Agarwal VP., 2022, The Pharma Innovat J, V11, P2716"
"   Ahmad M., 2021, Environ Sci Pollut Res, V28, P29832, DOI [10.1007/s11356-020-12210-0, DOI 10.1007/S11356-020-12210-0]"
"   Ahmad M, 2019, SOIL SCI PLANT NUTR, V65, P183, DOI 10.1080/00380768.2019.1570333"
"   Ahmad P, 2020, PLANTS-BASEL, V9, DOI 10.3390/plants9070825"
"   Ahmed T, 2022, J HAZARD MATER, V423, DOI 10.1016/j.jhazmat.2021.127024"
"   Ahmed T, 2021, ENVIRON POLLUT, V288, DOI 10.1016/j.envpol.2021.117785"
"   Aka RJN, 2016, INT J PHYTOREMEDIAT, V18, P200, DOI 10.1080/15226514.2015.1073671"
"   Al Mahmud J., 2019, Reactive oxygen species metabolism and antioxidant defense in plants under metal/metalloid stress, DOI [10.1007/978-3-030-06118-0_10, DOI 10.1007/978-3-030-06118-0_10]"
"   AL-Huqail AA, 2022, INT J ENVIRON SCI TE, V19, P5301, DOI 10.1007/s13762-021-03532-7"
"   Albert Q, 2018, CHEMOSPHERE, V196, P386, DOI 10.1016/j.chemosphere.2017.12.156"
"   Alengebawy A, 2021, TOXICS, V9, DOI 10.3390/toxics9030042"
"   Ali A, 2024, PLOS ONE, V19, DOI 10.1371/journal.pone.0297764"
"   Ali A, 2017, SCI REP-UK, V7, DOI 10.1038/srep46087"
"   Ali H., 2022, Environ. Res., V207, DOI [10.1016/j.envres.2021.112160, DOI 10.1016/J.ENVRES.2021.112160]"
"   Ali H, 2019, J CHEM-NY, V2019, DOI 10.1155/2019/6730305"
"   Ali H, 2013, CHEMOSPHERE, V91, P869, DOI 10.1016/j.chemosphere.2013.01.075"
"   Ali S., 2021, Environ Pollut, V283, DOI [10.1016/j.envpol.2021.117071, DOI 10.1016/J.ENVPOL.2021.117071]"
"   Ali S., 2022, Environ Pollut, V293, DOI [10.1016/j.envpol.2021.118533, DOI 10.1016/J.ENVPOL.2021.118533]"
"   Aljohani MS, 2024, INT J BIOL MACROMOL, V259, DOI 10.1016/j.ijbiomac.2023.129145"
"   Alori ET, 2022, FRONT SOIL SCI, V2, DOI 10.3389/fsoil.2022.937186"
"   Ameen F, 2023, FUNCT PLANT BIOL, V50, P623, DOI 10.1071/FP23027"
"   An QY, 2024, J HAZARD MATER, V476, DOI 10.1016/j.jhazmat.2024.135028"
"   Ansari F, 2024, TOXIN REV, V43, P63, DOI 10.1080/15569543.2023.2283768"
"   Aponte H, 2020, SCI TOTAL ENVIRON, V737, DOI 10.1016/j.scitotenv.2020.139744"
"   Aqeel U, 2023, PLANT STRESS, V8, DOI 10.1016/j.stress.2023.100161"
"   Asghar N, 2024, J NANOBIOTECHNOL, V22, DOI 10.1186/s12951-023-02151-3"
"   Ashraf S., 2022, Environ Pollut, V310, DOI [10.1016/j.envpol.2022.119845, DOI 10.1016/J.ENVPOL.2022.119845]"
"   Atigh Z. Biglari Quchan, 2020, Iranian Journal of Energy and Environment, V11, P89, DOI [10.5829/ijee.2020.11.02.01, 10.5829/ijee.2020.11.02.01]"
"   Ayangbenro AS, 2017, INT J ENV RES PUB HE, V14, DOI 10.3390/ijerph14010094"
"   Azimi B, 2024, J POLYM ENVIRON, V32, P345, DOI 10.1007/s10924-023-02989-6"
"   Babashpour-Asl M, 2022, ENVIRON SCI POLLUT R, V29, P80021, DOI 10.1007/s11356-022-19941-1"
"   Badawy AA, 2024, AGRONOMY-BASEL, V14, DOI 10.3390/agronomy14040797"
"   Baig U, 2022, ADV COLLOID INTERFAC, V303, DOI 10.1016/j.cis.2022.102635"
"   Barhoum A, 2022, NANOMATERIALS-BASEL, V12, DOI 10.3390/nano12020177"
"   Beiyuan J, 2017, CHEMOSPHERE, V178, P110, DOI 10.1016/j.chemosphere.2017.03.022"
"   Belhassan M, 2024, BRAZ J MICROBIOL, V55, P843, DOI 10.1007/s42770-024-01263-8"
"   Benettayeb A, 2024, Environmental Technology Reviews, V13, P398, DOI [10.1080/21622515.2024.2354519, 10.1080/21622515.2024.2354519, DOI 10.1080/21622515.2024.2354519]"
"   Bolan N., 2023, Sci Total Environ, V868, DOI [10.1016/j.scitotenv.2023.161733 161733, DOI 10.1016/J.SCITOTENV.2023.161733161733]"
"   Boriov?? K, 2014, LETT APPL MICROBIOL, V59, P217, DOI 10.1111/lam.12266"
"   Bosch-Serra AD, 2020, J ENVIRON MANAGE, V273, DOI 10.1016/j.jenvman.2020.111092"
"   Bozzi AT, 2021, J MOL BIOL, V433, DOI 10.1016/j.jmb.2021.166991"
"   Bruno LB, 2021, J ENVIRON MANAGE, V289, DOI 10.1016/j.jenvman.2021.112553"
"   Cabral L, 2015, WORLD J MICROB BIOT, V31, P1655, DOI 10.1007/s11274-015-1918-y"
"   Cecchi G, 2017, J ENVIRON SCI HEAL B, V52, P191, DOI 10.1080/03601234.2017.1261549"
"   ??evik B, 2025, PROTEIN J, V44, P308, DOI 10.1007/s10930-025-10258-9"
"   Chaitanya M.V.N.L., 2024, ORGANIC MICROPOLLUTA, P67, DOI DOI 10.1007/978-3-031-48977-8_4"
"   Chakraborty S, 2021, BIOCATAL AGR BIOTECH, V38, DOI 10.1016/j.bcab.2021.102204"
"   Chatterjee S., 2023, Front Microbiol, V14, P1121230, DOI [10.3389/fmicb.2023.1121230, DOI 10.3389/FMICB.2023.1121230]"
"   Chen F, 2022, ENVIRON POLLUT, V308, DOI 10.1016/j.envpol.2022.119606"
"   Chen Z, 2024, ENVIRON SCI-NANO, V11, P3457, DOI [10.1039/D4EN00271G, 10.1039/d4en00271g]"
"   Chen ZQ, 2024, SCI TOTAL ENVIRON, V932, DOI 10.1016/j.scitotenv.2024.173029"
"   Cheng FL, 2024, ENVIRON RES, V241, DOI 10.1016/j.envres.2023.117504"
"   Chernyshova I, 2023, CURR OPIN COLLOID IN, V68, DOI 10.1016/j.cocis.2023.101763"
"   Chibuike G.U., 2014, APPL ENVIRON SOIL SC, DOI [10.1155/2014/752708, DOI 10.1155/2014/752708]"
"   Chmielowska-Bak J, 2014, FRONT PLANT SCI, V5, DOI 10.3389/fpls.2014.00245"
"   Chmielowska-Bak J, 2013, J PLANT PHYSIOL, V170, P1585, DOI 10.1016/j.jplph.2013.06.019"
"   Coninx L, 2017, ADV BOT RES, V83, P127, DOI 10.1016/bs.abr.2016.12.005"
"   Conte S, 2024, J HAZARD MATER, V474, DOI 10.1016/j.jhazmat.2024.134657"
"   Curry-Lindahl K., 2019, Curry-Lindahl K. Conservation Problems of Savannahs and Other Grasslands, P359"
"   Dar MI., 2018, Heavy metal hyperaccumulation and hypertolerance in Brassicaceae, DOI [10.1007/978-981-13-2242-6_10, DOI 10.1007/978-981-13-2242-6_10]"
"   Das J, 2018, SCI TOTAL ENVIRON, V624, P1106, DOI 10.1016/j.scitotenv.2017.12.157"
"   Das N., 2022, Plant metal tolerance proteins: insight into their roles in metal transport and homeostasis for future biotechnological applications"
"   Dawood MFA, 2023, PLANT SOIL, V483, P225, DOI 10.1007/s11104-022-05732-7"
"   Dell'Anno F, 2022, MICROORGANISMS, V10, DOI 10.3390/microorganisms10050993"
"   Deng SX, 2024, BIOTECHNOL ADV, V72, DOI 10.1016/j.biotechadv.2024.108337"
"   Dey M, 2021, HELIYON, V7, DOI 10.1016/j.heliyon.2021.e08625"
"   Ding YF, 2020, J AGR FOOD CHEM, V68, P1958, DOI 10.1021/acs.jafc.9b07468"
"   Direk A, 2024, PLANT STRESS, V12, DOI 10.1016/j.stress.2024.100458"
"   Dixit R, 2015, SUSTAINABILITY-BASEL, V7, P2189, DOI 10.3390/su7022189"
"   Dutta Subhayan, 2021, Journal of Applied Biology & Biotechnology, V9, P69, DOI [10.7324/jabb.2021.9409, 10.7324/JABB.2021.9409]"
"   EL-khawaga HA, 2024, SCI REP-UK, V14, DOI 10.1038/s41598-024-61936-0"
"   El-Mahdy OM, 2021, ENVIRON SCI POLLUT R, V28, P67608, DOI 10.1007/s11356-021-15382-4"
"   Elik ??, 2025, LIFE-BASEL, V15, DOI 10.3390/life15020310"

"   Elsehly EM., 2024, InCarbon Nanotubes-Recent Advances, Perspectives and Applications, DOI [10.5772/intechopen.114885, DOI 10.5772/INTECHOPEN.114885]"
"   Emamverdian Abolghassem, 2015, ScientificWorldJournal, V2015, P756120, DOI [10.1155/2015/756120, 10.1155/2015/756120]"
"   Evangelou M., 2015, PHYTOREMEDIATION, P253, DOI DOI 10.1007/978-3-319-10395-2_17"
"   Faiz S, 2022, CHEMOSPHERE, V290, DOI 10.1016/j.chemosphere.2021.133200"
"   Faiz S, 2022, INT J PHYTOREMEDIAT, V24, P364, DOI 10.1080/15226514.2021.1949263"
"   Faizan M, 2022, METALS-BASEL, V12, DOI 10.3390/met12122030"
"   Farooq MA, 2016, S AFR J BOT, V104, P61, DOI 10.1016/j.sajb.2015.11.006"
"   Fashola MO, 2016, INT J ENV RES PUB HE, V13, DOI 10.3390/ijerph13111047"
"   Filippi L, 2021, INT J MOL SCI, V22, DOI 10.3390/ijms22063036"
"   Freedman Z, 2012, APPL ENVIRON MICROB, V78, P6568, DOI 10.1128/AEM.01060-12"
"   Fu XX, 2023, SUSTAINABILITY-BASEL, V15, DOI 10.3390/su15065361"
"   Gao ML, 2024, SCI TOTAL ENVIRON, V924, DOI 10.1016/j.scitotenv.2024.171633"
"   Geioushy RA, 2024, ENVIRON SCI POLLUT R, DOI 10.1007/s11356-024-32625-2"
"   Ghanaim AM, 2025, BMC MICROBIOL, V25, DOI 10.1186/s12866-024-03703-9"
"   Ghanaim AM, 2025, MICROB CELL FACT, V24, DOI 10.1186/s12934-025-02730-z"
"   Gupta DK., 2021, Environ Nanotechnol Monit Manag, V16, DOI [DOI 10.1016/J.ENMM.2021.100538, 10.1016/j.enmm.2021.100538]"
"   Gupta DK., 2022, Environ Sci Pollut Res, V29, P22340, DOI [10.1007/s11356-022-19177-y, DOI 10.1007/S11356-022-19177-Y]"
"   Gupta Pratima, 2017, Biotechnol Rep (Amst), V13, P58, DOI [10.1016/j.btre.2016.12.006, 10.1016/j.btre.2016.12.006]"
"   Gupta S., 2023, J Hazard Mater, V448, DOI [10.1016/j.jhazmat.2023.130847, DOI 10.1016/J.JHAZMAT.2023.130847]"
"   Hamid Y, 2019, SCI TOTAL ENVIRON, V660, P80, DOI 10.1016/j.scitotenv.2018.12.419"
"   Hao SH, 2021, HORTICULTURAE, V7, DOI 10.3390/horticulturae7060132"
"   Hasanuzzaman M., 2022, Plants, V11, P345, DOI [DOI 10.3390/PLANTS11030345, 10.3390/plants11030345]"
"   He J, 2019, INT BIODETER BIODEGR, V140, P67, DOI 10.1016/j.ibiod.2019.03.012"
"   He P, 2023, FRONT PLANT SCI, V14, DOI 10.3389/fpls.2023.1080881"
"   Hern??ndez-Guerrero AA, 2025, PROCESSES, V13, DOI 10.3390/pr13010276"
"   Hu T, 2022, SCI TOTAL ENVIRON, V814, DOI 10.1016/j.scitotenv.2021.152630"
"   Huang D, 2025, WORLD J MICROB BIOT, V41, DOI 10.1007/s11274-024-04247-w"
"   Huang Q, 2024, ENVIRON POLLUT, V342, DOI 10.1016/j.envpol.2023.123013"
"   Huang YH, 2025, CHEM ENG J, V506, DOI 10.1016/j.cej.2025.160030"
"   Iftikhar U., 2021, Strategies to reduce heavy metal contamination in soil-plant system, DOI [10.1201/9781003155089-13, DOI 10.1201/9781003155089-13]"
"   Isaac CPJ, 2013, DESALIN WATER TREAT, V51, P7700, DOI 10.1080/19443994.2013.778218"
"   Islam M. S., 2022, SOIL HLTH ENV SUSTAI, DOI [DOI 10.1007/978-3-031-09270-1_27, 10.1007/978-3-031-09270-1_27]"
"   Islam MS, 2024, Environmental Advances, V15, P100468, DOI [10.1016/j.envadv.2023.100468, DOI 10.1016/J.ENVADV.2023.100468, 10.1016/j.envadv.2023.100468]"
"   Jacinto MJ, 2024, INT J ENVIRON SCI TE, V21, P5865, DOI 10.1007/s13762-023-05444-0"
"   Jahan I, 2022, J POLYM ENVIRON, V30, P1709, DOI 10.1007/s10924-021-02312-1"
"   Jain Harshita, 2021, Nanotechnology for Environmental Engineering, V6, DOI [10.1007/s41204-021-00161-w, 10.1007/s41204-021-00161-w]"
"   Jalil Sanaullah, 2023, Chemosphere, V338, P139566, DOI 10.1016/j.chemosphere.2023.139566"
"   Jeyakumar P, 2023, ENVIRON ENG RES, V28, DOI 10.4491/eer.2021.631"
"   Ji Y, 2022, ENVIRON SCI POLLUT R, V29, P25568, DOI 10.1007/s11356-021-17760-4"
"   Jin CW, 2013, PLANT SOIL, V371, P387, DOI 10.1007/s11104-013-1699-8"
"   Jorjani Sarah, 2024, International Journal of Secondary Metabolite, V11, P169, DOI [10.21448/ijsm.1323494, 10.21448/ijsm.1323494]"
"   Kaamoush M, 2022, ENVIRON SCI POLLUT R, V29, P78942, DOI 10.1007/s11356-022-21328-1"
"   Kanwal H, 2024, ENVIRON SCI POLLUT R, DOI 10.1007/s11356-024-32516-6"
"   Kaur J, 2020, ECOTOX ENVIRON SAFE, V195, DOI 10.1016/j.ecoenv.2020.110480"
"   Kaur R, 2021, PHYSIOL PLANTARUM, V173, P430, DOI 10.1111/ppl.13491"
"   Kessler A, 2022, NANOMATERIALS-BASEL, V12, DOI 10.3390/nano12111922"
"   Kessler A, 2018, ANNU REV ECOL EVOL S, V49, P115, DOI 10.1146/annurev-ecolsys-110617-062406"
"   Khalid SA., 2022, Polymeric and Natural Composites"
"   Khamare Y, 2023, FRONT AGRON, V5, DOI 10.3389/fagro.2023.1235196"
"   Khan I, 2019, ENVIRON MONIT ASSESS, V191, DOI 10.1007/s10661-019-7781-9"
"   Khan I, 2019, ENVIRON MONIT ASSESS, V191, DOI 10.1007/s10661-019-7769-5"
"   Khan MU., 2022, Environ Technol Innov, V27, DOI [10.1016/j.eti.2022.102456, DOI 10.1016/J.ETI.2022.102456]"
"   Khan MA, 2022, CHEMOSPHERE, V294, DOI 10.1016/j.chemosphere.2022.133796"
"   Khidr R, 2025, ENVIRON GEOCHEM HLTH, V47, DOI 10.1007/s10653-025-02392-1"
"   Kiany T, 2022, ENVIRON SCI POLLUT R, V29, P34725, DOI 10.1007/s11356-021-17927-z"
"   Kim S, 2018, FRONT PHYSIOL, V9, DOI 10.3389/fphys.2018.00885"
"   Koleva L, 2022, FRONT PLANT SCI, V13, DOI 10.3389/fpls.2022.806781"
"   Komis G, 2018, ANNU REV PLANT BIOL, V69, P237, DOI 10.1146/annurev-arplant-042817-040314"
"   Kouki R, 2021, FOOD SCI NUTR, V9, P3280, DOI 10.1002/fsn3.2294"
"   Kumar K, 2020, J PLANT BIOCHEM BIOT, V29, P700, DOI 10.1007/s13562-020-00596-3"
"   Kumar S, 2023, INT J ENVIRON AN CH, V103, P7961, DOI 10.1080/03067319.2021.1977930"
"   Kumar V., 2015, Environ Sci Pollut Res, V22, P15306, DOI [10.1007/s11356-015-4717-1, DOI 10.1007/S11356-015-4717-1]"
"   Kumari S., 2022, Ecotoxicol Environ Saf, V238, DOI [DOI 10.1016/J.ECOENV.2022.113589, 10.1016/j.ecoenv.2022.113589]"
"   Lakshmi DS, 2024, WASTE BIOMASS VALORI, V15, P6067, DOI 10.1007/s12649-024-02601-5"
"   Le Gall H., 2021, Plants, V10, P142, DOI [10.3390/plants10010142, DOI 10.3390/PLANTS10010142]"
"   Li C, 2020, ENVIRON POLLUT, V260, DOI 10.1016/j.envpol.2020.114075"
"   Li D, 2022, J HAZARD MATER, V422, DOI 10.1016/j.jhazmat.2021.126936"
"   Li SC, 2022, INT J MOL SCI, V23, DOI 10.3390/ijms23084463"
"   Li X, 2024, J ENVIRON MANAGE, V366, DOI 10.1016/j.jenvman.2024.121751"
"   Lin WT, 2016, J ENVIRON SCI-CHINA, V45, P94, DOI 10.1016/j.jes.2015.12.010"

"   Liosis C, 2021, MATERIALS, V14, DOI 10.3390/ma14247500"
"   Liu HW, 2019, INT J PHYTOREMEDIAT, V21, P699, DOI 10.1080/15226514.2018.1556589"
"   Liu LW, 2018, SCI TOTAL ENVIRON, V633, P206, DOI 10.1016/j.scitotenv.2018.03.161"
"   Liu P, 2024, PLANT PHYSIOL BIOCH, V212, DOI 10.1016/j.plaphy.2024.108740"
"   Liu YN, 2019, ECOL INDIC, V101, P249, DOI 10.1016/j.ecolind.2019.01.029"
"   Liu YK, 2019, BIOCHEM BIOPH RES CO, V516, P747, DOI 10.1016/j.bbrc.2019.06.116"
"   Lu CC, 2025, GENES-BASEL, V16, DOI 10.3390/genes16060628"
"   Lu JZ, 2019, CHEMOSPHERE, V225, P395, DOI 10.1016/j.chemosphere.2019.03.021"
"   Ma Y, 2016, FRONT PLANT SCI, V7, DOI 10.3389/fpls.2016.00918"
"   Mafane D, 2025, SUSTAINABILITY-BASEL, V17, DOI [10.3390/su17083567, 10.3390/su17083567]"
"   Mahbub KR, 2017, APPL MICROBIOL BIOT, V101, P963, DOI 10.1007/s00253-016-8079-2"
"   Malik S, 2022, J BASIC MICROB, V62, P361, DOI 10.1002/jobm.202100555"
"   Mani A., 2022, Plant Metal and Metalloid Transporters"
"   Mao Q, 2024, ENVIRON POLLUT, V341, DOI 10.1016/j.envpol.2023.123001"
"   Mariz-Ponte N, 2021, PLANT PHYSIOL BIOCH, V167, P1, DOI 10.1016/j.plaphy.2021.07.021"
"   MathuMitha C., 2021, International Journal of Zoological Investigations, V7, P762, DOI 10.33745/ijzi.2021.v07i02.061"
"   Mathur J, 2024, ENVIRON SCI POLLUT R, DOI 10.1007/s11356-024-32499-4"
"   Mazibuko MT, 2024, SUSTAINABILITY-BASEL, V16, DOI 10.3390/su16083265"
"   Meena NL., 2024, Antioxidant defense mechanism and high-temperature stress tolerance in plants, DOI [10.1007/978-981-97-1699-9_8, DOI 10.1007/978-981-97-1699-9_8]"
"   Mireles-Arriaga A, 2021, POL J ENVIRON STUD, V30, P5185, DOI 10.15244/pjoes/135760"
"   Mishra J, 2017, FRONT MICROBIOL, V8, DOI 10.3389/fmicb.2017.01706"
"   Mohamed Heba I., 2024, Notulae Scientia Biologicae, V16, DOI [10.55779/nsb16312001, 10.55779/nsb16312001]"
"   Mohamed RR., 2024, Smart Polymers and Their Different Applications, DOI [10.1007/978-981-97-2761-2_11, DOI 10.1007/978-981-97-2761-2_11]"
"   Mohasin M, 2022, International Journal of Environment and Climate Change, P1231, DOI [10.9734/ijecc/2022/v12i121563, DOI 10.9734/IJECC/2022/V12I121563, 10.9734/ijecc/2022/v12i121563]"
"   Mondal S., 2023, Modern Approaches in Waste Bioremediation"
"   Mondal S, 2022, PLANTA, V255, DOI 10.1007/s00425-022-03869-4"
"   Morita AKM, 2024, INT J ENVIRON SCI TE, V21, P4307, DOI 10.1007/s13762-023-05306-9"
"   Motesharezadeh B, 2017, POLLUTION, V3, P575, DOI 10.22059/POLL.2017.62774"
"   Muhammad T, 2019, MOLECULES, V24, DOI 10.3390/molecules24030556"
"   Mustapha M. U., 2015, Journal of Microbial and Biochemical Technology, V7, P253, DOI 10.4172/1948-5948.1000219"
"   Nazir MM, 2022, FRONT PLANT SCI, V13, DOI 10.3389/fpls.2022.843795"
"   Ni H, 2012, CHEM ENG J, V204, P264, DOI 10.1016/j.cej.2012.05.029"
"   Niri SR, 2025, CAN GEOTECH J, V62, DOI 10.1139/cgj-2024-0069"
"   Noctor G., 2023, Plant J, V113, P412, DOI [DOI 10.1111/TPJ.16261, 10.1111/tpj.16261]"
"   Noctor G, 2024, J EXP BOT, V75, P4549, DOI 10.1093/jxb/erae194"
"   Nogueira MA., 2021, Environ Exp Bot, V186, DOI [10.1016/j.envexpbot.2021.104441, DOI 10.1016/J.ENVEXPBOT.2021.104441]"
"   Noman M, 2020, ECOTOX ENVIRON SAFE, V192, DOI 10.1016/j.ecoenv.2020.110303"
"   Nyabadza A, 2024, J ENVIRON CHEM ENG, V12, DOI 10.1016/j.jece.2024.112643"
"   Ojuederie OB, 2017, INT J ENV RES PUB HE, V14, DOI 10.3390/ijerph14121504"
"   Okereafor U, 2020, INT J ENV RES PUB HE, V17, DOI 10.3390/ijerph17072204"
"   Oladipo OG, 2018, BRAZ J MICROBIOL, V49, P29, DOI 10.1016/j.bjm.2017.06.003"
"   Opdenakker K, 2012, ENVIRON EXP BOT, V83, P53, DOI 10.1016/j.envexpbot.2012.04.003"
"   Osman Hanan E., 2024, Environmental Science and Pollution Research International, V31, P47655, DOI [10.1007/s11356-024-34223-8, 10.1007/s11356-024-34223-8]"
"   Osman HE, 2023, Journal of Umm Al-Qura University for Applied Sciences, V9, P77, DOI [10.1007/s43994-022-00022-5, 10.1007/s43994-022-00022-5, DOI 10.1007/S43994-022-00022-5]"
"   Osterberg JT, 2018, ADV BOT RES, V87, P57, DOI 10.1016/bs.abr.2018.09.004"
"   Pan FS, 2017, INT J PHYTOREMEDIAT, V19, P281, DOI 10.1080/15226514.2016.1225280"
"   Pande V, 2022, FRONT MICROBIOL, V13, DOI 10.3389/fmicb.2022.824084"
"   Pandey C, 2020, INTERNATIONAL JOURNAL OF PLANT AND ENVIRONMENT, V6, P264, DOI [10.18811/ijpen.v6i04.04, 10.18811/ijpen.v6i04.04, DOI 10.18811/IJPEN.V6I04.04, 10.18811/ijpen.v6i04., DOI 10.18811/IJPEN.V6I04]"
"   Pervaiz I, 2013, APPL BIOCHEM MICRO+, V49, P437, DOI 10.1134/S0003683813050098"
"   Perveen S., 2024, Nanotechnological approaches for restoring metalloid contaminated soil, DOI [10.1201/9781003414964-12, DOI 10.1201/9781003414964-12]"
"   Phukunkamkaew S, 2023, ENVIRON GEOCHEM HLTH, V45, P187, DOI 10.1007/s10653-022-01291-z"
"   Povedano-Priego C, 2017, MINER ENG, V106, P46, DOI 10.1016/j.mineng.2016.11.007"
"   Prakash V, 2022, CHEMOSPHERE, V303, DOI 10.1016/j.chemosphere.2022.134554"
"   Praveen A, 2018, J PLANT GROWTH REGUL, V37, P612, DOI 10.1007/s00344-017-9760-0"
"   Pujari M., 2021, Heavy Metals in the Environment, P1, DOI [10.1016/B978-0-12-821656-9.00001-8, DOI 10.1016/B978-0-12-821656-9.00001-8]"
"   Qader MQ, 2025, BIOREMEDIAT J, DOI 10.1080/10889868.2025.2496988"
"   Qattan SYA, 2025, ENVIRON SCI EUR, V37, DOI 10.1186/s12302-025-01103-y"
"   Qi WJ, 2025, PLANTS-BASEL, V14, DOI [10.3390/plants14081191, 10.3390/plants14081191]"
"   Qian XY, 2017, J CLEAN PROD, V164, P198, DOI 10.1016/j.jclepro.2017.06.195"
"   Qu JH, 2024, CHEM ENG J, V488, DOI 10.1016/j.cej.2024.150925"
"   Radziemska M, 2017, INT J ENV RES PUB HE, V14, DOI 10.3390/ijerph14090958"
"   Raheem Nelofer Abdul, 2025, Environmental Science and Pollution Research International, V32, P22036, DOI [10.1007/s11356-024-34602-1, 10.1007/s11356-024-34602-1]"
"   Rahman A., 2024, Chemosphere, V351, DOI [10.1016/j.chemosphere.2024.139817, DOI 10.1016/J.CHEMOSPHERE.2024.139817]"
"   Rai GK, 2021, PHYSIOL PLANTARUM, V173, P287, DOI 10.1111/ppl.13433"
"   Raklami A, 2022, INT J MOL SCI, V23, DOI 10.3390/ijms23095031"
"   Raklami A, 2019, MICROORGANISMS, V7, DOI 10.3390/microorganisms7070212"
"   Ramanathan S, 2024, J ENVIRON CHEM ENG, V12, DOI 10.1016/j.jece.2024.112931"
"   Rao KP, 2011, ARCH BIOCHEM BIOPHYS, V506, P73, DOI 10.1016/j.abb.2010.11.006"
"   Revin VV, 2023, INT J MOL SCI, V24, DOI 10.3390/ijms241914608"
"   Riaz M, 2021, J HAZARD MATER, V402, DOI 10.1016/j.jhazmat.2020.123919"

"   Rigoletto M, 2020, APPL SCI-BASEL, V10, DOI 10.3390/app10103528"
"   Riyazuddin R, 2022, BIOMOLECULES, V12, DOI 10.3390/biom12010043"
"   Rodriguez L., 2024, J Environ Manag, V344, DOI [10.1016/j.jenvman.2024.119213, DOI 10.1016/J.JENVMAN.2024.119213]"
"   Roy R, 2024, APPL BIOCHEM BIOTECH, V196, P1712, DOI 10.1007/s12010-023-04614-7"
"   Rudnicki Konrad, 2024, Science of The Total Environment, V944, P173941, DOI [10.1016/j.scitotenv.2024.173941, 10.1016/j.scitotenv.2024.173941]"
"   Saboor A, 2021, SAUDI J BIOL SCI, V28, P6339, DOI 10.1016/j.sjbs.2021.06.096"
"   Saeed HZ, 2025, RUSS J PLANT PHYSL+, V72, DOI 10.1134/S102144372560196X"
"   Saleem K, 2023, BIOL BULL+, V50, P390, DOI 10.1134/S1062359022602245"
"   Saleem Y, 2024, J AGR FOOD RES, V15, DOI 10.1016/j.jafr.2023.100953"
"   Salem SS., 2024, Advanced (nano) materials, DOI [10.1016/B978-0-323-99278-7.00011-0, DOI 10.1016/B978-0-323-99278-7.00011-0]"
"   Santos M, 2025, SOIL SYST, V9, DOI 10.3390/soilsystems9020035"
"   Senila M, 2024, HELIYON, V10, DOI 10.1016/j.heliyon.2024.e25303"
"   Serrano M, 2014, FRONT PLANT SCI, V5, DOI 10.3389/fpls.2014.00274"
"   Seshadri B, 2015, J SOIL SCI PLANT NUT, V15, P524"
"   Shafi Z, 2024, APPL BIOCHEM BIOTECH, V196, P3949, DOI 10.1007/s12010-023-04730-4"
"   Shafiq T, 2022, ANTIOXIDANTS-BASEL, V11, DOI 10.3390/antiox11112156"
"   Shahzad A, 2024, BMC PLANT BIOL, V24, DOI 10.1186/s12870-024-04812-3"
"   Shahzad K, 2024, SCI REP-UK, V14, DOI 10.1038/s41598-024-64596-2"
"   Sharma Priyanka, 2024, Environmental Nanotechnology, Monitoring and Management, V22, DOI [10.1016/j.enmm.2024.100970, 10.1016/j.enmm.2024.100970]"
"   Sharma P., 2024, Front Microbiol, V15, P1245786, DOI [10.3389/fmicb.2024.1245786, DOI 10.3389/FMICB.2024.1245786]"
"   Sharma P, 2021, BIORESOURCE TECHNOL, V320, DOI 10.1016/j.biortech.2020.124353"
"   Sharma SS, 2021, TRENDS PLANT SCI, V26, P452, DOI 10.1016/j.tplants.2020.11.003"
"   Shikha Srivastava Shikha Srivastava, 2016, Journal of Bioremediation and Biodegradation, V7, P323, DOI 10.4172/2155-6199.1000323"
"   Shrivastava M., 2019, Plant-Metal Interactions, DOI [10.1007/978-3-030-20732-8, DOI 10.1007/978-3-030-20732-8]"
"   Singh G, 2019, SOIL SEDIMENT CONTAM, V28, P729, DOI 10.1080/15320383.2019.1657381"
"   Singh R., 2023, Front Plant Sci, V14, P1126789, DOI [10.3389/fpls.2023.1126789, DOI 10.3389/FPLS.2023.1126789]"
"   Singh S, 2016, FRONT PLANT SCI, V6, DOI 10.3389/fpls.2015.01143"
"   Sivapullaiah PV, 2015, J ELECTROCHEM SCI EN, V5, DOI [10.5599/jese.2015.0055, 10.5599/jese.2015.0055, DOI 10.5599/JESE.2015.0055]"
"   Smail Harem Othman, 2024, Journal of Experimental and Molecular Biology, V25, P133, DOI [10.47743/jemb-2024-187, 10.47743/jemb-2024-187]"
"   Sobhanardakani S, 2019, BIOL TRACE ELEM RES, V187, P602, DOI 10.1007/s12011-018-1383-1"
"   Song Y, 2016, ENVIRON SCI POLLUT R, V23, P10577, DOI 10.1007/s11356-015-5966-5"
"   Srichandan H, 2014, HYDROMETALLURGY, V150, P130, DOI 10.1016/j.hydromet.2014.09.019"
"   Sumathi S, 2021, BIOREMEDIAT J, V25, P91, DOI 10.1080/10889868.2020.1853029"
"   Swamynaidu M, 2024, CONSTR BUILD MATER, V431, DOI 10.1016/j.conbuildmat.2024.136533"
"   Tajudin SAA, 2016, IOP CONF SER-MAT SCI, V136, DOI 10.1088/1757-899X/136/1/012043"
"   Tang HY, 2024, FRONT PLANT SCI, V15, DOI 10.3389/fpls.2024.1420408"
"   Tanveer Y, 2022, ENVIRON POLLUT, V300, DOI 10.1016/j.envpol.2022.118889"
"   Tauqeer H.M., 2021, Approaches in remediation of inorganic pollutants, P155, DOI DOI 10.1007/978-981-15-6221-1_8"
"   Thatoi H, 2014, J ENVIRON MANAGE, V146, P383, DOI 10.1016/j.jenvman.2014.07.014"
"   Timothy N, 2019, International Journal of Environmental Chemistry, V3, P72, DOI [10.11648/j.ijec.20190302.14, DOI 10.11648/J.IJEC.20190302.14, 10.11648/J.IJEC.20190302.14]"
"   Tirry N, 2021, J GENET ENG BIOTECHN, V19, DOI 10.1186/s43141-021-00254-8"
"   Tong T, 2024, COORDIN CHEM REV, V500, DOI 10.1016/j.ccr.2023.215498"
"   Topuz B, 2025, B ENVIRON CONTAM TOX, V114, DOI 10.1007/s00128-025-04067-0"
"   Tripathi M, 2023, FRONT MICROBIOL, V14, DOI 10.3389/fmicb.2023.1168954"
"   Tufail MK, 2025, SEP PURIF TECHNOL, V354, DOI 10.1016/j.seppur.2024.128739"
"   Ullah I, 2023, WATER AIR SOIL POLL, V234, DOI 10.1007/s11270-023-06675-0"
"   Rehman Z, 2023, SCI TOTAL ENVIRON, V867, DOI 10.1016/j.scitotenv.2023.161468"
"   Usoltsev VA, 2019, ECOL QUEST, V30, P29, DOI 10.12775/EQ.2019.021"
"   van Loon LC, 2006, ANNU REV PHYTOPATHOL, V44, P135, DOI 10.1146/annurev.phyto.44.070505.143425"
"   Vaseem H, 2017, ECOTOX ENVIRON SAFE, V145, P42, DOI 10.1016/j.ecoenv.2017.07.001"
"   Viana RDR, 2024, J ENVIRON MANAGE, V353, DOI 10.1016/j.jenvman.2024.120204"
"   Wang X, 2021, ENVIRON POLLUT, V277, DOI 10.1016/j.envpol.2021.116758"
"   Wang YT, 2024, J MOL LIQ, V400, DOI 10.1016/j.molliq.2024.124484"
"   Wu DX, 2021, MEMBRANES-BASEL, V11, DOI 10.3390/membranes11120984"
"   Xie Y, 2021, ENVIRON RES, V200, DOI 10.1016/j.envres.2021.111730"
"   Xu ED, 2024, INT J MOL SCI, V25, DOI 10.3390/ijms25136993"
"   Xu ZG, 2019, ECOTOX ENVIRON SAFE, V171, P301, DOI 10.1016/j.ecoenv.2018.12.084"
"   Xue WX, 2020, BMC PLANT BIOL, V20, DOI 10.1186/s12870-020-02500-6"
"   Yadav S., 2020, J Plant Biol, V63, P101, DOI [10.1007/s12374-020-09255-3, DOI 10.1007/S12374-020-09255-3]"
"   Yadav VK, 2023, WATER-SUI, V15, DOI 10.3390/w15050908"
"   Yan SW, 2021, BMC PLANT BIOL, V21, DOI 10.1186/s12870-021-02929-3"
"   Ye Y, 2013, PLANT CELL ENVIRON, V36, P1, DOI 10.1111/j.1365-3040.2012.02543.x"
"   Yenginar Y, 2024, B ENG GEOL ENVIRON, V83, DOI 10.1007/s10064-024-03800-z"
"   Yin M, 2024, ECOTOX ENVIRON SAFE, V271, DOI 10.1016/j.ecoenv.2024.115951"
"   Yu J, 2024, SCI HORTIC-AMSTERDAM, V324, DOI 10.1016/j.scienta.2023.112554"
"   Yue ZB, 2015, BIORESOURCE TECHNOL, V194, P399, DOI 10.1016/j.biortech.2015.07.042"
"   Zamora-Ledezma C, 2021, ENVIRON TECHNOL INNO, V22, DOI 10.1016/j.eti.2021.101504"
"   Zanganeh F, 2022, ENVIRON SCI POLLUT R, V29, P6040, DOI 10.1007/s11356-021-16061-0"
"   Zhan FD, 2019, INT J PHYTOREMEDIAT, V21, P849, DOI 10.1080/15226514.2019.1577353"
"   Zhang DZ, 2023, J HAZARD MATER, V445, DOI 10.1016/j.jhazmat.2022.130499"
"   Zhang FG, 2019, SCI TOTAL ENVIRON, V655, P1150, DOI 10.1016/j.scitotenv.2018.11.317"

"   Zhang MM, 2022, J INTEGR PLANT BIOL, V64, P301, DOI 10.1111/jipb.13215"
"   Zhao C, 2024, INT J BIOL MACROMOL, V274, DOI 10.1016/j.ijbiomac.2024.133071"
"   Zhao FY, 2014, ACTA PHYSIOL PLANT, V36, P1879, DOI 10.1007/s11738-014-1564-2"
"   Zhou L., 2023, Front Plant Sci, V14, P1123459, DOI [10.3389/fpls.2023.1123459, DOI 10.3389/FPLS.2023.1123459]"
"   Zhou W., 2022, J Hazard Mat, V436, DOI [10.1016/j.jhazmat.2022.129214, DOI 10.1016/J.JHAZMAT.2022.129214]"
"   Zunaidi AA, 2024, HELIYON, V10, DOI 10.1016/j.heliyon.2024.e29528"
NR 297
TC 21
Z9 24
U1 47
U2 54
PU SPRINGER HEIDELBERG
PI HEIDELBERG
"PA TIERGARTENSTRASSE 17, D-69121 HEIDELBERG, GERMANY"
EI 2197-4365
J9 BIORESOUR BIOPROCESS
JI Bioresour. Bioprocess.
PD SEP 4
PY 2025
VL 12
IS 1
AR 95
DI 10.1186/s40643-025-00930-4
PG 44
WC Biotechnology & Applied Microbiology
WE Science Citation Index Expanded (SCI-EXPANDED)
SC Biotechnology & Applied Microbiology
GA 6WZ8D
UT WOS:001564024600001
PM 40906247
"OA Green Submitted, gold"
DA 2026-04-06
ER

PT J
"AU Chun, SJ"
"   Kim, YJ"
"   Cui, Y"
"   Nam, KH"
"AF Chun, Seong-Jun"
"   Kim, Young-Joong"
"   Cui, Yingshun"
"   Nam, Kyong-Hee"
TI Ecological network analysis reveals distinctive microbial modules
   associated with heavy metal contamination of abandoned mine soils in
   Korea
SO ENVIRONMENTAL POLLUTION
LA English
DT Article
DE Abandoned mines; Bioremediation; Heavy metal contamination; Microbial
   community; Network analysis; Microbial interaction
ID BACTERIAL COMMUNITIES; RISK-ASSESSMENT; DIVERSITY; GROWTH;
   BIOREMEDIATION; COOCCURRENCE; REMEDIATION; POLLUTION; SITES
"AB Heavy metal pollution in soil around abandoned mine sites is one of the most critical environmental issues worldwide. Soil microbes form complex communities and perform ecological functions individually or in cooperation with other organisms to adapt to harsh environments. In this study, we investigated the distribution patterns of bacterial and fungal communities in non-contaminated and heavy metal-contaminated soil of the abandoned Samkwang mine in Korea to explore microbial interaction mechanisms and their modular structures. As expected, the bacterial and fungal community structures showed large differences depending on the degree of heavy metal contamination. The microbial network was divided into three modules based on the levels of heavy metal pollution: heavy metal-tolerant (HM-Tol), heavy metal-mid-tolerant (HM-mTol), and heavy metal-sensitive (HM-Sens) modules. Taxonomically, microbes assigned to Vicinamibacterales, Pedosphaeraceae, Nitrosomonadaceae, and Gemmatimonadales were the major groups constituting the HM-Tol module. Among the detected heavy metals (As, Pb, Cd, Cu, and Zn), copper concentrations played a key role in the formation of the HM-Tol module. In addition, filamentous fungi (Fusarium and Mortierella) showed potential interactions with bacteria (Nitrosomonadaceae) that could contribute to module stability in heavy metal-contaminated areas. Overall, heavy metal contamination was accompanied by distinct microbial communities, which could participate in the bioremediation of heavy metals. Analysis of the microbial interactions among bacteria and fungi in the presence of heavy metals could provide fundamental information for developing bioremediation mechanisms for the recovery of heavy metal-contaminated soil."
"C1 [Chun, Seong-Jun; Kim, Young-Joong; Nam, Kyong-Hee] Natl Inst Ecol, LMO Res Team, 1210 Geumgang Ro, Maseo Myeon 33657, Seocheon, South Korea."
"   [Cui, Yingshun] Korea Res Inst Biosci & Biotechnol, Cell Factory Res Ctr, 125 Gwahak Ro, Daejeon 34141, South Korea."
"   [Kim, Young-Joong] Natl Inst Ecol, Div Restorat Res Team, 23,Gowol Gil, Yeongyang 36531, South Korea."
C3 National Institute of Ecology; Korea Research Institute of Bioscience &
   Biotechnology (KRIBB); National Institute of Ecology
"RP Nam, KH (corresponding author), Natl Inst Ecol, LMO Res Team, 1210 Geumgang Ro, Maseo Myeon 33657, Seocheon, South Korea."
EM khnam@nie.re.kr
"OI Chun, Seong Jun/0000-0001-7037-6968; Kim,"
   Young-Joong/0000-0001-5569-2065

FU National Institute of Ecology (NIE) - Ministry of Environment (MOE) of
"   the Republic of Korea [NIE-A-2021-04, NIE-A-2021-10]"
FX This research was supported by the National Institute of Ecology (NIE)
   funded by the Ministry of Environment (MOE) of the Republic of Korea
   (NIE-A-2021-04 and NIE-A-2021-10).
"CR Ahmed AM, 2018, FRONT MICROBIOL, V9, DOI 10.3389/fmicb.2018.01852"
"   Assenov Y, 2008, BIOINFORMATICS, V24, P282, DOI 10.1093/bioinformatics/btm554"
"   Barber??n A, 2012, ISME J, V6, P343, DOI 10.1038/ismej.2011.119"
"   Bourceret A, 2016, MICROB ECOL, V71, P711, DOI 10.1007/s00248-015-0682-8"
"   Bradley IM, 2016, APPL ENVIRON MICROB, V82, P5878, DOI 10.1128/AEM.01630-16"
"   Callahan BJ, 2016, NAT METHODS, V13, P581, DOI [10.1038/NMETH.3869, 10.1038/nmeth.3869]"
"   Chain P, 2003, J BACTERIOL, V185, P2759, DOI 10.1128/JB.185.9.2759-2773.2003"
"   Chen JW, 2020, ECOL INDIC, V111, DOI 10.1016/j.ecolind.2020.106072"
"   Chon HT, 2005, ECON ENVIRON GEOL, V38, P101"
"   Chun SJ, 2020, WATER RES, V170, DOI 10.1016/j.watres.2019.115326"
"   Chun SJ, 2019, FRONT MICROBIOL, V10, DOI 10.3389/fmicb.2019.01637"
"   Crognale S, 2017, FRONT MICROBIOL, V8, DOI 10.3389/fmicb.2017.02202"
"   Csardi G., 2006, INT J COMPLEX SYSTEM, V1695, P1, DOI DOI 10.3724/SP.J.1087.2009.02191"
"   Cui Y, 2020, SCI TOTAL ENVIRON, V721, DOI 10.1016/j.scitotenv.2020.137725"
"   Dominiak DM, 2011, ENVIRON MICROBIOL, V13, P710, DOI 10.1111/j.1462-2920.2010.02375.x"
"   Faust K, 2012, NAT REV MICROBIOL, V10, P538, DOI 10.1038/nrmicro2832"
"   Fierer N, 2017, NAT REV MICROBIOL, V15, P579, DOI 10.1038/nrmicro.2017.87"
"   Friedman J, 2012, PLOS COMPUT BIOL, V8, DOI 10.1371/journal.pcbi.1002687"
"   Gao H, 2019, ENVIRON SCI-PROC IMP, V21, P867, DOI [10.1039/c8em00588e, 10.1039/C8EM00588E]"
"   Giller KE, 1998, SOIL BIOL BIOCHEM, V30, P1389, DOI 10.1016/S0038-0717(97)00270-8"
"   Golebiewski M, 2014, MICROB ECOL, V67, P635, DOI 10.1007/s00248-013-0344-7"
"   GORDON AS, 1993, APPL ENVIRON MICROB, V59, P60, DOI 10.1128/AEM.59.1.60-66.1993"
"   Grilli J, 2016, NAT COMMUN, V7, DOI 10.1038/ncomms12031"
"   Guo HH, 2017, ECOTOX ENVIRON SAFE, V144, P300, DOI 10.1016/j.ecoenv.2017.06.048"
"   Harrell F., 2019, VERSION 42 0 PACKAGE"
"   Harrison JJ, 2007, NAT REV MICROBIOL, V5, P928, DOI 10.1038/nrmicro1774"
"   Hemmat-Jou MH, 2018, ECOTOXICOLOGY, V27, P1281, DOI 10.1007/s10646-018-1981-x"
"   Herlemann DPR, 2011, ISME J, V5, P1571, DOI 10.1038/ismej.2011.41"
"   Hermans SM, 2018, MOL ECOL RESOUR, V18, P557, DOI 10.1111/1755-0998.12762"
"   Hong C, 2015, ENVIRON SCI POLLUT R, V22, P10788, DOI 10.1007/s11356-015-4186-3"
"   Hooper DU, 1998, ECOL MONOGR, V68, P121, DOI 10.1890/0012-9615(1998)068[0121:EOPCAD]2.0.CO;2"
"   Humphries MD, 2008, PLOS ONE, V3, DOI 10.1371/journal.pone.0002051"
"   HURLBERT SH, 1971, ECOLOGY, V52, P577, DOI 10.2307/1934145"
"   Kachenko A, 2006, WATER AIR SOIL POLL, V169, P101, DOI 10.1007/s11270-006-2027-1"
"   Kang CH, 2016, ECOL ENG, V89, P64, DOI 10.1016/j.ecoleng.2016.01.023"
"   Kasemodel MC, 2019, SCI TOTAL ENVIRON, V675, P367, DOI 10.1016/j.scitotenv.2019.04.223"
"   Keshvardoust P, 2019, NPJ BIOFILMS MICROBI, V5, DOI 10.1038/s41522-019-0095-4"
"   Knudsen BE, 2013, ENVIRON POLLUT, V181, P122, DOI 10.1016/j.envpol.2013.06.013"
"   Koechler S, 2015, RES MICROBIOL, V166, P764, DOI 10.1016/j.resmic.2015.03.008"
"   Kowalchuk GA, 2002, ANTON LEEUW INT J G, V81, P509, DOI 10.1023/A:1020565523615"
"   Kuramae EE, 2012, FEMS MICROBIOL ECOL, V79, P12, DOI 10.1111/j.1574-6941.2011.01192.x"
"   Kurtz ZD, 2015, PLOS COMPUT BIOL, V11, DOI 10.1371/journal.pcbi.1004226"
"   Kwon JC, 2012, APPL GEOCHEM, V27, P1020, DOI 10.1016/j.apgeochem.2011.11.015"
"   Lebeau T, 2008, ENVIRON POLLUT, V153, P497, DOI 10.1016/j.envpol.2007.09.015"
"   Lee C.H., 1998, EC ENV GEOL, V31, P111"
"   Li XQ, 2017, ENVIRON POLLUT, V231, P908, DOI 10.1016/j.envpol.2017.08.057"
"   Li X, 2016, RSC ADV, V6, P65816, DOI 10.1039/c6ra11767h"
"   Li ZY, 2014, SCI TOTAL ENVIRON, V468, P843, DOI 10.1016/j.scitotenv.2013.08.090"
"   Lim HS, 2008, J GEOCHEM EXPLOR, V96, P223, DOI 10.1016/j.gexplo.2007.04.008"
"   Lin YB, 2020, ENVIRON SCI POLLUT R, V27, P27819, DOI 10.1007/s11356-020-09049-9"
"   Lin YB, 2019, ECOTOX ENVIRON SAFE, V180, P557, DOI 10.1016/j.ecoenv.2019.05.057"
"   Liu SH, 2017, BIORESOURCE TECHNOL, V224, P25, DOI 10.1016/j.biortech.2016.11.095"
"   Ma Y, 2011, J HAZARD MATER, V195, P230, DOI 10.1016/j.jhazmat.2011.08.034"
"   Maiti R. K., 2004, Proceedings of the Indian National Science Academy Part B Biological Sciences, V70, P1"
"   MIRECO, 2019, YB MIRECO STAT"
"   Nam KH, 2020, SUSTAINABILITY-BASEL, V12, DOI 10.3390/su122310110"
"   Navarro MC, 2008, J GEOCHEM EXPLOR, V96, P183, DOI 10.1016/j.gexplo.2007.04.011"
"   NIAST, 2000, METH SOIL PLANT AN"
"   NIER, 2018, SOIL POLL PROC TEST"
"   Nilsson RH, 2019, NUCLEIC ACIDS RES, V47, pD259, DOI 10.1093/nar/gky1022"
"   NRIAGU JO, 1979, NATURE, V279, P409, DOI 10.1038/279409a0"
"   Oksanen Jari., 2017, COMMUNITY ECOLOGY PA, P1"
"   Oliveira A, 2006, J BIOSCI BIOENG, V102, P157, DOI 10.1263/jbb.102.157"
"   Pennafirme S, 2015, RADIAT PHYS CHEM, V116, P116, DOI 10.1016/j.radphyschem.2015.05.040"
"   Petrovich M, 2017, FEMS MICROBIOL ECOL, V93, DOI 10.1093/femsec/fix047"
"   Pii Y, 2015, BIOL FERT SOILS, V51, P403, DOI 10.1007/s00374-015-0996-1"
"   Poirel J, 2013, RES MICROBIOL, V164, P457, DOI 10.1016/j.resmic.2013.01.012"
"   Prosser JI., 2014, The Prokaryotes, P901, DOI [DOI 10.1007/978-3-642-30197-1372, 10.1007/978-3-642-30197-1_372/COVER, DOI 10.1007/978-3-642-30197-1_372]"
"   Quast C, 2013, NUCLEIC ACIDS RES, V41, pD590, DOI 10.1093/nar/gks1219"
"   Qurbani K, 2020, ARCH MICROBIOL, V202, P1397, DOI 10.1007/s00203-020-01853-8"
"   Rajkumar M, 2010, TRENDS BIOTECHNOL, V28, P142, DOI 10.1016/j.tibtech.2009.12.002"
"   Raspanti E, 2009, CHEMOSPHERE, V76, P48, DOI 10.1016/j.chemosphere.2009.02.030"
"   Shannon P, 2003, GENOME RES, V13, P2498, DOI 10.1101/gr.1239303"
"   Sibanda T, 2019, SCI TOTAL ENVIRON, V650, P2199, DOI 10.1016/j.scitotenv.2018.09.380"
"   Solioz M, 2010, J BIOL INORG CHEM, V15, P3, DOI 10.1007/s00775-009-0588-3"
"   Strickland MS, 2010, SOIL BIOL BIOCHEM, V42, P1385, DOI 10.1016/j.soilbio.2010.05.007"
"   Tamura K, 2013, MOL BIOL EVOL, V30, P2725, DOI 10.1093/molbev/mst197"

"   Team R.C., 2013, R LANG ENV STAT COMP"
"   Ternjej I, 2013, ECOTOX ENVIRON SAFE, V98, P119, DOI 10.1016/j.ecoenv.2013.09.014"
"   Thakur MP, 2017, TRENDS ECOL EVOL, V32, P884, DOI 10.1016/j.tree.2017.09.014"
"   Tosadori Gabriele, 2016, F1000Res, V5, P2524, DOI 10.12688/f1000research.9203.2"
"   Uehling J, 2017, ENVIRON MICROBIOL, V19, P2964, DOI 10.1111/1462-2920.13669"
"   Uehling JK, 2019, FRONT MICROBIOL, V10, DOI 10.3389/fmicb.2019.02163"
"   Verma P., 2016, INT J ENV SCI TOXICO, V4, P61"
"   Wang H., 2005, ACTA ECOL SIN, V25, P598"
"   Xiao EZ, 2021, ENVIRON POLLUT, V268, DOI 10.1016/j.envpol.2020.115834"
"   Yan CC, 2020, SCI TOTAL ENVIRON, V707, DOI 10.1016/j.scitotenv.2019.136116"
"   Ye SJ, 2017, CRIT REV ENV SCI TEC, V47, P1528, DOI 10.1080/10643389.2017.1386951"
"   Zhao HL, 2006, SOIL TILL RES, V87, P175, DOI 10.1016/j.still.2005.03.009"
NR 89
TC 93
Z9 103
U1 17
U2 342
PU ELSEVIER SCI LTD
PI London
"PA 125 London Wall, London, the United Kingdom"
SN 0269-7491
EI 1873-6424
J9 ENVIRON POLLUT
JI Environ. Pollut.
PD NOV 15
PY 2021
VL 289
AR 117851
DI 10.1016/j.envpol.2021.117851
EA AUG 2021
PG 13
WC Environmental Sciences
WE Science Citation Index Expanded (SCI-EXPANDED)
SC Environmental Sciences & Ecology
GA UR3KQ
UT WOS:000696651300005
PM 34358869
DA 2026-04-06
ER

PT J
"AU Xu, W"
"   Jin, Y"
"   Zeng, G"
"AF Xu, Wei"
"   Jin, Yuan"
"   Zeng, Gang"
TI Introduction of heavy metals contamination in the water and soil: a
"   review on source, toxicity and remediation methods"
SO GREEN CHEMISTRY LETTERS AND REVIEWS
LA English
DT Review
DE Heavy metals; contaminated water; soil contamination; sources of heavy
   metals; heavy metal toxicity
ID AQUEOUS-SOLUTIONS; WASTE-WATER; REMOVAL; BIOSORPTION; IONS;
   PHYTOREMEDIATION; BIOREMEDIATION; ENVIRONMENT; MANAGEMENT; STRESS
"AB Heavy metal contamination in water and soil presents a growing global issue that poses significant risks to environmental integrity and human well-being. Various heavy metals, including arsenic (As), lead (Pb), mercury (Hg), cadmium (Cd), and chromium (Cr), contaminate ecosystems. These metals enter the environment through both natural processes and human activities such as coal mining, leather production, metal processing, agriculture, and industrial waste disposal. With their high toxicity and tendency to accumulate in organisms, heavy metals induce oxidative stress in cells, resulting in organelle damage. This toxicity can lead to genetic mutations and histone alterations. Given the severe effects of heavy metals, urgent actions are required to eliminate them from polluted soil and water. While physicochemical techniques like membrane filtration, precipitation, oxidation, and reduction exist, they have limitations. Hence, there is a pressing need to devise environmentally friendly and cost-efficient approaches for heavy metal removal. This article examines heavy metal contamination in water and soil, its adverse impacts, and the cleanup of heavy metals using eco-friendly methods. [GRAPHICAL ABSTRACT]"
"C1 [Xu, Wei; Zeng, Gang] 2nd Geol Brigade Sichuan, Chengdu, Peoples R China."
"   [Jin, Yuan] Shanghai Kangshi Food Technol Co Ltd, Shanghai 201100, Peoples R China."
"RP Jin, Y (corresponding author), Shanghai Kangshi Food Technol Co Ltd, Shanghai 201100, Peoples R China."
EM jinyuan01@hotmail.com
"FU Shanghai Kangshi Food Technology Co., Ltd, Shanghai"
"FX Authors are thankful to the Shanghai Kangshi Food Technology Co., Ltd,"
   Shanghai for necessary support for this work.
"CR Abdelbasir SM, 2020, FRONT CHEM, V8, DOI 10.3389/fchem.2020.00782"
"   Abe C, 2022, MOLECULES, V27, DOI 10.3390/molecules27175451"
"   Abu-Danso E, 2018, ENVIRON POLLUT, V242, P1988, DOI 10.1016/j.envpol.2018.07.044"
"   Adeoye A. O., 2022, PHYTOREMEDIATION, P227, DOI [10.1016/B978-0-323-89874-4.00002-9, DOI 10.1016/B978-0-323-89874-4.00002-9]"
"   Agnello AC, 2016, SCI TOTAL ENVIRON, V563, P693, DOI 10.1016/j.scitotenv.2015.10.061"
"   Ahemad Munees, 2015, Journal of Genetic Engineering and Biotechnology, V13, P51, DOI [10.1016/j.jgeb.2015.02.001, 10.1016/j.jgeb.2015.02.001]"
"   Ahluwalia S. S., 2014, International Journal of Current Microbiology and Applied Sciences, V3, P383"

"   Akhtar N, 2021, WATER-SUI, V13, DOI 10.3390/w13192660"
"   Akpor O., 2014, Advances in Bioscience and Bioengineering, V2, P37, DOI DOI 10.11648/J.ABB.20140204.11"
"   AKSU Z, 1992, ENVIRON TECHNOL, V13, P579, DOI 10.1080/09593339209385186"
"   Al Masud MA, 2023, SCI TOTAL ENVIRON, V904, DOI 10.1016/j.scitotenv.2023.166813"
"   Al-Enezi G, 2004, J ENVIRON SCI HEAL A, V39, P455, DOI 10.1081/ESE-120027536"
"   Alengebawy A, 2021, TOXICS, V9, DOI 10.3390/toxics9030042"
"   Ali H, 2019, J CHEM-NY, V2019, DOI 10.1155/2019/6730305"
"   Ali Q, 2023, Hybrid Advances, V4, P100091, DOI [10.1016/j.hybadv.2023.100091, 10.1016/j.hybadv.2023.100091, DOI 10.1016/J.HYBADV.2023.100091]"
"   Alnaimy MA, 2021, WATER-SUI, V13, DOI 10.3390/w13162245"
"   Alves ARA, 2022, SCI TOTAL ENVIRON, V838, DOI 10.1016/j.scitotenv.2022.156435"
"   Angon PB, 2024, HELIYON, V10, DOI 10.1016/j.heliyon.2024.e28357"
"   Anirudhan TS, 2011, J ENVIRON SCI, V23, P1989, DOI 10.1016/S1001-0742(10)60515-3"
"   Anonymous, 2005, P1"
"   [Anonymous], 1999, Phytoremediation of Contaminated Soil and Water, DOI [10.1201/9781439822654, DOI 10.1201/9781439822654]"
"   Ariffin N, 2017, MATEC WEB CONF, V97, DOI 10.1051/matecconf/20179701023"
"   Ayangbenro AS, 2017, INT J ENV RES PUB HE, V14, DOI 10.3390/ijerph14010094"
"   Ayilara MS, 2023, FRONT AGRON, V5, DOI 10.3389/fagro.2023.1183691"
"   Bakshe P, 2023, J HAZARD MATER ADV, V10, DOI 10.1016/j.hazadv.2023.100293"
"   Bala S, 2022, TOXICS, V10, DOI 10.3390/toxics10080484"
"   Balali-Mood M, 2021, FRONT PHARMACOL, V12, DOI 10.3389/fphar.2021.643972"
"   Bhandary B, 2013, INT J MOL SCI, V14, P434, DOI 10.3390/ijms14010434"
"   Bhat SA, 2022, CHEMOSPHERE, V303, DOI 10.1016/j.chemosphere.2022.134788"
"   Bhattacharjee C, 2020, Environmental Advances, V2, P100007, DOI [10.1016/j.envadv.2020.100007, 10.1016/j.envadv.2020.100007, DOI 10.1016/J.ENVADV.2020.100007]"
"   Bl??cher C, 2003, WATER RES, V37, P4018, DOI 10.1016/S0043-1354(03)00314-2"
"   Boufekane A, 2019, GROUNDWATER SUST DEV, V8, P179, DOI 10.1016/j.gsd.2018.11.003"
"   Briffa J, 2020, HELIYON, V6, DOI 10.1016/j.heliyon.2020.e04691"
"   Chaney RL, 1997, CURR OPIN BIOTECH, V8, P279, DOI 10.1016/S0958-1669(97)80004-3"
"   Chen HG, 2016, CHEMOSPHERE, V155, P163, DOI 10.1016/j.chemosphere.2016.04.044"
"   Chen HY, 2019, ACS OMEGA, V4, P14105, DOI 10.1021/acsomega.9b02023"
"   Chen S, 2023, SUSTAINABILITY-BASEL, V15, DOI 10.3390/su152215863"
"   Cheng H, 2021, TOXICS, V9, DOI 10.3390/toxics9060142"
"   Chojnacka K, 2010, ENVIRON INT, V36, P299, DOI 10.1016/j.envint.2009.12.001"
"   Chuai Y, 2023, BMC ORAL HEALTH, V23, DOI 10.1186/s12903-023-02929-9"
"   Chwastowski J, 2023, MOLECULES, V28, DOI 10.3390/molecules28176345"
"   Collin MS, 2022, J HAZARD MATER ADV, V7, DOI 10.1016/j.hazadv.2022.100094"
"   Costa OYA, 2018, FRONT MICROBIOL, V9, DOI 10.3389/fmicb.2018.01636"
"   Coxon T, 2019, ENVIRON GEOCHEM HLTH, V41, P783, DOI 10.1007/s10653-018-0172-2"
"   Cunningham SD, 1996, PLANT PHYSIOL, V110, P715, DOI 10.1104/pp.110.3.715"
"   d'Halluin M, 2017, ACS SUSTAIN CHEM ENG, V5, P1965, DOI 10.1021/acssuschemeng.6b02768"
"   Dao PU, 2024, SCI TOTAL ENVIRON, V912, DOI 10.1016/j.scitotenv.2023.169241"
"   Das O, 2022, RENEW SUST ENERG REV, V158, DOI 10.1016/j.rser.2021.112054"
"   Das S, 2023, ENVIRON HEALTH INSIG, V17, DOI 10.1177/11786302231201259"
"   Deng SX, 2024, BIOTECHNOL ADV, V72, DOI 10.1016/j.biotechadv.2024.108337"
"   Deng SB, 2010, WATER RES, V44, P5188, DOI 10.1016/j.watres.2010.06.038"
"   Dev H., 1994, Health Saf. Plan (Revision 2), DOI [10.2172/10108753, DOI 10.2172/10108753]"
"   Devi B. D., 2012, Journal of Microbiology and Biotechnology Research, V2, P727"
"   Dias MA, 2001, APPL BIOCHEM BIOTECH, V91-3, P413, DOI 10.1385/ABAB:91-93:1-9:413"
"   Diep P, 2018, FRONT BIOENG BIOTECH, V6, DOI 10.3389/fbioe.2018.00157"
"   Ding BB, 2024, J HYDROL, V632, DOI 10.1016/j.jhydrol.2024.130761"
"   Dixit R, 2015, SUSTAINABILITY-BASEL, V7, P2189, DOI 10.3390/su7022189"
"   Djedidi Z, 2009, J HAZARD MATER, V172, P1372, DOI 10.1016/j.jhazmat.2009.07.144"
"   Duwiejuah AB, 2020, J HEALTH POLLUT, V10"
"   El-Naggar NE, 2020, SCI REP-UK, V10, DOI 10.1038/s41598-020-70473-5"
"   Elbasiouny H, 2021, ENVIRON MONIT ASSESS, V193, DOI 10.1007/s10661-021-09236-2"
"   Elgarahy AM, 2024, J CLEAN PROD, V451, DOI 10.1016/j.jclepro.2024.141974"
"   Ethaib S, 2022, ENVIRONMENTS, V9, DOI 10.3390/environments9100123"
"   Fashola MO, 2016, INT J ENV RES PUB HE, V13, DOI 10.3390/ijerph13111047"
"   Ferronato N, 2019, INT J ENV RES PUB HE, V16, DOI 10.3390/ijerph16061060"
"   Freitas H, 2004, ENVIRON INT, V30, P65, DOI 10.1016/S0160-4120(03)00149-1"
"   Fu FL, 2011, J ENVIRON MANAGE, V92, P407, DOI 10.1016/j.jenvman.2010.11.011"
"   Genchi G, 2020, INT J ENV RES PUB HE, V17, DOI 10.3390/ijerph17030679"
"   Gill EZ, 2021, SUSTAIN ENERGY TECHN, V47, DOI 10.1016/j.seta.2021.101321"
"   Guo BR, 2024, ACS APPL MATER INTER, V16, P8939, DOI 10.1021/acsami.3c19077"
"   Haghighizadeh A, 2024, ARAB J CHEM, V17, DOI 10.1016/j.arabjc.2024.105777"
"   Han J, 2016, J LIPID RES, V57, P1329, DOI 10.1194/jlr.R067595"
"   He X, 2023, CARBON, V213, DOI 10.1016/j.carbon.2023.118200"
"   Henson MW, 2015, PEERJ, V3, DOI 10.7717/peerj.1395"
"   Hu SG, 2021, J HAZARD MATER, V405, DOI 10.1016/j.jhazmat.2020.124072"
"   Humphries AC, 2005, BIOTECHNOL BIOENG, V90, P589, DOI 10.1002/bit.20450"
"   Ibrahim ASS, 2012, EXTREMOPHILES, V16, P659, DOI 10.1007/s00792-012-0464-x"
"   Iddrisu L, 2024, FOODS, V13, DOI 10.3390/foods13060853"
"   Islam MM, 2024, RSC SUSTAIN, V2, P1269, DOI 10.1039/d3su00440f"
"   Jacob JM, 2018, J ENVIRON MANAGE, V217, P56, DOI 10.1016/j.jenvman.2018.03.077"
"   Jain M, 2023, WASTE MANAGE B, V1, P34, DOI [10.1016/j.wmb.2023.06.004, 10.1016/j.wmb.2023.06.004]"
"   Jaishankar Monisha, 2014, Interdiscip Toxicol, V7, P60, DOI 10.2478/intox-2014-0009"
"   Jakubowicz P, 2022, SUSTAINABILITY-BASEL, V14, DOI 10.3390/su14031105"
"   Jaradat AQ, 2021, WATER SCI TECHNOL, V84, P3817, DOI 10.2166/wst.2021.493"
"   Jardine CG, 2003, J TOXICOL ENV HEAL B, V6, P569, DOI 10.1080/10937400390208608"
"   Jena AB, 2023, BIOMED PHARMACOTHER, V162, DOI 10.1016/j.biopha.2023.114606"
"   Johnson-Arbor K, 2021, HEALTH SCI REP-US, V4, DOI 10.1002/hsr2.293"
"   Jomova K, 2023, ARCH TOXICOL, V97, P2499, DOI 10.1007/s00204-023-03562-9"

"   Joutey NT, 2015, REV ENVIRON CONTAM T, V233, P45, DOI 10.1007/978-3-319-10479-9_2"
"   Kafle A, 2022, Environmental Advances, V8, P100203, DOI [10.1016/j.envadv.2022.100203, 10.1016/j.envadv.2022.100203, DOI 10.1016/J.ENVADV.2022.100203, 10.1016/J.ENVADV.2022.100203]"
"   Kathren R.L., 1996, Annual report, DOI [10.2172/770672, DOI 10.2172/770672]"
"   Kehrer JP, 2000, TOXICOLOGY, V149, P43, DOI 10.1016/S0300-483X(00)00231-6"
"   Khulbe KC, 2018, APPL WATER SCI, V8, DOI 10.1007/s13201-018-0661-6"
"   Kubrak OI, 2010, COMP BIOCHEM PHYS C, V152, P360, DOI 10.1016/j.cbpc.2010.06.003"
"   Kumar M, 2021, ENVIRON SUSTAIN IND, V12, DOI 10.1016/j.indic.2021.100155"
"   Kumar MS, 2013, B ENVIRON CONTAM TOX, V90, P730, DOI 10.1007/s00128-013-0984-9"
"   Kumar S, 2020, ENVIRON MONIT ASSESS, V192, DOI 10.1007/s10661-020-8181-x"
"   Lan MM, 2020, INT J ENV RES PUB HE, V17, DOI 10.3390/ijerph17051661"
"   Li KJ, 2023, J CARDIOVASC DEV DIS, V10, DOI 10.3390/jcdd10040171"
"   Li RQ, 2020, SCI TOTAL ENVIRON, V704, DOI 10.1016/j.scitotenv.2019.135284"
"   Liang A, 2024, FOOD CHEM, V449, DOI 10.1016/j.foodchem.2024.139238"
"   Lindman A., 2017, Towards Sustainability in Nordic Mining, DOI [10.6027/TN2016-560, DOI 10.6027/TN2016-560]"
"   Liu CX, 2020, ECOTOX ENVIRON SAFE, V190, DOI 10.1016/j.ecoenv.2019.110073"
"   Liu JL, 2024, J ENVIRON MANAGE, V359, DOI 10.1016/j.jenvman.2024.121003"
"   Liu LW, 2018, SCI TOTAL ENVIRON, V633, P206, DOI 10.1016/j.scitotenv.2018.03.161"
"   Magalhaes M. C. F., 2007, THERMODYNAMICS SOLUB, P327, DOI DOI 10.1016/B978-044452707-3/50020-3"
"   Mahe R, 2024, REV FISH SCI AQUAC, V32, P505, DOI 10.1080/23308249.2024.2347627"
"   Mandal M, 2022, ADV REDOX RES, V5, DOI 10.1016/j.arres.2022.100039"
"   Manjunatha BR, 2001, ENVIRON GEOL, V40, P1462"
"   Martemucci G, 2022, OXYGEN-BASEL, V2, P48, DOI [10.3390/oxygen2020006, 10.3390/oxygen2020006]"
"   Martin T.B., 2004, REMEDIATION J, V14, P35, DOI DOI 10.1002/REM.20011"
"   Matovic V, 2015, FOOD CHEM TOXICOL, V78, P130, DOI [10.1016/jict.2015.02.011, 10.1016/j.fct.2015.02.011]"
"   McLaren RG, 2005, AUST J SOIL RES, V43, P159, DOI 10.1071/SR04109"
"   Mehrpooya M, 2021, RENEW ENERG, V172, P1314, DOI 10.1016/j.renene.2021.03.111"
"   Mishra A, 2015, INT J ENVIRON SCI TE, V12, P1415, DOI 10.1007/s13762-014-0516-0"
"   Mitra S, 2022, J KING SAUD UNIV SCI, V34, DOI 10.1016/j.jksus.2022.101865"
"   Mocek-Pl??ciniak A, 2023, PLANTS-BASEL, V12, DOI 10.3390/plants12081653"
"   Mohanty B, 2023, AQUA-UK, V72, P851, DOI 10.2166/aqua.2023.122"
"   Morales ME, 2016, PLOS ONE, V11, DOI 10.1371/journal.pone.0151367"
"   Naja GM, 2009, ADV IND HAZARD WASTE, P247"
"   Nedjimi B, 2021, SN APPL SCI, V3, DOI 10.1007/s42452-021-04301-4"
"   Nita M, 2016, OXID MED CELL LONGEV, V2016, DOI 10.1155/2016/3164734"
"   Nong XY, 2020, B ENVIRON CONTAM TOX, V105, P283, DOI 10.1007/s00128-020-02940-8"
"   Nzihou A, 2013, J HAZARD MATER, V256, P56, DOI 10.1016/j.jhazmat.2013.02.050"
"   Ojuederie OB, 2017, INT J ENV RES PUB HE, V14, DOI 10.3390/ijerph14121504"
"   Pandit G.K., 2023, Bentham Science Publishers EBooks, P203, DOI [10.2174/9789815123739123010013, DOI 10.2174/9789815123739123010013]"
"   Perumal S, 2022, CHEMOSPHERE, V286, DOI 10.1016/j.chemosphere.2021.131760"
"   Poland CA, 2021, TOXICOLOGY, V463, DOI 10.1016/j.tox.2021.152969"
"   Popescu SM, 2024, FRONT ENV SCI-SWITZ, V12, DOI 10.3389/fenvs.2024.1336088"
"   Popoola LT, 2023, HELIYON, V9, DOI 10.1016/j.heliyon.2023.e19788"
"   Pritchard DL, 2010, WATER SCI TECHNOL, V62, P48, DOI 10.2166/wst.2010.274"
"   Priya AK, 2023, TOXICS, V11, DOI 10.3390/toxics11050422"
"   Qasem NAA, 2021, NPJ CLEAN WATER, V4, DOI 10.1038/s41545-021-00127-0"
"   Qu F, 2024, TOXICS, V12, DOI 10.3390/toxics12060388"
"   Rahimzadeh MR, 2017, CASP J INTERN MED, V8, P135, DOI 10.22088/cjim.8.3.135"
"   Rahman Z, 2019, ENVIRON MONIT ASSESS, V191, DOI 10.1007/s10661-019-7528-7"
"   Rai PK, 2008, INT J PHYTOREMEDIAT, V10, P133, DOI 10.1080/15226510801913918"
"   Rajendran S, 2022, CHEMOSPHERE, V287, DOI 10.1016/j.chemosphere.2021.132369"
"   Raklami A, 2022, INT J MOL SCI, V23, DOI 10.3390/ijms23095031"
"   Rashid A, 2023, AGRONOMY-BASEL, V13, DOI 10.3390/agronomy13061521"
"   Ravenscroft P., 2011, Arsenic Pollution"
"   Ray PD, 2014, FRONT GENET, V5, DOI 10.3389/fgene.2014.00201"
"   Renu K, 2021, CHEMOSPHERE, V271, DOI 10.1016/j.chemosphere.2021.129735"
"   Robins G, 2011, PUBLIC ADMIN, V89, P1293, DOI 10.1111/j.1467-9299.2010.01884.x"
"   Romantschuk M, 2023, FRONT MICROBIOL, V14, DOI 10.3389/fmicb.2023.1258148"
"   Sabreena, 2022, PLANTS-BASEL, V11, DOI 10.3390/plants11091255"
"   Sasakova N, 2018, FRONT SUSTAIN FOOD S, V2, DOI 10.3389/fsufs.2018.00042"
"   Shahane AA, 2021, FRONT AGRON, V3, DOI 10.3389/fagro.2021.680456"
"   Sharma JK, 2023, FRONT PLANT SCI, V14, DOI 10.3389/fpls.2023.1076876"
"   Shen Q, 2023, FRONT PHARMACOL, V14, DOI 10.3389/fphar.2023.1220983"
"   Shi XL, 2024, COLLOID SURFACE A, V694, DOI 10.1016/j.colsurfa.2024.134160"
"   Silva EA, 2003, BRAZ J CHEM ENG, V20, P213, DOI 10.1590/S0104-66322003000300002"
"   Singh A, 2024, J ENVIRON CHEM ENG, V12, DOI 10.1016/j.jece.2024.113032"
"   Singh N, 2022, LIFE-BASEL, V12, DOI 10.3390/life12122094"
"   Singh R, 2011, INDIAN J PHARMACOL, V43, P246, DOI 10.4103/0253-7613.81505"
"   Singh V, 2024, NANOCOMPOSITES, V10, P214, DOI 10.1080/20550324.2024.2347804"
"   Singh V, 2024, SCI REP-UK, V14, DOI 10.1038/s41598-024-58061-3"
"   Singh V, 2024, BIOREMEDIAT J, V28, P245, DOI 10.1080/10889868.2023.2269188"
"   Singh V, 2023, GREEN CHEM LETT REV, V16, DOI 10.1080/17518253.2023.2267079"
"   Singh V, 2023, TOXICS, V11, DOI 10.3390/toxics11020147"
"   Singh V, 2022, ANTIOXIDANTS-BASEL, V11, DOI 10.3390/antiox11122375"
"   Singh V, 2023, BIOMETALS, V36, P829, DOI 10.1007/s10534-022-00476-4"
"   Singh V, 2022, SEP SCI TECHNOL, V57, P1609, DOI 10.1080/01496395.2021.1993921"
"   Singh V, 2021, J ENVIRON CHEM ENG, V9, DOI 10.1016/j.jece.2021.106279"
"   Singh V, 2021, J ENVIRON CHEM ENG, V9, DOI 10.1016/j.jece.2021.105124"
"   Singh V, 2020, DESALIN WATER TREAT, V197, P300, DOI 10.5004/dwt.2020.25996"
"   Soltanian S, 2022, RENEW SUST ENERG REV, V156, DOI 10.1016/j.rser.2021.111975"
"   Song B, 2017, ENVIRON INT, V105, P43, DOI 10.1016/j.envint.2017.05.001"
"   Srivastava V, 2017, FRONT ENV SCI-SWITZ, V5, DOI 10.3389/fenvs.2017.00064"

"   Stancheva M., 2017, CASE STUDY 3 BURGAS:LAND-SEA INTERACTIONS Report on the Project Cross Border Maritime Spatial Planning in the Black Sea-Romania and Bulgaria (MARSPLAN-BS Project), DOI [10.7546/io.lsi.17.06.22, DOI 10.7546/IO.LSI.17.06.22]"
"   Suman J, 2018, FRONT PLANT SCI, V9, DOI 10.3389/fpls.2018.01476"
"   Sun QY, 2022, TOXICOLOGY, V469, DOI 10.1016/j.tox.2022.153136"
"   Tamm L, 2022, AGRONOMY-BASEL, V12, DOI 10.3390/agronomy12030673"
"   Tchounwou Paul B, 2012, Exp Suppl, V101, P133, DOI 10.1007/978-3-7643-8340-4_6"
"   Tekerlekopoulou AG, 2013, WATER RES, V47, P623, DOI 10.1016/j.watres.2012.10.034"
"   Tian F, 2024, ROCK MECH ROCK ENG, V57, P4835, DOI 10.1007/s00603-024-03788-8"
"   Tilman D, 2011, P NATL ACAD SCI USA, V108, P20260, DOI 10.1073/pnas.1116437108"
"   Timkov?? I, 2018, SEPARATIONS, V5, DOI 10.3390/separations5040054"
"   Tripathi DurgeshKuman., 2014, Improvement Of Crops In The Era Of Climatic Changes, V2, P197, DOI DOI 10.1007/978-1-4614-8824-8_8"
"   Trujillo-Gonz??lez JM, 2017, AGRICULTURE-BASEL, V7, DOI 10.3390/agriculture7070052"
"   Tsezos M, 1997, COMP BIOCHEM PHYS A, V118, P481, DOI 10.1016/S0300-9629(97)00009-1"
"   Udayanga WDC, 2018, FUEL, V226, P721, DOI 10.1016/j.fuel.2018.04.045"
"   Ugrina M, 2023, PROCESSES, V11, DOI 10.3390/pr11123270"
"   Vardhan KH, 2019, J MOL LIQ, V290, DOI 10.1016/j.molliq.2019.111197"
"   Verma Neelam, 2017, Recent Pat Biotechnol, V11, P171, DOI [10.2174/1872208311666170111111631, 10.2174/1872208311666170111111631]"
"   Violante A, 2010, J SOIL SCI PLANT NUT, V10, P268, DOI 10.4067/S0718-95162010000100005"
"   Vocciante M, 2021, SUSTAINABILITY-BASEL, V13, DOI 10.3390/su13020770"
"   Volesky B, 2007, WATER RES, V41, P4017, DOI 10.1016/j.watres.2007.05.062"
"   VS A.K., 2021, J. Adv. Sci. Res, V12, P255"
"   Wai KM, 2017, INT J ENV RES PUB HE, V14, DOI 10.3390/ijerph14111339"
"   Wang YL, 2024, EUR J MED RES, V29, DOI 10.1186/s40001-024-01681-2"
"   Weggler K, 2004, J ENVIRON QUAL, V33, P496, DOI 10.2134/jeq2004.0496"
"   White PJ, 2010, ANN BOT-LONDON, V105, P1073, DOI 10.1093/aob/mcq085"
"   Williamson B, 2014, PUBLIC POLICY ADMIN, V29, P292, DOI 10.1177/0952076714529139"
"   Witkowska D, 2021, MOLECULES, V26, DOI 10.3390/molecules26196060"
"   Wolowiec M, 2019, MINERALS-BASEL, V9, DOI 10.3390/min9080487"
"   Yadati T, 2020, CELLS-BASEL, V9, DOI 10.3390/cells9071679"
"   Yadav N, 2024, J PURE APPL MICROBIO, V18, P823, DOI 10.22207/JPAM.18.2.44"
"   Yan A, 2020, FRONT PLANT SCI, V11, DOI 10.3389/fpls.2020.00359"
"   Yan GW, 2023, FRONT ENV SCI-SWITZ, V11, DOI 10.3389/fenvs.2023.1131204"
"   Yang QW, 2022, ECOTOX ENVIRON SAFE, V241, DOI 10.1016/j.ecoenv.2022.113716"
"   Yilmazer P, 2009, J CHEM TECHNOL BIOT, V84, P604, DOI 10.1002/jctb.2088"
"   Yuan Z, 2022, INT J ENV RES PUB HE, V19, DOI 10.3390/ijerph19116572"
"   Zaynab M, 2022, J KING SAUD UNIV SCI, V34, DOI 10.1016/j.jksus.2021.101653"
"   Zeng X, 2023, TOXICS, V11, DOI 10.3390/toxics11030249"
"   Zeweld W, 2020, LAND USE POLICY, V95, DOI 10.1016/j.landusepol.2019.01.002"
"   Zhang FS, 2012, INT J ENV RES PUB HE, V9, P2658, DOI 10.3390/ijerph9082658"
"   Zhang PB, 2023, J ENVIRON CHEM ENG, V11, DOI 10.1016/j.jece.2023.109658"
"   Zhang P, 2023, TOXICS, V11, DOI 10.3390/toxics11100828"
"   Zhang WQ, 2020, SCI REP-UK, V10, DOI 10.1038/s41598-020-73570-7"
"   Zhang WH, 2023, EUR J MED RES, V28, DOI 10.1186/s40001-023-01247-8"
"   Zhen WY, 2024, IMETA, V3, DOI 10.1002/imt2.197"
"   Zhou XX, 2021, SEMIN CANCER BIOL, V76, P86, DOI 10.1016/j.semcancer.2021.05.009"
"   Zhou XX, 2016, ONCOTARGET, V7, P80482, DOI 10.18632/oncotarget.12613"
NR 214
TC 81
Z9 89
U1 103
U2 344
PU TAYLOR & FRANCIS LTD
PI ABINGDON
"PA 2-4 PARK SQUARE, MILTON PARK, ABINGDON OR14 4RN, OXON, the United Kingdom"
SN 1751-8253
EI 1751-7192
J9 GREEN CHEM LETT REV
JI Green Chem. Lett. Rev.
PD DEC 31
PY 2024
VL 17
IS 1
AR 2404235
DI 10.1080/17518253.2024.2404235
PG 25
"WC Chemistry, Multidisciplinary; Green & Sustainable Science & Technology"
WE Science Citation Index Expanded (SCI-EXPANDED)
SC Chemistry; Science & Technology - Other Topics
GA G3M2W
UT WOS:001315711100001
"OA Green Submitted, gold"
DA 2026-04-06
ER

PT J
"AU Moshood, AY"
"   Abdulraheem, MI"
"   Li, LZ"
"   Zhang, YY"
"   Zhang, W"
"   Gen, K"

"   Zang, YH"
"   Raghavan, V"
"   Hu, JD"
"AF Moshood, Abiodun Yusuff"
"   Abdulraheem, Mukhtar Iderawumi"
"   Li, Linze"
"   Zhang, Yanyan"
"   Zhang, Wei"
"   Gen, Kuan"
"   Zang, Yiheng"
"   Raghavan, Vijaya"
"   Hu, Jiandong"
TI Sustainable forest soil management for remediation of heavy metals
   contamination: integrating technologies and ecosystem health
SO ANTONIE VAN LEEUWENHOEK INTERNATIONAL JOURNAL OF GENERAL AND MOLECULAR
   MICROBIOLOGY
LA English
DT Review
DE Forest soil management; Heavy metals; Environmental remediation; Soil
   contamination; Phytoremediation; In-situ immobilization
ID BIOCHAR APPLICATION; PB IMMOBILIZATION; RESEARCH PROGRESS; TREE GROWTH;
   POLLUTION; PHYTOREMEDIATION; PRECIPITATION; ACCUMULATION; ADSORPTION;
   PHOSPHATE
"AB Due to its persistence and non-biodegradability, heavy metal pollution in forest soils poses an ecological disaster and a severe environmental concern, necessitating sustainable mitigation solutions. Forest soils exhibit physicochemical and biological properties, such as high organic matter content, abundant clay minerals, and diverse microbial interactions, which facilitate key processes such as adsorption, precipitation, and microbial-mediated immobilization of heavy metals. These attributes collectively enhance their capacity for sustainable remediation. Despite extensive research efforts in environmental remediation, there remains a gap in synthesizing the diverse approaches and technologies used for addressing forest soil contamination by heavy metals. Hence, this review aims to bridge this gap by consolidating existing knowledge and highlighting areas that require further investigation by providing an overview of research progress, prospects, and trends in soil remediation of heavy metal contamination. We discuss various remediation methods (e.g., phytoremediation, microbial interactions, and soil amendments), evaluating their effectiveness in reducing heavy metal concentrations and their impact on ecosystem health. A detailed analysis of these methods is presented, including their limitations, applicability, and the need to consider site-specific factors, cost-effectiveness, sustainability, and long-term efficacy. Additionally, we stress the importance of continuous innovation and collaboration among researchers, policymakers, and industry stakeholders to develop multidisciplinary, technology-driven solutions for restoring contaminated soils and safeguarding human and ecosystem health. By integrating multidisciplinary approaches and leveraging emerging technologies, sustainable solutions can be developed to restore contaminated soils and protect human health and ecosystems. Future research should focus on exploring the long-term impacts of forest soil management practices on heavy metal bioavailability and mobility, as well as optimizing existing techniques, exploring novel approaches, assessing ecosystem responses, and integrating forest soil management into broader environmental restoration strategies."
"C1 [Moshood, Abiodun Yusuff; Abdulraheem, Mukhtar Iderawumi; Li, Linze; Zhang, Yanyan; Zhang, Wei; Gen, Kuan; Zang, Yiheng; Hu, Jiandong] Henan Agr Univ, Dept Elect Engn, Zhengzhou 450002, Peoples R China."
"   [Moshood, Abiodun Yusuff; Abdulraheem, Mukhtar Iderawumi; Li, Linze; Zhang, Yanyan; Zhang, Wei; Gen, Kuan; Zang, Yiheng; Hu, Jiandong] Henan Int Joint Lab Laser Technol Agr Sci, Zhengzhou 450002, Peoples R China."
"   [Moshood, Abiodun Yusuff; Abdulraheem, Mukhtar Iderawumi; Li, Linze; Zhang, Wei; Gen, Kuan; Zang, Yiheng; Hu, Jiandong] State Key Lab Wheat & Maize Crop Sci, Zhengzhou 450002, Peoples R China."
"   [Raghavan, Vijaya] McGill Univ, Fac Agr & Environm Studies, Dept Bioresource Engn, Sainte Anne De Bellevue, PQ H9X 3V9, Canada."
C3 Henan Agricultural University; McGill University
"RP Abdulraheem, MI; Hu, JD (corresponding author), Henan Agr Univ, Dept Elect Engn, Zhengzhou 450002, Peoples R China."
EM yusufbinmasud01@stu.henau.edu.cn; abdulraheem@stu.henau.edu.cn;
   lilinze@henau.edu.cn; zyanyan0923@henau.edu.cn; wz@henau.edu.cn;
   kuankuan0208@163.com; yzang@stu.henau.edu.cn; vijaya.raghavan@mcgill.ca;
   jdhu@henau.edu.cn
"RI HU, Jiandong/JJC-4051-2023; Abdulraheem, Mukhtar Iderawumi/AAV-1818-2021"
"OI Abdulraheem, Mukhtar Iderawumi/0000-0002-6745-378X"
FU Key Technologies R & D Program of Henan Province; Major Science and
   Technology projects of Henan Province [221100320200]; Henan Center for
   Outstanding Overseas Scientists [GZS2021007]; [221111320700]
FX This research was funded by the Key Technologies R & D Program of Henan
"   Province (221111320700), Major Science and Technology projects of Henan"
   Province (221100320200) and supported by Henan Center for Outstanding
   Overseas Scientists (GZS2021007)
"CR Abdulraheem MI, 2024, AGRICULTURE-BASEL, V14, DOI 10.3390/agriculture14070976"
"   Abdulraheem MI, 2024, PLANTS-BASEL, V13, DOI 10.3390/plants13020163"
"   Adnan M, 2025, SUSTAIN HORIZ, V14, DOI 10.1016/j.horiz.2024.100126"
"   Adnan M, 2022, TOXICS, V10, DOI 10.3390/toxics10050231"
"   Ahmad A, 2022, CHEMOSPHERE, V306, DOI 10.1016/j.chemosphere.2022.135634"
"   Ahmed S, 2022, PLANTA, V255, DOI 10.1007/s00425-022-03903-5"
"   Alengebawy A, 2021, TOXICS, V9, DOI 10.3390/toxics9030042"
"   Alloway B.J., 2013, Heavy Metals in Soils, P11, DOI [10.1007/978-94-007-4470-7-2, DOI 10.1007/978-94-007-4470-7-2, 10.1002/9781119438922.ch18, DOI 10.1007/978-94-007-4470-72]"
"   Angon PB, 2024, HELIYON, V10, DOI 10.1016/j.heliyon.2024.e28357"
"   Animashaun IM, 2016, International Journal of Scientific Research in Environmental Sciences, V4, P240, DOI [10.12983/ijsres-2016-p0240-0246, 10.12983/ijsres-2016-p0240-0246, DOI 10.12983/IJSRES-2016-P0240-0246]"
"   [Anonymous], 2012, Bio-Geo interactions in metal-contaminated soils, DOI DOI 10.1007/978-3-642-23327-210"
"   Arora B, 2020, J COMPOS SCI, V4, DOI 10.3390/jcs4030135"

"   Asad SA, 2019, CHEMOSPHERE, V217, P925, DOI 10.1016/j.chemosphere.2018.11.021"
"   Ashwood F, 2018, EUR J SOIL BIOL, V87, P46, DOI 10.1016/j.ejsobi.2018.05.003"
"   Awasthi G, 2022, MINERALS-BASEL, V12, DOI 10.3390/min12010085"
"   Bakshi S, 2018, BURL DODDS AGR SCI, V49, P63, DOI 10.19103/AS.2017.0033.20"
"   Ballesteros D, 2020, FORESTS, V11, DOI 10.3390/f11121281"
"   Belaicha N, 2014, PROCEDIA ENGINEER, V83, P403, DOI 10.1016/j.proeng.2014.09.048"
"   Bhandary S., 2025, Forest fungi nanotechnology and their applications Forest Fungi, P489"
"   Bhantana P, 2021, SYMBIOSIS, V84, P19, DOI 10.1007/s13199-021-00756-6"
"   Bhat SA, 2022, CHEMOSPHERE, V303, DOI 10.1016/j.chemosphere.2022.134788"
"   Bhatia A, 2015, WATER ENVIRON RES, V87, P2027, DOI 10.2175/106143015X14362865226833"
"   Brown R., 2012, Biochar for environmental management, P159, DOI DOI 10.4324/9781849770552-15/BIOCHAR-PRODUCTIONTECHNOLOGY-ROBERT-BROWN"
"   Bruckman VJ, 2019, DEV SOIL SCI, V36, P427, DOI 10.1016/B978-0-444-63998-1.00017-3"
"   Brunner I, 2008, ENVIRON POLLUT, V152, P559, DOI 10.1016/j.envpol.2007.07.006"
"   Bykova MV, 2021, ENVIRON GEOCHEM HLTH, V43, P2331, DOI 10.1007/s10653-020-00802-0"
"   Chalise D, 2019, SOIL SYST, V3, DOI 10.3390/soilsystems3010012"
"   Chamba-Eras I, 2022, PLANTS-BASEL, V11, DOI 10.3390/plants11091186"
"   Chen LH, 2024, EARTH SYST SCI DATA, V16, P1543, DOI 10.5194/essd-16-1543-2024"
"   Chojnacka K, 2010, ENVIRON INT, V36, P299, DOI 10.1016/j.envint.2009.12.001"
"   Chowardhara Bhaben, 2024, Chemosphere, V359, P142178, DOI [10.1016/j.chemosphere.2024.142178, 10.1016/j.chemosphere.2024.142178]"
"   Chrastny V, 2012, ENVIRON MONIT ASSESS, V184, P2517, DOI 10.1007/s10661-011-2135-2"
"   Cui JL, 2021, SCI TOTAL ENVIRON, V767, DOI 10.1016/j.scitotenv.2021.145021"
"   Cui SZ, 2023, ENVIRON SCI POLLUT R, V30, P85198, DOI 10.1007/s11356-023-28448-2"
"   D'Auria M, 2021, FORESTS, V12, DOI 10.3390/f12010073"
"   Dagdag O., 2023, HEAVY METAL ENV MANA, V2023, P3, DOI [DOI 10.1021/BK-2023-1456.CH001, 10.1021/bk-2023-1456.ch001.ACSSymposiumSeries]"
"   Aparicio JD, 2022, J ENVIRON CHEM ENG, V10, DOI 10.1016/j.jece.2022.107141"
"   Desai M, 2019, SCI TOTAL ENVIRON, V656, P670, DOI 10.1016/j.scitotenv.2018.11.327"
"   Dhanapal AR, 2024, ACS OMEGA, V9, P13522, DOI 10.1021/acsomega.3c09776"
"   Di XY, 2019, CATENA, V174, P308, DOI 10.1016/j.catena.2018.11.028"
"   Dippong T, 2022, ECOL INDIC, V143, DOI 10.1016/j.ecolind.2022.109347"
"   Drake JA, 2015, SCI TOTAL ENVIRON, V514, P359, DOI 10.1016/j.scitotenv.2015.02.012"
"   Du BY, 2020, SCI TOTAL ENVIRON, V720, DOI 10.1016/j.scitotenv.2020.137585"
"   Du CC, 2024, GLOBAL PLANET CHANGE, V243, DOI 10.1016/j.gloplacha.2024.104602"
"   Emurotu J. E., 2017, Environmental Systems Research, V6, DOI [10.1186/s40068-017-0098-1, 10.1186/s40068-017-0098-1]"
"   Gadegaonkar SS, 2023, J WATER PROCESS ENG, V53, DOI 10.1016/j.jwpe.2023.103788"
"   Gardiner ES, 2018, FORESTS, V9, DOI 10.3390/f9110693"
"   G??mez L, 2019, ADV BOT RES, V89, P281, DOI 10.1016/bs.abr.2018.11.010"
"   Gong YY, 2018, WATER RES, V147, P440, DOI 10.1016/j.watres.2018.10.024"
"   Gong ZY, 2021, NANOMATERIALS-BASEL, V11, DOI 10.3390/nano11071792"
"   Henao SG, 2021, FRONT ENV SCI-SWITZ, V9, DOI 10.3389/fenvs.2021.604216"
"   Gourkhede PH, 2025, POL J ENVIRON STUD, DOI 10.15244/pjoes/203051"
"   Gufwan LA, 2025, ARCH AGRON SOIL SCI, V71, P1, DOI 10.1080/03650340.2025.2508763"
"   Guo FY, 2018, ECOTOX ENVIRON SAFE, V161, P164, DOI 10.1016/j.ecoenv.2018.05.088"
"   Han YH, 2024, SCI TOTAL ENVIRON, V926, DOI 10.1016/j.scitotenv.2024.171922"
"   Hassanzadeh Moghimi O., 2022, Environ. Energy Econ. Res, V6, P1"
"   Hauptvogl M, 2020, POL J ENVIRON STUD, V29, P505, DOI 10.15244/pjoes/101621"
"   Helmisaari HS, 2007, J ENVIRON QUAL, V36, P1145, DOI 10.2134/jeq2006.0319"
"   Hernandez L, 2003, SCI TOTAL ENVIRON, V312, P195, DOI 10.1016/S0048-9697(03)00223-7"
"   Herraiz-Carbon M, 2024, SCI TOTAL ENVIRON, V926, DOI 10.1016/j.scitotenv.2024.171848"
"   Hu YC, 2024, SCI TOTAL ENVIRON, V918, DOI 10.1016/j.scitotenv.2024.170577"
"   Huang JQ, 2021, J HAZARD MATER, V416, DOI 10.1016/j.jhazmat.2021.125942"
"   Indu S., 2020, Trace metals in the environment"
"   Isebrands JG, 2014, POPLARS AND WILLOWS: TREES FOR SOCIETY AND THE ENVIRONMENT, P258, DOI 10.1079/9781780641089.0258"
"   Jacob JM, 2018, J ENVIRON MANAGE, V217, P56, DOI 10.1016/j.jenvman.2018.03.077"
"   Janga Jagadeesh Kumar, 2023, Chemosphere, V345, P140476, DOI [10.1016/j.chemosphere.2023.140476, 10.1016/j.chemosphere.2023.140476]"
"   Ji CH, 2021, WATER RES, V189, DOI 10.1016/j.watres.2020.116599"
"   Kang J, 2022, FORESTS, V13, DOI 10.3390/f13060884"
"   Karami E, 2021, MINER ENG, V170, DOI 10.1016/j.mineng.2021.107029"
"   Karna RR, 2017, J ENVIRON QUAL, V46, P1225, DOI 10.2134/jeq2016.10.0387"
"   Kumar V, 2022, Cleaner Chemical Engineering, V2, P100030, DOI [10.1016/j.clce.2022.100030, 10.1016/j.clce.2022.100030, DOI 10.1016/J.CLCE.2022.100030]"
"   Kumpiene J, 2017, CHEMOSPHERE, V184, P13, DOI 10.1016/j.chemosphere.2017.05.140"
"   Kupper T, 2014, WASTE MANAGE, V34, P867, DOI 10.1016/j.wasman.2014.02.007"
"   Larson C, 2014, SCIENCE, V343, P1415, DOI 10.1126/science.343.6178.1415"
"   Li K, 2019, PEERJ, V7, DOI 10.7717/peerj.6631"
"   Li LP, 2022, INT J ENV RES PUB HE, V19, DOI 10.3390/ijerph191610164"
"   Li N, 2019, FORESTS, V10, DOI 10.3390/f10100856"
"   Li Y, 2022, ECOTOX ENVIRON SAFE, V240, DOI 10.1016/j.ecoenv.2022.113701"
"   Li YF, 2018, J SOIL SEDIMENT, V18, P546, DOI 10.1007/s11368-017-1906-y"
"   Li Y, 2019, J CLEAN PROD, V212, P81, DOI 10.1016/j.jclepro.2018.11.294"
"   Li ZG, 2011, APPL GEOCHEM, V26, P160, DOI 10.1016/j.apgeochem.2010.11.014"
"   Lin JJ, 2025, TRAC-TREND ANAL CHEM, V183, DOI 10.1016/j.trac.2024.118082"
"   Liu HY, 2024, FRONT ENV SCI-SWITZ, V12, DOI 10.3389/fenvs.2024.1413047"
"   Liu Lu, 2024, Chemosphere, V366, P143485, DOI [10.1016/j.chemosphere.2024.143485, 10.1016/j.chemosphere.2024.143485]"
"   Liu RL, 2018, FRONT MICROBIOL, V9, DOI 10.3389/fmicb.2018.00041"
"   Liu R, 2016, ENVIRON POLLUT, V210, P174, DOI 10.1016/j.envpol.2015.11.044"
"   Liu ZC, 2020, J HAZARD MATER, V400, DOI 10.1016/j.jhazmat.2020.123138"
"   Lukaszek-Chmielewska A, 2025, SCI REP-UK, V15, DOI 10.1038/s41598-025-88754-2"

"   Luo Q, 2025, RESOUR CONSERV RECY, V215, DOI 10.1016/j.resconrec.2024.108054"
"   OM, 2016, Journal of Bioremediation & Biodegradation, V07, DOI [10.4172/2155-6199.1000334, 10.4172/2155-6199.1000334, DOI 10.4172/2155-6199.1000334, DOI 10.4172/2155-6199.10003342]"
"   Mai XR, 2025, J ENVIRON SCI, V149, P1, DOI 10.1016/j.jes.2024.01.045"
"   Mallampati SR, 2016, ENVIRON PROG SUSTAIN, V35, P1693, DOI 10.1002/ep.12418"
"   Malunguja GK, 2022, ENVIRON PROCESS, V9, DOI 10.1007/s40710-022-00567-x"
"   Mandal RR, 2025, J ENVIRON MANAGE, V375, DOI 10.1016/j.jenvman.2025.124199"
"   Masarovicova E, 2018, Phytoremediation: Management of Environmental Contaminants, P319, DOI [DOI 10.1007/978-3-319-99651-6_14, 10.1007/978-3-319-99651-6_14]"
"   Matovic D, 2011, ENERGY, V36, P2011, DOI 10.1016/j.energy.2010.09.031"
"   McElligott K., 2011, Res Note RMRS-RN-46 Fort Collins, CO US Dep Agric For Serv Rocky Mt Res Stn, V14, P46"
"   Minnikova T, 2022, ENVIRON PROCESS, V9, DOI 10.1007/s40710-022-00604-9"
"   Miranda LS, 2022, J HAZARD MATER, V421, DOI 10.1016/j.jhazmat.2021.126743"
"   Mishra J, 2017, FRONT MICROBIOL, V8, DOI 10.3389/fmicb.2017.01706"
"   MISHRA S., 2019, Environmental biotechnology: For sustainable future, P103, DOI [DOI 10.1007/978-981-10-7284-0_5, DOI 10.1007/978-981-10-7284-05]"
"   Mitra S, 2022, J KING SAUD UNIV SCI, V34, DOI 10.1016/j.jksus.2022.101865"
"   Mohammed A. S., 2011, BIOMANAGEMENT METAL, P1, DOI [10.1007/978-94-007-1914-9_1, DOI 10.1007/978-94-007-1914-9_1]"
"   Molina L, 2021, PLANTS-BASEL, V10, DOI 10.3390/plants10112305"
"   Mosa A, 2023, SUSTAINABILITY-BASEL, V15, DOI 10.3390/su15021206"
"   Moshood AY, 2025, FUNCT INTEGR GENOMIC, V25, DOI 10.1007/s10142-025-01551-y"
"   Nabavi E, 2023, WATER AIR SOIL POLL, V234, DOI 10.1007/s11270-023-06099-w"
"   Nag R, 2022, SCI TOTAL ENVIRON, V802, DOI 10.1016/j.scitotenv.2021.149839"
"   Nsanganwimana F, 2014, J ENVIRON MANAGE, V143, P123, DOI 10.1016/j.jenvman.2014.04.027"
"   O'Connor D, 2018, SCI TOTAL ENVIRON, V619, P815, DOI 10.1016/j.scitotenv.2017.11.132"
"   Osman K.T., 2013, Forest Soils Properties and Management, V1st, DOI [DOI 10.1007/978-3-319-02541-4, 10.1007/978-3-319-02541-4_2, DOI 10.1007/978-3-319-02541-4_2]"
"   Parvez S, 2023, ACS OMEGA, V8, P15990, DOI 10.1021/acsomega.2c07681"
"   Pathak HK, 2024, ENVIRON RES, V255, DOI 10.1016/j.envres.2024.119136"
"   Patil DS, 2016, J ENVIRON CHEM ENG, V4, P468, DOI 10.1016/j.jece.2015.11.028"
"   Paul ALD, 2021, PLANT J, V107, P1040, DOI 10.1111/tpj.15362"
"   Pedron F, 2022, ENVIRONMENTS, V9, DOI 10.3390/environments9110139"
"   Pendke MS, 2025, SCI REP-UK, V15, DOI [10.1038/s41598-025-95116-5, 10.1038/s41598-025-95116-5]"
"   Pracke K, 2022, J SOIL SEDIMENT, V22, P905, DOI 10.1007/s11368-021-03104-9"
"   Pritchard HW, 2014, FOREST ECOL MANAG, V333, P88, DOI 10.1016/j.foreco.2014.08.012"
"   Priya AK, 2023, TOXICS, V11, DOI 10.3390/toxics11050422"
"   Qasem NAA, 2021, NPJ CLEAN WATER, V4, DOI 10.1038/s41545-021-00127-0"
"   Raffa CM, 2021, APPL SCI-BASEL, V11, DOI 10.3390/app11094134"
"   Rahamathulla M, 2021, MATERIALS, V14, DOI 10.3390/ma14216707"
"   Rahman Z, 2021, MATER TODAY-PROC, V37, P2730, DOI 10.1016/j.matpr.2020.08.541"
"   Raklami A, 2022, INT J MOL SCI, V23, DOI 10.3390/ijms23095031"
"   Rashid A, 2023, AGRONOMY-BASEL, V13, DOI 10.3390/agronomy13061521"
"   Rathore S. S., 2019, Proceedings of the Indian National Science Academy Part B Biological Sciences, V89, P419, DOI [10.1007/s40011-017-0885-5, 10.1007/s40011-017-0885-5]"
"   Roshni NA, 2023, ENVIRON PROCESS, V10, DOI 10.1007/s40710-023-00656-5"
"   S??ez JA, 2021, J HAZARD MATER, V402, DOI 10.1016/j.jhazmat.2020.123481"
"   Saha P, 2019, HUM ECOL RISK ASSESS, V25, P966, DOI 10.1080/10807039.2018.1458595"
"   S??nchez-Castro I, 2023, HELIYON, V9, DOI 10.1016/j.heliyon.2023.e16692"
"   Sekar KC, 2024, Geology Ecology and Landscapes, V8, P559, DOI [10.1080/24749508.2022.2163606, DOI 10.1080/24749508.2022.2163606, 10.1080/24749508.2022.2163606]"
"   Selvi A, 2019, FRONT ENV SCI-SWITZ, V7, DOI 10.3389/fenvs.2019.00066"
"   Shah V, 2020, ENVIRON TECHNOL INNO, V18, DOI 10.1016/j.eti.2020.100774"
"   Sharma R., 2021, Contamination of water"
"   Shivhare N., 2019, Res J Chem Environ, V23, P134"
"   Song JM, 2021, J CLEAN PROD, V319, DOI 10.1016/j.jclepro.2021.128644"
"   Song PP, 2022, SCI TOTAL ENVIRON, V838, DOI 10.1016/j.scitotenv.2022.156417"
"   St-Denis A, 2017, APPL SOIL ECOL, V112, P1, DOI 10.1016/j.apsoil.2016.12.008"
"   Stanturf JA., 2024, Restoring forests and trees for sustainable development: policies, practices, impacts, and ways forward"
"   Sun J, 2023, RSC ADV, V13, P9903, DOI 10.1039/d2ra06106f"
"   Tang HM, 2021, ACTA GEOCHIM, V40, P300, DOI 10.1007/s11631-021-00453-7"
"   Tchounwou Paul B, 2012, Exp Suppl, V101, P133, DOI 10.1007/978-3-7643-8340-4_6"
"   Teklegiorgis S, 2024, Geology Ecology and Landscapes, P1, DOI [10.1080/24749508.2024.2373471, 10.1080/24749508.2024.2373471, DOI 10.1080/24749508.2024.2373471]"
"   Thomas SC, 2015, NEW FOREST, V46, P931, DOI 10.1007/s11056-015-9491-7"
"   Tomao A, 2020, FOREST ECOL MANAG, V457, DOI 10.1016/j.foreco.2019.117678"
"   Torres-Cruz TJ, 2018, APPL SOIL ECOL, V131, P66, DOI 10.1016/j.apsoil.2018.08.001"
"   Utermann J, 2019, ECOL STUD-ANAL SYNTH, V237, P199, DOI 10.1007/978-3-030-15734-0_7"
"   Varol M, 2011, J HAZARD MATER, V195, P355, DOI 10.1016/j.jhazmat.2011.08.051"
"   Verma S, 2019, ENVIRON TECHNOL INNO, V14, DOI 10.1016/j.eti.2019.100369"
"   Virkutyte J, 2002, SCI TOTAL ENVIRON, V289, P97, DOI 10.1016/S0048-9697(01)01027-0"
"   Volchko Y, 2017, SCI TOTAL ENVIRON, V605, P300, DOI 10.1016/j.scitotenv.2017.06.128"
"   Wan J, 2016, J HAZARD MATER, V320, P278, DOI 10.1016/j.jhazmat.2016.08.038"
"   Wang YY, 2024, J HAZARD MATER, V465, DOI 10.1016/j.jhazmat.2024.133490"
"   Wei WR, 2022, J HYDRAUL RES, V60, P445, DOI 10.1080/00221686.2021.2004250"
"   Wu XL, 2024, WATER RESOUR RES, V60, DOI 10.1029/2024WR037537"
"   Wuana R.A., 2011, ISRN Ecology, DOI [10.5402/2011/402647, DOI 10.5402/2011/402647]"
"   Xia WY, 2019, CONSTR BUILD MATER, V210, P278, DOI 10.1016/j.conbuildmat.2019.03.195"
"   Xiong YN, 2024, ENVIRON EARTH SCI, V83, DOI 10.1007/s12665-024-11930-6"
"   Yan A, 2020, FRONT PLANT SCI, V11, DOI 10.3389/fpls.2020.00359"
"   Yang Y, 2025, GLOBAL CHANGE BIOL, V31, DOI 10.1111/gcb.70033"
"   Zeng GM, 2017, J HAZARD MATER, V339, P354, DOI 10.1016/j.jhazmat.2017.05.038"

"   Zeng JQ, 2023, CHEM ENG J, V454, DOI 10.1016/j.cej.2022.140307"
"   Zhang LY, 2021, GCB BIOENERGY, V13, P1859, DOI 10.1111/gcbb.12898"
"   Zhang XK, 2013, ENVIRON SCI POLLUT R, V20, P8472, DOI 10.1007/s11356-013-1659-0"
"   Zhao HD, 2022, SCI REP-UK, V12, DOI 10.1038/s41598-022-07602-9"
"   Zou MM, 2021, ENVIRON POLLUT, V280, DOI 10.1016/j.envpol.2021.116965"
NR 167
TC 4
Z9 4
U1 16
U2 18
PU SPRINGER
PI DORDRECHT
"PA VAN GODEWIJCKSTRAAT 30, 3311 GZ DORDRECHT, NETHERLANDS"
SN 0003-6072
EI 1572-9699
J9 ANTON LEEUW INT J G
JI Antonie Van Leeuwenhoek
PD JUN 24
PY 2025
VL 118
IS 7
AR 95
DI 10.1007/s10482-025-02111-x
PG 25
WC Microbiology
WE Science Citation Index Expanded (SCI-EXPANDED)
SC Microbiology
GA 4CA0G
UT WOS:001514418300001
PM 40553236
DA 2026-04-06
ER

PT J
"AU Ma, MY"
"   Ha, Z"
"   Xu, XQ"
"   Lv, CY"
"   Li, CY"
"   Du, DY"
"   Chi, R"
"AF Ma, Mengyu"
"   Ha, Zhihao"
"   Xu, Xiangqun"
"   Lv, Chenyang"
"   Li, Changyi"
"   Du, Dongyun"
"   Chi, Ruan"
TI Simultaneous immobilization of multiple heavy metals in polluted soils amended with mechanical activation waste slag
SO SCIENCE OF THE TOTAL ENVIRONMENT
LA English
DT Article
DE Electrolytic manganese slags; Heavy metals; Adsorption; Soil remediation
ID ZERO-VALENT IRON; REMOVAL; ADSORPTION; AS(III); CD(II); PB(II)
"AB Heavy metal soil contamination has become an increasingly serious problem in industrial development. However, industrial byproducts used for remediation are one aspect of green remediation that can contribute to sustainable practices in waste recycling. In this study, electrolytic manganese slags (EMS) were mechanically activated and modified into a passivator (M-EMS), and the heavy metal adsorption performance of M-EMS, heavy metal passivation ability in soil, dissolved organic matter (DOM) change and its effect on the microbial community structure of soil were investigated. The findings revealed that the maximum adsorption capacities of As(V), Cd2+, Cu2+ and Pb2+ were 76.32 mg/g, 301.41 mg/g, 306.83 mg/g and 826.81 mg/g, respectively, indicating that M-EMS demonstrated remarkable removal performance for different heavy metals. The Langmuir model fits Cd2+, Cu2+ and Pb2+ better than the Freundlich model, and monolayer adsorption is the main process. Surface complexation played a major role in the As (V) adsorption's on the surface of metal oxides in M-EMS. The passivation effect was ranked as Pb > Cr > As>Ni > Cd > Cu, with the highest passivation rate of 97.59 % for Pb, followed by Cr (94.76 %), then As (71.99 %), Ni (65.17 %), Cd (61.44 %), and the worst one was Cu (25.17 %). In conclusion, the passivator has the effect of passivation for each heavy metal. The addition of passivating agent can enhance the diversity of microorganisms. Then it can change the dominant flora and induce the passivation of heavy metals through microorganisms. XRD, FTIR, XPS and the microbial community structure of soil indicated that M-EMS can stabilize heavy metals in contaminated soils through four main mechanisms: ion exchange, electrostatic adsorption, complex precipitation and the microbially induced stabilization. The results of this study may provide new insights into the ecological remediation of multiple heavy-metal-contaminated soils and water bodies and research on the strategy of waste reduction and harmlessness by using EMS-based composites in combination with heavy metals in soil."
"C1 [Ma, Mengyu; Chi, Ruan] Wuhan Inst Technol, Sch Chem & Environm Engn, Hubei Novel Reactor & Green Chem Technol Key Lab, Key Lab Green Chem Proc,Minist Educ, Wuhan 430074, Peoples R China."
"   [Ma, Mengyu; Ha, Zhihao; Xu, Xiangqun; Lv, Chenyang; Li, Changyi; Du, Dongyun] South Cent Minzu Univ, Coll Resources & Environm Sci, Wuhan 430074, Peoples R China."
"   [Ma, Mengyu; Ha, Zhihao; Xu, Xiangqun; Lv, Chenyang; Li, Changyi; Du, Dongyun] South Cent Minzu Univ, Coll Resources & Environm Sci, Engn Res Ctr Heavy Met Pollut Control Hubei Prov, Wuhan 430074, Peoples R China."
C3 Wuhan Institute of Technology; South Central Minzu University; South Central Minzu University

"RP Du, DY (corresponding author), South Cent Minzu Univ, Coll Resources & Environm Sci, Wuhan 430074, Peoples R China."
EM dydu666@mail.scuec.edu.cn
FU Major Innovation Projects of Hubei Province of China [2019ACA156]; Hubei Three Gorges Laboratory Open Fund [SK211010]; Key R amp;D Projects of Hubei Province [2022BCA061]
FX Acknowledgements This study was funded by Major Innovation Projects of
" Hubei Province of China (2019ACA156) , Hubei Three Gorges Laboratory"
" Open Fund (SK211010) ,and the Key R &D Projects of Hubei Province"
(2022BCA061) .
"CR Ananth MV, 1998, J POWER SOURCES, V75, P278, DOI 10.1016/S0378-7753(98)00100-1"
" Han H, 2020, ENVIRON POLLUT, V267, DOI 10.1016/j.envpol.2020.115432"
" Hou SY, 2020, J HAZARD MATER, V382, DOI 10.1016/j.jhazmat.2019.121093"
" Hu XS, 2021, SCI TOTAL ENVIRON, V775, DOI 10.1016/j.scitotenv.2021.144946"
" Huang Y, 2018, J HAZARD MATER, V354, P161, DOI 10.1016/j.jhazmat.2018.05.006"
" Imtiaz M, 2016, J ENVIRON MANAGE, V183, P521, DOI 10.1016/j.jenvman.2016.09.009"
" Jiang M., 2014, China Manganese Ind., V32, P16"
" Jiang M, 2023, CHEM ENG J, V457, DOI 10.1016/j.cej.2022.141182"
" Kakaei S, 2020, J MOL STRUCT, V1199, DOI 10.1016/j.molstruc.2019.126989"
" Khalid M, 2021, PEDOSPHERE, V31, P475, DOI 10.1016/S1002-0160(20)60091-1"
" Khan S, 2021, J HAZARD MATER, V417, DOI 10.1016/j.jhazmat.2021.126039"
" Lan J.-Q., 2006, CHINA MANG IND, V24, P23"
" Lan JR, 2020, CHEMOSPHERE, V252, DOI 10.1016/j.chemosphere.2020.126487"
" Lan JR, 2019, J ALLOY COMPD, V811, DOI 10.1016/j.jallcom.2019.151973"
" Li CX, 2015, J IND ENG CHEM, V23, P344, DOI 10.1016/j.jiec.2014.08.038"
" Li ZT, 2020, ENVIRON POLLUT, V260, DOI 10.1016/j.envpol.2020.114098"
" Li ZT, 2018, J HAZARD MATER, V344, P1, DOI 10.1016/j.jhazmat.2017.09.036"
" Liu K, 2020, ACS SUSTAIN CHEM ENG, V8, P3547, DOI 10.1021/acssuschemeng.9b07055"
" Liu YL, 2020, CHEMOSPHERE, V247, DOI 10.1016/j.chemosphere.2020.125850"
" L??pez EO, 2022, APPL SURF SCI, V571, DOI 10.1016/j.apsusc.2021.151310"
" Ma MY, 2020, SCI TOTAL ENVIRON, V748, DOI 10.1016/j.scitotenv.2020.141490"
" Martinson CA, 2009, J COLLOID INTERF SCI, V336, P406, DOI 10.1016/j.jcis.2009.04.075"
" Ning Y, 2020, FRONT MICROBIOL, V11, DOI 10.3389/fmicb.2020.01665"
" Palmer SJ, 2011, SPECTROCHIM ACTA A, V78, P1633, DOI 10.1016/j.saa.2011.02.019"
" Qin QD, 2011, CHEM ENG J, V172, P68, DOI 10.1016/j.cej.2011.05.066"
" Rauret G, 1999, J ENVIRON MONITOR, V1, P57, DOI 10.1039/a807854h"
" Shu JC, 2018, J TAIWAN INST CHEM E, V82, P351, DOI 10.1016/j.jtice.2017.11.020"
" Sun Y, 2021, J HAZARD MATER, V415, DOI 10.1016/j.jhazmat.2021.125601"
" T??th G, 2016, ENVIRON INT, V88, P299, DOI 10.1016/j.envint.2015.12.017"
" Wan J, 2022, CHEMOSPHERE, V286, DOI 10.1016/j.chemosphere.2021.131643"
" Wan XM, 2020, ENVIRON POLLUT, V261, DOI 10.1016/j.envpol.2020.114157"
" Wang C, 2014, J HAZARD MATER, V268, P124, DOI 10.1016/j.jhazmat.2014.01.009"
" Wang DY, 2022, J HAZARD MATER, V439, DOI 10.1016/j.jhazmat.2022.129693"
" Wang J, 2013, POWDER TECHNOL, V241, P12, DOI 10.1016/j.powtec.2013.03.003"
" Wang MM, 2018, ENVIRON SCI TECHNOL, V52, P13136, DOI 10.1021/acs.est.8b03469"
" Wang P, 2019, SCI TOTAL ENVIRON, V695, DOI 10.1016/j.scitotenv.2019.133893"
" Wu QH, 2022, ENVIRON SCI TECHNOL, V56, P8142, DOI 10.1021/acs.est.2c01393"
" Xiao GX, 2018, FOOD CONTROL, V89, P32, DOI 10.1016/j.foodcont.2018.01.032"
" Yang QQ, 2018, SCI TOTAL ENVIRON, V642, P690, DOI 10.1016/j.scitotenv.2018.06.068"
" Yang ZH, 2022, ENVIRON POLLUT, V312, DOI 10.1016/j.envpol.2022.119953"
" Young G, 2021, CHEMOSPHERE, V271, DOI 10.1016/j.chemosphere.2021.129546"
" Zhai WW, 2020, ENVIRON POLLUT, V258, DOI 10.1016/j.envpol.2019.113790"
" Zhang DF, 2020, ANTON LEEUW INT J G, V113, P1523, DOI 10.1007/s10482-020-01460-z"
" Zhang M, 2013, BIORESOURCE TECHNOL, V130, P457, DOI 10.1016/j.biortech.2012.11.132"
" Zhang YL, 2020, J HAZARD MATER, V390, DOI 10.1016/j.jhazmat.2020.122188"
" Zhang YT, 2023, CHEM ENG J, V457, DOI 10.1016/j.cej.2023.141311"
" Zhao JH, 2016, CHEM ENG J, V304, P737, DOI 10.1016/j.cej.2016.07.003"
" Zhou CB, 2014, J CLEAN PROD, V84, P707, DOI 10.1016/j.jclepro.2014.01.052"
" Zhu J, 2015, J HAZARD MATER, V286, P220, DOI 10.1016/j.jhazmat.2015.01.004"
NR 49
TC 32
Z9 34
U1 14
U2 139
PU ELSEVIER
PI AMSTERDAM
"PA RADARWEG 29, 1043 NX AMSTERDAM, NETHERLANDS"
SN 0048-9697
EI 1879-1026
J9 SCI TOTAL ENVIRON
JI Sci. Total Environ.
PD OCT 10
PY 2023
VL 894
AR 164730
DI 10.1016/j.scitotenv.2023.164730
EA JUN 2023
PG 14
WC Environmental Sciences
WE Science Citation Index Expanded (SCI-EXPANDED)
SC Environmental Sciences & Ecology
GA L7HX7
UT WOS:001024944000001
PM 37308014

DA 2026-04-06
ER

PT J
"AU Naidu, R"
"   Biswas, B"
"   Nuruzzaman, M"
"   Singh, BK"
"AF Naidu, Ravi"
"   Biswas, Bhabananda"
"   Nuruzzaman, Md"
"   Singh, Brajesh Kumar"
TI Bioremediation of heavy metal(loid)s in agricultural soils and crops
SO NATURE REVIEWS BIOENGINEERING
LA English
DT Review
ID CONTAMINATED SOILS; METALS; PHYTOREMEDIATION; NANOPARTICLES;
   ACCUMULATION; CD; PHYTOEXTRACTION; PB; HYPERACCUMULATION; TRANSFORMATION
"AB Heavy metal(loid) contamination in crops threatens the quality and safety of food and fodder. This contamination affects human health, as non-biodegradable heavy metal(loid)s can build up in the body through the food chain and bioaccumulation processes. Although heavy metal(loid)s exist naturally, their displacement and extensive use in chemicals and consumer products heighten the risk of accumulation in agricultural soils and crops. Managing or removing these hazardous materials from agricultural soils is challenging, owing to the large spatial distribution of contaminants, as well as limited technical and management options. However, concerns regarding agricultural soil quality and food safety have led to interest in environmentally friendly methods, such as the bioremediation of agricultural soils contaminated with heavy metal(loid)s. Given the low-to-moderate levels and diffused nature of heavy metal(loid) pollution in agricultural soils, one strategy is to cultivate crops that can tolerate these heavy metal(loid)s. Additionally, scalable bioremediation strategies include the bioengineering of crop varieties that are heavy metal(loid) resistant, and ecosystem-level bioengineering approaches for soil remediation. In this Review, we discuss trans-disciplinary approaches that integrate traditional bioremediation with crop bioengineering, microbiome engineering and nanotechnology to develop effective solutions for heavy metal(loid)-contaminated agricultural soils."
"C1 [Naidu, Ravi; Biswas, Bhabananda; Nuruzzaman, Md] Univ Newcastle, Global Ctr Environm Remediat, Callaghan, NSW, Australia."
"   [Naidu, Ravi; Biswas, Bhabananda; Nuruzzaman, Md] crcCARE Pty Ltd, ATC Bldg, Callaghan, NSW, Australia."
"   [Singh, Brajesh Kumar] Western Sydney Univ, Hawkesbury Inst Environm, Penrith, NSW, Australia."
C3 University of Newcastle; Western Sydney University
"RP Naidu, R (corresponding author), Univ Newcastle, Global Ctr Environm Remediat, Callaghan, NSW, Australia.; Naidu, R (corresponding author), crcCARE Pty Ltd, ATC Bldg, Callaghan, NSW, Australia."
EM Ravi.Naidu@newcastle.edu.au
"RI Biswas, Bhabananda/E-3904-2016; Nuruzzaman/U-3535-2018; Singh, Dr"
   Brajesh/HRB-4433-2023
"OI Biswas, Bhabananda/0000-0001-9473-4141; Nuruzzaman/0000-0002-8403-3867;"
"   Naidu, Ravi/0000-0002-1520-2495; "
FU Alexander von Humboldt Foundation; Australian Research Council (ARC)
"   [DP230102963, DP230101448]"
FX This work was supported by crcCARE's partner organizations. B.B. thanks
   the Alexander von Humboldt Foundation for financing his fellowship. R.N.
   and B.B. thank Australian Research Council (ARC) for the sustainable
   fertilizer project (DP230102963) and B.K.S. thanks ARC for microbial
   science project (DP230101448).
"CR AbdelRahim K, 2017, SAUDI J BIOL SCI, V24, P208, DOI 10.1016/j.sjbs.2016.02.025"
"   Afridi MS, 2024, MICROBIOL RES, V279, DOI 10.1016/j.micres.2023.127564"
"   Ahmed E, 2018, J SAUDI CHEM SOC, V22, P919, DOI 10.1016/j.jscs.2018.02.002"
"   Ahmed F, 2020, AGRICULTURE-BASEL, V10, DOI 10.3390/agriculture10120610"
"   Al-Khayri JM, 2023, PLANTS-BASEL, V12, DOI 10.3390/plants12020292"
"   Alaboudi KA, 2018, ANN AGR SCI-CAIRO, V63, P123, DOI 10.1016/j.aoas.2018.05.007"
"   Ameen F, 2020, J MOL STRUCT, V1202, DOI 10.1016/j.molstruc.2019.127233"
"   Anwar A, 2024, J HAZARD MATER, V468, DOI 10.1016/j.jhazmat.2024.133829"
"   Arora D, 2024, WATER AIR SOIL POLL, V235, DOI 10.1007/s11270-023-06874-9"
"   Atta MI, 2023, PEERJ, V11, DOI 10.7717/peerj.16067"
"   Azad MA, 2014, CHINESE SCI BULL, V59, P703, DOI 10.1007/s11434-013-0058-8"
"   Bai XH, 2024, J HAZARD MATER, V471, DOI 10.1016/j.jhazmat.2024.134370"
"   Ballabio C, 2024, SCI TOTAL ENVIRON, V912, DOI 10.1016/j.scitotenv.2023.168710"
"   Baritz R., 2018, International Yearbook of Soil Law and Policy 2017, P17, DOI DOI 10.1007/978-3-319-68885-5_3"
"   Barr ZK, 2023, PLANTS-BASEL, V12, DOI 10.3390/plants12163015"
"   Bassegio C, 2020, INT J PHYTOREMEDIAT, V22, P134, DOI 10.1080/15226514.2019.1647406"
"   Bhatt P, 2022, J HAZARD MATER, V427, DOI 10.1016/j.jhazmat.2021.128033"
"   Bhatti SS, 2016, SPRINGERPLUS, V5, DOI 10.1186/s40064-016-1777-5"
"   Bravo G, 2020, MICROORGANISMS, V8, DOI 10.3390/microorganisms8121952"
"   Burges A, 2018, INT J PHYTOREMEDIAT, V20, P384, DOI 10.1080/15226514.2017.1365340"
"   Cao YN, 2024, NAT FOOD, V5, DOI 10.1038/s43016-024-01063-1"
"   Celardo I, 2011, NANOSCALE, V3, P1411, DOI 10.1039/c0nr00875c"
"   Chang R.Y., 2018, 2018 IEEE Global Communications Conference (GLOBECOM), P1, DOI DOI 10.1109/GLOCOM.2018.8647261"
"   Charagh S, 2024, PHYSIOL PLANTARUM, V176, DOI 10.1111/ppl.14226"
"   Chen F, 2007, CHEMOSPHERE, V67, P2082, DOI 10.1016/j.chemosphere.2006.10.014"
"   Chen Q, 2020, J ZHEJIANG UNIV-SC B, V21, P442, DOI 10.1631/jzus.B1900576"
"   Cheng XR, 2024, J INTEGR AGR, V23, P3943, DOI 10.1016/j.jia.2024.09.012"
"   Clarke JM, 2006, CROP SCI, V46, P2306, DOI 10.2135/cropsci2005.12.0454"
"   Cotta SR, 2025, BRAZ J MICROBIOL, V56, P225, DOI 10.1007/s42770-024-01583-9"
"   D'Hondt K, 2021, NAT MICROBIOL, V6, P138, DOI 10.1038/s41564-020-00857-w"
"   Dhanapal AR, 2024, ACS OMEGA, V9, P13522, DOI 10.1021/acsomega.3c09776"

"   Drabesch S, 2024, COMMUN EARTH ENVIRON, V5, DOI 10.1038/s43247-024-01794-w"
"   Durand A, 2016, PLANT SOIL, V399, P179, DOI 10.1007/s11104-015-2691-2"
"   Edelstein M, 2018, SCI HORTIC-AMSTERDAM, V234, P431, DOI 10.1016/j.scienta.2017.12.039"
"   Ejaz U, 2023, FRONT PLANT SCI, V14, DOI 10.3389/fpls.2023.1154571"
"   El-Sappah AH, 2024, FRONT PLANT SCI, V15, DOI 10.3389/fpls.2024.1423625"
"   Espada JJ, 2022, SCI TOTAL ENVIRON, V840, DOI 10.1016/j.scitotenv.2022.156675"
"   Feng Hanlin, 2023, Int J Environ Res Public Health, V20, DOI [10.3390/ijerph20031869, 10.3390/ijerph20031869]"
"   Feng JR, 2023, CHEMOSPHERE, V313, DOI 10.1016/j.chemosphere.2022.137391"
"   Food and Agriculture Organization of the United Nations & UN Environment Programme, 2021, GLOBAL ASSESSMENT SO, V48"
"   Food and Agriculture Organization of the United Nations & UN Environment Programme, 2021, GLOBAL ASSESSMENT SO"
"   Gao Jun, 2023, Chemosphere, V335, P139088, DOI [10.1016/j.chemosphere.2023.139088, 10.1016/j.chemosphere.2023.139088]"
"   Godinho Diogo P, 2022, Plant Environ Interact, V3, P170, DOI [10.1002/pei3.10088, 10.1002/pei3.10088]"
"   Goodman RE, 2024, GM CROPS FOOD, V15, P40, DOI 10.1080/21645698.2024.2305944"
"   Guha T, 2021, FREE RADICAL BIO MED, V171, P11, DOI 10.1016/j.freeradbiomed.2021.05.005"
"   Guo YS, 2024, ENVIRON POLLUT, V358, DOI 10.1016/j.envpol.2024.124519"
"   Guzm??n-Moreno J, 2022, MICROBIOL SPECTR, V10, DOI 10.1128/spectrum.00656-22"
"   Hamid Y, 2019, SCI TOTAL ENVIRON, V660, P80, DOI 10.1016/j.scitotenv.2018.12.419"
"   Hanlon P, 2021, CRIT REV FOOD SCI, V61, P959, DOI 10.1080/10408398.2020.1749026"
"   Hannan F, 2024, J CLEAN PROD, V437, DOI 10.1016/j.jclepro.2024.140737"
"   He WW, 2012, BIOMATERIALS, V33, P7547, DOI 10.1016/j.biomaterials.2012.06.076"
"   Hong J, 2021, ENVIRON SCI-NANO, V8, P1196, DOI [10.1039/d0en01129k, 10.1039/D0EN01129K]"
"   Hou DY, 2020, NAT REV EARTH ENV, V1, P366, DOI 10.1038/s43017-020-0061-y"
"   Hou DY, 2020, SCI TOTAL ENVIRON, V729, DOI 10.1016/j.scitotenv.2020.138961"
"   Hu YC, 2024, SCI TOTAL ENVIRON, V918, DOI 10.1016/j.scitotenv.2024.170577"
"   Huang DL, 2018, ECOTOX ENVIRON SAFE, V153, P229, DOI 10.1016/j.ecoenv.2018.01.060"
"   Huang HY, 2024, J HAZARD MATER, V470, DOI 10.1016/j.jhazmat.2024.134227"
"   Huang Y, 2019, SCI TOTAL ENVIRON, V651, P3034, DOI 10.1016/j.scitotenv.2018.10.185"
"   Huang Y, 2018, J ENVIRON MANAGE, V207, P159, DOI 10.1016/j.jenvman.2017.10.072"
"   Inamuddin D., 2024, GENETICALLY ENGINEER"
"   Islam MM, 2024, RSC SUSTAIN, V2, P1269, DOI 10.1039/d3su00440f"
"   Islam S, 2017, SCI TOTAL ENVIRON, V599, P324, DOI 10.1016/j.scitotenv.2017.04.215"
"   Jamil M, 2024, BMC PLANT BIOL, V24, DOI 10.1186/s12870-024-04757-7"
"   Jia XQ, 2020, BIOTECHNOL BIOENG, V117, P3820, DOI 10.1002/bit.27525"
"   Jiang TH, 2024, SUSTAINABILITY-BASEL, V16, DOI 10.3390/su16062515"
"   Jimenez PAJ, 2023, SUSTAINABILITY-BASEL, V15, DOI 10.3390/su15053951"
"   Jurburg SD, 2022, NAT FOOD, V3, P557, DOI 10.1038/s43016-022-00576-x"
"   Kafle A, 2022, Environmental Advances, V8, P100203, DOI [10.1016/j.envadv.2022.100203, 10.1016/j.envadv.2022.100203, DOI 10.1016/J.ENVADV.2022.100203, 10.1016/J.ENVADV.2022.100203]"
"   Kakeshpour T, 2021, PHYSIOL PLANTARUM, V173, P1263, DOI 10.1111/ppl.13522"
"   Kalve S, 2011, CURR SCI INDIA, V100, P888"
"   Kasote DM, 2019, ACS SUSTAIN CHEM ENG, V7, P5142, DOI 10.1021/acssuschemeng.8b06013"
"   Kaur R, 2021, PHYSIOL PLANTARUM, V173, P430, DOI 10.1111/ppl.13491"
"   Khan I, 2020, PLANT PHYSIOL BIOCH, V156, P221, DOI 10.1016/j.plaphy.2020.09.018"
"   Khan IU, 2020, J CLEAN PROD, V272, DOI 10.1016/j.jclepro.2020.123152"
"   Lea-Smith DJ, 2025, NAT COMMUN, V16, DOI 10.1038/s41467-025-58492-0"
"   Li FB, 2023, ENGINEERING-PRC, V25, P28, DOI 10.1016/j.eng.2022.08.010"
"   Li SM, 2024, SCI TOTAL ENVIRON, V906, DOI 10.1016/j.scitotenv.2023.167627"
"   Lin HM, 2018, PLANT GROWTH REGUL, V86, P149, DOI 10.1007/s10725-018-0417-1"
"   Liu P, 2023, ENVIRON POLLUT, V330, DOI 10.1016/j.envpol.2023.121740"
"   Liu YQ, 2024, J HAZARD MATER, V469, DOI 10.1016/j.jhazmat.2024.134085"
"   Lu JZ, 2021, ECOL MODEL, V455, DOI 10.1016/j.ecolmodel.2021.109633"
"   Lv JT, 2019, ENVIRON SCI-NANO, V6, P41, DOI 10.1039/c8en00645h"
"   Macklin MG, 2023, SCIENCE, V381, P1345, DOI 10.1126/science.adg6704"
"   Mahar A, 2016, ECOTOX ENVIRON SAFE, V126, P111, DOI 10.1016/j.ecoenv.2015.12.023"
"   Maity S, 2024, ENVIRON QUAL MANAG, V33, P427, DOI 10.1002/tqem.22100"
"   Manara A., 2012, Plants and heavy metals, SpringerBriefs in Biometals, P27"
"   Mawari G, 2022, ENVIRON HEALTH INSIG, V16, DOI 10.1177/11786302221119151"
"   Melotto M, 2020, FRONT PLANT SCI, V11, DOI 10.3389/fpls.2020.00428"
"   Mishra R, 2024, WATER AIR SOIL POLL, V235, DOI 10.1007/s11270-024-07359-z"
"   Muehe EM, 2019, NAT COMMUN, V10, DOI 10.1038/s41467-019-12946-4"
"   Murugaiyan V, 2021, FRONT PLANT SCI, V12, DOI 10.3389/fpls.2021.753063"
"   Nahar N, 2017, J PLANT PHYSIOL, V218, P121, DOI 10.1016/j.jplph.2017.08.001"
"   Naidu R, 2021, ENVIRON INT, V156, DOI 10.1016/j.envint.2021.106616"
"   Narayanan M, 2023, J ENVIRON MANAGE, V345, DOI 10.1016/j.jenvman.2023.118732"
"   Narayanan M, 2023, ENVIRON RES, V222, DOI 10.1016/j.envres.2023.115413"
"   Ofori-Agyemang Felix, 2024, Chemosphere, V362, P142624, DOI [10.1016/j.chemosphere.2024.142624, 10.1016/j.chemosphere.2024.142624]"
"   Oladele SO, 2021, EMERG CONTAM, V7, P139, DOI 10.1016/j.emcon.2021.05.001"
"   Pande V, 2022, FRONT MICROBIOL, V13, DOI 10.3389/fmicb.2022.824084"
"   Pena-Garcia Y, 2021, J HAZARD MATER, V407, DOI 10.1016/j.jhazmat.2020.124831"
"   Pollard AJ, 1997, NEW PHYTOL, V135, P655, DOI 10.1046/j.1469-8137.1997.00689.x"
"   Qin GW, 2021, CHEMOSPHERE, V267, DOI 10.1016/j.chemosphere.2020.129205"
"   Qiu ZG, 2019, BIOTECHNOL ADV, V37, DOI 10.1016/j.biotechadv.2019.03.010"
"   Ragab G, 2021, J PLANT GROWTH REGUL, V40, P1894, DOI 10.1007/s00344-020-10240-y"
"   Rashid A, 2023, AGRONOMY-BASEL, V13, DOI 10.3390/agronomy13061521"
"   Ren J, 2021, J SOIL SEDIMENT, V21, P1001, DOI 10.1007/s11368-020-02841-7"
"   Rojas LA, 2011, PLOS ONE, V6, DOI 10.1371/journal.pone.0017555"
"   Rozas P, 2022, BIOL RES, V55"
"   Ruan ZP, 2024, NAT COMMUN, V15, DOI 10.1038/s41467-024-49098-z"
"   Sachdev S, 2021, ANTIOXIDANTS-BASEL, V10, DOI 10.3390/antiox10020277"

"   Saleem M, 2021, PLANT PHYSIOL BIOCH, V168, P381, DOI 10.1016/j.plaphy.2021.10.011"
"   Saravanan A, 2021, CHEMOSPHERE, V264, DOI 10.1016/j.chemosphere.2020.128580"
"   Sharma P, 2025, TRENDS PLANT SCI, V30, P539, DOI [10.1016/j.tplants.2025.01.009, 10.1016/j.tplants.2025.01.009]"
"   Sharma P, 2021, J HAZARD MATER, V416, DOI 10.1016/j.jhazmat.2021.125855"
"   Shayanthan A, 2022, FRONT AGRON, V4, DOI 10.3389/fagro.2022.896307"
"   Shen LA, 2024, ENVIRON SCI TECHNOL, V58, P15790, DOI 10.1021/acs.est.4c04009"
"   Shi An, 2024, Science of The Total Environment, V947, P174585, DOI [10.1016/j.scitotenv.2024.174585, 10.1016/j.scitotenv.2024.174585]"
"   Shu YX, 2025, GREEN ENERGY ENVIRON, V10, P869, DOI [10.1016/j.gee.2024.07.007, 10.1016/j.gee.2024.07.007]"
"   Singh J, 2024, J PLANT BIOCHEM BIOT, V33, P457, DOI 10.1007/s13562-024-00885-1"
"   Sladkovska T, 2022, AGRONOMY-BASEL, V12, DOI 10.3390/agronomy12102587"
"   Smyth SJ, 2020, GLOB FOOD SECUR-AGR, V26, DOI 10.1016/j.gfs.2020.100440"
"   Solomon W, 2024, PLANT PHYSIOL BIOCH, V206, DOI 10.1016/j.plaphy.2023.108290"
"   Su YM, 2019, ENVIRON SCI-NANO, V6, P2311, DOI [10.1039/c9en00461k, 10.1039/C9EN00461K]"
"   Sultana R, 2022, ENVIRON SYST RES, V11, DOI 10.1186/s40068-022-00261-9"
"   Tang HY, 2024, FRONT PLANT SCI, V15, DOI 10.3389/fpls.2024.1420408"
"   Tang Z, 2023, J INTEGR PLANT BIOL, V65, P570, DOI 10.1111/jipb.13440"
"   Thai TD, 2023, FRONT BIOENG BIOTECH, V11, DOI 10.3389/fbioe.2023.1178680"
"   Thijs S, 2016, FRONT MICROBIOL, V7, DOI 10.3389/fmicb.2016.00341"
"   Tom M, 2014, PLOS ONE, V9, DOI 10.1371/journal.pone.0112441"
"   Tongprung S., 2024, ENV CHALL, V14, P100845"
"   T??th G, 2016, SCI TOTAL ENVIRON, V565, P1054, DOI 10.1016/j.scitotenv.2016.05.115"
"   Ul Hussan Maqsood, 2024, Science of The Total Environment, V955, P176797, DOI [10.1016/j.scitotenv.2024.176797, 10.1016/j.scitotenv.2024.176797]"
"   Vamerali T, 2014, EUR J AGRON, V53, P56, DOI 10.1016/j.eja.2013.11.008"
"   V??tkov?? M, 2018, CHEMOSPHERE, V200, P217, DOI 10.1016/j.chemosphere.2018.02.118"
"   Wan YA, 2024, TOXICS, V12, DOI 10.3390/toxics12010063"
"   Wang CC, 2023, SCI TOTAL ENVIRON, V858, DOI 10.1016/j.scitotenv.2022.159819"
"   Wang Y, 2022, FRONT MICROBIOL, V13, DOI 10.3389/fmicb.2022.966226"
"   Wang Z, 2023, J AGR FOOD CHEM, V71, P5030, DOI 10.1021/acs.jafc.2c08017"
"   Weyens N, 2013, J SOIL SEDIMENT, V13, P176, DOI 10.1007/s11368-012-0582-1"
"   Wu HH, 2022, PLANT COMMUN, V3, DOI 10.1016/j.xplc.2022.100346"
"   Wu JJX, 2019, CHEM SOC REV, V48, P1004, DOI 10.1039/c8cs00457a"
"   Xiao WQ, 2024, TOXICS, V12, DOI 10.3390/toxics12060400"
"   Xu MQ, 2022, ECOTOX ENVIRON SAFE, V247, DOI 10.1016/j.ecoenv.2022.114244"
"   Xu M, 2022, ENVIRON POLLUT, V300, DOI 10.1016/j.envpol.2022.118982"
"   Yan A, 2020, FRONT PLANT SCI, V11, DOI 10.3389/fpls.2020.00359"
"   Yan X, 2022, ENVIRON SCI-NANO, V9, P2195, DOI [10.1039/d2en00211f, 10.1039/D2EN00211F]"
"   Yang D, 2022, SCI TOTAL ENVIRON, V814, DOI 10.1016/j.scitotenv.2021.152798"
"   Yao J, 2018, CHEM SCI, V9, P2927, DOI 10.1039/c7sc05476a"
"   Zhang BQ, 2020, CHEMOSPHERE, V247, DOI 10.1016/j.chemosphere.2020.125958"
"   Zhang SY, 2024, NAT SUSTAIN, V7, DOI 10.1038/s41893-024-01341-7"
"   Zhang XY, 2015, PLOS ONE, V10, DOI 10.1371/journal.pone.0135182"
"   Zhang YX, 2017, J ENVIRON SCI, V57, P329, DOI 10.1016/j.jes.2017.01.007"
"   Zhao FJ, 2022, MOL PLANT, V15, P27, DOI 10.1016/j.molp.2021.09.016"
"   Zhao LJ, 2022, NAT FOOD, V3, P829, DOI 10.1038/s43016-022-00596-7"
"   Zhou YY, 2024, NAT COMMUN, V15, DOI 10.1038/s41467-024-50685-3"
"   Zhu Zhen, 2025, Science of The Total Environment, V958, P177945, DOI [10.1016/j.scitotenv.2024.177945, 10.1016/j.scitotenv.2024.177945]"
NR 155
TC 12
Z9 12
U1 65
U2 79
PU SPRINGERNATURE
PI LONDON
"PA CAMPUS, 4 CRINAN ST, LONDON, N1 9XW, the United Kingdom"
EI 2731-6092
J9 NAT REV BIOENG
JI Nat. Rev. Bioeng.
PD DEC
PY 2025
VL 3
IS 12
BP 1005
EP 1018
DI 10.1038/s44222-025-00345-y
EA AUG 2025
PG 14
"WC Engineering, Biomedical; Materials Science, Biomaterials"
WE Science Citation Index Expanded (SCI-EXPANDED)
SC Engineering; Materials Science
GA L7165
UT WOS:001551567100001
DA 2026-04-06
ER

PT J
"AU Guo, XF"
"   Pei, Y"
"   Gao, Y"
"   Zhang, GX"

"   Wu, H"
"   Zhang, HY"
"AF Guo, Xiaofang"
"   Pei, Yu"
"   Gao, Yu"
"   Zhang, Guixiang"
"   Wu, Hao"
"   Zhang, Hongyu"
TI Effects of sulfate-reducing bacteria on the plant uptake of heavy metals
   in chelator-washed soil with residual heavy metal-chelant complexes
SO ENVIRONMENTAL GEOCHEMISTRY AND HEALTH
LA English
DT Article
DE Sulfate-reducing bacteria; Heavy metal-chelant complexes; Microbial
   community; Agricultural reuse
ID CONTAMINATED SOIL; REMEDIATION; EDTA; STABILIZATION; ACCUMULATION;
   COMBINATION; DRAINAGE; CADMIUM; COMPOST; MAIZE
"AB The application of sulfate-reducing bacteria (SRB) for stabilizing heavy metals in chelator-washed soils is critical for enabling agricultural reuse, yet its impact on plant uptake of heavy metals remains understudied. In this study, a pot experiment was conducted to examine the effects of SRB-which include Shewanella JN01 (S), Clostridium ZG01 (C), and a mixture of the two strains (M)-on stabilization of heavy metal-chelant complexes and the uptake of heavy metals (Cd, Pb, Cu, and Zn) by Chinese cabbage. The results revealed that all SRB treatments significantly reduced available Pb, Cu, and Zn by 8.23-34.85%, 8.78-15.72%, and 19.83-29.49% in the chelator-washed soils after planting Chinese cabbage, respectively. However, there was no significant change in available Cd contents in all SRB treatments because the formation of CdS with higher solubility constants was more difficult than the formation of CuS and PbS. Clostridium ZG01 reduced Cd, Pb, Cu, and Zn concentrations in shoots by 41.85%, 83.02%, 14.55%, and 23.49%, respectively, and lowered transfer coefficients by 48.18-90.00%, outperforming other treatments. This strain enhanced soil bacterial diversity and organic matter content, likely through metabolic synergies and rhizosphere interactions. Our findings demonstrated Clostridium ZG01 has excellent efficacy in stabilizing metal-chelant complexes and mitigating phytoavailability in alkaline soils, though crop selection or integrated remediation strategies are necessary for safe reuse. Collectively, this study provided an efficient, green and economical strategy for the remediation of heavy metals-contaminated farmland after being washed by chelators."
"C1 [Guo, Xiaofang; Pei, Yu; Gao, Yu; Zhang, Guixiang; Wu, Hao; Zhang, Hongyu] Taiyuan Univ Sci & Technol, Sch Environm & Resources, Taiyuan 030024, Shanxi, Peoples R China."
C3 Taiyuan University of Science & Technology
"RP Zhang, GX (corresponding author), Taiyuan Univ Sci & Technol, Sch Environm & Resources, Taiyuan 030024, Shanxi, Peoples R China."
EM zhanggx@tyust.edu.cn
"OI Zhang, Guixiang/0000-0003-1673-6728"
FU Special fund for Science and Technology Innovation Teams of Shanxi
   Province [202304051001037]; National Natural Science Foundation of China
   [22408256]; China Postdoctoral Science Foundation [2024M750604];
   Teaching Reform Innovation Project of Shanxi Province [J20230868]
FX This work was funded by the special fund for Science and Technology
"   Innovation Teams of Shanxi Province (202304051001037), the National"
"   Natural Science Foundation of China (22408256), the China Postdoctoral"
"   Science Foundation (2024M750604), and Teaching Reform Innovation Project"
   of Shanxi Province (J20230868).
"CR Alexandrino M, 2014, ENVIRON SCI TECHNOL, V48, P3378, DOI 10.1021/es4052044"
"   Beiyuan JZ, 2018, SCI TOTAL ENVIRON, V612, P1463, DOI 10.1016/j.scitotenv.2017.09.015"
"   Castro L, 2017, HYDROMETALLURGY, V168, P56, DOI 10.1016/j.hydromet.2016.08.002"
"   Chen C, 2023, WATER RES, V245, DOI 10.1016/j.watres.2023.120590"
"   Chen Zi-jie, 2021, Shengtaixue Zazhi, V40, P2315, DOI 10.13292/j.1000-4890.202108.009"
"   de Quadros PD, 2016, APPL SOIL ECOL, V98, P195, DOI 10.1016/j.apsoil.2015.10.016"
"   Fan MC, 2016, WATER RES, V96, P198, DOI 10.1016/j.watres.2016.03.061"
"   Fleming M, 2013, ENVIRON POLLUT, V177, P90, DOI 10.1016/j.envpol.2013.02.013"
"   Gomez-Montano L, 2013, SOIL BIOL BIOCHEM, V65, P50, DOI 10.1016/j.soilbio.2013.04.005"
"   Gu WZ, 2021, CHEMOSPHERE, V279, DOI 10.1016/j.chemosphere.2021.130437"
"   Guo XF, 2011, PEDOSPHERE, V21, P650, DOI 10.1016/S1002-0160(11)60167-7"
"   Guo XF, 2023, ENVIRON SCI POLLUT R, V30, P739, DOI 10.1007/s11356-022-22126-5"
"   Guo XF, 2020, ENVIRON TECHNOL INNO, V19, DOI 10.1016/j.eti.2020.101038"
"   Guo XF, 2018, J SOIL SEDIMENT, V18, P835, DOI 10.1007/s11368-017-1781-6"
"   Hakansson Torbjorn, 2012, International Journal of Environment and Waste Management, V9, P330, DOI 10.1504/IJEWM.2012.046396"
"   Hakim S, 2021, FRONT SUSTAIN FOOD S, V5, DOI 10.3389/fsufs.2021.617157"
"   Hazrati S, 2020, ECOTOX ENVIRON SAFE, V201, DOI 10.1016/j.ecoenv.2020.110807"
"   Hou DY, 2020, NAT REV EARTH ENV, V1, P366, DOI 10.1038/s43017-020-0061-y"
"   Hu JJ, 2022, ENVIRON SCI-PROC IMP, V24, P598, DOI [10.1039/d2em00036a, 10.1039/D2EM00036A]"
"   Hu PJ, 2014, CHEMOSPHERE, V117, P532, DOI 10.1016/j.chemosphere.2014.08.081"
"   Hu XS, 2021, SCI TOTAL ENVIRON, V775, DOI 10.1016/j.scitotenv.2021.144946"
"   Hu YN, 2020, J HAZARD MATER, V391, DOI 10.1016/j.jhazmat.2020.122244"
"   Jaskulak M, 2022, CHEMOSPHERE, V298, DOI 10.1016/j.chemosphere.2022.134321"
"   Jelusic M, 2014, SCI TOTAL ENVIRON, V475, P142, DOI 10.1016/j.scitotenv.2013.11.111"
"   Karami N, 2011, J HAZARD MATER, V191, P41, DOI 10.1016/j.jhazmat.2011.04.025"
"   Karlapudi Abraham Peele, 2018, Petroleum, V4, P241, DOI 10.1016/j.petlm.2018.03.007"
"   Kaurin A, 2020, CHEMOSPHERE, V260, DOI 10.1016/j.chemosphere.2020.127673"
"   Ke YX, 2023, ENVIRON TECHNOL INNO, V32, DOI 10.1016/j.eti.2023.103356"
"   Khalid S, 2017, J GEOCHEM EXPLOR, V182, P247, DOI 10.1016/j.gexplo.2016.11.021"
"   Kiran MG, 2017, J HAZARD MATER, V324, P62, DOI 10.1016/j.jhazmat.2015.12.042"
"   Lee YS, 2017, J GINSENG RES, V41, P60, DOI 10.1016/j.jgr.2015.12.012"
"   Li X, 2018, ECOTOX ENVIRON SAFE, V158, P162, DOI 10.1016/j.ecoenv.2018.04.025"
"   Li X, 2017, J HAZARD MATER, V324, P340, DOI 10.1016/j.jhazmat.2016.10.067"
"   Liu GN, 2018, GEODERMA, V312, P104, DOI 10.1016/j.geoderma.2017.10.013"

"   [?????? Liu Xing], 2020, [?????????????????????, Environmental Pollution & Control], V42, P507"
"   Lu R.H, 1999, Analytical methods of soil and agro-chemistry"
"   Lv Y, 2022, POL J ENVIRON STUD, V31, P1747, DOI 10.15244/pjoes/141326"
"   Matsuura N, 2021, APPL MICROBIOL BIOT, V105, P2181, DOI 10.1007/s00253-021-11113-4"
"   Mmotla K, 2025, ANN MICROBIOL, V75, DOI 10.1186/s13213-025-01793-y"
"   Nida K, 2024, J SOIL SCI PLANT NUT, V24, P4624, DOI 10.1007/s42729-024-01859-x"
"   Oldroyd GED, 2020, SCIENCE, V368, P45, DOI 10.1126/science.aba0196"
"   Rostami S, 2023, ENVIRON PROCESS, V10, DOI 10.1007/s40710-023-00643-w"
"   Sasse J, 2018, TRENDS PLANT SCI, V23, P25, DOI 10.1016/j.tplants.2017.09.003"
"   Scavino A.F., 2013, Bacteria in agrobiology: crop productivity, P265, DOI [10.1007/978-3-642-37241-4_11, DOI 10.1007/978-3-642-37241-4_11, DOI 10.1007/978-3-642-37241-411]"
"   Tu C, 2020, ENVIRON INT, V137, DOI 10.1016/j.envint.2020.105576"
"   Wang GY, 2020, ENVIRON RES, V186, DOI 10.1016/j.envres.2020.109554"
"   Wang GY, 2018, J CLEAN PROD, V197, P479, DOI 10.1016/j.jclepro.2018.06.225"
"   Wang QW, 2019, CHEMOSPHERE, V220, P1200, DOI 10.1016/j.chemosphere.2018.12.212"
"   Wang X, 2021, ENVIRON POLLUT, V277, DOI 10.1016/j.envpol.2021.116758"
"   Wieczorek J, 2023, SCI TOTAL ENVIRON, V882, DOI 10.1016/j.scitotenv.2023.163574"
"   Wu BH, 2024, SCI TOTAL ENVIRON, V949, DOI 10.1016/j.scitotenv.2024.175009"
"   Wu YF, 2022, RESOUR CONSERV RECY, V181, DOI 10.1016/j.resconrec.2022.106261"
"   Xia Y, 2024, SCI TOTAL ENVIRON, V934, DOI 10.1016/j.scitotenv.2024.173158"
"   Xu CM, 2021, MATERIALS, V14, DOI 10.3390/ma14226981"
"   Xu DM, 2021, J CLEAN PROD, V321, DOI 10.1016/j.jclepro.2021.128730"
"   Yang ZD, 2023, FRONT MICROBIOL, V14, DOI 10.3389/fmicb.2023.1306573"
"   Yoo JC, 2018, SCI TOTAL ENVIRON, V616, P572, DOI 10.1016/j.scitotenv.2017.10.310"
"   Yuan CL, 2021, J HAZARD MATER, V408, DOI 10.1016/j.jhazmat.2020.124417"
"   Zhai XQ, 2018, SCI TOTAL ENVIRON, V635, P92, DOI 10.1016/j.scitotenv.2018.04.119"
"   Zhang GX, 2023, J SOIL SEDIMENT, V23, P2457, DOI 10.1007/s11368-023-03487-x"
"   Zhao Q, 2021, J HAZARD MATER, V406, DOI 10.1016/j.jhazmat.2020.124680"
NR 61
TC 2
Z9 2
U1 11
U2 21
PU SPRINGER
PI DORDRECHT
"PA VAN GODEWIJCKSTRAAT 30, 3311 GZ DORDRECHT, NETHERLANDS"
SN 0269-4042
EI 1573-2983
J9 ENVIRON GEOCHEM HLTH
JI Environ. Geochem. Health
PD MAY 28
PY 2025
VL 47
IS 6
AR 235
DI 10.1007/s10653-025-02543-4
PG 17
"WC Engineering, Environmental; Environmental Sciences; Public,"
Environmental & Occupational Health; Water Resources
WE Science Citation Index Expanded (SCI-EXPANDED)
"SC Engineering; Environmental Sciences & Ecology; Public, Environmental &"
Occupational Health; Water Resources
GA 3EK5N
UT WOS:001498366500002
PM 40437111
DA 2026-04-06
ER

PT J
"AU Ma, JJ"
"   Luo, ZT"
"   Gao, GH"
"   Cui, YM"
"   Ying, M"
"   Huang, L"
"   Li, MT"
"AF Ma, Jiajun"
"   Luo, Zetian"
"   Gao, Guohui"
"   Cui, Yumeng"
"   Ying, Ming"
"   Huang, Lei"
"   Li, Meitong"
TI Biochar enhances the remediation of fluorene and Cd(II) co-contamination
by Burkholderia sp. FM-2
SO ECOTOXICOLOGY AND ENVIRONMENTAL SAFETY
LA English
DT Article
DE Polycyclic aromatic hydrocarbons; Fluorene; Heavy metal; Cadmium;
Biochar; Bioremediation
ID POLYCYCLIC AROMATIC-HYDROCARBONS; SOIL; BIOREMEDIATION; DEGRADATION;
ADSORPTION; TOLERANCE; EFFICIENT; EXPOSURE; BACTERIA; METALS
"AB This study investigated the fluorene degradation ability and metabolic pathway of Burkholderia sp. FM-2. The FM-2 demonstrated a 72.16 % degradation rate of 300 mg/L fluorene over a period of three days. HPLC-MS

analysis identified major metabolites including 9-fluorenol, 9-fluorenone, and phthalate. The impact of heavy metals on fluorene degradation was also assessed. FM-2 exhibited strong tolerance to heavy metals, with a minimum inhibitory concentration (MIC) of 1000 mg/L for Cd(II). The study found that the removal rate of 100 mg/L Cd(II) was 70.42 %, and it was observed that this concentration had a slightly enhancing effect on fluorene degradation. Proteomic analysis revealed that multicopper oxidases and proteins involved in metabolic pathways for instance glutathione metabolism, sulfur metabolism were up-regulated under Cd(II) stress, suggesting potential mechanisms for Cd(II) tolerance and co-metabolism. Moreover, the effect of biochar on FM-2 for the remediation of co-contamination with fluorene and Cd(II) was evaluated. After immobilizing FM-2 with modified biochar (BC-BW), the degradation efficiency of fluorene significantly increased from 72.58 % to 84.93 % and the removal efficiency of Cd(II) increased from 70.42 % to 84.50 %. In the contaminated soil remediation experiment, the removal rates of fluorene and Cd(II) by the immobilized bacteria were 72.59 % and 66.67 %, respectively. These findings demonstrate that modified biochar enhances the remediation efficiency of FM-2 in co-contaminated soils. This study provides theoretical support and practical guidance for the application of biochar-based immobilization techniques in microbial remediation of complex polluted environments."
"C1 [Ma, Jiajun; Luo, Zetian; Gao, Guohui; Cui, Yumeng; Ying, Ming; Huang, Lei; Li, Meitong] Tianjin Univ Technol, Coll Chem & Chem Engn, Tianjin Key Lab Organ Solar Cells & Photochem Cons, Tianjin 300384, Peoples R China."
C3 Tianjin University of Technology
"RP Huang, L; Li, MT (corresponding author), Tianjin Univ Technol, Coll Chem & Chem Engn, Tianjin Key Lab Organ Solar Cells & Photochem Cons, Tianjin 300384, Peoples R China."
EM martintin@stud.tjut.edu.cn; luozetian@stud.tjut.edu.cn;
gaoguohui@stud.tjut.edu.cn; cuiyumeng@stud.tjut.edu.cn; ym@tjut.edu.cn;
huanglei@tjut.edu.cn; tjutlmt@email.tjut.edu.cn
"OI Huang, Lei/0000-0002-3490-3393"
FU National Natural Science Foundation of China [42077212]; Tiankai Higher
Education Science and Technology Innovation Park Enterprise R & D
Special Project [23YFZXYC00019]
FX This work is supported by the National Natural Science Foundation of
" China (Grant No. 42077212) , Tiankai Higher Education Science and"
Technology Innovation Park Enterprise R & D Special Project
(23YFZXYC00019) .
"CR Abu Talha M, 2018, BIORESOURCE TECHNOL, V252, P37, DOI 10.1016/j.biortech.2017.12.081"
" Awual E, 2024, J IND ENG CHEM, V131, P585, DOI 10.1016/j.jiec.2023.10.062"
" Awual MR, 2023, SEP PURIF TECHNOL, V319, DOI 10.1016/j.seppur.2023.124088"
" Awual MR, 2020, MICROCHEM J, V154, DOI 10.1016/j.microc.2019.104585"
" Awual MR, 2019, J CLEAN PROD, V228, P1311, DOI 10.1016/j.jclepro.2019.04.325"
" Awual MR, 2019, COMPOS PART B-ENG, V171, P294, DOI 10.1016/j.compositesb.2019.05.078"
" Awual MR, 2019, J CLEAN PROD, V228, P778, DOI 10.1016/j.jclepro.2019.04.280"
" Awual MR, 2019, J MOL LIQ, V284, P502, DOI 10.1016/j.molliq.2019.03.157"
" Awual MR, 2016, CHEM ENG J, V303, P539, DOI 10.1016/j.cej.2016.06.040"
" Banerjee M, 2021, INT J ENVIRON RES, V15, P875, DOI 10.1007/s41742-021-00362-w"
" Bankole PO, 2021, ECOTOX ENVIRON SAFE, V208, DOI 10.1016/j.ecoenv.2020.111619"
" Bolan S, 2023, SCI TOTAL ENVIRON, V886, DOI 10.1016/j.scitotenv.2023.163968"
" Briggs CA, 2008, BRIT J PHARMACOL, V153, P1054, DOI 10.1038/sj.bjp.0707649"
" Cao HT, 2023, ACS APPL MATER INTER, V15, P7236, DOI 10.1021/acsami.2c22266"
" Casellas M, 1998, CAN J MICROBIOL, V44, P734, DOI 10.1139/cjm-44-8-734"
" Casellas M, 1997, APPL ENVIRON MICROB, V63, P819, DOI 10.1128/AEM.63.3.819-826.1997"
" Chen BL, 2012, J SOIL SEDIMENT, V12, P1350, DOI 10.1007/s11368-012-0554-5"
" Chu JB, 2009, ACTA CRYSTALLOGR E, V65, pO2009, DOI 10.1107/S1600536809027287"
" Das AS, 2022, CHEMISTRYSELECT, V7, DOI 10.1002/slct.202201097"
" Ganesan M, 2022, CHEMOSPHERE, V303, DOI 10.1016/j.chemosphere.2022.134956"
" Grass G, 2001, BIOCHEM BIOPH RES CO, V286, P902, DOI 10.1006/bbrc.2001.5474"
" Hale L, 2015, SOIL BIOL BIOCHEM, V81, P228, DOI 10.1016/j.soilbio.2014.11.023"
" Hossain MS, 2024, PROCESS SAF ENVIRON, V185, P367, DOI 10.1016/j.psep.2024.03.026"
" Islam A, 2025, INT J HYDROGEN ENERG, V140, P745, DOI 10.1016/j.ijhydene.2025.05.197"
" Islam A, 2025, ADV COLLOID INTERFAC, V343, DOI 10.1016/j.cis.2025.103558"
" Islam A, 2025, INT J HYDROGEN ENERG, V101, P173, DOI 10.1016/j.ijhydene.2024.12.300"
" Islam A, 2025, RENEW SUST ENERG REV, V208, DOI 10.1016/j.rser.2024.115033"
" Jiang YT, 2022, CHEMOSPHERE, V286, DOI 10.1016/j.chemosphere.2021.131721"
" Kim L, 2019, APPL BIOL CHEM, V62"
" Kubra KT, 2021, J MOL LIQ, V338, DOI 10.1016/j.molliq.2021.116667"
" Kubra KT, 2021, J MOL LIQ, V329, DOI 10.1016/j.molliq.2021.115541"
" Lehmann J., 2006, Mitigation and Adaptation Strategies for Global Change, V11, P403, DOI 10.1007/s11027-005-9006-5"
" Li B, 2024, WATER-SUI, V16, DOI 10.3390/w16172537"
" Li R, 2022, SCI TOTAL ENVIRON, V837, DOI 10.1016/j.scitotenv.2022.155563"
" Li X, 2022, ENVIRON TECHNOL, V43, P1068, DOI 10.1080/09593330.2020.1815858"
" Li XN, 2019, J HAZARD MATER, V364, P325, DOI 10.1016/j.jhazmat.2018.10.041"
" Liang JQ, 2021, J HAZARD MATER, V404, DOI 10.1016/j.jhazmat.2020.124037"
" Liu SH, 2017, BIORESOURCE TECHNOL, V224, P25, DOI 10.1016/j.biortech.2016.11.095"
" Liu XX, 2019, FRONT MICROBIOL, V10, DOI 10.3389/fmicb.2019.00408"
" Liu YY, 2025, J ENVIRON CHEM ENG, V13, DOI 10.1016/j.jece.2025.117330"
" Ma N, 2015, J BASIC MICROB, V55, P1002, DOI 10.1002/jobm.201400847"
" Mahdi Z, 2019, J ENVIRON CHEM ENG, V7, DOI 10.1016/j.jece.2019.103379"
" Mansour C, 2017, FISH SHELLFISH IMMUN, V67, P110, DOI 10.1016/j.fsi.2017.06.008"
" Miras R, 2008, J BIOL INORG CHEM, V13, P195, DOI 10.1007/s00775-007-0310-2"
" Nongkhlaw M, 2019, PLOS ONE, V14, DOI 10.1371/journal.pone.0216995"
" Peiffer J, 2016, NEUROTOXICOLOGY, V53, P321, DOI 10.1016/j.neuro.2015.11.006"
" Rajapaksha AU, 2015, ENVIRON GEOCHEM HLTH, V37, P931, DOI 10.1007/s10653-015-9694-z"
" Rehan AI, 2023, COLLOID SURFACE A, V673, DOI 10.1016/j.colsurfa.2023.131859"
" Salman MS, 2023, J MOL STRUCT, V1282, DOI 10.1016/j.molstruc.2023.135259"
" Thavamani P, 2012, BIODEGRADATION, V23, P823, DOI 10.1007/s10532-012-9572-7"
" Thomas KRJ, 2013, MATER TECHNOL, V28, P71, DOI 10.1179/1753555712Y.0000000036"

"   Tuersun N, 2024, ACS OMEGA, V9, P17989, DOI 10.1021/acsomega.3c09501"
"   Wang NN, 2023, WATER AIR SOIL POLL, V234, DOI 10.1007/s11270-023-06296-7"
"   Wattiau P, 2001, RES MICROBIOL, V152, P861, DOI 10.1016/S0923-2508(01)01269-4"
"   Wong KW, 2005, LETT APPL MICROBIOL, V40, P50, DOI 10.1111/j.1472-765X.2004.01623.x"
"   Xi JM, 2018, INT J MOL SCI, V19, DOI 10.3390/ijms19124044"
"   Xiao B, 2025, WATER AIR SOIL POLL, V236, DOI 10.1007/s11270-025-08111-x"
"   Yu HL, 2020, FRONT MICROBIOL, V11, DOI 10.3389/fmicb.2020.558104"
"   Yue ZB, 2007, BIORESOURCE TECHNOL, V98, P781, DOI 10.1016/j.biortech.2006.03.017"
"   Zhang M, 2019, INT J ENV RES PUB HE, V16, DOI 10.3390/ijerph16245098"
"   Zhang ZZ, 2011, BIORESOURCE TECHNOL, V102, P4111, DOI 10.1016/j.biortech.2010.12.064"
NR 61
TC 1
Z9 1
U1 16
U2 16
PU ACADEMIC PRESS INC ELSEVIER SCIENCE
PI SAN DIEGO
"PA 525 B ST, STE 1900, SAN DIEGO, CA 92101-4495 USA"
SN 0147-6513
EI 1090-2414
J9 ECOTOX ENVIRON SAFE
JI Ecotox. Environ. Safe.
PD OCT 1
PY 2025
VL 304
AR 119134
DI 10.1016/j.ecoenv.2025.119134
EA SEP 2025
PG 13
WC Environmental Sciences; Toxicology
WE Science Citation Index Expanded (SCI-EXPANDED)
SC Environmental Sciences & Ecology; Toxicology
GA 8HJ0H
UT WOS:001588699300005
PM 41032980
"OA Green Submitted, gold"
DA 2026-04-06
ER

PT J
"AU Song, YQ"
"   Shahir, S"
"   Abd Manan, F"
"AF Song, Yu Qing"
"   Shahir, Shafinaz"
"   Abd Manan, Fazilah"
TI Bacterial inoculant-assisted phytoremediation of heavy
   metal-contaminated soil: Inoculant development and the inoculation
   effects
SO BIOLOGIA
LA English
DT Review
DE Heavy metals; Bacterial inoculant-assisted phytoremediation; Plant
   growth-promoting bacteria; Plant-bacteria interaction; Inoculation
   effect
ID GROWTH-PROMOTING RHIZOBACTERIA; BIOAUGMENTATION; BIOREMEDIATION;
   EFFICIENCY; RESPONSES; REMOVAL; CIRCUIT; NICKEL; L.
"AB Heavy metal contamination is a worldwide concern and a challenge to resolve due to the toxicity poses a significant threat to all living organisms in the environment. Phytoremediation is an environmental-friendly approach to alleviate heavy metal contamination in the soil. Combining the plant and beneficial bacteria inoculant to remediate soil heavy metal is known as bacterial inoculant-assisted phytoremediation. Beneficial bacteria facilitate plant metal uptake by mobilizing the heavy metals in the soil and enhancing plant vigor. The inoculant formulations can be prepared as liquid, granules or powders. Nonetheless, the effective interaction between plant and bacteria is not promised in unsterilized soil, thus affecting the phytoremediation efficiency. The development of bacterial inoculants for phytoremediation and the effects of the inoculants in soil are overlooked in current research studies. Therefore, an ample understanding of the bacterial inoculant-assisted phytoremediation concept is pertinent to circumvent the limitations. This article discusses the relationship between beneficial bacteria inoculant and plant in different techniques of phytoremediation, recent advances in developing bacterial inoculants for phytoremediation and the fate of the inoculants in soil. The provided information in this article generates more insights on the optimization works, which ultimately leading to the possible field application."
"C1 [Song, Yu Qing; Shahir, Shafinaz; Abd Manan, Fazilah] Univ Teknol Malaysia, Fac Sci, Dept Biosci, Johor Baharu 81310, Malaysia."
C3 Universiti Teknologi Malaysia
"RP Abd Manan, F (corresponding author), Univ Teknol Malaysia, Fac Sci, Dept Biosci, Johor Baharu 81310, Malaysia."
EM m-fazilah@utm.my
"RI Abd Manan, Fazilah/IQT-9516-2023"
"OI Shahir, Shafinaz/0000-0001-5711-5659"
"FU Ministry of Higher Education Fundamental Research Grant Scheme, Malaysia"
   (FRGS) [R.J130000.7854.5F102]; Universiti Teknologi Malaysia's Geran
   Universiti Penyelidikan (GUP TIER 1) [17H14]
FX The financial support was provided through the Ministry of Higher
"   Education Fundamental Research Grant Scheme, Malaysia, (FRGS Grant No."

R.J130000.7854.5F102) and Universiti Teknologi Malaysia's Geran
Universiti Penyelidikan (GUP TIER 1 Grant No. 17H14). The authors would
" like to thank the colleagues of the Department of Biosciences, Faculty"
" of Science, Universiti Teknologi Malaysia for the ideas and supports"
given throughout the research process.
"CR Adeleke R, 2017, S AFR J BOT, V108, P393, DOI 10.1016/j.sajb.2016.09.002"
" Akhtar MJ, 2018, CHEMOSPHERE, V190, P234, DOI 10.1016/j.chemosphere.2017.09.136"
" Ali H, 2013, CHEMOSPHERE, V91, P869, DOI 10.1016/j.chemosphere.2013.01.075"
" Alka S, 2020, ENVIRON TECHNOL INNO, V17, DOI 10.1016/j.eti.2020.100602"
" Azizi N, 2021, ENVIRON EXP BOT, V187, DOI 10.1016/j.envexpbot.2021.104470"
" Bandara T, 2017, J SOIL SEDIMENT, V17, P665, DOI 10.1007/s11368-015-1243-y"
" Beans C, 2017, P NATL ACAD SCI USA, V114, P7475, DOI 10.1073/pnas.1707883114"
" Bell TH, 2014, TRENDS BIOTECHNOL, V32, P271, DOI 10.1016/j.tibtech.2014.02.008"
" Beolchini F, 2017, CHEM ECOL, V33, P103, DOI 10.1080/02757540.2017.1281252"
" Bernat P, 2019, CURR MICROBIOL, V76, P1320, DOI 10.1007/s00284-019-01757-x"
" Bruno LB, 2021, J ENVIRON MANAGE, V289, DOI 10.1016/j.jenvman.2021.112553"
" Chen L, 2014, SOIL BIOL BIOCHEM, V68, P300, DOI 10.1016/j.soilbio.2013.10.021"
" Chiboub M, 2018, J PLANT RES, V131, P99, DOI 10.1007/s10265-017-0971-z"
" Chuaphasuk C, 2019, ENVIRON SCI POLLUT R, V26, P23679, DOI 10.1007/s11356-019-05661-6"
" Daniel R, 2005, NAT REV MICROBIOL, V3, P470, DOI 10.1038/nrmicro1160"
" Fagorzi C, 2018, GENES-BASEL, V9, DOI 10.3390/genes9110542"
" Fan MC, 2018, CHEMOSPHERE, V197, P729, DOI 10.1016/j.chemosphere.2018.01.102"
" Garc??a-Gonzalo P, 2017, ENVIRON SCI POLLUT R, V24, P25713, DOI 10.1007/s11356-016-6667-4"
" Glick BR, 2012, SCIENTIFICA, V2012, DOI 10.6064/2012/963401"
" Glick BR, 2010, BIOTECHNOL ADV, V28, P367, DOI 10.1016/j.biotechadv.2010.02.001"
" Glick BR, 1997, BIOTECHNOL ADV, V15, P353, DOI 10.1016/S0734-9750(97)00004-9"
" Griffiths BS, 2013, FEMS MICROBIOL REV, V37, P112, DOI 10.1111/j.1574-6976.2012.00343.x"
" Guarino F, 2020, CHEMOSPHERE, V251, DOI 10.1016/j.chemosphere.2020.126310"
" Guo D, 2020, ENVIRON POLLUT, V265, DOI 10.1016/j.envpol.2020.114867"
" Guo Y, 2021, FRONT BIOENG BIOTECH, V9, DOI 10.3389/fbioe.2021.585617"
" Hossain Z, 2013, FRONT PLANT SCI, V3, DOI 10.3389/fpls.2012.00310"
" Hrynkiewicz K, 2018, ENVIRON REV, V26, P316, DOI 10.1139/er-2018-0023"
" Huang JL, 2016, J GEN APPL MICROBIOL, V62, P258, DOI 10.2323/jgam.2016.04.007"
" Irshad S, 2020, J HAZARD MATER, V381, DOI 10.1016/j.jhazmat.2019.120903"
" Ji SH, 2019, SCI REP-UK, V9, DOI 10.1038/s41598-018-38026-z"
" Jimoh AA, 2019, ECOTOX ENVIRON SAFE, V184, DOI 10.1016/j.ecoenv.2019.109607"
" Ju WL, 2019, ECOTOX ENVIRON SAFE, V167, P218, DOI 10.1016/j.ecoenv.2018.10.016"
" Karwowska E, 2014, J HAZARD MATER, V264, P203, DOI 10.1016/j.jhazmat.2013.11.018"
" Ke T, 2021, ENVIRON POLLUT, V271, DOI 10.1016/j.envpol.2020.116314"
" Khan A, 2015, ENVIRON SCI POLLUT R, V22, P13772, DOI 10.1007/s11356-015-4881-0"
" Kiers ET, 2013, NEW PHYTOL, V198, P4, DOI 10.1111/nph.12015"
" Kong ZY, 2019, ECOTOX ENVIRON SAFE, V183, DOI 10.1016/j.ecoenv.2019.109504"
" Kong ZY, 2017, ADV MICROB PHYSIOL, V71, P97, DOI 10.1016/bs.ampbs.2017.04.001"
" Kumar S, 2018, J PLANT NUTR, V41, P1035, DOI 10.1080/01904167.2018.1433211"
" Lee K, 2009, GLOB INST, P1"
" Li WC, 2010, PLANT SOIL, V326, P453, DOI 10.1007/s11104-009-0025-y"
" Li X, 2016, RSC ADV, V6, P65816, DOI 10.1039/c6ra11767h"
" Li Y, 2020, PLANT SOIL, V448, P439, DOI 10.1007/s11104-020-04447-x"
" Liu L, 2012, J BIOMED BIOTECHNOL, DOI 10.1155/2012/251364"
" Luna JM, 2016, PROCESS SAF ENVIRON, V102, P558, DOI 10.1016/j.psep.2016.05.010"
" Luo JM, 2014, PLOS ONE, V9, DOI 10.1371/journal.pone.0108185"
" Lupwayi NZ, 2000, FIELD CROP RES, V65, P259, DOI 10.1016/S0378-4290(99)00091-X"
" Ma Y, 2019, J HAZARD MATER, V379, DOI 10.1016/j.jhazmat.2019.120813"
" MacDonald IC, 2016, ADV DRUG DELIVER REV, V105, P20, DOI 10.1016/j.addr.2016.08.008"
" Manzoor M, 2019, CHEMOSPHERE, V227, P561, DOI 10.1016/j.chemosphere.2019.04.093"
" Marwa N, 2020, ECOTOX ENVIRON SAFE, V196, DOI 10.1016/j.ecoenv.2020.110498"
" Mrozik A, 2010, MICROBIOL RES, V165, P363, DOI 10.1016/j.micres.2009.08.001"
" Navazas A, 2021, SCI TOTAL ENVIRON, V769, DOI 10.1016/j.scitotenv.2020.144648"
" Naveed M, 2020, INT J ENV RES PUB HE, V17, DOI 10.3390/ijerph17238859"
" Nielsen UN, 2015, ANNU REV ENV RESOUR, V40, P63, DOI 10.1146/annurev-environ-102014-021257"
" Olanrewaju OS, 2017, WORLD J MICROB BIOT, V33, DOI 10.1007/s11274-017-2364-9"
" Plociniczak T, 2020, ECOTOX ENVIRON SAFE, V194, DOI 10.1016/j.ecoenv.2020.110434"
" Pollard AJ, 2014, PLANT SCI, V217, P8, DOI 10.1016/j.plantsci.2013.11.011"
" Prapagdee B, 2017, ENVIRON SCI POLLUT R, V24, P19249, DOI 10.1007/s11356-017-9591-3"
" Ren XM, 2019, FRONT MICROBIOL, V10, DOI 10.3389/fmicb.2019.01455"
" Romano I, 2020, FRONT PLANT SCI, V11, DOI 10.3389/fpls.2020.00006"
" Rylott EL, 2020, CURR OPIN CHEM BIOL, V58, P86, DOI 10.1016/j.cbpa.2020.07.004"
" SALT DE, 1995, BIO-TECHNOL, V13, P468, DOI 10.1038/nbt0595-468"
" Santos MS, 2019, AMB EXPRESS, V9, DOI 10.1186/s13568-019-0932-0"
" Sharma D, 2015, SPRINGERPLUS, V4, DOI 10.1186/2193-1801-4-4"
" Sharma S, 2018, 3 BIOTECH, V8, DOI 10.1007/s13205-018-1237-8"
" Silambarasan S, 2020, PLANT SOIL, V457, P241, DOI 10.1007/s11104-020-04734-7"
" Srichandan H, 2019, HYDROMETALLURGY, V189, DOI 10.1016/j.hydromet.2019.105122"
" Strawn DG, 2018, GEOCHEM T, V19, DOI 10.1186/s12932-018-0055-6"
" Tamburini E, 2017, B ENVIRON CONTAM TOX, V98, P310, DOI 10.1007/s00128-016-1866-8"
" Tang X, 2018, J ENVIRON MANAGE, V206, P633, DOI 10.1016/j.jenvman.2017.11.009"
" Tay PKR, 2017, ACS SYNTH BIOL, V6, P1841, DOI 10.1021/acssynbio.7b00137"
" Tchounwou Paul B, 2012, Exp Suppl, V101, P133, DOI 10.1007/978-3-7643-8340-4_6"
" Thijs S, 2016, FRONT MICROBIOL, V7, DOI 10.3389/fmicb.2016.00341"
" Thompson IP, 2005, ENVIRON MICROBIOL, V7, P909, DOI 10.1111/j.1462-2920.2005.00804.x"
" Todman LC, 2016, SCI REP-UK, V6, DOI 10.1038/srep28426"
" Trabelsi D, 2013, BIOMED RES INT-UK, V2013, DOI 10.1155/2013/863240"
" Tu C, 2020, ENVIRON INT, V137, DOI 10.1016/j.envint.2020.105576"
" Ullah A, 2015, ENVIRON EXP BOT, V117, P28, DOI 10.1016/j.envexpbot.2015.05.001"

"   Vryzas Z, 2016, FRONT MICROBIOL, V7, DOI 10.3389/fmicb.2016.01968"
"   Wang T, 2017, SCI REP-UK, V7, DOI 10.1038/s41598-017-12503-3"
"   Wang Y, 2020, ECOTOX ENVIRON SAFE, V192, DOI 10.1016/j.ecoenv.2020.110294"
"   Wen CQ, 2017, PLOS ONE, V12, DOI 10.1371/journal.pone.0176716"
"   Yahaghi Z, 2019, S AFR J BOT, V124, P573, DOI 10.1016/j.sajb.2019.01.006"
"   Ye JP, 2020, ENVIRON POLLUT, V267, DOI 10.1016/j.envpol.2020.115447"
"   Yu SM, 2019, J SOIL SEDIMENT, V19, P1064, DOI 10.1007/s11368-018-2111-3"
"   Zgorelec Z, 2020, SCI REP-UK, V10, DOI 10.1038/s41598-020-63488-5"
"   Zhang JR, 2018, ENVIRON POLLUT, V235, P710, DOI 10.1016/j.envpol.2017.12.106"
NR 88
TC 13
Z9 15
U1 7
U2 123
PU SPRINGER
PI NEW YORK
"PA ONE NEW YORK PLAZA, SUITE 4600, NEW YORK, NY, UNITED STATES"
SN 0006-3088
EI 1336-9563
J9 BIOLOGIA
JI Biologia
PD SEP
PY 2021
VL 76
IS 9
BP 2675
EP 2685
DI 10.1007/s11756-021-00804-y
EA JUN 2021
PG 11
WC Biology
WE Science Citation Index Expanded (SCI-EXPANDED)
SC Life Sciences & Biomedicine - Other Topics
GA UC6QO
UT WOS:000659023000002
DA 2026-04-06
ER

PT J
"AU Xing, YH"
"   Tan, SX"
"   Liu, S"
"   Xu, SZ"
"   Wan, WJ"
"   Huang, QY"
"   Chen, WL"
"AF Xing, Yonghui"
"   Tan, Shuxin"
"   Liu, Song"
"   Xu, Shaozu"
"   Wan, Wenjie"
"   Huang, Qiaoyun"
"   Chen, Wenli"
TI Effective immobilization of heavy metals via reactive barrier by
   rhizosphere bacteria and their biofilms
SO ENVIRONMENTAL RESEARCH
LA English
DT Article
DE Heavy metal; Bioremediation; Planktonic cell; Biofilm; Reactive barrier
ID CONTAMINATED SOILS; OXIDATIVE STRESS; CADMIUM; REMEDIATION;
   PHYTOREMEDIATION; BIOACCUMULATION; TOLERANCE; BINDING; COPPER
"AB As the portal of plants, rhizosphere microorganisms play an essential role in controlling the species, transformation, and bioavailability of heavy metals, yet the potential passivation mechanism is still unclear. In this study, two heavy metal resistant and growth-promoting rhizosphere bacteria were screened, and their mechanisms in dealing with external stress and immobilizing heavy metal were explored. The results showed that heavy metals inhibited the ability of Pseudomonas sp. H13 and Brevundomonas sp. H16 to promote plant growth, but stimulated the production of extracellular polysaccharides and inorganic labile sulfide, and enhanced biofilm formation, thereby significantly improved the removal efficiency of Cu-2+, Zn2+, Cd2+, and Pb2+. Compared with H16, the biofilm of H13 disintegrated rapidly in the later stage, so more metal ions were adsorbed on the planktonic cells. The C-OH and P-O groups related to polysaccharides play a crucial role in heavy metal adsorption, and the immobilization mechanism of the planktonic cell is mainly ion exchange and group complex, but for H16, intracellular enrichment cannot be ignored. Functional group complexes played a dominant role in biofilm, and the immobilized heavy metals were more difficult to release into the environment. This study highlighted the potential application prospects of biofilm bacteria in heavy metal remediation and explained the reactive barrier of rhizosphere bacteria to heavy metals."
"C1 [Xing, Yonghui; Tan, Shuxin; Liu, Song; Xu, Shaozu; Wan, Wenjie; Huang, Qiaoyun; Chen, Wenli] Huazhong Agr Univ, State Key Lab Agr Microbiol, Wuhan 430070, Peoples R China."
"   [Huang, Qiaoyun] Huazhong Agr Univ, Minist Agr, Key Lab Arable Land Conservat Middle & Lower Reac, Wuhan 430070, Peoples R China."
C3 Huazhong Agricultural University; Huazhong Agricultural University
"RP Chen, WL (corresponding author), Huazhong Agr Univ, State Key Lab Agr Microbiol, Wuhan 430070, Peoples R China."
EM wlchen@mail.hzau.edu.cn
"RI Wan, Wenjie/AAT-7226-2021; Xing, Yonghui/NGS-3691-2025; Huang,"

"   Qiaoyun/A-8418-2012; Chen, Wenli/D-5625-2014"
"OI Wan, Wenjie/0000-0001-7150-6138; Tan, Shuxin/0000-0003-2614-6991; "
FU National Key Research and Develop-ment Program of China
   [2017YFA0605001]; Major Technology Innovation of Hubei Province
   [2018ABA092]
FX This study was funded by The National Key Research and Develop-ment
"   Program of China (2017YFA0605001) , and the Major Technology Innovation"
   of Hubei Province (2018ABA092) .
"CR Abdelkrim S, 2020, ECOTOX ENVIRON SAFE, V192, DOI 10.1016/j.ecoenv.2020.110260"
"   AIKING H, 1982, APPL ENVIRON MICROB, V44, P938, DOI 10.1128/AEM.44.4.938-944.1982"
"   Bai J, 2014, J ENVIRON SCI-CHINA, V26, P2056, DOI 10.1016/j.jes.2014.07.015"
"   Bolan N, 2014, J HAZARD MATER, V266, P141, DOI 10.1016/j.jhazmat.2013.12.018"
"   Burmolle M, 2007, MICROB ECOL, V54, P352, DOI 10.1007/s00248-007-9222-5"
"   Chen X, 2017, J HAZARD MATER, V325, P319, DOI 10.1016/j.jhazmat.2016.12.009"
"   Chi YW, 2020, SCI TOTAL ENVIRON, V741, DOI 10.1016/j.scitotenv.2020.140422"
"   Dimkpa CO, 2009, J APPL MICROBIOL, V107, P1687, DOI 10.1111/j.1365-2672.2009.04355.x"
"   Etesami H, 2018, ECOTOX ENVIRON SAFE, V147, P175, DOI 10.1016/j.ecoenv.2017.08.032"
"   Fang LC, 2011, J HAZARD MATER, V190, P810, DOI 10.1016/j.jhazmat.2011.03.122"
"   Feng XC, 2020, ENVIRON RES, V185, DOI 10.1016/j.envres.2020.109390"
"   Flemming HC, 2019, NAT REV MICROBIOL, V17, P247, DOI 10.1038/s41579-019-0158-9"
"   Gong YY, 2018, WATER RES, V147, P440, DOI 10.1016/j.watres.2018.10.024"
"   Guo JK, 2020, CHEMOSPHERE, V246, DOI 10.1016/j.chemosphere.2020.125823"
"   Guo Q, 2020, SOIL BIOL BIOCHEM, V144, DOI 10.1016/j.soilbio.2020.107755"
"   Gupta P, 2019, J HAZARD MATER, V377, P391, DOI 10.1016/j.jhazmat.2019.05.054"
"   Hashim MA, 2011, J ENVIRON MANAGE, V92, P2355, DOI 10.1016/j.jenvman.2011.06.009"
"   Huang F, 2020, J CLEAN PROD, V272, DOI 10.1016/j.jclepro.2020.122743"
"   Islam F, 2014, ECOTOX ENVIRON SAFE, V104, P285, DOI 10.1016/j.ecoenv.2014.03.008"
"   Jia XL, 2020, J HAZARD MATER, V393, DOI 10.1016/j.jhazmat.2020.122424"
"   Kamran MA, 2016, ECOTOX ENVIRON SAFE, V126, P256, DOI 10.1016/j.ecoenv.2016.01.002"
"   Lennon JT, 2016, ENVIRON MICROBIOL, V18, P2732, DOI 10.1111/1462-2920.13331"
"   Li ZY, 2014, SCI TOTAL ENVIRON, V468, P843, DOI 10.1016/j.scitotenv.2013.08.090"
"   Liu LW, 2018, SCI TOTAL ENVIRON, V633, P206, DOI 10.1016/j.scitotenv.2018.03.161"
"   Liu WR, 2020, SCI TOTAL ENVIRON, V734, DOI 10.1016/j.scitotenv.2020.139023"
"   Manoj SR, 2020, J ENVIRON MANAGE, V254, DOI 10.1016/j.jenvman.2019.109779"
"   Nell RM, 2017, GEOCHIM COSMOCHIM AC, V199, P210, DOI 10.1016/j.gca.2016.11.039"
"   Pabst MW, 2010, CHEMOSPHERE, V81, P904, DOI 10.1016/j.chemosphere.2010.07.069"
"   Pramanik K, 2021, CHEMOSPHERE, V274, DOI 10.1016/j.chemosphere.2021.129819"
"   Pramanik K, 2018, J HAZARD MATER, V351, P317, DOI 10.1016/j.jhazmat.2018.03.009"
"   Qu CC, 2019, ENVIRON INT, V131, DOI 10.1016/j.envint.2019.104995"
"   Rajkumar M, 2012, BIOTECHNOL ADV, V30, P1562, DOI 10.1016/j.biotechadv.2012.04.011"
"   Ren DW, 2015, ISME J, V9, P81, DOI 10.1038/ismej.2014.96"
"   SALT DE, 1995, BIO-TECHNOL, V13, P468, DOI 10.1038/nbt0595-468"
"   Sarwar N, 2017, CHEMOSPHERE, V171, P710, DOI 10.1016/j.chemosphere.2016.12.116"
"   Siripornadulsil S, 2013, ECOTOX ENVIRON SAFE, V94, P94, DOI 10.1016/j.ecoenv.2013.05.002"
"   Sule P, 2009, LETT APPL MICROBIOL, V49, P299, DOI 10.1111/j.1472-765X.2009.02659.x"
"   Tang YK, 2020, SCI TOTAL ENVIRON, V707, DOI 10.1016/j.scitotenv.2019.135867"
"   Toner B, 2005, ENVIRON SCI TECHNOL, V39, P8288, DOI 10.1021/es050528+"
"   Wang CR, 2020, CHEMOSPHERE, V252, DOI 10.1016/j.chemosphere.2020.126603"
"   Wei X, 2011, ENVIRON POLLUT, V159, P1369, DOI 10.1016/j.envpol.2011.01.006"
"   Xiao R, 2019, CHEMOSPHERE, V230, P510, DOI 10.1016/j.chemosphere.2019.05.121"
"   Xing YH, 2020, BIORESOURCE TECHNOL, V315, DOI 10.1016/j.biortech.2020.123742"
"   Xiong X, 2021, SCI TOTAL ENVIRON, V773, DOI 10.1016/j.scitotenv.2021.145069"
"   Xu SZ, 2020, ENVIRON POLLUT, V258, DOI 10.1016/j.envpol.2019.113774"
"   Xu SZ, 2020, CHEMOSPHERE, V240, DOI 10.1016/j.chemosphere.2019.124893"
"   Xu SZ, 2019, APPL MICROBIOL BIOT, V103, P3887, DOI 10.1007/s00253-019-09700-7"
"   Xu XJ, 2015, FRONT MICROBIOL, V6, DOI 10.3389/fmicb.2015.01422"
"   Zhang DY, 2010, J HAZARD MATER, V175, P359, DOI 10.1016/j.jhazmat.2009.10.011"
"   Zhang JH, 2019, ECOTOX ENVIRON SAFE, V176, P162, DOI 10.1016/j.ecoenv.2019.03.091"
"   Zhang RF, 2017, CURR OPIN MICROBIOL, V37, P8, DOI 10.1016/j.mib.2017.03.008"
NR 51
TC 37
Z9 46
U1 10
U2 214
PU ACADEMIC PRESS INC ELSEVIER SCIENCE
PI SAN DIEGO
"PA 525 B ST, STE 1900, SAN DIEGO, CA 92101-4495 USA"
SN 0013-9351
EI 1096-0953
J9 ENVIRON RES
JI Environ. Res.
PD MAY 1
PY 2022
VL 207
AR 112080
DI 10.1016/j.envres.2021.112080
EA JAN 2022
PG 9
"WC Environmental Sciences; Public, Environmental & Occupational Health"
WE Science Citation Index Expanded (SCI-EXPANDED)
"SC Environmental Sciences & Ecology; Public, Environmental & Occupational"
   Health
GA YU2SO
UT WOS:000751897600004

PM 34563529
DA 2026-04-06
ER

PT J
"AU Arroyo-Herrera, I"
"   Rom??n-Ponce, B"
"   Res??ndiz-Mart??nez, AL"
"   de los Santos, PE"
"   Wang, ET"
"   V??squez-Murrieta, MS"
"AF Arroyo-Herrera, Ivan"
"   Roman-Ponce, Brenda"
"   Resendiz-Martinez, Ana Laura"
"   de los Santos, Paulina"
"   Wang, En Tao"
"   Vasquez-Murrieta, Maria Soledad"
TI Heavy-metal resistance mechanisms developed by bacteria from
   Lerma-Chapala basin
SO ARCHIVES OF MICROBIOLOGY
LA English
DT Article
DE Heavy metals; Resistance mechanism; Plant growth-promoting traits; Novel
   species
ID RIBOSOMAL-RNA GENE; LAKE CHAPALA; SOLUBILIZING BACTERIA; HEXAVALENT
   CHROMIUM; ENDOPHYTIC BACTERIA; GROWTH; BIOREMEDIATION; PLANT;
   SIDEROPHORES; TOXICITY
"AB Heavy-metal (HM) contamination is a huge environmental problem in many countries including Mexico. Currently, microorganisms with multiple heavy-metal resistance and/or plant-promoting characteristics have been widely used for bioremediation of HM-contaminated soils. The aim of the study was isolated bacteria with multiple heavy-metal resistance and to determinate the resistance mechanism developed by these organisms. A total of 138 aerobic bacteria were isolated from soil and sediments surrounding the Lerma-Chapala basin located in the boundary of the States of Michoacan and Jalisco states of Mexico. One hundred and eight strains showed at least 1 plant growth-promoting features. The Lerma-Chapala basin bacteria were also resistant to high concentrations of HMs including the metalloid arsenic. Sequence analysis of 16S RNA genes reveled that these bacteria were mainly affiliated to the phyla Proteobacteria (38%), Firmicutes (31%) and Actinobacteria (25%), covering 21 genera with Bacillus as the most abundant one. Among them, at least 27 putative novel species were detected in the genera Acinetobacter, Arthrobacter, Bacillus, Agrobacterium, Dyadobacter, Enterobacter, Exiguobacterium, Kluyvera, Micrococcus, Microbacterium and Psychrobacter. In addition, these bacteria developed various heavy-metal-resistance mechanisms, such as biosorption/bioaccumulation, immobilization and detoxification. Therefore, the bacteria isolated from soils and sediments of Lerma-Chapala basin could be used in bioremediation strategies."
"C1 [Arroyo-Herrera, Ivan; Roman-Ponce, Brenda; Resendiz-Martinez, Ana Laura; de los Santos, Paulina; Wang, En Tao; Vasquez-Murrieta, Maria Soledad] Escuela Nacl Ciencias Biol, Inst Politecn Nacl, Prolongac Carpio & Plan de Ayala S-N, Mexico City 11340, DF, Mexico."
"   [Roman-Ponce, Brenda] Dept Microbiol & Genet, Edificio Dept,Lab 214,Campus Unamuno, Salamanca 37007, Spain."
"   [Roman-Ponce, Brenda] Univ Politecn Estado Morelos, Blvd Cuauhnahuac 556, Jiutepec 62550, Morelos, Mexico."
C3 Instituto Politecnico Nacional - Mexico; Universidad Autonoma del Estado
   de Morelos
"RP V??squez-Murrieta, MS (corresponding author), Escuela Nacl Ciencias Biol, Inst Politecn Nacl, Prolongac Carpio & Plan de Ayala S-N, Mexico City 11340, DF, Mexico."
EM murrieta@hotmail.com
"RI ; Vasquez Murrieta, Maria Soledad/IXD-3351-2023; Estrada de los Santos,"
   Paulina/S-1238-2018
"OI arroyo herrera, ivan/0000-0002-1148-6267; Vasquez Murrieta, Maria"
"   Soledad/0000-0002-3362-5408; Estrada de los Santos,"
   Paulina/0000-0003-2407-0284
"FU Consejo Nacional de Ciencia y Tecnologia (CONACyT) [SIP20140353,"
"   SIP20140354, SIP20150311, SIP20150358]; BEIFI-IPN; CONACyT [323687];"
   Comision de Operacion y Fomento de Actividades Academicas-IPN; Estimulos
   al Desempeno de los Investigadores-IPN; Sistema Nacional de
   Investigadores-CONACyT
"FX This study was financially supported by the projects SIP20140353,"
"   SIP20140354, SIP20150311 and SIP20150358. I. Arroyo-Herrera received"
   scholarships from the Consejo Nacional de Ciencia y Tecnologia (CONACyT)
   and BEIFI-IPN. B. Roman-Ponce received postdoctoral scholarships from
"   the CONACyT-323687. P. Estrada-de los Santos, E. T. Wang and M. S."
   Vasquez-Murrieta appreciate the scholarships of Comision de Operacion y
   Fomento de Actividades Academicas-IPN and Estimulos al Desempeno de los
   Investigadores-IPN and Sistema Nacional de Investigadores-CONACyT.
"CR Abhijit Gupta Abhijit Gupta, 2016, Journal of Microbial and Biochemical Technology, V8, P364, DOI 10.4172/1948-5948.1000310"
"   Agami RA, 2013, ECOTOX ENVIRON SAFE, V94, P164, DOI 10.1016/j.ecoenv.2013.04.013"
"   ALEXANDER DB, 1991, BIOL FERT SOILS, V12, P39, DOI 10.1007/BF00369386"
"   Ayangbenro AS, 2017, INT J ENV RES PUB HE, V14, DOI 10.3390/ijerph14010094"
"   Benidire L, 2016, ENVIRON SCI POLLUT R, V23, P21751, DOI 10.1007/s11356-016-7378-6"
"   BERRAQUERO F.R., 1976, ARS PHARM, V17, P399"
"   Bondarczuk K, 2013, CELL BIOL TOXICOL, V29, P397, DOI 10.1007/s10565-013-9262-1"
"   Branco R, 2004, CAN J MICROBIOL, V50, P697, DOI [10.1139/w04-048, 10.1139/W04-048]"
"   BROWN NL, 1992, PLASMID, V27, P41, DOI 10.1016/0147-619X(92)90005-U"
"   Bruins MR, 2000, ECOTOX ENVIRON SAFE, V45, P198, DOI 10.1006/eesa.1999.1860"
"   Campanella JJ, 2003, BMC BIOINFORMATICS, V4, DOI 10.1186/1471-2105-4-29"

"   Chun J, 2007, INT J SYST EVOL MICR, V57, P2259, DOI 10.1099/ijs.0.64915-0"
"   Guill??n-Jim??nez FD, 2008, J IND MICROBIOL BIOT, V35, P1277, DOI 10.1007/s10295-008-0425-7"
"   Dipak Paul Dipak Paul, 2017, Annals of Agrarian Science, V15, P130, DOI [10.1016/j.aasci.2016.10.001, 10.1016/j.aasci.2016.10.001]"
"   Etesami H, 2018, ECOTOX ENVIRON SAFE, V147, P175, DOI 10.1016/j.ecoenv.2017.08.032"
"   Fasim F, 2002, FEMS MICROBIOL LETT, V213, P1"
"   Ford TE, 2000, J ENVIRON SCI HEAL A, V35, P313, DOI 10.1080/10934520009376973"
"   Galtier N, 1996, COMPUT APPL BIOSCI, V12, P543"
"   Gavrilescu M, 2004, ENG LIFE SCI, V4, P219, DOI 10.1002/elsc.200420026"
"   Glick BR, 2010, BIOTECHNOL ADV, V28, P367, DOI 10.1016/j.biotechadv.2010.02.001"
"   GLICKMANN E, 1995, APPL ENVIRON MICROB, V61, P793, DOI 10.1128/AEM.61.2.793-796.1995"
"   Gong YH, 2014, PLOS ONE, V9, DOI 10.1371/journal.pone.0102823"
"   Hansen AM, 2001, LERMA-CHAPALA WATERSHED: EVALUATION AND MANAGEMENT, P95"
"   He LY, 2010, APPL SOIL ECOL, V44, P49, DOI 10.1016/j.apsoil.2009.09.004"
"   Hemme CL, 2010, ISME J, V4, P660, DOI 10.1038/ismej.2009.154"
"   Hern??ndez A, 1998, APPL ENVIRON MICROB, V64, P4317"
"   HOFFMAN CS, 1987, GENE, V57, P267, DOI 10.1016/0378-1119(87)90131-4"
"   Huang Y, 2018, J ENVIRON MANAGE, V207, P159, DOI 10.1016/j.jenvman.2017.10.072"
"   Hussain A, 2015, PAK J AGR SCI, V52, P915"
"   JAIN DK, 1985, CAN J MICROBIOL, V31, P206, DOI 10.1139/m85-039"
"   Jay JA, 2001, LERMA-CHAPALA WATERSHED: EVALUATION AND MANAGEMENT, P123"
"   Jia MR, 2019, CHEMOSPHERE, V218, P1061, DOI 10.1016/j.chemosphere.2018.11.145"
"   Carlos MHJ, 2016, MICROBIOL RES, V188, P53, DOI 10.1016/j.micres.2016.05.001"
"   Joutey NT, 2015, REV ENVIRON CONTAM T, V233, P45, DOI 10.1007/978-3-319-10479-9_2"
"   Kang CH, 2016, ECOL ENG, V89, P64, DOI 10.1016/j.ecoleng.2016.01.023"
"   Khatri N, 2015, FRONT LIFE SCI, V8, P23, DOI 10.1080/21553769.2014.933716"
"   Kraepiel AML, 2009, BIOMETALS, V22, P573, DOI 10.1007/s10534-009-9222-7"
"   Kwok CK, 2014, ECOTOX ENVIRON SAFE, V106, P62, DOI 10.1016/j.ecoenv.2014.04.016"
"   Lara P, 2017, ENVIRON SCI POLLUT R, V24, P25513, DOI 10.1007/s11356-016-7191-2"
"   Larkin MA, 2007, BIOINFORMATICS, V23, P2947, DOI 10.1093/bioinformatics/btm404"
"   Layton AC, 2014, GENOME ANNOUNCEMENTS, V2, DOI 10.1128/genomeA.01170-14"
"   Li WW, 2014, BIORESOURCE TECHNOL, V160, P15, DOI 10.1016/j.biortech.2013.11.074"
"   Lim SR, 2010, J HAZARD MATER, V177, P251, DOI 10.1016/j.jhazmat.2009.12.025"
"   Lin YB, 2012, ECOTOX ENVIRON SAFE, V77, P7, DOI 10.1016/j.ecoenv.2011.09.016"
"   Lukasz D, 2014, BIOMED RES INT, V2014, DOI 10.1155/2014/841892"
"   Ma Y, 2011, BIOTECHNOL ADV, V29, P248, DOI 10.1016/j.biotechadv.2010.12.001"
"   Ma Y, 2015, FRONT PLANT SCI, V5, DOI 10.3389/fpls.2014.00757"
"   Malaviya P, 2016, CRIT REV MICROBIOL, V42, P607, DOI 10.3109/1040841X.2014.974501"
"   Mallick I, 2014, ECOTOX ENVIRON SAFE, V107, P236, DOI 10.1016/j.ecoenv.2014.06.007"
"   MARZLUF GA, 1970, J BACTERIOL, V102, P716, DOI 10.1128/JB.102.3.716-721.1970"
"   Menna P, 2006, SYST APPL MICROBIOL, V29, P315, DOI 10.1016/j.syapm.2005.12.002"
"   Mestre R. J. E. Mestre R. J. E., 1997, Water International, V22, P140, DOI 10.1080/02508069708686693"
"   Mishra J, 2017, FRONT MICROBIOL, V8, DOI 10.3389/fmicb.2017.01706"
"   Mishra V, 2016, INT J PHYTOREMEDIAT, V18, P697, DOI 10.1080/15226514.2015.1131231"
"   Mumtaz MZ, 2017, MICROBIOL RES, V202, P51, DOI 10.1016/j.micres.2017.06.001"
"   Naik MM, 2013, ECOTOX ENVIRON SAFE, V98, P1, DOI 10.1016/j.ecoenv.2013.09.039"
"   Nair A, 2007, WATER AIR SOIL POLL, V180, P199, DOI 10.1007/s11270-006-9263-2"
"   Nautiyal CS, 1999, FEMS MICROBIOL LETT, V170, P265, DOI 10.1111/j.1574-6968.1999.tb13383.x"
"   Nies DH, 1999, APPL MICROBIOL BIOT, V51, P730, DOI 10.1007/s002530051457"
"   Nies DH, 2000, EXTREMOPHILES, V4, P77, DOI 10.1007/s007920050140"
"   OHTAKE H, 1987, J BACTERIOL, V169, P3853, DOI 10.1128/jb.169.8.3853-3856.1987"
"   Olaniran AO, 2013, INT J MOL SCI, V14, P10197, DOI 10.3390/ijms140510197"
"   Ontiveros-Cuadras JF, 2014, SCI TOTAL ENVIRON, V468, P677, DOI 10.1016/j.scitotenv.2013.08.071"
"   Ort??zar M, 2020, SCI TOTAL ENVIRON, V739, DOI 10.1016/j.scitotenv.2020.139850"
"   Pande Amit, 2017, Journal of Genetic Engineering and Biotechnology, V15, P379, DOI [10.1016/j.jgeb.2017.06.005, 10.1016/j.jgeb.2017.06.005]"
"   PEPI M, 1992, BIOMETALS, V5, P179, DOI 10.1007/BF01061326"
"   P??rez-Miranda S, 2007, J MICROBIOL METH, V70, P127, DOI 10.1016/j.mimet.2007.03.023"
"   Pishchik VN, 2016, MICROBIOLOGY+, V85, P257, DOI 10.1134/S0026261716030097"
"   PUMPEL T, 1995, J IND MICROBIOL, V14, P213, DOI 10.1007/BF01569930"
"   Qamar N, 2017, J APPL MICROBIOL, V123, P748, DOI 10.1111/jam.13535"
"   Rajkumar M, 2010, TRENDS BIOTECHNOL, V28, P142, DOI 10.1016/j.tibtech.2009.12.002"
"   Ram??rez-D??az MI, 2008, BIOMETALS, V21, P321, DOI 10.1007/s10534-007-9121-8"
"   Rathnayake IVN, 2013, CHEMOSPHERE, V90, P1195, DOI 10.1016/j.chemosphere.2012.09.036"
"   Ray L., 2006, J HAZARD SUBST RES, DOI [10.4148/1090-7025.1032, DOI 10.4148/1090-7025.1032]"
"   Retamal-Morales G, 2018, ECOTOX ENVIRON SAFE, V157, P176, DOI 10.1016/j.ecoenv.2018.03.087"
"   Rodriguez E, 2012, PLANT PHYSIOL BIOCH, V53, P94, DOI 10.1016/j.plaphy.2012.01.013"
"   Rom??n-Ponce B, 2018, ARCH MICROBIOL, V200, P883, DOI 10.1007/s00203-018-1495-1"
"   Rom??n-Ponce B, 2016, ARCH MICROBIOL, V198, P941, DOI 10.1007/s00203-016-1252-2"
"   Saito MA, 2002, LIMNOL OCEANOGR, V47, P1629, DOI 10.4319/lo.2002.47.6.1629"
"   Saravanan VS, 2011, BACTERIA IN AGROBIOLOGY: PLANT NUTRIENT MANAGEMENT, P47, DOI 10.1007/978-3-642-21061-7_3"
"   Schalk IJ, 2011, ENVIRON MICROBIOL, V13, P2844, DOI 10.1111/j.1462-2920.2011.02556.x"
"   SCHWYN B, 1987, ANAL BIOCHEM, V160, P47, DOI 10.1016/0003-2697(87)90612-9"
"   Sharma SK, 2012, J MICROBIOL BIOTECHN, V22, P352, DOI 10.4014/jmb.1106.05063"
"   Silva Filho G. N., 2000, Revista Brasileira de Ciencia do Solo, V24, P311, DOI 10.1590/S0100-06832000000200008"
"   Simeonova DD, 2004, FEMS MICROBIOL LETT, V237, P249, DOI 10.1111/j.1574-6968.2004.tb09703.x"
"   Soufiane B, 2013, ANTON LEEUW INT J G, V103, P195, DOI 10.1007/s10482-012-9800-5"
"   Stackebrandt E., 2006, Microbiology Today, V33, P152"
"   Stong T, 2013, J ENVIRON SCI HEAL A, V48, P1835, DOI 10.1080/10934529.2013.823340"
"   Tamura K, 2011, MOL BIOL EVOL, V28, P2731, DOI 10.1093/molbev/msr121"
"   Thatoi H, 2014, J ENVIRON MANAGE, V146, P383, DOI 10.1016/j.jenvman.2014.07.014"
"   Torres Z, 2016, B ENVIRON CONTAM TOX, V97, P418, DOI 10.1007/s00128-016-1892-6"

"   Torres Z, 2014, ENVIRON SCI TECHNOL, V48, P6359, DOI 10.1021/es4048076"
"   Trasande L, 2010, ENVIRON HEALTH-GLOB, V9, DOI 10.1186/1476-069X-9-1"
"   VALLEE BL, 1993, PHYSIOL REV, V73, P79, DOI 10.2466/pr0.1993.73.1.79"
"   Veglio F, 1997, PROCESS BIOCHEM, V32, P99, DOI 10.1016/S0032-9592(96)00047-7"
"   Verma P, 2019, SAUDI J BIOL SCI, V26, P1882, DOI 10.1016/j.sjbs.2016.01.042"
"   Vinuesa P, 2005, SYST APPL MICROBIOL, V28, P702, DOI 10.1016/j.syapm.2005.05.007"
"   Wani PA, 2007, CHEMOSPHERE, V70, P36, DOI 10.1016/j.chemosphere.2007.07.028"
"   Weeger W, 1999, BIOMETALS, V12, P141, DOI 10.1023/A:1009255012328"
"   Wu C, 2018, ENVIRON SCI POLLUT R, V25, P20792, DOI 10.1007/s11356-018-2268-8"
"   Wu Q, 2013, J APPL MICROBIOL, V114, P713, DOI 10.1111/jam.12093"
"   Wuana R.A., 2011, ISRN Ecology, DOI [10.5402/2011/402647, DOI 10.5402/2011/402647]"
"   Yan G, 2019, CURR GENET, V65, P329, DOI 10.1007/s00294-018-0894-9"
NR 103
TC 19
Z9 20
U1 3
U2 38
PU SPRINGER HEIDELBERG
PI HEIDELBERG
"PA TIERGARTENSTRASSE 17, D-69121 HEIDELBERG, GERMANY"
SN 0302-8933
EI 1432-072X
J9 ARCH MICROBIOL
JI Arch. Microbiol.
PD MAY
PY 2021
VL 203
IS 4
BP 1807
EP 1823
DI 10.1007/s00203-020-02140-2
EA JAN 2021
PG 17
WC Microbiology
WE Science Citation Index Expanded (SCI-EXPANDED)
SC Microbiology
GA RP0HM
UT WOS:000610914800001
PM 33491161
DA 2026-04-06
ER

PT J
"AU Gao, JM"
"   Jia, ZZ"
"   Song, LZ"
"   Yang, YX"
"   Liu, J"
"   Han, Y"
"   Ma, LJ"
"   Ma, L"
"AF Gao, Jianmin"
"   Jia, Zhenzhen"
"   Song, Leizhuang"
"   Yang, Yuxian"
"   Liu, Jie"
"   Han, Yi"
"   Ma, Linjing"
"   Ma, Lei"
TI Enhancing composting efficiency and heavy metal stabilization through
   thermophilic microbial inoculation
SO ENVIRONMENTAL TECHNOLOGY
LA English
DT Article
DE Composting; humification; heavy metal; microbial inoculation;
   thermophiles
ID HUMIFICATION; COMMUNITY
"AB Agricultural waste management is critical for reducing environmental pollution and enhancing soil health, particularly the treatment of organic waste containing heavy metals. This study investigates the distinct effects of mesophilic and thermophilic microbial inoculation on composting. Using rice straw and swine manure as feedstocks, composting piles were inoculated with mesophilic (Bacillus subtilis and Trichoderma reesei) or thermophilic (Geobacillus stearothermophilus and Aspergillus fumigatus) microorganisms, alongside a control group with no inoculation. The results revealed that thermophilic inoculation significantly enhanced organic degradation and humification, leading to more efficient stabilization of heavy metals such as Pb, Zn, and Cr, especially during the thermophilic and mature phases. Microbial community analysis showed that thermophilic inoculation created a more connected microbial network and boosted the microbial functionality. Spearman correlation analysis indicated that the enhanced key metabolic pathways of IT, particularly those involved in organic matter degradation and heavy metal detoxification, were associated with reduced metal bioavailability during thermophilic phase. These findings highlighting the potential of thermophilic inoculants for sustainable waste management and environmental protection."
"C1 [Gao, Jianmin; Yang, Yuxian; Liu, Jie; Han, Yi; Ma, Lei] Henan Univ Urban Construct, Coll Life Sci & Engn, Pingdingshan, Peoples R China."
"   [Jia, Zhenzhen; Song, Leizhuang] Pingdingshan Acad Agr Sci, Pingdingshan, Peoples R China."
"   [Ma, Linjing] Nanyang Acad Sci, Nanyang 473000, Henan, Peoples R China."

C3 Henan University of Urban Construction
"RP Ma, LJ (corresponding author), Nanyang Acad Sci, Nanyang 473000, Henan, Peoples R China."
EM 15936157679@163.com
"RI han, yi/F-7602-2016"
FU Henan Province science and technology attack project [242102110367];
"   Natural Science Foundation of Henan [242300421555, 252300420651]; Henan"
   Province Key Research Projects Plan for Higher Education Institutions
   [24B550001]
FX This research was financially supported by the Henan Province science
"   and technology attack project (242102110367), Natural Science Foundation"
"   of Henan (242300421555, 252300420651), and Henan Province Key Research"
   Projects Plan for Higher Education Institutions (24B550001).
"CR Abdellah YAY, 2024, BIOCHAR, V6, DOI 10.1007/s42773-024-00347-w"
"   Abdellah YAY, 2022, ENVIRON POLLUT, V307, DOI 10.1016/j.envpol.2022.119549"
"   Bao JF, 2022, BIORESOURCE TECHNOL, V362, DOI 10.1016/j.biortech.2022.127797"
"   Barthod J, 2018, AGRON SUSTAIN DEV, V38, DOI 10.1007/s13593-018-0491-9"
"   Bolyen E, 2019, NAT BIOTECHNOL, V37, P852, DOI 10.1038/s41587-019-0209-9"
"   Cao Y, 2022, J ENVIRON MANAGE, V316, DOI 10.1016/j.jenvman.2022.115215"
"   Capra EJ, 2012, ANNU REV MICROBIOL, V66, P325, DOI 10.1146/annurev-micro-092611-150039"
"   Castro C, 2019, BIORESOURCE TECHNOL, V279, P317, DOI 10.1016/j.biortech.2019.02.028"
"   Chang RX, 2019, J ENVIRON MANAGE, V230, P119, DOI 10.1016/j.jenvman.2018.09.076"
"   Chen HX, 2017, APPL SURF SCI, V425, P728, DOI 10.1016/j.apsusc.2017.07.053"
"   Chen L, 2023, WASTE MANAGE, V158, P93, DOI 10.1016/j.wasman.2022.12.040"
"   Chen XM, 2020, J ENVIRON MANAGE, V272, DOI 10.1016/j.jenvman.2020.111109"
"   Comel J, 2021, J ENVIRON CHEM ENG, V9, DOI 10.1016/j.jece.2021.105450"
"   Cui H, 2020, WASTE MANAGE, V118, P360, DOI 10.1016/j.wasman.2020.08.043"
"   Dan YT, 2023, ENVIRON POLLUT, V320, DOI 10.1016/j.envpol.2023.121109"
"   Douglas GM, 2020, NAT BIOTECHNOL, V38, P685, DOI 10.1038/s41587-020-0548-6"
"   Du S, 2023, ENVIRON INT, V172, DOI 10.1016/j.envint.2023.107789"
"   El-Shazly MM., 2020, ROLE TRICHODERMA SPP"
"   Finore I, 2023, CHEM BIOL TECHNOL AG, V10, DOI 10.1186/s40538-023-00381-z"
"   Fu Y, 2021, J HAZARD MATER, V417, DOI 10.1016/j.jhazmat.2021.126041"
"   del Pulgar EMG, 2014, CRIT REV MICROBIOL, V40, P236, DOI 10.3109/1040841X.2013.776512"
"   Islam MA, 2020, SEP PURIF TECHNOL, V247, DOI 10.1016/j.seppur.2020.116949"
"   Kong XL, 2023, J ENVIRON MANAGE, V326, DOI 10.1016/j.jenvman.2022.116747"
"   Kong YL, 2024, CHEM ENG J, V489, DOI 10.1016/j.cej.2024.151386"
"   Koyama M, 2018, BIORESOURCE TECHNOL, V265, P207, DOI 10.1016/j.biortech.2018.05.109"
"   Lashermes G, 2010, CHEMOSPHERE, V79, P455, DOI 10.1016/j.chemosphere.2010.01.041"
"   Li CN, 2021, J HAZARD MATER, V415, DOI 10.1016/j.jhazmat.2021.125559"
"   Li HJ, 2024, J ENVIRON MANAGE, V353, DOI 10.1016/j.jenvman.2024.120145"
"   Li T, 2024, CHEM ENG J, V502, DOI 10.1016/j.cej.2024.158087"
"   Li XN, 2022, J HAZARD MATER, V427, DOI 10.1016/j.jhazmat.2021.127915"
"   Li Y, 2023, FRONT MICROBIOL, V14, DOI 10.3389/fmicb.2023.1281633"
"   Liu QM, 2023, CHEM ENG J, V451, DOI 10.1016/j.cej.2022.139087"
"   LIU Y, 2022, SCI TOTAL ENVIRON, V836, DOI DOI 10.1016/J.SCITOTENV.2022.155727"
"   Luisa BG., 2012, CELLULAR ENERGY META"
"   Marano RBM, 2021, ENVIRON SCI TECHNOL, V55, P6814, DOI 10.1021/acs.est.1c00612"
"   Quast C, 2013, NUCLEIC ACIDS RES, V41, pD590, DOI 10.1093/nar/gks1219"
"   Sahu A, 2019, J ENVIRON MANAGE, V244, P144, DOI 10.1016/j.jenvman.2019.04.015"
"   Sarlaki E, 2024, CHEM ENG J, V498, DOI 10.1016/j.cej.2024.155521"
"   Shan GC, 2024, J HAZARD MATER, V471, DOI 10.1016/j.jhazmat.2024.134398"
"   Shu WH, 2021, J HAZARD MATER, V414, DOI 10.1016/j.jhazmat.2021.125520"
"   Sun Y, 2023, FRONT MICROBIOL, V14, DOI 10.3389/fmicb.2023.1145784"
"   Town RM, 2018, ENVIRON SCI TECHNOL, V52, P11682, DOI 10.1021/acs.est.8b02896"
"   Valle-P??rez AU, 2021, WASTE BIOMASS VALORI, V12, P5885, DOI 10.1007/s12649-021-01397-y"
"   Wang CQ, 2024, GLOBAL CHANGE BIOL, V30, DOI 10.1111/gcb.17349"
"   Wang F, 2022, WASTE MANAGE, V149, P333, DOI 10.1016/j.wasman.2022.06.015"
"   Wang MM, 2022, BIORESOURCE TECHNOL, V354, DOI 10.1016/j.biortech.2022.127195"
"   Wei YQ, 2015, BIORESOURCE TECHNOL, V189, P349, DOI 10.1016/j.biortech.2015.04.031"
"   Wichuk KM, 2011, COMPOST SCI UTIL, V19, P244, DOI 10.1080/1065657x.2011.10737008"
"   Wu D, 2024, IND CROP PROD, V208, DOI 10.1016/j.indcrop.2023.117838"
"   Xu JQ, 2019, J ENVIRON MANAGE, V243, P240, DOI 10.1016/j.jenvman.2019.05.008"
"   Yang C, 2020, CHINESE CHEM LETT, V31, P2693, DOI 10.1016/j.cclet.2020.04.001"
"   Yang F, 2021, CHEM SOC REV, V50, P6221, DOI 10.1039/d0cs01363c"
"   Zhang J, 2024, SCI TOTAL ENVIRON, V927, DOI 10.1016/j.scitotenv.2024.172304"
"   Zhang LX, 2023, ENVIRON TECHNOL INNO, V32, DOI 10.1016/j.eti.2023.103306"
"   Zhang LL, 2021, J HAZARD MATER, V416, DOI 10.1016/j.jhazmat.2021.126141"
"   Zhang WM, 2020, BIORESOURCE TECHNOL, V297, DOI 10.1016/j.biortech.2019.122410"
"   Zhao YX, 2022, WASTE MANAGE, V144, P11, DOI 10.1016/j.wasman.2022.03.006"
"   Zhong XB, 2023, SCI TOTAL ENVIRON, V903, DOI 10.1016/j.scitotenv.2023.166274"
"   Zhou YW, 2021, J HAZARD MATER, V420, DOI 10.1016/j.jhazmat.2021.126562"
"   Zhu JJ, 2019, NAT REV MOL CELL BIO, V20, P436, DOI 10.1038/s41580-019-0123-5"
"   Zhu N, 2021, BIORESOURCE TECHNOL, V319, DOI 10.1016/j.biortech.2020.124149"
NR 61
TC 0
Z9 0
U1 19
U2 19
PU TAYLOR & FRANCIS LTD
PI ABINGDON
"PA 2-4 PARK SQUARE, MILTON PARK, ABINGDON OR14 4RN, OXON, the United Kingdom"
SN 0959-3330
EI 1479-487X
J9 ENVIRON TECHNOL

JI Environ. Technol.
PD JAN 15
PY 2026
VL 47
IS 2
BP 242
EP 257
DI 10.1080/09593330.2025.2573836
EA OCT 2025
PG 16
WC Environmental Sciences
WE Science Citation Index Expanded (SCI-EXPANDED)
SC Environmental Sciences & Ecology
GA AW7NU
UT WOS:001599947900001
PM 41139227
DA 2026-04-06
ER

PT J
"AU Shan, SP"
"   Guo, ZH"
"   Lei, P"
"   Li, YL"
"   Wang, YS"
"   Zhang, M"
"   Cheng, W"
"   Wu, SD"
"   Wu, MX"
"   Du, DX"
"AF Shan, Shiping"
"   Guo, Zhaohui"
"   Lei, Ping"
"   Li, Yilu"
"   Wang, Yushuang"
"   Zhang, Min"
"   Cheng, Wei"
"   Wu, Shandong"
"   Wu, Minxi"
"   Du, Dongxia"
TI Increased biomass and reduced tissue cadmium accumulation in rice via
   indigenous Citrobacter sp. XT1-2-2 and its mechanisms
SO SCIENCE OF THE TOTAL ENVIRONMENT
LA English
DT Article
DE Microbial remediation; Heavy metals; Citrobacter; Rice biomass; Cd
   speciation
ID HEAVY-METAL RESISTANCE; ORYZA-SATIVA L.; CD ACCUMULATION; SOIL;
   BACTERIA; BIOSORPTION; BIOREMEDIATION; TOLERANT; REMOVAL; MINE
"AB Microbial remediation is a promising technique to remediate heavy metals contaminated soils. In this study, the cadmium (Cd)- resistant Citrobacter sp. XT1-2-2, isolated from heavy metals contaminated paddy soils, was investigated to evaluate the effect of this strain on soil Cd speciation, cellular Cd distribution, tissue Cd accumulation and rice biomass. The percentage of Cd2+ removal by Citrobacter sp. XT1-2-2 was up to 82.3 +/- 2.1% within 240 min in the solution. The average content of soil soluble plus exchangeable and carbonate-bound fractions of Cd decreased, whereas Fe/Mn oxide-bound, organic matter-bound and residual fractions increased with bacteria inoculation. For the paddy soil inoculated with the XT1-2-2 strain, Cd concentrations of roots, culms, leaves and grains were significantly reduced by 24.1%, 46.9%, 41.5% and 66.7%, respectively. In addition, inoculation bacteria significantly increased the biomass of the roots, above-ground tissues and the rice grains. All results indicated that the XT1-2-2 strain had the ability to immobilize soil Cd and decrease Cd accumulation in rice grains. Therefore, the XT1-2-2 strain has potential for application to remediate Cd-contaminated paddy soils. It is possible to exploit a new bacterial-assisted technique for the remediation in Cd-contaminated paddy soils. (C) 2019 Elsevier B.V. All rights reserved."
"C1 [Shan, Shiping; Guo, Zhaohui; Lei, Ping; Li, Yilu; Wang, Yushuang; Zhang, Min; Cheng, Wei; Wu, Shandong; Wu, Minxi; Du, Dongxia] Hunan Inst Microbiol, Changsha 410009, Hunan, Peoples R China."
"   [Shan, Shiping; Guo, Zhaohui; Cheng, Wei] Hunan Engn Res Ctr Safe & Efficient Utilizat Heav, Changsha 410083, Hunan, Peoples R China."
"RP Guo, ZH; Du, DX (corresponding author), Hunan Inst Microbiol, Changsha 410009, Hunan, Peoples R China."
EM 157263033@qq.com; xiaoping310@126.com
"RI min, zhang/IQU-8773-2023; cheng, wei/V-4156-2017"
FU Changsha Municipal Science and Technology Major Project [kq1804016];
   Science Research Project of the Science and Technology Department in
   Hunan Province [2017NK2144]; Hunan Agricultural Science and Technology
   Innovation Alliance Project [2017LM0305]
FX This project was supported by Changsha Municipal Science and Technology
"   Major Project (kq1804016), Science Research Project of the Science and"
   Technology Department in Hunan Province (2017NK2144) and Hunan
   Agricultural Science and Technology Innovation Alliance Project
   (2017LM0305).
"CR Alam MZ, 2011, ENVIRON MONIT ASSESS, V178, P281, DOI 10.1007/s10661-010-1689-8"
"   [Anonymous], MANUAL SOIL CHEM FER"
"   [Anonymous], 2014, CHINAS OCEAN DEV REP"
"   Ayangbenro AS, 2017, INT J ENV RES PUB HE, V14, DOI 10.3390/ijerph14010094"
"   Aziz R, 2015, J AGR FOOD CHEM, V63, P3599, DOI 10.1021/jf505557g"

"   Bai HJ, 2008, BIORESOURCE TECHNOL, V99, P7716, DOI 10.1016/j.biortech.2008.01.071"
"   Chen Z, 2017, ENVIRON POLLUT, V231, P549, DOI 10.1016/j.envpol.2017.08.055"
"   Choudhary S, 2009, BIORESOURCE TECHNOL, V100, P2482, DOI 10.1016/j.biortech.2008.12.015"
"   Dixit R, 2015, SUSTAINABILITY-BASEL, V7, P2189, DOI 10.3390/su7022189"
"   DUDKA S, 1994, WATER AIR SOIL POLL, V76, P333, DOI 10.1007/BF00482710"
"   Felske A, 1999, FEMS MICROBIOL ECOL, V30, P137, DOI 10.1111/j.1574-6941.1999.tb00642.x"
"   Hadia NMA, 2014, J NANOSCI NANOTECHNO, V14, P5449, DOI 10.1166/jnn.2014.8917"
"   Hu YL, 2009, PLANT GROWTH REGUL, V59, P51, DOI 10.1007/s10725-009-9387-7"
"   Huang YZ., 2009, Acta Ecologica Sinica, V29, P320, DOI [DOI 10.1016/J.CHNAES.2009.09.011, 10.1016/j.chnaes.2009.09.011]"
"   Kiran MG, 2017, J HAZARD MATER, V324, P62, DOI 10.1016/j.jhazmat.2015.12.042"
"   KUYUCAK N, 1989, BIOTECHNOL BIOENG, V33, P823, DOI 10.1002/bit.260330705"
"   Lebeau T, 2002, SCI TOTAL ENVIRON, V291, P73, DOI 10.1016/S0048-9697(01)01093-2"
"   Lee PK, 2015, CHEMOSPHERE, V119, P1411, DOI 10.1016/j.chemosphere.2014.10.028"
"   Li FL, 1998, ENVIRON POLLUT, V102, P269, DOI 10.1016/S0269-7491(98)00063-3"
"   Li Y, 2017, ECOTOX ENVIRON SAFE, V138, P56, DOI 10.1016/j.ecoenv.2016.12.024"
"   Liang J, 2017, CHEMOSPHERE, V189, P198, DOI 10.1016/j.chemosphere.2017.09.046"
"   Liao SJ, 2011, ENVIRON EARTH SCI, V62, P1047, DOI 10.1007/s12665-010-0593-4"
"   Limcharoensuk T, 2015, ECOTOX ENVIRON SAFE, V122, P322, DOI 10.1016/j.ecoenv.2015.08.013"
"   Lin XY, 2016, SCI TOTAL ENVIRON, V569, P97, DOI 10.1016/j.scitotenv.2016.06.121"
"   Liu YL, 2018, ECOTOX ENVIRON SAFE, V163, P223, DOI 10.1016/j.ecoenv.2018.07.081"
"   Loukidou MX, 2003, WATER RES, V37, P4544, DOI 10.1016/S0043-1354(03)00415-9"
"   Luo SL, 2014, J HAZARD MATER, V274, P145, DOI 10.1016/j.jhazmat.2014.03.062"
"   MACASKIE LE, 1987, BIOTECHNOL BIOENG, V30, P66, DOI 10.1002/bit.260300110"
"   Onwudiwe DC, 2015, SPECTROCHIM ACTA A, V135, P1080, DOI 10.1016/j.saa.2014.08.004"
"   Peng WH, 2018, CHEMOSPHERE, V197, P33, DOI 10.1016/j.chemosphere.2018.01.017"
"   Punjee P, 2018, CAN J MICROBIOL, V64, P131, DOI 10.1139/cjm-2017-0198"
"   Rajkumar M, 2013, J ENVIRON MANAGE, V128, P973, DOI 10.1016/j.jenvman.2013.07.001"
"   Shan SP, 2019, ECOTOX ENVIRON SAFE, V169, P292, DOI 10.1016/j.ecoenv.2018.11.030"
"   Shi XY, 2018, J HAZARD MATER, V357, P431, DOI 10.1016/j.jhazmat.2018.06.027"
"   Shuman LM, 1999, J ENVIRON QUAL, V28, P1442, DOI 10.2134/jeq1999.00472425002800050008x"
"   Siripornadulsil S, 2013, ECOTOX ENVIRON SAFE, V94, P94, DOI 10.1016/j.ecoenv.2013.05.002"
"   Suksabye P, 2016, ENVIRON SCI POLLUT R, V23, P962, DOI 10.1007/s11356-015-4590-8"
"   Sun YB, 2016, ENVIRON POLLUT, V208, P739, DOI 10.1016/j.envpol.2015.10.054"
"   TAKAHASHI M, 1993, J ELECTROANAL CHEM, V359, P281, DOI 10.1016/0022-0728(93)80415-E"
"   Tangaromsuk J, 2002, BIORESOURCE TECHNOL, V85, P103, DOI 10.1016/S0960-8524(02)00066-4"
"   TESSIER A, 1979, ANAL CHEM, V51, P844, DOI 10.1021/ac50043a017"
"   Voica C, 2012, ROM REP PHYS, V64, P221"
"   Volesky B, 2001, HYDROMETALLURGY, V59, P203, DOI 10.1016/S0304-386X(00)00160-2"
"   Wang Q, 2018, ECOTOX ENVIRON SAFE, V148, P269, DOI 10.1016/j.ecoenv.2017.10.036"
"   Wang XY, 2018, J ENVIRON MANAGE, V226, P194, DOI 10.1016/j.jenvman.2018.08.042"
"   Xu SZ, 2019, APPL MICROBIOL BIOT, V103, P3887, DOI 10.1007/s00253-019-09700-7"
"   Xu XJ, 2015, FRONT MICROBIOL, V6, DOI 10.3389/fmicb.2015.01422"
"   Xue DW, 2014, J HAZARD MATER, V280, P269, DOI 10.1016/j.jhazmat.2014.08.010"
"   Yu H, 2006, SCI TOTAL ENVIRON, V370, P302, DOI 10.1016/j.scitotenv.2006.06.013"
"   Zhou GT, 2016, WATER AIR SOIL POLL, V227, DOI 10.1007/s11270-016-3040-7"
NR 50
TC 45
Z9 52
U1 0
U2 139
PU ELSEVIER
PI AMSTERDAM
"PA RADARWEG 29, 1043 NX AMSTERDAM, NETHERLANDS"
SN 0048-9697
EI 1879-1026
J9 SCI TOTAL ENVIRON
JI Sci. Total Environ.
PD MAR 15
PY 2020
VL 708
AR 135224
DI 10.1016/j.scitotenv.2019.135224
PG 11
WC Environmental Sciences
WE Science Citation Index Expanded (SCI-EXPANDED)
SC Environmental Sciences & Ecology
GA KB0TV
UT WOS:000506214900051
PM 31796275
DA 2026-04-06
ER

PT J
"AU Jacob, JM"
"   Karthik, C"
"   Saratale, RG"
"   Kumar, SS"
"   Prabakar, D"
"   Kadirvelu, K"
"   Pugazhendhi, A"
"AF Jacob, Jaya Mary"
"   Karthik, Chinnannan"
"   Saratale, Rijuta Ganesh"

"   Kumar, Smita S."
"   Prabakar, Desika"
"   Kadirvelu, K."
   Arivalagan Pugazhendhi
TI Biological approaches to tackle heavy metal pollution: A survey of
   literature
SO JOURNAL OF ENVIRONMENTAL MANAGEMENT
LA English
DT Review
DE Heavy metals; Bioremediation; Microorganisms; Reduction; Adsorption
ID POLYCYCLIC AROMATIC-HYDROCARBONS; AQUEOUS-SOLUTION; HEXAVALENT CHROMIUM;
   CR(VI) REDUCTION; CADMIUM(II) BIOSORPTION; UREOLYTIC BACTERIA;
   PHENOLIC-COMPOUNDS; CONTAMINATED SOIL; PROTEIN-QUALITY; AQUATIC PLANTS
"AB Pollution by heavy metals has been identified as a global threat since the inception of industrial revolution. Heavy metal contamination induces serious health and environmental hazards due to its toxic nature. Remediation of heavy metals by conventional methods is uneconomical and generates a large quantity of secondary wastes. On the other hand, biological agents such as plants, microorganisms etc. offer easy and eco-friendly ways for metal removal; hence, considered as efficient and alternative tools for metal removal. Bioremediation involves adsorption, reduction or removal of contaminants from the environment through biological resources (both microorganisms and plants). The heavy metal remediation properties of microorganisms stem from their self defense mechanisms such as enzyme secretion, cellular morphological changes etc. These defence mechanisms comprise the active involvement of microbial enzymes such as oxidoreductases, oxygenases etc, which influence the rates of bioremediation. Further, immobilization techniques are improving the practice at industrial scales. This article summarizes the various strategies inherent in the biological sorption and remediation of heavy metals. (C) 2018 Elsevier Ltd. All rights reserved."
"C1 [Jacob, Jaya Mary] APJ Abdul Kalam Kerala Technol Univ, Sree Buddha Coll Engn, Dept Biotechnol & Biochem Engn, Thiruvananthapuram, Kerala, India."
"   [Karthik, Chinnannan; Kadirvelu, K.] Bharathiar Univ Campus, DRDO BU CLS, Coimbatore 46, Tamil Nadu, India."
"   [Saratale, Rijuta Ganesh] Dongguk Univ Seoul, Res Inst Biotechnol & Med Converged Sci, Goyang Si, Gyeonggido, South Korea."
"   [Kumar, Smita S.] Indian Inst Technol Delhi, Ctr Rural Dev & Technol, Delhi 110016, India."
"   [Prabakar, Desika] Anna Univ, ACT Campus, Madras, Tamil Nadu, India."
"   [Arivalagan Pugazhendhi] Ton Duc Thang Univ, Fac Environm & Labour Safety, Innovat Green Prod Synth & Renewable Environm Dev, Ho Chi Minh City, Vietnam."
C3 Bharathiar University; Dongguk University; Indian Institute of
   Technology System (IIT System); Indian Institute of Technology (IIT) -
   Delhi; Anna University; Anna University Chennai; Ton Duc Thang
   University
"RP Pugazhendhi, A (corresponding author), Ton Duc Thang Univ, Fac Environm & Labour Safety, Innovat Green Prod Synth & Renewable Environm Dev, Ho Chi Minh City, Vietnam."
EM arivalagan.pugazhendhi@tdt.edu.vn
"RI Dakshinamoorthy, Prabakar/ACU-1739-2022; Kumar, Smita/O-4532-2019;"
"   Chinnannan, Karthik/KCK-4224-2024; Pugazhendhi, Arivalagan/F-5411-2018;"
"   kadirvelu, krishnagounder/ABE-4918-2020"
"OI Kumar, Smita/0000-0002-9294-7634; Chinnannan,"
"   Karthik/0000-0001-6548-4083; Pugazhendhi,"
"   Arivalagan/0000-0002-9529-3306; kadirvelu,"
   krishnagounder/0000-0001-7912-8282
"FU Science and Engineering Research Board (SERB) [PDF/2016/002058,"
   PDF/2017/002783]
FX Chinnannan Karthik and Smita S Kumar were thankful to the Science and
   Engineering Research Board (SERB) for providing National Post-doctoral
   Fellowship (File No: PDF/2016/002058 and PDF/2017/002783).
"CR Abbas S.H., 2014, Journal of Chemical Science and Technology, V3, P74"
"   Ajjabi LC, 2009, J ENVIRON MANAGE, V90, P3485, DOI 10.1016/j.jenvman.2009.06.001"
"   Akar T, 2005, BIOCHEM ENG J, V25, P227, DOI 10.1016/j.bej.2005.05.006"
"   Akhtar N, 2003, BIORESOURCE TECHNOL, V88, P163, DOI 10.1016/S0960-8524(02)00289-4"
"   Aksu Z, 2001, SEP PURIF TECHNOL, V21, P285, DOI 10.1016/S1383-5866(00)00212-4"
"   Ali H, 2013, CHEMOSPHERE, V91, P869, DOI 10.1016/j.chemosphere.2013.01.075"
"   [Anonymous], 2013, Thermophilic Microbes in Environmental and Industrial Biotechnology, DOI [10.1007/978-94-007-5899-5_6, DOI 10.1007/978-94-007-5899-5_6]"
"   Arivalagan P, 2014, ECOL ENG, V71, P728, DOI 10.1016/j.ecoleng.2014.08.005"
"   Ayangbenro AS, 2017, INT J ENV RES PUB HE, V14, DOI 10.3390/ijerph14010094"
"   Ayotamuno Josiah M., 2006, Journal of Food Agriculture & Environment, V4, P218"
"   Bachate SP, 2013, CHEMOSPHERE, V90, P2273, DOI 10.1016/j.chemosphere.2012.10.081"
"   Banfalvi G, 2011, CELLULAR EFFECTS OF HEAVY METALS, P1, DOI 10.1007/978-94-007-0428-2"
"   Barria C, 2013, MICROBIOL-SGM, V159, P2437, DOI 10.1099/mic.0.052209-0"
"   Basha S. A., 2014, International Journal of Current Microbiology and Applied Sciences, V3, P785"
"   Beena AK., 2010, DEV MICROBIOLOGY MOL, V1, P113"
"   Bishnoi NR, 2007, J HAZARD MATER, V145, P142, DOI 10.1016/j.jhazmat.2006.10.093"
"   Boopathy R, 2000, BIORESOURCE TECHNOL, V74, P63, DOI 10.1016/S0960-8524(99)00144-3"
"   Borker AR, 2013, EMIR J FOOD AGR, V25, P443, DOI 10.9755/ejfa.v25i6.13970"
"   Brake SS, 2004, GEOMICROBIOL J, V21, P331, DOI 10.1080/01490450490454074"
"   Braud A, 2009, CHEMOSPHERE, V74, P280, DOI 10.1016/j.chemosphere.2008.09.013"
"   Bwapwa J. K., 2017, South African Journal of Chemical Engineering, V24, P62, DOI [10.1016/j.sajce.2017.06.005, 10.1016/j.sajce.2017.06.005]"
"   Carolin CF, 2017, J ENVIRON CHEM ENG, V5, P2782, DOI 10.1016/j.jece.2017.05.029"
"   Castro HF, 2010, APPL ENVIRON MICROB, V76, P999, DOI 10.1128/AEM.02874-09"
"   Chandra R, 2015, ENVIRON SCI-PROC IMP, V17, P326, DOI [10.1039/c4em00627e, 10.1039/C4EM00627E]"
"   Chatterjee S., 2013, Plant-Based Remediation Processes, V35, P1, DOI DOI 10.1007/978-3-642-35564-6_1"
"   Chen M, 2015, BIOTECHNOL ADV, V33, P745, DOI 10.1016/j.biotechadv.2015.05.003"
"   Clausen T, 2011, NAT REV MOL CELL BIO, V12, P152, DOI 10.1038/nrm3065"

"   Congeevaram S, 2007, J HAZARD MATER, V146, P270, DOI 10.1016/j.jhazmat.2006.12.017"
"   Das D, 2010, J HAZARD MATER, V184, P765, DOI 10.1016/j.jhazmat.2010.08.105"
"   De Windt W, 2006, ANTON LEEUW INT J G, V90, P377, DOI 10.1007/s10482-006-9088-4"
"   Deng DM, 2008, J INTEGR PLANT BIOL, V50, P691, DOI 10.1111/j.1744-7909.2008.00669.x"
"   Dhanarani S, 2016, J TAIWAN INST CHEM E, V69, P61, DOI 10.1016/j.jtice.2016.09.032"
"   Dixit R, 2015, SUSTAINABILITY-BASEL, V7, P2189, DOI 10.3390/su7022189"
"   D??nmez G, 2002, PROCESS BIOCHEM, V38, P751, DOI 10.1016/S0032-9592(02)00204-2"
"   Dursun AY, 2003, PROCESS BIOCHEM, V38, P1647, DOI 10.1016/S0032-9592(02)00075-4"
"   Elbaz-Poulichet F, 2000, WATER RES, V34, P3222, DOI 10.1016/S0043-1354(00)00073-7"
"   Emamverdian A., 2015, SCI WORLD J, DOI DOI 10.1155/2015/756120"
"   Fadel M., 2017, HBRC Journal, V13, P106, DOI [10.1016/j.hbrcj.2014.12.006, 10.1016/j.hbrcj.2014.12.006]"
"   Fan JJ, 2014, J HAZARD MATER, V279, P236, DOI 10.1016/j.jhazmat.2014.07.016"
"   Fashola MO, 2016, INT J ENV RES PUB HE, V13, DOI 10.3390/ijerph13111047"
"   Favara P, 2017, J ENVIRON MANAGE, V204, P730, DOI 10.1016/j.jenvman.2017.03.097"
"   Finocchio E, 2010, CHEM ENG J, V156, P264, DOI 10.1016/j.cej.2009.10.015"
"   Flores-Chaparro CE, 2017, J ENVIRON MANAGE, V193, P126, DOI 10.1016/j.jenvman.2017.02.005"
"   Francov?? A, 2017, ENVIRON POLLUT, V220, P286, DOI 10.1016/j.envpol.2016.09.062"
"   Frey SD, 2008, SOIL BIOL BIOCHEM, V40, P2904, DOI 10.1016/j.soilbio.2008.07.020"
"   Fu YongQian Fu YongQian, 2012, African Journal of Biotechnology, V11, P1403"
"   Fujii K, 2013, SOIL BIOL BIOCHEM, V57, P109, DOI 10.1016/j.soilbio.2012.07.007"
"   Gadd GM, 2010, MICROBIOL-SGM, V156, P609, DOI 10.1099/mic.0.037143-0"
"   Garc??a-Delgado C, 2015, J HAZARD MATER, V285, P259, DOI 10.1016/j.jhazmat.2014.12.002"
"   Ghalib AK, 2014, BRAZ ARCH BIOL TECHN, V57, P554, DOI [10.1590/S1516-8913201402001, 10.1590/S1982-88372014000100013]"
"   Gianfreda L., 1999, BIOREMEDIAT J, V3, P1, DOI [DOI 10.1080/10889869991219163, 10.1080/10889869991219163]"
"   Gokhale SV, 2008, BIORESOURCE TECHNOL, V99, P3600, DOI 10.1016/j.biortech.2007.07.039"
"   Gong RM, 2005, CHEMOSPHERE, V58, P125, DOI 10.1016/j.chemosphere.2004.08.055"
"   Govarthanan M, 2016, 3 BIOTECH, V6, DOI 10.1007/s13205-016-0560-1"
"   Gramss G, 1999, CHEMOSPHERE, V38, P1481, DOI 10.1016/S0045-6535(98)00369-5"
"   Gu YL, 2015, ENVIRON SCI POLLUT R, V22, P6271, DOI 10.1007/s11356-014-3856-x"
"   Gundacker C, 2010, MUTAT RES-REV MUTAT, V705, P130, DOI 10.1016/j.mrrev.2010.06.003"
"   Gupta VK, 2008, J HAZARD MATER, V153, P759, DOI 10.1016/j.jhazmat.2007.09.021"
"   Gupta VK, 2006, J COLLOID INTERF SCI, V296, P59, DOI 10.1016/j.jcis.2005.08.033"
"   Tran HT, 2016, J ENVIRON CHEM ENG, V4, P2529, DOI 10.1016/j.jece.2016.04.038"
"   Haq F, 2016, DESALIN WATER TREAT, V57, P19946, DOI 10.1080/19443994.2015.1109561"
"   Hautphenne C, 2016, ENVIRON TECHNOL INNO, V5, P250, DOI 10.1016/j.eti.2016.04.001"
"   Hayaishi O., 2012, Molecular mechanisms of oxygen activation"
"   He ZG, 2009, J HAZARD MATER, V163, P869, DOI 10.1016/j.jhazmat.2008.07.041"
"   Hemambika B., 2011, Journal of Ecology and the Natural Environment, V3, P168"
"   Hobman JL, 2015, J MED MICROBIOL, V64, P471, DOI 10.1099/jmm.0.023036-0"
"   Huang W, 2013, COLLOID SURFACE B, V105, P113, DOI 10.1016/j.colsurfb.2012.12.040"
"   Huang ZZ, 2015, J HAZARD MATER, V289, P174, DOI 10.1016/j.jhazmat.2015.02.043"
"   Husain Q, 2006, CRIT REV BIOTECHNOL, V26, P201, DOI 10.1080/07388550600969936"
"   Ibrahim WM, 2016, EGYPT J BAS APPL SCI, V3, P241, DOI [10.1016/j.ejbas.2016.07.005, 10.1016/J.EJBAS.2016.07.005, DOI 10.1016/J.EJBAS.2016.07.005, 10.1016/j.ejbas.2016.07.005]"
"   Incharoensakdi A, 2002, CURR MICROBIOL, V45, P261, DOI 10.1007/s00284-002-3747-0"
"   Jacob JM, 2017, J HAZARD MATER, V324, P54, DOI 10.1016/j.jhazmat.2015.12.056"
"   Jaishankar Monisha, 2014, Interdiscip Toxicol, V7, P60, DOI 10.2478/intox-2014-0009"
"   Jin Y, 2017, 3 BIOTECH, V7, DOI 10.1007/s13205-017-0721-x"
"   Joshi PK, 2011, INDIAN J MICROBIOL, V51, P482, DOI 10.1007/s12088-011-0110-9"
"   Juturu V, 2014, RENEW SUST ENERG REV, V33, P188, DOI 10.1016/j.rser.2014.01.077"
"   Kalyani S, 2004, CHEMOSPHERE, V57, P1225, DOI 10.1016/j.chemosphere.2004.08.057"
"   Kang CH, 2016, ECOL ENG, V97, P304, DOI 10.1016/j.ecoleng.2016.10.016"
"   Kang CH, 2015, ECOL ENG, V74, P402, DOI 10.1016/j.ecoleng.2014.10.009"
"   Karigar C. S., 2011, Enzyme Research, V2011, P805187, DOI 10.4061/2011/805187"
"   Kariuki Zacharia, 2017, South African Journal of Chemical Engineering, V23, P62, DOI [10.1016/j.sajce.2017.02.001, 10.1016/j.sajce.2017.02.001]"
"   Karthik C, 2017, ARCH AGRON SOIL SCI, V63, P1058, DOI 10.1080/03650340.2016.1261116"
"   Karthik C, 2017, J HAZARD MATER, V333, P42, DOI 10.1016/j.jhazmat.2017.03.037"
"   Karthik C, 2017, J TAIWAN INST CHEM E, V70, P282, DOI 10.1016/j.jtice.2016.11.006"
"   Karthik C, 2016, J ADV RES, V7, P839, DOI 10.1016/j.jare.2016.08.007"
"   Khan MS, 2009, ENVIRON CHEM LETT, V7, P1, DOI 10.1007/s10311-008-0155-0"
"   Kidd P, 2009, ENVIRON EXP BOT, V67, P243, DOI 10.1016/j.envexpbot.2009.06.013"
"   Kim IH, 2015, J MICROBIOL BIOTECHN, V25, P1542, DOI 10.4014/jmb.1504.04067"
"   Kotrba P, 2009, BIOTECHNOL ADV, V27, P799, DOI 10.1016/j.biotechadv.2009.06.003"
"   Kumar JIN, 2012, J ENVIRON BIOL, V33, P27"
"   Kumar P, 2017, J IND ENG CHEM, V54, P30, DOI 10.1016/j.jiec.2017.06.010"
"   Kuppusamy S, 2017, CHEMOSPHERE, V168, P944, DOI 10.1016/j.chemosphere.2016.10.115"
"   Lee YC, 2011, BIORESOURCE TECHNOL, V102, P5297, DOI 10.1016/j.biortech.2010.12.103"
"   Li KF, 2011, J HAZARD MATER, V189, P531, DOI 10.1016/j.jhazmat.2011.02.075"
"   Li Q, 2007, PROCESS BIOCHEM, V42, P379, DOI 10.1016/j.procbio.2006.09.020"
"   Long DY, 2013, J HAZARD MATER, V256, P24, DOI 10.1016/j.jhazmat.2013.04.020"
"   Lu LL, 2008, J EXP BOT, V59, P3203, DOI 10.1093/jxb/ern174"
"   Lu WB, 2006, J HAZARD MATER, V134, P80, DOI 10.1016/j.jhazmat.2005.10.036"
"   OM, 2016, Journal of Bioremediation & Biodegradation, V07, DOI [10.4172/2155-6199.1000334, 10.4172/2155-6199.1000334, DOI 10.4172/2155-6199.1000334, DOI 10.4172/2155-6199.10003342]"
"   Magyarosy A, 2002, APPL MICROBIOL BIOT, V59, P382, DOI 10.1007/s00253-002-1020-x"
"   Maheswari S, 2009, ENVIRON TECHNOL, V30, P921, DOI 10.1080/09593330902971279"
"   Manafi Z, 2012, INT J MINER PROCESS, V112, P43, DOI 10.1016/j.minpro.2012.07.003"
"   Margesin R, 1999, BIOTECHNOL TECH, V13, P859, DOI 10.1023/A:1008928308695"
"   Medina JDC, 2017, CURRENT DEVELOPMENTS IN BIOTECHNOLOGY AND BIOENGINEERING: PRODUCTION, ISOLATION AND PURIFICATION OF INDUSTRIAL PRODUCTS, P217, DOI 10.1016/B978-0-444-63662-1.00010-5"
"   MeenaKumari M, 2015, SPECTROCHIM ACTA A, V135, P632, DOI 10.1016/j.saa.2014.07.037"

"   Mehta SK, 2001, ECOL ENG, V18, P1, DOI 10.1016/S0925-8574(00)00174-9"
"   Milner MJ, 2008, ANN BOT-LONDON, V102, P3, DOI 10.1093/aob/mcn063"
"   Mishra RR, 2012, J HAZARD MATER, V227, P219, DOI 10.1016/j.jhazmat.2012.05.063"
"   Mohsenzadeh F, 2014, J ENVIRON HEALTH SCI, V12, DOI 10.1186/2052-336X-12-102"
"   Mondal Naba Kumar, 2017, Journal of Genetic Engineering and Biotechnology, V15, P151, DOI [10.1016/j.jgeb.2017.01.006, 10.1016/j.jgeb.2017.01.006]"
"   Monteiro CM, 2011, ENVIRON CHEM LETT, V9, P169, DOI 10.1007/s10311-009-0258-2"
"   Mukherjee T, 2017, J ENVIRON MANAGE, V201, P435, DOI 10.1016/j.jenvman.2017.06.030"
"   Muter O, 2002, PROCESS BIOCHEM, V38, P123, DOI 10.1016/S0032-9592(02)00065-1"
"   Mwinyihija M, 2010, ECOTOXICOLOGICAL DIAGNOSIS IN THE TANNING INDUSTRY, P1, DOI 10.1007/978-1-4419-6266-9_1"
"   Nagajyoti PC, 2010, ENVIRON CHEM LETT, V8, P199, DOI 10.1007/s10311-010-0297-8"
"   Naik UC, 2012, ENVIRON SCI POLLUT R, V19, P3005, DOI 10.1007/s11356-012-0811-6"
"   Nie M, 2013, ECOL LETT, V16, P234, DOI 10.1111/ele.12034"
"   Olaniran AO, 2013, INT J MOL SCI, V14, P10197, DOI 10.3390/ijms140510197"
"   Olowu R A., 2015, American Journal of Chemistry, V5, P40, DOI DOI 10.5923/J.CHEMISTRY.20150501.06"
"   Oves M, 2013, EUR J SOIL BIOL, V56, P72, DOI 10.1016/j.ejsobi.2013.02.002"
"   ??zdemir S, 2013, BIOREMEDIAT J, V17, P86, DOI 10.1080/10889868.2012.751961"
"   Panda J, 2012, ENVIRON SCI POLLUT R, V19, P1809, DOI 10.1007/s11356-011-0702-2"
"   Park JK, 2002, KOREAN J CHEM ENG, V19, P68, DOI 10.1007/BF02706876"
"   Park JW, 2006, ARCH ENVIRON CON TOX, V50, P191, DOI 10.1007/s00244-004-0119-8"
"   Pereira ARB, 2014, AFR J BIOTECHNOL, V13"
"   Popenda A, 2014, DESALIN WATER TREAT, V52, P3918, DOI 10.1080/19443994.2014.887449"
"   Pradhan S, 2000, WORLD J MICROB BIOT, V16, P579, DOI 10.1023/A:1008987908001"
"   Prasher SO, 2004, ENVIRON TECHNOL, V25, P1097, DOI 10.1080/09593332508618378"
"   Pratas J, 2014, ECOL ENG, V69, P170, DOI 10.1016/j.ecoleng.2014.03.046"
"   Puyen ZM, 2012, BIORESOURCE TECHNOL, V126, P233, DOI 10.1016/j.biortech.2012.09.036"
"   Rahman MS, 2016, INT J ENVIRON SCI TE, V13, P1973, DOI 10.1007/s13762-016-1022-3"
"   Raja C.P, 2015, J ENV ENG, V142"
"   Rajkumar M, 2012, BIOTECHNOL ADV, V30, P1562, DOI 10.1016/j.biotechadv.2012.04.011"
"   Rezania S, 2016, J HAZARD MATER, V318, P587, DOI 10.1016/j.jhazmat.2016.07.053"
"   Rezania S, 2015, J ENVIRON MANAGE, V163, P125, DOI 10.1016/j.jenvman.2015.08.018"
"   Rhodes CJ, 2014, CHEM SPEC BIOAVAILAB, V26, P196, DOI 10.3184/095422914X14047407349335"
"   Rizwan Md, 2014, Journal of Nanoparticles, DOI 10.1155/2014/431787"
"   Roberts TL, 2014, PROCEDIA ENGINEER, V83, P52, DOI 10.1016/j.proeng.2014.09.012"
"   Romera E, 2007, BIORESOURCE TECHNOL, V98, P3344, DOI 10.1016/j.biortech.2006.09.026"
"   Ruiz JM, 2001, STUD NAT PROD CHEM, V25, P651"
"   Sakakibara M., 2010, WATER ENERGY, V26"
"   Salehizadeh H, 2003, WATER RES, V37, P4231, DOI 10.1016/S0043-1354(03)00418-4"
"   Samuel J, 2012, IND ENG CHEM RES, V51, P3740, DOI 10.1021/ie201796s"
"   de Groot NS, 2006, FEBS LETT, V580, P6471, DOI 10.1016/j.febslet.2006.10.071"
"   Saratale G, 2007, J BASIC MICROB, V47, P400, DOI 10.1002/jobm.200710337"
"   Saratale GD, 2017, J TAIWAN INST CHEM E, V76, P51, DOI 10.1016/j.jtice.2017.03.027"
"   Saratale GD, 2012, BIOMASS BIOENERG, V47, P302, DOI 10.1016/j.biombioe.2012.09.030"
"   Saratale GD, 2011, INT BIODETER BIODEGR, V65, P494, DOI 10.1016/j.ibiod.2011.01.012"
"   Saratale RG, 2011, J TAIWAN INST CHEM E, V42, P138, DOI 10.1016/j.jtice.2010.06.006"
"   Saratale RG, 2016, J ENVIRON SCI HEAL A, V51, P164, DOI 10.1080/10934529.2015.1087747"
"   Saratale RG, 2017, CHEMOSPHERE, V177, P35, DOI 10.1016/j.chemosphere.2017.02.132"
"   Saravanan VS, 2007, CHEMOSPHERE, V66, P1794, DOI 10.1016/j.chemosphere.2006.07.067"
"   Schaider LA, 2014, SCI TOTAL ENVIRON, V490, P456, DOI 10.1016/j.scitotenv.2014.04.126"
"   Schut S, 2011, INT J FOOD MICROBIOL, V145, P126, DOI 10.1016/j.ijfoodmicro.2010.11.039"
"   Seth CS, 2012, BOT REV, V78, P32, DOI 10.1007/s12229-011-9092-x"
"   Shafiquzzaman Siddiquee Shafiquzzaman Siddiquee, 2015, Journal of Microbial and Biochemical Technology, V7, P384, DOI 10.4172/1948-5948.1000243"
"   Shamuyarira KK, 2014, INT J ENV RES PUB HE, V11, P2569, DOI 10.3390/ijerph110302569"
"   Sharma D., 2011, Biotechnology, V10, P23, DOI [DOI 10.3923/BIOTECH.2011.23.40, DOI 10.3923/BI0TECH.2011.23.40]"
"   Singh A, 2012, WATER RES, V46, P779, DOI 10.1016/j.watres.2011.11.050"
"   Singh R, 2010, J HAZARD MATER, V174, P623, DOI 10.1016/j.jhazmat.2009.09.097"
"   Sinha A, 2012, INT BIODETER BIODEGR, V71, P1, DOI 10.1016/j.ibiod.2011.12.014"
"   Sobol Z, 2012, ENVIRON MOL MUTAGEN, V53, P94, DOI 10.1002/em.20679"
"   Sooksawat N, 2016, BIOREMEDIAT J, V20, P240, DOI 10.1080/10889868.2016.1212810"
"   Sowerby A, 2005, SOIL BIOL BIOCHEM, V37, P1805, DOI 10.1016/j.soilbio.2005.02.023"
"   Srinath T, 2002, CHEMOSPHERE, V48, P427, DOI 10.1016/S0045-6535(02)00089-9"
"   Srivastava J, 2014, INT J ENVIRON SCI TE, V11, P1787, DOI 10.1007/s13762-013-0412-z"
"   Sultan S, 2012, AFR J MICROBIOL RES, V6, P3329, DOI 10.5897/AJMR12.226"
"   Taiwo AM, 2016, J HAZARD MATER, V304, P166, DOI 10.1016/j.jhazmat.2015.10.061"
"   Tangahu BV, 2011, INT J CHEM ENG, V2011, DOI 10.1155/2011/939161"
"   Taqieddin E, 2004, BIOMATERIALS, V25, P1937, DOI 10.1016/j.biomaterials.2003.08.034"
"   Tarangini K., 2009, J ENV RES DEV, V3"
"   Tchounwou P., 2012, MOL CLIN ENV TOXICOL, V101, P133, DOI DOI 10.1007/978-3-7643-8340-4"
"   Terry PA, 2002, CHEMOSPHERE, V47, P249, DOI 10.1016/S0045-6535(01)00303-4"
"   Thakur S., 2016, ENVIRON MONIT ASSESS, V188, P154"
"   Thatoi H, 2014, J ENVIRON MANAGE, V146, P383, DOI 10.1016/j.jenvman.2014.07.014"
"   Thomson B., 2017, BIOGEOSCI DISCUSS, V2017, P1"
"   Tratnyek PG, 2006, NANO TODAY, V1, P44, DOI 10.1016/S1748-0132(06)70048-2"
"   Tuzen M, 2010, CHEM ENG J, V158, P200, DOI 10.1016/j.cej.2009.12.041"
"   Ullah A, 2015, ENVIRON EXP BOT, V117, P28, DOI 10.1016/j.envexpbot.2015.05.001"
"   Vasileva-Tonkova E, 2003, Z NATURFORSCH C, V58, P87"
"   Vasudevan P, 2002, BIORESOURCE TECHNOL, V82, P285, DOI 10.1016/S0960-8524(01)00181-X"
"   V??zquez S, 2006, WATER AIR SOIL POLL, V177, P349, DOI 10.1007/s11270-006-9178-y"
"   Venil C., 2011, ISRN MICROBIOL, V2011"
"   Verma A, 2013, ECOL ENG, V61, P486, DOI 10.1016/j.ecoleng.2013.10.008"
"   Verma RC, 2012, INT J PHARM SCI RES, V3, P1832, DOI 10.13040/IJPSR.0975-8232.3(6).1832-38"

"   Vijayaraghavan K, 2005, SEP PURIF TECHNOL, V44, P53, DOI 10.1016/j.seppur.2004.12.003"
"   Vijayaraghavan K, 2015, J ENVIRON MANAGE, V160, P283, DOI 10.1016/j.jenvman.2015.06.030"
"   Vogel M, 2010, SCI TOTAL ENVIRON, V409, P384, DOI 10.1016/j.scitotenv.2010.10.011"
"   Volland S, 2013, CHEMOSPHERE, V91, P448, DOI 10.1016/j.chemosphere.2012.11.064"
"   Wang NJ, 2014, APPL SURF SCI, V305, P267, DOI 10.1016/j.apsusc.2014.03.054"
"   Wang Y, 2017, FOOD SCI NUTR, V5, P215, DOI 10.1002/fsn3.384"
"   Watts MP, 2015, APPL ENVIRON MICROB, V81, P5511, DOI 10.1128/AEM.00853-15"
"   Weerasundara L, 2017, SCI TOTAL ENVIRON, V584, P803, DOI 10.1016/j.scitotenv.2017.01.121"
"   Wuana R.A., 2011, ISRN Ecology, DOI [10.5402/2011/402647, DOI 10.5402/2011/402647]"
"   Wyszkowska J, 2013, J ELEMENTOL, V18, P769, DOI 10.5601/jelem.2013.18.4.455"
"   Xu L, 2011, J HAZARD MATER, V185, P1169, DOI 10.1016/j.jhazmat.2010.10.028"
"   Xu XF, 2015, SCI REP-UK, V5, DOI 10.1038/srep17445"
"   Yal??in S, 2012, ENVIRON SCI POLLUT R, V19, P3118, DOI 10.1007/s11356-012-0807-2"
"   Yal??inkaya Y, 2002, TURK J CHEM, V26, P441"
"   Yan GY, 2001, BIORESOURCE TECHNOL, V78, P243, DOI 10.1016/S0960-8524(01)00020-7"
"   Yuan L, 2015, ENVIRON SCI TECHNOL, V49, P824, DOI 10.1021/es504207c"
"   Zeraatkar AK, 2016, J ENVIRON MANAGE, V181, P817, DOI 10.1016/j.jenvman.2016.06.059"
"   Zhou ZD, 2010, INT J BIOL MACROMOL, V47, P21, DOI 10.1016/j.ijbiomac.2010.04.001"
"   Zouboulis AI, 2004, PROCESS BIOCHEM, V39, P909, DOI 10.1016/S0032-9592(03)00200-0"
NR 204
TC 476
Z9 555
U1 38
U2 1047
PU ACADEMIC PRESS LTD- ELSEVIER SCIENCE LTD
PI LONDON
"PA 24-28 OVAL RD, LONDON NW1 7DX, the United Kingdom"
SN 0301-4797
EI 1095-8630
J9 J ENVIRON MANAGE
JI J. Environ. Manage.
PD JUL 1
PY 2018
VL 217
BP 56
EP 70
DI 10.1016/j.jenvman.2018.03.077
PG 15
WC Environmental Sciences
WE Science Citation Index Expanded (SCI-EXPANDED)
SC Environmental Sciences & Ecology
GA GG2GO
UT WOS:000432508400006
PM 29597108
DA 2026-04-06
ER

PT J
"AU Mai, XR"
"   Tang, J"
"   Tang, JX"
"   Zhu, XY"
"   Yang, ZH"
"   Liu, X"
"   Zhuang, XJ"
"   Feng, G"
"   Tang, L"
"AF Mai, Xurui"
"   Tang, Jing"
"   Tang, Juexuan"
"   Zhu, Xinyue"
"   Yang, Zhenhao"
"   Liu, Xi"
"   Zhuang, Xiaojie"
"   Feng, Guang"
"   Tang, Lin"
TI Research progress on the environmental risk assessment and remediation
   technologies of heavy metal pollution in agricultural soil
SO JOURNAL OF ENVIRONMENTAL SCIENCES
LA English
DT Review
DE Source; Migration transformation; Risk assessment; Remediation; Heavy
   metals; Agricultural soils
ID ELECTROKINETIC REMEDIATION; ASPERGILLUS-NIGER; REMOVAL; BIOREMEDIATION;
   ADSORPTION; CHROMIUM; IMPACT; WATER
"AB Controlling heavy metal pollution in agricultural soil has been a significant challenge. These heavy metals seriously threaten the surrounding ecological environment and human health. The effective assessment and remediation of heavy metals in agricultural soils are crucial. These two aspects support each other, forming a close and complete decisionmaking chain. Therefore, this review systematically summarizes the distribution characteristics of soil heavy metal pollution, the correlation between soil and crop heavy metal contents, the presence pattern and migration and transformation mode of heavy metals in the soil-crop system. The advantages and disadvantages of the risk evaluation tools and models of heavy metal pollution in farmland are further outlined, which provides important guidance for an in-depth understanding of the characteristics of heavy metal pollution in farmland soils and the assessment of the environmental risk. Soil remediation

strategies involve multiple physical, chemical, biological and even combined technologies, and this paper compares the potential and effect of the above current remediation technologies in heavy metal polluted farmland soils. Finally, the main problems and possible research directions of future heavy metal risk assessment and remediation technologies in agricultural soils are prospected. This review provides new ideas for effective assessment and selection of remediation technologies based on the characterization of soil heavy metals. (c) 2024 The Research Center for Eco-Environmental Sciences, Chinese Academy of Sciences. Published by Elsevier B.V."
"C1 [Mai, Xurui; Tang, Jing; Tang, Juexuan; Zhu, Xinyue; Yang, Zhenhao; Tang, Lin] Hunan Univ, Coll Environm Sci & Engn, Changsha 410082, Peoples R China."
"   [Mai, Xurui; Tang, Jing; Tang, Juexuan; Zhu, Xinyue; Yang, Zhenhao; Tang, Lin] Hunan Univ, Key Lab Environm Biol & Pollut Control, Minist Educ, Changsha 410082, Peoples R China."
"   [Liu, Xi; Zhuang, Xiaojie; Feng, Guang] Power China Zhongnan Engn Corp Ltd, Changsha 410014, Peoples R China."
C3 Hunan University; Hunan University
"RP Tang, J; Tang, L (corresponding author), Hunan Univ, Coll Environm Sci & Engn, Changsha 410082, Peoples R China.; Tang, J; Tang, L (corresponding author), Hunan Univ, Key Lab Environm Biol & Pollut Control, Minist Educ, Changsha 410082, Peoples R China."
EM jingtang@hnu.edu.cn; tanglin@hnu.edu.cn
"RI ; Tang, Lin/M-8040-2014"
"OI Mai, Xurui/0009-0001-9920-0162; Tang, Jing/0000-0002-8745-9177; "
"FU National Natural Science Foundation of China [52100184, U22A20617]"
FX This work was supported by the National Natural Science Foundation of
"   China (Nos. 52100184 , and U22A20617) ."
"CR Bart S, 2016, CATENA, V136, P44, DOI 10.1016/j.catena.2015.07.008"
"   Bian FangYuan Bian FangYuan, 2018, Scientia Silvae Sinicae, V54, P106"
"   Bolan N, 2014, J HAZARD MATER, V266, P141, DOI 10.1016/j.jhazmat.2013.12.018"
"   Cai Yanzi, 2018, Journal of Beijing Normal University (Natural Science), V54, P48, DOI 10.16360/j.cnki.jbnuns.2018.01.007"
"   [????????? Chen Weiping], 2018, [????????????, Acta Pedologica Sinica], V55, P261"
"   [????????? Chen Yujia], 2022, [????????????, Environmental Science], V43, P5728"
"   Cui JunYi Cui JunYi, 2018, Environmental Science & Technology (China), V41, P77"
"   [????????? Cui Xiaofeng], 2012, [????€?????????????, Ecology and Environmental Sciences], V21, P130"
"   Deng B, 2022, J PLANT GROWTH REGUL, V41, P639, DOI 10.1007/s00344-021-10327-0"
"   Ding L, 2017, ENVIRON SCI POLLUT R, V24, P3430, DOI 10.1007/s11356-016-8069-z"
"   [????????? Duan Mingyu], 2017, [??????????????????, Journal of Triticeae Crops], V37, P985"
"   Fan S., 2010, Chinese Agricultural Science Bulletin, V26, P310"
"   [?????? Fan Ting], 2013, [????€?????????????, Ecology and Environmental Sciences], V22, P1727, DOI 10.12677/aep.2017.71004"
"   Farhadian M, 2008, BIORESOURCE TECHNOL, V99, P5296, DOI 10.1016/j.biortech.2007.10.025"
"   Fei XF, 2023, J HAZARD MATER, V452, DOI 10.1016/j.jhazmat.2023.131231"
"   Feng RW, 2021, J HAZARD MATER, V402, DOI 10.1016/j.jhazmat.2020.123570"
"   Fu RB, 2017, CHEM ENG J, V316, P601, DOI 10.1016/j.cej.2017.01.092"
"   Fu TG, 2018, GEODERMA, V310, P230, DOI 10.1016/j.geoderma.2017.09.027"
"   Gao Jun, 2023, Chemosphere, V335, P139088, DOI [10.1016/j.chemosphere.2023.139088, 10.1016/j.chemosphere.2023.139088]"
"   Gao P., 2014, Master's thesis"
"   Guo GH, 2023, SCI TOTAL ENVIRON, V887, DOI 10.1016/j.scitotenv.2023.163897"
"   Guo L, 2017, INT J ENV RES PUB HE, V14, DOI 10.3390/ijerph14121475"
"   Gusiatin ZM, 2020, INT J ENV RES PUB HE, V17, DOI 10.3390/ijerph17176220"
"   Hamdi FM, 2025, J ENVIRON SCI, V147, P424, DOI 10.1016/j.jes.2023.11.005"
"   Han R, 2021, CHEMOSPHERE, V278, DOI 10.1016/j.chemosphere.2021.130446"
"   He YiLin He YiLin, 2014, Research of Environmental Sciences, V27, P1074"
"   Hu H., 2023, Chemosphere, V327"
"   Hu Peng-Jie, 2018, Research of Agricultural Modernization, V39, P535"
"   Huang QQ, 2018, ENVIRON SCI POLLUT R, V25, P31175, DOI 10.1007/s11356-018-3068-x"
"   Huang S, 2019, INT J ENV RES PUB HE, V16, DOI 10.3390/ijerph16010097"
"   Huang Y, 2018, J ANIM ECOL, V87, P863, DOI 10.1111/1365-2656.12809"
"   Islam MN, 2024, J HAZARD MATER, V464, DOI 10.1016/j.jhazmat.2023.132931"
"   Jalali M, 2007, GEODERMA, V140, P156, DOI 10.1016/j.geoderma.2007.03.016"
"   Ji Dong-li, 2019, Research of Environmental Sciences, V31, P1895"
"   Jia Lin Jia Lin, 2009, Journal of Agro-Environment Science, V28, P2270"
"   Jiang F, 2023, APPL GEOCHEM, V152, DOI 10.1016/j.apgeochem.2023.105632"
"   Kimball S, 2015, RESTOR ECOL, V23, P800, DOI 10.1111/rec.12261"
"   Li J.Y., 2021, Master's thesis"
"   Li Ji, 2015, Huanjing Huaxue-Environmental Chemistry, V34, P1043, DOI 10.7524/j.issn.0254-6108.2015.06.2014111301"
"   Li YC, 2024, J ENVIRON CHEM ENG, V12, DOI 10.1016/j.jece.2023.111628"
"   Li YF, 2015, PLANT SOIL, V391, P195, DOI 10.1007/s11104-015-2418-4"
"   Li YL, 2022, CHEMOSPHERE, V286, DOI 10.1016/j.chemosphere.2021.131470"
"   Liu F, 2024, SCI TOTAL ENVIRON, V912, DOI 10.1016/j.scitotenv.2023.168990"
"   Liu Ping, 2007, Shengtaixue Zazhi, V26, P933"
"   Liu X.Y., 2021, Master's thesis"
"   Liu YM, 2020, ENVIRON POLLUT, V257, DOI 10.1016/j.envpol.2019.113581"
"   L?? QX, 2021, CHEMOSPHERE, V270, DOI 10.1016/j.chemosphere.2020.128626"
"   Lyu X.B., 2020, Master's thesis"
"   Ma Q., 2019, PhD Thesis"
"   Ma Qiang, 2023, Huan Jing Ke Xue, V44, P1668, DOI 10.13227/j.hjkx.202204269"
"   Mao CP, 2019, CATENA, V175, P339, DOI 10.1016/j.catena.2018.12.029"
"   Maxted AP, 2007, PLANT SOIL, V290, P157, DOI 10.1007/s11104-006-9149-5"
"   Minasny B, 2010, GEODERMA, V155, P132, DOI 10.1016/j.geoderma.2009.04.024"
"   Ni RX, 2018, PLOS ONE, V13, DOI 10.1371/journal.pone.0199460"
"   Pandey B, 2022, CHEMOSPHERE, V307, DOI 10.1016/j.chemosphere.2022.135889"
"   Peng, 2019, YUNNAN CHEM TECHNOL, V46, P88"
"   [????????? Qian Qingteng], 2016, [????????????????????, Environmental Pollution & Control], V38, P43"

"   Qu RongHui Qu RongHui, 2016, Zhongguo Shengtai Nongye Xuebao / Chinese Journal of Eco-Agriculture, V24, P517"
"   Ren SY, 2022, SCI TOTAL ENVIRON, V806, DOI 10.1016/j.scitotenv.2021.150322"
"   Ryu SR, 2017, J TAIWAN INST CHEM E, V70, P252, DOI 10.1016/j.jtice.2016.10.058"
"   Saxena G, 2020, REV ENVIRON CONTAM T, V249, P71, DOI 10.1007/398_2019_24"
"   Shahid M, 2014, REV ENVIRON CONTAM T, V232, P1, DOI 10.1007/978-3-319-06746-9_1"
"   Shi JD., 2022, ENVIRON ECOL, V4, P1"
"   Shi JD, 2023, SCI TOTAL ENVIRON, V871, DOI 10.1016/j.scitotenv.2023.161768"
"   Shi Y., 2018, NONFERROUS METALS SC, V9, P66, DOI DOI 10.13264/J.CNKI.YSJSKX.2018.05.012"
"   Silva KNO, 2018, J ELECTROANAL CHEM, V816, P171, DOI 10.1016/j.jelechem.2018.03.051"
"   Sodango TH, 2018, J HEALTH POLLUT, V8, P53, DOI 10.5696/2156-9614-8.17.53"
"   Srivastava S, 2006, SOIL BIOL BIOCHEM, V38, P1904, DOI 10.1016/j.soilbio.2005.12.016"
"   Sun XY, 2024, J ENVIRON SCI, V139, P516, DOI 10.1016/j.jes.2023.06.015"
"   Tang L, 2017, WATER RES, V117, P175, DOI 10.1016/j.watres.2017.03.059"
"   Tao Q, 2020, CHEMOSPHERE, V243, DOI 10.1016/j.chemosphere.2019.125290"
"   [?????? TENG Ying], 2007, [??????, Soils], V39, P497"
"   Tomczyk P, 2023, ECOL INDIC, V156, DOI 10.1016/j.ecolind.2023.111161"
"   Tsui L, 2022, ENVIRON RES, V215, DOI 10.1016/j.envres.2022.114239"
"   Wan XM, 2020, ENVIRON POLLUT, V266, DOI 10.1016/j.envpol.2020.115193"
"   [????????? Wan Yushan], 2018, [??????????????????, Chinese Journal of Environmental Engineering], V12, P2075"
"   Wang CC, 2023, SCI TOTAL ENVIRON, V882, DOI 10.1016/j.scitotenv.2023.163361"
"   Wang DH, 2021, ECOTOX ENVIRON SAFE, V208, DOI 10.1016/j.ecoenv.2020.111734"
"   Wang FE, 2023, ENVIRON POLLUT, V316, DOI 10.1016/j.envpol.2022.120608"
"   Wang J, 2021, ECOTOX ENVIRON SAFE, V207, DOI 10.1016/j.ecoenv.2020.111261"
"   Wang N, 2022, SCI REP-UK, V12, DOI 10.1038/s41598-022-20865-6"
"   Wang Y., 2023, J. Saf. Environ., P1"
"   Wang YuJun Wang YuJun, 2017, Journal of Agro-Environment Science, V36, P2365"
"   Wang ZH, 2025, J ENVIRON SCI, V147, P179, DOI 10.1016/j.jes.2023.10.002"
"   Waseem M, 2024, PLANT STRESS, V11, DOI 10.1016/j.stress.2023.100297"
"   Wei BG, 2010, MICROCHEM J, V94, P99, DOI 10.1016/j.microc.2009.09.014"
"   Wei S.H., 2004, Solanum nigrum L. Chin. Sci. Bull., P2568"
"   Wu Lie-shan, 2015, Huanjing Kexue, V36, P309"
"   Wu YF, 2022, RESOUR CONSERV RECY, V181, DOI 10.1016/j.resconrec.2022.106261"
"   Xia F, 2024, J HAZARD MATER, V465, DOI 10.1016/j.jhazmat.2023.133215"
"   Xiang MT, 2021, ENVIRON POLLUT, V278, DOI 10.1016/j.envpol.2021.116911"
"   [?????? Xiong Zi], 2016, [????????????????????????, Journal of Agro-Environment Science], V35, P2275"
"   [?????? XU Yan], 2008, [?????????????????, Journal of Anhui Agricultural Sciences], V36, P4615"
"   [?????? Yan Xiao], 2021, [??€?????????, Chinese Journal of Rare Metals], V45, P240"
"   Yang GD, 2014, RSC ADV, V4, P58362, DOI 10.1039/c4ra08432b"
"   Yang GD, 2015, CHEM ENG J, V259, P854, DOI 10.1016/j.cej.2014.08.081"
"   Yang HL., 2023, N HORTIC, V05, P123"
"   Yi YY, 2023, ACS EST WATER, V3, P793, DOI 10.1021/acsestwater.2c00580"
"   [????????? Yin Jingxuan], 2020, [??????????????????, Acta Scientiae Circumstantiae], V40, P2212"
"   Yin K, 2019, CHEM ENG J, V360, P1553, DOI [10.1016/j.cej.2018.10.226, 10.1016/j.jhazmat.2024.134410]"
"   Yu G, 2020, ENVIRON POLLUT, V266, DOI 10.1016/j.envpol.2020.115408"
"   Yu Hong-Yan, 2023, Southwest China Journal of Agricultural Sciences, V36, P1066, DOI 10.16213/j.cnki.scjas.2023.05.020"
"   Zaltauskaite J, 2022, J HAZARD MATER, V424, DOI 10.1016/j.jhazmat.2021.127316"
"   Zeng X, 2023, PLOS ONE, V18, DOI 10.1371/journal.pone.0285116"
"   Zhai WW, 2020, ENVIRON POLLUT, V258, DOI 10.1016/j.envpol.2019.113790"
"   Zhang BL, 2023, ENVIRON POLLUT, V330, DOI 10.1016/j.envpol.2023.121607"
"   Zhang D.X., 2017, Guangdong Chem. Ind., V44, P205"
"   Zhang SC, 2022, INT J ENV RES PUB HE, V19, DOI 10.3390/ijerph192113880"
"   [????????? Zhang Yongzhi], 2009, [????€?????????????, Ecology and Environmental Sciences], V18, P515"
"   [????????? Zhao Ajuan], 2020, [??????????????????, Acta Tabacaria Sinica], V26, P90"
"   Zhao B, 2023, J HAZARD MATER, V448, DOI 10.1016/j.jhazmat.2023.130879"
"   Zhao KeLi Zhao KeLi, 2016, Zhongguo Shengtai Nongye Xuebao / Chinese Journal of Eco-Agriculture, V24, P226"
"   Zhao SN, 2016, PROCEDIA ENVIRON SCI, V31, P274, DOI 10.1016/j.proenv.2016.02.036"
"   Zhong L., 2023, Jiangsu Agric. Sci, V51, P34"
"   Zhong SF, 2021, ENVIRON SCI TECHNOL, V55, P12741, DOI 10.1021/acs.est.1c01339"
"   Zhou XB, 2020, FRONT PLANT SCI, V11, DOI 10.3389/fpls.2020.586421"
NR 139
TC 137
Z9 150
U1 531
U2 1872
PU SCIENCE PRESS
PI BEIJING
"PA 16 DONGHUANGCHENGGEN NORTH ST, BEIJING 100717, PEOPLES R CHINA"
SN 1001-0742
EI 1878-7320
J9 J ENVIRON SCI
JI J. Environ. Sci.
PD MAR
PY 2025
VL 149
BP 1
EP 20
DI 10.1016/j.jes.2024.01.045
EA FEB 2024
PG 20
WC Environmental Sciences

```
WE Science Citation Index Expanded (SCI-EXPANDED)
SC Environmental Sciences & Ecology
GA NF3W2
UT WOS:001199008700001
PM 39181626
DA 2026-04-06
ER

PT J
"AU Wang, X"
"   Yang, ZL"
"   Zeng, Q"
"   Wang, XL"
"   Liu, S"
"   Wang, EG"
"   Wu, YJ"
"   Zeng, YN"
"   He, ML"
"   Wang, Y"
"   Shen, GQ"
"   Jing, XM"
"   Ping, R"
"   Zhang, X"
"   Chen, BD"
"AF Wang, Xia"
"   Yang, Zhonglin"
"   Zeng, Qin"
"   Wang, Xueli"
"   Liu, Song"
"   Wang, Engui"
"   Wu, Yangjin"
"   Zeng, Yinan"
"   He, Maolin"
"   Wang, Yan"
"   Shen, Guoqiang"
"   Jing, Xuemin"
"   Ping, Ren"
"   Zhang, Xin"
"   Chen, Baodong"
TI Chitosan hydrogel microspheres loaded with Bacillus
   subtilis promote plant growth and reduce chromium uptake
SO INTERNATIONAL JOURNAL OF BIOLOGICAL MACROMOLECULES
LA English
DT Article
DE Bacterial-hydrogel microsphere; Heavy metal soil pollution; Microbial
   remediation
ID CONTAMINATED SOIL; PHYTOREMEDIATION; PERFORMANCE; MANAGEMENT; BACTERIA;
   TOXICITY; STRESS; METALS
"AB Cr contamination can lead to reduced crop yields and threaten food security. Eliminating soil Cr
contamination or improving crop resistance to Cr is challenging in terms of costly, environment and
biodiversity risk. Here, we used chitosan hydrogel microspheres loaded with Bacillus subtilis to cope with
plant stress caused by Cr contamination. The addition of chitosan hydrogel microspheres loaded with Bacillus
subtilis increased shoot biomass by 88 % and decreased the plant Cr concentration by 17 %. The bacterial
chitosan hydrogel microsphere treatment enhanced the decomposition of organic matter and facilitated the
uptake of nutrients by the plants. It also significantly increased the abundance of anti-heavy metal stress
functional bacteria (Proteobacteria, Actinobacteria, and Chloroflexi), strongly promoting interactions and
correlations between microbes. This technology of bacterial-chitosan hydrogel microspheres presents promising
opportunities for sustainable strategies for addressing heavy metal pollution and enhancing agricultural
productivity."
"C1 [Wang, Xia; Yang, Zhonglin; Zeng, Qin; Wang, Xueli; Liu, Song; Wu, Yangjin; Zeng, Yinan; He, Maolin;
Wang, Yan; Shen, Guoqiang; Ping, Ren] Sichuan Normal Univ, Coll Geog & Resources, Key Lab Land Resources
Evaluat & Monitoring Southw, Chengdu 610066, Peoples R China."
"   [Wang, Xia; Jing, Xuemin; Zhang, Xin; Chen, Baodong] Chinese Acad Sci, Res Ctr Ecoenvironm Sci, Beijing
100085, Peoples R China."
"   [Wang, Engui; Zhang, Xin; Chen, Baodong] Univ Chinese Acad Sci, Beijing 100049, Peoples R China."
C3 Sichuan Normal University; Chinese Academy of Sciences; Research Center
"   for Eco-Environmental Sciences (RCEES), CAS; Chinese Academy of"
"   Sciences; University of Chinese Academy of Sciences, CAS"
"RP Wang, X; Zhang, X (corresponding author), Chinese Acad Sci, Res Ctr Ecoenvironm Sci, Beijing 100085,
Peoples R China."
EM wangxia114@mails.ucas.ac.cn; xinzhang@rcees.ac.cn
"RI Chen, Baodong/AAI-2012-2020"
"OI Wang, Engui/0009-0005-6983-3929"
FU National Natural Science Foundation of China [42207050]; Sichuan
   Sci-ence and Technology Program [2024NSFSC0860]; Sichuan Province
   Science and Technology Program [2023NSFSC1979]; National Key Research
   and Development Program of China [2023YFF1306004]
FX We are grateful to Prof. Han Ouyang (UCAS) for their cooperation and
   advice in this study. This work was supported by National Natural
"   Science Foundation of China grants (No. 42207050) , the Sichuan Sci-ence"
"   and Technology Program (No. 2024NSFSC0860) , and the Sichuan Province"
   Science and Technology Program (No. 2023NSFSC1979) . This study was
   financially supported by the National Key Research and Development
   Program of China (2023YFF1306004) .
```

"CR Adki VS, 2013, ENVIRON SCI POLLUT R, V20, P1173, DOI 10.1007/s11356-012-1125-4"
"   Ahemad M, 2019, ARAB J CHEM, V12, P1365, DOI 10.1016/j.arabjc.2014.11.020"
"   Ali S, 2011, SOIL SCI PLANT NUTR, V57, P68, DOI 10.1080/00380768.2010.549444"
"   Bashan Y, 2014, PLANT SOIL, V378, P1, DOI 10.1007/s11104-013-1956-x"
"   Cheng GJ, 2009, EUR J SOIL BIOL, V45, P483, DOI 10.1016/j.ejsobi.2009.06.009"
"   Chhetri G, 2022, J MICROBIOL, V60, P1"
"   Choudhary B, 2017, ENVIRON SCI POLLUT R, V24, P16786, DOI 10.1007/s11356-017-9322-9"
"   Elshamly AMS, 2023, J SOIL SCI PLANT NUT, V23, P1913, DOI 10.1007/s42729-023-01146-1"
"   Etesami H, 2018, ECOTOX ENVIRON SAFE, V147, P175, DOI 10.1016/j.ecoenv.2017.08.032"
"   Han Lingzhuo, 2023, Chemosphere, V344, P140307, DOI [10.1016/j.chemosphere.2023.140307, 10.1016/j.chemosphere.2023.140307]"
"   Hazirah HM, 2017, BIOSCI RES, V14, P1074"
"   Hidangmayum A, 2019, PHYSIOL MOL BIOL PLA, V25, P313, DOI 10.1007/s12298-018-0633-1"
"   Hirsch AM, 2010, SOIL BIOL BIOCHEM, V42, P536, DOI 10.1016/j.soilbio.2009.11.023"
"   Hu J, 2016, MBIO, V7, DOI 10.1128/mBio.01790-16"
"   Huo JL, 2024, J ENVIRON MANAGE, V356, DOI 10.1016/j.jenvman.2024.120655"
"   Jia R.Y., 2015, Adv. Mater. Res., V1073, P672"
"   Kamari A, 2011, J ENVIRON MANAGE, V92, P2675, DOI 10.1016/j.jenvman.2011.06.005"
"   Kumar G.K., 2016, Plant Growth Promoting Characteristics of Rhizobial Strains Isolated From Root Nodules of Vigna trilobata Cultivars"
"   Kumari A, 2023, ENVIRON RES, V223, DOI 10.1016/j.envres.2023.115485"
"   Li XY, 2024, ENVIRON RES, V251, DOI 10.1016/j.envres.2024.118716"
"   Liu SY, 2023, J HAZARD MATER, V441, DOI 10.1016/j.jhazmat.2022.129975"
"   L??pez-Bucio JS, 2022, ENVIRON POLLUT, V312, DOI 10.1016/j.envpol.2022.120084"
"   Lu C., 2009, ACTA AGR JIANGXI, V21, P142, DOI DOI 10.19386/J.CNKI.JXNYXB.2009.08.049"
"   Mahapatra S, 2022, J APPL MICROBIOL, V132, P3543, DOI 10.1111/jam.15480"
"   Martignoni MM, 2020, ECOL MODEL, V438, DOI 10.1016/j.ecolmodel.2020.109321"
"   Modrzejewska Z, 2006, SEP SCI TECHNOL, V41, P111, DOI 10.1080/01496390500446160"
"   Nanayakkara KGN, 2011, MAR POLLUT BULL, V63, P119, DOI 10.1016/j.marpolbul.2011.03.003"
"   Nayak AK, 2018, INT J PHYTOREMEDIAT, V20, P682, DOI 10.1080/15226514.2017.1413332"
"   Nebesk?? D, 2021, ECOTOX ENVIRON SAFE, V224, DOI 10.1016/j.ecoenv.2021.112630"
"   O'Callaghan M, 2016, APPL MICROBIOL BIOT, V100, P5729, DOI 10.1007/s00253-016-7590-9"
"   Patra DK, 2018, CHEMOSPHERE, V193, P793, DOI 10.1016/j.chemosphere.2017.11.062"
"   Pei YX, 2018, FRONT MICROBIOL, V9, DOI 10.3389/fmicb.2018.00038"
"   Polti MA, 2009, J BASIC MICROB, V49, P285, DOI 10.1002/jobm.200800239"
"   Poopal AC, 2009, J HAZARD MATER, V169, P539, DOI 10.1016/j.jhazmat.2009.03.126"
"   Qin GW, 2021, CHEMOSPHERE, V267, DOI 10.1016/j.chemosphere.2020.129205"
"   Qiu TY, 2024, IMETA, V3, DOI 10.1002/imt2.187"
"   Rizvi A., 2017, Microbial Strategies for Vegetable Production, P99, DOI [DOI 10.1007/978-3-319-54401-4_5, 10.1007/978-3-319-54401-4_5]"
"   Rizvi A, 2023, PLANT GROWTH REGUL, V99, P449, DOI 10.1007/s10725-022-00928-4"
"   Rizvi A, 2022, ENVIRON EXP BOT, V200, DOI 10.1016/j.envexpbot.2022.104911"
"   Rizvi A, 2021, MICROORGANISMS, V9, DOI 10.3390/microorganisms9122451"
"   Rizvi A, 2021, FRONT PLANT SCI, V12, DOI 10.3389/fpls.2021.746780"
"   Rizvi A, 2020, RSC ADV, V10, P38379, DOI 10.1039/d0ra05610c"
"   Rizvi A, 2019, RSC ADV, V9, P6125, DOI 10.1039/c9ra00333a"
"   Rizvi A, 2019, ECOTOXICOLOGY, V28, P302, DOI 10.1007/s10646-019-02023-3"
"   Sethuraman P., 2010, Int J Eng Sci Technol, P1811, DOI DOI 10.3390/MA812"
"   Shahid M, 2017, CHEMOSPHERE, V178, P513, DOI 10.1016/j.chemosphere.2017.03.074"
"   Shahrajabian MH, 2021, BIOMOLECULES, V11, DOI 10.3390/biom11060819"
"   Sinha VR, 2004, INT J PHARMACEUT, V274, P1, DOI 10.1016/j.ijpharm.2003.12.026"
"   Tao JM, 2018, FRONT MICROBIOL, V9, DOI 10.3389/fmicb.2018.03087"
"   VANELSAS JD, 1990, BIOL FERT SOILS, V10, P127, DOI 10.1007/BF00336248"
"   Vodyanitskii YN, 2013, EURASIAN SOIL SCI+, V46, P793, DOI 10.1134/S1064229313050153"
"   Wakeel A, 2020, INT J MOL SCI, V21, DOI 10.3390/ijms21030728"
"   Wu ZH, 2024, ECOTOX ENVIRON SAFE, V282, DOI 10.1016/j.ecoenv.2024.116691"
"   Yang ZP, 2012, J INTEGR AGR, V11, P655, DOI 10.1016/S2095-3119(12)60053-7"
"   Zayed AM, 2003, PLANT SOIL, V249, P139, DOI 10.1023/A:1022504826342"
"   Zheng XJ, 2024, SUSTAINABILITY-BASEL, V16, DOI 10.3390/su16135715"
NR 56
TC 10
Z9 10
U1 21
U2 77
PU ELSEVIER
PI AMSTERDAM
"PA RADARWEG 29, 1043 NX AMSTERDAM, NETHERLANDS"
SN 0141-8130
EI 1879-0003
J9 INT J BIOL MACROMOL
JI Int. J. Biol. Macromol.
PD JAN
PY 2025
VL 286
AR 138401
DI 10.1016/j.ijbiomac.2024.138401
EA DEC 2024
PG 11
"WC Biochemistry & Molecular Biology; Chemistry, Applied; Polymer Science"
WE Science Citation Index Expanded (SCI-EXPANDED)
SC Biochemistry & Molecular Biology; Chemistry; Polymer Science
GA P0K6V
UT WOS:001374912500001
PM 39643173

DA 2026-04-06
ER

PT J
AU Imran
"   Khan, AA"
"   Kamal, A"
"   Alrefaei, AF"
"   Zaman, W"
AF Imran
"   Khan, Asad Ali"
"   Kamal, Abid"
"   Alrefaei, Abdulwahed Fahad"
"   Zaman, Wajid"
TI Utilization and Characterization of Microbes for Heavy Metal Remediation
SO POLISH JOURNAL OF ENVIRONMENTAL STUDIES
LA English
DT Article
DE contamination; heavy metal; soil and ecosystem health; bioremediation;
   microbes
ID SOIL; CONTAMINATION; CD
"AB Heavy metals in forest soils have substantial ecological implications, affecting the soil and the surrounding ecosystem. These naturally occurring elements, with high atomic weights, become toxic at elevated concentrations. Notable heavy metals in forest soils include lead, cadmium, mercury, and arsenic. The profound toxicity of heavy metal pollution poses a significant risk to modern agriculture, with the potential for accumulation in crops and soils, threatening food security. A crucial aspect of addressing this challenge involves promptly restoring disrupted agricultural land. This study contributes to agricultural soil restoration by employing a combination of microorganisms, which have proven effective in alleviating heavy metal pollution. When paired with Sunflower (Helianthus annus), the combination ensures soil restoration and enhances food security. The study investigated microorganisms from contaminated soil, revealing Gram-negative bacilli and cocci arrangements. The colony characteristics, including hues and diameters, were assessed, with notable findings in samples 1, 2, and 5. The microbial ability to remove heavy metals (Pb, As, Hg, Ni, and Cd) was quantified, highlighting the diverse capacities among isolates. Selected isolates (1, 3, 6, 7, and 10) exhibited 25% higher biomass accumulation than the control, extracting at least 40 mg/L of each metal. Bacterial identification using a Vitek 2 Compact analyzer revealed Pantoea sp., Achromobacter denitrificans, Klebsiella oxytoca, Rhizobium radiobacter, and Pseudomonas fluorescens. Biocompatibility testing led to the formation of consortia for soil remediation, with Coalition D (Achromobacter denitrificans, Klebsiella oxytoca, and Rhizobium radiobacter in a 1:1:2 ratio) confirming the effective removal of Ni and Pb. Various consortia showed differing performances in removing composite contaminants, with Coalition D being promising, indicating a potential for phytoremediation. Optimal cultivation conditions were identified, with Coalition D excelling at metal removal and biomass accumulation. The temperature, pH, and soil conditions were crucial for Coalition D efficiency, and combining them with phytoremediation techniques showed promise. Laboratory experiments with sunflower seedlings confirmed the efficacy of Coalition D in enhancing soil phytoremediation, improving plant survival, and removing mixed heavy metals."
"C1 [Imran] South China Agr Univ, Coll Engn, Agr Aviat Innovat Lab, Guangzhou, Peoples R China."
"   [Imran] Govt Khyber Pakhtunkhwa, Minist Agr, Extens Wing, Peshawar, Pakistan."
"   [Khan, Asad Ali] Univ Agr, Dept Agron, Peshawar, Pakistan."
"   [Kamal, Abid] Univ Agr, Dept Soil & Environm Sci, Peshawar, Pakistan."
"   [Alrefaei, Abdulwahed Fahad] King Saud Univ, Dept Zool, Coll Sci, POB 62521, Riyadh 11451, Saudi Arabia."
"   [Zaman, Wajid] Yeungnam Univ, Dept Life Sci, Gyongsan 38541, South Korea."
C3 South China Agricultural University; University of Agriculture
   Faisalabad; Agricultural University Peshawar; Agricultural University
   Peshawar; University of Agriculture Faisalabad; King Saud University;
   Yeungnam University
"RP Zaman, W (corresponding author), Yeungnam Univ, Dept Life Sci, Gyongsan 38541, South Korea."
EM wajidzaman@yu.ac.kr
"RI Alrefaei, Abdulwahed/AAX-7443-2021; Zaman, Wajid/J-6491-2019; Imran,"
   Imran/AEI-0727-2022
"OI Imran, Imran/0000-0002-9459-0130"
"FU King Saud University, Riyadh, Saudi Arabia [RSP2024R218]"
FX We extend our appreciation to the Researchers Supporting Project (no.
"   RSP2024R218) , King Saud University, Riyadh, Saudi Arabia."
"CR Ayangbenro AS, 2017, INT J ENV RES PUB HE, V14, DOI 10.3390/ijerph14010094"
"   Babu AG, 2015, J ENVIRON MANAGE, V151, P160, DOI 10.1016/j.jenvman.2014.12.045"
"   Bai B, 2024, ENVIRON TECHNOL INNO, V33, DOI 10.1016/j.eti.2023.103485"
"   Bai B, 2023, POWDER TECHNOL, V416, DOI 10.1016/j.powtec.2023.118242"
"   Bai B, 2020, INT J HEAT MASS TRAN, V153, DOI 10.1016/j.ijheatmasstransfer.2020.119573"
"   Bai LY, 2015, ENVIRON SCI POLLUT R, V22, P5359, DOI 10.1007/s11356-014-3763-1"
"   BEKKERING C.S., 2023, CourseSource, V10"
"   Debonne N, 2021, GLOBAL ENVIRON CHANG, V66, DOI 10.1016/j.gloenvcha.2020.102221"
"   Defarge N, 2018, TOXICOL REP, V5, P156, DOI 10.1016/j.toxrep.2017.12.025"
"   Drozdova MY, 2021, FOOD RAW MATER, V9, P406"
"   Du KQ, 2024, IEEE T GEOSCI REMOTE, V62, DOI 10.1109/TGRS.2024.3351141"
"   Durand A, 2016, PLANT SOIL, V399, P179, DOI 10.1007/s11104-015-2691-2"
"   Fajardo C, 2019, APPL SOIL ECOL, V135, P56, DOI 10.1016/j.apsoil.2018.10.022"
"   Gong YY, 2018, WATER RES, V147, P440, DOI 10.1016/j.watres.2018.10.024"
"   Guan DX, 2018, ENVIRON SCI POLLUT R, V25, P31666, DOI 10.1007/s11356-018-3143-3"
"   Guan QY, 2022, SCI TOTAL ENVIRON, V833, DOI 10.1016/j.scitotenv.2022.155096"
"   Haque S, 2022, SCI TOTAL ENVIRON, V833, DOI 10.1016/j.scitotenv.2022.155222"
"   He MY, 2023, SPECTROCHIM ACTA B, V209, DOI 10.1016/j.sab.2023.106781"
"   He MY, 2021, GEOCHIM COSMOCHIM AC, V299, P151, DOI 10.1016/j.gca.2021.02.021"
"   Hu QL, 2022, AGR WATER MANAGE, V261, DOI 10.1016/j.agwat.2021.107345"
"   Jia Q, 2024, MICROBIOL SPECTR, V12, DOI 10.1128/spectrum.03427-23"
"   Jiang C, 2023, ENVIRON MONIT ASSESS, V195, DOI 10.1007/s10661-023-11821-6"

"   Jiang HH, 2020, SCI TOTAL ENVIRON, V701, DOI 10.1016/j.scitotenv.2019.134466"
"   Kapusta-Duch J, 2011, ECOL FOOD NUTR, V50, P137, DOI 10.1080/03670244.2011.552372"
"   Khalid S, 2017, J GEOCHEM EXPLOR, V182, P247, DOI 10.1016/j.gexplo.2016.11.021"
"   Khan I, 2019, ENVIRON MONIT ASSESS, V191, DOI 10.1007/s10661-019-7769-5"
"   Li CF, 2019, SOIL SEDIMENT CONTAM, V28, P380, DOI 10.1080/15320383.2019.1592108"
"   Li XQ, 2022, SPECTROCHIM ACTA B, V188, DOI 10.1016/j.sab.2021.106348"
"   Li Y, 2019, J APPL MICROBIOL, V126, P919, DOI 10.1111/jam.14165"
"   Liu J, 2023, CATENA, V231, DOI 10.1016/j.catena.2023.107357"
"   Liu J, 2024, SCI TOTAL ENVIRON, V916, DOI 10.1016/j.scitotenv.2024.169996"
"   Ma J, 2022, ENVIRON SCI TECHNOL, V56, P3524, DOI 10.1021/acs.est.1c07574"
"   Mitra S, 2018, MICROBIOL RES, V210, P12, DOI 10.1016/j.micres.2018.03.003"
"   Mo F, 2020, ECOTOX ENVIRON SAFE, V195, DOI 10.1016/j.ecoenv.2020.110499"
"   Mogal CS, 2022, HELIYON, V8, DOI 10.1016/j.heliyon.2022.e09532"
"   Nizamutdinov TiImur I., 2022, Food Processing: Techniques and Technology, V52, P350, DOI [10.21603/2074-9414-2022-2-2369, 10.21603/2074-9414-2022-2-2369]"
"   Oliveira VHD, 2020, ECOTOX ENVIRON SAFE, V202, DOI 10.1016/j.ecoenv.2020.110917"
"   Qiu SJ, 2023, CATENA, V220, DOI 10.1016/j.catena.2022.106708"
"   Rahman Z, 2020, ENVIRON SCI POLLUT R, V27, P27563, DOI 10.1007/s11356-020-08903-0"
"   Rillig MC, 2019, SCIENCE, V366, P886, DOI 10.1126/science.aay2832"
"   Sarkar S, 2017, COMMUN SOIL SCI PLAN, V48, P1008, DOI 10.1080/00103624.2017.1323087"
"   Singh K, 2023, J ENVIRON MANAGE, V332, DOI 10.1016/j.jenvman.2023.117294"
"   Sun RG, 2020, CHEMOSPHERE, V254, DOI 10.1016/j.chemosphere.2020.126828"
"   Sun SC, 2021, ARCH MICROBIOL, V203, P2699, DOI 10.1007/s00203-021-02271-0"
"   TONG J., 2020, Effects of land use and cover change on soil organic carbon and total nitrogen in southwest China Karst region: A Meta-analysis"
"   Twarog A, 2020, ENVIRON GEOCHEM HLTH, V42, P1453, DOI 10.1007/s10653-019-00435-y"
"   Uchimiya M, 2020, J AGR FOOD CHEM, V68, P12856, DOI 10.1021/acs.jafc.0c00183"
"   Wang TY, 2021, ADV CIV ENG, V2021, DOI 10.1155/2021/6646272"
"   Wang XY, 2022, NAT ECOL EVOL, V6, P890, DOI 10.1038/s41559-022-01774-3"
"   Wang YN, 2023, BIORESOURCE TECHNOL, V377, DOI 10.1016/j.biortech.2023.128978"
"   Yaashikaa PR, 2022, ENVIRON POLLUT, V312, DOI 10.1016/j.envpol.2022.120031"
"   Yan A, 2020, FRONT PLANT SCI, V11, DOI 10.3389/fpls.2020.00359"
"   Yu ZF, 2024, J HAZARD MATER, V469, DOI 10.1016/j.jhazmat.2024.133861"
"   Zeng XX, 2020, SCI REP-UK, V10, DOI 10.1038/s41598-020-58259-1"
"   Zhang SR, 2021, EARTHS FUTURE, V9, DOI 10.1029/2020EF001938"
"   Zhang T, 2023, LAND DEGRAD DEV, V34, P3340, DOI 10.1002/ldr.4687"
"   Zhang XC, 2022, GEOCHIM COSMOCHIM AC, V321, P115, DOI 10.1016/j.gca.2022.01.023"
"   Zhao Y, 2024, CATENA, V238, DOI 10.1016/j.catena.2024.107896"
"   Zhao YY, 2023, CHEM ENG J, V475, DOI 10.1016/j.cej.2023.146286"
"   Zhou Y, 2022, ENVIRON POLLUT, V307, DOI 10.1016/j.envpol.2022.119487"
"   Zhu GF, 2022, HYDROL EARTH SYST SC, V26, P3771, DOI 10.5194/hess-26-3771-2022"
NR 61
TC 10
Z9 10
U1 48
U2 77
PU HARD
PI OLSZTYN 5
"PA POST-OFFICE BOX, 10-718 OLSZTYN 5, POLAND"
SN 1230-1485
EI 2083-5906
J9 POL J ENVIRON STUD
JI Pol. J. Environ. Stud.
PY 2025
VL 34
IS 2
BP 1179
EP 1190
DI 10.15244/pjoes/186937
PG 12
WC Environmental Sciences
WE Science Citation Index Expanded (SCI-EXPANDED)
SC Environmental Sciences & Ecology
GA R8W8A
UT WOS:001394188400009
OA gold
DA 2026-04-06
ER

PT J
"AU Younas, H"
"   Nazir, A"
   Firdaus e Bareen
"AF Younas, Hajira"
"   Nazir, Aisha"
"   Firdaus e Bareen, Firdaus e"
"TI From sources to solutions: integrated approaches for Cd, Hg, and Pb"
   remediation- a comprehensive review
SO PLANT AND SOIL
LA English
DT Review
DE Heavy metals; Metalliferous sites; Conventional strategies;
   Microbes-dependent remediation; Genetically engineered microbes
ID GENETICALLY-MODIFIED ORGANISMS; HEAVY-METAL ACCUMULATION; CONTAMINATED

PADDY SOILS; BACILLUS-CEREUS RC-1; RICE ORYZA-SATIVA; ELECTROKINETIC
REMEDIATION; PSEUDOMONAS-PUTIDA; AQUEOUS-SOLUTION; PLANT-GROWTH;
ANTIBIOTIC-RESISTANCE
"AB BackgroundUrbanization, industrialization, and various other anthropogenic and natural activities increase the release of heavy metals (HMs) into the environment, posing a severe threat to nearby flora, fauna, and humans. HMs are toxic, non-biodegradable, silent, and subtle killers that alter the soil attributes and threaten plants, animals, and humans, impacting biodiversity and human health.ObjectiveHMs such as Cd, Hg, and Pb disturb physiological processes in plants and animals, reducing growth, impaired reproduction, and negative impacts on human beings upon bioaccumulation in food chains. This results in the dire need for effective remediation strategies to clean up HMs in soil and water.MethodsVarious conventional and non-conventional approaches remove and detoxify HMs from the substrate. Metal immobilization, detoxification, and removal via microbial approaches, i.e., biosorption, bioaccumulation, bioaugmentation, bioleaching, and bio-volatilization, are effective in remediating Cd, Hg, and Pb.Results Additionally, microbes utilize direct and indirect mechanisms to help the remediation of Cd, Hg, and Pb from contaminated sites. Genetically engineered microbes (GEMs) are also being explored for the remediation of these metals from contaminated soil, offering both opportunities and challenges regarding associated risks and management practices. This review emphasizes the possible heavy metal sources responsible for increasing the concentration of HMs in the environment and their effect on plants and animals. We will discuss various conventional remediation strategies that can help remediate Cd, Hg, and Pb.ConclusionBy elucidating the role of microbial-dependent remediation approaches, including GEMs, this review also contributes to advancing sustainable approaches for mitigating Cd, Hg, and Pb-contaminated soil and safeguarding environment."
"C1 [Younas, Hajira] Cornell Univ, Sch Integrat Plant Sci, Ithaca, NY 14850 USA."
" [Nazir, Aisha] Univ Punjab, Inst Bot, Lahore 54000, Pakistan."
" [Firdaus e Bareen, Firdaus e] Univ Lahore, Inst Microbiol & Mol Genet, Lahore 54000, Pakistan."
C3 Cornell University; University of Punjab; University of Lahore
"RP Younas, H (corresponding author), Cornell Univ, Sch Integrat Plant Sci, Ithaca, NY 14850 USA."
EM hajira.younas10@gmail.com
"CR Ab Rahman NNN, 2016, DESALIN WATER TREAT, V57, P13106, DOI 10.1080/19443994.2015.1057767"
" Abbas HA, 2018, AFR HEALTH SCI, V18, P11, DOI 10.4314/ahs.v18i1.3"
" Abdi O., 2015, Journal of Materials and Environmental Sciences, V6, P1386"
" Afef NH, 2012, FRESEN ENVIRON BULL, V21, P665"
" Agrawal K, 2024, CRIT REV BIOTECHNOL, V44, P429, DOI 10.1080/07388551.2023.2170862"
" Agyeman PC, 2021, ENVIRON GEOCHEM HLTH, V43, P601, DOI 10.1007/s10653-020-00743-8"
" Ahanger MA, 2019, BMC PLANT BIOL, V19, DOI 10.1186/s12870-019-2085-3"
" Ahemad M, 2015, 3 BIOTECH, V5, P111, DOI 10.1007/s13205-014-0206-0"
" Ahmad A, 2023, CURR MICROBIOL, V80, DOI 10.1007/s00284-023-03316-x"
" Ahmad G., 2020, The Plant Family Fabaceae: Biology and Physiological Responses to Environmental Stresses"
" Ahmed W, 2021, SCI TOTAL ENVIRON, V761, DOI 10.1016/j.scitotenv.2020.144216"
" Aka RJN, 2017, BIOREMEDIAT J, V21, P1, DOI 10.1080/10889868.2017.1282933"
" Akhtar S, 2013, SOIL ENVRON, V32, P55"
" Akhter K, 2017, PAK J ZOOL, V49, P1841, DOI [10.11758/journal.pjz/2017.49.5.1841.1846, 10.17582/journal.pjz/2017.49.5.1841.1846]"
" Akinkunmi WA, 2016, Journal of Biochemistry Microbiology and Biotechnology, V4, P1, DOI [10.54987/jobimb.v4i2.303, DOI 10.54987/JOBIMB.V4I2.303]"
" Akram M, 2021, Journal CleanWAS, V5, P05, DOI [10.26480/jcleanwas.01.2021.05.09, 10.26480/jcleanwas.01.2021.05.09, DOI 10.26480/JCLEANWAS.01.2021.05.09]"
" Al-Hagara OE., 2020, SCI ASIA, V46, P1"
" Alaboudi KA, 2019, ANN AGR SCI-CAIRO, V64, P95, DOI 10.1016/j.aoas.2019.04.002"
" Alamri SA, 2018, J PLANT INTERACT, V13, P409, DOI 10.1080/17429145.2018.1491067"
" Albert Q, 2018, CHEMOSPHERE, V196, P386, DOI 10.1016/j.chemosphere.2017.12.156"
" Alengebawy A, 2021, TOXICS, V9, DOI 10.3390/toxics9030042"
" Alkhatib R, 2019, PLANT BIOLOGY, V21, P1132, DOI 10.1111/plb.13021"
" Alotaibi BS, 2021, MICROORGANISMS, V9, DOI 10.3390/microorganisms9081628"
" Alsamhary K, 2023, CHEMOSPHERE, V311, DOI 10.1016/j.chemosphere.2022.136931"
" Alvarez-Bernal D, 2006, APPL SOIL ECOL, V33, P269, DOI 10.1016/j.apsoil.2005.10.007"
" An XY, 2022, ENVIRON EARTH SCI, V81, DOI 10.1007/s12665-022-10629-w"
" Anderson, 1993, SOIL WASHINGSOIL FLU"
" [Anonymous], 2010, Final Review of Scientific Information on Lead"
" [Anonymous], 2000, Supplementary Guidance for Conducting Health Risk Assessment of Chemical Mixtures"
" [Anonymous], 2016, Mineral Commodity Summaries, DOI [10.3133/70140094, DOI 10.3133/70140094]"
" [Anonymous], 2010, PREVENTING DIS HLTH, DOI DOI 10.1016/J.ECOENV.2011.12.007"
" [Anonymous], 2012, Toxicological Profile for Manganese (Draft for Public Comment)"
" Araki R, 2011, PLANT CELL PHYSIOL, V52, P1931, DOI 10.1093/pcp/pcr126"
" Arregui G, 2021, FRONT PLANT SCI, V11, DOI 10.3389/fpls.2020.560768"
" Ashraf MY, 2016, COMMUN SOIL SCI PLAN, V47, P405, DOI 10.1080/00103624.2015.1118117"
" Ashraf U, 2015, ENVIRON SCI POLLUT R, V22, P18318, DOI 10.1007/s11356-015-5463-x"
" Aslam F, 2020, INT MICROBIOL, V23, P253, DOI 10.1007/s10123-019-00098-w"
" Awasthi MK, 2021, RENEW SUST ENERG REV, V144, DOI 10.1016/j.rser.2021.110837"
" Aydin Mehmet Hadi, 2022, Turkey Tarimsal Arastirmalar Dergisi, V9, P118, DOI [10.19159/tutad.1004550, 10.19159/tutad.1004550]"
" Ayele Abate, 2021, ScientificWorldJournal, V2021, P5588111, DOI [10.1155/2021/5588111, 10.1155/2021/5588111]"
" Azizi Iraj, 2021, Journal of the Saudi Society of Agricultural Sciences, V20, P417, DOI [10.1016/j.jssas.2021.05.003, 10.1016/j.jssas.2021.05.003]"
" Bai YC, 2020, MICROORGANISMS, V8, DOI 10.3390/microorganisms8050694"
" Baldantoni D, 2016, ECOTOX ENVIRON SAFE, V123, P89, DOI 10.1016/j.ecoenv.2015.05.019"
" Bali S, 2019, CHEMOSPHERE, V235, P734, DOI 10.1016/j.chemosphere.2019.06.188"
" Banta G, 2023, Journal of Applied Science Engineering Technology and Education, V5, P150, DOI [10.35877/454ri.asci1915, 10.35877/454RI.asci1915, DOI 10.35877/454RI.ASCI1915]"
" Barman MK, 2023, VIETNAM J CHEM, V61, P533, DOI 10.1002/vjch.202300027"
" Baselt R C., 1995, Disposition of Toxic Drugs and Chemicals in Man, V4th Edn, P105"
" Bashir A, 2019, ENVIRON CHEM LETT, V17, P729, DOI 10.1007/s10311-018-00828-y"
" Baskaran V., 2020, ENV NANOTECHNO MONIT, V14"
" Beauvais-Fl??ck R, 2017, J HAZARD MATER, V338, P140, DOI 10.1016/j.jhazmat.2017.05.028"

"   Belimov AA, 2004, MICROBIOLOGY+, V73, P99, DOI 10.1023/B:MICI.0000016377.62060.d3"
"   Beni NN, 2023, COMMUN EARTH ENVIRON, V4, DOI 10.1038/s43247-023-00691-y"
"   Bertagnolli C, 2016, IND ENG CHEM RES, V55, P2461, DOI 10.1021/acs.iecr.5b04683"
"   BETOWSKI LD, 1988, ENVIRON SCI TECHNOL, V22, P1430, DOI 10.1021/es00177a007"
"   Bhatia A, 2015, WATER ENVIRON RES, V87, P2027, DOI 10.2175/106143015X14362865226833"
"   Binish MB, 2015, IJMS, V5, P1, DOI DOI 10.5376/IJMS.2015.05.0050"
"   Bjuhr J., 2007, Trace metals in soils irrigated with waste water in a periurban area downstream Hanoi city"
"   Boffetta P, 2020, SCI REP-UK, V10, DOI 10.1038/s41598-020-77027-9"
"   Bondarenko O, 2008, SENSORS-BASEL, V8, P6899, DOI 10.3390/s8116899"
"   Botelho AB, 2019, MINING METALL EXPLOR, V36, P199, DOI 10.1007/s42461-018-0016-8"
"   Brim H, 2000, NAT BIOTECHNOL, V18, P85, DOI 10.1038/71986"
"   Burgess-Conforti J. R., 2016, Liming characteristics of a high-calcium, dry flue gas desulfurization by-product and its effects on runoff water quality"
"   Bushra S, 2022, PHYTON-INT J EXP BOT, V91, P1987, DOI 10.32604/phyton.2022.019957"
"   Cao Y, 2018, SCI REP-UK, V8, DOI 10.1038/s41598-018-22782-z"
"   Carne G, 2021, SCI TOTAL ENVIRON, V760, DOI 10.1016/j.scitotenv.2020.143374"
"   Castagno LN, 2021, ANN APPL BIOL, V178, P256, DOI 10.1111/aab.12673"
"   Chang JJ, 2019, SCI TOTAL ENVIRON, V671, P676, DOI 10.1016/j.scitotenv.2019.03.409"
"   Chen SY, 2019, METALS-BASEL, V9, DOI 10.3390/met9090994"
"   Chen SH, 2020, INT J ENVIRON RES, V14, P235, DOI 10.1007/s41742-020-00253-6"
"   Chen XY, 2020, HUM ECOL RISK ASSESS, V26, P571, DOI 10.1080/10807039.2018.1539639"
"   Chen XW, 2018, PLANT SOIL, V425, P309, DOI 10.1007/s11104-018-3583-z"
"   Chibuike G.U., 2014, APPL ENVIRON SOIL SC, DOI [10.1155/2014/752708, DOI 10.1155/2014/752708]"
"   ??ikili Y, 2020, T??rk Tar??m ve Do??a Bilimleri Dergisi, V7, P1098, DOI [10.30910/turkjans.682480, 10.30910/turkjans.682480, DOI 10.30910/TURKJANS.682480]"
"   Ciopec M., 2023, Microbial Electrochemical Technologies, P201, DOI [10.1002/9783527839001.ch7, DOI 10.1002/9783527839001.CH7]"
"   Clarkson TW, 2006, CRIT REV TOXICOL, V36, P609, DOI 10.1080/10408440600845619"
"   Conner JR, 1998, CRIT REV ENV SCI TEC, V28, P397, DOI 10.1080/10643389891254250"
"   CWC, 2019, WATER RELATED STAT"
"   Czuprynski P, 2022, RSC ADV, V12, P5145, DOI 10.1039/d1ra09426b"
"   Dalyan E, 2020, RAD HEA MET ENVIR, P129, DOI 10.1007/978-3-030-21638-2_8"
"   Aparicio JD, 2022, J ENVIRON CHEM ENG, V10, DOI 10.1016/j.jece.2022.107141"
"   Danquah CA, 2022, ANTIBIOTICS-BASEL, V11, DOI 10.3390/antibiotics11030285"
"   Dary M, 2010, J HAZARD MATER, V177, P323, DOI 10.1016/j.jhazmat.2009.12.035"
"   Das S, 2014, ELSEV INSIGHT, P23, DOI 10.1016/B978-0-12-800021-2.00002-9"
"   Vega-P??ez JD, 2019, MATERIALS, V12, DOI 10.3390/ma12081296"
"   Deng X, 2007, J HAZARD MATER, V139, P340, DOI 10.1016/j.jhazmat.2006.06.043"
"   Deng X, 2011, BIORESOURCE TECHNOL, V102, P3083, DOI 10.1016/j.biortech.2010.10.051"
"   Devos Y, 2014, TRANSGENIC RES, V23, P1, DOI 10.1007/s11248-013-9741-4"
"   Dhaliwal SS, 2020, SOIL SEDIMENT CONTAM, V29, P638, DOI 10.1080/15320383.2020.1758031"
"   Diao JR, 2017, FRESEN ENVIRON BULL, V26, P4817"
"   Diep P, 2018, FRONT BIOENG BIOTECH, V6, DOI 10.3389/fbioe.2018.00157"
"   Dimkic I, 2022, PHYSIOL MOL PLANT P, V117, DOI 10.1016/j.pmpp.2021.101754"
"   Dissanayake DMDC, 2023, FEMS MICROBIOL LETT, V370, DOI 10.1093/femsle/fnad092"
"   Dixit V. K., 2021, SOIL BIOREMEDIATION, P193, DOI DOI 10.1002/9781119547976.CH9"
"   Duan PF, 2020, ARAB J CHEM, V13, P6949, DOI 10.1016/j.arabjc.2020.06.041"
"   Ducic Tanja, 2005, Braz. J. Plant Physiol., V17, P103, DOI 10.1590/S1677-04202005000100009"
"   Dushyantha N, 2020, ORE GEOL REV, V122, DOI 10.1016/j.oregeorev.2020.103521"
"   Eaton MC, 1985, AM SOC MECH ENG PAP, V85"
"   Eckley CS, 2020, SCI TOTAL ENVIRON, V707, DOI 10.1016/j.scitotenv.2019.136031"
"   Ekawanti Ardiana, 2015, J Health Pollut, V5, P25, DOI [10.5696/2156-9614-5-9.25, 10.5696/2156-9614-5-9.25]"
"   Ekwere AS., 2021, SE NIGERIA ENV NANOT, V15"
"   El-Bondkly AMA, 2022, HELIYON, V8, DOI 10.1016/j.heliyon.2022.e09854"
"   El-Gendy MMAA, 2017, MYCOBIOLOGY, V45, P73, DOI 10.5941/MYCO.2017.45.2.73"
"   El-Maraghy SS, 2020, HELIYON, V6, DOI 10.1016/j.heliyon.2020.e04384"
"   Emenike CU, 2017, CLEAN-SOIL AIR WATER, V45, DOI 10.1002/clen.201500802"
"   Essa MH, 2015, SOIL SEDIMENT CONTAM, V24, P30, DOI 10.1080/15320383.2014.911720"
"   Fan Y, 2018, CHEMOSPHERE, V197, P382, DOI 10.1016/j.chemosphere.2018.01.055"
"   Fang YL, 2022, FRONT BIOENG BIOTECH, V10, DOI 10.3389/fbioe.2022.996456"
"   Farooqi ZUR, 2022, CLIMATE CHANGE MICRO, P1"
"   Fasani E, 2018, PLANT CELL ENVIRON, V41, P1201, DOI 10.1111/pce.12963"
"   Fatima G, 2019, INDIAN J CLIN BIOCHE, V34, P371, DOI 10.1007/s12291-019-00839-8"
"   Feng WL, 2019, ECOTOX ENVIRON SAFE, V180, P160, DOI 10.1016/j.ecoenv.2019.04.090"
"   Feng Y, 2022, AGRONOMY-BASEL, V12, DOI 10.3390/agronomy12071639"
"   Ferrari M, 2021, AGRONOMY-BASEL, V11, DOI 10.3390/agronomy11112138"
"   Firdaus-e-Bareen, 2012, J HAZARD MATER, V237, P186, DOI 10.1016/j.jhazmat.2012.08.033"
"   Firmanda A, 2022, BIOFUEL BIOPROD BIOR, V16, P1909, DOI 10.1002/bbb.2433"
"   Foong LK, 2020, RSC ADV, V10, P15430, DOI 10.1039/d0ra00762e"
"   Fosso-Kankeu E, 2018, HEAVY METALS IN THE ENVIRONMENT: MICROORGANISMS AND BIOREMEDIATION, P43"
"   Fran??ois F, 2012, APPL ENVIRON MICROB, V78, P1097, DOI 10.1128/AEM.06522-11"
"   Frye MD, 2022, USING PORTABLE XRAY"
"   Gadd GM, 2013, PROGR SOIL SCI, P115, DOI 10.1007/978-94-007-4177-5_6"
"   Gao MY, 2021, J HAZARD MATER, V416, DOI 10.1016/j.jhazmat.2021.125894"
"   Gautam A, 2022, CURR MICROBIOL, V79, DOI 10.1007/s00284-021-02734-z"
"   Gelbicova T, 2021, FRONT MICROBIOL, V11, DOI 10.3389/fmicb.2020.599882"
"   George B, 2012, BIOREMEDIAT J, V16, P195, DOI 10.1080/10889868.2012.731442"
"   Gharehbaghli N, 2022, S AFR J BOT, V150, P161, DOI 10.1016/j.sajb.2022.07.014"
"   Goldberg S., 2013, Reference Module in Earth Systems and Environmental Sciences"
"   Gonz??lez D, 2021, INT J ENV RES PUB HE, V18, DOI 10.3390/ijerph18189867"
"   Gopalakrishnan S, 2015, 3 BIOTECH, V5, P355, DOI 10.1007/s13205-014-0241-x"
"   Grandjean P, 2010, ENVIRON HEALTH PERSP, V118, P1429, DOI 10.1289/ehp.1002289"

"   Grimison C, 2023, J HAZARD MATER, V445, DOI 10.1016/j.jhazmat.2022.130441"
"   Gupta DK, 2013, ECOTOXICOLOGY, V22, P1403, DOI 10.1007/s10646-013-1126-1"
"   Gutierrez-Ruiz M, 2022, J ENVIRON MANAGE, V314, DOI 10.1016/j.jenvman.2022.115032"
"   Hadi F, 2010, CHEMOSPHERE, V80, P457, DOI 10.1016/j.chemosphere.2010.04.020"
"   Halim MA, 2020, J AGR FOOD CHEM, V68, P13497, DOI 10.1021/acs.jafc.0c04579"
"   Hamanaka RB, 2018, FRONT ENDOCRINOL, V9, DOI 10.3389/fendo.2018.00680"
"   Hao LJ, 2021, ECOTOX ENVIRON SAFE, V212, DOI 10.1016/j.ecoenv.2021.111996"
"   Hassan A, 2020, J SOIL SEDIMENT, V20, P66, DOI 10.1007/s11368-019-02394-4"
"   Hazen RM, 2012, AM MINERAL, V97, P1013, DOI 10.2138/am.2012.3922"
"   He F, 2015, ENVIRON SCI POLLUT R, V22, P8124, DOI 10.1007/s11356-015-4316-y"
"   He HD, 2017, CHEMOSPHERE, V189, P247, DOI 10.1016/j.chemosphere.2017.09.069"
"   He S, 2024, FRONT PLANT SCI, V15, DOI 10.3389/fpls.2024.1310328"
"   Heidari P, 2020, INT J ENV RES PUB HE, V17, DOI 10.3390/ijerph17114059"
"   Hertz-Picciotto I, 2000, AM J IND MED, V38, P300, DOI 10.1002/1097-0274(200009)38:3<300::AID-AJIM9>3.0.CO;2-C"
"   HIGHAM DP, 1986, ENVIRON HEALTH PERSP, V65, P5, DOI 10.2307/3430155"
"   Hlihor RM, 2017, PROCESS SAF ENVIRON, V108, P44, DOI 10.1016/j.psep.2016.06.016"
"   Hong Young-Seoub, 2012, J Prev Med Public Health, V45, P353, DOI 10.3961/jpmph.2012.45.6.353"
"   Hosseinniaee S, 2023, ENVIRON MONIT ASSESS, V195, DOI 10.1007/s10661-023-11551-9"
"   Hrynkiewicz K, 2018, ENVIRON REV, V26, P316, DOI 10.1139/er-2018-0023"
"   Huang F, 2018, ECOTOX ENVIRON SAFE, V157, P216, DOI 10.1016/j.ecoenv.2018.03.077"
"   Huang F, 2013, COLLOID SURFACE B, V107, P11, DOI 10.1016/j.colsurfb.2013.01.062"
"   Hummel M, 2018, SCI REP-UK, V8, DOI 10.1038/s41598-018-33952-4"
"   Hussain I, 2018, SCI TOTAL ENVIRON, V628-629, P1582, DOI 10.1016/j.scitotenv.2018.02.037"
"   Igiri BE, 2018, J TOXICOL-US, V2018, DOI 10.1155/2018/2568038"
"   Iloms E, 2020, INT J ENV RES PUB HE, V17, DOI 10.3390/ijerph17031096"
"   Imran M, 2019, CHEMOSPHERE, V215, P846, DOI 10.1016/j.chemosphere.2018.10.114"
"   Imron MF, 2021, SUSTAIN ENVIRON RES, V31, DOI 10.1186/s42834-021-00088-6"
"   Jaiswal S, 2020, FRONT MICROBIOL, V11, DOI 10.3389/fmicb.2020.00808"
"   Jalloh MA, 2009, J HAZARD MATER, V162, P1081, DOI 10.1016/j.jhazmat.2008.05.146"
"   Jana S, 2022, J PHYS-CONDENS MAT, V34, DOI 10.1088/1361-648X/ac3075"
"   Janssen DB, 2020, ENVIRON SCI-PROC IMP, V22, P487, DOI [10.1039/c9em00601j, 10.1039/C9EM00601J]"
"   Javanbakht V, 2014, WATER SCI TECHNOL, V69, P1775, DOI 10.2166/wst.2013.718"
"   Jayakumar Manickam, 2021, Arabian Journal of Geosciences, DOI [10.1007/s12517-021-08543-9, 10.1007/s12517-021-08543-9]"
"   Jiang ZS, 2021, ORE GEOL REV, V139, DOI 10.1016/j.oregeorev.2021.104527"
"   Jing X, 2023, INT J ENVIRON SCI TE, V20, P3039, DOI 10.1007/s13762-022-04158-z"
"   Jones L, 2015, ANAL METHODS-UK, V7, P7976, DOI 10.1039/c5ay01059d"
"   Jong MC, 2020, ENVIRON SCI TECHNOL, V54, P14984, DOI 10.1021/acs.est.0c03714"
"   Joshi, 2022, NEW FUTURE DEV MICRO, P369"
"   Joshi S, 2023, FRONT MICROBIOL, V14, DOI 10.3389/fmicb.2023.1229828"
"   Jung MC, 2008, SENSORS-BASEL, V8, P2413, DOI 10.3390/s8042413"
"   Kafle A, 2022, Environmental Advances, V8, P100203, DOI [10.1016/j.envadv.2022.100203, 10.1016/j.envadv.2022.100203, DOI 10.1016/J.ENVADV.2022.100203, 10.1016/J.ENVADV.2022.100203]"
"   Kagami T, 2013, WATER RES, V47, P1361, DOI 10.1016/j.watres.2012.12.001"
"   Kalayu G, 2019, INT J AGRON, V2019, DOI 10.1155/2019/4917256"
"   Kang SH, 2007, APPL ENVIRON MICROB, V73, P6317, DOI 10.1128/AEM.01237-07"
"   Kanwal A, 2020, SCI REP-UK, V10, DOI 10.1038/s41598-020-68208-7"
"   Karlsson S, 2002, CRITICAL REV"
"   Kaur G., 2017, Int. J. Innovat. Res. Sci. Eng, V3, P179"
"   Kaur G, 2015, PEDOSPHERE, V25, P428, DOI 10.1016/S1002-0160(15)30010-2"
"   Kaushik CP, 2014, PROC MAT SCI, V7, P16, DOI 10.1016/j.mspro.2014.10.004"
"   Khan A, 2018, MICROBIOL RES, V212, P103, DOI 10.1016/j.micres.2017.10.012"
"   Khan I, 2019, ENVIRON MONIT ASSESS, V191, DOI 10.1007/s10661-019-7769-5"
"   Kharytonov M, 2023, AGRICULTURE-BASEL, V13, DOI 10.3390/agriculture13061129"
"   Khatib I, 2022, J XENOBIOTICS, V12, P236, DOI 10.3390/jox12030018"
"   Khatun J, 2022, TOXICOLOGY, V477, DOI 10.1016/j.tox.2022.153274"
"   Khullar S, 2019, BIOMETALS, V32, P101, DOI 10.1007/s10534-018-00164-2"
"   Kim SK, 2005, J BIOSCI BIOENG, V99, P109, DOI 10.1263/jbb.99.109"
"   Kinuthia GK, 2020, SCI REP-UK, V10, DOI 10.1038/s41598-020-65359-5"
"   Kirkelund GM, 2010, J APPL ELECTROCHEM, V40, P1049, DOI 10.1007/s10800-009-0059-0"
"   Kiyono M, 1999, BIOL PHARM BULL, V22, P910"
"   Klapstein SJ, 2018, B ENVIRON CONTAM TOX, V100, P14, DOI 10.1007/s00128-017-2236-x"
"   Kobayashi T, 2012, ANNU REV PLANT BIOL, V63, P131, DOI 10.1146/annurev-arplant-042811-105522"
"   Kolseth AK, 2015, AGR ECOSYST ENVIRON, V214, P96, DOI 10.1016/j.agee.2015.08.021"
"   Kopru S, 2024, OPT QUANT ELECTRON, V56, DOI 10.1007/s11082-023-06166-w"
"   Korohoda W, 2008, CELL MOL BIOL LETT, V13, P312, DOI 10.2478/s11658-008-0004-y"
"   Korotkova NA, 2022, METALS-BASEL, V12, DOI 10.3390/met12081308"
"   Kulshreshtha A., 2014, Journal of Environmental Science, Toxicology and Food Technology, V8, P44, DOI [10.9790/2402-08714450, DOI 10.9790/2402-08714450]"
"   Kumar SS, 2020, CHEMOSPHERE, V241, DOI 10.1016/j.chemosphere.2019.124824"
"   Kumar V, 2023, HELIYON, V9, DOI 10.1016/j.heliyon.2023.e15472"
"   Kumar V, 2019, CHEMOSPHERE, V237, DOI 10.1016/j.chemosphere.2019.124567"
"   Kuo SL, 2021, WATER-SUI, V13, DOI 10.3390/w13243594"
"   Kurniawan SB, 2022, HELIYON, V8, DOI 10.1016/j.heliyon.2022.e08995"
"   Laperche V, 2021, MINERALS-BASEL, V11, DOI 10.3390/min11010033"
"   Lee SW, 2001, APPL ENVIRON MICROB, V67, P1437, DOI 10.1128/AEM.67.4.1437-1444.2001"
"   Li B, 2019, BIORESOURCE TECHNOL, V284, P231, DOI 10.1016/j.biortech.2019.03.128"
"   Li CS, 2013, BIORESOURCE TECHNOL, V143, P46, DOI 10.1016/j.biortech.2013.05.114"
"   Li WY, 2022, ENVIRON RES, V212, DOI 10.1016/j.envres.2022.113267"
"   Li ZT, 2018, J HAZARD MATER, V344, P1, DOI 10.1016/j.jhazmat.2017.09.036"
"   Liaquat F, 2021, ENVIRON TECHNOL INNO, V21, DOI 10.1016/j.eti.2020.101237"
"   Lin XY, 2016, SCI TOTAL ENVIRON, V569, P97, DOI 10.1016/j.scitotenv.2016.06.121"
"   Liu GW, 2024, ARCH MICROBIOL, V206, DOI 10.1007/s00203-023-03755-x"

"   Liu J, 2022, INT J ENVIRON SCI TE, V19, P601, DOI 10.1007/s13762-021-03144-1"
"   Liu JJ, 2020, COLD REG SCI TECHNOL, V172, DOI 10.1016/j.coldregions.2020.102992"
"   Liu LN, 2019, SCI TOTAL ENVIRON, V667, P444, DOI 10.1016/j.scitotenv.2019.02.390"
"   Liu RH, 2014, BIODEGRADATION, V25, P903, DOI 10.1007/s10532-014-9709-y"
"   Liu SL, 2015, ECOTOX ENVIRON SAFE, V119, P35, DOI 10.1016/j.ecoenv.2015.04.053"
"   Liu S, 2011, J ENVIRON SCI-CHINA, V23, P1544, DOI 10.1016/S1001-0742(10)60570-0"
"   Lopareva-Pohu A, 2011, ENVIRON POLLUT, V159, P1721, DOI 10.1016/j.envpol.2011.02.030"
"   Lu ZW, 2013, ECOTOX ENVIRON SAFE, V91, P147, DOI 10.1016/j.ecoenv.2013.01.017"
"   Madhav S, 2024, INT J ENVIRON SCI TE, V21, P1099, DOI 10.1007/s13762-023-04950-5"
"   Magnin JP, 2014, CAN J MICROBIOL, V60, P829, DOI 10.1139/cjm-2014-0231"
"   Mahar A, 2016, ECOTOX ENVIRON SAFE, V126, P111, DOI 10.1016/j.ecoenv.2015.12.023"
"   Mahbub KR, 2017, SCI TOTAL ENVIRON, V576, P303, DOI 10.1016/j.scitotenv.2016.10.083"
"   Mahmood A, 2014, ARAB J CHEM, V7, P91, DOI 10.1016/j.arabjc.2013.07.002"
"   Mai ZM, 2021, BIOCHEM BIOPH RES CO, V585, P42, DOI 10.1016/j.bbrc.2021.10.069"
"   Maity Aniruddha, 2018, Proceedings of the Indian National Science Academy Part B Biological Sciences, V88, P595, DOI [10.1007/s40011-016-0796-x, 10.1007/s40011-016-0796-x]"
"   Maji P., 2022, INDIA POLLUT RES J, V41, P1008, DOI [10.53550/PR.2022.v41i03.036, DOI 10.53550/PR.2022.V41I03.036]"
"   Majumdar A., 2022, Global Arsenic Hazard: Ecotoxicology and Remediation, P291, DOI DOI 10.1007/978-3-031-16360-9_14"
"   Makino T, 2007, ENVIRON POLLUT, V147, P112, DOI 10.1016/j.envpol.2006.08.037"
"   Mallikarjunaiah S, 2020, NANOTECH LIFE SCI, P297, DOI 10.1007/978-3-030-31938-0_13"
"   Mantovani M, 2023, ENVIRON SCI POLLUT R, V30, P6730, DOI 10.1007/s11356-022-22506-x"
"   Marreiro DD, 2017, ANTIOXIDANTS-BASEL, V6, DOI 10.3390/antiox6020024"
"   Negrete JLM, 2013, REV FAC ING-UNIV ANT, P136"
"   Marrugo-Negrete J, 2017, ENVIRON RES, V154, P380, DOI 10.1016/j.envres.2017.01.021"
"   Martinez-Finley Ebany J, 2014, Curr Environ Health Rep, V1, P163, DOI 10.1007/s40572-014-0014-z"
"   Massah J, 2016, AMA-AGR MECH ASIA AF, V47, P44"
"   Masto RE, 2015, SOLID EARTH, V6, P811, DOI 10.5194/se-6-811-2015"
"   Mattsson K, 2021, CURR OPIN COLLOID IN, V56, DOI 10.1016/j.cocis.2021.101513"
"   McLaughlin MJ, 2000, AUST J SOIL RES, V38, P1037, DOI 10.1071/SR99128"
"   MCLAUGHLIN MJ, 1988, AUST J SOIL RES, V26, P333, DOI 10.1071/SR9880333"
"   Medina Ramirez A., 2013, Journal of Water Resource and Protection, V5, P58"
"   Megharaj M, 2003, CURR MICROBIOL, V47, P51, DOI 10.1007/s00284-002-3889-0"
"   Mehrotra T, 2021, J ENVIRON CHEM ENG, V9, DOI 10.1016/j.jece.2021.105920"
"   Migahed F., 2015, J CHEM BIOL, V6, P18"
"   Millaleo R, 2010, J SOIL SCI PLANT NUT, V10, P476, DOI 10.4067/S0718-95162010000200008"
"   Mishra A, 2013, CRIT REV ENV SCI TEC, V43, P1162, DOI 10.1080/10934529.2011.627044"
"   Mishra V, 2014, APPL WATER SCI, V4, P311, DOI 10.1007/s13201-013-0150-x"
"   Miszczak E, 2020, SCI TOTAL ENVIRON, V705, DOI 10.1016/j.scitotenv.2019.135776"
"   Mitra S, 2022, J KING SAUD UNIV SCI, V34, DOI 10.1016/j.jksus.2022.101865"
"   Mohamed AEA, 2020, MICRO NANO TECHNOL, P21, DOI 10.1016/B978-0-12-819786-8.00002-5"
"   Mohasin M, 2022, International Journal of Environment and Climate Change, P1231, DOI [10.9734/ijecc/2022/v12i121563, DOI 10.9734/IJECC/2022/V12I121563, 10.9734/ijecc/2022/v12i121563]"
"   Morcillo RJL, 2021, METABOLITES, V11, DOI 10.3390/metabo11060337"
"   Mossa AW, 2020, SCI TOTAL ENVIRON, V720, DOI 10.1016/j.scitotenv.2020.137441"
"   Mu'azu ND, 2016, SOIL SEDIMENT CONTAM, V25, P757, DOI 10.1080/15320383.2016.1213700"
"   Muddarisna N., 2013, PLANT SCI FEED, V3, P50"
"   Mukherjee P, 2019, FRONT MICROBIOL, V10, DOI 10.3389/fmicb.2019.01207"
"   Myhr AI, 2002, J AGR ENVIRON ETHIC, V15, P73, DOI 10.1023/A:1013814108502"
"   Naciri R, 2021, SCI REP-UK, V11, DOI 10.1038/s41598-021-84990-4"
"   Nag R, 2019, SCI TOTAL ENVIRON, V690, P460, DOI 10.1016/j.scitotenv.2019.06.512"
"   Navarro A, 2013, INT J MINER PROCESS, V119, P65, DOI 10.1016/j.minpro.2012.12.002"
"   Navas-Acien A, 2007, ENVIRON HEALTH PERSP, V115, P472, DOI 10.1289/ehp.9785"
"   Naz T, 2016, TOXICOL IND HEALTH, V32, P1619, DOI 10.1177/0748233715569900"
"   Nazarian H, 2016, PAK J BOT, V48, P945"
"   2019, ENVIRON EXP BIOL, V17, DOI [10.22364/eeb.17.08, DOI 10.22364/eeb.17.08]"
"   Ng Shee Ping, 2009, BMC Res Notes, V2, P38, DOI 10.1186/1756-0500-2-38"
"   Nguyen TH, 2021, CHEMOSPHERE, V282, DOI 10.1016/j.chemosphere.2021.131108"
"   Niam MI, 2021, INT J PHYS C SER, V1725"
"   Nwodo UU, 2012, INT J MOL SCI, V13, P14002, DOI 10.3390/ijms131114002"
"   Obire O., 2008, INT J AGR TECHNOLOGY, V4, P81"
"   Obrist D, 2018, AMBIO, V47, P116, DOI 10.1007/s13280-017-1004-9"
"   Odumo BO, 2014, ENVIRON SCI POLLUT R, V21, P12426, DOI 10.1007/s11356-014-3190-3"
"   Oladipo OG, 2018, BRAZ J MICROBIOL, V49, P29, DOI 10.1016/j.bjm.2017.06.003"
"   Olubode T. P., 2022, African Journal of Microbiology Research, V16, P268, DOI 10.5897/AJMR2022.9634"
"   Orozco-Mosqueda MD, 2023, PLANTS-BASEL, V12, DOI 10.3390/plants12030606"
"   Ouyang JP, 2013, MICROBIOL RES, V168, P455, DOI 10.1016/j.micres.2013.01.007"
"   Oves M, 2013, SAUDI J BIOL SCI, V20, P121, DOI 10.1016/j.sjbs.2012.11.006"
"   Ozyigit Ibrahim Ilker, 2021, International Journal of Life Sciences and Biotechnology, V4, P235, DOI [10.38001/ijlsb.833553, 10.38001/ijlsb.833553]"
"   Pacyna EG, 2010, ATMOS ENVIRON, V44, P2487, DOI 10.1016/j.atmosenv.2009.06.009"
"   Padhan D., 2021, Sustain. Technol., P285, DOI DOI 10.1002/9781119547976.CH12"
"   Page V, 2015, AGRONOMY-BASEL, V5, P447, DOI 10.3390/agronomy5030447"
"   Pandey B, 2012, J ENVIRON MANAGE, V101, P59, DOI 10.1016/j.jenvman.2012.01.029"
"   Pant G, 2021, J HAZARD MATER, V405, DOI 10.1016/j.jhazmat.2020.124631"
"   Park JH, 2011, PLANT SOIL, V348, P439, DOI 10.1007/s11104-011-0948-y"
"   Pat-Espadas AM, 2018, WATER-SUI, V10, DOI 10.3390/w10111685"
"   Patel J, 2010, APPL BIOCHEM BIOTECH, V160, P232, DOI 10.1007/s12010-009-8540-0"
"   Pathak A, 2009, J ENVIRON MANAGE, V90, P2343, DOI 10.1016/j.jenvman.2008.11.005"
"   Patriarca M, 2022, J ANAL ATOM SPECTROM, V37, P410, DOI [10.1039/d2ja90005j, 10.1039/D2JA90005J]"
"   Pazos M, 2006, CHEMOSPHERE, V62, P817, DOI 10.1016/j.chemosphere.2005.04.071"
"   Pedroza-Garcia JA, 2022, PLANT J, V109, P490, DOI 10.1111/tpj.15567"
"   Pei SL, 2020, J HAZARD MATER, V398, DOI 10.1016/j.jhazmat.2020.122959"

"   Peter R., 2011, ENCY ENV HLTH, V2, P199, DOI DOI 10.1016/B978-0-444-63951-6.00481-2"
"   Piao HS, 2006, ENVIRON POLLUT, V139, P498, DOI 10.1016/j.envpol.2005.06.005"
"   Pietri GP, 2021, FRONT MOL BIOSCI, V8, DOI 10.3389/fmolb.2021.745360"
"   Pietro-Souza W, 2020, CHEMOSPHERE, V240, DOI 10.1016/j.chemosphere.2019.124874"
"   Popa RG, 2021, NAT GEOSCI, V14, P781, DOI 10.1038/s41561-021-00827-9"
"   Pratush A, 2018, INT MICROBIOL, V21, P97, DOI 10.1007/s10123-018-0012-3"
"   Priyadarshanee M, 2021, J ENVIRON CHEM ENG, V9, DOI 10.1016/j.jece.2020.104686"
"   Priyadarshini E, 2021, CHEMOSPHERE, V274, DOI 10.1016/j.chemosphere.2021.129976"
"   Pushie MJ, 2014, CHEM REV, V114, P8499, DOI 10.1021/cr4007297"
"   Qaswar M, 2020, J SOIL SEDIMENT, V20, P2093, DOI 10.1007/s11368-020-02576-5"
"   Qian C, 2018, TRAC-TREND ANAL CHEM, V109, P19, DOI 10.1016/j.trac.2018.09.021"
"   Qin HQ, 2020, ENVIRON POLLUT, V258, DOI 10.1016/j.envpol.2019.113777"
"   Qiu JJ, 2021, APPL MICROBIOL BIOT, V105, P6477, DOI 10.1007/s00253-021-11497-3"
"   Quiton KG, 2018, SUSTAIN ENVIRON RES, V28, P206, DOI 10.1016/j.serj.2018.04.002"
"   Rahman Z, 2020, ENVIRON SCI POLLUT R, V27, P27563, DOI 10.1007/s11356-020-08903-0"
"   Rahman Z, 2020, J HAZARD MATER, V396, DOI 10.1016/j.jhazmat.2020.122682"
"   Rahman Z, 2019, J BASIC MICROB, V59, P477, DOI 10.1002/jobm.201900024"
"   Rai R., 2016, Plant responses to xenobiotics, P127, DOI [DOI 10.1007/978-981-10-2860-16, DOI 10.1007/978-981-10-2860-1_6]"
"   Rajkumar M, 2010, TRENDS BIOTECHNOL, V28, P142, DOI 10.1016/j.tibtech.2009.12.002"
"   Rajmohan KS, 2020, INDIAN J MICROBIOL, V60, P125, DOI 10.1007/s12088-019-00841-x"
"   Rajouriya K, 2020, POLLUTION, V6, P555, DOI 10.22059/poll.2020.294483.728"
"   Ramya D., 2021, FUNGI BIOPROSPECTS S, V2, P331, DOI [10.1016/B978-0-12-821925-6.00015-0, DOI 10.1016/B978-0-12-821925-6.00015-0]"
"   Ren J, 2020, J SOIL SEDIMENT, V20, P3041, DOI 10.1007/s11368-019-02403-6"
"   Ribeiro e Sousa L, 2020, SOFT ROCK MECH ENG"
"   Richardson AE, 2001, AUST J PLANT PHYSIOL, V28, P897, DOI 10.1071/PP01093"
"   Rizvi A, 2020, ENVIRON MONIT ASSESS, V192, DOI 10.1007/s10661-020-08758-5"
"   Robles I, 2015, ELECTROCHIM ACTA, V181, P68, DOI 10.1016/j.electacta.2015.04.099"
"   Rohde M, 2019, MICROBIOL SPECTR, V7, DOI 10.1128/microbiolspec.GPP3-0044-2018"
"   Rosestolato D, 2015, CHEM ENG J, V264, P16, DOI 10.1016/j.cej.2014.11.074"
"   Rout GR, 2015, Reviews in Agricultural Science, V3, P1, DOI [10.7831/ras.3.1, 10.7831/ras.3.1, DOI 10.7831/RAS.3.1]"
"   Ruiz ON, 2009, CURR OPIN BIOTECH, V20, P213, DOI 10.1016/j.copbio.2009.02.010"
"   Ruqia N., 2015, JOURNALOFPHARMACEUTI, V7, P89"
"   Sadej W, 2020, J ENVIRON SCI HEAL A, V55, P82, DOI 10.1080/10934529.2019.1667671"
"   Sader JA, 2020, GEOCHEM-EXPLOR ENV A, V20, P167, DOI 10.1144/geochem2019-049"
"   Safari F, 2019, ECOTOX ENVIRON SAFE, V183, DOI 10.1016/j.ecoenv.2019.109542"
"   Sandeep G., 2019, Int. J. Chem. Stud., V7, P1612"
"   Santhoshkumar P, 2023, INT J PLANT SOIL SCI, V35"
"   Soares TFSN, 2020, ECOTOX ENVIRON SAFE, V193, DOI 10.1016/j.ecoenv.2020.110296"
"   Saravanan A, 2022, CHEMOSPHERE, V298, DOI 10.1016/j.chemosphere.2022.134341"
"   Sarubbo LA, 2015, CHEM ECOL, V31, P707, DOI 10.1080/02757540.2015.1095293"
"   Sasaki Y, 2005, BIOSCI BIOTECH BIOCH, V69, P1394, DOI 10.1271/bbb.69.1394"
"   Sati M., 2014, International Journal of Advanced Research, V2, P923"
"   Sayqal A, 2021, APPL BIONICS BIOMECH, V2021, DOI 10.1155/2021/1609149"
"   Scammell MK, 2019, SEMIN NEPHROL, V39, P230, DOI 10.1016/j.semnephrol.2019.02.001"
"   Schippers A, 2014, ADV BIOCHEM ENG BIOT, V141, P1, DOI 10.1007/10_2013_216"
"   Schneider L, 2018, MAR POLLUT BULL, V131, P468, DOI 10.1016/j.marpolbul.2018.04.046"
"   Selim S, 2021, PHYSIOL PLANTARUM, V173, P978, DOI 10.1111/ppl.13496"
"   Shabaan M, 2021, INT J PHYTOREMEDIAT, V23, P837, DOI 10.1080/15226514.2020.1859988"
"   Shabbir Z, 2020, CHEMOSPHERE, V259, DOI 10.1016/j.chemosphere.2020.127436"
"   Shafiquzzaman Siddiquee Shafiquzzaman Siddiquee, 2015, Journal of Microbial and Biochemical Technology, V7, P384, DOI 10.4172/1948-5948.1000243"
"   Shah KT, 2017, International Journal of Life-Sciences Scientific Research, V3, P1142, DOI [10.21276/ijlssr.2017.3.4.7, 10.21276/ijlssr.2017.3.4.7, DOI 10.21276/IJLSSR.2017.3.4.7]"
"   Shahzad B, 2018, PLANT PHYSIOL BIOCH, V132, P641, DOI 10.1016/j.plaphy.2018.10.014"
"   Shao W, 2019, INT J ENV RES PUB HE, V16, DOI 10.3390/ijerph16122172"
"   Sharma B, 2021, BIORESOURCE TECHNOL, V320, DOI 10.1016/j.biortech.2020.124330"
"   Sharma KR, 2023, INT J ENVIRON SCI TE, V20, P943, DOI 10.1007/s13762-022-03914-5"
"   Sharma P, 2021, J HAZARD MATER, V416, DOI 10.1016/j.jhazmat.2021.125855"
"   Sharma S, 2018, 3 BIOTECH, V8, DOI 10.1007/s13205-018-1237-8"
"   Sharma V., 2014, J DRUG DELIVERY THER, V4, P115, DOI [10.22270/jddt.v4i4.925, DOI 10.22270/JDDT.V4I4.925]"
"   Sharma Y, 2022, SOIL SEDIMENT CONTAM, V31, P1043, DOI 10.1080/15320383.2022.2041542"
"   Shen SL, 2023, J HAZARD MATER, V449, DOI 10.1016/j.jhazmat.2023.131040"
"   Shen ZM, 2007, ENVIRON POLLUT, V150, P193, DOI 10.1016/j.envpol.2007.02.004"
"   Sheng GP, 2013, WATER RES, V47, P607, DOI 10.1016/j.watres.2012.10.037"
"   Silva RA, 2018, MIN PROC EXT MET REV, V39, P395, DOI 10.1080/08827508.2018.1459619"
"   Singh JS, 2011, GENE, V480, P1, DOI 10.1016/j.gene.2011.03.001"
"   Singh P, 2022, FRONT MICROBIOL, V13, DOI 10.3389/fmicb.2022.898979"
"   Singh R, 2020, J BIOTECHNOL, V323, P283, DOI 10.1016/j.jbiotec.2020.09.004"
"   Singh S, 2020, ENVIRON SCI POLLUT R, V27, P27181, DOI 10.1007/s11356-019-04974-w"
"   Singh V. B., 2023, Weathering and erosion processes in the natural environment"
"   Deb AKS, 2023, ACS OMEGA, V8, P32204, DOI 10.1021/acsomega.3c05116"
"   Sivarajan S. P., 2017, Science and policy: nutrient management challenges for the next generation, P1"
"   SL B., 2014, India Int J Curr Eng Technol, V4, P1314"
"   Smily J. R. M. B., 2017, Hayati Journal of Biosciences, V24, P65, DOI [10.1016/j.hjb.2017.08.005, 10.1016/j.hjb.2017.08.005]"
"   Smith L.A., 1995, REMEDIAL OPTIONS MET"
"   Song QB, 2022, FRONT MICROBIOL, V13, DOI 10.3389/fmicb.2022.952021"
"   Sperdouli I, 2022, TOXICS, V10, DOI 10.3390/toxics10120715"
"   Spinosa L, 2023, Standards, V3, P385, DOI [10.3390/standards3040027, 10.3390/standards3040027, DOI 10.3390/STANDARDS3040027]"

"   Sriprang R, 2003, APPL ENVIRON MICROB, V69, P1791, DOI 10.1128/AEM.69.3.1791-1796.2003"
"   Srivastava N, 2022, MINIATURIZED ANALYTI, P279, DOI DOI 10.1002/9783527827213.CH13"
"   Srivastava S, 2012, APPL MICROBIOL BIOT, V95, P1275, DOI 10.1007/s00253-012-3976-5"
"   Srivastava S, 2023, J SOIL SCI PLANT NUT, V23, P22, DOI 10.1007/s42729-022-00958-x"
"   Stabile P, 2023, MINERALS-BASEL, V13, DOI 10.3390/min13111378"
"   Streets DG, 2017, ENVIRON SCI TECHNOL, V51, P5969, DOI 10.1021/acs.est.7b00451"
"   Sun Yingqi, 2023, 2023 IEEE 16th International Conference on Electronic Measurement & Instruments (ICEMI), P189, DOI 10.1109/ICEMI59194.2023.10270694"
"   Suvarapu LN, 2017, TOXICOL IND HEALTH, V33, P79, DOI 10.1177/0748233716654827"
"   Suzuki T, 2014, COLLOID SURFACE A, V440, P145, DOI 10.1016/j.colsurfa.2012.09.050"
"   Syed A, 2021, ACS OMEGA, V6, P25076, DOI 10.1021/acsomega.1c04396"
"   Tamariz-Angeles C, 2021, MICROBIOL RES, V250, DOI 10.1016/j.micres.2021.126811"
"   Tang XQ, 2018, CHEM ENG J, V337, P210, DOI 10.1016/j.cej.2017.12.111"
"   Tariq SR, 2016, SCIENTIFICA, V2016, DOI 10.1155/2016/7575239"
"   Tauriainen S, 1998, BIOSENS BIOELECTRON, V13, P931, DOI 10.1016/S0956-5663(98)00027-X"
"   Tchounwou PB, 2003, TOXICOL PATHOL, V31, P575, DOI 10.1080/01926230390242007"
"   Tian D, 2018, ENVIRON SCI POLLUT R, V25, P21118, DOI 10.1007/s11356-018-2243-4"
"   Timofeeva AM, 2022, PLANTS-BASEL, V11, DOI 10.3390/plants11223065"
"   Torimiro N, 2021, International journal of environmental bioremediation & biodegradation, V9, P8, DOI [10.12691/ijebb-9-1-2, DOI 10.12691/IJEBB-9-1-2]"
"   Torres-Quiroz C, 2021, INT J ENV RES PUB HE, V18, DOI 10.3390/ijerph18052530"
"   Trellu C, 2016, J HAZARD MATER, V306, P149, DOI 10.1016/j.jhazmat.2015.12.008"
"   Trifunovi?? V, 2021, Zastita Materijala, V62, P166, DOI [10.5937/zasmat2103166t, 10.5937/zasmat2103166T, DOI 10.5937/ZASMAT2103166T]"
"   Tsezos M, 2009, ADV MATER RES-SWITZ, V71-73, P527, DOI 10.4028/www.scientific.net/AMR.71-73.527"
"   Tsoi MF, 2021, SCI REP-UK, V11, DOI 10.1038/s41598-021-82435-6"
"   Tyagi M, 2011, BIODEGRADATION, V22, P231, DOI 10.1007/s10532-010-9394-4"
"   Ungureanu N, 2020, SUSTAINABILITY-BASEL, V12, DOI 10.3390/su12219055"
"   Rehman Z, 2023, SCI TOTAL ENVIRON, V867, DOI 10.1016/j.scitotenv.2023.161468"
"   Ur??k M, 2007, WATER AIR SOIL POLL, V186, P337, DOI 10.1007/s11270-007-9489-7"
"   USEPA, 1992, EPA625R92002 OFF RES"
"   Ustiatik R, 2022, BIOREMEDIAT J, V26, P208, DOI 10.1080/10889868.2021.1973950"
"   Vacar CL, 2021, J FUNGI, V7, DOI 10.3390/jof7050386"
"   Valls M, 2000, NAT BIOTECHNOL, V18, P661, DOI 10.1038/76516"
"   Va??inkov?? M, 2021, IOP Conference Series Earth and Environmental Science, V900, P012048, DOI [10.1088/1755-1315/900/1/012048, 10.1088/1755-1315/900/1/012048, DOI 10.1088/1755-1315/900/1/012048]"
"   Vazquez A., 2020, PLANT PHYSIOL BIOCH, V151, P166"
"   Waalkes M., 1996, Toxicology of metals"
"   WAALKES MP, 1992, FUND APPL TOXICOL, V19, P512, DOI 10.1016/0272-0590(92)90089-Z"
"   Wang F, 2024, HELIYON, V10, DOI 10.1016/j.heliyon.2024.e26023"
"   Wang JX, 2012, J HAZARD MATER, V221, P1, DOI 10.1016/j.jhazmat.2012.04.035"
"   Wang LW, 2020, CRIT REV ENV SCI TEC, V50, P2724, DOI 10.1080/10643389.2019.1705724"
"   Wang T, 2014, J HAZARD MATER, V278, P483, DOI 10.1016/j.jhazmat.2014.06.028"
"   Wang ZZ, 2020, ECOTOX ENVIRON SAFE, V203, DOI 10.1016/j.ecoenv.2020.110981"
"   Wei WH, 2018, INT J ENV RES PUB HE, V15, DOI 10.3390/ijerph15091987"
"   White CC., 2012, SYSTEMATIC APPROACH"
"   World Health Organization. Regional Office for the Western Pacific, 1996, WHO W PACIFIC REGION"
"   Wu CH, 2006, APPL ENVIRON MICROB, V72, P1129, DOI 10.1128/AEM.72.2.1129-1134.2006"
"   Wu C, 2021, J ENVIRON MANAGE, V296, DOI 10.1016/j.jenvman.2021.113185"
"   Wu CC, 2020, SCI TOTAL ENVIRON, V703, DOI 10.1016/j.scitotenv.2019.134812"
"   Wuana R.A., 2011, ISRN Ecology, DOI [10.5402/2011/402647, DOI 10.5402/2011/402647]"
"   Wygel CM, 2019, GEOHEALTH, V3, P338, DOI 10.1029/2018GH000181"
"   Xu HY, 2021, CAN J MICROBIOL, V67, P789, DOI 10.1139/cjm-2020-0571"
"   Xu JY, 2015, ENVIRON INT, V74, P42, DOI 10.1016/j.envint.2014.09.007"
"   Yaciuk PA., 2022, NW ARGENTINA APPL GE, V136, DOI [10.1016/j.apgeochem.2021.105152, DOI 10.1016/J.APGEOCHEM.2021.105152]"
"   Yang LM, 2016, FRONT PLANT SCI, V7, DOI 10.3389/fpls.2016.00190"
"   Yang P, 2018, INT J ENV RES PUB HE, V15, DOI 10.3390/ijerph15071330"
"   Yang YJ, 2020, SCI TOTAL ENVIRON, V708, DOI 10.1016/j.scitotenv.2019.135186"
"   Yazdi MD, 2021, LANCET PLANET HEALTH, V5, pE689, DOI 10.1016/S2542-5196(21)00204-7"
"   Yi SW, 2022, J HAZARD MATER, V424, DOI 10.1016/j.jhazmat.2021.127514"
"   Yildiz H, 2018, PROCESS BIOCHEM, V72, P41, DOI 10.1016/j.procbio.2018.06.009"
"   Yin Kun Yin Kun, 2019, Chemical Engineering Journal, V360, P1553, DOI 10.1016/j.cej.2018.10.226"
"   Younas H, 2022, ENVIRON SCI POLLUT R, V29, P57669, DOI 10.1007/s11356-022-19913-5"
"   Younas H, 2022, WATER AIR SOIL POLL, V233, DOI 10.1007/s11270-022-05749-9"
"   Yuan HY, 2021, J CLEAN PROD, V318, DOI 10.1016/j.jclepro.2021.128589"
"   Zahir F, 2005, ENVIRON TOXICOL PHAR, V20, P351, DOI 10.1016/j.etap.2005.03.007"
"   Zha FS, 2020, ENVIRON EARTH SCI, V79, DOI 10.1007/s12665-020-08990-9"
"   Zhang HY, 2020, CHEM ENG J, V398, DOI 10.1016/j.cej.2020.125657"
"   Zhang R, 2015, V2, DOI [10.13130/2283-3927/4785, 10.13130/2283-3927/4785, DOI 10.13130/2283-3927/4785]"
"   Zhang WW, 2012, APPL MICROBIOL BIOT, V93, P1305, DOI 10.1007/s00253-011-3454-5"
"   Zhang Y, 2023, GEOTEXT GEOMEMBRANES, V51, P179, DOI 10.1016/j.geotexmem.2023.06.004"
"   Zhao XW, 2005, PROCESS BIOCHEM, V40, P1611, DOI 10.1016/j.procbio.2004.06.014"
"   Zheng XJ, 2022, SUSTAINABILITY-BASEL, V14, DOI 10.3390/su142013058"
"   Zhou DM, 2004, CHEMOSPHERE, V56, P265, DOI 10.1016/j.chemosphere.2004.02.033"
"   Zhou HD, 2021, J HAZARD MATER, V408, DOI 10.1016/j.jhazmat.2020.124885"
"   Zhou Jun, 2013, Shengtaixue Zazhi, V32, P1532"
"   Zhu NL, 2020, ACS APPL MATER INTER, V12, P22948, DOI 10.1021/acsami.0c04512"
"   Zulfiqar U, 2023, PLANTS-BASEL, V12, DOI 10.3390/plants12173147"
NR 438
TC 11
Z9 12
U1 12
U2 46

PU SPRINGER
PI DORDRECHT
"PA VAN GODEWIJCKSTRAAT 30, 3311 GZ DORDRECHT, NETHERLANDS"
SN 0032-079X
EI 1573-5036
J9 PLANT SOIL
JI Plant Soil
PD MAY
PY 2025
VL 510
IS 1-2
BP 1
EP 47
DI 10.1007/s11104-024-06944-9
EA SEP 2024
PG 47
WC Agronomy; Plant Sciences; Soil Science
WE Science Citation Index Expanded (SCI-EXPANDED)
SC Agriculture; Plant Sciences
GA 2XG6V
UT WOS:001318928700001
DA 2026-04-06
ER

PT J
"AU Sun, YJ"
"   Yu, PY"
"   Chen, JZ"
"   Li, SM"
"   Jiang, LJ"
"AF Sun, Y. J."
"   Yu, P. Y."
"   Chen, J. Z."
"   Li, S. M."
"   Jiang, L. J."
TI EFFECTS OF SLIPPERY JACK (SUILLUS LUTEUS) ON THE HEAVY METAL
   ACCUMULATION AND SOIL PROPERTIES OF MASSON'S PINE (PINUS
   MASSONIANA LAMB) IN A MINING AREA OF CHINA
SO APPLIED ECOLOGY AND ENVIRONMENTAL RESEARCH
LA English
DT Article
DE bioremediation; heavy metal toxicity; enzyme activities; mycorrhiza;
   symbiosis systems
ID PHYSIOLOGICAL-RESPONSES; ECTOMYCORRHIZAL FUNGI; PLANT-GROWTH; SEEDLINGS;
   TOXICITY; PHYTOREMEDIATION; TOLERANCE; SITES
"AB Plant-microbial bioremediation is an efficient way to treat heavy metal contaminated soils. In order to reveal the potential mechanisms of ectomycorrhizal fungi mutualistic symbiosis for remediating heavy metal contaminated soils, the masson's pine associated with ectomycorrhizal fungi (Suillus luteus) and control treatments (plant roots without ectomycorrhizal fungi) were planted in a Pb and Zn contaminated mining area in the south of China The results showed that the growth of masson's pine seedlings were significantly higher in the treated group than in the control group. The bioconcentration factor (BCF) of heavy metals inoculated with Suillus luteus was 1 to 5 times higher than that of those in control group. However, the translocation factor (TF) of heavy metals in treated group decreased by 50% compare to the control group. Both selenium and potassium content significantly improved the functions of oxidation and stress resistance in symbiotic systems. The activity of the urease and alkaline phosphatase in the soil was significantly improved in treated group. The soil available N, P and K contents, and the ratio of C/N was increased compared to the control. In conclusion, the ectomycorrhizal fungi could improve soil physicochemical properties, and enhance nutrient absorption by the plants, which would be favorable to the growth of plants in the soil contaminated with heavy metals."
"C1 [Sun, Y. J.; Yu, P. Y.; Li, S. M.; Jiang, L. J.] Cent South Univ Forestry & Technol, Coll Life Sci & Technol, 498 South Shaoshan Rd, Changsha 410004, Hunan, Peoples R China."
"   [Chen, J. Z.] Hunan Acad Forestry, 658 South Shaoshan Rd, Changsha 410004, Hunan, Peoples R China."
C3 Central South University of Forestry & Technology; Hunan Academy of
   Forestry
"RP Jiang, LJ (corresponding author), Cent South Univ Forestry & Technol, Coll Life Sci & Technol, 498 South Shaoshan Rd, Changsha 410004, Hunan, Peoples R China."
EM znljiang2542@163.com
FU Key R&D Project of Science and Technology by Hunan Government
   [2019Sk2191]; Scientific Innovation Fund for Post-graduates of Central
   South University of Forestry and Technology [20183038]; Changsha
   Municipal Government [kq1801080]
FX This work supported by The Key R&D Project of Science and Technology
"   Granted by Hunan Government (2019Sk2191), Scientific Innovation Fund for"
   Post-graduates of Central South University of Forestry and Technology
   (20183038) and Changsha Municipal Government (kq1801080). And we helpful
   comments by the following graduate students for their assistance in
"   laboratory chemical analysis: Xiao Zhou, Xuechun Feng and Qinyi Wang."
"CR Adriaensen K, 2006, MYCORRHIZA, V16, P553, DOI 10.1007/s00572-006-0072-7"
"   Angelovicov?? L, 2015, CARPATH J EARTH ENV, V10, P193"
"   [Anonymous], BRIT MYCOLOGICAL SOC"
"   [Anonymous], 2014, ENVIRON EXP BOT, DOI DOI 10.1016/j.envexpbot.2013.10.018"
"   Ayangbenro AS, 2017, INT J ENV RES PUB HE, V14, DOI 10.3390/ijerph14010094"
"   Bao S D., 2010, Soil Agro-Chemistrical Analysis, V3rd"
"   Becerra-Castro C, 2012, J HAZARD MATER, V217, P350, DOI 10.1016/j.jhazmat.2012.03.039"

"   Burke DJ, 2011, SOIL BIOL BIOCHEM, V43, P795, DOI 10.1016/j.soilbio.2010.12.014"
"   Cairney JWG, 2011, PLANT SOIL, V344, P51, DOI 10.1007/s11104-011-0731-0"
"   Colpaert JV, 2005, MYCORRHIZA, V15, P628, DOI 10.1007/s00572-005-0009-6"
"   Eichlerov?? I, 2015, FUNGAL ECOL, V13, P10, DOI 10.1016/j.funeco.2014.08.002"
"   Guan YM., 1986, Soil Enzyme and Its Research Method"
"   Guo JK, 2020, J ENVIRON SCI, V88, P361, DOI 10.1016/j.jes.2019.10.001"
"   Huai WX, 2003, MYCORRHIZA, V13, P265, DOI 10.1007/s00572-003-0227-8"
"   Huang J, 2012, MYCORRHIZA, V22, P589, DOI 10.1007/s00572-012-0436-0"
"   Jentschke G, 2000, PHYSIOL PLANTARUM, V109, P107, DOI 10.1034/j.1399-3054.2000.100201.x"
"   K??hler J, 2018, NEW PHYTOL, V220, P1200, DOI 10.1111/nph.15208"
"   Krznaric E, 2010, ENVIRON MICROBIOL, V12, P2133, DOI 10.1111/j.1462-2920.2009.02082.x"
"   Krznaric E, 2009, ENVIRON POLLUT, V157, P1581, DOI 10.1016/j.envpol.2008.12.030"
"   Kurzawova V, 2012, NEW BIOTECHNOL, V30, P15, DOI 10.1016/j.nbt.2012.06.004"
"   Lalancette S, 2019, MYCOBIOLOGY, V47, P415, DOI 10.1080/12298093.2019.1660297"
"   Li K, 2019, PEERJ, V7, DOI 10.7717/peerj.6631"
"   Ma Y, 2019, J HAZARD MATER, V379, DOI 10.1016/j.jhazmat.2019.120813"
"   Nannipieri P, 2018, BIOL FERT SOILS, V54, P11, DOI 10.1007/s00374-017-1245-6"
"   Leguizamo MAO, 2017, CHEMOSPHERE, V168, P1230, DOI 10.1016/j.chemosphere.2016.10.075"
"   Perotto S, 2001, MINERVA BIOTECNOL, V13, P55"
"   Reddy MS, 2016, ENVIRON MICROBIOL, V18, P2446, DOI 10.1111/1462-2920.13149"
"   Sheoran V, 2011, CRIT REV ENV SCI TEC, V41, P168, DOI 10.1080/10643380902718418"
"   Shi X, 2011, J ENVIRON SCI, V23, P266, DOI 10.1016/S1001-0742(10)60402-0"
"   Shukla OP, 2011, WASTE MANAGE, V31, P115, DOI 10.1016/j.wasman.2010.08.022"
"   Sinsabaugh RL, 2010, SOIL BIOL BIOCHEM, V42, P391, DOI 10.1016/j.soilbio.2009.10.014"
"   Sousa NR, 2012, SCI TOTAL ENVIRON, V414, P63, DOI 10.1016/j.scitotenv.2011.10.053"
"   Sozoniuk M, 2019, PHYSIOL MOL BIOL PLA, V25, P1377, DOI 10.1007/s12298-019-00712-1"
"   Sun FF, 2010, J ENVIRON SCI, V22, P1006, DOI 10.1016/S1001-0742(09)60211-4"
"   TAM PCF, 1995, MYCORRHIZA, V5, P181, DOI 10.1007/BF00203335"
"   Taniguchi T, 2008, SOIL BIOL BIOCHEM, V40, P1235, DOI 10.1016/j.soilbio.2007.12.017"
"   Wang ZH, 2019, GEOCHEM-EXPLOR ENV A, V19, P1, DOI 10.1144/geochem2017-046"
"   Wuana R.A., 2011, ISRN Ecology, DOI [10.5402/2011/402647, DOI 10.5402/2011/402647]"
"   Xue L, 2014, CLEAN-SOIL AIR WATER, V42, P81, DOI 10.1002/clen.201200490"
"   Yin D, 2014, CURR MICROBIOL, V69, P334, DOI 10.1007/s00284-014-0589-5"
"   Yu PY, 2020, J HAZARD MATER, V381, DOI 10.1016/j.jhazmat.2019.121203"
"   Zhang T, 2017, ACTA PHYSIOL PLANT, V39, DOI 10.1007/s11738-017-2392-y"
"   Zhang X, 2018, J ENVIRON MANAGE, V223, P132, DOI 10.1016/j.jenvman.2018.06.025"
"   Zhang Y, 2019, J ENVIRON MANAGE, V246, P796, DOI 10.1016/j.jenvman.2019.06.030"
"   Zhao X, 2013, ENVIRON SCI POLLUT R, V21, P5076"
NR 45
TC 5
Z9 8
U1 4
U2 50
PU ALOKI Applied Ecological Research and Forensic Inst Ltd
PI Budapest
"PA Kassa u. 118, Budapest, HUNGARY"
SN 1589-1623
EI 1785-0037
J9 APPL ECOL ENV RES
JI Appl. Ecol. Environ. Res.
PY 2020
VL 18
IS 2
BP 3741
EP 3755
DI 10.15666/aeer/1802_37413755
PG 15
WC Ecology; Environmental Sciences
WE Science Citation Index Expanded (SCI-EXPANDED)
SC Environmental Sciences & Ecology
GA LK2VS
UT WOS:000530720800108
OA gold
DA 2026-04-06
ER

PT J
"AU Zhang, XR"
"   Gong, ZQ"
"   Allinson, G"
"   Li, XJ"
"   Jia, CY"
"AF Zhang, Xiaorong"
"   Gong, Zongqiang"
"   Allinson, Graeme"
"   Li, Xiaojun"
"   Jia, Chunyun"
TI Joint effects of bacterium and biochar in remediation of
   antibiotic-heavy metal contaminated soil and responses of resistance
   gene and microbial community
SO CHEMOSPHERE
LA English
DT Article
DE Bacterium; Biochar; Resistance genes; Network analysis

ID SWINE MANURE; OXYTETRACYCLINE; TETRACYCLINE; ENVIRONMENT; PREVALENCE;
MECHANISMS; WATER; ASSOCIATION; RESISTOME; ABUNDANCE
"AB Soils containing both veterinary antibiotics (VAs) and heavy metals necessitate effective remediation approaches, and microbial and molecular levels of the results should be further examined. Here, a novel material combining waste fungus chaff-based biochar (WFCB) and Herbaspirillum huttiense (HHS1) was established to immobilize copper (Cu) and zinc (Zn) and degrade oxytetracycline (OTC) and enrofloxacin (ENR). Results showed that the combined material exhibited high immobilization of Cu (85.5%) and Zn (64.4%) and great removals of OTC (41.9%) and ENR (40.7%). Resistance genes including tet(PB), tetH, tetR, tetS, tetT, tetM, aacA/aphD, aacC, aadA9, and czcA were reduced. Abundances of potential hosts of antibiotic resistance genes (ARGs) including phylum Proteobacteria and genera Brevundimonas and Rhodanobacter were altered. Total phosphorus and pH were the factors driving the VA degrading microorganisms and potential hosts of ARGs. The combination of WFCB and HHS1 can serve as an important bioresource for immobilizing heavy metals and removing VAs in the contaminated soil."
"C1 [Zhang, Xiaorong; Gong, Zongqiang; Li, Xiaojun; Jia, Chunyun] Chinese Acad Sci, Inst Appl Ecol, Key Lab Pollut Ecol & Environm Engn, Shenyang 110016, Peoples R China."
" [Zhang, Xiaorong] Univ Chinese Acad Sci, Beijing 100049, Peoples R China."
" [Allinson, Graeme] RMIT Univ, Sch Sci, Melbourne, Vic 3000, Australia."
"C3 Chinese Academy of Sciences; Shenyang Institute of Applied Ecology, CAS;"
" Chinese Academy of Sciences; University of Chinese Academy of Sciences,"
CAS; Royal Melbourne Institute of Technology (RMIT)
"RP Gong, ZQ (corresponding author), Chinese Acad Sci, Inst Appl Ecol, Key Lab Pollut Ecol & Environm Engn, Shenyang 110016, Peoples R China."
EM zhangxiaorong191@mails.ucas.ac.cn; zgong@iae.ac.cn;
graeme.allinson@rmit.edu.au; lixiaojun@iae.ac.cn; jiachunyun@iae.ac.cn
"RI li, xiaojun/O-6854-2016; Zhang, Xiaorong/LGZ-0883-2024"
FU National Key Research and Development Program of China [2019YFD1100504]
FX Acknowledgements This work was financially supported by the National Key
Research and Development Program of China [2019YFD1100504] .
"CR Allen HK, 2010, NAT REV MICROBIOL, V8, P251, DOI 10.1038/nrmicro2312"
" [Anonymous], 2018, Soil environmental quality construction land soil pollution risk control standard"
" Bayat Zeynab, 2015, Open Microbiol J, V9, P48, DOI 10.2174/1874285801509010048"
" Chen JF, 2019, BIORESOURCE TECHNOL, V276, P236, DOI 10.1016/j.biortech.2019.01.006"
" Chen XL, 2022, SCI TOTAL ENVIRON, V814, DOI 10.1016/j.scitotenv.2021.152852"
" Cummings DE, 2011, ENVIRON SCI TECHNOL, V45, P447, DOI 10.1021/es1029206"
" Cytryn E., 2018, FRONT MICROBIOL, V9, P1"
" Duan ML, 2019, ECOTOX ENVIRON SAFE, V180, P114, DOI 10.1016/j.ecoenv.2019.05.005"
" Fan NS, 2020, J ENVIRON MANAGE, V262, DOI 10.1016/j.jenvman.2020.110375"
" Fu YL, 2022, PROCESS SAF ENVIRON, V160, P62, DOI 10.1016/j.psep.2022.02.006"
" Gao FZ, 2020, SCI TOTAL ENVIRON, V741, DOI 10.1016/j.scitotenv.2020.140482"
" Gillings MR, 2015, ISME J, V9, P1269, DOI 10.1038/ismej.2014.226"
" Gu JY, 2021, SCI TOTAL ENVIRON, V776, DOI 10.1016/j.scitotenv.2021.145959"
" Guo HH, 2020, BIORESOURCE TECHNOL, V309, DOI 10.1016/j.biortech.2020.122802"
" Guo T, 2018, SCI TOTAL ENVIRON, V635, P995, DOI 10.1016/j.scitotenv.2018.04.194"
" Guo X, 2021, J HAZARD MATER, V413, DOI 10.1016/j.jhazmat.2021.125444"
" Guo X, 2020, ECOTOX ENVIRON SAFE, V205, DOI 10.1016/j.ecoenv.2020.111362"
" Guo X, 2019, BIORESOURCE TECHNOL, V291, DOI 10.1016/j.biortech.2019.121916"
" Harrabi M, 2019, SCI TOTAL ENVIRON, V648, P962, DOI 10.1016/j.scitotenv.2018.08.193"
" H??lzel CS, 2012, ENVIRON RES, V113, P21, DOI 10.1016/j.envres.2012.01.002"
" Hossain MZ, 2020, BIOCHAR, V2, P379, DOI 10.1007/s42773-020-00065-z"
" Hsu JT, 2014, J HAZARD MATER, V277, P34, DOI 10.1016/j.jhazmat.2014.02.016"
" Hu W, 2012, INT J ENVIRON AN CH, V92, P698, DOI 10.1080/03067319.2010.520122"
" Huang HN, 2016, BIORESOURCE TECHNOL, V218, P1284, DOI 10.1016/j.biortech.2016.07.097"
" Huang J., 2021, BIORESOUR TECHNOL RE, V15"
" Huang JJ, 2020, ECOTOX ENVIRON SAFE, V193, DOI 10.1016/j.ecoenv.2020.110349"
" Igun OT, 2019, INT BIODETER BIODEGR, V142, P1, DOI 10.1016/j.ibiod.2019.04.008"
" Islam MS, 2021, SCI TOTAL ENVIRON, V785, DOI 10.1016/j.scitotenv.2021.147163"
" Jia X, 2013, CELL, V152, P68, DOI 10.1016/j.cell.2012.12.019"
" Jiang YT, 2022, CHEMOSPHERE, V286, DOI 10.1016/j.chemosphere.2021.131721"
" Kwon G, 2020, J HAZARD MATER, V400, DOI 10.1016/j.jhazmat.2020.123242"
" Li D, 2020, CHEMOSPHERE, V241, DOI 10.1016/j.chemosphere.2019.125039"
" Li J, 2012, ENVIRON HEALTH PERSP, V120, P1144, DOI 10.1289/ehp.1104776"
" Li ZJ, 2019, J INTEGR AGR, V18, P1953, DOI 10.1016/S2095-3119(18)62121-5"
" Liang ZJ, 2013, CHEMOSPHERE, V92, P1383, DOI 10.1016/j.chemosphere.2013.05.014"
" Liu CX, 2020, SCI TOTAL ENVIRON, V710, DOI 10.1016/j.scitotenv.2019.136329"
" Ma J, 2019, SCI TOTAL ENVIRON, V673, P357, DOI 10.1016/j.scitotenv.2019.04.103"
" Mazhar SH, 2021, SCI TOTAL ENVIRON, V755, DOI 10.1016/j.scitotenv.2020.142702"
" Meng J, 2017, CHEMOSPHERE, V180, P93, DOI 10.1016/j.chemosphere.2017.04.009"
" Peng S, 2017, J HAZARD MATER, V340, P16, DOI 10.1016/j.jhazmat.2017.06.059"
" Qian X, 2019, SCI TOTAL ENVIRON, V674, P383, DOI 10.1016/j.scitotenv.2019.04.197"
" Qian X, 2016, J HAZARD MATER, V315, P61, DOI 10.1016/j.jhazmat.2016.05.002"
" Qiu LL, 2021, J HAZARD MATER, V417, DOI 10.1016/j.jhazmat.2021.126022"
" Roberts MC, 2016, J ENVIRON QUAL, V45, P576, DOI 10.2134/jeq2015.04.0207"
" Ryan MP, 2018, VIRULENCE, V9, P480, DOI 10.1080/21505594.2017.1419116"
" Santos F, 2019, ECOTOX ENVIRON SAFE, V169, P894, DOI 10.1016/j.ecoenv.2018.11.078"
" Sardar MF, 2021, BIORESOURCE TECHNOL, V320, DOI 10.1016/j.biortech.2020.124403"
" Sergeant MJ, 2014, PLOS ONE, V9, DOI 10.1371/journal.pone.0091941"
" Shannon P, 2003, GENOME RES, V13, P2498, DOI 10.1101/gr.1239303"
" Shen Q, 2021, ECOTOX ENVIRON SAFE, V213, DOI 10.1016/j.ecoenv.2021.111981"
" Slipko K, 2021, J ENVIRON CHEM ENG, V9, DOI 10.1016/j.jece.2020.104783"
" Song W, 2017, BIORESOURCE TECHNOL, V231, P1, DOI 10.1016/j.biortech.2017.01.054"
" Subirats J, 2019, WATER RES, V167, DOI 10.1016/j.watres.2019.114895"
" Sun J, 2019, ELECTROCHIM ACTA, V299, P500, DOI 10.1016/j.electacta.2019.01.036"
" Tuo XX, 2018, CHEMOSPHERE, V197, P643, DOI 10.1016/j.chemosphere.2018.01.033"
" Wang H, 2017, ISME J, V11, P100, DOI 10.1038/ismej.2016.103"

"   Wang JH, 2018, SCI TOTAL ENVIRON, V624, P900, DOI 10.1016/j.scitotenv.2017.12.096"
"   Wang SL, 2013, MAR POLLUT BULL, V76, P7, DOI 10.1016/j.marpolbul.2013.08.025"
"   Wang Y, 2022, CHEM ENG J, V430, DOI 10.1016/j.cej.2021.130478"
"   Weber K, 2018, FUEL, V217, P240, DOI 10.1016/j.fuel.2017.12.054"
"   Xia L, 2019, BIOCHEM ENG J, V151, DOI 10.1016/j.bej.2019.107339"
"   Xie HJ, 2018, WATER RES, V143, P457, DOI 10.1016/j.watres.2018.05.061"
"   Xu Y, 2021, RESOUR CONSERV RECY, V168, DOI 10.1016/j.resconrec.2020.105342"
"   Yang L, 2018, ENVIRON SCI POLLUT R, V25, P104, DOI 10.1007/s11356-016-8062-6"
"   Yang R, 2021, CHEMOSPHERE, V268, DOI 10.1016/j.chemosphere.2020.129208"
"   Yin YN, 2017, FRONT MICROBIOL, V8, P1, DOI 10.3389/fmicb.2017.00344"
"   Zenati K, 2017, J GLOB ANTIMICROB RE, V10, P148, DOI 10.1016/j.jgar.2017.05.015"
"   Zhang FL, 2018, ENVIRON SCI POLLUT R, V25, P9547, DOI 10.1007/s11356-018-1251-8"
"   Zhang LL, 2021, SCI TOTAL ENVIRON, V788, DOI 10.1016/j.scitotenv.2021.147873"
"   Zhang Q, 2019, SCI TOTAL ENVIRON, V668, P1183, DOI 10.1016/j.scitotenv.2019.03.091"
"   Zhang XR, 2021, J HAZARD MATER, V413, DOI 10.1016/j.jhazmat.2021.125460"
"   Zhao XY, 2016, WASTE MANAGE, V56, P79, DOI 10.1016/j.wasman.2016.07.018"
"   Zhu XM, 2017, ENVIRON POLLUT, V227, P98, DOI [10.1016/j.envpol.2017.04.032, 10.1016/j.envPol.2017.04.032]"
"   Zhu YJ, 2017, CHEM ENG J, V325, P300, DOI 10.1016/j.cej.2017.05.073"
"   Zhu YG, 2017, NAT MICROBIOL, V2, DOI 10.1038/nmicrobiol.2016.270"
NR 75
TC 54
Z9 61
U1 20
U2 252
PU PERGAMON-ELSEVIER SCIENCE LTD
PI OXFORD
"PA THE BOULEVARD, LANGFORD LANE, KIDLINGTON, OXFORD OX5 1GB, the United Kingdom"
SN 0045-6535
EI 1879-1298
J9 CHEMOSPHERE
JI Chemosphere
PD JUL
PY 2022
VL 299
AR 134333
DI 10.1016/j.chemosphere.2022.134333
EA MAR 2022
PG 10
WC Environmental Sciences
WE Science Citation Index Expanded (SCI-EXPANDED)
SC Environmental Sciences & Ecology
GA 1C5QR
UT WOS:000793174000005
PM 35304205
DA 2026-04-06
ER

PT J
"AU Han, H"
"   Cai, H"
"   Wang, XY"
"   Hu, XM"
"   Chen, ZJ"
"   Yao, LG"
"AF Han, Hui"
"   Cai, Hong"
"   Wang, Xiaoyu"
"   Hu, Xiaomin"
"   Chen, Zhaojin"
"   Yao, Lunguang"
TI Heavy metal-immobilizing bacteria increase the biomass and reduce the Cd and Pb uptake by pakchoi (Brassica chinensis L.) in heavy metal-contaminated soil
SO ECOTOXICOLOGY AND ENVIRONMENTAL SAFETY
LA English
DT Article
DE Heavy metal immobilization; Plant growth-promoting bacteria; Extracellular adsorption; Bioprecipitation; Pakchoi
ID GROWTH PROMOTING BACTERIA; PLANT-GROWTH; CADMIUM UPTAKE; BACILLUS-SUBTILIS; ABSCISIC-ACID; ACCUMULATION; TOXICITY; TOMATO; RHIZOBACTERIA; INOCULATION
"AB Microbial immobilization is a novel and environmentally friendly technology that uses microbes to reduce metal availability in soil and accumulation of heavy metals in plants. We used urea agar plates to isolate urease-producing bacteria from the rhizosphere soil of pakchoi in Cd- and Pb-contaminated farmland and investigated their effects on Cd and Pb accumulation in pakchoi and the underlying mechanisms. The results showed that two urease-producing bacteria, Bacillus megaterium N3 and Serratia liquefaciens H12, were identified by screening. They had higher ability to produce urease (57.5 ms cm(-1) min(-1) OD600-1 and 76.4 ms cm(-1) min(-1) OD600-1, respectively). The two strains allowed for the immobilization of Cd and Pb by extracellular adsorption, bioprecipitation, and increasing the pH (from 6.94 to 7.05-7.09), NH4+ content (69.1%-127%), and NH4+/NO3- ratio (from 1.37 to 1.67-2.11), thereby reducing the DTPA-extractable Cd (35.3%-58.8%) and Pb (37.8%-62.2%) contents in the pakchoi rhizosphere soils and the Cd (76.5%-79.7%) and Pb (76.3%-83.5%) contents in the leaves (edible tissue) of pakchoi. The strains were highly resistant to heavy metal toxicity; produced IAA, siderophores and abscisic acid; and increased the NH4+/NO3- ratio, which might be

related to the two strains protecting pakchoi against the toxic effect of Cd and Pb and increasing pakchoi biomass. Thus, the results were supposed to strain resources and a theoretical basis for the remediation of Cd- and Pb-contaminated farmlands for the safe production of vegetables."
"C1 [Han, Hui; Cai, Hong; Wang, Xiaoyu; Hu, Xiaomin; Chen, Zhaojin; Yao, Lunguang] Nanyang Normal Univ, Coll Agr Engn, Collaborat Innovat Ctr Water Secur Water Source R, Nanyang 473061, Peoples R China."
"   [Han, Hui; Chen, Zhaojin; Yao, Lunguang] Nanyang Normal Univ, Henan Prov Academician Workstn Water Secur Water, Nanyang 473061, Peoples R China."
C3 Nanyang Normal University; Nanyang Normal University
"RP Chen, ZJ; Yao, LG (corresponding author), Nanyang Normal Univ, Coll Agr Engn, Collaborat Innovat Ctr Water Secur Water Source R, Nanyang 473061, Peoples R China."
EM Zhaojin_chen@163.com; lunguangyao@nynu.edu.cn
"RI Han, Hui/GYJ-6725-2022; Hu, Xiaomin/GRY-1539-2022; wang,"
xiaoyu/HJP-6901-2023
"OI Han, Hui/0000-0002-5218-0109; ?????????, Zhaojin Chen/0000-0003-1088-6821; "
"FU National Natural Science Foundation of China [41907143, 41601332]; Key"
Research Project of Colleges and Universities of Henan Province
Education Department [20A180023]
FX This work was supported by the National Natural Science Foundation of
"   China (Grant No. 41907143, 41601332) and the Key Research Project of"
Colleges and Universities of Henan Province Education Department (Grant
No. 20A180023).
"CR Bai J, 2014, J ENVIRON SCI-CHINA, V26, P2056, DOI 10.1016/j.jes.2014.07.015"
"   Banik A, 2018, SCI TOTAL ENVIRON, V630, P231, DOI 10.1016/j.scitotenv.2018.02.227"
"   Bhattacharya A, 2018, J ENVIRON MANAGE, V215, P143, DOI 10.1016/j.jenvman.2018.03.055"
"   Bolund P, 1999, ECOL ECON, V29, P293, DOI 10.1016/S0921-8009(99)00013-0"
"   Chang J, 2010, AQUAT BOT, V93, P216, DOI 10.1016/j.aquabot.2010.08.002"
"   Chen L, 2016, J HAZARD MATER, V312, P123, DOI 10.1016/j.jhazmat.2016.03.042"
"   Cohen AC, 2008, PLANT GROWTH REGUL, V54, P97, DOI 10.1007/s10725-007-9232-9"
"   Cohen AC, 2015, PHYSIOL PLANTARUM, V153, P79, DOI 10.1111/ppl.12221"
"   El-Meihy RM, 2019, ENVIRON EXP BOT, V162, P295, DOI 10.1016/j.envexpbot.2019.03.005"
"   Etesami H, 2018, ECOTOX ENVIRON SAFE, V147, P175, DOI 10.1016/j.ecoenv.2017.08.032"
"   Fan SK, 2014, FRONT PLANT SCI, V5, DOI 10.3389/fpls.2014.00721"
"   Fisher KA, 2017, GEODERMA, V285, P1, DOI 10.1016/j.geoderma.2016.09.012"
"   Gao X, 2019, J ENVIRON MANAGE, V251, DOI 10.1016/j.jenvman.2019.109610"
"   Govarthanan M, 2015, RSC ADV, V5, P54564, DOI 10.1039/c5ra06945a"
"   Grobelak A, 2018, J ENVIRON MANAGE, V227, P1, DOI 10.1016/j.jenvman.2018.08.075"
"   Han H, 2018, ECOTOX ENVIRON SAFE, V161, P526, DOI 10.1016/j.ecoenv.2018.06.033"
"   Han H, 2018, J HAZARD MATER, V353, P280, DOI 10.1016/j.jhazmat.2018.04.024"
"   He LY, 2009, ECOTOX ENVIRON SAFE, V72, P1343, DOI 10.1016/j.ecoenv.2009.03.006"
"   Kannan SK, 2006, ARCH MICROBIOL, V185, P202, DOI 10.1007/s00203-006-0088-6"
"   Kim SW, 2017, J AGR FOOD CHEM, V65, P1239, DOI 10.1021/acs.jafc.6b04339"
"   Kumari D, 2014, INT BIODETER BIODEGR, V94, P98, DOI 10.1016/j.ibiod.2014.07.007"
"   Li Y, 2017, ECOTOX ENVIRON SAFE, V138, P56, DOI 10.1016/j.ecoenv.2016.12.024"
"   Liu HL, 2019, J HAZARD MATER, V362, P9, DOI 10.1016/j.jhazmat.2018.09.032"
"   Lu Q, 2020, ENVIRON POLLUT, V257, DOI 10.1016/j.envpol.2019.113497"
"   Luo BF, 2012, J EXP BOT, V63, P3127, DOI 10.1093/jxb/ers036"
"   Madhaiyan M, 2007, CHEMOSPHERE, V69, P220, DOI 10.1016/j.chemosphere.2007.04.017"
"   Mao QQ, 2014, PLANT PHYSIOL, V166, P934, DOI 10.1104/pp.114.243766"
"   Moreira H, 2019, GEODERMA, V334, P72, DOI 10.1016/j.geoderma.2018.07.025"
"   Mori M, 2016, CHEMOSPHERE, V148, P487, DOI 10.1016/j.chemosphere.2016.01.012"
"   Nicholson FA, 2003, SCI TOTAL ENVIRON, V311, P205, DOI 10.1016/S0048-9697(03)00139-6"
"   Omoregie AI, 2017, ECOL ENG, V109, P65, DOI 10.1016/j.ecoleng.2017.09.012"
"   Pan W, 2019, ECOTOX ENVIRON SAFE, V177, P100, DOI 10.1016/j.ecoenv.2019.04.010"
"   Park JH, 2011, SCI TOTAL ENVIRON, V409, P853, DOI 10.1016/j.scitotenv.2010.11.003"
"   Pietri JCA, 2008, SOIL BIOL BIOCHEM, V40, P1856, DOI [10.1016/j.soilbio.2008.03.020, 10.1016/i.soilbio.2008.03.020]"
"   Rajkumar M, 2013, J ENVIRON MANAGE, V128, P973, DOI 10.1016/j.jenvman.2013.07.001"
"   Rehman MZU, 2018, ENVIRON POLLUT, V241, P557, DOI 10.1016/j.envpol.2018.06.005"
"   Rouillon M, 2017, ENVIRON POLLUT, V222, P557, DOI 10.1016/j.envpol.2016.11.024"
"   Saadani O, 2016, ECOTOX ENVIRON SAFE, V130, P263, DOI 10.1016/j.ecoenv.2016.04.032"
"   Saleem M, 2018, CHEMOSPHERE, V195, P606, DOI 10.1016/j.chemosphere.2017.12.117"
"   Sarwar N, 2010, J SCI FOOD AGR, V90, P925, DOI 10.1002/jsfa.3916"
"   Singh N, 2016, ECOTOX ENVIRON SAFE, V125, P25, DOI 10.1016/j.ecoenv.2015.11.020"
"   Singh VK, 2018, AGR ECOSYST ENVIRON, V267, P129, DOI 10.1016/j.agee.2018.08.020"
"   SSICA (Soil Sci Ch. Acad.), 1980, DISSERTATION"
"   Stefanowicz AM, 2014, GEODERMA, V235, P1, DOI 10.1016/j.geoderma.2014.06.020"
"   Sun LN, 2010, BIORESOURCE TECHNOL, V101, P501, DOI 10.1016/j.biortech.2009.08.011"
"   Teng ZD, 2019, J ENVIRON MANAGE, V231, P189, DOI 10.1016/j.jenvman.2018.10.012"
"   Tylova-Munzarova E, 2005, AQUAT BOT, V81, P326, DOI 10.1016/j.aquabot.2005.01.006"
"   Uchimiya M, 2010, J AGR FOOD CHEM, V58, P5538, DOI 10.1021/jf9044217"
"   Udeigwe TK, 2011, ENVIRON INT, V37, P258, DOI 10.1016/j.envint.2010.08.008"
"   Wan YA, 2018, ENVIRON POLLUT, V240, P523, DOI 10.1016/j.envpol.2018.04.044"
"   Wang DF, 2017, J AGR FOOD CHEM, V65, P8999, DOI 10.1021/acs.jafc.7b02370"
"   WATANABE F. S., 1965, SOIL SCI SOC AMER PROC, V29, P677"
"   Wess??n E, 2010, APPL SOIL ECOL, V45, P193, DOI 10.1016/j.apsoil.2010.04.003"
"   Wheaton G, 2015, MINERALS-BASEL, V5, P397, DOI 10.3390/min5030397"
"   Xie HL, 2009, PLANT SOIL, V318, P205, DOI 10.1007/s11104-008-9830-y"
"   Xu QR, 2018, J AGR FOOD CHEM, V66, P5229, DOI 10.1021/acs.jafc.8b00598"
"   Yun SW, 2016, GEODERMA, V270, P10, DOI 10.1016/j.geoderma.2015.11.036"
"   Zhang LX, 2018, J HAZARD MATER, V360, P32, DOI 10.1016/j.jhazmat.2018.07.075"
"   Zhang YF, 2011, J HAZARD MATER, V186, P1720, DOI 10.1016/j.jhazmat.2010.12.069"
"   Zhao XQ, 2019, INT J ENV RES PUB HE, V16, DOI 10.3390/ijerph16020268"
"   Zhu J, 2019, J EXP BOT, V70, P6363, DOI 10.1093/jxb/erz374"
"   Zhu XL, 2019, GEODERMA, V350, P1, DOI 10.1016/j.geoderma.2019.04.015"

NR 62
TC 91
Z9 116
U1 7
U2 218
PU ACADEMIC PRESS INC ELSEVIER SCIENCE
PI SAN DIEGO
"PA 525 B ST, STE 1900, SAN DIEGO, CA 92101-4495 USA"
SN 0147-6513
EI 1090-2414
J9 ECOTOX ENVIRON SAFE
JI Ecotox. Environ. Safe.
PD JUN 1
PY 2020
VL 195
AR 110375
DI 10.1016/j.ecoenv.2020.110375
PG 9
WC Environmental Sciences; Toxicology
WE Science Citation Index Expanded (SCI-EXPANDED)
SC Environmental Sciences & Ecology; Toxicology
GA LD0ZU
UT WOS:000525762800025
PM 32200142
DA 2026-04-06
ER

PT J
"AU Khatoon, Z"
"   Orozco-Mosqueda, MD"
"   Santoyo, G"
"AF Khatoon, Zobia"
"   Orozco-Mosqueda, Ma. del Carmen"
"   Santoyo, Gustavo"
TI Microbial Contributions to Heavy Metal Phytoremediation in Agricultural
   Soils: A Review
SO MICROORGANISMS
LA English
DT Review
DE PGPB; microbial synergies; meta-organism; plant-microbe associations;
   endophytes
ID ARBUSCULAR MYCORRHIZAL FUNGI; GROWTH-PROMOTING RHIZOBACTERIA;
   PLANT-GROWTH; TRANSPORTERS EXPRESSION; TRICHODERMA-HARZIANUM; ENDOPHYTIC
   BACTERIA; CADMIUM RESISTANCE; AMF COLONIZATION; RISK-ASSESSMENT; GENES
"AB Phytoremediation is a sustainable technique that employs plants to reinforce polluted environments such as agroecosystems. In recent years, new strategies involving the plant microbiome as an adjuvant in remediation processes have been reported. By leveraging this microbial assistance to remediate soils contaminated with heavy metals such As, Pb, Cd, Hg, and Cr, plants can sequester, degrade, or stabilize contaminants more efficiently. Remarkably, some plant species are known for their hyper-accumulative traits in synergy with their microbial partners and can successfully mitigate heavy metal pollutants. This sustainable biotechnology based on plant-microbe associations not only aids in environmental cleanup but also enhances biodiversity, improves soil structure, and promotes plant growth and health, making it a promising solution for addressing agro-pollution challenges worldwide. The current review article emphasizes the potential of synergistic plant-microbe interactions in developing practical and sustainable solutions for heavy metal remediation in agricultural systems, which are essential for food security."
"C1 [Khatoon, Zobia; Santoyo, Gustavo] Univ Michoacana, Inst Chem & Biol Res, Morelia 58030, Mexico."
"   [Orozco-Mosqueda, Ma. del Carmen] Tecnol Nacl Mex Celaya, Dept Ingn Bioquim & Ambiental, Celaya 38010, Mexico."
C3 Universidad Michoacana de San Nicolas de Hidalgo
"RP Santoyo, G (corresponding author), Univ Michoacana, Inst Chem & Biol Res, Morelia 58030, Mexico.; Orozco-Mosqueda, MD (corresponding author), Tecnol Nacl Mex Celaya, Dept Ingn Bioquim & Ambiental, Celaya 38010, Mexico."
EM carmen.orozco@itcelaya.edu.mx; gustavo.santoyo@umich.mx
"RI ; Santoyo, Gustavo/AAC-7178-2020"
"OI Orozco-Mosqueda, Ma del Carmen/0000-0002-4782-9751; Santoyo,"
   Gustavo/0000-0002-0374-9661
FU ICTI-Michoacn and CIC-UMSNH; CIC-UMSNH
FX This research was funded by the ICTI-Michoacan and CIC-UMSNH.
"CR Abbaszadeh-Dahaji P., 2016, PLANT MICROBE INTERA, P187"
"   Abou Alhamed MF, 2012, PLANT BIOLOGY, V14, P859, DOI 10.1111/j.1438-8677.2012.00608.x"
"   Abou-Aly HE, 2021, BIOCATAL AGR BIOTECH, V31, DOI 10.1016/j.bcab.2021.101911"
"   Abou-Shanab RAI, 2007, CHEMOSPHERE, V68, P360, DOI 10.1016/j.chemosphere.2006.12.051"
"   Achal V, 2012, J HAZARD MATER, V201, P178, DOI 10.1016/j.jhazmat.2011.11.067"
"   Adams P, 2007, MICROB ECOL, V54, P306, DOI 10.1007/s00248-006-9203-0"
"   Al Mamun A, 2024, TRANSGENIC RES, V33, P175, DOI 10.1007/s11248-024-00393-x"
"   Amna, 2015, INT J PHYTOREMEDIAT, V17, P981, DOI [10.1080/15226514.2014.989311, DOI 10.1080/15226514.2014.989311]"
"   Arriagada C, 2009, BIORESOURCE TECHNOL, V100, P6250, DOI 10.1016/j.biortech.2009.07.010"
"   Asad SA, 2019, CHEMOSPHERE, V217, P925, DOI 10.1016/j.chemosphere.2018.11.021"
"   Babu AG, 2014, ECOL ENG, V69, P186, DOI 10.1016/j.ecoleng.2014.03.055"
"   Babu AG, 2014, SCI TOTAL ENVIRON, V476, P561, DOI 10.1016/j.scitotenv.2013.12.119"
"   Baghaie AH, 2019, ENVIRON SCI POLLUT R, V26, P30794, DOI 10.1007/s11356-019-06237-0"
"   Balali-Mood M, 2021, FRONT PHARMACOL, V12, DOI 10.3389/fphar.2021.643972"
"   Becerra-Castro C, 2012, J HAZARD MATER, V217, P350, DOI 10.1016/j.jhazmat.2012.03.039"

"   Belimov AA, 2005, SOIL BIOL BIOCHEM, V37, P241, DOI 10.1016/j.soilbio.2004.07.033"
"   Berthelot C, 2016, FEMS MICROBIOL ECOL, V92, DOI 10.1093/femsec/fiw144"
"   Bibi S, 2018, CHEMOSPHERE, V211, P653, DOI 10.1016/j.chemosphere.2018.07.197"
"   Branco R, 2008, J BACTERIOL, V190, P6996, DOI 10.1128/JB.00289-08"
"   Cai XC, 2019, ECOTOX ENVIRON SAFE, V181, P472, DOI 10.1016/j.ecoenv.2019.06.036"
"   Cao LX, 2008, CHEMOSPHERE, V71, P1769, DOI 10.1016/j.chemosphere.2008.01.066"
"   Carlier JD, 2024, Environmental Advances, V15, P100479, DOI [10.1016/j.envadv.2023.100479, 10.1016/j.envadv.2023.100479, DOI 10.1016/J.ENVADV.2023.100479]"
"   Carvalho LM, 2006, PLANT SOIL, V285, P161, DOI 10.1007/s11104-006-9001-y"
"   Chakraborty J, 2014, ENVIRON SCI POLLUT R, V21, P14188, DOI 10.1007/s11356-014-3308-7"
"   Chamba I, 2017, CHEMOSPHERE, V188, P633, DOI 10.1016/j.chemosphere.2017.08.160"
"   Chang Q, 2018, ENVIRON POLLUT, V241, P607, DOI 10.1016/j.envpol.2018.06.003"
"   Chaouni LBA, 1996, PLASMID, V36, P1, DOI 10.1006/plas.1996.0025"
"   Chaturyedi R, 2018, ECOTOX ENVIRON SAFE, V148, P318, DOI 10.1016/j.ecoenv.2017.10.048"
"   Chen BD, 2003, CHEMOSPHERE, V50, P839, DOI 10.1016/S0045-6535(02)00228-X"
"   Chen GJ, 2015, SCI REP-UK, V5, DOI 10.1038/srep17419"
"   Chen LH, 2015, ECOTOX ENVIRON SAFE, V113, P460, DOI 10.1016/j.ecoenv.2014.12.033"
"   Chen XW, 2013, ENVIRON EXP BOT, V87, P92, DOI 10.1016/j.envexpbot.2012.08.002"
"   Chen XW, 2019, GEODERMA, V337, P749, DOI 10.1016/j.geoderma.2018.10.029"
"   Chen XW, 2012, CHEMOSPHERE, V89, P1248, DOI 10.1016/j.chemosphere.2012.07.054"
"   Chen YH, 2015, MYCORRHIZA, V25, P561, DOI 10.1007/s00572-015-0629-4"
"   Chen ZQ, 2024, SCI TOTAL ENVIRON, V932, DOI 10.1016/j.scitotenv.2024.173029"
"   Chiang HC, 2006, ENVIRON SCI TECHNOL, V40, P6792, DOI 10.1021/es061432y"
"   Chillappagari S, 2009, J BACTERIOL, V191, P2362, DOI 10.1128/JB.01616-08"
"   Coc??rta DM, 2016, INT J ENVIRON SCI TE, V13, P2025, DOI 10.1007/s13762-016-1031-2"
"   Deng SG, 2024, ENVIRON SCI TECHNOL, V58, P7870, DOI 10.1021/acs.est.4c00066"
"   Dimitroula H, 2015, J HAZARD MATER, V281, P114, DOI 10.1016/j.jhazmat.2014.08.005"
"   Ding SF, 2024, J HAZARD MATER, V469, DOI 10.1016/j.jhazmat.2024.134110"
"   Duprey A, 2014, J BIOL ENG, V8, DOI 10.1186/1754-1611-8-19"
"   Durand A, 2016, PLANT SOIL, V399, P179, DOI 10.1007/s11104-015-2691-2"
"   Franchi E, 2017, J SOIL SEDIMENT, V17, P1224, DOI 10.1007/s11368-015-1346-5"
"   Pinter IF, 2017, APPL SOIL ECOL, V109, P60, DOI 10.1016/j.apsoil.2016.10.003"
"   Ge ZM, 1996, FEMS MICROBIOL LETT, V145, P181, DOI 10.1016/S0378-1097(96)00405-3"
"   Glick BR, 2007, CRIT REV PLANT SCI, V26, P227, DOI 10.1080/07352680701572966"
"   Glick BR, 2010, BIOTECHNOL ADV, V28, P367, DOI 10.1016/j.biotechadv.2010.02.001"
"   G??hre V, 2006, PLANTA, V223, P1115, DOI 10.1007/s00425-006-0225-0"
"   Gola D, 2021, J ENVIRON CHEM ENG, V9, DOI 10.1016/j.jece.2021.105552"
"   Gola D, 2016, BIORESOURCE TECHNOL, V218, P388, DOI 10.1016/j.biortech.2016.06.096"
"   Gou JH, 2024, WATER RES, V254, DOI 10.1016/j.watres.2024.121423"
"   Grass G, 2005, BIOMETALS, V18, P437, DOI 10.1007/s10534-005-3718-6"
"   Grass G, 2000, J BACTERIOL, V182, P1390, DOI 10.1128/JB.182.5.1390-1398.2000"
"   Grobelak A, 2015, ECOL ENG, V84, P22, DOI 10.1016/j.ecoleng.2015.07.019"
"   Guarino F, 2020, CHEMOSPHERE, V251, DOI 10.1016/j.chemosphere.2020.126310"
"   Guo JK, 2020, CHEMOSPHERE, V246, DOI 10.1016/j.chemosphere.2020.125823"
"   Hansda A, 2017, 3 BIOTECH, V7, DOI 10.1007/s13205-017-0757-y"
"   Hao SY, 2024, FRONT PLANT SCI, V15, DOI 10.3389/fpls.2024.1327649"
"   Hao XW, 2024, MICROORGANISMS, V12, DOI 10.3390/microorganisms12061117"
"   Hassan Auwalu, 2024, Environmental Science and Pollution Research International, V31, P28671, DOI [10.1007/s11356-024-33018-1, 10.1007/s11356-024-33018-1]"
"   He N, 2024, DESALIN WATER TREAT, V317, DOI 10.1016/j.dwt.2024.100070"
"   Henne KL, 2009, BMC MICROBIOL, V9, DOI 10.1186/1471-2180-9-199"
"   Hnini M, 2024, PLANT STRESS, V11, DOI 10.1016/j.stress.2024.100391"
"   Hu YH, 2009, J MICROBIOL, V47, P277, DOI 10.1007/s12275-008-0278-9"
"   Huang CC, 2002, GENE, V301, P13, DOI 10.1016/S0378-1119(02)01086-7"
"   Huang Wenhao, 2024, Chemosphere, V363, P142869, DOI [10.1016/j.chemosphere.2024.142869, 10.1016/j.chemosphere.2024.142869]"
"   Huang WH, 2024, J ENVIRON CHEM ENG, V12, DOI 10.1016/j.jece.2024.112573"
"   Hui CY, 2023, ECOTOX ENVIRON SAFE, V267, DOI 10.1016/j.ecoenv.2023.115662"
"   Jacob JM, 2018, J ENVIRON MANAGE, V217, P56, DOI 10.1016/j.jenvman.2018.03.077"
"   Carlos MHJ, 2016, MICROBIOL RES, V188, P53, DOI 10.1016/j.micres.2016.05.001"
"   Jourand P, 2014, J PLANT PHYSIOL, V171, P164, DOI 10.1016/j.jplph.2013.10.011"
"   Kamnev AA, 2005, J TRACE ELEM MED BIO, V19, P91, DOI 10.1016/j.jtemb.2005.03.002"
"   Kang X, 2018, FRONT MICROBIOL, V9, DOI 10.3389/fmicb.2018.01853"
"   Ke T, 2021, ENVIRON POLLUT, V271, DOI 10.1016/j.envpol.2020.116314"
"   Khalid M, 2021, PEDOSPHERE, V31, P475, DOI 10.1016/S1002-0160(20)60091-1"
"   Khan MS, 2009, ENVIRON CHEM LETT, V7, P1, DOI 10.1007/s10311-008-0155-0"
"   Kong ZY, 2015, ENVIRON SCI POLLUT R, V22, P12479, DOI 10.1007/s11356-015-4530-7"
"   Konkolewska A, 2020, ENVIRON SCI POLLUT R, V27, P13809, DOI 10.1007/s11356-020-07885-3"
"   Kumar V, 2021, ENVIRON SCI POLLUT R, V28, P10375, DOI 10.1007/s11356-020-11491-8"
"   Kumar V, 2019, ECOTOX ENVIRON SAFE, V185, DOI 10.1016/j.ecoenv.2019.109734"
"   Kumar V, 2019, CHEMOSPHERE, V216, P449, DOI 10.1016/j.chemosphere.2018.10.066"
"   Leonh??user J, 2007, J BIOTECHNOL, V132, P469, DOI 10.1016/j.jbiotec.2007.08.018"
"   Li H, 2020, PLANT SOIL, V455, P121, DOI 10.1007/s11104-020-04678-y"
"   Li XR, 2023, ECOTOX ENVIRON SAFE, V255, DOI 10.1016/j.ecoenv.2023.114776"
"   Liu AR, 2022, ENVIRON POLLUT, V314, DOI 10.1016/j.envpol.2022.120303"
"   Liu YQ, 2024, J HAZARD MATER, V469, DOI 10.1016/j.jhazmat.2024.134085"
"   Loha A, 2018, BIOCHEM BIOPH RES CO, V507, P414, DOI 10.1016/j.bbrc.2018.11.053"
"   Lu RR, 2020, ECOTOX ENVIRON SAFE, V203, DOI 10.1016/j.ecoenv.2020.110988"
"   Ma Y, 2011, BIOTECHNOL ADV, V29, P248, DOI 10.1016/j.biotechadv.2010.12.001"
"   Ma Y, 2019, J HAZARD MATER, V379, DOI 10.1016/j.jhazmat.2019.120813"
"   Ma Y, 2016, J ENVIRON MANAGE, V174, P14, DOI 10.1016/j.jenvman.2016.02.047"
"   Ma Y, 2015, J ENVIRON MANAGE, V156, P62, DOI 10.1016/j.jenvman.2015.03.024"
"   Mahajan P, 2018, J TOXICOL-US, V2018, DOI 10.1155/2018/4864365"
"   Mallick I, 2018, SCI TOTAL ENVIRON, V610, P1239, DOI 10.1016/j.scitotenv.2017.07.234"

"   Mandal D, 2024, MICROBE-NETH, V4, DOI [10.1016/j.microb.2024.100132, 10.1016/j.microb.2024.100132]"
"   Manoj SR, 2020, J ENVIRON MANAGE, V254, DOI 10.1016/j.jenvman.2019.109779"
"   Marques APGC, 2013, CHEMOSPHERE, V92, P74, DOI 10.1016/j.chemosphere.2013.02.055"
"   Mishra S, 2022, CHEMOSPHERE, V294, DOI 10.1016/j.chemosphere.2022.133609"
"   Mitra A, 2024, INT J ENVIRON SCI TE, V21, P6405, DOI 10.1007/s13762-024-05466-2"
"   Montes-Robledo A, 2024, ENVIRON TECHNOL INNO, V33, DOI 10.1016/j.eti.2023.103521"
"   Moreira H, 2016, GEODERMA, V278, P1, DOI 10.1016/j.geoderma.2016.05.003"
"   Naik MM, 2012, ECOTOX ENVIRON SAFE, V79, P129, DOI 10.1016/j.ecoenv.2011.12.015"
"   Nanda M, 2019, AQUAT TOXICOL, V212, P1, DOI 10.1016/j.aquatox.2019.04.011"
"   Orozco-Mosqueda MD, 2022, MICROBIOL RES, V263, DOI 10.1016/j.micres.2022.127137"
"   Oyewole OA, 2019, SN APPL SCI, V1, DOI 10.1007/s42452-019-0879-4"
"   Oza T, 2024, J HAZARD MATER LETT, V5, DOI 10.1016/j.hazl.2023.100097"
"   Petrus AK, 2015, APPL ENVIRON MICROB, V81, P7833, DOI 10.1128/AEM.01982-15"
"   Pinto A P., 2015, Phytoremediation, P133, DOI [DOI 10.1007/978-3-319-10395-2_10, 10.1007/978-3-319-10395-2_10]"
"   Qin HC, 2024, MICROORGANISMS, V12, DOI 10.3390/microorganisms12040700"
"   Quan Lingtong, 2023, Chemosphere, V334, P138857, DOI [10.1016/j.chemosphere.2023.138857, 10.1016/j.chemosphere.2023.138857]"
"   Rakic T, 2021, PLANT SOIL, V462, P349, DOI 10.1007/s11104-021-04865-5"
"   Ren ZM, 2023, WATER AIR SOIL POLL, V234, DOI 10.1007/s11270-023-06061-w"
"   Rodrigue A, 2005, J BACTERIOL, V187, P2912, DOI 10.1128/JB.187.8.2912-2916.2005"
"   Ruiz ON, 2011, BMC BIOTECHNOL, V11, DOI 10.1186/1472-6750-11-82"
"   Santoyo G, 2021, AGRONOMY-BASEL, V11, DOI 10.3390/agronomy11020219"
"   Sasaki Y, 2006, TRANSGENIC RES, V15, P615, DOI 10.1007/s11248-006-9008-4"
"   Shi YA, 2017, ENVIRON SCI POLLUT R, V24, P417, DOI 10.1007/s11356-016-7693-y"
"   Singh SK, 2004, J BACTERIOL, V186, P7815, DOI 10.1128/JB.186.22.7815-7817.2004"
"   SMITH K, 1972, J BACTERIOL, V112, P761, DOI 10.1128/JB.112.2.761-772.1972"
"   Song JX, 2024, ENVIRON INT, V183, DOI 10.1016/j.envint.2023.108394"
"   Sun SM, 2023, CHEMOSPHERE, V330, DOI 10.1016/j.chemosphere.2023.138728"
"   Szuba A, 2017, PLANT SOIL, V412, P253, DOI 10.1007/s11104-016-3062-3"
"   Taghavi S, 2009, ANTON LEEUW INT J G, V96, P171, DOI 10.1007/s10482-008-9289-0"
"   Thijs S, 2016, FRONT MICROBIOL, V7, DOI 10.3389/fmicb.2016.00341"
"   Timmusk S, 2023, FRONT MICROBIOL, V14, DOI 10.3389/fmicb.2023.1141862"
"   Tirry N., 2018, Journal of Genetic Engineering and Biotechnology, V16, P613, DOI [10.1016/j.jgeb.2018.06.004, 10.1016/j.jgeb.2018.06.004]"
"   Tirry N, 2021, J GENET ENG BIOTECHN, V19, DOI 10.1186/s43141-021-00254-8"
"   T??th G, 2016, ENVIRON INT, V88, P299, DOI 10.1016/j.envint.2015.12.017"
"   Ullah I, 2022, ENVIRON POLLUT, V293, DOI 10.1016/j.envpol.2021.118508"
"   Wang H, 2024, TOXICS, V12, DOI 10.3390/toxics12050333"
"   Wang JL, 2016, SCI REP-UK, V6, DOI 10.1038/srep22028"
"   Wang ZW, 2024, J ENVIRON SCI, V137, P237, DOI 10.1016/j.jes.2023.02.020"
"   Xing YH, 2022, ENVIRON RES, V207, DOI 10.1016/j.envres.2021.112080"
"   Xu XH, 2018, CURR MICROBIOL, V75, P1231, DOI 10.1007/s00284-018-1515-z"
"   Yang QQ, 2018, SCI TOTAL ENVIRON, V642, P690, DOI 10.1016/j.scitotenv.2018.06.068"
"   Yang X, 2021, J HAZARD MATER, V406, DOI 10.1016/j.jhazmat.2020.124325"
"   Zainab N, 2024, PLANT PHYSIOL BIOCH, V211, DOI 10.1016/j.plaphy.2024.108652"
"   Zhang MM, 2024, J HAZARD MATER, V464, DOI 10.1016/j.jhazmat.2023.132948"
"   Zhang SJ, 2024, ENVIRON POLLUT, V347, DOI 10.1016/j.envpol.2024.123804"
"   Zhu QY, 2022, APPL SOIL ECOL, V177, DOI 10.1016/j.apsoil.2022.104501"
"   Zhu Yaxin, 2023, Chemosphere, V338, P139475, DOI [10.1016/j.chemosphere.2023.139475, 10.1016/j.chemosphere.2023.139475]"
"   Zuo YY, 2023, ECOTOX ENVIRON SAFE, V262, DOI 10.1016/j.ecoenv.2023.115190"
NR 144
TC 37
Z9 41
U1 46
U2 114
PU MDPI
PI BASEL
"PA ST ALBAN-ANLAGE 66, CH-4052 BASEL, SWITZERLAND"
EI 2076-2607
J9 MICROORGANISMS
JI Microorganisms
PD OCT
PY 2024
VL 12
IS 10
AR 1945
DI 10.3390/microorganisms12101945
PG 20
WC Microbiology
WE Science Citation Index Expanded (SCI-EXPANDED)
SC Microbiology
GA K3V5Z
UT WOS:001343189600001
PM 39458255
"OA Green Submitted, gold"
DA 2026-04-06
ER

PT J
"AU Oziegbe, O"
"   Oluduro, AO"
"   Oziegbe, EJ"

"   Ahuekwe, EF"
"   Olorunsola, SJ"
"AF Oziegbe, O."
"   Oluduro, A. O."
"   Oziegbe, E. J."
"   Ahuekwe, E. F."
"   Olorunsola, S. J."
TI Assessment of heavy metal bioremediation potential of bacterial isolates
   from landfill soils
SO SAUDI JOURNAL OF BIOLOGICAL SCIENCES
LA English
DT Article
DE Landfill soil; Heavy metal contamination; Cadmium; Lead; Indigenous
   bacteria; Soil reclamation
ID PSEUDOMONAS-AERUGINOSA; CONTAMINATED SOILS; RESISTANCE; REMOVAL; IMPACT;
   BIOAVAILABILITY; COPPER; OIL; BIODEGRADATION; IDENTIFICATION
"AB Indiscriminate disposal of wastes on landfills has led to increase in heavy metal contamination in landfill soils. However, the ability of the indigenous microorganisms to remediate the polluted environment can be of great influence in reclamation of such soils. The objectives of this study were to assess the bioremediation potential of the screened indigenous bacteria and evaluate the effects of carbon source and pH in the enhancement of the bioremediation process. Bacterial isolates from landfill sites were screened for their capability to utilize heavy metal (Cd and Pb). Nutrient Agar was supplemented with five different concentrations of each metal (25 to 600 mgL(-1)). Viable counts of the isolates were taken four times at two days interval. Pseudomonas aeruginosa, Klebsiella edwardsii and Enterobacter cloacae were selected based on their tolerance to heavy metal for remediation process. Peptone broth was also supplemented using different concentrations of heavy metals. The remediation process was assessed by monitoring the growth of biomass using UV spectrophotometer at 600 nm and the residual heavy metal was evaluated after 8 days of incubation using AAS. Pseudomonas aeruginosa exhibited the highest bioremediation potential among the bacterial isolates with 58.80 and 33.67 remediation percentage in 50 mg Cd L-1 and 300 mg Pb L-1 . However, higher remediation percentage (79.87 and 92.41) was observed by Klebsiella edwardsii through addition of carbon source (5 g/L) and varying the pH (6) of the media in the heavy metal contaminated medium. The results of this study indicate that the effectiveness of the indigenous bacteria in remediation process can be enhanced through the addition of carbon source and increase pH for effective reclamation of contaminated soil. (C) 2021 The Author(s). Published by Elsevier B.V. on behalf of King Saud University."
"C1 [Oziegbe, O.; Ahuekwe, E. F.; Olorunsola, S. J.] Covenant Univ, Dept Biol Sci, Ota, Nigeria."
"   [Oluduro, A. O.] Obafemi Awolowo Univ, Dept Microbiol, Ife, Nigeria."
"   [Oziegbe, E. J.] Univ Lagos, Dept Geosci, Akoka, Nigeria."
C3 Covenant University; Obafemi Awolowo University; University of Lagos
"RP Oziegbe, O (corresponding author), Covenant Univ, Dept Biol Sci, Ota, Nigeria."
EM olubukola.oziegbe@covenantuniversity.edu.ng
"RI Oluduro, Anthonia/IUO-7972-2023; AHUEKWE, EZE FRANK/GON-8521-2022"
"OI Oziegbe, Ehitua Julius/0000-0001-5772-0461; AHUEKWE, EZE"
   FRANK/0000-0003-1477-4050
FU Covenant University
FX The authors are immensely grateful to Covenant University for the
   financial support. The authors also appreciate the Central Science
   Laboratory of Obafemi Awolowo University for all the assistance rendered
   during the remediation experiment.
"CR Abioye OP, 2018, Brazilian Journal of Biological Sciences, V5, P25, DOI [10.21472/bjbs.050903, 10.21472/bjbs.050903, DOI 10.21472/BJBS.050903]"
"   Aka RJN, 2017, BIOREMEDIAT J, V21, P1, DOI 10.1080/10889868.2017.1282933"
"   Akob DM, 2007, FEMS MICROBIOL ECOL, V59, P95, DOI 10.1111/j.1574-6941.2006.00203.x"
"   Al-Dhabi NA, 2019, INT J ENV RES PUB HE, V16, DOI 10.3390/ijerph16234788"
"   Alexander PD, 2006, ENVIRON POLLUT, V144, P736, DOI 10.1016/j.envpol.2006.03.001"
"   Ameen FA, 2020, SCI REP-UK, V10, DOI 10.1038/s41598-019-57210-3"
"   [Anonymous], 2013, Int. J. Environ. Res."
"   Ashokkumar P., 2017, INT J MICROBIOL MYCO, V6, P32, DOI DOI 10.1016/S0304-386X(00)00195-X"
"   Bennisse R, 2004, WORLD J MICROB BIOT, V20, P759, DOI 10.1007/s11274-004-5812-2"
"   Brownfield M.E., 2006, 2207C US GEOL SURV"
"   Chen QG, 2021, ENVIRON TECHNOL INNO, V22, DOI 10.1016/j.eti.2021.101456"
"   Choudhary S, 2011, J HAZARD MATER, V186, P336, DOI 10.1016/j.jhazmat.2010.11.004"
"   Das S, 2016, APPL MICROBIOL BIOT, V100, P2967, DOI 10.1007/s00253-016-7364-4"
"   Fomina M, 2014, BIORESOURCE TECHNOL, V160, P3, DOI 10.1016/j.biortech.2013.12.102"
"   Franklin NM, 2000, AQUAT TOXICOL, V48, P275, DOI 10.1016/S0166-445X(99)00042-9"
"   Gbadamosi MR, 2018, J GEOCHEM EXPLOR, V190, P187, DOI 10.1016/j.gexplo.2018.03.006"
"   Giovanella P, 2017, ECOTOX ENVIRON SAFE, V140, P162, DOI 10.1016/j.ecoenv.2017.02.010"
"   GUIBAL E, 1994, REACT POLYM, V23, P147, DOI 10.1016/0923-1137(94)90015-9"
"   Hassan A, 2020, J SOIL SEDIMENT, V20, P66, DOI 10.1007/s11368-019-02394-4"
"   Hassen A, 1998, BIORESOURCE TECHNOL, V64, P7, DOI 10.1016/S0960-8524(97)00161-2"
"   Hoornweg Daniel, 2012, WHAT WASTE GLOBAL RE, V15"
"   Jain P. K., 2011, Research Journal of Environmental Toxicology, V5, P1, DOI 10.3923/rjet.2011.1.26"
"   Jia L, 2020, CHEM ENG PROCESS, V158, DOI 10.1016/j.cep.2020.108177"
"   Jin YY, 2018, APPL SCI-BASEL, V8, DOI 10.3390/app8081336"
"   JOSHI MM, 1995, FRESEN ENVIRON BULL, V4, P617"
"   Kaczorek E, 2012, POL J ENVIRON STUD, V21, P901"
"   Kamunda C, 2016, INT J ENV RES PUB HE, V13, DOI 10.3390/ijerph13070663"
"   Karak T, 2012, CRIT REV ENV SCI TEC, V42, P1509, DOI 10.1080/10643389.2011.569871"
"   Khan S, 2008, ENVIRON POLLUT, V152, P686, DOI 10.1016/j.envpol.2007.06.056"
"   Koshlaf E, 2019, GEODERMA, V338, P216, DOI 10.1016/j.geoderma.2018.12.001"
"   Ling W, 2007, AUST J SOIL RES, V45, P618, DOI 10.1071/SR07079"
"   Liu SH, 2017, BIORESOURCE TECHNOL, V224, P25, DOI 10.1016/j.biortech.2016.11.095"
"   Loh KC, 1997, BIODEGRADATION, V8, P329, DOI 10.1023/A:1008267607634"
"   Machado MD, 2008, BIORESOURCE TECHNOL, V99, P2107, DOI 10.1016/j.biortech.2007.05.047"
"   Malik A, 2004, ENVIRON INT, V30, P261, DOI 10.1016/j.envint.2003.08.001"

"   McLaughlin MJ, 2000, AUST J SOIL RES, V38, P1037, DOI 10.1071/SR99128"
"   Tarekegn MM, 2020, COGENT FOOD AGR, V6, DOI 10.1080/23311932.2020.1783174"
"   Mehrzad F, 2015, J PURE APPL MICROBIO, V9, P2185"
"   Misra GP, 2018, The Journal of Solid Waste Technology and Management, V44, P130, DOI
[10.5276/jswtm.2018.130, 10.5276/JSWTM.2018.130, DOI 10.5276/JSWTM.2018.130]"
"   Morris S, 2018, International Journal of Environmental Bioremediation & Biodegradation, V6, P26, DOI
[10.12691/ijebb-6-1-4, 10.12691/ijebb-6-1-4, DOI 10.12691/IJEBB-6-1-4]"
"   Mounaouer B, 2014, DESALIN WATER TREAT, V52, P7037, DOI 10.1080/19443994.2013.823565"
"   Mukherjee S, 2015, CRIT REV ENV SCI TEC, V45, P472, DOI 10.1080/10643389.2013.876524"
"   Nandish M.S., 2005, THESIS U AGR SCI BAN"
"   Nies DH, 2003, FEMS MICROBIOL REV, V27, P313, DOI 10.1016/S0168-6445(03)00048-2"
"   Njoku KL, 2020, SCI AFR, V10, DOI 10.1016/j.sciaf.2020.e00545"
"   Nwinyi Obinna C., 2019, IOP Conference Series: Earth and Environmental Science, V331, DOI [10.1088/1755-
1315/331/1/012058, 10.1088/1755-1315/331/1/012058]"
"   Nwinyi OC, 2014, BRAZ ARCH BIOL TECHN, V57, P789, DOI 10.1590/S1516-8913201402250"
"   Ojewumi ME, 2018, J ECOL ENG, V19, P226, DOI 10.12911/22998993/83564"
"   Okoro EE, 2020, SCI REP-UK, V10, DOI 10.1038/s41598-020-63107-3"
"   Olabode SO, 2016, International Journal of Geosciences, V07, P210, DOI [10.4236/ijg.2016.72017,
10.4236/ijg.2016.72017, DOI 10.4236/IJG.2016.72017]"
"   Oladipo O. G., 2014, Environmental and Experimental Biology, V12, P179"
"   Oladipo OG, 2018, J ENVIRON MANAGE, V212, P357, DOI 10.1016/j.jenvman.2018.01.038"
"   Olaniran AO, 2013, INT J MOL SCI, V14, P10197, DOI 10.3390/ijms140510197"
"   Olorunfemi F.B., 2011, Journal of Geography and Regional Planning, V4, P656"
"   Piddock LJV, 2006, NAT REV MICROBIOL, V4, P629, DOI 10.1038/nrmicro1464"
"   Pourfadakari S, 2019, J HAZARD MATER, V379, DOI 10.1016/j.jhazmat.2019.120839"
"   Priyadarshanee M, 2021, J ENVIRON CHEM ENG, V9, DOI 10.1016/j.jece.2020.104686"
"   Raimondo EE, 2020, J ENVIRON MANAGE, V276, DOI 10.1016/j.jenvman.2020.111309"
"   Randelovic D, 2015, BIOREMEDIAT J, V19, P231, DOI 10.1080/10889868.2014.995370"
"   Raskin Ilya, 1994, Current Opinion in Biotechnology, V5, P285, DOI 10.1016/0958-1669(94)90030-2"
"   Raulinaitis M., 2012, EKOLOGILA, V58, P33, DOI DOI 10.6001/EKOLOGIJA.V58I1.2348"
"   Rizvi A, 2017, CHEMOSPHERE, V185, P942, DOI 10.1016/j.chemosphere.2017.07.088"
"   Rubinos DA, 2018, CRIT REV ENV SCI TEC, V48, P376, DOI 10.1080/10643389.2018.1461495"
"   SALT DE, 1995, BIO-TECHNOL, V13, P468, DOI 10.1038/nbt0595-468"
"   Samlafo VB, 2017, American Journal of Chemistry, V7, P145"
"   Sanchez-Clemente R, 2020, GENES-BASEL, V11, DOI 10.3390/genes11111292"
"   Scott J, 2005, CRIT REV ENV SCI TEC, V35, P239, DOI 10.1080/10643380590917969"
"   Shuaib M, 2021, MICROB PATHOGENESIS, V150, DOI 10.1016/j.micpath.2020.104713"
"   Singh R, 2013, BIORESOURCE TECHNOL, V138, P222, DOI 10.1016/j.biortech.2013.03.100"
"   Tang X, 2018, SCI TOTAL ENVIRON, V636, P1355, DOI 10.1016/j.scitotenv.2018.04.229"
"   Teng Y, 2010, BIODEGRADATION, V21, P167, DOI 10.1007/s10532-009-9291-x"
"   Varjani S, 2020, SCI TOTAL ENVIRON, V737, DOI 10.1016/j.scitotenv.2020.139766"
"   Wang JL, 2009, BIOTECHNOL ADV, V27, P195, DOI 10.1016/j.biotechadv.2008.11.002"
"   Wuana R.A., 2011, ISRN Ecology, DOI [10.5402/2011/402647, DOI 10.5402/2011/402647]"
"   Xia SP, 2019, CRIT REV ENV SCI TEC, V49, P1027, DOI 10.1080/10643389.2018.1564526"
"   Yin K, 2016, WATER RES, V103, P383, DOI 10.1016/j.watres.2016.07.053"
"   Zahoor A, 2009, J ENVIRON SCI-CHINA, V21, P814, DOI 10.1016/S1001-0742(08)62346-3"
"   Zhang MK, 2010, COMMUN SOIL SCI PLAN, V41, P820, DOI 10.1080/00103621003592341"
"   Zhang X, 2020, BIOCHEM ENG J, V157, DOI 10.1016/j.bej.2020.107551"
NR 79
TC 43
Z9 53
U1 2
U2 49
PU ELSEVIER
PI AMSTERDAM
"PA RADARWEG 29, 1043 NX AMSTERDAM, NETHERLANDS"
SN 1319-562X
EI 2213-7106
J9 SAUDI J BIOL SCI
JI Saudi J. Biol. Sci.
PD JUL
PY 2021
VL 28
IS 7
BP 3948
EP 3956
DI 10.1016/j.sjbs.2021.03.072
EA JUN 2021
PG 9
WC Biology
WE Science Citation Index Expanded (SCI-EXPANDED)
SC Life Sciences & Biomedicine - Other Topics
GA TC3PB
UT WOS:000668551400012
PM 34220251
"OA Green Submitted, hybrid"
DA 2026-04-06
ER

PT J
"AU Liu, YR"
"   Huang, LK"
"   Wen, ZY"
"   Fu, YY"

"   Liu, QZ"
"   Xu, SA"
"   Li, ZS"
"   Liu, CJ"
"   Yu, C"
"   Feng, Y"
"AF Liu, Yaru"
"   Huang, Lukuan"
"   Wen, Zheyu"
"   Fu, Yingyi"
"   Liu, Qizhen"
"   Xu, Shunan"
"   Li, Zhesi"
"   Liu, Chanjuan"
"   Yu, Chao"
"   Feng, Ying"
TI Effects of intercropping on safe agricultural production and
   phytoremediation of heavy metal-contaminated soils
SO SCIENCE OF THE TOTAL ENVIRONMENT
LA English
DT Article
DE Interplanting; Cd; Main plant; Bioremediation; meta-analysis
ID MICROBIAL COMMUNITY STRUCTURE; CHROMIUM SPECIATION; ORGANIC-ACIDS;
   FIELD-SCALE; CADMIUM; PLANTS; REMEDIATION; INTENSIFICATION;
   DETOXIFICATION; MECHANISMS
"AB Intercropping with hyperaccumulators is believed to be an important and efficient way to achieve simultaneous safe agricultural production and phytoremediation of polluted soils. However, some studies have suggested that this tech-nique might facilitate the uptake of heavy metals by crops. To investigate the effects of intercropping on the heavy metal contents of plants and soil, data from 135 global studies were collected and analyzed by meta-analysis. The re-sults showed that intercropping could significantly reduce the contents of heavy metals in the main plants and soils. Plant species was the main factor that affected plant and soil metal contents in the intercropping system, and the heavy metal content could be significantly reduced when members of the Poaceae and Crassulaceae were used as main plants or when legumes were used as intercropped plants. Among all the intercropped plants, the best one for removing heavy metals from the soil was a Crassulaceae hyperaccumulator. These results not only highlight the main factors affecting intercropping systems but also provide reliable reference information for the practice of safe ag-ricultural production coupled with phytoremediation of heavy metal-contaminated farmland."
"C1 [Liu, Yaru; Huang, Lukuan; Wen, Zheyu; Fu, Yingyi; Liu, Qizhen; Xu, Shunan; Li, Zhesi; Liu, Chanjuan; Feng, Ying] Zhejiang Univ, Coll Environm & Resource Sci, MOE Key Lab Environm Remediat & Ecol Hlth, Hangzhou 310058, Peoples R China."
"   [Yu, Chao] Livestock Ind Dev Ctr Shengzhou, Shengzhou 312400, Peoples R China."
"   [Feng, Ying] Zhejiang Univ, Coll Environm & Resource Sci, Hangzhou 310058, Peoples R China."
C3 Zhejiang University; Zhejiang University
"RP Feng, Y (corresponding author), Zhejiang Univ, Coll Environm & Resource Sci, Hangzhou 310058, Peoples R China."
EM yfeng@zju.edu.cn
"RI FENG, YING/KWU-9364-2024; Liu, Chanjuan/ADS-1118-2022"
FU National Natural Science Foundation of China [42277002]; Zhejiang
   Provincial Natural Science Foundation of China [LZ22D010002]
FX This work was supported by the National Natural Science Foundation of
   China (No. 42277002) and Zhejiang Provincial Natural Science Foundation
   of China (No. LZ22D010002) .
"CR Agnello AC, 2014, CRIT REV ENV SCI TEC, V44, P2531, DOI 10.1080/10643389.2013.829764"
"   Alvarez A, 2017, CHEMOSPHERE, V166, P41, DOI 10.1016/j.chemosphere.2016.09.070"
"   Arivalagan P, 2014, ECOL ENG, V71, P728, DOI 10.1016/j.ecoleng.2014.08.005"
"   Bian FY, 2021, J HAZARD MATER, V416, DOI 10.1016/j.jhazmat.2021.125898"
"   Brooker RW, 2015, NEW PHYTOL, V206, P107, DOI 10.1111/nph.13132"
"   Brumbarova T, 2015, TRENDS PLANT SCI, V20, P124, DOI 10.1016/j.tplants.2014.11.004"
"   Cui EP, 2021, SCI TOTAL ENVIRON, V784, DOI 10.1016/j.scitotenv.2021.147093"
"   Cui T, 2018, J CHEM-NY, V2018, DOI 10.1155/2018/7803408"
"   Duchene O, 2017, AGR ECOSYST ENVIRON, V240, P148, DOI 10.1016/j.agee.2017.02.019"
"   Duval S, 2000, BIOMETRICS, V56, P455, DOI 10.1111/j.0006-341X.2000.00455.x"
"   Elzinga EJ, 2010, J HAZARD MATER, V183, P145, DOI 10.1016/j.jhazmat.2010.06.130"
"   Gavrilescu M, 2022, CURR OPIN BIOTECH, V74, DOI 10.1016/j.copbio.2021.10.024"
"   Ghori NH, 2019, INT J ENVIRON SCI TE, V16, P1807, DOI 10.1007/s13762-019-02215-8"
"   Gong YY, 2018, WATER RES, V147, P440, DOI 10.1016/j.watres.2018.10.024"
"   Hedges L.V., 1985, Statistical Methods for Meta-Analysis, P75, DOI 10.1016/B978-0-08-057065-5.50010-5"
"   Hou SZ, 2019, J SOIL SEDIMENT, V19, P3512, DOI 10.1007/s11368-019-02309-3"
"   Hu RP, 2019, ACTA AGR SCAND B-S P, V69, P304, DOI 10.1080/09064710.2018.1564786"
"   Hussein Y, 2019, BIOSCI RES, V16, P2678"
"   Kim S, 2010, J ENVIRON SCI HEAL A, V45, P389, DOI 10.1080/10934520903467832"
"   Krishnamurti GSR, 1997, J ENVIRON QUAL, V26, P271, DOI 10.2134/jeq1997.00472425002600010038x"
"   Kumar A, 2020, INT J ENV RES PUB HE, V17, DOI 10.3390/ijerph17072179"
"   Kumar V, 2021, CHEMOSPHERE, V262, DOI 10.1016/j.chemosphere.2020.127810"
"   Lai HL, 2022, AGRONOMY-BASEL, V12, DOI 10.3390/agronomy12081900"
"   Landrot G, 2012, CHEMOSPHERE, V88, P1196, DOI 10.1016/j.chemosphere.2012.03.069"
"   Li CJ, 2020, NAT PLANTS, V6, P653, DOI 10.1038/s41477-020-0680-9"
"   Li KF, 2011, J HAZARD MATER, V189, P531, DOI 10.1016/j.jhazmat.2011.02.075"
"   Li NY, 2009, WATER AIR SOIL POLL, V199, P45, DOI 10.1007/s11270-008-9858-x"
"   Li ZR, 2019, INT J PHYTOREMEDIAT, V21, P4, DOI 10.1080/15226514.2018.1523867"
"   Light R.J., 1984, Summing up: The science of reviewing research"
"   Lin H, 2022, CHEMOSPHERE, V305, DOI 10.1016/j.chemosphere.2022.135457"
"   Ma LY, 2020, ENVIRON POLLUT, V264, DOI 10.1016/j.envpol.2020.114796"
"   Mahmoud R, 2022, AGRON SUSTAIN DEV, V42, DOI 10.1007/s13593-022-00754-y"

"   Maitra S, 2021, AGRONOMY-BASEL, V11, DOI 10.3390/agronomy11020343"
"   Martin-Guay MO, 2018, SCI TOTAL ENVIRON, V615, P767, DOI 10.1016/j.scitotenv.2017.10.024"
"   Mei XY, 2022, ENVIRON SCI POLLUT R, V29, P23026, DOI 10.1007/s11356-021-17353-1"
"   Mendoza-C??zatl DG, 2011, CURR OPIN PLANT BIOL, V14, P554, DOI 10.1016/j.pbi.2011.07.004"
"   Neugschwandtner RW, 2008, GEODERMA, V144, P446, DOI 10.1016/j.geoderma.2007.11.021"
"   Page MJ, 2021, PLOS MED, V18, DOI 10.1371/journal.pmed.1003583"
"   [????????? Peng Yunxiao], 2019, [?????????????????, Jiangsu Journal of Agricultural Sciences], V35, P1368"
"   Rai PK, 2019, ENVIRON INT, V125, P365, DOI 10.1016/j.envint.2019.01.067"
"   Rajendran S, 2022, CHEMOSPHERE, V287, DOI 10.1016/j.chemosphere.2021.132369"
"   Rehman AU, 2021, J MOL LIQ, V321, DOI 10.1016/j.molliq.2020.114455"
"   ROSENTHAL R, 1979, PSYCHOL BULL, V86, P638, DOI 10.1037/0033-2909.86.3.638"
"   Roth U, 2006, J EXP BOT, V57, P4003, DOI 10.1093/jxb/erl170"
"   Rusinamhodzi L, 2012, FIELD CROP RES, V136, P12, DOI 10.1016/j.fcr.2012.07.014"
"   Shah V, 2020, ENVIRON TECHNOL INNO, V18, DOI 10.1016/j.eti.2020.100774"
"   Shahid M, 2012, J HAZARD MATER, V219, P1, DOI 10.1016/j.jhazmat.2012.01.060"
"   Shahid M, 2017, CHEMOSPHERE, V178, P513, DOI 10.1016/j.chemosphere.2017.03.074"
"   Shahid M, 2017, REV ENVIRON CONTAM T, V241, P73, DOI 10.1007/398_2016_8"
"   Singh S, 2016, FRONT PLANT SCI, V6, DOI 10.3389/fpls.2015.01143"
"   Stangoulis JCR, 2022, PLANT SOIL, V474, P57, DOI 10.1007/s11104-022-05330-7"
"   Stanton C, 2022, MOL PLANT, V15, P65, DOI 10.1016/j.molp.2021.12.008"
"   Viechtbauer W, 2010, RES SYNTH METHODS, V1, P112, DOI 10.1002/jrsm.11"
"   Wallace BC, 2017, METHODS ECOL EVOL, V8, P941, DOI 10.1111/2041-210X.12708"
"   [????????? WEI Ze-bin], 2010, [????????????????????????, Journal of Agro-Environment Science], V29, P267"
"   Xiao ZH, 2020, ECOTOX ENVIRON SAFE, V205, DOI 10.1016/j.ecoenv.2020.111162"
"   Yaashikaa PR, 2022, ENVIRON POLLUT, V301, DOI 10.1016/j.envpol.2022.119035"
"   Yang Jia-Jie, 2020, Journal of Agro-Environment Science, V39, P2122, DOI 10.11654/jaes.2020-0568"
"   Zeng P, 2019, GEODERMA, V345, P5, DOI 10.1016/j.geoderma.2019.03.013"
"   Zhan FD, 2016, RSC ADV, V6, P33240, DOI 10.1039/c5ra26601g"
"   Zhang F, 2014, J PLANT INTERACT, V9, P249, DOI 10.1080/17429145.2013.819944"
"   Zou JC, 2021, CHEMOSPHERE, V276, DOI 10.1016/j.chemosphere.2021.130223"
"   Zuo Y, 2009, AGRON SUSTAIN DEV, V29, P63, DOI 10.1051/agro:2008055"
NR 63
TC 39
Z9 48
U1 19
U2 254
PU ELSEVIER
PI AMSTERDAM
"PA RADARWEG 29, 1043 NX AMSTERDAM, NETHERLANDS"
SN 0048-9697
EI 1879-1026
J9 SCI TOTAL ENVIRON
JI Sci. Total Environ.
PD JUN 1
PY 2023
VL 875
AR 162700
DI 10.1016/j.scitotenv.2023.162700
EA MAR 2023
PG 10
WC Environmental Sciences
WE Science Citation Index Expanded (SCI-EXPANDED)
SC Environmental Sciences & Ecology
GA 0A0RE
UT WOS:000951536600001
PM 36906036
DA 2026-04-06
ER

PT J
"AU Wei, YX"
"   Ma, JJ"
"   Liu, KK"
"   Zhang, S"
"   Wang, JQ"
"AF Wei, Yuxin"
"   Ma, Jingjing"
"   Liu, Kuankuan"
"   Zhang, Shuai"
"   Wang, Junqi"
"TI Biochar-Based Remediation of Heavy Metal-Contaminated Soils: Mechanisms,"
"   Synergies, and Sustainable Prospects"
SO NANOMATERIALS
LA English
DT Review
DE biochar; heavy metal pollution; soil remediation; adsorption mechanism;
   sustainable development
ID LOADED BIOCHAR; IMMOBILIZATION; PB; CD; AVAILABILITY; MOBILITY;
   SORPTION; BIOAVAILABILITY; STABILIZATION; PHYTOTOXICITY
"AB This study systematically explores the mechanisms and application potential of biochar in remediating heavy metal-contaminated soils. Particular emphasis is placed on the role of raw materials and pyrolysis conditions in modulating key physicochemical properties of biochar, including its aromatic structure, porosity, cation exchange capacity, and ash content, which collectively enhance heavy metal immobilization.

The direct remediation mechanisms are categorized into six pathways: physical adsorption, electrostatic interactions, precipitation, ion exchange, organic functional group complexation, and redox reactions, with particular emphasis on the reduction in toxic Cr6+ and the oxidation of mobile As3+. In addition to direct interactions, biochar indirectly facilitates remediation by enhancing soil carbon sequestration, improving soil physicochemical characteristics, stimulating microbial activity, and promoting plant growth, thereby generating synergistic effects. The study evaluates combined remediation strategies integrating biochar with phytoremediation and microbial remediation, highlighting their enhanced efficiency. Moreover, practical challenges related to the long-term stability, ecological risks, and economic feasibility in field applications are critically analyzed. By synthesizing recent theoretical advancements and practical findings, this research provides a scientific foundation for optimizing biochar-based soil remediation technologies."
"C1 [Wang, Junqi] Xi An Jiao Tong Univ, Technol Innovat Ctr Land Engn & Human Settlements, Shaanxi Land Engn Construct Grp Co Ltd, Xian 710049, Peoples R China."
"   Xi An Jiao Tong Univ, Sch Human Settlements & Civil Engn, Xian 710049, Peoples R China."
C3 Xi'an Jiaotong University; Xi'an Jiaotong University
"RP Wang, JQ (corresponding author), Xi An Jiao Tong Univ, Technol Innovat Ctr Land Engn & Human Settlements, Shaanxi Land Engn Construct Grp Co Ltd, Xian 710049, Peoples R China."
EM weiyuxin5924@stu.xjtu.edu.cn; mjj20010218@stu.xjtu.edu.cn;
   lk20010422@stu.xjtu.edu.cn; zs0209@stu.xjtu.edu.cn; wjq@xjtu.edu.cn
"RI ; Ma, Jingjing/OUI-5527-2025"
"OI Wang, Junqi/0000-0002-0955-3934; "
FU Technology Innovation Center for Land Engineering and Human Settlements
   [201912131-D6]; Technology Innovation Center for Land Engineering
FX The authors are grateful for the financial support provided by the
"   Technology Innovation Center for Land Engineering and Human Settlements,"
"   Shaanxi Land Engineering Construction Group Co., Ltd. and Xi'an Jiaotong"
   University (201912131-D6).
"CR Abdelhafez AA, 2014, CHEMOSPHERE, V117, P66, DOI 10.1016/j.chemosphere.2014.05.086"
"   Ahmad J, 2021, ENVIRON TECHNOL INNO, V21, DOI 10.1016/j.eti.2020.101310"
"   Ahmad M, 2017, J SOIL SEDIMENT, V17, P717, DOI 10.1007/s11368-015-1339-4"
"   Ahmad M, 2016, J ENVIRON MANAGE, V166, P131, DOI 10.1016/j.jenvman.2015.10.006"
"   Ahmad M, 2018, CHEMOSPHERE, V194, P327, DOI 10.1016/j.chemosphere.2017.11.156"
"   Ambika S, 2022, CHEM ENG J, V439, DOI 10.1016/j.cej.2022.135716"
"   Arabi Z, 2021, ENVIRON POLLUT, V286, DOI 10.1016/j.envpol.2021.117199"
"   Awa SH, 2020, WATER AIR SOIL POLL, V231, DOI 10.1007/s11270-020-4426-0"
"   Awang NA, 2023, J CHEM TECHNOL BIOT, V98, P22, DOI 10.1002/jctb.7182"
"   Cai T, 2021, J CLEAN PROD, V281, DOI 10.1016/j.jclepro.2020.125390"
"   Chandra S, 2023, SUSTAINABILITY-BASEL, V15, DOI 10.3390/su15053973"
"   Chen HM, 2019, ENVIRON INT, V127, P395, DOI 10.1016/j.envint.2019.03.068"
"   Chen HH, 2020, WATER AIR SOIL POLL, V231, DOI 10.1007/s11270-020-4425-1"
"   Chen JQ, 2018, ECOTOX ENVIRON SAFE, V163, P172, DOI 10.1016/j.ecoenv.2018.07.071"
"   Cui XQ, 2016, SCI TOTAL ENVIRON, V562, P517, DOI 10.1016/j.scitotenv.2016.03.248"
"   Diao YZ, 2022, ENVIRON SCI POLLUT R, V29, P71871, DOI 10.1007/s11356-022-20750-9"
"   Ding H, 2023, FRONT ENV SCI-SWITZ, V11, DOI 10.3389/fenvs.2023.1239842"
"   Ding KL, 2021, J HAZARD MATER, V420, DOI 10.1016/j.jhazmat.2021.126551"
"   Duan CQ, 2023, SUSTAINABILITY-BASEL, V15, DOI 10.3390/su152416753"
"   El-Naggar A, 2021, J HAZARD MATER, V419, DOI 10.1016/j.jhazmat.2021.126421"
"   Fan JJ, 2020, J HAZARD MATER, V388, DOI 10.1016/j.jhazmat.2020.122037"
"   Fan J, 2020, J HAZARD MATER, V398, DOI 10.1016/j.jhazmat.2020.122901"
"   Fang J, 2015, CHEM ENG J, V267, P253, DOI 10.1016/j.cej.2015.01.026"
"   Fang SE, 2016, CHEMOSPHERE, V149, P263, DOI 10.1016/j.chemosphere.2016.01.060"
"   Farhangi-Abriz S, 2023, CHEMOSPHERE, V313, DOI 10.1016/j.chemosphere.2022.137365"
"   Feng DD, 2021, CHEM ENG J, V410, DOI 10.1016/j.cej.2020.127707"
"   Freitas AM, 2020, FRONT SUSTAIN FOOD S, V4, DOI 10.3389/fsufs.2020.510982"
"   Fu HC, 2021, CHEMOSPHERE, V281, DOI 10.1016/j.chemosphere.2021.130990"
"   Gao YN, 2022, J ENVIRON MANAGE, V313, DOI 10.1016/j.jenvman.2022.114973"
"   Gholizadeh M, 2021, J ENVIRON CHEM ENG, V9, DOI 10.1016/j.jece.2021.105830"
"   Gong HF, 2020, ECOTOX ENVIRON SAFE, V205, DOI 10.1016/j.ecoenv.2020.111132"
"   Gong HB, 2022, J HAZARD MATER, V432, DOI 10.1016/j.jhazmat.2022.128668"
"   Tran HN, 2019, J HAZARD MATER, V373, P258, DOI 10.1016/j.jhazmat.2019.03.018"
"   Hamid Y, 2020, ENVIRON POLLUT, V257, DOI 10.1016/j.envpol.2019.113609"
"   He LZ, 2019, ENVIRON POLLUT, V252, P846, DOI 10.1016/j.envpol.2019.05.151"
"   Huang DL, 2018, J HAZARD MATER, V348, P109, DOI 10.1016/j.jhazmat.2018.01.024"
"   Igalavithana AD, 2019, ENVIRON INT, V127, P190, DOI 10.1016/j.envint.2019.03.031"
"   Islam MS, 2021, CHEMOSPHERE, V283, DOI 10.1016/j.chemosphere.2021.131102"
"   Islam MS, 2021, SCI TOTAL ENVIRON, V785, DOI 10.1016/j.scitotenv.2021.147163"
"   Ji YN, 2024, ENVIRON POLLUT, V357, DOI 10.1016/j.envpol.2024.124445"
"   Jiang JH, 2023, ENVIRON RES, V236, DOI 10.1016/j.envres.2023.116731"
"   Karimi A, 2019, J SOIL SCI PLANT NUT, V19, P851, DOI 10.1007/s42729-019-00084-1"
"   Katebe FM, 2023, AGRONOMY-BASEL, V13, DOI 10.3390/agronomy13020357"
"   Kumar R, 2022, SCI TOTAL ENVIRON, V836, DOI 10.1016/j.scitotenv.2022.155619"
"   Leng LJ, 2020, BIORESOURCE TECHNOL, V298, DOI 10.1016/j.biortech.2019.122286"
"   Li AY, 2022, CHEM ENG J, V439, DOI 10.1016/j.cej.2022.135785"
"   Li G, 2018, J HAZARD MATER, V348, P100, DOI 10.1016/j.jhazmat.2018.01.031"
"   Li MH, 2020, WATER AIR SOIL POLL, V231, DOI 10.1007/s11270-020-04875-6"
"   Li XN, 2024, ENVIRON INT, V185, DOI 10.1016/j.envint.2024.108498"
"   Lin H, 2022, CHEMOSPHERE, V305, DOI 10.1016/j.chemosphere.2022.135457"
"   Liu BL, 2017, SCI TOTAL ENVIRON, V609, P822, DOI 10.1016/j.scitotenv.2017.07.215"
"   Liu HK, 2018, SCI TOTAL ENVIRON, V645, P702, DOI 10.1016/j.scitotenv.2018.07.115"
"   Liu JH, 2019, CERAM INT, V45, P1379, DOI 10.1016/j.ceramint.2018.08.311"
"   Liu J, 2020, ENVIRON RES, V188, DOI 10.1016/j.envres.2020.109886"
"   Liu JJ, 2020, CATALYSTS, V10, DOI 10.3390/catal10070742"
"   Liu SC, 2021, ENVIRON TECHNOL INNO, V22, DOI 10.1016/j.eti.2021.101388"
"   Liu SM, 2020, ENVIRON SCI POLLUT R, V27, P16069, DOI 10.1007/s11356-020-08282-6"
"   Liu YL, 2020, CHEMOSPHERE, V247, DOI 10.1016/j.chemosphere.2020.125850"

"   Lobzenko I, 2022, AGRICULTURE-BASEL, V12, DOI 10.3390/agriculture12101689"
"   Lu HP, 2018, SCI TOTAL ENVIRON, V622, P892, DOI 10.1016/j.scitotenv.2017.12.056"
"   Lu YL, 2015, ENVIRON INT, V77, P5, DOI 10.1016/j.envint.2014.12.010"
"   Lyu P, 2024, J CLEAN PROD, V434, DOI 10.1016/j.jclepro.2023.140136"
"   Mahar A, 2016, ECOTOX ENVIRON SAFE, V126, P111, DOI 10.1016/j.ecoenv.2015.12.023"
"   Manikandan SK, 2023, MOLECULES, V28, DOI 10.3390/molecules28020719"
"   Oni BA, 2019, ANN AGR SCI-CAIRO, V64, P222, DOI 10.1016/j.aoas.2019.12.006"
"   Oustriere N, 2016, SCI TOTAL ENVIRON, V566, P816, DOI 10.1016/j.scitotenv.2016.05.091"
"   Park JH, 2011, PLANT SOIL, V348, P439, DOI 10.1007/s11104-011-0948-y"
"   Rechberger MV, 2019, CHEMOSPHERE, V216, P463, DOI 10.1016/j.chemosphere.2018.10.094"
"   Rees F, 2014, EUR J SOIL SCI, V65, P149, DOI 10.1111/ejss.12107"
"   Rodriguez A, 2019, J HEALTH POLLUT, V9, DOI 10.5696/2156-9614-9.23.190907"
"   Sharma JK, 2023, FRONT PLANT SCI, V14, DOI 10.3389/fpls.2023.1076876"
"   Shen ZT, 2019, ENVIRON INT, V122, P357, DOI 10.1016/j.envint.2018.11.045"
"   Singh H, 2022, BIOCHAR, V4, DOI 10.1007/s42773-022-00138-1"
"   Song PP, 2022, J CLEAN PROD, V378, DOI 10.1016/j.jclepro.2022.134510"
"   Song PP, 2022, SCI TOTAL ENVIRON, V838, DOI 10.1016/j.scitotenv.2022.156417"
"   Song ZG, 2014, CHEM ENG J, V242, P36, DOI 10.1016/j.cej.2013.12.061"
"   Soria RI, 2020, HELIYON, V6, DOI 10.1016/j.heliyon.2020.e05388"
"   Sui X, 2021, SUSTAINABILITY-BASEL, V13, DOI 10.3390/su13169267"
"   Sun T, 2021, ENVIRON POLLUT, V271, DOI 10.1016/j.envpol.2020.116308"
"   Tan XF, 2015, CHEMOSPHERE, V125, P70, DOI 10.1016/j.chemosphere.2014.12.058"
"   Tan ZX, 2017, ENVIRON SCI POLLUT R, V24, P24844, DOI 10.1007/s11356-017-0109-9"
"   Tomczyk A, 2020, REV ENVIRON SCI BIO, V19, P191, DOI 10.1007/s11157-020-09523-3"
"   Trakal L, 2014, BIORESOURCE TECHNOL, V171, P442, DOI 10.1016/j.biortech.2014.08.108"
"   Ullah MH, 2024, HELIYON, V10, DOI 10.1016/j.heliyon.2024.e36869"
"   Veronica N, 2020, J GEOCHEM EXPLOR, V209, DOI 10.1016/j.gexplo.2019.106430"
"   Wan XM, 2020, ENVIRON POLLUT, V261, DOI 10.1016/j.envpol.2020.114157"
"   Wang K, 2020, CHEMOSPHERE, V241, DOI 10.1016/j.chemosphere.2019.125044"
"   Wang L, 2021, J HAZARD MATER, V414, DOI 10.1016/j.jhazmat.2021.125494"
"   Wang YF, 2024, J HAZARD MATER, V477, DOI 10.1016/j.jhazmat.2024.135356"
"   Wu C, 2018, ENVIRON SCI POLLUT R, V25, P20792, DOI 10.1007/s11356-018-2268-8"
"   Wu JZ, 2020, J HAZARD MATER, V387, DOI 10.1016/j.jhazmat.2019.122010"
"   Xiang MT, 2021, ENVIRON POLLUT, V278, DOI 10.1016/j.envpol.2021.116911"
"   Xie LH, 2023, SCI TOTAL ENVIRON, V905, DOI 10.1016/j.scitotenv.2023.167399"
"   Xiong XN, 2025, BIOCHAR, V7, DOI 10.1007/s42773-025-00438-2"
"   Xu DM, 2021, J CLEAN PROD, V321, DOI 10.1016/j.jclepro.2021.128730"
"   Xu XY, 2019, ENVIRON POLLUT, V244, P423, DOI 10.1016/j.envpol.2018.10.068"
"   Yang F, 2021, ENV POLLUT BIOAVAIL, V33, P55, DOI 10.1080/26395940.2021.1916407"
"   Yang JW, 2022, NANOMATERIALS-BASEL, V12, DOI 10.3390/nano12193541"
"   Yang TT, 2021, ENVIRON POLLUT, V291, DOI 10.1016/j.envpol.2021.118129"
"   Yang X, 2019, APPL SCI-BASEL, V9, DOI 10.3390/app9071365"
"   Yu Y, 2020, BIORESOURCE TECHNOL, V297, DOI 10.1016/j.biortech.2019.122466"
"   Yu ZH, 2017, CHEMOSPHERE, V168, P341, DOI 10.1016/j.chemosphere.2016.10.069"
"   Zhang GX, 2016, ENVIRON POLLUT, V218, P513, DOI 10.1016/j.envpol.2016.07.031"
"   Zhang PZ, 2021, BIORESOURCE TECHNOL, V331, DOI 10.1016/j.biortech.2021.125013"
"   Zhang W, 2022, BIOCHAR, V4, DOI 10.1007/s42773-022-00181-y"
"   Zhang Y, 2023, J CLEAN PROD, V382, DOI 10.1016/j.jclepro.2022.135380"
"   Zhang ZY, 2021, BIORESOURCES, V16, P8509, DOI 10.15376/biores.16.4.Zhang"
"   Zhong DL, 2019, ENVIRON SCI TECHNOL, V53, P9034, DOI 10.1021/acs.est.9b00756"
"   Zhou QW, 2018, SCI TOTAL ENVIRON, V615, P115, DOI 10.1016/j.scitotenv.2017.09.220"
"   Zhou YM, 2013, CHEM ENG J, V231, P512, DOI 10.1016/j.cej.2013.07.036"
"   Zhu YF, 2020, INT J ENV RES PUB HE, V17, DOI 10.3390/ijerph17207654"
NR 111
TC 6
Z9 7
U1 57
U2 57
PU MDPI
PI BASEL
"PA MDPI AG, Grosspeteranlage 5, CH-4052 BASEL, SWITZERLAND"
EI 2079-4991
J9 NANOMATERIALS-BASEL
JI Nanomaterials
PD SEP 29
PY 2025
VL 15
IS 19
AR 1487
DI 10.3390/nano15191487
PG 25
"WC Chemistry, Multidisciplinary; Nanoscience & Nanotechnology; Materials"
"   Science, Multidisciplinary; Physics, Applied"
WE Science Citation Index Expanded (SCI-EXPANDED)
SC Chemistry; Science & Technology - Other Topics; Materials Science;
   Physics
GA 8OV6K
UT WOS:001593786300001
PM 41090833
"OA Green Submitted, gold"
DA 2026-04-06
ER

PT J

"AU Liu, P"
"   Cheng, Y"
"   Shao, GH"
"AF Liu, Peng"
"   Cheng, Yu"
"   Shao, Guanghui"
TI Study on the remediation of Pb-contaminated soil under the combined
   action of carbonic anhydrase (CA) and bacterial-induced mineralization
   (BIM)
SO JOURNAL OF ENVIRONMENTAL MANAGEMENT
LA English
DT Article
DE Lead (Pb) stabilization; Bacterial-induced mineralization (BIM);
   Carbonic anhydrase (CA); Bioremediation
ID METAL REMOVAL; HEAVY-METALS
"AB Bacterial-induced mineralization (BIM) is an environmentally friendly and cost-effective technology for remediating heavy metal-contaminated sites. Bacteria immobilize heavy metals and render them less hazardous primarily through biomineralization, thereby preventing their further spread or leaching into the environment. In this study, carbonic anhydrase (CA) was employed to catalyze the CO2 hydration reaction, which significantly increased the production rate of CO32- and HCO3- during the BIM process (by a factor of 108) and accelerated carbonate precipitation, thereby enhancing the remediation effect of BIM on contaminated soil. Through experiments, we investigated the effects of CA dosage, Pb2+ concentrations in contaminated soil, cementation solution concentration, and curing time on the leaching behavior and chemical speciation distribution of Pb. By combining these experiments with microscopic analyses (e.g., XRD and SEM), the influencing mechanism of CA on the BIM remediation process of Pb-contaminated soil was elucidated. The results demonstrated that CA substantially improved BIM efficiency in remediating Pb-contaminated soil, with the leaching concentration of heavy metal ions decreasing initially and stabilizing as the CA dosage increased. Compared to BIM-treated soil without CA, the incorporation of a 3 % mass percentage of a CA-producing bacterial agent reduced the leaching concentration of Pb2+ by 49.0 %. Furthermore, CA promoted the transformation of exchangeable Pb2+ into more stable forms, effectively reducing the bioavailability and mobility of heavy metal ions. These findings hold significant implications for optimizing BIM and its application in remediating heavy metal-contaminated soil."
"C1 [Liu, Peng; Shao, Guanghui] Nanjing Forestry Univ, Sch Civil Engn, 159 Longpan Rd, Nanjing 210037, Peoples R China."
"   [Cheng, Yu] Shandong Univ Sci & Technol, Sch Transportat, 579 Qianwangang Rd, Qingdao 266590, Peoples R China."
C3 Nanjing Forestry University; Shandong University of Science & Technology
"RP Liu, P (corresponding author), Nanjing Forestry Univ, Sch Civil Engn, 159 Longpan Rd, Nanjing 210037, Peoples R China."
EM liupengreal@sina.com; ycheng1979@163.com; gh_shao@njfu.edu.cn
FU National Natural Science Foundation of China [51809139]
FX <BOLD>Funding</BOLD> This work is funded by the National Natural Science
   Foundation of China (No.51809139) .
"CR Achal V, 2012, J MICROBIOL BIOTECHN, V22, P244, DOI 10.4014/jmb.1108.08033"
"   Ali H, 2013, CHEMOSPHERE, V91, P869, DOI 10.1016/j.chemosphere.2013.01.075"
"   Cai L.X., 2019, J. Bioeng., P1, DOI [10.13345/j.cjb.180108, DOI 10.13345/J.CJB.180108]"
"   Chen M.J., 2020, Nonferrous Metal Engineering, V12, P128"
"   Chen Yue-ru, 2024, Huanjing Kexue, V45, P2939, DOI 10.13227/j.hjkx.202305138"
"   Dai H.R., 2022, Master's Thesis, DOI [10.27242/d.cnki.gnjlu.2022.000853, DOI 10.27242/D.CNKI.GNJLU.2022.000853]"
"   Davoodbasha M, 2024, APPL BIOCHEM BIOTECH, V196, P7064, DOI 10.1007/s12010-024-04908-4"
"   Dejong JT, 2013, GEOTECHNIQUE, V63, P287, DOI 10.1680/geot.SIP13.P.017"
"   Dermont G, 2008, J HAZARD MATER, V152, P1, DOI 10.1016/j.jhazmat.2007.10.043"
"   Favre N, 2009, J MOL CATAL B-ENZYM, V60, P163, DOI 10.1016/j.molcatb.2009.04.018"
"   Fu Jiajia, 2021, People's Changjiang, V1, P172, DOI [10.16232/j.cnki.1001-4179.2021.01.027, DOI 10.16232/J.CNKI.1001-4179.2021.01.027]"
"   Han FX, 2001, SOIL SCI, V166, P18, DOI 10.1097/00010694-200101000-00005"
"   Khan FI, 2004, J ENVIRON MANAGE, V71, P95, DOI 10.1016/j.jenvman.2004.02.003"
"   Lanphear B.P., 2019, Lowlevel environmental lead exposure and Children"
"   Li M, 2013, INT BIODETER BIODEGR, V76, P81, DOI 10.1016/j.ibiod.2012.06.016"
"   Liu J.X., 2020, Low Temperature Construction Technology, P17, DOI [10.13905/j.cnki.dwjz.2020.05.004, DOI 10.13905/J.CNKI.DWJZ.2020.05.004]"
"   Ma Rui, 2014, Res. Environ. Sci., P1205, DOI [10.13198/j.issn.1001-6929.2014.10.17, DOI 10.13198/J.ISSN.1001-6929.2014.10.17]"
"   Qian Chun-Xiang, 2011, Environ. Sci. Technol., P33"
"   Seifan M, 2018, APPL MICROBIOL BIOT, V102, P2167, DOI 10.1007/s00253-018-8782-2"
"   Shao G., 2017, Journal of Nanjing Forestry University (Natural Science Edition), P129, DOI [10.3969/j.issn.1000-2006.2017.02.019,02, DOI 10.3969/J.ISSN.1000-2006.2017.02.019,02]"
"   Sharma M, 2022, ENVIRON SCI POLLUT R, V29, P51827, DOI 10.1007/s11356-022-19551-x"
"   TESSIER A, 1979, ANAL CHEM, V51, P844, DOI 10.1021/ac50043a017"
"   [????????? Wang Xiaohui], 2015, [????????????????????????. ???????????????, Journal of Shaanxi Normal University. Natural Science Edition], V43, P80"
"   Xiao H., 2022, Journal of Three Gorges University (Natural Science Edition), V6, P66, DOI [10.13393/j.cnki.issn.1672- 948x.2022.06.009, DOI 10.13393/J.CNKI.ISSN.1672-948X.2022.06.009]"
"   Xue ZF, 2023, ENVIRON POLLUT, V322, DOI 10.1016/j.envpol.2023.121218"
"   Yang Q.Q., 2018, Industrial Building, P38, DOI [10.13204/j.gyjz201807007, DOI 10.13204/J.GYJZ201807007]"
"   Zha FS, 2021, CRYSTALS, V11, DOI 10.3390/cryst11111303"
"   Zhang Y., 2024, J. GeoTech. Eng., V46, P2352, DOI [10.11779/CJGE20230749, DOI 10.11779/CJGE20230749]"
"   Zhang Yinfeng, 2021, J. China Inst. Water Resour. Hydropower Res., P246, DOI [10.13244/j.cnki.jiwhr.20200250, DOI 10.13244/J.CNKI.JIWHR.20200250]"
NR 29
TC 1
Z9 1
U1 6

U2 22
PU ACADEMIC PRESS LTD- ELSEVIER SCIENCE LTD
PI LONDON
"PA 24-28 OVAL RD, LONDON NW1 7DX, the United Kingdom"
SN 0301-4797
EI 1095-8630
J9 J ENVIRON MANAGE
JI J. Environ. Manage.
PD JUN
PY 2025
VL 384
AR 125517
DI 10.1016/j.jenvman.2025.125517
EA MAY 2025
PG 10
WC Environmental Sciences
WE Science Citation Index Expanded (SCI-EXPANDED)
SC Environmental Sciences & Ecology
GA 2NU6I
UT WOS:001487059500001
PM 40315649
DA 2026-04-06
ER

PT J
"AU Zakaria, Z"
"   Fadzil, FNM"
"   Mohamad, MAN"
"   Hamid, AAA"
"   Chowdhury, AJK"
"   Harumain, ZAS"
"AF Zakaria, Zahiruddeen"
"   Fadzil, Fariha Nafeesa Md"
"   Mohamad, Mohd Azrul Naim"
"   Hamid, Azzmer Azzar Abdul"
"   Chowdhury, Ahmed Jalal Khan"
"   Harumain, Zakuan Azizi Shamsul"
TI Metagenomic analysis of bacterial communities in heavy metal
"   leachate-contaminated soils at Jalan Lipis Sanitary Landfill, Pahang,"
   Malaysia
SO DESALINATION AND WATER TREATMENT
LA English
DT Article
DE Metagenomics; Leachate-contaminated soil; Heavy metals; Bacteria;
   Bioremediation
ID DIVERSITY; WASTE; MANGANESE; POLLUTION; IRON
"AB A variety of bacterial communities can be found in heavy metal leachate-contaminated soil. These bacteria might have molecular defenses that allow them to endure a high heavy metal concentration. However, there aren't enough datasets on bacterial populations to be exploited for bioremediation, especially the biotreatment of leachate contaminated with heavy metals. This research examined the diversity of the bacterial population in heavy metal leachate-contaminated soil from the Jalan Lipis Sanitary Landfill in Pahang, Malaysia. The soil samples were taken from three (3) different locations. pH analysis showed that the heavy metal leachate-contaminated soil possesses alkaline pH. ICPMS analysis showed high concentration of nickel (112.96 mg/kg) followed by manganase (89.83 mg/kg), arsenic (43.84 mg/kg), and lead (3.62 mg/kg). The three most prevalent bacteria in the heavy metal leachate-contaminated soil from the site were Pseudomonas C (Proteobacteria), Flavobacterium (Bacteroidota), and Proteiniclasticum (Firmicutes), according to metagenomic sequencing of the 16 s rRNA gene. The alpha and beta bacterial diversity analysis indicate that each location with different heavy metal concentration differs significantly on its bacterial diversity providing a valuable information to be applied in heavy metal bioremediation exclusively from landfill leachate."
"C1 [Zakaria, Zahiruddeen; Fadzil, Fariha Nafeesa Md; Mohamad, Mohd Azrul Naim; Hamid, Azzmer Azzar Abdul; Harumain, Zakuan Azizi Shamsul] Int Islamic Univ Malaysia IIUM, Kulliyyah Sci, Dept Biotechnol, Kuantan 25200, Pahang, Malaysia."
"   [Mohamad, Mohd Azrul Naim; Hamid, Azzmer Azzar Abdul; Harumain, Zakuan Azizi Shamsul] Int Islamic Univ Malaysia, Kulliyyah Sci, Res Unit Bioinformat & Computat Biol RUBIC, Kuantan 25200, Pahang, Malaysia."
"   [Mohamad, Mohd Azrul Naim; Hamid, Azzmer Azzar Abdul; Harumain, Zakuan Azizi Shamsul] Int Islamic Univ Malaysia, Environm Biol & Biotechnol Res Unit EBBRU, Kulliyyah Sci, Kuantan 25200, Pahang, Malaysia."
"   [Chowdhury, Ahmed Jalal Khan] Sultan Sharif Ali Islamic Univ UNISSA, Fac Agr, Km 33 Jalan Tutong,Kg Sinaut, Tutong, Brunei."
C3 International Islamic University Malaysia; International Islamic
   University Malaysia; International Islamic University Malaysia; Sultan
   Sharif Ali Islamic University
"RP Harumain, ZAS (corresponding author), Int Islamic Univ Malaysia IIUM, Kulliyyah Sci, Dept Biotechnol, Kuantan 25200, Pahang, Malaysia.; Chowdhury, AJK (corresponding author), Sultan Sharif Ali Islamic Univ UNISSA, Fac Agr, Km 33 Jalan Tutong,Kg Sinaut, Tutong, Brunei."
EM ahmed.chowdhury@unissa.edu.bn; zak@iium.edu.my
"RI Naim, Mohd Azrul/D-2955-2015; harumain, zakuan/R-9457-2019"
"OI Zakaria, Zahiruddeen/0009-0006-4306-8218"
FU Ministry of Higher Education [FRGS/1/2019/WAB08/UIAM/03/1]
FX The authors wish to thank the Fundamental Research Grant Scheme
   (FRGS/1/2019/WAB08/UIAM/03/1) from the Ministry of Higher Education for
   financial support for this work.
"CR Alain N, 2018, SOIL CONTAMINATION L"
"   Ao L, 2014, INT J SYST EVOL MICR, V64, P3369, DOI 10.1099/ijs.0.063248-0"
"   Barasarathi J, 2022, ENVIRON SCI POLLUT R, V29, P22069, DOI 10.1007/s11356-021-17389-3"

"   Behjati S, 2013, ARCH DIS CHILDHOOD-E, V98, P236, DOI 10.1136/archdischild-2013-304340"
"   Bokulich NA, 2018, MICROBIOME, V6, DOI 10.1186/s40168-018-0470-z"
"   Bolyen E, 2019, NAT BIOTECHNOL, V37, P852, DOI 10.1038/s41587-019-0209-9"
"   Brzoska RM, 2022, Bacteria, V1, P33, DOI [10.3390/bacteria1010004, 10.3390/bacteria1010004, DOI 10.3390/BACTERIA1010004]"
"   Callahan BJ, 2016, NAT METHODS, V13, P581, DOI [10.1038/NMETH.3869, 10.1038/nmeth.3869]"
"   Calle ML, 2019, Genom Inf, V17, P1"
"   Chen SF, 2018, BIOINFORMATICS, V34, P884, DOI 10.1093/bioinformatics/bty560"
"   Chong J, 2020, NAT PROTOC, V15, P799, DOI 10.1038/s41596-019-0264-1"
"   Chukwuma OB, 2021, LIFE-BASEL, V11, DOI 10.3390/life11060493"
"   Cordi A, 2015, ENVIRON SCI POLLUT R, V22, P13753, DOI 10.1007/s11356-014-3840-5"
"   Department of Environment, 2010, Environ Requir: A Guide Invest"
"   Drewniak L, 2016, FRONT MICROBIOL, V7, DOI 10.3389/fmicb.2016.01252"
"   Eitinger T, 2000, ARCH MICROBIOL, V173, P1, DOI 10.1007/s002030050001"
"   Fauziah SH, 2017, International Journal of Bioscience Biochemistry and Bioinformatics, V7, P230, DOI [10.17706/ijbbb.2017.7.4.230-237, DOI 10.17706/IJBBB.2017.7.4.230-237]"
"   Fern??ndez S, 2019, FOLIA MICROBIOL, V64, P19, DOI 10.1007/s12223-018-0627-y"
"   Gao TP, 2021, WATER AIR SOIL POLL, V232, DOI 10.1007/s11270-021-05338-2"
"   Gu ZP, 2022, CHEMOSPHERE, V287, DOI 10.1016/j.chemosphere.2021.132155"
"   Hao XL, 2021, COMPUT STRUCT BIOTEC, V19, P94, DOI 10.1016/j.csbj.2020.12.006"
"   Haque MA, 2016, J CLEAN PROD, V139, P99, DOI 10.1016/j.jclepro.2016.08.028"
"   Hemmat-Jou MH, 2018, ECOTOXICOLOGY, V27, P1281, DOI 10.1007/s10646-018-1981-x"
"   How FNF, 2020, POLLUTION, V6, P801, DOI 10.22059/poll.2020.301198.783"
"   Hu LF, 2021, J HAZARD MATER, V403, DOI 10.1016/j.jhazmat.2020.123687"
"   Ismail S., 2013, Journal of Toxicology and Environmental Health Sciences, V5, P86, DOI [10.5897/JTEHS12.20, DOI 10.5897/JTEHS12.20, 10.5897/jtehs12.058, DOI 10.5897/JTEHS12.058]"
"   Jacob JM, 2018, J ENVIRON MANAGE, V217, P56, DOI 10.1016/j.jenvman.2018.03.077"
"   Jayanthi B, 2017, INT BIODETER BIODEGR, V119, P467, DOI 10.1016/j.ibiod.2016.10.053"
"   Jiang YX, 2021, INT BIODETER BIODEGR, V164, DOI 10.1016/j.ibiod.2021.105292"
"   Kamaruddin MA, 2017, ENVIRON SCI POLLUT R, V24, P26988, DOI 10.1007/s11356-017-0303-9"
"   Kiddee P, 2013, WASTE MANAGE, V33, P1237, DOI 10.1016/j.wasman.2013.01.006"
"   Klindworth A, 2013, NUCLEIC ACIDS RES, V41, DOI 10.1093/nar/gks808"
"   Kunin V, 2008, MICROBIOL MOL BIOL R, V72, P557, DOI 10.1128/MMBR.00009-08"
"   Li YD, 2009, WASTE MANAGE, V29, P2147, DOI 10.1016/j.wasman.2009.02.005"
"   Liang ZT, 2021, ENVIRON SCI POLLUT R, V28, P56418, DOI 10.1007/s11356-021-14337-z"
"   Lindamulla L, 2022, CURR POLLUT REP, V8, P273, DOI 10.1007/s40726-022-00222-x"
"   Liu YX, 2021, PROTEIN CELL, V12, P315, DOI 10.1007/s13238-020-00724-8"
"   Lopez S, 2017, SOIL BIOL BIOCHEM, V114, P121, DOI 10.1016/j.soilbio.2017.07.010"
"   Martin M., 2011, EMBnet J, V17, P3, DOI [10.14806/ej.17.1.200, DOI 10.14806/EJ.17.1.200]"
"   Mat Ali QA, 2016, Assessment of glossostigma elatinoides and Bacopa caroliniana to remediate landfill leachate through landscape ecology model system"
"   Mat Salleh N.F. D., 2013, The Malaysian Journal of Analytical Sciences, V17, P24"
"   Mckee KP, 2016, J ENVIRON SCI HEAL A, V51, P523, DOI 10.1080/10934529.2016.1141618"
"   Mohd Latiff NH, 2019, Study on Landfill Spatial Contaminant Distribution and Potential Terrestrial Fern Species as Phytoremediation Agent"
"   Mojiri A, 2021, WATER REUSE, V11, P66, DOI 10.2166/wrd.2020.079"
"   Mwaikono KS, 2016, BMC MICROBIOL, V16, DOI 10.1186/s12866-016-0758-8"
"   Oberacker P, 2019, PLOS BIOL, V17, DOI 10.1371/journal.pbio.3000107"
"   Parks DH, 2020, NAT BIOTECHNOL, V38, P1079, DOI 10.1038/s41587-020-0501-8"
"   Puthusseri RM, 2021, J ENVIRON MANAGE, V298, DOI 10.1016/j.jenvman.2021.113492"
"   Rehan M., 2016, Environ Chem Recent Pollut Control Approaches, V11, P13"
"   Rosli RA, 2021, REMEDIATION, V31, P49, DOI 10.1002/rem.21688"
"   Rupert R, 2020, DATA BRIEF, V33, DOI 10.1016/j.dib.2020.106351"
"   Saadah N, 2014, Int J Innov, Manag Technol, V5, P61, DOI [10.7763/ijimt.2014.v5.487, DOI 10.7763/IJIMT.2014.V5.487]"
"   Sathendra ER, 2018, ENERGY ENV SUSTAIN, P249, DOI 10.1007/978-981-10-7413-4_13"
"   Sekhohola-Dlamini L, 2020, BIODEGRADATION, V31, P1, DOI 10.1007/s10532-019-09890-x"
"   Song LY, 2015, APPL MICROBIOL BIOT, V99, P7745, DOI 10.1007/s00253-015-6633-y"
"   Sutherland KM, 2021, GEOBIOLOGY, V19, P450, DOI 10.1111/gbi.12448"
"   Tajudin MTF, 2017, Microbial Degradation of Polychlorinated Biphenyls and Dibenzofuran by Burkholderia xenovurans LB400 Isolated from Landfill Leacahete"
"   Wen P, 2021, CHEMOSPHERE, V262, DOI 10.1016/j.chemosphere.2020.127822"
"   Xu MJ, 2023, INT J ENV RES PUB HE, V20, DOI 10.3390/ijerph20010604"
"   Yang L, 2014, BIORESOURCE TECHNOL, V156, P384, DOI 10.1016/j.biortech.2014.01.067"
"   Yang ZN, 2022, ENVIRON SCI ECOTECH, V10, DOI 10.1016/j.ese.2022.100169"
"   Yin HQ, 2015, SCI REP-UK, V5, DOI 10.1038/srep14266"
"   Yin K, 2019, CHEM ENG J, V360, P1553, DOI [10.1016/j.cej.2018.10.226, 10.1016/j.jhazmat.2024.134410]"
"   Yin Kun Yin Kun, 2019, Chemical Engineering Journal, V360, P1553, DOI 10.1016/j.cej.2018.10.226"
"   Yin Y, 2023, J HAZARD MATER, V442, DOI 10.1016/j.jhazmat.2022.130063"
"   Yusoff I, 2013, SCIENCEASIA, V39, P392, DOI 10.2306/scienceasia1513-1874.2013.39.392"
"   Zainun MY, 2018, SCI TOTAL ENVIRON, V616, P269, DOI 10.1016/j.scitotenv.2017.10.266"
"   Zeng TT, 2022, INT J ENV RES PUB HE, V19, DOI 10.3390/ijerph19116529"
"   Zhang GY, 2014, GEOMICROBIOL J, V31, P562, DOI 10.1080/01490451.2013.854428"
"   Zhao XQ, 2019, ECOTOX ENVIRON SAFE, V170, P218, DOI 10.1016/j.ecoenv.2018.11.136"
"   Zhou Q, 2014, SCI REP-UK, V4, DOI 10.1038/srep06957"
NR 71
TC 4
Z9 5
U1 8
U2 26
PU ELSEVIER SCIENCE INC
PI NEW YORK
"PA STE 800, 230 PARK AVE, NEW YORK, NY 10169 USA"
SN 1944-3994

EI 1944-3986
J9 DESALIN WATER TREAT
JI Desalin. Water Treat.
PD JUL
PY 2024
VL 319
AR 100512
DI 10.1016/j.dwt.2024.100512
EA JUN 2024
PG 13
"WC Engineering, Chemical; Water Resources"
WE Science Citation Index Expanded (SCI-EXPANDED)
SC Engineering; Water Resources
GA XE2C7
UT WOS:001259932500001
OA gold
DA 2026-04-06
ER

PT J
"AU Xu, YL"
"   Seshadri, B"
"   Bolan, N"
"   Sarkar, B"
"   Ok, YS"
"   Zhang, W"
"   Rumpel, C"
"   Sparks, D"
"   Farrell, M"
"   Hall, T"
"   Dong, ZM"
"AF Xu, Yilu"
"   Seshadri, Balaji"
"   Bolan, Nanthi"
"   Sarkar, Binoy"
"   Ok, Yong Sik"
"   Zhang, Wei"
"   Rumpel, Cornelia"
"   Sparks, Donald"
"   Farrell, Mark"
"   Hall, Tony"
"   Dong, Zhaomin"
TI Microbial functional diversity and carbon use feedback in soils as
   affected by heavy metals
SO ENVIRONMENT INTERNATIONAL
LA English
DT Article
DE Heavy metals; Soil organic carbon; Microbial carbon decomposition;
   Microbial activity; Microbial community composition; PLFAs
ID COMMUNITY STRUCTURE; BACTERIAL COMMUNITY; ENZYME-ACTIVITIES; BIOMASS
   CARBON; CADMIUM; PB; WASTE; CD; BIOAVAILABILITY; LEAD
"AB Soil microorganisms are an important indicator of soil fertility and health. However, our state of knowledge about soil microbial activities, community compositions and carbon use patterns under metal contaminations is still poor. This study aimed to evaluate the influences of heavy metals (Cd and Pb) on soil microorganisms by investigating the microbial community composition and carbon use preferences. Metal pollution was approached both singly and jointly with low (25 and 2500 mg kg(-1)) and high (50 and 5000 mg kg(-1)) concentrations of Cd and Pb, respectively, in an artificially contaminated soil. In a laboratory incubation experiment, bio-available and potentially bio-available metal concentrations, selected soil properties (pH, electrical conductivity, total organic carbon and total nitrogen), and microbial parameters (microbial activity as basal respiration, microbial biomass carbon (MBC) and microbial functional groups) were determined at two sampling occasions (7 and 49 days). Metal contamination had no effect on the selected soil properties, while it significantly inhibited both microbial activity and MBC formation. Contaminated soils had higher microbial quotient (qCO(2)), suggesting there was higher energy demand with less microbially immobilized carbon as MBC. Notably, the efficiency of microbial carbon use was repressed as the metal concentration increased, yet no difference was observed between metal types (p > 0.05). Based on the microbial phospholipid fatty acids (PLFA) analysis, total PLFAs decreased significantly under metal stress at the end of incubation. Heavy metals had a greater negative influence on the fungal population than bacteria with respective 5-35 and 8-32% fall in abundances. The contaminant-driven (metal concentrations and types) variation of soil PLFA biomarkers demonstrated that the heavy metals led to the alteration of soil microbial community compositions and their activities, which consequently had an adverse impact on soil microbial carbon immobilization."
"C1 [Xu, Yilu; Seshadri, Balaji; Bolan, Nanthi] Univ Newcastle, Global Ctr Environm Remediat, Callaghan, NSW 2308, Australia."
"   [Xu, Yilu] Swansea Univ, Coll Engn, Bay Campus, Swansea SA1 8EN, W Glam, the United Kingdom."
"   [Sarkar, Binoy] Univ Sheffield, Dept Anim & Plant Sci, Sheffield S10 2TN, S Yorkshire, the United Kingdom."
"   [Sarkar, Binoy; Zhang, Wei] Univ South Australia, Future Ind Inst, Mawson Lakes, SA 5095, Australia."
"   [Ok, Yong Sik] Korea Univ, O Jeong Ecoresilience Inst, Korea Biochar Res Ctr, Seoul, South Korea."
"   [Ok, Yong Sik] Korea Univ, Div Environm Sci & Ecol Engn, Seoul, South Korea."
"   [Zhang, Wei] Univ South Australia, Sch Nat & Built Environm, Mawson Lakes, SA 5095, Australia."
"   [Rumpel, Cornelia] CNRS, Inst Ecol & Environm Paris, IEES, CNRS INRA UPMC UPEC IRD, F-78850 Thiverval Grignon, France."
"   [Sparks, Donald] Univ Delaware, Delaware Environm Inst, Dept Plant & Soil Sci, Newark, DE 19711 USA."
"   [Farrell, Mark] CSIRO Agr & Food, Locked Bag 2, Glen Osmond, SA 5064, Australia."

"   [Hall, Tony] Univ Adelaide, Sprigg Geobiol Ctr, Adelaide, SA 5005, Australia."
"   [Hall, Tony] Univ Adelaide, Dept Earth Sci, Adelaide, SA 5005, Australia."
"   [Dong, Zhaomin] Beihang Univ, Sch Space & Environm, Beijing 100191, Peoples R China."
C3 University of Newcastle; Swansea University; University of Sheffield;
Adelaide University; University of South Australia; Korea University;
Korea University; Adelaide University; University of South Australia;
Sorbonne Universite; Universite Paris-Est-Creteil-Val-de-Marne (UPEC);
INRAE; Centre National de la Recherche Scientifique (CNRS); CNRS -
Institute of Ecology & Environment (INEE); Universite Paris Cite;
University of Delaware; Commonwealth Scientific & Industrial Research
Organisation (CSIRO); CSIRO Agriculture & Food; Adelaide University;
University of Adelaide; Adelaide University; University of Adelaide;
Beihang University
"RP Dong, ZM (corresponding author), Beihang Univ, Sch Space & Environm, Beijing 100191, Peoples R China."
EM dongzm@buaa.edu.cn
"RI Zhang, Wei/A-3823-2012; Sarkar, Binoy/A-5187-2019; Bolan,"
"   Nanthi/E-8535-2011; Ok, Yong Sik/R-6739-2016; Rumpel,"
"   Cornelia/A-2001-2015; Farrell, Mark/C-3676-2011; dong,"
zhaomin/AAG-4062-2019
"OI Zhang, Wei/0000-0003-3129-2918; Sarkar, Binoy/0000-0002-4196-1225;"
"   Bolan, Nanthi/0000-0003-2056-1692; Ok, Yong Sik/0000-0003-3401-0912;"
"   Rumpel, Cornelia/0000-0003-2131-9451; Farrell, Mark/0000-0003-4562-2738;"
"   Seshai, Balaji/0000-0003-1855-045X; "
"FU University of Newcastle; Department of Education and Training,"
Government of Australia; Australian Research Council [DP140100323]
FX Yilu Xu is thankful to the University of Newcastle and Department of
"   Education and Training, Government of Australia, for awarding her PhD"
Scholarship. This research was partly supported by an Australian
Research Council Discovery-Project (DP140100323).
"CR Abhijit Gupta Abhijit Gupta, 2016, Journal of Microbial and Biochemical Technology, V8, P364, DOI 10.4172/1948-5948.1000310"
"   Adriano DC, 2004, GEODERMA, V122, P121, DOI 10.1016/j.geoderma.2004.01.003"
"   Ahmad M, 2016, J ENVIRON MANAGE, V166, P131, DOI 10.1016/j.jenvman.2015.10.006"
"   ANDERSON TH, 1989, SOIL BIOL BIOCHEM, V21, P471, DOI 10.1016/0038-0717(89)90117-X"
"   ANDERSON TH, 1993, SOIL BIOL BIOCHEM, V25, P393, DOI 10.1016/0038-0717(93)90140-7"
"   Bailey VL, 2002, SOIL BIOL BIOCHEM, V34, P997, DOI 10.1016/S0038-0717(02)00033-0"
"   Blagodatskaya E, 2013, SOIL BIOL BIOCHEM, V67, P192, DOI 10.1016/j.soilbio.2013.08.024"
"   BLIGH EG, 1959, CAN J BIOCHEM PHYS, V37, P911"
"   Bolan N, 2014, J HAZARD MATER, V266, P141, DOI 10.1016/j.jhazmat.2013.12.018"
"   Bossio DA, 1998, MICROBIAL ECOL, V36, P1, DOI 10.1007/s002489900087"
"   Caliz J, 2012, CHEMOSPHERE, V89, P494, DOI 10.1016/j.chemosphere.2012.05.002"
"   Carney KM, 2005, ECOSYSTEMS, V8, P928, DOI 10.1007/s10021-005-0047-0"
"   Chen JH, 2014, FEMS MICROBIOL ECOL, V87, P164, DOI 10.1111/1574-6941.12212"
"   Chenery SR, 2012, SCI TOTAL ENVIRON, V433, P547, DOI 10.1016/j.scitotenv.2012.03.004"
"   Chodak M, 2013, APPL SOIL ECOL, V64, P7, DOI 10.1016/j.apsoil.2012.11.004"
"   Choppala G, 2014, CRIT REV PLANT SCI, V33, P374, DOI 10.1080/07352689.2014.903747"
"   Creamer CA, 2015, SOIL BIOL BIOCHEM, V80, P175, DOI 10.1016/j.soilbio.2014.10.008"
"   Dai J, 2004, APPL SOIL ECOL, V25, P99, DOI 10.1016/j.apsoil.2003.09.003"
"   Dilly O, 2005, SOIL BIOL, V3, P123"
"   Doran J. W., 1996, Methods for assessing soil quality., P25"
"   El-Naggar AH, 2015, CHEMOSPHERE, V138, P67, DOI 10.1016/j.chemosphere.2015.05.052"
"   Epelde L, 2008, SCI TOTAL ENVIRON, V401, P21, DOI 10.1016/j.scitotenv.2008.03.024"
"   Farrell M, 2014, SOIL BIOL BIOCHEM, V77, P261, DOI 10.1016/j.soilbio.2014.07.003"
"   Farrell M, 2010, ENVIRON POLLUT, V158, P55, DOI 10.1016/j.envpol.2009.08.027"
"   Fern??ndez-Calvi??o D, 2010, APPL SOIL ECOL, V46, P276, DOI 10.1016/j.apsoil.2010.08.001"
"   FLIESSBACH A, 1994, SOIL BIOL BIOCHEM, V26, P1201, DOI 10.1016/0038-0717(94)90144-9"
"   Frey SD, 2001, SOIL BIOL BIOCHEM, V33, P1759, DOI 10.1016/S0038-0717(01)00101-8"
"   FROSTEGARD A, 1993, APPL ENVIRON MICROB, V59, P3605"
"   Frosteg??rd ??, 2011, SOIL BIOL BIOCHEM, V43, P1621, DOI 10.1016/j.soilbio.2010.11.021"
"   Gadd GM, 2004, GEODERMA, V122, P109, DOI 10.1016/j.geoderma.2004.01.002"
"   Garc??a-Orenes F, 2013, PLOS ONE, V8, DOI 10.1371/journal.pone.0080522"
"   Gentry T.J., 2015, ENV MICROBIOLOGY, P415"
"   Giller KE, 2009, SOIL BIOL BIOCHEM, V41, P2031, DOI 10.1016/j.soilbio.2009.04.026"
"   Gupta Pratima, 2017, Biotechnol Rep (Amst), V13, P58, DOI [10.1016/j.btre.2016.12.006, 10.1016/j.btre.2016.12.006]"
"   Hinojosa MB, 2004, SOIL BIOL BIOCHEM, V36, P1559, DOI 10.1016/j.soilbio.2004.07.003"
"   Houben D, 2013, CHEMOSPHERE, V92, P1450, DOI 10.1016/j.chemosphere.2013.03.055"
"   Hui N, 2012, BOREAL ENVIRON RES, V17, P46"
"   Igalavithana AD, 2017, ENVIRON GEOCHEM HLTH, V39, P1531, DOI 10.1007/s10653-017-9998-2"
"   Igalavithana AD, 2017, CHEMOSPHERE, V174, P593, DOI 10.1016/j.chemosphere.2017.01.148"
"   Isbell R., 2016, The Australian soil classification"
"   Jiang JP, 2010, EUR J SOIL BIOL, V46, P18, DOI 10.1016/j.ejsobi.2009.10.001"
"   Kallenbach CM, 2016, NAT COMMUN, V7, DOI 10.1038/ncomms13630"
"   Khan S, 2007, J ENVIRON SCI, V19, P834, DOI 10.1016/S1001-0742(07)60139-9"
"   Khan S, 2010, ENVIRON SCI POLLUT R, V17, P288, DOI 10.1007/s11356-009-0134-4"
"   KILLHAM K, 1985, ENVIRON POLLUT A, V38, P283, DOI 10.1016/0143-1471(85)90133-3"
"   Kim RY, 2015, ENVIRON GEOCHEM HLTH, V37, P1041, DOI 10.1007/s10653-015-9695-y"
"   Knight BP, 1997, APPL ENVIRON MICROB, V63, P39, DOI 10.1128/AEM.63.1.39-43.1997"
"   Lamb DT, 2016, ECOTOXICOLOGY, V25, P1563, DOI 10.1007/s10646-016-1712-0"
"   Lehmann J, 2015, NATURE, V528, P60, DOI 10.1038/nature16069"
"   LEITA L, 1993, J PLANT NUTR, V16, P2001, DOI 10.1080/01904169309364670"
"   Lenart A, 2013, B ENVIRON CONTAM TOX, V90, P85, DOI 10.1007/s00128-012-0869-3"
"   Liu Yongzhuo, 2012, PLoS One, V7, pe38858, DOI 10.1371/journal.pone.0038858"
"   Lu AX, 2005, GEODERMA, V125, P225, DOI 10.1016/j.geoderma.2004.08.002"

"   Lu M, 2013, CHEMOSPHERE, V91, P491, DOI 10.1016/j.chemosphere.2012.12.009"
"   Margesin R, 2011, CHEMOSPHERE, V82, P1583, DOI 10.1016/j.chemosphere.2010.11.056"
"   Markowicz A, 2016, ARCH ENVIRON PROT, V42, P3, DOI 10.1515/aep-2016-0041"
"   MILLER WP, 1987, COMMUN SOIL SCI PLAN, V18, P1, DOI 10.1080/00103628709367799"
"   Mine N. A. C, 2014, TOPSOIL MANAGEMENT P"
"   Ming H, 2016, GEODERMA, V268, P60, DOI 10.1016/j.geoderma.2016.01.021"
"   Mudhoo A, 2013, INT J ENVIRON SCI TE, V10, P1472"
"   Neethu CS, 2015, CAN J MICROBIOL, V61, P429, DOI 10.1139/cjm-2014-0803"
"   Noble AD, 1999, AGROFOREST SYST, V46, P147, DOI 10.1023/A:1006299615488"
"   Noble AD, 1996, PLANT SOIL, V179, P293, DOI 10.1007/BF00009340"
"   Ok YS, 2011, ENVIRON GEOCHEM HLTH, V33, P83, DOI 10.1007/s10653-010-9329-3"
"   Ok YS, 2011, ENVIRON GEOCHEM HLTH, V33, P31, DOI 10.1007/s10653-010-9362-2"
"   Pocknee S, 1997, SOIL SCI SOC AM J, V61, P86, DOI 10.2136/sssaj1997.03615995006100010014x"
"   Rinnan R, 2009, APPL ENVIRON MICROB, V75, P3611, DOI 10.1128/AEM.02865-08"
"   Ross D S., 1995, Recommended Methods for Determining Soil Cation Exchange Capacity, P75"
"   Salimi M., 2012, INT J ENVIRON HEALTH, V1, P57, DOI [DOI 10.4103/2277-9183.94395, 10.4103/2277-9183.94395]"
"   Schimel J, 2007, ECOLOGY, V88, P1386, DOI 10.1890/06-0219"
"   Seshadri B, 2017, CHEMOSPHERE, V184, P197, DOI 10.1016/j.chemosphere.2017.05.172"
"   Seshadri B, 2015, J SOIL SCI PLANT NUT, V15, P524"
"   Shi WL, 2017, ANN AGR ENV MED, V24, P722, DOI 10.26444/aaem/80920"
"   Six J, 2006, SOIL SCI SOC AM J, V70, P555, DOI 10.2136/sssaj2004.0347"
"   Sparks D. L., 1996, Methods of soil analysis. Part 3 - chemical methods., P1275"
"   Tchounwou Paul B, 2012, Exp Suppl, V101, P133, DOI 10.1007/978-3-7643-8340-4_6"
"   Tipayno SC, 2018, ECOL EVOL, V8, P6157, DOI 10.1002/ece3.4170"
"   Tripathy S, 2014, ECOL ENG, V70, P25, DOI 10.1016/j.ecoleng.2014.04.013"
"   VANCE ED, 1987, SOIL BIOL BIOCHEM, V19, P703, DOI 10.1016/0038-0717(87)90052-6"
"   Wang QK, 2013, AGR FOREST METEOROL, V178, P152, DOI 10.1016/j.agrformet.2013.04.021"
"   Wang YP, 2007, ECOTOX ENVIRON SAFE, V67, P75, DOI 10.1016/j.ecoenv.2006.03.007"
"   Wertz S, 2007, ENVIRON MICROBIOL, V9, P2211, DOI 10.1111/j.1462-2920.2007.01335.x"
"   Wuana R.A., 2011, ISRN Ecology, DOI [10.5402/2011/402647, DOI 10.5402/2011/402647]"
"   Xu YL, 2018, SCI TOTAL ENVIRON, V621, P148, DOI 10.1016/j.scitotenv.2017.11.214"
"   Yan F, 1996, SOIL BIOL BIOCHEM, V28, P617, DOI 10.1016/0038-0717(95)00180-8"
"   Yang YR, 2015, SOIL BIOL BIOCHEM, V86, P146, DOI 10.1016/j.soilbio.2015.03.018"
"   Zelles L, 1999, BIOL FERT SOILS, V29, P111, DOI 10.1007/s003740050533"
"   Zhang FP, 2010, APPL SOIL ECOL, V45, P144, DOI 10.1016/j.apsoil.2010.03.006"
"   Zhang Y, 2008, PEDOSPHERE, V18, P1, DOI 10.1016/S1002-0160(07)60097-6"
NR 89
TC 165
Z9 190
U1 23
U2 319
PU PERGAMON-ELSEVIER SCIENCE LTD
PI OXFORD
"PA THE BOULEVARD, LANGFORD LANE, KIDLINGTON, OXFORD OX5 1GB, the United Kingdom"
SN 0160-4120
EI 1873-6750
J9 ENVIRON INT
JI Environ. Int.
PD APR
PY 2019
VL 125
BP 478
EP 488
DI 10.1016/j.envint.2019.01.071
PG 11
WC Environmental Sciences
WE Science Citation Index Expanded (SCI-EXPANDED)
SC Environmental Sciences & Ecology
GA HL8PN
UT WOS:000459005200045
PM 30771648
"OA Green Submitted, gold"
DA 2026-04-06
ER

PT J
"AU Bai, GC"
"   Yang, J"
"   Xia, JG"
"   Li, QQ"
"AF Bai, Gen-chuan"
"   Yang, Juan"
"   Xia, Jian-guo"
"   Li, Qi-quan"
TI RECENT ADVANCES ON REMEDIATION OF HEAVY METAL CONTAMINATED SOIL VIA
   BIOCHAR
SO FRESENIUS ENVIRONMENTAL BULLETIN
LA English
DT Article
DE Biochar; heavy metal; soil remediation; load material
ID ENZYME-ACTIVITIES; PARTICLE-SIZE; RICE; CADMIUM; BIOAVAILABILITY;
   NITRIFICATION; TECHNOLOGIES; MECHANISMS; SPECIATION; FERTILIZER

"AB Soil contamination with persistent and potentially (eco)toxic heavy metal(loid)s is ubiquitous around the globe. Concentration of these heavy metal (loid)s in soil has increased drastically over the last three decades, thus posing risk to the environment and human health. Some technologies have long been in use to remediate the hazardous heavy metal(loid)s. Conventional remediation methods for heavy metal(loid)s are generally based on physical, chemical and biological approaches, which may be used in combination with one another to clean-up heavy metal(loid) contaminated soils to an acceptable and safe level. Biochar is a stable carbon-rich by-product synthesized through pyrolysis/carbonization of plant- and animal-based biomass."
"   This review addresses: (1) the functional properties of biochar, and microbial cycling of nutrients in soil; (2) bioremediation, especially treatment of metal and organic contaminants in soils using biochar, and restoration of degraded land. (3) the mechanism involved in soil heavy metal remediation, environmental impacts. (4) the potential risk of being chemical precursors as well as the potential of being load material."
"C1 [Bai, Gen-chuan; Yang, Juan; Xia, Jian-guo; Li, Qi-quan] Sichuan Agr Univ, Coll Resources, 211 Huimin Rd, Chengdu, Sichuan, Peoples R China."
C3 Sichuan Agricultural University
"RP Xia, JG (corresponding author), Sichuan Agr Univ, Coll Resources, 211 Huimin Rd, Chengdu, Sichuan, Peoples R China."
EM xiajianguo@126.com
"RI xia, jianguo/K-3316-2017"
"CR [Anonymous], 2009, CSIRO LAND WATER S B"
"   Bargmann I, 2013, J AGRON CROP SCI, V199, P360, DOI 10.1111/jac.12024"
"   Bhargava A, 2012, J ENVIRON MANAGE, V105, P103, DOI 10.1016/j.jenvman.2012.04.002"
"   Bian LC, 2013, ARCH APPL MECH, V83, P445, DOI 10.1007/s00419-012-0692-7"
"   Erdem H, 2017, FRESEN ENVIRON BULL, V26, P7393"
"   Fang GD, 2015, BIORESOURCE TECHNOL, V176, P210, DOI 10.1016/j.biortech.2014.11.032"
"   FOrstner U., 1995, NONLINEAR RELEASE ME, P247"
"   J??rup L, 2003, BRIT MED BULL, V68, P167, DOI 10.1093/bmb/ldg032"
"   Kappler A, 2014, ENVIRON SCI TECH LET, V1, P339, DOI 10.1021/ez5002209"
"   Khalid S, 2017, J GEOCHEM EXPLOR, V182, P247, DOI 10.1016/j.gexplo.2016.11.021"
"   King GM, 2011, TRENDS MICROBIOL, V19, P75, DOI 10.1016/j.tim.2010.11.006"
"   Lehmann J., 2009, EARTHSCAN"
"   Libra JA, 2011, BIOFUELS-UK, V2, P71, DOI [10.4155/bfs.10.81, 10.4155/BFS.10.81]"
"   Minnikova TV, 2017, J GEOCHEM EXPLOR, V174, P70, DOI 10.1016/j.gexplo.2016.06.007"
"   Morillo E, 2017, SCI TOTAL ENVIRON, V586, P576, DOI 10.1016/j.scitotenv.2017.02.020"
"   Pattnaik BK, 2016, J GEOCHEM EXPLOR, V168, P127, DOI 10.1016/j.gexplo.2016.06.011"
"   Pierart A, 2015, J HAZARD MATER, V289, P219, DOI 10.1016/j.jhazmat.2015.02.011"
"   Qiao JT, 2018, CHEMOSPHERE, V195, P260, DOI [10.1016/j.chemosphere.2017., 10.1016/j.chemosphere.2017.12.081]"
"   Shackley S, 2012, ENERG POLICY, V42, P49, DOI 10.1016/j.enpol.2011.11.026"
"   Shahid M, 2014, SOIL SEDIMENT CONTAM, V23, P389, DOI 10.1080/15320383.2014.831029"
"   Shahid M, 2012, J HAZARD MATER, V219, P1, DOI 10.1016/j.jhazmat.2012.01.060"
"   Shahid M, 2012, CHEM SPEC BIOAVAILAB, V24, P248, DOI 10.3184/095422912X13495331697627"
"   Shahid M, 2012, BIOL FERT SOILS, V48, P689, DOI 10.1007/s00374-012-0662-9"
"   Van Zwieten L, 2010, PLANT SOIL, V327, P235, DOI 10.1007/s11104-009-0050-x"
"   Venegas A, 2016, GEODERMA, V279, P132, DOI 10.1016/j.geoderma.2016.06.010"
"   Verbruggen N, 2009, NEW PHYTOL, V181, P759, DOI 10.1111/j.1469-8137.2008.02748.x"
"   VERHEIJEN F, 2010, EUR 24099"
"   Wang CY, 2018, ECOTOX ENVIRON SAFE, V150, P62, DOI 10.1016/j.ecoenv.2017.12.036"
"   Wardle DA, 2008, SCIENCE, V320, P629, DOI 10.1126/science.1154960"
"   Weyers Sharon L., 2011, Applied and Environmental Soil Science, V2011, P1, DOI 10.1155/2011/541592"
"   Xiong TT, 2016, J ENVIRON SCI, V46, P16, DOI 10.1016/j.jes.2015.08.029"
"   Yang Y, 2017, CHEMOSPHERE, V188, P148, DOI 10.1016/j.chemosphere.2017.08.140"
"   Yang ZM, 2016, ECOTOX ENVIRON SAFE, V132, P224, DOI 10.1016/j.ecoenv.2016.06.008"
"   Yao ZT, 2012, PROCEDIA ENVIRON SCI, V16, P722, DOI 10.1016/j.proenv.2012.10.099"
"   Yuan JH, 2011, BIORESOURCE TECHNOL, V102, P3488, DOI 10.1016/j.biortech.2010.11.018"
"   Zhang KL, 2017, BIOL FERT SOILS, V53, P77, DOI 10.1007/s00374-016-1154-0"
"   Zhang MY, 2017, CHEMOSPHERE, V184, P892, DOI 10.1016/j.chemosphere.2017.06.081"
"   Zhang Q, 2015, APPL SOIL ECOL, V94, P59, DOI 10.1016/j.apsoil.2015.05.005"
"   Zhang RH, 2017, ECOL ENG, V98, P183, DOI 10.1016/j.ecoleng.2016.10.057"
"   Zhang WM, 2016, FRESEN ENVIRON BULL, V25, P602"
"   Zhang W, 2011, CHEMOSPHERE, V85, P1306, DOI 10.1016/j.chemosphere.2011.07.042"
"   Zhu XM, 2017, ENVIRON POLLUT, V227, P98, DOI [10.1016/j.envpol.2017.04.032, 10.1016/j.envPol.2017.04.032]"
"   Zimmerman AR, 2011, SOIL BIOL BIOCHEM, V43, P1169, DOI 10.1016/j.soilbio.2011.02.005"
"   Zimmerman AR, 2010, ENVIRON SCI TECHNOL, V44, P1295, DOI 10.1021/es903140c"
"   Zimmermann M, 2012, GLOBAL CHANGE BIOL, V18, P3306, DOI 10.1111/j.1365-2486.2012.02796.x"
"   Zurbrugg C., 2002, P ISWA2002 ANN C IST"
NR 46
TC 0
Z9 1
U1 1
U2 53
PU PARLAR SCIENTIFIC PUBLICATIONS (P S P)
PI FREISING
"PA ANGERSTR. 12, 85354 FREISING, GERMANY"
SN 1018-4619
EI 1610-2304
J9 FRESEN ENVIRON BULL
JI Fresenius Environ. Bull.
PY 2018
VL 27
IS 8
BP 5455
EP 5461

PG 7
WC Environmental Sciences
WE Science Citation Index Expanded (SCI-EXPANDED)
SC Environmental Sciences & Ecology
GA GT5EF
UT WOS:000444528000032
DA 2026-04-06
ER

PT J
"AU Wang, Y"
"   Luo, Y"
"   Zeng, GQ"
"   Wu, XD"
"   Wu, B"
"   Li, X"
"   Xu, H"
"AF Wang, Ying"
"   Luo, Yao"
"   Zeng, Guoquan"
"   Wu, Xudong"
"   Wu, Bin"
"   Li, Xue"
"   Xu, Heng"
TI Characteristics and in situ remediation effects of heavy metal
   immobilizing bacteria on cadmium and nickel co-contaminated soil
SO ECOTOXICOLOGY AND ENVIRONMENTAL SAFETY
LA English
DT Article
DE Heavy metal; Bacteria; Characteristic; Immobilization; Soil
ID MICROBIAL COMMUNITY; BIOCHAR; CD; BIOSORPTION; REMOVAL;
   EXOPOLYSACCHARIDE; BIOREMEDIATION; PHENANTHRENE; PERFORMANCE; MECHANISM
"AB Cadmium (Cd) and nickel (Ni) in soil have caused serious environmental problems and increased healthy risks to humans and biota, it is vital important and necessary to develop effective methods to resolve the combined contaminated problems. In this study, strains L5 and L6 with good heavy metal resistant and immobilizing capacities were isolated from Cd and Ni contaminated soil. Bacterial characteristic experiment illustrated that many functional groups (-OH, -NH2 and -COO et al.) were distributed on the surface of 1.5 and L6. Under the stress of heavy metals, bacterial appearances were distorted. The pot experiment indicated that the concentrations of HOAc-extractable Cd and Ni in soil reduced 6.26-15.33% and 13.31-19.53% with the inoculation of L5 and L6. In addition, the immobilization rates on Cd and Ni improved 61.27-128.50% and 23.69-39.66% with re-inoculation of strains L5 and L6 at 30 days, respectively. After inoculation of strains L5 and L6 for 60 days, the activities of FDA hydrolysis, acid phosphatase, urease, invertase and dehydrogenase in soil increased obviously. Furthermore, bacterial diversity indexes and community structure of soil were also improved. Thus, given the beneficial remediation effects of the isolated strains, L5 and L6 have great potentials for heavy metals contaminated soil remediation."
"C1 [Wang, Ying; Luo, Yao; Zeng, Guoquan; Wu, Xudong; Wu, Bin; Xu, Heng] Sichuan Univ, Coll Life Sci, Key Lab Bioresource & Ecoenvironm, Minist Educ, Chengdu 610065, Sichuan, Peoples R China."
"   [Li, Xue] Chongqing Univ Technol, Chongqing 400054, Peoples R China."
C3 Sichuan University; Chongqing University of Technology
"RP Li, X (corresponding author), Chongqing Univ Technol, Chongqing 400054, Peoples R China.; Xu, H (corresponding author), Sichuan Univ, 24 South Sect 1,Yihuan Rd, Chengdu 610065, Peoples R China."
EM leexcoco217@163.com; xuheng64@sina.com
"RI ; wu, xudong/KGL-1857-2024"
"OI Xu, Heng/0000-0003-1307-4896; Luo, Yao/0000-0001-5040-8045; "
FU National Key Research and Development Program [2018YFC1802605]; Key
"   Research and Development Program of Sichuan Province [2017SZ0181,"
   2018NZ0008]; Science and Technology Project of Sichuan Province
"   [2019JDRC0092, 2018RZ0110]"
FX This study was financially supported by the National Key Research and
"   Development Program (2018YFC1802605), the Key Research and Development"
"   Program of Sichuan Province (2017SZ0181, 2018NZ0008) and the Science and"
"   Technology Project of Sichuan Province (2019JDRC0092, 2018RZ0110). The"
   authors also wish to thank Professor Guanglei Cheng and Hui Wang from
   Sichuan University for the technical assistance.
"CR Abdulla H, 2009, MICROB ECOL, V58, P753, DOI 10.1007/s00248-009-9549-1"
"   Acosta MP, 2005, WORLD J MICROB BIOT, V21, P1157, DOI 10.1007/s11274-005-0381-6"
"   Adam G, 2001, SOIL BIOL BIOCHEM, V33, P943, DOI 10.1016/S0038-0717(00)00244-3"
"   Ahemad M, 2019, ARAB J CHEM, V12, P1365, DOI 10.1016/j.arabjc.2014.11.020"
"   ANKE M, 1984, IARC SCI PUBL, P339"
"   [Anonymous], NAT SOIL POLL SURV B"
"   [Anonymous], SPRINGER NATURE"
"   [Anonymous], MICROB ECOL"
"   Antony R, 2011, CURR MICROBIOL, V62, P840, DOI 10.1007/s00284-010-9784-1"
"   Ashok Kumar Ashok Kumar, 2010, Journal of Biological & Environmental Sciences, V4, P97"
"   CHAO A, 1984, SCAND J STAT, V11, P265"
"   Chen T, 2010, PROG POLYM SCI, V35, P94, DOI 10.1016/j.progpolymsci.2009.11.004"
"   Emenike CU, 2017, J GEOCHEM EXPLOR, V182, P275, DOI 10.1016/j.gexplo.2016.10.002"
"   Ge ZL, 2011, SCI CHINA CHEM, V54, P1273, DOI 10.1007/s11426-011-4327-6"
"   Gotelli NJ., 1995, PRIMER ECOL, P340"
"   Gu Y, 2009, EUR J SOIL BIOL, V45, P436, DOI 10.1016/j.ejsobi.2009.06.003"
"   Gullotto A, 2015, ENVIRON TECHNOL, V36, P538, DOI 10.1080/09593330.2014.980334"
"   Guo XF, 2016, CHEMOSPHERE, V147, P412, DOI 10.1016/j.chemosphere.2015.12.087"
"   HAUSINGER RP, 1994, SCI TOTAL ENVIRON, V148, P157, DOI 10.1016/0048-9697(94)90392-1"
"   Hua Yu-mei, 2011, China Environmental Science, V31, P795"

"   Jiang C, 2009, J HAZARD MATER, V167, P1170, DOI 10.1016/j.jhazmat.2009.01.107"
"   Jiang J, 2015, ECOTOX ENVIRON SAFE, V120, P386, DOI 10.1016/j.ecoenv.2015.06.028"
"   Jiang XT, 2013, MICROB ECOL, V66, P96, DOI 10.1007/s00248-013-0238-8"
"   Jin L, 2002, LANGMUIR, V18, P9765, DOI 10.1021/la025917l"
"   Karakagh Rahim Mohammadzadeh, 2012, Ecohydrology & Hydrobiology, V12, P191, DOI 10.1016/S1642-3593(12)70203-3"
"   Kiran MG, 2017, J HAZARD MATER, V324, P62, DOI 10.1016/j.jhazmat.2015.12.042"
"   Krishnaswamy R, 2000, APPL ENVIRON MICROB, V66, P5383, DOI 10.1128/AEM.66.12.5383-5386.2000"
"   Kumar S, 2016, MOL BIOL EVOL, V33, P1, DOI [10.1093/molbev/msv279, DOI 10.1109/CVPR.2016.181, 10.1093/molbev/msw054, DOI 10.1093/molbev/msw054]"
"   Li X, 2016, RSC ADV, V6, P65816, DOI 10.1039/c6ra11767h"
"   Liu HK, 2018, SCI TOTAL ENVIRON, V645, P702, DOI 10.1016/j.scitotenv.2018.07.115"
"   Lu WB, 2006, J HAZARD MATER, V134, P80, DOI 10.1016/j.jhazmat.2005.10.036"
"   Lu Y, 2007, ENVIRON MONIT ASSESS, V134, P429, DOI 10.1007/s10661-007-9634-1"
"   Luo XG, 2011, J HAZARD MATER, V187, P182, DOI 10.1016/j.jhazmat.2011.01.019"
"   Ma L, 2013, CHEM ENG J, V225, P59, DOI 10.1016/j.cej.2013.03.044"
"   McLaughlin MJ, 1999, FIELD CROP RES, V60, P143, DOI 10.1016/S0378-4290(98)00137-3"
"   Mori H, 2014, DNA RES, V21, P217, DOI 10.1093/dnares/dst052"
"   Morillo JA, 2006, CURR MICROBIOL, V53, P189, DOI 10.1007/s00284-005-0438-7"
"   P??rez JAM, 2008, WORLD J MICROB BIOT, V24, P2699, DOI 10.1007/s11274-008-9800-9"
"   Nannipieri P., 1994, Soil biota: management in sustainable farming systems., P238"
"   Nepple BB, 1999, MICROBIOL RES, V154, P191"
"   Netherlands, 2008, ENCY GENETICS GENOMI, P1087"
"   Pan JL, 2010, ENVIRON GEOCHEM HLTH, V32, P1, DOI 10.1007/s10653-009-9273-2"
"   Pueyo M, 2003, J ENVIRON QUAL, V32, P2054, DOI 10.2134/jeq2003.2054"
"   Rajdeep Chakravarty Rajdeep Chakravarty, 2007, Research Journal of Microbiology, V2, P742"
"   Rajkumar M, 2012, BIOTECHNOL ADV, V30, P1562, DOI 10.1016/j.biotechadv.2012.04.011"
"   Rau N, 2009, SOIL BIOL BIOCHEM, V41, P813, DOI 10.1016/j.soilbio.2009.01.022"
"   Sarret G, 1998, ENVIRON SCI TECHNOL, V32, P1648, DOI 10.1021/es9709684"
"   Satarug S, 2004, TOXICOL LETT, V148, P177, DOI 10.1016/j.toxlet.2003.09.015"
"   Shrestha B, 2013, J HAZARD MATER, V261, P188, DOI 10.1016/j.jhazmat.2013.07.031"
"   [?????? Sun Tao], 2014, [????????????, Geology of China], V41, P1986"
"   Sungur A, 2015, SOIL SEDIMENT CONTAM, V24, P1, DOI 10.1080/15320383.2014.907238"
"   Tabatabai M. A., 1969, Soil Biology and Biochemistry, V1, P301, DOI 10.1016/0038-0717(69)90012-1"
"   Tan H, 2020, J HAZARD MATER, V386, DOI 10.1016/j.jhazmat.2019.121628"
"   Wang C, 2016, RSC ADV, V6, P37274, DOI 10.1039/c6ra05457a"
"   Wang Q, 2007, APPL ENVIRON MICROB, V73, P5261, DOI 10.1128/AEM.00062-07"
"   Wang T, 2014, EUR J SOIL BIOL, V65, P62, DOI 10.1016/j.ejsobi.2014.10.001"
"   Wang Y, 2018, J SOIL SEDIMENT, V18, P2136, DOI 10.1007/s11368-017-1762-9"
"   Wang Y, 2018, SCI TOTAL ENVIRON, V621, P1057, DOI 10.1016/j.scitotenv.2017.10.121"
"   Wei BG, 2010, MICROCHEM J, V94, P99, DOI 10.1016/j.microc.2009.09.014"
"   Wu B, 2019, SCI TOTAL ENVIRON, V686, P719, DOI 10.1016/j.scitotenv.2019.06.041"
"   Wu B, 2018, ECOTOX ENVIRON SAFE, V166, P116, DOI 10.1016/j.ecoenv.2018.09.076"
"   Wu B, 2016, SCI TOTAL ENVIRON, V562, P732, DOI 10.1016/j.scitotenv.2016.04.079"
"   Wu MH, 2019, J HAZARD MATER, V368, P412, DOI 10.1016/j.jhazmat.2019.01.059"
"   Xiao KM, 2017, ACS APPL MATER INTER, V9, P30448, DOI 10.1021/acsami.7b09520"
"   Xu N, 2016, EUR J SOIL BIOL, V74, P1, DOI 10.1016/j.ejsobi.2016.02.004"
"   Yao ZT, 2012, PROCEDIA ENVIRON SCI, V16, P722, DOI 10.1016/j.proenv.2012.10.099"
"   Yin DX, 2016, CHEMOSPHERE, V152, P196, DOI 10.1016/j.chemosphere.2016.01.044"
"   [????????? Yuan Shanshan], 2012, [?????????????????????, Environmental Pollution & Control], V34, P51"
"   Zhan XH, 2010, J ENVIRON SCI, V22, P607, DOI [10.1016/S1001-0742(09)60139-x, 10.1016/S1001-0742(09)60139-X]"
NR 69
TC 75
Z9 84
U1 3
U2 171
PU ACADEMIC PRESS INC ELSEVIER SCIENCE
PI SAN DIEGO
"PA 525 B ST, STE 1900, SAN DIEGO, CA 92101-4495 USA"
SN 0147-6513
EI 1090-2414
J9 ECOTOX ENVIRON SAFE
JI Ecotox. Environ. Safe.
PD APR 1
PY 2020
VL 192
AR 110294
DI 10.1016/j.ecoenv.2020.110294
PG 9
WC Environmental Sciences; Toxicology
WE Science Citation Index Expanded (SCI-EXPANDED)
SC Environmental Sciences & Ecology; Toxicology
GA KS7QX
UT WOS:000518502300034
PM 32044601
DA 2026-04-06
ER

PT J
"AU Li, H"
"   Wang, T"
"   Du, HX"
"   Guo, P"

"   Wang, SF"
"   Ma, M"
"AF Li, Hong"
"   Wang, Tao"
"   Du, Hongxia"
"   Guo, Pan"
"   Wang, Shufeng"
"   Ma, Ming"
TI Research Progress in the Joint Remediation of Plants-Microbes-Soil for
   Heavy Metal-Contaminated Soil in Mining Areas: A Review
SO SUSTAINABILITY
LA English
DT Review
DE joint remediation of plant-microbe; phytoremediation; microbes; HMs;
   bioremediation
ID ARBUSCULAR MYCORRHIZAL FUNGI; GENOME-WIDE ANALYSIS;
   ROBINIA-PSEUDOACACIA; CADMIUM ACCUMULATION; RISK-ASSESSMENT;
   HEALTH-RISKS; CD UPTAKE; GROWTH; TOLERANCE; STRESS
"AB Plants growing in heavy metal (HM)-contaminated soil have evolved a special detoxification mechanism. The rhizosphere gathers many living substances and their secretions at the center of plant roots, which has a unique ecological remediation effect. It is of great significance to thoroughly understand the ecological process of rhizosphere pollution under heavy metals (HMs) stress and develop biotechnology for joint remediation using plants and their coexisting microbial systems according to the mechanism of rhizosphere stress. Microbes can weaken the toxicity of HM pollutants by transforming the existing forms or reducing the bioavailability in the rhizosphere. Microbes survive in the HM-polluted soils through the production of stress-resistant substances, the participation of proteins, and the expression of heavy metal resistance genes, which strengthens the resistance of plants. Moreover, microbes can improve the nutritional status of plants to improve plant resistance to HMs. Plants, in turn, provide a habitat for microbes to survive and reproduce, which greatly accelerates the process of bioremediation. Briefly, the combined remediation of soil HMs pollution by plants and microbes is a promising, green, and sustainable strategy. Here, we mainly elucidate the joint remediation mechanism of plant-microbe symbiosis and introduce the coping characteristics of plants, microbes, and their symbiotic system, hoping to provide a scientific basis for the remediation of HM-contaminated soil in mining areas and the sustainable development of the ecological environment."
"C1 [Li, Hong; Wang, Shufeng; Ma, Ming] Southwest Univ, Coll Resources & Environm, Key Lab Three Gorges Reservoir Area, Minist Educ, Chongqing 400715, Peoples R China."
"   [Wang, Tao; Guo, Pan; Wang, Shufeng; Ma, Ming] Southwest Univ, Coll Resources & Environm, Ctr Mol Ecophysiol CMEP, Chongqing 400715, Peoples R China."
"   [Du, Hongxia] Southwest Univ, Coll Resources & Environm, Chongqing Key Lab Bioresource Bioenergy, Chongqing 400715, Peoples R China."
C3 Southwest University - China; Southwest University - China; Southwest
   University - China
"RP Wang, SF (corresponding author), Southwest Univ, Coll Resources & Environm, Key Lab Three Gorges Reservoir Area, Minist Educ, Chongqing 400715, Peoples R China.; Wang, SF (corresponding author), Southwest Univ, Coll Resources & Environm, Ctr Mol Ecophysiol CMEP, Chongqing 400715, Peoples R China."
EM haru023@swu.edu.cn; 15340571977@139.com; duhx@swu.edu.cn;
   wo05422@163.com; wangsf2020@swu.edu.cn; maming@swu.edu.cn
"RI Ma, Ming/KYP-0980-2024"
"OI Ma, Ming/0000-0001-6915-0953; Wang, Shufeng/0000-0002-0775-3617"
FU National Natural Science Foundation of China [42177198]; Fundamental
   Research Funds for the Central Universities [SWU019019]
FX This research was supported by the National Natural Science Foundation
   of China (grant number 42177198) and Fundamental Research Funds for the
   Central Universities (grant number SWU019019).
"CR Adebayo AS, 2022, ENVIRON EARTH SCI, V81, DOI 10.1007/s12665-022-10581-9"
"   Agrawal K, 2024, CRIT REV BIOTECHNOL, V44, P429, DOI 10.1080/07388551.2023.2170862"
"   Ahammed GJ, 2023, ENVIRON POLLUT, V327, DOI 10.1016/j.envpol.2023.121597"
"   Aloui A, 2011, BMC PLANT BIOL, V11, DOI 10.1186/1471-2229-11-75"
"   Salas-Lu??vano MA, 2017, ENVIRON EARTH SCI, V76, DOI 10.1007/s12665-017-7139-y"
"   Armendariz AL, 2019, PLANT PHYSIOL BIOCH, V138, P26, DOI 10.1016/j.plaphy.2019.02.018"
"   Asensio V, 2013, SCI TOTAL ENVIRON, V443, P446, DOI 10.1016/j.scitotenv.2012.09.069"
"   Beckers F, 2017, CRIT REV ENV SCI TEC, V47, P693, DOI 10.1080/10643389.2017.1326277"
"   Boughattas I, 2016, ENVIRON POLLUT, V218, P530, DOI 10.1016/j.envpol.2016.07.033"
"   Bruno LB, 2021, J ENVIRON MANAGE, V289, DOI 10.1016/j.jenvman.2021.112553"
"   Cai LM, 2015, CHEMOSPHERE, V127, P127, DOI 10.1016/j.chemosphere.2015.01.027"
"   Cardoso P, 2018, ECOTOX ENVIRON SAFE, V150, P260, DOI 10.1016/j.ecoenv.2017.12.002"
"   Chen GC, 2013, ENVIRON SCI POLLUT R, V20, P5665, DOI 10.1007/s11356-013-1506-3"
"   Chen L, 2014, SOIL BIOL BIOCHEM, V68, P300, DOI 10.1016/j.soilbio.2013.10.021"
"   Chen XW, 2019, GEODERMA, V337, P749, DOI 10.1016/j.geoderma.2018.10.029"
"   Chen YF, 2021, CHEMOSPHERE, V269, DOI 10.1016/j.chemosphere.2020.128673"
"   Cheng XY, 2022, ENVIRON SCI POLLUT R, V29, P89557, DOI 10.1007/s11356-022-21711-y"
"   Christou A, 2017, J GEOCHEM EXPLOR, V178, P16, DOI 10.1016/j.gexplo.2017.03.012"
"   Chu Ngoc Kien, 2011, Tropical Agriculture and Development, V55, P135"
"   Cicatelli A, 2014, ENVIRON SCI POLLUT R, V21, P1723, DOI 10.1007/s11356-013-2072-4"
"   Clemens S, 2016, ANNU REV PLANT BIOL, V67, P489, DOI 10.1146/annurev-arplant-043015-112301"
"   Cornejo P, 2013, SOIL BIOL BIOCHEM, V57, P925, DOI 10.1016/j.soilbio.2012.10.031"
"   Cui GJ, 2019, ECOTOX ENVIRON SAFE, V171, P231, DOI 10.1016/j.ecoenv.2018.12.093"
"   Cui JL, 2020, J HAZARD MATER, V381, DOI 10.1016/j.jhazmat.2019.121208"
"   Dai DC, 2024, FORESTS, V15, DOI 10.3390/f15010115"
"   Dawuda MM, 2019, PEERJ, V7, DOI 10.7717/peerj.7530"
"   De Oliveira VH, 2020, ENVIRON EXP BOT, V171, DOI 10.1016/j.envexpbot.2019.103925"
"   Deepika KV, 2016, ECOTOX ENVIRON SAFE, V134, P1, DOI 10.1016/j.ecoenv.2016.08.008"
"   Dhalaria R, 2020, AGRONOMY-BASEL, V10, DOI 10.3390/agronomy10060815"
"   Di Giuseppe EC, 2024, TOXICS, V12, DOI 10.3390/toxics12040261"
"   Dom??nguez MT, 2011, CHEMOSPHERE, V83, P1166, DOI 10.1016/j.chemosphere.2011.01.002"

"   Du BG, 2019, SCI TOTAL ENVIRON, V688, P333, DOI 10.1016/j.scitotenv.2019.06.245"
"   Du HX, 2021, ENVIRON POLLUT, V273, DOI 10.1016/j.envpol.2021.116431"
"   Edulamudi P, 2023, CURR MICROBIOL, V80, DOI 10.1007/s00284-023-03278-0"
"   Escarr?? J, 2011, WATER AIR SOIL POLL, V216, P485, DOI 10.1007/s11270-010-0547-1"
"   Fan MC, 2018, CHEMOSPHERE, V197, P729, DOI 10.1016/j.chemosphere.2018.01.102"
"   Faraz A, 2020, J PLANT GROWTH REGUL, V39, P641, DOI 10.1007/s00344-019-10007-0"
"   Fatnassi IC, 2015, CR BIOL, V338, P241, DOI 10.1016/j.crvi.2015.02.001"
"   Feng JJ, 2018, PLANT BIOTECHNOL J, V16, P558, DOI 10.1111/pbi.12795"
"   Festin ES, 2019, ECOL ENG, V138, P118, DOI 10.1016/j.ecoleng.2019.07.010"
"   Gao F, 2022, PLOS ONE, V17, DOI 10.1371/journal.pone.0274319"
"   Gao HL, 2018, NEW PHYTOL, V217, P179, DOI 10.1111/nph.14783"
"   Gatasheh MK, 2024, BMC PLANT BIOL, V24, DOI 10.1186/s12870-024-05359-z"
"   Gil-Cardeza ML, 2018, SCI TOTAL ENVIRON, V625, P1113, DOI 10.1016/j.scitotenv.2017.12.278"
"   Gonz??lez A, 2021, FRONT PLANT SCI, V12, DOI 10.3389/fpls.2021.669096"
"   Gonz??lez-Guerrero M, 2010, MYCORRHIZA, V20, P137, DOI 10.1007/s00572-009-0273-y"
"   Gonz??lez-M??ndez B, 2022, ENVIRON EARTH SCI, V81, DOI 10.1007/s12665-022-10285-0"
"   Guarino C, 2019, SCI TOTAL ENVIRON, V650, P3156, DOI 10.1016/j.scitotenv.2018.09.343"
"   Guo JK, 2018, ECOTOX ENVIRON SAFE, V157, P491, DOI 10.1016/j.ecoenv.2018.04.010"
"   Guo JK, 2014, PLANT SOIL, V375, P205, DOI 10.1007/s11104-013-1952-1"
"   Gupta DK, 2017, PLANT CELL ENVIRON, V40, P509, DOI 10.1111/pce.12711"
"   Hao QingJu Hao QingJu, 2015, Acta Ecologica Sinica - International Journal, V35, P46, DOI 10.1016/j.chnaes.2015.01.002"
"   Hao XL, 2015, ENVIRON SCI TECHNOL, V49, P2328, DOI 10.1021/es504956a"
"   He BY, 2017, CHEMOSPHERE, V171, P588, DOI 10.1016/j.chemosphere.2016.12.085"
"   He YM, 2020, ENVIRON POLLUT, V263, DOI 10.1016/j.envpol.2020.114406"
"   Hu PJ, 2015, PLANT SOIL, V388, P267, DOI 10.1007/s11104-014-2321-4"
"   Huber M, 2022, MINING-BASEL, V2, P616, DOI [10.3390/mining2030033, 10.3390/mining2030033]"
"   Ike A, 2007, CHEMOSPHERE, V66, P1670, DOI 10.1016/j.chemosphere.2006.07.058"
"   Inouhe M., 2015, REACTIVE OXYGEN SPEC, P219, DOI DOI 10.1007/978-3-319-20421-59"
"   Jaiswal SK, 2018, BIOL FERT SOILS, V54, P309, DOI 10.1007/s00374-018-1262-0"
"   Jalmi SK, 2018, FRONT PLANT SCI, V9, DOI 10.3389/fpls.2018.00012"
"   Jia BB, 2023, LAND DEGRAD DEV, V34, P506, DOI 10.1002/ldr.4475"
"   Jia ZM, 2018, SCI REP-UK, V8, DOI 10.1038/s41598-018-21569-6"
"   Jian LR, 2019, PEERJ, V7, DOI 10.7717/peerj.6875"
"   Kamran M, 2021, CHEMOSPHERE, V263, DOI [10.1016/j.chemosphere.2020.128169, 10.1016/J.chemosphere.2020.128169]"
"   Khan AHA, 2023, CHIANG MAI J SCI, V50, DOI 10.12982/CMJS.2023.008"
"   Khan IU, 2023, PLANTS-BASEL, V12, DOI 10.3390/plants12040725"
"   Khan IU, 2020, J CLEAN PROD, V272, DOI 10.1016/j.jclepro.2020.123152"
"   Khan NM, 2024, BMC PLANT BIOL, V24, DOI 10.1186/s12870-024-05201-6"
"   Lago-Vila M, 2019, J ENVIRON MANAGE, V235, P152, DOI 10.1016/j.jenvman.2019.01.058"
"   Lakzian A., 2007, viciae in heavy metal contaminated soils. Soil Biol. Biochem, V39, P1066, DOI [10.1016/j.soilbio.2006.12.018, DOI 10.1016/J.SOILBIO.2006.12.018]"
"   Lanquar V, 2010, PLANT PHYSIOL, V152, P1986, DOI 10.1104/pp.109.150946"
"   Latef AAHA, 2018, LAND DEGRAD DEV, V29, P1065, DOI 10.1002/ldr.2780"
"   Li JX, 2024, INT J MOL SCI, V25, DOI 10.3390/ijms25010613"
"   Li NN, 2017, J EXP BOT, V68, P5669, DOI 10.1093/jxb/erx370"
"   Li QQ, 2024, TOXICS, V12, DOI 10.3390/toxics12040291"
"   Li SQ, 2018, SCI TOTAL ENVIRON, V618, P1314, DOI 10.1016/j.scitotenv.2017.09.252"
"   Li XM, 2023, QUAL ASSUR SAF CROP, V15, P59, DOI 10.15586/qas.v15i2.1182"
"   Li YL, 2023, ENVIRON POLLUT, V325, DOI 10.1016/j.envpol.2023.121433"
"   Liu H, 2017, NEW PHYTOL, V215, P687, DOI 10.1111/nph.14622"
"   Liu H, 2015, APPL SOIL ECOL, V89, P44, DOI 10.1016/j.apsoil.2015.01.006"
"   Long JM, 2018, HUM ECOL RISK ASSESS, V24, P679, DOI 10.1080/10807039.2017.1396531"
"   Lu JZ, 2019, ENVIRON POLLUT, V255, DOI 10.1016/j.envpol.2019.113255"
"   Lu QY, 2020, ENVIRON SCI POLLUT R, V27, P8719, DOI 10.1007/s11356-019-07512-w"
"   Mani D, 2014, INT J ENVIRON SCI TE, V11, P843, DOI 10.1007/s13762-013-0299-8"
"   Marrugo-Negrete J, 2016, SCI TOTAL ENVIRON, V542, P809, DOI 10.1016/j.scitotenv.2015.10.117"
"   Modabberi S, 2023, ENVIRON GEOCHEM HLTH, V45, P4795, DOI 10.1007/s10653-023-01537-4"
"   Mondal S, 2023, PLANT MOL BIOL REP, V41, P15, DOI 10.1007/s11105-022-01350-w"
"   Monfared SH, 2013, J FORESTRY RES, V24, P391, DOI 10.1007/s11676-012-0313-x"
"   Mushtaq MU, 2020, ENVIRON SCI POLLUT R, V27, P39807, DOI 10.1007/s11356-020-08839-5"
"   Nouri Mohamed, 2016, Ekologia (Bratislava), V35, P32, DOI [10.1515/eko-2016-0003, 10.1515/eko-2016-0003]"
"   Ogunlaja A, 2019, SCI TOTAL ENVIRON, V651, P2943, DOI 10.1016/j.scitotenv.2018.09.386"
"   Ovecka M, 2014, BIOTECHNOL ADV, V32, P73, DOI 10.1016/j.biotechadv.2013.11.011"
"   Palusinska M, 2020, BMC PLANT BIOL, V20, DOI 10.1186/s12870-020-2255-3"
"   Panqing Y, 2023, SCI REP-UK, V13, DOI 10.1038/s41598-023-33986-3"
"   Peroni P, 2024, PLANTS-BASEL, V13, DOI 10.3390/plants13131866"
"   Pittman JK, 2016, PLANT BIOLOGY, V18, P741, DOI 10.1111/plb.12460"
"   Radi N, 2023, ENVIRON MONIT ASSESS, V195, DOI 10.1007/s10661-022-10862-7"
"   Radu VM, 2023, SUSTAINABILITY-BASEL, V15, DOI 10.3390/su152115293"
"   Reeves RD, 2018, NEW PHYTOL, V218, P407, DOI 10.1111/nph.14907"
"   Riaz M, 2021, J HAZARD MATER, V402, DOI 10.1016/j.jhazmat.2020.123919"
"   Rubio-Santiago J, 2023, PLANTS-BASEL, V12, DOI 10.3390/plants12030498"
"   Sahoo R, 2024, DISCOV APPL SCI, V6, DOI 10.1007/s42452-024-06024-8"
"   Saleem K, 2023, BIOL BULL+, V50, P390, DOI 10.1134/S1062359022602245"
"   Saleem MH, 2020, CHEMOSPHERE, V248, DOI 10.1016/j.chemosphere.2020.126032"
"   Samara T, 2020, SUSTAINABILITY-BASEL, V12, DOI 10.3390/su12114453"
"   S??nchez-Pardo B, 2014, ECOTOX ENVIRON SAFE, V102, P1, DOI 10.1016/j.ecoenv.2014.01.016"
"   Sarwar N, 2017, CHEMOSPHERE, V171, P710, DOI 10.1016/j.chemosphere.2016.12.116"
"   Schneider L, 2019, SCI TOTAL ENVIRON, V656, P250, DOI 10.1016/j.scitotenv.2018.11.241"
"   Sheng YB, 2019, PLANT CELL ENVIRON, V42, P891, DOI 10.1111/pce.13457"
"   Shi WG, 2019, PLANT CELL ENVIRON, V42, P1087, DOI 10.1111/pce.13471"
"   Shin MN, 2012, J HAZARD MATER, V199, P314, DOI 10.1016/j.jhazmat.2011.11.010"

"   Shoaib M, 2021, ECOTOX ENVIRON SAFE, V217, DOI 10.1016/j.ecoenv.2021.112244"
"   Skrobala V, 2022, MIN MINER DEPOSITS, V16, P71, DOI 10.33271/mining16.04.071"
"   Stefanescu L, 2020, LAND USE POLICY, V95, DOI 10.1016/j.landusepol.2018.10.011"
"   Tao Q, 2017, J EXP BOT, V68, P739, DOI 10.1093/jxb/erw453"
"   Tepanosyan G, 2020, ENVIRON POLLUT, V261, DOI 10.1016/j.envpol.2020.114210"
"   Thomas J, 2023, BIOCATAL AGR BIOTECH, V47, DOI 10.1016/j.bcab.2022.102592"
"   Timofeev I, 2018, SCI TOTAL ENVIRON, V642, P63, DOI 10.1016/j.scitotenv.2018.06.042"
"   Torres D, 2022, J SOIL SCI PLANT NUT, V22, P4669, DOI 10.1007/s42729-022-00948-z"
"   Ullah A, 2015, ENVIRON EXP BOT, V117, P28, DOI 10.1016/j.envexpbot.2015.05.001"
"   Velasco-Arroyo B, 2024, NEW BIOTECHNOL, V79, P50, DOI 10.1016/j.nbt.2023.12.003"
"   Veskovic J, 2024, METALS-BASEL, V14, DOI 10.3390/met14060719"
"   Vezza ME, 2022, ENVIRON SCI POLLUT R, V29, P88066, DOI 10.1007/s11356-022-21443-z"
"   Vezza ME, 2020, ENVIRON SCI POLLUT R, V27, P2287, DOI 10.1007/s11356-019-06959-1"
"   Wang FY, 2022, PEDOSPHERE, V32, P844, DOI 10.1016/j.pedsph.2022.06.011"
"   Wang LH, 2020, CHEMOSPHERE, V252, DOI 10.1016/j.chemosphere.2020.126522"
"   Wang Y, 2020, ECOTOX ENVIRON SAFE, V192, DOI 10.1016/j.ecoenv.2020.110294"
"   Wekesa C, 2022, CELLS-BASEL, V11, DOI 10.3390/cells11050873"
"   Wu KR, 2018, ENVIRON SCI POLLUT R, V25, P21844, DOI 10.1007/s11356-018-2322-6"
"   Wu YJ, 2020, SCI TOTAL ENVIRON, V726, DOI 10.1016/j.scitotenv.2020.138554"
"   Xiao R, 2017, ECOTOX ENVIRON SAFE, V141, P17, DOI 10.1016/j.ecoenv.2017.03.002"
"   Xu DM, 2017, ENVIRON SCI POLLUT R, V24, P24387, DOI 10.1007/s11356-017-0052-9"
"   Xu S, 2017, BIOCHEM BIOPH RES CO, V490, P528, DOI 10.1016/j.bbrc.2017.06.073"
"   Yamagata A, 2022, NAT COMMUN, V13, DOI 10.1038/s41467-022-34930-1"
"   Yang S, 2019, CHINA GEOL, V2, P501, DOI 10.31035/cg2018128"
"   Yildirim D, 2017, J GEOCHEM EXPLOR, V182, P228, DOI 10.1016/j.gexplo.2016.11.005"
"   Yin YC, 2015, RSC ADV, V5, P47584, DOI 10.1039/c5ra05717e"
"   Yoneyama T, 2015, INT J MOL SCI, V16, P19111, DOI 10.3390/ijms160819111"
"   Yu HY, 2021, ENVIRON SCI POLLUT R, V28, P25432, DOI 10.1007/s11356-021-12373-3"
"   Zhakypbek Y, 2024, PLANTS-BASEL, V13, DOI 10.3390/plants13111534"
"   Zhang ZZ, 2017, PLANT MOL BIOL REP, V35, P534, DOI 10.1007/s11105-017-1042-2"
"   Zhang P, 2019, PLANT PHYSIOL, V180, P529, DOI 10.1104/pp.18.01380"
"   Zhang XQ, 2024, MICROORGANISMS, V12, DOI 10.3390/microorganisms12071255"
"   Zhang YF, 2011, J HAZARD MATER, V186, P1720, DOI 10.1016/j.jhazmat.2010.12.069"
"   Zhao MF, 2020, ECOL ENG, V148, DOI 10.1016/j.ecoleng.2020.105790"
"   Zhou BR, 2014, INT J MOL SCI, V15, P10398, DOI 10.3390/ijms150610398"
"   Zhou H, 2024, SCI TOTAL ENVIRON, V906, DOI 10.1016/j.scitotenv.2023.167772"
"   Zhu G, 2020, J CLEAN PROD, V257, DOI 10.1016/j.jclepro.2020.120664"
NR 149
TC 14
Z9 18
U1 51
U2 175
PU MDPI
PI BASEL
"PA MDPI AG, Grosspeteranlage 5, CH-4052 BASEL, SWITZERLAND"
EI 2071-1050
J9 SUSTAINABILITY-BASEL
JI Sustainability
PD OCT
PY 2024
VL 16
IS 19
AR 8464
DI 10.3390/su16198464
PG 21
WC Green & Sustainable Science & Technology; Environmental Sciences;
   Environmental Studies
WE Science Citation Index Expanded (SCI-EXPANDED); Social Science Citation Index (SSCI)
SC Science & Technology - Other Topics; Environmental Sciences & Ecology
GA I8T0M
UT WOS:001332912500001
OA gold
DA 2026-04-06
ER

PT J
"AU Kim, JH"
"   Lee, JY"
"AF Kim, Joon-Ha"
"   Lee, Jai-Young"
TI An optimum condition of MICP indigenous bacteria with contaminated
   wastes of heavy metal
SO JOURNAL OF MATERIAL CYCLES AND WASTE MANAGEMENT
LA English
DT Article
DE CaCO3; Heavy metal; Soil; Mine tailing; S; S; Indigenous bacteria
ID CALCIUM-CARBONATE; BIOREMEDIATION; PRECIPITATION; SOIL;
   BIOMINERALIZATION; MICROORGANISMS; ANHYDRASE; CEMENT; UREASE; PB
"AB Heavy metals are naturally occurring trace elements present in the environment, including soil, water, and air. However, high heavy metal concentration contamination from waste is a serious environmental problem brought on by industrial activities. The research in this study evaluated different biomineralized CaCO3 forms from individual bacteria species in heavy-metal-contaminated soils and mine tailings. Scanning electron microscope (SEM) images of the crystals were used to characterize the precipitated CaCO3. Generally, urea-hydrolysis bacteria form minerals via a microbiologically induced calcite precipitation (MICP) process. These

bacteria produce the urease enzyme, which leads to urea-hydrolysis. These bacteria were isolated from heavy-metal-contaminated soils and characterized for their potential utilization in the S/S process. Optimum conditions for indigenous bacterial growth were 30 degrees C and a pH range of 7-8; and growth patterns were further affected by the growth medium salinity. SEM and X-ray diffraction (XRD) analyses demonstrated that bioaccumulated heavy metal ions were deposited around the cell envelope as rhombohedral and sphere shaped crystalline carbonate minerals in optimum conditions. In this study, the authors hypothesize that the indigenous bacteria can effectively precipitate heavy metals in soil and mine tailing with the urea-hydrolysis enzyme, and play an important role in heavy metal stabilization."
"C1 [Kim, Joon-Ha; Lee, Jai-Young] Univ Seoul, Dept Environm Engn, Seoul, South Korea."
C3 University of Seoul
"RP Lee, JY (corresponding author), Univ Seoul, Dept Environm Engn, Seoul, South Korea."
EM leejy@uos.ac.kr
FU Korea Ministry of Environment (MOE) as part of the GAIA Project
(Geo-Advanced Innovative Action Project) [2015000550007]
FX This study was supported by the Korea Ministry of Environment (MOE) as
part of the GAIA Project (Geo-Advanced Innovative Action Project; No.
2015000550007).
"CR Abo-El-Enein SA., 2012, Housing and Building National Research Center Journal, V8, P185, DOI [10.1016/j.hbrcj.2012.10.002, DOI 10.1016/J.HBRCJ.2013.02.001, 10.1016/j.hbrcj.2013.02.001]"
" Achal V, 2009, EXCELLENCE IN CONCRETE CONSTRUCTION THROUGH INNOVATION, P27"
" Achal V, 2012, J MICROBIOL BIOTECHN, V22, P244, DOI 10.4014/jmb.1108.08033"
" Alpaslan B, 2002, WATER AIR SOIL POLL, V133, P253, DOI 10.1023/A:1012977829536"
" Anne S, 2010, CONSTR BUILD MATER, V24, P1036, DOI 10.1016/j.conbuildmat.2009.11.014"
" Bang SS, 2001, ENZYME MICROB TECH, V28, P404, DOI 10.1016/S0141-0229(00)00348-3"
" Bauerlein E., 2007, Handbook of biomineralization, 3 volume set"
" Bazylinski DA, 2007, GEOMICROBIOL J, V24, P465, DOI 10.1080/01490450701572259"
" Braissant O, 2003, J SEDIMENT RES, V73, P485, DOI 10.1306/111302730485"
" Chen QY, 2009, WASTE MANAGE, V29, P390, DOI 10.1016/j.wasman.2008.01.019"
" Cheung KH, 2007, INT BIODETER BIODEGR, V59, P8, DOI 10.1016/j.ibiod.2006.05.002"
" Cheung KH, 2006, J MICROBIOL BIOTECHN, V16, P855"
" De Yoreo JJ, 2003, REV MINERAL GEOCHEM, V54, P57, DOI 10.2113/0540057"
" DeJong JT, 2006, J GEOTECH GEOENVIRON, V132, P1381, DOI 10.1061/(ASCE)1090-0241(2006)132:11(1381)"
" Dhami NK, 2014, APPL BIOCHEM BIOTECH, V172, P2552, DOI 10.1007/s12010-013-0694-0"
" Dhami NK, 2013, J MICROBIOL BIOTECHN, V23, P707, DOI 10.4014/jmb.1212.11087"
" Dick J, 2006, BIODEGRADATION, V17, P357, DOI 10.1007/s10532-005-9006-x"
" Dupraz S, 2009, CHEM GEOL, V265, P54, DOI 10.1016/j.chemgeo.2008.12.012"
" Favre N, 2009, J MOL CATAL B-ENZYM, V60, P163, DOI 10.1016/j.molcatb.2009.04.018"
" FERRER MR, 1988, GEOMICROBIOL J, V6, P49, DOI 10.1080/01490458809377821"
" Ferris FG, 2004, GEOCHIM COSMOCHIM AC, V68, P1701, DOI 10.1016/S0016-7037(03)00503-9"
" GOLLAPUDI UK, 1995, CHEMOSPHERE, V30, P695, DOI 10.1016/0045-6535(94)00435-W"
" Gorospe CM, 2013, BIOTECHNOL BIOPROC E, V18, P903, DOI 10.1007/s12257-013-0030-0"
" Hammes F, 2003, APPL ENVIRON MICROB, V69, P4901, DOI 10.1128/AEM.69.8.4901-4909.2003"
" Hammes F., 2002, ENV SCI BIO TECHNOLO, V1, P3, DOI DOI 10.1023/A:1015135629155"
" Hwang Balbina Y, 2005, THESIS"
" Ivanov Volodymyr, 2008, Reviews in Environmental Science and Bio/Technology, V7, P139, DOI 10.1007/s11157-007-9126-3"
" Jang A, 2000, MINER ENG, V13, P1659, DOI 10.1016/S0892-6875(00)00151-5"
" Kang CH, 2014, APPL BIOCHEM BIOTECH, V172, P1929, DOI 10.1007/s12010-013-0626-z"
" Kawaguchi T, 2002, J CRYST GROWTH, V240, P230, DOI 10.1016/S0022-0248(02)00918-1"
" Lee J., 2011, THESIS"
" Lee JY, 2001, SOIL ENV ENG"
" Lee S, 2007, SOIL TEST METHOD"
" Liu DX, 2006, LANGMUIR, V22, P5566, DOI 10.1021/la060612i"
" Loewenthal RE, 1978, CARBONATE CHEM AQUAT"
" MILLER LT, 1982, J CLIN MICROBIOL, V16, P584, DOI 10.1128/JCM.16.3.584-586.1982"
" Mitchell AC, 2005, GEOCHIM COSMOCHIM AC, V69, P4199, DOI 10.1016/j.gca.2005.03.014"
" MOBLEY HLT, 1995, MICROBIOL REV, V59, P451, DOI 10.1128/MMBR.59.3.451-480.1995"
" Okwadha GDO, 2010, CHEMOSPHERE, V81, P1143, DOI 10.1016/j.chemosphere.2010.09.066"
" Olayinka K. O., 2011, Journal of Soil Science and Environmental Management, V2, P220"
" Park SJ, 2010, J MICROBIOL BIOTECHN, V20, P782, DOI 10.4014/jmb.0911.11015"
" Phae CG, 2002, SOIL CONTAMINATION M"
" Pruvot C, 2006, J SOIL SEDIMENT, V6, P215, DOI 10.1065/jss2006.10.186"
" Ramachandran SK, 2001, ACI MATER J, V98, P3"
" Sarda D, 2009, J IND MICROBIOL BIOT, V36, P1111, DOI 10.1007/s10295-009-0581-4"
" ?????????, 2003, [Economic and Environmental Geology, ??????????????€???], V36, P27"
" Soon NW, 2013, KSCE J CIV ENG, V17, P718, DOI 10.1007/s12205-013-0149-8"
" Stocks-Fischer S, 1999, SOIL BIOL BIOCHEM, V31, P1563, DOI 10.1016/S0038-0717(99)00082-6"
" Tai CY, 1998, AICHE J, V44, P1790, DOI 10.1002/aic.690440810"
" Weaver R.W., 1994, METHODS SOIL ANAL 2"
" Williams MSR, 1998, MAT SCI ENG C-BIOMIM, V6, P47, DOI 10.1016/S0928-4931(98)00034-4"
" Zhuang P, 2009, ENVIRON GEOCHEM HLTH, V31, P707, DOI 10.1007/s10653-009-9248-3"
NR 52
TC 53
Z9 60
U1 4
U2 128
PU SPRINGER
PI NEW YORK
"PA 233 SPRING ST, NEW YORK, NY 10013 USA"
SN 1438-4957
EI 1611-8227
J9 J MATER CYCLES WASTE
JI J. Mater. Cycles Waste Manag.
PD MAR

PY 2019
VL 21
IS 2
BP 239
EP 247
DI 10.1007/s10163-018-0779-5
PG 9
WC Environmental Sciences
WE Science Citation Index Expanded (SCI-EXPANDED)
SC Environmental Sciences & Ecology
GA HN2HO
UT WOS:000460006500003
DA 2026-04-06
ER

PT J
"AU Yaashikaa, PR"
"   Kumar, PS"
"   Jeevanantham, S"
"   Saravanan, R"
"AF Yaashikaa, P. R."
"   Kumar, P. Senthil"
"   Jeevanantham, S."
"   Saravanan, R."
TI A review on bioremediation approach for heavy metal detoxification and
   accumulation in plants
SO ENVIRONMENTAL POLLUTION
LA English
DT Review
DE Phytoremediation; Heavy metals; Detoxification; Rhizosphere microflora;
   Transporters
ID GROWTH-PROMOTING RHIZOBACTERIA; ARSENIC TOXICITY; CADMIUM STRESS;
   ABIOTIC STRESS; CROP PLANTS; GENE FAMILY; PHYTOREMEDIATION; TOLERANCE;
   COPPER; ZINC
"AB Nowadays, the accumulation of toxic heavy metals in soil and water streams is considered a serious environmental problem that causes various harmful effects on plants and animals. Phytoremediation is an effective, green, and economical bioremediation approach by which the harmful heavy metals in the contaminated ecosystem can be detoxified and accumulated in the plant. Hyperaccumulators exude molecules called transporters that carry and translocate the heavy metals present in the soil to different plant parts. The hyper accumulator plant genes can confine higher concentrations of toxic heavy metals in their tissues. The efficiency of phytoremediation relies on various parameters such as soil properties (pH and soil type), organic matters in soil, heavy metal type, nature of rhizosphere, characteristics of rhizosphere microflora, etc. The present review comprehensively discusses the toxicity effect of heavy metals on the environment and different phytoremediation mechanisms for the transport and accumulation of heavy metals from polluted soil. This review gave comprehensive insights into plants tolerance for the higher heavy metal concentration their responses for heavy metal accumulation and the different mechanisms involved for heavy metal tolerance. The current status and the characteristic features that need to be improved in the phytoremediation process are also reviewed in detail."
"C1 [Yaashikaa, P. R.] SIMATS, Saveetha Sch Engn, Dept Biotechnol, Chennai 602105, Tamil Nadu, India."
"   [Kumar, P. Senthil] Sri Sivasubramaniya Nadar Coll Engn, Dept Chem Engn, Chennai 603110, Tamil Nadu, India."
"   [Kumar, P. Senthil] Sri Sivasubramaniya Nadar Coll Engn, Ctr Excellence Water Res CEWAR, Chennai 603110, Tamil Nadu, India."
"   [Jeevanantham, S.] Rajalakshmi Engn Coll, Dept Biotechnol, Chennai 602105, Tamil Nadu, India."
"   [Saravanan, R.] Univ Tarapaca, Dept Mech Engn, Arica, Chile."
C3 Saveetha Institute of Medical & Technical Science; Saveetha School of
   Engineering; SSN College of Engineering; SSN College of Engineering;
   Rajalakshmi Engineering College; Universidad de Tarapaca
"RP Kumar, PS (corresponding author), Sri Sivasubramaniya Nadar Coll Engn, Dept Chem Engn, Chennai 603110, Tamil Nadu, India."
EM senthilkumarp@ssn.edu.in
"RI Senthil Kumar, P/D-1869-2012; , P R Yaashikaa/AAI-2934-2021; Sathasivam,"
"   Jeevanantham/HGD-5615-2022; RAJENDRAN, Saravanan/J-3287-2014"
"OI Senthil Kumar, P/0000-0001-9389-5541; , P R"
"   Yaashikaa/0000-0002-3408-4447; Sathasivam,"
   Jeevanantham/0000-0003-1897-6792;
"CR AbdElgawad H, 2020, ENVIRON POLLUT, V258, DOI 10.1016/j.envpol.2019.113705"
"   Abdelkrim S, 2020, ECOTOX ENVIRON SAFE, V192, DOI 10.1016/j.ecoenv.2020.110260"
"   Adamowski M, 2015, PLANT CELL, V27, P20, DOI 10.1105/tpc.114.134874"
"   Adediran GA, 2015, J HAZARD MATER, V283, P490, DOI 10.1016/j.jhazmat.2014.09.064"
"   Afaj Adnan H., 2017, International Journal of Environmental Studies, V74, P119, DOI [10.1080/00207233.2016.1220723, 10.1080/00207233.2016.1220723]"
"   Ahemad M, 2019, ARAB J CHEM, V12, P1365, DOI 10.1016/j.arabjc.2014.11.020"
"   Ahmad MSA, 2011, REV ENVIRON CONTAM T, V214, P125, DOI 10.1007/978-1-4614-0668-6_6"
"   Akhtar Nahid, 2020, Biotechnology Reports, V26, pe00452, DOI [10.1016/j.btre.2020.e00452, 10.1016/j.btre.2020.e00452]"
"   Alaraidh IA, 2018, S AFR J BOT, V115, P90, DOI 10.1016/j.sajb.2018.01.012"
"   Alvarez A, 2017, CHEMOSPHERE, V166, P41, DOI 10.1016/j.chemosphere.2016.09.070"
"   Amm I, 2014, BBA-MOL CELL RES, V1843, P182, DOI 10.1016/j.bbamcr.2013.06.031"
"   Antoniadis V, 2017, EARTH-SCI REV, V171, P621, DOI 10.1016/j.earscirev.2017.06.005"
"   Armendariz AL, 2016, PLANT PHYSIOL BIOCH, V98, P119, DOI 10.1016/j.plaphy.2015.11.021"
"   Aryal M, 2015, INT J ENVIRON RES, V9, P1147"
"   Ashraf S, 2019, ECOTOX ENVIRON SAFE, V174, P714, DOI 10.1016/j.ecoenv.2019.02.068"
"   Azubuike CC, 2016, WORLD J MICROB BIOT, V32, DOI 10.1007/s11274-016-2137-x"

"   Balaji S, 2014, CLEAN-SOIL AIR WATER, V42, P1790, DOI 10.1002/clen.201300478"
"   Bali AS, 2020, ENVIRON CHEM LETT, V18, P1243, DOI 10.1007/s10311-020-01012-x"
"   Bashri G, 2015, ACTA PHYSIOL PLANT, V37, DOI 10.1007/s11738-014-1745-z"
"   Benatti MR, 2014, NEW PHYTOL, V202, P940, DOI 10.1111/nph.12718"
"   BRAHMBHATT N. H., 2016, INT J ENG RES GEN SC, V4, P443"
"   Broadhurst CL, 2004, ENVIRON SCI TECHNOL, V38, P5797, DOI 10.1021/es0493796"
"   Bucker-Neto L, 2017, GENET MOL BIOL, V40, P373, DOI [10.1590/1678-4685-GMB-2016-0087, 10.1590/1678-4685-gmb-2016-0087]"
"   Buer CS, 2010, J INTEGR PLANT BIOL, V52, P98, DOI 10.1111/j.1744-7909.2010.00905.x"
"   Cenkci S, 2010, ENVIRON EXP BOT, V67, P467, DOI 10.1016/j.envexpbot.2009.10.001"
"   Charfeddine M, 2017, PLANT CELL TISS ORG, V128, P521, DOI 10.1007/s11240-016-1130-2"
"   Chauhan R, 2020, J HAZARD MATER, V390, DOI 10.1016/j.jhazmat.2020.122122"
"   Chen SN, 2014, BIORESOURCE TECHNOL, V158, P181, DOI 10.1016/j.biortech.2014.02.020"
"   Chen YT, 2017, CURR OPIN PLANT BIOL, V39, P66, DOI 10.1016/j.pbi.2017.06.004"
"   Chibuike G.U., 2014, APPL ENVIRON SOIL SC, DOI [10.1155/2014/752708, DOI 10.1155/2014/752708]"
"   Claus J, 2012, PLOS ONE, V7, DOI 10.1371/journal.pone.0037193"
"   Clemens S, 2016, ANNU REV PLANT BIOL, V67, P489, DOI 10.1146/annurev-arplant-043015-112301"
"   Cristaldi A, 2020, ENVIRON RES, V185, DOI 10.1016/j.envres.2020.109427"
"   Cui XQ, 2021, J HAZARD MATER, V405, DOI 10.1016/j.jhazmat.2020.123832"
"   Cui ZJ, 2017, J ENVIRON CHEM ENG, V5, P3616, DOI 10.1016/j.jece.2017.07.021"
"   DalCorso G, 2013, METALLOMICS, V5, P1117, DOI 10.1039/c3mt00038a"
"   DalCorso G, 2010, PLANT SIGNAL BEHAV, V5, P663, DOI 10.4161/psb.5.6.11425"
"   de Souza LT, 2016, BRAGANTIA, V75, P19, DOI 10.1590/1678-4499.142"
"   Deng JC, 2020, BIORESOURCE TECHNOL, V303, DOI 10.1016/j.biortech.2020.122963"
"   Dhalaria R, 2020, AGRONOMY-BASEL, V10, DOI 10.3390/agronomy10060815"
"   Diaconu M, 2020, NEW BIOTECHNOL, V56, P130, DOI 10.1016/j.nbt.2020.01.003"
"   Dixit G, 2015, J HAZARD MATER, V298, P241, DOI 10.1016/j.jhazmat.2015.06.008"
"   Dobrowolski R, 2017, BIORESOURCE TECHNOL, V225, P113, DOI 10.1016/j.biortech.2016.11.040"
"   Ehsan S, 2014, ECOTOX ENVIRON SAFE, V106, P164, DOI 10.1016/j.ecoenv.2014.03.007"
"   Ertani A, 2017, WATER AIR SOIL POLL, V228, DOI 10.1007/s11270-017-3356-y"
"   Etesami H, 2018, ECOTOX ENVIRON SAFE, V147, P175, DOI 10.1016/j.ecoenv.2017.08.032"
"   Farooq MA, 2015, ENVIRON SCI POLLUT R, V22, P10699, DOI 10.1007/s11356-015-4269-1"
"   Farshian Shadi, 2007, Pak J Biol Sci, V10, P2363, DOI 10.3923/pjbs.2007.2363.2367"
"   Feng Z, 2021, TRAC-TREND ANAL CHEM, V143, DOI 10.1016/j.trac.2021.116402"
"   Finnegan PM, 2012, FRONT PHYSIOL, V3, DOI 10.3389/fphys.2012.00182"
"   Fuente V, 2016, J STRUCT BIOL, V193, P23, DOI 10.1016/j.jsb.2015.11.005"
"   Gall JE, 2015, ENVIRON MONIT ASSESS, V187, DOI 10.1007/s10661-015-4436-3"
"   Gangadhar BH, 2016, FRONT PLANT SCI, V7, DOI 10.3389/fpls.2016.01228"
"   Garg N., 2017, Mycorrhiza-Nutrient Uptake, Biocontrol, Ecorestoration, P75, DOI [10.1007/978-3-319-68867, DOI 10.1007/978-3-319-68867-1_4]"
"   Ghori NH, 2019, INT J ENVIRON SCI TE, V16, P1807, DOI 10.1007/s13762-019-02215-8"
"   Glavac NK, 2017, ARH HIG RADA TOKSIKO, V68, P236, DOI 10.1515/aiht-2017-68-2990"
"   Gong XM, 2019, SCI TOTAL ENVIRON, V666, P1126, DOI 10.1016/j.scitotenv.2019.02.215"
"   Guigues S, 2014, PLANT SOIL, V381, P367, DOI 10.1007/s11104-014-2138-1"
"   Guo JK, 2020, J ENVIRON SCI, V88, P361, DOI 10.1016/j.jes.2019.10.001"
"   Gupta M., 2019, GENOMICS ASSISTED BR, V2, DOI [10.1007/978-3-319-99573-1_2, DOI 10.1007/978-3-319-99573-1_2]"
"   Hall JL, 2002, J EXP BOT, V53, P1, DOI 10.1093/jexbot/53.366.1"
"   Harada E, 2010, PLANT CELL PHYSIOL, V51, P1627, DOI 10.1093/pcp/pcq118"
"   Hasan MK, 2015, FRONT PLANT SCI, V6, DOI 10.3389/fpls.2015.00601"
"   Hayat S, 2013, ACTA BOT CROAT, V72, P323, DOI 10.2478/v10184-012-0019-3"
"   He CQ, 2020, CHEMOSPHERE, V252, DOI 10.1016/j.chemosphere.2020.126471"
"   Hejna M, 2020, CHEMOSPHERE, V241, DOI 10.1016/j.chemosphere.2019.125018"
"   Hooper AM, 2010, PHYTOCHEMISTRY, V71, P904, DOI 10.1016/j.phytochem.2010.02.015"
"   Htwe T, 2020, COMMUN SOIL SCI PLAN, V51, P2349, DOI 10.1080/00103624.2020.1836200"
"   Huang HG, 2011, BIORESOURCE TECHNOL, V102, P11034, DOI 10.1016/j.biortech.2011.09.067"
"   Ibrahim N, 2020, HELIYON, V6, DOI 10.1016/j.heliyon.2020.e04445"
"   Imtiaz M, 2016, J ENVIRON MANAGE, V183, P521, DOI 10.1016/j.jenvman.2016.09.009"
"   Jaafari J, 2019, CHEMOSPHERE, V217, P447, DOI 10.1016/j.chemosphere.2018.10.205"
"   Jeevanantham S, 2019, ENVIRON TECHNOL INNO, V13, P264, DOI 10.1016/j.eti.2018.12.007"
"   Jin CX, 2015, SOIL SEDIMENT CONTAM, V24, P454, DOI 10.1080/15320383.2015.981648"
"   Johnsen IV, 2019, SCI TOTAL ENVIRON, V687, P41, DOI 10.1016/j.scitotenv.2019.06.086"
"   Jung MC, 2008, SENSORS-BASEL, V8, P2413, DOI 10.3390/s8042413"
"   Kamran MA, 2016, ECOTOX ENVIRON SAFE, V126, P256, DOI 10.1016/j.ecoenv.2016.01.002"
"   Kerkeb L, 2003, PLANT PHYSIOL, V131, P716, DOI 10.1104/pp102.010686"
"   Khalid S, 2017, J GEOCHEM EXPLOR, V182, P247, DOI 10.1016/j.gexplo.2016.11.021"
"   Khan I, 2019, INT J ENVIRON SCI TE, V16, P6473, DOI 10.1007/s13762-019-02431-2"
"   Khan I, 2019, ENVIRON MONIT ASSESS, V191, DOI 10.1007/s10661-019-7781-9"
"   Khan I, 2021, ECOTOX ENVIRON SAFE, V222, DOI 10.1016/j.ecoenv.2021.112510"
"   Khanthom S, 2021, ENVIRON TECHNOL INNO, V22, DOI 10.1016/j.eti.2021.101419"
"   Kolaj-Robin O, 2015, FEBS LETT, V589, P1283, DOI 10.1016/j.febslet.2015.04.007"
"   Krzeslowska M, 2011, ACTA PHYSIOL PLANT, V33, P35, DOI 10.1007/s11738-010-0581-z"
"   Kumar A., 2021, HDB BIOREMEDIATION, P23, DOI [DOI 10.1016/B978-0-12-819382-2.00003-X, 10.1016/B978-0-12-819382-2.00003-X]"
"   Kumar A, 2019, FUNG BIOL-US, P437, DOI 10.1007/978-3-030-25506-0_18"
"   Kumar B, 2017, ARAB J CHEM, V10, pS2335, DOI 10.1016/j.arabjc.2013.08.010"
"   Kumari S, 2019, ENVIRON SCI POLLUT R, V26, P28763, DOI 10.1007/s11356-019-05916-2"
"   Leitenmaier B, 2013, FRONT PLANT SCI, V4, DOI 10.3389/fpls.2013.00374"
"   Lin YF, 2012, CELL MOL LIFE SCI, V69, P3187, DOI 10.1007/s00018-012-1089-z"
"   Ling T., 2010, Research Journal of Phytochemistry, V4, P225"
"   Liu CJ, 2021, CHEMOSPHERE, V272, DOI 10.1016/j.chemosphere.2021.129816"
"   Liu CJ, 2020, ECOTOX ENVIRON SAFE, V202, DOI 10.1016/j.ecoenv.2020.110958"
"   Liu J, 2015, J HAZARD MATER, V294, P99, DOI 10.1016/j.jhazmat.2015.03.060"
"   Liu JX, 2016, NEW PHYTOL, V211, P418, DOI 10.1111/nph.13915"

"   Liu XS, 2019, BMC PLANT BIOL, V19, DOI 10.1186/s12870-019-1899-3"
"   Logro??o W, 2017, CHEMOSPHERE, V176, P378, DOI 10.1016/j.chemosphere.2017.02.099"
"   Maalej H, 2015, J APPL MICROBIOL, V118, P356, DOI 10.1111/jam.12688"
"   Maestri E., 2012, METAL TOXICITY PLANT, P41, DOI DOI 10.1007/978-3-642-22081-4_3"
"   Manara A., 2012, Plants and heavy metals, SpringerBriefs in Biometals, P27"
"   Mani D, 2014, INT J ENVIRON SCI TE, V11, P843, DOI 10.1007/s13762-013-0299-8"
"   Manoj SR, 2020, J ENVIRON MANAGE, V254, DOI 10.1016/j.jenvman.2019.109779"
"   Maron LG, 2010, PLANT J, V61, P728, DOI 10.1111/j.1365-313X.2009.04103.x"
"   Masood F, 2015, SEP SCI TECHNOL, V50, P892, DOI 10.1080/01496395.2014.969378"
"   Memon A.R., 2016, Phytoremediat.: Manag. Environ. Contam., V3, P239, DOI [DOI 10.1007/978-3-319-40148-58, 10.1007/]"
"   Mesa J, 2015, J HAZARD MATER, V300, P263, DOI 10.1016/j.jhazmat.2015.07.006"
"   Migeon A, 2010, CELL MOL LIFE SCI, V67, P3763, DOI 10.1007/s00018-010-0445-0"
"   Mirshekali H., 2012, International Journal of Agriculture: Research and Review, V2, P247"
"   Mishra D, 2018, HEAT SHOCK PROTEINS, V13, P41, DOI 10.1007/978-3-319-74715-6_3"
"   Mondal NK, 2015, ENVIRON MONIT ASSESS, V187, DOI 10.1007/s10661-015-4484-8"
"   Morsy A.A., 2012, Eurasian Journal of Biosciences, V6, P1, DOI DOI 10.5053/EJOBIOS.2012.6.0.1"
"   Mwamba TM, 2016, INT J ENVIRON SCI TE, V13, P2163, DOI 10.1007/s13762-016-1040-1"
"   Naghipour D, 2018, DATA BRIEF, V21, P1409, DOI 10.1016/j.dib.2018.10.111"
"   Niu H, 2021, CHEMOSPHERE, V269, DOI 10.1016/j.chemosphere.2020.128765"
"   Noctor G, 2013, PLANT PHYSIOL, V163, P665, DOI 10.1104/pp.113.223545"
"   O'Sullivan CA, 2019, AGR SYST, V174, P133, DOI 10.1016/j.agsy.2019.05.007"
"   Osmolovskaya Natalia, 2018, Biological Communications, V63, P9, DOI [10.21638/spbu03.2018.103, 10.21638/spbu03.2018.103]"
"   Page V, 2015, AGRONOMY-BASEL, V5, P447, DOI 10.3390/agronomy5030447"
"   Pasricha S., 2021, ENV CHALL, V4, P100197, DOI [10.1016/j.envc.2021.100197, DOI 10.1016/J.ENVC.2021.100197]"
"   Patra DK, 2020, ENVIRON TECHNOL INNO, V18, DOI 10.1016/j.eti.2020.100672"
"   Patra DK, 2019, CHEMOSPHERE, V218, P1082, DOI 10.1016/j.chemosphere.2018.11.211"
"   Pochodylo AL, 2017, ENVIRON CHEM LETT, V15, P495, DOI 10.1007/s10311-017-0609-3"
"   Pramanik K, 2018, J HAZARD MATER, V351, P317, DOI 10.1016/j.jhazmat.2018.03.009"
"   Rai KK, 2021, ECOTOX ENVIRON SAFE, V208, DOI 10.1016/j.ecoenv.2020.111750"
"   Rai PK, 2020, SCI TOTAL ENVIRON, V705, DOI 10.1016/j.scitotenv.2019.135858"
"   Raklami A, 2021, J ENVIRON SCI, V99, P210, DOI 10.1016/j.jes.2020.06.032"
"   Rascio N, 2011, PLANT SCI, V180, P169, DOI 10.1016/j.plantsci.2010.08.016"
"   Ravanbakhsh M, 2019, J HAZARD MATER, V379, DOI 10.1016/j.jhazmat.2019.120787"
"   Reeves RD, 2018, NEW PHYTOL, V218, P407, DOI 10.1111/nph.14907"
"   Rehman AU, 2021, J MOL LIQ, V321, DOI 10.1016/j.molliq.2020.114455"
"   Ricachenevsky FK, 2013, FRONT PLANT SCI, V4, DOI 10.3389/fpls.2013.00144"
"   Saavedra R, 2018, BIORESOURCE TECHNOL, V263, P49, DOI 10.1016/j.biortech.2018.04.101"
"   S??cky J, 2014, FUNGAL GENET BIOL, V67, P3, DOI 10.1016/j.fgb.2014.03.003"
"   Sakakibara M, 2011, CLEAN-SOIL AIR WATER, V39, P735, DOI 10.1002/clen.201000488"
"   Sasaki A, 2012, PLANT CELL, V24, P2155, DOI 10.1105/tpc.112.096925"
"   Sassykova LR, 2020, MATER TODAY-PROC, V33, P1187, DOI 10.1016/j.matpr.2020.07.660"
"   Sati M., 2014, International Journal of Advanced Research, V2, P923"
"   Shah V, 2020, ENVIRON TECHNOL INNO, V18, DOI 10.1016/j.eti.2020.100774"
"   Sharma J, 2017, SCI TOTAL ENVIRON, V579, P359, DOI 10.1016/j.scitotenv.2016.11.089"
"   Sharma P, 2021, ENVIRON TECHNOL INNO, V23, DOI 10.1016/j.eti.2021.101725"
"   Sharma SS, 2016, PLANT CELL ENVIRON, V39, P1112, DOI 10.1111/pce.12706"
"   Shen ZJ, 2017, ECOTOX ENVIRON SAFE, V143, P19, DOI 10.1016/j.ecoenv.2017.04.042"
"   Shrivastava M., 2019, PLANT METAL INTERACT, P79, DOI [10.1007/978-3-030-20732-8_5, DOI 10.1007/978-3-030-20732-8_5, https://doi.org/10.1007/978-3-030-20732-8_5]"
"   Singh HP, 2013, ENVIRON CHEM LETT, V11, P229, DOI 10.1007/s10311-013-0407-5"
"   Singh JS, 2019, ENVIRON SUSTAIN IND, V3-4, DOI 10.1016/j.indic.2019.100008"
"   Singh RK, 2016, SCI REP-UK, V6, DOI 10.1038/srep32641"
"   Singh S, 2016, FRONT PLANT SCI, V6, DOI 10.3389/fpls.2015.01143"
"   Singh VK, 2018, AGR ECOSYST ENVIRON, V267, P129, DOI 10.1016/j.agee.2018.08.020"
"   Sobariu DL, 2017, NEW BIOTECHNOL, V39, P125, DOI 10.1016/j.nbt.2016.09.002"
"   Sreekanth TVM, 2013, INT J ENVIRON SCI TE, V10, P1129, DOI 10.1007/s13762-013-0245-9"
"   Su ZL, 2019, INT J AGR BIOL ENG, V12, P147, DOI 10.25165/j.ijabe.20191203.4403"
"   Sun RL, 2007, ENVIRON EXP BOT, V60, P468, DOI 10.1016/j.envexpbot.2007.01.004"
"   Sun Z, 2016, ECOL ENG, V86, P60, DOI 10.1016/j.ecoleng.2015.10.023"
"   Talukdar D, 2020, ENVIRON TECHNOL INNO, V20, DOI 10.1016/j.eti.2020.101050"
"   Tangahu BV, 2011, INT J CHEM ENG, V2011, DOI 10.1155/2011/939161"
"   Tirry N., 2018, Journal of Genetic Engineering and Biotechnology, V16, P613, DOI [10.1016/j.jgeb.2018.06.004, 10.1016/j.jgeb.2018.06.004]"
"   Tiwari J, 2019, PHYTOMANAGEMENT OF POLLUTED SITES: MARKET OPPORTUNITIES IN SUSTAINABLE PHYTOREMEDIATION, P179, DOI 10.1016/B978-0-12-813912-7.00005-3"
"   Tkalec M, 2014, PLOS ONE, V9, DOI 10.1371/journal.pone.0087582"
"   Tran Lam-Son Phan, 2010, GM Crops, V1, P32, DOI 10.4161/gmcr.1.1.10569"
"   Upadhyay A, 2017, FRONT MICROBIOL, V8, DOI 10.3389/fmicb.2017.00284"
"   Verma S, 2019, ENVIRON TECHNOL INNO, V14, DOI 10.1016/j.eti.2019.100369"
"   Villiers F, 2011, PROTEOMICS, V11, P1650, DOI 10.1002/pmic.201000645"
"   Volland S, 2013, CHEMOSPHERE, V91, P448, DOI 10.1016/j.chemosphere.2012.11.064"
"   Wakeel A, 2020, INT J MOL SCI, V21, DOI 10.3390/ijms21030728"
"   Wang LW, 2020, CRIT REV ENV SCI TEC, V50, P2724, DOI 10.1080/10643389.2019.1705724"
"   Wang SQ, 2019, SCI TOTAL ENVIRON, V654, P1023, DOI 10.1016/j.scitotenv.2018.11.032"
"   Wang Y, 2015, SCI REP-UK, V5, DOI 10.1038/srep18296"
"   Wang Z, 2017, J HAZARD MATER, V328, P108, DOI 10.1016/j.jhazmat.2016.12.059"
"   Wei CY, 2015, ENVIRON EXP BOT, V109, P31, DOI 10.1016/j.envexpbot.2014.08.002"
"   Wood JL, 2016, SOIL BIOL BIOCHEM, V103, P131, DOI 10.1016/j.soilbio.2016.08.021"
"   Xiang QJ, 2013, GENE, V522, P133, DOI 10.1016/j.gene.2013.03.069"
"   Xiao AW, 2020, CHEMOSPHERE, V242, DOI 10.1016/j.chemosphere.2019.125136"
"   Xin JP, 2020, ECOTOX ENVIRON SAFE, V203, DOI 10.1016/j.ecoenv.2020.111024"

"   Yamaji N, 2013, PLANT PHYSIOL, V162, P927, DOI 10.1104/pp.113.216564"
"   Ying MC, 2019, ENVIRON EXP BOT, V165, P53, DOI 10.1016/j.envexpbot.2019.05.009"
"   Yuan HM, 2013, PLANT SIGNAL BEHAV, V8, DOI 10.4161/psb.24671"
"   Yuan M, 2014, J INTEGR PLANT BIOL, V56, P559, DOI 10.1111/jipb.12173"
"   Yuan M, 2011, BMC PLANT BIOL, V11, DOI 10.1186/1471-2229-11-69"
"   Zhang J, 2017, FEBS LETT, V591, P656, DOI 10.1002/1873-3468.12576"
"   Zhao FJ, 2000, PLANT CELL ENVIRON, V23, P507, DOI 10.1046/j.1365-3040.2000.00569.x"
"   Zhu J, 2012, CHEM ENG J, V179, P26, DOI 10.1016/j.cej.2011.07.011"
NR 182
TC 311
Z9 359
U1 56
U2 1487
PU ELSEVIER SCI LTD
PI London
"PA 125 London Wall, London, the United Kingdom"
SN 0269-7491
EI 1873-6424
J9 ENVIRON POLLUT
JI Environ. Pollut.
PD MAY 15
PY 2022
VL 301
AR 119035
DI 10.1016/j.envpol.2022.119035
EA FEB 2022
PG 15
WC Environmental Sciences
WE Science Citation Index Expanded (SCI-EXPANDED)
SC Environmental Sciences & Ecology
GA 0W9PK
UT WOS:000789350100001
PM 35196562
DA 2026-04-06
ER

PT J
"AU Karmakar, V"
"   Kar, A"
"   Banerjee, A"
"   Sengupta, P"
"   Dutta, S"
"   Gorain, B"
"AF Karmakar, Varnita"
"   Kar, Arijit"
"   Banerjee, Adrija"
"   Sengupta, Pallav"
"   Dutta, Sulagna"
"   Gorain, Bapi"
TI BIOREMEDIATION AND DETOXIFICATION STRATEGIES USING MICROORGANISMS:
   MOLECULAR ASPECT TO COMBAT HEAVY METAL CONTAMINATION
SO ENVIRONMENTAL ENGINEERING AND MANAGEMENT JOURNAL
LA English
DT Article
DE bioremediation; detoxification; heavy metals; microorganisms; molecular
   mechanisms
ID PSEUDOMONAS-PUTIDA; AQUEOUS-SOLUTIONS; CADMIUM UPTAKE; BIOSORPTION;
   RESISTANCE; BACTERIA; COPPER; TOLERANCE; STRAIN; ZINC
"AB The increased contamination of air, soil, and water resources with heavy metals around us is due to the industrial revolution and other anthropogenic activities, which create serious health problems. Remediation of such non-biodegradable heavy metals from the sources is of utmost importance considering their persisting nature in the environment and simultaneous toxic effects on living systems. The removal of such harmful elements from contaminated environments using microorganisms could help in cleaning polluted ecosystems. Bioremediation involves the interaction of the contaminated environment containing heavy metals with biologically active microbes and the elimination of heavy metals through different mechanisms, such as adsorption, complexation, precipitation, methylation, etc. These living microorganisms have opened up avenues to detoxify heavy metals through several biological processes including active efflux or protein sequestration, bio-stimulation, and biosorption. An overview of the impact of heavy metals on human health, aquatic life, plants, or soil and the mechanism of detoxification of heavy metals by various microorganisms is presented in this article. The study elucidates how microbial enzymes catalyze the conversion of toxic metalions, offering a molecular perspective on bioremediation. Through genetic adaptations, microbes develop specialized mechanismsto sequester or expel heavy metals, enhancing possibilities in removing contamination. This comprehensive understanding underscores the importance of leveraging bioremediation to mitigate health risks associated with heavy metal exposure, necessitating further research and application in contaminated environments."
"C1 [Karmakar, Varnita; Gorain, Bapi] Birla Inst Technol, Dept Pharmaceut Sci & Technol, Ranchi 835215, Jharkhand, India."
"   [Kar, Arijit; Banerjee, Adrija] Eminent Coll Pharmaceut Technol, Dept Pharmacol, Barasat 700126, West Bengal, India."
"   [Sengupta, Pallav] Gulf Med Univ, Coll Med, Dept Biomed Sci, Ajman, U Arab Emirates."
"   [Dutta, Sulagna] Bharath Inst Higher Educ & Res BIHER, Sch Med Sci, Chennai, Tamil Nadu, India."
C3 Birla Institute of Technology Mesra; Bharath Institute of Higher
   Education & Research

"RP Gorain, B (corresponding author), Birla Inst Technol, Dept Pharmaceut Sci & Technol, Ranchi 835215, Jharkhand, India."
EM bapi.gn@gmail.com
"RI Sengupta, Pallav/E-3392-2016; Gorain, Bapi/M-9706-2017; Dutta,"
Sulagna/W-5151-2017
"OI Sengupta, Pallav/0000-0002-1928-5048; "
"CR Abo-Alkasem MI, 2023, B NATL RES CENT, V47, DOI [10.1186/s42269-023-01006-z, 10.1186/s42269-023-01006-z, DOI 10.1186/S42269-023-01006-Z]"
" Ackerley DF, 2006, J BACTERIOL, V188, P3371, DOI 10.1128/JB.188.9.3371-3381.2006"
" Al-Dhabi NA, 2019, INT J ENV RES PUB HE, V16, DOI 10.3390/ijerph16234788"
" Alharbi RM, 2023, TOXICS, V11, DOI 10.3390/toxics11050439"
" Ali H, 2019, J CHEM-NY, V2019, DOI 10.1155/2019/6730305"
" Redha AA, 2020, Arab Journal of Basic and Applied Sciences, V27, P183, DOI [10.1080/25765299.2020.1756177, 10.1080/25765299.2020.1756177, DOI 10.1080/25765299.2020.1756177]"
" Apandi NM, 2022, SUSTAINABILITY-BASEL, V14, DOI 10.3390/su14095090"
" Ares A, 2022, ENVIRON MICROBIOL, V24, P6144, DOI 10.1111/1462-2920.16255"
" Azevedo BF, 2012, J BIOMED BIOTECHNOL, DOI 10.1155/2012/949048"
" Babich H, 1978, Adv Appl Microbiol, V23, P55, DOI 10.1016/S0065-2164(08)70065-0"
" Bala S, 2022, TOXICS, V10, DOI 10.3390/toxics10080484"
" Balali-Mood M, 2021, FRONT PHARMACOL, V12, DOI 10.3389/fphar.2021.643972"
" Baldrian P, 2002, FEMS MICROBIOL LETT, V206, P69, DOI 10.1111/j.1574-6968.2002.tb10988.x"
" Barkay T, 2003, FEMS MICROBIOL REV, V27, P355, DOI 10.1016/S0168-6445(03)00046-9"
" Bazylinski DA, 2007, GEOMICROBIOL J, V24, P465, DOI 10.1080/01490450701572259"
" Ben Fekih I, 2018, FRONT MICROBIOL, V9, DOI 10.3389/fmicb.2018.02473"
" BEVERIDGE TJ, 1985, CAN J EARTH SCI, V22, P1893, DOI 10.1139/e85-204"
" Bhalerao TS, 2007, INT BIODETER BIODEGR, V59, P315, DOI 10.1016/j.ibiod.2006.09.002"
" Bhaskar PV, 2006, ENVIRON INT, V32, P191, DOI 10.1016/j.envint.2005.08.010"
" Bissen M, 2003, ACTA HYDROCH HYDROB, V31, P9, DOI 10.1002/aheh.200390025"
" Blaga AC, 2021, POLYMERS-BASEL, V13, DOI 10.3390/polym13172893"
" Blaudez D, 2000, MICROBIOL-SGM, V146, P1109, DOI 10.1099/00221287-146-5-1109"
" Bodor A, 2020, INT J ENV RES PUB HE, V17, DOI 10.3390/ijerph17114106"
" Boyd SM, 2022, ANTIBIOTICS-BASEL, V11, DOI 10.3390/antibiotics11060711"
" Bravo G, 2020, MICROORGANISMS, V8, DOI 10.3390/microorganisms8121952"
" Broussard LA, 2002, LAB MED, V33, P614"
" Bruins MR, 2000, ECOTOX ENVIRON SAFE, V45, P198, DOI 10.1006/eesa.1999.1860"
" C??novas D, 2003, ENVIRON MICROBIOL, V5, P1242, DOI [10.1111/j.1462-2920.2003.00463.x, 10.1046/j.1462-2920.2003.00463.x]"
" Cavalca L, 2000, RES MICROBIOL, V151, P877, DOI 10.1016/S0923-2508(00)01155-4"
" Cervantes C, 2001, FEMS MICROBIOL REV, V25, P335, DOI 10.1111/j.1574-6976.2001.tb00581.x"
" Chandrashekharaiah PS, 2022, RESOUR CONSERV RECY, V180, DOI 10.1016/j.resconrec.2022.106211"
" Chen X., 2021, Advances, V11, P17080, DOI [10.1039/D0RA09744F, DOI 10.1039/D0RA09744F]"
" Chen XC, 2005, COLLOID SURFACE B, V46, P101, DOI 10.1016/j.colsurfb.2005.10.003"
" Clarkson TW, 2006, CRIT REV TOXICOL, V36, P609, DOI 10.1080/10408440600845619"
" Darma A, 2022, BIOLOGY-BASEL, V11, DOI 10.3390/biology11030472"
" Das S, 2016, APPL MICROBIOL BIOT, V100, P2967, DOI 10.1007/s00253-016-7364-4"
" Davis TA, 2003, WATER RES, V37, P4311, DOI 10.1016/S0043-1354(03)00293-8"
" Dhankhar R, 2011, ENVIRON TECHNOL, V32, P467, DOI 10.1080/09593330.2011.572922"
" Diep P, 2018, FRONT BIOENG BIOTECH, V6, DOI 10.3389/fbioe.2018.00157"
" Ding CL, 2022, FRONT ENV SCI-SWITZ, V10, DOI 10.3389/fenvs.2022.920957"
" Doroszkiewicz J, 2023, INT J MOL SCI, V24, DOI 10.3390/ijms242115721"
" Dupraz C, 2009, EARTH-SCI REV, V96, P141, DOI 10.1016/j.earscirev.2008.10.005"
" Dutta S, 2022, ENVIRON SCI POLLUT R, V29, P62067, DOI 10.1007/s11356-021-16581-9"
" Eggleton J, 2004, ENVIRON INT, V30, P973, DOI 10.1016/j.envint.2004.03.001"
" El-Bondkly AMA, 2022, HELIYON, V8, DOI 10.1016/j.heliyon.2022.e09854"
" Esfandiar N, 2022, J HAZARD MATER, V423, DOI 10.1016/j.jhazmat.2021.126938"
" Fashola MO, 2016, INT J ENV RES PUB HE, V13, DOI 10.3390/ijerph13111047"
" Fomina M, 2014, BIORESOURCE TECHNOL, V160, P3, DOI 10.1016/j.biortech.2013.12.102"
" Frankel RB, 2003, REV MINERAL GEOCHEM, V54, P95, DOI 10.2113/0540095"
" Gadd GM, 2012, ENV MICROBIOL REP, V4, P270, DOI 10.1111/j.1758-2229.2011.00283.x"
" Gadd GM, 2009, J CHEM TECHNOL BIOT, V84, P13, DOI 10.1002/jctb.1999"
" Gao MZ, 2024, FRONT MICROBIOL, V15, DOI 10.3389/fmicb.2024.1374275"
" Genchi G, 2020, INT J ENV RES PUB HE, V17, DOI 10.3390/ijerph17113782"
" Henao SG, 2021, FRONT ENV SCI-SWITZ, V9, DOI 10.3389/fenvs.2021.604216"
" Green-Ruiz C, 2006, BIORESOURCE TECHNOL, V97, P1907, DOI 10.1016/j.biortech.2005.08.014"
" Grennan AK, 2011, PLANT PHYSIOL, V155, P1750, DOI 10.1104/pp.111.900407"
" Grotz N, 2006, BBA-MOL CELL RES, V1763, P595, DOI 10.1016/j.bbamcr.2006.05.014"
" Guin?? V, 2007, CHEM GEOL, V236, P266, DOI 10.1016/j.chemgeo.2006.10.001"
" Gupta Pratima, 2017, Biotechnol Rep (Amst), V13, P58, DOI [10.1016/j.btre.2016.12.006, 10.1016/j.btre.2016.12.006]"
" Lima AIG, 2006, ENZYME MICROB TECH, V39, P763, DOI 10.1016/j.enzmictec.2005.12.009"
" Gworek B, 2016, WATER AIR SOIL POLL, V227, DOI 10.1007/s11270-016-3060-3"
" Hassan A, 2019, WATER AIR SOIL POLL, V230, DOI 10.1007/s11270-019-4227-5"
" Hawkes SJ, 1997, J CHEM EDUC, V74, P1374, DOI 10.1021/ed074p1374"
" Hetzer A, 2006, APPL ENVIRON MICROB, V72, P4020, DOI 10.1128/AEM.00295-06"
" HIGHAM DP, 1986, ENVIRON HEALTH PERSP, V65, P5, DOI 10.2307/3430155"
" Hlihor RM, 2009, ENVIRON ENG MANAG J, V8, P1199"
" Huang H, 2020, HIGH-RISK POLLUTANTS IN WASTEWATER, P209, DOI 10.1016/B978-0-12-816448-8.00009-5"
" Hussain S, 2022, FRONT MICROBIOL, V13, DOI 10.3389/fmicb.2022.900740"
" Igiri BE, 2018, J TOXICOL-US, V2018, DOI 10.1155/2018/2568038"
" Ansari MI, 2007, BIORESOURCE TECHNOL, V98, P3149, DOI 10.1016/j.biortech.2006.10.008"
" Iyer A, 2005, MAR POLLUT BULL, V50, P340, DOI 10.1016/j.marpolbul.2004.11.012"
" Jaishankar Monisha, 2014, Interdiscip Toxicol, V7, P60, DOI 10.2478/intox-2014-0009"
" J??rup L, 2003, BRIT MED BULL, V68, P167, DOI 10.1093/bmb/ldg032"
" Kadukov?? J, 2005, ENVIRON INT, V31, P227, DOI 10.1016/j.envint.2004.09.020"

"   Kang CH, 2016, ECOL ENG, V89, P64, DOI 10.1016/j.ecoleng.2016.01.023"
"   Kapoor A, 1998, BIORESOURCE TECHNOL, V63, P109, DOI 10.1016/S0960-8524(97)00118-1"
"   Kazy SK, 2002, WORLD J MICROB BIOT, V18, P583, DOI 10.1023/A:1016354713289"
"   Kemner KM, 2004, SCIENCE, V306, P686, DOI 10.1126/science.1103524"
"   KUYUCAK N, 1988, BIOTECHNOL LETT, V10, P137, DOI 10.1007/BF01024641"
"   Lecomte SM, 2018, FRONT ENV SCI-SWITZ, V6, DOI 10.3389/fenvs.2018.00139"
"   Lee JU, 2005, ENVIRON GEOCHEM HLTH, V27, P159, DOI 10.1007/s10653-005-0133-4"
"   Lee SW, 2001, APPL ENVIRON MICROB, V67, P1437, DOI 10.1128/AEM.67.4.1437-1444.2001"
"   Leonard J, 2022, GEOMICROBIOL J, V39, P328, DOI 10.1080/01490451.2021.1998257"
"   Li QR, 2023, BMC MICROBIOL, V23, DOI 10.1186/s12866-023-02795-z"
"   Li XZ, 1997, J BACTERIOL, V179, P6127, DOI 10.1128/JB.179.19.6127-6132.1997"
"   Liang G, 2019, INT J ENV RES PUB HE, V16, DOI 10.3390/ijerph16060909"
"   Liaqat I, 2023, MOL BIOL REP, V50, P3985, DOI 10.1007/s11033-023-08334-3"
"   Liu HZ, 2021, INT J MOL SCI, V22, DOI 10.3390/ijms22189697"
"   Lopez-Fernandez M, 2021, MICROB BIOTECHNOL, V14, P810, DOI 10.1111/1751-7915.13718"
"   Luptakova A, 2005, HYDROMETALLURGY, V77, P97, DOI 10.1016/j.hydromet.2004.10.019"
"   Ma Q, 2019, INT J ENV RES PUB HE, V16, DOI 10.3390/ijerph16112047"
"   Ma Y., 2022, Advances in Microbe-assisted Phytoremediation Polluted Sites., P103, DOI [10.1016/B978-0-12-823443-3.00012-0, DOI 10.1016/B978-0-12-823443-3.00012-0]"
"   Mandal BK, 2002, TALANTA, V58, P201, DOI 10.1016/S0039-9140(02)00268-0"
"   Mathivanan K, 2021, ECOTOX ENVIRON SAFE, V226, DOI 10.1016/j.ecoenv.2021.112863"
"   Medina-Bellver JI, 2005, ENVIRON MICROBIOL, V7, P773, DOI 10.1111/j.1462-2920.2005.00742.x"
"   Mergeay M, 2003, FEMS MICROBIOL REV, V27, P385, DOI 10.1016/S0168-6445(03)00045-7"
"   MERGEAY M, 1985, J BACTERIOL, V162, P328, DOI 10.1128/JB.162.1.328-334.1985"
"   Michalak I, 2013, APPL BIOCHEM BIOTECH, V170, P1389, DOI 10.1007/s12010-013-0269-0"
"   Mire CE, 2004, APPL ENVIRON MICROB, V70, P855, DOI 10.1128/AEM.70.2.855-864.2004"
"   Mishra A, 2013, CRIT REV ENV SCI TEC, V43, P1162, DOI 10.1080/10934529.2011.627044"
"   MITRA RS, 1978, J BACTERIOL, V133, P75, DOI 10.1128/JB.133.1.75-80.1978"
"   Mitra S, 2022, J KING SAUD UNIV SCI, V34, DOI 10.1016/j.jksus.2022.101865"
"   Mohammadi-Moghadam F, 2022, INT J CHEM ENG, V2022, DOI 10.1155/2022/5294170"
"   Nadzir MM, 2021, POLYMERS-BASEL, V13, DOI 10.3390/polym13040530"
"   Mohseni M, 2018, ECOTOX ENVIRON SAFE, V148, P555, DOI 10.1016/j.ecoenv.2017.11.002"
"   Morcillo RJL, 2021, METABOLITES, V11, DOI 10.3390/metabo11060337"
"   Muthu M, 2017, POLYMERS-BASEL, V9, DOI 10.3390/polym9120721"
"   Nagvenkar GS, 2010, ECOTOXICOLOGY, V19, P604, DOI 10.1007/s10646-009-0429-8"
"   Neethu CS, 2015, CAN J MICROBIOL, V61, P429, DOI 10.1139/cjm-2014-0803"
"   NELSON MJ, 1990, ENVIRON PROG, V9, P190, DOI 10.1002/ep.670090322"
"   Nies DH, 2003, FEMS MICROBIOL REV, V27, P313, DOI 10.1016/S0168-6445(03)00048-2"
"   Nies DH, 1999, APPL MICROBIOL BIOT, V51, P730, DOI 10.1007/s002530051457"
"   Norton L, 2004, ADV ENVIRON RES, V8, P629, DOI 10.1016/S1093-0191(03)00035-2"
"   ODERMATT A, 1993, J BIOL CHEM, V268, P12775"
"   Ojuederie OB, 2017, INT J ENV RES PUB HE, V14, DOI 10.3390/ijerph14121504"
"   Oyewole OA, 2019, SN APPL SCI, V1, DOI 10.1007/s42452-019-0879-4"
"   Padgaonkar Amruta, 2022, Mediterranean Agricultural Sciences, V35, P135, DOI [10.29136/mediterranean.1097816, 10.29136/mediterranean.1097816]"
"   Pande V, 2022, FRONT MICROBIOL, V13, DOI 10.3389/fmicb.2022.824084"
"   Pardo R, 2003, ANAL BIOANAL CHEM, V376, P26, DOI 10.1007/s00216-003-1843-z"
"   Partridge SR, 2018, CLIN MICROBIOL REV, V31, DOI 10.1128/CMR.00088-17"
"   Perron K, 2004, J BIOL CHEM, V279, P8761, DOI 10.1074/jbc.M312080200"
"   Pinto E, 2003, J PHYCOL, V39, P1008, DOI 10.1111/j.0022-3646.2003.02-193.x"
"   Presentato A, 2020, MICROORGANISMS, V8, DOI 10.3390/microorganisms8122027"
"   Priya AK, 2022, CHEMOSPHERE, V307, DOI 10.1016/j.chemosphere.2022.135957"
"   Purwanti IF, 2020, HELIYON, V6, DOI 10.1016/j.heliyon.2020.e05004"
"   Qin W, 2020, ADV MATER, V32, DOI 10.1002/adma.201907833"
"   Raff J.D., 2020, Elements of Environmental Chemistry, V3rd"
"   Raklami A, 2022, INT J MOL SCI, V23, DOI 10.3390/ijms23095031"
"   Rasheed PA, 2019, CURR OPIN CHEM ENG, V25, P35, DOI 10.1016/j.coche.2019.06.003"
"   Rashid A, 2023, AGRONOMY-BASEL, V13, DOI 10.3390/agronomy13061521"
"   Rensing C, 1999, J BACTERIOL, V181, P5891, DOI 10.1128/JB.181.19.5891-5897.1999"
"   Richau JA, 1997, RES MICROBIOL, V148, P251, DOI 10.1016/S0923-2508(97)85245-X"
"   Rickard D, 2007, CHEM REV, V107, P514, DOI 10.1021/cr0503658"
"   Saravanan A, 2022, CHEMOSPHERE, V298, DOI 10.1016/j.chemosphere.2022.134341"
"   Sauge-Merle S, 2012, CHEMOSPHERE, V88, P918, DOI 10.1016/j.chemosphere.2012.04.015"
"   SCOTT JA, 1990, APPL MICROBIOL BIOT, V33, P221, DOI 10.1007/BF00176529"
"   Sharma PK, 2000, APPL ENVIRON MICROB, V66, P3083, DOI 10.1128/AEM.66.7.3083-3087.2000"
"   Singh A., 2014, Virology Mycology, V3, P127, DOI [10.4172/2161-0517.1000127, DOI 10.4172/2161-0517.1000127, https://doi.org/10.4172/2161-0517.1000127]"
"   Singh K, 2015, J BACTERIOL, V197, P2545, DOI 10.1128/JB.02433-14"
"   Stanciu A, 2024, ENVIRON ENG MANAG J, V23, DOI 10.30638/eemj.2024.185"
"   STOHS SJ, 1995, FREE RADICAL BIO MED, V18, P321, DOI 10.1016/0891-5849(94)00159-H"
"   Sunanda, 2022, J BASIC MICROB, V62, P428, DOI 10.1002/jobm.202100384"
"   Taniguchi J, 2000, APPL MICROBIOL BIOT, V54, P581, DOI 10.1007/s002530000415"
"   Tchounwou Paul B, 2012, Exp Suppl, V101, P133, DOI 10.1007/978-3-7643-8340-4_6"
"   Teitzel GM, 2003, APPL ENVIRON MICROB, V69, P2313, DOI 10.1128/AEM.69.4.2313-2320.2003"
"   Thelwell C, 1998, P NATL ACAD SCI USA, V95, P10728, DOI 10.1073/pnas.95.18.10728"
"   TIMMIS KN, 1994, ANNU REV MICROBIOL, V48, P525, DOI 10.1146/annurev.mi.48.100194.002521"
"   Tyagi B., 2021, BIOREMEDIATION ENV S, P3, DOI DOI 10.1016/B978-0-12-820524-2.00001-8"
"   Valencia EY, 2013, BMC MICROBIOL, V13, DOI 10.1186/1471-2180-13-79"
"   Valls M, 2002, FEMS MICROBIOL REV, V26, P327, DOI 10.1111/j.1574-6976.2002.tb00618.x"
"   Vats N, 2001, MICROBIOL-UK, V147, P653, DOI 10.1099/00221287-147-3-653"
"   Vijayaraghavan K, 2008, BIOTECHNOL ADV, V26, P266, DOI 10.1016/j.biotechadv.2008.02.002"
"   Volesky B, 2007, WATER RES, V41, P4017, DOI 10.1016/j.watres.2007.05.062"
"   Wang CL, 2002, BIOTECHNOL LETT, V24, P637, DOI 10.1023/A:1015043324584"
"   Wang X, 2023, ENVIRON POLLUT, V317, DOI 10.1016/j.envpol.2022.120740"

"   White VE, 2000, ARCH MICROBIOL, V173, P373, DOI 10.1007/s002030000157"
"   WILLIAMS JR, 1993, APPL ENVIRON MICROB, V59, P2531, DOI 10.1128/AEM.59.8.2531-2537.1993"
"   Witkowska D, 2021, MOLECULES, V26, DOI 10.3390/molecules26196060"
"   Wrobel Monika, 2023, Int J Environ Res Public Health, V20, DOI [10.3390/ijerph20064964, 10.3390/ijerph20064964]"
"   Wuana R.A., 2011, ISRN Ecology, DOI [10.5402/2011/402647, DOI 10.5402/2011/402647]"
"   Yilmaz EI, 2003, RES MICROBIOL, V154, P409, DOI 10.1016/S0923-2508(03)00116-5"
"   Yu FB, 2013, J ENVIRON SCI HEAL B, V48, P198, DOI 10.1080/03601234.2013.730299"
"   Zhou B, 2023, APPL SCI-BASEL, V13, DOI 10.3390/app13148439"
NR 163
TC 0
Z9 0
U1 8
U2 17
PU GH ASACHI TECHNICAL UNIV IASI
PI IASI
"PA 71 MANGERON BLVD, IASI, 700050, ROMANIA"
SN 1582-9596
EI 1843-3707
J9 ENVIRON ENG MANAG J
JI Environ. Eng. Manag. J.
PD MAR
PY 2025
VL 24
IS 3
DI 10.30638/eemj.2025.043
PG 232
WC Environmental Sciences
WE Science Citation Index Expanded (SCI-EXPANDED)
SC Environmental Sciences & Ecology
GA 3HI8T
UT WOS:001500367100008
OA gold
DA 2026-04-06
ER

PT J
"AU Deng, SJ"
"   Lu, XW"
"   Chen, XD"
"   Lei, K"
"   Zhu, T"
"   Zhang, YS"
"   Yu, B"
"AF Deng, Sijia"
"   Lu, Xinwei"
"   Chen, Xiuduan"
"   Lei, Kai"
"   Zhu, Tong"
"   Zhang, Yingsen"
"   Yu, Bo"
TI Research on biochar remediation of heavy metal contaminated soil based
   on CiteSpace visual analysis
SO CLEAN TECHNOLOGIES AND ENVIRONMENTAL POLICY
LA English
DT Review
DE Bibliometrics; Biochar remediation; CiteSpace software; Cadmium;
   Polluted soil; Research hotspots
ID PYROLYSIS TEMPERATURE; AQUEOUS-SOLUTIONS; RICE STRAW; PHYSIOCHEMICAL
   PROPERTIES; BIBLIOMETRIC ANALYSIS; EMERGING TRENDS; POTENTIAL ROLE;
   WATER; CO; IMMOBILIZATION
"AB Biochar remediation technology is increasingly being valued due to its economic and environmental advantages in treating heavy metal contaminated soil. In order to comprehensively grasp the research progress in this field, we analyzed the current research status, hotspots, and development directions of biochar passivation remediation of heavy metal contaminated soil based on the core collection database of Web of Science from 2008 to 2022, using bibliometrics and CiteSpace visualization technology. The analysis results indicate that since 2014, research on biochar remediation technology has become a hot topic in the treatment of heavy metal contaminated soil, and the number of literature in this field has grown rapidly. The application scope, mechanism of action, application effect, preparation method, and optimization technology of biochar are the main focus of this field. The selection of iron-modified biochar in the rice-soil system, biochar-based nanocomposites, and the combined application of phytoremediation and biochar are current research hotspots. The interaction mechanism between biochar and soil system, microbial remediation technology based on biochar, potential long-term effects of biochar use, and separation technology between biochar and soil are future research directions in the field of biochar remediation of heavy metal contaminated soil."
"C1 [Deng, Sijia; Lu, Xinwei; Zhu, Tong; Zhang, Yingsen; Yu, Bo] Shaanxi Normal Univ, Sch Geog & Tourism, Dept Environm Sci, Xian 710119, Peoples R China."
"   [Chen, Xiuduan] Shaanxi Xueqian Normal Univ, Sch Econ & Management, Xian 710100, Peoples R China."
"   [Lei, Kai] Xian Univ, Sch Biol & Environm Engn, Xian 710065, Peoples R China."
C3 Shaanxi Normal University; Shaanxi Xueqian Normal University; Xi'an
   University
"RP Lu, XW (corresponding author), Shaanxi Normal Univ, Sch Geog & Tourism, Dept Environm Sci, Xian 710119, Peoples R China."
EM luxinwei@snnu.edu.cn

FU National Natural Science Foundation of China
FX No Statement Available
"CR Abdeljaoued E, 2020, CLEAN TECHNOL ENVIR, V22, P1967, DOI 10.1007/s10098-020-01969-x"
"   Adekiya Aruna Olasekan, 2020, ScientificWorldJournal, V2020, P9391630, DOI [10.1155/2020/4610515, 10.1155/2020/9391630, 10.1155/2020/9391630]"
"   Agrafioti E, 2013, J ANAL APPL PYROL, V101, P72, DOI 10.1016/j.jaap.2013.02.010"
"   Ahmad M, 2014, CHEMOSPHERE, V99, P19, DOI 10.1016/j.chemosphere.2013.10.071"
"   Ahmed MB, 2016, BIORESOURCE TECHNOL, V214, P836, DOI 10.1016/j.biortech.2016.05.057"
"   Alkharabsheh HM, 2021, AGRONOMY-BASEL, V11, DOI 10.3390/agronomy11050993"
"   Alma Mehmet Hakki, 2021, Journal of the Institute of Science and Technology, V11, P2599, DOI 10.21597/jist.931246"
"   Amini S, 2016, J SOIL SEDIMENT, V16, P939, DOI 10.1007/s11368-015-1293-1"
"   An XY, 2011, SCIENTOMETRICS, V88, P133, DOI 10.1007/s11192-011-0374-1"
"   Baig SA, 2016, MATER SCI-POLAND, V34, P597, DOI 10.1515/msp-2016-0112"
"   Bakshi S, 2015, CRIT REV ENV SCI TEC, V45, P861, DOI 10.1080/10643389.2014.921975"
"   Baruah N, 2020, J ENVIRON CHEM ENG, V8, DOI 10.1016/j.jece.2020.104480"
"   Bashir S, 2018, B ENVIRON CONTAM TOX, V100, P834, DOI 10.1007/s00128-018-2332-6"
"   Beesley L, 2011, ENVIRON POLLUT, V159, P3269, DOI 10.1016/j.envpol.2011.07.023"
"   Bolan N, 2014, J HAZARD MATER, V266, P141, DOI 10.1016/j.jhazmat.2013.12.018"
"   Borggaard OK, 2019, GEODERMA, V343, P235, DOI 10.1016/j.geoderma.2019.02.041"
"   Chathurika JAS, 2016, CAN J SOIL SCI, V96, P472, DOI 10.1139/cjss-2015-0094"
"   Chausali N, 2021, J AGR FOOD RES, V5, DOI 10.1016/j.jafr.2021.100191"
"   Chen Chaomei, 2020, Front Res Metr Anal, V5, P607286, DOI [10.3389/frma.2020.607286, 10.3389/frma.2020.607286]"
"   Chen CM, 2017, J DATA INFO SCI, V2, P1, DOI 10.1515/jdis-2017-0006"
"   Chen CM, 2012, EXPERT OPIN BIOL TH, V12, P593, DOI 10.1517/14712598.2012.674507"
"   Chen CM, 2010, J AM SOC INF SCI TEC, V61, P1386, DOI 10.1002/asi.21309"
"   Chen CM, 2006, J AM SOC INF SCI TEC, V57, P359, DOI 10.1002/asi.20317"
"   Chen T, 2014, BIORESOURCE TECHNOL, V164, P47, DOI 10.1016/j.biortech.2014.04.048"
"   Cho J, 2014, J INF SCI, V40, P386, DOI 10.1177/0165551514524686"
"   Dai ZM, 2017, SCI TOTAL ENVIRON, V581, P601, DOI [10.1016/j.scitotenv2016.12.169, 10.1016/j.scitotenv.2016.12.169]"
"   Demirbas A, 2004, J ANAL APPL PYROL, V72, P215, DOI 10.1016/j.jaap.2004.06.005"
"   Dong DM, 2009, J HAZARD MATER, V162, P1261, DOI 10.1016/j.jhazmat.2008.06.032"
"   Dong XD, 2014, BIORESOURCE TECHNOL, V160, P123, DOI 10.1016/j.biortech.2014.01.006"
"   Fan JJ, 2020, J HAZARD MATER, V388, DOI 10.1016/j.jhazmat.2020.122037"
"   Fang Y, 2018, J SUSTAIN TOUR, V26, P108, DOI 10.1080/09669582.2017.1329310"
"   FREEMAN LC, 1979, SOC NETWORKS, V1, P215, DOI 10.1016/0378-8733(78)90021-7"
"   Gao L, 2017, J SOIL SCI PLANT NUT, V17, P884, DOI 10.4067/S0718-95162017000400004"
"   Gasc?? G, 2019, CHEMOSPHERE, V231, P562, DOI 10.1016/j.chemosphere.2019.05.168"
"   Gasp??ri J, 2018, ENVIRON SCI POLLUT R, V25, P23559, DOI 10.1007/s11356-016-8005-2"
"   Gong HB, 2022, J HAZARD MATER, V432, DOI 10.1016/j.jhazmat.2022.128668"
"   Gong XM, 2019, SCI TOTAL ENVIRON, V666, P1126, DOI 10.1016/j.scitotenv.2019.02.215"
"   Gul S, 2015, AGR ECOSYST ENVIRON, V206, P46, DOI 10.1016/j.agee.2015.03.015"
"   He LZ, 2019, ENVIRON POLLUT, V252, P846, DOI 10.1016/j.envpol.2019.05.151"
"   He X, 2022, ENVIRON POLLUT, V313, DOI 10.1016/j.envpol.2022.120175"
"   Houben D, 2013, CHEMOSPHERE, V92, P1450, DOI 10.1016/j.chemosphere.2013.03.055"
"   Hu BW, 2020, BIOCHAR, V2, P47, DOI 10.1007/s42773-020-00044-4"
"   Ibrahim M, 2016, INT J ENVIRON SCI TE, V13, P2467, DOI 10.1007/s13762-016-1081-5"
"   International Biochar Initiative, 2015, STAND PROD DEF PROD"
"   Inyang M, 2014, CHEM ENG J, V236, P39, DOI 10.1016/j.cej.2013.09.074"
"   Ippolito JA, 2020, BIOCHAR, V2, P421, DOI 10.1007/s42773-020-00067-x"
"   Irshad MK, 2020, SCI TOTAL ENVIRON, V717, DOI 10.1016/j.scitotenv.2020.137086"
"   Jing XR, 2014, CHEM ENG J, V248, P168, DOI 10.1016/j.cej.2014.03.006"
"   Joseph S, 2021, GCB BIOENERGY, V13, P1731, DOI 10.1111/gcbb.12885"
"   Karami N, 2011, J HAZARD MATER, V191, P41, DOI 10.1016/j.jhazmat.2011.04.025"
"   Khalid S, 2017, J GEOCHEM EXPLOR, V182, P247, DOI 10.1016/j.gexplo.2016.11.021"
"   Khum-in V, 2020, PROCESS SAF ENVIRON, V141, P222, DOI 10.1016/j.psep.2020.05.008"
"   Lee YG, 2021, ENERGIES, V14, DOI 10.3390/en14051297"
"   Lehmann J., 2015, Biochar for Environmental Management: Science, Technology and Implementation, DOI [DOI 10.4324/9780203762264, 10.4324/9780203762264]"
"   Lehmann J, 2007, NATURE, V447, P143, DOI 10.1038/447143a"
"   Li B, 2017, CHEMOSPHERE, V175, P332, DOI 10.1016/j.chemosphere.2017.02.061"
"   Li DY, 2020, ENVIRON SCI POLLUT R, V27, P2898, DOI 10.1007/s11356-019-06870-9"
"   Li HB, 2017, CHEMOSPHERE, V178, P466, DOI 10.1016/j.chemosphere.2017.03.072"
"   Li Q, 2017, DESALIN WATER TREAT, V82, P179, DOI 10.5004/dwt.2017.20962"
"   Li X, 2021, CHEMOSPHERE, V274, DOI 10.1016/j.chemosphere.2021.129766"
"   Li YZ, 2022, ENVIRON MODELL SOFTW, V149, DOI 10.1016/j.envsoft.2022.105329"
"   Liang LP, 2021, BIOCHAR, V3, P255, DOI 10.1007/s42773-021-00101-6"
"   Ling LL, 2017, ENVIRON SCI TECHNOL, V51, P10081, DOI 10.1021/acs.est.7b02382"
"   Liu MS, 2022, SCI TOTAL ENVIRON, V806, DOI 10.1016/j.scitotenv.2021.150531"
"   Liu YN, 2020, J SOIL SEDIMENT, V20, P1513, DOI 10.1007/s11368-019-02519-9"
"   Lu KP, 2017, J ENVIRON MANAGE, V186, P285, DOI 10.1016/j.jenvman.2016.05.068"
"   Lwin CS, 2018, SOIL SCI PLANT NUTR, V64, P156, DOI 10.1080/00380768.2018.1440938"
"   Ma YF, 2020, SCI TOTAL ENVIRON, V718, DOI 10.1016/j.scitotenv.2020.137299"
"   Memon KA, 2020, OPT SWITCH NETW, V39, DOI 10.1016/j.osn.2020.100586"
"   Meng J, 2018, SCI TOTAL ENVIRON, V633, P300, DOI 10.1016/j.scitotenv.2018.03.199"
"   Menzembere ERGY, 2023, PEDOSPHERE, V33, P23, DOI 10.1016/j.pedsph.2022.06.030"
"   Mottaleb KA, 2019, AGR WATER MANAGE, V222, P242, DOI 10.1016/j.agwat.2019.05.038"
"   Nawab J, 2018, ENVIRON SCI POLLUT R, V25, P5558, DOI 10.1007/s11356-017-0764-x"
"   Nejad ZD, 2018, ENVIRON GEOCHEM HLTH, V40, P927, DOI 10.1007/s10653-017-9964-z"
"   Ok YS, 2015, ENVIRON TECHNOL INNO, V4, P206, DOI 10.1016/j.eti.2015.08.003"
"   Pan WW, 2019, SCIENTOMETRICS, V121, P1407, DOI 10.1007/s11192-019-03256-z"
"   Peng P, 2016, ECOL ENG, V90, P225, DOI 10.1016/j.ecoleng.2016.01.039"

"   Qin FZ, 2022, SCI TOTAL ENVIRON, V818, DOI 10.1016/j.scitotenv.2021.151774"
"   Rajendran M, 2019, CHEMOSPHERE, V222, P314, DOI 10.1016/j.chemosphere.2019.01.149"
"   Rajendran S, 2022, CHEMOSPHERE, V287, DOI 10.1016/j.chemosphere.2021.132369"
"   Rees F, 2020, SCI TOTAL ENVIRON, V707, DOI 10.1016/j.scitotenv.2019.135654"
"   Rosestolato D, 2015, CHEM ENG J, V264, P16, DOI 10.1016/j.cej.2014.11.074"
"   SMALL H, 1973, J AM SOC INFORM SCI, V24, P265, DOI 10.1002/asi.4630240406"
"   Soares MAR, 2015, J ENVIRON MANAGE, V164, P137, DOI 10.1016/j.jenvman.2015.08.042"
"   Sun T, 2021, CHEMOSPHERE, V280, DOI 10.1016/j.chemosphere.2021.130606"
"   Tang JY, 2019, J ENVIRON MANAGE, V242, P121, DOI 10.1016/j.jenvman.2019.04.061"
"   Tripathi M, 2016, RENEW SUST ENERG REV, V55, P467, DOI 10.1016/j.rser.2015.10.122"
"   Uchimiya M, 2011, J AGR FOOD CHEM, V59, P2501, DOI 10.1021/jf104206c"
"   Uchimiya M, 2010, J AGR FOOD CHEM, V58, P5538, DOI 10.1021/jf9044217"
"   Vithanage M, 2017, CARBON, V113, P219, DOI 10.1016/j.carbon.2016.11.032"
"   Wang B, 2017, CRIT REV ENV SCI TEC, V47, P2158, DOI 10.1080/10643389.2017.1418580"
"   Wang DJ, 2013, ENVIRON SCI TECHNOL, V47, P5154, DOI 10.1021/es305337r"
"   Wang JL, 2019, J CLEAN PROD, V227, P1002, DOI 10.1016/j.jclepro.2019.04.282"
"   Wang L, 2022, J HAZARD MATER, V435, DOI 10.1016/j.jhazmat.2022.128991"
"   Wang SS, 2019, J HAZARD MATER, V373, P820, DOI 10.1016/j.jhazmat.2019.03.080"
"   Wang SS, 2015, BIORESOURCE TECHNOL, V175, P391, DOI 10.1016/j.biortech.2014.10.104"
"   Wang YY, 2019, J CLEAN PROD, V231, P556, DOI 10.1016/j.jclepro.2019.04.407"
"   Wei SY, 2019, CHEMOSPHERE, V218, P624, DOI 10.1016/j.chemosphere.2018.11.177"
"   Xiao R, 2017, BIORESOURCE TECHNOL, V246, P203, DOI 10.1016/j.biortech.2017.07.090"
"   Xu CB, 2020, ENVIRON POLLUT, V261, DOI 10.1016/j.envpol.2020.114133"
"   Yan JC, 2015, BIORESOURCE TECHNOL, V175, P269, DOI 10.1016/j.biortech.2014.10.103"
"   Yang H, 2014, SCIENCE, V344, P691, DOI 10.1126/science.344.6185.691-b"
"   Yang H, 2019, SUSTAINABILITY-BASEL, V11, DOI 10.3390/su11184908"
"   Yang R, 2021, J CLEAN PROD, V278, DOI 10.1016/j.jclepro.2020.123402"
"   Yang X, 2016, ENVIRON SCI POLLUT R, V23, P974, DOI 10.1007/s11356-015-4233-0"
"   Yao Q, 2017, SOIL BIOL BIOCHEM, V110, P56, DOI 10.1016/j.soilbio.2017.03.005"
"   Ye SJ, 2019, RESOUR CONSERV RECY, V140, P278, DOI 10.1016/j.resconrec.2018.10.004"
"   Yin DX, 2017, CHEMOSPHERE, V186, P928, DOI 10.1016/j.chemosphere.2017.07.126"
"   Yu DJ, 2020, INT J MACH LEARN CYB, V11, P715, DOI 10.1007/s13042-019-01028-y"
"   Yu HW, 2019, J ENVIRON MANAGE, V232, P8, DOI 10.1016/j.jenvman.2018.10.117"
"   Zeng XY, 2020, J ENVIRON MANAGE, V255, DOI 10.1016/j.jenvman.2019.109885"
"   Zhang CS, 2017, APPL SOIL ECOL, V112, P90, DOI 10.1016/j.apsoil.2016.12.005"
"   Zhang M, 2012, SCI TOTAL ENVIRON, V435, P567, DOI 10.1016/j.scitotenv.2012.07.038"
"   Zhang QG, 2021, ENVIRON POLLUT, V272, DOI 10.1016/j.envpol.2020.115989"
"   Zhang RH, 2017, ECOL ENG, V98, P183, DOI 10.1016/j.ecoleng.2016.10.057"
"   Zhang WH, 2015, J ENVIRON QUAL, V44, P1119, DOI 10.2134/jeq2014.12.0536"
"   Zhao Y, 2023, ENVIRON SCI POLLUT R, V30, P67953, DOI 10.1007/s11356-023-27295-5"
"   Zou HW, 2021, J HAZARD MATER, V413, DOI 10.1016/j.jhazmat.2021.125252"
NR 118
TC 6
Z9 6
U1 37
U2 219
PU SPRINGER
PI NEW YORK
"PA ONE NEW YORK PLAZA, SUITE 4600, NEW YORK, NY, UNITED STATES"
SN 1618-954X
EI 1618-9558
J9 CLEAN TECHNOL ENVIR
JI Clean Technol. Environ. Policy
PD SEP
PY 2024
VL 26
IS 9
BP 2801
EP 2817
DI 10.1007/s10098-024-02767-5
EA MAR 2024
PG 17
"WC Green & Sustainable Science & Technology; Engineering, Environmental;"
   Environmental Sciences
WE Science Citation Index Expanded (SCI-EXPANDED)
SC Science & Technology - Other Topics; Engineering; Environmental Sciences
   & Ecology
GA E7E3Z
UT WOS:001174984800001
DA 2026-04-06
ER

PT J
"AU Di, Z"
"   Chaoyang, L"
"   Mengxi, Z"
"   Yunlin, Z"
"   Zhenggang, X"
"   Guiyan, Y"
"AF Di, Zhao"
"   Chaoyang, Li"
"   Mengxi, Zheng"
"   Yunlin, Zhao"
"   Zhenggang, Xu"

"   Guiyan, Yang"
"TI Curvularia coatesiae XK8, a Potential Bioadsorbent Material for"
   Adsorbing Cd(II) and Sb(III) Compound Pollution: Characteristics and
   Effects
SO FRONTIERS IN MICROBIOLOGY
LA English
DT Article
DE fungi; heavy metal pollution; biosorption; microbial remediation;
   response surface experiment
ID HEAVY-METALS; BIOSORPTION; ANTIMONY; REMOVAL; CADMIUM; MECHANISM; MINE;
   OPTIMIZATION; PERFORMANCE; COPPER
"AB Soil heavy metal pollution is a common problem in mining areas. The soil of the Xikuangshan located in Lengshuijiang, Hunan Province, China contains various excessive heavy metals, especially antimony and cadmium. Previous studies have shown that heavy metal-tolerant microorganisms screened from mining areas have the potential to adsorb heavy metals. In this study, we screened out a cadmium and antimony tolerant fungus named XK8 from the slags collected from the Xikuangshan. Then, we explored the single and binary biosorption characteristics of Cd(II) and Sb(III) on it. In our results, the fungus XK8 was identified as Curvularia coatesiae XK8 by ITS sequencing analysis. Under the optimal conditions, in binary biosorption of the XK8, the main effect of the initial cadmium concentration on the cadmium removal rate of XK8 is negative, while the main effect of the initial antimony concentration, biosorption time, and initial pH on the cadmium removal rate of XK8 is positive. The initial pH has the greatest impact on the biosorption of cadmium on XK8, followed by the biosorption time; moreover, the effects of both are stronger than the coexisting ions. SAS analysis shows that under the optimal conditions, the theoretical maximum cadmium removal rate of XK8 is 100%, and the actual removal rate is 67.57%. Compared to the single biosorption with binary biosorption, the maximum biosorption capacity of XK8 for cadmium in the composite biosorption system increased to 23.6 mg g(-1). It shows that under the background of high antimony, Sb(III) has a promoting effect on the biosorption of Cd(II) on XK8. In summary, a cadmium and antimony tolerant fungus with strong cadmium biosorption ability under the background of high antimony was screened out. It provides a potential microbial material for the bioremediation of heavy metal pollution."
"C1 [Di, Zhao; Yunlin, Zhao; Zhenggang, Xu] Cent South Univ Forestry & Technol, Hunan Res Ctr Engn Technol Utilizat Environm & Re, Changsha, Peoples R China."
"   [Chaoyang, Li] Cent South Inventory & Planning Inst Natl Forestr, Changsha, Peoples R China."
"   [Mengxi, Zheng; Zhenggang, Xu; Guiyan, Yang] Northwest A&F Univ, Key Lab Natl Forestry & Grassland Adm Management, Coll Forestry, Xianyang, Peoples R China."
C3 Central South University of Forestry & Technology; Northwest A&F
   University - China
"RP Yunlin, Z; Zhenggang, X (corresponding author), Cent South Univ Forestry & Technol, Hunan Res Ctr Engn Technol Utilizat Environm & Re, Changsha, Peoples R China.; Zhenggang, X (corresponding author), Northwest A&F Univ, Key Lab Natl Forestry & Grassland Adm Management, Coll Forestry, Xianyang, Peoples R China."
EM zyl8291290@163.com; xuzhenggang@nwafu.edu.cn
"RI ; li, chaoyang/AAP-3209-2020"
"OI Zhenggang, Xu/0000-0002-7401-5163; "
FU Key Projects of the National Forestry and Grassland Bureau [201801];
   Major Science and Technology Program of Hunan Province [2017NK1014];
   Open Fund of Key Laboratory of Microbial Resources Collection and
"   Preservation, Ministry of Agriculture and Rural Affairs [KLMRCP2021-07];"
   Experimental Demonstration Station (Base) Science and Technology
   Innovation and Achievement Transformation Project from Northwest
   Agriculture and Forestry University [TGZX2021-41]
FX This work was supported by Key Projects of the National Forestry and
   Grassland Bureau (201801); Major Science and Technology Program of Hunan
   Province (2017NK1014); Open Fund of Key Laboratory of Microbial
"   Resources Collection and Preservation, Ministry of Agriculture and Rural"
   Affairs (KLMRCP2021-07); and Experimental Demonstration Station (Base)
   Science and Technology Innovation and Achievement Transformation Project
   from Northwest Agriculture and Forestry University (TGZX2021-41).
"CR Bhuiyan MAH, 2010, J HAZARD MATER, V173, P384, DOI 10.1016/j.jhazmat.2009.08.085"
"   Bin L., 2014, THESIS GUANGXI U"
"   Cai Y, 2018, INT J ENV RES PUB HE, V15, DOI 10.3390/ijerph15040702"
"   Chien CC, 2013, BIOCHEM ENG J, V78, P132, DOI 10.1016/j.bej.2013.01.014"
"   Choinska-Pulit A, 2018, ECOTOX ENVIRON SAFE, V149, P275, DOI 10.1016/j.ecoenv.2017.12.008"
"   Deng XH, 2012, J HAZARD MATER, V233, P25, DOI 10.1016/j.jhazmat.2012.06.054"
"   Guo XJ, 2014, J ENVIRON SCI-CHINA, V26, P1549, DOI 10.1016/j.jes.2014.05.022"
"   Hassan SHA, 2018, CHEMOSPHERE, V202, P750, DOI 10.1016/j.chemosphere.2018.03.114"
"   He MC, 2007, ENVIRON GEOCHEM HLTH, V29, P209, DOI 10.1007/s10653-006-9066-9"
"   He MC, 2012, SCI TOTAL ENVIRON, V421, P41, DOI 10.1016/j.scitotenv.2011.06.009"
"   Huang HM, 2018, PLOS ONE, V13, DOI 10.1371/journal.pone.0203285"
"   Jacob JM, 2018, J ENVIRON MANAGE, V217, P56, DOI 10.1016/j.jenvman.2018.03.077"
"   Kumar V, 2019, CHEMOSPHERE, V237, DOI 10.1016/j.chemosphere.2019.124567"
"   Li JK, 2018, GEOMICROBIOL J, V35, P713, DOI 10.1080/01490451.2018.1454556"
"   Li J, 2019, J ENVIRON MANAGE, V237, P552, DOI 10.1016/j.jenvman.2019.02.057"
"   [????????? Li Shuangshuang], 2009, [????????????, Environmental Protection of Chemical Industry], V29, P131"
"   Li YC, 2016, WATER SCI TECHNOL, V73, P2039, DOI 10.2166/wst.2016.044"
"   Liu RP, 2015, J HAZARD MATER, V300, P847, DOI 10.1016/j.jhazmat.2015.08.020"
"   Mohite BV, 2018, APPL BIOCHEM BIOTECH, V186, P199, DOI 10.1007/s12010-018-2727-1"
"   Noormohamadi HR, 2019, CHEMOSPHERE, V216, P124, DOI 10.1016/j.chemosphere.2018.10.113"
"   Ren Jie, 2020, Huanjing Huaxue-Environmental Chemistry, V39, P3436, DOI 10.7524/j.issn.0254-6108.2019090701"
"   [????????? RU ShuHua], 2006, [??????????€?????????????, Chinese journal of eco-agriculture], V14, P29"
"   Su JF, 2019, CHEMOSPHERE, V222, P970, DOI 10.1016/j.chemosphere.2019.01.192"
"   Tian D, 2019, ENVIRON MICROBIOL, V21, P471, DOI 10.1111/1462-2920.14478"
"   Wang JL, 2009, BIOTECHNOL ADV, V27, P195, DOI 10.1016/j.biotechadv.2008.11.002"

"   [????????? Wang Jingwen], 2015, [????????????????????????, Journal of Agro-Environment Science], V34, P1693"
"   Wang NN, 2017, BIORESOURCE TECHNOL, V233, P58, DOI 10.1016/j.biortech.2017.02.069"
"   Wang ZQ, 2018, ECOTOX ENVIRON SAFE, V154, P329, DOI 10.1016/j.ecoenv.2018.02.048"
"   Xia L, 2015, INT J MOL SCI, V16, P15670, DOI 10.3390/ijms160715670"
"   Xu XJ, 2018, GEOMICROBIOL J, V35, P40, DOI 10.1080/01490451.2017.1310331"
"   Xu XJ, 2015, FRONT MICROBIOL, V6, DOI 10.3389/fmicb.2015.01422"
"   Xu XJ, 2012, ENVIRON TECHNOL, V33, P1661, DOI 10.1080/09593330.2011.641591"
"   Yan C, 2008, ENG LIFE SCI, V8, P363, DOI 10.1002/elsc.200820237"
"   Yang RJ, 2019, J SOIL SEDIMENT, V19, P1319, DOI 10.1007/s11368-018-2140-y"
NR 34
TC 15
Z9 17
U1 4
U2 103
PU FRONTIERS MEDIA SA
PI LAUSANNE
"PA AVENUE DU TRIBUNAL FEDERAL 34, LAUSANNE, CH-1015, SWITZERLAND"
EI 1664-302X
J9 FRONT MICROBIOL
JI Front. Microbiol.
PD JAN 27
PY 2022
VL 12
AR 816312
DI 10.3389/fmicb.2021.816312
PG 13
WC Microbiology
WE Science Citation Index Expanded (SCI-EXPANDED)
SC Microbiology
GA YW7XK
UT WOS:000753627500001
PM 35154041
"OA Green Submitted, gold"
DA 2026-04-06
ER

PT J
"AU Lin, YB"
"   Ye, YM"
"   Hu, YM"
"   Shi, HK"
"AF Lin, Yaoben"
"   Ye, Yanmei"
"   Hu, Yiming"
"   Shi, Haokun"
TI The variation in microbial community structure under different heavy
   metal contamination levels in paddy soils
SO ECOTOXICOLOGY AND ENVIRONMENTAL SAFETY
LA English
DT Article
DE Heavy metal contamination; Soil bacteria; Soil fungi; High throughput
   sequencing
ID ARBUSCULAR MYCORRHIZAL FUNGI; HUMAN HEALTH; DIVERSITY; FOREST; IMPACTS;
   CHINA; RICE; RISK
"AB As a global pollution problem, heavy metal contamination poses a serious hazard to soil microorganisms which play an extremely important role in soil chemical cycling and ecological persistence. However, the effects that different levels of heavy metal contamination in soils have on microorganisms and the interactions between them are still unclear. The purpose of this research is to analyze the microbial structure under different levels of heavy metal contamination, find out heavy metal tolerant species under different environmental conditions, then provide useful reference for the bioremediation of contaminated farmland. In this study, 16s rRNA high throughput sequencing technology was used to investigate the microbial communities in severe level (SL), moderate level (ML), light level (LL) and clean level (CL) of heavy metal contaminated soils, and the relationships between environment variables and microorganisms were analyzed. The results showed that the concentrations of heavy metals and soil physicochemical properties had various impacts on microbial community composition under different heavy metal contamination levels. Most dominant bacteria were in significant negative correlation with Cd in ML region, and significantly correlated with TN and OM in LL region. However, there was no significant correlation between dominant fungi and the physicochemical properties in LL region. And most of the dominant fungi were significantly correlated with the heavy metal concentrations in SL region. The bacterial phyla such as Proteobacteria, Acidobacteria and Bacteroidetes showed more tolerance with heavy metal contamination in SL, ML and LL regions, respectively. Meanwhile, the dominant fungi of Ascomycota, Basidiomycota, Chytridiomycota, Glomeromycota, Zygomycota and Rozellomycota showed stronger correlations with heavy metal contamination in SL and LL regions. These results indicated that some microorganisms had strong tolerance to heavy metal contamination and had certain heavy metals digestion ability, which can create an appropriate soil environment for the growth of food crops."
"C1 [Lin, Yaoben; Ye, Yanmei; Hu, Yiming; Shi, Haokun] Zhejiang Univ, LAND, Hangzhou 310058, Zhejiang, Peoples R China."
"   [Lin, Yaoben; Ye, Yanmei; Hu, Yiming; Shi, Haokun] Land Ecol Restorat Engn Technol Res Ctr Shandong, Binzhou 256600, Peoples R China."
C3 Zhejiang University
"RP Ye, YM (corresponding author), Zhejiang Univ, LAND, Hangzhou 310058, Zhejiang, Peoples R China."
EM yeyanmei@zju.edu.cn
FU Social Science Foundation of China [14ZDA039]; Shandong Yellow River
   delta land use and ecological engineering technology research center

project; Shandong province saline-alkali land comprehensive development demonstration project
FX This research was supported by the Social Science Foundation of China
"   through project No.14ZDA039, Shandong Yellow River delta land use and"
"   ecological engineering technology research center project, and Shandong"
province saline-alkali land comprehensive development demonstration project.
"CR Al-Sadi AM, 2017, INT J AGRIC BIOL, V19, P682, DOI 10.17957/IJAB/15.0328"
"   Alsaleh KAM, 2018, J ENVIRON MANAGE, V206, P731, DOI 10.1016/j.jenvman.2017.11.026"
"   Baldoncini M, 2019, GEODERMA, V335, P69, DOI 10.1016/j.geoderma.2018.08.012"
"   Barnes AD, 2017, NAT ECOL EVOL, V1, P1511, DOI 10.1038/s41559-017-0275-7"
"   Blackwood CB, 2007, ENVIRON MICROBIOL, V9, P1306, DOI 10.1111/j.1462-2920.2007.01250.x"
"   Caban JR, 2018, APPL SOIL ECOL, V129, P72, DOI 10.1016/j.apsoil.2018.04.013"
"   DONG FQ, 2018, FORESTS, P98"
"   [????????? FAN Yun-hui], 2009, [???????????????, Geophysical and Geochemical Exploration], V33, P132"
"   Fierer N, 2017, NAT REV MICROBIOL, V15, P579, DOI 10.1038/nrmicro.2017.87"
"   Frey B, 2006, SOIL BIOL BIOCHEM, V38, P1745, DOI 10.1016/j.soilbio.2005.11.032"
"   Guo HH, 2017, ECOTOX ENVIRON SAFE, V144, P300, DOI 10.1016/j.ecoenv.2017.06.048"
"   Guo QX, 2019, SOIL BIOL BIOCHEM, V128, P127, DOI 10.1016/j.soilbio.2018.10.018"
"   Halter D, 2011, RES MICROBIOL, V162, P877, DOI 10.1016/j.resmic.2011.06.001"
"   Ho J, 2019, SOIL BIOL BIOCHEM, V130, P122, DOI 10.1016/j.soilbio.2018.12.004"
"   Jasper DA, 2007, RESTOR ECOL, V15, pS74, DOI 10.1111/j.1526-100X.2007.00295.x"
"   Jiang B, 2019, J HAZARD MATER, V362, P187, DOI 10.1016/j.jhazmat.2018.08.060"
"   Keshavarzi A, 2019, INT J ENVIRON HEAL R, V29, P544, DOI 10.1080/09603123.2018.1555638"
"   Kirkham MB, 2006, GEODERMA, V137, P19, DOI 10.1016/j.geoderma.2006.08.024"
"   Kuang JL, 2013, ISME J, V7, P1038, DOI 10.1038/ismej.2012.139"
"   Langille MGI, 2013, NAT BIOTECHNOL, V31, P814, DOI 10.1038/nbt.2676"
"   Legrand F, 2018, APPL SOIL ECOL, V132, P135, DOI 10.1016/j.apsoil.2018.08.016"
"   Li J, 2018, EUR J SOIL SCI, V69, P1056, DOI 10.1111/ejss.12714"
"   Li XQ, 2017, ENVIRON POLLUT, V231, P908, DOI 10.1016/j.envpol.2017.08.057"
"   Liang XQ, 2018, LAND DEGRAD DEV, V29, P2189, DOI 10.1002/ldr.2851"
"   Liu Chun-zao, 2012, Huanjing Kexue, V33, P260"
"   Liu J, 2008, BIODEGRADATION, V19, P695, DOI 10.1007/s10532-007-9174-y"
"   Luneberg K, 2018, SCI REPORTS, V8, P1, DOI [10.1038/s41598-017-17765-5, DOI 10.1038/S41598-017-17765-5]"
"   Ma B, 2018, ISME J, V12, P2492, DOI 10.1038/s41396-018-0232-8"
"   Malik AA, 2018, NAT COMMUN, V9, DOI 10.1038/s41467-018-05980-1"
"   Mao CP, 2019, CATENA, V175, P339, DOI 10.1016/j.catena.2018.12.029"
"   Mierzwa-Hersztek M, 2018, ENVIRON TOXICOL CHEM, V37, P1197, DOI 10.1002/etc.4045"
"   Nabulo G, 2010, SCI TOTAL ENVIRON, V408, P5338, DOI 10.1016/j.scitotenv.2010.06.034"
"   Nottingham AT, 2018, ECOLOGY, V99, P2455, DOI 10.1002/ecy.2482"
"   Obade VD, 2019, SCI TOTAL ENVIRON, V651, P2036, DOI 10.1016/j.scitotenv.2018.10.106"
"   Obi CC, 2016, FRONT MICROBIOL, V7, DOI 10.3389/fmicb.2016.01213"
"   Oliveira A, 2006, J BIOSCI BIOENG, V102, P157, DOI 10.1263/jbb.102.157"
"   Pasqualetti M, 2012, CRYPTOGAMIE MYCOL, V33, P43, DOI 10.7872/crym.v33.iss1.2012.043"
"   Peng H, 2019, SCI TOTAL ENVIRON, V660, P776, DOI 10.1016/j.scitotenv.2019.01.066"
"   Quan SX, 2015, ENVIRON SCI POLLUT R, V22, P1290, DOI 10.1007/s11356-014-3420-8"
"   Rahman Z, 2018, SAUDI J BIOL SCI, V25, P1687, DOI 10.1016/j.sjbs.2016.09.018"
"   S??nchez-Castro I, 2017, SCI TOTAL ENVIRON, V598, P121, DOI 10.1016/j.scitotenv.2017.04.084"
"   Shao PS, 2019, SOIL BIOL BIOCHEM, V128, P56, DOI 10.1016/j.soilbio.2018.10.004"
"   Singh BK, 2014, ENVIRON MICROBIOL, V16, P2408, DOI 10.1111/1462-2920.12353"
"   Smith MB, 2015, MBIO, V6, DOI 10.1128/mBio.00326-15"
"   Song XD, 2018, CATENA, V171, P588, DOI 10.1016/j.catena.2018.07.045"
"   Tian T, 2017, ENVIRON SCI POLLUT R, V24, P23236, DOI 10.1007/s11356-017-9837-0"
"   Wu HP, 2017, CRIT REV BIOTECHNOL, V37, P754, DOI [10.24966/ctb-9107/100009, 10.1080/07388551.2016.1232696]"
"   Wu SH, 2019, J HAZARD MATER, V364, P108, DOI 10.1016/j.jhazmat.2018.09.094"
"   Yousef M, 2018, FRONT PLANT SCI, V9, DOI 10.3389/fpls.2018.00001"
"   Zafar S, 2007, BIORESOURCE TECHNOL, V98, P2557, DOI 10.1016/j.biortech.2006.09.051"
"   Zarei M, 2010, ENVIRON POLLUT, V158, P2757, DOI 10.1016/j.envpol.2010.04.017"
"   Zhang HS, 2011, BIOL FERT SOILS, V47, P543, DOI 10.1007/s00374-011-0563-3"
"   Zhang WH, 2019, ECOTOX ENVIRON SAFE, V174, P100, DOI 10.1016/j.ecoenv.2019.02.048"
"   Zhu YX, 2013, PLOS ONE, V8, DOI 10.1371/journal.pone.0056386"
NR 54
TC 213
Z9 251
U1 16
U2 483
PU ACADEMIC PRESS INC ELSEVIER SCIENCE
PI SAN DIEGO
"PA 525 B ST, STE 1900, SAN DIEGO, CA 92101-4495 USA"
SN 0147-6513
EI 1090-2414
J9 ECOTOX ENVIRON SAFE
JI Ecotox. Environ. Safe.
PD SEP 30
PY 2019
VL 180
BP 557
EP 564
DI 10.1016/j.ecoenv.2019.05.057
PG 8
WC Environmental Sciences; Toxicology
WE Science Citation Index Expanded (SCI-EXPANDED)
SC Environmental Sciences & Ecology; Toxicology

GA IG5IG
UT WOS:000473836100064
PM 31128554
DA 2026-04-06
ER

PT J
"AU Gupta, R"
"   Khan, F"
"   Alqahtani, FM"
"   Hashem, M"
"   Ahmad, F"
"AF Gupta, Rishil"
"   Khan, Faryad"
"   Alqahtani, Fatmah M."
"   Hashem, Mohamed"
"   Ahmad, Faheem"
TI Plant Growth-Promoting Rhizobacteria (PGPR) Assisted Bioremediation of
   Heavy Metal Toxicity
SO APPLIED BIOCHEMISTRY AND BIOTECHNOLOGY
LA English
DT Review
DE Bioremediation; Heavy metals; Non-degradable; PGPR; Sustainable
   agriculture
ID MICROBIAL CARBONATE PRECIPITATION; HEXAVALENT CHROMIUM; ROOT-SYSTEM;
   SOIL; REMEDIATION; BACTERIA; REMOVAL; CADMIUM; CD; PHYTOEXTRACTION
"AB Due to a variety of natural and anthropogenic processes, heavy metal toxicity of soil constitutes a substantial hazard to all living beings in the environment. The heavy metals alter the soil properties, which directly or indirectly influence the agriculture systems. Thus, plant growth-promoting rhizobacteria (PGPR)-assisted bioremediation is a promising, eco-friendly, and sustainable method for eradicating heavy metals. PGPR cleans up the heavy metal-contaminated environment using various approaches including efflux systems, siderophores and chelation, biotransformation, biosorption, bioaccumulation, precipitation, ACC deaminase activity, biodegradation, and biomineralization methods. These PGPRs have been found effective to bioremediate the heavy metal-contaminated soil through increased plant tolerance to metal stress, improved nutrient availability in soil, alteration of heavy metal pathways, and by producing some chemical compounds like siderophores and chelating ions. Many heavy metals are non-degradable; hence, another remediation approach with a broader scope of contamination removal is needed. This article also briefly emphasized the role of genetically modified PGPR strains which improve the soil's degradation rate of heavy metals. In this regard, genetic engineering, a molecular approach, could improve bioremediation efficiency and be helpful. Thus, the ability of PGPRs can aid in heavy metal bioremediation and promote a sustainable agricultural soil system."
"C1 [Gupta, Rishil; Khan, Faryad; Ahmad, Faheem] Aligarh Muslim Univ, Dept Bot, Aligarh 202002, UP, India."
"   [Alqahtani, Fatmah M.; Hashem, Mohamed] King Khalid Univ, Coll Sci, Dept Biol, Abha 61413, Saudi Arabia."
C3 Aligarh Muslim University; King Khalid University
"RP Ahmad, F (corresponding author), Aligarh Muslim Univ, Dept Bot, Aligarh 202002, UP, India."
EM faheem.bt@amu.ac.in
"RI Ahmad, Faheem/ABH-6335-2020; Alqahtani, Fatmah/AAG-3894-2020; Khan,"
"   Faryad/IXN-6776-2023; Alqahtani, Fatmah/JXN-4032-2024; Hashem,"
   Mohamed/AAG-3894-2020
"OI Ahmad, Faheem/0000-0002-7450-0900; Alqahtani,"
"   Fatmah/0000-0002-2819-5431; Khan, Faryad/0000-0002-3786-0341; Alqahtani,"
"   Fatmah/0000-0002-2819-5431; Hashem, Mohamed/0000-0003-2593-3387"
"FU Deanship of Scientific Research, King Khalid University [R.G.P."
   2/205/44]
FX AcknowledgementsThe corresponding author is grateful to the Aligarh
"   Muslim University, Aligarh, for providing facilities to accomplish the"
   work. Fatmah M. Alqahtani and Mohamed Hashem extend their appreciation
"   to the Deanship of Scientific Research, King Khalid University, for"
   funding the work through the research groups program under grant number
   R.G.P. 2/205/44.
"CR Abatenh E., 2017, OPEN J ENV BIOL, DOI DOI 10.17352/OJEB.000007"
"   Achal V, 2011, ECOL ENG, V37, P1601, DOI 10.1016/j.ecoleng.2011.06.008"
"   Ahemad M., 2013, Biochem. Mol. Biol, V1, P19, DOI [DOI 10.12966/bmb.06.02.2013, 10.12966/bmb.06.02.2013, DOI 10.12966/BMB.06.02.2013]"
"   Ahemad M, 2019, ARAB J CHEM, V12, P1365, DOI 10.1016/j.arabjc.2014.11.020"
"   Alcalde-Rico M, 2016, FRONT MICROBIOL, V7, DOI 10.3389/fmicb.2016.01483"
"   Ambrosini VG, 2018, PLANT PHYSIOL BIOCH, V128, P89, DOI 10.1016/j.plaphy.2018.05.011"
"   [Anonymous], 2016, Int. J. Pharmacol. Biol. Sci."
"   Asadulla, 2021, PAK J BOT, V53, P31, DOI 10.30848/PJB2021-1(39)"
"   Azubuike CC, 2016, WORLD J MICROB BIOT, V32, DOI 10.1007/s11274-016-2137-x"
"   Bai J, 2019, J ENVIRON SCI, V78, P328, DOI 10.1016/j.jes.2018.11.012"
"   Bandowe BAM, 2014, ENVIRON INT, V65, P135, DOI 10.1016/j.envint.2013.12.018"
"   Barr D, 2002, BIOL METHODS ASSESSM"
"   Belogolova GA, 2019, IOP C SER EARTH ENV, V381, DOI 10.1088/1755-1315/381/1/012007"
"   Bhakat K, 2019, ENVIRON SCI POLLUT R, V26, P12907, DOI 10.1007/s11356-019-04827-6"
"   Briffa J, 2020, HELIYON, V6, DOI 10.1016/j.heliyon.2020.e04691"
"   Burd GI, 1998, APPL ENVIRON MICROB, V64, P3663"
"   Chao S., 2014, ENV SKEPTICS CRITICS, V3, P24, DOI DOI 10.1037/A0036071"
"   Chauhan PS, 2018, INT J ENVIRON SCI TE, V15, P2447, DOI 10.1007/s13762-017-1583-9"
"   CHIBUIKE G, 2014, APPL ENVIRON SOIL SC, V2014, P1, DOI [10.1155/2014/752708, DOI 10.1155/2014/752708]"
"   Compant S, 2010, SOIL BIOL BIOCHEM, V42, P669, DOI 10.1016/j.soilbio.2009.11.024"
"   Cui JL, 2020, J HAZARD MATER, V381, DOI 10.1016/j.jhazmat.2019.121208"
"   Cui JL, 2019, ENVIRON INT, V126, P717, DOI 10.1016/j.envint.2019.02.045"
"   Dabrowska G, 2017, INT J PHYTOREMEDIAT, V19, P597, DOI 10.1080/15226514.2016.1244157"

"   DalCorso G, 2019, INT J MOL SCI, V20, DOI 10.3390/ijms20143412"
"   Das J, 2018, SCI TOTAL ENVIRON, V624, P1106, DOI 10.1016/j.scitotenv.2017.12.157"
"   Diels L, 2003, HYDROMETALLURGY, V71, P235, DOI 10.1016/S0304-386X(03)00161-0"
"   Dimkpa C, 2008, CAN J MICROBIOL, V54, P163, DOI [10.1139/W07-130, 10.1139/w07-130]"
"   Dixit G, 2015, SCI REP-UK, V5, DOI 10.1038/srep16205"
"   Dotaniya M., 2016, Conserv. Agric., P225, DOI DOI 10.1007/978-981-10-2558-7_9"
"   Dotaniya M. L., 2014, Agricultural Reviews, V35, P153, DOI 10.5958/0976-0741.2014.00094.4"
"   Fashola MO, 2016, INT J ENV RES PUB HE, V13, DOI 10.3390/ijerph13111047"
"   Frankenberger WT, 2002, BOOK SOIL P, P363"
"   Friesl-Hanl W, 2009, ENVIRON GEOCHEM HLTH, V31, P581, DOI 10.1007/s10653-009-9256-3"
"   Gao XY, 2019, ENVIRON SCI TECHNOL, V53, P4959, DOI 10.1021/acs.est.8b07243"
"   Gauthier PT, 2014, AQUAT TOXICOL, V154, P253, DOI 10.1016/j.aquatox.2014.05.026"
"   Gavrilescu M., 2010, Dynamic Biochemistry, Process Biotechnology and Molecular Biology, V4, P1"
"   Gilis A, 1998, J IND MICROBIOL BIOT, V20, P61, DOI 10.1038/sj.jim.2900478"
"   Glick BR, 2005, FEMS MICROBIOL LETT, V251, P1, DOI 10.1016/j.femsle.2005.07.030"
"   Gontia-Mishra I, 2016, J PLANT GROWTH REGUL, V35, P1000, DOI 10.1007/s00344-016-9598-x"
"   Gopinath A., 2020, MODERN AGE WASTE WAT, P309, DOI DOI 10.1007/978-3-030-08283-3_15"
"   Gupta S, 2015, INT J ENVIRON SCI TE, V12, P995, DOI 10.1007/s13762-013-0484-9"
"   Gupta S, 2019, FRONT MICROBIOL, V10, DOI 10.3389/fmicb.2019.01506"
"   Hamzah A., 2016, INT J ENV AGR RES, V2, P8"
"   Hansda A, 2017, 3 BIOTECH, V7, DOI 10.1007/s13205-017-0757-y"
"   Harmsen J, 2007, J ENVIRON QUAL, V36, P1112, DOI 10.2134/jeq2006.0163"
"   Hassen W, 2018, FRONT MICROBIOL, V9, DOI 10.3389/fmicb.2018.00034"
"   He Jing, 2020, Microbiology and Biotechnology Letters, V48, P223, DOI [10.4014/mbl.1912.12011, 10.4014/mbl.1912.12011]"
"   He J, 2019, INT BIODETER BIODEGR, V140, P67, DOI 10.1016/j.ibiod.2019.03.012"
"   Hlihor RM, 2017, BIOMED RES INT, V2017, DOI 10.1155/2017/6327610"
"   Hu BF, 2017, INT J ENV RES PUB HE, V14, DOI 10.3390/ijerph14091042"
"   Huang JL, 2016, J GEN APPL MICROBIOL, V62, P258, DOI 10.2323/jgam.2016.04.007"
"   Igiri BE, 2018, J TOXICOL-US, V2018, DOI 10.1155/2018/2568038"
"   Iqbal HH, 2016, Bull. Environ. Stud, V1, P10"
"   Jing XB, 2012, CAN J MICROBIOL, V58, P45, DOI [10.1139/W11-110, 10.1139/w11-110]"
"   Joutey N.T., 2013, BOOK AGR BIOLOGICAL, DOI [DOI 10.5772/56194, 10.1061/40745(146)46, DOI 10.1061/40745(146)46]"
"   Kamran M, 2020, J PLANT GROWTH REGUL, V39, P266, DOI 10.1007/s00344-019-09980-3"
"   Karthik C, 2017, J HAZARD MATER, V333, P42, DOI 10.1016/j.jhazmat.2017.03.037"
"   Khanna K, 2019, SCI REP-UK, V9, DOI 10.1038/s41598-019-41899-3"
"   KINGSLEY DM, 1994, GENE DEV, V8, P133, DOI 10.1101/gad.8.2.133"
"   Konkolewska A, 2020, ENVIRON SCI POLLUT R, V27, P13809, DOI 10.1007/s11356-020-07885-3"
"   Kotoky R, 2019, ENVIRON SUSTAIN, V2, P135, DOI [10.1007/s42398-019-00055-3, 10.1007/s42398-019-00055-3, DOI 10.1007/S42398-019-00055-3]"
"   KRUMME ML, 1994, ENVIRON SCI TECHNOL, V28, P1134, DOI 10.1021/es00055a025"
"   Kumar A., 2011, International Journal of Environmental Sciences, V1, P1079"
"   Kumar PS, 2018, ENERGY ENV SUSTAIN, P165, DOI 10.1007/978-981-10-7485-1_9"
"   Kumar P, 2019, SCI REP-UK, V9, DOI 10.1038/s41598-018-37396-8"
"   Lepp NW, 2012, Effect of heavy metal pollution on plants: metals in the environment, V2"
"   Li DD, 2017, J ENVIRON MANAGE, V196, P8, DOI 10.1016/j.jenvman.2017.02.076"
"   Li XJ, 2018, RSC ADV, V8, P30902, DOI 10.1039/c8ra06270f"
"   Liu XX, 2019, J PLANT GROWTH REGUL, V38, P1314, DOI 10.1007/s00344-019-09935-8"
"   Ma Y, 2016, FRONT PLANT SCI, V7, DOI 10.3389/fpls.2016.00918"
"   Mahatma Gandhi Chitrakoot Gramodaya Vishwavidhyalaya Chitrakoot (M.P.) India, 2016, International Journal of Pure & Applied Bioscience, V4, P88, DOI [10.18782/2320-7051.2424, 10.18782/2320-7051.2424, DOI 10.18782/2320-7051.2424]"
"   Mahrukh Zubair Mahrukh Zubair, 2016, Environmental Technology Reviews, V5, P112, DOI [10.1080/21622515.2016.1259358, 10.1080/21622515.2016.1259358]"
"   Malik A, 2004, ENVIRON INT, V30, P261, DOI 10.1016/j.envint.2003.08.001"
"   Manara A., 2012, Plants and heavy metals, SpringerBriefs in Biometals, P27"
"   Megharaj M, 2017, MICROB BIOTECHNOL, V10, P1244, DOI 10.1111/1751-7915.12840"
"   Mishra A, 2013, CRIT REV ENV SCI TEC, V43, P1162, DOI 10.1080/10934529.2011.627044"
"   Mishra SK, 2017, ANTON LEEUW INT J G, V110, P253, DOI 10.1007/s10482-016-0796-0"
"   Mitra S, 2018, ECOTOX ENVIRON SAFE, V156, P183, DOI 10.1016/j.ecoenv.2018.03.001"
"   Mohamed RM, 2012, J BASIC MICROB, V52, P53, DOI 10.1002/jobm.201100133"
"   Mu'minah Mu'minah, 2015, International Journal of Current Microbiology and Applied Sciences, V4, P341"
"   Mwandira W, 2020, SCI REP-UK, V10, DOI 10.1038/s41598-020-78187-4"
"   Naik MM, 2013, ECOTOX ENVIRON SAFE, V98, P1, DOI 10.1016/j.ecoenv.2013.09.039"
"   Nawar N., 2013, AJIS, V2, P85, DOI [DOI 10.5901/AJIS.2013.V2N6P85, 10.5901/ajis.2013.v2n6p85.]"
"   Nikolaides A, 2015, HIGHWAY ENGINEERING: PAVEMENTS, MATERIALS AND CONTROL OF QUALITY, P1"
"   Okereafor U, 2020, INT J ENV RES PUB HE, V17, DOI 10.3390/ijerph17072204"
"   Parnell JJ, 2016, FRONT PLANT SCI, V7, DOI 10.3389/fpls.2016.01110"
"   Pathak A, 2009, J ENVIRON MANAGE, V90, P2343, DOI 10.1016/j.jenvman.2008.11.005"
"   Pingoliya KK, 2015, LEGUME RES, V38, P558, DOI 10.5958/0976-0571.2015.00137.X"
"   Prasad MNV, 2003, RUSS J PLANT PHYSL+, V50, P686, DOI 10.1023/A:1025604627496"
"   Radice F, 2006, CURR MICROBIOL, V52, P395, DOI 10.1007/s00284-005-0355-9"
"   Rahman A, 2015, J ENVIRON SCI HEAL A, V50, P1136, DOI 10.1080/10934529.2015.1047670"
"   Rahman Z, 2019, ENVIRON MONIT ASSESS, V191, DOI 10.1007/s10661-019-7528-7"
"   Raiesi F, 2019, ECOTOX ENVIRON SAFE, V168, P221, DOI 10.1016/j.ecoenv.2018.10.079"
"   Rajendran S. K., 2020, International Journal of Agricultural Technology, V16, P365"
"   Rajkumar M, 2010, TRENDS BIOTECHNOL, V28, P142, DOI 10.1016/j.tibtech.2009.12.002"
"   Rehan M., 2019, Environmental Chemistry and Recent Pollution Control Approaches p, P145, DOI [10.5772/INTECHOPEN.88339, DOI 10.5772/INTECHOPEN.88339, 10.5772/intechopen.88339]"
"   Rehman K, 2018, J CELL BIOCHEM, V119, P157, DOI 10.1002/jcb.26234"
"   Rizvi A, 2020, ENVIRON MONIT ASSESS, V192, DOI 10.1007/s10661-020-08758-5"
"   Rodrigues AAZ, 2017, J ENVIRON SCI HEAL B, V52, P850, DOI 10.1080/03601234.2017.1359049"
"   Sagar A, 2020, INDIAN J EXP BIOL, V58, P115"

"   Salam LB, 2019, B NATL RES CENT, V43, DOI [10.1186/s42269-019-0208-5, 10.1186/s42269-019-0208-5, DOI 10.1186/S42269-019-0208-5]"
"   Sayqal A, 2021, APPL BIONICS BIOMECH, V2021, DOI 10.1155/2021/1609149"
"   Sayyed RZ, 2019, ENVIRON SUSTAIN, V2, P117, DOI [10.1007/s42398-019-00070-4, 10.1007/s42398-019-00070-4, DOI 10.1007/S42398-019-00070-4, 10.1007/s42398-019, DOI 10.1007/S42398-019]"
"   Schalk IJ, 2011, ENVIRON MICROBIOL, V13, P2844, DOI 10.1111/j.1462-2920.2011.02556.x"
"   Seneviratne M, 2017, AGRO-ENVIRONMENTAL SUSTAINABILITY, VOL 2: MANAGING ENVIRONMENTAL POLLUTION, P147, DOI 10.1007/978-3-319-49727-3_8"
"   Sharma H.D., 2004, GEOENVIRONMENTAL ENG"
"   Sharma I., 2020, Trace Metals in the Environment-New Approaches and Recent Advances, P12, DOI [10.5772/intechopen.90453, DOI 10.5772/INTECHOPEN.90453]"
"   Sheoran V, 2011, CRIT REV ENV SCI TEC, V41, P168, DOI 10.1080/10643380902718418"
"   Sheoran V, 2016, PEDOSPHERE, V26, P148, DOI 10.1016/S1002-0160(15)60032-7"
"   Shishir TA, 2019, POLLUTION, V5, P555, DOI 10.22059/poll.2019.272339.558"
"   Silver S, 2005, J IND MICROBIOL BIOT, V32, P587, DOI 10.1007/s10295-005-0019-6"
"   Singh JS, 2011, GENE, V480, P1, DOI 10.1016/j.gene.2011.03.001"
"   Sostaric TD, 2018, J MOL LIQ, V259, P340, DOI 10.1016/j.molliq.2018.03.055"
"   Stearns JC, 2005, PLANT PHYSIOL BIOCH, V43, P701, DOI 10.1016/j.plaphy.2005.05.010"
"   Sung CY, 2018, LANDSCAPE URBAN PLAN, V177, P38, DOI 10.1016/j.landurbplan.2018.04.013"
"   Taamalli M, 2014, ECOL ENG, V71, P623, DOI 10.1016/j.ecoleng.2014.08.013"
"   Tara N, 2019, J CLEAN PROD, V217, P541, DOI 10.1016/j.jclepro.2019.01.258"
"   Tighadouini S, 2018, NEW J CHEM, V42, P13229, DOI 10.1039/c8nj01918e"
"   Vardar G, 2005, APPL MICROBIOL BIOT, V66, P696, DOI 10.1007/s00253-004-1685-4"
"   Velkova Z, 2018, ENG LIFE SCI, V18, P871, DOI 10.1002/elsc.201800017"
"   Weyens N, 2009, TRENDS BIOTECHNOL, V27, P591, DOI 10.1016/j.tibtech.2009.07.006"
"   Whelan MJ, 2015, CHEMOSPHERE, V131, P232, DOI 10.1016/j.chemosphere.2014.10.088"
"   Wood TK, 2008, CURR OPIN BIOTECH, V19, P572, DOI 10.1016/j.copbio.2008.10.003"
"   Wu CH, 2006, APPL ENVIRON MICROB, V72, P1129, DOI 10.1128/AEM.72.2.1129-1134.2006"
"   Wu XY, 2016, ENVIRON SCI POLLUT R, V23, P8244, DOI 10.1007/s11356-016-6333-x"
"   Xu CR, 2017, CHEMOSPHERE, V173, P622, DOI 10.1016/j.chemosphere.2017.01.005"
"   Yan A, 2020, FRONT PLANT SCI, V11, DOI 10.3389/fpls.2020.00359"
"   Yan GX, 2016, CLEAN-SOIL AIR WATER, V44, P984, DOI 10.1002/clen.201500571"
"   Yang PP, 2017, J MATER CHEM A, V5, P17933, DOI 10.1039/c6ta10022h"
"   Zhao YP, 2018, SCI TOTAL ENVIRON, V621, P772, DOI 10.1016/j.scitotenv.2017.11.189"
"   Zhou J, 2018, PLOS ONE, V13, DOI 10.1371/journal.pone.0191139"
"   Zhu XJ, 2016, ENVIRON POLLUT, V219, P149, DOI 10.1016/j.envpol.2016.10.047"
NR 132
TC 103
Z9 122
U1 23
U2 155
PU SPRINGER
PI NEW YORK
"PA ONE NEW YORK PLAZA, SUITE 4600, NEW YORK, NY, UNITED STATES"
SN 0273-2289
EI 1559-0291
J9 APPL BIOCHEM BIOTECH
JI Appl. Biochem. Biotechnol.
PD MAY
PY 2024
VL 196
IS 5
SI SI
BP 2928
EP 2956
DI 10.1007/s12010-023-04545-3
EA APR 2023
PG 29
WC Biochemistry & Molecular Biology; Biotechnology & Applied Microbiology
WE Science Citation Index Expanded (SCI-EXPANDED)
SC Biochemistry & Molecular Biology; Biotechnology & Applied Microbiology
GA SM3O9
UT WOS:000979683400002
PM 37097400
DA 2026-04-06
ER

PT J
"AU Ouyang, P"
"   Narayanan, M"
"   Shi, XJ"
"   Chen, X"
"   Li, ZL"
"   Luo, YM"
"   Ma, Y"
"AF Ouyang, Peng"
"   Narayanan, Mathiyazhagan"
"   Shi, Xiaojun"
"   Chen, Xinping"
"   Li, Zhenlun"
"   Luo, Yongming"
"   Ma, Ying"
TI Integrating biochar and bacteria for sustainable remediation of
   metal-contaminated soils

SO BIOCHAR
LA English
DT Review
DE Biochar; Heavy metals; Bioremediation; Plant-beneficial bacteria; Soil
   pollution
ID MECHANISMS; REDUCTION; REMOVAL; IMPROVE; CR(VI); STRAIN
"AB Due to anthropogenic activities, heavy metal (HM) pollution in soils has increased, resulting in severe ecological problems and posing a constant threat to human health. Among various remediation methods, bacterial remediation is a relatively clean, efficient, and minimally negative approach. However, bacterial agents face multiple environmental stresses, making them challenging to achieve long-lasting and stable restoration effects. To address this issue, supportive organic substances such as biochar can be added to the soil with bacteria. According to bibliometric studies, integrating biochar and bacteria is extensively researched and widely used for HM-contaminated soil remediation. By integrating biochar and bacteria, heavy metals in the soil can be remediated, and soil conditions can be improved over time. Bacteria can also better promote plant growth or contribute effectively to phytoremediation processes when assisted by biochar. However, the remediation agents integrating biochar and bacteria are still some distance away from large-scale use because of their high cost and possible environmental problems. Therefore, further discussion on the interaction between biochar and bacteria and the integration approach, along with their remediation efficiency and environmental friendliness, is needed to achieve sustainable remediation of HM- contaminated soils by integrating biochar and bacteria. This paper discusses the potential mechanisms of biochar-bacteria-metal interactions, current advancements in biochar-bacteria combinations for HM-contaminated soil treatment, and their application in sustainable remediation, analyzes the interaction between biochar and bacteria and compares the remediation effect of different ways and feedstocks to integrate biochar and bacteria. Finally, future directions of biochar-bacteria combinations are presented, along with evidence and strategies for improving their commercialization and implementation."
"C1 [Ouyang, Peng; Shi, Xiaojun; Chen, Xinping; Li, Zhenlun; Ma, Ying] Southwest Univ, Coll Resources & Environm, Chongqing 400716, Peoples R China."
"   [Narayanan, Mathiyazhagan] Saveetha Inst Med & Techn Sci, Saveetha Sch Engn, Div Res & Innovat, Dept Biotechnol, Chennai 602105, Tamil Nadu, India."
"   [Luo, Yongming] Chinese Acad Sci, Inst Soil Sci, Key Lab Soil Environm & Pollut Remediat, Nanjing 210008, Peoples R China."
C3 Southwest University - China; Saveetha Institute of Medical & Technical
   Science; Saveetha School of Engineering; Chinese Academy of Sciences;
"   Nanjing Institute of Soil Science, CAS"
"RP Ma, Y (corresponding author), Southwest Univ, Coll Resources & Environm, Chongqing 400716, Peoples R China."
EM cathymaying@hotmail.com
"RI Ma, Ying/J-1152-2014; Narayanan, Mathiyazhagan/AAN-2571-2021; Ouyang,"
   Peng/HJP-7292-2023
"OI Ouyang, Peng/0000-0003-0117-2755"
FU Fundamental Research Funds for the Central Universities [SWU 020010]
"FX This work is carried out at the College of Resources and Environment,"
"   Southwest University, supported by the Fundamental Research Funds for"
   the Central Universities (No. SWU 020010).
"CR Ahmad Z, 2018, J CLEAN PROD, V180, P437, DOI 10.1016/j.jclepro.2018.01.133"
"   Bali AS, 2021, CHEMOSPHERE, V283, DOI 10.1016/j.chemosphere.2021.131050"
"   Pascual MB, 2020, FEMS MICROBIOL ECOL, V96, DOI 10.1093/femsec/fiaa133"
"   Bolan S, 2023, SCI TOTAL ENVIRON, V886, DOI 10.1016/j.scitotenv.2023.163968"
"   Brichi L, 2023, SOIL USE MANAGE, V39, P686, DOI 10.1111/sum.12892"
"   Cao MY, 2023, ENVIRON RES, V217, DOI 10.1016/j.envres.2022.114924"
"   Chen X, 2021, WATER AIR SOIL POLL, V232, DOI 10.1007/s11270-021-05206-z"
"   Chen YY, 2021, FRONT MICROBIOL, V12, DOI 10.3389/fmicb.2021.641913"
"   Cheng C, 2020, SCI TOTAL ENVIRON, V740, DOI 10.1016/j.scitotenv.2020.139972"
"   Chiaranunt P, 2023, PLANTS-BASEL, V12, DOI 10.3390/plants12020400"
"   Orozco-Mosqueda MD, 2021, AGRONOMY-BASEL, V11, DOI 10.3390/agronomy11061167"
"   Dhaliwal SS, 2020, ENVIRON SCI POLLUT R, V27, P1319, DOI 10.1007/s11356-019-06967-1"
"   Gholizadeh M, 2021, J ENVIRON CHEM ENG, V9, DOI 10.1016/j.jece.2021.105830"
"   Giovanella P, 2016, NEW BIOTECHNOL, V33, P216, DOI 10.1016/j.nbt.2015.05.006"
"   Godlewska P, 2021, J HAZARD MATER, V403, DOI 10.1016/j.jhazmat.2020.123833"
"   Henao SG, 2021, FRONT ENV SCI-SWITZ, V9, DOI 10.3389/fenvs.2021.604216"
"   Gou ZC, 2023, ENVIRON POLLUT, V317, DOI 10.1016/j.envpol.2022.120790"
"   Guo MX, 2020, FRONT ENV SCI-SWITZ, V8, DOI 10.3389/fenvs.2020.521512"
"   Guo SS, 2022, BIORESOURCE TECHNOL, V350, DOI 10.1016/j.biortech.2022.126901"
"   Harindintwali JD, 2020, ECOTOX ENVIRON SAFE, V204, DOI 10.1016/j.ecoenv.2020.111020"
"   Hu FN, 2021, GEODERMA, V403, DOI 10.1016/j.geoderma.2021.115276"
"   Hua Y, 2020, BIORESOURCE TECHNOL, V300, DOI 10.1016/j.biortech.2019.122708"
"   Huang F, 2020, J CLEAN PROD, V272, DOI 10.1016/j.jclepro.2020.122743"
"   Huang K, 2023, PLANT SOIL ENVIRON, V69, P45, DOI 10.17221/348/2022-PSE"
"   Ji XW, 2022, SCI TOTAL ENVIRON, V811, DOI 10.1016/j.scitotenv.2021.152112"
"   Kapoor A, 2022, J PLANT NUTR, V45, P2380, DOI 10.1080/01904167.2022.2027980"
"   Kari A, 2021, APPL SOIL ECOL, V160, DOI 10.1016/j.apsoil.2020.103856"
"   Konczak M, 2020, SCI TOTAL ENVIRON, V723, DOI 10.1016/j.scitotenv.2020.137796"
"   Kuzyakov Y, 2009, SOIL BIOL BIOCHEM, V41, P210, DOI 10.1016/j.soilbio.2008.10.016"
"   Lai WW, 2022, AGRONOMY-BASEL, V12, DOI 10.3390/agronomy12051003"
"   Li HX, 2019, GEODERMA, V352, P96, DOI 10.1016/j.geoderma.2019.06.006"
"   Li XP, 2020, SCI TOTAL ENVIRON, V711, DOI 10.1016/j.scitotenv.2019.134847"
"   Liang S, 2023, PLANTS-BASEL, V12, DOI 10.3390/plants12020284"
"   Liao XL, 2022, GEODERMA, V411, DOI 10.1016/j.geoderma.2021.115692"
"   Lin H, 2023, INT BIODETER BIODEGR, V178, DOI 10.1016/j.ibiod.2022.105544"
"   Liu LC, 2021, J HAZARD MATER, V405, DOI 10.1016/j.jhazmat.2020.124246"
"   Liu YB, 2023, SCI TOTAL ENVIRON, V858, DOI 10.1016/j.scitotenv.2022.160158"
"   Lyu C, 2022, ENVIRON POLLUT, V310, DOI 10.1016/j.envpol.2022.119818"
"   Ma H, 2020, J HAZARD MATER, V388, DOI 10.1016/j.jhazmat.2020.122065"
"   Ma Y, 2011, BIOTECHNOL ADV, V29, P248, DOI 10.1016/j.biotechadv.2010.12.001"

"   Ma Y, 2022, FRONT PLANT SCI, V13, DOI 10.3389/fpls.2022.1024243"
"   Ma Y, 2020, FRONT PLANT SCI, V11, DOI 10.3389/fpls.2020.591911"
"   Ma Y, 2016, FRONT PLANT SCI, V7, DOI 10.3389/fpls.2016.00918"
"   Ma Y, 2015, J ENVIRON MANAGE, V156, P62, DOI 10.1016/j.jenvman.2015.03.024"
"   Narayanan M, 2022, FRONT MICROBIOL, V13, DOI 10.3389/fmicb.2022.929730"
"   Nivetha N, 2023, CHEMOSPHERE, V312, DOI 10.1016/j.chemosphere.2022.137099"
"   Ouyang P, 2022, CHEMOSPHERE, V294, DOI 10.1016/j.chemosphere.2022.133702"
"   Paquete CM, 2022, BIORESOURCE TECHNOL, V347, DOI 10.1016/j.biortech.2022.126705"
"   Pei JM, 2021, GEODERMA, V382, DOI 10.1016/j.geoderma.2020.114710"
"   Peng JY, 2022, SCI TOTAL ENVIRON, V816, DOI 10.1016/j.scitotenv.2021.151632"
"   Prodana M, 2019, ECOTOX ENVIRON SAFE, V174, P120, DOI 10.1016/j.ecoenv.2019.02.044"
"   Qiu MQ, 2022, BIOCHAR, V4, DOI 10.1007/s42773-022-00146-1"
"   Qu JH, 2022, J HAZARD MATER, V434, DOI 10.1016/j.jhazmat.2022.128841"
"   Qu JH, 2022, J HAZARD MATER, V423, DOI 10.1016/j.jhazmat.2021.127043"
"   Quan GX, 2020, J HAZARD MATER, V382, DOI 10.1016/j.jhazmat.2019.121071"
"   Rafiq MK, 2020, SCI TOTAL ENVIRON, V717, DOI 10.1016/j.scitotenv.2019.135296"
"   Rajendran S, 2022, CHEMOSPHERE, V287, DOI 10.1016/j.chemosphere.2021.132369"
"   Rostami S, 2019, CHEMOSPHERE, V220, P818, DOI 10.1016/j.chemosphere.2018.12.203"
"   Sabir A, 2020, J ENVIRON MANAGE, V265, DOI 10.1016/j.jenvman.2020.110522"
"   Simiele M, 2021, ENVIRON EXP BOT, V182, DOI 10.1016/j.envexpbot.2020.104335"
"   Sreedevi PR, 2022, J WATER PROCESS ENG, V48, DOI 10.1016/j.jwpe.2022.102884"
"   Tan LS, 2020, J SOIL SEDIMENT, V20, P340, DOI 10.1007/s11368-019-02398-0"
"   Tomczyk A, 2020, REV ENVIRON SCI BIO, V19, P191, DOI 10.1007/s11157-020-09523-3"
"   Tu C, 2020, ENVIRON INT, V137, DOI 10.1016/j.envint.2020.105576"
"   Ullah S, 2023, MICROORGANISMS, V11, DOI 10.3390/microorganisms11020527"
"   Van der Zee FR, 2009, BIOTECHNOL ADV, V27, P256, DOI 10.1016/j.biotechadv.2009.01.004"
"   Wan J, 2022, J ENVIRON CHEM ENG, V10, DOI 10.1016/j.jece.2022.108403"
"   Wan J, 2020, FUEL, V269, DOI 10.1016/j.fuel.2020.117142"
"   Wang HH, 2022, ECOLOGIES-BASEL, V3, P539, DOI 10.3390/ecologies3040040"
"   Wang JL, 2019, J CLEAN PROD, V227, P1002, DOI 10.1016/j.jclepro.2019.04.282"
"   Wang LW, 2020, ENVIRON SCI TECHNOL, V54, P14797, DOI 10.1021/acs.est.0c04033"
"   Wang L, 2021, J HAZARD MATER, V414, DOI 10.1016/j.jhazmat.2021.125494"
"   Wang L, 2021, SCI TOTAL ENVIRON, V750, DOI 10.1016/j.scitotenv.2020.141672"
"   Wang YY, 2021, ECOTOX ENVIRON SAFE, V207, DOI 10.1016/j.ecoenv.2020.111294"
"   Wang Y, 2022, FRONT MICROBIOL, V13, DOI 10.3389/fmicb.2022.966226"
"   Wu B, 2019, SCI TOTAL ENVIRON, V686, P719, DOI 10.1016/j.scitotenv.2019.06.041"
"   Wu JW, 2021, SCI TOTAL ENVIRON, V754, DOI 10.1016/j.scitotenv.2020.142150"
"   Xia X, 2021, J HAZARD MATER, V401, DOI [10.1016/j.jhazmat.2020.143685, 10.1016/j.jhazmat.2020.123685]"
"   Xia X, 2018, J HAZARD MATER, V342, P499, DOI 10.1016/j.jhazmat.2017.08.051"
"   Xu JC, 2019, CHEMOSPHERE, V237, DOI 10.1016/j.chemosphere.2019.124540"
"   Xu XY, 2019, ENVIRON POLLUT, V244, P423, DOI 10.1016/j.envpol.2018.10.068"
"   Xu YL, 2018, SCI TOTAL ENVIRON, V621, P148, DOI 10.1016/j.scitotenv.2017.11.214"
"   Yan S, 2022, SCI TOTAL ENVIRON, V802, DOI 10.1016/j.scitotenv.2021.149835"
"   Yang Q, 2021, BIOCHAR, V3, P23, DOI 10.1007/s42773-021-00087-1"
"   Yin K, 2019, CHEM ENG J, V360, P1553, DOI [10.1016/j.cej.2018.10.226, 10.1016/j.jhazmat.2024.134410]"
"   Zeba N, 2022, PLOS ONE, V17, DOI 10.1371/journal.pone.0265663"
"   Zha RD, 2015, CATENA, V125, P183, DOI 10.1016/j.catena.2014.10.026"
"   Zhang GX, 2020, GEODERMA, V375, DOI 10.1016/j.geoderma.2020.114497"
"   Zhang H, 2020, CHEMOSPHERE, V261, DOI 10.1016/j.chemosphere.2020.127694"
"   Zhang KK, 2019, FRONT ENV SCI ENG, V13, DOI 10.1007/s11783-019-1106-7"
"   Zhang MY, 2023, APPL SOIL ECOL, V182, DOI 10.1016/j.apsoil.2022.104700"
"   Zhang MY, 2021, J CLEAN PROD, V292, DOI 10.1016/j.jclepro.2021.126062"
"   Zhang WW, 2012, APPL MICROBIOL BIOT, V93, P1305, DOI 10.1007/s00253-011-3454-5"
"   Zhang XQ, 2019, ENVIRON SCI TECHNOL, V53, P6660, DOI 10.1021/acs.est.9b01345"
"   Zhang YF, 2021, CATENA, V202, DOI 10.1016/j.catena.2021.105284"
"   Zhao B, 2018, J CLEAN PROD, V174, P977, DOI 10.1016/j.jclepro.2017.11.013"
"   Zhao JT, 2021, BIOTECHNOL ADV, V53, DOI 10.1016/j.biotechadv.2020.107682"
"   Zhao L, 2020, WATER RES, V172, DOI 10.1016/j.watres.2020.115494"
"   Zheng XM, 2022, J HAZARD MATER, V438, DOI 10.1016/j.jhazmat.2022.129557"
"   Zhou XG, 2022, CHEMOSPHERE, V307, DOI 10.1016/j.chemosphere.2022.136138"
"   Zhu XM, 2017, ENVIRON POLLUT, V227, P98, DOI [10.1016/j.envpol.2017.04.032,
10.1016/j.envPol.2017.04.032]"
NR 101
TC 53
Z9 63
U1 49
U2 253
PU SPRINGER SINGAPORE PTE LTD
PI SINGAPORE
"PA 152 Beach Road, #21-01 Gateway East, SINGAPORE, SINGAPORE"
SN 2524-7972
EI 2524-7867
J9 BIOCHAR
JI Biochar
PD OCT
PY 2023
VL 5
IS 1
AR 63
DI 10.1007/s42773-023-00265-3
PG 19
WC Environmental Sciences; Soil Science
WE Science Citation Index Expanded (SCI-EXPANDED)
SC Environmental Sciences & Ecology; Agriculture

GA LJ8U3
UT WOS:001186531200001
"OA Green Submitted, gold"
DA 2026-04-06
ER

PT J
"AU Ma, LL"
"   Li, ZT"
"   Qiao, MD"
"   Liu, J"
"   Jia, B"
"   Yang, B"
"   Liu, YC"
"AF Ma, Lili"
"   Li, Zetong"
"   Qiao, Mengdan"
"   Liu, Jie"
"   Jia, Bing"
"   Yang, Bing"
"   Liu, Yucheng"
TI Enhancing electrokinetic remediation of TPH-Cr(VI) co-contaminated soils
   with biochar-immobilized bacteria as biological permeable reactive
   barriers
SO CHEMICAL ENGINEERING JOURNAL
LA English
DT Article
DE Soil remediation; Enhanced electrokinetics; Biochar-immobilized
   bacteria; Biological permeable reactive barriers; Petroleum
   hydrocarbons; Cr(VI)
ID PETROLEUM-HYDROCARBONS; COMMUNITY STRUCTURE; DEGRADING BACTERIA;
   HEAVY-METALS; BIOREMEDIATION; REMOVAL; OIL; BIOAUGMENTATION;
   BIODEGRADATION; TECHNOLOGIES
"AB The co-contamination soil caused by petroleum pollution poses a significant challenge for remediation. The complexity of pollution composition and interaction increases the difficulty of the remediation process. This study established a methodology that employs biochar-immobilized bacteria to enhance the electrokinetic (EK) remediation technique. A 25-day experiment was conducted in the laboratory to remediate co-contaminated soil with total petroleum hydrocarbons(TPH) (9252.51 mg/kg) and Cr (VI) (147.17 mg/kg). The results indicate that adding biochar-immobilized bacteria leads to the most effective remediation performance in the EK system. Compared to the standalone EK remediation, TPH removal efficiency increased by 22.57 %, and the removal rate of Cr (VI) increased by 53.15 %. The study also examined the response of microbial communities in the remediation system. The study results on microbial diversity reveal that introducing the EK system reduces the diversity of soil microbial communities. In contrast, incorporating biochar-immobilized bacteria impacts the microbial community structure in the soil, enhancing microbial communities' stability. This study demonstrates the effectiveness of adding biochar-immobilized bacteria in enhancing EK remediation of co-contaminated soil and its impact on microbial community structure."
"C1 [Ma, Lili; Li, Zetong; Qiao, Mengdan; Yang, Bing; Liu, Yucheng] Southwest Petr Univ, Res Inst Ind Hazardous Waste Disposal & Resource U, Coll Chem & Chem Engn, Chengdu 610500, Peoples R China."
"   [Liu, Jie] Lanzhou Univ, Ctr Grassland Microbiome, State Key Lab Herbage Improvement & Grassland Agro, Lanzhou 730000, Peoples R China."
"   [Jia, Bing] Chinese Acad Sci, Key Lab Ecohydrol Inland River Basin, Northwest Inst Ecoenvironm & Resources, Lanzhou 730000, Peoples R China."
C3 Southwest Petroleum University; Lanzhou University; Chinese Academy of
   Sciences
"RP Ma, LL; Liu, YC (corresponding author), Southwest Petr Univ, Res Inst Ind Hazardous Waste Disposal & Resource U, Coll Chem & Chem Engn, Chengdu 610500, Peoples R China."
EM swpumall@163.com; rehuo2013@sina.cn
"RI Liu, Jie/GRN-8750-2022; YANG, BING/O-3051-2013; Ma, Lili/NWI-0472-2025;"
"   Jia, Binghao/AAL-6374-2020"
"OI Liu, Jie/0000-0002-3849-6414; YANG, BING/0000-0002-9749-1349; "
FU Natural Science Foundation of Sichuan Province of China [2022NSFSC0241];
   Qilian Mountains Eco-environment Research Center in Gansu Province
   [QLS202004]; Science and Technology Plan of Gansu Province [21JR7RA522]
FX The authors are grateful to the financial support from the Natural
   Science Foundation of Sichuan Province of China (2022NSFSC0241) and the
   Qilian Mountains Eco-environment Research Center in Gansu Province
   (QLS202004) . Science and Technology Plan of Gansu Province (21JR7RA522)
   .
"CR Agnello AC, 2016, SCI TOTAL ENVIRON, V563, P693, DOI 10.1016/j.scitotenv.2015.10.061"
"   Alisi C, 2009, SCI TOTAL ENVIRON, V407, P3024, DOI 10.1016/j.scitotenv.2009.01.011"
"   Alshawabkeh AN, 2009, SEP SCI TECHNOL, V44, P2171, DOI 10.1080/01496390902976681"
"   ALSHAWABKEH AN, 1992, J ENVIRON SCI HEAL A, VA27, P1835, DOI 10.1080/10934529209375828"
"   Assil Z, 2021, SCI TOTAL ENVIRON, V762, DOI 10.1016/j.scitotenv.2020.143084"
"   Cang L, 2013, CHEMOSPHERE, V90, P2326, DOI 10.1016/j.chemosphere.2012.10.062"
"   Cao XF, 2022, J HAZARD MATER, V426, DOI 10.1016/j.jhazmat.2021.128096"
"   Cavaliere M, 2017, ENVIRON MICROBIOL, V19, P2949, DOI 10.1111/1462-2920.13767"
"   Cavazzoli S, 2022, ENVIRON POLLUT, V307, DOI 10.1016/j.envpol.2022.119569"
"   Cha??neau CH, 2005, SOIL BIOL BIOCHEM, V37, P1490, DOI 10.1016/j.soilbio.2005.01.012"
"   Chang JH, 2006, J HAZARD MATER, V129, P186, DOI 10.1016/j.jhazmat.2005.08.030"
"   Chuaphasuk C, 2019, ENVIRON SCI POLLUT R, V26, P23679, DOI 10.1007/s11356-019-05661-6"
"   CLARKE KR, 1993, AUST J ECOL, V18, P117, DOI 10.1111/j.1442-9993.1993.tb00438.x"
"   Coyte KZ, 2015, SCIENCE, V350, P663, DOI 10.1126/science.aad2602"
"   Ejaz M, 2021, APPL SCI-BASEL, V11, DOI 10.3390/app11083504"
"   Fan RJ, 2022, J HAZARD MATER, V422, DOI 10.1016/j.jhazmat.2021.126959"

"   Fan RJ, 2020, SCI TOTAL ENVIRON, V748, DOI 10.1016/j.scitotenv.2020.142463"
"   Floris B, 2017, INORG CHIM ACTA, V455, P429, DOI 10.1016/j.ica.2016.04.003"
"   Ghobadi R, 2020, CHEMOSPHERE, V252, DOI 10.1016/j.chemosphere.2020.126607"
"   Gidudu B, 2022, MOLECULES, V27, DOI 10.3390/molecules27217381"
"   Guo SH, 2014, CHEMOSPHERE, V109, P226, DOI 10.1016/j.chemosphere.2014.02.007"
"   He CQ, 2021, CHEM ENG J, V407, DOI 10.1016/j.cej.2020.126923"
"   HERMAN DC, 1995, ENVIRON SCI TECHNOL, V29, P2280, DOI 10.1021/es00009a019"
"   Hong Y, 2022, SCI TOTAL ENVIRON, V851, DOI 10.1016/j.scitotenv.2022.158033"
"   Huang DQ, 2012, INT J ELECTROCHEM SC, V7, P4528"
"   Huang YJ, 2022, ENVIRON SCI POLLUT R, V29, P44893, DOI 10.1007/s11356-022-18972-y"
"   IJAH U J J, 1992, Waste Management, V12, P55, DOI 10.1016/0956-053X(92)90009-8"
"   Jiao S, 2016, SOIL BIOL BIOCHEM, V98, P64, DOI 10.1016/j.soilbio.2016.04.005"
"   Kuznetsova AI, 2020, MICROBIOLOGY+, V89, P670, DOI 10.1134/S0026261720060119"
"   Lan JR, 2021, J HAZARD MATER, V420, DOI 10.1016/j.jhazmat.2021.126588"
"   Lan J, 2023, ENVIRON SCI ECOTECH, V16, DOI 10.1016/j.ese.2023.100278"
"   Lear G, 2004, SOIL BIOL BIOCHEM, V36, P1751, DOI 10.1016/j.soilbio.2004.04.032"
"   Li JX, 2011, CHEM ENG J, V166, P551, DOI 10.1016/j.cej.2010.11.018"
"   Li JY, 2022, J AGR FOOD CHEM, DOI 10.1021/acs.jafc.2c05612"
"   Li S, 2023, ENVIRON TECHNOL, V44, P3050, DOI 10.1080/09593330.2022.2049891"
"   Li YL, 2021, J ENVIRON CHEM ENG, V9, DOI 10.1016/j.jece.2021.105190"
"   Ling H, 2023, CHEMOSPHERE, V322, DOI 10.1016/j.chemosphere.2023.138225"
"   Liu Q, 2022, ENVIRON TECHNOL INNO, V28, DOI 10.1016/j.eti.2022.102835"
"   Lu L, 2014, ENVIRON SCI TECHNOL, V48, P4021, DOI 10.1021/es4057906"
"   Lu Q, 2020, CHEMOSPHERE, V254, DOI 10.1016/j.chemosphere.2020.126861"
"   Ma CZ, 2022, ENVIRON SCI POLLUT R, V29, P11966, DOI 10.1007/s11356-021-16357-1"
"   Ma LL, 2021, J HAZARD MATER, V405, DOI 10.1016/j.jhazmat.2020.124086"
"   Ma YJ, 2023, APPL SOIL ECOL, V182, DOI 10.1016/j.apsoil.2022.104718"
"   Ma YS, 2018, CHEM ENG J, V353, P410, DOI 10.1016/j.cej.2018.07.131"
"   Man ML, 2023, J WATER PROCESS ENG, V55, DOI 10.1016/j.jwpe.2023.104115"
"   Mena E, 2016, ELECTROCHIM ACTA, V190, P294, DOI 10.1016/j.electacta.2015.12.133"
"   Mena E, 2015, J HAZARD MATER, V283, P131, DOI 10.1016/j.jhazmat.2014.08.069"
"   Neutel AM, 2002, SCIENCE, V296, P1120, DOI 10.1126/science.1068326"
"   NODA Y, 1984, PHYSIOL CHEM PHYS ME, V16, P263"
"   Qiao M., 2022, Applied Chemical Industry, V51, DOI [10.16581/j.cnki.issn1671-3206.20220322.011, DOI 10.16581/J.CNKI.ISSN1671-3206.20220322.011]"
"   Reddy KR, 2010, GEOTECH GEOL ENG, V28, P211, DOI 10.1007/s10706-008-9235-y"
"   Reddy KR, 2014, J ENVIRON ENG, V140, DOI 10.1061/(ASCE)EE.1943-7870.0000872"
"   Saini A, 2021, ENVIRON TECHNOL INNO, V23, DOI 10.1016/j.eti.2021.101585"
"   Schaller J, 2011, SCI TOTAL ENVIRON, V409, P3211, DOI 10.1016/j.scitotenv.2011.05.014"
"   Shu JC, 2015, ENVIRON SCI POLLUT R, V22, P16004, DOI 10.1007/s11356-015-4817-8"
"   Song LC, 2021, CHEMOSPHERE, V279, DOI 10.1016/j.chemosphere.2021.130427"
"   Tabinda A.B., 2023, Phytoremediation: management of environmental contaminants, VVolume 7, P231, DOI DOI 10.1007/978-3-031-17988-412"
"   Tang J, 2024, INT J ENVIRON AN CH, V104, P5999, DOI 10.1080/03067319.2022.2135999"
"   Tucci M, 2021, CHEM ENG J, V419, DOI 10.1016/j.cej.2021.130008"
"   Villalobos M, 2008, WATER AIR SOIL POLL, V194, P151, DOI 10.1007/s11270-008-9704-1"
"   Wang WB, 2023, ACS EST WATER, V3, P2024, DOI 10.1021/acsestwater.3c00188"
"   Wick LY, 2004, ENVIRON SCI TECHNOL, V38, P4596, DOI 10.1021/es0354420"
"   Wu CC, 2022, ENVIRON RES, V212, DOI 10.1016/j.envres.2022.113226"
"   Xiao J, 2023, Carbon Research, V2, DOI [10.1007/s44246-023-00053-5, 10.1007/s44246-023-00053-5, DOI 10.1007/S44246-023-00053-5]"
"   Xie N, 2021, J CLEAN PROD, V308, DOI 10.1016/j.jclepro.2021.127433"
"   Xiong BJ, 2017, CHEMOSPHERE, V182, P316, DOI 10.1016/j.chemosphere.2017.05.020"
"   Xu H, 2018, BIORESOURCE TECHNOL, V263, P266, DOI 10.1016/j.biortech.2018.05.015"
"   Xu M, 2023, BIOCHAR, V5, DOI 10.1007/s42773-023-00222-0"
"   Ye SJ, 2017, CRIT REV BIOTECHNOL, V37, P1062, DOI 10.1080/07388551.2017.1304357"
"   Yi ML, 2022, J HAZARD MATER, V431, DOI 10.1016/j.jhazmat.2022.128632"
"   Zhang BF, 2019, RSC ADV, V9, P35304, DOI 10.1039/c9ra06726d"
"   Zhang Y, 2023, BIOCHAR, V5, DOI 10.1007/s42773-023-00241-x"
"   Zhao SN, 2016, PROCEDIA ENVIRON SCI, V31, P274, DOI 10.1016/j.proenv.2016.02.036"
"   Zhong YQW, 2022, IMETA, V1, DOI 10.1002/imt2.18"
NR 74
TC 24
Z9 25
U1 16
U2 128
PU ELSEVIER SCIENCE SA
PI LAUSANNE
"PA PO BOX 564, 1001 LAUSANNE, SWITZERLAND"
SN 1385-8947
EI 1873-3212
J9 CHEM ENG J
JI Chem. Eng. J.
PD DEC 15
PY 2023
VL 478
AR 147301
DI 10.1016/j.cej.2023.147301
EA NOV 2023
PG 12
"WC Engineering, Environmental; Engineering, Chemical"
WE Science Citation Index Expanded (SCI-EXPANDED)
SC Engineering
GA AF3M8

UT WOS:001117011600001
DA 2026-04-06
ER

PT J
"AU Abbas, HMM"
"   Rais, U"
"   Altaf, MM"
"   Rasul, F"
"   Shah, A"
"   Tahir, A"
"   Nafees-Ur-Rehman, M"
"   Shaukat, M"
"   Sultan, H"
"   Zou, RL"
"   Khan, MN"
"   Nie, LX"
"AF Abbas, Hafiz Muhammad Mazhar"
"   Rais, Ummah"
"   Altaf, Muhammad Mohsin"
"   Rasul, Fahd"
"   Shah, Asad"
"   Tahir, Ashar"
"   Nafees-Ur-Rehman, Muhammad"
"   Shaukat, Muhammad"
"   Sultan, Haider"
"   Zou, Ruilong"
"   Khan, Mohammad Nauman"
"   Nie, Lixiao"
TI Microbial-inoculated biochar for remediation of salt and heavy metal
   contaminated soils
SO SCIENCE OF THE TOTAL ENVIRONMENT
LA English
DT Article
DE Microbial biochar; Co-application; Contaminated soil; Soil health;
   Adsorption; Evapotranspiration; Plant growth; Bioremediation;
   Sustainable agriculture
ID SHOOTING RANGE SOIL; SALINITY STRESS; PLANT-GROWTH; ENZYME-ACTIVITIES;
   PYROLYSIS TEMPERATURE; PYROLIGNEOUS SOLUTION; IMMOBILIZED BACTERIA;
   MYCORRHIZAL FUNGI; CR(VI) REDUCTION; ORGANIC-MATTER
"AB Numerous harmful contaminants (i.e. salt and heavy metals) have become major threats to soil and are being introduced into the soil through human and geological activities. These contaminants are raising global concerns about their toxic effects on food safety, human health and reclamation mechanisms. Microbial-inoculated biochar can improve soil environment by immobilizing and transforming contaminants in soil and altering the physico-chemical and biochemical properties of soil. In this review we will discuss the positive effects of microbial-modified biochar on physicochemical properties of contaminated soil. It can decrease the pH, EC while increase CEC, OM and other biochemical properties of soil. Additionally, we discuss the efficacy of biochar as a microbial carrier for salt and heavy metals-contaminated soil and plant growth in those soils. This review provides a better understanding of the potential of microbial biochar can be used for bioremediation of contaminated soil, which will help the researcher to modify biochar in a targeted way for specific applications."
"C1 [Abbas, Hafiz Muhammad Mazhar; Shah, Asad; Sultan, Haider; Zou, Ruilong; Khan, Mohammad Nauman; Nie, Lixiao] Hainan Univ, Sanya Inst Breeding & Multiplicat, Sch Breeding & Multiplicat, Sanya 572025, Peoples R China."
"   [Rais, Ummah] Islamia Univ Bahawalpur, Dept Zool, Bahawalpur 63100, Pakistan."
"   [Altaf, Muhammad Mohsin] Hainan Univ, Inst Trop Agr & Forestry, Haikou 570228, Hainan, Peoples R China."
"   [Rasul, Fahd] Univ Agr Faisalabad, Dept Agron, Faisalabad 38040, Punjab, Pakistan."
"   [Tahir, Ashar] Chinese Acad Trop Agr Sci, Rubber Res Inst, Danzhou 571700, Hainan, Peoples R China."
"   [Nafees-Ur-Rehman, Muhammad] Hainan Univ, Sch Life Sci, Lab Microbiol Engn, Haikou 570228, Hainan, Peoples R China."
"   [Shaukat, Muhammad] Allama Iqbal Open Univ Islamabad, Fac Sci, Dept Biol & Environm Sci, Islamabad 44310, Pakistan."
C3 Hainan University; Islamia University of Bahawalpur; Hainan University;
   University of Agriculture Faisalabad; Chinese Academy of Tropical
   Agricultural Sciences; Hainan University
"RP Nie, LX (corresponding author), Hainan Univ, Sanya Inst Breeding & Multiplicat, Sch Breeding & Multiplicat, Sanya 572025, Peoples R China."
EM 184268@hainanu.edu.cn; lxnie@hainanu.edu.cn
"RI Altaf, Muhammad/KCK-2763-2024; RASUL, Dr.FAHD/H-6016-2011; Khan,"
"   Mohammad/KFS-3277-2024; abbas, Muhammad/AAD-7630-2019; Shaukat,"
"   Muhammad/AAF-2976-2019; Sultan, Haider/MGA-3296-2025"
"OI Shaukat, Muhammad/0000-0001-8635-8692; Sultan,"
   Haider/0009-0005-2091-9991
FU Hainan Major Science and Technology Projects [ZDKJ202001]; Research
   Initiation Fund of Hainan University (KYQD) [19104]
FX This work was supported by the Hainan Major Science and Technology
   Projects (ZDKJ202001) . Research Initiation Fund of Hainan University
   (KYQD (ZR) 19104) .
"CR Abbas T, 2017, ECOTOX ENVIRON SAFE, V140, P37, DOI 10.1016/j.ecoenv.2017.02.028"
"   Abd El-Mageed TA, 2020, SCI HORTIC-AMSTERDAM, V261, DOI 10.1016/j.scienta.2019.108930"
"   Abel S, 2013, GEODERMA, V202, P183, DOI 10.1016/j.geoderma.2013.03.003"
"   Abit SM, 2012, ENVIRON SCI TECHNOL, V46, P8097, DOI 10.1021/es300797z"
"   Agbna G., 2017, Int J Eng Works, V4, P49, DOI [10.5281/zenodo.555942, DOI 10.5281/ZENODO.555942]"
"   Ahmad K, 2019, B ENVIRON CONTAM TOX, V102, P822, DOI 10.1007/s00128-019-02605-1"

"   Ahmad M, 2014, CHEMOSPHERE, V99, P19, DOI 10.1016/j.chemosphere.2013.10.071"
"   Ahmad M, 2012, J HAZARD MATER, V209, P392, DOI 10.1016/j.jhazmat.2012.01.047"
"   Ahmad M, 2020, AGRONOMY-BASEL, V10, DOI 10.3390/agronomy10071055"
"   Akhtar M, 2012, COMMUN SOIL SCI PLAN, V43, P1674, DOI 10.1080/00103624.2012.681738"
"   Akhtar SS, 2015, FUNCT PLANT BIOL, V42, P770, DOI 10.1071/FP15054"
"   Akhtar SS, 2015, AGR WATER MANAGE, V158, P61, DOI 10.1016/j.agwat.2015.04.010"
"   Akter R, 2023, HYDROBIOLOGY-BASEL, V2, P162, DOI [10.3390/hydrobiology2010011, 10.3390/hydrobiology2010011]"
"   Al-Wabel MI, 2013, BIORESOURCE TECHNOL, V131, P374, DOI 10.1016/j.biortech.2012.12.165"
"   Ali A, 2019, ECOTOX ENVIRON SAFE, V184, DOI 10.1016/j.ecoenv.2019.109635"
"   Ameloot N, 2013, EUR J SOIL SCI, V64, P379, DOI 10.1111/ejss.12064"
"   Ameur D, 2018, PEDOBIOLOGIA, V69, P1, DOI 10.1016/j.pedobi.2018.06.001"
"   Ana PP, 2018, DOI [10.2760/093804, 10.2760/093804, DOI 10.2760/093804]"
"   Andrenelli MC, 2016, AGR WATER MANAGE, V163, P190, DOI 10.1016/j.agwat.2015.09.017"
"   Arshad M, 2017, APPL SOIL ECOL, V114, P90, DOI 10.1016/j.apsoil.2017.02.021"
"   Azadi N, 2021, APPL SOIL ECOL, V167, DOI 10.1016/j.apsoil.2021.104043"
"   Bandara T, 2017, J SOIL SEDIMENT, V17, P665, DOI 10.1007/s11368-015-1243-y"
"   Benavente I, 2018, SCI TOTAL ENVIRON, V634, P1308, DOI 10.1016/j.scitotenv.2018.04.120"
"   Bhaduri D, 2016, CHEMOSPHERE, V148, P86, DOI 10.1016/j.chemosphere.2015.12.130"
"   Bhatta M, 2021, CURR OPIN PLANT BIOL, V60, DOI 10.1016/j.pbi.2020.101986"
"   Biswas D, 2021, PLANT GENE, V26, DOI 10.1016/j.plgene.2021.100286"
"   Blanco-Canqui H, 2017, SOIL SCI SOC AM J, V81, P687, DOI 10.2136/sssaj2017.01.0017"
"   Bolan S, 2023, SCI TOTAL ENVIRON, V886, DOI 10.1016/j.scitotenv.2023.163968"
"   Burrell LD, 2016, GEODERMA, V282, P96, DOI 10.1016/j.geoderma.2016.07.019"
"   Chaganti VN, 2015, AGR WATER MANAGE, V158, P255, DOI 10.1016/j.agwat.2015.05.016"
"   Chahal SS, 2017, ARCH AGRON SOIL SCI, V63, P1875, DOI 10.1080/03650340.2017.1308491"
"   Chen BL, 2012, J SOIL SEDIMENT, V12, P1350, DOI 10.1007/s11368-012-0554-5"
"   Chen HM, 2019, ENVIRON INT, V127, P395, DOI 10.1016/j.envint.2019.03.068"
"   Cheng C, 2020, SCI TOTAL ENVIRON, V740, DOI 10.1016/j.scitotenv.2020.139972"
"   Cui Q, 2021, SCI TOTAL ENVIRON, V756, DOI 10.1016/j.scitotenv.2020.143801"
"   Das SK, 2023, BIOMASS CONVERS BIOR, V13, P12193, DOI 10.1007/s13399-021-01880-5"
"   Das SK, 2011, SOIL BIOL, V22, P25, DOI 10.1007/978-3-642-14225-3_2"
"   Demisie W, 2014, CATENA, V121, P214, DOI 10.1016/j.catena.2014.05.020"
"   Dineshkumar R, 2018, WASTE BIOMASS VALORI, V9, P793, DOI 10.1007/s12649-017-9873-5"
"   Do HT, 2020, ECOTOX ENVIRON SAFE, V198, DOI 10.1016/j.ecoenv.2020.110672"
"   Dong XL, 2014, J HAZARD MATER, V267, P62, DOI 10.1016/j.jhazmat.2013.12.027"
"   Du ZJ, 2022, AGRICULTURE-BASEL, V12, DOI 10.3390/agriculture12081106"
"   Egamberdieva D, 2018, ENVIRON SUSTAIN, V1, P19, DOI [10.1007/s42398-018-0010-6, 10.1007/s42398-018-0010-6, DOI 10.1007/S42398-018-0010-6]"
"   Epelde L, 2009, SOIL BIOL BIOCHEM, V41, P1788, DOI 10.1016/j.soilbio.2008.04.001"
"   Farhangi-Abriz S, 2017, ECOTOX ENVIRON SAFE, V137, P64, DOI 10.1016/j.ecoenv.2016.11.029"
"   Farooq M, 2020, CHEMOSPHERE, V260, DOI 10.1016/j.chemosphere.2020.127652"
"   Farrell M, 2013, SCI TOTAL ENVIRON, V465, P288, DOI 10.1016/j.scitotenv.2013.03.090"
"   Fazal A, 2016, COMMUN SOIL SCI PLAN, V47, P1985, DOI 10.1080/00103624.2016.1216562"
"   Fei YH, 2019, SOIL TILL RES, V193, P59, DOI 10.1016/j.still.2019.05.024"
"   Fidel RB, 2018, SCI REP-UK, V8, DOI 10.1038/s41598-018-35534-w"
"   Fox A, 2014, FEMS MICROBIOL ECOL, V90, P78, DOI 10.1111/1574-6941.12374"
"   Gao XD, 2016, ACS OMEGA, V1, P226, DOI 10.1021/acsomega.6b00085"
"   Gasc?? G, 2016, ECOL ENG, V95, P19, DOI 10.1016/j.ecoleng.2016.06.039"
"   Gontikaki E, 2013, PLOS ONE, V8, DOI 10.1371/journal.pone.0067722"
"   Gul S, 2015, AGR ECOSYST ENVIRON, V206, P46, DOI 10.1016/j.agee.2015.03.015"
"   Gunarathne V, 2020, BIOCHAR, V2, P107, DOI 10.1007/s42773-020-00036-4"
"   Hagemann N, 2017, NAT COMMUN, V8, DOI 10.1038/s41467-017-01123-0"
"   Haider FU, 2021, ECOTOX ENVIRON SAFE, V214, DOI 10.1016/j.ecoenv.2021.112112"
"   Hale L, 2015, SOIL BIOL BIOCHEM, V81, P228, DOI 10.1016/j.soilbio.2014.11.023"
"   Hammer EC, 2015, APPL SOIL ECOL, V96, P114, DOI 10.1016/j.apsoil.2015.07.014"
"   Han FP, 2016, CATENA, V137, P554, DOI 10.1016/j.catena.2015.11.002"
"   Han YX, 2016, J PLANT NUTR, V39, P1654, DOI 10.1080/01904167.2015.1089903"
"   Hashimoto Y, 2009, J ENVIRON QUAL, V38, P1420, DOI 10.2134/jeq2008.0427"
"   Hasnine MT, 2017, Environment and Ecology Research, V5, P510, DOI [10.13189/eer.2017.050707, 10.13189/eer.2017.050707, DOI 10.13189/EER.2017.050707]"
"   He MJ, 2021, J HAZARD MATER, V414, DOI 10.1016/j.jhazmat.2021.125378"
"   Hossain MZ, 2021, J ANAL APPL PYROL, V155, DOI 10.1016/j.jaap.2021.105043"
"   Hu J, 2021, P ROY SOC B-BIOL SCI, V288, DOI 10.1098/rspb.2021.1396"
"   Huang DL, 2017, CHEMOSPHERE, V174, P545, DOI 10.1016/j.chemosphere.2017.01.130"
"   [???????€? Huang Mingyi], 2021, [?????????????????, Transactions of the Chinese Society for Agricultural Machinery], V52, P238"
"   Hussain A, 2019, ITAL J AGRON, V14, P101, DOI 10.4081/ija.2019.1413"
"   Ijaz M, 2019, BRAZ J MICROBIOL, V50, P449, DOI 10.1007/s42770-019-00043-z"
"   Irfan M, 2019, ARAB J GEOSCI, V12, DOI 10.1007/s12517-019-4239-x"
"   Jaganathan D, 2018, FRONT PLANT SCI, V9, DOI 10.3389/fpls.2018.00985"
"   James RA, 2011, J EXP BOT, V62, P2939, DOI 10.1093/jxb/err003"
"   J??rup L, 2003, BRIT MED BULL, V68, P167, DOI 10.1093/bmb/ldg032"
"   Jiang J, 2012, J HAZARD MATER, V229, P145, DOI 10.1016/j.jhazmat.2012.05.086"
"   Jien SH, 2013, CATENA, V110, P225, DOI 10.1016/j.catena.2013.06.021"
"   Jones DL, 2012, SOIL BIOL BIOCHEM, V45, P113, DOI 10.1016/j.soilbio.2011.10.012"
"   Kanwal S, 2018, J PLANT NUTR, V41, P526, DOI 10.1080/01904167.2017.1392568"
"   Kazemi R, 2019, J SOIL SCI PLANT NUT, V19, P758, DOI 10.1007/s42729-019-00075-2"
"   Kim HS, 2016, CHEMOSPHERE, V142, P153, DOI 10.1016/j.chemosphere.2015.06.041"
"   Kiran, 2022, MATER TODAY-PROC, V51, P880, DOI 10.1016/j.matpr.2021.06.278"
"   Kolb SE, 2009, SOIL SCI SOC AM J, V73, P1173, DOI 10.2136/sssaj2008.0232"
"   Kolton M, 2017, NEW PHYTOL, V213, P1393, DOI 10.1111/nph.14253"
"   Kumar D., 2016, Plant Responses to Xenobiotics, P19, DOI DOI 10.1007/978-981-10-2860-1_2"
"   Lashari MS, 2015, J SCI FOOD AGR, V95, P1321, DOI 10.1002/jsfa.6825"

"   Lashari MS, 2013, FIELD CROP RES, V144, P113, DOI 10.1016/j.fcr.2012.11.015"
"   Lata S, 2019, INT J PHARM SCI RES, V10, P4120, DOI 10.13040/IJPSR.0975-8232.10(9).4120-28"
"   Lee XQ, 2022, CATENA, V211, DOI 10.1016/j.catena.2022.106018"
"   Lemon E. R., 1956, Soil Science Society of America. Proceedings, V20, P120"
"   Li H, 2019, SCI TOTAL ENVIRON, V669, P258, DOI 10.1016/j.scitotenv.2019.03.104"
"   Li LJ, 2018, SCI TOTAL ENVIRON, V640, P1034, DOI 10.1016/j.scitotenv.2018.05.287"
"   Li XP, 2014, ENVIRON REV, V22, P41, DOI 10.1139/er-2013-0035"
"   Lian F, 2017, ENVIRON SCI TECHNOL, V51, P13517, DOI 10.1021/acs.est.7b02528"
"   Liang S, 2023, PLANTS-BASEL, V12, DOI 10.3390/plants12020284"
"   Limcharoensuk T, 2015, ECOTOX ENVIRON SAFE, V122, P322, DOI 10.1016/j.ecoenv.2015.08.013"
"   Liu C, 2017, J AGRON CROP SCI, V203, P131, DOI 10.1111/jac.12185"
"   Liu HK, 2018, SCI TOTAL ENVIRON, V645, P702, DOI 10.1016/j.scitotenv.2018.07.115"
"   Liu YL, 2020, CHEMOSPHERE, V247, DOI 10.1016/j.chemosphere.2020.125850"
"   Lu HP, 2015, CHEMOSPHERE, V119, P209, DOI 10.1016/j.chemosphere.2014.06.024"
"   Lu KP, 2017, J ENVIRON MANAGE, V186, P285, DOI 10.1016/j.jenvman.2016.05.068"
"   Lu SG, 2014, CATENA, V114, P37, DOI 10.1016/j.catena.2013.10.014"
"   Luo MK, 2020, SCI TOTAL ENVIRON, V717, DOI 10.1016/j.scitotenv.2020.137014"
"   Ma H, 2020, J HAZARD MATER, V388, DOI 10.1016/j.jhazmat.2020.122065"
"   Ma H, 2022, PLANTS-BASEL, V11, DOI 10.3390/plants11030423"
"   Manfrin J, 2021, J ENVIRON CHEM ENG, V9, DOI 10.1016/j.jece.2020.104980"
"   Mehmood S, 2022, SCI TOTAL ENVIRON, V826, DOI 10.1016/j.scitotenv.2022.154043"
"   Mehmood S, 2021, ENVIRON POLLUT, V289, DOI 10.1016/j.envpol.2021.117916"
"   Mehmood S, 2018, PLANT PHYSIOL BIOCH, V132, P345, DOI 10.1016/j.plaphy.2018.09.019"
"   Mierzwa-Hersztek M, 2016, APPL SOIL ECOL, V105, P144, DOI 10.1016/j.apsoil.2016.04.006"
"   Mishra AK, 2023, FRONT ENV SCI-SWITZ, V10, DOI 10.3389/fenvs.2022.962581"
"   Moradi S, 2019, ENVIRON MONIT ASSESS, V191, DOI 10.1007/s10661-019-7393-4"
"   Mukherjee S, 2022, ENVIRON POLLUT, V308, DOI 10.1016/j.envpol.2022.119609"
"   Nehela Y, 2021, PLANTS-BASEL, V10, DOI 10.3390/plants10091960"
"   Nelissen V, 2015, EUR J AGRON, V62, P65, DOI 10.1016/j.eja.2014.09.006"
"   Ha NTH, 2022, CLEAN TECHNOL ENVIR, V24, P1073, DOI 10.1007/s10098-020-01996-8"
"   Nie CR, 2018, CHEMOSPHERE, V200, P274, DOI 10.1016/j.chemosphere.2018.02.134"
"   Oh SY, 2013, J ENVIRON QUAL, V42, P815, DOI 10.2134/jeq2012.0411"
"   Palansooriya KN, 2019, J ENVIRON MANAGE, V234, P52, DOI 10.1016/j.jenvman.2018.12.085"
"   Pan XH, 2014, WATER RES, V55, P21, DOI 10.1016/j.watres.2014.01.066"
"   Pan YS, 2022, SCI TOTAL ENVIRON, V823, DOI 10.1016/j.scitotenv.2022.153547"
"   Parihar P, 2015, ENVIRON SCI POLLUT R, V22, P4056, DOI 10.1007/s11356-014-3739-1"
"   Peake LR, 2014, GEODERMA, V235, P182, DOI 10.1016/j.geoderma.2014.07.002"
"   Poli A, 2009, RES MICROBIOL, V160, P99, DOI 10.1016/j.resmic.2008.10.012"
"   Preena PG, 2021, REV AQUACULT, V13, P2053, DOI 10.1111/raq.12558"
"   Qi X, 2022, J HAZARD MATER, V436, DOI 10.1016/j.jhazmat.2022.129147"
"   Qi X, 2021, J HAZARD MATER, V401, DOI 10.1016/j.jhazmat.2020.123823"
"   Qian LB, 2015, J SOIL SEDIMENT, V15, P1130, DOI 10.1007/s11368-015-1073-y"
"   Qin GW, 2021, CHEMOSPHERE, V267, DOI 10.1016/j.chemosphere.2020.129205"
"   Qu JH, 2022, J HAZARD MATER, V423, DOI 10.1016/j.jhazmat.2021.127043"
"   Rahnama A, 2010, FUNCT PLANT BIOL, V37, P255, DOI 10.1071/FP09148"
"   Rizwan M, 2018, J ENVIRON MANAGE, V206, P676, DOI 10.1016/j.jenvman.2017.10.035"
"   Rizwan M, 2016, ECOTOX ENVIRON SAFE, V130, P43, DOI 10.1016/j.ecoenv.2016.04.001"
"   Rousk J, 2010, ISME J, V4, P1340, DOI 10.1038/ismej.2010.58"
"   Sabir A, 2020, J ENVIRON MANAGE, V265, DOI 10.1016/j.jenvman.2020.110522"
"   Saifullah, 2018, SCI TOTAL ENVIRON, V625, P320, DOI 10.1016/j.scitotenv.2017.12.257"
"   Sajid M., 2023, Aquaculture Sediments Amended with Biochar Improved Soil Health and Plant Growth in a Degraded Soil"
"   Seneviratne M, 2017, J ENVIRON MANAGE, V186, P293, DOI 10.1016/j.jenvman.2016.07.024"
"   Serkalem W.M., 2015, Masters Thesis"
"   Shaaban M., 2021, SORBENTS MAT CONTROL, P189, DOI [10.1016/B978-0-12-820042-1.00001-8, DOI 10.1016/B978-0-12-820042-1.00001-8]"
"   Shaaban M, 2018, J ENVIRON MANAGE, V228, P429, DOI 10.1016/j.jenvman.2018.09.006"
"   Shar AG, 2021, SAUDI J BIOL SCI, V28, P4155, DOI 10.1016/j.sjbs.2021.04.074"
"   Sharma RK, 2008, ENVIRON MONIT ASSESS, V142, P269, DOI 10.1007/s10661-007-9924-7"
"   Shi RY, 2019, CHEMOSPHERE, V234, P43, DOI 10.1016/j.chemosphere.2019.06.030"
"   Shi RY, 2018, J ENVIRON MANAGE, V210, P171, DOI 10.1016/j.jenvman.2018.01.028"
"   Simiele M, 2021, ENVIRON EXP BOT, V182, DOI 10.1016/j.envexpbot.2020.104335"
"   Singh R, 2019, CLEAN-SOIL AIR WATER, V47, DOI 10.1002/clen.201700656"
"   Song LC, 2021, CHEMOSPHERE, V279, DOI 10.1016/j.chemosphere.2021.130427"
"   Song Y, 2017, INT J PHYTOREMEDIAT, V19, P133, DOI 10.1080/15226514.2016.1207598"
"   Sun DQ, 2016, BIOL FERT SOILS, V52, P515, DOI 10.1007/s00374-016-1093-9"
"   Sun HJ, 2017, SCI TOTAL ENVIRON, V575, P820, DOI 10.1016/j.scitotenv.2016.09.137"
"   Sun XQ, 2021, SOIL TILL RES, V205, DOI 10.1016/j.still.2020.104809"
"   Tan HY, 2021, Chemical engineering transactions, V83, P235, DOI [10.3303/cet2183040, 10.3303/CET2183040]"
"   Tang JW, 2020, SCI TOTAL ENVIRON, V731, DOI 10.1016/j.scitotenv.2020.138938"
"   Tao SY, 2018, BIORESOURCES, V13, P1773, DOI 10.15376/biores.13.1.1773-1786"
"   Tao Y, 2019, SCI TOTAL ENVIRON, V682, P59, DOI 10.1016/j.scitotenv.2019.05.134"
"   Teng FZ, 2010, EARTH PLANET SC LETT, V300, P63, DOI 10.1016/j.epsl.2010.09.036"
"   Thomas SC, 2013, J ENVIRON MANAGE, V129, P62, DOI 10.1016/j.jenvman.2013.05.057"
"   Tomczyk A, 2020, REV ENVIRON SCI BIO, V19, P191, DOI 10.1007/s11157-020-09523-3"
"   Tu C, 2020, ENVIRON INT, V137, DOI 10.1016/j.envint.2020.105576"
"   Vanek S. J., 2016, Journal of Microbial and Biochemical Technology, V8, P296, DOI 10.4172/1948-5948.1000300"
"   Vlajkov V, 2023, PLANTS-BASEL, V12, DOI 10.3390/plants12051024"
"   Wang JH, 2020, ECOTOX ENVIRON SAFE, V190, DOI 10.1016/j.ecoenv.2019.110124"
"   Wang Y, 2017, FUEL PROCESS TECHNOL, V160, P55, DOI 10.1016/j.fuproc.2017.02.019"
"   Wang Y, 2014, J ANAL APPL PYROL, V110, P375, DOI 10.1016/j.jaap.2014.10.006"
"   Wang YY, 2019, J CLEAN PROD, V231, P556, DOI 10.1016/j.jclepro.2019.04.407"
"   Wang Y, 2022, ENVIRON GEOTECH, V9, P135, DOI 10.1680/jenge.18.00091"

"   Wardle DA, 2008, SCIENCE, V320, P629, DOI 10.1126/science.1154960"
"   Wei J, 2019, ENVIRON POLLUT, V251, P56, DOI 10.1016/j.envpol.2019.04.128"
"   Wei T, 2022, J HAZARD MATER, V435, DOI 10.1016/j.jhazmat.2022.129006"
"   Weintraub SR, 2013, BIOGEOCHEMISTRY, V114, P313, DOI 10.1007/s10533-012-9812-2"
"   Wu B, 2019, SCI TOTAL ENVIRON, V686, P719, DOI 10.1016/j.scitotenv.2019.06.041"
"   Wu CC, 2022, ENVIRON RES, V212, DOI 10.1016/j.envres.2022.113226"
"   Wu HP, 2017, CRIT REV BIOTECHNOL, V37, P754, DOI [10.24966/ctb-9107/100009, 10.1080/07388551.2016.1232696]"
"   Xian Y, 2015, CHEMOSPHERE, V139, P604, DOI 10.1016/j.chemosphere.2014.12.060"
"   Xiao KM, 2017, ACS APPL MATER INTER, V9, P30448, DOI 10.1021/acsami.7b09520"
"   Xie LP, 2018, ECOTOX ENVIRON SAFE, V162, P71, DOI 10.1016/j.ecoenv.2018.06.072"
"   Xing J, 2024, SCI TOTAL ENVIRON, V924, DOI 10.1016/j.scitotenv.2024.171641"
"   Xu HQ, 2022, ECO-ENVIRON HEALTH, V1, DOI 10.1016/j.eehl.2022.04.003"
"   Xu HJ, 2014, ENVIRON SCI TECHNOL, V48, P9391, DOI 10.1021/es5021058"
"   Xu YL, 2013, BIORESOURCE TECHNOL, V146, P485, DOI 10.1016/j.biortech.2013.07.086"
"   Yaghoubian Y, 2019, ENVIRON SCI POLLUT R, V26, P7863, DOI 10.1007/s11356-019-04255-6"
"   Yang QQ, 2018, SCI TOTAL ENVIRON, V642, P690, DOI 10.1016/j.scitotenv.2018.06.068"
"   Yao RJ, 2022, IRRIGATION SCI, V40, P267, DOI 10.1007/s00271-021-00763-x"
"   Youngwilai A, 2020, SCI TOTAL ENVIRON, V713, DOI 10.1016/j.scitotenv.2020.136708"
"   Yuan JH, 2011, SOIL USE MANAGE, V27, P110, DOI 10.1111/j.1475-2743.2010.00317.x"
"   Yuan P, 2019, SCI TOTAL ENVIRON, V659, P473, DOI 10.1016/j.scitotenv.2018.12.400"
"   Zhang FG, 2019, SCI TOTAL ENVIRON, V655, P1150, DOI 10.1016/j.scitotenv.2018.11.317"
"   Zhang H, 2022, ECO-ENVIRON HEALTH, V1, P46, DOI 10.1016/j.eehl.2022.04.002"
"   Zhang LX, 2018, J HAZARD MATER, V360, P32, DOI 10.1016/j.jhazmat.2018.07.075"
"   Zhang Shuxuan, 2023, Chemosphere, V340, P139850, DOI [10.1016/j.chemosphere.2023.139850, 10.1016/j.chemosphere.2023.139850]"
"   Zhang XQ, 2017, POL J ENVIRON STUD, V26, P413, DOI 10.15244/pjoes/64908"
"   Zhao FJ, 2015, ENVIRON SCI TECHNOL, V49, P750, DOI 10.1021/es5047099"
"   Zhao GM, 2015, IND CROP PROD, V64, P175, DOI 10.1016/j.indcrop.2014.10.058"
"   Zhao L, 2020, WATER RES, V172, DOI 10.1016/j.watres.2020.115494"
"   Zheng H, 2018, PLANT CELL ENVIRON, V41, P517, DOI 10.1111/pce.12944"
"   Zheng H, 2013, GEODERMA, V206, P32, DOI 10.1016/j.geoderma.2013.04.018"
"   Zhou X, 2019, ECOL ENG, V128, P57, DOI 10.1016/j.ecoleng.2018.12.028"
"   Zhou YW, 2021, BIORESOURCE TECHNOL, V337, DOI 10.1016/j.biortech.2021.125451"
"   Zhu XM, 2017, ENVIRON POLLUT, V227, P98, DOI [10.1016/j.envpol.2017.04.032, 10.1016/j.envPol.2017.04.032]"
"   Zielinska A, 2016, CHEMOSPHERE, V163, P480, DOI 10.1016/j.chemosphere.2016.08.072"
"   ,, 2020, The state of food security and nutrition in the world 2020: transforming food systems for affordable healthy diets, DOI [10.26596/wn.201910395-97, 10.4060/ca9692en]"
NR 202
TC 28
Z9 28
U1 37
U2 127
PU ELSEVIER
PI AMSTERDAM
"PA RADARWEG 29, 1043 NX AMSTERDAM, NETHERLANDS"
SN 0048-9697
EI 1879-1026
J9 SCI TOTAL ENVIRON
JI Sci. Total Environ.
PD DEC 1
PY 2024
VL 954
AR 176104
DI 10.1016/j.scitotenv.2024.176104
EA SEP 2024
PG 14
WC Environmental Sciences
WE Science Citation Index Expanded (SCI-EXPANDED)
SC Environmental Sciences & Ecology
GA H1N2D
UT WOS:001321168500001
PM 39250966
DA 2026-04-06
ER

PT J
"AU Li, S"
"   Wu, X"
"   Xie, JM"
"AF Li, Shuang"
"   Wu, Xiang"
"   Xie, Jiamin"
TI Biomineralization technology for solidification/stabilization of heavy
   metals in ecosystem: status and perspective
SO FRONTIERS IN ECOLOGY AND EVOLUTION
LA English
DT Article
DE heavy metal; biomineralization; polluted water; contaminated soil;
   precipitation; coprecipitation
ID CARBONATE PRECIPITATION; BIOREMEDIATION; SOIL
"AB Biomineralization technology offers an eco-friendly and efficient method for stabilizing heavy metals (HMs) in ecosystem. This technology comprises two primary methods: microbially induced carbonate

precipitation (MICP) and enzyme-induced carbonate precipitation (EICP). Biomineralization provides a superior alternative to stabilize heavy metals due to its low energy consumption, reduced carbon dioxide emissions, and superior biocompatibility. In the process of biomineralization, heavy metal ions precipitate and co-precipitate with calcium carbonate, forming a solidified and stabilized product. Despite its many advantages, little attention has been paid to the impact of biomineralization on mitigation of ammonia nitrogen of bio-treated polluted water and the strength of contaminated soil, limiting its further applications in ecological environment restoration. This paper summarizes recent advancements in biomineralization for solidifying and stabilizing (S/S) heavy metals in contaminated water and soil. Key factors inhibiting this method's application include the concentration and combinations of heavy metal ions, the concentration of ammonia nitrogen in polluted water, and the properties of contaminated soil. Finally, this paper offers recommendations on the optimization of further research and experimental design of biomineralization on S/S polluted water and contaminated soil."
"C1 [Li, Shuang] Fuzhou Univ, Coll Civil Engn, Fuzhou, Peoples R China."
"   [Wu, Xiang] Zhongnan Univ Econ & Law, Sch Publ Adm, Wuhan, Peoples R China."
"   [Xie, Jiamin] Shantou Polytech, Sch Construt & Ecol, Shantou, Peoples R China."
C3 Fuzhou University; Zhongnan University of Economics & Law
"RP Xie, JM (corresponding author), Shantou Polytech, Sch Construt & Ecol, Shantou, Peoples R China."
EM jmxie@stpt.edu.cn
"RI Li, Shuang/PJT-3686-2026"
FU Project of Young Innovative Talents for General Colleges in Guangdong Province [2022KQNCX2372022]; Scientific Research Project in Shantou Polytechnic [SZK2023Z01]
FX Funding This work was financially supported by the Project of Young Innovative Talents for General Colleges in Guangdong Province (2022KQNCX2372022) and the Scientific Research Project in Shantou Polytechnic (SZK2023Z01).
"CR Achal V, 2012, J HAZARD MATER, V201, P178, DOI 10.1016/j.jhazmat.2011.11.067"
"   Benalia MC, 2022, ARAB J SCI ENG, V47, P5587, DOI 10.1007/s13369-021-05525-7"
"   BLAKELEY RL, 1983, INORG CHIM A-BIOINOR, V79, P11, DOI 10.1016/S0020-1693(00)95029-1"
"   Chen MJ, 2021, J HAZARD MATER, V411, DOI 10.1016/j.jhazmat.2021.125103"
"   Chu J, 2014, ACTA GEOTECH, V9, P277, DOI 10.1007/s11440-013-0278-8"
"   Fu FL, 2012, CHEM ENG J, V189, P283, DOI 10.1016/j.cej.2012.02.073"
"   Fu ZJ, 2022, WATER RES, V222, DOI 10.1016/j.watres.2022.118888"
"   Gowthaman S., 2022, Low Carbon Stabilization and Solidification of Hazardous Wastes, P49, DOI [DOI 10.1016/B978-0-12-824004, 10.1016/B978-0-12-824004-5.00019-0]"
"   Han LJ, 2022, CONSTR BUILD MATER, V314, DOI 10.1016/j.conbuildmat.2021.125577"
"   Han LJ, 2023, J HAZARD MATER, V448, DOI 10.1016/j.jhazmat.2023.130860"
"   Hassan A, 2020, J CLEAN PROD, V245, DOI 10.1016/j.jclepro.2019.118762"
"   Jiang NJ, 2020, SOIL USE MANAGE, V36, P185, DOI 10.1111/sum.12571"
"   Jiang NJ, 2019, SCI TOTAL ENVIRON, V672, P722, DOI 10.1016/j.scitotenv.2019.03.294"
"   Kang CH, 2016, ECOL ENG, V89, P64, DOI 10.1016/j.ecoleng.2016.01.023"
"   Li HF, 2023, SEP PURIF TECHNOL, V319, DOI 10.1016/j.seppur.2023.124058"
"   Li Shuang, 2022, Arabian Journal of Geosciences, V15, DOI [10.1007/s12517-022-09752-6, 10.1007/s12517-022-09752-6]"
"   Liu JR, 2021, J HAZARD MATER, V416, DOI 10.1016/j.jhazmat.2021.125776"
"   Liu P, 2021, BIOCHEM ENG J, V166, DOI 10.1016/j.bej.2020.107856"
"   Makinda Jodin, 2021, E3S Web of Conferences, V331, DOI [10.1051/e3sconf/202133103001, 10.1051/e3sconf/202133103001]"
"   Meng H, 2021, GEODERMA, V383, DOI 10.1016/j.geoderma.2020.114723"
"   Morales L, 2019, APPL CLAY SCI, V174, P15, DOI 10.1016/j.clay.2019.03.018"
"   Mwandira W, 2017, ECOL ENG, V109, P57, DOI 10.1016/j.ecoleng.2017.09.011"
"   Mymrin V, 2019, J ENVIRON MANAGE, V236, P206, DOI 10.1016/j.jenvman.2019.01.065"
"   Qiao SY, 2021, CHEMOSPHERE, V274, DOI 10.1016/j.chemosphere.2021.129661"
"   Song HW, 2022, SEP PURIF TECHNOL, V297, DOI 10.1016/j.seppur.2022.121540"
"   Tsesarsky M, 2018, ENVIRON GEOTECH, V5, P69, DOI 10.1680/jenge.15.00070"
"   Wang YJ, 2022, ACTA GEOTECH, V17, P4217, DOI 10.1007/s11440-022-01491-y"
"   Wang ZJ, 2022, ENVIRON SCI TECHNOL, V56, P11343, DOI 10.1021/acs.est.2c01311"
"   Xu JW, 2019, NAT COMMUN, V10, DOI 10.1038/s41467-019-10472-x"
"   Xue ZF, 2022, J HAZARD MATER, V426, DOI 10.1016/j.jhazmat.2021.128090"
"   Zeng GQ, 2021, J HAZARD MATER, V412, DOI 10.1016/j.jhazmat.2021.125156"
"   Zhang J, 2019, APPL GEOCHEM, V103, P68, DOI 10.1016/j.apgeochem.2019.02.011"
NR 32
TC 8
Z9 8
U1 12
U2 97
PU FRONTIERS MEDIA SA
PI LAUSANNE
"PA AVENUE DU TRIBUNAL FEDERAL 34, LAUSANNE, CH-1015, SWITZERLAND"
SN 2296-701X
J9 FRONT ECOL EVOL
JI Front. Ecol. Evol.
PD JUN 16
PY 2023
VL 11
AR 1189356
DI 10.3389/fevo.2023.1189356
PG 5
WC Ecology
WE Science Citation Index Expanded (SCI-EXPANDED)
SC Environmental Sciences & Ecology
GA K5AU1
UT WOS:001016572400001
"OA Green Submitted, gold"

DA 2026-04-06
ER

PT J
AU Tamannaa
" Kumar, V"
" Chandel, S"
" Rana, S"
" Singh, D"
AF Tamannaa
" Kumar, Vijay"
" Chandel, Shamli"
" Rana, Samiksha"
" Singh, Dharam"
TI Comparative genomics and functional analysis of Rahnella sp.
PCH160 revealed metal stress tolerance and plant growth-promotion traits
SO MICROBIOLOGICAL RESEARCH
LA English
DT Article
DE Rahnella; Genomic analysis; Heavy metal tolerance; Plant
growth-promoting rhizobacterium; Pangi-Chamba Himalaya
ID ENDOPHYTIC BACTERIA; SEQUENCE
"AB Heavy metals (HM) are rapidly emerging as the primary pollutants in agriculture, posing significant environmental and health concerns. Employing heavy metal tolerant plant growth-promoting rhizobacteria (PGPR) for the remediation of HM-induced phytotoxicity is a sustainable and effective strategy. This study investigates a putative novel plant growth-promoting rhizobacterium (PGPR) Rahnella sp. PCH160, isolated from the Pangi-Chamba Himalayan region (PCH). Whole-genome sequencing and comparative genomics identified unique genes associated with heavy metal tolerance, phosphate solubilization, siderophore production, nitrogen fixation, and indole-3-acetic acid (IAA) synthesis. The bacterium exhibits exceptional tolerance to heavy metals, even under chemically defined medium, particularly copper (Cu2+) and nickel (Ni2+), as well as multiple plant growth-promoting traits. Additionally, PCH160 showed biocontrol potential through different enzymatic activities. Seed-based bioassays highlighted significant enhancements in root and shoot length and dry biomass under heavy metal stress. This research highlights the promising capabilities of Rahnella sp. PCH160 as both a biofertilizer and a biocontrol agent. There are limited reports of the positive impact of PGPR on plant growth and development in the Himalayan regions. However, to the best of our knowledge, tolerance of PGPR to heavy metals has not been characterized from higher-altitude regions. In conclusion, this study provides insights into sustainable agriculture practices in contaminated soils at high altitudes. It has also demonstrated that a PGPR isolated from the Pangi-Chamba Himalayan region has the potential to enhance plant growth and development in the presence of heavy metal stress."
"C1 [Tamannaa; Kumar, Vijay; Chandel, Shamli; Rana, Samiksha; Singh, Dharam] CSIR Inst Himalayan Bioresource Technol, Biotechnol Div, Post Box 6, Palampur 176061, Himachal Prades, India."
" [Tamannaa; Chandel, Shamli; Rana, Samiksha; Singh, Dharam] Acad Sci & Innovat Res AcSIR, Ghaziabad 201002, India."
" [Kumar, Vijay] Guru Nanak Dev Univ, Dept Microbiol, Amritsar 143005, Punjab, India."
C3 Council of Scientific & Industrial Research (CSIR) - India; CSIR -
Institute of Himalayan Bioresource Technology (IHBT); Academy of
Scientific & Innovative Research (AcSIR); Guru Nanak Dev University
"RP Singh, D (corresponding author), CSIR Inst Himalayan Bioresource Technol CSIR IHBT, Biotechnol Div, Mol & Microbial Genet Lab, Palampur 176061, Himachal Prades, India."
EM vijay.hpu@gmail.com; shamli.chandel43@gmail.com;
samiksharana99@gmail.com; dharamsingh@ihbt.res.in
"OI Kumar, Vijay/0000-0002-5581-4365; Singh, Dharam/0000-0001-8655-2997"
"FU Council of Scientific and Industrial Research (CSIR) , Government of"
" India [MLP201, MLP0143]"
FX Funding D.S. duly acknowledges Council of Scientific and
" Industrial Research (CSIR) , Government of India for financial support"
under MLP201 and MLP0143 projects.
"CR Adhikary S, 2024, GEOMICROBIOL J, DOI 10.1080/01490451.2024.2340517"
" Ansari FA, 2018, Advances in Microbiology, V08, P235, DOI [10.4236/aim.2018.83016, 10.4236/aim.2018.83016, DOI 10.4236/AIM.2018.83016]"
" Backer R, 2018, FRONT PLANT SCI, V9, DOI 10.3389/fpls.2018.01473"
" Bhadrecha P, 2023, ARCH MICROBIOL, V205, DOI 10.1007/s00203-023-03502-2"
" Blin K, 2023, NUCLEIC ACIDS RES, V51, pW46, DOI 10.1093/nar/gkad344"
" Borker SS, 2021, BMC GENOMICS, V22, DOI 10.1186/s12864-021-07681-4"
" Boubekri K, 2021, MICROORGANISMS, V9, DOI 10.3390/microorganisms9030470"
" Cen XY, 2024, PLANTS-BASEL, V13, DOI 10.3390/plants13192703"
" Chamkhi I, 2023, IND CROP PROD, V197, DOI 10.1016/j.indcrop.2023.116606"
" Chang WY, 2024, J PLANT GROWTH REGUL, V43, P76, DOI 10.1007/s00344-023-11056-2"
" Dar Gh. H., 2018, Biodiversitas: Journal of Biological Diversity, V19, P712, DOI [10.13057/biodiv/d190245, 10.13057/biodiv/d190245]"
" Dey G, 2022, MAR POLLUT BULL, V181, DOI 10.1016/j.marpolbul.2022.113905"
" Dilshad R, 2023, OPEN AGRIC, V8, DOI 10.1515/opag-2022-0238"
" Dixit S, 2024, PLANT PHYSIOL REP, V29, P6, DOI 10.1007/s40502-023-00766-0"
" Dukariya G, 2020, Journal of Advances in Biology & Biotechnology, P39, DOI [10.9734/jabb/2020/v23i130137, 10.9734/JABB/2020/v23i130137, DOI 10.9734/JABB/2020/V23I130137, 10.9734/jabb/2020/, DOI 10.9734/JABB/2020]"
" Fadiji AE, 2023, MICROBIOL RES, V271, DOI 10.1016/j.micres.2023.127368"
" Fan MC, 2018, MICROBIOL RES, V217, P51, DOI 10.1016/j.micres.2018.09.002"
" Fujita M, 2019, COMMUN BIOL, V2, DOI 10.1038/s42003-019-0676-z"
" Ghorbani A, 2024, J NANOBIOTECHNOL, V22, DOI 10.1186/s12951-024-02371-1"
" Glick BR, 2012, SCIENTIFICA, V2012, DOI 10.6064/2012/963401"
" GLICK BR, 1995, CAN J MICROBIOL, V41, P109, DOI 10.1139/m95-015"
" GORDON SA, 1951, PLANT PHYSIOL, V26, P192, DOI 10.1104/pp.26.1.192"
" Goris J, 2007, INT J SYST EVOL MICR, V57, P81, DOI 10.1099/ijs.0.64483-0"
" Goswami D, 2014, J PLANT INTERACT, V9, P566, DOI 10.1080/17429145.2013.871650"

"   Guo YB, 2012, J BACTERIOL, V194, P6646, DOI 10.1128/JB.01769-12"
"   Hassoun A, 2024, COMPR REV FOOD SCI F, V23, DOI 10.1111/1541-4337.70040"
"   Helal DS, 2022, J GENET ENG BIOTECHN, V20, DOI 10.1186/s43141-022-00361-0"
"   Kamath A, 2023, EUR J PLANT PATHOL, V165, P407, DOI 10.1007/s10658-022-02618-3"
"   Kaviraj M, 2024, ARCH MICROBIOL, V206, DOI 10.1007/s00203-024-04009-0"
"   Khare G, 2017, PLOS ONE, V12, DOI 10.1371/journal.pone.0169545"
"   Kim M, 2014, INT J SYST EVOL MICR, V64, P346, DOI 10.1099/ijs.0.059774-0"
"   Kryuchkova YV, 2024, MICROBIOLOGY+, V93, P478, DOI 10.1134/S0026261723605122"
"   Kulus D, 2025, PLANT CELL TISS ORG, V160, DOI 10.1007/s11240-024-02955-7"
"   Kumar Ajay, 2019, Vegetos, V32, P103, DOI [10.1007/s42535-019-00013-8, 10.1007/s42535-019-00013-8]"
"   Kumar R, 2021, GENOMICS, V113, P1448, DOI 10.1016/j.ygeno.2021.03.019"
"   Kumar V, 2022, FRONT MICROBIOL, V13, DOI 10.3389/fmicb.2022.881873"
"   Kumar V, 2022, EXTREMOPHILES, V26, DOI 10.1007/s00792-021-01252-x"
"   Kumar V, 2021, BIORESOURCE TECHNOL, V319, DOI 10.1016/j.biortech.2020.124235"
"   Kumar V, 2020, GENOMICS, V112, P637, DOI 10.1016/j.ygeno.2019.04.016"
"   Kumar V, 2018, FEMS MICROBIOL LETT, V365, DOI 10.1093/femsle/fny144"
"   Kumar V, 2023, ARCH MICROBIOL, V205, DOI 10.1007/s00203-023-03456-5"
"   Lee SY, 2024, INT J ENVIRON SCI TE, V21, P1419, DOI 10.1007/s13762-023-05078-2"
"   Leontidou K, 2020, SCI REP-UK, V10, DOI 10.1038/s41598-020-71652-0"
"   Li PS, 2023, FORESTS, V14, DOI 10.3390/f14061110"
"   Meier-Kolthoff JP, 2019, NAT COMMUN, V10, DOI 10.1038/s41467-019-10210-3"
"   Miller MB, 2001, ANNU REV MICROBIOL, V55, P165, DOI 10.1146/annurev.micro.55.1.165"
"   Monchy S, 2006, INT J ENVIRON AN CH, V86, P677, DOI 10.1080/03067310600583824"
"   Musah BI, 2024, ENVIRON MONIT ASSESS, V197, DOI 10.1007/s10661-024-13490-5"
"   Page AJ, 2015, BIOINFORMATICS, V31, P3691, DOI 10.1093/bioinformatics/btv421"
"   Pailin GT, 2001, LETT APPL MICROBIOL, V33, P45, DOI 10.1046/j.1472-765X.2001.00954.x"
"   Pal C, 2017, ADV MICROB PHYSIOL, V70, P261, DOI 10.1016/bs.ampbs.2017.02.001"
"   Palmieri D, 2020, NAT COMMUN, V11, DOI 10.1038/s41467-020-18994-5"
"   Park SC, 2019, FRONT MICROBIOL, V10, DOI 10.3389/fmicb.2019.00834"
"   P??rez G, 2011, WORLD J MICROB BIOT, V27, P47, DOI 10.1007/s11274-010-0425-4"
"   Pikovskaia R. I., 1948, Mikrobiologiya, V17, P362"
"   Qadir M, 2020, CHEMOSPHERE, V258, DOI 10.1016/j.chemosphere.2020.127386"
"   Raghavan R. Shree, 2024, Biosciences Biotechnology Research Asia, V21, P947"
"   Rana Kusam Lata, 2021, Journal of Applied Biology & Biotechnology, V9, P41, DOI [10.7324/jabb.2021.9306, 10.7324/JABB.2021.9306]"
"   Roskova Z, 2022, SCI TOTAL ENVIRON, V819, DOI 10.1016/j.scitotenv.2022.153144"
"   Roychowdhury R, 2024, PHYSIOL PLANTARUM, V176, DOI 10.1111/ppl.14399"
"   Sahoo R, 2024, DISCOV APPL SCI, V6, DOI 10.1007/s42452-024-06024-8"
"   SCHWYN B, 1987, ANAL BIOCHEM, V160, P47, DOI 10.1016/0003-2697(87)90612-9"
"   Shimoyama Y., 2022, COGclassifier: A tool for classifying prokaryote protein sequences into COG functional category [Computer software]"
"   Singh AA, 2024, WORLD J MICROB BIOT, V41, DOI 10.1007/s11274-024-04232-3"
"   Smith E, 2025, MICROBIOL-SGM, V171, DOI 10.1099/mic.0.001557"
"   Tariq A, 2025, STRESS BIOL, V5, DOI 10.1007/s44154-024-00168-8"
"   Tatusova T, 2016, NUCLEIC ACIDS RES, V44, P6614, DOI 10.1093/nar/gkw569"
"   Tettelin H, 2005, P NATL ACAD SCI USA, V102, P13950, DOI 10.1073/pnas.0506758102"
"   Timkov?? I, 2024, BIOMETALS, V37, P371, DOI 10.1007/s10534-023-00555-0"
"   Tinna D, 2020, SCI HORTIC-AMSTERDAM, V270, DOI 10.1016/j.scienta.2020.109432"
"   Wang LY, 2013, PLOS GENET, V9, DOI 10.1371/journal.pgen.1003865"
"   Wang Y, 2022, ENVIRON SCI POLLUT R, V29, P3888, DOI 10.1007/s11356-021-15996-8"
"   Wang ZH, 2022, BMC MICROBIOL, V22, DOI 10.1186/s12866-022-02715-7"
"   Yuan LF, 2020, ARCH MICROBIOL, V202, P483, DOI 10.1007/s00203-019-01758-1"
"   Zouagui R, 2024, BMC GENOMICS, V25, DOI 10.1186/s12864-024-10088-6"
NR 75
TC 2
Z9 2
U1 16
U2 35
PU ELSEVIER GMBH
PI MUNICH
"PA HACKERBRUCKE 6, 80335 MUNICH, GERMANY"
SN 0944-5013
EI 1618-0623
J9 MICROBIOL RES
JI Microbiol. Res.
PD OCT
PY 2025
VL 299
AR 128264
DI 10.1016/j.micres.2025.128264
EA JUL 2025
PG 13
WC Microbiology
WE Science Citation Index Expanded (SCI-EXPANDED)
SC Microbiology
GA 4RA8S
UT WOS:001524627300002
PM 40602306
DA 2026-04-06
ER

PT J
"AU Xiao, KM"
"   Li, YZ"

"   Sun, Y"
"   Liu, RY"
"   Li, JJ"
"   Zhao, Y"
"   Xu, H"
"AF Xiao, Kemeng"
"   Li, Yunzhen"
"   Sun, Yang"
"   Liu, Ruyue"
"   Li, Junjie"
"   Zhao, Yun"
"   Xu, Heng"
TI Remediation Performance and Mechanism of Heavy Metals by a Bottom Up
   Activation and Extraction System Using Multiple Biochemical Materials
SO ACS APPLIED MATERIALS & INTERFACES
LA English
DT Article
DE biochemical materials; heavy metals; extraction; mechanism; bacterial
   community
ID GROWTH-PROMOTING BACTERIA; PLANT-GROWTH; DEHYDROGENASE-ACTIVITY;
   CONTAMINATED SOILS; COLORIMETRIC DETERMINATION; INDOLEACETIC-ACID;
   CLITOCYBE-MAXIMA; ORGANIC-ACIDS; BIOCHAR; ALGINATE
"AB Soil contamination with heavy metals has caused serious environmental problems and increased the risks to humans and biota. Herein, we developed an effective bottom up metals removal system based on the synergy between the activation of immobilization metal-resistant bacteria and the extraction of bioaccumulator material (Stropharia rugosoannulata). In this system, the advantages of biochar produced at 400 degrees C and sodium alginate were integrated to immobilize bacteria. Optimized by response surface methodology, the biochar and bacterial suspension were mixed at a ratio of 1:20 (w:v) for 12 h when 2.5% sodium alginate was added to the mixture. Results demonstrated that the system significantly increased the proportion of acid soluble Cd and Cu and improved the soil microecology (microbial counts, soil respiration, and enzyme activities). The maximum extractions of Cd and Cu were 8.79 and 77.92 mg kg(-1), respectively. Moreover, details of the possible mechanistic insight into the metal removal are discussed, which indicate positive correlation with the acetic acid extractable metals and soil microecology. Meanwhile, the ""dilution effect"" in S. rugosoannulata probably plays an important role in the metal removal process. Furthermore, the metal-resistant bacteria in this system were successfully colonized, and the soil bacteria community were evaluated to understand the microbial diversity in metal-contaminated soil after remediation."
"C1 [Xiao, Kemeng; Sun, Yang; Liu, Ruyue; Li, Junjie; Zhao, Yun; Xu, Heng] Sichuan Univ, Coll Life Sci, Key Lab Bioresources & Ecoenvironm, Minist Educ, Chengdu 610064, Sichuan, Peoples R China."
"   [Li, Yunzhen] Sichuan Univ, Civil Engn Postdoctoral Res Stn, Chengdu 610065, Sichuan, Peoples R China."
C3 Sichuan University; Sichuan University
"RP Zhao, Y; Xu, H (corresponding author), Sichuan Univ, Coll Life Sci, Key Lab Bioresources & Ecoenvironm, Minist Educ, Chengdu 610064, Sichuan, Peoples R China."
EM zhaoyun@scu.edu.cn; xuheng64@sina.com
"RI Xu, Heng/AAS-3465-2021; Xiao, Kemeng/OIC-5102-2025; zhao,"
"   yun/AAJ-4105-2020; Liu, Ruyue/AGF-7070-2022"
"OI Xu, Heng/0000-0003-1307-4896; Xiao, Kemeng/0000-0002-8970-3196; "
"FU NSFC [41171253, J1103518]; National Science and Technology Supporting"
   Program [2015BAD05B01-5]; Science and Technology Support Program of
   Sichuan Province [2016NZ0050]
"FX This study was financially supported by the NSFC (41171253, J1103518),"
"   the National Science and Technology Supporting Program (2015BAD05B01-5),"
   and the Science and Technology Support Program of Sichuan Province
   (2016NZ0050). The authors thank Professor Guanglei Cheng from Sichuan
   University for technical assistance.
"CR Adam G, 2001, SOIL BIOL BIOCHEM, V33, P943, DOI 10.1016/S0038-0717(00)00244-3"
"   ANDERSON TH, 1990, SOIL BIOL BIOCHEM, V22, P251, DOI 10.1016/0038-0717(90)90094-G"
"   [Anonymous], [No title captured]"
"   Bai J, 2016, ACS APPL MATER INTER, V8, P25289, DOI 10.1021/acsami.6b08059"
"   BENEFIELD CB, 1977, SOIL BIOL BIOCHEM, V9, P67, DOI 10.1016/0038-0717(77)90063-3"
"   Bolan N, 2014, J HAZARD MATER, V266, P141, DOI 10.1016/j.jhazmat.2013.12.018"
"   BRIC JM, 1991, APPL ENVIRON MICROB, V57, P535, DOI 10.1128/AEM.57.2.535-538.1991"
"   Burd GI, 2000, CAN J MICROBIOL, V46, P237, DOI 10.1139/cjm-46-3-237"
"   Burns RG, 2000, SOIL BIOL BIOCHEM, V32, P1815, DOI 10.1016/S0038-0717(00)00154-1"
"   Byers HK, 1998, FEMS MICROBIOL ECOL, V25, P391, DOI 10.1016/S0168-6496(98)00013-0"
"   Chen BL, 2012, J SOIL SEDIMENT, V12, P1350, DOI 10.1007/s11368-012-0554-5"
"   Chen L, 2014, SOIL BIOL BIOCHEM, V68, P300, DOI 10.1016/j.soilbio.2013.10.021"
"   Davidson CM, 1998, ANAL CHIM ACTA, V363, P45, DOI 10.1016/S0003-2670(98)00057-9"
"   Diaz MA, 2015, SOIL SEDIMENT CONTAM, V24, P16, DOI 10.1080/15320383.2014.907239"
"   DOELMAN P, 1979, SOIL BIOL BIOCHEM, V11, P475, DOI 10.1016/0038-0717(79)90005-1"
"   Epelde L, 2009, SOIL BIOL BIOCHEM, V41, P1788, DOI 10.1016/j.soilbio.2008.04.001"
"   Fellet G, 2014, SCI TOTAL ENVIRON, V468, P598, DOI 10.1016/j.scitotenv.2013.08.072"
"   Fiske CH, 1925, J BIOL CHEM, V66, P375"
"   Gao YZ, 2003, ENVIRON INT, V29, P613, DOI 10.1016/S0160-4120(03)00048-5"
"   Ge ZL, 2011, SCI CHINA CHEM, V54, P1273, DOI 10.1007/s11426-011-4327-6"
"   GIANFREDA L, 1993, PESTIC SCI, V39, P237, DOI 10.1002/ps.2780390310"
"   Glick BR, 1998, J THEOR BIOL, V190, P63, DOI 10.1006/jtbi.1997.0532"
"   G??lser F, 2008, ENVIRON MONIT ASSESS, V145, P127, DOI 10.1007/s10661-007-0022-7"
"   Hale L, 2015, SOIL BIOL BIOCHEM, V81, P228, DOI 10.1016/j.soilbio.2014.11.023"
"   Hu LB, 2009, NEW BIOTECHNOL, V26, P205, DOI 10.1016/j.nbt.2009.09.001"
"   Jarosz-Wilkolazka A, 2002, ENZYME MICROB TECH, V30, P566, DOI 10.1016/S0141-0229(02)00022-4"
"   KANDELER E, 1988, BIOL FERT SOILS, V6, P68, DOI 10.1007/BF00257924"
"   Kauffman JS, 2009, ACS APPL MATER INTER, V1, P1287, DOI 10.1021/am900167r"
"   Lee JW, 2010, ENVIRON SCI TECHNOL, V44, P7970, DOI 10.1021/es101337x"
"   Li FY, 2009, ENVIRON GEOL, V57, P1815, DOI 10.1007/s00254-008-1469-8"

"   Li JH, 2010, J ENVIRON MONITOR, V12, P466, DOI 10.1039/b916389a"
"   Li X, 2017, J HAZARD MATER, V324, P340, DOI 10.1016/j.jhazmat.2016.10.067"
"   Li XZ, 2017, J HAZARD MATER, V330, P1, DOI 10.1016/j.jhazmat.2017.01.047"
"   Li X, 2016, RSC ADV, V6, P65816, DOI 10.1039/c6ra11767h"
"   Liu HY, 2015, ENVIRON SCI POLLUT R, V22, P17182, DOI 10.1007/s11356-015-4941-5"
"   Liu HY, 2015, J HAZARD MATER, V294, P121, DOI 10.1016/j.jhazmat.2015.04.004"
"   Lu KP, 2014, AGR ECOSYST ENVIRON, V191, P124, DOI 10.1016/j.agee.2014.04.010"
"   Lu M, 2014, ENVIRON SCI TECHNOL, V48, P1158, DOI 10.1021/es403337t"
"   Ma Y, 2011, BIOTECHNOL ADV, V29, P248, DOI 10.1016/j.biotechadv.2010.12.001"
"   Ma Y, 2016, J HAZARD MATER, V320, P36, DOI 10.1016/j.jhazmat.2016.08.009"
"   Ma Y, 2009, CHEMOSPHERE, V75, P719, DOI 10.1016/j.chemosphere.2009.01.056"
"   Madhaiyan M, 2013, ANTON LEEUW INT J G, V103, P475, DOI 10.1007/s10482-012-9828-6"
"   Major J, 2012, J ENVIRON QUAL, V41, P1076, DOI 10.2134/jeq2011.0128"
"   Manabe K, 2015, ACS APPL MATER INTER, V7, P4763, DOI 10.1021/am508393n"
"   OBBARD JP, 1994, SCI TOTAL ENVIRON, V142, P157, DOI 10.1016/0048-9697(94)90323-9"
"   Park JH, 2011, PLANT SOIL, V348, P439, DOI 10.1007/s11104-011-0948-y"
"   Patten CL, 2002, APPL ENVIRON MICROB, V68, P3795, DOI 10.1128/AEM.68.8.3795-3801.2002"
"   Qian LB, 2013, ENVIRON SCI TECHNOL, V47, P2737, DOI 10.1021/es3047872"
"   Sangeetha D., 2012, International Journal of Pharmaceutical and Biological Archives, V3, P170"
"   SCHWYN B, 1987, ANAL BIOCHEM, V160, P47, DOI 10.1016/0003-2697(87)90612-9"
"   Tang X, 2016, J ENVIRON MANAGE, V181, P646, DOI 10.1016/j.jenvman.2016.08.043"
"   Valentao P, 2005, J AGR FOOD CHEM, V53, P3626, DOI 10.1021/jf040465z"
"   VANELSAS JD, 1992, BIOL FERT SOILS, V14, P14, DOI 10.1007/BF00336297"
"   Vieira FCS, 2005, MICROBIOL RES, V160, P197, DOI 10.1016/j.micres.2005.01.004"
"   Wang SY, 2017, CHEM ENG J, V313, P567, DOI 10.1016/j.cej.2016.12.039"
"   Wang Y., 2017, COMPOS B, V114, P149, DOI [10.1016/j.compositesb.2017.01.045, DOI 10.1016/J.COMPOSITESB.2017.03.038]"
"   Wu B, 2016, SCI TOTAL ENVIRON, V562, P732, DOI 10.1016/j.scitotenv.2016.04.079"
"   Xiao KM, 2016, RSC ADV, V6, P86068, DOI 10.1039/c6ra14417a"
"   Yao GB, 2015, ANGEW CHEM INT EDIT, V54, P2966, DOI 10.1002/anie.201410895"
"   Zhang GX, 2016, ENVIRON POLLUT, V218, P513, DOI 10.1016/j.envpol.2016.07.031"
"   Zhang HR, 2016, J ENVIRON SCI, V47, P7, DOI 10.1016/j.jes.2015.12.023"
"   Zhang WB, 2014, ACS APPL MATER INTER, V6, P11783, DOI 10.1021/am502541g"
NR 62
TC 54
Z9 64
U1 4
U2 108
PU AMER CHEMICAL SOC
PI WASHINGTON
"PA 1155 16TH ST, NW, WASHINGTON, DC 20036 USA"
SN 1944-8244
J9 ACS APPL MATER INTER
JI ACS Appl. Mater. Interfaces
PD SEP 13
PY 2017
VL 9
IS 36
BP 30448
EP 30457
DI 10.1021/acsami.7b09520
PG 10
"WC Nanoscience & Nanotechnology; Materials Science, Multidisciplinary"
WE Science Citation Index Expanded (SCI-EXPANDED)
SC Science & Technology - Other Topics; Materials Science
GA FH3JR
UT WOS:000411043600023
PM 28836755
DA 2026-04-06
ER

PT J
"AU Liu, SJ"
"   Liu, WZ"
"   Zhang, YG"
"AF Liu, Shujiao"
"   Liu, Weizhong"
"   Zhang, Yonggang"
"TI Application of Substrate Materials Derived from Plant Roots, Stems,"
"   Leaves, and Microbes for Remediation of Soil Heavy Metal Pollution"
SO JOURNAL OF BIOBASED MATERIALS AND BIOENERGY
LA English
DT Article
DE Heavy Metals; Soil; Plants; Substrate Materials
ID ELECTROCHEMICAL BIOSENSOR
"AB This study focuses on the remediation of chromium (Cr), a heavy metal, in soil using substrate mate-rials synthesized from plant roots, stems, leaves, and microbes. Corn was chosen as the source material for biochar production through high-temperature pyrolysis of corn roots, stems, and leaves. The biochar was then modified with an iron solution, and microbial adsorption was employed to remediate heavy metal Cr in the soil. The study involved physical and infrared analyses of the substrate materials and experiments to determine the adsorption contribution of different pH con-ditions, sites, and substrate materials to metal Cr. The effectiveness of plant roots, stems, leaves, and microbial combinations in remediating soil heavy metal pollution was verified. The experimental results demonstrate a decrease in the Zeta potential values of both materials as the pH increases. Moreover, as pH gradually increases, the form of Cr ions changes, and the

reproductive ability of microbes weakens. The substrate materials exhibit a notable increase in negative charge, leading to enhanced repulsion towards Cr, which impacts their adsorption performance. At neutral pH, the IP: 203.8.109.20 On: Tue, 05 Dec 2023 13:24:01 adsorption capacity of the substrate materials for Cr is enhanced. Tcontribution rate of the com-Copyright: American Scientific Publishe rs posite substrate materials in the adsorption process is 6.78%, while that of microbial organisms is Delivered by Ingenta 56.45%, significantly higher than that of Fe-CBC. The main reason is that microbial adsorption activ-ity for Cr is enhanced under higher pH conditions, with the contribution rate of microbial organisms continuing to increase significantly at a pH of 9."
"C1 [Liu, Shujiao; Zhang, Yonggang] Shuozhou Vocatioanl Tech, Shuozhou 036002, Shanxi, Peoples R China."
"   [Liu, Weizhong] Shanxi Normal Univ, Sch Life Sci, Taiyuan 030031, Shanxi, Peoples R China."
C3 Shanxi Normal University
"RP Liu, WZ (corresponding author), Shanxi Normal Univ, Sch Life Sci, Taiyuan 030031, Shanxi, Peoples R China."
EM liuweizhong072707@163.com
"RI Liu, Shujiao/HSH-8079-2023"
FU general research project of agricultural and rural develop-ment
   reform-Research on Rural Bioeconomy Development [NYKJ9284]
FX This work was supported by Shanxi Province Soft Science Research Program
"   Project (Project Number: 2018041001-3) , The key project of the Ministry"
   of Agriculture and Rural Affairs-the sub project of thegeneral research
   project of agricultural and rural development reform-Research on Rural
   Bioeconomy Development (project number: NYKJ9284) .r general research
   project of agricultural and rural develop-ment reform-Research on Rural
   Bioeconomy Development (project number: NYKJ9284) .
"CR Ahmed T, 2021, ECOTOX ENVIRON SAFE, V227, DOI 10.1016/j.ecoenv.2021.112888"
"   Ameen F, 2021, ECOTOX ENVIRON SAFE, V213, DOI 10.1016/j.ecoenv.2021.112027"
"   Andrade-Zavaleta K, 2022, MOLECULES, V27, DOI 10.3390/molecules27041367"
"   Awasthi G, 2022, MINERALS-BASEL, V12, DOI 10.3390/min12010085"
"   Azeez L, 2022, INT J PHYTOREMEDIAT, V24, P384, DOI 10.1080/15226514.2021.1949578"
"   El-Saadony MT, 2021, J ENVIRON SCI, V106, P1, DOI 10.1016/j.jes.2021.01.012"
"   Guo WF, 2021, J NANOBIOTECHNOL, V19, DOI 10.1186/s12951-021-00914-4"
"   Li JH, 2022, SCI TOTAL ENVIRON, V803, DOI 10.1016/j.scitotenv.2021.149866"
"   Li ZS, 2022, RSC ADV, V12, P5062, DOI 10.1039/d1ra08376g"
"   Liu Y, 2018, J BIOMED NANOTECHNOL, V14, P2156, DOI 10.1166/jbn.2018.2655"
"   Liu Y, 2017, J BIOMED NANOTECHNOL, V13, P1253, DOI 10.1166/jbn.2017.2424"
"   Ma J, 2020, ENVIRON POLLUT, V256, DOI 10.1016/j.envpol.2019.113463"
"   Mensah MB, 2021, ROY SOC OPEN SCI, V8, DOI 10.1098/rsos.201485"
"   Nedjimi B, 2021, SN APPL SCI, V3, DOI 10.1007/s42452-021-04301-4"
"   Pehlivan N, 2021, J HAZARD MATER, V403, DOI 10.1016/j.jhazmat.2020.123609"
"   Priyadarshini E, 2021, CHEMOSPHERE, V274, DOI 10.1016/j.chemosphere.2021.129976"
"   Singh A, 2021, ENVIRON SCI POLLUT R, V28, P3942, DOI 10.1007/s11356-020-10737-9"
"   Sun WJ, 2021, CRIT REV ENV SCI TEC, V51, P973, DOI 10.1080/10643389.2020.1740546"
"   Wu J, 2019, J COLLOID INTERF SCI, V555, P403, DOI 10.1016/j.jcis.2019.07.108"
NR 19
TC 1
Z9 1
U1 2
U2 21
PU AMER SCIENTIFIC PUBLISHERS
PI VALENCIA
"PA 26650 THE OLD RD, STE 208, VALENCIA, CA 91381-0751 USA"
SN 1556-6560
EI 1556-6579
J9 J BIOBASED MATER BIO
JI J. Biobased Mater. Bioenergy
PD FEB
PY 2023
VL 18
IS 1
BP 58
EP 63
DI 10.1166/jbmb.2024.2333
PG 6
"WC Chemistry, Applied; Energy & Fuels; Materials Science, Biomaterials"
WE Science Citation Index Expanded (SCI-EXPANDED)
SC Chemistry; Energy & Fuels; Materials Science
GA AG9M9
UT WOS:001117431300001
DA 2026-04-06
ER

PT J
"AU Shah, SA"
"   Aslam, S"
"AF Shah, Sajid Ali"
"   Aslam, Saima"
TI Nanoparticle-mediated mitigation of heavy metal stress in plants: a
   comprehensive review
SO JOURNAL OF NANOPARTICLE RESEARCH
LA English
DT Review
DE Heavy metals; Anthropogenic activities; Reactive oxygen species;
   Nanotechnology; Bioremediation Sustainable agriculture; Food crops
ID ZEA-MAYS L.; ZINC-OXIDE NANOPARTICLES; CADMIUM ACCUMULATION;

GLYCINE-MAX; TOXICITY; MOLYBDENUM; TOLERANCE; SEEDLINGS; GROWTH; SOILS
"AB Heavy metals' soil contamination seriously threatens plant health and agricultural productivity. In response, nanoparticles have emerged as a promising solution to mitigate heavy metal-induced stress in plants. This review examines the effects of various heavy metals such as lead, chromium, arsenic, zinc, cadmium, copper, mercury, and nickel, which are highly toxic to plants and other organisms, whereas metals like barium, antimony, molybdenum (Mo), thallium, and tin are generally considered less harmful. This review focuses on the role of nanoparticles in reducing metal toxicity in plants and improving their physiology. Nanoparticles such as zinc oxide (ZnO), iron oxide (Fe3O4), titanium dioxide (TiO2), and silicon dioxide (SiO2) have demonstrated the ability to boost plant growth, enhance photosynthetic efficiency, and strengthen antioxidant defenses under heavy metal stress. These nanoparticles reduce the uptake of harmful metals, improve nutrient absorption, and regulate gene expression related to stress responses. Additionally, Mo, an essential micronutrient, helps mitigate the effects of heavy metals by enhancing antioxidant enzyme activity, reducing oxidative damage, and facilitating osmolyte accumulation. Gene suppression or regulation induced by heavy metal stress and the upregulation of specific genes by nanoparticles is critical to stress alleviation in various plant species. The combined action of Mo and nanoparticles presents a promising approach to increasing plant tolerance to heavy metal toxicity. This review emphasizes the importance of understanding the mechanisms through which nanoparticles alleviate stress and the potential of Mo in conjunction with nanotechnology as a sustainable strategy to address heavy metal pollution in agricultural systems."
"C1 [Shah, Sajid Ali; Aslam, Saima] Baba Ghulam Shah Badshah Univ, Dept Biotechnol, Rajouri 185234, J&K, India."
"RP Aslam, S (corresponding author), Baba Ghulam Shah Badshah Univ, Dept Biotechnol, Rajouri 185234, J&K, India."
EM saima@bgsbu.ac.in
"CR Abasi F, 2024, BIONANOSCIENCE, V14, P3934, DOI 10.1007/s12668-024-01558-y"
"   Abd Elnabi MK, 2023, TOXICS, V11, DOI 10.3390/toxics11070580"
"   Abdulmajeed AM, 2023, NOT BOT HORTI AGROBO, V51, DOI 10.15835/nbha51113043"
"   Adejumo SA, 2021, ENVIRON GEOCHEM HLTH, V43, P1537, DOI 10.1007/s10653-020-00649-5"
"   Adnan M, 2022, TOXICS, V10, DOI 10.3390/toxics10050231"
"   Afshan S, 2015, ENVIRON SCI POLLUT R, V22, P11679, DOI 10.1007/s11356-015-4396-8"
"   Ahmad J, 2024, 3. Mercuryinduced changes in plant proteomes, DOI [10.22541/au.170665336.64203223/v1, DOI 10.22541/AU.170665336.64203223/V1]"
"   Ahmad Z, 2024, Foliar applied silicon nanoparticles (SiNPs) imparted tolerance to arsenic toxicity by modulating physiological and antioxidant in wheat cultivars, DOI [10.21203/rs.3.rs-4309874/v1, DOI 10.21203/RS.3.RS-4309874/V1]"
"   Ahmed S, 2023, ENVIRON SCI POLLUT R, V30, P41002, DOI 10.1007/s11356-023-25189-0"
"   Ahmed S, 2025, INT J PHYTOREMEDIAT, V27, P733, DOI [10.1080/15226514.2024.2440110, 10.1080/15226514.2024.2383657]"
"   Ahmed T, 2023, J HAZARD MATER, V459, DOI 10.1016/j.jhazmat.2023.132070"
"   Ahmed T, 2021, ENVIRON POLLUT, V288, DOI 10.1016/j.envpol.2021.117785"
"   Alabdallah NM, 2024, HELIYON, V10, DOI 10.1016/j.heliyon.2024.e26537"
"   Alamri S, 2022, ENVIRON POLLUT, V292, DOI 10.1016/j.envpol.2021.118268"
"   Alengebawy A, 2021, TOXICS, V9, DOI 10.3390/toxics9030042"
"   Alhammad BA, 2023, AGRONOMY-BASEL, V13, DOI 10.3390/agronomy13041174"
"   Ali MM., 2021, HEAVY METALS, DOI DOI 10.5772/INTECHOPEN.96805"
"   Ali MA, 2019, REACTIVE OXYGEN, NITROGEN AND SULFUR SPECIES IN PLANTS: PRODUCTION, METABOLISM, SIGNALING AND DEFENSE MECHANISMS, VOLS 1-2, P353"
"   Ali N, 2023, S AFR J BOT, V162, P160, DOI 10.1016/j.sajb.2023.09.004"
"   Ali S, 2023, PLANTS-BASEL, V12, DOI 10.3390/plants12071502"
"   Alshaal T, 2013, ENVIRON CHEM LETT, V11, P295, DOI 10.1007/s10311-013-0406-6"
"   Alshegaihi RM, 2024, J PLANT GROWTH REGUL, V43, P1519, DOI 10.1007/s00344-023-11204-8"
"   Altaf MM, 2024, SCI HORTIC-AMSTERDAM, V338, DOI 10.1016/j.scienta.2024.113635"
"   Altaf MM, 2024, S AFR J BOT, V170, P348, DOI 10.1016/j.sajb.2024.05.038"
"   Anderson AJ, 2018, J AGR FOOD CHEM, V66, P6513, DOI 10.1021/acs.jafc.7b01302"
"   Ansari Mohd Kafeel Ahmad, 2024, Agricultural Reviews, V45, P400, DOI [10.18805/ag.rf-321, 10.18805/ag.RF-321]"
"   Anwar A, 2024, J HAZARD MATER, V468, DOI 10.1016/j.jhazmat.2024.133829"
"   Ashraf H, 2024, J ENVIRON MANAGE, V368, DOI 10.1016/j.jenvman.2024.122239"
"   Asiminicesei DM, 2024, PLANTS-BASEL, V13, DOI 10.3390/plants13060913"
"   Aslam A, 2024, BOT STUD, V65, DOI 10.1186/s40529-024-00420-4"
"   Avila-Quezada GD, 2024, Impact of nanoparticles on plant growth and their entry route into the plants, P83, DOI [10.1016/b978-0-443-15570-3.00015-6, DOI 10.1016/B978-0-443-15570-3.00015-6]"
"   Bano N, 2024, ENVIRON POLLUT, V344, DOI 10.1016/j.envpol.2024.123300"
"   Baraga??o D, 2022, J HAZARD MATER, V433, DOI 10.1016/j.jhazmat.2022.128748"
"   Belowitz R, 2013, AQUAT TOXICOL, V138, P70, DOI 10.1016/j.aquatox.2013.04.013"
"   Bhalerao S A., 2015, International Journal of Pure and Applied Bioscience, V3, P345, DOI [DOI 10.1086/349961, 10.1007/s11356-019-04892-x]"
"   Bharathi C, 2025, PLANT SCI TODAY, V12, DOI 10.14719/pst.5192"
"   Boffito C, 1991, Kirk-Othmer encyclopedia of chemical technology, V4th, P902, DOI [10.1002/0471238961.0201180902150606.a01.pub2, DOI 10.1002/0471238961.0201180902150606.A01.PUB2]"
"   Bolan NS, 2013, ADV AGRON, V119, P183, DOI 10.1016/B978-0-12-407247-3.00004-4"
"   Boojar MMA, 2011, J PLANT NUTR, V34, P1532, DOI 10.1080/01904167.2011.585209"
"   Borkar P, 2024, Computational approaches in biomedical engineering, DOI [10.1201/9781032699882, DOI 10.1201/9781032699882]"
"   Brise??o-Bugar??n J, 2024, ENVIRONMENTS, V11, DOI 10.3390/environments11030043"
"   Brisson BA, 2022, Determination of the toxic effects of heavy metals on the morpho-anatomical responses of the leaf of Typha latifolia as a biomonitoring tool, DOI [10.21203/rs.3.rs-2290740/v1, DOI 10.21203/RS.3.RS-2290740/V1]"
"   Cakaj A, 2024, ENVIRON RES, V256, DOI 10.1016/j.envres.2024.119222"
"   Cao YH, 2025, FRONT PLANT SCI, V15, DOI 10.3389/fpls.2024.1510482"
"   Chai SY, 2024, J HAZARD MATER, V470, DOI 10.1016/j.jhazmat.2024.134245"
"   Chakrabarty D, 2024, FRONT PLANT SCI, V15, DOI 10.3389/fpls.2024.1502021"
"   Chang HF, 2024, ECOTOX ENVIRON SAFE, V276, DOI 10.1016/j.ecoenv.2024.116290"
"   Chanu L. B., 2016, Current World Environment, V11, P218, DOI [10.12944/cwe.11.1.27, 10.12944/CWE.11.1.27]"

"   Chaplygin VA, 2024, EURASIAN SOIL SCI+, V57, P1746, DOI 10.1134/S1064229324601501"
"   Chen CY, 2009, CLEAN-SOIL AIR WATER, V37, P304, DOI 10.1002/clen.200800199"
"   Chen F, 2022, ENVIRON POLLUT, V308, DOI 10.1016/j.envpol.2022.119606"
"   Chen H, 2024, J NANOBIOTECHNOL, V22, DOI 10.1186/s12951-024-02667-2"
"   Chen Z, 2024, J HAZARD MATER, V469, DOI 10.1016/j.jhazmat.2024.133917"
"   Daler S, 2024, HORTICULTURAE, V10, DOI 10.3390/horticulturae10101103"
"   Dashtey A, 2024, International Journal of Scientific Research and Management (IJSRM), V12, P202, DOI [10.18535/ijsrm/v12i12.c01, 10.18535/ijsrm/v12i12.c01, DOI 10.18535/IJSRM/V12I12.C01]"
"   DiBello PM, 1991, Kirk-Othmer encyclopedia of chemical technology, V4th, P909, DOI [10.1002/0471238961.0201180904090205.a01, DOI 10.1002/0471238961.0201180904090205.A01]"
"   Dixit V, 2024, PLANT SCI TODAY, V11, P569, DOI 10.14719/pst.3715"
"   Donnat-Aracil C, 2023, Phytotoxicity responses and defence mechanisms of heavy metal and metal-based nanoparticles, P59, DOI [10.1007/978-981-99-2419-6_4, DOI 10.1007/978-981-99-2419-6_4]"
"   Doria-Manzur A, 2023, INT J PHYTOREMEDIAT, V25, P98, DOI 10.1080/15226514.2022.2060934"
"   Nguyen DTC, 2023, SCI TOTAL ENVIRON, V857, DOI 10.1016/j.scitotenv.2022.159278"
"   Emamverdian A, 2022, FRONT PLANT SCI, V13, DOI 10.3389/fpls.2022.841501"
"   Ertani A, 2017, WATER AIR SOIL POLL, V228, DOI 10.1007/s11270-017-3356-y"
"   Espinosa F, 2023, ANTIOXIDANTS-BASEL, V12, DOI 10.3390/antiox12030678"
"   Ewubare PO, 2024, 1. An academic review on heavy metals in the environment: effects on soil, plants human health, and possible solutions, DOI [10.54536/ajee.v3i1.3261, DOI 10.54536/AJEE.V3I1.3261]"
"   Faizan M, 2023, NANOMATERIALS-BASEL, V13, DOI 10.3390/nano13060974"
"   Faizan M, 2023, SUSTAINABILITY-BASEL, V15, DOI 10.3390/su15021446"
"   Fan SX, 2024, 1. Screening and stress responsive characteristics of potential hyperaccumulator of Pb, Zn, and Cd compound heavy metals, DOI [10.13227/j.hjkx.202308262, DOI 10.13227/J.HJKX.202308262]"
"   Farhat F, 2022, FRONT PLANT SCI, V13, DOI 10.3389/fpls.2022.904893"
"   Farmand M, 2022, 3 BIOTECH, V12, DOI 10.1007/s13205-022-03175-6"
"   Fathy WA, 2024, INT J PHYTOREMEDIAT, V26, P1952, DOI 10.1080/15226514.2024.2370022"
"   Fazal H, 2016, Environ Sci Pollution Res., DOI [10.1007/S11356-016-7230-Z, DOI 10.1007/S11356-016-7230-Z]"
"   Ferreira P.A.A., 2018, Physiological, biochemical changes, and phytotoxicity remediation in agricultural plant species cultivated in soils contaminated with copper and zinc, P29, DOI DOI 10.1007/978-981-13-2242-6_2"
"   Ferronato C, 2016, WATER AIR SOIL POLL, V227, DOI 10.1007/s11270-016-3027-4"
"   Francis DV, 2024, ENVIRON INT, V190, DOI 10.1016/j.envint.2024.108859"
"   Gao JY, 2024, J ENVIRON MANAGE, V361, DOI 10.1016/j.jenvman.2024.121289"
"   Gaozhong Pu, 2017, Journal of Environmental Science and Engineering - A, V6, DOI [10.17265/2162-5298/2017.01.002, 10.17265/2162-5298/2017.01.002, DOI 10.17265/2162-5298/2017.01.002]"
"   Garg T, 2024, S AFR J BOT, V168, P296, DOI 10.1016/j.sajb.2024.03.020"
"   Gatasheh MK, 2024, BMC PLANT BIOL, V24, DOI 10.1186/s12870-024-05359-z"
"   Ghorbani A, 2024, J NANOBIOTECHNOL, V22, DOI 10.1186/s12951-024-02371-1"
"   Ghosh S, 2022, Metal stress removal and nanotechnology-driven solutions, P129, DOI [10.1016/b978-0-323-91908-1.00004-3, DOI 10.1016/B978-0-323-91908-1.00004-3]"
"   Ghouri F, 2024, SCI REP-UK, V14, DOI 10.1038/s41598-024-55810-2"
"   Gohari G, 2020, SCI REP-UK, V10, DOI 10.1038/s41598-020-57794-1"
"   Gomez-Gonzalez MA, 2015, SCI TOTAL ENVIRON, V536, P268, DOI 10.1016/j.scitotenv.2015.07.033"
"   Gouranga U, 2024, 4. Potential of silver and zinc nanoparticles in mediating abiotic stress tolerance in crop plants, DOI [10.1007/978-981-99-7673-7_8, DOI 10.1007/978-981-99-7673-7_8]"
"   Gracia-Rodriguez C, 2024, HELIYON, V10, DOI 10.1016/j.heliyon.2024.e29140"
"   Guo J, 2021, ECOTOX ENVIRON SAFE, V226, DOI 10.1016/j.ecoenv.2021.112857"
"   Gupta N, 2022, PLANTS-BASEL, V11, DOI 10.3390/plants11172227"
"   Haghaninia M, 2024, HORTICULTURAE, V10, DOI 10.3390/horticulturae10060659"
"   Han ZX, 2020, INT J ENV RES PUB HE, V17, DOI 10.3390/ijerph17072355"
"   Hasan A, 2024, ENVIRON QUAL MANAG, V34, DOI 10.1002/tqem.70018"
"   Hasanuzzaman M, 2020, ANTIOXIDANTS-BASEL, V9, DOI 10.3390/antiox9080681"
"   Hassan KM, 2024, HORTICULTURAE, V10, DOI 10.3390/horticulturae10080806"
"   Hassan SH, 2023, Lead toxicity and tolerance in plants, P373, DOI [10.1002/9781119906506.ch18, DOI 10.1002/9781119906506.CH18]"
"   Hawezy HJS, 2025, REV INORG CHEM, V45, P587, DOI 10.1515/revic-2024-0090"
"   Helaoui S, 2023, 2. Nickel toxicity and tolerance in plants, DOI [10.1002/9781119906506.ch11, DOI 10.1002/9781119906506.CH11]"
"   Hoque MN, 2021, INT J MOL SCI, V22, DOI 10.3390/ijms222111445"
"   Hu CX, 2020, Biomedical Journal of Scientific & Technical Research, V24, DOI [10.26717/bjstr.2020.24.004104, https://doi.org/10.26717/BJSTR.2020.24.004104, DOI 10.26717/BJSTR.2020.24.004104]"
"   Hu ZW, 2023, AGRONOMY-BASEL, V13, DOI 10.3390/agronomy13102601"
"   Huang Q, 2024, ENVIRON POLLUT, V342, DOI 10.1016/j.envpol.2023.123013"
"   Huang YM, 2024, J ENVIRON SCI, V135, P10, DOI 10.1016/j.jes.2023.01.001"
"   Huang YQ, 2024, ECOTOX ENVIRON SAFE, V273, DOI 10.1016/j.ecoenv.2024.116166"
"   Imran M, 2021, ANTIOXIDANTS-BASEL, V10, DOI 10.3390/antiox10060838"
"   Imran M, 2020, BIOMOLECULES, V10, DOI 10.3390/biom10111582"
"   ing S, 2024, 4. Enhanced cadmium absorption and tolerance of rice epiphytic microbes by iron oxide nanoparticles, DOI [10.1016/j.ibiod.2024.105770, DOI 10.1016/J.IBIOD.2024.105770]"
"   Iqbal I, 2024, PAK J BOT, V56, P2129, DOI 10.30848/PJB2024-6(10)"
"   Jalil S, 2023, FUNCT PLANT BIOL, V50, P870, DOI 10.1071/FP23021"
"   Jebarani H, 2025, Nanoparticle-assisted abiotic remediation of contaminated soil, P239, DOI [10.1201/9781003477730-17, DOI 10.1201/9781003477730-17]"
"   Jesitha K., 2021, NANOMATERIALS SOIL R, P463, DOI [DOI 10.1016/B978-0-12-822891-3.00027-X, 10.1016/B978-0-12-822891-3.00027-X]"
"   Jojevic ST, 2024, LAND-BASEL, V13, DOI 10.3390/land13122075"
"   Jorjani Sarah, 2024, International Journal of Secondary Metabolite, V11, P169, DOI [10.21448/ijsm.1323494, 10.21448/ijsm.1323494]"
"   Juan CA, 2021, INT J MOL SCI, V22, DOI 10.3390/ijms22094642"
"   Jutsz AM, 2015, ARCH ENVIRON PROT, V41, P104, DOI 10.1515/aep-2015-0045"
"   Kabata-Pendias Alina, 2010, P1"

"   Kappler U, 2015, J BIOL INORG CHEM, V20, P253, DOI 10.1007/s00775-014-1197-3"
"   Kaur H, 2021, PLANTA, V253, DOI 10.1007/s00425-021-03642-z"
"   Kaur P, 2022, SUSTAINABILITY-BASEL, V14, DOI 10.3390/su14020935"
"   Khalid HI, 2023, PAK J AGR SCI, V60, P377, DOI 10.21162/PAKJAS/23.65"
"   Khalofah A, 2021, PLANTS-BASEL, V10, DOI 10.3390/plants10071349"
"   Khan S, 2021, TOXICS, V9, DOI 10.3390/toxics9010002"
"   Khan ZA, 2024, 4. Exogenous GABA enhances copper stress resilience in rice plants via antioxidant, DOI [10.3390/antiox13060700, DOI 10.3390/ANTIOX13060700]"
"   Khan Z, 2023, PLANT GROWTH REGUL, V100, P409, DOI 10.1007/s10725-023-00988-0"
"   Khushbu S, 2024, 4. Mercury phytovolatilization: an overview of the mechanism and mitigation, DOI [10.1016/b978-0-443-15291-7.00031-6, DOI 10.1016/B978-0-443-15291-7.00031-6]"
"   Kiany T, 2022, ENVIRON SCI POLLUT R, V29, P34725, DOI 10.1007/s11356-021-17927-z"
"   Kohli SK, 2020, REV ENVIRON CONTAM T, V249, P153, DOI 10.1007/398_2019_25"
"   Konate A, 2017, SUSTAINABILITY-BASEL, V9, DOI 10.3390/su9050790"
"   Kondak S, 2024, ENVIRON RES, V260, DOI 10.1016/j.envres.2024.119665"
"   Kreslavski VD, 2023, FUNCT PLANT BIOL, V50, P932, DOI 10.1071/FP23085"
"   Kumar A, 2023, J PLANT NUTR, V46, P2351, DOI 10.1080/01904167.2022.2155542"
"   Kumar D, 2024, SCI TOTAL ENVIRON, V930, DOI 10.1016/j.scitotenv.2024.172413"
"   Kumari N, 2024, REV ENVIRON CONTAM T, V262, DOI 10.1007/s44169-023-00053-x"
"   Kumchai J, 2013, GENET MOL RES, V12, P5589, DOI 10.4238/2013.November.18.8"
"   Lamb DT, 2013, ENVIRON SCI TECHNOL, V47, P4670, DOI 10.1021/es302053d"
"   Lawson-Wood K, 2021, ENVIRON SCI POLLUT R, V28, P48929, DOI 10.1007/s11356-021-13902-w"
"   Lebaka VR, 2025, NANOMATERIALS-BASEL, V15, DOI 10.3390/nano15100754"
"   Leela P, 2024, Interactions of lead, zinc and nickel toxicity in their effects on growth responses of cicer arietinum l. and brassica nigra l, P90, DOI [10.58532/v3bkbt21p1ch8, DOI 10.58532/V3BKBT21P1CH8]"
"   Li YF, 2022, BIOL TRACE ELEM RES, V200, P2727, DOI 10.1007/s12011-021-02889-7"
"   Li YY, 2020, ENVIRON SCI-NANO, V7, P1807, DOI [10.1039/d0en00091d, 10.1039/D0EN00091D]"
"   Lisboa TP, 2020, J ANAL TOXICOL, V44, P514, DOI 10.1093/jat/bkz106"
"   Liu HD, 2023, PLANTS-BASEL, V12, DOI 10.3390/plants12010044"
"   Llugany M, 2000, ARCH ENVIRON CON TOX, V39, P440, DOI 10.1007/s002440010125"
"   Lu QH, 2019, APPL GEOCHEM, V106, P142, DOI 10.1016/j.apgeochem.2019.05.010"
"   Madejon P, 2013, Heavy metals in soilsTrace metals and metalloids in soils and their bioavailability, V3rd, P507, DOI [10.1007/978-94-007-4470-7_19, DOI 10.1007/978-94-007-4470-7_19]"
"   Madjar RM, 2025, SUSTAINABILITY-BASEL, V17, DOI 10.3390/su17167368"
"   Mahas ??S, 2024, Problems of applied mathematics and mathematic modeling, P119, DOI [10.15421/322412, 10.1016/b978-0-443-18927-2.00019-4, DOI 10.1016/B978-0-443-18927-2.00019-4]"
"   Mahmood S, 2025, RUSS J PLANT PHYSL+, V72, DOI 10.1134/S1021443724610693"
"   Mahmood T, 2012, Metallic phytoremediationand extractionofnanoparticles"
"   Majumder B, 2023, Arsenic toxicity and tolerance in plants, P293, DOI [10.1002/9781119906506.ch14, DOI 10.1002/9781119906506.CH14]"
"   Malik S, 2024, Adv Environ Eng Green Technol Book Ser, DOI [10.4018/979-8-3693-1471-5.ch009, DOI 10.4018/979-8-3693-1471-5.CH009]"
"   Manna I, 2021, FRONT PLANT SCI, V12, DOI 10.3389/fpls.2021.586509"
"   Mansoor S, 2023, PLANTS-BASEL, V12, DOI 10.3390/plants12163003"
"   Manzoor N, 2023, ECOTOX ENVIRON SAFE, V264, DOI 10.1016/j.ecoenv.2023.115382"
"   Maqsood M, 2024, Earth Environ Sci Library, DOI [10.1007/978-3-031-48817-7_2, DOI 10.1007/978-3-031-48817-7_2]"
"   Maqsood MF, 2023, PLANT STRESS, V10, DOI 10.1016/j.stress.2023.100281"
"   Masruri MK, 2024, Vegetative growth response of rice in heavy metal contaminated soil due to NPK fertilizer based ameliorant application, DOI [10.55677/ijlsar/v03i3y2024-05, DOI 10.55677/IJLSAR/V03I3Y2024-05]"
"   Mathur S, 2022, FRONT SUSTAIN FOOD S, V6, DOI 10.3389/fsufs.2022.932424"
"   Mazhar S, 2023, BIOSCIENTIFIC REV, V5, P55, DOI [10.32350/bsr.53.06, 10.32350/bsr.53.06, DOI 10.32350/BSR.53.06]"
"   Meena V, 2022, SILICON-NETH, V14, P4447, DOI 10.1007/s12633-021-01200-6"
"   Mendel RR, 2024, MOLECULES, V29, DOI 10.3390/molecules29184458"
"   Mir AR, 2022, PLANT PHYSIOL BIOCH, V185, P244, DOI 10.1016/j.plaphy.2022.06.006"
"   Miranda-Villag??mez E, 2019, J NANOMATER, V2019, DOI 10.1155/2019/5368027"
"   Mishra S., 2024, De in Various sources of heavy metals contamination, including industrial activities, mining, agriculture, and urbanization, P71, DOI [10.1201/9781032685793, DOI 10.1201/9781032685793]"
"   Mittal D, 2020, FRONT NANOTECHNOL, V2, DOI 10.3389/fnano.2020.579954"
"   Mocek-Pl??ciniak A, 2023, PLANTS-BASEL, V12, DOI 10.3390/plants12081653"
"   Modi G, 2023, International Journal of Advances in Agricultural Science and Technology, V10, P28, DOI [10.47856/ijaast.2022.v10i01.003, DOI 10.47856/IJAAST.2022.V10I01.003]"
"   Mohammad F, 2023, Sustainability, DOI [10.3390/su15021446, DOI 10.3390/SU15021446]"
"   Mondal S, 2022, PLANTA, V255, DOI 10.1007/s00425-022-03869-4"
"   Mukarram M, 2024, FRONT PLANT SCI, V15, DOI 10.3389/fpls.2024.1377964"
"   Mustafa N, 2022, MOLECULES, V27, DOI 10.3390/molecules27154884"
"   Natasha, 2020, INT J PHYTOREMEDIAT, V22, P617, DOI 10.1080/15226514.2019.1701980"
"   Nazir MM., 2020, PLANT FAMILY BRASSIC, P379, DOI [10.1007/978-981-15-6345-4_13, DOI 10.1007/978-981-15-6345-4_13]"
"   Nazir MM, 2022, FRONT PLANT SCI, V13, DOI 10.3389/fpls.2022.843795"
"   Nilima K, 2023, Rev Environ Contam Toxicol, DOI [10.1007/s44169-023-00053-x, DOI 10.1007/S44169-023-00053-X]"
"   Ni??o-Savala AG, 2019, FRONT AGRIC SCI ENG, V6, P419, DOI 10.15302/J-FASE-2019273"
"   Nofal A, 2024, Journal of Sustainable Agricultural and Environmental Sciences, V3, P98, DOI 10.21608/jsaes.2024.270332.1080"
"   Okereafor U, 2020, INT J ENV RES PUB HE, V17, DOI 10.3390/ijerph17072204"
"   Oskarsson A, 2015, Barium, P625, DOI [10.1016/B978-0-444-59453-2.00029-9, DOI 10.1016/B978-0-444-59453-2.00029-9]"
"   Ouyang LN, 2024, FRONT PLANT SCI, V15, DOI 10.3389/fpls.2024.1444227"
"   Palmer CM, 2009, NAT CHEM BIOL, V5, P333, DOI 10.1038/nchembio.166"
"   Pande A, 2022, FRONT PLANT SCI, V13, DOI 10.3389/fpls.2022.1019647"

" Pathak K, 2021, 5. Molybdenum mediated mitigation of oxidative stress in Triticum durum (HI 8737) seedlings caused due to aluminium, DOI [10.1007/S40502-021-00598-W, DOI 10.1007/S40502-021-00598-W]"
" Pathak Kratika, 2024, Vegetos, V38, P1970, DOI [10.1007/s42535-024-00981-6, 10.1007/s42535-024-00981-6]"
" Pena A, 2023, Environ Health Perspectives, DOI [10.1289/isee.2023.ep-008, DOI 10.1289/ISEE.2023.EP-008]"
" Petersen E, 2023, ENVIRON INT, V173, DOI 10.1016/j.envint.2022.107650"
" Pirzadah TB, 2024, 2. Proteomic response of Tartary buckwheat under mercury-induced stress, DOI [10.21203/rs.3.rs-3957652/v1, DOI 10.21203/RS.3.RS-3957652/V1]"
" Prakash D.V.S., 2025, Nanoparticle Uptake, Translocation, and Biological Impact in Plants, P79, DOI [10.1201/9781003477730-7, DOI 10.1201/9781003477730-7]"
" Pronold-Gunthner F, 2023, Determination of trace concentrations of barium extracted from aqueous systems, DOI [10.31390/gradschool_disstheses.8375, DOI 10.31390/GRADSCHOOL_DISSTHESES.8375]"
" Putros MD, 2024, Al-Salam Journal for Engineering and Technology, V4, P27, DOI [10.55145/ajest.2025.04.01.003, 10.55145/ajest.2025.04.01.003, DOI 10.55145/AJEST.2025.04.01.003]"
" Qin M, 2024, ACS SUSTAIN CHEM ENG, V12, P4767, DOI 10.1021/acssuschemeng.4c00385"
" Qu LL, 2024, J HAZARD MATER, V461, DOI 10.1016/j.jhazmat.2023.132641"
" Radic S, 2009, ENVIRON TOXICOL CHEM, V28, P189, DOI 10.1897/08-188.1"
" Raghu V, 2001, ENVIRON GEOL, V40, P1265, DOI 10.1007/s002540100308"
" Rahman SU, 2024, HELIYON, V10, DOI 10.1016/j.heliyon.2024.e27724"
" Rahman SU, 2024, Heliyon, DOI [10.2139/ssrn.4620053, DOI 10.2139/SSRN.4620053]"
" Rai P, 2022, PLANT NANO BIOL, V2, DOI 10.1016/j.plana.2022.100017"
" Rai PK, 2023, SCI TOTAL ENVIRON, V902, DOI 10.1016/j.scitotenv.2023.166064"
" Raj P, 2024, Asian Journal of Soil Science and Plant Nutrition, V10, P684, DOI [10.9734/ajsspn/2024/v10i4439, DOI 10.9734/AJSSPN/2024/V10I4439]"
" Rajan R, 2025, Future scope of nanotechnology in agriculture, P256, DOI [10.1201/9781003354253-16, DOI 10.1201/9781003354253-16]"
" Rajput VD, 2024, Adv Environ Eng Green Technol Book Ser, DOI [10.4018/979-8-3693-1471-5.ch005, DOI 10.4018/979-8-3693-1471-5.CH005]"
" Rajput VD, 2021, BIOLOGY-BASEL, V10, DOI 10.3390/biology10080791"
" Raza HZ, 2024, Unravelling the role silica nanoparticles to ameliorate chromium heavy metal stress in Mexican marigold (Tagetes erecta L.), DOI [10.21203/rs.3.rs-4924549/v1, DOI 10.21203/RS.3.RS-4924549/V1]"
" Rehman A, 2024, J HAZARD MATER, V470, DOI 10.1016/j.jhazmat.2024.134130"
" Renkema H, 2015, ENVIRON SCI POLLUT R, V22, P2166, DOI 10.1007/s11356-014-3490-7"
" Rizwan M, 2019, ENVIRON POLLUT, V248, P358, DOI 10.1016/j.envpol.2019.02.031"
" Rob MM, 2024, J PLANT PHYSIOL, V302, DOI 10.1016/j.jplph.2024.154314"
" Romele A, 2022, 5. Physiological role, toxicity, hyperaccumulation, and tolerance of nickel in plants, DOI [10.1016/b978-0-323-85621-8.00001-7, DOI 10.1016/B978-0-323-85621-8.00001-7]"
" Roy A, 2024, GREEN PROCESS SYNTH, V13, DOI 10.1515/gps-2024-0150"
" Roy TK, 2024, J CROP HEALTH, V77, DOI 10.1007/s10343-024-01069-6"
" Roychoudhury A, 2022, PLANT NUTR FOOD SECU, P255, DOI DOI 10.1016/B978-0-12-822916-3.00021-4"
" Rudra S, 2024, Harnessing nature's power for eco-friendly metal nanoparticle synthesis and applications, DOI [10.22541/au.173307436.68964338/v1, DOI 10.22541/AU.173307436.68964338/V1]"
" Sachdev S, 2021, 2. Role of nanomaterials in regulating oxidative stress in plants, DOI [10.1007/978-3-030-73606-4_13, DOI 10.1007/978-3-030-73606-4_13]"
" Saeed W, 2024, FRONT PLANT SCI, V15, DOI 10.3389/fpls.2024.1332426"
" Samani M, 2024, BMC PLANT BIOL, V24, DOI 10.1186/s12870-024-05311-1"
" Samar G, 2024, Plant Soil, DOI [10.1007/s11104-024-06581-2, DOI 10.1007/S11104-024-06581-2]"
" Sameer A, 2024, INT J PHYTOREMEDIAT, V26, P1728, DOI 10.1080/15226514.2024.2351508"
" S??nchez-Chapul L, 2023, FRONT GENET, V14, DOI 10.3389/fgene.2023.1168713"
" Sarraf M, 2022, PLANTS-BASEL, V11, DOI 10.3390/plants11030316"
" Sati A, 2025, ACS OMEGA, V10, P7549, DOI 10.1021/acsomega.4c11045"
" Satyaki B, 2024, Plant Physiol Biochem, DOI [10.1016/j.plaphy.2024.108956, DOI 10.1016/J.PLAPHY.2024.108956]"
" Sebastian A, 2016, ENVIRON SCI POLLUT R, V23, P1224, DOI 10.1007/s11356-015-5346-1"
" Sehrish AK, 2024, SCI REP-UK, V14, DOI 10.1038/s41598-024-70204-0"
" Sehrish Adiba Khan, 2024, Chemosphere, V364, P143113, DOI [10.1016/j.chemosphere.2024.143113, 10.1016/j.chemosphere.2024.143113]"
" Sehrish AK, 2024, Comparative efficacy of titanium oxide nanoparticles and zinc oxide nanoparticles against lead tolerance, growth performance and nutrient profiling of Brassica Napus L. grown under Lead contaminated soil, DOI [10.21203/rs.3.rs-3684389/v1, DOI 10.21203/RS.3.RS-3684389/V1]"
" Sembada Anca Awal, 2024, BIO Web of Conferences, V91, DOI 10.1051/bioconf/20249101005"
" Sethi S., 2023, Phytochelatins: heavy metal detoxifiers in plants, P361, DOI DOI 10.1007/978-981-99-2598-8_16"
" Shah AA, 2024, PLANT PHYSIOL BIOCH, V210, DOI 10.1016/j.plaphy.2024.108624"
" Shah TR, 2024, ENVIRON EXP BOT, V220, DOI 10.1016/j.envexpbot.2024.105697"
" Shakoor Noman, 2024, Chemosphere, V356, P141767, DOI [10.1016/j.chemosphere.2024.141767, 10.1016/j.chemosphere.2024.141767]"
" Shang CJ, 2024, ECOTOX ENVIRON SAFE, V281, DOI 10.1016/j.ecoenv.2024.116609"
" Sharma N, 2024, FRONT MICROBIOL, V15, DOI 10.3389/fmicb.2024.1374303"
" Sharma P, 2024, Asian Journal of Advances in Agricultural Research, V24, P75, DOI [10.9734/ajaar/2024/v24i12574, 10.9734/ajaar/2024/v24i12574, DOI 10.9734/AJAAR/2024/V24I12574]"
" Sharma S, 2023, ENVIRON POLLUT, V335, DOI 10.1016/j.envpol.2023.122031"
" Shelke DB, 2023, BIOCATAL AGR BIOTECH, V52, DOI 10.1016/j.bcab.2023.102805"
" Shen X, 2024, ADV MATER, V36, DOI 10.1002/adma.202402745"
" Siddiqi KS, 2017, NANOSCALE RES LETT, V12, DOI 10.1186/s11671-017-1861-y"
" Siddiqui MH, 2021, ENVIRON POLLUT, V290, DOI 10.1016/j.envpol.2021.117953"
" SIEGEL BZ, 1976, BIOINORG CHEM, V6, P341, DOI 10.1016/S0006-3061(00)80019-8"
" Sikdar Ashim, 2022, Agrochemicals in soil and environment: impacts and remediation, P367, DOI 10.1007/978-981-16-9310-6_16"
" Singh A, 2025, Nanotechnology-mediated stress mitigation in plants, P147, DOI [10.1201/9781003354253-9, DOI 10.1201/9781003354253-9]"
" Singh A L., 2010, Plant nutrition and abiotic stress tolerance II-plant stress, P31"
" Singh HP, 2013, ENVIRON CHEM LETT, V11, P229, DOI 10.1007/s10311-013-0407-5"
" Singh J, 2016, J ENVIRON MANAGE, V170, P88, DOI 10.1016/j.jenvman.2016.01.015"
" Singh Y, 2024, IND CROP PROD, V212, DOI 10.1016/j.indcrop.2024.118378"

"   Soumya V, 2022, PLANT SCI TODAY, V9, P623, DOI 10.14719/pst.1621"
"   Student of M.Sc Guru Nanak Institute of Pharmaceutical Science and Technology Kolkata West Bengal - 700114 India., 2023, International Journal of Advanced Research, V11, P168, DOI [10.21474/ijar01/16236, 10.21474/IJAR01/16236, DOI 10.21474/IJAR01/16236]"
"   Sturikova H, 2018, J HAZARD MATER, V349, P101, DOI 10.1016/j.jhazmat.2018.01.040"
"   Sultan MW, 2025, ENVIRON RES, V265, DOI 10.1016/j.envres.2024.120457"
"   Syed Mazhar Irfan, 2023, Proceedings of the Pakistan Academy of Sciences: B. Life and Environmental Sciences, V60, DOI [10.53560/ppasb(60-3)854, DOI 10.53560/PPASB(60-3)854]"
"   Thompson CW, 2022, Toxic thallium effects on cells, DOI [10.1016/b978-0-323-95531-7.00005-7, DOI 10.1016/B978-0-323-95531-7.00005-7]"
"   Tippani R, 2025, Green nanoparticles used in agriculture to reduce ROS and increase plant resilience against abiotic stress, P94, DOI [10.1201/9781003477730-8, DOI 10.1201/9781003477730-8]"
"   Tombuloglu G, 2024, PLANT PHYSIOL BIOCH, V210, DOI 10.1016/j.plaphy.2024.108616"
"   Tran TT, 2024, J HORTIC SCI-INDIA, V19, DOI 10.24154/jhs.v19i1.2267"
"   Tumolo M, 2020, INT J ENV RES PUB HE, V17, DOI 10.3390/ijerph17155438"
"   Ullah J, 2024, BMC PLANT BIOL, V24, DOI 10.1186/s12870-024-05627-y"
"   Ullah S, 2020, J ENVIRON MANAGE, V263, DOI 10.1016/j.jenvman.2020.110365"
"   Rahim HU, 2021, NANOMATERIALS-BASEL, V11, DOI 10.3390/nano11082068"
"   Uzma S, 2021, 3. Role of nanomaterials in regulating reactive species as a signaling molecule of abiotic stress in plants, DOI [10.1007/978-3-030-73606-4_12, DOI 10.1007/978-3-030-73606-4_12]"
"   Vaishnavi J, 2025, FRONT SOIL SCI, V5, DOI 10.3389/fsoil.2025.1484039"
"   Vas D, 2023, Bull Pure Appl Sci Sec B-Botany, DOI [10.48165/bpas.2023.42b.1.7, DOI 10.48165/BPAS.2023.42B.1.7]"
"   Vasilachi IC, 2023, AGRICULTURE-BASEL, V13, DOI 10.3390/agriculture13101983"
"   Verma N, 2024, FRONT CHEM, V12, DOI 10.3389/fchem.2024.1432469"
"   Vijay P, 2025, Mobility of nanoparticles in plants, P125, DOI [10.1201/9781003477730-9, DOI 10.1201/9781003477730-9]"
"   Vyas T. K., 2024, Improving stress resilience in plants by nanoparticles, P73, DOI DOI 10.1016/B978-0-443-18927-2.00023-6"
"   Vyas TK, 2024, 6. Improving stress resilience in plants by nanoparticles, DOI [10.1016/b978-0-443-18927-2.00023-6, DOI 10.1016/B978-0-443-18927-2.00023-6]"
"   Wahab A, 2023, SUSTAINABILITY-BASEL, V15, DOI 10.3390/su152014792"
"   Wang B, 2024, PLANT PHYSIOL BIOCH, V217, DOI 10.1016/j.plaphy.2024.109303"
"   Wang M, 2024, ENVIRON SCI TECHNOL, V58, P6900, DOI 10.1021/acs.est.3c10506"
"   Wang RX, 2024, ENVIRON EXP BOT, V218, DOI 10.1016/j.envexpbot.2023.105590"
"   Wang XK, 2017, ACTA PHYSIOL PLANT, V39, DOI 10.1007/s11738-017-2424-7"
"   Wang ZJ, 2023, AGRONOMY-BASEL, V13, DOI 10.3390/agronomy13123060"
"   Wani KI, 2022, ENVIRON POLLUT, V315, DOI 10.1016/j.envpol.2022.120350"
"   Wen J, 2024, 5. Current advances of the valorization technologies for heavy metal containing hyperaccumulators, DOI [10.1016/j.indcrop.2024.118051, DOI 10.1016/J.INDCROP.2024.118051]"
"   Wu MM, 2024, J HAZARD MATER, V472, DOI 10.1016/j.jhazmat.2024.134516"
"   Wu SW, 2020, J EXP BOT, V71, P5074, DOI 10.1093/jxb/eraa215"
"   Xie WJ, 2011, PLANT SOIL ENVIRON, V57, P128, DOI 10.17221/225/2010-PSE"
"   Xie WJ, 2011, AFR J AGR RES, V6, P1469"
"   Xin JL, 2024, ENVIRON POLLUT, V348, DOI 10.1016/j.envpol.2024.123890"
"   Xu SJ, 2018, PLANT PHYSIOL BIOCH, V123, P75, DOI 10.1016/j.plaphy.2017.11.023"
"   Yadav A, 2023, Copper and molybdenum management in crop production, P137"
"   Yadav A, 2023, Agrochemicals, V2, P220, DOI [10.3390/agrochemicals2020016, 10.3390/agrochemicals2020016, DOI 10.3390/AGROCHEMICALS2020016, 10.3390/AGROCHEMICALS2020016]"
"   Yan A, 2020, FRONT PLANT SCI, V11, DOI 10.3389/fpls.2020.00359"
"   Yan GC, 2024, FRONT PLANT SCI, V15, DOI 10.3389/fpls.2024.1393458"
"   Yao J, 2018, CHEM SCI, V9, P2927, DOI 10.1039/c7sc05476a"
"   Yasin Muhammad Umair, 2024, Chemosphere, V354, P141672, DOI [10.1016/j.chemosphere.2024.141672, 10.1016/j.chemosphere.2024.141672]"
"   Yavas Ilkay, 2022, Turkish Journal of Agriculture - Food Science and Technology, V10, P1468, DOI [10.24925/turjaf.v10i8.1468-1481.5220, 10.24925/turjaf.v10i8.1468-1481.5220]"
"   Yi L, 2022, J Geosci Environ Protect, DOI [10.4236/gep.2022.109006, DOI 10.4236/GEP.2022.109006]"
"   Younas A., 2024, Revolutionizing agriculture: A comprehensive exploration of agri-nanotechnology, P17, DOI DOI 10.1007/978-3-031-76000-6_2"
"   Yuan HY, 2021, J CLEAN PROD, V318, DOI 10.1016/j.jclepro.2021.128589"
"   Zadeh RR, 2019, J PLANT INTERACT, V14, P474, DOI 10.1080/17429145.2019.1626922"
"   Zaidi S, 2024, PLANT PHYSIOL BIOCH, V213, DOI 10.1016/j.plaphy.2024.108811"
"   Zhai XW, 2013, SYST BIOL REPROD MED, V59, P251, DOI 10.3109/19396368.2013.791347"
"   Zhang S, 2019, Mechanism of migration and transformation of nano selenium and mitigates cadmium stress in plants"
"   Zhao LJ, 2020, J AGR FOOD CHEM, V68, P1935, DOI 10.1021/acs.jafc.9b06615"
"   Zhou JR, 2024, POL J ENVIRON STUD, V33, P5991, DOI 10.15244/pjoes/183161"
"   Zhou PF, 2023, ENVIRON SCI TECHNOL, V57, P7547, DOI 10.1021/acs.est.3c00959"
"   Zhou TN, 2021, WATER AIR SOIL POLL, V232, DOI 10.1007/s11270-021-05227-8"
"   Zhou X, 2025, FRONT PLANT SCI, V15, DOI 10.3389/fpls.2024.1509047"
"   Zhu JP, 2024, HORTICULTURAE, V10, DOI 10.3390/horticulturae10070751"
"   Zhu YX, 2023, CRIT REV ENV SCI TEC, V53, P2071, DOI 10.1080/10643389.2023.2210985"
"   Zhu YY, 2022, ECOTOX ENVIRON SAFE, V240, DOI 10.1016/j.ecoenv.2022.113681"
"   Zulfiqar HF, 2024, 2. Nanoparticles in agriculture: enhancing crop resilience and productivity against abiotic stresses, DOI [10.5772/intechopen.114843, DOI 10.5772/INTECHOPEN.114843]"
NR 300
TC 1
Z9 1
U1 13
U2 13
PU SPRINGER
PI DORDRECHT
"PA VAN GODEWIJCKSTRAAT 30, 3311 GZ DORDRECHT, NETHERLANDS"
SN 1388-0764

EI 1572-896X
J9 J NANOPART RES
JI J. Nanopart. Res.
PD DEC
PY 2025
VL 27
IS 12
AR 324
DI 10.1007/s11051-025-06520-x
PG 56
"WC Chemistry, Multidisciplinary; Nanoscience & Nanotechnology; Materials"
"   Science, Multidisciplinary"
WE Science Citation Index Expanded (SCI-EXPANDED)
SC Chemistry; Science & Technology - Other Topics; Materials Science
GA R2773
UT WOS:001635430300003
DA 2026-04-06
ER

PT J
"AU Chen, MJ"
"   Li, YF"
"   Jiang, XR"
"   Zhao, DR"
"   Liu, XF"
"   Zhou, JL"
"   He, ZF"
"   Zheng, CL"
"   Pan, XL"
"AF Chen, Minjie"
"   Li, Yafei"
"   Jiang, Xiaoru"
"   Zhao, Dingran"
"   Liu, Xuefeng"
"   Zhou, Jianlin"
"   He, Zhanfei"
"   Zheng, Chunli"
"   Pan, Xiangliang"
TI Study on soil physical structure after the bioremediation of Pb
   pollution using microbial-induced carbonate precipitation methodology
SO JOURNAL OF HAZARDOUS MATERIALS
LA English
DT Article
DE Pollution; Pb; Soil; Bioremediation; Soil aggregation
ID SIZE FRACTIONS; CONTAMINATED SOIL; HEAVY-METALS; IMPROVEMENT; STABILITY;
   BACTERIA; UREASE
"AB Soil structure is an important index to evaluate soil quality; however, previous researchers have only paid attention to the effect and economic benefits of soil heavy metal remediation. In this study, microbial-induced carbonate precipitation (MICP) technology was used to remediate soil Pb pollution, and its effect on soil structure was studied by sieving and X-ray computed tomography techniques. The results showed that the leaching amount of heavy metals in soil decreased by 76.34% after remediation. Interestingly, due to the addition of organic matter and microorganisms, the soil particle size changed from microaggregates to large aggregates, and the large soil particle size (diameter > 2 mm) increased significantly by 71.43%. The soil porosity increased by 73.78%, which enhanced the soil permeability and increased the soil hydraulic conductivity. Therefore, MICP bioremediation not only remediated soil heavy metal pollution but also promoted the soil aggregation structure, which has important significance for soil remediation and improvement."
"C1 [Chen, Minjie; Li, Yafei; Jiang, Xiaoru; Zhao, Dingran; Liu, Xuefeng; Zhou, Jianlin; Zheng, Chunli] Inner Mongolia Univ Sci & Technol, Sch Energy & Environm, Baotou 014010, Peoples R China."
"   [Chen, Minjie; Li, Yafei; Jiang, Xiaoru; Zhao, Dingran; Liu, Xuefeng; Zhou, Jianlin; Zheng, Chunli] Inner Mongolia Engn Res Ctr Evaluat & Restorat Mi, Baotou 014010, Peoples R China."
"   [Zheng, Chunli] Key Lab Integrated Exploitat Bayan Obo Multimetal, Baotou 014010, Peoples R China."
"   [He, Zhanfei; Pan, Xiangliang] Zhejiang Univ Technol, Coll Environm, Key Lab Microbial Technol Ind Pollut Control, Hangzhou 310014, Peoples R China."
C3 Inner Mongolia University of Science & Technology; Zhejiang University
   of Technology
"RP Zheng, CL (corresponding author), 7 Arding St, Baotou 014010, Inner Mongolia, Peoples R China.; Pan, XL (corresponding author), 18 Chaowang St, Hangzhou 014010, Zhejiang, Peoples R China."
EM zhengchunli1979@163.com; xiangliangpan@163.com
"RI he, zhanfei/H-6902-2015; Chen, minjie/KXC-7011-2024; Liu,"
"   xuefeng/IUP-1483-2023; pan, xiangliang/I-1466-2014"
FU National key research and development program [2018YFC1802904]; National
"   Science Foundation of China [41561094, 41867061]; Inner Mongolia Science"
   & Technology Plan; Special Fund for the Transformation of Scientific
   and?Technological Achievements in Inner Mongolia [2019CG062]
FX We sincerely appreciate five anonymous reviewers for their critical and
   valuable comments to help improve this manuscript. This work was
   financially supported by National key research and development program
"   (2018YFC1802904) , the National Science Foundation of China (41561094"
"   and 41867061) , Inner Mongolia Science & Technology Plan, and the"
   Special Fund for the Transformation of Scientific and?Technological
   Achievements in Inner Mongolia (2019CG062) .
"CR Achal V, 2009, J IND MICROBIOL BIOT, V36, P981, DOI 10.1007/s10295-009-0578-z"
"   Achal V, 2011, ECOL ENG, V37, P1601, DOI 10.1016/j.ecoleng.2011.06.008"
"   Ajmone-Marsan F, 2008, ENVIRON POLLUT, V152, P73, DOI 10.1016/j.envpol.2007.05.020"

"   Almajed A, 2019, SCI REP-UK, V9, DOI 10.1038/s41598-018-38361-1"
"   Am??zketa E, 1999, J SUSTAIN AGR, V14, P83, DOI 10.1300/J064v14n02_08"
"   AMEZKETA E, 1995, SOIL SCI, V159, P287, DOI 10.1097/00010694-199505000-00001"
"   [????????? AN Shaoshan], 2006, [???????€????, Journal of Soil Science], V37, P45"
"   Ayangbenro AS, 2017, INT J ENV RES PUB HE, V14, DOI 10.3390/ijerph14010094"
"   BOQUET E, 1973, NATURE, V246, P527, DOI 10.1038/246527a0"
"   Chai YJ, 2019, CATENA, V173, P312, DOI 10.1016/j.catena.2018.10.026"
"   Chen S, 2019, POL J ENVIRON STUD, V28, P3635, DOI 10.15244/pjoes/99205"
"   Chu J, 2013, GEOTECHNIQUE, V63, P871, DOI [10.1680/geot.SIP13.P007, 10.1680/geot.SIP13.P.007]"
"   Chung H, 2020, SCI TOTAL ENVIRON, V740, DOI 10.1016/j.scitotenv.2020.140194"
"   Dejong JT, 2013, GEOTECHNIQUE, V63, P287, DOI 10.1680/geot.SIP13.P.017"
"   Delelegn YT, 2017, SCI REP-UK, V7, DOI 10.1038/s41598-017-14128-y"
"   DEXTER AR, 1988, SOIL TILL RES, V11, P199, DOI 10.1016/0167-1987(88)90002-5"
"   Ekperusi OA, 2015, 3 BIOTECH, V5, P957, DOI 10.1007/s13205-015-0298-1"
"   ELLIOTT ET, 1986, SOIL SCI SOC AM J, V50, P627, DOI 10.2136/sssaj1986.03615995005000030017x"
"   Epelde L, 2014, APPL SOIL ECOL, V75, P1, DOI 10.1016/j.apsoil.2013.10.003"
"   Fan GQ, 2017, SCI REP-UK, V7, DOI 10.1038/s41598-017-01360-9"
"   Feng MH, 2005, CHEMOSPHERE, V59, P939, DOI 10.1016/j.chemosphere.2004.11.056"
"   Fern??ndez R, 2010, SOIL TILL RES, V109, P103, DOI 10.1016/j.still.2010.05.002"
"   Fujita Y, 2008, ENVIRON SCI TECHNOL, V42, P3025, DOI 10.1021/es702643g"
"   Haghi AH, 2019, SCI REP-UK, V9, DOI 10.1038/s41598-019-51263-0"
"   Harms H, 2011, NAT REV MICROBIOL, V9, P177, DOI 10.1038/nrmicro2519"
"   Hashem A, 2019, SAUDI J BIOL SCI, V26, P1291, DOI 10.1016/j.sjbs.2019.05.004"
"   Huang B, 2017, ENVIRON SCI POLLUT R, V24, P23939, DOI 10.1007/s11356-017-0012-4"
"   Ilg K, 2004, GEODERMA, V123, P153, DOI 10.1016/j.geoderma.2004.02.006"
"   Kumari D, 2016, ADV APPL MICROBIOL, V94, P79, DOI 10.1016/bs.aambs.2015.12.002"
"   Li M, 2013, INT BIODETER BIODEGR, V76, P81, DOI 10.1016/j.ibiod.2012.06.016"
"   Liu B, 2020, ENG GEOL, V264, DOI 10.1016/j.enggeo.2019.105389"
"   Liu L, 2016, HUM ECOL RISK ASSESS, V22, P424, DOI 10.1080/10807039.2015.1078226"
"   Luo Y., 2011, ACT ECOL SIN, V31"
"   Murillo-Amador B, 2015, FRONT PLANT SCI, V6, DOI 10.3389/fpls.2015.00549"
"   Oduor CO, 2018, BMC ECOL, V18, DOI 10.1186/s12898-018-0202-z"
"   Peng DH, 2020, J HAZARD MATER, V400, DOI 10.1016/j.jhazmat.2020.123116"
"   Quinton JN, 2007, ENVIRON SCI TECHNOL, V41, P3495, DOI 10.1021/es062147h"
"   QUIRK JP, 1955, J SOIL SCI, V6, P163, DOI 10.1111/j.1365-2389.1955.tb00841.x"
"   Repas TS, 2017, PLOS ONE, V12, DOI 10.1371/journal.pone.0186704"
"   Robles P, 2018, INT J MOL SCI, V19, DOI 10.3390/ijms19082388"
"   Sajjadi SA, 2015, SOLID EARTH, V6, P311, DOI 10.5194/se-6-311-2015"
"   Seifan M, 2016, APPL MICROBIOL BIOT, V100, P2591, DOI 10.1007/s00253-016-7316-z"
"   Sherameti I., 2010, SOIL HEAVY METALS, P455, DOI [10.4028/www.scientific.net/AMR.455-456.1285, DOI 10.4028/WWW.SCIENTIFIC.NET/AMR.455-456.1285]"
"   Shiau YJ, 2017, SCI REP-UK, V7, DOI 10.1038/srep40561"
"   Tak HI, 2013, REV ENVIRON CONTAM T, V223, P33, DOI 10.1007/978-1-4614-5577-6_2"
"   Tang FK, 2016, SOLID EARTH, V7, P141, DOI 10.5194/se-7-141-2016"
"   Tian J, 2015, APPL SOIL ECOL, V90, P1, DOI 10.1016/j.apsoil.2015.01.014"
"   von L??tzow M, 2007, SOIL BIOL BIOCHEM, V39, P2183, DOI 10.1016/j.soilbio.2007.03.007"
"   Wang Q, 2017, SCI REP-UK, V7, DOI 10.1038/s41598-017-12809-2"
"   Whiffin VS, 2007, GEOMICROBIOL J, V24, P417, DOI 10.1080/01490450701436505"
"   Yang C, 2019, GEODERMA, V337, P444, DOI 10.1016/j.geoderma.2018.10.002"
"   Yu X, 2016, EUR J SOIL SCI, V67, P109, DOI 10.1111/ejss.12305"
"   Zhang JM, 2019, SCI REP-UK, V9, DOI 10.1038/s41598-019-48406-8"
"   Zhang WX, 2019, ECOL EVOL, V9, P7586, DOI 10.1002/ece3.5308"
"   Zhou D, 2017, INT J ENV RES PUB HE, V14, DOI 10.3390/ijerph14070681"
"   Zhu SC, 2020, WATER AIR SOIL POLL, V231, DOI 10.1007/s11270-020-04815-4"
"   Zhu XL, 2018, AMB EXPRESS, V8, DOI 10.1186/s13568-018-0590-7"
"   Zu YQ, 2004, ENVIRON INT, V30, P567, DOI 10.1016/j.envint.2003.10.012"
NR 58
TC 106
Z9 119
U1 20
U2 422
PU ELSEVIER
PI AMSTERDAM
"PA RADARWEG 29, 1043 NX AMSTERDAM, NETHERLANDS"
SN 0304-3894
EI 1873-3336
J9 J HAZARD MATER
JI J. Hazard. Mater.
PD JUN 5
PY 2021
VL 411
AR 125103
DI 10.1016/j.jhazmat.2021.125103
EA JAN 2021
PG 11
"WC Engineering, Environmental; Environmental Sciences"
WE Science Citation Index Expanded (SCI-EXPANDED)
SC Engineering; Environmental Sciences & Ecology
GA RK1PO
UT WOS:000638075400007
PM 33858089
DA 2026-04-06
ER

PT J
"AU Tarun, A"
"   Lakshmi, TDS"
"   Jha, AK"
"AF Tarun, Abhishek"
"   Lakshmi, Tallapudi Divya Sri"
"   Jha, Arvind Kumar"
TI Immobilization of Heavy Metal Contaminants and Their Effect on the
   Strength Behavior of MICP-Treated Sand
SO JOURNAL OF ENVIRONMENTAL ENGINEERING
LA English
DT Article
DE Bioremediation; Calcite; Metal contamination; Microbially induced
   calcite precipitation (MICP); Strength
ID CARBONATE PRECIPITATION; OXIDE NANOPARTICLES; BACTERIAL-ACTIVITY;
   ACTIVATED CARBON; GANGA RIVER; SEDIMENTS; REMOVAL; WATER;
   BIOREMEDIATION; ZINC
"AB Microbially Induced Calcite Precipitation (MICP) is a bio-mediated process used for contaminant remediation through calcite formation. While previous research has focused on heavy metal immobilization at low concentrations, the underlying interaction mechanisms remain unclear. This study investigates (i) the effect of heavy metal concentrations on urease-producing Bacillus sp.; (ii) bio-precipitation mechanisms and removal efficiencies; (iii) the role of bio-augmentation in metal immobilization; and (iv) the impact of heavy metals on MICP-treated soil strength. Experiments examined Bacillus sp.'s zeta potential and growth behavior in metal-contaminated environments. Results revealed that the presence of heavy metals influenced the zeta potential of the bacteria. The toxicity of heavy metals varies depending on the specific metal salt. In this study, zinc was found to significantly inhibit bacterial growth, whereas lead had only a minimal effect. Microanalysis confirmed the formation of carbonate precipitates after treating the heavy metal-contaminated bacterial solution. Additionally, bio-augmentation in contaminated sand was evaluated to assess heavy metal leaching and the impact of MICP on soil strength. After 18 days, more than 90% of heavy metals were immobilized, but strength improvement declined by up to 50% in samples containing highly toxic metals. The decrease in strength was linked to a reduction in density, as observed through the ultrasonic pulse velocity test. These findings offer valuable insights into optimizing MICP for effective heavy metal remediation. This work provides insights for advancing the MICP method for effective heavy metal removal."
"   The soil naturally contains trace amounts of heavy metals essential for the local ecosystem's flora and fauna. However, human activities have significantly elevated these concentrations to hazardous levels, adversely impacting both humans and animals. Conventional remediation methods often demand high energy or produce toxic byproducts, posing further ecological risks. This study explores the eco-friendly microbially induced calcite precipitation (MICP) process as a sustainable solution for heavy metal immobilization. It investigates the process of remediation of heavy metal-contaminated soils using ureolytic bacteria while addressing the adverse effects on soil microbiomes. The effect on the bacterial activity and their electrostatic properties affecting their interaction with contaminated soil have also been discussed. Dealing with such contamination in field scenarios is unavoidable. This study also examines the effect of MICP treatment on the soil mechanical properties, highlighting the adverse effects and changes that arise with it. It emphasizes the practical considerations for applying this method in real-world field conditions."
"C1 [Tarun, Abhishek; Lakshmi, Tallapudi Divya Sri; Jha, Arvind Kumar] Indian Inst Technol Patna, Patna 801106, Bihar, India."
C3 Indian Institute of Technology (IIT) - Patna; Indian Institute of
   Technology System (IIT System)
"RP Jha, AK (corresponding author), Indian Inst Technol Patna, Patna 801106, Bihar, India."
EM abhishek_2021ce04@iitp.ac.in; tallapudi_2001ce60@iitp.ac.in;
   jhaarvind@iitp.ac.in
"OI Tallapudi, Divya/0009-0002-7100-4888"
"CR Abbas Q, 2020, ENVIRON INT, V138, DOI 10.1016/j.envint.2020.105646"
"   Achal V, 2013, CHEMOSPHERE, V93, P1352, DOI 10.1016/j.chemosphere.2013.08.008"
"   Achal V, 2011, ECOL ENG, V37, P554, DOI 10.1016/j.ecoleng.2010.11.009"
"   Al Qabany A, 2012, J GEOTECH GEOENVIRON, V138, P992, DOI 10.1061/(ASCE)GT.1943-5606.0000666"
"   Anbu P, 2016, SPRINGERPLUS, V5, DOI 10.1186/s40064-016-1869-2"
"   Bai H, 2021, INT BIODETER BIODEGR, V165, DOI 10.1016/j.ibiod.2021.105311"
"   Bhattacharyya A, 2014, PHYSIOL REV, V94, P329, DOI 10.1152/physrev.00040.2012"
"   BIS, 1980, Methods of test for soils, part 3: Determination of specific gravity"
"   BIS (Bureau Indian Standards), 1970, IS 1498"
"   BIS (Bureau Indian Standards), 1893, Criteria for earthquake resistant design of structures, Part 1: General provisions and buildings"
"   BIS (Bureau Indian Standards), 1986, Methods of test for soils, part 17: Laboratory determination of permeability"
"   BIS (Bureau Indian Standards), 1985, Methods of test for soils, part 4: Grain size analysis"
"   Cheung KH, 2007, INT BIODETER BIODEGR, V59, P8, DOI 10.1016/j.ibiod.2006.05.002"
"   Chhaya, 2024, Environmental Nanotechnology, Monitoring and Management, V21, DOI [10.1016/j.enmm.2024.100933, 10.1016/j.enmm.2024.100933]"
"   Chhaya, 2020, Microbial Bioremediation spsampsps Biodegradation, P425, DOI DOI 10.1007/978-981-15-1812-616"
"   Choi SG, 2020, CONSTR BUILD MATER, V246, DOI 10.1016/j.conbuildmat.2020.118415"
"   Chrysikopoulos CV, 2017, TRANSPORT POROUS MED, V119, P181, DOI 10.1007/s11242-017-0879-z"
"   Cui LM, 2015, CHEM ENG J, V281, P1, DOI 10.1016/j.cej.2015.06.043"
"   Duarte-Nass C, 2020, J ENVIRON MANAGE, V256, DOI 10.1016/j.jenvman.2019.109938"
"   Fang LY, 2021, SCI TOTAL ENVIRON, V787, DOI 10.1016/j.scitotenv.2021.147627"
"   Fujita Y, 2010, ENVIRON SCI TECHNOL, V44, P7652, DOI 10.1021/es101752p"
"   Hao HJ, 2022, J ENVIRON ENG, V148, DOI 10.1061/(ASCE)EE.1943-7870.0002022"
"   Harkes MP, 2010, ECOL ENG, V36, P112, DOI 10.1016/j.ecoleng.2009.01.004"
"   He ZF, 2023, CHEMOSPHERE, V311, DOI 10.1016/j.chemosphere.2022.136981"
"   Heera Rajandas, 2015, BMC Res Notes, V8, P754, DOI [10.1186/s13104-015-1726-3, 10.1186/s13104-015-1726-3]"
"   Jalilvand N, 2020, J SOIL SCI PLANT NUT, V20, P206, DOI 10.1007/s42729-019-00121-z"
"   Jha AK, 2022, J HAZARD TOXIC RADIO, V26, DOI 10.1061/(ASCE)HZ.2153-5515.0000686"

"   Jiang NJ, 2019, SCI TOTAL ENVIRON, V672, P722, DOI 10.1016/j.scitotenv.2019.03.294"
"   Jogawat A, 2021, PHYSIOL PLANTARUM, V173, P259, DOI 10.1111/ppl.13370"
"   Kim Y, 2021, FRONT MICROBIOL, V12, DOI 10.3389/fmicb.2021.646748"
"   Kumar A, 2023, CHEMOSPHERE, V318, DOI 10.1016/j.chemosphere.2023.137894"
"   Kumar RN, 2013, ENVIRON MONIT ASSESS, V185, P359, DOI 10.1007/s10661-012-2558-4"
"   Lage OM, 2012, ARCH MICROBIOL, V194, P847, DOI 10.1007/s00203-012-0818-x"
"   Laspidou CS, 2002, WATER RES, V36, P1983, DOI 10.1016/S0043-1354(01)00414-6"
"   Li A, 2013, J ENVIRON SCI, V25, P2281, DOI 10.1016/S1001-0742(12)60293-9"
"   Li LY, 2001, J ENVIRON ENG, V127, P420, DOI 10.1061/(ASCE)0733-9372(2001)127:5(420)"
"   Li MM, 2021, ADV MATER, V33, DOI 10.1002/adma.202104829"
"   Liang LY, 2018, ACS SYNTH BIOL, V7, P2497, DOI 10.1021/acssynbio.8b00194"
"   Liu Cheng-Han Michael, 2024, Chemosphere, V347, P140539, DOI [10.1016/j.chemosphere.2023.140539, 10.1016/j.chemosphere.2023.140539]"
"   Matta G, 2023, INT J RIVER BASIN MA, V21, P759, DOI 10.1080/15715124.2022.2098756"
"   Mita L, 2015, J HAZARD MATER, V291, P129, DOI 10.1016/j.jhazmat.2015.02.072"
"   Morales-Barrera L, 2022, PROCESSES, V10, DOI 10.3390/pr10050886"
"   Mwandira W, 2017, ECOL ENG, V109, P57, DOI 10.1016/j.ecoleng.2017.09.011"
"   Nosrati SA, 2024, J ENVIRON ENG, V150, DOI 10.1061/JOEEDU.EEENG-7232"
"   Omoregie AI, 2025, MICROORGANISMS, V13, DOI 10.3390/microorganisms13010174"
"   Pandey J, 2010, TROP ECOL, V51, P365"
"   Paul Dipak, 2017, Annals of Agrarian Science, V15, P278, DOI 10.1016/j.aasci.2017.04.001"
"   Peng Dinghua, 2024, Chemosphere, V366, P143441, DOI [10.1016/j.chemosphere.2024.143441, 10.1016/j.chemosphere.2024.143441]"
"   Qiao SY, 2021, CHEMOSPHERE, V274, DOI 10.1016/j.chemosphere.2021.129661"
"   Rajasekar A, 2021, PLOS ONE, V16, DOI 10.1371/journal.pone.0254676"
"   Ramaye Y, 2021, MATERIALS, V14, DOI 10.3390/ma14020290"
"   Raychoudhury T, 2015, WATER SCI TECH-W SUP, V15, P990, DOI 10.2166/ws.2015.057"
"   Rzetala MA, 2015, SOIL SEDIMENT CONTAM, V24, P49, DOI 10.1080/15320383.2014.911721"
"   Salgin S, 2012, INT J ELECTROCHEM SC, V7, P12404"
"   Shan B, 2021, ENVIRON SCI POLLUT R, V28, P30486, DOI 10.1007/s11356-021-14045-8"
"   Sharma M, 2021, INT J GEOTECH ENG, V15, P220, DOI 10.1080/19386362.2019.1691322"
"   Sharma M, 2022, ENVIRON SCI POLLUT R, V29, P51827, DOI 10.1007/s11356-022-19551-x"
"   Silver S, 1996, ANNU REV MICROBIOL, V50, P753, DOI 10.1146/annurev.micro.50.1.753"
"   Singh VK, 2005, ENVIRON MONIT ASSESS, V105, P43, DOI 10.1007/s10661-005-2816-9"
"   Song MZ, 2022, CHEMOSPHERE, V290, DOI 10.1016/j.chemosphere.2021.133229"
"   Stocks-Fischer S, 1999, SOIL BIOL BIOCHEM, V31, P1563, DOI 10.1016/S0038-0717(99)00082-6"
"   Su ZM, 2023, INT BIODETER BIODEGR, V178, DOI 10.1016/j.ibiod.2023.105563"
"   Sun PD, 2015, SCI TOTAL ENVIRON, V505, P189, DOI 10.1016/j.scitotenv.2014.09.095"
"   Taharia M, 2024, ECOTOX ENVIRON SAFE, V271, DOI 10.1016/j.ecoenv.2024.115990"
"   Tang YH, 2021, CONSTR BUILD MATER, V306, DOI 10.1016/j.conbuildmat.2021.124950"
"   Tarun A, 2025, J HAZARD TOXIC RADIO, V29, DOI 10.1061/JHTRBP.HZENG-1432"
"   Tarun A, 2024, J ENVIRON ENG, V150, DOI 10.1061/JOEEDU.EEENG-7477"
"   Tchounwou Paul B, 2012, Exp Suppl, V101, P133, DOI 10.1007/978-3-7643-8340-4_6"
"   Van Paassen L.A., 2009, BIOGROUT GROUND IMPR"
"   Vazquez-S??nchez AY, 2023, MOLECULES, V28, DOI 10.3390/molecules28176387"
"   Wallace AR, 2022, J ENVIRON ENG, V148, DOI [10.1061/(ASCE)EE.1943-7870.0002000, 10.1061/(asce)ee.1943-7870.0002000]"
"   Wang Yuxuan, 2024, Chemosphere, V362, P142762, DOI [10.1016/j.chemosphere.2024.142762, 10.1016/j.chemosphere.2024.142762]"
"   Wang ZY, 2021, MAR GEORESOUR GEOTEC, V39, P1459, DOI 10.1080/1064119X.2020.1850949"
"   Yang QQ, 2018, SCI TOTAL ENVIRON, V642, P690, DOI 10.1016/j.scitotenv.2018.06.068"
"   Yin J, 2023, SUSTAINABILITY-BASEL, V15, DOI 10.3390/su15010434"
"   Yuan LJ, 2024, BUILDINGS-BASEL, V14, DOI 10.3390/buildings14071977"
"   Zhang WC, 2023, FRONT MICROBIOL, V14, DOI 10.3389/fmicb.2023.1116970"
"   Zhang Y, 2023, BIOGEOTECHNICS, V1, DOI [10.1016/j.bgtech.2023.100003, 10.1016/j.bgtech.2023.100003]"
NR 78
TC 1
Z9 2
U1 12
U2 15
PU ASCE-AMER SOC CIVIL ENGINEERS
PI RESTON
"PA 1801 ALEXANDER BELL DR, RESTON, VA 20191-4400 USA"
SN 0733-9372
EI 1943-7870
J9 J ENVIRON ENG
JI J. Environ. Eng.-ASCE
PD SEP 1
PY 2025
VL 151
IS 9
AR 04025052
DI 10.1061/JOEEDU.EEENG-8148
PG 13
"WC Engineering, Environmental; Engineering, Civil; Environmental Sciences"
WE Science Citation Index Expanded (SCI-EXPANDED)
SC Engineering; Environmental Sciences & Ecology
GA 4YK5N
UT WOS:001529636900009
DA 2026-04-06
ER

PT J
"AU Zhang, HY"

"   Yuan, XZ"
"   Xiong, T"
"   Wang, H"
"   Jiang, LB"
"AF Zhang, Hanyan"
"   Yuan, Xingzhong"
"   Xiong, Ting"
"   Wang, Hou"
"   Jiang, Longbo"
TI Bioremediation of co-contaminated soil with heavy metals and pesticides:
"   Influence factors, mechanisms and evaluation methods"
SO CHEMICAL ENGINEERING JOURNAL
LA English
DT Article
DE Co-contaminated soil; Bioremediation; Heavy metal; Pesticide; Evaluation
   method
ID POLYCYCLIC AROMATIC-HYDROCARBONS; IN-SITU REMEDIATION; WEIGHT
   ORGANIC-ACIDS; ZEA-MAYS L.; HYPERACCUMULATOR SEDUM-ALFREDII; EARTHWORM
   EISENIA-FOETIDA; RADIAL OXYGEN LOSS; RISK-ASSESSMENT;
   MICROBIAL-DEGRADATION; ORGANOCHLORINE PESTICIDES
"AB Soil co-contaminated with heavy metals and pesticides has caused large global environmental issues. The interaction of heavy metals and pesticides make the contamination situation more complicated. It is crucial to remediate the co-contaminated soil by heavy metals and pesticides. Bioremediation has the advantages of high efficiency (especially for large, low contamination areas), low cost, easy available, harmless for the ecosystem and high public acceptance, which has been widely developed in remediation of combined pollution of heavy metals and pesticides. This paper reviews the bioremediation applications of combined contaminated soil with heavy metals and pesticides. Abiotic and biotic factors (e.g. pH, temperature, bioavailability of pollutants, the interactions between pollutants, biological competitiveness and biological state) can all affect the bioremediation of heavy metals and pesticides. Meanwhile, the present review summarizes the bioremediation mechanisms of heavy metals and pesticides by microorganisms and plants. The effectiveness evaluation methods of soil remediation are also reviewed. Based on above, this review proposes the future investigations required for this field."
"C1 [Zhang, Hanyan; Yuan, Xingzhong; Xiong, Ting; Wang, Hou; Jiang, Longbo] Hunan Univ, Coll Environm Sci & Engn, Changsha 410082, Peoples R China."
"   [Zhang, Hanyan; Yuan, Xingzhong; Xiong, Ting; Wang, Hou; Jiang, Longbo] Hunan Univ, Key Lab Environm Biol & Pollut Control, Minist Educ, Changsha 410082, Peoples R China."
"   [Wang, Hou] Nanyang Technol Univ, Sch Chem & Biomed Engn, Singapore 637459, Singapore."
C3 Hunan University; Hunan University; Nanyang Technological University
"RP Yuan, XZ; Xiong, T (corresponding author), Hunan Univ, Coll Environm Sci & Engn, Changsha 410082, Peoples R China."
EM yxz@hnu.edu.cn; xiongt_hnu@163.com
"RI Jiang, Longbo/AER-3617-2022; Xingzhong, Yuan/E-2109-2016; Xiong,"
"   T./LSL-0703-2024; Wang, Hou/E-5264-2013"
"OI Jiang, Longbo/0000-0002-8913-179X; "
"FU National Natural Science Foundation of China [51739004, 21776066,"
"   51521006, 71431006]; Fundamental Research Funds for the Central"
   Universities [531118010394]
FX The authors gratefully acknowledge the financial support provided by the
"   National Natural Science Foundation of China (51739004, 21776066,"
"   51521006, 71431006), the Fundamental Research Funds for the Central"
   Universities (531118010394).
"CR ABBASZADEHDAHAJ.P, 2017, PLANT MICROBE INTERA, P187, DOI DOI 10.1007/978-981-10-2854-0_9"
"   Abhilash PC, 2011, ENVIRON EXP BOT, V74, P127, DOI 10.1016/j.envexpbot.2011.05.009"
"   Abhilash PC, 2010, J SOIL SEDIMENT, V10, P272, DOI 10.1007/s11368-009-0085-x"
"   Adamkovicova Maria, 2014, ScientificWorldJournal, V2014, P632581, DOI 10.1155/2014/632581"
"   Adhikari T, 2004, J FOOD AGRIC ENVIRON, V2, P266"
"   Aislabie J, 2005, FEMS MICROBIOL ECOL, V52, P279, DOI 10.1016/j.femsec.2004.11.012"
"   Aksu Z, 2005, PROCESS BIOCHEM, V40, P997, DOI 10.1016/j.procbio.2004.04.008"
"   Al-Turki A. I., 2009, Research Journal of Environmental Toxicology, V3, P1, DOI 10.3923/rjet.2009.1.8"
"   Ali H, 2013, CHEMOSPHERE, V91, P869, DOI 10.1016/j.chemosphere.2013.01.075"
"   Almeida CMR, 2008, MAR ENVIRON RES, V66, P311, DOI 10.1016/j.marenvres.2008.04.005"
"   Alvarez A, 2017, CHEMOSPHERE, V166, P41, DOI 10.1016/j.chemosphere.2016.09.070"
"   Amoakwah E, 2014, B ENVIRON CONTAM TOX, V93, P187, DOI 10.1007/s00128-014-1319-1"
"   Rodr??guez-Li??bana JA, 2014, SCI TOTAL ENVIRON, V497, P561, DOI 10.1016/j.scitotenv.2014.08.010"
"   Armstrong WD., 1979, ADV BOT RES, V7, P225, DOI DOI 10.1016/S0065-2296(08)60089-0"
"   Arora K, 2016, INT J PHYTOREMEDIAT, V18, P704, DOI 10.1080/15226514.2015.1131232"
"   Arslan M, 2017, ENVIRON SCI POLLUT R, V24, P4322, DOI 10.1007/s11356-015-4935-3"
"   Asha Latha P, 2013, ENV POLLUT INT J BAS, P2277"
"   Ashmore MH, 2008, ENVIRON INT, V34, P1192, DOI 10.1016/j.envint.2008.03.012"
"   Ashraf S, 2019, ECOTOX ENVIRON SAFE, V174, P714, DOI 10.1016/j.ecoenv.2019.02.068"
"   ATLAS RM, 1981, MICROBIOL REV, V45, P180, DOI 10.1128/MMBR.45.1.180-209.1981"
"   Ayangbenro AS, 2017, INT J ENV RES PUB HE, V14, DOI 10.3390/ijerph14010094"
"   Azad MA, 2014, CHINESE SCI BULL, V59, P703, DOI 10.1007/s11434-013-0058-8"
"   Bal??zs HE, 2020, APPL SOIL ECOL, V150, DOI 10.1016/j.apsoil.2019.103467"
"   Bandowe BAM, 2014, ENVIRON INT, V65, P135, DOI 10.1016/j.envint.2013.12.018"
"   Batty LC, 2008, INT J PHYTOREMEDIAT, V10, P236, DOI 10.1080/15226510801997549"
"   BELFROID A, 1995, ECOTOX ENVIRON SAFE, V31, P185, DOI 10.1006/eesa.1995.1061"
"   Benckiser G, 1997, Fauna in soil ecosystems: recycling processes, nutrient fluxes, and agricultural production"
"   Benimeli CS, 2003, BIORESOURCE TECHNOL, V89, P133, DOI 10.1016/S0960-8524(03)00061-0"
"   Bicalho STT, 2012, J ENVIRON SCI HEAL B, V47, P505, DOI 10.1080/03601234.2012.665659"
"   Bolan NS, 2011, ADV AGRON, V112, P145, DOI 10.1016/B978-0-12-385538-1.00004-4"
"   Boughattas I, 2019, J SOIL SEDIMENT, V19, P296, DOI 10.1007/s11368-018-2038-8"
"   Pereira ACC, 2013, SOIL SEDIMENT CONTAM, V22, P376, DOI 10.1080/15320383.2013.733446"

"   Pereira RC, 2008, ENVIRON POLLUT, V155, P350, DOI 10.1016/j.envpol.2007.11.009"
"   Ceci A, 2019, APPL MICROBIOL BIOT, V103, P53, DOI 10.1007/s00253-018-9451-1"
"   Ceci A, 2018, APPL MICROBIOL BIOT, V102, P1019, DOI 10.1007/s00253-017-8614-9"
"   C??r??monie H, 2006, FEMS MICROBIOL LETT, V257, P243, DOI 10.1111/j.1574-6968.2006.00188.x"
"   Chen C, 2015, ENVIRON INT, V77, P95, DOI 10.1016/j.envint.2015.01.014"
"   Chen C, 2014, CHEMOSPHERE, V112, P393, DOI 10.1016/j.chemosphere.2014.04.070"
"   Chen F, 2019, CHEMOSPHERE, V236, DOI 10.1016/j.chemosphere.2019.124406"
"   Chen YT, 2017, CURR OPIN PLANT BIOL, V39, P66, DOI 10.1016/j.pbi.2017.06.004"
"   Chen YX, 2004, PLANT SOIL, V261, P127, DOI 10.1023/B:PLSO.0000035581.92021.f2"
"   Cheng H, 2014, PLANT SOIL, V375, P137, DOI 10.1007/s11104-013-1945-0"
"   Cheng SP, 2007, INT J PHYTOREMEDIAT, V9, P453, DOI 10.1080/15226510701709531"
"   Cheraghi M, 2011, BIOL TRACE ELEM RES, V144, P1133, DOI 10.1007/s12011-009-8359-0"
"   Chigbo C, 2013, CHEMOSPHERE, V90, P2542, DOI 10.1016/j.chemosphere.2012.11.007"
"   Chung MK, 2007, SEP PURIF TECHNOL, V54, P355, DOI 10.1016/j.seppur.2006.10.008"
"   Clausen GB, 2002, FEMS MICROBIOL ECOL, V41, P221, DOI 10.1111/j.1574-6941.2002.tb00983.x"
"   Comte S, 2008, J HAZARD MATER, V151, P185, DOI 10.1016/j.jhazmat.2007.05.070"
"   Contreras-Ramos SM, 2008, SOIL BIOL BIOCHEM, V40, P1954, DOI 10.1016/j.soilbio.2008.04.009"
"   Cui XY, 2013, ENVIRON POLLUT, V172, P223, DOI 10.1016/j.envpol.2012.09.013"
"   Cycon M, 2017, CHEMOSPHERE, V172, P52, DOI 10.1016/j.chemosphere.2016.12.129"
"   Aparicio JD, 2019, ECOTOX ENVIRON SAFE, V181, P255, DOI 10.1016/j.ecoenv.2019.06.019"
"   Aparicio JD, 2018, J ENVIRON CHEM ENG, V6, P2310, DOI 10.1016/j.jece.2018.03.030"
"   Aparicio JD, 2018, J HAZARD MATER, V342, P408, DOI 10.1016/j.jhazmat.2017.08.041"
"   Das AJ, 2017, INT J ENVIRON SCI TE, V14, P1343, DOI 10.1007/s13762-016-1183-0"
"   Davis TA, 2003, WATER RES, V37, P4311, DOI 10.1016/S0043-1354(03)00293-8"
"   Deng ZJ, 2017, CHEMOSPHERE, V168, P1100, DOI 10.1016/j.chemosphere.2016.10.097"
"   DIBBLE JT, 1979, APPL ENVIRON MICROB, V37, P729, DOI 10.1128/AEM.37.4.729-739.1979"
"   Diepens NJ, 2014, CRIT REV ENV SCI TEC, V44, P255, DOI 10.1080/01496395.2012.718945"
"   Dixit R, 2015, SUSTAINABILITY-BASEL, V7, P2189, DOI 10.3390/su7022189"
"   Dong XL, 2014, J HAZARD MATER, V267, P62, DOI 10.1016/j.jhazmat.2013.12.027"
"   Donnelly C, 2013, NATURE, V499, P154, DOI 10.1038/499154d"
"   Doucette W, 2013, ENVIRON SCI TECHNOL, V47, P5813, DOI 10.1021/es304115c"
"   Dub?? JS, 2002, CAN GEOTECH J, V39, P279, DOI 10.1139/T01-113"
"   Emenike CU, 2018, ENVIRON REV, V26, P156, DOI 10.1139/er-2017-0045"
"   Erakhrumen, 2007, ED RES REV, V2, P151, DOI DOI 10.5897/ERR.9000255"
"   Estevez E, 2016, SCI TOTAL ENVIRON, V551, P186, DOI 10.1016/j.scitotenv.2016.01.177"
"   Falis M, 2014, ACTA VET BRNO, V83, P95, DOI 10.2754/avb201483020095"
"   Fang H, 2012, J ENVIRON SCI-CHINA, V24, P1036, DOI 10.1016/S1001-0742(11)60895-4"
"   Fang LC, 2010, BIORESOURCE TECHNOL, V101, P5774, DOI 10.1016/j.biortech.2010.02.075"
"   Fayiga AO, 2019, ENVIRON CHEM, V16, P81, DOI 10.1071/EN18196"
"   Felix H, 1997, Z PFLANZ BODENKUNDE, V160, P525, DOI 10.1002/jpln.19971600414"
"   Feng JF, 2018, J HAZARD MATER, V345, P97, DOI 10.1016/j.jhazmat.2017.11.013"
"   Feng NX, 2017, SCI TOTAL ENVIRON, V583, P352, DOI 10.1016/j.scitotenv.2017.01.075"
"   Fenner K, 2013, SCIENCE, V341, P752, DOI 10.1126/science.1236281"
"   Fliessbach A, 2004, BIOL FERT SOILS, V40, P268, DOI 10.1007/s00374-004-0776-9"
"   Fomina M, 2014, BIORESOURCE TECHNOL, V160, P3, DOI 10.1016/j.biortech.2013.12.102"
"   Freitas EV, 2013, CHEMOSPHERE, V92, P213, DOI 10.1016/j.chemosphere.2013.01.103"
"   Gadd GM, 2009, J CHEM TECHNOL BIOT, V84, P13, DOI 10.1002/jctb.1999"
"   Gallego SM, 2012, ENVIRON EXP BOT, V83, P33, DOI 10.1016/j.envexpbot.2012.04.006"
"   Garc??a-G??mez C, 2019, SCI TOTAL ENVIRON, V688, P199, DOI 10.1016/j.scitotenv.2019.06.083"
"   Garrison AW, 2000, ENVIRON SCI TECHNOL, V34, P1663, DOI 10.1021/es990265h"
"   Gauthier PT, 2015, ENVIRON SCI TECHNOL, V49, P11780, DOI 10.1021/acs.est.5b03233"
"   Gerhardt KE, 2009, PLANT SCI, V176, P20, DOI 10.1016/j.plantsci.2008.09.014"
"   Ghosh M., 2005, Applied Ecology and Environmental Research, V3, P1"
"   Gjorgieva D, 2011, ARCH ENVIRON CON TOX, V60, P233, DOI 10.1007/s00244-010-9543-0"
"   Glick BR, 2010, BIOTECHNOL ADV, V28, P367, DOI 10.1016/j.biotechadv.2010.02.001"
"   Gokel GW, 2001, COORDIN CHEM REV, V222, P127, DOI 10.1016/S0010-8545(01)00380-0"
"   GOLAB Z, 1995, WATER AIR SOIL POLL, V82, P713, DOI 10.1007/BF00479421"
"   Goldblum DK, 2006, J HAZARD MATER, V136, P406, DOI 10.1016/j.jhazmat.2005.11.047"
"   Gong JL, 2009, J HAZARD MATER, V164, P1517, DOI 10.1016/j.jhazmat.2008.09.072"
"   Gonz??lez-Pleiter M, 2013, WATER RES, V47, P2050, DOI 10.1016/j.watres.2013.01.020"
"   Govantes F, 2009, MICROB BIOTECHNOL, V2, P178, DOI 10.1111/j.1751-7915.2008.00073.x"
"   Guo S., 2018, Twenty Years of Research and Development on Soil Pollution and Remediation in China, P703, DOI [10.1007/978-981-10-6029-8_44, DOI 10.1007/978-981-10-6029-8_44]"
"   Guti??rrez-Gin??s MJ, 2014, J ENVIRON MANAGE, V143, P197, DOI 10.1016/j.jenvman.2014.04.028"
"   Haimi J, 2000, ENVIRON POLLUT, V107, P233, DOI 10.1016/S0269-7491(99)00142-6"
"   Hansda A, 2016, WORLD J MICROB BIOT, V32, DOI 10.1007/s11274-016-2117-1"
"   Hao YQ, 2018, ENVIRON POLLUT, V242, P728, DOI 10.1016/j.envpol.2018.07.029"
"   He W, 2020, CRIT REV BIOTECHNOL, V40, P31, DOI 10.1080/07388551.2019.1675582"
"   Hechmi N, 2014, ENVIRON SCI POLLUT R, V21, P1304, DOI 10.1007/s11356-013-1997-y"
"   Hechmi N, 2013, INT J PHYTOREMEDIAT, V15, P703, DOI 10.1080/15226514.2012.723067"
"   Heys KA, 2016, RSC ADV, V6, P47844, DOI 10.1039/c6ra05406d"
"   Hickman ZA, 2008, ENVIRON INT, V34, P1072, DOI 10.1016/j.envint.2008.02.013"
"   Hinsinger P, 2005, NEW PHYTOL, V168, P293, DOI 10.1111/j.1469-8137.2005.01512.x"
"   Hong HB, 2000, WATER RES, V34, P349, DOI 10.1016/S0043-1354(99)00100-1"
"   Hong YW, 2015, ENVIRON SCI POLLUT R, V22, P7071, DOI 10.1007/s11356-014-3912-6"
"   Houben D, 2013, CHEMOSPHERE, V92, P1450, DOI 10.1016/j.chemosphere.2013.03.055"
"   Hrynkiewicz K, 2018, ENVIRON REV, V26, P316, DOI 10.1139/er-2018-0023"
"   Huang H.G., 2012, PROCESSES MECH AGRON"
"   Huang HL, 2006, J AGR FOOD CHEM, V54, P9377, DOI 10.1021/jf061699g"
"   Huang HG, 2011, BIORESOURCE TECHNOL, V102, P11034, DOI 10.1016/j.biortech.2011.09.067"
"   Huang W, 2013, COLLOID SURFACE B, V105, P113, DOI 10.1016/j.colsurfb.2012.12.040"
"   Hussain I, 2018, SCI TOTAL ENVIRON, V628-629, P1582, DOI 10.1016/j.scitotenv.2018.02.037"
"   Hussain I, 2018, ENVIRON EXP BOT, V147, P202, DOI 10.1016/j.envexpbot.2017.12.016"
"   Hussain S, 2009, CRIT REV ENV SCI TEC, V39, P843, DOI 10.1080/10643380801910090"

" Hussain S, 2009, ADV AGRON, V102, P159, DOI 10.1016/S0065-2113(09)01005-0"
" Husson O, 2013, PLANT SOIL, V362, P389, DOI 10.1007/s11104-012-1429-7"
" Iqbal M, 2016, CHEMOSPHERE, V144, P785, DOI 10.1016/j.chemosphere.2015.09.048"
" Jabeen R, 2009, BOT REV, V75, P339, DOI 10.1007/s12229-009-9036-x"
" Jan AT, 2014, CRIT REV ENV SCI TEC, V44, P519, DOI 10.1080/10643389.2012.728811"
" Janicki T, 2018, J HAZARD MATER, V360, P661, DOI 10.1016/j.jhazmat.2018.08.047"
" Janicki T, 2016, BIORESOURCE TECHNOL, V200, P223, DOI 10.1016/j.biortech.2015.10.034"
" Perez-Vazquez FJ, 2015, ENVIRON MONIT ASSESS, V187, DOI 10.1007/s10661-014-4119-5"
" Jeelani N, 2018, INT J PHYTOREMEDIAT, V20, P773, DOI 10.1080/15226514.2018.1425666"
" Jia ZL, 2017, J SOIL SEDIMENT, V17, P315, DOI 10.1007/s11368-016-1562-7"
" Jiang R, 2018, SCI TOTAL ENVIRON, V626, P1047, DOI 10.1016/j.scitotenv.2018.01.135"
" Jobby R, 2018, CHEMOSPHERE, V207, P255, DOI 10.1016/j.chemosphere.2018.05.050"
" Johnsen K, 2001, BIOL FERT SOILS, V33, P443, DOI 10.1007/s003740100351"
" Jones KD, 1999, ENVIRON SCI TECHNOL, V33, P580, DOI 10.1021/es9803207"
" Kachur AV, 1998, FREE RADICAL RES, V28, P259, DOI 10.3109/10715769809069278"
" Kalia A, 2011, ARCH AGRON SOIL SCI, V57, P569, DOI 10.1080/03650341003787582"
" Karaca A, 2010, SOIL BIOL, V19, P237, DOI 10.1007/978-3-642-02436-8_11"
" Kataoka R, 2013, APPL MICROBIOL BIOT, V97, P3285, DOI 10.1007/s00253-013-4774-4"
" Khalid S, 2017, J GEOCHEM EXPLOR, V182, P247, DOI 10.1016/j.gexplo.2016.11.021"
" Khan S, 2013, CHEMOSPHERE, V90, P1317, DOI 10.1016/j.chemosphere.2012.09.045"
" Kirkham MB, 2006, GEODERMA, V137, P19, DOI 10.1016/j.geoderma.2006.08.024"
" Kumar S, 2018, PEDOSPHERE, V28, P190, DOI 10.1016/S1002-0160(18)60017-7"
" Kushwaha A, 2018, ECOTOX ENVIRON SAFE, V147, P1035, DOI 10.1016/j.ecoenv.2017.09.049"
" Laemmli CM, 2000, J BACTERIOL, V182, P4165, DOI 10.1128/JB.182.15.4165-4172.2000"
" LEAHY JG, 1990, MICROBIOL REV, V54, P305, DOI 10.1128/MMBR.54.3.305-315.1990"
" Lei X., 2014, CHIN AGR SCI B"
" Li H, 2017, ENVIRON POLLUT, V224, P622, DOI 10.1016/j.envpol.2017.01.087"
" Li M, 2013, INT BIODETER BIODEGR, V76, P81, DOI 10.1016/j.ibiod.2012.06.016"
" Li XX, 2017, GEOMICROBIOL J, V34, P346, DOI 10.1080/01490451.2016.1193571"
" Li XZ, 2019, SCI TOTAL ENVIRON, V646, P893, DOI 10.1016/j.scitotenv.2018.07.380"
" Liang J, 2015, J ENVIRON SCI HEAL A, V50, P100, DOI 10.1080/10934529.2015.964636"
" Liang XJ, 2016, ENVIRON MICROBIOL, V18, P219, DOI 10.1111/1462-2920.13003"
" Liang XF, 2014, GEODERMA, V235, P9, DOI 10.1016/j.geoderma.2014.06.029"
" Limmer M, 2016, ENVIRON SCI TECHNOL, V50, P6632, DOI 10.1021/acs.est.5b04113"
" Lin Q, 2008, J HAZARD MATER, V150, P515, DOI 10.1016/j.jhazmat.2007.04.132"
" Lin Q, 2006, SCI TOTAL ENVIRON, V368, P814, DOI 10.1016/j.scitotenv.2006.03.024"
" Lin WT, 2016, J ENVIRON SCI-CHINA, V45, P94, DOI 10.1016/j.jes.2015.12.010"
" Lin Z, 2012, J ENVIRON MONITOR, V14, P1551, DOI 10.1039/c2em30160a"
" Liu HY, 2015, J HAZARD MATER, V294, P121, DOI 10.1016/j.jhazmat.2015.04.004"
" Liu LW, 2018, SCI TOTAL ENVIRON, V633, P206, DOI 10.1016/j.scitotenv.2018.03.161"
" Liu SH, 2017, BIORESOURCE TECHNOL, V224, P25, DOI 10.1016/j.biortech.2016.11.095"
" Liu T, 2019, SCI TOTAL ENVIRON, V651, P2247, DOI 10.1016/j.scitotenv.2018.10.087"
" Loureiro S, 2009, ENVIRON POLLUT, V157, P625, DOI 10.1016/j.envpol.2008.08.010"
" Lu M, 2014, ENVIRON SCI TECHNOL, V48, P1158, DOI 10.1021/es403337t"
" Lu M, 2013, J HAZARD MATER, V262, P311, DOI 10.1016/j.jhazmat.2013.08.067"
" Luo L, 2006, CHEMOSPHERE, V63, P1273, DOI 10.1016/j.chemosphere.2005.10.013"
" Ma SS, 2015, RSC ADV, V5, P40000, DOI 10.1039/c5ra04075b"
" Ma Y, 2011, BIOTECHNOL ADV, V29, P248, DOI 10.1016/j.biotechadv.2010.12.001"
" Ma Y, 2016, FRONT PLANT SCI, V7, DOI 10.3389/fpls.2016.00918"
" Mahar A, 2016, ECOTOX ENVIRON SAFE, V126, P111, DOI 10.1016/j.ecoenv.2015.12.023"
" Malik A, 2004, ENVIRON INT, V30, P261, DOI 10.1016/j.envint.2003.08.001"
" Manoj SR, 2020, J ENVIRON MANAGE, V254, DOI 10.1016/j.jenvman.2019.109779"
" Marr LC, 2006, ENVIRON SCI TECHNOL, V40, P5560, DOI 10.1021/es060087+"
" Marziali L, 2017, SCI TOTAL ENVIRON, V593, P809, DOI 10.1016/j.scitotenv.2017.03.219"
" Mastretta Chiara, 2006, Biotechnol Genet Eng Rev, V23, P175"
" Mattina MI, 2003, ENVIRON POLLUT, V124, P375, DOI 10.1016/S0269-7491(03)00060-5"
" Megharaj M, 2011, ENVIRON INT, V37, P1362, DOI 10.1016/j.envint.2011.06.003"
" MEHLICH A, 1984, COMMUN SOIL SCI PLAN, V15, P1409, DOI 10.1080/00103628409367568"
" Mena E, 2016, CHEM ENG J, V299, P30, DOI 10.1016/j.cej.2016.04.049"
" Meng LJ, 2016, J HAZARD MATER, V305, P219, DOI 10.1016/j.jhazmat.2015.11.048"
" Mercado-Borrayo BM, 2015, INT J PHYTOREMEDIAT, V17, P701, DOI 10.1080/15226514.2014.964841"
" Montiel-Rozas MM, 2016, ENVIRON POLLUT, V216, P273, DOI 10.1016/j.envpol.2016.05.080"
" Morillo E, 2017, SCI TOTAL ENVIRON, V586, P576, DOI 10.1016/j.scitotenv.2017.02.020"
" Mulligan CN, 2005, ENVIRON POLLUT, V133, P183, DOI 10.1016/j.envpol.2004.06.009"
" Nagpal Varima, 2006, Indian Journal of Biotechnology, V5, P400"
" Ndubueze Ezinne. U., 2018, POTENTIAL 5 PLANT SP"
" Niti C, 2013, RES J CHEM ENVIRON, V17, P88"
" Nykiel-Szymanska J, 2018, ECOTOX ENVIRON SAFE, V162, P1, DOI 10.1016/j.ecoenv.2018.06.060"
" Odukkathil G, 2013, REV ENVIRON SCI BIO, V12, P421, DOI 10.1007/s11157-013-9320-4"
" Ojuederie OB, 2017, INT J ENV RES PUB HE, V14, DOI 10.3390/ijerph14121504"
" Olaniran AO, 2013, INT J MOL SCI, V14, P10197, DOI 10.3390/ijms140510197"
" Olaniran AO, 2009, J ENVIRON SCI, V21, P661, DOI 10.1016/S1001-0742(08)62322-0"
" Oner E.T., 2015, MICROBIAL PRODUCTION, P35"
" ??zer A, 2003, J HAZARD MATER, V100, P219, DOI 10.1016/S0304-3894(03)00109-2"
" Padmavathiamma PK, 2007, WATER AIR SOIL POLL, V184, P105, DOI 10.1007/s11270-007-9401-5"
" Pan LW, 2012, GROUND WATER MONIT R, V32, P96, DOI 10.1111/j.1745-6592.2011.01377.x"
" Parveen S, 2005, J ENVIRON SCI, V17, P631"
" Passatore L, 2014, J HAZARD MATER, V278, P189, DOI 10.1016/j.jhazmat.2014.05.051"
" Pepper IL, 2002, ENVIRON HEALTH PERSP, V110, P943, DOI 10.1289/ehp.02110s6943"
" Pino N, 2011, INT BIODETER BIODEGR, V65, P827, DOI 10.1016/j.ibiod.2011.06.001"
" Polti MA, 2014, INT BIODETER BIODEGR, V88, P48, DOI 10.1016/j.ibiod.2013.12.004"
" Prigione V, 2009, BIORESOURCE TECHNOL, V100, P2770, DOI 10.1016/j.biortech.2009.01.002"
" Priya M, 2014, ELSEV INSIGHT, P521, DOI 10.1016/B978-0-12-800021-2.00023-6"
" Purnomo AS, 2011, INT BIODETER BIODEGR, V65, P921, DOI 10.1016/j.ibiod.2011.07.011"

"   Qin F, 2004, CHEMOSPHERE, V57, P253, DOI 10.1016/j.chemosphere.2004.06.010"
"   Rajtor M, 2016, CHEMOSPHERE, V162, P105, DOI 10.1016/j.chemosphere.2016.07.071"
"   Rani A, 2013, ANN MICROBIOL, V63, P923, DOI 10.1007/s13213-012-0545-1"
"   Rasheed T, 2018, SCI TOTAL ENVIRON, V615, P476, DOI 10.1016/j.scitotenv.2017.09.126"
"   Regoli F, 2005, ENVIRON TOXICOL CHEM, V24, P1475, DOI 10.1897/04-514R.1"
"   Rodriguez-Iruretagoiena A, 2016, MAR POLLUT BULL, V111, P95, DOI 10.1016/j.marpolbul.2016.07.025"
"   S??nchez V, 2020, SEP PURIF TECHNOL, V233, DOI 10.1016/j.seppur.2019.116044"
"   Sandrin ToddR., 2007, Environmental Bioremediation Technologies, P1, DOI DOI 10.1007/978-3-540-34793-4_1"
"   Sandrin TR, 2003, ENVIRON HEALTH PERSP, V111, P1093, DOI 10.1289/ehp.5840"
"   Sarma H, 2019, ENVIRON TECHNOL INNO, V13, P318, DOI 10.1016/j.eti.2018.12.004"
"   Schreck E, 2012, SCI TOTAL ENVIRON, V427, P253, DOI 10.1016/j.scitotenv.2012.03.051"
"   SCHROLL R, 1994, CHEMOSPHERE, V28, P297, DOI 10.1016/0045-6535(94)90126-0"
"   Scott C, 2008, INDIAN J MICROBIOL, V48, P65, DOI 10.1007/s12088-008-0007-4"
"   Sheoran V, 2016, PEDOSPHERE, V26, P148, DOI 10.1016/S1002-0160(15)60032-7"
"   Sidhu GK, 2019, CRIT REV ENV SCI TEC, V49, P1135, DOI 10.1080/10643389.2019.1565554"
"   Sola MZS, 2017, J BASIC MICROB, V57, P1037, DOI 10.1002/jobm.201700324"
"   Sol?? MZS, 2019, CHEMOSPHERE, V222, P679, DOI 10.1016/j.chemosphere.2019.01.197"
"   Singh AK, 1999, BIOSENS BIOELECTRON, V14, P703, DOI 10.1016/S0956-5663(99)00044-5"
"   Singh N, 2017, FRONT CHEM, V5, DOI 10.3389/fchem.2017.00070"
"   Singh T, 2017, INT J PHYTOREMEDIAT, V19, P834, DOI 10.1080/15226514.2017.1290579"
"   Slaba M, 2009, SCI TOTAL ENVIRON, V407, P4127, DOI 10.1016/j.scitotenv.2009.03.023"
"   Song B, 2019, CRIT REV ENV SCI TEC, V49, P791, DOI 10.1080/10643389.2018.1558891"
"   Song B, 2017, ENVIRON INT, V105, P43, DOI 10.1016/j.envint.2017.05.001"
"   Song B, 2017, CHEMOSPHERE, V172, P449, DOI 10.1016/j.chemosphere.2017.01.032"
"   Suanon F, 2020, CHEM ENG J, V385, DOI 10.1016/j.cej.2019.123934"
"   Subashchandrabose SR, 2015, ENVIRON SCI POLLUT R, V22, P8876, DOI 10.1007/s11356-013-1679-9"
"   Svenningsen NB, 2017, BMC MICROBIOL, V17, DOI 10.1186/s12866-017-1119-y"
"   Tariq SR, 2016, SCIENTIFICA, V2016, DOI 10.1155/2016/7575239"
"   Tejada M, 2016, ECOL ENG, V86, P39, DOI 10.1016/j.ecoleng.2015.09.058"
"   Teng Y, 2017, ENVIRON SCI POLLUT R, V24, P9538, DOI 10.1007/s11356-017-8640-2"
"   Teng Y, 2011, J HAZARD MATER, V186, P1271, DOI 10.1016/j.jhazmat.2010.11.126"
"   Thompson IP, 2005, ENVIRON MICROBIOL, V7, P909, DOI 10.1111/j.1462-2920.2005.00804.x"
"   Tiwary M, 2016, INT BIODETER BIODEGR, V108, P42, DOI 10.1016/j.ibiod.2015.11.025"
"   TRAPP S, 1994, ENVIRON TOXICOL CHEM, V13, P413, DOI [10.1897/1552-8618(1994)13[413:MFUOXI]2.0.CO;2, 10.1002/etc.5620130308]"
"   Tyagi M, 2011, BIODEGRADATION, V22, P231, DOI 10.1007/s10532-010-9394-4"
"   Uwizeyimana H, 2017, ENVIRON TOXICOL PHAR, V55, P20, DOI 10.1016/j.etap.2017.08.001"
"   van Herwijnen R, 2003, FEMS MICROBIOL ECOL, V46, P105, DOI [10.1016/S0168-6496(03)00202-2, 10.1016/s0168-6496(03)00202-2]"
"   Vangronsveld J, 2009, ENVIRON SCI POLLUT R, V16, P765, DOI 10.1007/s11356-009-0213-6"
"   Verma JP, 2014, REV ENVIRON SCI BIO, V13, P429, DOI 10.1007/s11157-014-9341-7"
"   Verma S, 2019, ENVIRON TECHNOL INNO, V14, DOI 10.1016/j.eti.2019.100369"
"   Villa RD, 2010, J HAZARD MATER, V174, P770, DOI 10.1016/j.jhazmat.2009.09.118"
"   Wang FL, 2014, J SOIL SEDIMENT, V14, P451, DOI 10.1007/s11368-013-0827-7"
"   Wang H, 2013, APPL SURF SCI, V279, P432, DOI 10.1016/j.apsusc.2013.04.133"
"   Wang J, 2018, ENVIRON POLLUT, V238, P462, DOI 10.1016/j.envpol.2018.02.082"
"   Wang K., 2012, Enhancement and Mechanism of Phytoremediation for Cd and Pahs cocontaminated Soils"
"   Wang LW, 2020, CRIT REV ENV SCI TEC, V50, P2724, DOI 10.1080/10643389.2019.1705724"
"   Wang XX, 2008, BIOCHEM BIOPH RES CO, V365, P453, DOI 10.1016/j.bbrc.2007.10.193"
"   Wang XD, 2018, ENVIRON POLLUT, V242, P136, DOI 10.1016/j.envpol.2018.06.070"
"   Wang Y, 2018, J SOIL SEDIMENT, V18, P2136, DOI 10.1007/s11368-017-1762-9"
"   Wang YB, 2017, B ENVIRON CONTAM TOX, V98, P465, DOI [10.1007/s00128-017-2042-5, 10.10]"
"   Wcislo E, 2016, ENVIRON INT, V94, P436, DOI 10.1016/j.envint.2016.05.028"
"   WEISSENFELS WD, 1992, APPL MICROBIOL BIOT, V36, P689, DOI 10.1007/BF00183251"
"   Wenzel WW, 2009, PLANT SOIL, V321, P385, DOI 10.1007/s11104-008-9686-1"
"   Willscher S, 2013, HYDROMETALLURGY, V131, P46, DOI 10.1016/j.hydromet.2012.08.012"
"   Wu D, 2014, SCI TOTAL ENVIRON, V481, P209, DOI 10.1016/j.scitotenv.2014.02.041"
"   Wu LH, 2003, CHEMOSPHERE, V50, P819, DOI 10.1016/S0045-6535(02)00225-4"
"   Xiao WD, 2013, ENVIRON SCI POLLUT R, V20, P380, DOI 10.1007/s11356-012-0902-4"
"   Xiong T, 2020, ECOTOX ENVIRON SAFE, V192, DOI 10.1016/j.ecoenv.2020.110326"
"   Xiong T, 2020, J HAZARD MATER, V389, DOI 10.1016/j.jhazmat.2020.122154"
"   Xiong T, 2018, J ENVIRON MANAGE, V205, P99, DOI 10.1016/j.jenvman.2017.09.067"
"   Yan DYS, 2013, ENVIRON POLLUT, V178, P15, DOI 10.1016/j.envpol.2013.02.030"
"   Yan GX, 2016, CLEAN-SOIL AIR WATER, V44, P984, DOI 10.1002/clen.201500571"
"   Yan GY, 2003, WATER RES, V37, P4486, DOI 10.1016/S0043-1354(03)00409-3"
"   Yang C, 2016, J BASIC MICROB, V56, P820, DOI 10.1002/jobm.201500559"
"   Yang JX, 2017, ECOL ENG, V107, P56, DOI 10.1016/j.ecoleng.2017.06.052"
"   Yang JX, 2014, PLANT SOIL, V374, P815, DOI 10.1007/s11104-013-1922-7"
"   Yang P, 2018, INT J ENV RES PUB HE, V15, DOI 10.3390/ijerph15071330"
"   Yang Q., 2004, PHYTOREMEDIATION SOI"
"   Yang T, 2015, TRAC-TREND ANAL CHEM, V66, P90, DOI 10.1016/j.trac.2014.11.016"
"   Yang XE, 2004, J PLANT NUTR, V27, P1963, DOI [10.1081/PLN-200030082, 10.1081/LPLA-200030082]"
"   Ye M, 2014, J ENVIRON MANAGE, V141, P161, DOI 10.1016/j.jenvman.2014.03.025"
"   Ye SJ, 2017, CRIT REV ENV SCI TEC, V47, P1528, DOI 10.1080/10643389.2017.1386951"
"   Yuan XZ, 2018, ENVIRON SCI POLLUT R, V25, P32562, DOI 10.1007/s11356-018-3140-6"
"   Zhang C, 2019, ENVIRON SCI POLLUT R, V26, P26844, DOI 10.1007/s11356-019-05811-w"
"   Zhang D, 2012, ENVIRON POLLUT, V160, P178, DOI 10.1016/j.envpol.2011.08.053"
"   Zhang KJ, 2019, CHEMOSPHERE, V224, P272, DOI 10.1016/j.chemosphere.2019.02.140"
"   Zhang MY, 2019, GEODERMA, V348, P115, DOI 10.1016/j.geoderma.2019.04.031"
"   Zhang XK, 2013, ENVIRON SCI POLLUT R, V20, P8472, DOI 10.1007/s11356-013-1659-0"
"   Zhang XY, 2020, INT J PHYTOREMEDIAT, V22, P313, DOI 10.1080/15226514.2019.1663481"
"   Zhang ZH, 2012, GEODERMA, V175, P1, DOI 10.1016/j.geoderma.2012.01.020"
"   Zhang ZH, 2011, J HAZARD MATER, V189, P119, DOI 10.1016/j.jhazmat.2011.02.007"

"   Zhou D.M., 2000, Soil Environ. Sci., P143, DOI [10.3969/j.issn.1674-5906.2000.02.016, DOI 10.3969/J.ISSN.1674-5906.2000.02.016]"
"   Zhu TY, 2015, B ENVIRON CONTAM TOX, V94, P129, DOI 10.1007/s00128-014-1412-5"
"   Zhu YG, 2009, CURR OPIN BIOTECH, V20, P220, DOI 10.1016/j.copbio.2009.02.011"
"   Zhu ZQ, 2012, J HAZARD MATER, V235, P144, DOI 10.1016/j.jhazmat.2012.07.033"
"   Zouboulis AI, 2004, PROCESS BIOCHEM, V39, P909, DOI 10.1016/S0032-9592(03)00200-0"
NR 285
TC 330
Z9 392
U1 47
U2 884
PU ELSEVIER SCIENCE SA
PI LAUSANNE
"PA PO BOX 564, 1001 LAUSANNE, SWITZERLAND"
SN 1385-8947
EI 1873-3212
J9 CHEM ENG J
JI Chem. Eng. J.
PD OCT 15
PY 2020
VL 398
AR 125657
DI 10.1016/j.cej.2020.125657
PG 19
"WC Engineering, Environmental; Engineering, Chemical"
WE Science Citation Index Expanded (SCI-EXPANDED)
SC Engineering
GA OC1KU
UT WOS:000578922200013
DA 2026-04-06
ER

PT J
"AU El-Sharkawy, M"
"   Alotaibi, MO"
"   Li, J"
"   Du, DL"
"   Mahmoud, E"
"AF El-Sharkawy, Mahmoud"
"   Alotaibi, Modhi O."
"   Li, Jian"
"   Du, Daolin"
"   Mahmoud, Esawy"
TI Heavy Metal Pollution in Coastal Environments: Ecological Implications
   and Management Strategies: A Review
SO SUSTAINABILITY
LA English
DT Review
DE contamination sources; coastal ecosystems; ecological impact; future
   research; pollution control
ID IN-SITU REMEDIATION; REDOX CONDITIONS; RAINFALL-RUNOFF; SEDIMENTS
   IMPLICATIONS; MERCURY METHYLATION; ECOSYSTEM SERVICES; CONTAMINATED
   SOILS; SURFACE SEDIMENTS; WATER-QUALITY; BIOAVAILABILITY
"AB Heavy metals originating from industrial runoff, agricultural practices, urbanization, and natural geological processes persist in coastal sediments due to their low degradation rates and high stability. Their cycling is influenced by sediment dynamics, water circulation, and complex interactions with biological and chemical factors. Heavy metal pollution demonstrates serious risks to coastal biota, including fish, shellfish, algae, and marine mammals through mechanisms such as bioaccumulation and biomagnification. These processes lead to biodiversity loss, habitat degradation, and reduced ecosystem functionality. Current mitigation strategies for pollution control regulations and remediation techniques show promise but face challenges in implementation. Emerging technologies such as nanotechnology and bioremediation offer innovative solutions but require further validation. Knowledge gaps persist in understanding the long-term ecological impacts of heavy metal contamination and optimizing management strategies for diverse coastal ecosystems. Coastal ecosystems are vital for supporting biodiversity and providing essential ecosystem services, but they are increasingly threatened by heavy metal pollution-a pervasive environmental challenge that demands urgent attention. This review investigates the sources, characteristics, pathways, ecological impacts, and management strategies associated with heavy metal contamination in coastal environments. The review synthesizes findings from recent literature, employing a systematic approach to analyze natural and anthropogenic sources, contamination pathways, and the biogeochemical processes governing heavy metal cycling. Future research should focus on addressing these gaps through interdisciplinary approaches, integrating advanced modeling techniques, stakeholder engagement, and sustainable management practices. By prioritizing these efforts, we can safeguard coastal ecosystems and their essential services from the escalating threats of heavy metal pollution."
"C1 [El-Sharkawy, Mahmoud; Mahmoud, Esawy] Tanta Univ, Fac Agr, Soil & Water Dept, Tanta 31527, Egypt."
"   [Alotaibi, Modhi O.] Princess Nourah bint Abdulrahman Univ, Coll Sci, Dept Biol, POB 84428, Riyadh 11671, Saudi Arabia."
"   [Alotaibi, Modhi O.] Princess Nourah bint Abdulrahman Univ, Nat & Hlth Sci Res Ctr, Environm & Biomat Unit, POB 84428, Riyadh 11671, Saudi Arabia."
"   [Li, Jian; Du, Daolin] Jiangsu Univ, Sch Environm & Safety Engn, Sch Emergency Management, Zhenjiang 212013, Peoples R China."
C3 Egyptian Knowledge Bank (EKB); Tanta University; Princess Nourah bint
   Abdulrahman University; Princess Nourah bint Abdulrahman University;
   Jiangsu University

"RP El-Sharkawy, M (corresponding author), Tanta Univ, Fac Agr, Soil & Water Dept, Tanta 31527, Egypt.; Alotaibi, MO (corresponding author), Princess Nourah bint Abdulrahman Univ, Coll Sci, Dept Biol, POB 84428, Riyadh 11671, Saudi Arabia.; Alotaibi, MO (corresponding author), Princess Nourah bint Abdulrahman Univ, Nat & Hlth Sci Res Ctr, Environm & Biomat Unit, POB 84428, Riyadh 11671, Saudi Arabia."
EM mahmoud.elsharkawy@agr.tanta.edu.eg; mouotaebe@pnu.edu.sa;
jianli@ujs.edu.cn; daolindu@ujs.edu.cn; esawy.rezk@agr.tanta.edu.eg
"RI Du, Daolin/HHC-9359-2022; mahmoud, esawy/AFL-9839-2022; Alotaibi, Modhi"
" O/AAN-1939-2021; El-Sharkawy, Mahmoud/AER-2401-2022"
"OI mahmoud, esawy/0000-0002-1006-779X; Alotaibi, Modhi"
" O/0000-0002-6604-1652; El-Sharkawy, Mahmoud/0000-0003-2927-4657"
FU Princess Nourah bint Abdulrahman University Researchers Supporting
" Project; Princess Nourah bint Abdulrahman University, Riyadh, Saudi"
Arabia; [PNURSP2025R101]
FX This research was funded by Princess Nourah bint Abdulrahman University
" Researchers Supporting Project No. (PNURSP2025R101), Princess Nourah"
" bint Abdulrahman University, Riyadh, Saudi Arabia."
"CR Ahiablame LM, 2012, WATER AIR SOIL POLL, V223, P4253, DOI 10.1007/s11270-012-1189-2"
" Akcil A, 2015, J CLEAN PROD, V86, P24, DOI 10.1016/j.jclepro.2014.08.009"
" Alexander D.E., 1999, Environmental Geology: Encyclopedia of Earth Science, P43, DOI [10.1007/1-4020-4494-1_31, DOI 10.1007/1-4020-4494-1_31]"
" Alg??l F, 2020, SCI REP-UK, V10, DOI 10.1038/s41598-020-68833-2"
" Ali H, 2019, J CHEM-NY, V2019, DOI 10.1155/2019/6730305"
" Ali Q, 2023, Hybrid Advances, V4, P100091, DOI [10.1016/j.hybadv.2023.100091, 10.1016/j.hybadv.2023.100091, DOI 10.1016/J.HYBADV.2023.100091]"
" Ashayeri SY, 2023, MAR POLLUT BULL, V186, DOI 10.1016/j.marpolbul.2022.114389"
" Awa SH, 2020, WATER AIR SOIL POLL, V231, DOI 10.1007/s11270-020-4426-0"
" Ayangbenro AS, 2017, INT J ENV RES PUB HE, V14, DOI 10.3390/ijerph14010094"
" Bai S, 2021, ACTA GEOCHIM, V40, P961, DOI 10.1007/s11631-021-00492-0"
" Baker HM, 2009, ENVIRON MONIT ASSESS, V157, P319, DOI 10.1007/s10661-008-0537-6"
" Barkay T, 2021, ACS ENVIRON AU, V2, P77, DOI 10.1021/acsenvironau.1c00022"
" Benedet L, 2020, ENVIRON GEOCHEM HLTH, V42, P3281, DOI 10.1007/s10653-020-00572-9"
" Bennett EM, 2015, CURR OPIN ENV SUST, V14, P76, DOI 10.1016/j.cosust.2015.03.007"
" Bhat SA, 2022, CHEMOSPHERE, V288, DOI 10.1016/j.chemosphere.2021.132606"
" Bigham GN, 2017, INTEGR ENVIRON ASSES, V13, P249, DOI 10.1002/ieam.1822"
" Bjerregaard P, 2022, Handbook on the toxicology of metals, VFifth, P593"
" Borja A, 2010, ESTUAR COAST, V33, P1249, DOI 10.1007/s12237-010-9347-5"
" Briffa J, 2020, HELIYON, V6, DOI 10.1016/j.heliyon.2020.e04691"
" Caetano M, 2003, WATER AIR SOIL POLL, V143, P23, DOI 10.1023/A:1022877120813"
" Cardoso Simone Jaqueline, 2019, Acta Limnologica Brasiliensia, V31, pe201, DOI 10.1590/s2179-975x8918"
" Carolin CF, 2017, J ENVIRON CHEM ENG, V5, P2782, DOI 10.1016/j.jece.2017.05.029"
" Chahouri A, 2023, MAR POLLUT BULL, V192, DOI 10.1016/j.marpolbul.2023.115076"
" Chai MW, 2019, ENVIRON GEOCHEM HLTH, V41, P175, DOI 10.1007/s10653-018-0107-y"
" Charlatchka R, 2000, WATER AIR SOIL POLL, V118, P143, DOI 10.1023/A:1005195920876"
" Chavez-Rivera A D., 2023, TRANSLATIONAL SURG, P223, DOI [DOI 10.1016/B978-0-323-90300-4.00074-4, 10.1016/b978-0-323-90300-4.00074-4]"
" Chen MS, 2021, WATER RES, V190, DOI 10.1016/j.watres.2020.116711"
" Chen YM, 2016, CONT SHELF RES, V124, P125, DOI 10.1016/j.csr.2016.06.002"
" Chen Z, 2016, J HAZARD MATER, V311, P20, DOI 10.1016/j.jhazmat.2016.02.069"
" Chenier KA, 2024, SUSTAINABILITY-BASEL, V16, DOI 10.3390/su16114823"
" Choi Y, 2021, AQUAT TOXICOL, V238, DOI 10.1016/j.aquatox.2021.105883"
" Chowardhara Bhaben, 2024, Chemosphere, V359, P142178, DOI [10.1016/j.chemosphere.2024.142178, 10.1016/j.chemosphere.2024.142178]"
" Chris DI, 2023, ENVIRON SCI POLLUT R, V30, P80055, DOI 10.1007/s11356-023-27932-z"
" Clements WH, 2009, ENVIRON TOXICOL CHEM, V28, P1789, DOI 10.1897/09-140.1"
" Costanza R, 2014, GLOBAL ENVIRON CHANG, V26, P152, DOI 10.1016/j.gloenvcha.2014.04.002"
" Couture P., 2011, 9Field Studies on Metal Accumulation and Effects in Fish Homeostasis and Toxicology of Essential Metals, V473, P1546"
" Cristaldi A, 2017, ENVIRON TECHNOL INNO, V8, P309, DOI 10.1016/j.eti.2017.08.002"
" Cui BS, 2011, ENVIRON POLLUT, V159, P1297, DOI 10.1016/j.envpol.2011.01.024"
" Dale P, 2019, MAR POLICY, V103, P42, DOI 10.1016/j.marpol.2019.02.029"
" Danovaro R, 2023, COMMUN BIOL, V6, DOI 10.1038/s42003-023-05539-x"
" Dauvin JC, 2008, MAR POLLUT BULL, V57, P160, DOI 10.1016/j.marpolbul.2007.10.012"
" de Montaudouin X, 2023, MAR POLLUT BULL, V194, DOI 10.1016/j.marpolbul.2023.115278"
" Ding KB, 2018, J SOIL SEDIMENT, V18, P380, DOI 10.1007/s11368-016-1547-6"
" Dong JY, 2021, MAR POLLUT BULL, V167, DOI 10.1016/j.marpolbul.2021.112292"
" Dong YR, 2024, FRONT MICROBIOL, V15, DOI 10.3389/fmicb.2024.1352430"
" Du ZW, 2014, J HAZARD MATER, V274, P443, DOI 10.1016/j.jhazmat.2014.04.038"
" Duan PZ, 2022, J HAZARD MATER, V436, DOI 10.1016/j.jhazmat.2022.129175"
" Eisler R, 2004, REV ENVIRON CONTAM T, V183, P21"
" El Ouaty O, 2024, SUSTAINABILITY-BASEL, V16, DOI 10.3390/su16051921"
" Emenike EC, 2022, BIOL TRACE ELEM RES, V200, P4476, DOI 10.1007/s12011-021-03037-x"
" Emon FJ, 2023, TOXICS, V11, DOI 10.3390/toxics11060510"
" Fang SM, 2023, ENVIRON SCI POLLUT R, V30, P112445, DOI 10.1007/s11356-023-30239-8"
" Feng XH, 2007, ENVIRON POLLUT, V147, P366, DOI 10.1016/j.envpol.2006.05.028"
" Fujita S., 2023, Design of Materials and Technologies for Environmental Remediation, P507"
" Gavrilescu M, 2022, CURR OPIN BIOTECH, V74, DOI 10.1016/j.copbio.2021.10.024"
" Geng N, 2024, PROCESSES, V12, DOI 10.3390/pr12020311"
" Ghassemzadeh F., 2008, International Journal of Environmental Studies, V65, P587, DOI 10.1080/00207230802273387"
" Gill LW, 2017, SCI TOTAL ENVIRON, V601, P32, DOI 10.1016/j.scitotenv.2017.05.182"
" Gilmour CC, 2013, ENVIRON SCI TECHNOL, V47, P11810, DOI 10.1021/es403075t"
" Giovanni L, 2018, SCI TOTAL ENVIRON, V621, P85, DOI 10.1016/j.scitotenv.2017.11.229"
" Gnanasekaran S., 2023, Heavy Metals-Recent Advances"
" G??ng??r EBO, 2010, GEODERMA, V159, P131, DOI 10.1016/j.geoderma.2010.07.004"

"   Hamzeh M, 2014, WATER AIR SOIL POLL, V225, DOI 10.1007/s11270-014-1878-0"
"   Haynes RJ, 2022, ADV AGRON, V171, P191, DOI 10.1016/bs.agron.2021.08.004"
"   Holland KT, 2008, EARTH-SCI REV, V89, P116, DOI 10.1016/j.earscirev.2008.03.003"
"   Hossain MB, 2022, MAR POLLUT BULL, V185, DOI 10.1016/j.marpolbul.2022.114246"
"   Hou DY, 2023, NAT REV EARTH ENV, V4, P271, DOI 10.1038/s43017-023-00404-1"
"   Houhou J, 2009, SCI TOTAL ENVIRON, V407, P6052, DOI 10.1016/j.scitotenv.2009.08.019"
"   Hu Y, 2016, ENVIRON SCI POLLUT R, V23, P13964, DOI 10.1007/s11356-016-6589-1"
"   Ilyinskaya E, 2021, COMMUN EARTH ENVIRON, V2, DOI 10.1038/s43247-021-00146-2"
"   Ismanto A, 2024, ENVIRON QUAL MANAG, V33, P37, DOI 10.1002/tqem.21978"
"   Ives JT, 2019, FRESHWATER BIOL, V64, P1, DOI 10.1111/fwb.13203"
"   Jayachandran S, 2018, MAR POLLUT BULL, V133, P920, DOI 10.1016/j.marpolbul.2018.03.054"
"   Ji YH, 2024, LAND-BASEL, V13, DOI 10.3390/land13030384"
"   Jia ZZ, 2021, ENVIRON POLLUT, V287, DOI 10.1016/j.envpol.2021.117277"
"   Jiang H, 2012, ENVIRON MONIT ASSESS, V184, P6325, DOI 10.1007/s10661-011-2422-y"
"   Joziasse J., 2007, Sediment Risk Management and Communication, V3, P9"
"   Ju SH, 2011, J HAZARD MATER, V192, P554, DOI 10.1016/j.jhazmat.2011.05.049"
"   Karimian N, 2018, CHEMOSPHERE, V197, P803, DOI 10.1016/j.chemosphere.2018.01.096"
"   Khalef RN., 2022, Environmental Impact and Remediation of Heavy Metals"
"   Khan BN, 2023, HELIYON, V9, DOI 10.1016/j.heliyon.2023.e20968"
"   Kim Y, 2018, J HAZARD MATER, V353, P118, DOI 10.1016/j.jhazmat.2018.04.016"
"   Kleber M, 2021, NAT REV EARTH ENV, V2, P402, DOI 10.1038/s43017-021-00162-y"
"   Komy ZR, 2014, CHEMOSPHERE, V99, P117, DOI 10.1016/j.chemosphere.2013.10.048"
"   Kumar H., 2021, New Trends in Removal of Heavy Metals from Industrial Wastewater, P571, DOI [10.1016/B978-0-12-822965-1.00023-4, DOI 10.1016/B978-0-12-822965-1.00023-4]"
"   Kumar R., 2024, Biodiversity and Bioeconomy, P347, DOI DOI 10.1016/B978-0-323-95482-2.00016-X"
"   Kuppusamy S, 2016, REV ENVIRON CONTAM T, V236, P1, DOI 10.1007/978-3-319-20013-2_1"
"   L??zaro-Lobo A, 2021, WETLANDS, V41, DOI 10.1007/s13157-021-01462-1"
"   Lee G, 2019, MAR POLLUT BULL, V149, DOI 10.1016/j.marpolbul.2019.110509"
"   Lewis A, 2017, ENV CHEM SUSTAIN WOR, V8, P101, DOI 10.1007/978-3-319-58622-9_4"
"   Li CF, 2019, SOIL SEDIMENT CONTAM, V28, P380, DOI 10.1080/15320383.2019.1592108"
"   Li M, 2016, MAR POLLUT BULL, V103, P227, DOI 10.1016/j.marpolbul.2015.12.014"
"   Li QS, 2007, ENVIRON POLLUT, V149, P158, DOI 10.1016/j.envpol.2007.01.006"
"   Li Z, 2023, J ENVIRON MANAGE, V329, DOI 10.1016/j.jenvman.2022.117035"
"   Liang RZ, 2023, MAR POLLUT BULL, V186, DOI 10.1016/j.marpolbul.2022.114445"
"   Liang RS, 2024, SCI REP-UK, V14, DOI 10.1038/s41598-023-50754-5"
"   Lin Q, 2016, CATENA, V145, P193, DOI 10.1016/j.catena.2016.06.003"
"   Liu FD, 2019, RSC ADV, V9, P30615, DOI 10.1039/c9ra05332h"
"   Liu J, 2023, CATENA, V231, DOI 10.1016/j.catena.2023.107357"
"   Liu JN, 2008, J HAZARD MATER, V151, P261, DOI 10.1016/j.jhazmat.2007.08.016"
"   Liu XZ, 2024, SCI TOTAL ENVIRON, V912, DOI 10.1016/j.scitotenv.2023.169537"
"   Liu YZ, 2017, SCI TOTAL ENVIRON, V601, P580, DOI 10.1016/j.scitotenv.2017.05.212"
"   Liu YZ, 2015, SCI TOTAL ENVIRON, V511, P298, DOI 10.1016/j.scitotenv.2014.12.077"
"   Liu YZ, 2015, J ENVIRON MANAGE, V147, P12, DOI 10.1016/j.jenvman.2014.09.005"
"   Lofrano G, 2017, ENVIRON SCI POLLUT R, V24, P5189, DOI 10.1007/s11356-016-8281-x"
"   Loredo-Portales R, 2020, ENVIRON SCI POLLUT R, V27, P26605, DOI 10.1007/s11356-020-09051-1"
"   Lu YL, 2018, ENVIRON POLLUT, V239, P670, DOI 10.1016/j.envpol.2018.04.016"
"   Luo MH, 2022, SCI TOTAL ENVIRON, V807, DOI 10.1016/j.scitotenv.2021.151070"
"   Magalhaes DD, 2015, ENVIRON CHEM LETT, V13, P69, DOI 10.1007/s10311-015-0491-9"
"   Maier R. M., 2015, ENV MICROBIOLOGY, P377"
"   Maletic SP, 2019, J HAZARD MATER, V365, P467, DOI 10.1016/j.jhazmat.2018.11.020"
"   Malyan SK, 2021, WATER ENVIRON RES, V93, P1882, DOI 10.1002/wer.1599"
"   Mao LC, 2015, CHEMOSPHERE, V131, P201, DOI 10.1016/j.chemosphere.2015.03.035"
"   Marchand C, 2006, MAR CHEM, V98, P1, DOI 10.1016/j.marchem.2005.06.001"
"   Martino LE, 2016, ENVIRON EARTH SCI, V75, DOI 10.1007/s12665-016-6219-8"
"   Mathew R, 2020, OCEAN DYNAM, V70, P435, DOI 10.1007/s10236-020-01345-9"
"   Mattuck R, 1996, J CONTAM HYDROL, V23, P213, DOI 10.1016/0169-7722(95)00097-6"
"   Mayanna S, 2015, CHEM GEOL, V402, P6, DOI 10.1016/j.chemgeo.2015.02.029"
"   Meng L, 2024, J HAZARD MATER, V476, DOI 10.1016/j.jhazmat.2024.134930"
"   Miao XY, 2021, ECOTOX ENVIRON SAFE, V214, DOI 10.1016/j.ecoenv.2021.112099"
"   Miranda LS, 2022, J HAZARD MATER, V421, DOI 10.1016/j.jhazmat.2021.126743"
"   Miranda LS, 2021, WATER RES, V202, DOI 10.1016/j.watres.2021.117386"
"   Miranda LS, 2021, SCI TOTAL ENVIRON, V763, DOI 10.1016/j.scitotenv.2020.142984"
"   V??lez JMM, 2018, ENVIRON SCI POLICY, V88, P72, DOI 10.1016/j.envsci.2018.06.016"
"   Mukherjee J, 2022, ENVIRON SCI POLLUT R, V29, P21365, DOI 10.1007/s11356-021-17297-6"
"   Munson KM, 2018, BIOGEOSCIENCES, V15, P6451, DOI 10.5194/bg-15-6451-2018"
"   Murphy P, 2006, J ENVIRON ENG, V132, P787, DOI 10.1061/(ASCE)0733-9372(2006)132:7(787)"
"   Mustafa SA, 2024, EGYPT J AQUATIC RES, V50, P225, DOI 10.1016/j.ejar.2024.02.006"
"   Naser HA, 2013, MAR POLLUT BULL, V72, P6, DOI 10.1016/j.marpolbul.2013.04.030"
"   Nie X, 2024, ENVIRON EARTH SCI, V83, DOI 10.1007/s12665-024-11561-x"
"   Nnaji ND, 2023, SN APPL SCI, V5, DOI 10.1007/s42452-023-05351-6"
"   Noe GB, 2020, WIRES WATER, V7, DOI 10.1002/wat2.1454"
"   Nordmyr L, 2008, ESTUAR COAST SHELF S, V76, P141, DOI 10.1016/j.ecss.2007.07.002"
"   O'Brien RM, 2021, CRIT REV ENV SCI TEC, V51, P1259, DOI 10.1080/10643389.2020.1755203"
"   Olaniran AO, 2013, INT J MOL SCI, V14, P10197, DOI 10.3390/ijms140510197"
"   Olsen M., 2019, Contaminated Sediments: Review of Solutions for Protecting Aquatic Environments"
"   Ondrasek G, 2012, ABIOTIC STRESS RESPONSES IN PLANTS: METABOLISM, PRODUCTIVITY AND SUSTAINABILITY, P403, DOI 10.1007/978-1-4614-0634-1_22"
"   Otunola BO, 2020, ENVIRON TECHNOL INNO, V18, DOI 10.1016/j.eti.2020.100692"
"   Pal A, 2022, CRIT REV MICROBIOL, V48, P327, DOI 10.1080/1040841X.2021.1970512"
"   Palermo M., 2014, PROCESSES ASSESSMENT, P365, DOI DOI 10.1007/978-1-4614-6726-7_13"
"   Palleiro L, 2016, CATENA, V144, P34, DOI 10.1016/j.catena.2016.04.019"
"   Pan F, 2021, J HAZARD MATER, V418, DOI 10.1016/j.jhazmat.2021.126200"
"   Parsons CT, 2013, ENVIRON POLLUT, V178, P254, DOI 10.1016/j.envpol.2013.02.028"
"   Pedersen HD, 2006, GEOCHIM COSMOCHIM AC, V70, P4116, DOI 10.1016/j.gca.2006.06.1370"

"   Peng WH, 2018, J SOIL SEDIMENT, V18, P1701, DOI 10.1007/s11368-018-1921-7"
"   Persaud N., 2021, The Role of Monitoring and Evaluation in the UN 2030 SDGs Agenda, P89"
"   Phillipson J, 2012, J ENVIRON MANAGE, V95, P56, DOI 10.1016/j.jenvman.2011.10.005"
"   Pontier H, 2004, SCI TOTAL ENVIRON, V319, P215, DOI 10.1016/S0048-9697(03)00410-8"
"   Qi XY, 2023, HYDROL RES, V54, P491, DOI 10.2166/nh.2023.093"
"   Qian Y, 2012, ENVIRON POLLUT, V166, P23, DOI 10.1016/j.envpol.2012.02.027"
"   Qu CC, 2019, ENVIRON INT, V131, DOI 10.1016/j.envint.2019.104995"
"   Ram R, 2021, MINER ENG, V163, DOI 10.1016/j.mineng.2021.106801"
"   Rebai N, 2022, J MAR SCI ENG, V10, DOI 10.3390/jmse10111625"
"   Reed MS, 2008, BIOL CONSERV, V141, P2417, DOI 10.1016/j.biocon.2008.07.014"
"   Remaili TM, 2016, ENVIRON POLLUT, V208, P590, DOI 10.1016/j.envpol.2015.10.033"
"   Saidon NB, 2024, ENVIRON POLLUT, V340, DOI 10.1016/j.envpol.2023.122815"
"   Saroop S., 2021, HEAVY METALS ENV, P57, DOI DOI 10.1016/B978-0-12-821656-9.00004-3"
"   Sarwar N, 2017, CHEMOSPHERE, V171, P710, DOI 10.1016/j.chemosphere.2016.12.116"
"   Schaefer JK, 2020, ENVIRON MICROBIOL, V22, P3685, DOI 10.1111/1462-2920.15134"
"   Shah S., 2021, Heavy Metals in Scleractinian Corals. SpringerBriefs in Earth Sciences, DOI [10.1007/978-3-030-73613-2_1, DOI 10.1007/978-3-030-73613-2_1, 10.1007/978-3-030-73613-21]"
"   Shahsavari S., 2017, PhD Thesis"
"   Sharma N, 2022, ACS EARTH SPACE CHEM, V6, P1331, DOI 10.1021/acsearthspacechem.2c00031"
"   Shi MQ, 2021, SCI TOTAL ENVIRON, V752, DOI 10.1016/j.scitotenv.2020.141930"
"   Silva TP, 2024, INT SOIL WATER CONSE, V12, P121, DOI 10.1016/j.iswcr.2023.06.003"
"   Singh D, 2008, COMMUN SOIL SCI PLAN, V39, P2971, DOI 10.1080/00103620802432873"
"   Skyllberg U, 2021, GEOCHIM COSMOCHIM AC, V294, P106, DOI 10.1016/j.gca.2020.11.022"
"   Song YA, 2019, SCI TOTAL ENVIRON, V663, P568, DOI 10.1016/j.scitotenv.2019.01.347"
"   Stankovic S, 2013, ENV CHEM SUSTAIN WOR, V3, P151, DOI 10.1007/978-94-007-6836-9_5"
"   Stein ED, 2009, MAR POLLUT BULL, V59, P91, DOI 10.1016/j.marpolbul.2009.02.025"
"   Szalinska E, 2021, SCI TOTAL ENVIRON, V755, DOI 10.1016/j.scitotenv.2020.142898"
"   Tanaskovski B, 2014, MACED J CHEM CHEM EN, V33, P137"
"   Tian K, 2020, ENVIRON INT, V136, DOI 10.1016/j.envint.2020.105512"
"   Todaro F, 2020, ENVIRON SCI POLLUT R, V27, P21407, DOI 10.1007/s11356-020-08562-1"
"   Tong XE, 2021, WATER RES, V200, DOI 10.1016/j.watres.2021.117298"
"   Verma S, 2019, ENVIRON TECHNOL INNO, V14, DOI 10.1016/j.eti.2019.100369"
"   Walker TR, 2013, REMEDIATION, V23, P123, DOI 10.1002/rem.21371"
"   Wang HM, 2015, BIOTECHNOL ADV, V33, P317, DOI 10.1016/j.biotechadv.2015.04.003"
"   Wang L, 2017, CHEMOSPHERE, V184, P594, DOI 10.1016/j.chemosphere.2017.06.025"
"   Wang LW, 2021, SOIL USE MANAGE, V37, P936, DOI 10.1111/sum.12717"
"   Wang Q, 2022, SCI TOTAL ENVIRON, V807, DOI 10.1016/j.scitotenv.2021.150973"
"   Wang Q, 2020, ENVIRON POLLUT, V263, DOI 10.1016/j.envpol.2020.114565"
"   Wang XX, 2020, ENVIRON SCI TECHNOL, V54, P14124, DOI 10.1021/acs.est.0c05459"
"   Wang YP, 2013, J GEOPHYS RES-OCEANS, V118, P5591, DOI 10.1002/jgrc.20340"
"   Xie MW, 2019, ENVIRON POLLUT, V250, P87, DOI 10.1016/j.envpol.2019.04.003"
"   Xiong RW, 2024, J ENVIRON MANAGE, V349, DOI 10.1016/j.jenvman.2023.119428"
"   Xu DM, 2021, J CLEAN PROD, V321, DOI 10.1016/j.jclepro.2021.128730"
"   Xu H, 2021, ENVIRON SCI POLLUT R, V28, P10718, DOI 10.1007/s11356-020-11110-6"
"   Xu QQ, 2022, INT J ENV RES PUB HE, V19, DOI 10.3390/ijerph192416767"
"   Xu XY, 2022, ENVIRON MONIT ASSESS, V194, DOI 10.1007/s10661-022-10258-7"
"   Xu Z, 2022, CHEM ENG J, V429, DOI 10.1016/j.cej.2021.131688"
"   Yadav KK, 2023, MAR POLLUT BULL, V188, DOI 10.1016/j.marpolbul.2022.114569"
"   Yan FQ, 2023, LAND USE POLICY, V127, DOI 10.1016/j.landusepol.2023.106555"
"   Yang H, 2024, J CLEAN PROD, V435, DOI 10.1016/j.jclepro.2023.140529"
"   Yu FR, 2023, ENVIRON SCI POLLUT R, V30, P96055, DOI 10.1007/s11356-023-29059-7"
"   Yu KH, 2023, WATER RES, V242, DOI 10.1016/j.watres.2023.120185"
"   Yu PD, 2024, ECOHYDROL HYDROBIOL, V24, P820, DOI 10.1016/j.ecohyd.2022.01.002"
"   Zhang C, 2016, ENVIRON SCI POLLUT R, V23, P4370, DOI 10.1007/s11356-016-6076-8"
"   Zhang C, 2014, ENVIRON INT, V73, P270, DOI 10.1016/j.envint.2014.08.010"
"   Zhang HY, 2020, J SOIL SEDIMENT, V20, P109, DOI 10.1007/s11368-019-02413-4"
"   Zhang M, 2022, MAR POLLUT BULL, V181, DOI 10.1016/j.marpolbul.2022.113872"
"   Zhang WH, 2024, CATENA, V246, DOI 10.1016/j.catena.2024.108360"
"   Zhao SN, 2024, TOXICS, V12, DOI 10.3390/toxics12070494"
"   Zhou CY, 2020, ENVIRON INT, V145, DOI 10.1016/j.envint.2020.106140"
"   Zhou YF, 2010, CRIT REV ENV SCI TEC, V40, P909, DOI 10.1080/10643380802586857"
"   Zhuang QF, 2018, CATENA, V170, P386, DOI 10.1016/j.catena.2018.06.037"
"   Zhuang YH, 2016, J SOIL WATER CONSERV, V71, p98A, DOI 10.2489/jswc.71.4.98A"
"   Zhuo TY, 2023, WATER RES, V245, DOI 10.1016/j.watres.2023.120647"
NR 207
TC 103
Z9 116
U1 70
U2 159
PU MDPI
PI BASEL
"PA MDPI AG, Grosspeteranlage 5, CH-4052 BASEL, SWITZERLAND"
EI 2071-1050
J9 SUSTAINABILITY-BASEL
JI Sustainability
PD JAN
PY 2025
VL 17
IS 2
AR 701
DI 10.3390/su17020701
PG 29
WC Green & Sustainable Science & Technology; Environmental Sciences;
   Environmental Studies

WE Science Citation Index Expanded (SCI-EXPANDED); Social Science Citation Index (SSCI)
SC Science & Technology - Other Topics; Environmental Sciences & Ecology
GA T5I1O
UT WOS:001405329700001
"OA Green Submitted, gold"
DA 2026-04-06
ER

PT J
"AU Liu, SH"
" Zeng, GM"
" Niu, QY"
" Liu, Y"
" Zhou, L"
" Jiang, LH"
" Tan, XF"
" Xu, P"
" Zhang, C"
" Cheng, M"
"AF Liu, Shao-Heng"
" Zeng, Guang-Ming"
" Niu, Qiu-Ya"
" Liu, Yang"
" Zhou, Lu"
" Jiang, Lu-Hua"
" Tan, Xiao-fei"
" Xu, Piao"
" Zhang, Chen"
" Cheng, Min"
TI Bioremediation mechanisms of combined pollution of PAHs and heavy metals by bacteria and fungi: A mini review
SO BIORESOURCE TECHNOLOGY
LA English
DT Review
DE PAHs; Heavy metals; Bioremediation mechanisms; Bacteria; Fungi
ID POLYCYCLIC AROMATIC-HYDROCARBONS; WEIGHT ORGANIC-ACIDS; CONTAMINATED-SOIL; AQUEOUS-SOLUTION; PHANEROCHAETE-CHRYSOSPORIUM; MICROBIAL ACTIVITY; ESCHERICHIA-COLI; OXIDATIVE DAMAGE; HUMIC SUBSTANCES; DEGRADATION
"AB In recent years, knowledge in regard to bioremediation of combined pollution of polycyclic aromatic hydrocarbons (PAHs) and heavy metals by bacteria and fungi has been widely developed. This paper reviews the species of bacteria and fungi which can tackle with various types of PAHs and heavy metals entering into environment simultaneously or successively. Microbial activity, pollutants bioavailability and environmental factors (e.g. pH, temperature, low molecular weight organic acids and humic acids) can all affect the bioremediation of PAHs and heavy metals. Moreover, this paper summarizes the remediation mechanisms of PAHs and heavy metals by microbes via elucidating the interaction mechanisms of heavy metals with heavy metals, PAHs/PAHs metabolites with PAHs and PAHs with heavy metals. Based on the above reviews, this paper also discusses the potential research needs for this field. (C) 2016 Elsevier Ltd. All rights reserved."
"C1 [Zeng, Guang-Ming] Hunan Univ, Coll Environm Sci & Engn, Changsha 410082, Hunan, Peoples R China."
" Hunan Univ, Minist Educ, Key Lab Environm Biol & Pollut Control, Changsha 410082, Hunan, Peoples R China."
C3 Hunan University; Hunan University
"RP Zeng, GM (corresponding author), Hunan Univ, Coll Environm Sci & Engn, Changsha 410082, Hunan, Peoples R China."
EM zgming@hnu.edu.cn
"RI guangming, zeng/AHD-0360-2022; Tan, Xiaofei/ABD-6971-2021; Cheng,"
" Min/AAM-6401-2021; Liu, Yang/IYS-9887-2023; Zhang, Chen/AAY-8585-2021"
"OI Tan, Xiaofei/0000-0002-5750-8441; Cheng, Min/0000-0003-1086-4008; Liu,"
" Yang/0000-0002-8993-5771; Zhang, Chen/0000-0002-3579-6980"
"FU National Natural Science Foundation of China [51521006, 51378190,"
51108166]; Program for New Century Excellent Talents in University [NCET-13-0186]; Program for Changjiang Scholars and Innovative Research Team in University [IRT-13R17]
FX This research was financially supported by the National Natural Science
" Foundation of China (51521006, 51378190, 51108166), the Program for New"
Century Excellent Talents in University (NCET-13-0186) and the Program for Changjiang Scholars and Innovative Research Team in University (IRT-13R17).
"CR Al-Turki A. I., 2009, Research Journal of Environmental Toxicology, V3, P1, DOI 10.3923/rjet.2009.1.8"
" Atagana HI, 2009, AFR J BIOTECHNOL, V8, P5780"
" Baldrian P, 2000, APPL ENVIRON MICROB, V66, P2471, DOI 10.1128/AEM.66.6.2471-2478.2000"
" Bamforth SM, 2005, J CHEM TECHNOL BIOT, V80, P723, DOI 10.1002/jctb.1276"
" Bandowe BAM, 2014, ENVIRON INT, V65, P135, DOI 10.1016/j.envint.2013.12.018"
" Barmo C, 2011, CHEMOSPHERE, V83, P1087, DOI 10.1016/j.chemosphere.2011.01.037"
" BENEDETTI MF, 1995, ENVIRON SCI TECHNOL, V29, P446, DOI 10.1021/es00002a022"
" Biswas B, 2015, J HAZARD MATER, V298, P129, DOI 10.1016/j.jhazmat.2015.05.009"
" Booth SC, 2015, FRONT MICROBIOL, V6, DOI 10.3389/fmicb.2015.00827"
" Brito EMS, 2015, SCI TOTAL ENVIRON, V521, P1, DOI 10.1016/j.scitotenv.2015.02.098"
" Chaturvedi AD, 2015, WORLD J MICROB BIOT, V31, P1595, DOI 10.1007/s11274-015-1911-5"
" Chen M, 2015, BIOTECHNOL ADV, V33, P745, DOI 10.1016/j.biotechadv.2015.05.003"
" Chen SN, 2014, BIORESOURCE TECHNOL, V158, P181, DOI 10.1016/j.biortech.2014.02.020"
" Cheng M, 2016, CHEM ENG J, V284, P582, DOI 10.1016/j.cej.2015.09.001"
" Chung MK, 2007, SEP PURIF TECHNOL, V54, P355, DOI 10.1016/j.seppur.2006.10.008"
" Cirillo T, 2012, ARCH ENVIRON CON TOX, V62, P118, DOI 10.1007/s00244-011-9676-9"

"   Dimkpa CO, 2008, CHEMOSPHERE, V74, P19, DOI 10.1016/j.chemosphere.2008.09.079"
"   Ehrlich HL, 1997, APPL MICROBIOL BIOT, V48, P687, DOI 10.1007/s002530051116"
"   Fan T, 2008, J HAZARD MATER, V160, P655, DOI 10.1016/j.jhazmat.2008.03.038"
"   Farmer PB, 2003, MUTAT RES-REV MUTAT, V544, P397, DOI 10.1016/j.mrrev.2003.09.002"
"   Feng YA, 2010, CHEM ENG J, V162, P487, DOI 10.1016/j.cej.2010.05.049"
"   Galazka A, 2015, POL J MICROBIOL, V64, P241, DOI 10.5604/01.3001.0009.2119"
"   Gao YZ, 2015, SOIL TILL RES, V145, P103, DOI 10.1016/j.still.2014.09.008"
"   Gauthier PT, 2015, ENVIRON SCI TECHNOL, V49, P11780, DOI 10.1021/acs.est.5b03233"
"   Gauthier PT, 2014, AQUAT TOXICOL, V154, P253, DOI 10.1016/j.aquatox.2014.05.026"
"   Giner-Lamia J, 2014, PLOS ONE, V9, DOI 10.1371/journal.pone.0108912"
"   Gong JL, 2009, J HAZARD MATER, V164, P1517, DOI 10.1016/j.jhazmat.2008.09.072"
"   Gorria M, 2006, ANN NY ACAD SCI, V1090, P108, DOI 10.1196/annals.1378.011"
"   Guo HJ, 2010, BIORESOURCE TECHNOL, V101, P8599, DOI 10.1016/j.biortech.2010.06.085"
"   Guo PY, 2015, INT J ENVIRON SCI TE, V12, P2339, DOI 10.1007/s13762-014-0633-9"
"   Gupta S, 2015, REV ENVIRON SCI BIO, V14, P241, DOI 10.1007/s11157-014-9353-3"
"   Haritash AK, 2009, J HAZARD MATER, V169, P1, DOI 10.1016/j.jhazmat.2009.03.137"
"   Hildebrandt U, 2007, PHYTOCHEMISTRY, V68, P139, DOI 10.1016/j.phytochem.2006.09.023"
"   Hu XJ, 2011, J HAZARD MATER, V185, P306, DOI 10.1016/j.jhazmat.2010.09.034"
"   Huang DL, 2008, ENVIRON SCI TECHNOL, V42, P4946, DOI 10.1021/es800072c"
"   Huang RY, 2015, AER ADV ENG RES, V17, P1151"
"   Jiang J, 2015, ECOTOX ENVIRON SAFE, V120, P386, DOI 10.1016/j.ecoenv.2015.06.028"
"   Karaca A, 2010, SOIL BIOL, V19, P237, DOI 10.1007/978-3-642-02436-8_11"
"   Ke L, 2010, BIORESOURCE TECHNOL, V101, P6950, DOI 10.1016/j.biortech.2010.04.011"
"   Khan Z, 2015, APPL MICROBIOL BIOT, V99, P10745, DOI 10.1007/s00253-015-6901-x"
"   Kuang D, 2013, ENVIRON SCI TECHNOL, V47, P7446, DOI 10.1021/es401639x"
"   Kuppusamy S, 2016, INT BIODETER BIODEGR, V108, P149, DOI 10.1016/j.ibiod.2015.12.013"
"   Li F, 2015, ENVIRON SCI TECHNOL, V49, P3698, DOI 10.1021/es504673j"
"   Li H, 2016, AER ADV ENG RES, V75, P1"
"   Li HL, 2014, BIORESOURCE TECHNOL, V163, P193, DOI 10.1016/j.biortech.2014.04.042"
"   Liang C, 2003, BIORESOURCE TECHNOL, V86, P131, DOI 10.1016/S0960-8524(02)00153-0"
"   Liu SH, 2015, RSC ADV, V5, P55812, DOI 10.1039/c5ra04867b"
"   Lu M, 2014, ENVIRON SCI TECHNOL, V48, P1158, DOI 10.1021/es403337t"
"   Luna JM, 2016, PROCESS SAF ENVIRON, V102, P558, DOI 10.1016/j.psep.2016.05.010"
"   Ma XK, 2016, APPL MICROBIOL BIOT, V100, P7741, DOI 10.1007/s00253-016-7595-4"
"   Ma XK, 2014, APPL MICROBIOL BIOT, V98, P9817, DOI 10.1007/s00253-014-5905-2"
"   Ma Y, 2016, J ENVIRON MANAGE, V174, P14, DOI 10.1016/j.jenvman.2016.02.047"
"   Mahmoudi N, 2011, CAN J MICROBIOL, V57, P623, DOI [10.1139/W11-049, 10.1139/w11-049]"
"   Manzano R, 2014, ENVIRON SCI POLLUT R, V21, P4539, DOI 10.1007/s11356-013-2412-4"
"   Meng PP, 2014, BIORESOURCE TECHNOL, V157, P316, DOI 10.1016/j.biortech.2014.01.095"
"   Michalec FG, 2016, AQUAT TOXICOL, V170, P310, DOI 10.1016/j.aquatox.2015.08.010"
"   Momose Y, 2008, ANTON LEEUW INT J G, V94, P165, DOI 10.1007/s10482-008-9222-6"
"   Moscoso F, 2012, BIORESOURCE TECHNOL, V119, P270, DOI 10.1016/j.biortech.2012.05.095"
"   Mulligan CN, 1999, ENVIRON SCI TECHNOL, V33, P3812, DOI 10.1021/es9813055"
"   Niederer M, 1995, ENVIRON SCI POLLUT R, V2, P83, DOI 10.1007/BF02986721"
"   Nouha K, 2016, BIORESOURCE TECHNOL, V212, P120, DOI 10.1016/j.biortech.2016.04.021"
"   Osman D, 2008, ADV APPL MICROBIOL, V65, P217, DOI 10.1016/S0065-2164(08)00608-4"
"   Patel V, 2012, BIORESOURCE TECHNOL, V107, P122, DOI 10.1016/j.biortech.2011.12.056"
"   Poulsen TG, 2010, ENVIRON SCI TECHNOL, V44, P5086, DOI 10.1021/es9038243"
"   Qin F, 2004, CHEMOSPHERE, V57, P253, DOI 10.1016/j.chemosphere.2004.06.010"
"   Saha R, 2013, J BASIC MICROB, V53, P303, DOI 10.1002/jobm.201100552"
"   Shen GQ, 2006, ECOTOX ENVIRON SAFE, V63, P474, DOI 10.1016/j.ecoenv.2005.01.009"
"   Shen GQ, 2005, CHEMOSPHERE, V61, P1175, DOI 10.1016/j.chemosphere.2005.02.074"
"   Su HL, 2009, BIOMATERIALS, V30, P5979, DOI 10.1016/j.biomaterials.2009.07.030"
"   Tang L, 2008, ENVIRON SCI TECHNOL, V42, P1207, DOI 10.1021/es7024593"
"   Thavamani P, 2012, J ENVIRON MANAGE, V99, P10, DOI 10.1016/j.jenvman.2011.12.030"
"   van de Kreeke J, 2010, TRAC-TREND ANAL CHEM, V29, P928, DOI 10.1016/j.trac.2010.04.009"
"   Wang JL, 2009, BIOTECHNOL ADV, V27, P195, DOI 10.1016/j.biotechadv.2008.11.002"
"   Wang MH, 2010, ECOTOXICOLOGY, V19, P273, DOI 10.1007/s10646-009-0410-6"
"   Wang YJ, 2009, J HAZARD MATER, V162, P1135, DOI 10.1016/j.jhazmat.2008.06.001"
"   WEISSENFELS WD, 1992, APPL MICROBIOL BIOT, V36, P689, DOI 10.1007/BF00183251"
"   Wen JW, 2011, INT BIODETER BIODEGR, V65, P600, DOI 10.1016/j.ibiod.2011.03.003"
"   Wiatrowska K, 2015, J ELEMENTOL, V20, P743, DOI 10.5601/jelem.2014.19.2.675"
"   WILLIAMS JW, 1984, ENZYME MICROB TECH, V6, P530, DOI 10.1016/0141-0229(84)90081-4"
"   Won EJ, 2011, COMP BIOCHEM PHYS C, V154, P82, DOI 10.1016/j.cbpc.2011.03.008"
"   Wu G, 2010, J HAZARD MATER, V174, P1, DOI 10.1016/j.jhazmat.2009.09.113"
"   Wu LH, 2003, CHEMOSPHERE, V50, P819, DOI 10.1016/S0045-6535(02)00225-4"
"   Xiao JJ, 2010, J HAZARD MATER, V174, P818, DOI 10.1016/j.jhazmat.2009.09.126"
"   Xu PA, 2012, SCI TOTAL ENVIRON, V424, P1, DOI 10.1016/j.scitotenv.2012.02.023"
"   Yan GY, 2003, WATER RES, V37, P4486, DOI 10.1016/S0043-1354(03)00409-3"
"   Zeng GM, 2012, ENVIRON SCI TECHNOL, V46, P7818, DOI 10.1021/es301006j"
"   Zeng GM, 2013, NATURE, V499, P154, DOI 10.1038/499154c"
"   Zeng GM, 2013, SCIENCE, V340, P1403, DOI 10.1126/science.340.6139.1403-a"
"   Zhang C, 2016, WATER RES, V95, P103, DOI 10.1016/j.watres.2016.03.014"
"   Zhang C, 2014, RSC ADV, V4, P55511, DOI 10.1039/c4ra09563d"
"   Zhang WH, 2015, J SOIL SEDIMENT, V15, P1463, DOI 10.1007/s11368-015-1112-8"
"   Zhang WH, 2011, CHEMOSPHERE, V83, P302, DOI 10.1016/j.chemosphere.2010.12.056"
"   Zhang XX, 2006, PEDOSPHERE, V16, P555, DOI 10.1016/S1002-0160(06)60088-X"
"   Zhang Y, 2007, BIOSENS BIOELECTRON, V22, P2121, DOI 10.1016/j.bios.2006.09.030"
"   Zhang YD, 2013, SCI WORLD J, DOI 10.1155/2013/130134"
"   Zhou YY, 2016, SENSOR ACTUAT B-CHEM, V223, P280, DOI 10.1016/j.snb.2015.09.090"
"   Zhu DQ, 2004, J ENVIRON QUAL, V33, P276, DOI 10.2134/jeq2004.0276"
"   Zloch M, 2016, CHEMOSPHERE, V156, P312, DOI 10.1016/j.chemosphere.2016.04.130"
"   Zouboulis AI, 2004, PROCESS BIOCHEM, V39, P909, DOI 10.1016/S0032-9592(03)00200-0"
NR 99

TC 410
Z9 477
U1 27
U2 1715
PU ELSEVIER SCI LTD
PI OXFORD
"PA THE BOULEVARD, LANGFORD LANE, KIDLINGTON, OXFORD OX5 1GB, OXON, the United Kingdom"
SN 0960-8524
EI 1873-2976
J9 BIORESOURCE TECHNOL
JI Bioresour. Technol.
PD JAN
PY 2017
VL 224
BP 25
EP 33
DI 10.1016/j.biortech.2016.11.095
PG 9
WC Agricultural Engineering; Biotechnology & Applied Microbiology; Energy &
   Fuels
WE Science Citation Index Expanded (SCI-EXPANDED)
SC Agriculture; Biotechnology & Applied Microbiology; Energy & Fuels
GA EN0HW
UT WOS:000395691900003
PM 27916498
DA 2026-04-06
ER

PT J
"AU Zhang, JH"
"   Wang, NY"
"   Zhang, FS"
AF Zhang Jianhua
   Wang Naiyan
   Zhang Fengshou
TI Analysis of Accumulating Ability of Heavy Metals in Lotus (Nelumbo
   nucifera) Improved by Ion Implantation
SO PLASMA SCIENCE & TECHNOLOGY
LA English
DT Article
DE ion implantation; lotus (Nelumbo nucifera); heavy metal; improvement
"AB Heavy metals have seriously contaminated soil and water, and done harm to public health. Academician WANG Naiyan proposed that ion-implantation technique should be exploited for environmental bioremediation by mutating and breeding plants or microbes. By implanting N+ into Taikonglian No.1, we have selected and bred two lotus cultivars, Jingguang No.1 and Jingguang No.2. The present study aims at analyzing the feasibility that irradiation can be used for remediation of soil and water from heavy metals. Compared with parent Taikonglian No.1, the uptaking and accumulating ability of heavy metals in two mutated cultivars was obviously improved. So ion implantation technique can indeed be used in bioremediation of heavy metals in soil and water, but it is hard to select and breed a cultivar which can remedy the soil and water from all the heavy metals."
"C1 [Zhang Jianhua; Wang Naiyan; Zhang Fengshou] Beijing Normal Univ, Coll Nucl Sci & Technol, Minist Educ, Key Lab Beam Technol & Mat Modificat, Beijing 100875, Peoples R China."
"   [Zhang Jianhua; Wang Naiyan; Zhang Fengshou] Beijing Radiat Ctr, Beijing 100875, Peoples R China."
C3 Beijing Normal University; Beijing Academy of Science & Technology
"RP Wang, NY (corresponding author), Beijing Normal Univ, Coll Nucl Sci & Technol, Minist Educ, Key Lab Beam Technol & Mat Modificat, Beijing 100875, Peoples R China."
EM wangny@public.bta.net.cn
"RI Zhang, Fengshou/K-1522-2013"
FU National Natural Science Foundation of China [11075019]; Beijing
   Ion-Irradiating-Breeding Research Platform Project of China
FX supported by National Natural Science Foundation of China (No.
"   11075019), Beijing Ion-Irradiating-Breeding Research Platform Project of"
   China
"CR [?????? Bao Tong], 2008, [????€???????, Ecology and Environment], V17, P858"
"   Chen X, 1999, TOBACCO SCI TECHNOLO, V2, P35"
"   Qu Ran, 2008, Shengtaixue Zazhi, V27, P626"
"   Tan CY, 1999, CHINESE PHYS LETT, V16, P123, DOI 10.1088/0256-307X/16/2/017"
"   Vajpayee P, 2000, CHEMOSPHERE, V41, P1075, DOI 10.1016/S0045-6535(99)00426-9"
"   Wang Q, 2010, ENVIRON SCI TECHNOL, V5, P96"
"   Wu YJ, 1990, J NUCL AGR SCI, V11, P4"
"   Wu YJ, 1989, J ANHUI AGR SCI, V2, P12"
"   ?????????, 2004, [????????????, Acta Agriculturae Nucleatae Sinica], V18, P303"
"   Xu LS, 2010, EARTH ENV, V3, P372"
"   Yu Zengliang, 2006, P1, DOI 10.1109/VTCF.2006.218"
"   Yu ZL, 1991, J ANHUI AGR COLL, V18, P276"
"   Yu ZL, 1989, J ANHUI AGR SCI, V1, P26"
"   Zhang L, 2009, CHINESE J SPECTROSCO, V2, P316"
"   Zheng C, 2009, NEW PHYTOL, V1, P1"
NR 15
TC 2
Z9 4
U1 2
U2 37
PU IOP Publishing Ltd

PI BRISTOL
"PA TEMPLE CIRCUS, TEMPLE WAY, BRISTOL BS1 6BE, the United Kingdom"
SN 1009-0630
EI 2058-6272
J9 PLASMA SCI TECHNOL
JI Plasma Sci. Technol.
PD MAY
PY 2012
VL 14
IS 5
BP 424
EP 426
DI 10.1088/1009-0630/14/5/21
PG 3
"WC Physics, Fluids & Plasmas"
WE Science Citation Index Expanded (SCI-EXPANDED)
SC Physics
GA 947CO
UT WOS:000304404400021
DA 2026-04-06
ER

PT J
"AU Al Disi, Z"
"   Al-Ghouti, MA"
"   Zouari, N"
"AF Al Disi, Zulfa"
"   Al-Ghouti, Mohammad A."
"   Zouari, Nabil"
TI Investigating the simultaneous removal of hydrocarbons and heavy metals
   by highly adapted Bacillus and Pseudomonas strains
SO ENVIRONMENTAL TECHNOLOGY & INNOVATION
LA English
DT Article
DE Weathered oil; Diesel Range Organics; Hydrocarbon-degrading bacteria;
   Heavy metals; Bacillus; Pseudomonas
ID CONTAMINATED SOILS; MALDI-TOF; OIL; REMEDIATION; BIOAUGMENTATION;
   BIOAVAILABILITY; BIODEGRADATION; BIOREMEDIATION; METALLOIDS; DIVERSITY
"AB The status, content and availability of heavy metals and hydrocarbons are highly affected by weathering processes - particularly - in areas characterized by harsh conditions. Their concomitant removal by highly adapted strains of Bacillus and Pseudomonas to weathered oil components was investigated. Indeed, weathered soil collected from Dukhan dumpsite was shown to contain 14 heavy metals with concentrations exceeding the US-EPA limits. Their distribution in such soil was not affected by the soil organics, which is important for the remediation processes. Most of them were strongly bonded to the iron-manganese oxide and the residual fractions, justifying their bioremediation. This study used 18 adapted bacterial strains isolated from extremely weathered oily soils, including the studied soil, all shown tolerant up to 5 mM and above of heavy metals. 4 Bacillus and 3 Pseudomonas strains exhibited the capability to remove 70%-80% of the heavy metals. Concomitantly, they removed up to 73% of the diesel-range organics. Using the PCA methodology with multivariate (bacterial type and adaptation and heavy metals properties), the efficiency of heavy metals removal was shown not related to the adapted bacteria, but to the heavy metal status. This means that the adaptation of these bacterial strains was at the level of the cells' structure and/or their exopolymeric substances which immobilize the heavy metals and reduce their toxicity allowing their growth and removing hydrocarbons. It is then, a double-adaptation route, leading to concomitant removal of organics and heavy metals, which is of high importance from the practical point of view. (C) 2022 The Authors. Published by Elsevier B.V."
"C1 [Al Disi, Zulfa; Al-Ghouti, Mohammad A.; Zouari, Nabil] Qatar Univ, Coll Arts & Sci, Dept Biol & Environm Sci, Environm Sci Program, POB 2713, Doha, Qatar."
C3 Qatar University
"RP Zouari, N (corresponding author), Qatar Univ, Coll Arts & Sci, Dept Biol & Environm Sci, Environm Sci Program, POB 2713, Doha, Qatar."
EM Nabil.Zouari@qu.edu.qa
"RI Al-Ghouti, Mohammad/ONJ-6577-2025; Al Disi, Zulfa/J-1630-2019"
"OI Al Disi, Zulfa/0000-0003-0935-4545"
"FU Qatar National Research Fund, Qatar (a member of Qatar Foundation)"
   [PDRA5-0425-19007]; Qatar National Library
FX This publication was made possible by PDRA Grant No. PDRA5-0425-19007
"   from the Qatar National Research Fund, Qatar (a member of Qatar"
   Foundation) . The statements made herein are solely the responsibility
   of the authors. Open Access funding provided by the Qatar National
   Library.
"CR Agnello AC, 2016, SCI TOTAL ENVIRON, V563, P693, DOI 10.1016/j.scitotenv.2015.10.061"
"   Al Disi Z, 2017, BIOMED RES INT, V2017, DOI 10.1155/2017/8649350"
"   Al-Kaabi N, 2018, J PETROL SCI ENG, V168, P569, DOI 10.1016/j.petrol.2018.05.013"
"   Al-Kaabi NS, 2017, J PETROL SCI ENG, V149, P107, DOI 10.1016/j.petrol.2016.10.034"
"   AlKaabi Nasser, 2020, Biotechnology Reports, V28, pe00543, DOI [10.1016/j.btre.2020.e00543, 10.1016/j.btre.2020.e00543]"
"   Allen RJ, 2019, REP PROG PHYS, V82, DOI 10.1088/1361-6633/aae546"
"   Arora NK, 2018, ENVIRON SUSTAIN, V1, P305, DOI [10.1007/s42398-018-00036-y, 10.1007/s42398-018-00036-y, DOI 10.1007/S42398-018-00036-Y]"
"   Attar A, 2017, RSC ADV, V7, P45920, DOI 10.1039/c7ra04704e"
"   Ayangbenro AS, 2018, SUSTAINABILITY-BASEL, V10, DOI 10.3390/su10093028"
"   Ayangbenro AS, 2017, INT J ENV RES PUB HE, V14, DOI 10.3390/ijerph14010094"
"   Biswas B, 2018, SOIL SYST, V2, DOI 10.3390/soilsystems2030051"
"   Briffa J, 2020, HELIYON, V6, DOI 10.1016/j.heliyon.2020.e04691"

"   Caporale AG, 2016, CURR POLLUT REP, V2, P15, DOI 10.1007/s40726-015-0024-y"
"   Cih??k M, 2017, FRONT MICROBIOL, V8, DOI 10.3389/fmicb.2017.02495"
"   Das Nilanjana, 2011, Biotechnol Res Int, V2011, P941810, DOI 10.4061/2011/941810"
"   Dell'Anno F, 2020, MICROORGANISMS, V8, DOI 10.3390/microorganisms8091402"
"   Fayeulle A, 2014, ENVIRON SCI POLLUT R, V21, P3515, DOI 10.1007/s11356-013-2324-3"
"   Filote C, 2021, PROCESSES, V9, DOI 10.3390/pr9101696"
"   Govarthanan M, 2016, 3 BIOTECH, V6, DOI 10.1007/s13205-016-0560-1"
"   Gupta Pratima, 2017, Biotechnol Rep (Amst), V13, P58, DOI [10.1016/j.btre.2016.12.006, 10.1016/j.btre.2016.12.006]"
"   Gurav R, 2017, ENVIRON SCI POLLUT R, V24, P11392, DOI 10.1007/s11356-017-8446-2"
"   Igiri BE, 2018, J TOXICOL-US, V2018, DOI 10.1155/2018/2568038"
"   Izidoro JD, 2012, ADV MATER RES-SWITZ, V356-360, P1900, DOI 10.4028/www.scientific.net/AMR.356-360.1900"
"   Jallouli W, 2010, PROCESS BIOCHEM, V45, P706, DOI 10.1016/j.procbio.2010.01.007"
"   Jiang Y, 2016, CHEMOSPHERE, V161, P300, DOI 10.1016/j.chemosphere.2016.07.032"
"   Joutey N.T., 2013, BOOK AGR BIOLOGICAL, DOI [DOI 10.5772/56194, 10.1061/40745(146)46, DOI 10.1061/40745(146)46]"
"   Juwarkar AA, 2010, REV ENVIRON SCI BIO, V9, P215, DOI 10.1007/s11157-010-9215-6"
"   Karlapudi Abraham Peele, 2018, Petroleum, V4, P241, DOI 10.1016/j.petlm.2018.03.007"
"   Khalid FE, 2021, J MAR SCI ENG, V9, DOI 10.3390/jmse9020155"
"   Kuippers G., 2021, MICROBIOLOGY NUCL WA, DOI [DOI 10.1016/B978-0-12-818695-4.00008-3, 10.1016/B978-0-12-818695-4.00008-3]"
"   Kwon MJ, 2017, WATER AIR SOIL POLL, V228, DOI 10.1007/s11270-017-3368-7"
"   Lala S., 2021, FRONT MICROBIOL, V12"
"   Li N, 2021, CHEMOSPHERE, V270, DOI 10.1016/j.chemosphere.2020.128662"
"   Liu SH, 2017, BIORESOURCE TECHNOL, V224, P25, DOI 10.1016/j.biortech.2016.11.095"
"   Ma YL, 2015, APPL BIOCHEM BIOTECH, V175, P1294, DOI 10.1007/s12010-014-1347-7"
"   Manasi, 2016, Journal of Genetic Engineering and Biotechnology, V14, P177, DOI [10.1016/j.jgeb.2016.02.002, 10.1016/j.jgeb.2016.02.002]"
"   Marzan LW, 2017, EGYPT J AQUATIC RES, V43, P65, DOI [10.1016/j.ejar.2016.11.002, 10.1016/j.ejar.2016.11.002, DOI 10.1016/j.ejar.2016.11.002, DOI 10.1016/J.EJAR.2016.11.002]"
"   Tarekegn MM, 2020, COGENT FOOD AGR, V6, DOI 10.1080/23311932.2020.1783174"
"   Migoni D, 2021, APPL SCI-BASEL, V11, DOI 10.3390/app11052115"
"   Mohanakrishna G, 2019, RSC ADV, V9, P41409, DOI 10.1039/c9ra06957g"
"   Olaniran AO, 2013, INT J MOL SCI, V14, P10197, DOI 10.3390/ijms140510197"
"   Oualha M, 2019, J ENVIRON MANAGE, V250, DOI 10.1016/j.jenvman.2019.109455"
"   Peng M, 2015, INT J ENV RES PUB HE, V12, P12002, DOI 10.3390/ijerph121012002"
"   Pulles T, 2012, ATMOS ENVIRON, V61, P641, DOI 10.1016/j.atmosenv.2012.07.022"
"   Ram??rez V, 2019, FRONT MICROBIOL, V10, DOI 10.3389/fmicb.2019.01833"
"   Rosenthal AZ, 2018, ELIFE, V7, DOI 10.7554/eLife.33099"
"   Samad RA, 2020, RSC ADV, V10, P14606, DOI 10.1039/d0ra01229g"
"   Singh SK, 2019, PGPR AMELIORATION IN SUSTAINABLE AGRICULTURE: FOOD SECURITY AND ENVIRONMENTAL MANAGEMENT, P239, DOI 10.1016/B978-0-12-815879-1.00012-4"
"   Timkov?? I, 2018, SEPARATIONS, V5, DOI 10.3390/separations5040054"
"   Ukaogo PO., 2020, Microorganisms for Sustainable Environment and Health, P419, DOI [DOI 10.1016/B978-0-12-819001-2.00021-8, https://doi.org/10.1016/B978-0-12-819001-, DOI 10.1016/B978-0-12-819001]"
"   Usman MM, 2016, AIMS BIOENG, V3, P289, DOI 10.3934/bioeng.2016.3.289"
"   Uysal A, 2017, WATER SCI TECHNOL, V76, P2733, DOI 10.2166/wst.2017.450"
"   Violante A, 2010, J SOIL SCI PLANT NUT, V10, P268, DOI 10.4067/S0718-95162010000100005"
"   Xu XJ, 2018, FRONT MICROBIOL, V9, DOI 10.3389/fmicb.2018.02885"
"   Zaynab M, 2022, J KING SAUD UNIV SCI, V34, DOI 10.1016/j.jksus.2021.101653"
"   Ziyath A. M., 2011, Journal of Water Resource and Protection, V3, P758, DOI 10.4236/jwarp.2011.310086"
NR 56
TC 28
Z9 31
U1 3
U2 50
PU ELSEVIER
PI AMSTERDAM
"PA RADARWEG 29, 1043 NX AMSTERDAM, NETHERLANDS"
SN 2352-1864
J9 ENVIRON TECHNOL INNO
JI Environ. Technol. Innov.
PD AUG
PY 2022
VL 27
AR 102513
DI 10.1016/j.eti.2022.102513
EA APR 2022
PG 14
"WC Biotechnology & Applied Microbiology; Engineering, Environmental;"
   Environmental Sciences
WE Science Citation Index Expanded (SCI-EXPANDED)
SC Biotechnology & Applied Microbiology; Engineering; Environmental
   Sciences & Ecology
GA 3I7EY
UT WOS:000832876300017
"OA Green Submitted, gold"
DA 2026-04-06
ER

PT J
"AU Hou, DY"
"   O'Connor, D"
"   Igalavithana, AD"

"   Alessi, DS"
"   Luo, J"
"   Tsang, DCW"
"   Sparks, DL"
"   Yamauchi, Y"
"   Rinklebe, J"
"   Ok, YS"
"AF Hou, Deyi"
"   O'Connor, David"
"   Igalavithana, Avanthi D."
"   Alessi, Daniel S."
"   Luo, Jie"
"   Tsang, Daniel C. W."
"   Sparks, Donald L."
"   Yamauchi, Yusuke"
"   Rinklebe, Jorg"
"   Ok, Yong Sik"
TI Metal contamination and bioremediation of agricultural soils for food safety and sustainability
SO NATURE REVIEWS EARTH & ENVIRONMENT
LA English
DT Review
ID HEALTH-RISK ASSESSMENT; SHORT-ROTATION COPPICE; PTERIS-VITTATA L.; HEAVY-METAL; IN-SITU; TRACE-ELEMENTS; ECOLOGICAL RISK; FIELD-SCALE; CADMIUM PHYTOREMEDIATION; SPATIAL-DISTRIBUTION
"AB Agricultural soil is a non-renewable natural resource that requires careful stewardship in order to achieve the United Nations' Sustainable Development Goals. However, industrial and agricultural activity is often detrimental to soil health and can distribute heavy metal(loid)s into the soil environment, with harmful effects on human and ecosystem health. In this Review, we examine processes that can lead to the contamination of agricultural land with heavy metal(loid)s, which range from mine tailings runoff entering local irrigation channels to the atmospheric deposition of incinerator and coal-fired power-plant emissions. We discuss the relationship between heavy metal(loid) biogeochemical transformations in the soil and their bioavailability. We then review two biological solutions for remediation of contaminated agricultural land, plant-based remediation and microbial bioremediation, which offer cost-effective and sustainable alternatives to traditional physical or chemical remediation technologies. Finally, we discuss how integrating these innovative technologies with profitable and sustainable land use could lead to green and sustainable remediation strategies, and conclude by identifying research challenges and future directions for the biological remediation of agricultural soils."
"   Contamination of agricultural soils by heavy metals and metalloids has severe consequences on human and ecosystem health. This Review discusses the sources of heavy metal(loid) contamination, the mechanisms by which these contaminants interact with biological and geochemical soil elements, and plant-based and microorganism-based remediation strategies."
"C1 [Hou, Deyi; O'Connor, David] Tsinghua Univ, Sch Environm, Beijing, Peoples R China."
"   [Igalavithana, Avanthi D.; Ok, Yong Sik] Korea Univ, Korea Biochar Res Ctr, APRU Sustainable Waste Management Program, Seoul, South Korea."
"   [Igalavithana, Avanthi D.; Ok, Yong Sik] Korea Univ, Div Environm Sci & Ecol Engn, Seoul, South Korea."
"   [Alessi, Daniel S.] Univ Alberta, Dept Earth & Atmospher Sci, Edmonton, AB, Canada."
"   [Luo, Jie] Yangtze Univ, Coll Resources & Environm, Wuhan, Peoples R China."
"   [Tsang, Daniel C. W.] Hong Kong Polytech Univ, Dept Civil & Environm Engn, Kowloon, Hong Kong, Peoples R China."
"   [Sparks, Donald L.] Univ Delaware, Dept Plant & Soil Sci, Newark, DE 19717 USA."
"   [Sparks, Donald L.] Univ Delaware, Delaware Environm Inst, Newark, DE USA."
"   [Yamauchi, Yusuke] Univ Queensland, Australian Inst Bioengn & Nanotechnol AIBN, Brisbane, Qld, Australia."
"   [Yamauchi, Yusuke] Natl Inst Mat Sci NIMS, Int Ctr Mat Nanoarchitecton, Tsukuba, Ibaraki, Japan."
"   [Rinklebe, Jorg] Univ Wuppertal, Inst Fdn Engn, Sch Architecture & Civil Engn, Wuppertal, Germany."
C3 Tsinghua University; Korea University; Korea University; University of Alberta; Yangtze University; Hong Kong Polytechnic University; University of Delaware; University of Delaware; University of Queensland; National Institute for Materials Science; University of Wuppertal
"RP Ok, YS (corresponding author), Korea Univ, Korea Biochar Res Ctr, APRU Sustainable Waste Management Program, Seoul, South Korea.; Ok, YS (corresponding author), Korea Univ, Div Environm Sci & Ecol Engn, Seoul, South Korea."
EM yongsikok@korea.ac.kr
"RI Rinklebe, Joerg/Y-2398-2019; Alessi, Daniel/AAI-9106-2020; O'Connor,"
"   David/ACC-9467-2022; Hou, Deyi/B-1030-2016; Yamauchi,"
"   Yusuke/D-2780-2015; Tsang, Dan/E-5442-2012; Ok, Yong Sik/R-6739-2016;"
"   Igalavithana, Avanthi Deshani/J-5198-2019"
"OI O'Connor, David/0000-0003-3858-9247; Hou, Deyi/0000-0002-0511-5806;"
"   Yamauchi, Yusuke/0000-0001-7854-927X; Tsang, Dan/0000-0002-6850-733X;"
"   Ok, Yong Sik/0000-0003-3401-0912; Igalavithana, Avanthi"
Deshani/0000-0001-5322-8584
FU Cooperative Research Program for Agriculture Science and Technology
"   Development, Rural Development Administration, Republic of Korea"
[PJ01475801]; National Research Foundation of Korea (NRF) [NRF-2015R1A2A2A11001432]; NRF Germany-Korea Partnership Program (GEnKO Program) (2018-2020); KU Future Research Grant (KU FRG) Fund from the
"   Korea University, Republic of Korea; Korea Biochar Research Center"
"   (KBRC) Fund from the Korea University, Republic of Korea; Association of"
Pacific Rim Universities (APRU) Sustainable Waste Management Program
"   from the Korea University, Republic of Korea; National Key Research and"
Development Program of China [2018YFC1801300]; National Water Pollution Control and Treatment Science and Technology Major Project

[2018ZX07109-003]; Ministry of Ecology and Environment's National Soil
Pollution Investigation Project in China
FX This work was carried out with the support of the Cooperative Research
Program for Agriculture Science and Technology Development (project no.
"   PJ01475801), Rural Development Administration, Republic of Korea. This"
work was also supported by the National Research Foundation of Korea
(NRF) (NRF-2015R1A2A2A11001432) and the NRF Germany-Korea Partnership
Program (GEnKO Program) (2018-2020). Y.S. O. and A.D.I. were partly
"   supported by the KU Future Research Grant (KU FRG) Fund, Korea Biochar"
Research Center (KBRC) Fund and the Association of Pacific Rim
Universities (APRU) Sustainable Waste Management Program from the Korea
"   University, Republic of Korea. D.H. and D.O.C. were supported by the"
National Key Research and Development Program of China (grant no.
"   2018YFC1801300), the National Water Pollution Control and Treatment"
Science and Technology Major Project (no. 2018ZX07109-003) and the
Ministry of Ecology and Environment's National Soil Pollution
Investigation Project in China.
"CR Aboughalma H, 2008, J ENVIRON SCI HEAL A, V43, P926, DOI 10.1080/10934520801974459"
"   Achal V, 2011, ECOL ENG, V37, P1601, DOI 10.1016/j.ecoleng.2011.06.008"
"   Ahemad M, 2019, ARAB J CHEM, V12, P1365, DOI 10.1016/j.arabjc.2014.11.020"
"   Ahmad P, 2015, PLOS ONE, V10, DOI [10.46568/pjgs.v10i1.222, 10.1371/journal.pone.0114571]"
"   Al-Ahmad H, 2004, MOL ECOL, V13, P697, DOI 10.1046/j.1365-294X.2004.02092.x"
"   Alessi DS, 2014, ENVIRON SCI TECHNOL, V48, P12842, DOI 10.1021/es502701u"
"   Alsbou EME, 2018, ENVIRON MONIT ASSESS, V190, DOI 10.1007/s10661-017-6409-1"
"   Anantharaman K, 2016, NAT COMMUN, V7, DOI 10.1038/ncomms13219"
"   Andersson-Sk??ld Y, 2014, BIOFUELS-UK, V5, P487, DOI 10.1080/17597269.2014.996728"
"   Andrew RM, 2018, EARTH SYST SCI DATA, V10, P195, DOI 10.5194/essd-10-195-2018"
"   Angassa K, 2019, ENVIRON PROCESS, V6, P65, DOI 10.1007/s40710-019-00358-x"
"   Angelova V, 2004, IND CROP PROD, V19, P197, DOI 10.1016/j.indcrop.2003.10.001"
"   Antoniadis V, 2019, ENVIRON INT, V127, P819, DOI 10.1016/j.envint.2019.03.039"
"   Antoniadis V, 2017, EARTH-SCI REV, V171, P621, DOI 10.1016/j.earscirev.2017.06.005"
"   Antoniadis V, 2017, ENVIRON GEOCHEM HLTH, V39, P319, DOI 10.1007/s10653-016-9882-5"
"   Atafar Z, 2010, ENVIRON MONIT ASSESS, V160, P83, DOI 10.1007/s10661-008-0659-x"
"   BAKER AJM, 1994, RESOUR CONSERV RECY, V11, P41, DOI 10.1016/0921-3449(94)90077-9"
"   Balkhair KS, 2016, SAUDI J BIOL SCI, V23, pS32, DOI 10.1016/j.sjbs.2015.09.023"
"   Barbieri M, 2018, J GEOCHEM EXPLOR, V195, P78, DOI 10.1016/j.gexplo.2017.11.014"
"   Bargar JR, 2013, P NATL ACAD SCI USA, V110, P4506, DOI 10.1073/pnas.1219198110"
"   Bashan Y, 2002, BIOL FERT SOILS, V35, P359, DOI 10.1007/s00374-002-0481-5"
"   Beckers F, 2017, CRIT REV ENV SCI TEC, V47, P693, DOI 10.1080/10643389.2017.1326277"
"   Bennett LE, 2003, J ENVIRON QUAL, V32, P432, DOI 10.2134/jeq2003.0432"
"   Bhuiyan MSI, 2017, P INDIAN NATL SCI AC, V83, P51, DOI 10.16943/ptinsa/2016/48857"
"   Bi XY, 2006, ENVIRON INT, V32, P883, DOI 10.1016/j.envint.2006.05.010"
"   Blissett RS, 2012, FUEL, V97, P1, DOI 10.1016/j.fuel.2012.03.024"
"   Borrelli P, 2017, NAT COMMUN, V8, DOI 10.1038/s41467-017-02142-7"
"   Boussen S, 2013, GEODERMA, V192, P227, DOI 10.1016/j.geoderma.2012.08.029"
"   Braud A, 2009, CHEMOSPHERE, V74, P280, DOI 10.1016/j.chemosphere.2008.09.013"
"   Burges A, 2018, INT J PHYTOREMEDIAT, V20, P384, DOI 10.1080/15226514.2017.1365340"
"   Cameselle C, 2019, J HAZARD MATER, V361, P95, DOI 10.1016/j.jhazmat.2018.08.062"
"   Cao XD, 2003, SCI TOTAL ENVIRON, V307, P179, DOI 10.1016/S0048-9697(02)00543-0"
"   Caporale AG, 2016, CURR POLLUT REP, V2, P15, DOI 10.1007/s40726-015-0024-y"
"   Cassidy ES, 2013, ENVIRON RES LETT, V8, DOI 10.1088/1748-9326/8/3/034015"
"   Chalot M, 2012, ENVIRON SCI TECHNOL, V46, P13361, DOI 10.1021/es3017478"
"   Chandra R, 2016, FOR SCI TECHNOL, V12, P55, DOI 10.1080/21580103.2015.1044024"
"   Chang CY, 2019, ENVIRON SCI POLLUT R, V26, P6818, DOI 10.1007/s11356-019-04137-x"
"   Chaoua S., 2019, Journal of the Saudi Society of Agricultural Sciences, V18, P429, DOI 10.1016/j.jssas.2018.02.003"
"   Che-Castaldo JP, 2015, AUST J BOT, V63, P141, DOI 10.1071/BT15045"
"   Chen HY, 2015, SCI TOTAL ENVIRON, V512, P143, DOI 10.1016/j.scitotenv.2015.01.025"
"   Chen YH, 2004, APPL GEOCHEM, V19, P1553, DOI 10.1016/j.apgeochem.2004.02.003"
"   Chen YW, 2019, J GEOCHEM EXPLOR, V205, DOI 10.1016/j.gexplo.2019.106349"
"   Cheng SF, 2015, ECOTOX ENVIRON SAFE, V111, P72, DOI 10.1016/j.ecoenv.2014.09.024"
"   Chibuike G.U., 2014, APPL ENVIRON SOIL SC, DOI [10.1155/2014/752708, DOI 10.1155/2014/752708]"
"   Clemens S, 2006, BIOCHIMIE, V88, P1707, DOI 10.1016/j.biochi.2006.07.003"
"   Cohen CK, 1998, PLANT PHYSIOL, V116, P1063, DOI 10.1104/pp.116.3.1063"
"   Conesa HM, 2012, SCI WORLD J, DOI 10.1100/2012/173829"
"   Congeevaram S, 2007, J HAZARD MATER, V146, P270, DOI 10.1016/j.jhazmat.2006.12.017"
"   da Rocha U. N., 2019, MODERN SOIL MICROBIO, P295"
"   Damian GE, 2019, J AGR ENVIRON ETHIC, V32, P27, DOI 10.1007/s10806-019-09767-2"
"   Dary M, 2010, J HAZARD MATER, V177, P323, DOI 10.1016/j.jhazmat.2009.12.035"
"   Davison J, 2005, J IND MICROBIOL BIOT, V32, P639, DOI 10.1007/s10295-005-0242-1"
"   Deng XH, 2019, CAN J MICROBIOL, V65, P629, DOI 10.1139/cjm-2018-0624"
"   Deng XH, 2012, J HAZARD MATER, V233, P25, DOI 10.1016/j.jhazmat.2012.06.054"
"   Diels L, 1999, MOL BIOTECHNOL, V12, P149, DOI 10.1385/MB:12:2:149"
"   Diep P, 2018, FRONT BIOENG BIOTECH, V6, DOI 10.3389/fbioe.2018.00157"
"   Dimitrijevic MD, 2016, INT J ENVIRON SCI TE, V13, P615, DOI 10.1007/s13762-015-0905-z"
"   Din BU, 2020, PLANT PHYSIOL BIOCH, V146, P249, DOI 10.1016/j.plaphy.2019.11.010"
"   Dominish E., 2019, Responsible Minerals Sourcing for Renewable Energy"
"   Donati ER, 2019, FRONT MICROBIOL, V10, DOI 10.3389/fmicb.2019.01851"
"   D??ring RA, 2003, SCI TOTAL ENVIRON, V313, P227, DOI 10.1016/S0048-9697(03)00294-8"
"   Elghali A, 2019, SCI TOTAL ENVIRON, V654, P118, DOI 10.1016/j.scitotenv.2018.11.066"
"   Evangelou M.W. H., 2015, PHYTOREMEDIATION, P115, DOI [DOI 10.1007/978-3-319-10395-2_9, 10.1007/978-3-319-10395-2_9]"
"   Ewel JJ, 2019, TRENDS PLANT SCI, V24, P121, DOI 10.1016/j.tplants.2018.10.008"
"   Facchinelli A, 2001, ENVIRON POLLUT, V114, P313, DOI 10.1016/S0269-7491(00)00243-8"

" Fang W, 2016, J HAZARD MATER, V310, P1, DOI 10.1016/j.jhazmat.2016.02.025"
" Farhate CVV, 2019, SOIL USE MANAGE, V35, P273, DOI 10.1111/sum.12479"
" Favas PJC, 2019, PHYTOMANAGEMENT OF POLLUTED SITES: MARKET OPPORTUNITIES IN SUSTAINABLE PHYTOREMEDIATION, P277, DOI 10.1016/B978-0-12-813912-7.00010-7"
" Favas PJC, 2014, ENVIRONMENTAL RISK ASSESSMENT OF SOIL CONTAMINATION, P485, DOI 10.5772/57469"
" Feng XB, 2008, SCI TOTAL ENVIRON, V400, P227, DOI 10.1016/j.scitotenv.2008.05.040"
" Fisher-Power LM, 2016, CHEMOSPHERE, V144, P1973, DOI 10.1016/j.chemosphere.2015.10.109"
" Foley JA, 2011, NATURE, V478, P337, DOI 10.1038/nature10452"
" Food and Agriculture Organization of the United Nations, 2019, STAT FODD SEC NUTR"
" Fu YM, 2017, SCI HORTIC-AMSTERDAM, V214, P51, DOI 10.1016/j.scienta.2016.11.020"
" Gadd GM, 2010, MICROBIOL-SGM, V156, P609, DOI 10.1099/mic.0.037143-0"
" GADD GM, 1990, EXPERIENTIA, V46, P834, DOI 10.1007/BF01935534"
" Gadd GM, 2000, CURR OPIN BIOTECH, V11, P271, DOI 10.1016/S0958-1669(00)00095-1"
" Gadd GM, 2004, GEODERMA, V122, P109, DOI 10.1016/j.geoderma.2004.01.002"
" Galende MA, 2014, J GEOCHEM EXPLOR, V145, P181, DOI 10.1016/j.gexplo.2014.06.006"
" Greinert A, 2015, J SOIL SEDIMENT, V15, P1725, DOI 10.1007/s11368-014-1054-6"
" Grobelak A, 2017, J CLEAN PROD, V155, P189, DOI 10.1016/j.jclepro.2016.10.005"
" Guidi Nissim W, 2016, ECOL ENG, V91, P472, DOI 10.1016/j.ecoleng.2016.03.008"
" Gupta S., 2017, ADV ENV BIOTECHNOLOG, P197"
" Gusev A., 2019, ASSESSMENT TRANSBOUN"
" Hemen Sarma Hemen Sarma, 2011, Journal of Environmental Science and Technology, V4, P118"
" Hesse E, 2019, P ROY SOC B-BIOL SCI, V286, DOI [10.1101/441873, 10.1098/rspb.2019.0804]"
" Hesse E, 2018, ECOL LETT, V21, P117, DOI 10.1111/ele.12878"
" Hettiarachchi GM, 2004, ENVIRON PROG, V23, P78, DOI 10.1002/ep.10004"
" Hider RC, 2010, NAT PROD REP, V27, P637, DOI 10.1039/b906679a"
" Hou D, 2020, SUSTAINABLE REMEDIATION OF CONTAMINATED SOIL AND GROUNDWATER: MATERIALS, PROCESSES, AND ASSESSMENT, P1, DOI 10.1016/C2018-0-03003-0"
" Hou DY, 2018, LAND DEGRAD DEV, V29, P1005, DOI 10.1002/ldr.2748"
" Hou DY, 2019, NATURE, V566, P455, DOI 10.1038/d41586-019-00669-x"
" Hou DY, 2017, ENVIRON POLLUT, V231, P1188, DOI 10.1016/j.envpol.2017.07.021"
" Hou DY, 2017, LAND DEGRAD DEV, V28, P2315, DOI 10.1002/ldr.2741"
" Hou DY, 2014, ENVIRON SCI POLICY, V39, P25, DOI 10.1016/j.envsci.2014.02.003"
" Hou XL, 2018, FLORA, V240, P82, DOI 10.1016/j.flora.2018.01.006"
" Hu RP, 2019, ACTA AGR SCAND B-S P, V69, P304, DOI 10.1080/09064710.2018.1564786"
" Hu YN, 2016, ENVIRON INT, V92-93, P515, DOI 10.1016/j.envint.2016.04.042"
" Huang H, 2018, BIORESOURCE TECHNOL, V249, P487, DOI 10.1016/j.biortech.2017.10.020"
" Iqbal M, 2012, ENVIRON POLLUT, V170, P222, DOI 10.1016/j.envpol.2012.07.008"
" Jadhav U, 2016, RSC ADV, V6, P43442, DOI 10.1039/c6ra04169h"
" Jansson JK, 2020, NAT REV MICROBIOL, V18, P35, DOI 10.1038/s41579-019-0265-7"
" J??rup L, 2003, BRIT MED BULL, V68, P167, DOI 10.1093/bmb/ldg032"
" Jeong S, 2015, CHEMOSPHERE, V122, P1, DOI 10.1016/j.chemosphere.2014.10.003"
" Ji PH, 2011, ENVIRON POLLUT, V159, P762, DOI 10.1016/j.envpol.2010.11.029"
" Jiang JP, 2010, EUR J SOIL BIOL, V46, P18, DOI 10.1016/j.ejsobi.2009.10.001"
" Jiang YB, 2019, ECOL ENG, V129, P38, DOI 10.1016/j.ecoleng.2019.01.009"
" Jin YL, 2019, ENVIRON INT, V124, P320, DOI 10.1016/j.envint.2019.01.024"
" Jing F, 2020, SOIL USE MANAGE, V36, P320, DOI 10.1111/sum.12557"
" Kallmeyer J, 2012, P NATL ACAD SCI USA, V109, P16213, DOI 10.1073/pnas.1203849109"
" Kang SH, 2007, APPL ENVIRON MICROB, V73, P6317, DOI 10.1128/AEM.01237-07"
" Kasemodel MC, 2019, SCI TOTAL ENVIRON, V675, P367, DOI 10.1016/j.scitotenv.2019.04.223"
" Kavamura VN, 2010, BIOTECHNOL ADV, V28, P61, DOI 10.1016/j.biotechadv.2009.09.002"
" Keller C, 2005, ENVIRON SCI TECHNOL, V39, P3359, DOI 10.1021/es0484101"
" Khan S, 2008, ENVIRON POLLUT, V152, P686, DOI 10.1016/j.envpol.2007.06.056"
" Khaokaew S, 2015, CHEMOSPHERE, V138, P883, DOI 10.1016/j.chemosphere.2014.09.108"
" Kidd P, 2015, INT J PHYTOREMEDIAT, V17, P1005, DOI 10.1080/15226514.2014.1003788"
" Kim RY, 2015, ENVIRON GEOCHEM HLTH, V37, P1041, DOI 10.1007/s10653-015-9695-y"
" Knechtenhofer LA, 2003, J PLANT NUTR SOIL SC, V166, P84, DOI 10.1002/jpln.200390017"
" Kopittke PM, 2019, ENVIRON INT, V132, DOI 10.1016/j.envint.2019.105078"
" Kumar V, 2019, SOIL USE MANAGE, V35, P303, DOI 10.1111/sum.12473"
" Lawniczak L, 2020, MOLECULES, V25, DOI 10.3390/molecules25040856"
" Lebeau T., 2011, SOIL BIOL, V108, P129"
" Li B, 2016, INORG CHEM FRONT, V3, P175, DOI 10.1039/c5qi00187k"
" Li C, 2015, ENVIRON POLLUT, V206, P264, DOI 10.1016/j.envpol.2015.07.007"
" Li D, 2013, SEP PURIF TECHNOL, V120, P52, DOI 10.1016/j.seppur.2013.09.018"
" Li JT, 2009, CHEMOSPHERE, V76, P1233, DOI 10.1016/j.chemosphere.2009.05.042"
" Li NY, 2016, PEDOSPHERE, V26, P717, DOI 10.1016/S1002-0160(15)60082-0"
" Li WL, 2014, SCI TOTAL ENVIRON, V472, P407, DOI 10.1016/j.scitotenv.2013.11.046"
" Li XD, 2004, ENVIRON POLLUT, V129, P113, DOI 10.1016/j.envpol.2003.09.030"
" Li XX, 2019, ECOTOX ENVIRON SAFE, V168, P1, DOI 10.1016/j.ecoenv.2018.10.012"
" Li Z, 2018, CHEMOSPHERE, V194, P432, DOI 10.1016/j.chemosphere.2017.12.005"
" Li Z, 2016, ENVIRON POLLUT, V209, P123, DOI 10.1016/j.envpol.2015.11.015"
" Liang LC, 2017, ENVIRON REV, V25, P269, DOI 10.1139/er-2016-0063"
" Liang XJ, 2017, MICROB BIOTECHNOL, V10, P1199, DOI 10.1111/1751-7915.12767"
" Liang YZ, 2019, SCI TOTAL ENVIRON, V656, P521, DOI 10.1016/j.scitotenv.2018.11.386"
" Lim JE, 2017, ENVIRON GEOCHEM HLTH, V39, P431, DOI 10.1007/s10653-016-9867-4"
" Lovley DR, 2003, NAT REV MICROBIOL, V1, P35, DOI 10.1038/nrmicro731"
" Lovley DR, 1997, CURR OPIN BIOTECH, V8, P285, DOI 10.1016/S0958-1669(97)80005-5"
" Lu AX, 2012, SCI TOTAL ENVIRON, V425, P66, DOI 10.1016/j.scitotenv.2012.03.003"
" Luj??n AM, 2015, BIOL LETTERS, V11, DOI 10.1098/rsbl.2014.0934"
" Luo CL, 2011, J HAZARD MATER, V186, P481, DOI 10.1016/j.jhazmat.2010.11.024"
" Luo J, 2019, CHEMOSPHERE, V227, P462, DOI 10.1016/j.chemosphere.2019.04.083"
" Luo J, 2016, J CLEAN PROD, V119, P25, DOI 10.1016/j.jclepro.2016.01.043"
" Ma J, 2018, CHEMOSPHERE, V194, P737, DOI 10.1016/j.chemosphere.2017.11.135"
" Ma LQ, 2001, NATURE, V409, P579, DOI 10.1038/35054664"
" Madsen EL, 2011, CURR OPIN BIOTECH, V22, P456, DOI 10.1016/j.copbio.2011.01.008"

"   Mangalassery S, 2015, EUR J SOIL BIOL, V68, P9, DOI 10.1016/j.ejsobi.2015.03.001"
"   Marrugo-Negrete J, 2017, ENVIRON RES, V154, P380, DOI 10.1016/j.envres.2017.01.021"
"   Mayerov?? M, 2017, ENVIRON SCI POLLUT R, V24, P14706, DOI 10.1007/s11356-017-9051-0"
"   Meers E, 2010, CHEMOSPHERE, V78, P35, DOI 10.1016/j.chemosphere.2009.08.015"
"   MEHARG AA, 1992, J EXP BOT, V43, P519, DOI 10.1093/jxb/43.4.519"
"   Mertens J, 2006, ENVIRON SCI TECHNOL, V40, P1962, DOI 10.1021/es051225i"
"   Mesa J, 2015, J HAZARD MATER, V300, P263, DOI 10.1016/j.jhazmat.2015.07.006"
"   Mic?? C, 2006, CHEMOSPHERE, V65, P863, DOI 10.1016/j.chemosphere.2006.03.016"
"   Ministry of Environmental Protection, 2014, CHIN NAT SOIL CONT S"
"   Montgomery DR, 2007, P NATL ACAD SCI USA, V104, P13268, DOI 10.1073/pnas.0611508104"
"   Moreno F. N., 2004, Environmental Practice, V6, P165, DOI 10.1017/S1466046604000274"
"   Mulligan CN, 2001, ENG GEOL, V60, P193, DOI 10.1016/S0013-7952(00)00101-0"
"   Murtaza G, 2017, INT J PHYTOREMEDIAT, V19, P199, DOI 10.1080/15226514.2016.1207605"
"   Navarro MC, 2008, J GEOCHEM EXPLOR, V96, P183, DOI 10.1016/j.gexplo.2007.04.011"
"   Newsome L, 2014, CHEM GEOL, V363, P164, DOI 10.1016/j.chemgeo.2013.10.034"
"   Niazi NK, 2017, INT J PHYTOREMEDIAT, V19, P670, DOI 10.1080/15226514.2016.1278427"
"   Nicholson FA, 2003, SCI TOTAL ENVIRON, V311, P205, DOI 10.1016/S0048-9697(03)00139-6"
"   Nies DH, 2000, EXTREMOPHILES, V4, P77, DOI 10.1007/s007920050140"
"   Noli F, 2016, SCI TOTAL ENVIRON, V563, P377, DOI 10.1016/j.scitotenv.2016.04.098"
"   Nziguheba G, 2008, SCI TOTAL ENVIRON, V390, P53, DOI 10.1016/j.scitotenv.2007.09.031"
"   O'Brien S, 2015, EMBO REP, V16, P1241, DOI 10.15252/embr.201541064"
"   O'Connor D, 2020, NAT SUSTAIN, V3, P77, DOI 10.1038/s41893-020-0475-z"
"   O'Connor D, 2019, ENVIRON INT, V126, P747, DOI 10.1016/j.envint.2019.03.019"
"   O'Connor D, 2018, SCI TOTAL ENVIRON, V619, P815, DOI 10.1016/j.scitotenv.2017.11.132"
"   Ogunkunle CO, 2013, POL J ENVIRON STUD, V22, P487"
"   Okoro H.K., 2012, OPEN ACCESS SCI REPO, V1, P1, DOI [10.4172/scientificreports.181, DOI 10.4172/SCIENTIFICREPORTS.181]"
"   Palumbo B, 2000, GEODERMA, V95, P247, DOI 10.1016/S0016-7061(99)00090-7"
"   Panda D, 2018, INT J PHYTOREMEDIAT, V20, P538, DOI 10.1080/15226514.2017.1393394"
"   Park JH, 2011, J HAZARD MATER, V185, P549, DOI 10.1016/j.jhazmat.2010.09.082"
"   Paya Perez A., 2018, Status of Local Soil Contamination in Europe"
"   Plewniak F, 2018, FRONT MICROBIOL, V9, DOI 10.3389/fmicb.2018.00820"
"   Pratas J, 2017, ENVIRON EARTH SCI, V76, DOI 10.1007/s12665-017-6459-2"
"   Praveen A, 2019, ECOTOX ENVIRON SAFE, V182, DOI 10.1016/j.ecoenv.2019.109462"
"   Pushpanathan M, 2014, ELSEV INSIGHT, P407, DOI 10.1016/B978-0-12-800021-2.00017-0"
"   Rajendran P., 2003, Indian Journal of Experimental Biology, V41, P935"
"   Rajkumar M, 2012, BIOTECHNOL ADV, V30, P1562, DOI 10.1016/j.biotechadv.2012.04.011"
"   Rascio N, 2011, PLANT SCI, V180, P169, DOI 10.1016/j.plantsci.2010.08.016"
"   Rasoulnia P, 2016, WASTE MANAGE, V52, P309, DOI 10.1016/j.wasman.2016.04.004"
"   Rehman K, 2018, J HAZARD MATER, V349, P242, DOI 10.1016/j.jhazmat.2018.02.013"
"   Rehman MZU, 2017, ECOTOX ENVIRON SAFE, V143, P236, DOI 10.1016/j.ecoenv.2017.05.038"
"   Ren WX, 2009, J HAZARD MATER, V167, P164, DOI 10.1016/j.jhazmat.2008.12.104"
"   Rieuwerts JS, 1998, CHEM SPEC BIOAVAILAB, V10, P61, DOI 10.3184/095422998782775835"
"   Robinson BH, 2009, CRIT REV PLANT SCI, V28, P240, DOI 10.1080/07352680903035424"
"   Mart??n JAR, 2006, ENVIRON POLLUT, V144, P1001, DOI 10.1016/j.envpol.2006.01.045"
"   Rodriguez-Eugenio N., 2018, Soil Pollution: A Hidden Reality, pvi"
"   Rogich D., 2008, The global flows of metals and minerals"
"   Romic M, 2003, ENVIRON GEOL, V43, P795, DOI 10.1007/s00254-002-0694-9"
"   Ruttens A, 2011, INT J PHYTOREMEDIAT, V13, P194, DOI 10.1080/15226514.2011.568543"
"   Salehi FM, 2016, TRIBOL LETT, V63, DOI 10.1007/s11249-016-0704-9"
"   Sampanpanish P, 2019, B ENVIRON CONTAM TOX, V102, P140, DOI 10.1007/s00128-018-2502-6"
"   Schreurs E, 2011, BIOMASS BIOENERG, V35, P4469, DOI 10.1016/j.biombioe.2011.09.005"
"   Schr??der P, 2018, SCI TOTAL ENVIRON, V616, P1101, DOI 10.1016/j.scitotenv.2017.10.209"
"   Serna-Chavez HM, 2013, GLOBAL ECOL BIOGEOGR, V22, P1162, DOI 10.1111/geb.12070"
"   Shaheen SM, 2014, GEODERMA, V228, P142, DOI 10.1016/j.geoderma.2013.10.012"
"   Shahid M, 2017, J HAZARD MATER, V325, P36, DOI 10.1016/j.jhazmat.2016.11.063"
"   Shahid M, 2017, REV ENVIRON CONTAM T, V241, P73, DOI 10.1007/398_2016_8"
"   Shan YS, 2013, J SOIL SEDIMENT, V13, P720, DOI 10.1007/s11368-012-0637-3"
"   Shen ZJ, 2017, HORTIC ENVIRON BIOTE, V58, P93, DOI 10.1007/s13580-017-0077-5"
"   Shenker M, 2005, SOIL SCI PLANT NUTR, V51, P1, DOI 10.1111/j.1747-0765.2005.tb00001.x"
"   Shrestha P, 2019, INT J ENV RES PUB HE, V16, DOI 10.3390/ijerph16071261"
"   Smith E, 2008, SCI TOTAL ENVIRON, V392, P277, DOI 10.1016/j.scitotenv.2007.11.023"
"   Sneller FEC, 2000, J AGR FOOD CHEM, V48, P4014, DOI 10.1021/jf9903105"
"   Song YA, 2019, SCI TOTAL ENVIRON, V663, P568, DOI 10.1016/j.scitotenv.2019.01.347"
"   SRIVASTAVA SK, 1989, WATER RES, V23, P1161, DOI 10.1016/0043-1354(89)90160-7"
"   Tao HY, 2019, SOIL USE MANAGE, V35, P314, DOI 10.1111/sum.12483"
"   Thornton SF, 2020, J HAZARD MATER, V391, DOI 10.1016/j.jhazmat.2020.122046"
"   Tilman D, 2011, P NATL ACAD SCI USA, V108, P20260, DOI 10.1073/pnas.1116437108"
"   T??th G, 2016, ENVIRON INT, V88, P299, DOI 10.1016/j.envint.2015.12.017"
"   Touceda-Gonz??lez M, 2017, J ENVIRON MANAGE, V186, P301, DOI 10.1016/j.jenvman.2016.09.019"
"   Tozs??r D, 2018, ENVIRON SCI POLLUT R, V25, P3275, DOI 10.1007/s11356-017-0699-2"
"   T??rkdogan MK, 2003, ENVIRON TOXICOL PHAR, V13, P175, DOI 10.1016/S1382-6689(02)00156-4"
"   United Nations Environment Assembly of the United Nations Environment Programme, 2018, MAN SOIL POLL ACH SU"
"   United Nations Environment Assembly of the United Nations Environment Programme, 2019, IMPL PLAN POLL FREE"
"   Unver IK, 2018, J MIN ENVIRON, V9, P641, DOI 10.22044/jme.2018.6855.1518"
"   Valls M, 2002, FEMS MICROBIOL REV, V26, P327, DOI 10.1111/j.1574-6976.2002.tb00618.x"
"   Van Huysen T, 2003, PLANTA, V218, P71, DOI 10.1007/s00425-003-1070-z"
"   Van Slycken S, 2013, INT J PHYTOREMEDIAT, V15, P677, DOI 10.1080/15226514.2012.723070"
"   Vangronsveld J, 2009, ENVIRON SCI POLLUT R, V16, P765, DOI 10.1007/s11356-009-0213-6"
"   Visoottiviseth P, 2002, ENVIRON POLLUT, V118, P453, DOI 10.1016/S0269-7491(01)00293-7"
"   Vromman D, 2018, ENVIRON SCI POLLUT R, V25, P34473, DOI 10.1007/s11356-018-3351-x"
"   Wallschlager D, 1998, J ENVIRON QUAL, V27, P1034, DOI 10.2134/jeq1998.00472425002700050008x"

"   Walter I, 2006, ENVIRON POLLUT, V139, P507, DOI 10.1016/j.envpol.2005.05.020"
"   Wan XM, 2017, SCI TOTAL ENVIRON, V579, P1467, DOI 10.1016/j.scitotenv.2016.11.148"
"   Wan XM, 2016, SCI TOTAL ENVIRON, V563, P796, DOI 10.1016/j.scitotenv.2015.12.080"
"   Wang J, 2019, ENVIRON SCI TECHNOL, V53, P2500, DOI 10.1021/acs.est.8b06863"
"   Wang L, 2017, CHEMOSPHERE, V184, P594, DOI 10.1016/j.chemosphere.2017.06.025"
"   Wang LW, 2020, CRIT REV ENV SCI TEC, V50, P2724, DOI 10.1080/10643389.2019.1705724"
"   Wang XJ, 2018, ANN BOT-LONDON, V121, P143, DOI 10.1093/aob/mcx138"
"   Wang XT, 2018, INT J BIOL MACROMOL, V113, P655, DOI 10.1016/j.ijbiomac.2018.02.081"
"   Wang X, 2018, SCI ADV, V4, DOI 10.1126/sciadv.aaq0210"
"   Wang X, 2019, ENVIRON SCI TECHNOL, V53, P10665, DOI 10.1021/acs.est.9b02386"
"   White C, 1998, NAT BIOTECHNOL, V16, P572, DOI 10.1038/nbt0698-572"
"   Wilkin RT, 2008, MINE WATER ENVIRON, V27, P251, DOI 10.1007/s10230-008-0049-1"
"   Willscher S, 2013, HYDROMETALLURGY, V131, P46, DOI 10.1016/j.hydromet.2012.08.012"
"   Xie ZY, 2020, NAT SUSTAIN, V3, P9, DOI 10.1038/s41893-019-0433-9"
"   Yadav SK, 2010, S AFR J BOT, V76, P167, DOI 10.1016/j.sajb.2009.10.007"
"   Yao AJ, 2006, APPL GEOCHEM, V21, P446, DOI 10.1016/j.apgeochem.2005.10.003"
"   Yergeau E, 2014, ISME J, V8, P344, DOI 10.1038/ismej.2013.163"
"   Yin WJ, 2015, J MATER CHEM A, V3, P8926, DOI 10.1039/c4ta05033a"
"   Young CC, 2006, BIOTECHNOL BIOENG, V95, P76, DOI 10.1002/bit.20957"
"   Zayed AM, 2003, PLANT SOIL, V249, P139, DOI 10.1023/A:1022504826342"
"   Zhang F, 2012, INT J ENV RES PUB HE, V9, P1715, DOI 10.3390/ijerph9051715"
"   Zhang L, 2019, PLANTA, V249, P1007, DOI 10.1007/s00425-018-3057-9"
"   Zhang XY, 2020, CHEMOSPHERE, V241, DOI 10.1016/j.chemosphere.2019.125027"
"   Zhang YH, 2019, CRIT REV ENV SCI TEC, V49, P1386, DOI 10.1080/10643389.2019.1571354"
"   Zhao FJ, 2015, ENVIRON SCI TECHNOL, V49, P750, DOI 10.1021/es5047099"
"   Zhao FJ, 2003, PLANT SOIL, V249, P37, DOI 10.1023/A:1022530217289"
"   Zhao HR, 2012, SCI TOTAL ENVIRON, V417, P45, DOI 10.1016/j.scitotenv.2011.12.047"
"   Zhou J, 2016, ECOTOXICOLOGY, V25, P380, DOI 10.1007/s10646-015-1596-4"
NR 256
TC 868
Z9 983
U1 137
U2 1588
PU SPRINGERNATURE
PI LONDON
"PA CAMPUS, 4 CRINAN ST, LONDON, N1 9XW, the United Kingdom"
EI 2662-138X
J9 NAT REV EARTH ENV
JI Nat. Rev. Earth Environ.
PD JUL
PY 2020
VL 1
IS 7
BP 366
EP 381
DI 10.1038/s43017-020-0061-y
PG 16
"WC Environmental Sciences; Geosciences, Multidisciplinary; Meteorology &"
   Atmospheric Sciences
WE Science Citation Index Expanded (SCI-EXPANDED)
SC Environmental Sciences & Ecology; Geology; Meteorology & Atmospheric
   Sciences
GA SA6YP
UT WOS:000649447900009
DA 2026-04-06
ER

PT J
"AU Ye, CS"
"   Pan, XC"
"   Feng, HP"
"   Zhou, QW"
"   Wu, WH"
"   Jin, MQ"
"   Fu, L"
"   Ji, WY"
"   Zhan, LC"
"AF Ye, Chenshuai"
"   Pan, Xingchen"
"   Feng, Haopeng"
"   Zhou, Qingwei"
"   Wu, Weihong"
"   Jin, Meiqing"
"   Fu, Li"
"   Ji, Weiying"
"   Zhan, Lichuan"
TI Effects of Phosphorus-Containing Compounds on the Remediation and Soil
"   Microbiota in Rice Contaminated by Lead, Zinc, and Cadmium"
SO EURASIAN SOIL SCIENCE
LA English
DT Article
DE exogenous phosphorus; BCR; PLFA; microbial community
ID HEAVY-METALS; ZN; COMMUNITY; CD; NI; L.; CU

"AB In the present study, a wetland root box experiment was conducted to investigate the impact of exogenous phosphorus (calcium dihydrogen phosphate) on heavy metal distribution and soil microbial population of rice using Bureau Community of Reference (BCR) sequential extraction and Phospholipid Fatty Acid (PLFA) analysis. The experimental results demonstrate that adding calcium dihydrogen phosphate could reduce levels of acid-soluble, reduced, and oxidized heavy metals while minimizing their toxicity to rice. However, an exception was observed with cadmium, which showed an adverse effect at the maturity stage of rice. Furthermore, the addition of calcium dihydrogen phosphate significantly enhanced soil microbial biomass by 133% during the jointing stage and by 154% at the maturity stage. An increase in the fungi-to-bacteria (F/B) ratio was also observed, alongside improvements in the microbial community structure, contributing to greater soil ecosystem sustainability. Notably, the most favorable outcomes were achieved with a P : Pb molar ratio of 2 : 1, leading to effective reduction in heavy metal activity, promotion of microbial growth, and mitigation of heavy metal accumulation in plants."
"C1 [Ye, Chenshuai; Zhou, Qingwei; Wu, Weihong; Jin, Meiqing; Fu, Li] Hangzhou Dianzi Univ, Coll Mat & Environm Engn, Hangzhou 310018, Peoples R China."
"   [Pan, Xingchen] Zhejiang Super Dimens Semicond Co LTD, Huzhou 313309, Peoples R China."
"   [Feng, Haopeng] Hangzhou Youyuan Environm Protect Technol Co, Hangzhou 310000, Peoples R China."
"   [Ji, Weiying] Zhejiang Prov Cultivated Land Qual & Fertilizer A, Hangzhou 310020, Peoples R China."
"   [Zhan, Lichuan] Shengzhou Agr Technol Extens Ctr, Shaoxing 312400, Peoples R China."
C3 Hangzhou Dianzi University
"RP Zhou, QW (corresponding author), Hangzhou Dianzi Univ, Coll Mat & Environm Engn, Hangzhou 310018, Peoples R China.; Zhan, LC (corresponding author), Shengzhou Agr Technol Extens Ctr, Shaoxing 312400, Peoples R China."
EM zhouqw@hdu.edu.cn; 25051652@qq.com
"RI Feng, Haopeng/ABE-2311-2021; Wu, Weihong/A-3516-2019"
"OI ye, chenshuai/0009-0001-3495-9397"
"FU National Science Foundation of China [42173073, 42277017]; The ""Pioneer"""
"   and ""Leading Goose"" RAMP;D Programs of Zhejiang [2022C02022]; Ningbo"
   Science and Technique Plan [2022S110]
"FX This work was supported by National Science Foundation of China, grant"
"   nos. 42173073 and 42277017, ""Pioneer"" and ""Leading Goose"" R&D Programs"
"   of Zhejiang, grant no. 2022C02022, and Ningbo Science and Technique"
"   Plan, project no. 2022S110."
"CR Ahmad M, 2018, CHEMOSPHERE, V194, P327, DOI 10.1016/j.chemosphere.2017.11.156"
"   Barra Caracciolo A., 2021, RHIZOSPHERE MICROBIA, V9, P1462"
"   Beattie RE, 2018, SOIL BIOL BIOCHEM, V126, P57, DOI 10.1016/j.soilbio.2018.08.011"
"   Borggaard OK, 2019, GEODERMA, V343, P235, DOI 10.1016/j.geoderma.2019.02.041"
"   Chen T, 2018, ENVIRON SCI POLLUT R, V25, P36702, DOI 10.1007/s11356-018-3493-x"
"   Ferri M, 2019, APPL SURF SCI, V475, P397, DOI 10.1016/j.apsusc.2018.12.264"
"   Ge QL, 2022, CHEMOSPHERE, V291, DOI 10.1016/j.chemosphere.2021.132835"
"   Gupta Amrita, 2022, Int J Environ Res Public Health, V19, DOI 10.3390/ijerph19053141"
"   Hao XL, 2021, COMPUT STRUCT BIOTEC, V19, P94, DOI 10.1016/j.csbj.2020.12.006"
"   Huang HL, 2021, J HAZARD MATER, V401, DOI 10.1016/j.jhazmat.2020.123393"
"   Huang HL, 2024, ENVIRON RES, V251, DOI 10.1016/j.envres.2024.118389"
"   Kastury F, 2019, ENVIRON INT, V131, DOI 10.1016/j.envint.2019.104967"
"   Lal Rattan, 2017, Current Sustainable/Renewable Energy Reports, V4, P117, DOI 10.1007/s40518-017-0082-4"
"   Lei C, 2020, ECOTOX ENVIRON SAFE, V206, DOI 10.1016/j.ecoenv.2020.111409"
"   Li Q, 2022, SCI TOTAL ENVIRON, V825, DOI 10.1016/j.scitotenv.2022.153862"
"   Liang SX, 2018, B ENVIRON CONTAM TOX, V101, P250, DOI 10.1007/s00128-018-2390-9"
"   Liang XQ, 2018, LAND DEGRAD DEV, V29, P2189, DOI 10.1002/ldr.2851"
"   Liu CJ, 2022, J HAZARD MATER, V431, DOI 10.1016/j.jhazmat.2022.128546"
"   Liu HY, 2021, J SOIL SEDIMENT, V21, P3057, DOI 10.1007/s11368-021-03005-x"
"   Liu L, 2019, J ENVIRON MANAGE, V250, DOI 10.1016/j.jenvman.2019.109525"
"   Liu YY, 2024, EURASIAN SOIL SCI+, V57, P446, DOI 10.1134/S1064229323602640"
"   Lwin CS, 2018, SOIL SCI PLANT NUTR, V64, P156, DOI 10.1080/00380768.2018.1440938"
"   Mishra J, 2017, FRONT MICROBIOL, V8, DOI 10.3389/fmicb.2017.01706"
"   Natasha N, 2022, SCI TOTAL ENVIRON, V808, DOI 10.1016/j.scitotenv.2021.152024"
"   Nishimura Ibuki, 2021, Japanese Geotechnical Society Special Publication, V9, P319, DOI [10.3208/jgssp.v09.cpeg017, 10.3208/jgssp.v09.cpeg017]"
"   Palit K, 2022, ENVIRON SCI POLLUT R, V29, P32467, DOI 10.1007/s11356-022-19048-7"
"   Raj A., 2019, SUSTAINABLE AGR FORE"
"   Ren BZ, 2020, J CONTAM HYDROL, V232, DOI 10.1016/j.jconhyd.2020.103605"
"   Sun T, 2021, CHEMOSPHERE, V280, DOI 10.1016/j.chemosphere.2021.130606"
"   Tahervand S, 2017, J GEOCHEM EXPLOR, V181, P148, DOI 10.1016/j.gexplo.2017.07.005"
"   Tan ZX, 2020, J HAZARD MATER, V384, DOI 10.1016/j.jhazmat.2019.121370"
"   Ubuza LJA, 2020, ENVIRON ENG RES, V25, P977, DOI 10.4491/eer.2019.258"
"   Wang JC, 2020, ECOTOX ENVIRON SAFE, V189, DOI 10.1016/j.ecoenv.2019.109997"
"   Wei W, 2021, CHEMOSPHERE, V285, DOI 10.1016/j.chemosphere.2021.131411"
"   Xu DM, 2021, J CLEAN PROD, V321, DOI 10.1016/j.jclepro.2021.128730"
"   Xu DM, 2017, ENVIRON SCI POLLUT R, V24, P24387, DOI 10.1007/s11356-017-0052-9"
"   YAQOOB AA, 2021, FUEL CELLS, V13, DOI DOI 10.3390/POLYM13010135"
"   Yu M., 2023, RESPONSE SOIL ENV MI, V15, P1986"
"   Yu XJ, 2024, SCI TOTAL ENVIRON, V926, DOI 10.1016/j.scitotenv.2024.171614"
"   Yuhu Luo, 2021, IOP Conference Series: Earth and Environmental Science, V769, DOI [10.1088/1755-1315/769/2/022032, 10.1088/1755-1315/769/2/022032]"
"   Zhang H, 2021, ENVIRON POLLUT, V285, DOI 10.1016/j.envpol.2021.117459"
"   Zhang JR, 2020, BIOELECTROCHEMISTRY, V136, DOI 10.1016/j.bioelechem.2020.107596"
NR 42
TC 0
Z9 0
U1 6
U2 13
PU PLEIADES PUBLISHING LTD
PI TORTOLA
"PA TROPIC ISLE BLDG, PO BOX 3331. ROAD TOWN, TORTOLA, BRITISH VIRGIN ISL"

SN 1064-2293
EI 1556-195X
J9 EURASIAN SOIL SCI+
JI Eurasian Soil Sci.
PD APR 18
PY 2025
VL 58
IS 4
AR 51
DI 10.1134/S1064229324602774
PG 8
WC Soil Science
WE Science Citation Index Expanded (SCI-EXPANDED)
SC Agriculture
GA 1QE0Z
UT WOS:001471024900007
DA 2026-04-06
ER

PT J
"AU Tian, ZJ"
"   Li, GW"
"   Tang, WZ"
"   Zhu, QH"
"   Li, XG"
"   Du, CL"
"   Li, CL"
"   Li, JX"
"   Zhao, C"
"   Zhang, LY"
"AF Tian, Zhenjun"
"   Li, Guowen"
"   Tang, Wenzhong"
"   Zhu, Qiuheng"
"   Li, Xiaoguang"
"   Du, Caili"
"   Li, Caole"
"   Li, Jiaxi"
"   Zhao, Chen"
"   Zhang, Lieyu"
TI Role of Sedum alfredii and soil microbes in the
   remediation of ultra-high content heavy metals contaminated soil
SO AGRICULTURE ECOSYSTEMS & ENVIRONMENT
LA English
DT Article
DE Soil health; Phytoremediation; Sedum alfredii; Soil microbes;
   Patescibacteria
ID POLLUTION; PHYTOREMEDIATION; PHYTOEXTRACTION; BACTERIA; CHINA; RISK;
   ACCUMULATION; REMOVAL; CADMIUM; RIVER
"AB Ultra-high content of heavy metals (UHHMs) poses a threat to soil health and agricultural products safety, in turn causing harm to human health through the food chain. Phytoremediation has proven to be an effective technique for restoration of soil contaminated with a single or several conventional content of heavy metals, whereas the effectiveness of phytoremediation for UHHMs-contaminated soil is unclear. In this study, we investigated the effectiveness of phytoremediation for UHHMs-contaminated soil and the role of soil microbes in the process of phytoremediation. Results showed that the Sedum alfredii grew well in UHHMs-contaminated soil, although the shoot height, biomass, and accumulation of heavy metals were slightly reduced due to the stress of UHHMs. Soil microbiome analysis demonstrated that both the number and diversity of microbes decreased in UHHMs-contaminated soil. However, the dominant species Patescibacteria proliferated to varying degrees under the exposure of various UHHMs. Patescibacteria is closely related to the functional genes regarding transport of various heavy metals. Given that Patescibacteria exhibits a strong migration ability in soil, it is likely involved in the transport of various heavy metals from the bulk soil to the rhizosphere. This enhanced the availability of heavy metals in the rhizosphere, which in turn improved the phytoremediation efficiency of the Sedum alfredii. This was further confirmed by structural equation modeling analysis and microbial phenotypes prediction. Findings suggest that using Sedum alfredii to remedy UHHMs-contaminated soil is feasible owing to the facili-tation by soil microbes."
"C1 [Tian, Zhenjun; Li, Guowen; Zhu, Qiuheng; Li, Xiaoguang; Du, Caili; Li, Caole; Li, Jiaxi; Zhao, Chen; Zhang, Lieyu] Chinese Res Inst Environm Sci, State Key Lab Environm Criteria & Risk Assessment, Beijing 100012, Peoples R China."
"   [Tian, Zhenjun; Zhu, Qiuheng] Beijing Normal Univ, Coll Water Sci, Beijing 100875, Peoples R China."
"   [Tang, Wenzhong] Chinese Acad Sci, Res Ctr Ecoenvironm Sci, State Key Lab Environm Aquat Chem, Beijing 100085, Peoples R China."
"   [Du, Caili] Tongji Univ, Coll Environm Sci & Engn, Shanghai 200092, Peoples R China."
C3 Chinese Research Academy of Environmental Sciences; Beijing Normal
   University; Chinese Academy of Sciences; Research Center for
"   Eco-Environmental Sciences (RCEES), CAS; Tongji University"
"RP Zhang, LY (corresponding author), Chinese Res Inst Environm Sci, State Key Lab Environm Criteria & Risk Assessment, Beijing 100012, Peoples R China."
EM zhanglieyu@163.com
"RI Tang, Wenzhong/AAM-6971-2020; Tian, Zhenjun/HTP-4121-2023; Zhu,"
   Qiuheng/AAR-5235-2020
"OI Tang, Wenzhong/0000-0001-8347-8592; Tian, Zhenjun/0000-0003-4047-9194;"
"   Zhu, Qiuheng/0000-0002-5771-9513"
FU Central-level Public Welfare Research Institute Basic Research
   Operations Special Project of Chinese Research Academy of Environmental

Sciences [2019YSKY-019]; National Key Research and Development Program of China [2020YFC1807904]
FX Acknowledgments We would like to thank the Central-level Public Welfare Research Institute Basic Research Operations Special Project of Chinese Research Academy of Environmental Sciences (Grant No. 2019YSKY-019) and the National Key Research and Development Program of China (Grant No. 2020YFC1807904) for the financial supports of this work.
"CR Ashraf S, 2019, ECOTOX ENVIRON SAFE, V174, P714, DOI 10.1016/j.ecoenv.2019.02.068"
"   Berni R, 2019, ENVIRON EXP BOT, V161, P98, DOI 10.1016/j.envexpbot.2018.10.017"
"   Chaudhari NM, 2021, ENVIRON MICROBIOME, V16, DOI 10.1186/s40793-021-00395-w"
"   Chen L, 2022, CHEMOSPHERE, V293, DOI 10.1016/j.chemosphere.2022.133577"
"   Chen XY, 2021, SCI TOTAL ENVIRON, V785, DOI 10.1016/j.scitotenv.2021.147180"
"   Deng DM, 2008, J INTEGR PLANT BIOL, V50, P691, DOI 10.1111/j.1744-7909.2008.00669.x"
"   Douglas GM, 2020, NAT BIOTECHNOL, V38, P685, DOI 10.1038/s41587-020-0548-6"
"   Dyla M, 2019, BIOCHEM SOC T, V47, P1247, DOI 10.1042/BST20190124"
"   Fan Yi, 2016, Ecological Processes, V5, P19, DOI 10.1186/s13717-016-0063-3"
"   Gasiorek M, 2017, CHEMOSPHERE, V179, P148, DOI 10.1016/j.chemosphere.2017.03.106"
"   He C, 2021, NAT MICROBIOL, V6, P354, DOI 10.1038/s41564-020-00840-5"
"   He GD, 2020, GENES-BASEL, V11, DOI 10.3390/genes11111269"
"   Herrmann M, 2019, FRONT MICROBIOL, V10, DOI 10.3389/fmicb.2019.01407"
"   Hou DD, 2018, APPL ENVIRON MICROB, V84, DOI [10.1128/AEM.02797-17, 10.1128/aem.02797-17]"
"   Hou SN, 2019, ENVIRON INT, V128, P430, DOI 10.1016/j.envint.2019.04.046"
"   Hu BF, 2017, INT J ENV RES PUB HE, V14, DOI 10.3390/ijerph14091042"
"   Hu JL, 2013, CHEMOSPHERE, V93, P1359, DOI 10.1016/j.chemosphere.2013.07.089"
"   Hu PJ, 2019, AGR ECOSYST ENVIRON, V286, DOI 10.1016/j.agee.2019.106651"
"   Ji ZH, 2022, CHEMOSPHERE, V286, DOI 10.1016/j.chemosphere.2021.131564"
"   Khan KS, 2016, GEODERMA, V271, P115, DOI 10.1016/j.geoderma.2016.02.019"
"   Khanam R, 2020, SCI TOTAL ENVIRON, V699, DOI 10.1016/j.scitotenv.2019.134330"
"   Lemos LN, 2019, MOL ECOL, V28, P4259, DOI 10.1111/mec.15208"
"   Lemos LN, 2020, ENV MICROBIOL REP, V12, P651, DOI 10.1111/1758-2229.12880"
"   Li WC, 2012, J HAZARD MATER, V209, P421, DOI 10.1016/j.jhazmat.2012.01.055"
"   Li WC, 2010, J SOIL SEDIMENT, V10, P313, DOI 10.1007/s11368-009-0165-y"
"   Lin ZW, 2020, J SOIL SEDIMENT, V20, P320, DOI 10.1007/s11368-019-02405-4"
"   Liu J, 2021, AGR ECOSYST ENVIRON, V321, DOI 10.1016/j.agee.2021.107632"
"   Ma Y, 2013, CHEMOSPHERE, V93, P1386, DOI 10.1016/j.chemosphere.2013.06.077"
"   Mahar A, 2016, ECOTOX ENVIRON SAFE, V126, P111, DOI 10.1016/j.ecoenv.2015.12.023"
"   Meharg AA, 2003, ENVIRON SCI TECHNOL, V37, P229, DOI 10.1021/es0259842"
"   Mishra J, 2017, FRONT MICROBIOL, V8, DOI 10.3389/fmicb.2017.01706"
"   Nayfach S, 2021, NAT BIOTECHNOL, V39, P499, DOI 10.1038/s41587-020-0718-6"
"   Pires C, 2017, GEOMICROBIOL J, V34, P760, DOI 10.1080/01490451.2016.1261968"
"   S??nchez-Osuna M, 2017, ENVIRON MICROBIOL, V19, P3465, DOI 10.1111/1462-2920.13826"
"   Sessitsch A, 2013, SOIL BIOL BIOCHEM, V60, P182, DOI 10.1016/j.soilbio.2013.01.012"
"   Shah V, 2020, ENVIRON TECHNOL INNO, V18, DOI 10.1016/j.eti.2020.100774"
"   Szeri F, 2021, INT J MOL SCI, V22, DOI 10.3390/ijms22136910"
"   Tang WZ, 2017, ENVIRON TOXICOL CHEM, V36, P1149, DOI 10.1002/etc.3650"
"   Tang WZ, 2014, PLOS ONE, V9, DOI 10.1371/journal.pone.0108996"
"   Tian RM, 2020, MICROBIOME, V8, DOI 10.1186/s40168-020-00825-w"
"   Tian XS, 2013, ENVIRON EARTH SCI, V69, P2129, DOI 10.1007/s12665-012-2042-z"
"   Wan XM, 2016, SCI TOTAL ENVIRON, V563, P796, DOI 10.1016/j.scitotenv.2015.12.080"
"   Wang L, 2021, J HAZARD MATER, V413, DOI 10.1016/j.jhazmat.2021.125427"
"   Ward T, 2017, bioRxiv, DOI [10.1101/133462, DOI 10.1101/133462, 10.1101/133462]"
"   Xv LL, 2020, ENVIRON POLLUT, V263, DOI 10.1016/j.envpol.2020.114401"
"   Yan A, 2020, FRONT PLANT SCI, V11, DOI 10.3389/fpls.2020.00359"
"   Yang QQ, 2018, SCI TOTAL ENVIRON, V642, P690, DOI 10.1016/j.scitotenv.2018.06.068"
"   Yang WH, 2014, J SOIL SEDIMENT, V14, P1385, DOI 10.1007/s11368-014-0875-7"
"   Yang XE, 2006, PLANT SOIL, V284, P109, DOI 10.1007/s11104-006-0033-0"
"   Zeng HA, 2009, PEDOSPHERE, V19, P562, DOI 10.1016/S1002-0160(09)60150-8"
NR 50
TC 49
Z9 55
U1 9
U2 159
PU ELSEVIER
PI AMSTERDAM
"PA RADARWEG 29, 1043 NX AMSTERDAM, NETHERLANDS"
SN 0167-8809
EI 1873-2305
J9 AGR ECOSYST ENVIRON
JI Agric. Ecosyst. Environ.
PD NOV 1
PY 2022
VL 339
AR 108090
DI 10.1016/j.agee.2022.108090
EA JUL 2022
PG 11
"WC Agriculture, Multidisciplinary; Ecology; Environmental Sciences"
WE Science Citation Index Expanded (SCI-EXPANDED)
SC Agriculture; Environmental Sciences & Ecology
GA 5J6WF
UT WOS:000869180700005
DA 2026-04-06
ER

PT J
"AU Kou, B"
"   Yuan, Y"
"   Zhu, XL"
"   Ke, YX"
"   Wang, H"
"   Yu, TQ"
"   Tan, WB"
"AF Kou, Bing"
"   Yuan, Ying"
"   Zhu, Xiaoli"
"   Ke, Yuxin"
"   Wang, Hui"
"   Yu, Tingqiao"
"   Tan, Wenbing"
TI Effect of soil organic matter-mediated electron transfer on heavy metal
   remediation: Current status and perspectives
SO SCIENCE OF THE TOTAL ENVIRONMENT
LA English
DT Article
DE Soil organic matter; Microorganism; Electron transfer; Heavy metal
   remediation; Reduction
ID DISSIMILATORY IRON REDUCTION; HUMIC SUBSTANCES; SHEWANELLA-ONEIDENSIS;
   MICROBIAL REDUCTION; HEXAVALENT CHROMIUM; MERCURY REDUCTION;
   GEOBACTER-SULFURREDUCENS; TRANSFER CAPACITY;
"   ANTHRAQUINONE-2,6-DISULFONATE AQDS; TRANSPORT ELECTRONS"
"AB Soil contamination by heavy metals poses major risks to human health and the environment. Given the current status of heavy metal pollution, many remediation techniques have been tested at laboratory and contaminated sites. The effects of soil organic matter-mediated electron transfer on heavy metal remediation have not been adequately studied, and the key mechanisms underlying this process have not yet been elucidated. In this review, microbial extracellular electron transfer pathways, organic matter electron transfer for heavy metal reduction, and the factors affecting these processes were discussed to enhance our understanding of heavy metal pollution. It was found that microbial extracellular electrons delivered by electron shuttles have the longest distance among the three electron transfer pathways, and the application of exogenous electron shuttles lays the foundation for efficient and persistent remediation of heavy metals. The organic matter-mediated electron transfer process, wherein organic matter acts as an electron shuttle, promotes the conversion of high valence state metal ions, such as Cr(VI), Hg(II), and U(VI), into less toxic and morphologically stable forms, which inhibits their mobility and bioavailability. Soil type, organic matter structural and content, heavy metal concentrations, and environmental factors (e.g., pH, redox potential, oxygen conditions, and temperature) all influence organic matter-mediated electron transfer processes and bioremediation of heavy metals. Organic matter can more effectively mediate electron transfer for heavy metal remediation under anaerobic conditions, as well as when the heavy metal"
"C1 [Kou, Bing; Zhu, Xiaoli; Ke, Yuxin] Northwest Univ, Coll Urban & Environm Sci, Xian 710127, Peoples R China."
"   [Kou, Bing; Yuan, Ying; Wang, Hui; Tan, Wenbing] Chinese Res Inst Environm Sci, State Environm Protect Key Lab Simulat & Control, Beijing 100012, Peoples R China."
"   [Yu, Tingqiao] Beijing Vocat Coll Agr, Dept Hort, Beijing 102442, Peoples R China."
C3 Northwest University Xi'an; Chinese Research Academy of Environmental
   Sciences
"RP Zhu, XL (corresponding author), Northwest Univ, Coll Urban & Environm Sci, Xian 710127, Peoples R China.; Yuan, Y (corresponding author), Chinese Res Inst Environm Sci, State Environm Protect Key Lab Simulat & Control, Beijing 100012, Peoples R China."
EM yuanying19880214@126.com; xiaolizhu@nwu.edu.cn
"OI Tan, Wenbing/0000-0001-9896-7604"
FU National Key Research and Devel- opment Program of China
   [2021YFC1808902]; Key Research and Development Program of Shaanxi
   Province [2020ZDLNY06- 06]; Shandong Provincial Natural Science
"   Foundation, China [2020CXGC011403]; Northwest University Graduate"
   Innovation Project [363052204001]
FX This work was supported by the National Key Research and Devel- opment
"   Program of China (2021YFC1808902) , Key Research and Development Program"
"   of Shaanxi Province (Grant No. 2020ZDLNY06- 06) , Shandong Provincial"
"   Natural Science Foundation, China (Grant No. 2020CXGC011403) , and"
   Northwest University Graduate Innovation Project (Grant No.
   363052204001) .
"CR Aeschbacher M., 2011, Electrochemical Redox Characterization of Humic Substances"
"   Aeschbacher M, 2012, ENVIRON SCI TECHNOL, V46, P4916, DOI 10.1021/es300039h"
"   ALBERTS JJ, 1974, SCIENCE, V184, P895, DOI 10.1126/science.184.4139.895"
"   Argyropoulos DS, 1998, J AGR FOOD CHEM, V46, P4628, DOI 10.1021/jf9804802"
"   Azeez NA, 2021, CHEMOSPHERE, V266, DOI 10.1016/j.chemosphere.2020.129204"
"   Bae S, 2013, GEOCHIM COSMOCHIM AC, V114, P144, DOI 10.1016/j.gca.2013.03.041"
"   Bai Y, 2020, ENVIRON SCI TECHNOL, V54, P4131, DOI 10.1021/acs.est.9b07134"
"   Bauer I, 2009, ENVIRON SCI TECHNOL, V43, P4902, DOI 10.1021/es900179s"
"   Bauer M, 2007, ENVIRON SCI TECHNOL, V41, P139, DOI 10.1021/es061323j"
"   Beblawy S, 2018, MOL MICROBIOL, V109, P571, DOI 10.1111/mmi.14067"
"   Bonneville S, 2004, CHEM GEOL, V212, P255, DOI 10.1016/j.chemgeo.2004.08.015"
"   Borggaard OK, 2019, GEODERMA, V343, P235, DOI 10.1016/j.geoderma.2019.02.041"
"   Bretschger O, 2007, APPL ENVIRON MICROB, V73, P7003, DOI 10.1128/AEM.01087-07"
"   Brutinel ED, 2012, APPL MICROBIOL BIOT, V93, P41, DOI 10.1007/s00253-011-3653-0"
"   Cai JJ, 2021, J ENERGY INST, V96, P140, DOI 10.1016/j.joei.2021.03.005"
"   Cai XX, 2020, J HAZARD MATER, V391, DOI 10.1016/j.jhazmat.2020.122213"
"   Cervantes FJ, 2003, BIOTECHNOL LETT, V25, P39, DOI 10.1023/A:1021721929815"
"   Cervantes FJ, 2002, ENVIRON MICROBIOL, V4, P51, DOI 10.1046/j.1462-2920.2002.00258.x"
"   Che LN, 2022, CATENA, V208, DOI 10.1016/j.catena.2021.105721"

" Chen H, 2011, PHYS CHEM EARTH, V36, P451, DOI 10.1016/j.pce.2010.05.002"
" Chen J, 2003, SCI TOTAL ENVIRON, V307, P167, DOI 10.1016/S0048-9697(02)00538-7"
" Childers SE, 2002, NATURE, V416, P767, DOI 10.1038/416767a"
" Coursolle D, 2010, J BACTERIOL, V192, P467, DOI 10.1128/JB.00925-09"
" Cubadda F, 2017, SCI TOTAL ENVIRON, V579, P1228, DOI 10.1016/j.scitotenv.2016.11.108"
" Cutting RS, 2009, GEOCHIM COSMOCHIM AC, V73, P4004, DOI 10.1016/j.gca.2009.04.009"
" de Graef MR, 1999, J BACTERIOL, V181, P2351, DOI 10.1128/JB.181.8.2351-2357.1999"
" Deng BL, 1996, ENVIRON SCI TECHNOL, V30, P463, DOI 10.1021/es950156c"
" Dietrich LEP, 2008, SCIENCE, V321, P1203, DOI 10.1126/science.1160619"
" EPC, 2014, National soil pollution investigation government bulletins"
" Filipovic L, 2018, ECOTOX ENVIRON SAFE, V147, P824, DOI 10.1016/j.ecoenv.2017.09.041"
" Fonseca BM, 2013, BIOCHEM J, V449, P101, DOI 10.1042/BJ20121467"
" Fredrickson JK, 2000, APPL ENVIRON MICROB, V66, P2006, DOI 10.1128/AEM.66.5.2006-2011.2000"
" Fredrickson JK, 2000, GEOCHIM COSMOCHIM AC, V64, P3085, DOI 10.1016/S0016-7037(00)00397-5"
" Fulda B, 2013, ENVIRON SCI TECHNOL, V47, P12775, DOI 10.1021/es401997d"
" Glasser NR, 2017, ANNU REV MICROBIOL, V71, P731, DOI 10.1146/annurev-micro-090816-093913"
" Gong YY, 2018, WATER RES, V147, P440, DOI 10.1016/j.watres.2018.10.024"
" Gorby YA, 2006, P NATL ACAD SCI USA, V103, P11358, DOI 10.1073/pnas.0604517103"
" Gu BH, 2011, P NATL ACAD SCI USA, V108, P1479, DOI 10.1073/pnas.1008747108"
" Gu BH, 2005, ENVIRON SCI TECHNOL, V39, P4841, DOI 10.1021/es050011y"
" Gu BH, 2005, ENVIRON SCI TECHNOL, V39, P5268, DOI 10.1021/es050350r"
" Gu BH, 2003, GEOCHIM COSMOCHIM AC, V67, P3575, DOI 10.1016/S0016-7037(03)00162-5"
" Gustave W, 2019, CHEMOSPHERE, V237, DOI 10.1016/j.chemosphere.2019.124459"
" Han JC, 2017, ENVIRON SCI TECH LET, V4, P350, DOI 10.1021/acs.estlett.7b00227"
" Han RX, 2022, ENVIRON SCI TECHNOL, V56, P16419, DOI 10.1021/acs.est.2c03799"
" Hartshorne RS, 2009, P NATL ACAD SCI USA, V106, P22169, DOI 10.1073/pnas.0900086106"
" He XS, 2014, J HAZARD MATER, V268, P256, DOI 10.1016/j.jhazmat.2014.01.030"
" Hernandez ME, 2001, CELL MOL LIFE SCI, V58, P1562, DOI 10.1007/PL00000796"
" Hong YG, 2012, APPL MICROBIOL BIOT, V93, P2661, DOI 10.1007/s00253-011-3577-8"
" Hu HY, 2013, ENVIRON SCI TECHNOL, V47, P10922, DOI 10.1021/es400527m"
" Hu SW, 2021, J COLLOID INTERF SCI, V583, P544, DOI 10.1016/j.jcis.2020.09.074"
" Hu TT, 2023, J BIOSCI BIOENG, V135, P238, DOI 10.1016/j.jbiosc.2022.12.003"
" Huang WF, 2021, SCI TOTAL ENVIRON, V755, DOI 10.1016/j.scitotenv.2020.142687"
" Huang XN, 2019, ENVIRON INT, V129, P86, DOI 10.1016/j.envint.2019.05.016"
" Jeyasingh J, 2005, J HAZARD MATER, V118, P113, DOI 10.1016/j.jhazmat.2004.10.003"
" Jiang B, 2019, J HAZARD MATER, V365, P205, DOI 10.1016/j.jhazmat.2018.10.070"
" Jiang J, 2008, ENVIRON SCI TECHNOL, V42, P3563, DOI 10.1021/es7023803"
" [?????? Jiang Jie], 2023, [?????????????€????, Microbiology China], V50, P4694"
" ?????????, 2014, [The Journal of Korean Society of Soil and Groundwater Environment, ??€?????????????????], V19, P16"
" Kang Y, 2022, CHEM ENG J, V439, DOI 10.1016/j.cej.2022.135742"
" Kappler A, 2004, FEMS MICROBIOL ECOL, V47, P85, DOI 10.1016/S0168-6496(03)00245-9"
" Kavita B, 2012, 3 BIOTECH, V2, P79, DOI 10.1007/s13205-011-0038-0"
" Kavita B, 2015, DESALIN WATER TREAT, V53, P221, DOI 10.1080/19443994.2013.838521"
" Keller JK, 2013, ECOSPHERE, V4, DOI 10.1890/ES12-00382.1"
" Kerscher Stefan, 2008, V45, P185, DOI 10.1007/400_2007_028"
" Kl??pfel L, 2014, NAT GEOSCI, V7, P195, DOI 10.1038/NGEO2084"
" Klupfel L., 2009, Term Paper FS, P1"
" Kopecky M, 2021, SOIL TILL RES, V207, DOI 10.1016/j.still.2020.104865"
" Kotloski NJ, 2013, MBIO, V4, DOI 10.1128/mBio.00553-12"
" Kumar A, 2017, NAT REV CHEM, V1, DOI 10.1038/s41570-017-0024"
" Kumar M, 2021, ENVIRON SUSTAIN IND, V12, DOI 10.1016/j.indic.2021.100155"
" Kumarathilaka P, 2018, WATER RES, V140, P403, DOI 10.1016/j.watres.2018.04.034"
" Lan S, 2019, GEOCHIM COSMOCHIM AC, V247, P175, DOI 10.1016/j.gca.2018.12.018"
" Lee S, 2018, CHEMOSPHERE, V194, P515, DOI 10.1016/j.chemosphere.2017.12.007"
" [????????? LI Haoran], 2007, [????????????????????????. ???????????????, Journal of China University of Mining & Technology], V36, P680"
" Li KM, 2023, J ENVIRON MANAGE, V345, DOI 10.1016/j.jenvman.2023.118596"
" Li M, 2022, SCI TOTAL ENVIRON, V819, DOI 10.1016/j.scitotenv.2022.153157"
" Li T, 2020, ENVIRON SCI TECHNOL, V54, P8658, DOI 10.1021/acs.est.9b07473"
" Li YR, 2009, J HAZARD MATER, V161, P657, DOI 10.1016/j.jhazmat.2008.04.007"
" Li YH, 2021, J ENVIRON CHEM ENG, V9, DOI 10.1016/j.jece.2021.106823"
" Light SH, 2018, NATURE, V562, P140, DOI 10.1038/s41586-018-0498-z"
" Liu TX, 2016, SCI REP-UK, V6, DOI 10.1038/srep29592"
" Liu W, 2019, FRONT MICROBIOL, V10, DOI 10.3389/fmicb.2019.00464"
" Liu YR, 2023, SCI TOTAL ENVIRON, V875, DOI 10.1016/j.scitotenv.2023.162700"
" Liu YH, 2022, J POWER SOURCES, V527, DOI 10.1016/j.jpowsour.2022.231212"
" Logan BE, 2019, NAT REV MICROBIOL, V17, P307, DOI 10.1038/s41579-019-0173-x"
" Lovley DR, 2011, ENERG ENVIRON SCI, V4, P4896, DOI 10.1039/c1ee02229f"
" LOVLEY DR, 1993, ANNU REV MICROBIOL, V47, P263, DOI 10.1146/annurev.micro.47.1.263"
" Lovley DR, 1999, APPL ENVIRON MICROB, V65, P4252"
" LOVLEY DR, 1991, NATURE, V350, P413, DOI 10.1038/350413a0"
" LOVLEY DR, 1994, APPL ENVIRON MICROB, V60, P726, DOI 10.1128/AEM.60.2.726-728.1994"
" Lovley DR, 1996, NATURE, V382, P445, DOI 10.1038/382445a0"
" Lu T, 2022, J CLEAN PROD, V365, DOI 10.1016/j.jclepro.2022.132794"
" Luo WQ, 2023, J HAZARD MATER, V446, DOI 10.1016/j.jhazmat.2022.130655"
" Lv JT, 2018, ENVIRON SCI TECHNOL, V52, P11660, DOI 10.1021/acs.est.8b03940"
" Malvankar NS, 2015, MBIO, V6, DOI 10.1128/mBio.00084-15"
" Malvankar NS, 2014, CURR OPIN BIOTECH, V27, P88, DOI 10.1016/j.copbio.2013.12.003"
" Malvankar NS, 2011, NAT NANOTECHNOL, V6, P573, DOI [10.1038/nnano.2011.119, 10.1038/NNANO.2011.119]"
" Markiewicz M, 2018, APPL SOIL ECOL, V123, P509, DOI 10.1016/j.apsoil.2017.04.022"
" Marritt SJ, 2012, BIOCHEM J, V444, P465, DOI 10.1042/BJ20120197"
" Marsili E, 2008, P NATL ACAD SCI USA, V105, P3968, DOI 10.1073/pnas.0710525105"
" Meng Y, 2018, SCI TOTAL ENVIRON, V640, P591, DOI 10.1016/j.scitotenv.2018.05.331"

"   Merino C, 2021, SCI TOTAL ENVIRON, V761, DOI 10.1016/j.scitotenv.2020.143194"
"   Meunier L, 2005, AQUAT SCI, V67, P292, DOI 10.1007/s00027-005-0779-0"
"   Mich??lkov?? Z, 2014, J ENVIRON MANAGE, V146, P226, DOI 10.1016/j.jenvman.2014.08.004"
"   Michelson K, 2017, ENVIRON SCI TECHNOL, V51, P11660, DOI 10.1021/acs.est.7b02531"
"   Moe B, 2016, J ENVIRON SCI, V49, P113, DOI 10.1016/j.jes.2016.10.004"
"   Mohamed A, 2020, CHEMOSPHERE, V247, DOI 10.1016/j.chemosphere.2020.125902"
"   Myers JM, 2004, APPL ENVIRON MICROB, V70, P1405, DOI 10.1128/AEM.70.3.1405-1412.2004"
"   Nurmi JT, 2002, ENVIRON SCI TECHNOL, V36, P617, DOI 10.1021/es0110731"
"   Nyman Jennifer L., 2002, Bioremediation Journal, V6, P39, DOI 10.1080/10889860290777468"
"   Ochoa PA, 2016, CATENA, V140, P31, DOI 10.1016/j.catena.2016.01.011"
"   Okamoto A, 2014, CHEMELECTROCHEM, V1, P1808, DOI 10.1002/celc.201402151"
"   Okamoto A, 2014, ANGEW CHEM INT EDIT, V53, P10988, DOI 10.1002/anie.201407004"
"   Okamoto A, 2014, ENERG ENVIRON SCI, V7, P1357, DOI 10.1039/c3ee43674h"
"   Okamoto A, 2013, P NATL ACAD SCI USA, V110, P7856, DOI 10.1073/pnas.1220823110"
"   Oremland RS, 2003, SCIENCE, V300, P939, DOI 10.1126/science.1081903"
"   Palmer NE, 2006, ENVIRON CHEM, V3, P131, DOI 10.1071/EN05081"
"   Parks JM, 2013, SCIENCE, V339, P1332, DOI 10.1126/science.1230667"
"   Peretyazhko T, 2006, GEODERMA, V137, P140, DOI 10.1016/j.geoderma.2006.08.004"
"   P??rez-Mej??as G, 2022, COORDIN CHEM REV, V450, DOI 10.1016/j.ccr.2021.214233"
"   Pfeffer C, 2012, NATURE, V491, P218, DOI 10.1038/nature11586"
"   Pham AN, 2012, J PHYS CHEM A, V116, P6590, DOI 10.1021/jp300995h"
"   Pirbadian S, 2014, P NATL ACAD SCI USA, V111, P12883, DOI 10.1073/pnas.1410551111"
"   Pirbadian S, 2012, PHYS CHEM CHEM PHYS, V14, P13802, DOI 10.1039/c2cp41185g"
"   Price-Whelan A, 2006, NAT CHEM BIOL, V2, P71, DOI 10.1038/nchembio764"
"   Qiao JT, 2019, ENVIRON SCI TECHNOL, V53, P5034, DOI 10.1021/acs.est.8b06333"
"   Rabaey K, 2005, TRENDS BIOTECHNOL, V23, P291, DOI 10.1016/j.tibtech.2005.04.008"
"   Rabaey K, 2005, ENVIRON SCI TECHNOL, V39, P3401, DOI 10.1021/es048563o"
"   Ratasuk N, 2007, ENVIRON SCI TECHNOL, V41, P7844, DOI 10.1021/es071389u"
"   Reguera G, 2005, NATURE, V435, P1098, DOI 10.1038/nature03661"
"   Rehnlund D, 2022, ISCIENCE, V25, DOI 10.1016/j.isci.2022.103853"
"   Rendon J., 2016, Biochim. Biophys. Acta-Bioenerg., V1857, P50"
"   Rhodes Z, 2021, ISCIENCE, V24, DOI 10.1016/j.isci.2021.103033"
"   Roden EE, 2010, NAT GEOSCI, V3, P417, DOI 10.1038/NGEO870"
"   Rosenbaum MA, 2012, PLOS ONE, V7, DOI 10.1371/journal.pone.0030827"
"   Ross DE, 2009, APPL ENVIRON MICROB, V75, P5218, DOI 10.1128/AEM.00544-09"
"   Rush JE, 2021, GEODERMA, V401, DOI 10.1016/j.geoderma.2021.115303"
"   Sarkar B, 2013, ENVIRON SCI TECHNOL, V47, P13629, DOI 10.1021/es401568k"
"   Sathishkumar K, 2017, ANN MICROBIOL, V67, P91, DOI 10.1007/s13213-016-1240-4"
"   Schuetz B, 2009, APPL ENVIRON MICROB, V75, P7789, DOI 10.1128/AEM.01834-09"
"   Scott DT, 1998, ENVIRON SCI TECHNOL, V32, P2984, DOI 10.1021/es980272q"
"   Shi L, 2016, NAT REV MICROBIOL, V14, P651, DOI 10.1038/nrmicro.2016.93"
"   Shi LA, 2009, ENV MICROBIOL REP, V1, P220, DOI 10.1111/j.1758-2229.2009.00035.x"
"   Shi Z, 2012, ENVIRON SCI TECHNOL, V46, P11644, DOI 10.1021/es301544b"
"   Si YB, 2015, CHEMOSPHERE, V122, P206, DOI 10.1016/j.chemosphere.2014.11.054"
"   Siciliano SD, 2002, ENVIRON SCI TECHNOL, V36, P3064, DOI 10.1021/es010774v"
"   Simoska O, 2019, ACS SENSORS, V4, P170, DOI 10.1021/acssensors.8b01152"
"   SKOGERBOE RK, 1981, ANAL CHEM, V53, P228, DOI 10.1021/ac00225a023"
"   Stevenson F. J., 1982, Humus chemistry, genesis, composition, reactions."
"   Struyk Z, 2001, GEODERMA, V102, P329, DOI 10.1016/S0016-7061(01)00040-4"
"   Stumm W., 1992, Chemistry of the Solid-Water Interface, p309e335"
"   Summers ZM, 2010, SCIENCE, V330, P1413, DOI 10.1126/science.1196526"
"   Sun YY, 2022, FRONT MICROBIOL, V13, DOI 10.3389/fmicb.2022.997732"
"   SUNDA WG, 1994, NATURE, V367, P62, DOI 10.1038/367062a0"
"   SUZUKI T, 1992, J BACTERIOL, V174, P5340, DOI 10.1128/JB.174.16.5340-5345.1992"
"   Suzuki Y, 2010, PHYS CHEM CHEM PHYS, V12, P10081, DOI 10.1039/c0cp00339e"
"   Tabaraki R, 2018, ECOTOX ENVIRON SAFE, V166, P35, DOI 10.1016/j.ecoenv.2018.09.063"
"   Tan WB, 2020, SCI TOTAL ENVIRON, V708, DOI 10.1016/j.scitotenv.2019.134683"
"   Tan WB, 2019, SCI TOTAL ENVIRON, V676, P378, DOI 10.1016/j.scitotenv.2019.04.288"
"   Tan WB, 2019, GEODERMA, V338, P1, DOI 10.1016/j.geoderma.2018.11.037"
"   Tan WB, 2018, SCI TOTAL ENVIRON, V610, P333, DOI 10.1016/j.scitotenv.2017.08.060"
"   Tan WB, 2017, ENVIRON SCI TECHNOL, V51, P3176, DOI 10.1021/acs.est.6b04131"
"   Tongesayi T, 2006, ENVIRON CHEM, V3, P137, DOI 10.1071/EN05095"
"   Tsui MTK, 2019, ENVIRON SCI TECHNOL, V53, P2434, DOI 10.1021/acs.est.8b06053"
"   Urrutia MM, 1999, ENVIRON SCI TECHNOL, V33, P4022, DOI 10.1021/es990447b"
"   Van Brusselen D, 2020, LANCET PLANET HEALTH, V4, pE158, DOI 10.1016/S2542-5196(20)30059-0"
"   Venkataraman A, 2011, ENERG ENVIRON SCI, V4, P4550, DOI 10.1039/c1ee01377g"
"   VISSER SA, 1964, NATURE, V204, P581, DOI 10.1038/204581a0"
"   Voelker BM, 1997, ENVIRON SCI TECHNOL, V31, P1004, DOI 10.1021/es9604018"
"   von Canstein H, 2008, APPL ENVIRON MICROB, V74, P615, DOI 10.1128/AEM.01387-07"
"   Walpen N, 2016, ENVIRON SCI TECHNOL, V50, P6423, DOI 10.1021/acs.est.6b01120"
"   Wang FB, 2019, CELL, V177, P361, DOI 10.1016/j.cell.2019.03.029"
"   Wang JB, 2015, SOIL SEDIMENT CONTAM, V24, P103, DOI [10.1080/15320383.2014.920292, 10.1080/15320383.2016.1085833]"
"   Wang J, 2019, ENVIRON SCI TECHNOL, V53, P2500, DOI 10.1021/acs.est.8b06863"
"   Wang JH, 2021, J HAZARD MATER, V417, DOI 10.1016/j.jhazmat.2021.126090"
"   Wang XB, 2022, BIORESOURCE TECHNOL, V345, DOI 10.1016/j.biortech.2021.126522"
"   Wang Y, 2010, J BACTERIOL, V192, P365, DOI 10.1128/JB.01188-09"
"   Wang YQ, 2022, SCI TOTAL ENVIRON, V807, DOI 10.1016/j.scitotenv.2021.150979"
"   Wittbrodt PR, 1996, ENVIRON SCI TECHNOL, V30, P2470, DOI 10.1021/es950731c"
"   WITTBRODT PR, 1995, ENVIRON SCI TECHNOL, V29, P255, DOI 10.1021/es00001a033"
"   Wolf M, 2009, ENVIRON SCI TECHNOL, V43, P5679, DOI 10.1021/es803647r"
"   Wu C, 2021, J ENVIRON MANAGE, V296, DOI 10.1016/j.jenvman.2021.113185"
"   Xi BD, 2018, RSC ADV, V8, P32588, DOI 10.1039/c8ra04278k"
"   Xia X, 2021, J HAZARD MATER, V401, DOI [10.1016/j.jhazmat.2020.143685, 10.1016/j.jhazmat.2020.123685]"

"   Xiao X, 2019, CHEMOSPHERE, V222, P757, DOI 10.1016/j.chemosphere.2019.01.173"
"   Xie JC, 2018, SCI TOTAL ENVIRON, V610, P1321, DOI 10.1016/j.scitotenv.2017.08.184"
"   Xie JC, 2017, J HAZARD MATER, V339, P347, DOI 10.1016/j.jhazmat.2017.06.054"
"   Yamamura S, 2018, J HAZARD MATER, V342, P571, DOI 10.1016/j.jhazmat.2017.08.071"
"   Yang G, 2022, INT J ENV RES PUB HE, V19, DOI 10.3390/ijerph191912128"
"   Yang WL, 2022, INT J ENV RES PUB HE, V19, DOI 10.3390/ijerph192315650"
"   Yang Y, 2015, ACS SYNTH BIOL, V4, P815, DOI 10.1021/sb500331x"
"   Yuan CL, 2021, J ENVIRON SCI, V104, P204, DOI 10.1016/j.jes.2020.11.016"
"   Yuan CL, 2019, J HAZARD MATER, V378, DOI 10.1016/j.jhazmat.2019.05.065"
"   Yuan Y, 2018, CHEMOSPHERE, V208, P77, DOI 10.1016/j.chemosphere.2018.05.160"
"   Yuan Y, 2018, ENVIRON SCI POLLUT R, V25, P13026, DOI 10.1007/s11356-018-1494-4"
"   Yuan Y, 2017, J HAZARD MATER, V339, P378, DOI 10.1016/j.jhazmat.2017.06.047"
"   [?????? Yuan Ying], 2014, [????????????, Environmental Chemistry], V33, P2048"
"   Zaiets O, 2016, GEODERMA, V272, P83, DOI 10.1016/j.geoderma.2016.03.006"
"   Zhang F, 2022, J MOL LIQ, V351, DOI 10.1016/j.molliq.2022.118622"
"   Zhang HC, 2009, ENVIRON SCI TECHNOL, V43, P1042, DOI 10.1021/es8017072"
"   Zhang J, 2019, SCI TOTAL ENVIRON, V651, P2975, DOI 10.1016/j.scitotenv.2018.10.165"
"   Zhang JR, 2021, ENVIRON RES, V198, DOI 10.1016/j.envres.2021.111217"
"   Zhang JR, 2020, BIOELECTROCHEMISTRY, V136, DOI 10.1016/j.bioelechem.2020.107596"
"   Zhang XY, 2015, PLOS ONE, V10, DOI 10.1371/journal.pone.0135182"
"   Zhang YL, 2019, ECOTOX ENVIRON SAFE, V171, P594, DOI 10.1016/j.ecoenv.2019.01.024"
"   Zhao XY, 2020, ENVIRON INT, V135, DOI 10.1016/j.envint.2019.105380"
"   Zheng W, 2013, ENVIRON SCI TECHNOL, V47, P12827, DOI 10.1021/es402697u"
"   Zheng W, 2012, ENVIRON SCI TECHNOL, V46, P292, DOI 10.1021/es203402p"
"   Zheng Z, 2015, INT BIODETER BIODEGR, V97, P90, DOI 10.1016/j.ibiod.2014.10.017"
"   Zhilin DM, 2004, ENVIRON CHEM LETT, V2, P141, DOI 10.1007/s10311-004-0085-4"
"   Zhou QX, 2023, ECO-ENVIRON HEALTH, V2, P3, DOI 10.1016/j.eehl.2023.01.002"
"   Zhou Z, 2020, ENVIRON SCI TECHNOL, V54, P9445, DOI 10.1021/acs.est.0c03062"
"   Zhu YP, 2019, CHEM ENG J, V357, P75, DOI 10.1016/j.cej.2018.09.061"
"   Zulfiqar U, 2023, PLANTS-BASEL, V12, DOI 10.3390/plants12173147"
NR 214
TC 55
Z9 62
U1 73
U2 488
PU ELSEVIER
PI AMSTERDAM
"PA RADARWEG 29, 1043 NX AMSTERDAM, NETHERLANDS"
SN 0048-9697
EI 1879-1026
J9 SCI TOTAL ENVIRON
JI Sci. Total Environ.
PD MAR 20
PY 2024
VL 917
AR 170451
DI 10.1016/j.scitotenv.2024.170451
EA FEB 2024
PG 14
WC Environmental Sciences
WE Science Citation Index Expanded (SCI-EXPANDED)
SC Environmental Sciences & Ecology
GA KA0F3
UT WOS:001177116300001
PM 38296063
DA 2026-04-06
ER

PT J
"AU Song, LC"
"   Niu, XG"
"   Zhou, B"
"   Xiao, YN"
"   Zou, HT"
"AF Song, Lichao"
"   Niu, Xuguang"
"   Zhou, Bin"
"   Xiao, Yinong"
"   Zou, Hongtao"
TI Application of biochar-immobilized Bacillus sp. KSB7 to enhance the
   phytoremediation of PAHs and heavy metals in a coking plant
SO CHEMOSPHERE
LA English
DT Article
DE Bacterium; Biochar-immobilized; Co -contamination; Kochia scoparia;
   Rhizoremediation; Strain KSB7
ID POLYCYCLIC AROMATIC-HYDROCARBONS; GROWTH; SOIL; PHENANTHRENE;
   DEGRADATION; RHIZOSPHERE; BACTERIA; SUBTILIS; STRESS; PYRENE
"AB The co-existence of heavy metals and polycyclic aromatic hydrocarbons (PAHs) challenges the remediation of polluted soil. This study aimed to investigate whether a combined amendment of biochar-immobilized bacterium (BM) could enhance the phytoremediation of heavy metals and PAHs in co-contaminated soil. The Bacillus sp. KSB7 with the capabilities of plant-growth promotion, metal tolerance, and PAH degradation was immobilized on the peanut shell biochar prepared at 400 degrees C and 600 degrees C (PBM4 and PBM6, respectively). After 90 days, PBM4 treatment increased the removal of PAHs by 94.17% and decreased the

amounts of diethylenetriamine pentaacetic acid-extractable Zn, Pb, Cr, and Cu by 58.46%, 53.42%, 84.94%, and 83.15%, respectively, compared with Kochia scoparia-alone treatment. Meanwhile, PBM4 was more effective in promoting K. scoparia growth and reducing the uptake of co-contaminants. The abundance of Gram-negative PAH-degrader and 1-aminocyclopropane-1-carboxylic deaminase-producing bacteria within rhizosphere soil was significantly improved after PBM4 treatment. Moreover, the relative abundance of the Bacillus genus increased by 0.66 and 2.05 times under PBM4 treatment compared with biochar alone and KSB7, indicating that KSB7 could colonize in the rhizosphere soil of K. scoparia. However, the removal of PAHs and heavy metals after PBM6 and 600 degrees C biochar-alone treatments caused no obvious difference. This study suggested that low-temperature BM-amended plant cultivation would be an effective approach to remove PAHs and heavy metals in co-contaminated soil."
"C1 [Song, Lichao; Niu, Xuguang; Zhou, Bin; Xiao, Yinong; Zou, Hongtao] Shenyang Agr Univ, Coll Land & Environm, Shenyang 110866, Peoples R China."
"   [Song, Lichao; Niu, Xuguang; Zhou, Bin; Xiao, Yinong; Zou, Hongtao] Shenyang Agr Univ, Key Lab Northeast Arable Land Conservat, Minist Agr, Shenyang 110866, Peoples R China."
C3 Shenyang Agricultural University; Shenyang Agricultural University
"RP Zou, HT (corresponding author), Shenyang Agr Univ, Coll Land & Environm, Shenyang 110866, Peoples R China.; Zou, HT (corresponding author), Shenyang Agr Univ, Key Lab Northeast Arable Land Conservat, Minist Agr, Shenyang 110866, Peoples R China."
EM Hongtaozou208@126.com
"RI ???, ??????/AAC-7936-2019"
FU National Natural Science Foundation of China [41703103]; scientific research fund project of the Liaoning Provincial Department of Education [LJKZ0665]
FX This study was financially supported by the National Natural Science Foundation of China (No. 41703103) and the scientific research fund project of the Liaoning Provincial Department of Education (LJKZ0665) .
"CR Ahmad M, 2019, J HAZARD MATER, V380, DOI 10.1016/j.jhazmat.2019.120863"
"   Ali H, 2013, CHEMOSPHERE, V91, P869, DOI 10.1016/j.chemosphere.2013.01.075"
"   Ali M, 2022, ENVIRON POLLUT, V295, DOI 10.1016/j.envpol.2021.118686"
"   Bao HY, 2020, J HAZARD MATER, V385, DOI 10.1016/j.jhazmat.2019.121595"
"   Bouffaud ML, 2018, MICROBIOME, V6, DOI 10.1186/s40168-018-0503-7"
"   Bragato C, 2009, DESALINATION, V246, P35, DOI 10.1016/j.desal.2008.02.036"
"   Cao XF, 2022, J HAZARD MATER, V426, DOI 10.1016/j.jhazmat.2021.128096"
"   C??bron A, 2008, J MICROBIOL METH, V73, P148, DOI 10.1016/j.mimet.2008.01.009"
"   Chen HB, 2020, J ENVIRON MANAGE, V261, DOI 10.1016/j.jenvman.2020.110246"
"   Czarny J, 2020, J HAZARD MATER, V383, DOI 10.1016/j.jhazmat.2019.121168"
"   Dai YY, 2020, ENVIRON INT, V136, DOI 10.1016/j.envint.2019.105421"
"   Gao YZ, 2006, CHEMOSPHERE, V65, P1355, DOI 10.1016/j.chemosphere.2006.04.030"
"   Guo MX, 2017, SOIL BIOL BIOCHEM, V113, P130, DOI 10.1016/j.soilbio.2017.06.006"
"   Hale L, 2015, SOIL BIOL BIOCHEM, V81, P228, DOI 10.1016/j.soilbio.2014.11.023"
"   Hashem A, 2019, SAUDI J BIOL SCI, V26, P1291, DOI 10.1016/j.sjbs.2019.05.004"
"   Jin J, 2014, SCI TOTAL ENVIRON, V473, P308, DOI 10.1016/j.scitotenv.2013.12.033"
"   Li GR, 2020, ENVIRON POLLUT, V265, DOI 10.1016/j.envpol.2020.114887"
"   Li JB, 2019, SOIL BIOL BIOCHEM, V128, P100, DOI 10.1016/j.soilbio.2018.10.008"
"   Li N, 2021, SCI TOTAL ENVIRON, V754, DOI 10.1016/j.scitotenv.2020.141198"
"   Liu AM, 2016, SCI HORTIC-AMSTERDAM, V198, P311, DOI 10.1016/j.scienta.2015.11.030"
"   Liu SH, 2017, BIORESOURCE TECHNOL, V224, P25, DOI 10.1016/j.biortech.2016.11.095"
"   Liu WX, 2014, CHEMOSPHERE, V117, P303, DOI 10.1016/j.chemosphere.2014.07.026"
"   Liu XY, 2018, ECOTOX ENVIRON SAFE, V154, P69, DOI 10.1016/j.ecoenv.2018.02.028"
"   Lucena C, 2015, FRONT PLANT SCI, V6, DOI 10.3389/fpls.2015.01056"
"   Ma H, 2020, J HAZARD MATER, V388, DOI 10.1016/j.jhazmat.2020.122065"
"   Moubasher HA, 2015, INT BIODETER BIODEGR, V98, P113, DOI 10.1016/j.ibiod.2014.11.019"
"   N??dudvari AA, 2021, J HAZARD MATER, V412, DOI 10.1016/j.jhazmat.2021.125244"
"   Pandit NR, 2018, SCI TOTAL ENVIRON, V625, P1380, DOI 10.1016/j.scitotenv.2018.01.022"
"   Qi X, 2021, J HAZARD MATER, V401, DOI 10.1016/j.jhazmat.2020.123823"
"   Quilliam RS, 2013, SOIL BIOL BIOCHEM, V65, P287, DOI 10.1016/j.soilbio.2013.06.004"
"   Rahman Z, 2020, J HAZARD MATER, V396, DOI 10.1016/j.jhazmat.2020.122682"
"   Ravanbakhsh M, 2019, J HAZARD MATER, V379, DOI 10.1016/j.jhazmat.2019.120787"
"   Sarma H, 2019, ECOTOX ENVIRON SAFE, V176, P288, DOI 10.1016/j.ecoenv.2019.03.081"
"   Saxena J, 2013, SCI HORTIC-AMSTERDAM, V162, P351, DOI 10.1016/j.scienta.2013.08.002"
"   Singh VK, 2018, AGR ECOSYST ENVIRON, V267, P129, DOI 10.1016/j.agee.2018.08.020"
"   Song LC, 2021, CHEMOSPHERE, V279, DOI 10.1016/j.chemosphere.2021.130427"
"   Song LC, 2021, CHEMOSPHERE, V264, DOI 10.1016/j.chemosphere.2020.128513"
"   Sun L, 2017, ENVIRON POLLUT, V230, P862, DOI 10.1016/j.envpol.2017.07.037"
"   Tao KY, 2019, CHEMOSPHERE, V222, P831, DOI 10.1016/j.chemosphere.2019.01.133"
"   Tu C, 2020, ENVIRON INT, V137, DOI 10.1016/j.envint.2020.105576"
"   Wahla AQ, 2020, J HAZARD MATER, V390, DOI 10.1016/j.jhazmat.2019.121493"
"   Wang Q, 2019, CHEMOSPHERE, V234, P769, DOI 10.1016/j.chemosphere.2019.06.132"
"   Wang SX, 2012, J ENVIRON SCI, V24, P1662, DOI 10.1016/S1001-0742(11)60963-7"
"   Wang T, 2015, ENVIRON RES, V140, P405, DOI 10.1016/j.envres.2015.04.013"
"   Wang T, 2014, J HAZARD MATER, V278, P483, DOI 10.1016/j.jhazmat.2014.06.028"
"   Xiong BJ, 2017, CHEMOSPHERE, V182, P316, DOI 10.1016/j.chemosphere.2017.05.020"
"   Yang MY, 2020, APPL SOIL ECOL, V150, DOI 10.1016/j.apsoil.2019.103472"
"   Ye M, 2017, PEDOSPHERE, V27, P452, DOI [10.1016/S1002-0160(17)60341-2, 10.1016/s1002-0160(17)60341-2]"
"   Zhang XY, 2020, CHEMOSPHERE, V241, DOI 10.1016/j.chemosphere.2019.125027"
"   Zheng TY, 2021, CHEM ENG J, V407, DOI 10.1016/j.cej.2020.126343"
NR 50
TC 45
Z9 58
U1 13
U2 192
PU PERGAMON-ELSEVIER SCIENCE LTD
PI OXFORD
"PA THE BOULEVARD, LANGFORD LANE, KIDLINGTON, OXFORD OX5 1GB, the United Kingdom"

SN 0045-6535
EI 1879-1298
J9 CHEMOSPHERE
JI Chemosphere
PD NOV
PY 2022
VL 307
AR 136084
DI 10.1016/j.chemosphere.2022.136084
EA AUG 2022
PN 4
PG 8
WC Environmental Sciences
WE Science Citation Index Expanded (SCI-EXPANDED)
SC Environmental Sciences & Ecology
GA 4X2RB
UT WOS:000860693800007
PM 35988767
DA 2026-04-06
ER

PT J
"AU Khalid, M"
"   Ur-Rahman, S"
"   Hassani, D"
"   Hayat, K"
"   Zhou, P"
"   Hui, N"
"AF Khalid, Muhammad"
"   Ur-Rahman, Saeed"
"   Hassani, Danial"
"   Hayat, Kashif"
"   Zhou, Pei"
"   Hui, Nan"
TI Advances in fungal-assisted phytoremediation of heavy metals: A review
SO PEDOSPHERE
LA English
DT Review
DE arbuscular mycorrhizal fungi; bioremediation; endophytic fungi;
   environmental stress; metal tolerance; microbe; plant transporter; soil
   contaminant
ID ARBUSCULAR MYCORRHIZAL FUNGI; SEPTATE ENDOPHYTIC FUNGUS;
   PENICILLIUM-JANTHINELLUM LK5; VAR. FOLIOSA BAILEY; SOLANUM-NIGRUM L.;
   PLANT-GROWTH; CONTAMINATED SOILS; PIRIFORMOSPORA-INDICA; CADMIUM
   TOLERANCE; ENHANCED PHYTOREMEDIATION
"AB Trace metals such as manganese (Mn), copper (Cu), zinc (Zn), and iron (Fe) are essential for many biological processes in plant life cycles. However, in excess, they can be toxic and disrupt plant growth processes, which is economically undesirable for crop production. For this reason, processes such as homeostasis and transport control of these trace metals are of constant interest to scientists studying heavily contaminated habitats. Phytoremediation is a promising cleanup technology for soils polluted with heavy metals. However, this technique has some disadvantages, such as the slow growth rate of metal-accumulating plant species, low bioavailability of heavy metals, and long duration of remediation. Microbial-assisted phytoremediation is a promising strategy for hyperaccumulating, detoxifying, or remediating soil contaminants. Arbuscular mycorrhizal fungi (AMF) are found in association with almost all plants, contributing to their healthy performance and providing resistance against environmental stresses. They colonize plant roots and extend their hyphae to the rhizosphere region, assisting in mineral nutrient uptake and regulation of heavy metal acquisition. Endophytic fungi exist in every healthy plant tissue and provide enormous services to their host plants, including growth enhancement by nutrient acquisition, detoxification of heavy metals, secondary metabolite regulation, and enhancement of abiotic/biotic stress tolerance. The aim of the present work is to review the recent literature regarding the role of AMF and endophytic fungi in plant heavy metal tolerance in terms of its regulation in highly contaminated conditions."
"C1 [Khalid, Muhammad; Hayat, Kashif; Zhou, Pei; Hui, Nan] Shanghai Jiao Tong Univ, Sch Agr & Biol, Key Lab Urban Agr, Shanghai 200240, Peoples R China."
"   [Ur-Rahman, Saeed; Hassani, Danial] Shanghai Jiao Tong Univ, Fudan SJTU Nottingham Plant Biotechnol R&D Ctr, Sch Agr & Biol,Minist Agr,Plant Biotechnol Res Ct, Joint Int Res Lab Metab & Dev Sci,Key Lab Urban A, Shanghai 200240, Peoples R China."
C3 Shanghai Jiao Tong University; Shanghai Jiao Tong University
"RP Zhou, P; Hui, N (corresponding author), Shanghai Jiao Tong Univ, Sch Agr & Biol, Key Lab Urban Agr, Shanghai 200240, Peoples R China."
EM peizhousjtu@163.com; nan.hui@sjtu.edu.cn
"RI Khalid, Muhammad/AAU-4383-2021; Hui, Nan/H-3560-2018; Hayat,"
"   Kashif/ABG-4302-2020; hassani, danial/AGZ-8246-2022"
"OI Hui, Nan/0000-0001-8517-8454; hassani, danial/0000-0001-8024-0837;"
"   Rahman, Saeed ur/0000-0002-7082-5281"
FU National Key Research and Development Program of China [2016YFD0800807];
"   National Nature Science Foundation of China [31702003, 31902105]; Young"
   Elite Scientists Sponsorship Program of China Association for Science
   and Technology (CAST) [2017QNRC001]; China Postdoctoral Science
"   Foundation [2019 M651505]; ""Chenguang Program"" of the Shanghai Education"
"   Development Foundation; Shanghai Municipal Education Commission, China"
   [17CG07]; Shanghai Agriculture Science and Technology Development
"   Project, China [2018-4-13]"
FX This work was supported by the National Key Research and Development
"   Program of China (No. 2016YFD0800807), the National Nature Science"

"   Foundation of China (Nos. 31702003 and 31902105), the Young Elite"
   Scientists Sponsorship Program of China Association for Science and
"   Technology (CAST) (No. 2017QNRC001), the China Postdoctoral Science"
"   Foundation (No. 2019 M651505), the ""Chenguang Program"" of the Shanghai"
   Education Development Foundation and Shanghai Municipal Education
"   Commission, China (No. 17CG07), and the Shanghai Agriculture Science and"
"   Technology Development Project, China (No. 2018-4-13)."
"CR Abbaslou H, 2017, POLLUTION, V3, P471, DOI [10.7508/pj.2017.03.0, 10.7508/pj.2017.03.012]"
"   Abou Alhamed MF, 2012, PLANT BIOLOGY, V14, P859, DOI 10.1111/j.1438-8677.2012.00608.x"
"   Adams P, 2007, MICROB ECOL, V54, P306, DOI 10.1007/s00248-006-9203-0"
"   Ahmed MMM, 2017, SOIL ENVRON, V36, P1, DOI 10.25252/SE/17/41160"
"   Aly AH, 2011, APPL MICROBIOL BIOT, V90, P1829, DOI 10.1007/s00253-011-3270-y"
"   Amna, 2015, INT J PHYTOREMEDIAT, V17, P981, DOI [10.1080/15226514.2014.989311, DOI 10.1080/15226514.2014.989311]"
"   Arora K, 2016, INT J PHYTOREMEDIAT, V18, P704, DOI 10.1080/15226514.2015.1131232"
"   Arriagada C, 2009, BIORESOURCE TECHNOL, V100, P6250, DOI 10.1016/j.biortech.2009.07.010"
"   Assun??ao AGL, 2003, NEW PHYTOL, V159, P351, DOI 10.1046/j.1469-8137.2003.00820.x"
"   Ferreira PAA, 2018, REV BRAS CIENC SOLO, V42, DOI 10.1590/18069657rbcs20170245"
"   Babu AG, 2014, ECOL ENG, V69, P186, DOI 10.1016/j.ecoleng.2014.03.055"
"   Babu AG, 2014, SCI TOTAL ENVIRON, V476, P561, DOI 10.1016/j.scitotenv.2013.12.119"
"   Baghaie AH, 2019, ENVIRON SCI POLLUT R, V26, P30794, DOI 10.1007/s11356-019-06237-0"
"   Bais HP, 2004, PLANT PHYSIOL, V134, P307, DOI 10.1104/pp.103.028712"
"   Ban YH, 2017, PEDOSPHERE, V27, P283, DOI [10.1016/s1002-0160(17)60316-3, 10.1016/S1002-0160(17)60316-3]"
"   Baum C, 2006, J PLANT NUTR SOIL SC, V169, P516, DOI 10.1002/jpln.200521925"
"   Berthelot C, 2016, FEMS MICROBIOL ECOL, V92, DOI 10.1093/femsec/fiw144"
"   Bibi S, 2018, CHEMOSPHERE, V211, P653, DOI 10.1016/j.chemosphere.2018.07.197"
"   Cabral L, 2015, WORLD J MICROB BIOT, V31, P1655, DOI 10.1007/s11274-015-1918-y"
"   Cao LX, 2008, CHEMOSPHERE, V71, P1769, DOI 10.1016/j.chemosphere.2008.01.066"
"   Carvalho LM, 2006, PLANT SOIL, V285, P161, DOI 10.1007/s11104-006-9001-y"
"   Cevnik M, 2000, PHYTON-ANN REI BOT A, V40, P61"
"   Chamba I, 2017, CHEMOSPHERE, V188, P633, DOI 10.1016/j.chemosphere.2017.08.160"
"   Chang Q, 2018, ENVIRON POLLUT, V241, P607, DOI 10.1016/j.envpol.2018.06.003"
"   Chaturvedi R, 2018, INT J PHYTOREMEDIAT, V20, P885, DOI 10.1080/15226514.2018.1438360"
"   Chaturyedi R, 2018, ECOTOX ENVIRON SAFE, V148, P318, DOI 10.1016/j.ecoenv.2017.10.048"
"   Chen BD, 2003, CHEMOSPHERE, V50, P839, DOI 10.1016/S0045-6535(02)00228-X"
"   Chen LH, 2015, ECOTOX ENVIRON SAFE, V113, P460, DOI 10.1016/j.ecoenv.2014.12.033"
"   Chen XW, 2013, ENVIRON EXP BOT, V87, P92, DOI 10.1016/j.envexpbot.2012.08.002"
"   Chen XW, 2019, GEODERMA, V337, P749, DOI 10.1016/j.geoderma.2018.10.029"
"   Chen XW, 2012, CHEMOSPHERE, V89, P1248, DOI 10.1016/j.chemosphere.2012.07.054"
"   Chen Y, 2013, APPL SOIL ECOL, V67, P20, DOI 10.1016/j.apsoil.2013.02.004"
"   Chiapello M, 2015, METALLOMICS, V7, P805, DOI [10.1039/c5mt00024f, 10.1039/C5MT00024F]"
"   Chibucos MC, 2009, BMC MICROBIOL, V9, DOI 10.1186/1471-2180-9-S1-S6"
"   Christie P, 2004, PLANT SOIL, V261, P209, DOI 10.1023/B:PLSO.0000035542.79345.1b"
"   Cicatelli A, 2014, IFOREST, V7, P333, DOI 10.3832/ifor1045-007"
"   Coninx L, 2017, ADV BOT RES, V83, P127, DOI 10.1016/bs.abr.2016.12.005"
"   Cozzolino V, 2016, ENVIRON SCI POLLUT R, V23, P11312, DOI 10.1007/s11356-016-6337-6"
"   D'Amico M, 2008, MYCOL RES, V112, P100, DOI 10.1016/j.mycres.2007.11.007"
"   de Souza LA, 2012, ACTA PHYSIOL PLANT, V34, P523, DOI 10.1007/s11738-011-0849-y"
"   Debeljak M, 2018, CHEMOSPHERE, V212, P1076, DOI 10.1016/j.chemosphere.2018.08.147"
"   Deng ZJ, 2017, CHEMOSPHERE, V168, P1100, DOI 10.1016/j.chemosphere.2016.10.097"
"   Deng ZJ, 2014, SOIL BIOL BIOCHEM, V77, P31, DOI 10.1016/j.soilbio.2014.06.005"
"   Deng ZJ, 2014, ENVIRON SCI POLLUT R, V21, P2346, DOI 10.1007/s11356-013-2163-2"
"   Deng ZJ, 2013, CHEMOSPHERE, V91, P41, DOI 10.1016/j.chemosphere.2012.11.065"
"   Deng ZJ, 2012, WATER AIR SOIL POLL, V223, P5321, DOI 10.1007/s11270-012-1282-6"
"   Diene O, 2014, PLOS ONE, V9, DOI 10.1371/journal.pone.0109233"
"   Diffels JF, 2006, BIOCHIMIE, V88, P1639, DOI 10.1016/j.biochi.2006.08.008"
"   Ding ZiLi Ding ZiLi, 2016, African Journal of Microbiology Research, V10, P1080, DOI [10.5897/ajmr2016.8126, 10.5897/AJMR2016.8126]"
"   Dur??n N, 2011, APPL MICROBIOL BIOT, V90, P1609, DOI 10.1007/s00253-011-3249-8"
"   Dushenkov S, 1997, ENVIRON SCI TECHNOL, V31, P3468, DOI 10.1021/es970220l"
"   Ehrnstorfer IA, 2014, NAT STRUCT MOL BIOL, V21, P990, DOI 10.1038/nsmb.2904"
"   El Faiz A, 2015, INT J PHYTOREMEDIAT, V17, P503, DOI 10.1080/15226514.2014.950408"
"   Emamverdian A., 2015, SCI WORLD J, DOI DOI 10.1155/2015/756120"
"   Firmin S, 2015, SCI TOTAL ENVIRON, V527, P91, DOI 10.1016/j.scitotenv.2015.04.116"
"   Fomina MA, 2005, SOIL BIOL BIOCHEM, V37, P851, DOI 10.1016/j.soilbio.2004.10.013"
"   Freeman JL, 2004, PLANT CELL, V16, P2176, DOI 10.1105/tpc.104.023036"
"   Gadd GM, 2007, MYCOL RES, V111, P3, DOI 10.1016/j.mycres.2006.12.001"
"   Gaude N, 2012, PLANT J, V69, P510, DOI 10.1111/j.1365-313X.2011.04810.x"
"   Ghosh M., 2005, Applied Ecology and Environmental Research, V3, P1"
"   Gill SS, 2010, PLANT PHYSIOL BIOCH, V48, P909, DOI 10.1016/j.plaphy.2010.08.016"
"   Glick BR, 2010, BIOTECHNOL ADV, V28, P367, DOI 10.1016/j.biotechadv.2010.02.001"
"   Glick BR, 2003, BIOTECHNOL ADV, V21, P383, DOI 10.1016/S0734-9750(03)00055-7"
"   G??hre V, 2006, PLANTA, V223, P1115, DOI 10.1007/s00425-006-0225-0"
"   Corr??a RCG, 2019, REF SER PHYTOCHEM, P609, DOI 10.1007/978-3-319-90484-9_24"
"   Gong B, 2017, BRAZ J MICROBIOL, V48, P530, DOI 10.1016/j.bjm.2016.10.027"
"   Gonz??lez-Ch??vez MDA, 2011, FUNGAL BIOL-UK, V115, P1197, DOI 10.1016/j.funbio.2011.08.005"
"   Govarthanan M, 2018, ECOTOX ENVIRON SAFE, V151, P273, DOI 10.1016/j.ecoenv.2018.01.020"
"   Gu HH, 2017, INT J PHYTOREMEDIAT, V19, P739, DOI 10.1080/15226514.2017.1284751"
"   Gucwa-Przepi??ra E, 2013, ACTA SOC BOT POL, V82, P251, DOI 10.5586/asbp.2013.033"
"   Gunathilakae Nimasha, 2018, Groundwater for Sustainable Development, V7, P477, DOI [10.1016/j.gsd.2018.03.008, 10.1016/j.gsd.2018.03.008]"
"   Handa Y, 2015, PLANT CELL PHYSIOL, V56, P1490, DOI 10.1093/pcp/pcv071"
"   Harada E, 2010, PLANT CELL PHYSIOL, V51, P1627, DOI 10.1093/pcp/pcq118"
"   He YM, 2017, ENVIRON SCI POLLUT R, V24, P18494, DOI 10.1007/s11356-017-9459-6"

"   Hijikata N, 2010, NEW PHYTOL, V186, P285, DOI 10.1111/j.1469-8137.2009.03168.x"
"   Hildebrandt U, 2007, PHYTOCHEMISTRY, V68, P139, DOI 10.1016/j.phytochem.2006.09.023"
"   Hogekamp C, 2011, PLANT PHYSIOL, V157, P2023, DOI 10.1104/pp.111.186635"
"   Hrishikesh Upadhyaya Hrishikesh Upadhyaya, 2010, Journal of Phytology, V2, P16"
"   Hristozkova M, 2016, APPL SOIL ECOL, V101, P57, DOI 10.1016/j.apsoil.2016.01.008"
"   Hristozkova M, 2015, TURK J BIOL, V39, P461, DOI 10.3906/biy-1408-47"
"   Huang XC, 2018, ECOTOX ENVIRON SAFE, V149, P43, DOI 10.1016/j.ecoenv.2017.11.011"
"   Hui FQ, 2015, J ENVIRON SCI, V37, P184, DOI 10.1016/j.jes.2015.06.005"
"   Ikram M, 2018, PLOS ONE, V13, DOI 10.1371/journal.pone.0208150"
"   Jaffr?? T, 2013, FRONT PLANT SCI, V4, DOI 10.3389/fpls.2013.00279"
"   Janouskov?? M, 2006, CHEMOSPHERE, V65, P1959, DOI 10.1016/j.chemosphere.2006.07.007"
"   Jiang M, 2008, LETT APPL MICROBIOL, V47, P561, DOI 10.1111/j.1472-765X.2008.02454.x"
"   [?????? JIANG Shu], 2010, [?????????, Chinese Traditional and Herbal Drugs], V41, P121"
"   Jin HQ, 2018, SYMBIOSIS, V74, P89, DOI 10.1007/s13199-017-0485-4"
"   Alvarado-L??pez CJ, 2019, ENVIRON SCI POLLUT R, V26, P381, DOI 10.1007/s11356-018-3569-7"
"   Kafil M, 2019, INT J PHYTOREMEDIAT, V21, P92, DOI 10.1080/15226514.2018.1474443"
"   Kaldorf M, 1999, J PLANT PHYSIOL, V154, P718, DOI 10.1016/S0176-1617(99)80250-8"
"   Kamran MA, 2014, ENVIRON SCI POLLUT R, V21, P801, DOI 10.1007/s11356-013-2187-7"
"   Kangwankraiphaisan T, 2013, BIOMETALS, V26, P489, DOI 10.1007/s10534-013-9634-2"
"   Karimi A, 2011, AFR J MICROBIOL RES, V5, P1571"
"   Khan AL, 2013, BMC PLANT BIOL, V13, DOI 10.1186/1471-2229-13-86"
"   Khan AL, 2015, J HAZARD MATER, V295, P70, DOI 10.1016/j.jhazmat.2015.04.008"
"   Khan AL, 2014, BIOL FERT SOILS, V50, P75, DOI 10.1007/s00374-013-0833-3"
"   Khan AR, 2017, ENVIRON EXP BOT, V135, P126, DOI 10.1016/j.envexpbot.2016.03.005"
"   Khan AR, 2017, ECOTOX ENVIRON SAFE, V136, P180, DOI 10.1016/j.ecoenv.2016.03.014"
"   Khan Z., 2011, Current Topics in Plant Biology, Volume 11, P97"
"   Klauberg-Filho O., 2002, Rev. Bras. Ci??nc. Solo, V26, P125, DOI 10.1590/S0100-06832002000100012"
"   Kobae Y, 2014, SOIL SCI PLANT NUTR, V60, P843, DOI 10.1080/00380768.2014.949854"
"   Kotrba P, 2009, BIOTECHNOL ADV, V27, P799, DOI 10.1016/j.biotechadv.2009.06.003"
"   Lacercat-Didier L, 2016, MYCORRHIZA, V26, P657, DOI 10.1007/s00572-016-0699-y"
"   Lam CM, 2018, ECOTOX ENVIRON SAFE, V162, P563, DOI 10.1016/j.ecoenv.2018.07.047"
"   Lanfranco L, 2002, PLANT PHYSIOL, V130, P58, DOI 10.1104/pp.003525"
"   Lasat MM, 2002, J ENVIRON QUAL, V31, P109, DOI 10.2134/jeq2002.0109"
"   Latef AAHA, 2016, J PLANT BIOL, V59, P407, DOI 10.1007/s12374-016-0237-7"
"   Lermen C, 2015, IND CROP PROD, V76, P734, DOI 10.1016/j.indcrop.2015.07.009"
"   Li HY, 2012, FUNGAL DIVERS, V54, P11, DOI 10.1007/s13225-012-0165-x"
"   Li HY, 2012, FUNGAL ECOL, V5, P309, DOI 10.1016/j.funeco.2011.06.002"
"   Li H, 2018, SCI TOTAL ENVIRON, V613, P447, DOI 10.1016/j.scitotenv.2017.09.066"
"   Li H, 2016, SCI TOTAL ENVIRON, V571, P1183, DOI 10.1016/j.scitotenv.2016.07.124"
"   Li JM, 2017, SCI TOTAL ENVIRON, V599, P1462, DOI 10.1016/j.scitotenv.2017.05.063"
"   Li T, 2011, SCI TOTAL ENVIRON, V409, P1069, DOI 10.1016/j.scitotenv.2010.12.012"
"   Li XN, 2017, PLANT PHYSIOL BIOCH, V114, P38, DOI 10.1016/j.plaphy.2017.02.014"
"   Li X, 2019, CHEMOSPHERE, V220, P910, DOI 10.1016/j.chemosphere.2018.12.211"
"   Li XM, 2015, J HAZARD MATER, V295, P79, DOI 10.1016/j.jhazmat.2015.04.015"
"   Li XM, 2012, J HAZARD MATER, V213, P55, DOI 10.1016/j.jhazmat.2012.01.052"
"   Li YY, 2020, INT J PHYTOREMEDIAT, V22, P451, DOI 10.1080/15226514.2019.1671796"
"   Likar M, 2013, PLANT SOIL, V370, P593, DOI 10.1007/s11104-013-1656-6"
"   Likar M, 2009, SCI TOTAL ENVIRON, V407, P6179, DOI 10.1016/j.scitotenv.2009.08.045"
"   Lingua G, 2008, ENVIRON POLLUT, V153, P137, DOI 10.1016/j.envpol.2007.07.012"
"   Liu CG, 2018, ENVIRON POLLUT, V240, P557, DOI 10.1016/j.envpol.2018.04.138"
"   L??pez A, 2000, J IND MICROBIOL BIOT, V24, P146, DOI 10.1038/sj.jim.2900793"
"   de Andrade SAL, 2008, INT J PHYTOREMEDIAT, V10, P1, DOI 10.1080/15226510701827002"
"   de Andrade SAL, 2015, CHEMOSPHERE, V134, P141, DOI 10.1016/j.chemosphere.2015.04.023"
"   Ma Y, 2019, J HAZARD MATER, V379, DOI 10.1016/j.jhazmat.2019.120813"
"   Ma Y, 2016, J HAZARD MATER, V320, P36, DOI 10.1016/j.jhazmat.2016.08.009"
"   Ma Y, 2011, J HAZARD MATER, V195, P230, DOI 10.1016/j.jhazmat.2011.08.034"
"   Mani D, 2015, ECOTOX ENVIRON SAFE, V111, P86, DOI 10.1016/j.ecoenv.2014.09.019"
"   Mesjasz-Przybylowicz J, 2004, ACTA BIOL CRACOV BOT, V46, P75"
"   Mirzahosseini Z, 2014, EUR J SOIL BIOL, V63, P33, DOI 10.1016/j.ejsobi.2014.05.004"
"   Mohd S, 2019, ECOTOX ENVIRON SAFE, V176, P108, DOI 10.1016/j.ecoenv.2019.03.053"
"   Mohd S, 2017, FRONT MICROBIOL, V8, DOI 10.3389/fmicb.2017.00754"
"   Monnet F, 2001, PHYSIOL PLANTARUM, V113, P557, DOI 10.1034/j.1399-3054.2001.1130415.x"
"   Moore FP, 2006, SYST APPL MICROBIOL, V29, P539, DOI 10.1016/j.syapm.2005.11.012"
"   Morel M, 2009, PLANT PHYSIOL, V149, P894, DOI 10.1104/pp.108.130294"
"   Muhammad Zain-ul-Arifeen Muhammad Zain-ul-Arifeen, 2015, American-Eurasian Journal of Agricultural & Environmental Sciences, V15, P1155"
"   M??ller CB, 2005, CURR OPIN PLANT BIOL, V8, P450, DOI 10.1016/j.pbi.2005.05.007"
"   Na G, 2011, ENVIRON EXP BOT, V72, P18, DOI 10.1016/j.envexpbot.2010.04.004"
"   Nafady NA, 2018, INT J PHYTOREMEDIAT, V20, P869, DOI 10.1080/15226514.2018.1438358"
"   Nanda R, 2018, ECOTOX ENVIRON SAFE, V156, P409, DOI 10.1016/j.ecoenv.2018.03.016"
"   Neagoe A, 2017, REV CHIM-BUCHAREST, V68, P789, DOI 10.37358/rc.17.4.5554"
"   Nguyen THH, 2011, BIORESOURCE TECHNOL, V102, P2228, DOI 10.1016/j.biortech.2010.10.014"
"   Padmavathiamma PK, 2007, WATER AIR SOIL POLL, V184, P105, DOI 10.1007/s11270-007-9401-5"
"   Park J, 2012, PLANT J, V69, P278, DOI 10.1111/j.1365-313X.2011.04789.x"
"   Pastor J, 2015, INT J PHYTOREMEDIAT, V17, P988, DOI 10.1080/15226514.2014.1003786"
"   Pawlowska TE, 2004, APPL ENVIRON MICROB, V70, P6643, DOI 10.1128/AEM.70.11.6643-6649.2004"
"   Pedroso DD, 2018, B ENVIRON CONTAM TOX, V101, P386, DOI 10.1007/s00128-018-2405-6"
"   Pietro-Souza W, 2017, PLOS ONE, V12, DOI 10.1371/journal.pone.0182017"
"   Rafique M, 2019, ENVIRON SCI POLLUT R, V26, P20689, DOI 10.1007/s11356-019-05323-7"
"   Rajkumar M, 2010, TRENDS BIOTECHNOL, V28, P142, DOI 10.1016/j.tibtech.2009.12.002"
"   Rascio N, 2011, PLANT SCI, V180, P169, DOI 10.1016/j.plantsci.2010.08.016"
"   Rasouli-Sadaghiani M., 2016, Water Soil, V30, P542, DOI DOI 10.22067/JSW.V30I2.40077"
"   Rasouli-Sadaghiani MH, 2019, J PLANT GROWTH REGUL, V38, P113, DOI 10.1007/s00344-018-9815-x"
"   Ren AZ, 2011, INT J PHYTOREMEDIAT, V13, P233, DOI 10.1080/15226511003671387"

"   Ren CG, 2019, CHEMOSPHERE, V217, P773, DOI 10.1016/j.chemosphere.2018.11.085"
"   Rodriguez RJ, 2009, NEW PHYTOL, V182, P314, DOI 10.1111/j.1469-8137.2009.02773.x"
"   Rollon R. J. C., 2017, AAB Bioflux, V9, P57"
"   Rozpadek P, 2018, ENVIRON EXP BOT, V147, P31, DOI 10.1016/j.envexpbot.2017.11.009"
"   Ruscitti M, 2017, THEOR EXP PLANT PHYS, V29, P37, DOI 10.1007/s40626-016-0081-7"
"   Ruytinx J, 2013, METALLOMICS, V5, P1225, DOI 10.1039/c3mt00061c"
"   Sabra M, 2018, FRONT PLANT SCI, V9, DOI 10.3389/fpls.2018.01726"
"   SALT DE, 1995, BIO-TECHNOL, V13, P468, DOI 10.1038/nbt0595-468"
"   Schneider J, 2017, ENVIRON POLLUT, V224, P125, DOI 10.1016/j.envpol.2017.01.071"
"   Schneider J, 2016, SCI TOTAL ENVIRON, V572, P86, DOI 10.1016/j.scitotenv.2016.07.185"
"   Schneider J, 2013, J HAZARD MATER, V262, P1105, DOI 10.1016/j.jhazmat.2012.09.063"
"   Schneider J, 2013, J HAZARD MATER, V262, P1245, DOI 10.1016/j.jhazmat.2012.05.091"
"   Setyaningsih L, 2017, IOP C SER EARTH ENV, V58, DOI 10.1088/1755-1315/58/1/012053"
"   Shahabivand S, 2018, Journal of Genetic Resources, V4, P44, DOI 10.22080/jgr.2018.14168.1102"
"   Shahabivand S, 2017, ECOTOX ENVIRON SAFE, V145, P496, DOI 10.1016/j.ecoenv.2017.07.064"
"   Shahabivand S, 2012, PLANT PHYSIOL BIOCH, V60, P53, DOI 10.1016/j.plaphy.2012.07.018"
"   Sheikh-Assadi M, 2015, INT J PHYTOREMEDIAT, V17, P556, DOI 10.1080/15226514.2014.922928"
"   Shen M, 2013, FUNGAL ECOL, V6, P539, DOI 10.1016/j.funeco.2013.08.001"
"   Sheng XF, 2008, J HAZARD MATER, V155, P17, DOI 10.1016/j.jhazmat.2007.10.107"
"   Shi YA, 2017, ENVIRON SCI POLLUT R, V24, P417, DOI 10.1007/s11356-016-7693-y"
"   Sim CSF, 2018, WATER AIR SOIL POLL, V229, DOI 10.1007/s11270-018-3733-1"
"   Singh G, 2019, SOIL SEDIMENT CONTAM, V28, P729, DOI 10.1080/15320383.2019.1657381"
"   Smith SE, 2008, MYCORRHIZAL SYMBIOSIS, 3RD EDITION, P13, DOI 10.1016/B978-012370526-6.50003-9"
"   Soleimani M, 2010, CHEMOSPHERE, V81, P1084, DOI 10.1016/j.chemosphere.2010.09.034"
"   Soleimani M, 2010, INT J PHYTOREMEDIAT, V12, P535, DOI 10.1080/15226510903353187"
"   Stadler M, 2012, IMA FUNGUS, V3, P51"
"   Stepniewska Z, 2013, APPL MICROBIOL BIOT, V97, P9589, DOI 10.1007/s00253-013-5235-9"
"   Sun LQ, 2017, ENVIRON SCI POLLUT R, V24, P9681, DOI 10.1007/s11356-017-8637-x"
"   Sut M, 2016, ECOTOXICOLOGY, V25, P1260, DOI 10.1007/s10646-016-1678-y"
"   Tangahu BV, 2011, INT J CHEM ENG, V2011, DOI 10.1155/2011/939161"
"   Tisserant E, 2013, P NATL ACAD SCI USA, V110, P20117, DOI 10.1073/pnas.1313452110"
"   Usuki F, 2007, MYCOLOGIA, V99, P175, DOI 10.3852/mycologia.99.2.175"
"   Vadassery J, 2009, PLANT J, V59, P193, DOI 10.1111/j.1365-313X.2009.03867.x"
"   van der Ent A, 2015, ENVIRON SCI TECHNOL, V49, P4773, DOI 10.1021/es506031u"
"   Wang FY, 2007, INT J PHYTOREMEDIAT, V9, P345, DOI 10.1080/15226510701476214"
"   Wang FY, 2018, CHEMOSPHERE, V209, P421, DOI 10.1016/j.chemosphere.2018.06.099"
"   Wang J, 2016, SCI REP-UK, V6, DOI 10.1038/srep20728"
"   Wang S, 2018, APPL ECOL ENV RES, V16, P5987, DOI 10.15666/aeer/1605_59875999"
"   Wang WF, 2013, INT J PHYTOREMEDIAT, V15, P488, DOI 10.1080/15226514.2012.716101"
"   Waqas M, 2014, BIOL FERT SOILS, V50, P1155, DOI 10.1007/s00374-014-0937-4"
"   Watts-Williams SJ, 2013, PLANT SOIL, V371, P299, DOI 10.1007/s11104-013-1670-8"
"   Wei YF, 2016, ECOTOX ENVIRON SAFE, V124, P363, DOI 10.1016/j.ecoenv.2015.11.008"
"   WEISSENHORN I, 1994, PLANT SOIL, V167, P189, DOI 10.1007/BF00007944"
"   Weyens N, 2009, ENVIRON SCI TECHNOL, V43, P9413, DOI 10.1021/es901997z"
"   Wezowicz K, 2015, ENVIRON SCI POLLUT R, V22, P19400, DOI 10.1007/s11356-015-5706-x"
"   White JF, 2010, PHYSIOL PLANTARUM, V138, P440, DOI 10.1111/j.1399-3054.2009.01332.x"
"   Wintz H, 2003, J BIOL CHEM, V278, P47644, DOI 10.1074/jbc.M309338200"
"   Wong MH, 2003, CHEMOSPHERE, V50, P775, DOI 10.1016/S0045-6535(02)00232-1"
"   Wu JT, 2020, PLANT BIOLOGY, V22, P62, DOI 10.1111/plb.13039"
"   Wu L., 1990, Heavy metal tolerance in plants: evolutionary aspects., P269"
"   Wu LS, 2013, CURR MICROBIOL, V66, P40, DOI 10.1007/s00284-012-0235-z"
"   Wu SL, 2016, ENVIRON EXP BOT, V122, P10, DOI 10.1016/j.envexpbot.2015.08.006"
"   Wu SL, 2015, ENVIRON SCI TECHNOL, V49, P14036, DOI 10.1021/acs.est.5b03659"
"   Xiao X, 2010, BIORESOURCE TECHNOL, V101, P1668, DOI 10.1016/j.biortech.2009.09.083"
"   Xiao Yi Xiao Yi, 2014, African Journal of Microbiology Research, V8, P1535"
"   Xin G, 2009, MYCOL RES, V113, P973, DOI 10.1016/j.mycres.2009.06.001"
"   Yamaji K, 2016, PLOS ONE, V11, DOI 10.1371/journal.pone.0169089"
"   Yang Y, 2017, SCI REP-UK, V7, DOI 10.1038/s41598-017-05834-8"
"   Yang YQ, 2015, PLOS ONE, V10, DOI [10.1371/journal.pone.0145726, 10.1371/journal.pone.0117475]"
"   Yang YR, 2016, SCI REP-UK, V6, DOI 10.1038/srep20469"
"   Yoon J, 2006, SCI TOTAL ENVIRON, V368, P456, DOI 10.1016/j.scitotenv.2006.01.016"
"   Zahoor M, 2017, ECOTOX ENVIRON SAFE, V142, P139, DOI 10.1016/j.ecoenv.2017.04.005"
"   Zamani N, 2015, INT J PHYTOREMEDIAT, V17, P456, DOI 10.1080/15226514.2014.922919"
"   Zarei M, 2008, ENVIRON POLLUT, V156, P1277, DOI 10.1016/j.envpol.2008.03.006"
"   Zarei M, 2008, EUR J SOIL BIOL, V44, P381, DOI 10.1016/j.ejsobi.2008.06.004"
"   Zhan FD, 2018, ENVIRON SCI POLLUT R, V25, P24338, DOI 10.1007/s11356-018-2487-z"
"   Zhang FG, 2019, SCI TOTAL ENVIRON, V655, P1150, DOI 10.1016/j.scitotenv.2018.11.317"
"   Zhang XX, 2012, SCI CHINA LIFE SCI, V55, P793, DOI 10.1007/s11427-012-4359-y"
"   Zhang XX, 2010, J HAZARD MATER, V175, P703, DOI 10.1016/j.jhazmat.2009.10.066"
"   Zhang YF, 2011, J HAZARD MATER, V186, P1720, DOI 10.1016/j.jhazmat.2010.12.069"
"   Zhang Y, 2018, SCI TOTAL ENVIRON, V628-629, P282, DOI 10.1016/j.scitotenv.2018.01.331"
"   Zhang YJ, 2008, J MICROBIOL, V46, P624, DOI 10.1007/s12275-008-0163-6"
"   Zhao FJ, 2003, PLANT SOIL, V249, P37, DOI 10.1023/A:1022530217289"
"   Zhou XJ, 2013, BMC GENOMICS, V14, DOI 10.1186/1471-2164-14-238"
"   Zhu LL, 2018, ENVIRON SCI POLLUT R, V25, P35232, DOI 10.1007/s11356-018-3456-2"
"   Zhu SC, 2015, J CENT SOUTH UNIV, V22, P88, DOI 10.1007/s11771-015-2498-6"
NR 233
TC 91
Z9 106
U1 17
U2 484
PU SCIENCE PRESS
PI BEIJING
"PA 16 DONGHUANGCHENGGEN NORTH ST, BEIJING 100717, PEOPLES R CHINA"

SN 1002-0160
EI 2210-5107
J9 PEDOSPHERE
JI Pedosphere
PD APR
PY 2021
VL 31
IS 3
BP 475
EP 495
DI 10.1016/S1002-0160(20)60091-1
PG 21
WC Soil Science
WE Science Citation Index Expanded (SCI-EXPANDED)
SC Agriculture
GA PO6TY
UT WOS:000605304200014
DA 2026-04-06
ER

PT J
"AU Wu, JW"
"   Tao, Q"
"   Khan, MB"
"AF Wu, Jiawen"
"   Tao, Qi"
"   Khan, Muhammad Bilal"
TI Editorial: Safe utilization of heavy metals pollution in soils for
   healthy food
SO FRONTIERS IN PLANT SCIENCE
LA English
DT Editorial Material
DE alleviation; bioremediation; crop; heavy metal; phytoremediation;
   detoxification; exogenous supply; inoculation
"C1 [Wu, Jiawen] Yanan Univ, Coll Life Sci, Yanan, Peoples R China."
"   [Tao, Qi] Sichuan Agr Univ, Coll Resources, Chengdu, Peoples R China."
"   [Khan, Muhammad Bilal] Ayub Agr Res Inst, Soil & Water Testing Lab Layyah, Faisalabad, Pakistan."
C3 Yanan University; Sichuan Agricultural University
"RP Wu, JW (corresponding author), Yanan Univ, Coll Life Sci, Yanan, Peoples R China."
EM wujiawende@126.com
"RI BILAL KHAN, MUHAMMAD/JLM-5504-2023; Tao, Qi/AAC-2514-2019"
"OI BILAL KHAN, MUHAMMAD/0000-0001-6658-6129; "
NR 0
TC 0
Z9 0
U1 2
U2 9
PU FRONTIERS MEDIA SA
PI LAUSANNE
"PA AVENUE DU TRIBUNAL FEDERAL 34, LAUSANNE, CH-1015, SWITZERLAND"
SN 1664-462X
J9 FRONT PLANT SCI
JI Front. Plant Sci.
PD NOV 11
PY 2024
VL 15
AR 1511483
DI 10.3389/fpls.2024.1511483
PG 2
WC Plant Sciences
WE Science Citation Index Expanded (SCI-EXPANDED)
SC Plant Sciences
GA N2P3T
UT WOS:001362815200001
PM 39588092
"OA Green Submitted, gold"
DA 2026-04-06
ER

PT J
"AU Fang, XH"
"   Zhong, XB"
"   Cui, ZY"
"   Zhang, Y"
"   Du, LN"
"   Yang, YJ"
"   Lv, JL"
"AF Fang, Xianhui"
"   Zhong, Xianbao"
"   Cui, Ziying"
"   Zhang, Yang"
"   Du, Lina"
"   Yang, Yajun"
"   Lv, Jialong"

TI Distribution and Remediation Techniques of Heavy Metals in Soil
   Aggregates Perspective: A Review
SO WATER AIR AND SOIL POLLUTION
LA English
DT Review
DE Soil aggregates; Heavy metals; Adsorption; Remediation methods
ID WEIGHT ORGANIC-ACIDS; CONTAMINATED PADDY SOILS; DIFFERENT SIZE
   FRACTIONS; PARTICLE-SIZE; CITRIC-ACID; MICROBIAL BIOREMEDIATION;
   COMPETITIVE ADSORPTION; SOURCE IDENTIFICATION; BINDING PROPERTIES;
   SURFACE SEDIMENTS
"AB Soil aggregates, formed by soil particles and organic matter, have significant impacts on the physical properties of soil and play a crucial role in the migration of nutrients and pollutants. Heavy metals, as harmful soil pollutants, pose important risks to human health and ecosystems. Therefore, understanding the distribution, forms, and migration characteristics of heavy metals in soil aggregates is crucial. This study aims to investigate heavy metal pollution from the perspective of microscopic soil aggregates. A bibliometric analysis was conducted to review studies on the distribution and remediation of heavy metals in soil aggregates over the past 30 years. Based on keyword analysis, the review focused on three main themes: different soil types, adsorption mechanisms and influencing factors of heavy metals on aggregates of different particle sizes, and biochemical remediation. Generally speaking, microaggregates have a strong enrichment capacity for most heavy metals due to their large surface area and numerous adsorption sites. However, the adsorption of aggregates on heavy metals is also related to the physicochemical properties of the soil. Currently, there is relatively little research on the impact of various soil heavy metal remediation techniques on soil aggregates. In the future, this can be strengthened through advanced analytical techniques and modeling methods. Understanding the interaction between heavy metals and soil aggregates is essential for comprehending migration and transformation mechanisms and developing effective remediation technologies. Remediation of heavy metal-contaminated soils using soil aggregates can enhance adsorption and immobilization of heavy metals and improve soil structure. Additionally, the restoration of soil aggregates is vital for soil remediation, enhancing stability, reducing erosion, and promoting the overall recovery of soil ecosystems. Thus, studying heavy metals in soil aggregates is of practical significance for sustainable agriculture and ecological protection."
"C1 [Fang, Xianhui; Zhong, Xianbao; Cui, Ziying; Zhang, Yang; Yang, Yajun; Lv, Jialong] Northwest A&F Univ, Coll Nat Resources & Environm, 3 Taicheng Rd, Yangling 712100, Shaanxi, Peoples R China."
"   [Fang, Xianhui; Zhong, Xianbao; Cui, Ziying; Zhang, Yang; Yang, Yajun; Lv, Jialong] Minist Agr, Key Lab Plant Nutr & Agrienvironm Northwest China, Yangling, Peoples R China."
"   [Du, Lina] Univ Alberta, Dept Renewable Resources, Edmonton, AB T6G 2G1, Canada."
C3 Northwest A&F University - China; University of Alberta
"RP Yang, YJ; Lv, JL (corresponding author), Northwest A&F Univ, Coll Nat Resources & Environm, 3 Taicheng Rd, Yangling 712100, Shaanxi, Peoples R China."
EM yajuny@nwsuaf.edu.cn; ljlll@nwsuaf.edu.cn
"RI Du, Lina/HJA-5282-2022; Yang, Yajun/JFJ-8563-2023"
"OI Du, Lina/0009-0008-8580-2964; Zhong, Xianbao/0009-0007-8994-9464; "
FU This study was supported by National natural science foundation of China
"   (Project No. 42077135), Talent Special Fund Grant from Northwest"
   Aamp;amp;F university (Project No. 2452021124) and Innovation and
   entrepreneurship training program for College Stude [42077135]; National
   natural science foundation of China [2452021124]; Talent Special Fund
   Grant from Northwest Aamp;amp;F university [S202210712582]; Innovation
   and entrepreneurship training program for College Students from
   Northwest Aamp;amp;F university [A314021402-202218]; Foundation of the
   State Key Laboratory of Soil Erosion and Dryland Farming on the Loess
   Plateau
FX This study was supported by National natural science foundation of China
"   (Project No. 42077135), Talent Special Fund Grant from Northwest A &"
   amp;F university (Project No. 2452021124) and Innovation and
   entrepreneurship training program for College Students from Northwest A
"   & amp;F university (S202210712582), Foundation of the State Key"
   Laboratory of Soil Erosion and Dryland Farming on the Loess Plateau
   (A314021402-202218).
"CR Abrahams PW, 2002, SCI TOTAL ENVIRON, V291, P1, DOI 10.1016/S0048-9697(01)01102-0"
"   Acosta JA, 2009, GEODERMA, V149, P101, DOI 10.1016/j.geoderma.2008.11.034"
"   Adimalla N, 2018, ARAB J GEOSCI, V11, DOI 10.1007/s12517-018-4028-y"
"   Ajmone-Marsan F, 2008, ENVIRON POLLUT, V152, P73, DOI 10.1016/j.envpol.2007.05.020"
"   Altin O, 1998, J COLLOID INTERF SCI, V198, P130, DOI 10.1006/jcis.1997.5246"
"   Alumaa P, 2002, INT J HYG ENVIR HEAL, V204, P375, DOI 10.1078/1438-4639-00114"
"   Ang L, 2021, INTEGR MED RES, V10"
"   Asad SA, 2019, CHEMOSPHERE, V217, P925, DOI 10.1016/j.chemosphere.2018.11.021"
"   Atkinson CA, 2007, CHEMOSPHERE, V69, P1428, DOI 10.1016/j.chemosphere.2007.04.068"
"   Ayrault S, 2014, ENVIRON SCI POLLUT R, V21, P4134, DOI 10.1007/s11356-013-2240-6"
"   Bagot D, 2006, ENVIRON CHEM LETT, V4, P207, DOI 10.1007/s10311-006-0047-0"
"   Bali AS, 2020, ENVIRON CHEM LETT, V18, P1243, DOI 10.1007/s10311-020-01012-x"
"   Bano A, 2018, CHEMOSPHERE, V199, P218, DOI 10.1016/j.chemosphere.2018.02.043"
"   Barros YJ, 2010, REV BRAS CIENC SOLO, V34, P1397, DOI 10.1590/S0100-06832010000400036"
"   BASTA NT, 1993, AGRON J, V85, P1054, DOI 10.2134/agronj1993.00021962008500050018x"
"   Baumert VL, 2018, FRONT ENV SCI-SWITZ, V6, DOI 10.3389/fenvs.2018.00140"
"   Begum ZA, 2012, CHEMOSPHERE, V87, P1161, DOI 10.1016/j.chemosphere.2012.02.032"
"   Bradl HB, 2004, J COLLOID INTERF SCI, V277, P1, DOI 10.1016/j.jcis.2004.04.005"
"   Cao DY, 2021, EUR J SOIL SCI, V72, P2165, DOI 10.1111/ejss.13095"
"   Castro L, 2018, HYDROMETALLURGY, V179, P44, DOI 10.1016/j.hydromet.2018.05.029"
"   Chen CL, 2016, GEODERMA, V275, P74, DOI 10.1016/j.geoderma.2016.04.015"
"   Chen S, 2007, AUST J SOIL RES, V45, P554, DOI 10.1071/SR07029"
"   Chen W, 2020, J HAZARD MATER, V393, DOI 10.1016/j.jhazmat.2020.122425"
"   Chen W, 2015, ENVIRON SCI TECHNOL, V49, P2052, DOI 10.1021/es5049495"
"   Chen WF, 2017, ENVIRON SCI POLLUT R, V24, P16344, DOI 10.1007/s11356-017-9272-2"
"   Chen YF, 2021, SCI TOTAL ENVIRON, V781, DOI 10.1016/j.scitotenv.2021.146773"

" Chen Y, 2016, ENVIRON SCI POLLUT R, V23, P4629, DOI 10.1007/s11356-015-5700-3"
" Chen YM, 2016, CONT SHELF RES, V124, P125, DOI 10.1016/j.csr.2016.06.002"
" Cheng SF, 2008, J CHIN INST ENG, V31, P231, DOI 10.1080/02533839.2008.9671376"
" Cheung KH, 2007, INT BIODETER BIODEGR, V59, P8, DOI 10.1016/j.ibiod.2006.05.002"
" Chowdhury S, 2011, CLEAN-SOIL AIR WATER, V39, P274, DOI 10.1002/clen.201000170"
" Covelo EF, 2008, J HAZARD MATER, V159, P342, DOI 10.1016/j.jhazmat.2008.02.025"
" Covelo EF, 2008, GEODERMA, V147, P75, DOI 10.1016/j.geoderma.2008.08.001"
" Crou?? JP, 2003, ENVIRON SCI TECHNOL, V37, P328, DOI 10.1021/es020676p"
" Cui HB, 2016, ENVIRON SCI POLLUT R, V23, P10808, DOI 10.1007/s11356-016-6271-7"
" Cui JY, 2021, SCI TOTAL ENVIRON, V761, DOI 10.1016/j.scitotenv.2020.144111"
" Deng AM, 2018, GLOB ECOL CONSERV, V16, DOI 10.1016/j.gecco.2018.e00465"
" Deng XH, 2012, J HAZARD MATER, V233, P25, DOI 10.1016/j.jhazmat.2012.06.054"
" Dercov?? K, 2005, CHEM LISTY, V99, P682"
" Dey CJ, 2020, P ROY SOC B-BIOL SCI, V287, DOI 10.1098/rspb.2020.0012"
" Dhaliwal SS, 2020, ENVIRON SCI POLLUT R, V27, P1319, DOI 10.1007/s11356-019-06967-1"
" Dhami NK, 2017, ECOL ENG, V103, P106, DOI 10.1016/j.ecoleng.2017.03.007"
" Dong J, 2021, APPL SCI-BASEL, V11, DOI 10.3390/app112210956"
" Dong XL, 2016, J SOIL SEDIMENT, V16, P1481, DOI 10.1007/s11368-015-1338-5"
" DRAZKIEWICZ M, 1994, FOLIA MICROBIOL, V39, P276, DOI 10.1007/BF02814312"
" Du JP, 2022, ENVIRON RES, V209, DOI 10.1016/j.envres.2022.112817"
" Eggleton J, 2004, ENVIRON INT, V30, P973, DOI 10.1016/j.envint.2004.03.001"
" Elbana TA, 2018, GEODERMA, V324, P80, DOI 10.1016/j.geoderma.2018.03.019"
" Emenike CU, 2018, ENVIRON REV, V26, P156, DOI 10.1139/er-2017-0045"
" Ertani A, 2017, WATER AIR SOIL POLL, V228, DOI 10.1007/s11270-017-3356-y"
" Fakhar A, 2022, CRIT REV ENV SCI TEC, V52, P1868, DOI 10.1080/10643389.2020.1863112"
" Fan JL, 2013, COMMUN SOIL SCI PLAN, V44, P1224, DOI 10.1080/00103624.2012.756002"
" Fan WH, 2002, MAR POLLUT BULL, V44, P820, DOI 10.1016/S0025-326X(02)00069-3"
" Farshadirad A, 2019, J SOIL SEDIMENT, V19, P1866, DOI 10.1007/s11368-018-2187-9"
" Feizi M, 2019, J HAZARD MATER, V379, DOI 10.1016/j.jhazmat.2019.04.050"
" Feng SP, 2012, J ENVIRON ENG-ASCE, V138, P483, DOI 10.1061/(ASCE)EE.1943-7870.0000486"
" Frankowski M, 2009, ENVIRON MONIT ASSESS, V155, P493, DOI 10.1007/s10661-008-0476-2"
" Gao JB, 2018, J SOIL SEDIMENT, V18, P1020, DOI 10.1007/s11368-017-1759-4"
" Gao RL, 2016, ENVIRON SCI POLLUT R, V23, P23271, DOI 10.1007/s11356-016-7560-x"
" Ge SQ, 2021, J CLEAN PROD, V324, DOI 10.1016/j.jclepro.2021.129184"
" Ghosh A, 2016, J ENVIRON ENG, V142, DOI 10.1061/(ASCE)EE.1943-7870.0000965"
" Ghosh S, 1999, INDIAN J CHEM A, V38, P10"
" Gluhar S, 2020, CHEMOSPHERE, V257, DOI 10.1016/j.chemosphere.2020.127226"
" G??mez-Garrido M, 2018, INT J PHYTOREMEDIAT, V20, P1033, DOI 10.1080/15226514.2018.1452189"
" Gong C, 2014, J GEOCHEM EXPLOR, V139, P109, DOI 10.1016/j.gexplo.2013.01.002"
" Gong Cang, 2013, Huanjing Kexue, V34, P1094"
" Gul S, 2015, POL J ENVIRON STUD, V24, P1423, DOI 10.15244/pjoes/26970"
" Guo L, 2014, ECOTOX ENVIRON SAFE, V104, P72, DOI 10.1016/j.ecoenv.2014.02.019"
" Guo N, 2015, J RADIOANAL NUCL CH, V303, P2185, DOI 10.1007/s10967-014-3768-8"
" Guo XF, 2018, CHEMOSPHERE, V209, P776, DOI 10.1016/j.chemosphere.2018.06.144"
" Guo ZH, 2021, ENVIRON ENG SCI, V38, P764, DOI 10.1089/ees.2020.0356"
" Gusiatin ZM, 2020, INT J ENV RES PUB HE, V17, DOI 10.3390/ijerph17176220"
" Hanif MA, 2010, ASIAN J CHEM, V22, P335"
" Harvey HJ, 2020, SOIL BIOL BIOCHEM, V148, DOI 10.1016/j.soilbio.2020.107870"
" He ZLL, 2005, J TRACE ELEM MED BIO, V19, P125, DOI 10.1016/j.jtemb.2005.02.010"
" Hernandez L, 2003, SCI TOTAL ENVIRON, V312, P195, DOI 10.1016/S0048-9697(03)00223-7"
" Ho YS, 2000, WATER RES, V34, P735, DOI 10.1016/S0043-1354(99)00232-8"
" Ho YS, 2019, ECOHYDROLOGY, V12, DOI 10.1002/eco.2111"
" Hong YK, 2022, TOXICS, V10, DOI 10.3390/toxics10020090"
" Huang B, 2020, ENVIRON SCI-PROC IMP, V22, P1596, DOI [10.1039/d0em00189a, 10.1039/D0EM00189A]"
" Huang B, 2017, ENVIRON SCI POLLUT R, V24, P23939, DOI 10.1007/s11356-017-0012-4"
" Huang B, 2014, J HAZARD MATER, V264, P176, DOI 10.1016/j.jhazmat.2013.10.074"
" Huang SH, 2019, SUSTAINABILITY-BASEL, V11, DOI 10.3390/su11195166"
" Huang Y, 2020, ECOTOX ENVIRON SAFE, V204, DOI 10.1016/j.ecoenv.2020.111129"
" Hur J, 2011, CHEMOSPHERE, V83, P1603, DOI 10.1016/j.chemosphere.2011.01.004"
" Ikegami M, 2014, RISK ANAL, V34, P1677, DOI 10.1111/risa.12215"
" Ilg K, 2004, GEODERMA, V123, P153, DOI 10.1016/j.geoderma.2004.02.006"
" Im J, 2015, CHEMOSPHERE, V138, P253, DOI 10.1016/j.chemosphere.2015.06.004"
" Ingelmo F, 2012, J ENVIRON MANAGE, V95, pS104, DOI 10.1016/j.jenvman.2011.04.015"
" Jadia CD, 2009, AFR J BIOTECHNOL, V8, P921"
" Javanbakht V, 2014, WATER SCI TECHNOL, V69, P1775, DOI 10.2166/wst.2013.718"
" Jia JJ, 2020, ENVIRON POLLUT, V259, DOI 10.1016/j.envpol.2019.113848"
" Jia ZY, 2020, ENVIRON POLLUT, V256, DOI 10.1016/j.envpol.2019.113505"
" Jung J, 2016, MAR GENOM, V30, P73, DOI 10.1016/j.margen.2016.07.002"
" Kamaludeen SPB, 2003, REV ENVIRON CONTAM T, V178, P93, DOI 10.1007/0-387-21728-2_4"
" Kamdem JP, 2019, FOOD CHEM, V294, P448, DOI 10.1016/j.foodchem.2019.05.021"
" Kang CH, 2016, ECOL ENG, V89, P64, DOI 10.1016/j.ecoleng.2016.01.023"
" Karer J, 2015, WATER AIR SOIL POLL, V226, DOI 10.1007/s11270-015-2584-2"
" Kavamura VN, 2010, BIOTECHNOL ADV, V28, P61, DOI 10.1016/j.biotechadv.2009.09.002"
" Khalid S, 2017, J GEOCHEM EXPLOR, V182, P247, DOI 10.1016/j.gexplo.2016.11.021"
" Kiem R, 1997, APPL SOIL ECOL, V5, P221, DOI 10.1016/S0929-1393(96)00132-1"
" Kinoshita H, 2013, RES MICROBIOL, V164, P701, DOI 10.1016/j.resmic.2013.04.004"
" Kong YL, 2022, SCI TOTAL ENVIRON, V824, DOI 10.1016/j.scitotenv.2022.153755"
" Koo BJ, 2010, ENVIRON POLLUT, V158, P2582, DOI 10.1016/j.envpol.2010.05.018"
" Kools SAE, 2005, SOIL BIOL BIOCHEM, V37, P1303, DOI 10.1016/j.soilbio.2004.11.026"
" Kordialik-Bogacka E, 2011, CENT EUR J CHEM, V9, P348, DOI 10.2478/s11532-011-0008-8"
" Kubier A, 2019, APPL GEOCHEM, V108, DOI 10.1016/j.apgeochem.2019.104388"
" Kumar PS, 2010, BRAZ J CHEM ENG, V27, P339, DOI 10.1590/S0104-66322010000200012"
" Kumpiene J, 2008, WASTE MANAGE, V28, P215, DOI 10.1016/j.wasman.2006.12.012"
" Labanowski J, 2008, ENVIRON POLLUT, V152, P693, DOI 10.1016/j.envpol.2007.06.054"

" Langmuir I, 1916, J AM CHEM SOC, V38, P2221, DOI 10.1021/ja02268a002"
" Lee MH, 2021, PROCESSES, V9, DOI 10.3390/pr9081431"
" Lehrsch GA, 2013, HYDROL PROCESS, V27, P1739, DOI 10.1002/hyp.9320"
" Leitenmaier B, 2013, FRONT PLANT SCI, V4, DOI 10.3389/fpls.2013.00374"
" Leterme B, 2015, ENVIRON SCI POLLUT R, V22, P16830, DOI 10.1007/s11356-015-4876-x"
" Colin VL, 2012, INT BIODETER BIODEGR, V69, P28, DOI 10.1016/j.ibiod.2011.12.001"
" Li CF, 2019, SOIL SEDIMENT CONTAM, V28, P380, DOI 10.1080/15320383.2019.1592108"
" Li DW, 2013, RES J CHEM ENVIRON, V17, P76"
" Li DW, 2012, RES J CHEM ENVIRON, V16, P127"
" Li K, 2019, PEERJ, V7, DOI 10.7717/peerj.6631"
" Li KF, 2011, J HAZARD MATER, V189, P531, DOI 10.1016/j.jhazmat.2011.02.075"
" Li L, 2011, J ENVIRON SCI-CHINA, V23, P108, DOI 10.1016/S1001-0742(10)60380-4"
" Li LY, 2000, CAN GEOTECH J, V37, P296, DOI 10.1139/cgj-37-2-296"
" Li NJ, 2020, FRESEN ENVIRON BULL, V29, P5152"
" Li PS, 2015, CRIT REV MICROBIOL, V41, P140, DOI 10.3109/1040841X.2013.813898"
" Li Q, 2018, ENVIRON POLLUT, V233, P569, DOI 10.1016/j.envpol.2017.10.091"
" Li Q, 2022, FRONT ENV SCI ENG, V16, DOI 10.1007/s11783-021-1488-1"
" Li SS, 2019, ENVIRON SCI POLLUT R, V26, P20929, DOI 10.1007/s11356-019-05431-4"
" Li YJ, 2015, ENVIRON SCI POLLUT R, V22, P5563, DOI 10.1007/s11356-014-3720-z"
" Li ZW, 2015, RSC ADV, V5, P90588, DOI 10.1039/c5ra16501f"
" Liang YZ, 2019, SCI TOTAL ENVIRON, V656, P521, DOI 10.1016/j.scitotenv.2018.11.386"
" Liu BL, 2016, J SOIL SEDIMENT, V16, P1547, DOI 10.1007/s11368-016-1351-3"
" Liu HL, 2017, ENVIRON TOXICOL CHEM, V36, P1005, DOI 10.1002/etc.3618"
" Liu L, 2006, ENVIRON GEOL, V50, P431, DOI 10.1007/s00254-006-0226-0"
" Liu LW, 2018, SCI TOTAL ENVIRON, V633, P206, DOI 10.1016/j.scitotenv.2018.03.161"
" Liu MX, 2017, PROG CHEM, V29, P1537, DOI 10.7536/PC170739"
" Liu MX, 2020, B ENVIRON CONTAM TOX, V104, P532, DOI 10.1007/s00128-020-02810-3"
" Liu XH, 2012, INT J AGRIC BIOL, V14, P975"
" LO KSL, 1992, TOXICOL ENVIRON CHEM, V34, P139, DOI 10.1080/02772249209357787"
" Lombi E, 2000, WATER AIR SOIL POLL, V124, P319, DOI 10.1023/A:1005230628958"
" Luo LQ, 2018, ENVIRON SCI POLLUT R, V25, P24111, DOI 10.1007/s11356-018-2482-4"
" Lwin CS, 2022, PEDOSPHERE, V32, P307, DOI 10.1016/S1002-0160(21)60075-9"
" Ma L, 2010, J ENVIRON SCI-CHINA, V22, P689, DOI 10.1016/S1001-0742(09)60164-9"
" Madrid F, 2008, ARCH ENVIRON CON TOX, V55, P21, DOI 10.1007/s00244-007-9086-1"
" Maity S, 2008, BIORESOURCE TECHNOL, V99, P7291, DOI 10.1016/j.biortech.2007.12.079"
" Makino T, 2008, CHEMOSPHERE, V70, P1035, DOI 10.1016/j.chemosphere.2007.07.080"
" Makino T, 2007, ENVIRON POLLUT, V147, P112, DOI 10.1016/j.envpol.2006.08.037"
" Manirakiza N, 2018, FRESEN ENVIRON BULL, V27, P8637"
" Mao XH, 2015, J HAZARD MATER, V285, P419, DOI 10.1016/j.jhazmat.2014.12.009"
" MATERECHERA SA, 1992, PLANT SOIL, V142, P69, DOI 10.1007/BF00010176"
" Mellis EV, 2004, SCI AGR, V61, P190, DOI 10.1590/S0103-90162004000200011"
" Miao XY, 2020, J COAST CONSERV, V24, DOI 10.1007/s11852-020-00786-0"
" Miao XY, 2019, J COAST CONSERV, V23, P643, DOI 10.1007/s11852-019-00695-x"
" Miransari M, 2011, BIOTECHNOL ADV, V29, P645, DOI 10.1016/j.biotechadv.2011.04.006"
" Mng'ong'o M, 2021, J ENVIRON MANAGE, V294, DOI 10.1016/j.jenvman.2021.112973"
" Moon DH, 2021, ENVIRON GEOCHEM HLTH, V43, P3343, DOI 10.1007/s10653-021-00815-3"
" Morton-Bermea O, 2009, J GEOCHEM EXPLOR, V101, P218, DOI 10.1016/j.gexplo.2008.07.002"
" Mosbah R, 2013, CENT EUR J CHEM, V11, P1412, DOI 10.2478/s11532-013-0268-6"
" Muthusaravanan S, 2018, ENVIRON CHEM LETT, V16, P1339, DOI 10.1007/s10311-018-0762-3"
" Nam IH, 2014, NEW BIOTECHNOL, V31, pS135, DOI 10.1016/j.nbt.2014.05.1947"
" Niu ZX, 2011, SOIL SEDIMENT CONTAM, V20, P877, DOI 10.1080/15320383.2011.620042"
" Niu ZX, 2013, INT J PHYTOREMEDIAT, V15, P690, DOI 10.1080/15226514.2012.723066"
" Nouri H, 2023, INT J ENVIRON AN CH, V103, P6017, DOI 10.1080/03067319.2021.1946688"
" Otunola BO, 2020, ENVIRON TECHNOL INNO, V18, DOI 10.1016/j.eti.2020.100692"
" Ouyang L, 2013, J SOIL SCI PLANT NUT, V13, P991, DOI 10.4067/S0718-95162013005000078"
" Pakdil NB, 2007, WATER SCI TECHNOL, V56, P87, DOI 10.2166/wst.2007.707"
" Palansooriya KN, 2020, ENVIRON INT, V134, DOI 10.1016/j.envint.2019.105046"
" Pan JW, 2022, SCI TOTAL ENVIRON, V805, DOI 10.1016/j.scitotenv.2021.150413"
" Peng LF, 2018, GEOCHIM COSMOCHIM AC, V224, P282, DOI 10.1016/j.gca.2018.01.014"
" Peng SM, 2018, SOIL SCI SOC AM J, V82, P839, DOI 10.2136/sssaj2018.03.0104"
" Pierattini EC, 2018, TREE PHYSIOL, V38, P109, DOI 10.1093/treephys/tpx155"
" Polti MA, 2009, J BASIC MICROB, V49, P285, DOI 10.1002/jobm.200800239"
" Priovashini C, 2022, AMBIO, V51, P241, DOI 10.1007/s13280-021-01543-9"
" Rabot E, 2018, GEODERMA, V314, P122, DOI 10.1016/j.geoderma.2017.11.009"
" Raffa CM, 2021, APPL SCI-BASEL, V11, DOI 10.3390/app11094134"
" Rassaei Farzad, 2021, Arabian Journal of Geosciences, DOI [10.1007/s12517-021-08594-y, 10.1007/s12517-021-08594-y]"
" REED BE, 1994, SEPAR SCI TECHNOL, V29, P1529, DOI 10.1080/01496399408007372"
" Ren C, 2022, AGRICULTURE-BASEL, V12, DOI 10.3390/agriculture12020307"
" Sangiumsak N, 2014, POL J ENVIRON STUD, V23, P853"
" Savenko AV, 2016, GEOCHEM INT+, V54, P719, DOI 10.1134/S0016702916060069"
" Schwab P, 2007, BIORESOURCE TECHNOL, V98, P2935, DOI 10.1016/j.biortech.2006.10.012"
" Serrano S, 2005, GEODERMA, V124, P91, DOI 10.1016/j.geoderma.2004.04.002"
" Shao Y, 2021, WATER AIR SOIL POLL, V232, DOI 10.1007/s11270-021-05368-w"
" Shen Q, 2022, INT J ENVIRON AN CH, V102, P4272, DOI 10.1080/03067319.2020.1781840"
" Shen Q, 2020, B ENVIRON CONTAM TOX, V104, P144, DOI 10.1007/s00128-019-02739-2"
" Shi Qiong-bin, 2016, Huan Jing Ke Xue, V37, P1923"
[truncated: 640,595 more chars]
